# Global incidence, prevalence, years lived with disability (YLDs), disability-adjusted life-years (DALYs), and healthy life expectancy (HALE) for 371 diseases and injuries in 204 countries and territories and 811 subnational locations, 1990–2021: a systematic analysis for the Global Burden of Disease Study 2021

**DOI:** 10.1016/S0140-6736(24)00757-8

**Published:** 2024-05-18

**Authors:** GBD 2021 Diseases and Injuries Collaborators, Alize J Ferrari, Damian Francesco Santomauro, Amirali Aali, Yohannes Habtegiorgis Abate, Cristiana Abbafati, Hedayat Abbastabar, Samar Abd ElHafeez, Michael Abdelmasseh, Sherief Abd-Elsalam, Arash Abdollahi, Auwal Abdullahi, Kedir Hussein Abegaz, Roberto Ariel Abeldaño Zuñiga, Richard Gyan Aboagye, Hassan Abolhassani, Lucas Guimarães Abreu, Hasan Abualruz, Eman Abu-Gharbieh, Niveen ME Abu-Rmeileh, Ilana N Ackerman, Isaac Yeboah Addo, Giovanni Addolorato, Akindele Olupelumi Adebiyi, Abiola Victor Adepoju, Habeeb Omoponle Adewuyi, Shadi Afyouni, Saira Afzal, Sina Afzal, Antonella Agodi, Aqeel Ahmad, Danish Ahmad, Firdos Ahmad, Shahzaib Ahmad, Ali Ahmed, Luai A Ahmed, Muktar Beshir Ahmed, Marjan Ajami, Karolina Akinosoglou, Mohammed Ahmed Akkaif, Syed Mahfuz Al Hasan, Samer O Alalalmeh, Ziyad Al-Aly, Mohammed Albashtawy, Robert W Aldridge, Meseret Desalegn Alemu, Yihun Mulugeta Alemu, Kefyalew Addis Alene, Adel Ali Saeed Al-Gheethi, Maryam Alharrasi, Robert Kaba Alhassan, Mohammed Usman Ali, Rafat Ali, Syed Shujait Shujait Ali, Sheikh Mohammad Alif, Syed Mohamed Aljunid, Sabah Al-Marwani, Joseph Uy Almazan, Mahmoud A Alomari, Basem Al-Omari, Zaid Altaany, Nelson Alvis-Guzman, Nelson J Alvis-Zakzuk, Hassan Alwafi, Mohammad Sami Al-Wardat, Yaser Mohammed Al-Worafi, Safwat Aly, Karem H Alzoubi, Azmeraw T Amare, Prince M Amegbor, Edward Kwabena Ameyaw, Tarek Tawfik Amin, Alireza Amindarolzarbi, Sohrab Amiri, Dickson A Amugsi, Robert Ancuceanu, Deanna Anderlini, David B Anderson, Pedro Prata Andrade, Catalina Liliana Andrei, Hossein Ansari, Catherine M Antony, Saleha Anwar, Sumadi Lukman Anwar, Razique Anwer, Philip Emeka Anyanwu, Juan Pablo Arab, Jalal Arabloo, Mosab Arafat, Daniel T Araki, Aleksandr Y Aravkin, Mesay Arkew, Benedetta Armocida, Michael Benjamin Arndt, Mahwish Arooj, Anton A Artamonov, Raphael Taiwo Aruleba, Ashokan Arumugam, Charlie Ashbaugh, Mubarek Yesse Ashemo, Muhammad Ashraf, Marvellous O Asika, Elaheh Askari, Thomas Astell-Burt, Seyyed Shamsadin Athari, Prince Atorkey, Maha Moh'd Wahbi Atout, Alok Atreya, Avinash Aujayeb, Marcel Ausloos, Abolfazl Avan, Adedapo Wasiu Awotidebe, Kofi Awuviry-Newton, Beatriz Paulina Ayala Quintanilla, Jose L Ayuso-Mateos, Sina Azadnajafabad, Rui M S Azevedo, Abraham Samuel Babu, Muhammad Badar, Ashish D Badiye, Soroush Baghdadi, Nasser Bagheri, Sulaiman Bah, Ruhai Bai, Jennifer L Baker, Shankar M Bakkannavar, Abdulaziz T Bako, Senthilkumar Balakrishnan, Kiran Bam, Palash Chandra Banik, Martina Barchitta, Mainak Bardhan, Erfan Bardideh, Suzanne Lyn Barker-Collo, Hiba Jawdat Barqawi, Amadou Barrow, Sandra Barteit, Lingkan Barua, Somaye Bashiri Aliabadi, Afisu Basiru, Sanjay Basu, Saurav Basu, Prapthi Persis Bathini, Kavita Batra, Bernhard T Baune, Nebiyou Simegnew Bayileyegn, Babak Behnam, Amir Hossein Behnoush, Maryam Beiranvand, Diana Fernanda Bejarano Ramirez, Michelle L Bell, Olorunjuwon Omolaja Bello, Apostolos Beloukas, Isabela M Bensenor, Zombor Berezvai, Eduardo Bernabe, Robert S Bernstein, Paulo J G Bettencourt, Akshaya Srikanth Bhagavathula, Neeraj Bhala, Dinesh Bhandari, Ashish Bhargava, Sonu Bhaskar, Vivek Bhat, Gurjit Kaur Bhatti, Jasvinder Singh Bhatti, Manpreet S Bhatti, Rajbir Bhatti, Zulfiqar A Bhutta, Boris Bikbov, Jessica Devin Bishai, Catherine Bisignano, Veera R Bitra, Tone Bjørge, Virginia Bodolica, Aadam Olalekan Bodunrin, Eyob Ketema Bogale, Milad Bonakdar Hashemi, Aime Bonny, Berrak Bora Basara, Hamed Borhany, Christopher Boxe, Oliver J Brady, Nicola Luigi Bragazzi, Dejana Braithwaite, Luisa C Brant, Michael Brauer, Susanne Breitner, Hermann Brenner, Julie Brown, Traolach Brugha, Norma B Bulamu, Danilo Buonsenso, Katrin Burkart, Richard A Burns, Reinhard Busse, Yasser Bustanji, Zahid A Butt, Justin Byun, Florentino Luciano Caetano dos Santos, Daniela Calina, Luis Alberto Cámera, Ismael R Campos-Nonato, Chao Cao, Angelo Capodici, Sinclair Carr, Giulia Carreras, Andrea Carugno, Márcia Carvalho, Joao Mauricio Castaldelli-Maia, Carlos A Castañeda-Orjuela, Giulio Castelpietra, Alberico L Catapano, Maria Sofia Cattaruzza, Arthur Caye, Luca Cegolon, Francieli Cembranel, Muthia Cenderadewi, Ester Cerin, Promit Ananyo Chakraborty, Jeffrey Shi Kai Chan, Raymond N C Chan, Rama Mohan Chandika, Eeshwar K Chandrasekar, Periklis Charalampous, Vijay Kumar Chattu, Victoria Chatzimavridou-Grigoriadou, Angela W Chen, An-Tian Chen, Catherine S Chen, Haowei Chen, Nathalie M Chen, Esther T W Cheng, Odgerel Chimed-Ochir, Ritesh Chimoriya, Patrick R Ching, William C S Cho, Sungchul Choi, Bryan Chong, Yuen Yu Chong, Sonali Gajanan Choudhari, Rajiv Chowdhury, Steffan Wittrup McPhee Christensen, Dinh-Toi Chu, Isaac Sunday Chukwu, Eric Chung, Eunice Chung, Muhammad Chutiyami, Mareli M Claassens, Rebecca M Cogen, Alyssa Columbus, Joao Conde, Paolo Angelo Cortesi, Ewerton Cousin, Michael H Criqui, Natália Cruz-Martins, Omid Dadras, Siyu Dai, Xiaochen Dai, Zhaoli Dai, Maxwell Ayindenaba Dalaba, Giovanni Damiani, Jai K Das, Saswati Das, Mohsen Dashti, Claudio Alberto Dávila-Cervantes, Kairat Davletov, Diego De Leo, Aklilu Tamire Debele, Shayom Debopadhaya, Nicole K DeCleene, Farah Deeba, Louisa Degenhardt, Cristian Del Bo', Ivan Delgado-Enciso, Andreas K Demetriades, Edgar Denova-Gutiérrez, Nikolaos Dervenis, Hardik Dineshbhai Desai, Rupak Desai, Keshab Deuba, Kuldeep Dhama, Samath Dhamminda Dharmaratne, Sameer Dhingra, Diana Dias da Silva, Daniel Diaz, Luis Antonio Diaz, Michael J Diaz, Adriana Dima, Delaney D Ding, M Ashworth Dirac, Thao Huynh Phuong Do, Camila Bruneli do Prado, Sushil Dohare, Regina-Mae Villanueva Dominguez, Wanyue Dong, Deepa Dongarwar, Mario D'Oria, E Ray Dorsey, Leila Doshmangir, Robert Kokou Dowou, Tim Robert Driscoll, Haneil Larson Dsouza, Viola Dsouza, John Dube, Samuel C Dumith, Bruce B Duncan, Andre Rodrigues Duraes, Senbagam Duraisamy, Oyewole Christopher Durojaiye, Paulina Agnieszka Dzianach, Arkadiusz Marian Dziedzic, Ejemai Eboreime, Alireza Ebrahimi, Hisham Atan Edinur, David Edvardsson, Terje Andreas Eikemo, Ebrahim Eini, Michael Ekholuenetale, Temitope Cyrus Ekundayo, Iman El Sayed, Maha El Tantawi, Iffat Elbarazi, Noha Mousaad Elemam, Ghada Metwally Tawfik ElGohary, Muhammed Elhadi, Omar Abdelsadek Abdou Elmeligy, Gihan ELNahas, Mohammed Elshaer, Ibrahim Elsohaby, Luchuo Engelbert Bain, Ryenchindorj Erkhembayar, Babak Eshrati, Kara Estep, Natalia Fabin, Adeniyi Francis Fagbamigbe, Luca Falzone, Mohammad Fareed, Carla Sofia e Sá Farinha, MoezAlIslam Ezzat Mahmoud Faris, Andre Faro, Pegah Farrokhi, Ali Fatehizadeh, Nelsensius Klau Fauk, Valery L Feigin, Xiaoqi Feng, Seyed-Mohammad Fereshtehnejad, Abdullah Hamid Feroze, Nuno Ferreira, Paulo H Ferreira, Florian Fischer, Joanne Flavel, David Flood, Luisa S Flor, Nataliya A Foigt, Morenike Oluwatoyin Folayan, Lisa M Force, Daniela Fortuna, Matteo Foschi, Richard Charles Franklin, Alberto Freitas, Takeshi Fukumoto, João M Furtado, Peter Andras Gaal, Muktar A Gadanya, Abhay Motiramji Gaidhane, Santosh Gaihre, Yaseen Galali, Mandukhai Ganbat, Aravind P Gandhi, Balasankar Ganesan, Mohd Ashraf Ganie, Mohammad Arfat Ganiyani, William M Gardner, Tilaye Gebru Gebi, Miglas W Gebregergis, Mesfin Gebrehiwot, Tesfay B B Gebremariam, Teferi Gebru Gebremeskel, Yibeltal Yismaw Gela, Simona Roxana Georgescu, Abera Getachew Obsa, Peter W Gething, Molla Getie, Keyghobad Ghadiri, Fataneh Ghadirian, Khalid Yaser Ghailan, Alireza Ghajar, MohammadReza Ghasemi, Ghazal Ghasempour Dabaghi, Afsaneh Ghasemzadeh, Ramy Mohamed Ghazy, Ali Gholamrezanezhad, Mahsa Ghorbani, Elena Ghotbi, Ruth Margaret Gibson, Tiffany K Gill, Themba G Ginindza, Alem Girmay, James C Glasbey, Laszlo Göbölös, Myron Anthony Godinho, Salime Goharinezhad, Mohamad Goldust, Mahaveer Golechha, Pouya Goleij, Philimon N Gona, Giuseppe Gorini, Alessandra C Goulart, Ayman Grada, Michal Grivna, Shi-Yang Guan, Giovanni Guarducci, Mohammed Ibrahim Mohialdeen Gubari, Mesay Dechasa Gudeta, Avirup Guha, Stefano Guicciardi, Snigdha Gulati, David Gulisashvili, Damitha Asanga Gunawardane, Cui Guo, Anish Kumar Gupta, Bhawna Gupta, Ishita Gupta, Mohak Gupta, Rajeev Gupta, Veer Bala Gupta, Vijai Kumar Gupta, Vivek Kumar Gupta, Reyna Alma Gutiérrez, Farrokh Habibzadeh, Parham Habibzadeh, Rasool Haddadi, Najah R Hadi, Nils Haep, Nima Hafezi-Nejad, Abdul Hafiz, Hailey Hagins, Esam S Halboub, Aram Halimi, Sebastian Haller, Rabih Halwani, Erin B Hamilton, Graeme J Hankey, Md Abdul Hannan, Md Nuruzzaman Haque, Harapan Harapan, Josep Maria Haro, Jan Hartvigsen, Ahmed I Hasaballah, Ikramul Hasan, Mohammad Hasanian, Md Saquib Hasnain, Amr Hassan, Johannes Haubold, Rasmus J Havmoeller, Simon I Hay, Khezar Hayat, Jeffrey J Hebert, Omar E Hegazi, Golnaz Heidari, Bartosz Helfer, Mehdi Hemmati, Delia Hendrie, Claire A Henson, Kamal Hezam, Yuta Hiraike, Nguyen Quoc Hoan, Ramesh Holla, Julia Hon, Md Mahbub Hossain, Hassan Hosseinzadeh, Mehdi Hosseinzadeh, Mihaela Hostiuc, Sorin Hostiuc, Johnathan M Hsu, Junjie Huang, Fernando N Hugo, Kiavash Hushmandi, Javid Hussain, Nawfal R Hussein, Chantal K Huynh, Hong-Han Huynh, Bing-Fang Hwang, Vincent C Iannucci, Audrey L Ihler, Adalia I Ikiroma, Kevin S Ikuta, Olayinka Stephen Ilesanmi, Irena M Ilic, Milena D Ilic, Mohammad Tarique Imam, Mustapha Immurana, Lalu Muhammad Irham, Md Rabiul Islam, Sheikh Mohammed Shariful Islam, Farhad Islami, Faisal Ismail, Nahlah Elkudssiah Ismail, Gaetano Isola, Masao Iwagami, Chidozie C D Iwu, Mahalaxmi Iyer, Jalil Jaafari, Kathryn H Jacobsen, Farhad Jadidi-Niaragh, Morteza Jafarinia, Khushleen Jaggi, Kasra Jahankhani, Nader Jahanmehr, Haitham Jahrami, Akhil Jain, Nityanand Jain, Ammar Abdulrahman Jairoun, Abhishek Jaiswal, Mihajlo Jakovljevic, Abubakar Ibrahim Jatau, Sabzali Javadov, Tahereh Javaheri, Sathish Kumar Jayapal, Shubha Jayaram, Sun Ha Jee, Jayakumar Jeganathan, Angeline Jeyakumar, Anil K Jha, Heng Jiang, Yinzi Jin, Jost B Jonas, Tamas Joo, Abel Joseph, Nitin Joseph, Charity Ehimwenma Joshua, Jacek Jerzy Jozwiak, Mikk Jürisson, Vaishali K, Billingsley Kaambwa, Ali Kabir, Zubair Kabir, Vidya Kadashetti, Rizwan Kalani, Leila R Kalankesh, Feroze Kaliyadan, Sanjay Kalra, Kaloyan Kamenov, Naser Kamyari, Thanigaivelan Kanagasabai, Himal Kandel, Arun R Kanmanthareddy, Kehinde Kazeem Kanmodi, Rami S Kantar, Ibraheem M Karaye, Asima Karim, Salah Eddin Karimi, Yeganeh Karimi, Hengameh Kasraei, Molly B Kassel, Joonas H Kauppila, Norito Kawakami, Gbenga A Kayode, Foad Kazemi, Sina Kazemian, Leila Keikavoosi-Arani, Cathleen Keller, John H Kempen, Jessica A Kerr, Kamyab Keshtkar, Emmanuelle Kesse-Guyot, Mohammad Keykhaei, Himanshu Khajuria, Amirmohammad Khalaji, Asaad Khalid, Nauman Khalid, Alireza Khalilian, Faham Khamesipour, Asaduzzaman Khan, Ikramullah Khan, Maseer Khan, Moien AB Khan, Shaghayegh Khanmohammadi, Khaled Khatab, Fatemeh Khatami, Moawiah Mohammad Khatatbeh, Amir M Khater, Hamid Reza Khayat Kashani, Feriha Fatima Khidri, Elaheh Khodadoust, Moein Khormali, Zahra Khorrami, Zemene Demelash Kifle, Min Seo Kim, Ruth W Kimokoti, Adnan Kisa, Sezer Kisa, Ann Kristin Skrindo Knudsen, Jonathan M Kocarnik, Sonali Kochhar, Hyun Yong Koh, Ali-Asghar Kolahi, Farzad Kompani, Gerbrand Koren, Oleksii Korzh, Soewarta Kosen, Sindhura Lakshmi Koulmane Laxminarayana, Kewal Krishan, Varun Krishna, Vijay Krishnamoorthy, Barthelemy Kuate Defo, Md Abdul Kuddus, Mohammed Kuddus, Ilari Kuitunen, Vishnutheertha Kulkarni, Manasi Kumar, Nithin Kumar, Rakesh Kumar, Om P Kurmi, Dian Kusuma, Hmwe Hmwe Kyu, Carlo La Vecchia, Ben Lacey, Muhammad Awwal Ladan, Lucie Laflamme, Alessandra Lafranconi, Chandrakant Lahariya, Daphne Teck Ching Lai, Dharmesh Kumar Lal, Ratilal Lalloo, Tea Lallukka, Judit Lám, Qing Lan, Tuo Lan, Iván Landires, Francesco Lanfranchi, Berthold Langguth, Ariane Laplante-Lévesque, Bagher Larijani, Anders O Larsson, Savita Lasrado, Paolo Lauriola, Huu-Hoai Le, Long Khanh Dao Le, Nhi Huu Hanh Le, Trang Diep Thanh Le, Janet L Leasher, Caterina Ledda, Munjae Lee, Paul H Lee, Sang-woong Lee, Seung Won Lee, Wei-Chen Lee, Yo Han Lee, Kate E LeGrand, Jacopo Lenzi, Elvynna Leong, Janni Leung, Ming-Chieh Li, Wei Li, Xiaopan Li, Yichong Li, Yongze Li, Lee-Ling Lim, Stephen S Lim, Megan Lindstrom, Shai Linn, Gang Liu, Runben Liu, Shiwei Liu, Wei Liu, Xiaofeng Liu, Xuefeng Liu, Erand Llanaj, Chun-Han Lo, Rubén López-Bueno, Arianna Maever Loreche, László Lorenzovici, Rafael Lozano, Jailos Lubinda, Giancarlo Lucchetti, Raimundas Lunevicius, Jay B Lusk, hengliang lv, Zheng Feei Ma, Nikolaos Machairas, Áurea M Madureira-Carvalho, Javier A Magaña Gómez, Azzam A Maghazachi, Preeti Maharjan, Phetole Walter Mahasha, Mina Maheri, Soleiman Mahjoub, Mansour Adam Mahmoud, Elham Mahmoudi, Azeem Majeed, Konstantinos Christos Makris, Elaheh Malakan Rad, Kashish Malhotra, Ahmad Azam Malik, Iram Malik, Deborah Carvalho Malta, Yosef Manla, Ali Mansour, Pejman Mansouri, Mohammad Ali Mansournia, Ana M Mantilla Herrera, Lorenzo Giovanni Mantovani, Emmanuel Manu, Hamid Reza Marateb, Parham Mardi, Gabriel Martinez, Ramon Martinez-Piedra, Daniela Martini, Francisco Rogerlândio Martins-Melo, Miquel Martorell, Wolfgang Marx, Sharmeen Maryam, Roy Rillera Marzo, Yasith Mathangasinghe, Stephanie Mathieson, Alexander G Mathioudakis, Jishanth Mattumpuram, Andrea Maugeri, Mahsa Mayeli, Mohsen Mazidi, Antonio Mazzotti, John J McGrath, Martin McKee, Anna Laura W McKowen, Michael A McPhail, Kamran Mehrabani-Zeinabad, Entezar Mehrabi Nasab, Tesfahun Mekene Meto, Walter Mendoza, Ritesh G Menezes, George A Mensah, Alexios-Fotios A Mentis, Sultan Ayoub Meo, Haftu Asmerom Meresa, Atte Meretoja, Tuomo J Meretoja, Abera M Mersha, Tomislav Mestrovic, Kukulege Chamila Dinushi Mettananda, Sachith Mettananda, Irmina Maria Michalek, Paul Anthony Miller, Ted R Miller, Edward J Mills, Le Huu Nhat Minh, Antonio Mirijello, Erkin M Mirrakhimov, Mizan Kiros Mirutse, Mohammad Mirza-Aghazadeh-Attari, Maryam Mirzaei, Roya Mirzaei, Awoke Misganaw, Ajay Kumar Mishra, Philip B Mitchell, Chaitanya Mittal, Babak Moazen, Madeline E Moberg, Jama Mohamed, Mouhand F H Mohamed, Nouh Saad Mohamed, Esmaeil Mohammadi, Soheil Mohammadi, Hussen Mohammed, Salahuddin Mohammed, Shafiu Mohammed, Robin M Mohr, Ali H Mokdad, Sabrina Molinaro, Sara Momtazmanesh, Lorenzo Monasta, Stefania Mondello, AmirAli Moodi Ghalibaf, Maryam Moradi, Yousef Moradi, Maziar Moradi-Lakeh, Paula Moraga, Lidia Morawska, Rafael Silveira Moreira, Negar Morovatdar, Shane Douglas Morrison, Jakub Morze, Abbas Mosapour, Jonathan F Mosser, Elias Mossialos, Rohith Motappa, Vincent Mougin, Simin Mouodi, Matías Mrejen, Ahmed Msherghi, Sumaira Mubarik, Ulrich Otto Mueller, Francesk Mulita, Kavita Munjal, Efrén Murillo-Zamora, BV Murlimanju, Ghulam Mustafa, Sathish Muthu, Muhammad Muzaffar, Woojae Myung, Ahamarshan Jayaraman Nagarajan, Pirouz Naghavi, Ganesh R Naik, Firzan Nainu, Sanjeev Nair, Hastyar Hama Rashid Najmuldeen, Vinay Nangia, Atta Abbas Naqvi, Aparna Ichalangod Narayana, Shumaila Nargus, Gustavo G Nascimento, Abdulqadir J Nashwan, Ali Nasrollahizadeh, Amir Nasrollahizadeh, Zuhair S Natto, Biswa Prakash Nayak, Vinod C Nayak, Sabina Onyinye Nduaguba, Hadush Negash, Ionut Negoi, Ruxandra Irina Negoi, Seyed Aria Nejadghaderi, Olivia D Nesbit, Henok Biresaw Netsere, Marie Ng, Georges Nguefack-Tsague, Josephine W Ngunjiri, Dang H Nguyen, Hien Quang Nguyen, Robina Khan Niazi, Taxiarchis Konstantinos Nikolouzakis, Ali Nikoobar, Fatemeh Nikoomanesh, Amin Reza Nikpoor, Chukwudi A Nnaji, Lawrence Achilles Nnyanzi, Efaq Ali Noman, Shuhei Nomura, Bo Norrving, Chisom Adaobi Nri-Ezedi, George Ntaios, Mpiko Ntsekhe, Dieta Nurrika, Chimezie Igwegbe Nzoputam, Ogochukwu Janet Nzoputam, Bogdan Oancea, Ismail A Odetokun, Martin James O'Donnell, Ayodipupo Sikiru Oguntade, James Odhiambo Oguta, Hassan Okati-Aliabad, Sylvester Reuben Okeke, Akinkunmi Paul Okekunle, Osaretin Christabel Okonji, Andrew T Olagunju, Omotola O Olasupo, Matthew Idowu Olatubi, Gláucia Maria Moraes Oliveira, Isaac Iyinoluwa Olufadewa, Bolajoko Olubukunola Olusanya, Jacob Olusegun Olusanya, Hany A Omar, Goran Latif Omer, Abidemi E Emmanuel Omonisi, Sandersan Onie, Obinna E Onwujekwe, Michal Ordak, Verner N Orish, Doris V Ortega-Altamirano, Alberto Ortiz, Edgar Ortiz-Brizuela, Wael M S Osman, Samuel M Ostroff, Uchechukwu Levi Osuagwu, Adrian Otoiu, Nikita Otstavnov, Stanislav S Otstavnov, Amel Ouyahia, Guoqing Ouyang, Mayowa O Owolabi, Mahesh Padukudru P A, Alicia Padron-Monedero, Jagadish Rao Padubidri, Tamás Palicz, Claudia Palladino, Feng Pan, Seithikurippu R Pandi-Perumal, Helena Ullyartha Pangaribuan, Georgios D Panos, Leonidas D Panos, Anca Mihaela Pantea Stoian, Shahina Pardhan, Romil R Parikh, Ava Pashaei, Maja Pasovic, Roberto Passera, Jay Patel, Sangram Kishor Patel, Shankargouda Patil, Dimitrios Patoulias, Venkata Suresh Patthipati, Shrikant Pawar, Hamidreza Pazoki Toroudi, Spencer A Pease, Amy E Peden, Paolo Pedersini, Minjin Peng, Umberto Pensato, Veincent Christian Filipino Pepito, Emmanuel K Peprah, Prince Peprah, João Perdigão, Maria Odete Pereira, Arokiasamy Perianayagam, Norberto Perico, Konrad Pesudovs, Fanny Emily Petermann-Rocha, William A Petri, Hoang Tran Pham, Anil K Philip, Michael R Phillips, Manon Pigeolet, David M Pigott, Julian David Pillay, Zahra Zahid Piracha, Saeed Pirouzpanah, Dietrich Plass, Evgenii Plotnikov, Dimitri Poddighe, Suzanne Polinder, Maarten J Postma, Naeimeh Pourtaheri, Sergio I Prada, Pranil Man Singh Pradhan, V Prakash, Manya Prasad, Elton Junio Sady Prates, Tina Priscilla, Natalie Pritchett, Pooja Puri, Jagadeesh Puvvula, Nameer Hashim Qasim, Ibrahim Qattea, Asma Saleem Qazi, Gangzhen Qian, Mehrdad Rabiee Rad, Raghu Anekal Radhakrishnan, Venkatraman Radhakrishnan, Hadi Raeisi Shahraki, Quinn Rafferty, Alberto Raggi, Pankaja Raghav Raghav, Md Jillur Rahim, Md Mosfequr Rahman, Mohammad Hifz Ur Rahman, Mosiur Rahman, Muhammad Aziz Rahman, Shayan Rahmani, Mohammad Rahmanian, Setyaningrum Rahmawaty, Sathish Rajaa, Mahmoud Mohammed Ramadan, Shakthi Kumaran Ramasamy, Premkumar Ramasubramani, Sheena Ramazanu, Kritika Rana, Chhabi Lal Ranabhat, Nemanja Rancic, Amey Rane, Chythra R Rao, Kumuda Rao, Mithun Rao, Sowmya J Rao, Mohammad-Mahdi Rashidi, Giridhara Rathnaiah Babu, Santosh Kumar Rauniyar, David Laith Rawaf, Salman Rawaf, Christian Razo, Murali Mohan Rama Krishna Reddy, Elrashdy Moustafa Mohamed Redwan, Lennart Reifels, Robert C Reiner Jr, Giuseppe Remuzzi, Andre M N Renzaho, Bhageerathy Reshmi, Luis Felipe Reyes, Nazila Rezaei, Negar Rezaei, Nima Rezaei, Peyman Rezaei Hachesu, Mohsen Rezaeian, Jennifer Rickard, Célia Fortuna Rodrigues, Jefferson Antonio Buendia Rodriguez, Leonardo Roever, Luca Ronfani, Gholamreza Roshandel, Kunle Rotimi, Himanshu Sekhar Rout, Bedanta Roy, Nitai Roy, Priyanka Roy, Enrico Rubagotti, Chandan S N, Aly M A Saad, Maha Mohamed Saber-Ayad, Siamak Sabour, Simona Sacco, Perminder S Sachdev, Basema Saddik, Adam Saddler, Bashdar Abuzed Sadee, Erfan Sadeghi, Masoumeh Sadeghi, Mohammad Reza Saeb, Umar Saeed, Sher Zaman Safi, Rajesh Sagar, Dominic Sagoe, Zahra Saif, Mirza Rizwan Sajid, Joseph W Sakshaug, Nasir Salam, Afeez Abolarinwa Salami, Luciane B Salaroli, Mohamed A Saleh, Marwa Rashad Salem, Mohammed Z Y Salem, Malik Sallam, Sara Samadzadeh, Saad Samargandy, Yoseph Leonardo Samodra, Abdallah M Samy, Juan Sanabria, Francesca Sanna, Itamar S Santos, Milena M Santric-Milicevic, Made Ary Sarasmita, Yaser Sarikhani, Rodrigo Sarmiento-Suárez, Gargi Sachin Sarode, Sachin C Sarode, Arash Sarveazad, Brijesh Sathian, Anudeep Sathyanarayan, Maheswar Satpathy, Monika Sawhney, Nikolaos Scarmeas, Benedikt Michael Schaarschmidt, Maria Inês Schmidt, Ione Jayce Ceola Schneider, Austin E Schumacher, David C Schwebel, Falk Schwendicke, Mansour Sedighi, Sabyasachi Senapati, Subramanian Senthilkumaran, Sadaf G Sepanlou, Yashendra Sethi, Soko Setoguchi, Allen Seylani, Jamileh Shadid, Mahan Shafie, Humaira Shah, Nilay S Shah, Pritik A Shah, Ataollah Shahbandi, Samiah Shahid, Wajeehah Shahid, Moyad Jamal Shahwan, Masood Ali Shaikh, Alireza Shakeri, Ali S Shalash, Sunder Sham, Muhammad Aaqib Shamim, Mohammad Ali Shamshirgaran, Mohammad Anas Shamsi, Mohd Shanawaz, Abhishek Shankar, Mohammed Shannawaz, Medha Sharath, Amin Sharifan, Javad Sharifi-Rad, Manoj Sharma, Rajesh Sharma, Saurab Sharma, Ujjawal Sharma, Vishal Sharma, Rajesh P Shastry, Amin Shavandi, Amir Mehdi Shayan, Maryam Shayan, Amr Mohamed Elsayed Shehabeldine, Pavanchand H Shetty, Kenji Shibuya, Jemal Ebrahim Shifa, Desalegn Shiferaw, Wondimeneh Shibabaw Shiferaw, Mika Shigematsu, Rahman Shiri, Nebiyu Aniley Shitaye, Aminu Shittu, K M Shivakumar, Velizar Shivarov, Zahra Shokati Eshkiki, Sina Shool, Sunil Shrestha, Kerem Shuval, Migbar Mekonnen Sibhat, Emmanuel Edwar Siddig, Inga Dora Sigfusdottir, Diego Augusto Santos Silva, João Pedro Silva, Luís Manuel Lopes Rodrigues Silva, Soraia Silva, Colin R Simpson, Anjali Singal, Abhinav Singh, Balbir Bagicha Singh, Harmanjit Singh, Jasvinder A Singh, Mahendra Singh, Paramdeep Singh, Søren T Skou, David A Sleet, Erica Leigh N Slepak, Ranjan Solanki, Sameh S M Soliman, Suhang Song, Yimeng Song, Reed J D Sorensen, Joan B Soriano, Ireneous N Soyiri, Michael Spartalis, Chandrashekhar T Sreeramareddy, Benjamin A Stark, Antonina V Starodubova, Caroline Stein, Dan J Stein, Caitlyn Steiner, Timothy J Steiner, Jaimie D Steinmetz, Paschalis Steiropoulos, Leo Stockfelt, Mark A Stokes, Narayan Subedi Subedi, Vetriselvan Subramaniyan, Claudia Kimie Suemoto, Muhammad Suleman, Rizwan Suliankatchi Abdulkader, Abida Sultana, Johan Sundström, Chandan Kumar Swain, Lukasz Szarpak, Payam Tabaee Damavandi, Rafael Tabarés-Seisdedos, Ozra Tabatabaei Malazy, Seyed-Amir Tabatabaeizadeh, Shima Tabatabai, Celine Tabche, Mohammad Tabish, Santosh Kumar Tadakamadla, Yasaman Taheri Abkenar, Moslem Taheri Soodejani, Amir Taherkhani, Jabeen Taiba, Iman M Talaat, Ashis Talukder, Mircea Tampa, Jacques Lukenze Tamuzi, Ker-Kan Tan, Sarmila Tandukar, Haosu Tang, Razieh Tavakoli Oliaee, Seyed Mohammad Tavangar, Mojtaba Teimoori, Mohamad-Hani Temsah, Masayuki Teramoto, Pugazhenthan Thangaraju, Kavumpurathu Raman Thankappan, Rekha Thapar, Rasiah Thayakaran, Sathish Thirunavukkarasu, Nihal Thomas, Nikhil Kenny Thomas, Chern Choong Chern Thum, Ales Tichopad, Jansje Henny Vera Ticoalu, Tala Tillawi, Tenaw Yimer Tiruye, Ruoyan Tobe-Gai, Marcello Tonelli, Roman Topor-Madry, Anna E Torre, Mathilde Touvier, Marcos Roberto Tovani-Palone, Jasmine T Tran, Mai Thi Ngoc Tran, Nghia Minh Tran, Ngoc-Ha Tran, Domenico Trico, Samuel Joseph Tromans, Thien Tan Tri Tai Truyen, Aristidis Tsatsakis, Guesh Mebrahtom Tsegay, Evangelia Eirini Tsermpini, Munkhtuya Tumurkhuu, Stefanos Tyrovolas, Arit Udoh, Muhammad Umair, Srikanth Umakanthan, Tungki Pratama Umar, Eduardo A Undurraga, Brigid Unim, Bhaskaran Unnikrishnan, Carolyn Anne Unsworth, Era Upadhyay, Daniele Urso, Jibrin Sammani Usman, Seyed Mohammad Vahabi, Asokan Govindaraj Vaithinathan, Jef Van den Eynde, Orsolya Varga, Ravi Prasad Varma, Priya Vart, Tommi Juhani Vasankari, Milena Vasic, Siavash Vaziri, Balachandar Vellingiri, Narayanaswamy Venketasubramanian, Massimiliano Veroux, Georgios-Ioannis Verras, Dominique Vervoort, Jorge Hugo Villafañe, Francesco S Violante, Vasily Vlassov, Stein Emil Vollset, Simona Ruxandra Volovat, Avina Vongpradith, Yasir Waheed, Cong Wang, Fang Wang, Ning Wang, Shu Wang, Yanzhong Wang, Yuan-Pang Wang, Paul Ward, Emebet Gashaw Wassie, Marcia R Weaver, Kosala Gayan Weerakoon, Robert G Weintraub, Daniel J Weiss, Abrha Hailay Weldemariam, Katherine M Wells, Yi Feng Wen, Joanna L Whisnant, Harvey A Whiteford, Taweewat Wiangkham, Dakshitha Praneeth Wickramasinghe, Nuwan Darshana Wickramasinghe, Angga Wilandika, Caroline Wilkerson, Peter Willeit, Anders Wimo, Demewoz H Woldegebreal, Axel Walter Wolf, Yen Jun Wong, Anthony D Woolf, Chenkai Wu, Felicia Wu, Xinsheng Wu, Zenghong Wu, Sarah Wulf Hanson, Yanjie Xia, Hong Xiao, Xiaoyue Xu, Yvonne Yiru Xu, Lalit Yadav, Ali Yadollahpour, Sajad Yaghoubi, Kazumasa Yamagishi, Lin Yang, Yuichiro Yano, Yao Yao, Habib Yaribeygi, Mohammad Hosein Yazdanpanah, Pengpeng Ye, Sisay Shewasinad Yehualashet, Subah Abderehim Yesuf, Saber Yezli, Arzu Yiğit, Vahit Yiğit, Zeamanuel Anteneh Yigzaw, Yazachew Yismaw, Dong Keon Yon, Naohiro Yonemoto, Mustafa Z Younis, Chuanhua Yu, Yong Yu, Hadiza Yusuf, Mondal Hasan Zahid, Fathiah Zakham, Leila Zaki, Nazar Zaki, Burhan Abdullah Zaman, Nelson Zamora, Ramin Zand, Ghazal G Z Zandieh, Heather J Zar, Armin Zarrintan, Mikhail Sergeevich Zastrozhin, Haijun Zhang, Ning Zhang, Yunquan Zhang, Hanqing Zhao, Chenwen Zhong, Panliang Zhong, Juexiao Zhou, Zhaohua Zhu, Makan Ziafati, Magdalena Zielińska, Stephanie R M Zimsen, Mohammad Zoladl, Alimuddin Zumla, Samer H Zyoud, Theo Vos, Christopher J L Murray

## Abstract

**Background:**

Detailed, comprehensive, and timely reporting on population health by underlying causes of disability and premature death is crucial to understanding and responding to complex patterns of disease and injury burden over time and across age groups, sexes, and locations. The availability of disease burden estimates can promote evidence-based interventions that enable public health researchers, policy makers, and other professionals to implement strategies that can mitigate diseases. It can also facilitate more rigorous monitoring of progress towards national and international health targets, such as the Sustainable Development Goals. For three decades, the Global Burden of Diseases, Injuries, and Risk Factors Study (GBD) has filled that need. A global network of collaborators contributed to the production of GBD 2021 by providing, reviewing, and analysing all available data. GBD estimates are updated routinely with additional data and refined analytical methods. GBD 2021 presents, for the first time, estimates of health loss due to the COVID-19 pandemic.

**Methods:**

The GBD 2021 disease and injury burden analysis estimated years lived with disability (YLDs), years of life lost (YLLs), disability-adjusted life-years (DALYs), and healthy life expectancy (HALE) for 371 diseases and injuries using 100 983 data sources. Data were extracted from vital registration systems, verbal autopsies, censuses, household surveys, disease-specific registries, health service contact data, and other sources. YLDs were calculated by multiplying cause-age-sex-location-year-specific prevalence of sequelae by their respective disability weights, for each disease and injury. YLLs were calculated by multiplying cause-age-sex-location-year-specific deaths by the standard life expectancy at the age that death occurred. DALYs were calculated by summing YLDs and YLLs. HALE estimates were produced using YLDs per capita and age-specific mortality rates by location, age, sex, year, and cause. 95% uncertainty intervals (UIs) were generated for all final estimates as the 2·5th and 97·5th percentiles values of 500 draws. Uncertainty was propagated at each step of the estimation process. Counts and age-standardised rates were calculated globally, for seven super-regions, 21 regions, 204 countries and territories (including 21 countries with subnational locations), and 811 subnational locations, from 1990 to 2021. Here we report data for 2010 to 2021 to highlight trends in disease burden over the past decade and through the first 2 years of the COVID-19 pandemic.

**Findings:**

Global DALYs increased from 2·63 billion (95% UI 2·44–2·85) in 2010 to 2·88 billion (2·64–3·15) in 2021 for all causes combined. Much of this increase in the number of DALYs was due to population growth and ageing, as indicated by a decrease in global age-standardised all-cause DALY rates of 14·2% (95% UI 10·7–17·3) between 2010 and 2019. Notably, however, this decrease in rates reversed during the first 2 years of the COVID-19 pandemic, with increases in global age-standardised all-cause DALY rates since 2019 of 4·1% (1·8–6·3) in 2020 and 7·2% (4·7–10·0) in 2021. In 2021, COVID-19 was the leading cause of DALYs globally (212·0 million [198·0–234·5] DALYs), followed by ischaemic heart disease (188·3 million [176·7–198·3]), neonatal disorders (186·3 million [162·3–214·9]), and stroke (160·4 million [148·0–171·7]). However, notable health gains were seen among other leading communicable, maternal, neonatal, and nutritional (CMNN) diseases. Globally between 2010 and 2021, the age-standardised DALY rates for HIV/AIDS decreased by 47·8% (43·3–51·7) and for diarrhoeal diseases decreased by 47·0% (39·9–52·9). Non-communicable diseases contributed 1·73 billion (95% UI 1·54–1·94) DALYs in 2021, with a decrease in age-standardised DALY rates since 2010 of 6·4% (95% UI 3·5–9·5). Between 2010 and 2021, among the 25 leading Level 3 causes, age-standardised DALY rates increased most substantially for anxiety disorders (16·7% [14·0–19·8]), depressive disorders (16·4% [11·9–21·3]), and diabetes (14·0% [10·0–17·4]). Age-standardised DALY rates due to injuries decreased globally by 24·0% (20·7–27·2) between 2010 and 2021, although improvements were not uniform across locations, ages, and sexes. Globally, HALE at birth improved slightly, from 61·3 years (58·6–63·6) in 2010 to 62·2 years (59·4–64·7) in 2021. However, despite this overall increase, HALE decreased by 2·2% (1·6–2·9) between 2019 and 2021.

**Interpretation:**

Putting the COVID-19 pandemic in the context of a mutually exclusive and collectively exhaustive list of causes of health loss is crucial to understanding its impact and ensuring that health funding and policy address needs at both local and global levels through cost-effective and evidence-based interventions. A global epidemiological transition remains underway. Our findings suggest that prioritising non-communicable disease prevention and treatment policies, as well as strengthening health systems, continues to be crucially important. The progress on reducing the burden of CMNN diseases must not stall; although global trends are improving, the burden of CMNN diseases remains unacceptably high. Evidence-based interventions will help save the lives of young children and mothers and improve the overall health and economic conditions of societies across the world. Governments and multilateral organisations should prioritise pandemic preparedness planning alongside efforts to reduce the burden of diseases and injuries that will strain resources in the coming decades.

**Funding:**

Bill & Melinda Gates Foundation.

## Introduction

Comprehensive estimates of the global burden of disease have a crucial role in improving understanding of the impact of diseases and injuries on population health and assessing progress towards international health targets. Since the early 1990s, the Global Burden of Diseases, Injuries, and Risk Factors Study (GBD) has systematically and comprehensively produced estimates of global health and health loss across stratified age groups, locations, and sexes.^1^, ^2^ In 2020, the COVID-19 pandemic shifted global health priorities to control transmission of SARS-CoV-2 and respond to added demands on health services. Since then, COVID-19 has transitioned from being a new threat requiring emergency response, to an infectious disease that populations need to live with and manage. Within this context, systematic and up-to-date analysis of disease burden by cause, age, sex, location, and year assumes an even more important function. The results generated by such analyses provide the evidence base on which the emergence of COVID-19 can be better understood, as well as the altered burden of disease landscape that ensued in 2020 and 2021 after the novel coronavirus circulated widely.

**Research in context**

**Evidence before this study**

The Global Burden of Diseases, Injuries, and Risk Factors Study (GBD) 2021 is a comprehensive study of global health loss. GBD 2021 provides current information on the distribution and burden of diseases and injuries across time, age, sex, location, and sociodemographic group. GBD quantifies burden using disability-adjusted life-years (DALYs), a metric introduced in the World Bank's 1993 World Development Report and since adopted as a key measure of disease burden by WHO, the UN, the World Bank, and governmental agencies around the world. The previous GBD cycle, GBD 2019, estimated prevalence, incidence, and burden as years lived with disability, years of life lost to premature mortality, DALYs, and healthy life expectancy for 369 diseases and injuries in 204 countries and territories, from 1990 to 2019, across age groups and by sex. While other groups have reported on DALYs and other population health metrics in recent years, including the WHO World Health Statistics 2020 report and national-level burden of disease studies, GBD remains the most comprehensive disease burden research effort to date.

**Added value of this study**

GBD 2021 includes 19 189 new data sources for DALYs, 12 new causes, and other important methodological updates. Notably, we disaggregated the under-5 age group, providing more detailed age-based estimates of burden for young children. GBD 2021 reports ten non-COVID-19-related causes for the first time: pulmonary arterial hypertension, hepatoblastoma, Burkitt lymphoma, other non-Hodgkin lymphoma, eye cancer (including retinoblastoma and other eye cancers as distinct causes), soft tissue and other extraosseous sarcomas, malignant neoplasm of bone and articular cartilage, neuroblastoma, and other peripheral nervous cell tumours. Another important advancement of GBD 2021 is the addition of the impact of the COVID-19 pandemic on disease burden. GBD 2021 includes the estimation of burden for COVID-19, post-COVID-19 condition (also known as long COVID), other pandemic-related outcomes, and the effect of COVID-19 on other selected diseases and injuries. By modelling the burden of COVID-19 and related outcomes within a mutually exclusive and collective exhaustive cause hierarchy, this Article provides both policy makers and practitioners with scientific and evidence-based insights to help guide decision making, priority setting, and resource allocation from subnational to global levels. Policy makers benefit from robust evidence to formulate effective policies, while practitioners gain actionable insights for programme design and implementation. This report's global perspective aids in coordinated resource allocation, fostering collaborative efforts to address interconnected health challenges efficiently, such that it provides a comprehensive toolkit to inform and enhance decision-making processes across various levels of governance and practice.

**Implications of all the available evidence**

Timely and comprehensive estimates of disease burden are crucial for making health-related policy decisions. At the global level, in this study we found that the burden of diseases and injuries shifted in 2021 compared with previous years, with COVID-19 imposing additional health loss as the leading cause of burden in 2021. Neonatal disorders, ischaemic heart disease, and stroke continued to be among the leading causes of DALYs globally in 2021, as in every year of the previous decade. These findings highlight the importance of continuing to prioritise non-communicable disease prevention and treatment policies, along with health system improvements and ongoing COVID-19 vaccination and other transmission-prevention efforts.

GBD 2021 highlights new and existing health threats that require prioritisation on international public health agendas. GBD 2021 estimates will allow up to date identification of health disparities within and between populations, enabling evaluation of how these have changed over time, quantify health gains, and, in turn, identify policies or interventions that present the most promising opportunities for impact in the post-COVID-19 era. GBD 2021 estimates are given for 371 diseases and injuries (including 95 communicable, maternal, neonatal, and nutritional [CMNN] diseases, 234 non-communicable diseases, and 40 injuries); 204 countries and territories; 21 countries with subnational locations; 25 age groups; females, males, and both sexes combined; and for the years 1990–2021. Here, we report prevalence, incidence, years lived with disability (YLDs; quantifying non-fatal health loss), years of life lost (YLLs; quantifying fatal health loss), disability-adjusted life-years (DALYs; quantifying both years lost to premature mortality and years lived with disability), and healthy life expectancy (HALE; quantifying expected years of life lived in good health). We focused on estimates between 2010 and 2021 to highlight trends in disease burden over the past decade and in 2020 and 2021, the first 2 years of the COVID-19 pandemic. In this manuscript we provide high-level findings of GBD 2021. The estimation of causes of deaths and YLLs for GBD 2021 are summarised elsewhere.^3^

There have been key data and methodological updates for GBD 2021, which are summarised in the Methods section. Notably, to our knowledge, GBD 2021 is the first publication to provide comprehensive burden estimates for COVID-19 as an infectious disease inclusive of post-COVID-19 condition (also known as long COVID; disability caused by remaining symptoms after initial SARS-CoV-2 infection has been cleared^4^), other COVID-19 pandemic-related outcomes, and the effect of COVID-19 on the burden of selected diseases. GBD 2021 findings therefore supersede those from GBD 2019 and all previous GBD rounds. This manscript was produced as part of the GBD Collaborator Network and in accordance with the GBD Protocol.^5^

## Methods

### Overview

In this section, we provide an overview of the burden estimation process for GBD 2021, with further details provided in appendix 1. Methods used to generate burden estimates closely followed those for GBD 2019; however, here we summarise new or updated processes since GBD 2019. Detailed descriptions of analytical methods and models for each cause of disease burden are also available in a searchable online tool. Here we report GBD 2021 estimates of incidence, point prevalence (hereafter referred to as prevalence), YLDs, YLLs, and DALYs for 371 diseases and injuries along with estimates of HALE. Estimates were produced by sex (female and male) and age (25 age groups from birth to age 95 years and older) for 204 countries and territories, including subnational estimates for 21 countries and territories. GBD 2021 provides annual estimates between 1990 and 2021; however, in this report we have focused on estimates between 2010 to 2021 to highlight trends over the past decade and the first 2 years of the COVID-19 pandemic. We also present these metrics stratified by Socio-demographic Index (SDI), a composite measure of lag-distributed income per capita, average years of education for those aged 15 years or older, and fertility rates among females younger than 25 years (appendix 1 section 4).

An international network of over 10 000 collaborators from more than 150 countries and territories provided, reviewed, or analysed the available data to generate GBD metrics. GBD 2021 complies with the Guidelines for Accurate and Transparent Health Estimates Reporting (GATHER);^6^ a completed GATHER checklist is provided in appendix 1 (table S3). Analyses were completed using Python (version 3.10.4), Stata (version 13.1), and R (version 4.2.1).

### Geographical hierarchy

GBD 2021 produced estimates for 204 countries and territories, grouped into 21 regions and seven super-regions. GBD regions, and in turn GBD super-regions, are made up of countries and territories that are geographically close, epidemiologically similar, and share similar distributions of causes of death. Subnational estimates were produced for the following 21 countries and territories: Brazil, China, Ethiopia, India, Indonesia, Italy, Iran, Japan, Kenya, Mexico, New Zealand, Nigeria, Norway, Pakistan, the Philippines, Poland, Russia, South Africa, Sweden, the UK, and the USA. Subnational analyses were conducted at the first level of administrative organisation within each country, except for New Zealand (which was conducted by Māori ethnicity), Sweden (by Stockholm and non-Stockholm), the UK (by local government authorities), Kenya (by county), and the Philippines (by province). Since GBD 2019, the GBD location hierarchy has included all WHO Member States. At the most detailed level, we generated estimates for 983 specific locations. A full list of the geographical hierarchy is in appendix 1 (table S1).

### Disease and injury hierarchy

The 371 diseases and injuries included in GBD 2021 were organised within a cause hierarchy with four levels plus the Level 0 aggregate of all causes. Level 1 consisted of other COVID-19 pandemic-related outcomes and three broad aggregate categories: CMNN diseases; non-communicable diseases; and injuries. Level 2 included 22 clusters of causes that each fell within a Level 1 category. Level 3 included 175 causes of which 132 were specific causes and 43 were clusters of Level 4 causes. Level 4 consisted of 302 specific causes, including 170 specific causes that each fell within the 43 Level 3 clusters of causes and the 132 Level 3 specific causes that were not further disaggregated at Level 4. Overall, 365 causes had non-fatal outcomes, and 288 causes had fatal outcomes. A full list of causes by level is in appendix 1 (table S2).

### Data sources

DALY estimates for GBD 2021 were informed by 100 983 data sources (including 19 189 sources newly used in 2021). Included among these data sources were 75 459 data sources on non-fatal causes, among which 36 916 were data sources on incidence, 22 236 were data sources on prevalence, and 45 were data sources on other epidemiological measures (eg, remission). Information on data sources for YLLs for GBD 2021 have been reported elsewhere.^3^ For non-fatal estimates, data sources included scientific literature, household survey data, epidemiological surveillance data, disease registry data, clinical informatics data, and other sources (appendix 1 section 3.1.1). Cause-specific literature reviews are described in detail in appendix 1 (section 8) and include searches of online research databases, government and international organisation websites, published reports, primary data sources, and contributions of datasets by GBD Collaborators. Multiple data types were included to capture the widest array of epidemiological information pertaining to a cause. Fatal estimates, methods, and data sources are discussed in full detail elsewhere.^3^ Data sources were identified from vital registration, verbal autopsy, registry, survey, police, or surveillance data across all countries and territories. As with any given release of GBD, we sought to incorporate as much available data from around the world as possible into our estimates. We receive data not only through literature reviews, but also through proactive data seeking and through the work of collaborators in our network to identify new datasets and sources.

### Prevalence, incidence, and YLDs

YLDs were calculated with a microsimulation process that used estimated age-sex-location-year-specific prevalent counts of non-fatal disease sequelae (consequences of a disease or injury) for each cause and disability weights for each sequela as the inputs.

#### Data processing

Epidemiological data with known biases (eg, alternative case definitions or measurement methods) were adjusted with correction factors estimated by network meta-regressions using MR-BRT (meta-regression—Bayesian, regularised, trimmed; appendix 1 section 3.3).^7^ MR-BRT encompasses a series of statistical models—namely, linear and non-linear mixed effects models—and fitting procedures (appendix 1 section 3.5). Input data to estimate correction factors consisted of pairs of estimates where two case definitions or measurement methods were available for the same age-sex-location-year. For example, the correction factor used to adjust past-year prevalence estimates of anxiety disorders down to reflect point prevalence (the gold-standard recall period for this cause) was informed by available past-year prevalence data matched with point prevalence data on age, sex, location, and year (cause-specific corrections applied to the data using MR-BRT are in appendix 1 [section 8]). Input data not reported by sex were adjusted using the cause-specific pooled within-study sex ratio. Input data not reported by age and sex underwent age-sex splitting. Age-specific estimates from sources reporting by age and by sex (but not by age-sex) were adjusted using the within-source sex ratio to provide age-sex-specific estimates. Input data representing an age span greater than 25 years were split into granular age-specific estimates via an alternative age pattern that was estimated with available data from other sources (appendix 1 section 3.3.5).

Clinical informatics data also underwent various forms of data processing, as detailed in appendix 1 (section 3.2). Clinical sources included data on inpatient hospital admissions, outpatient visits (including to general practitioners), and health insurance claims. Data from inpatient hospital admissions reporting a single diagnosis were adjusted to account for readmissions, non-primary diagnoses, and outpatient care. The fraction of unique inpatient cases and ratios between primary and non-primary diagnoses, and inpatient and outpatient care, were extracted from data sources containing this level of detail at the individual level. Age-sex-specific ratios were then estimated by cause using MR-BRT, and inpatient data were adjusted accordingly. Estimates of total inpatient admission rates per capita for each location, year, age, and sex were used to scale inpatient sources that were incomplete for the population. Inpatient sources were additionally transformed using a scalar derived from the Healthcare Access and Quality Index to account for varying health-care access by location.^8^ These adjustments aim to produce standardised, population-level clinical estimates of incidence and prevalence.

#### Statistical analyses of prevalence estimates

For most diseases and injuries, prevalence and incidence were modelled using DisMod-MR 2.1 (Disease Modelling Meta-Regression; version 2.1). DisMod-MR 2.1 is a Bayesian disease modelling meta-regression tool that generates internally consistent estimates of prevalence, incidence, remission, and mortality by sex, location, year, and age group. The tool also produced these measures for locations with missing raw epidemiological data by estimating prevalence across a cascade down the five levels of the GBD geographical hierarchy. Epidemiological data from locations higher in the hierarchy served as priors for the estimation of the epidemiological parameter from locations lower in the hierarchy. DisMod-MR 2.1 also uses location-level covariates to inform prevalence and incidence for locations with missing data. More detail on DisMod-MR 2.1 is in appendix 1 (section 3.6) and has been published elsewhere.^9^ For some causes, spatiotemporal Gaussian process regression (ST-GPR) disease models were used instead of DisMod-MR 2.1. ST-GPR is a set of regression methods that allow us to analyse heterogeneous and incomplete data requiring statistical smoothing over time, age, and location. More detail on ST-GPR is in appendix 1 (section 3.4). Custom models were used for causes where DisMod-MR 2.1 or ST-GPR could not adequately model prevalence or incidence. Details about cause-specific methods are in appendix 1 (section 8).

#### Severity distribution and disability weights

Prevalence and incidence were split into sequela-specific prevalence and incidence for non-fatal causes for which disability varied across a spectrum of severity. Sequela categories varied by non-fatal cause—for instance, categories could represent asymptomatic, mild, moderate, and severe sequelae. Estimates of the proportion of cases in each sequela category for most non-fatal causes were calculated via analysis of the Medical Expenditure Panel Survey (known as MEPS; appendix 1 section 3.8).^10^ Severity proportions for remaining non-fatal causes were estimated from an analysis of the US National Epidemiologic Survey on Alcohol and Related Conditions,^11^, ^12^ the 1997 Australian National Survey of Mental Health and Wellbeing,^13^ or from published survey data sourced from literature reviews. Details on severity proportions by cause are in appendix 1 (section 8). Crude YLD rates were estimated by multiplying sequela-specific prevalence by their respective disability weights. Disability weights represent health loss from a sequela on a scale ranging from 0 (equivalent to no health loss) to 1 (equivalent to death). Disability weights were derived from community surveys of the general population from Bangladesh, Hungary, Indonesia, Italy, Peru, Sweden, Tanzania, the Netherlands, and the USA, and an open internet survey available in English, Spanish, and Mandarin.^14^, ^15^ Participants of the surveys were presented pairs of lay descriptions of health states and asked which of the two was the healthier state. Lay descriptions were created by experts, used non-clinical language, and were 35 words or shorter in length. Responses were then anchored between 0 and 1 via population health equivalence questions where respondents were asked to compare the benefit of lifesaving or disease-prevention programmes. More detail on the estimation of disability weights is provided elsewhere^14^, ^15^ and in appendix 1 (section 3.9).

#### Comorbidity

YLDs underwent a comorbidity correction to account for the co-occurrence of non-fatal causes in the population and to allow for YLDs to be additive across the GBD 2021 cause hierarchy. The co-occurrence of non-fatal causes was simulated within a population of 20  000 simulated individuals for every age, sex, location, and year. The probability of each simulated individual having each sequela was equal to its prevalence. Each simulated individual was then assigned a cumulative disability weight on the basis of a multiplicative function of all the disability weights assigned to the simulated individual. Sequela-specific disability weights were adjusted accordingly and the YLD rate for each sequela was calculated. YLD counts were then estimated as the YLD rate multiplied by the age-sex-location-year-specific population. More detail on this comorbidity simulation is in appendix 1 (section 3.10).

#### New for GBD 2021

For GBD 2021, we further disaggregated all estimates for age groups younger than 5 years into those aged 0–6 days, 7–27 days, 1–5 months, 6–11 months, 12–23 months, and 2–4 years. The relatively broad grouping of 1–4-year-old children in previous iterations of the GBD study limited our ability to make targeted recommendations regarding the burden from neonatal disorders given that the risk factors, health services, supportive equipment needs, and caregiver needs vary considerably within this age group. GBD 2021 reports two new COVID-19-related causes: COVID-19 at Level 3 (including the burden due to long COVID, modelled as a sequela for COVID-19), and other COVID-19 pandemic-related outcomes at Level 1 (appendix 1 table S2). Other COVID-19 pandemic-related outcomes included excess mortality associated with the pandemic minus mortality directly due to COVID-19, lower respiratory infections, measles, malaria, and pertussis, because there was a reduction in deaths from these causes during the pandemic^3^ and, hence, we removed that effect on mortality first before calculating other pandemic-related deaths. For the first time, GBD 2021 also reports on five additional Level 3 causes (pulmonary arterial hypertension, eye cancer, soft tissue and other extraosseous sarcomas, malignant neoplasm of bone and articular cartilage, and neuroblastoma and other peripheral nervous cell tumours), and five additional Level 4 causes (hepatoblastoma, Burkitt lymphoma, other non-Hodgkin lymphoma, retinoblastoma, and other eye cancers; appendix 1 table S2). The decision to model and report new causes is manifold and considers such factors as data availability, policy concerns, research priorities, and methodological refinements. Processes used to incorporate these causes into GBD 2021 are in appendix 1 (section 8). Additionally, the prevalence estimates for major depressive disorder and anxiety disorders were adjusted for the impact of the COVID-19 pandemic using data sourced from a systematic review that informed a meta-regression in MR-BRT. This model was then used to adjust pre-pandemic prevalence of these two disorders estimated via DisMod-MR 2.1 (appendix 1 section 8).^16^

### Deaths and YLLs

YLLs were calculated as the product of estimated age-sex-location-year-specific deaths and the standard life expectancy at the age death occurred for a given cause. This process is summarised here, with detailed information provided in affiliated GBD 2021 publications featuring causes of death and YLLs,^3^ as well as updated demographic parameters.^17^

#### Data processing

GBD 2021 methods follow principles from the ICD, 11th edition, whereby each death is assigned to the underlying cause that initiated events leading to death. Vital registration data reporting assigned ICD so-called garbage codes (a term used in GBD to represent non-specific codes, implausible codes, or intermediate rather than underlying cause of death codes) were assigned to the most likely cause of death via redistribution algorithms.^18^ These algorithms were derived from published studies, expert consultation, or regression on data sources reporting multiple causes of death.^3^

#### Statistical analyses of mortality estimates

Cause of death estimates for most diseases and injuries were modelled via the Cause of Death Ensemble model (CODEm). CODEm uses an ensemble of statistical models while also systematically testing combinations of covariates on the basis of their out-of-sample predictive validity. It then combines results to estimate deaths by location, age, sex, and year for a given cause. CODEm was run by sex and separately for countries and territories with and without extensive complete vital registration data to decrease the likelihood of uncertainty inflation from data with high heterogeneity. Multiple iterations of out-of-sample predictive validity for each model were assessed, and models with the smallest root-mean-square error were weighted to generate an ensemble model for a given cause. Other customised modelling strategies were used to estimate deaths for a small group of causes with unique epidemiology, important changes in reporting practices, or a scarcity of data. These modelling strategies included the use of prevalence, incidence, case-fatality data, or data related to sub-causes to inform cause of death estimates; further discussions of these methods are available elsewhere.^3^

### DALYs and HALE

YLDs and YLLs were summed to calculate DALYs by location, age, sex, year, and cause (appendix 1 section 5). HALE was estimated as a complementary measure to DALYs, representing a population's average number of years of life spent in good health. HALE values were calculated using age-specific mortality rates and YLDs per capita. The method used to estimate HALE was unchanged from previous GBD cycles.^19^ The method was presented by Sullivan,^20^ and is described in appendix 1 (section 6). Both DALYs and HALE were estimated by location, age, sex, and year, and DALYs also by cause.

### Data presentation, annual rate of change, uncertainty, and SDI

GBD 2021 metrics were estimated as counts, all-age and age-specific rates per 100 000 population, and age-standardised rates per 100 000 population, calculated using the GBD standard population structure. We present percentage changes over specified time periods (eg, 2010–21), and annualised rates of change as the difference in the natural log of the values at the start and end of the time interval divided by the number of years in the interval. All calculations were conducted 500 times to generate draw-level estimates. The number of computations per process was reduced from 1000, as in previous GBD iterations, to 500 for GBD 2021 because simulation testing revealed the final estimates and their uncertainty were not affected by this reduction. Final estimates represent the mean estimate across 500 draws, and 95% uncertainty intervals (UIs) are represented by the 2·5th and 97·5th percentile values across the draws. Uncertainty was propagated at each step in the estimation process.

Sociodemographic development has been a leading contributor to health gains over the three decades for which GBD has previously tracked changes in burden by location.^21^ In this Article, we also present an analysis of burden for locations across SDI quintiles. SDI is a composite indicator representing the geometric mean of three parameters: the lag-distributed income per capita, average years of schooling, and the fertility rate in females younger than 25 years for a given location. SDI scores were rescaled from 0 (lowest income and years of schooling, and highest fertility) to 100 (highest income and years of schooling, and lowest fertility). This process is further explained in appendix 1 (section 4).

### Role of the funding source

The funder of this study had no role in study design, data collection, data analysis, data interpretation, or the writing of the report.

## Results

We generated results using GBD methods to analyse data between 2010 and 2021 to highlight trends in disease burden over the past decade and through the first 2 years of the COVID-19 pandemic (2020 and 2021). We present prevalence and incidence results for causes at Level 3 of the GBD cause hierarchy and DALY estimates for all causes combined and individual causes or cause groups at Levels 1–3 of the hierarchy. DALY estimates are presented at the global level, by SDI quintile, GBD regions, and countries and territories.

Estimates described in the Article are viewable in appendix 2. Detailed results are also available in searchable and downloadable form through the GBD Results tool and via visual exploration through the online tool GBD Compare. Summaries of results for each cause of disease burden included in the analysis are available online.

### Global trends in prevalence and incidence

The number of prevalent cases and age-standardised prevalence and their rank by cause and sex in 2010 and 2021 are presented in appendix 2 (tables S1–S6). Globally, the most prevalent Level 3 diseases and injuries during 2021 (across ages and sexes) were oral disorders (3·69 billion [95% UI 3·40–4·00] cases at any given point during the year), headache disorders (2·81 billion [2·60–3·03]), and haemoglobinopathies and haemolytic anaemias, which includes inherited blood disorders (2·19 billion [2·12–2·27]; appendix 2 table S4). These causes also had the highest age-standardised prevalence of all Level 3 causes (appendix 2 tables S1, S2, S3). There were only minimal changes among the 20 most prevalent causes since 2010.

The Level 3 causes with the highest incidence globally in 2021 (across ages and sexes) were upper respiratory infections (12·8 billion [95% UI 11·4–14·5] new cases), diarrhoeal diseases (4·67 billion [4·11–5·22]), and oral disorders (3·74 billion [3·31–4·22]). These three causes also had the highest incidence globally for all sexes combined since 2010. The number of incident cases and age-standardised incidence and rank by cause, age, and sex, for 2010 and 2021, are in appendix 2 (tables S7, S8, S9, S10, S11, S12).

There were 2·28 billion (95% UI 2·18–2·37) incident cases of COVID-19 globally in 2021, equivalent to an age-standardised incidence rate of 28 955·3 (27 708·3–30 143·2) per 100 000 population (appendix 2 tables S7, S10). This was an increase from 1·63 billion (1·55–1·69) incident cases of COVID-19 globally in 2020, equivalent to an age-standardised incidence rate of 20 819·8 (19 908·0–21 681·5) per 100 000 (appendix 2 table S7, S10). Although males and females had similar incidence rates of COVID-19 across years (appendix 2 tables S8, S9, S11, S12), females were affected by long COVID at twice the rate of males (63·2% [59·7–66·3] of people with long COVID were female and 36·8% [33·7–40·3] were male in 2020–21; sequela-level estimates not shown).

### Global trends in DALYs between 2010 and 2021

The global number of all-cause DALYs remained steady between 2010 (2·63 billion [95% UI 2·44–2·85]) and 2019 (2·61 billion [2·36–2·88]); however, global all-cause DALYs increased to 2·76 billion (2·50–3·04) in 2020 and to 2·88 billion (2·64–3·15) in 2021 (appendix 2 table S13). Yet, there was a decrease in the global age-standardised DALY rate per 100 000 population, which accounts for the impact of population growth and ageing, between 2010 (39 352·9 [36 554·8–42 641·1]) and 2019 (33 763·6 [30 685·4–37 225·2]), equivalent to a 14·2% (10·7–17·3) decrease in the global age-standardised all-cause burden (appendix 2 table S13). However, the global age-standardised DALY rate per 100 000 population increased between 2019 and 2020 by 4·1% (1·8–6·3), to 35 143·0 (32 052·1–38 678·8) in 2020, and by 7·2% (4·7–10·0) between 2019 and 2021, to 36 206·8 DALYs (33 039·5–39 638·7) in 2021 (appendix 2 table S13).

Across the Level 1 causes, non-communicable diseases contributed the highest burden globally and was the only disease group for which DALYs increased between 2010 and 2021, from 1·47 billion (95% UI 1·32–1·64) in 2010 to 1·73 billion (1·54–1·94) in 2021 (appendix 2 table S13). This increase was mostly due to population growth and ageing (165 of 204 countries increased in population between 2010 and 2021), because the age-standardised DALY rates decreased between 2010 and 2021 by 6·4% (3·5–9·5), from 22 209·8 (19 961·7–24 705·4) to 20 783·1 (18 495·1–23 377·4) DALYs per 100 000 population. DALYs for CMNN diseases decreased from 876 million (816–939) in 2010 to 831 million (761–909) in 2021. The age-standardised DALY rate for CMNN diseases decreased by 12·9% (5·1–19·2) between 2010 and 2021, from 13 062·8 (12 173·8–14 002·1) to 11 373·3 (10 360·3–12 517·3) DALYs per 100 000. DALYs due to injuries decreased from 284 million (266–305) in 2010 to 248 million (227–272) in 2021 (appendix 2 table S13). The age-standardised DALY rate due to injuries decreased by 24·0% (20·7–27·2) from 2010 to 2021, from 4080·1 (3821·4–4385·6) to 3098·9 (2837·2–3404·5) DALYs per 100 000. However, most of the health gains from injuries occurred between 2010 and 2019 (a decrease of 20·8% [17·7–23·6] in age-standardised DALY rate) as opposed to between 2019 and 2021 (a decrease of 4·1% [1·1–6·7]).

**Figure 1:**
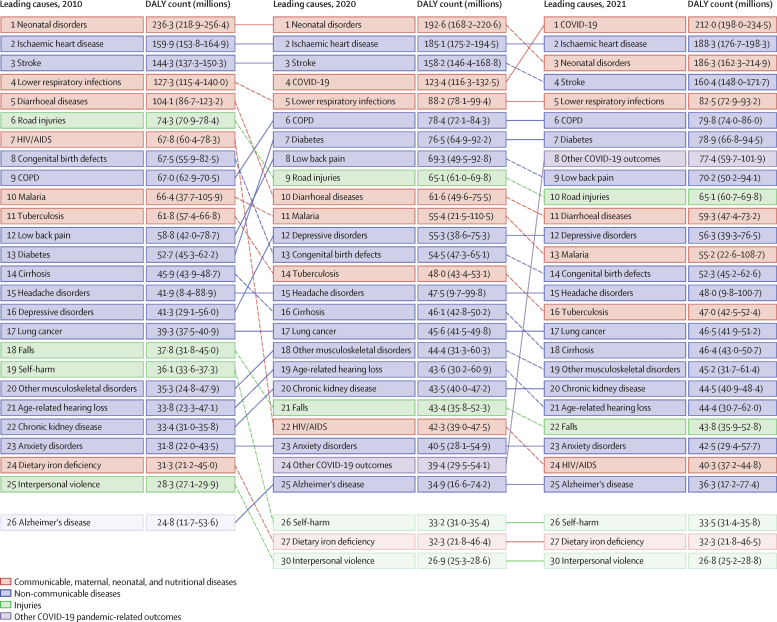
Leading 25 Level 3 causes of global DALYs in 2010, 2020, and 2021, for both sexes combined, and all ages Causes are connected by lines between time periods, where solid lines represent an increase or no change in rank and dashed lines represent a decrease in rank. Faded colours indicate that the cause is not within the top 25 causes of DALYs for that year. Data in parentheses are 95% uncertainty intervals. COPD=chronic obstructive pulmonary disease. DALY=disability-adjusted life-year.

In total, 15 non-communicable diseases, seven CMNN diseases, two types of injuries, and other COVID-19 pandemic-related outcomes featured within the 25 leading Level 3 causes of DALYs in 2021 (figure 1). Between 2010 and 2020, neonatal disorders, ischaemic heart disease, and stroke were the leading causes of DALYs for all ages and sexes combined. In 2020, COVID-19 emerged as the fourth leading cause of all-age DALYs. In 2021, it became the leading cause of DALYs, surpassing neonatal disorders, ischaemic heart disease, and stroke. Other COVID-19 pandemic-related outcomes was the 24th leading cause of DALYs in 2020 and the eighth leading cause in 2021. The emergence of both these causes in 2020 reversed the decline in DALYs due to CMNN diseases, causing an increase of 18·0% (95% UI 13·8–22·5) in age-standardised DALY rates between 2019 and 2021. However, there were substantial health gains among other leading CMNN diseases, most notably for HIV/AIDS (with a decrease in the age-standardised DALYs rate of 47·8% [43·3–51·7] between 2010 and 2021) and diarrhoeal diseases (decrease of 47·0% [39·9–52·9]). The smallest health gain among the CMNN diseases was observed for neonatal disorders (decrease in age-standardised DALY rate of 17·1% [4·9–25·9]), which was in the top three leading causes of DALYs in 69 of 204 countries and territories in 2021.

Among the non-communicable diseases featured in figure 1 as the leading Level 3 causes of DALYs in 2021, the largest health gains between 2010 and 2021 were observed for congenital birth defects (with a decrease in the age-standardised DALY rate of 20·9% [95% UI 2·7–30·4], cirrhosis and other chronic liver diseases (a decrease of 18·3% [10·9–26·5]), and stroke (a decrease of 16·9% [11·7–21·9]; appendix 2, table S25). However, age-standardised DALYs also increased by 14·0% (10·0–17·4) for diabetes, 16·4% (11·9–21·3) for depressive disorders, and 16·7% (14·0–19·8) for anxiety disorders. The two types of injury explaining the most burden globally in 2021 (figure 1) decreased in age-standardised DALY rates between 2010 and 2021. Between 2010 and 2021, age-standardised DALYs for road injuries decreased by 22·9% (18·5–27·1), from 1049·4 (1001·8–1108·3) per 100 000 in 2010 to 808·9 (752·7–865·8) per 100 000 in 2021 and falls decreased by 6·9% (4·0–10·2), from 570·9 (480·7–678·2) per 100 000 in 2010 to 531·2 (437·3–638·8) per 100 000 in 2021. However, most of this change occurred before 2019, when the rate was 839·9 (788·0–899·3) DALYs per 100 000 for road injuries and 536·7 (442·8–643·0) per 100 000 for falls. Age-standardised DALY rates and number of DALYs by cause and location for 2010, 2019, 2020, and 2021 are presented in appendix 2 table S13.

**Figure 2:**
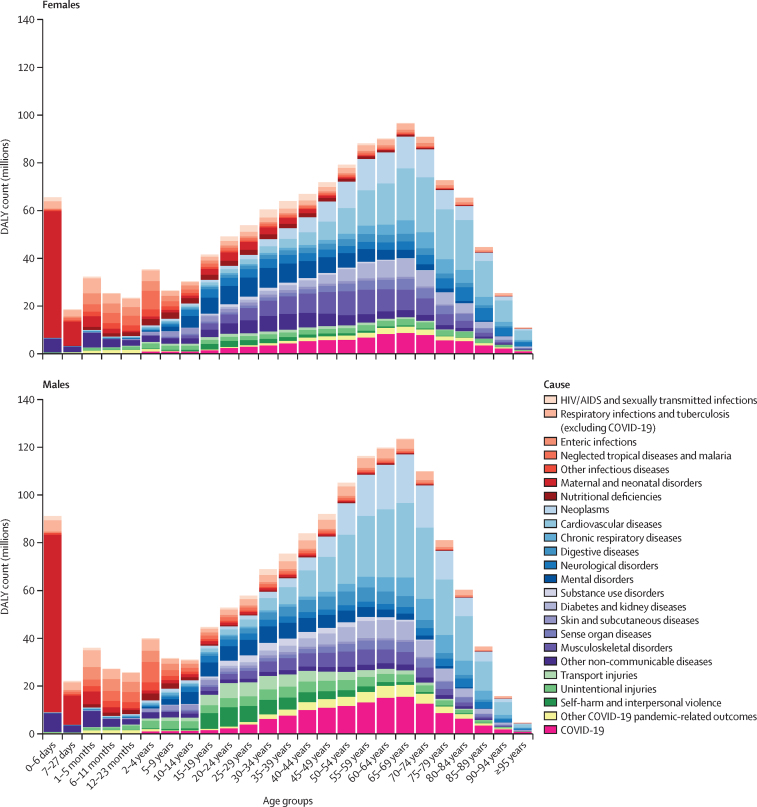
The distribution of global DALYs by age and sex for Level 2 causes, COVID-19, and other COVID-19 pandemic-related outcomes in 2021 DALY=disability-adjusted life-year.

Figure 2 shows the distribution of global DALYs across age groups for females and males in 2021 for Level 2 causes, COVID-19, and other COVID-19 pandemic-related outcomes (the equivalent plot for 2020 is in appendix 2 [figure S1]; age-specific rates for 2021 are in appendix 2 [figure S2]). In 2021, males accounted for 1·55 billion (95% UI 1·43–1·69) global DALYs and females for 1·33 billion (1·19–1·48) global DALYs. In females, COVID-19 was the leading Level 3 cause of DALYs in 2021 (80·2 million [73·1–92·5] DALYs) followed by neonatal disorders (79·9 million [70·3–91·0]; appendix 2 table S14). In males, COVID-19 was also the leading cause of DALYs (132 million [124–143] DALYs), followed by ischaemic heart disease (115 million [108–123]) and neonatal disorders (106 million [91·2–124]; appendix 2 table S15). Most of the burden due to neonatal disorders in 2021 occurred during the first 6 days after birth and burden imposed by neonatal conditions decreased sharply with age thereafter. Despite similar age-standardised incidence rates of COVID-19 between females and males, the age-standardised DALY rate for COVID-19 was greater among males (3247·9 [3057·9–3521·4] per 100 000 males; 8·5% [7·6–9·5] of male DALYs) than females (1822·6 [1651·0–2125·7] per 100 000 females; 6·1% [5·3–7·0] of female DALYs). Males also had a larger age-standardised rate of DALYs due to other COVID-19 pandemic-related outcomes (1220·4 [909·4–1630·9] DALYs per 100 000) than did females (705·2 [503·6–945·9] per 100 000; appendix 2 tables S14, S15). Older populations had greater health loss due to COVID-19 than did younger populations, with individuals aged 50–69 years having 5834·8 (5515·1–6302·5) DALYs per 100 000 and individuals aged 70 years and older having 11 584·1 (11 067·8–12 262·5) DALYs per 100 000 due to COVID-19 in 2021 (appendix 2 figure S2). This trend was also evident for other COVID-19 pandemic-related outcomes, which accounted for 1911·6 (1429·8–2677·6) DALYs per 100 000 population aged 50–69 years, and 3572·6 (2701·0–4791·8) per 100 000 population aged 70 years and older globally (appendix 2 figure S2).

### Decomposition of DALYs into YLDs and YLLs

Global all-cause DALYs in 2021 were composed of 907 million (95% UI 680–1170) YLDs (equivalent to 31·3% [25·4–37·5] of total DALYs and an age-standardised rate of 11 031·9 [8279·5–14 272·4] YLDs per 100 000; appendix 2 table S16) and 1·98 billion (1·87–2·10) YLLs (equivalent to 68·7% [62·5–74·6] of total DALYs and an age-standardised rate of 25 175·4 [23 693·7–26 906·6] YLLs per 100 000; appendix 2 table S19). Global YLDs increased from 738 million (550–959) in 2010 and YLLs increased from 1·89 billion (1·82–1·96) in 2010. All-cause, age-standardised YLD rates, which account for population growth and ageing, remained relatively stable between 2010 and 2021, with only a 2·6% (1·3–4·8) increase globally (appendix 2 table S16).

21 non-communicable diseases, three CMNN diseases, and one injury featured within the 25 leading Level 3 causes of YLDs globally in 2021 (table). The top three causes of YLDs, across all ages and sexes combined, were all non-communicable diseases: low back pain (70·2 million [95% UI 50·2–94·1] YLDs), depressive disorders (56·3 million [39·3–76·5] YLDs), and headache disorders (48·0 million [9·80–101] YLDs). Among the 25 leading Level 3 causes of YLDs, we observed the largest increase in age-standardised YLD rates between 2010 and 2021 in diabetes, anxiety disorders, depressive disorders, drug use disorders, and neonatal disorders (table). COVID-19, which was the leading contributor to the increase in DALYs in 2021 (figure 1), did not feature as prominently as a leading cause of YLDs, ranking as the 19th leading Level 3 cause of YLDs in 2021 (14·3 million [5·14–33·7] YLDs; table), up from 41st in 2020 (4·95 million [1·79–11·4] YLDs; appendix 2 table S16).

**Table:** Top 25 leading Level 3 causes of global YLDs, ranked from 1 to 25, in 2021 across all ages, for both sexes combined, YLD counts, age-standardised rates, and percentage change between 2010 and 2021

	**Cause**	**YLDs 2021**			**Percentage change, 2010–21**	
		Percentage of all-cause YLDs	Count (millions)	Age-standardised rate (per 100 000)	YLD count	Age-standardised rate
1	Low back pain	7·7% (6·5 to 9·1)	70·2 (50·2 to 94·1)	832·2 (595·9 to 1115·3)	19·4% (17·9 to 20·8)	−2·4% (−2·9 to −1·8)
2	Depressive disorders	6·2% (5·0 to 7·8)	56·3 (39·3 to 76·5)	681·2 (475·3 to 924·0)	36·5% (31·7 to 41·7)	16·4% (11·9 to 21·3)
3	Headache disorders	5·2% (1·2 to 10·4)	48·0 (9·80 to 101)	588·4 (117·6 to 1245·7)	14·4% (12·7 to 17·8)	0·3% (−1·1 to 1·4)
4	Age-related and other hearing loss	4·9% (3·9 to 5·9)	44·4 (30·7 to 62·0)	525·9 (364·2 to 731·9)	31·6% (30·0 to 33·1)	2·5% (1·7 to 3·3)
5	Other musculoskeletal disorders	4·8% (3·6 to 6·2)	43·0 (29·6 to 59·2)	507·2 (349·5 to 698·0)	28·7% (26·7 to 30·8)	6·3% (5·6 to 7·0)
6	Anxiety disorders	4·7% (3·6 to 6·0)	42·5 (29·4 to 57·7)	524·3 (363·3 to 716·2)	33·7% (30·7 to 37·0)	16·7% (14·0 to 19·8)
7	Diabetes mellitus	4·5% (3·9 to 5·2)	41·2 (29·0 to 56·5)	477·0 (336·1 to 653·3)	62·9% (60·3 to 65·8)	25·9% (24·0 to 28·1)
8	Dietary iron deficiency	3·6% (2·8 to 4·2)	32·3 (21·8 to 46·5)	423·7 (285·3 to 611·0)	3·2% (0·9 to 5·2)	−7·3% (−9·4 to −5·5)
9	Blindness and vision loss	3·2% (2·4 to 4·4)	29·2 (19·0 to 42·9)	342·8 (224·2 to 503·6)	32·9% (27·4 to 38·6)	2·6% (−2·5 to 7·7)
10	Gynaecological diseases	3·0% (2·5 to 3·7)	27·4 (19·1 to 38·2)	334·0 (232·7 to 467·5)	15·3% (14·0 to 16·8)	1·4% (0·6 to 2·3)
11	Falls	2·7% (2·3 to 3·0)	24·2 (16·8 to 32·8)	288·6 (200·9 to 391·7)	21·4% (19·2 to 23·3)	−3·5% (−4·7 to −2·6)
12	Oral disorders	2·6% (1·7 to 3·6)	23·2 (13·8 to 35·0)	275·9 (164·2 to 416·8)	25·1% (21·4 to 28·6)	0·4% (−2·6 to 3·3)
13	Neonatal disorders	2·4% (2·0 to 2·8)	21·7 (15·7 to 27·3)	282·7 (204·9 to 356·1)	24·9% (13·8 to 35·3)	13·5% (3·6 to 22·9)
14	Osteoarthritis	2·3% (1·4 to 4·5)	21·3 (10·2 to 42·9)	244·5 (117·1 to 493·1)	36·9% (35·2 to 38·5)	2·4% (1·3 to 3·5)
15	Neck pain	2·2% (1·7 to 2·9)	20·4 (13·6 to 28·9)	242·3 (162·6 to 342·8)	22·0% (19·4 to 24·6)	1·4% (1·0 to 1·9)
16	Stroke	1·7% (1·3 to 2·0)	15·2 (11·0 to 19·4)	178·7 (128·9 to 227·6)	29·1% (25·4 to 32·8)	−1·2% (−4·0 to 1·5)
17	COPD	1·7% (1·3 to 2·1)	14·9 (12·4 to 17·1)	174·4 (145·5 to 201·0)	29·1% (24·8 to 33·3)	−2·5% (−5·7 to 0·5)
18	Schizophrenia	1·7% (1·2 to 2·2)	14·8 (10·9 to 19·1)	177·8 (131·6 to 228·9)	16·3% (14·9 to 17·8)	0·0% (−0·9 to 0·9)
19	COVID-19	1·6% (0·6 to 3·6)	14·3 (5·14 to 33·7)	176·4 (63·1 to 418·9)	..	..
20	Alzheimer's disease and other dementias	1·3% (1·0 to 1·6)	11·6 (7·96 to 15·3)	141·9 (97·7 to 187·2)	45·7% (44·1 to 47·2)	2·6% (1·7 to 3·5)
21	Autism spectrum disorders	1·3% (0·8 to 2·0)	11·5 (7·84 to 16·3)	147·6 (100·2 to 208·1)	12·9% (11·9 to 13·7)	0·7% (−0·2 to 1·4)
22	Alcohol use disorders	1·2% (1·0 to 1·5)	11·0 (7·68 to 15·3)	132·3 (92·3 to 184·5)	6·8% (3·4 to 10·5)	−8·1% (−10·8 to −5·4)
23	Asthma	1·1% (0·9 to 1·4)	10·2 (6·50 to 15·0)	131·1 (84·0 to 194·7)	2·1% (0·2 to 4·4)	−11·1% (−12·8 to −8·9)
24	Drug use disorders	1·0% (0·8 to 1·3)	9·23 (6·54 to 11·8)	114·1 (80·9 to 145·5)	26·2% (22·5 to 30·1)	13·9% (10·9 to 17·2)
25	Other mental disorders	1·0% (0·7 to 1·3)	8·96 (5·72 to 13·5)	106·6 (68·2 to 160·7)	19·5% (18·4 to 21·0)	−0·2% (−0·8 to 0·2)

Data in parentheses are 95% uncertainty intervals. Count data are presented to three significant figures, and rates and percentage change data are presented to one decimal place. COPD=chronic obstructive pulmonary disease. YLDs=years lived with disability.

Global YLLs increased non-significantly (4·6% [95% UI –0·9 to 10·7]) between 2010 and 2021 (appendix 2 table S19). However, relative to YLDs, a larger decrease of 12·0% (6·4 to 16·8) in age-standardised YLL rates was found between 2010 (28 595·8 [27 568·9 to 29 636·5] per 100 000) and 2021 (25 175·4 [23 693·7 to 26 906·6] per 100 000). The top three causes of YLLs, across all ages and sexes, in 2021 were: COVID-19 (198 million [187–211] YLLs), ischaemic heart disease (184 million [173–195]), and neonatal disorders (165 million [141–191]; appendix 2 table S19). Age-standardised YLD and YLL rates, and the number of YLDs and YLLs by cause, location, and sex for 2010, 2019, 2020, and 2021 are in appendix 2 (tables S16, S17, S18, S19, S20, S21).

### Trends in DALYs by SDI, location, age, and sex

**Figure 3:**
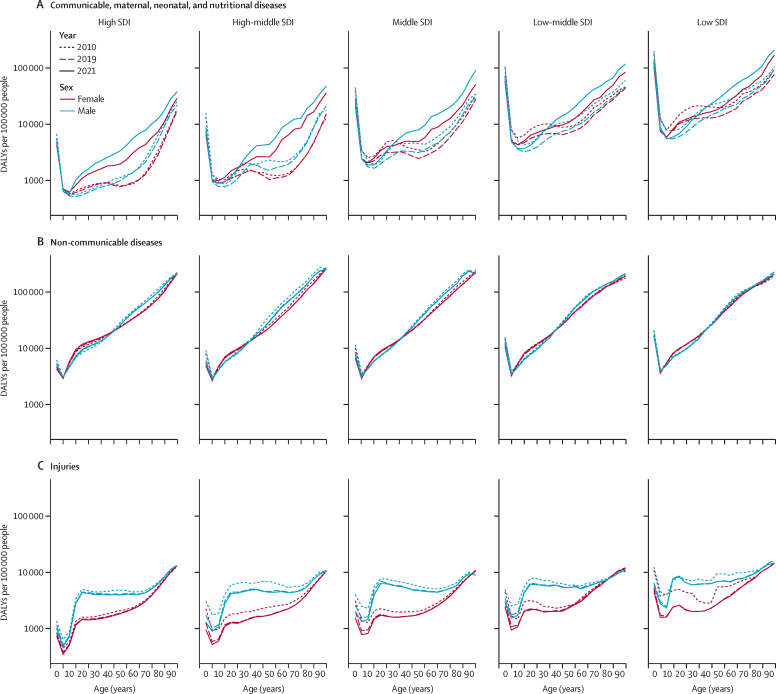
Age-specific DALY rates for Level 1 causes of communicable, maternal, neonatal, and nutritional diseases (A), non-communicable diseases (B), and injuries (C), by age, sex, year, and SDI quintile The y-axis shows DALYs per 100 000 population on a logarithmic scale. DALY=disability-adjusted life-year. SDI=Socio-demographic Index.

Trends in DALYs at the global level were informed by complex patterns of cause-specific burden across location, age, and sex. Age-standardised DALY rates for non-communicable diseases in males in 2021 ranged from 18 489·4 (95% UI 16 343·7–21 105·5) DALYs per 100 000 population in the high SDI quintile to 24 964·7 (22 503·7–27 556·8) per 100 000 in the low-middle SDI quintile. In females age-standardised DALY rates ranged from 17 167·4 (14 340·9–20 435·8) per 100 000 in the high SDI quintile to 24 072·6 (21 230·1–27 461·6) per 100 000 in the low SDI quintile (appendix 2 table S22). Across all SDI quintiles, age-specific DALY rates from non-communicable diseases decreased with increasing age from 0–6 days through to 5–9 years, and then increased gradually with age from this point on (figure 3).

Age-standardised DALY rates for CMNN diseases in males in 2021 ranged from 2902·3 (95% UI 2750·4 to 3092·7) per 100 000 in the high SDI quintile to 28 861·0 (26 081·7 to 32 375·0) per 100 000 in the low SDI quintile. In females, they ranged from 2014·2 (1816·0 to 2272·9) per 100 000 in the high SDI quintile to 24 040·3 (21 630·5 to 26 984·8) per 100 000 in the low SDI quintile (appendix 2 table S22). Age-standardised DALY rates for CMNN diseases decreased between 2010 and 2021 in the low SDI quintile (by 19·3% [12·2 to 25·8]) and the low-middle SDI quintile (by 13·9% [7·1 to 19·7]). Age-standardised DALY rates for CMNN diseases decreased at lower rates in the middle SDI quintile (by 4·9% [–2·0 to 10·8]), whereas (due to the emergence of COVID-19) rates increased for both the high-middle SDI quintile (by 26·9% [20·1 to 34·6]) and the high SDI quintile (by 63·9% [54·9 to 73·8]). Except for the high SDI quintile, age-specific DALY rates for CMNN diseases were higher in females than males in age groups younger than 25 years, and higher among males than females in age groups including those aged 25 years and older. In the high SDI quintile, DALY rates for CMNN diseases were higher among males than among females across most of the lifespan (figure 3).

Age-standardised DALY rates for injuries in males in 2021 ranged from 3050·8 (95% UI 2784·5–3395·9) per 100 000 in the high SDI quintile to 5958·8 (5266·7–6754·1) per 100 000 in the low SDI quintile. In females, age-standardised DALY rates ranged from 1384·7 (1206·2–1607·8) per 100 000 in the high-middle SDI quintile to 2787·9 (2414·0–3214·2) per 100 000 in the low SDI quintile (appendix 2 table S22). Age-standardised DALY rates for injuries decreased between 2010 and 2021 in all locations, ranging from a decrease of 31·7% (26·3–36·2) in the low SDI quintile to a decrease of 12·3% (10·4–14·2) in the high SDI quintile. Across all SDI quintiles, DALY rates for injuries emerged 0–6 days after birth, declined between the age groups of 7–27 days after birth and 5–9 years, and increased with age thereafter. From age 20 years and older, DALY rates for injuries in males remained relatively stable with increasing age and males aged 15–49 years in low SDI locations had similar DALY rates from injuries in 2021 (6854·7 [6203·9–7633·6] DALYs per 100 000) as they did in 2010 (7317·5 [6689·3–8064·0] per 100 000, a decrease of 6·3% [0·6–11·7]), whereas the DALY rates for females increased quite steadily with age from age 20 years (figure 3).

**Figure 4:**
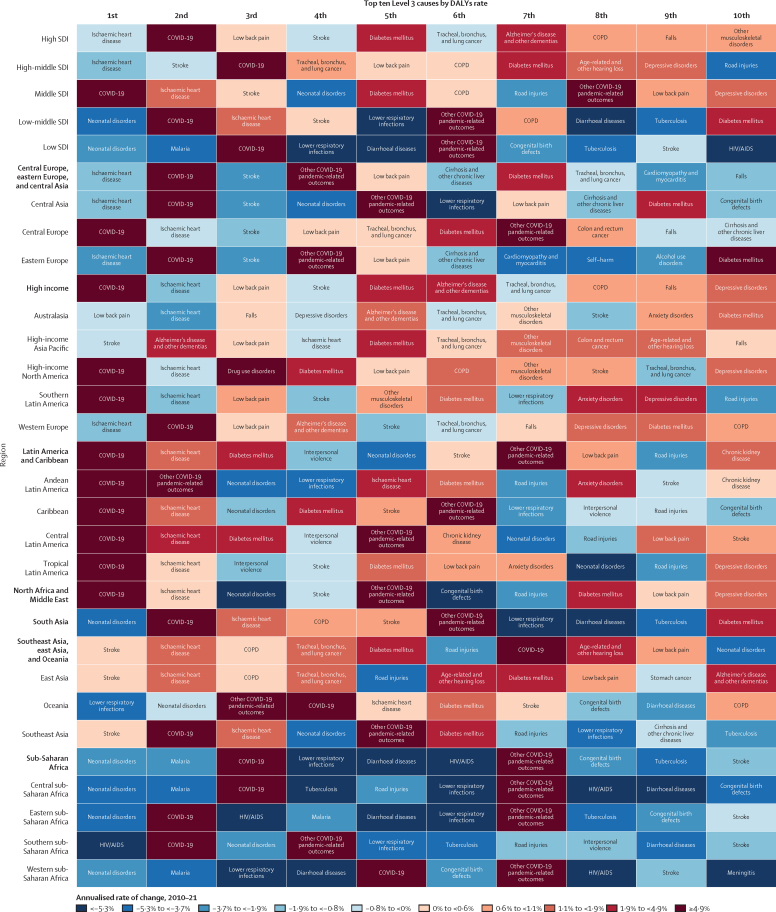
Leading ten Level 3 causes of DALYs in 2021 by SDI quintile, region, super-region, and annualised rate of change between 2010 and 2021 Level 3 causes are ranked by attributable DALYs from left (first) to right (tenth) for each GBD region and SDI quintile, with GBD super-regions in bold. Leading ten Level 3 causes of DALYs are ranked according to 2021 DALYs counts. COPD=chronic obstructive pulmonary disease. DALY=disability-adjusted life-year. SDI=Socio-demographic Index.

Drivers of changes in DALYs by location are further illustrated in figure 4, which shows the ten leading Level 3 causes of DALYs in 2021 and their annualised rate of change between 2010 and 2021 by region, super-region, and SDI quintile. In the low SDI quintile, seven of the ten leading Level 3 causes of DALYs were CMNN diseases, led by neonatal disorders (77·9 million [95% UI 65·4 to 92·8] DALYs), malaria (39·7 million [16·2 to 77·3]), and COVID-19 (33·7 million [30·8 to 38·2]; appendix 2 table S23). Age-standardised DALY rates per 100 000 due to these three causes were highest in the low SDI quintile (neonatal disorders: 4599·0 [3868·8 to 5449·4]; malaria: 2872·3 [1135·3 to 5709·5]; COVID-19: 5646·7 [5223·4 to 6170·2]), and lowest in the high SDI quintile (neonatal disorders: 536·1 [477·1 to 596·7]; malaria: 0·0 [0·0 to 0·1]; COVID-19: 1220·2 [1148·4 to 1341·9]; appendix 2 table S22). As SDI increased, more non-communicable diseases emerged in the top ten leading causes of DALYs (figure 4). In the high SDI quintile, eight of the ten leading Level 3 causes of DALYs were non-communicable diseases, led by ischaemic heart disease (23·5 million [21·5 to 24·7] DALYs), low back pain (15·9 million [11·5 to 21·2]), and stroke (15·2 million [13·7 to 16·4]); appendix 2 table S23). However, the age-standardised rate of DALYs due to ischaemic heart disease was lowest in the high SDI quintile (1133·9 [1053·0 to 1185·9] per 100 000) and highest in the low-middle SDI quintile (3137·8 [2912·4 to 3360·9] per 100 000). The largest changes in age-standardised DALY rates for ischaemic heart disease between 2010 and 2021 ranged from a decrease of 1·4% (–6·5 to 8·6) in low SDI locations to a decrease of 26·0% (20·6 to 31·3) in high-middle SDI locations. The age-standardised rate of DALYs due to low back pain was highest in the high SDI quintile (1094·3 [792·2 to 1459·2] per 100 000) and was lowest in the middle SDI quintile (717·5 [512·8 to 962·4] per 100 000). However, the age-standardised DALY rate due to stroke was lowest in high SDI locations (730·5 [665·3 to 786·6] per 100 000) and highest in low SDI locations (2462·9 [2203·8 to 2735·2] per 100 000). The smallest change in age-standardised DALY rates due to stroke was estimated to be for low SDI locations (decrease of 9·6% [2·5 to 15·8]), whereas the largest decrease was estimated to be for high-middle SDI locations (26·0% [20·6 to 31·3]).

For eight of 21 GBD regions, the leading cause of burden was COVID-19 (figure 4). However, COVID-19 did not feature within the top ten leading Level 3 causes of DALYs in Australasia, high-income Asia Pacific, or east Asia in 2021. The annualised rate of change for number of DALYs between 2010 and 2021 was largest for drug use disorders in high-income North America (6·5% [6·0–7·1]) and diabetes in eastern Europe (5·5% [4·9–6·2]).

**Figure 5:**
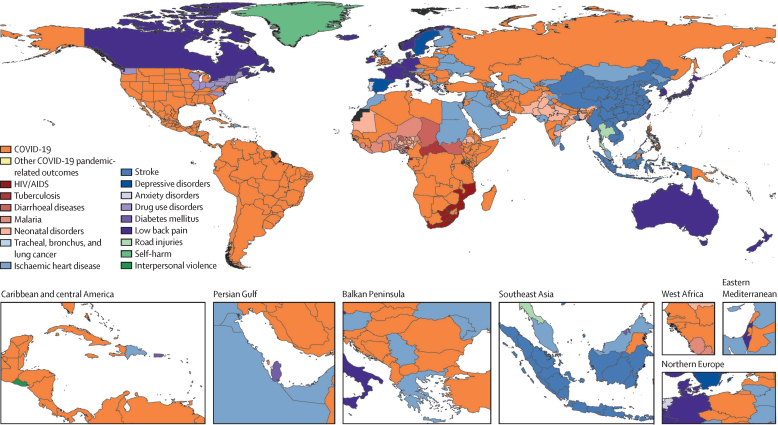
Leading Level 3 causes of age-standardised DALY rates by location, all ages, both sexes, in 2021 Dotted lines indicate disputed territories. DALY=disability-adjusted life-year.

The leading Level 3 causes of age-standardised DALY rates by location in 2021 are shown in figure 5 (the equivalent map for 2020, and for 2020 by sex, are in appendix 2 [figures S3, S4, S5, S6, S7]). COVID-19 was the leading cause of age-standardised DALY rates in 95 (47%) of 204 countries and territories. Despite the large burden from COVID-19 in these locations—including many in the super-region of sub-Saharan Africa—neonatal disorders, diarrhoeal diseases, malaria, or HIV/AIDS persisted as the leading Level 3 causes of burden in 17 countries and territories in sub-Saharan Africa. Ischaemic heart disease continued to be the leading cause of burden in nine countries and territories in north Africa and the Middle East, five countries and territories in western Europe, and six countries and territories in central Asia. Low back pain was the most burdensome cause in Canada (in high-income North America), and all locations in Australasia. Stroke was the most burdensome cause in all locations in east Asia.

### Trends in HALE by location and year

We estimated global HALE at birth of 61·3 years (95% UI 58·6 to 63·6) in 2010, 63·6 years (60·7 to 66·2) in 2019, and 62·2 years (59·4 to 64·7) in 2021 (appendix 2 table S24). HALE at birth increased by 1·4% (0·4 to 2·4) between 2010 and 2021. However, HALE decreased by 2·2% (1·6 to 2·9) between 2019 and 2021. For 2021, we estimated higher HALE at birth in high SDI locations (68·5 years [65·2 to 71·3]) than in low SDI locations (54·4 years [51·6 to 56·7]). We estimated the greatest change in HALE at birth between 2010 and 2021 for low SDI locations (a change of 4·2% [1·9 to 6·0], from 52·2 years [49·6–54·3] in 2010). By comparison, we estimated minimal changes in HALE at birth within high SDI locations between 2010 and 2021 (a change of –0·5% [–0·8 to –0·2], from 68·9 years [65·7 to 71·6] in 2010). We estimated that HALE at birth increased between 2010 and 2021 for 11 of 21 GBD regions, with the largest increases estimated for the Caribbean, where HALE increased by 19·7% (16·5 to 23·3), from 50·4 years (47·9 to 52·6) in 2010 to 60·4 years (57·4 to 63·0) in 2021. We also found that HALE at birth decreased between 2010 and 2021 in six of 21 GBD regions. The largest decrease in HALE at birth was observed in Andean Latin America, which decreased by 5·4% (3·8 to 7·0), from 65·9 years (62·8 to 68·3) in 2010 to 62·3 years (59·4 to 64·7) in 2021.

We estimated improvement in HALE at birth in 59 (29%) of 204 countries and territories between 2010 and 2021 (appendix 2 table S24). Among them, there were 30 countries and territories where HALE at birth increased by more than 2 years between 2010 and 2021. Haiti had the largest improvement in HALE at birth (an increase of 90·2% [95% UI 77·1–104·6], from 27·4 years [25·5–29·3] in 2010 to 52·1 years [48·2–55·4] in 2021) due to the Haiti earthquake in 2010 and recovery thereafter. The next largest improvements were observed in Eswatini (an increase of 11·8% [6·2–19·2], from 41·1 years [38·5–43·7] in 2010 to 45·9 years [43·1–48·7]) and Côte d'Ivoire (an increase of 9·7% [5·8–13·5], from 50·0 years [47·4–52·3] in 2010 to 54·9 years [51·8–57·8] in 2021). In 43 countries and territories, we estimated HALE at birth to decrease between 2010 and 2021, and in 21 countries and territories, HALE at birth decreased by more than 2 years between 2010 and 2021. The largest reduction in HALE at birth was observed in Peru (a decrease of 7·4% [5·2–9·8], from 68·0 years [64·6–70·7] in 2010 to 63·0 years [60·1–65·4] in 2021) and Venezuela (a decrease of 6·9% [3·7–10·1], from 65·4 years [62·4–68·0] in 2010 to 60·9 years [57·7–64·0] in 2021).

## Discussion

In 2020 and 2021, global health outcomes, as measured by age-standardised DALY rates, worsened for the first time in three decades. From 1990 to 2019, GBD analyses showed consistent and rather encouraging improvements in overall health outcomes at the population level. During this period, achievements by the global health community included reductions in vaccine-preventable deaths and improvements in under-5 mortality rates,^3^, ^17^ contributing to the trend of people living longer. However, as a global epidemiological transition occurs wherein the greatest share of disease burden shifts from communicable diseases to non-communicable diseases, populations are living longer but in poorer health. GBD 2021 reports a new global trend: the global number of DALYs and age-standardised DALY rates increased in both 2020 and 2021. This is a setback from the gains in overall human health at the global population level over the past three decades, and the evidence suggests that the COVID-19 pandemic was the inflection point for the reversal in progress. GBD 2021 quantified the health impacts of COVID-19 during some of the worst phases of the pandemic. Estimates of health loss caused by the COVID-19 pandemic provide the global health community—researchers, practitioners, and policy makers—with comprehensive health metrics about the immediate and long-term health needs of the populations most affected. The shared knowledge base provided by GBD 2021 can help stakeholders at all levels of responsibility—from multilateral agencies and national governments to local organisations—identify systemic inefficiencies that must be addressed in preparation for future pandemics and other global health crises.^22^ DALYs are a useful metric to understand the effects of the COVID-19 pandemic on population health because they capture overall disease burden by measuring how diseases and injuries reduce years of healthy living. The estimation of DALYs up to 2021 also provides an important new benchmark for evaluating progress towards Sustainable Development Goal 2030 targets. GBD 2021 presents new opportunities for mutual understanding and accountability by countries and territories in their responses to the health inequalities persisting in their populations.

### Important trends

The profound impact of the COVID-19 pandemic affected population health at the global level, the reverberations of which continue to be detected through population-level scientific studies.^16^, ^23^, ^24^, ^25^, ^26^ GBD 2021 found that the burden of COVID-19, as measured by DALYs, increased between 2020 and 2021. In 2020, the global all-age incidence of COVID-19 was 1·63 billion (95% UI 1·55–1·69) cases, equivalent to an age-standardised incidence rate of 20 819·8 (19 908·0–21 681·5) cases per 100 000. Global COVID-19 incidence increased to 2·28 billion (2·18–2·37) in 2021, equivalent to an age-standardised incidence rate of 28 955·3 (27 708·3–30 143·2) per 100 000. The distribution of DALYs due to COVID-19 was mostly driven by deaths and YLLs (as opposed to YLDs). Although in 2021 males and females had similar COVID-19 incidence rates, the age-standardised DALY rates for males were higher than for females. We found that, in addition to the sex disparities, both COVID-19 incidence and DALY rates were higher in low SDI locations than in high SDI locations. DALYs from other COVID-19 pandemic-related outcomes followed similar trends as DALYs due to COVID-19, in that they were higher in males than in females and highest in the low SDI quintile.

When shifting our focus from burden imposed directly by COVID-19 to the secondary impacts of the pandemic, other trends emerged. In the cases of both long COVID (data not shown) and depressive and anxiety disorders, the burden was driven by YLDs in 2020 and 2021 and was greater in females than males. The health consequences of the pandemic were unevenly distributed across demographic groups and geographical locations, which exacerbated existing socioeconomic inequities.^27^ Government responses to the health-care challenges presented by the COVID-19 pandemic were varied, and susceptible populations were most affected as a result. The delivery of care and services—whether basic and routine care like vaccinations or immediate COVID-19 medical aid—was hampered.^28^ Our estimation of burden for COVID-19 accounted for the roll-out of COVID-19 vaccines across countries and territories in 2021. Due to use of face masks, physical distancing, and other non-pharmaceutical interventions deployed during the COVID-19 pandemic, we found evidence that the burden of some other infectious diseases (eg, influenza and respiratory syncytial virus) in some locations was reduced, the effects of which were incorporated into our excess mortality estimations.^23^ Locations with strained and under-resourced health systems faced overwhelming structural challenges in securing swift and sufficient supplies of vaccine, which compromised vaccine delivery to high-risk populations,^29^ and further affected progress in responding to the burden imposed by the pandemic in under-resourced locations in 2021.^25^

As the pandemic recedes and COVID-19 becomes an endemic disease, a new global health landscape is becoming visible. In addition to COVID-19, six CMNN diseases were in the top 25 leading Level 3 causes of DALYs globally in 2021 (neonatal disorders, lower respiratory infections, diarrhoeal diseases, malaria, tuberculosis, and HIV/AIDS). DALY counts for each of these causes had decreased since 2010, with reductions in age-standardised DALY rates between 2010 and 2021 ranging from a decrease of 17·1% (95% UI 4·9 to 25·9) for neonatal disorders to a decrease of 47·8% (43·3 to 51·7) for HIV/AIDS. As anticipated with the global epidemiological transition,^3^, ^17^ the decrease in burden from CMNN diseases between 2010 and 2021 was largely driven by a decrease in age-standardised YLL rates (which decreased by 15·1% [6·7 to 22·2]) rather than YLD rates (which decreased by 4·5% [–3·6 to 20·7], appendix 2 tables S26, S27). The progress on reducing the burden of CMNN diseases must preserve momentum, particularly across low SDI locations. Neonatal disorders remained the third-leading cause of DALYs worldwide in 2021 and was in the top three leading causes of DALYs in 69 of 204 countries and territories. While the global community made progress in curtailing deaths and YLLs from neonatal disorders between 2010 and 2021 (19·5% [7·0 to 29·5] reduction in age-standardised YLL rates between 2010 and 2021),^3^ many still experience the non-fatal health consequences of neonatal disorders throughout their lifespan. The age-standardised DALY rates of diarrhoeal diseases decreased by 47·0% (39·9 to 52·9) between 2010 in 2021 due primarily to progress in the management of its risk factors among children younger than 5 years.^30^ However, the decrease in burden of diarrhoeal diseases was not evenly distributed across SDI locations or groups aged 5 years and older and remained one of the top ten leading causes of DALYs in 50 countries and territories in 2021. As with diarrhoeal diseases, the global burden of HIV/AIDS also decreased. Increasing treatment coverage and preventive strategies, particularly advances in antiretroviral therapy and the implementation of treatment-as-prevention programmes,^31^ reduced HIV/AIDS burden globally in 2021. In 2021, however, HIV/AIDS remained in the top ten leading causes of DALYs in 39 countries and territories and was the leading cause of DALYs in Congo (Brazzaville), Equatorial Guinea, Djibouti, Malawi, Mozambique, Zambia, Botswana, Eswatini, Lesotho, and South Africa. Global treatment coverage is not increasing at a sufficient rate to meet the joint UNAIDS targets to globally diagnose 95% of people living with HIV, provide treatment to 95% of people diagnosed, and achieve viral suppression in 95% of people on treatment by 2030.^32^ Progress to slow down transmission, increase treatment rates, and prevent HIV-related deaths is urgently required.

Between 2010 and 2021, DALYs from non-communicable diseases increased by 17·6% (95% UI 13·8 to 21·0) due to the ageing and growing global population,^17^ with only a moderate improvement in corresponding age-standardised DALY rates (decrease of 6·4% [3·5 to 9·5] between 2010 and 2021; appendix 2 table S25). The non-communicable disease burden was distributed widely across the world's populations, irrespective of age, sex, or socioeconomic circumstance, which presents both immediate and long-term challenges to health-care systems.^16^, ^33^, ^34^ Cardiovascular diseases—notably, Level 3 causes including ischaemic heart disease and stroke—were in the top five leading sources of global DALYs in 190 countries and territories in 2021. Changes in age-standardised DALY rates from 2010 to 2021 for stroke ranged from decreases of 9·6% (2·5 to 15·8) in low SDI locations to decreases of 24·9% (17·3 to 31·7) in high-middle SDI locations, whereas for ischaemic heart disease changes in age-standardised DALY rates ranged from a decrease of 1·4% (–6·5 to 8·6) in low SDI locations to a decrease of 26·0% (20·6 to 31·3) in high-middle SDI locations. Ischaemic heart disease and stroke share many modifiable risk factors and opportunities for intervention. The increasing global burden from diabetes between 2010 and 2021, alongside established risk factors for cardiovascular diseases (eg, high blood pressure, high cholesterol, high BMI, kidney dysfunction, ambient and household air pollution, physical inactivity, and tobacco use) are known contributors to stagnating progress on reducing the burden from cardiovascular diseases.^35^ Intervention strategies targeting modifiable risks, especially approaches with demonstrated success, such as tobacco control and blood pressure-lowering and cholesterol-lowering strategies,^35^ will help reduce the overall burden of non-communicable diseases. In addition to prevention through the management of risk factors, the negative health effects of cardiovascular disease can be mitigated through timely intervention for acute events and surgical procedures. However, geographical disparities between high-income countries and low-income and middle-income countries in access to and quality of cardiac surgical care must be addressed,^36^, ^37^ which would help populations for whom the burden of cardiovascular diseases is greatest receive the necessary care and treatment.

We estimated that 31·3% (95% UI 25·4–37·5) of global DALYs in 2021 were due to YLDs. The leading contributors of global YLDs were non-communicable diseases: low back pain, depressive disorders, and headache disorders. Low back pain was the largest contributor of global all-cause YLDs in 2021, with a decrease in the age-standardised YLD rate of only 2·4% (1·8–2·9) since 2010. Globally, prevalent cases and YLDs from low back pain increased with age—peaking in the 85–89 year age group—presenting huge challenges to countries with ageing populations.^38^ Clinical guidelines for treatment and prevention include education, self-care, physical therapy, medication, non-surgical treatments, and surgery.^39^, ^40^ Not only are some of these treatment options costly, but also, when used in isolation, rarely address the complex nature of low back pain. A holistic approach to the burden imposed by low back pain, addressing its biological, social, and psychological components, with further research into the efficacy of diagnostic and preventive options, would advance our ability to manage this considerable source of disability worldwide.

The efficacy and cost-effectiveness of treatment options for depressive disorders across low-income, middle-income, and high-income settings have been established by existing research.^16^ These include the use of self-care (eg, web-based therapy), primary care, and community outreach programmes to deliver psychological and pharmacological interventions, hospital care, and specialist services.^41^ Although mental health interventions are effective and cost-effective, and public opinions regarding the benefits of seeking treatment for mental disorders are becoming more supportive, we estimated there were 332 million (95% UI 298–376) cases of depressive disorders globally in 2021. Cases were distributed across the entire lifespan but were most common among females between age groups 15–19 years and 60–64 years. In many instances, depressive disorders are rarely detected at their onset, and only a small proportion of individuals receive the evidence-based treatment packages considered to be minimally adequate.^34^, ^41^ This is also the case for other disabling and burdensome mental disorders, such as anxiety disorders and schizophrenia, and substance use disorders. The findings of GBD 2021 underline the need for an enhanced response to address mental and substance use disorders through expanded financial commitments and improved service quality and access. The need to intensify our efforts to minimise the negative health consequences of mental and substance use disorders is urgent in the wake of shock events, like the COVID-19 pandemic, natural disasters, and military conflicts.^16^ Additionally, many mental disorders are prevalent during childhood or early adulthood, when other risk factors such as bullying victimisation and childhood maltreatment can occur.^16^, ^42^ Early interventions with population-based prevention strategies targeting these risk factors and promoting the social and emotional development of younger populations will lead to positive outcomes. Interventions that can address the increasing burden of drug use disorders are also urgently required, particularly for opioid use disorders. Opioid substitution therapy can decrease opioid use and health risks involved with injecting drugs. The effects of an opioid overdose can be reversed with use of the opioid antagonist naloxone, the availability of which ought to be expanded alongside training on safe and effective administration for pharmacists, health-care providers, individuals at risk of opioid overdose, their family members, and social service agencies that work with substance users.^43^

Approximately 2·81 billion (95% UI 2·60 to 3·03) individuals had migraine or tension-type headache in 2021, with females aged 15–49 years being most affected. Interventions targeting the management of symptoms exist, but we found no sign of improvement in the burden of these disorders between 2010 and 2021 (with a 0·3% [–1·1 to 1·4] increase in age-standardised YLD rates). In addition to opportunities to scale up treatment strategies, more work is required to establish the modifiable risk factors of headache disorders.^44^

Among the leading 25 causes of YLDs, we observed the largest increase in global age-standardised YLD rates for diabetes between 2010 and 2021 (25·9% [95% UI 24·0–28·1]). An increase in the age-standardised rate was evident in all 204 countries and territories between 2010 and 2021. This increase in age-standardised YLD rates for diabetes was largely driven by type 2 diabetes and the increasing rates of obesity globally. Type 2 diabetes is preventable and, in some instances, reversible with early detection and adequate care.^33^, ^45^, ^46^ GBD 2021 estimates that 525 million (490–565) people had diabetes in 2021; however, without global intervention, a separate GBD 2021 analysis has forecast that more than 1·31 billion people worldwide will have diabetes by 2050.^33^ Any coordinated response to the escalating burden of diabetes must target the underlying causes of obesity and help remove the social and logistical barriers to accessing care and services.

The overall burden imposed by injuries was driven by deaths and YLLs, both of which decreased between 2010 and 2021. Reductions in the burden of injuries were not distributed equally across time, sex, and location. The travel restrictions and physical distancing measures implemented in some countries and territories to control the spread of SARS-CoV-2 during the COVID-19 pandemic might have contributed to small decreases in road accidents in 2020 and 2021; however, data and evidence on this have been inconsistent.^3^ Our estimates indicated that most of the health gains from injuries occurred between 2010 and 2019 as opposed to between 2019 and 2021. Burden from injuries was highest in low SDI locations, where exposure to forces of nature (ie, natural disasters) increased the burden due to injuries in 2010 for both males and females. Despite this, males aged 15–49 years in low SDI locations had similar DALY rates from injuries in 2021 as they did in 2010. The leading injuries among males aged 15–49 years in the low SDI quintile in 2021 were road injuries, conflict and terrorism, and interpersonal violence (data not shown). Males were more affected by these injuries than their female counterparts, for whom we saw a substantial decrease in burden from injuries in the same period (decrease of 45·8% [40·9–50·1] since 2010; data not shown). Although populations in many countries and territories are living safer and longer lives, more effort is required to reach those same standards for many others, especially those living in low SDI locations.

Globally, HALE at birth improved from 61·3 years (95% UI 58·6–63·6) in 2010 to 62·2 years (59·4–64·7) in 2021. GBD 2021 provides an important baseline from which we can track the extent to which life expectancies rebound and progress in HALE accelerates after 2021. Between 2010 and 2021, we observed improvements in HALE at birth in 59 countries and territories, with people living in low SDI locations having larger gains than those living in high SDI locations. Faster progress in the sociodemographic circumstances in low SDI locations resulted in larger gains in life expectancy and HALE at birth between 2010 and 2021.

### Limitations

GBD estimates of health and health loss evolve across cycles because of the addition of new data, important improvements made to the burden estimation pipeline, and instability in datasets and processes, which we try to minimise wherever possible. Inconsistencies in the availability of primary epidemiological data remain a limitation and source of instability within GBD analyses. Our estimates depend on the out-of-sample predictive validity of modelling processes in cases where data are insufficient to produce burden estimates for all 204 countries and territories (by year, sex, and age). Although this approach cannot fully replace high quality primary data, it ensures that populations or causes with no or little data are not excluded from important benchmarking exercises intended for burden estimation. With any given GBD release, there might be extant data not identified or incorporated, which is a key part of the rationale for ongoing cycles of releases, rather than a single update. For the primary data available, our data processing methods account for known sources of variation wherever possible, but fully disentangling variation in our estimates is not always possible due to measurement error and reporting inaccuracies. There are problems with the quality and collection of primary data, such as flawed methodologies and potential under-reporting of illnesses, which is a recurring limitation for GBD that can be continually improved on by strengthening data-collection systems. Our identification of reference and alternative case definitions for epidemiological data undergoing bias correction can further inform survey design in future data collection efforts. Our estimation of 95% UIs is also an area requiring further improvement. Our analyses are intended to capture uncertainty from a range of data types and processes (eg, from stochastic variation in input data, age–sex splitting, bias corrections, and other data manipulations or statistical approaches), but it is difficult to capture all sources of uncertainty across the entire burden estimation pipeline.

Our estimation of YLDs is restricted by several factors. Due to data sparsity, severity distributions do not capture variation by access to treatments for major causes contributing to YLDs. The development of new approaches to model changes in severity by health-care access for depressive and anxiety disorders is underway and could provide a solution to this issue in future GBD cycles.^47^ The quality and accuracy of comorbidity corrections also requires improvement. Our comorbidity correction adjusted for the difference between the average disability weight for one sequela and the multiplicatively combined disability weight for multiple sequelae (ie, independent comorbidity). This approach underestimates the comorbidity between sequelae especially for causes (eg, mental disorders) where the comorbidity distribution is reliant on the range of disorders experienced. Limitations related to our estimation of YLLs are discussed in detail elsewhere.^3^

The estimation of DALYs for specific diseases and injuries also has distinct limitations, and these are presented in greater detail in appendix 1 (section 8) and topic-specific publications. Notably, for GBD 2021, challenges associated with collecting data and modelling a novel disease like COVID-19 were present. Modelling COVID-19, long COVID, and other COVID-19 pandemic-related outcomes presented analytical challenges for a range of reasons. First, data on COVID-19 outcomes (eg, cases, deaths, hospital admissions, and seroprevalence) were of highly variable quality and completeness, which required novel methods for standardisation.^23^, ^25^, ^26^ Second, approaches for estimating excess mortality, which were used to estimate the extent of other COVID-19 pandemic-related mortality, can produce quite divergent results in some locations.^17^ Third, we found considerable heterogeneity in case definition and instrumentation used to measure long COVID. We concentrated on estimates for three major symptom clusters associated with long-term health loss from COVID-19 because these would not be captured by GBD cause estimates. Cohort data suggest that these three clusters capture the most severe outcomes reported at 3 months or longer after initial infection but are not a complete capture of all long COVID symptoms.^24^ However, we had limited ability in GBD 2021 to capture the specific indirect effects of COVID-19, both in modelling fatal and non-fatal outcome estimations. In future analyses, the combined effect of changing virus variants, vaccinations, and past infection on the occurrence of long COVID will need to be evaluated. Due to sparsity of data from long COVID follow-up studies, particularly from low-income and middle-income settings, we were unable to fully assess variation in the occurrence, severity, or duration of long COVID between locations. As more data become available from the pandemic era, the ability to more fully and specifically estimate the indirect effect of the COVID-19 pandemic across many causes is likely to improve. Work to address these limitations in future data collection efforts is in development and will be incorporated into future iterations of GBD.^24^

### Future directions

Efforts by GBD research teams to include new causes and locations, secure new epidemiological data, more precisely correct for measurement error, and capture uncertainty within burden estimates are ongoing areas of priority. Because forecasts of future health trends are of great value to policy makers, forecasting the burden of disease under reference and alternative risk exposure scenarios is another ongoing area of high priority. There also remains a need to analyse the long-term effects of COVID-19 and develop tools and analytical frameworks that can measure the hidden burden of the pandemic. Developing and refining analytical tools to better understand the extent of the burden imposed by COVID-19 will also assist pandemic planning and preparedness. More streamlined incorporation of location-level covariates capturing the effect of the pandemic is being tested within our cause of death and prevalence modelling for the next GBD cycle. The use of DisMod-AT, an updated version of DisMod-MR, to estimate prevalence is also proceeding for several causes. We expect DisMod-AT to produce more accurate trends over age and time within our prevalence estimates, including the integration of the effect of population shock events. We also expect to incorporate severity distributions by health-care access for several causes in the next GBD cycle and continue work to eventually produce dependent comorbidity corrections. Our burden estimation processes are constantly evolving to provide the most comprehensive and accurate information to stakeholders. However, areas of innovation and methods development also need to be staggered to allow us to respond to emerging trends and the ever-evolving public health landscape.

### Conclusion

In 2020 and 2021, the global all-cause burden of disease increased for the first time in three decades. This reversal in progress was due to three main factors: (1) the direct health impacts of COVID-19; (2) the indirect health consequences of COVID-19 and the pandemic, such as the effect on mental disorders; and (3) fallout from overburdened health service systems and uncoordinated policy responses to the COVID-19 pandemic. The effect of the pandemic, as measured by DALYs, was unevenly distributed by age, sex, and location, exacerbating many existing inequalities. As the world starts to recover, continuing gains for other CMNN diseases must not stall. Urgent priority needs to be given to prevalent and disabling non-communicable diseases that have seen insufficient or no improvement despite several calls to action—notably, cardiovascular diseases, musculoskeletal disorders (ie, low back pain), and mental disorders. Ultimately, GBD 2021 results illustrate the need for our global community to come together in a multidisciplinary and coordinated response to deliver not just better survival rates, but healthier and safer outcomes for our ever growing and ageing population.

## Data sharing

To download the data used in these analyses, please visit the GBD 2021 Sources Tool. The statistical code used in GBD 2021 is available online.

For more on **cause-specific methods to generate GBD estimates** see https://www.healthdata.org/gbd/methods-appendices-2021

For detailed results see **GBD Results** https://www.vizhub.healthdata.org/gbd-results and **GBD Compare** https://www.vizhub.healthdata.org/gbd-compare

## Declaration of interests

S Afzal reports support for the present manuscript from King Edward Medical University including study material, research articles, valid data sources and authentic real time information for this manuscript; payment or honoraria for lectures, presentations, speakers bureaus, manuscript writing, or educational events from King Edward Medical University and collaborative partners including University of Johns Hopkins, University of California, University of Massachusetts, KEMCAANA, KEMCA-UK Scientific Conferences and Webinars; support for attending meetings and/or travel from King Edward Medical University to attend meetings; participation on a Data Safety Monitoring Board or Advisory Board with National Bioethics Committee Pakistan, King Edward Medical University Ethical Review Board, as well as Ethical Review Board Fatima Jinnah Medical University and Sir Ganga Ram Hospital; leadership or fiduciary roles in board, society, committee or advocacy groups, paid or unpaid with Pakistan Association of Medical Editors, Fellow of Faculty of Public Health Royal Colleges UK (FFPH), and Society of Prevention, Advocacy And Research, King Edward Medical University (SPARK); and other support as Dean of Public Health and Preventive Medicine at King Edward Medical University, as the Chief Editor Annals of King Edward Medical University, as the Director of Quality Enhancement Cell King Edward Medical University, as an international-level Fellow of Faculty of Public Health UK, as an Advisory Board Member and Chair Scientific Session KEMCA-UK, as a Chairperson of KEMCAANA (the International Scientific Conference), as a national-level member on the Research and Publications Higher Education Commission (HEC Pakistan), as a member of the Research and Journals Committee (Pakistan) the Medical and Dental Council (Pakistan), the National Bioethics Committee (Pakistan), the Corona Experts Advisory Group (Punjab), the Chair of the Dengue Experts Advisory Group, and a member of the Punjab Residency Program Research Committee; all outside the submitted work. R Ancuceanu reports consulting fees from AbbVie; payment or honoraria for lectures, presentations, speakers bureaus, manuscript writing or educational events from AbbVie, Sandoz, and B. Braun; all outside the submitted work. D B Anderson reports leadership or fiduciary roles in board, society, committee or advocacy groups, paid or unpaid with PM&R journal as Senior Editor, which includes an annual stipend, outside the submitted work. P Atorkey reports infrastructure support for the present manuscript from the Australian College of Applied Professions, Discipline of Psychological Sciences, Sydney, Australia and the School of Medicine and Public Health, The University of Newcastle, Australia. J L Baker reports grants paid to their institution from Novo Nordisk Foundation, World Cancer Research Fund, Independent Research Council Denmark, and EU Horizon; consulting fees from Novo Nordisk Denmark A/S; unpaid leadership or fiduciary role in other board, society, committee or advocacy group with European Association for the Study of Obesity; all outside the submitted work. S Barteit reports research grants from the Carl-Zeiss Foundation and the German Research Foundation (DFG); stock or stock options from CHEERS company, a for-profit company focusing on climate change and health evaluation and response systems; all outside the submitted work. M L Bell reports grants or contracts paid to their institution from US Environmental Protection Agency (EPA), US National Institutes of Health (NIH), High Tide Foundation, Health Effects Institute, Yale Women Faculty Forum, Environmental Defense Fund, Wellcome Trust Foundation, Yale Climate Change and Health Center, Robert Wood Johnson Foundation, and Hutchinson Postdoctoral Fellowship; consulting fees from Clinique and SciQuest; payment or honoraria for lectures, presentations, speakers bureaus, manuscript writing or educational events from Colorado School of Public Health, Duke University, University of Texas, Data4Justice, Korea University, Organization of Teratology Information Specialists, University of Pennsylvania, and Boston University for speaking events, IOP Publishing for editorial duties, NIH, Health Canada, PAC-10, UK Research and Innovation, and AXA Research Fund Fellowship for grant review, Korea University for adjunct teaching/research, Harvard University and University of Montana for external advisory committee membership; support for travel from Colorado School of Public Health, University of Texas, Duke University, Boston University, University of Pennsylvania, Harvard University, *American Journal of Public Health*, and Columbia University; paid leadership or fiduciary roles in board, society, committee or advocacy groups, with US EPA Clean Air Scientific Advisory Committee (CASAC), and unpaid roles with Johns Hopkins EHE Advisory Board, Harvard external advisory committee for training grant, WHO Global Air Pollution and Health Technical Advisory Group, National Academies Panels and Committees; all outside the submitted work. P J G Bettencourt reports patents planned, issued or pending WO2020229805A1, BR112021022592A2, EP3965809A1, OA1202100511, US2023173050A1, EP4265271A2 and EP4275700A2; other interests as project reviewer at Botnar Foundation; all outside the submitted work. S Bhaskar reports grants or contracts from a Grant-in-Aid for Scientific Research (KAKENHI) from Japan Society for the Promotion of Science (JSPS), Japanese Ministry of Education, Culture, Sports, Science and Technology (MEXT) and a Japan Society for the Promotion of Science (JSPS) International Fellowship from JSPS and the Australian Academy of Science; leadership or fiduciary roles in board, society, committee or advocacy groups, paid or unpaid, with Rotary District 9675 as District Chair, Diversity, Equity & Inclusion, with Global Health & Migration Hub Community, Global Health Hub Germany, Berlin, Germany as Chair and Manager, with *PLoS One, BMC Neurology, Frontiers in Neurology, Frontiers in Stroke, Frontiers in Public Health*, and *BMC Medical Research Methodology* as Editorial Board Member, and with College of Reviewers, Canadian Institutes of Health Research (CIHR), and Government of Canada as Member; all outside the submitted work. B Bikbov reports grants or contracts from European Commission and University of Rome; support for attending meetings and/or travel from European Renal Association via reimbursement of hotel stay for congress lecturers; leadership or fiduciary role in other board, society, committee or advocacy group, unpaid, with Advocacy Group, International Society of Nephrology and Western Europe Regional Board, International Society of Nephrology; other non-financial support from Scientific-Tools.Org for a public health consultancy; all outside the submitted work. M Carvalho reports other support from The Laboratório Associado para a Química Verde (LAQV/REQUIMTE), University of Porto, Porto, Portugal, and Foundation for Science and Technology (FCT) and the Portuguese Ministry of Science, Technology, and Higher Education (MCTES) under the scope of the project UIDP/50006/2020 (DOI 10.54499/UIDP/50006/2020), outside the submitted work. A L Catapano reports grants or contracts from Amryt Pharma, Menarini, and Ultragenyx; payment or honoraria for lectures, presentations, speakers bureaus, manuscript writing or educational events from Amarin, Amgen, Amryt Pharma, AstraZeneca, Daiichi Sankyo, Esperion, Ionis Pharmaceutical, Medscaper, Menarini, Merck, Novartis, Novo Nordisk, Peervoice, Pfizer, Recordati, Regeneron, Sandoz, Sanofi, The Corpus, Ultragenyx, and Viatris; all outside the submitted work. A Caye reports consulting fees from Knight Therapeutics outside the submitted work. J Conde reports grants or contracts from European Research Council Starting Grant (ERC-StG-2019-848325. Funding 1.5M€); patents planned, issued, or pending from Universidade Nova de Lisboa for Surfactant-Based Hydrogel, Methods and Uses Thereof; all outside the submitted work. S Das reports unpaid leadership or fiduciary roles in board, society, committee, or advocacy groups with Association for Diagnostics & Laboratory Medicine (ADLM) India Section as Program Chair, Clinical & Laboratory Standards Institute (CLSI) nominating committee as a member, Women in Global Health India as a member; all outside the submitted work. L Degenhardt reports untied educational grants to examine new opioid medications in Australia from Indivior and Seqirus, outside the submitted work. A K Demetriades reports leadership or fiduciary roles in board, society, committee or advocacy groups, paid or unpaid, with AO Knowledge Forum Degen as Steering Committee Member, Global Neuro Foundation as Board Member, and European Association of Neurosurgical Societies (EANS) as Board Member/Officer; all outside the submitted work. A Faro reports support for the present manuscript from the National Council for Scientific and Technological Development, CNPq, Brazil. D Flood reports grants or contracts from the National Heart, Lung, and Blood Institute (NHLBI; award number K23HL161271), the University of Michigan Claude D Pepper Older Americans Independence Center (award number 5P30AG024824), and the University of Michigan Caswell Diabetes Institute Clinical Translational Research Scholars Program; consulting fees paid to their institution serving as a diabetes consultant for WHO; unpaid leadership or fiduciary roles in board, society, committee, or advocacy groups with Maya Health Alliance, a non-governmental health organisation in Guatemala, as Staff Physician to carry out diabetes advocacy and solicit funding for clinical diabetes programs; all outside the submitted work. L M Force reports support for the present manuscript from the Bill & Melinda Gates foundation; grants or contracts from Conquer Cancer Foundation, St Jude Children's Research Hospital, St Baldrick's Foundation, and NIH Loan Repayment Program, outside the submitted work; unpaid leadership or fiduciary roles in board, society, committee or advocacy groups with *The Lancet Oncology* International Advisory Board, outside the submitted work. M Foschi reports consulting fees from Roche and Novartis; payment or honoraria for lectures, presentations, speakers bureaus, manuscript writing, or educational events from Roche, Novartis, Sanofi, Merck, Biogen, and Bristol Myers Squibb for speaking at sponsored congresses; support for attending meetings and/or travel from Roche, Novartis, Sanofi, Merck, Biogen, and Bristol Myers Squibb; unpaid leadership or fiduciary roles in board, society, committee or advocacy groups with MSBase scientific leadership group; all outside the submitted work. R C Franklin reports grants or contracts from Heatwaves in Queensland – Queensland Government, Mobile Plant Safety – Agrifutures, Arc Flash – Human Factors – Queensland Government; support for attending meetings and/or travel from Australasian College of Tropical Medicine (ACTM) – Tropical Medicine and Travel Medicine Conference 2022, 2023, and International Society of Travel Medicine (ISTM) – Travel Medicine Conference, Basel 2023; leadership or fiduciary roles in board, society, committee or advocacy groups, paid or unpaid, with Kidsafe as President/Director, Farmsafe as Director, Auschem as Director, PHAA Injury Prevention SIG Convenor, ISASH Governance Committee, and ACTM as Vice President; all outside the submitted work. A Guha reports grants or contracts from the American Heart Association and the US Department of Defense; consulting fees from Pfizer, Novartis, and Myovant; leadership or fiduciary roles in board, society, committee or advocacy groups, paid or unpaid, with ZERO Prostate Cancer – health equity task force and Doctorpedia as a founding medical partner; all outside the submitted work. A Hassan reports consulting fees from Novartis, Sanofi Genzyme, Biologix, Merck, Hikma Pharma, Janssen, Inspire Pharma, Future Pharma, and Elixir Pharma; payment or honoraria for lectures, presentations, speakers bureaus, manuscript writing, or educational events from Novartis, Allergan, Merck, Biologix, Janssen, Roche, Sanofi Genzyme, Bayer, Hikma Pharma, Al Andalus, Chemipharm, Lundbeck, Inspire Pharma, Future Pharma and Habib Scientific Office, and Everpharma; support for attending meetings and/or travel from Novartis, Allergan, Merck, Biologix, Roche, Sanofi Genzyme, Bayer, Hikma Pharma, Chemipharm, and Al Andalus and Clavita Pharm; all outside the submitted work. I M Ilic reports support for the present manuscript from Ministry of Science, Technological Development and Innovation of the Republic of Serbia (project no. 175042, 2011-2023). M D Ilic reports support for the present manuscript from Ministry of Science, Technological Development and Innovation of the Republic of Serbia (project no. 451-03-47/2023-01/200111). S M S Islam reports support for the present manuscript from an Investigator Grant from the National Health and Medical Research Council (NHMRC; Australia) and a Vanguard Grant from the Heart Foundation. N E Ismail reports leadership or fiduciary role in other board, society, committee, or advocacy group, paid or unpaid, with The Bursar (Council Member), Malaysia, and the Academy of Pharmacy, Malaysia, outside the submitted work. T Joo reports support for the present manuscript from National Research, Development, and Innovation Office in Hungary (RRF-2.3.1-21-2022-00006), Data-Driven Health Division of National Laboratory for Health Security. J J Jozwiak reports payment or honoraria for lectures, presentations, speakers bureaus, manuscript writing, or educational events from Novartis, Adamed, and Amgen, outside the submitted work. N Kawakami reports grants or contracts from Junpukai Foundation and Department of Digital Mental Health is an endowment department, supported with an unrestricted grant from 15 enterprises (https://dmh.m.u-tokyo.ac.jp/c); consulting fees from Riken Institute, JAXA, Sekisui Chemicals, and SB@WORK; leadership or fiduciary role in other board, society, committee, or advocacy group, paid or unpaid, with Japan Society for Occupational Health; all outside the submitted work. J H Kempen reports grants or contracts from Sight for Souls and Mass Eye and Ear Global Surgery Program; leadership or fiduciary role in other board, society, committee, or advocacy group, paid or unpaid, with the Sight for Souls board (US 501(c)(3)); stock or stock options in Betaliq and Tarsier; all outside the submitted work. K Krishan reports non-financial support from the UGC Centre of Advanced Study, CAS II, awarded to the Department of Anthropology, Panjab University, Chandigarh, India, outside the submitted work. B Lacey reports support for the present manuscript from UK Biobank, funded largely by the UK Medical Research Council and Wellcome. B Langguth reports support for the present manuscript from the EU paid to their institution; grants or contracts paid to their institution from German Research Foundation, German Bundesministerium für Bildung und Forschung, EU, Bavarian-Czech University Association, and Bavarian State; consulting fees from Schwabe, Neuromod, Sea Pharma, and Rovi; payment or honoraria for lectures, presentations, speakers bureaus, manuscript writing, or educational events from Schwabe and Neuromod; payment for expert testimony from Bavarian State; leadership or fiduciary role in other board, society, committee, or advocacy group, unpaid, with Tinnitus Research Initiative and the German Society for Brain Stimulation in Psychiatry; stock or stock options in Sea Pharma; receipt of equipment, materials, drugs, medical writing, gifts or other services from Neurocare and Daymed; all outside the submitted work. M Lee reports support for the present manuscript from the Ministry of Education of the Republic of Korea and the National Research Foundation of Korea (NRF-2023S1A3A2A05095298) and Bio-convergence Technology Education Program through the Korea Institute for Advancement Technology (KIAT) funded by the Ministry of Trade, Industry and Energy (No. P0017805). M-C Li reports grants or contracts form The National Science and Technology Council in Taiwan (NSTC 112-2410-H-003-031); leadership or fiduciary role in other board, society, committee, or advocacy group, paid or unpaid, with the *Journal of the American Heart Association* as Technical Editor; all outside the submitted work. P W Mahasha reports support for attending meetings and/or travel South African Medical Research Council, Department of Nuclear Medicine, School of Medicine, University of Pretoria, and Discipline of Traditional Medicine, School of Nursing and Public Health, University of KwaZulu Natal; participation on a Data Safety Monitoring Board or Advisory Board with the Journal Current Research in Public Health (CRPH) as Editorial Board Member, the journal of *Frontiers in Reproductive Health* section HIV and STIs as Associate Editorial Board Member, and the journal *BMC Public Health* as Editorial Board Member; leadership or fiduciary role in other board, society, committee or advocacy group, unpaid, as member of the Federation of Infectious Diseases Societies of Southern Africa (FIDSSA; November 2021–ongoing), Member of the International Society for Infectious Diseases (ISID; November, 2021–ongoing), Member of the COVID-19 Clinical Research Coalition (August, 2021–ongoing), Member of the Global Burden of Disease Collaborator Network (February, 2019–ongoing), Fellow Member of the Scholars Academic and Scientific Society (SAS Society; FSASS; August 2021–ongoing), Member of the South African Council for Natural Scientific Professions (SACNASP; 2018–ongoing), Member of the African Organisation for Standardisation (ARSO), ARSO TC 82 dealing with African Traditional Medicine standards (2023–ongoing, Member of the South African Bureau of Standards (SABS)'s National Committee dealing with African Traditional Medicine standards (2024–ongoing), Member of the Global Forum for Research Ethics and Integrity (GFREI; 2023–ongoing); all outside the submitted work. M A Mahmoud reports grants or contracts from Deputyship for Research & Innovation, Ministry of Education in Saudi Arabia for research through project number 445-5-762 outside the submitted work. L G Mantovani reports support for the present manuscript from the Italian Ministry of Health. H R Marateb reports grants or contracts outside the submitted work from The Beatriu de Pinós post-doctoral programme from the Office of the Secretary of Universities and Research from the Ministry of Business and Knowledge of the Government of Catalonia (programme: 2020 BP 00261) received through their institution, Universitat Politècnica de Catalunya · Barcelona Tech – UPC. A-F A Mentis reports grants or contract funding from 'MilkSafe: A novel pipeline to enrich formula milk using omics technologies', a research co-financed by the European Regional Development Fund of the EU and Greek national funds through the Operational Program Competitiveness, Entrepreneurship and Innovation, under the call RESEARCH - CREATE - INNOVATE (project code: T2EDK-02222), as well as from ELIDEK (Hellenic Foundation for Research and Innovation, MIMS-860; both outside of the present manuscript); payment for expert testimony from serving as external peer-reviewer for FONDAZIONE CARIPLO, ITALY; participation in a Data Safety Monitoring or Advisory Board as Editorial Board Member for “Systematic Reviews”, for *Annals of Epidemiology*, and as Associate Editor for *Translational Psychiatry*; stock or stock options from a family winery; and other financial interests as the current scientific officer for BGI Group; outside the submitted work. S A Meo reports support for the present manuscript from Research Supporting Project, King Saud University, Riyadh, Saudi Arabia (RSP-2024 R47). P B Mitchell reports payment or honoraria for lectures, presentations, speakers bureaus, manuscript writing, or educational events from Janssen (Australia); participation on a Data Safety Monitoring Board or Advisory Board with Janssen (Australia); all outside the submitted work. L Monasta reports support from the present manuscript from the Italian Ministry of Health (Ricerca Corrente 34/2017), payments made to the Institute for Maternal and Child Health IRCCS Burlo Garofolo. R S Moreira reports grants or contracts from the National Council for Scientific and Technological Development (CNPq) Research Productivity Scholarship (registration number 316607/2021-5), outside the submitted work. J F Mosser reports support for the present manuscripts from the Bill & Melinda Gates Foundation via grant funding; grants or contracts outside the submitted work from Gavi; support for attending meetings or travel from the Bill & Melinda Gates Foundation, outside the submitted work. S Muthu reports support for attending meetings or travel from AO Spine KF Degen LV Award 2024 (travel grants to attend GSC 2024); leadership or fiduciary roles in board, society, committee, or advocacy groups, paid or unpaid, with AOSpine KF Degen as Associate Member, SICOT Grants Committee as a member; ICRS Next Gen Committee as a member; all outside the submitted work. S Nomura reports grant support for the present manuscript from Ministry of Education, Culture, Sports, Science and Technology of Japan (21H03203) and Precursory Research for Embryonic Science and Technology from the Japan Science and Technology Agency (JPMJPR22R8). B Norrving reports participation on a Data Safety Monitoring Board with Simbec Orion in HOVID trial, outside the submitted work. A P Okekunle reports support for the present manuscript from the National Research Foundation of Korea funded by the Ministry of Science and ICT (2020H1D3A1A04081265); support for attending meetings and/or travel from National Research Foundation of Korea funded by the Ministry of Science and ICT (2020H1D3A1A04081265), outside the submitted work. S Onie reports support for attending meetings and/or travel from Black Dog Institute for travel to the International Association for Suicide Prevention World Congress, Piran, 2023; leadership or fiduciary roles in board, society, committee, or advocacy groups, paid or unpaid, with International Association for Suicide Prevention as Elected National Representative for Indonesia and Indonesian Association for Suicide Prevention as Founding President; all outside the submitted work. A Ortiz reports grants or contracts from Sanofi, Institution: IIS-FJD; consultancy or speaker fees or travel support from Advicciene, Astellas, AstraZeneca, Amicus, Amgen, Boehringer Ingelheim, Fresenius Medical Care, GSK, Bayer, Sanofi-Genzyme, Menarini, Mundipharma, Kyowa Kirin, Lilly, Alexion, Freeline, Idorsia, Chiesi, Otsuka, Novo Nordisk, Sysmex, and Vifor Fresenius Medical Care Renal Pharma and is Director of the Catedra Mundipharma-UAM of diabetic kidney disease and the Catedra Astrazeneca-UAM of chronic kidney disease and electrolytes; leadership or fiduciary roles in board, society, committee, or advocacy groups, paid or unpaid, with European Renal Association; stock or stock options in Telara Farma; all outside the submitted work. C Palladino reports grants or contracts from FCT – Fundação para a Ciência e a Tecnologia, I.P. (national funding), under a contract-programme as defined by DL No. 57/2016 and Law No. 57/2017 (DL57/2016/CP1376/CT0004). DOI 10.54499/DL57/2016/CP1376/CT0004 (https://doi.org/10.54499/DL57/2016/CP1376/CT0004), outside the submitted work. R Passera reports participation on a Data Safety Monitoring Board or Advisory Board, unpaid, as senior biostatistician member of the Data Safety Monitoring Board for the no-profit clinical trial “Consolidation with ADCT-402 (loncastuximab tesirine) after immunochemotherapy: a phase II study in BTKi-treated/ineligible Relapse/Refractory Mantle Cell Lymphoma (MCL) patients” - sponsor FIL, Fondazione Italiana Linfomi, Alessandria-I; leadership or fiduciary roles in board, society, committee, or advocacy groups, unpaid, as Member of the Statistical Committee of the EBMT, the European Society for Bone and Marrow Transplantation, Paris-F; all outside the submitted work. A E Peden reports support for the present manuscript from the Australian National Health and Medical Research Council (grant number: APP2009306). V C F Pepito reports grants or contracts from Sanofi Consumer Healthcare and the International Initiative for Impact Evaluation; outside the submitted work. M Pigeolet reports a grant from The Belgian Kids' Fund for Pediatric Research, outside the submitted work. A Rane reports stock or stock options in Agios as an employee. L F Reyes reports grants or contracts from MSD, Pfizer, and GSK; consulting fees from GSK and MSD; payment or honoraria for lectures, presentations, speakers bureaus, manuscript writing, or educational events from GSK, Pfizer, and MSD; support for attending meetings and/or travel from GSK and Pfizer; all outside the submitted work. L Ronfani reports support for the present manuscript from the Italian Ministry of Health (Ricerca Corrente 34/2017), payments made to the Institute for Maternal and Child Health IRCCS Burlo Garofolo. S Sacco reports grants or contracts from Novartis and Uriach; consulting fees from Novartis, Allergan-Abbvie, Teva, Lilly, Lundbeck, Pfizer, Novo Nordisk, Abbott, and AstraZeneca; payment or honoraria for lectures, presentations, speakers bureaus, manuscript writing or educational events from Novartis, Allergan-AbbVie, Teva, Lilly, Lundbeck, Pfizer, Novo Nordisk, Abbott, and AstraZeneca; support for attending meetings and/or travel from Lilly, Novartis, Teva, and Lundbeck; leadership or fiduciary role in other board, society, committee, or advocacy group, paid or unpaid, with the European Stroke Organisation as President-elect and the European Headache Federation as Second Vice-President; receipt of equipment, materials, drugs, medical writing, gifts or other services from Allergan-AbbVie and Novo Nordisk; all outside the submitted work. P S Sachdev reports grants or contracts from National Health and Medical Research Council of Australia and the NIH; Payment or honoraria for lectures from Alkem Labs for the Frontiers of Psychiatry June 2023 Seminar, Mumbai, India; Participation on a Data Safety Monitoring Board or Advisory Board with Biogen Australia and Roche Australia; leadership or fiduciary role in other board, society, committee or advocacy group, unpaid, with the VASCOG Society and the World Psychiatric Association; all outside the submitted work. Y L Samodra reports grants or contracts from Taipei Medical University; leadership or fiduciary role in other board, society, committee, or advocacy group, paid or unpaid, with the Benang Merah Research Center; all outside the submitted work. J Sanabria reports support for attending meetings and/or travel from the Department of Surgery, Marshall University School of Medicine (MUSOM); three patents pending; participation in quality assessment and assurance for surgeries of the Department of Surgery MUSOM; leadership or fiduciary role in other board, society, committee or advocacy group, paid or unpaid with SSAT, ASTS, AHPBA, IHPBA, and AASLD; all outside the submitted work. N Scarmeas reports grants or contracts with Novo Nordisk as the Local Principal Investigator of recruiting site for multinational, multicentre industry sponsored phase 3 treatment trial for Alzheimer's disease with funding paid to the institution; Participation on a Data Safety Monitoring Board or Advisory Board with Albert Einstein College of Medicine (NIH-funded study) as the Chair of Data Safety Monitoring Board; all outside the submitted work. B M Schaarschmidt reports research grants from Else Kröner-Fresenius Foundatuin, DFG, and PharmaCept; payment or honoraria for lectures, presentations, speakers bureaus, manuscript writing, or educational events from AstraZeneca; support for attending meetings and/or travel from Bayer AG; all outside the submitted work. S Setoguchi reports grants or contracts paid to their institution from NIH, Cystic Fibrosis Foundation, Pfizer Inc, Daiichi Sankyo, and Bristol Myers Squibb; consulting fees from Pfizer Japan, Merck Inc, Bristol Myers Squibb, and Regeneron for providing scientific advise on study design and analytic approaches for validation studies, post-market safety studies and pre-market studies for medications and medical conditions; all outside the submitted work. A Sharifan reports leadership or fiduciary role in other board, society, committee or advocacy group, unpaid with Cochrane as a steering member of the Cochrane Early Career Professionals Network; and receipt of 30 days of complimentary access to ScienceDirect, Scopus, Reaxys, and Geofacets after reviewing manuscripts for two journals published by Elsevier; outside the submitted work. S Sharma reports support for the present manuscript from the John J Bonica Postdoctoral Fellowship from the International Association for the Study of Pain (IASP; 2021-2023); payment or honoraria for lectures, presentations, speakers bureaus, manuscript writing, or educational events and a travel grant for delivering a talk on “Technologies for pain education in developing countries” conducted by the Pain Education SIG of the IASP at the World Pain Congress in Toronto (2022); outside the submitted work. V Sharma reports other financial or non-financial support from DFSS (MHA)'s research project (DFSS28(1)2019/EMR/6) at Institute of Forensic Science & Criminology, Panjab University, Chandigarh, India, outside the submitted work. V Shivarov reports one patent and one utility model with the Bulgarian Patent Office; stock or stock options from ICONplc (RSUs); and other financial interests from an ICONplc salary; all outside the submitted work. S Shrestha reports other financial interests from the Graduate Research Merit Scholarship from the School of Pharmacy at Monash University Malaysia, outside the submitted work. J P Silva reports support for the present manuscript from the Portuguese Foundation for Science and Technology through payment of their salary (contract with reference 2021.01789.CEECIND/CP1662/CT0014). C R Simpson reports grants or contracts from MBIE (New Zealand), HRC (New Zealand), Ministry of Health (New Zealand), Medical Research Council (UK), and Chief Scientist Office (UK); leadership or fiduciary role in other board, society, committee, or advocacy group, paid or unpaid with the New Zealand Government Data Ethics Advisory Group as the Chair; outside the submitted work. J A Singh reports consulting fees from AstraZeneca, Crealta/Horizon, Medisys, Fidia, PK Med, Two labs Inc, Adept Field Solutions, Clinical Care options, Clearview healthcare partners, Putnam associates, Focus forward, Navigant consulting, Spherix, MedIQ, Jupiter Life Science, UBM LLC, Trio Health, Medscape, WebMD, and Practice Point communications; and the NIH and the American College of Rheumatology; payment for lectures, presentations, speakers bureaus, manuscript writing, or educational events as a member of the speaker's bureau of Simply Speaking; support for attending meetings and/or travel as a past steering committee member of OMERACT; participation on a Data Safety Monitoring Board or Advisory Board with the US Food and Drug Administration Arthritis Advisory Committee; leadership or fiduciary role in other board, society, committees or advocacy group, paid or unpaid, as a past steering committee member of the OMERACT, an international organisation that develops measures for clinical trials and receives arm's length funding from 12 pharmaceutical companies, Co-Chair of the Veterans Affairs Rheumatology Field Advisory Committee, and editor and Director of the UAB Cochrane Musculoskeletal Group Satellite Center on Network Meta-analysis; stock or stock options in Atai life sciences, Kintara therapeutics, Intelligent Biosolutions, Acumen pharmaceutical, TPT Global Tech, Vaxart pharmaceuticals, Atyu biopharma, Adaptimmune Therapeutics, GeoVax Labs, Pieris Pharmaceuticals, Enzolytics Inc, Seres Therapeutics, Tonix Pharmaceuticals Holding Corp, and Charlotte's Web Holdings Inc, and previously owned stock options in Amarin, Viking, and Moderna pharmaceuticals; outside the submitted work. S T Skou reports grants or contracts from European Research Council paid to their university from the EU's Horizon 2020 research innovation program (grant agreement No 801790), EU's Horizon 2020 research innovation program paid to their hospital (grant agreement No 945377), and Region Zealand paid to their hospital; royalties or licenses from Munksgaard for book chapters and TrustMe-Ed for online lectures; payment or honoraria for lectures, presentations, speakers bureaus, manuscript writing, or educational events from Nestlé Health Science for a presentation at a webinar on osteoarthritis; participation on a Data Safety Monitoring Board or Advisory Board, paid or unpaid, with UK-based National Institute for Health and Care Research (NIHR)-funded trial PERFORM: Personalised Exercise-Rehabilitation FOR people with Multiple long-term conditions (multimorbidity, NIHR 202020); other support as co-founder of GLA:D, a not-for profit initiative hosted at University of Southern Denmark; all outside the submitted work. D J Stein reports consulting fees from Discovery Vitality, Johnson & Johnson, Kanna, L'Oreal, Lundbeck, Orion, Sanofi, Servier, Takeda, and Vistagen, outside the submitted work. A P Stoian reports consulting fees paid to their institution from AstraZeneca, Boehringer Ingelheim, Eli Lilly, Novo Nordisk, Novartis, Sandoz, Sanofi, and Servier; payment or honoraria paid to their institution for lectures, presentations, speakers bureaus, manuscript writing, or educational events from AstraZeneca, Boehringer Ingelheim, Eli Lilly, Novo Nordisk, Novartis, Medochemie, Sandoz, Sanofi, and Servier; support for attending meetings and/or travel, paid to their institution, from Novo Nordisk, Medochemie, and Sanofi; participation on a Data Safety Monitoring Board or Advisory Board, paid to their institution, with AstraZeneca, Eli Lilly, Novo Nordisk, and Sanofi; leadership or fiduciary roles in board, society, committee or advocacy groups, unpaid, with Central European Diabetes Association (CEDA) and Association for Renal-Metabolic & Nutritional Studies (ASRMN); receipt of medical writing (unpaid) from Novo Nordisk and Sanofi; all outside the submitted work. C K Suemoto reports grants or contracts Alzheimer's Association and São Paulo Research Foundation (FAPESP), paid to their institution, and CNPq, paid to them; support for attending meetings and/or travel from Alzheimer's Association; leadership or fiduciary roles in other board, society, committee, or advocacy group, unpaid, with Brazilian Society of Geriatrics and Gerontology; all outside the submitted work. J H V Ticoalu reports a leadership or fiduciary role in other board, society, committee or advocacy group with Benang Merah Research Center as Co-founder, outside the submitted work. S J Tromans reports grants or contracts from the 2023 Adult Psychiatric Morbidity Survey team, collecting epidemiological data on community-based adults living in England. This is a contracted study from NHS Digital, via the Department of Health and Social Care; outside the submitted work. P Willeit reports consulting fees from Novartis; outside the submitted work. A Wimo reports grants or contracts from VINNOVA program: PREDEM, EU-project JPND: ADDITION, EU-project JPND: EURO-FINGER, EU-project IHI: PROMINENT, EU-project JPND: PMI-AD, and EU-project H2020: PRODEMOS; royalties or licenses as license holder of RUD-instrument (part); leadership or fiduciary roles in other board, society, committee, or advocacy group, unpaid, with ADI: MSAP; all outside the submitted work. M Zielińska reports other financial interest as an AstraZeneca employee, outside the submitted work. A Zumla reports grants or contracts from The Pan-African Network on Emerging and Re-Emerging Infections (PANDORA-ID-NET, CANTAM-3, and EACCR-3) funded by the European and Developing Countries Clinical Trials Partnership, the EU Horizon 2020 Framework Programme, UK NIHR Senior Investigator, and Mahathir Science Award and EU-EDCTP Pascoal Mocumbi Prize Laureate; participation on a Data Safety Monitoring Board or Advisory Board member of the WHO Mass Gatherings Expert Group and WHO Health Emergencies Programme in Geneva, a member of the EU-EDCTP3-Global Health (Brussells) Scientific Committee; all outside the submitted work.

## References

[1] Murray CJL (2022). The Global Burden of Disease Study at 30 years. Nat Med.

[2] World Bank (1993).

[3] GBD 2021 Causes of Death Collaborators (2024). Global burden of 288 causes of death and life expectancy decomposition in 204 countries and territories and 811 subnational locations, 1990–2021: a systematic analysis for the Global Burden of Disease Study 2021. Lancet.

[4] Soriano JB, Murthy S, Marshall JC, Relan P, Diaz JV (2022). A clinical case definition of post-COVID-19 condition by a Delphi consensus. Lancet Infect Dis.

[5] Institute for Health Metrics and Evaluation (2020). https://www.healthdata.org/research-analysis/about-gbd/protocol.

[6] Stevens GA, Alkema L, Black RE (2016). Guidelines for Accurate and Transparent Health Estimates Reporting: the GATHER statement. Lancet.

[7] Zheng P, Barber R, Sorensen RJD, Murray CJL, Aravkin AY (2021). Trimmed constrained mixed effects models: formulations and algorithms. J Comput Graph Stat.

[8] GBD 2019 Healthcare Access and Quality Collaborators (2022). Assessing performance of the Healthcare Access and Quality Index, overall and by select age groups, for 204 countries and territories, 1990–2019: a systematic analysis from the Global Burden of Disease Study 2019. Lancet Glob Health.

[9] Flaxman AD, Vos T, Murray CJL, Kiyono P (2015).

[10] Agency for Healthcare Research and Quality Medical Expenditure Panel Survey (MEPS). https://www.ahrq.gov/data/meps.html.

[11] National Institutes of Health (April 8, 2014). https://healthdata.gov/dataset/National-Epidemiologic-Survey-on-Alcohol-and-Relat/vq4w-x6us/about_data.

[12] Hasin DS, Grant BF (2015). The National Epidemiologic Survey on Alcohol and Related Conditions (NESARC) Waves 1 and 2: review and summary of findings. Soc Psychiatry Psychiatr Epidemiol.

[13] Australian Bureau of Statistics (1997). 4326.0 - Mental health and wellbeing: profile of adults, Australia. https://www.abs.gov.au/AUSSTATS/abs@.nsf/Lookup/4326.0Main+Features11997?OpenDocument=.

[14] Salomon JA, Haagsma JA, Davis A (2015). Disability weights for the Global Burden of Disease 2013 study. Lancet Glob Health.

[15] Salomon JA, Vos T, Hogan DR (2012). Common values in assessing health outcomes from disease and injury: disability weights measurement study for the Global Burden of Disease Study 2010. Lancet.

[16] COVID-19 Mental Disorders Collaborators (2021). Global prevalence and burden of depressive and anxiety disorders in 204 countries and territories in 2020 due to the COVID-19 pandemic. Lancet.

[17] GBD 2021 Demographics Collaborators (2024). Global age-sex-specific mortality, life expectancy, and population estimates in 204 countries and territories and 811 subnational locations, 1950–2021, and the impact of the COVID-19 pandemic: a comprehensive demographic analysis for the Global Burden of Disease Study 2021. Lancet.

[18] Johnson SC, Cunningham M, Dippenaar IN (2021). Public health utility of cause of death data: applying empirical algorithms to improve data quality. BMC Med Inform Decis Mak.

[19] GBD 2019 Demographics Collaborators (2020). Global age-sex-specific fertility, mortality, healthy life expectancy (HALE), and population estimates in 204 countries and territories, 1950–2019: a comprehensive demographic analysis for the Global Burden of Disease Study 2019. Lancet.

[20] Sullivan DF (1971). A single index of mortality and morbidity. HSMHA Health Rep.

[21] GBD 2019 Diseases and Injuries Collaborators (2020). Global burden of 369 diseases and injuries in 204 countries and territories, 1990–2019: a systematic analysis for the Global Burden of Disease Study 2019. Lancet.

[22] GBD 2017 SDG Collaborators (2018). Measuring progress from 1990 to 2017 and projecting attainment to 2030 of the health-related Sustainable Development Goals for 195 countries and territories: a systematic analysis for the Global Burden of Disease Study 2017. Lancet.

[23] COVID-19 Excess Mortality Collaborators (2022). Estimating excess mortality due to the COVID-19 pandemic: a systematic analysis of COVID-19-related mortality, 2020–21. Lancet.

[24] Wulf Hanson S, Abbafati C, Aerts JG (2022). Estimated global proportions of individuals with persistent fatigue, cognitive, and respiratory symptom clusters following symptomatic COVID-19 in 2020 and 2021. JAMA.

[25] COVID-19 Cumulative Infection Collaborators (2022). Estimating global, regional, and national daily and cumulative infections with SARS-CoV-2 through Nov 14, 2021: a statistical analysis. Lancet.

[26] COVID-19 Forecasting Team (2022). Variation in the COVID-19 infection–fatality ratio by age, time, and geography during the pre-vaccine era: a systematic analysis. Lancet.

[27] Flor LS, Friedman J, Spencer CN (2022). Quantifying the effects of the COVID-19 pandemic on gender equality on health, social, and economic indicators: a comprehensive review of data from March, 2020, to September, 2021. Lancet.

[28] Lazarus JV, Romero D, Kopka CJ (2022). A multinational Delphi consensus to end the COVID-19 public health threat. Nature.

[29] Bollyky TJ, Murray CJL, Reiner RC (2021). Epidemiology, not geopolitics, should guide COVID-19 vaccine donations. Lancet.

[30] GBD 2017 Diarrhoeal Disease Collaborators (2020). Quantifying risks and interventions that have affected the burden of diarrhoea among children younger than 5 years: an analysis of the Global Burden of Disease Study 2017. Lancet Infect Dis.

[31] Fauci AS, Lane HC (2020). Four decades of HIV/AIDS - much accomplished, much to do. N Engl J Med.

[32] UNAIDS (2023).

[33] GBD 2021 Diabetes Collaborators (2023). Global, regional, and national burden of diabetes from 1990 to 2021, with projections of prevalence to 2050: a systematic analysis for the Global Burden of Disease Study 2021. Lancet.

[34] GBD 2019 Mental Disorders Collaborators (2022). Global, regional, and national burden of 12 mental disorders in 204 countries and territories, 1990–2019: a systematic analysis for the Global Burden of Disease Study 2019. Lancet Psychiatry.

[35] Roth GA, Mensah GA, Johnson CO (2020). Global Burden of Cardiovascular Diseases and Risk Factors, 1990–2019: update from the GBD 2019 study. J Am Coll Cardiol.

[36] Vervoort D, Lee G, Lin Y, Contreras Reyes JR, Kanyepi K, Tapaua N (2022). 6 billion people have no access to safe, timely, and affordable cardiac surgical care. JACC Adv.

[37] Vervoort D, Swain JD, Pezzella AT, Kpodonu J (2021). Cardiac surgery in low- and middle-income countries: a state-of-the-art review. Ann Thorac Surg.

[38] GBD 2021 Low Back Pain Collaborators (2023). Global, regional, and national burden of low back pain, 1990–2020, its attributable risk factors, and projections to 2050: a systematic analysis of the Global Burden of Disease Study 2021. Lancet Rheumatol.

[39] Knezevic NN, Candido KD, Vlaeyen JWS, Van Zundert J, Cohen SP (2021). Low back pain. Lancet.

[40] Foster NE, Anema JR, Cherkin D (2018). Prevention and treatment of low back pain: evidence, challenges, and promising directions. Lancet.

[41] Patel V, Saxena S, Lund C (2018). The *Lancet* Commission on global mental health and sustainable development. Lancet.

[42] GBD 2019 Risk Factors Collaborators (2020). Global burden of 87 risk factors in 204 countries and territories, 1990–2019: a systematic analysis for the Global Burden of Disease Study 2019. Lancet.

[43] GBD 2016 Alcohol and Drug Use Collaborators (2018). The global burden of disease attributable to alcohol and drug use in 195 countries and territories, 1990–2016: a systematic analysis for the Global Burden of Disease Study 2016. Lancet Psychiatry.

[44] GBD 2016 Headache Collaborators (2018). Global, regional, and national burden of migraine and tension-type headache, 1990–2016: a systematic analysis for the Global Burden of Disease Study 2016. Lancet Neurol.

[45] Afshin A, Forouzanfar MH, Reitsma MB (2017). Health effects of overweight and obesity in 195 countries over 25 years. N Engl J Med.

[46] Teufel F, Seiglie JA, Geldsetzer P (2021). Body-mass index and diabetes risk in 57 low-income and middle-income countries: a cross-sectional study of nationally representative, individual-level data in 685 616 adults. Lancet.

[47] Santomauro DF, Purcell C, Whiteford HA, Ferrari AJ, Vos T (2023). Grading disorder severity and averted burden by access to treatment within the GBD framework: a case study with anxiety disorders. Lancet Psychiatry.

