# Supplementary material for: Global incidence, prevalence, years lived with disability (YLDs), disability-adjusted life-years (DALYs), and healthy life expectancy (HALE) for 371 diseases and injuries in 204 countries and territories and 811 subnational locations, 1990–2021: a systematic analysis for the Global Burden of Disease Study 2021
Source: Lancet. 2024 May 18;403(10440):2133–61. doi: 10.1016/S0140-6736(24)00757-8 (PMC11122111; doi:10.1016/S0140-6736(24)00757-8)
Supplement: Supplementary appendix 3 [file mmc3.pdf]

THE LANCET

Supplementary appendix 3

This appendix formed part of the original submission and has been peer reviewed. We post it as supplied by the authors.

Supplement to: GBD 2021 Diseases and Injuries Collaborators. Global incidence, prevalence, years lived with disability (YLDs), disability-adjusted life-years (DALYs), and healthy life expectancy (HALE) for 371 diseases and injuries in 204 countries and territories and 811 subnational locations, 1990–2021: a systematic analysis for the Global Burden of Disease Study 2021. *Lancet* 2024; **403:** 2133–61.

Appendix 3: Authorship appendix to “Global incidence, prevalence, years lived with disability (YLDs), disability-adjusted life-years (DALYs), and healthy life expectancy (HALE) for 371 diseases and injuries in 204 countries and territories and 811 subnational locations, 1990–2021: a systematic analysis for the Global Burden of Disease Study 2021”

This appendix provides further authorship detail for “Global incidence, prevalence, years lived with disability (YLDs), disability-adjusted life-years (DALYs), and healthy life expectancy (HALE) for 371 diseases and injuries in 204 countries and territories and 811 subnational locations, 1990–2021: a systematic analysis for the Global Burden of Disease Study 2021”

Table of Contents

GBD 2021 DALYs Collaborators 2

Affiliations 8

Authors’ Contributions........ 40

- Managing the overall research enterprise........ 40
- Writing the first draft of the manuscript 40
- Primary responsibility for applying analytical methods to produce estimates 40
- Primary responsibility for seeking, cataloguing, extracting, or cleaning data; designing or coding figures and tables........ 40
- Providing data or critical feedback on data sources........ 40
- Developing methods or computational machinery 43
- Providing critical feedback on methods or results 43
- Drafting the work or revising it critically for important intellectual content 48
- Managing the estimation or publications process........ 53

GBD 2021 DALYs Collaborators

Alize J Ferrari*, Damian Francesco Santomauro*, Amirali Aali, Yohannes Habtegiorgis Abate, Cristiana Abbafati, Hedayat Abbastabar, Samar Abd ElHafeez, Michael Abdelmasseh, Sherief Abd-Elsalam, Arash Abdollahi, Auwal Abdullahi, Kedir Hussein Abegaz, Roberto Ariel Abeldaño Zuñiga, Richard Gyan Aboagye, Hassan Abolhassani, Lucas Guimarães Abreu, Hasan Abualruz, Eman Abu-Gharbieh, Niveen ME Abu-Rmeileh, Ilana N Ackerman, Isaac Yeboah Addo, Giovanni Addolorato, Akindele Olupelumi Adebiyi, Abiola Victor Adepoju, Habeeb Omoponle Adewuyi, Shadi Afyouni, Saira Afzal, Sina Afzal, Antonella Agodi, Aqeel Ahmad, Danish Ahmad, Firdos Ahmad, Shahzaib Ahmad, Ali Ahmed, Luai A Ahmed, Muktar Beshir Ahmed, Marjan Ajami, Karolina Akinosoglou, Mohammed Ahmed Akkaif, Syed Mahfuz Al Hasan, Samer O Alalalmeh, Ziyad Al-Aly, Mohammed Albashtawy, Robert W Aldridge, Meseret Desalegn Alemu, Yihun Mulugeta Alemu, Kefyalew Addis Alene, Adel Ali Saeed Al-Gheethi, Maryam Alharrasi, Robert Kaba Alhassan, Mohammed Usman Ali, Rafat Ali, Syed Shujait Shujait Ali, Sheikh Mohammad Alif, Syed Mohamed Aljunid, Sabah Al-Marwani, Joseph Uy Almazan, Mahmoud A Alomari, Basem Al-Omari, Zaid Altaany, Nelson Alvis-Guzman, Nelson J Alvis-Zakzuk, Hassan Alwafi, Mohammad Sami Al-Wardat, Yaser Mohammed Al-Worafi, Safwat Aly, Karem H Alzoubi, Azmeraw T Amare, Prince M Amegbor, Edward Kwabena Ameyaw, Tarek Tawfik Amin, Alireza Amindarolzarbi, Sohrab Amiri, Dickson A Amugsi, Robert Ancuceanu, Deanna Anderlini, David B Anderson, Pedro Prata Andrade, Catalina Liliana Andrei, Hossein Ansari, Catherine M Antony, Saleha Anwar, Sumadi Lukman Anwar, Razique Anwer, Philip Emeka Anyanwu, Juan Pablo Arab, Jalal Arabloo, Mosab Arafat, Daniel T Araki, Aleksandr Y Aravkin, Mesay Arkew, Benedetta Armocida, Michael Benjamin Arndt, Mahwish Arooj, Anton A Artamonov, Raphael Taiwo Aruleba, Ashokan Arumugam, Charlie Ashbaugh, Mubarek Yesse Ashemo, Muhammad Ashraf, Marvellous O Asika, Elaheh Askari, Thomas Astell-Burt, Seyyed Shamsadin Athari, Prince Atorkey, Maha Moh'd Wahbi Atout, Alok Atreya, Avinash Aujayeb, Marcel Ausloos, Abolfazl Avan, Adedapo Wasiu Awotidebe, Kofi Awuviry-Newton, Beatriz Paulina Ayala Quintanilla, Jose L Ayuso-Mateos, Sina Azadnajafabad, Rui M S Azevedo, Abraham Samuel Babu, Muhammad Badar, Ashish D Badiye, Soroush Baghdadi, Nasser Bagheri, Sulaiman Bah, Ruhai Bai, Jennifer L Baker, Shankar M Bakkannavar, Abdulaziz T Bako, Senthilkumar Balakrishnan, Kiran Bam, Palash Chandra Banik, Martina Barchitta, Mainak Bardhan, Erfan Bardideh, Suzanne Lyn Barker-Collo, Hiba Jawdat Barqawi, Amadou Barrow, Sandra Barteit, Lingkan Barua, Somaye Bashiri Aliabadi, Afisu Basiru, Sanjay Basu, Saurav Basu, Prapthi Persis Bathini, Kavita Batra, Bernhard T Baune, Nebiyou Simegnew Bayileyegn, Babak Behnam, Amir Hossein Behnoush, Maryam Beiranvand, Diana Fernanda Bejarano Ramirez, Michelle L Bell, Olorunjuwon Omolaja Bello, Apostolos Beloukas, Isabela M Bensenor, Zombor Berezvai, Eduardo Bernabe, Robert S Bernstein, Paulo J G Bettencourt, Akshaya Srikanth Bhagavathula, Neeraj Bhala, Dinesh Bhandari, Ashish Bhargava, Sonu Bhaskar, Vivek Bhat, Gurjit Kaur Bhatti, Jasvinder Singh Bhatti, Manpreet S Bhatti, Rajbir Bhatti, Zulfiqar A Bhutta, Boris Bikbov, Jessica Devin Bishai, Catherine Bisignano, Veera R Bitra, Tone Bjørge, Virginia Bodolica, Aadam Olalekan Bodunrin, Eyob Ketema Bogale, Milad Bonakdar Hashemi, Aime Bonny, Berrak Bora Basara, Hamed Borhany, Christopher Boxe, Oliver J Brady, Nicola Luigi Bragazzi, Dejana Braithwaite, Luisa C Brant, Michael Brauer, Susanne Breitner, Hermann Brenner, Julie Brown, Traolach Brugha, Norma B Bulamu, Danilo Buonsenso, Katrin Burkart, Richard A Burns, Reinhard Busse, Yasser Bustanji, Zahid A Butt, Justin Byun, Florentino Luciano Caetano dos Santos, Daniela Calina, Luis Alberto Cámera, Ismael R Campos-Nonato, Chao Cao, Angelo Capodici, Sinclair Carr, Giulia Carreras, Andrea Carugno, Márcia Carvalho, Joao Mauricio Castaldelli-Maia, Carlos A Castañeda-Orjuela, Giulio Castelpietra, Alberico L Catapano, Maria Sofia Cattaruzza, Arthur Caye, Luca Cegolon, Francieli Cembranel, Muthia Cenderadewi, Ester Cerin, Promit Ananyo Chakraborty, Jeffrey Shi Kai Chan,

Raymond N C Chan, Rama Mohan Chandika, Eeshwar K Chandrasekar, Periklis Charalampous, Vijay Kumar Chattu, Victoria Chatzimavridou-Grigoriadou, Angela W Chen, An-Tian Chen, Catherine S Chen, Haowei Chen, Nathalie M Chen, Esther T W Cheng, Odgerel Chimed-Ochir, Ritesh Chimoriya, Patrick R Ching, William C S Cho, Sungchul Choi, Bryan Chong, Yuen Yu Chong, Sonali Gajanan Choudhari, Rajiv Chowdhury, Steffan Wittrup McPhee Christensen, Dinh-Toi Chu, Isaac Sunday Chukwu, Eric Chung, Eunice Chung, Muhammad Chutiyami, Mareli M Claassens, Rebecca M Cogen, Alyssa Columbus, Joao Conde, Paolo Angelo Cortesi, Ewerton Cousin, Michael H Criqui, Natália Cruz-Martins, Omid Dadras, Siyu Dai, Xiaochen Dai, Zhaoli Dai, Maxwell Ayindenaba Dalaba, Giovanni Damiani, Jai K Das, Saswati Das, Mohsen Dashti, Claudio Alberto Dávila-Cervantes, Kairat Davletov, Diego De Leo, Aklilu Tamire Debele, Shayom Debopadhaya, Nicole K DeCleene, Farah Deeba, Louisa Degenhardt, Cristian Del Bo', Ivan Delgado-Enciso, Andreas K Demetriades, Edgar Denova-Gutiérrez, Nikolaos Dervenis, Hardik Dineshbhai Desai, Rupak Desai, Keshab Deuba, Kuldeep Dhama, Samath Dhamminda Dharmaratne, Sameer Dhingra, Diana Dias da Silva, Daniel Diaz, Luis Antonio Diaz, Michael J Diaz, Adriana Dima, Delaney D Ding, M Ashworth Dirac, Thao Huynh Phuong Do, Camila Bruneli do Prado, Sushil Dohare, Regina-Mae Villanueva Dominguez, Wanyue Dong, Deepa Dongarwar, Mario D'Oria, E Ray Dorsey, Leila Doshmangir, Robert Kokou Dowou, Tim Robert Driscoll, Haneil Larson Dsouza, Viola Dsouza, John Dube, Samuel C Dumith, Bruce B Duncan, Andre Rodrigues Duraes, Senbagam Duraisamy, Oyewole Christopher Durojaiye, Paulina Agnieszka Dzianach, Arkadiusz Marian Dziedzic, Ejemai Eboreime, Alireza Ebrahimi, Hisham Atan Edinur, David Edvardsson, Terje Andreas Eikemo, Ebrahim Eini, Michael Ekholuenetale, Temitope Cyrus Ekundayo, Iman El Sayed, Maha El Tantawi, Iffat Elbarazi, Noha Mousaad Elemam, Ghada Metwally Tawfik ElGohary, Muhammed Elhadi, Omar Abdelsadek Abdou Elmeligy, Gihan ELNahas, Mohammed Elshaer, Ibrahim Elsohaby, Luchuo Engelbert Bain, Ryenchindorj Erkhembayar, Babak Eshrati, Kara Estep, Natalia Fabin, Adeniyi Francis Fagbamigbe, Luca Falzone, Mohammad Fareed, Carla Sofia e Sá Farinha, MoezAlIslam Ezzat Mahmoud Faris, Andre Faro, Pegah Farrokhi, Ali Fatehizadeh, Nelsensius Klau Fauk, Valery L Feigin, Xiaoqi Feng, Seyed-Mohammad Fereshtehnejad, Abdullah Hamid Feroze, Nuno Ferreira, Paulo H Ferreira, Florian Fischer, Joanne Flavel, David Flood, Luisa S Flor, Nataliya A Foigt, Morenike Oluwatoyin Folayan, Lisa M Force, Daniela Fortuna, Matteo Foschi, Richard Charles Franklin, Alberto Freitas, Takeshi Fukumoto, João M Furtado, Peter Andras Gaal, Muktar A Gadanya, Abhay Motiramji Gaidhane, Santosh Gaihre, Yaseen Galali, Mandukhai Ganbat, Aravind P Gandhi, Balasankar Ganesan, Mohd Ashraf Ganie, Mohammad Arfat Ganiyani, William M Gardner, Tilaye Gebru Gebi, Miglas W Gebregergis, Mesfin Gebrehiwot, Tesfay B B Gebremariam, Teferi Gebru Gebremeskel, Yibeltal Yismaw Gela, Simona Roxana Georgescu, Abera Getachew Obsa, Peter W Gething, Molla Getie, Keyghobad Ghadiri, Fataneh Ghadirian, Khalid Yaser Ghailan, Alireza Ghajar, MohammadReza Ghasemi, Ghazal Ghasempour Dabaghi, Afsaneh Ghasemzadeh, Ramy Mohamed Ghazy, Ali Gholamrezanezhad, Mahsa Ghorbani, Elena Ghotbi, Ruth Margaret Gibson, Tiffany K Gill, Themba G Ginindza, Alem Girmay, James C Glasbey, Laszlo Göbölös, Myron Anthony Godinho, Salime Goharinezhad, Mohamad Goldust, Mahaveer Golechha, Pouya Goleij, Philimon N Gona, Giuseppe Gorini, Alessandra C Goulart, Ayman Grada, Michal Grivna, Shi-Yang Guan, Giovanni Guarducci, Mohammed Ibrahim Mohialdeen Gubari, Mesay Dechasa Gudeta, Avirup Guha, Stefano Guicciardi, Snigdha Gulati, David Gulisashvili, Damitha Asanga Gunawardane, Cui Guo, Anish Kumar Gupta, Bhawna Gupta, Ishita Gupta, Mohak Gupta, Rajeev Gupta, Veer Bala Gupta, Vijai Kumar Gupta, Vivek Kumar Gupta, Reyna Alma Gutiérrez, Farrokh Habibzadeh, Parham Habibzadeh, Rasool Haddadi, Najah R Hadi, Nils Haep, Nima Hafezi-Nejad, Abdul Hafiz, Hailey Hagins, Esam S Halboub, Aram Halimi, Sebastian Haller, Rabih Halwani, Erin B Hamilton, Graeme J Hankey, Md Abdul Hannan, Md Nuruzzaman Haque, Harapan Harapan, Josep Maria Haro, Jan

Hartvigsen, Ahmed I Hasaballah, Ikramul Hasan, Mohammad Hasanian, Md Saquib Hasnain, Amr Hassan, Johannes Haubold, Rasmus J Havmoeller, Simon I Hay, Khezar Hayat, Jeffrey J Hebert, Omar E Hegazi, Golnaz Heidari, Bartosz Helfer, Mehdi Hemmati, Delia Hendrie, Claire A Henson, Kamal Hezam, Yuta Hiraike, Nguyen Quoc Hoan, Ramesh Holla, Julia Hon, Md Mahbub Hossain, Hassan Hosseinzadeh, Mehdi Hosseinzadeh, Mihaela Hostiuc, Sorin Hostiuc, Johnathan M Hsu, Junjie Huang, Fernando N Hugo, Kiavash Hushmandi, Javid Hussain, Nawfal R Hussein, Chantal K Huynh, Hong-Han Huynh, Bing-Fang Hwang, Vincent C Iannucci, Audrey L Ihler, Adalia I Ikiroma, Kevin S Ikuta, Olayinka Stephen Ilesanmi, Irena M Ilic, Milena D Ilic, Mohammad Tarique Imam, Mustapha Immurana, Lalu Muhammad Irham, Md Rabiul Islam, Sheikh Mohammed Shariful Islam, Farhad Islami, Faisal Ismail, Nahlah Elkudssiah Ismail, Gaetano Isola, Masao Iwagami, Chidozie C D Iwu, Mahalaxmi Iyer, Jalil Jaafari, Kathryn H Jacobsen, Farhad Jadidi-Niaragh, Morteza Jafarinia, Khushleen Jaggi, Kasra Jahankhani, Nader Jahanmehr, Haitham Jahrami, Akhil Jain, Nityanand Jain, Ammar Abdulrahman Jairoun, Abhishek Jaiswal, Mihajlo Jakovljevic, Abubakar Ibrahim Jatau, Sabzali Javadov, Tahereh Javaheri, Sathish Kumar Jayapal, Shubha Jayaram, Sun Ha Jee, Jayakumar Jeganathan, Angeline Jeyakumar, Anil K Jha, Heng Jiang, Yinzi Jin, Jost B Jonas, Tamas Joo, Abel Joseph, Nitin Joseph, Charity Ehimwenma Joshua, Jacek Jerzy Jozwiak, Mikk Jürisson, Vaishali K, Billingsley Kaambwa, Ali Kabir, Zubair Kabir, Vidya Kadashetti, Rizwan Kalani, Leila R Kalankesh, Feroze Kaliyadan, Sanjay Kalra, Kaloyan Kamenov, Naser Kamyari, Thanigaivelan Kanagasabai, Himal Kandel, Arun R Kanmanthareddy, Kehinde Kazeem Kanmodi, Rami S Kantar, Ibraheem M Karaye, Asima Karim, Salah Eddin Karimi, Yeganeh Karimi, Hengameh Kasraei, Molly B Kassel, Joonas H Kauppila, Norito Kawakami, Gbenga A Kayode, Foad Kazemi, Sina Kazemian, Leila Keikavoosi-Arani, Cathleen Keller, John H Kempen, Jessica A Kerr, Kamyab Keshtkar, Emmanuelle Kesse-Guyot, Mohammad Keykhaei, Himanshu Khajuria, Amirmohammad Khalaji, Asaad Khalid, Nauman Khalid, Alireza Khalilian, Faham Khamesipour, Asaduzzaman Khan, Ikramullah Khan, Maseer Khan, Moien AB Khan, Shaghayegh Khanmohammadi, Khaled Khatab, Fatemeh Khatami, Moawiah Mohammad Khatatbeh, Amir M Khater, Hamid Reza Khayat Kashani, Feriha Fatima Khidri, Elaheh Khodadoust, Moein Khormali, Zahra Khorrami, Zemene Demelash Kifle, Min Seo Kim, Ruth W Kimokoti, Adnan Kisa, Sezer Kisa, Ann Kristin Skrindo Knudsen, Jonathan M Kocarnik, Sonali Kochhar, Hyun Yong Koh, Ali-Asghar Kolahi, Farzad Kompani, Gerbrand Koren, Oleksii Korzh, Soewarta Kosen, Sindhura Lakshmi Koulmane Laxminarayana, Kewal Krishan, Varun Krishna, Vijay Krishnamoorthy, Barthelemy Kuate Defo, Md Abdul Kuddus, Mohammed Kuddus, Ilari Kuitunen, Vishnutheertha Kulkarni, Manasi Kumar, Nithin Kumar, Rakesh Kumar, Om P Kurmi, Dian Kusuma, Hmwe Hmwe Kyu, Carlo La Vecchia, Ben Lacey, Muhammad Awwal Ladan, Lucie Laflamme, Alessandra Lafranconi, Chandrakant Lahariya, Daphne Teck Ching Lai, Dharmesh Kumar Lal, Ratilal Lalloo, Tea Lallukka, Judit Lám, Qing Lan, Tuo Lan, Iván Landires, Francesco Lanfranchi, Berthold Langguth, Ariane Laplante-Lévesque, Bagher Larijani, Anders O Larsson, Savita Lasrado, Paolo Lauriola, Huu-Hoai Le, Long Khanh Dao Le, Nhi Huu Hanh Le, Trang Diep Thanh Le, Janet L Leasher, Caterina Ledda, Munjae Lee, Paul H Lee, Sang-woong Lee, Seung Won Lee, Wei-Chen Lee, Yo Han Lee, Kate E LeGrand, Jacopo Lenzi, Elvynna Leong, Janni Leung, Ming-Chieh Li, Wei Li, Xiaopan Li, Yichong Li, Yongze Li, Lee-Ling Lim, Stephen S Lim, Megan Lindstrom, Shai Linn, Gang Liu, Runben Liu, Shiwei Liu, Wei Liu, Xiaofeng Liu, Xuefeng Liu, Erand Llanaj, Chun-Han Lo, Rubén López-Bueno, Arianna Maever Loreche, László Lorenzovici, Rafael Lozano, Jailos Lubinda, Giancarlo Lucchetti, Raimundas Lunevicius, Jay B Lusk, hengliang lv, Zheng Feei Ma, Nikolaos Machairas, Áurea M Madureira-Carvalho, Javier A Magaña Gómez, Azzam A Maghazachi, Preeti Maharjan, Phetole Walter Mahasha, Mina Maheri, Soleiman Mahjoub, Mansour Adam Mahmoud, Elham Mahmoudi, Azeem Majeed, Konstantinos Christos Makris, Elaheh Malakan Rad, Kashish Malhotra, Ahmad Azam Malik, Iram Malik, Deborah Carvalho Malta, Yosef Manla,

Ali Mansour, Pejman Mansouri, Mohammad Ali Mansournia, Ana M Mantilla Herrera, Lorenzo Giovanni Mantovani, Emmanuel Manu, Hamid Reza Marateb, Parham Mardi, Gabriel Martinez, Ramon Martinez-Piedra, Daniela Martini, Francisco Rogerlândio Martins-Melo, Miquel Martorell, Wolfgang Marx, Sharmeen Maryam, Roy Rillera Marzo, Yasith Mathangasinghe, Stephanie Mathieson, Alexander G Mathioudakis, Jishanth Mattumpuram, Andrea Maugeri, Mahsa Mayeli, Mohsen Mazidi, Antonio Mazzotti, John J McGrath, Martin McKee, Anna Laura W McKowen, Michael A McPhail, Kamran Mehrabani-Zeinabad, Entezar Mehrabi Nasab, Tesfahun Mekene Meto, Walter Mendoza, Ritesh G Menezes, George A Mensah, Alexios-Fotios A Mentis, Sultan Ayoub Meo, Haftu Asmerom Meresa, Atte Meretoja, Tuomo J Meretoja, Abera M Mersha, Tomislav Mestrovic, Kukulege Chamila Dinushi Mettananda, Sachith Mettananda, Irmina Maria Michalek, Paul Anthony Miller, Ted R Miller, Edward J Mills, Le Huu Nhat Minh, Antonio Mirijello, Erkin M Mirrakhimov, Mizan Kiros Mirutse, Mohammad Mirza-Aghazadeh-Attari, Maryam Mirzaei, Roya Mirzaei, Awoke Misganaw, Ajay Kumar Mishra, Philip B Mitchell, Chaitanya Mittal, Babak Moazen, Madeline E Moberg, Jama Mohamed, Mouhand F H Mohamed, Nouh Saad Mohamed, Esmaeil Mohammadi, Soheil Mohammadi, Hussen Mohammed, Salahuddin Mohammed, Shafiu Mohammed, Robin M Mohr, Ali H Mokdad, Sabrina Molinaro, Sara Momtazmanesh, Lorenzo Monasta, Stefania Mondello, AmirAli Moodi Ghalibaf, Maryam Moradi, Yousef Moradi, Maziar Moradi-Lakeh, Paula Moraga, Lidia Morawska, Rafael Silveira Moreira, Negar Morovatdar, Shane Douglas Morrison, Jakub Morze, Abbas Mosapour, Jonathan F Mosser, Elias Mossialos, Rohith Motappa, Vincent Mougin, Simin Mouodi, Matías Mrejen, Ahmed Msherghi, Sumaira Mubarik, Ulrich Otto Mueller, Francesk Mulita, Kavita Munjal, Efrén Murillo-Zamora, BV Murlimanju, Ghulam Mustafa, Sathish Muthu, Muhammad Muzaffar, Woojae Myung, Ahamarshan Jayaraman Nagarajan, Pirouz Naghavi, Ganesh R Naik, Firzan Nainu, Sanjeev Nair, Hastyar Hama Rashid Najmuldeen, Vinay Nangia, Atta Abbas Naqvi, Aparna Ichalangod Narayana, Shumaila Nargus, Gustavo G Nascimento, Abdulqadir J Nashwan, Ali Nasrollahizadeh, Amir Nasrollahizadeh, Zuhair S Natto, Biswa Prakash Nayak, Vinod C Nayak, Sabina Onyinye Nduaguba, Hadush Negash, Ionut Negoi, Ruxandra Irina Negoi, Seyed Aria Nejadghaderi, Olivia D Nesbit, Henok Biresaw Netsere, Marie Ng, Georges Nguefack-Tsague, Josephine W Ngunjiri, Dang H Nguyen, Hien Quang Nguyen, Robina Khan Niazi, Taxiarchis Konstantinos Nikolouzakis, Ali Nikoobar, Fatemeh Nikoomanesh, Amin Reza Nikpoor, Chukwudi A Nnaji, Lawrence Achilles Nnyanzi, Efaq Ali Noman, Shuhei Nomura, Bo Norrving, Chisom Adaobi Nri-Ezedi, George Ntaios, Mpiko Ntsekhe, Dieta Nurrika, Chimezie Igwegbe Nzoputam, Ogochukwu Janet Nzoputam, Bogdan Oancea, Ismail A Odetokun, Martin James O'Donnell, Ayodipupo Sikiru Oguntade, James Odhiambo Oguta, Hassan Okati-Aliabad, Sylvester Reuben Okeke, Akinkunmi Paul Okekunle, Osaretin Christabel Okonji, Andrew T Olagunju, Omotola O Olasupo, Matthew Idowu Olatubi, Gláucia Maria Moraes Oliveira, Isaac Iyinoluwa Olufadewa, Bolajoko Olubukunola Olusanya, Jacob Olusegun Olusanya, Hany A Omar, Goran Latif Omer, Abidemi E Emmanuel Omonisi, Sandersan Onie, Obinna E Onwujekwe, Michal Ordak, Verner N Orish, Doris V Ortega-Altamirano, Alberto Ortiz, Edgar Ortiz-Brizuela, Wael M S Osman, Samuel M Ostroff, Uchechukwu Levi Osuagwu, Adrian Otoiu, Nikita Otstavnov, Stanislav S Otstavnov, Amel Ouyahia, Guoqing Ouyang, Mayowa O Owolabi, Mahesh Padukudru P A, Alicia Padron-Monedero, Jagadish Rao Padubidri, Tamás Palicz, Claudia Palladino, Feng Pan, Seithikurippu R Pandi-Perumal, Helena Ullyartha Pangaribuan, Georgios D Panos, Leonidas D Panos, Anca Mihaela Pantea Stoian, Shahina Pardhan, Romil R Parikh, Ava Pashaei, Maja Pasovic, Roberto Passera, Jay Patel, Sangram Kishor Patel, Shankargouda Patil, Dimitrios Patoulias, Venkata Suresh Patthipati, Shrikant Pawar, Hamidreza Pazoki Toroudi, Spencer A Pease, Amy E Peden, Paolo Pedersini, Minjin Peng, Umberto Pensato, Veincent Christian Filipino Pepito, Emmanuel K Peprah, Prince Peprah,

João Perdigão, Maria Odete Pereira, Arokiasamy Perianayagam, Norberto Perico, Konrad Pesudovs, Fanny Emily Petermann-Rocha, William A Petri, Hoang Tran Pham, Anil K Philip, Michael R Phillips, Manon Pigeolet, David M Pigott, Julian David Pillay, Zahra Zahid Piracha, Saeed Pirouzpanah, Dietrich Plass, Evgenii Plotnikov, Dimitri Poddighe, Suzanne Polinder, Maarten J Postma, Naeimeh Pourtaheri, Sergio I Prada, Pranil Man Singh Pradhan, V Prakash, Manya Prasad, Elton Junio Sady Prates, Tina Priscilla, Natalie Pritchett, Pooja Puri, Jagadeesh Puvvula, Nameer Hashim Qasim, Ibrahim Qattea, Asma Saleem Qazi, Gangzhen Qian, Mehrdad Rabiee Rad, Raghu Anekal Radhakrishnan, Venkatraman Radhakrishnan, Hadi Raeisi Shahraki, Quinn Rafferty, Alberto Raggi, Pankaja Raghav Raghav, Md Jillur Rahim, Md Mosfequr Rahman, Mohammad Hifz Ur Rahman, Mosiur Rahman, Muhammad Aziz Rahman, Shayan Rahmani, Mohammad Rahmanian, Setyaningrum Rahmawaty, Sathish Rajaa, Mahmoud Mohammed Ramadan, Shakthi Kumaran Ramasamy, Premkumar Ramasubramani, Sheena Ramazanu, Kritika Rana, Chhabi Lal Ranabhat, Nemanja Rancic, Amey Rane, Chythra R Rao, Kumuda Rao, Mithun Rao, Sowmya J Rao, Mohammad-Mahdi Rashidi, Giridhara Rathnaiah Babu, Santosh Kumar Rauniyar, David Laith Rawaf, Salman Rawaf, Christian Razo, Murali Mohan Rama Krishna Reddy, Elrashdy Moustafa Mohamed Redwan, Lennart Reifels, Robert C Reiner Jr, Giuseppe Remuzzi, Andre M N Renzaho, Bhageerathy Reshmi, Luis Felipe Reyes, Nazila Rezaei, Negar Rezaei, Nima Rezaei, Peyman Rezaei Hachesu, Mohsen Rezaeian, Jennifer Rickard, Célia Fortuna Rodrigues, Jefferson Antonio Buendia Rodriguez, Leonardo Roever, Luca Ronfani, Gholamreza Roshandel, Kunle Rotimi, Himanshu Sekhar Rout, Bedanta Roy, Nitai Roy, Priyanka Roy, Enrico Rubagotti, Chandan S N, Aly M A Saad, Maha Mohamed Saber-Ayad, Siamak Sabour, Simona Sacco, Perminder S Sachdev, Basema Saddik, Adam Saddler, Bashdar Abuzed Sadee, Erfan Sadeghi, Masoumeh Sadeghi, Mohammad Reza Saeb, Umar Saeed, Sher Zaman Safi, Rajesh Sagar, Dominic Sagoe, Zahra Saif, Mirza Rizwan Sajid, Joseph W Sakshaug, Nasir Salam, Afeez Abolarinwa Salami, Luciane B Salaroli, Mohamed A Saleh, Marwa Rashad Salem, Mohammed Z Y Salem, Malik Sallam, Sara Samadzadeh, Saad Samargandy, Yoseph Leonardo Samodra, Abdallah M Samy, Juan Sanabria, Francesca Sanna, Itamar S Santos, Milena M Santric-Milicevic, Made Ary Sarasmita, Yaser Sarikhani, Rodrigo Sarmiento-Suárez, Gargi Sachin Sarode, Sachin C Sarode, Arash Sarveazad, Brijesh Sathian, Anudeep Sathyanarayan, Maheswar Satpathy, Monika Sawhney, Nikolaos Scarmeas, Benedikt Michael Schaarschmidt, Maria Inês Schmidt, Ione Jayce Ceola Schneider, Austin E Schumacher, David C Schwebel, Falk Schwendicke, Mansour Sedighi, Sabyasachi Senapati, Subramanian Senthilkumaran, Sadaf G Sepanlou, Yashendra Sethi, Soko Setoguchi, Allen Seylani, Jamileh Shadid, Mahan Shafie, Humaira Shah, Nilay S Shah, Pritik A Shah, Ataollah Shahbandi, Samiah Shahid, Wajeehah Shahid, Moyad Jamal Shahwan, Masood Ali Shaikh, Alireza Shakeri, Ali S Shalash, Sunder Sham, Muhammad Aaqib Shamim, Mohammad Ali Shamshirgaran, Mohammad Anas Shamsi, Mohd Shanawaz, Abhishek Shankar, Mohammed Shannawaz, Medha Sharath, Amin Sharifan, Javad Sharifi-Rad, Manoj Sharma, Rajesh Sharma, Saurab Sharma, Ujjawal Sharma, Vishal Sharma, Rajesh P Shastry, Amin Shavandi, Amir Mehdi Shayan, Maryam Shayan, Amr Mohamed Elsayed Shehabeldine, Pavanchand H Shetty, Kenji Shibuya, Jemal Ebrahim Shifa, Desalegn Shiferaw, Wondimeneh Shibabaw Shiferaw, Mika Shigematsu, Rahman Shiri, Nebiyu Aniley Shitaye, Aminu Shittu, K M Shivakumar, Velizar Shivarov, Zahra Shokati Eshkiki, Sina Shool, Sunil Shrestha, Kerem Shuval, Migbar Mekonnen Sibhat, Emmanuel Edwar Siddig, Inga Dora Sigfusdottir, Diego Augusto Santos Silva, João Pedro Silva, Luís Manuel Lopes Rodrigues Silva, Soraia Silva, Colin R Simpson, Anjali Singal, Abhinav Singh, Balbir Bagicha Singh, Harmanjit Singh, Jasvinder A Singh, Mahendra Singh, Paramdeep Singh, Søren T Skou, David A Sleet, Erica Leigh N Slepak, Ranjan Solanki, Sameh S M Soliman, Suhang Song, Yimeng Song, Reed J D Sorensen, Joan B Soriano, Ireneous N Soyiri, Michael Spartalis, Chandrashekhar T

Sreeramareddy, Benjamin A Stark, Antonina V Starodubova, Caroline Stein, Dan J Stein, Caitlyn Steiner, Timothy J Steiner, Jaimie D Steinmetz, Paschalis Steiropoulos, Leo Stockfelt, Mark A Stokes, Narayan Subedi Subedi, Vetriselvan Subramaniyan, Claudia Kimie Suemoto, Muhammad Suleman, Rizwan Suliankatchi Abdulkader, Abida Sultana, Johan Sundström, Chandan Kumar Swain, Lukasz Szarpak, Payam Tabaee Damavandi, Rafael Tabarés-Seisdedos, Ozra Tabatabaei Malazy, Seyed-Amir Tabatabaeizadeh, Shima Tabatabai, Celine Tabche, Mohammad Tabish, Santosh Kumar Tadakamadla, Yasaman Taheri Abkenar, Moslem Taheri Soodejani, Amir Taherkhani, Jabeen Taiba, Iman M Talaat, Ashis Talukder, Mircea Tampa, Jacques Lukenze Tamuzi, Ker-Kan Tan, Sarmila Tandukar, Haosu Tang, Razieh Tavakoli Oliaee, Seyed Mohammad Tavangar, Mojtaba Teimoori, Mohamad-Hani Temsah, Masayuki Teramoto, Pugazhenthan Thangaraju, Kavumpurathu Raman Thankappan, Rekha Thapar, Rasiah Thayakaran, Sathish Thirunavukkarasu, Nihal Thomas, Nikhil Kenny Thomas, Chern Choong Chern Thum, Ales Tichopad, Jansje Henny Vera Ticoalu, Tala Tillawi, Tenaw Yimer Tiruye, Ruoyan Tobe-Gai, Marcello Tonelli, Roman Topor-Madry, Anna E Torre, Mathilde Touvier, Marcos Roberto Tovani-Palone, Jasmine T Tran, Mai Thi Ngoc Tran, Nghia Minh Tran, Ngoc-Ha Tran, Domenico Trico, Samuel Joseph Tromans, Thien Tan Tri Tai Truyen, Aristidis Tsatsakis, Guesh Mebrahtom Tsegay, Evangelia Eirini Tsermpini, Munkhtuya Tumurkhuu, Stefanos Tyrovolas, Arit Udoh, Muhammad Umair, Srikanth Umakanthan, Tungki Pratama Umar, Eduardo A Undurraga, Brigid Unim, Bhaskaran Unnikrishnan, Carolyn Anne Unsworth, Era Upadhyay, Daniele Urso, Jibrin Sammani Usman, Seyed Mohammad Vahabi, Asokan Govindaraj Vaithinathan, Jef Van den Eynde, Orsolya Varga, Ravi Prasad Varma, Priya Vart, Tommi Juhani Vasankari, Milena Vasic, Siavash Vaziri, Balachandar Vellingiri, Narayanaswamy Venketasubramanian, Massimiliano Veroux, Georgios-Ioannis Verras, Dominique Vervoort, Jorge Hugo Villafañe, Francesco S Violante, Vasily Vlassov, Stein Emil Vollset, Simona Ruxandra Volovat, Avina Vongpradith, Yasir Waheed, Cong Wang, Fang Wang, Ning Wang, Shu Wang, Yanzhong Wang, Yuan-Pang Wang, Paul Ward, Emebet Gashaw Wassie, Marcia R Weaver, Kosala Gayan Weerakoon, Robert G Weintraub, Daniel J Weiss, Abrha Hailay Weldemariam, Katherine M Wells, Yi Feng Wen, Joanna L Whisnant, Harvey A Whiteford, Taweewat Wiangkham, Dakshitha Praneeth Wickramasinghe, Nuwan Darshana Wickramasinghe, Angga Wilandika, Caroline Wilkerson, Peter Willeit, Anders Wimo, Demewoz H Woldegebreal, Axel Walter Wolf, Yen Jun Wong, Anthony D Woolf, Chenkai Wu, Felicia Wu, Xinsheng Wu, Zenghong Wu, Sarah Wulf Hanson, Yanjie Xia, Hong Xiao, Xiaoyue Xu, Yvonne Yiru Xu, Lalit Yadav, Ali Yadollahpour, Sajad Yaghoubi, Kazumasa Yamagishi, Lin Yang, Yuichiro Yano, Yao Yao, Habib Yaribeygi, Mohammad Hosein Yazdanpanah, Pengpeng Ye, Sisay Shewasinad Yehualashet, Subah Abderehim Yesuf, Saber Yezli, Arzu Yiğit, Vahit Yiğit, Zeamanuel Anteneh Yigzaw, Yazachew Yismaw, Dong Keon Yon, Naohiro Yonemoto, Mustafa Z Younis, Chuanhua Yu, Yong Yu, Hadiza Yusuf, Mondal Hasan Zahid, Fathiah Zakham, Leila Zaki, Nazar Zaki, Burhan Abdullah Zaman, Nelson Zamora, Ramin Zand, Ghazal G Z Zandieh, Heather J Zar, Armin Zarrintan, Mikhail Sergeevich Zastrozhin, Haijun Zhang, Ning Zhang, Yunquan Zhang, Hanqing Zhao, Chenwen Zhong, Panliang Zhong, Juexiao Zhou, Zhaohua Zhu, Makan Ziafati, Magdalena Zielińska, Stephanie R M Zimsen, Mohammad Zoladl, Alimuddin Zumla, Samer H Zyoud, Theo Vos†, and Christopher J L Murray†.

*Joint first authors

†Joint senior authors

Affiliations

School of Public Health (A J Ferrari PhD, D F Santomauro PhD, A M Mantilla Herrera PhD, P A Miller PhD, J Shadid BSc, Prof H A Whiteford PhD), Centre for Sensorimotor Performance (D Anderlini MD), Department of Urology (Prof E Chung MD), School of Health and Rehabilitation Sciences (A Khan PhD), School of Dentistry (R Lalloo PhD), Queensland Brain Institute (Prof J J McGrath MD), The University of Queensland, Brisbane, QLD, Australia; Institute for Health Metrics and Evaluation (A J Ferrari PhD, D F Santomauro PhD, C M Antony MA, D T Araki MPH, A Y Aravkin PhD, M B Arndt PhD, C Ashbaugh MA, J D Bishai BA, C Bisignano MPH, Prof M Brauer DSc, K Burkart PhD, J Byun BS, C S Chen BA, N M Chen BA, E Chung MSc, R M Cogen BA, E Cousin PhD, X Dai PhD, N K DeCleene BS, Prof L Degenhardt PhD, Prof S D Dharmaratne MD, M A Dirac MD, R V Dominguez BS, K Estep MPA, Prof V L Feigin PhD, L S Flor MPH, L M Force MD, W M Gardner MPH, H Hagins MSPH, E B Hamilton MPH, Prof S I Hay FMedSci, C A Henson MPH, J Hon MLS, J M Hsu BA, C K Huynh BA, V C Iannucci BA, A L Ihler PSM, K S Ikuta MD, M B Kassel BA, C Keller MPH, J M Kocarnik PhD, H H Kyu PhD, K E LeGrand MPH, Prof S S Lim PhD, M Lindstrom PhD, Prof R Lozano MD, A W McKowen MA, T Mestrovic PhD, P A Miller PhD, M E Moberg MS, R M Mohr MA, A H Mokdad PhD, J F Mosser MD, V Mougin BA, O D Nesbit MA, S M Ostroff PhD, M Pasovic MEd, S A Pease BS, D M Pigott PhD, N Pritchett DrPH, Q Rafferty BA, C Razo PhD, R C Reiner Jr PhD, A E Schumacher PhD, J Shadid BSc, E N Slepak MLIS, R J D Sorensen PhD, B A Stark MA, C Stein PhD, C Steiner MPH, J D Steinmetz PhD, A E Torre BS, Prof S E Vollset DrPH, A Vongpradith BA, Prof M R Weaver PhD, K M Wells BA, J L Whisnant MPH, Prof H A Whiteford PhD, C Wilkerson MPH, S Wulf Hanson PhD, Y Xu MPH, S R M Zimsen MA, Prof T Vos PhD, Prof C J L Murray DPhil), Department of Applied Mathematics (A Y Aravkin PhD), Department of Health Metrics Sciences, School of Medicine (A Y Aravkin PhD, K Burkart PhD, E Cousin PhD, X Dai PhD, Prof S D Dharmaratne MD, M A Dirac MD, L S Flor MPH, L M Force MD, Prof S I Hay FMedSci, H H Kyu PhD, Prof S S Lim PhD, Prof R Lozano MD, A Misganaw PhD, A H Mokdad PhD, D M Pigott PhD, R C Reiner Jr PhD, C Stein PhD, Prof S E Vollset DrPH, Prof M R Weaver PhD, Prof T Vos PhD, Prof C J L Murray DPhil), Department of Global Health (M B Arndt PhD, S Kochhar MD, R J D Sorensen PhD), Department of Health Systems and Population Health (A W Chen MSc), Department of Family Medicine (M A Dirac MD), Division of Pediatric Hematology-Oncology (L M Force MD), Department of Neurology (R Kalani MD), Department of Anesthesiology & Pain Medicine (V Krishnamoorthy MD), Division of Plastic and Reconstructive Surgery (S D Morrison MD), Henry M Jackson School of International Studies (S M Ostroff PhD), University of Washington, Seattle, WA, USA; Policy and Epidemiology Group (D F Santomauro PhD), West Moreton Hospital Health Services (A M Mantilla Herrera PhD), Queensland Centre for Mental Health Research, Wacol, QLD, Australia; Faculty of Medicine (A Aali MD), Dental Research Center (E Bardideh DDS), Department of Orthodontics (M Ghorbani DDS), Clinical Research Development Unit (N Morovatdar MD), Mashhad University of Medical Sciences, Mashhad, Iran; Department of Clinical Governance and Quality Improvement (Y H Abate MSc), Aleta Wondo Hospital, Aleta Wondo, Ethiopia; Department of Juridical and Economic Studies (C Abbafati PhD), Department of Public Health and Infectious Diseases (M S Cattaruzza PhD), La Sapienza University, Rome, Italy; Advanced Diagnostic and Interventional Radiology Research Center (H Abbastabar PhD), Research Center for Immunodeficiencies (H Abolhassani PhD, Prof N Rezaei PhD), Non-communicable Diseases Research Center (S Azadnajafabad MD, M Keykhaei MD, S Momtazmanesh MD, S Rahmani MD, M Rashidi MD, N Rezaei MD, N Rezaei PhD), School of Medicine (A Behnoush BS, N Hafezi-Nejad MD, A Khalaji BS, S Khanmohammadi MD, M Mayeli MD, S Mohammadi MD, S Momtazmanesh MD), Iranian Research Center for HIV/AIDS (O Dadras DrPH), Cardiac Primary Prevention Research Center (S Kazemian

MD), Department of Cardiac Electrophysiology (S Kazemian MD), Students' Scientific Research Center (SSRC) (M Keykhaei MD), Center for Research and Training in Skin Diseases and Leprosy (F Khamesipour PhD), Urology Research Center (Prof F Khatami PhD), Sina Trauma and Surgery Research Center (M Khormali MD, S Shool MD), Children's Medical Center (F Kompani MD), Endocrinology and Metabolism Research Institute (Prof B Larijani FACE, N Rezaei PhD, O Tabatabaei Malazy PhD), Department of Cardiology (E Mahmoudi MD, P Mansouri MD), Department of Pediatric Cardiology (Prof E Malakan Rad MD), Department of Epidemiology and Biostatistics (M Mansournia PhD), Tehran Heart Center (E Mehrabi Nasab MD, A Nasrollahizadeh MD), Water Quality Research Center (R Mirzaei PhD), Faculty of Medicine (E Mohammadi MD, A Nasrollahizadeh MD, S Vahabi MD), Digestive Diseases Research Institute (S G Sepanlou MD, M Yazdanpanah MD), Department of Neurology (M Shafie MD), Department of Medicine (A Shahbandi MD), Department of Pharmaceutical Care (A Sharifan PharmD), Research Center for Rational Use of Drugs (A Sharifan PharmD), Department of Pathology (Prof S Tavangar MD), Tehran University of Medical Sciences, Tehran, Iran (E Mohammadi MD); Department of Epidemiology (S Abd ElHafeez DrPH), Biomedical Informatics and Medical Statistics Department (I El Sayed PhD), Department of Pediatric Dentistry and Dental Public Health (Prof M El Tantawi PhD), Pediatric Dentistry and Dental Public Health Department (Prof O A A Elmeligy PhD), Department of Tropical Health and Parasitology (R M Ghazy PhD), Department of Pathology (Prof I M Talaat PhD), Alexandria University, Alexandria, Egypt; Department of Surgery (M Abdelmasseh MD, Prof J Sanabria MD), Marshall University, Huntington, WV, USA; Department of Tropical Medicine and Infectious Diseases (S Abd-Elsalam PhD), Tanta University, Tanta, Egypt; Minimally Invasive Surgery Research Center (A Abdollahi MD, A Kabir MD), Health Management and Economics Research Center (J Arabloo PhD, A Nasrollahizadeh MD), Preventive Medicine and Public Health Research Center (B Eshrati PhD, S Goharinezhad PhD, M Moradi-Lakeh MD), School of Medicine (Y Karimi MD), Eye Research Center (H Kasraei MD), Educational Development Center (E Khodadoust MD), Comprehensive Research Laboratory (R Mirzaei PhD), Gastrointestinal and Liver Diseases Research Center (M Moradi-Lakeh MD), Department of Physiology (H Pazoki Toroudi PhD), Physiology Research Center (H Pazoki Toroudi PhD), Colorectal Research Center (A Sarveazad PhD), Department of Ophthalmology (M Ziafati MD), Iran University of Medical Sciences, Tehran, Iran (M Moradi MD); Department of Physiotherapy (A Abdullahi PhD, A W Awotidebe PhD, J S Usman PhD), Department of Community Medicine (Prof M A Gadanya FMCPH), Department of Nursing Science (M Ladan PhD), Bayero University Kano, Kano, Nigeria; Department of Rehabilitation Sciences (A Abdullahi PhD, M U Ali MSc, J S Usman PhD), School of Nursing (S Tyrovolas PhD), Hong Kong Polytechnic University, Hong Kong, China; Department of Biostatistics (K H Abegaz PhD), Near East University, Nicosia, Turkiye; Department of Biostatistics and Health Informatics (K H Abegaz PhD), Madda Walabu University, Bale Robe, Ethiopia; Postgraduate Department (Prof R A Abeldaño Zuñiga PhD), University of Sierra Sur, Miahuatlan de Porfirio Diaz, Mexico; National Research Council of Mexico, Mexico City, Mexico (Prof R A Abeldaño Zuñiga PhD); Department of Family and Community Health (R G Aboagye MPH), Institute of Health Research (R K Alhassan PhD, M Immurana PhD), Institute of Health and Allied Sciences (M A Dalaba PhD), Department of Epidemiology and Biostatistics (R K Dowou Mphil Applied Epidemiology), Department of Population and Behavioural Sciences (E Manu PhD), Department of Microbiology and Immunology (V N Orish PhD), University of Health and Allied Sciences, Ho, Ghana; Department of Medical Biochemistry and Biophysics (H Abolhassani PhD), Department of Global Public Health (K Deuba DrPH, Prof L Laflamme PhD), Department of Neurobiology, Care Sciences, and Society (S Fereshtehnejad PhD), Department of Molecular Medicine and Surgery (Prof J H Kauppila MD), Karolinska Institute, Stockholm, Sweden;

Department of Pediatric Dentistry (Prof L G Abreu PhD), Department of Internal Medicine (Prof L C Brant PhD), Department of Maternal and Child Nursing and Public Health (Prof D C Malta PhD, E J S Prates BS), Department of Applied Nursing (Prof M O Pereira PhD), Federal University of Minas Gerais, Belo Horizonte, Brazil; Department of Nursing (H Abualruz PhD), Al Zaytoonah University of Jordan, Amman, Jordan; Clinical Sciences Department (E Abu-Gharbieh PhD, H J Barqawi MPhil, Prof R Halwani PhD, Prof A A Maghazachi PhD, M M Saber-Ayad MD, Prof I M Talaat PhD), College of Medicine (F Ahmad PhD, Prof R Halwani PhD, Prof B Saddik PhD, M A Saleh PhD), Department of Pharmacy Practice and Pharmacotherapeutics (Prof K H Alzoubi PhD, Prof H A Omar PhD), Department of Physiotherapy (A Arumugam PhD), Department of Basic Biomedical Sciences (Y Bustanji PhD), Sharjah Institute for Medical Research (N M Elemam PhD), Department of Clinical Nutrition and Dietetics (M E M Faris PhD), Department of Basic Medical Sciences (A Karim PhD), Department of Clinical Science (Prof M M Ramadan PhD), Department of Medicinal Chemistry (S S M Soliman PhD), University of Sharjah, Sharjah, United Arab Emirates; Institute of Community and Public Health (Prof N M Abu-Rmeileh PhD), Birzeit University, Ramallah, Palestine; School of Public Health and Preventive Medicine (I N Ackerman PhD, S M Alif PhD, P Maharjan MPH), Monash University, Melbourne, VIC, Australia; Centre for Social Research in Health (I Y Addo PhD, S R Okeke PhD), National Drug and Alcohol Research Centre (Prof L Degenhardt PhD), School of Population Health (X Feng PhD), School of Psychiatry (Prof P B Mitchell MD), Black Dog Institute, Centre for Research Excellence in Suicide Prevention (S Onie PhD), School of Public Health and Community Medicine (A E Peden PhD), Centre for Primary Health Care and Equity (CPHCE) (P Peprah MSc), School of Optometry and Vision Science (Prof K Pesudovs PhD), Faculty of Medicine and Health (S Sharma PhD), The George Institute for Global Health (P Ye MPH), University of New South Wales, Sydney, NSW, Australia; Quality and Systems Performance Unit (I Y Addo PhD), Cancer Institute NSW, Sydney, NSW, Australia; Internal Medicine and Alcohol Related Disease Unit (Prof G Addolorato MD), Department of Woman and Child Health and Public Health (D Buonsenso MD), Fondazione Policlinico Universitario A. Gemelli IRCCS (Agostino Gemelli University Polyclinic IRCCS), Rome, Italy; Department of Medical and Surgical Sciences (Prof G Addolorato MD), Università Cattolica di Roma (Catholic University of Rome), Rome, Italy; Department of Community Medicine (A O Adebiyi MD, O S Ilesanmi PhD), Department of Educational Counselling and Developmental Psychology (H O Adewuyi PhD), Department of Epidemiology and Medical Statistics (A F Fagbamigbe PhD), College of Medicine (A P Okekunle PhD), Faculty of Public Health (I I Olufadewa MHS), Department of Medicine (Prof M O Owolabi DrM), University of Ibadan, Ibadan, Nigeria; Department of Community Medicine (A O Adebiyi MD, O S Ilesanmi PhD), Department of Medicine (A S Oguntade MSc, Prof M O Owolabi DrM), Department of Oral and Maxillofacial Surgery (A A Salami BDS), University College Hospital, Ibadan, Ibadan, Nigeria; Department of HIV and Infectious Diseases (A V Adepoju MD), Jhpiego, Abuja, Nigeria; Department of Adolescent Research and Care (A V Adepoju MD), Adolescent Friendly Research Initiative and Care, Ado Ekiti, Nigeria; Department of Educational Psychology (H O Adewuyi PhD), University of Johannesburg, Johannesburg, South Africa (A Jeyakumar PhD); Department of Radiology and Radiological Science (S Afyouni PhD, A Amindarolzarbi MD, N Hafezi-Nejad MD, G G Z Zandieh MD), Department of Biostatistics (A Columbus MS), Department of Neurosurgery (F Kazemi MD), Department of Health Policy and Management (D Vervoort MD), Department of International Health (H Zhang MS), Johns Hopkins University, Baltimore, MD, USA; Department of Community Medicine (Prof S Afzal PhD), King Edward Memorial Hospital, Lahore, Pakistan; Department of Public Health (Prof S Afzal PhD), Public Health Institute, Lahore, Pakistan; Department of Orthopedic Surgery (S Afzal MD), National Nutrition and Food Technology Research Institute (M Ajami PhD), Urology Department (M Bonakdar Hashemi MD), Internal

Medicine Department of SBMU (H Borhany MD), Psychiatric Nursing and Management Department (F Ghadirian PhD), Department of Medical Genetics (M Ghasemi PhD), Center for Comprehensive Genetic Services (M Ghasemi PhD), Obstetrics and Gynecology Department (E Ghotbi MD), Research Institute for Endocrine Sciences (A Halimi BSc), Department of Immunology (K Jahankhani MSc), Department of Health Policy and Management (N Jahanmehr PhD), Safety Promotion and Injury Prevention Research Center (N Jahanmehr PhD), Department of Neurosurgery (H Khayat Kashani MD), Department of Ophthalmology and Vision Science (Z Khorrami PhD), Social Determinants of Health Research Center (A Kolahi MD, A Nikoobar DipSc, M Rashidi MD), School of Medicine (S Nejadghaderi MD, S Rahmani MD), Student Research Committee (M Rahmanian MD), Department of Epidemiology (S Sabour PhD), Department of Anesthesiology (A Shakeri MD), Ophthalmic Research Center (ORC) (M Shayan MD), Emergency Department (S Shool MD), Department of Medical Education (S Tabatabai PhD), Shahid Beheshti University of Medical Sciences, Tehran, Iran; Department of Medical and Surgical Sciences and Advanced Technologies "GF Ingrassia" (Prof A Agodi PhD, M Barchitta PhD, A Maugeri PhD, Prof M Veroux PhD), Department of Biomedical and Biotechnological Sciences (L Falzone PhD), Department of General Surgery and Medical-Surgical Specialties (Prof G Isola PhD), Department of Clinical and Experimental Medicine (C Ledda PhD), University of Catania, Catania, Italy; Department of Medical Biochemistry (A Ahmad PhD), Department of Pediatrics (Prof G Mustafa MD), Department of Pharmacology (M Tabish MPharm), Shaqra University, Shaqra, Saudi Arabia; School of Medicine and Psychology (D Ahmad PhD), National Centre for Epidemiology and Population Health (Y Alemu MPH), Research School of Population Health (N Bagheri PhD, R A Burns PhD), Australian National University, Canberra, ACT, Australia; Public Health Foundation of India, Gandhinagar, India (D Ahmad PhD); Department of Medical Oncology (S Ahmad MD), Department of Medicine (M Ganiyani MD), Miami Cancer Institute, Miami, FL, USA; Department of Community Medicine and Preventive Health (S Ahmad MD), King Edward Medical University Lahore, Lahore, Pakistan; Department of Pharmacy Practice (A Ahmed PhD), Riphah Institute of Pharmaceutical Sciences, Islamabad, Pakistan; Division of Infectious Diseases and Global Public Health (IDGPH) (A Ahmed PhD), University of California, San Diego, CA, USA; Institute of Public Health (L A Ahmed PhD, I Elbarazi DrPH), College of Medicine and Health Sciences (Prof M Grivna PhD), Family Medicine Department (M A Khan MSc), Department of Computer Science and Software Engineering (Prof N Zaki PhD), United Arab Emirates University, Al Ain, United Arab Emirates; Department of Epidemiology (M B Ahmed MPH, D Shiferaw MPH), Department of Public Health (M Y Ashemo PhD), Department of Surgery (N S Bayileyegn MD), Jimma University, Jimma, Ethiopia; Australian Center for Precision Health (M B Ahmed MPH), Department of Allied Health and Human Performance (T Y Tiruye PhD), University of South Australia, Adelaide, SA, Australia; Department of Food and Nutrition Policy and Planning Research (M Ajami PhD), National Institute of Nutrition, Tehran, Iran; Department of Internal Medicine (K Akinosoglou PhD), University of Patras, Patras, Greece; Department of Internal Medicine and Infectious Diseases (K Akinosoglou PhD), University General Hospital of Patras, Patras, Greece; Department of Cardiology (M A Akkaif PhD), Department of Health Management Center (X Li PhD), Fudan University, Shanghai, China; Division of Public Health Sciences (S Al Hasan PhD), Washington University School of Medicine, St. Louis, MO, USA; Department of Clinical Sciences (S O Alalalmeh BPharm, O E Hegazi BPharm), Center for Medical and Bio-Allied Health Sciences Research (Prof M J Shahwan PhD, M A Shamsi PhD, S H Zyoud PhD), Ajman University, Ajman, United Arab Emirates; John T. Milliken Department of Internal Medicine (Z Al-Aly MD), Department of Surgery (T Lan PhD, C Wang MPH), Brown School (C Wang MPH), Washington University in St. Louis, St. Louis, MO, USA; Clinical Epidemiology Center (Z Al-Aly MD), US Department of Veterans Affairs (VA), St Louis,

MO, USA; Department of Community and Mental Health (Prof M Albashtawy PhD), Al al-Bayt University, Mafraq, Jordan; Institute of Health Informatics (R W Aldridge PhD), Division of Psychology and Language Sciences (M Kumar PhD), Institute of Cardiovascular Science (A S Oguntade MSc), Division of Surgery and Interventional Science (T Umar MD), Department of Infection (Prof A Zumla PhD), University College London, London, UK; Independent Consultant, Hosaena, Ethiopia (M D Alemu MPH); Department of Epidemiology and Biostatistics (Y Alemu MPH), College of Medicine and Health Science (A Amare PhD), Department of Health Promotion and Behavioural Science (E K Bogale MPH, Z A Yigzaw MPH), Department of Surgery (N A Shitaye MD), Department of Pharmacology (Y Yismaw MSc), Bahir Dar University, Bahir Dar, Ethiopia; Faculty of Health Sciences (K A Alene MPH), School of Population Health (Prof P W Gething PhD, D J Weiss PhD), School of Public Health (D Hendrie PhD, T R Miller PhD), Curtin University, Perth, WA, Australia; Wesfarmers Centre of Vaccines and Infectious Diseases (K A Alene MPH), Child Health Analytics Research Program (P A Dzianach PhD, Prof P W Gething PhD, F Sanna PhD, D J Weiss PhD), Geospatial Health and Development Team (J Lubinda PhD, A Saddler PhD), The Malaria Atlas Project (M A McPhail PhD), Telethon Kids Institute, Perth, WA, Australia; Global Centre for Environmental Remediation (A A S Al-Gheethi PhD), School of Medicine and Public Health (P Atorkey MPhil), Department of Public Health and Medicine (K Awuviry-Newton PhD), University of Newcastle, Newcastle, NSW, Australia; Cooperative Research Centre for Contamination Assessment and Remediation of the Environment, Newcastle, NSW, Australia (A A S Al-Gheethi PhD); Adult Health and Critical Care Department (M Alharrasi PhD), Sultan Qaboos University, College of Nursing, Alseeb, Oman; Department of Medical Rehabilitation (Physiotherapy) (M U Ali MSc), Department of Clinical Pharmacy and Pharmacy Administration (H Yusuf PhD), University of Maiduguri, Maiduguri, Nigeria; Department of Biosciences (R Ali MPhil, N Salam PhD), Centre for Interdisciplinary Research in Basic Sciences (CIRBSc) (S Anwar PhD), Centre For Interdisciplinary Research In Basic Sciences (CIRBSc) (M A Shamsi PhD), Jamia Millia Islamia, New Delhi, India; Center for Biotechnology and Microbiology (S S Ali PhD), University of Swat, Swat, Pakistan; Department of Health Policy and Management (Prof S M Aljunid PhD), Kuwait University, Kuwait, Kuwait; International Centre for Casemix and Clinical Coding (Prof S M Aljunid PhD), National University of Malaysia, Bandar Tun Razak, Malaysia; Department of Dentistry (S Al-Marwani MSc), Independent Consultant, Sana'a, Yemen; Public Health and Community Medicine (S Al-Marwani MSc), Independent Consultant, Irbid, Jordan; Department of Medicine (J U Almazan PhD, Prof D Poddighe PhD), Nazarbayev University, Astana, Kazakhstan; Department of Physical Education (Prof M A Alomari PhD), Social and Economic Survey Research Institute (Prof A Perianayagam PhD), Department of Population Medicine (Prof G Rathnaiah Babu PhD), Qatar University, Doha, Qatar; Department of Rehabilitation Sciences and Physical Therapy (Prof M A Alomari PhD), Department of Rehabilitation Sciences (M S Al-Wardat PhD), Department of Clinical Pharmacy (Prof K H Alzoubi PhD), Jordan University of Science and Technology, Irbid, Jordan; Department of Epidemiology and Population Health (B Al-Omari PhD), Khalifa University of Science, Technology & Research, Abu Dhabi, United Arab Emirates; Department of Basic Sciences (Z Altaany PhD), Yarmouk Univeristy, Irbid, Jordan; Research Group in Hospital Management and Health Policies (Prof N Alvis-Guzman PhD), Department of Economic Sciences (N J Alvis-Zakzuk MSc), Universidad de la Costa (University of the Coast), Barranquilla, Colombia; Research Group in Health Economics (Prof N Alvis-Guzman PhD), University of Cartagena, Cartagena, Colombia; National Health Observatory (N J Alvis-Zakzuk MSc), Colombian National Health Observatory (C A Castañeda-Orjuela MD), National Institute of Health, Bogota, Colombia; Department of Clinical Pharmacology and Toxicology (H Alwafi PhD), Umm Al-Qura University, Makkah, Saudi Arabia; Department of Medical Sciences (Prof Y M Al-Worafi PhD), Azal University for Human Development,

Sana'a, Yemen; Department of Clinical Sciences (Prof Y M Al-Worafi PhD), University of Science and Technology of Fujairah, Fujairah, United Arab Emirates; Department of Pediatric Cardiology (S Aly MD), Boston Children's Hospital, Boston, MA, USA; Department of Pediatrics (S Aly MD), Center for Primary Care (S Basu PhD), Harvard Business School (F Caetano dos Santos PhD), Department of Epidemiology (S Carr MS), Department of Neurological Surgery at Brigham and Women's Hospital (A H Feroze MD), Department of Ophthalmology (Prof J H Kempen MD), Department of Health Policy and Oral Epidemiology (Z S Natto DrPH), Department of Global Health and Social Medicine (M Pigeolet MD), T.H. Chan School of Public Health (P M S Pradhan MD), Harvard University, Boston, MA, USA; School of Medicine (A Amare PhD), Adelaide Medical School (T K Gill PhD, L Yadav PhD), School of Public Health (K Malhotra MBBS), University of Adelaide, Adelaide, SA, Australia; School of Global Public Health (P M Amegbor PhD, E K Peprah PhD), New York University, New York, NY, USA; School of Graduate Studies (E K Ameyaw MPhil), Lingnan University, Hong Kong, China; Public Health and Community Medicine Department (Prof T T Amin MD), Department of Neurology (A Hassan MD), National Hepatology and Tropical Medicine Research Institute (A M Khater MD), Cairo University, Cairo, Egypt; Medicine, Quran and Hadith Research Center (S Amiri PhD), Nephrology and Urology Research Center (K Hushmandi PhD), Baqiyatallah University of Medical Sciences, Tehran, Iran; Department of Maternal and Child Wellbeing (D A Amugsi PhD), African Population and Health Research Center, Nairobi, Kenya; Faculty of Pharmacy (Prof R Ancuceanu PhD), Department of Cardiology (C Andrei PhD), Department of Dermatology and Venereology (Prof S R Georgescu PhD), Department of Internal Medicine (M Hostiuc PhD), Department of Legal Medicine and Bioethics (S Hostiuc PhD), Department of General Surgery (I Negoi PhD), Department of Anatomy and Embryology (R I Negoi PhD), Department of Diabetes, Nutrition and Metabolic Diseases (A Pantea Stoian PhD), Department of Dermatology (M Tampa PhD), Carol Davila University of Medicine and Pharmacy, Bucharest, Romania; Neurology Department (D Anderlini MD), Royal Brisbane and Women's Hospital, Brisbane, QLD, Australia; Faculty of Medicine (D B Anderson PhD), School of Architecture, Design, and Planning (Prof T Astell-Burt PhD), Westmead Clinical School (R Chimoriya PhD), School of Pharmacy and Charles Perkins Centre (Z Dai PhD), School of Public Health (Prof T R Driscoll PhD), Faculty of Medicine and Health (Prof P H Ferreira PhD), Sydney Medical School (S Islam PhD), Save Sight Institute (H Kandel PhD), Kolling Institute (S Mathieson PhD), Sydney Musculoskeletal Health (S Mathieson PhD), School of Chemical & Biomolecular Engineering (E A Noman PhD), School of Veterinary Science (B B Singh PhD), University of Sydney, Sydney, NSW, Australia; Department of Health Care Management (P P Andrade MD, Prof R Busse PhD, S Mohammed PhD), Technical University of Berlin, Berlin, Germany; European University, Lisbon, Portugal (P P Andrade MD); Department of Epidemiology and Biostatistics (Prof H Ansari PhD), Health Promotion Research Center (H Okati-Aliabad PhD), Zahedan University of Medical Sciences, Zahedan, Iran; School of Chemical and Life Sciences (SCLS) (S Anwar PhD), Jamia Hamdard, New Delhi, India; Department of Surgery (S Anwar PhD), Gadjah Mada University, Yogyakarta, Indonesia; Department of Pathology (R Anwer PhD), Imam Mohammad Ibn Saud Islamic University, Riyadh, Saudi Arabia; Warwick Medical School (P E Anyanwu PhD), University of Warwick, Coventry, UK (J W Sakshaug PhD); Department of Medicine (J Arab MD), Western University, London, ON, Canada; Gastroenterology Department (J Arab MD), Department of Gastroenterology, School of Medicine (L A Diaz MD), Pontifical Catholic University of Chile, Santiago, Chile; College of Pharmacy (M Arafat PhD), Al Ain University, Abu Dhabi, United Arab Emirates; University of Texas Health Science Center, Houston, TX, USA (D T Araki MPH); Department of Medical Laboratory Sciences (M Arkew MSc), Department of Health Policy and Management (A T Debele MSc), Health Sciences Department of Oncology Nursing (T G Gebi MSc), Department of Clinical Pharmacy (M D

Gudeta MSc), School of Medical Laboratory Sciences (H A Meresa MSc), Haramaya University, Harar, Ethiopia; Department of Cardiovascular, Endocrine-Metabolic Diseases and Aging (B Armocida MSc), Istituto Superiore di Sanità, Rome, Italy; University College of Medicine & Dentistry (Prof M Arooj PhD), Institute of Molecular Biology and Biotechnology (Prof M Ashraf PhD, S Shahid PhD), University Institute of Public Health (A A Malik PhD, S Nargus PhD), Department of Technology (M Muzaffar MBA), Research Centre for Health Sciences (RCHS) (M Muzaffar MBA, S Shahid PhD), Department of Physics (W Shahid PhD), The University of Lahore, Lahore, Pakistan; Department of Biophysics (A A Artamonov PhD), Russian Academy of Sciences, Moscow, Russia; Department of Computer Science (A O Bodunrin MSc), Department of Cardiovascular Sciences (A Ghajar MD), Department of Physiology (M Tumurkhuu PhD), East Carolina University, Greenville, NC, USA (R T Aruleba PhD); Department of Community Medicine and Rehabilitation (A Arumugam PhD), Department of Nursing (Prof D Edvardsson PhD), Umeå University, Umea, Sweden; Department of Public Health (M Y Ashemo PhD), Wachemo University, Hossana, Ethiopia; Department of Medical Laboratory Sciences (M O Asika BMLS), Department of Pharmacology and Therapeutics (Prof O E Onwujekwe PhD), University of Nigeria Nsukka, Enugu, Nigeria; Department of Telemedicine (M O Asika BMLS), Society For Disease Prevention, Inc., Hummelstown, PA, USA; Department of Nutrition (E Askari PhD), Lorestan University of Medical Sciences, Khorramabad, Iran; Department of Immunology (S Athari PhD), Zanjan University of Medical Sciences, Zanjan, Iran; Hunter New England Population Health, Wallsend, NSW, Australia (P Atorkey MPhil); Faculty of Nursing (M M W Atout PhD), Philadelphia University, Amman, Jordan; Department of Forensic Medicine (A Atreya MD), Lumbini Medical College, Palpa, Nepal; Northumbria HealthCare NHS Foundation Trust, Newcastle upon Tyne, UK (A Aujayeb MBBS); School of Business (Prof M Ausloos PhD), Department of Health Sciences (Prof T Brugha MD, P H Lee PhD, S J Tromans PhD), University of Leicester, Leicester, UK; Department of Statistics and Econometrics (Prof M Ausloos PhD, A Otoiu PhD), Faculty of Management (A Dima PhD), Bucharest University of Economic Studies, Bucharest, Romania; Robarts Research Institute (A Avan MD), The University of Western Ontario, London, ON, Canada; School of Nursing and Public Health (A W Awotidebe PhD), Discipline of Public Health Medicine (T G Ginindza PhD), University of KwaZulu-Natal, Durban, South Africa; The Judith Lumley Centre (B Ayala Quintanilla PhD), School of Nursing and Midwifery (Prof D Edvardsson PhD, M Rahman PhD), Department of Public Health (H Jiang PhD), La Trobe University, Melbourne, VIC, Australia; San Martin de Porres University, Lima, Peru (B Ayala Quintanilla PhD); Department of Psychiatry (Prof J L Ayuso-Mateos PhD), Universidad Europea de Madridn (European University of Madrid), Madrid, Spain; Biomedical Research Networking Center for Mental Health Network (CIBERSAM) (Prof J L Ayuso-Mateos PhD), National School of Public Health (A Padron-Monedero PhD), Institute of Health Carlos III, Madrid, Spain; Department of Sciences (Prof R M S Azevedo PhD), Toxicology Research Unit (TOXRUN) (Á M Madureira-Carvalho PhD), Cooperativa de Ensino Superior Politécnico e Universitário (CESPU) (University Polytechnic Higher Education Cooperative), Gandra, Portugal; Department of Physiotherapy (A S Babu PhD, Prof V K PhD), Department of Forensic Medicine and Toxicology (S M Bakkannavar MD, Prof V C Nayak MD), Prasanna School of Public Heath (PSPH) (V Dsouza MSc), Kasturba Medical College, Mangalore (R Holla MD, M Rao MD), Manipal College of Dental Sciences (Prof A I Narayana PhD, Prof R A Radhakrishnan PhD), Manipal TATA Medical College (M Rahman PhD), Department of Community Medicine (C R Rao MD), Department of Health Information Management (B Reshmi PhD), Manipal Academy of Higher Education, Manipal, India; Gomal Center of Biochemistry and Biotechnology (M Badar PhD), Gomal University, Dera Ismail Khan, Pakistan; Department of Forensic Science (A D Badiye PhD), Government Institute of Forensic Science, Nagpur, India; Division of Orthopaedics (S Baghdadi

MD), Children's Hospital of Philadelphia, Philadelphia, PA, USA; Health Research Institute (N Bagheri PhD), University of Canberra, Canberra, ACT, Australia; Department of Public Health (Prof S Bah PhD), Division of Forensic Medicine (Prof R G Menezes MD), Imam Abdulrahman Bin Faisal University, Dammam, Saudi Arabia; School of Public Affairs (R Bai MD), Nanjing University of Science and Technology, Nanjing, China; Center for Clinical Research and Prevention (J L Baker PhD), Bispebjerg University Hospital, Frederiksberg, Denmark; Department of Neurosurgery (A T Bako PhD), Houston Methodist Hospital, Houston, TX, USA; Division of Biological Sciences (S Balakrishnan PhD), Tamil Nadu State Council for Science and Technology, Chennai, India; Department of Medicine (K Bam MPH), School of Nursing and Midwifery (D Bhandari PhD), Australian Regenerative Medicine Institute (Y Mathangasinghe MD), Department of Neuroscience (Prof C A Unsworth PhD), Monash University, Clayton, VIC, Australia; Department of Non-communicable Diseases (P C Banik MPhil, L Barua MPH), Bangladesh University of Health Sciences, Dhaka, Bangladesh; Miami Cancer Institute (M Bardhan MD), Baptist Health South Florida, Miami, FL, USA; School of Psychology (Prof S L Barker-Collo PhD), University of Auckland, Auckland, New Zealand; Department of Epidemiology (A Barrow MPH, D Braithwaite PhD, D D Ding BS), College of Medicine (M J Diaz BS), Department of Computer and Information Science and Engineering (P Naghavi MSc), Biology & Emerging Pathogens Institute (M H Zahid PhD), University of Florida, Gainesville, FL, USA; Department of Public & Environmental Health (A Barrow MPH), University of The Gambia, Brikama, The Gambia; Heidelberg Institute of Global Health (HIGH) (S Barteit PhD, B Moazen MSc), Heidelberg University, Heidelberg, Germany; Burn and Regenerative Medicine Research Center (S Bashiri Aliabadi MD), Department of Environmental Health Engineering (J Jaafari PhD), Guilan University of Medical Sciences, Rasht, Iran; Department of Veterinary Physiology and Biochemistry (A Basiru PhD), Department of Veterinary Public Health and Preventive Medicine (I A Odetokun PhD), University of Ilorin, Ilorin, Nigeria; School of Public Health (S Basu PhD), Department of Primary Care and Public Health (Prof A Majeed MD, Prof S Rawaf MD, C Tabche MSc), Department of Surgery and Cancer (Prof E Mossialos PhD), WHO Collaborating Centre for Public Health Education and Training (D L Rawaf MRCS), Division of Brain Sciences (Prof T J Steiner PhD), Imperial College London, London, UK; Department of Academics (S Basu MD), Indian Institute of Public Health, Gurgaon, India; Department of Clinical Pharmacology (Prof P P Bathini MD), Department of Dermatology, Venereology and Leprosy-DVL (Prof T Priscilla MD), Apollo Institute of Medical Sciences and Research, Hyderabad, India; Department of Medical Education (K Batra PhD), Department of Social and Behavioral Health (Prof M Sharma PhD), University of Nevada Las Vegas, Las Vegas, NV, USA; Department of Psychiatry (Prof B T Baune PhD), University of Münster, Münster, Germany; Department of Psychiatry (Prof B T Baune PhD), Melbourne Medical School, Melbourne, VIC, Australia; Department of Regulatory Affairs (B Behnam MD), NSF International, Ann Arbor, GA, USA; Department of Regulatory Affairs (B Behnam MD), Amarex Clinical Research, Germantown, MD, USA; Endocrinology and Metabolism Research Institute (A Khalaji BS), Department of Epidemiology (S Khanmohammadi MD, S Nejadghaderi MD), Non-Communicable Diseases Research Center (NCDRC), Tehran, Iran (A Behnoush BS); Division of Pulmonary, Critical Care, and Sleep (M Beiranvand PhD), University of Florida, Jacksonville, FL, USA; Department of Medicine (D F Bejarano Ramirez BN), El Bosque University, Bogota, Colombia; Transplant Service (D F Bejarano Ramirez BN), University Hospital Foundation Santa Fe de Bogotá, Bogota, Colombia; School of the Environment (Prof M L Bell PhD, Y Song PhD), Department of Dermatology (M Goldust MD), Department of Psychiatry (W Li PhD), Department of Radiology and Biomedical Imaging (X Liu PhD), Department of Genetics (S Pawar PhD), Yale University, New Haven, CT, USA; Department of Microbiology (O O Bello PhD), Department of Biological Sciences (T C Ekundayo PhD), University of Medical Sciences, Ondo, Ondo,

Nigeria; Department of Biomedical Sciences (Prof A Beloukas PhD), University of West Attica, Athens, Greece; Institute of Infection and Global Health (Prof A Beloukas PhD), Department of Surgery (Prof R Lunevicius DSc), University of Liverpool, Liverpool, UK; Department of Internal Medicine (I M Bensenor PhD, I S Santos PhD), Department of Psychiatry (Prof J Castaldelli-Maia PhD, Y Wang PhD), Center for Clinical and Epidemiological Research (I S Santos PhD), University of São Paulo, São Paulo, Brazil; Institute of Marketing (Z Berezvai PhD), Corvinus University of Budapest, Budapest, Hungary; Competition Economics and Market Research Section (Z Berezvai PhD), Hungarian Competition Authority, Budapest, Hungary; Faculty of Dentistry, Oral & Craniofacial Sciences (E Bernabe PhD), Department of Twin Research and Genetic Epidemiology (M Mazidi PhD), Institute of Psychiatry, Psychology & Neuroscience (D Urso MD), School of Population Health and Environmental Sciences (Y Wang PhD), King's College London, London, UK; Hubert Department of Global Health (R S Bernstein MD), Rollins School of Public Health (Prof D A Sleet PhD), Department of Family and Preventive Medicine (S Thirunavukkarasu PhD), Emory University, Atlanta, GA, USA; Butte County Department of Public Health, Chico, CA, USA (R S Bernstein MD); Faculty of Medicine (P J G Bettencourt PhD), Catholic University of Portugal, Rio de Mouro, Portugal; Department of Public Health (A S Bhagavathula PhD), North Dakota State University, Fargo, ND, USA; Institutes of Applied Health Research and Translational Medicine (N Bhala PhD), Queen Elizabeth Hospital Birmingham, Birmingham, UK; Institute of Applied Health Research (N Bhala PhD, K Malhotra MBBS, R Thayakaran PhD), NIHR Global Health Research Unit on Global Surgery (J C Glasbey MSc), University of Birmingham, Birmingham, UK; Public Health Research Laboratory (D Bhandari PhD), Department of Community Medicine (P M S Pradhan MD), Central Department of Public Health (N S Subedi MPH), Tribhuvan University, Kathmandu, Nepal; Department of Internal Medicine (A Bhargava MD), Wayne State University, Detroit, MI, USA; Global Health Neurology Lab (S Bhaskar PhD), NSW Brain Clot Bank, Sydney, NSW, Australia; Department of Neurology and Neurophysiology (S Bhaskar PhD), South West Sydney Local Heath District and Liverpool Hospital, Sydney, NSW, Australia; Department of Internal Medicine (V Bhat MBBS), St. John's National Academy of Health Sciences, Bangalore, India; Department of Medical Lab Technology (G K Bhatti PhD), University Centre for Research and Development (S Kalra DM), Chandigarh University, Mohali, India; Department of Human Genetics and Molecular Medicine (Prof J S Bhatti PhD, S Senapati PhD, U Sharma PhD), Department of Zoology (B Vellingiri PhD), Central University of Punjab, Bathinda, India; Department of Botanical and Environmental Sciences (Prof M S Bhatti PhD), Department of Pharmaceutical Sciences (R Bhatti PhD), Guru Nanak Dev University, Amritsar, India; Centre for Global Child Health (Prof Z A Bhutta PhD), Temerty Faculty of Medicine (V Chattu MD), Division of Neurology (S Fereshtehnejad PhD), University of Toronto, Toronto, ON, Canada; Centre of Excellence in Women & Child Health (Prof Z A Bhutta PhD), Division of Women and Child Health (J K Das MD), Aga Khan University, Karachi, Pakistan; Scientific-Tools.Org, Bergamo, Italy (B Bikbov MD); Faculty of Health Sciences (V R Bitra PhD), University of Botswana, Gaborone, Botswana; Department of Global Public Health and Primary Care (Prof T Bjørge PhD), Department of Psychosocial Science (D Sagoe PhD), University of Bergen, Bergen, Norway; Cancer Registry of Norway, Oslo, Norway (Prof T Bjørge PhD); School of Business Administration (Prof V Bodolica PhD), American University of Sharjah, Sharjah, United Arab Emirates; Faculty of Medicine and Pharmaceutical Sciences (A Bonny MD), University of Douala, Douala, Cameroon; Department of Cardiology (A Bonny MD), Centre Hospitalier Montfermeil (Montfermeil Hospital Center), Montfermeil, France; General Directorate of Health Information Systems (B Bora Basara PhD), Ministry of Health, Ankara, Turkiye; Department of Earth, Environment, and Equity (C Boxe PhD), Howard University, Washington, DC, USA; Department of Infectious Disease Epidemiology (O J Brady PhD), MSc

Epidemiology Programme (A Hafiz PhD), Department of Non-Communicable Disease Epidemiology (M Iwagami PhD), Department of Health Services Research and Policy (Prof M McKee DSc), London School of Hygiene & Tropical Medicine, London, UK; Department of Health Sciences (DISSAL) (F Lanfranchi MD), University of Genoa, Genoa, Italy (N L Bragazzi PhD); Cancer Population Sciences Program (D Braithwaite PhD), University of Florida Health Cancer Center, Gainesville, FL, USA; School of Population and Public Health (Prof M Brauer DSc, P A Chakraborty MPH), School of Nursing (A Pashaei MSc), University of British Columbia, Vancouver, BC, Canada; Institute for Medical Information Processing, Biometry, and Epidemiology (S Breitner DSc), Ludwig Maximilian University of Munich, Munich, Germany; Institute of Epidemiology (S Breitner DSc), Helmholtz Zentrum München German Research Center for Environmental Health, Neuherberg, Germany; Division of Clinical Epidemiology and Aging Research (Prof H Brenner MD), German Cancer Research Center, Heidelberg, Germany; Department of Injury (J Brown PhD), The George Institute for Global Health, Newtown, NSW, Australia; Faculty of Medicine (J Brown PhD), School of Population Health (Z Dai PhD, X Xu PhD), School of Psychiatry (Prof P S Sachdev MD), University of New South Wales, Kensington, NSW, Australia; Flinders Health and Medical Research Institute (N B Bulamu PhD), College of Medicine and Public Health (T G Gebremeskel PhD, B Kaambwa PhD, G R Naik PhD), Health Economics Unit (B Kaambwa PhD), Flinders University, Adelaide, SA, Australia; Global Health Research Institute (D Buonsenso MD), Università Cattolica del Sacro Cuore (Catholic University of Sacred Heart), Rome, Italy; Department of Biopharmaceutics and Clinical Pharmacy (Y Bustanji PhD), The University of Jordan, Amman, Jordan; School of Public Health Sciences (Z A Butt PhD), University of Waterloo, Waterloo, ON, Canada; Al Shifa School of Public Health (Z A Butt PhD), Al Shifa Trust Eye Hospital, Rawalpindi, Pakistan; Department of Clinical Pharmacy (Prof D Calina PhD), University of Medicine and Pharmacy of Craiova, Craiova, Romania; Department of Internal Medicine (Prof L A Cámera MD), Hospital Italiano de Buenos Aires (Italian Hospital of Buenos Aires), Buenos Aires, Argentina; Board of Directors (Prof L A Cámera MD), Argentine Society of Medicine, Buenos Aires, Argentina; Center for Nutrition and Health Research (I R Campos-Nonato PhD, E Denova-Gutiérrez DSc), Center for Health Systems Research (D V Ortega-Altamirano DrPH), National Institute of Public Health, Cuernavaca, Mexico; Dana-Farber Cancer Institute, Boston, MA, USA (C Cao MPH); Department of Biomedical and Neuromotor Sciences (A Capodici MD, S Guicciardi MD, J Lenzi PhD, A Mazzotti PhD), Department of Medical and Surgical Sciences (Prof F S Violante MD), University of Bologna, Bologna, Italy; Management and Healthcare (EMbeDS) (A Capodici MD), Sant'Anna School of Advanced Studies, Pisa, Italy; Oncological Network, Prevention and Research Institute (G Gorini MD), Institute for Cancer Research, Prevention and Clinical Network, Florence, Italy (G Carreras PhD); Dermatology Unit (A Carugno MD), Azienda Socio Sanitaria Territoriale Papa Giovanni XXIII (Territorial Healthcare Company Pope John XXIII), Bergamo, Italy; Instituto de Investigação, Inovação e Desenvolvimento (Institute of Research Innovation and Development) (M Carvalho PhD), University Fernando Pessoa, Porto, Portugal; Associated Laboratory for Green Chemistry (LAQV) (M Carvalho PhD), Institute for Research and Innovation in Health (Prof N Cruz-Martins PhD), Research Unit on Applied Molecular Biosciences (UCIBIO) (Prof D Dias da Silva PhD, J P Silva PhD), Department of Community Medicine, Information and Health Decision Sciences (A Freitas PhD), Department of Chemical Engineering (Prof C F Rodrigues PhD), University of Porto, Porto, Portugal; Epidemiology and Public Health Evaluation Group (C A Castañeda-Orjuela MD), National University of Colombia, Bogota, Colombia; Department of Medicine (G Castelpietra PhD), University of Udine, Udine, Italy; Department of Mental Health (G Castelpietra PhD), Healthcare Agency "Friuli Occidentale", Pordenone, Italy; Department of Pharmacological and Biomolecular Sciences (Prof A L Catapano PhD), Department of Clinical Sciences and Community Health

(Prof C La Vecchia MD), Department of Food, Environmental and Nutritional Sciences (Prof D Martini PhD), University of Milan, Milan, Italy; MultiMedica Sesto San Giovanni IRCCS, Sesto S. Giovanni, Italy (Prof A L Catapano PhD); Department of Psychiatry (A Caye PhD), Postgraduate Program in Epidemiology (Prof B B Duncan MD, Prof M I Schmidt MD), Department of Preventive and Social Dentistry (F N Hugo PhD), Federal University of Rio Grande do Sul, Porto Alegre, Brazil; Department of Medical, Surgical & Health Sciences (L Cegolon PhD, Prof M D'Oria MD), University of Trieste, Trieste, Italy; Public Health Unit (L Cegolon PhD), University Health Agency Giuliano-Isontina (ASUGI), Trieste, Italy; Department of Nutrition (Prof F Cembranel DSc), Department of Physical Education (Prof D A S Silva PhD), Federal University of Santa Catarina, Florianópolis, Brazil; College of Public Health, Medical, and Veterinary Sciences (M Cenderadewi MPHTM, Prof R C Franklin PhD, A E Peden PhD), James Cook University, Townsville, QLD, Australia; Department of Public Health (M Cenderadewi MPHTM), University of Mataram, Mataram, Indonesia; Mary MacKillop Institute for Health Research (Prof E Cerin PhD), Australian Catholic University, Melbourne, VIC, Australia; School of Public Health (Prof E Cerin PhD), Department of Urban Planning and Design (C Guo PhD), University of Hong Kong, Hong Kong, China; Heart Failure and Structural Heart Disease Unit (J Chan MBChB), Cardiovascular Analytics Group, Hong Kong, China; Department of Medicine and Therapeutic (R N C Chan MBChB (GPS) (CUHK)), Prince of Wales Hospital, Hong Kong, China; Department of Clinical Nutrition (R M Chandika PhD), Department of Epidemiology (S Dohare MD, K Y Ghailan PhD, M Khan MD), Department of Maxillofacial Surgery and Diagnostic Sciences (E S Halboub PhD), Health Research Center (Prof A Khalid PhD), Department of Health Education and Promotion (M Shanawaz MD), Jazan University, Jazan, Saudi Arabia; Department of Anesthesiology and Perioperative Medicine (E K Chandrasekar MD), University of Rochester, Rochester, NY, USA (E Dorsey MD); Department of Public Health (P Charalampous PhD, S Polinder PhD), Erasmus University Medical Center, Rotterdam, Netherlands; Department of Community Medicine (V Chattu MD), Datta Meghe Institute of Medical Sciences, Sawangi, India; Department of Endocrinology (V Chatzimavridou-Grigoriadou MD), Division of Immunology, Immunity to Infection and Respiratory Medicine (A G Mathioudakis PhD), University of Manchester, Manchester, UK; Department of Endocrinology (V Chatzimavridou-Grigoriadou MD), Christie Hospital NHS Foundation Trust, Manchester, UK; Fuwai Hospital (A Chen PhD), Chinese Academy of Medical Sciences & Peking Union Medical College, Beijing, China; Department of Computer Science (A Chen PhD), University of Texas Austin, Austin, TX, USA; Clinical Research Center (H Chen MB), Zhujiang Hospital (Z Zhu PhD), Southern Medical University, Guangzhou, China; Department of Paediatrics (E T W Cheng MBChB, S Dai PhD), The Nethersole School of Nursing (Y Chong PhD), Jockey Club School of Public Health and Primary Care (J Huang MD, C Zhong MD), Department of Medicine and Therapeutics (L Lim MRCP), The Chinese University of Hong Kong, Hong Kong, China; Department of Public Health and Health Policy (O Chimed-Ochir PhD), Hiroshima University, Hiroshima, Japan; Translational Health Research Institute (R Chimoriya PhD), Department of Engineering (G R Naik PhD), Western Sydney University, Sydney, NSW, Australia; Division of Infectious Diseases (P R Ching MD), Virginia Commonwealth University, Richmond, VA, USA; Department of Clinical Oncology (W C S Cho PhD), Queen Elizabeth Hospital, Hong Kong, China; College of Medicine (S Choi MD program student), Department of Epidemiology and Health Promotion (Prof S Jee PhD), Yonsei University, Seoul, South Korea; Department of Medicine (B Chong MBBS), School of Medicine (M Ng PhD), Leadership Institute for Global Health Transformation (LIGHT) (S Ramazanu PhD), Department of Surgery (K Tan PhD), Yong Loo Lin School of Medicine (Prof N Venketasubramanian MBBS), National University of Singapore, Singapore, Singapore; Department of Community Medicine (Prof S G Choudhari MD, Prof A M Gaidhane MD), Datta Meghe Institute of Medical Sciences, Wardha, India; Florida

International University, Miami, FL, USA (Prof R Chowdhury PhD); Department of Epidemiology (Prof R Chowdhury PhD), Institute of Social and Preventive Medicine (M Ganbat MPH), Department of Neurology (L D Panos MD), University of Bern, Bern, Switzerland; Department of Health Science and Technology (S W M Christensen PhD), Aalborg University, Aalborg, Denmark; Department of Physiotherapy (S W M Christensen PhD), University College of Northern Denmark, Aalborg, Denmark; Center for Biomedicine and Community Health (D Chu PhD), Viet Nam National University-International School, Hanoi, Vietnam; Department of Paediatric Surgery (I S Chukwu BMedSc), Federal Medical Centre, Umuahia, Nigeria; Department of AndroUrology (Prof E Chung MD), AndroUrology Centre, Brisbane, QLD, Australia; School of Nursing and Midwifery (M Chutiyami PhD), School of Biomedical Engineering (N Tran MSc), University of Technology Sydney, Sydney, NSW, Australia; Department of Biochemistry and Microbiology (M M Claassens PhD), University of Namibia, Windhoek, Namibia; Department of Paediatrics and Child Health (M M Claassens PhD), Stellenbosch University, Tygerberg, South Africa; Nova Medical School (J Conde PhD), Nova University of Lisbon, Lisbon, Portugal; School of Medicine and Surgery (P A Cortesi PhD, A Lafranconi MD, Prof L G Mantovani DSc), University of Milan Bicocca, Monza, Italy; Department of Family Medicine and Public Health (Prof M H Criqui MD), University of California San Diego, La Jolla, CA, USA; Therapeutic and Diagnostic Technologies Department (Prof N Cruz-Martins PhD), Cooperativa de Ensino Superior Politécnico e Universitário (Polytechnic and University Higher Education Cooperative), Gandra, Portugal; School of Clinical Medicine (S Dai PhD), Hangzhou Normal University, Hangzhou, China; IRCCS Istituto Ortopedico Galeazzi (G Damiani MD), Galeazzi Orthopedic Institute IRCCS (University of Milan), Milan, Italy; Department of Dermatology (G Damiani MD), Lerner College of Medicine (L Göbölös PhD), Harrington Heart and Vascular Institute (A Guha MD), Department of Quantitative Health Science (X Liu PhD), Department of Neonatology (I Qattea MD), Department of Nutrition and Preventive Medicine (Prof J Sanabria MD), Case Western Reserve University, Cleveland, OH, USA; Department of Biochemistry (S Das MD), Ministry of Health and Welfare, New Delhi, India; Department of Radiology (M Dashti MD, A Ghasemzadeh MD, M Mirza-Aghazadeh-Attari MD, A Zarrintan MD), Department of Health Policy and Management (L Doshmangir PhD), Department of Immunology (F Jadidi-Niaragh PhD), School of Management and Medical Informatics (L R Kalankesh PhD), Social Determinants of Health Research Center (S Karimi PhD), Molecular Medicine Research Center (S Pirouzpanah PhD), Department of Health Information Technology (P Rezaei Hachesu PhD), Tabriz University of Medical Sciences, Tabriz, Iran; Department of Population and Development (C A Dávila-Cervantes PhD), Latin American Faculty of Social Sciences Mexico, Mexico City, Mexico; Health Research Institute (K Davletov PhD), Asfendiyarov Kazakh National Medical University, Almaty, Kazakhstan; Australian Institute for Suicide Research and Prevention (Prof D De Leo DSc), Griffith University, Mount Gravatt, QLD, Australia; Medical College (S Debopadhaya BS), Albany Medical College, Albany, NY, USA; Centre for Interdisciplinary Research in Basic Sciences (CIRBSc) (F Deeba PhD), Jamia Millia Islamia, Delhi, India; Department of Food, Environmental and Nutritional Sciences (C Del Bo' PhD), Università degli Studi di Milano (University of Milan), Milano, Italy; School of Medicine (I Delgado-Enciso DSc), University of Colima, Colima, Mexico; Department of Research (I Delgado-Enciso DSc), Colima State Health Services, Colima, Mexico; Department of Neurosurgery (A K Demetriades MD), Global Health Governance Programme (J Patel BSc), Usher Institute (Prof C R Simpson PhD), College of Medicine and Veterinary Medicine (G Verras MD), University of Edinburgh, Edinburgh, UK; Department of Neurosurgery (A K Demetriades MD), National Health Service (NHS) Scotland, Edinburgh, UK; St Paul's Eye Unit (N Dervenis MD), Royal Liverpool University Hospital, Liverpool, UK; Department of Ophthalmology (N Dervenis MD), Second Department of Cardiology (D Patoulias PhD),

Aristotle University of Thessaloniki, Thessaloniki, Greece; Graduate Medical Education (H D Desai MD), Gujarat Adani Institute of Medical Sciences, Bhuj, India; Division of Cardiology (R Desai MBBS), Atlanta Veterans Affairs Medical Center, Decatur, GA, USA; National Centre for AIDS and STD Control (K Deuba DrPH), Save the Children, Kathmandu, Nepal; Division of Pathology (K Dhama PhD), ICAR-Indian Veterinary Research Institute, Bareilly, India; Department of Community Medicine (Prof S D Dharmaratne MD), University of Peradeniya, Peradeniya, Sri Lanka; Department of Pharmacy Practice (S Dhingra PhD), National Institute of Pharmaceutical Education and Research, Hajipur, India; Toxicology Research Unit (TOXRUN) (Prof D Dias da Silva PhD), Cooperativa de Ensino Superior Politécnico e Universitário (University Polytechnic Higher Education Cooperative) (CESP), Gandra, Portugal; Faculty of Science (Prof D Diaz PhD), School of Medicine (Prof R Lozano MD), National Autonomous University of Mexico, Mexico City, Mexico; Department of Medicine (T H Do MD), Can Tho University of Medicine and Pharmacy, Can Tho, Vietnam; Center for Health Sciences (C B do Prado MSc), Department of Integrated Health Education (Prof L B Salaroli PhD), Federal University of Espírito Santo, Vitória, Brazil; School of Elderly Care Services and Management (W Dong MD), Nanjing University of Chinese Medicine, Nanjing, China; Health Science Center (D Dongarwar MS), University of Texas, Houston, TX, USA; Cardio-Thoraco-Vascular Department (Prof M D'Oria MD), Azienda Sanitaria Universitaria Giuliano Isontina, Trieste, Italy; Department of Forensic Medicine and Toxicology (H L Dsouza MD, V Krishna MD, P H Shetty MD), Department of General Medicine (J Jeganathan MD), Department of Community Medicine (N Joseph MD, N Kumar MD, R Motappa MD, R Thapar MD), Department of Anatomy (B Murlimanju MD), Department of Internal Medicine (M M R Reddy MD), Kasturba Medical College, Mangalore (Prof B Unnikrishnan MD), Manipal Academy of Higher Education, Mangalore, India; Department of Forensic Medicine and Toxicology (H L Dsouza MD), Kasturba Medical College Mangalore, Mangalore, India; Office of Institutional Analysis (J Dube MA), University of Windsor, Windsor, ON, Canada; Post-graduate Program in Health Sciences (S C Dumith PhD), Federal University of Rio Grande, Rio Grande, Brazil; School of Medicine (Prof A R Duraes PhD), Federal University of Bahia, Salvador, Brazil; Department of Internal Medicine (Prof A R Duraes PhD), Escola Bahiana de Medicina e Saúde Pública (Bahiana School of Medicine and Public Health), Salvador, Brazil; Department of Biotechnology (S Duraisamy PhD), SRM Institute of Science and Technology, Kattankulathur, India; Department of Infection and Tropical Medicine (O C Durojaiye MPH), School of Health and Related Research (J O Oguta MSc), Department of Psychology (A Yadollahpour PhD), University of Sheffield, Sheffield, UK; Department of Conservative Dentistry with Endodontics (A M Dziedzic DSc), Medical University of Silesia, Katowice, Poland; Department of Psychiatry (E Eboreime PhD, E Tsermpini PhD), Dalhousie University, Halifax, NS, Canada; Department of Psychiatry (E Eboreime PhD), University of Alberta, Edmonton, AB, Canada; Department of Orthopaedic Surgery (A Ebrahimi MD), Department of Radiology (X Liu PhD), Division of Cardiology (D H Nguyen BS), Massachusetts General Hospital, Boston, MA, USA; School of Health Sciences (H A Edinur PhD), Universiti Sains Malaysia (University of Science Malaysia), Kubang Kerian, Malaysia; Centre for Global Health Inequalities Research (CHAIN) (Prof T Eikemo PhD), Department of Neuromedicine and Movement Science (Prof T J Steiner PhD), Norwegian University of Science and Technology, Trondheim, Norway; Department of Orthodontics (E Eini DDS), Alimentary Tract Research Center (Z Shokati Eshkiki PhD), Ahvaz Jundishapur University of Medical Sciences, Ahvaz, Iran; Faculty of Science and Health (M Ekholuenetale PhD), University of Portsmouth, Hampshire, UK; Department of Internal Medicine and Hematology Unit (Prof G M T ElGohary MD), Department of Neuropsychiatry (Prof G ELNahas MD), Department of Entomology (A M Samy PhD), Medical Ain Shams Research Institute (MASRI) (A M Samy PhD), Department of Neurology (Prof A S Shalash PhD), Ain Shams University, Cairo, Egypt; Section of

Adult Hematology (Prof G M T ElGohary MD), Department of Physiology (Prof S A Meo PhD), Pediatric Intensive Care Unit (M Temsah MD), King Saud University, Riyadh, Saudi Arabia; Faculty of Medicine (M Elhadi MD), Department of Radiology (A Msherghi MD), University of Tripoli, Tripoli, Libya; Department of Pediatric Dentistry (Prof O A A Elmeligy PhD), Rabigh Faculty of Medicine (A A Malik PhD), Department of Dental Public Health (Z S Natto DrPH), Department of Community Medicine (S Samargandy PhD), King Abdulaziz University, Jeddah, Saudi Arabia; Executive Committee (Prof G ELNahas MD), International Association for Women Mental Health, New York, MD, USA; Department of Clinical Pathology (M Elshaer MD), Department of Cardiology (Prof M M Ramadan PhD), Faculty of Pharmacy (M A Saleh PhD), Mansoura University, Mansoura, Egypt; Department of Infectious Diseases and Public Health (I Elsohaby PhD), City University of Hong Kong, Hong Kong, China; Department of Animal Medicine (I Elsohaby PhD), Cardiovascular Department (Prof A M A Saad MD), Zagazig University, Zagazig, Egypt; Lincoln International Institute for Rural Health (L Engelbert Bain PhD), University of Lincoln, Lincoln, UK; Department of International Cyber Education (R Erkhembayar MD), Mongolian National University of Medical Sciences, Ulaanbaatar, Mongolia; Independent Consultant, Bologna, Italy (N Fabin MD); Research Centre for Healthcare and Community (A F Fagbamigbe PhD), Faculty of Health and Life Sciences (O P Kurmi PhD), Coventry University, Coventry, UK; Epidemiology and Biostatistics Unit (L Falzone PhD), IRCCS Pascale, Naples, Italy; Center for Global Health Research (M Fareed PhD), Saveetha Medical College and Hospitals (S R Pandi-Perumal MSc), Saveetha Dental College and Hospitals (M R Tovani-Palone PhD), Saveetha Institute of Medical and Technical Sciences (SIMATS), Chennai, India; Dissemination Division (C S e Farinha MSc), National Institute of Statistics, Lisbon, Portugal; Activity Planning and Control Unit (C S e Farinha MSc), Directorate-General of Health (DGS), Lisbon, Portugal; Department of Psychology (Prof A Faro PhD), Federal University of Sergipe, São Cristóvão, Brazil; Social and Administrative Pharmacy (P Farrokhi PharmD), Department of Epidemiology and Community Health (R R Parikh MD), Department of Surgery (J Rickard MD), University of Minnesota, Minneapolis, MN, USA; Clinical Pharmacy and Pharmacy Practice (P Farrokhi PharmD), Department of Environmental Health Engineering (A Fatehizadeh PhD), School of Medicine (G Ghasempour Dabaghi MD, M Rabiee Rad MD), Cardiac Rehabilitation Research Center (K Mehrabani-Zeinabad PhD, Prof M Sadeghi MD), Faculty of Medicine (A Nasrollahizadeh MD), Isfahan University of Medical Sciences, Isfahan, Iran; Centre for Health Policy Research (Prof P Ward PhD), Torrens University Australia, Adelaide, SA, Australia (N K Fauk MSc); Institute of Resource Governance and Social Change, Kupang, Indonesia (N K Fauk MSc); National Institute for Stroke and Applied Neurosciences (Prof V L Feigin PhD), Auckland University of Technology, Auckland, New Zealand; Research Center of Neurology, Moscow, Russia (Prof V L Feigin PhD); National Institute of Environmental Health (X Feng PhD), National Center for Chronic and Noncommunicable Disease Control and Prevention (N Wang PhD, P Ye MPH), Chinese Center for Disease Control and Prevention, Beijing, China (Prof S Liu PhD); Department of Social Sciences (Prof N Ferreira PhD), University of Nicosia, Nicosia, Cyprus; Institute of Public Health (F Fischer PhD), Department of Surgery (N Haep MD), Department of Neurology (S Samadzadeh MD), Charité Universitätsmedizin Berlin (Charité Medical University Berlin), Berlin, Germany; School of Social Sciences (J Flavel PhD), Stretton Health Equity, Adelaide, SA, Australia; Center for Research in Indigenous Health (D Flood MD), Maya Health Alliance, Tecpán, Guatemala; Department of Internal Medicine (D Flood MD), University of Michigan, Ann Arbor, MI, USA; Institute of Gerontology (N A Foigt PhD), National Academy of Medical Sciences of Ukraine, Kyiv, Ukraine; Department of Child Dental Health (Prof M O Folayan FWACS), Obafemi Awolowo University, Ile-Ife, Nigeria; Clinical Science Department (Prof M O Folayan FWACS), Nigerian Institute of Medical Research, Yaba, Nigeria; Healthcare Innovation Department (D Fortuna MSc),

Regional Agency for Health and Social Care of Emilia-Romagna, Bologna, Italy; Department of Biotechnological and Applied Clinical Sciences (DISCAB) (M Foschi MD), Department of Neurology (Prof S Sacco MD), University of L'Aquila, L'Aquila, Italy; Department of Neuroscience (M Foschi MD), Hospital Santa Maria delle Croci, Ravenna, Italy; Center for Health Technology and Services Research (CINTESIS), Porto, Portugal (A Freitas PhD); Department of Dermatology (T Fukumoto PhD), Kobe University, Kobe, Japan; Division of Ophthalmology (J M Furtado MD), University of São Paulo, Ribeirão Preto, Brazil; Health Services Management Training Centre (P A Gaal PhD, T Joo PhD, J Lám PhD, T Palicz MD), Semmelweis University, Budapest, Hungary; Department of Applied Social Sciences (P A Gaal PhD), Sapientia Hungarian University of Transylvania, Târgu-Mureş, Romania; Department of Community Medicine (Prof M A Gadanya FMCPH), Aminu Kano Teaching Hospital, Kano, Nigeria; Institute of Applied Health Sciences (S Gaihre PhD), University of Aberdeen, Aberdeen, UK; Department of Food Technology (Y Galali ResM, B A Sadee PhD), Salahaddin University-Erbil, Erbil, Iraq; Department of Nutrition and Dietetics (Y Galali ResM, B A Sadee PhD), Cihan University-Erbil, Erbil, Iraq; Department of Research Publication (M Ganbat MPH), Zaigal Research Institute, Ulaanbaatar, Mongolia; Department of Community Medicine and Family Medicine (A P Gandhi MD), All India Institute of Medical Sciences, Nagpur, India; Institute of Health and Wellbeing (B Ganesan PhD), Federation University, Churchill, VIC, Australia; Department of Endocrinology (Prof M Ganie MD), Sheri Kashmir Institute of Medical Sciences, Srinagar J& K, India; Department of Endocrinology and Metabolism (Prof M Ganie MD), All India Institute of Medical Sciences, Delhi, India; Department of General Medicine (M Ganiyani MD), Grant Medical College & Sir J.J. Group of Hospitals, Mumbai, India; Department of Midwifery (M W Gebregergis MSc), Department of Medical Laboratory Sciences (H Negash MSc), Adigrat University, Adigrat, Ethiopia; Department of Environmental Health (M Gebrehiwot DSc), Wollo University, Dessie, Ethiopia; Department of Public Health Nutrition (T B B Gebremariam MPH), Aksum University, Mekelle, Ethiopia; Department of Reproductive and Family Health (T G Gebremeskel PhD), Axum College of Health Science, Axum, Ethiopia; Department of Human Physiology (Y Gela MSc), Department of Pharmacology (Z D Kifle MSc), School of Nursing (H B Netsere MS), University of Gondar, Gondar, Ethiopia; Department of Dermatology (Prof S R Georgescu PhD), "Victor Babes" Clinical Hospital of Infectious and Tropical Diseases, Bucharest, Romania; School of Psychology (A Getachew Obsa MA), Department of Medical Laboratory Science (M Getie MSc), School of Public Health (D H Woldegebreal MPH), Addis Ababa University, Addis Ababa, Ethiopia; Infectious Disease Research Center (Prof K Ghadiri MD), Pediatric Department (Prof K Ghadiri MD), Universal Scientific Education and Research Network (USERN) (P Goleij MSc), Department of Rehabilitation and Sports Medicine (M Mirzaei MSc), Department of Infectious Disease (Prof S Vaziri MD), Kermanshah University of Medical Sciences, Kermanshah, Iran; Center of Health Management (K Y Ghailan PhD), Aden University, Aden, Yemen; Department of Radiology (A Gholamrezanezhad MD), University of Southern California, Los Angeles, CA, USA; Department of Medicine (R M Gibson PhD), Stanford University, Palo Alto, CA, USA; Department of Nursing (A Girmay MSc, G M Tsegay MSc), Department of Adult Health Nursing (A H Weldemariam MSc), Aksum University, Aksum, Ethiopia; Department of Cardiac Surgery (L Göbölös PhD), Cleveland Clinic Abu Dhabi, Abu Dhabi, United Arab Emirates; Westmead Applied Research Centre (M A Godinho MBBS), University of Sydney, Westmead, NSW, Australia; Department of Health Systems and Policy Research (M Golechha PhD), Indian Institute of Public Health, Gandhinagar, India; Department of Genetics (P Goleij MSc), Sana Institute of Higher Education, Sari, Iran; Department of Exercise and Health Sciences (P N Gona PhD), University of Massachusetts Boston, Boston, MA, USA; Department of Epidemiology (Prof A C Goulart PhD), Universidade de São Paulo (University of São Paulo), São Paulo, Brazil; Department of

Dermatology (A Grada MD), Health Informatics Lab (T Javaheri PhD), Boston University, Boston, MA, USA; Department of Public Health and Preventive Medicine (Prof M Grivna PhD), Charles University, Prague, Czech Republic; Department of Epidemiology and Biostatistics (S Guan MD), Anhui Medical University, Hefei, China; Post Graduate School of Public Health (G Guarducci MD), University of Siena, Siena, Italy; Department of Family and Community Medicine (M I M Gubari PhD), University Of Sulaimani, Sulaimani, Iraq; Division of Cardiovascular Medicine (A Guha MD), Ohio State University, Columbus, OH, USA; Health Directorate (S Guicciardi MD), Local Health Authority of Bologna, Bologna, Italy; Department of General Surgery (S Gulati MD), Dignity Health, Phoenix, AZ, USA; Diagnostic Radiology and Nuclear Medicine (D Gulisashvili MD), School of Medicine (P Habibzadeh MD), University of Maryland, Baltimore, MD, USA; Department of Community Medicine (D A Gunawardane MD), University of Peradeniya, Kandy, Sri Lanka; Department of Internal Medicine (A K Gupta PharmD), Shree Guru Gobind Singh Tricentenary University, Gurugram, India; Non-communicable Division (NCD) (A K Gupta PharmD), Indian Council of Medical Research, Delhi, India; Department of Public Health (B Gupta PhD), Torrens University Australia, Melbourne, VIC, Australia; Department of Internal Medicine (I Gupta MD), Independent Consultant, Bharatpur, India; NGO (I Gupta MD), Independent Consultant, Delhi, India; Department of Internal Medicine (M Gupta MD), Lerner Research Institute (X Liu PhD), Cleveland Clinic, Cleveland, OH, USA; Department of Preventive Cardiology (Prof R Gupta MD), Eternal Heart Care Centre & Research Institute, Jaipur, India; Department of Medicine (Prof R Gupta MD), Mahatma Gandhi University Medical Sciences, Jaipur, India; School of Medicine (V Gupta PhD), Institute for Mental and Physical Health and Clinical Translation (IMPACT) (W Marx PhD), Deakin University, Geelong, VIC, Australia; School of Biotechnology (V Gupta PhD), Dublin City University, Glasnevin, Ireland; Faculty of Medicine Health and Human Sciences (Prof V K Gupta PhD), Macquarie University, Sydney, NSW, Australia; Department of Epidemiology and Psychosocial Research (R A Gutiérrez PhD), Ramón de la Fuente Muñiz National Institute of Psychiatry, Mexico City, Mexico; Global Virus Network, Middle East Region, Shiraz, Iran (F Habibzadeh MD); Department of Pharmacology and Toxicology (R Haddadi PhD), Research Center for Molecular Medicine (A Taherkhani PhD), Hamadan University of Medical Sciences, Hamadan, Iran; Department of Clinical Pharmacology and Medicine (Prof N R Hadi PhD), University of Kufa, Najaf, Iraq; Clinician Scientist Program (N Haep MD), Berlin Institute of Health, Berlin, Germany; College of Medicine (A Hafiz PhD), Umm AL Qura University, Makkah, Saudi Arabia; Department of Infectious Disease Epidemiology (S Haller MD), Robert Koch Institute, Berlin, Germany; Department of Public Health (S Haller MD), Charité Institute of Public Health, Berlin, Germany; Centre for Neuromuscular and Neurological Disorders (Prof G J Hankey MD), The University of Western Australia, Perth, WA, Australia; Perron Institute for Neurological and Translational Science, Perth, WA, Australia (Prof G J Hankey MD); Department of Biochemistry and Molecular Biology (Prof M Hannan PhD), Bangladesh Agricultural University, Mymensingh, Bangladesh; Department of Anatomy (Prof M Hannan PhD), Dongguk University, Gyeongju, South Korea; Department of Population Science and Human Resource Development (Prof M Haque PhD, Prof M Rahman PhD, M Rahman DrPH), Department of Mathematics (M Kuddus PhD), University of Rajshahi, Rajshahi, Bangladesh; Medical Research Unit (H Harapan PhD), Universitas Syiah Kuala (Syiah Kuala University), Banda Aceh, Indonesia; Research Unit (J M Haro MD), University of Barcelona, Barcelona, Spain; Biomedical Research Networking Center for Mental Health Network (CiberSAM), Barcelona, Spain (J M Haro MD); Department of Sports Science and Clinical Biomechanics (Prof J Hartvigsen PhD, Prof S T Skou PhD), Department of Neurology (S Samadzadeh MD), University of Southern Denmark, Odense, Denmark; Research Department (Prof J Hartvigsen PhD), Nordic Institute of Chiropractic and Clinical Biomechanics, Odense, Denmark;

Department of Zoology and Entomology (A I Hasaballah PhD), Botany and Microbiology Department (A M E Shehabeldine PhD), Al-Azhar University, Cairo, Egypt; Department of Pharmaceutical Technology (I Hasan MPharm), University of Dhaka, Dhaka, Bangladesh; Department of Radiology (M Hasanian MD), Arak University of Medical Sciences, Arak, Iran; Department of Pharmacy (Prof M S Hasnain PhD), Palamau Institute of Pharmacy, Daltonganj, India; Department of Diagnostic and Interventional Radiology and Neuroradiology (J Haubold MD, Prof B M Schaarschmidt MD), Institute of Artificial Intelligence in Medicine (J Haubold MD), University Hospital Essen, Essen, Germany; Skaane University Hospital (R J Havmoeller PhD), Skaane County Council, Malmö, Sweden; Institute of Pharmaceutical Sciences (K Hayat MS), University of Veterinary and Animal Sciences, Lahore, Pakistan; Department of Pharmacy Administration and Clinical Pharmacy (K Hayat MS), Xian Jiaotong University, Xian, China; Faculty of Kinesiology (Prof J J Hebert PhD), University of New Brunswick, Fredericton, NB, Canada; School of Allied Health (Prof J J Hebert PhD), Murdoch University, Murdoch, WA, Australia; Independent Consultant, Santa Clara, CA, USA (G Heidari MD); Institute of Psychology (B Helfer PhD), University of Wroclaw, Wroclaw, Poland; Meta Research Centre (B Helfer PhD), University of Wrocław, Wroclaw, Poland; Department of Medicine (M Hemmati MD), MedStar Health, Columbia, MD, USA; Department of Medicine (M Hemmati MD), Georgetown University, Washington, DC, USA; Department of Microbiology (K Hezam PhD), Taiz University, Taiz, Yemen; School of Medicine (K Hezam PhD), Nankai University, Tianjin, China; Division for Health Service Promotion (Y Hiraike PhD), Department of Mental Health (Prof N Kawakami PhD), Department of Global Health Policy (S Nomura PhD, S K Rauniyar PhD), University of Tokyo, Tokyo, Japan; School of Dentistry (N Q Hoan DDS), Hanoi Medical University, Hanoi, Vietnam; Social and Environmental Health Research (M Hossain MPH), Nature Study Society of Bangladesh, Khulna, Bangladesh; Department of Health Promotion and Community Health Sciences (M Hossain MPH), Texas A&M University, College Station, TX, USA; School of Health and Society (H Hosseinzadeh PhD), University of Wollongong, Wollongong, NSW, Australia; Institute of Research and Development (Prof M Hosseinzadeh PhD), Duy Tan University, Da Nang, Vietnam; Department of Computer Science (Prof M Hosseinzadeh PhD), University of Human Development, Sulaymaniyah, Iraq; Department of Clinical Legal Medicine (S Hostiuc PhD), National Institute of Legal Medicine Mina Minovici, Bucharest, Romania; Department of Biological Sciences and Chemistry (Prof J Hussain PhD), School of Pharmacy (A K Philip PhD), University of Nizwa, Nizwa, Oman; Department of Biomolecular Sciences (N R Hussein PhD), University of Zakho, Zakho, Iraq; International Master Program for Translational Science (H Huynh BS), International Ph.D. Program in Medicine (L Minh MD), Research Center for Artificial Intelligence in Medicine (L Minh MD), School of Public Health (Y L Samodra MPH, Y L Samodra MPH), Department of Clinical Pharmacy (M A Sarasmita PharmD), Taipei Medical University, Taipei, Taiwan; Department of Occupational Safety and Health (Prof B Hwang PhD), China Medical University, Taichung, Taiwan; Department of Occupational Therapy (Prof B Hwang PhD), Asia University, Taiwan, Taichung, Taiwan; Department of Clinical Effectiveness (A I Ikiroma PhD), NHS National Services Scotland, Edinburgh, Scotland; Division of Infectious Diseases (K S Ikuta MD), Veterans Affairs Greater Los Angeles, Los Angeles, CA, USA; Faculty of Medicine (I M Ilic PhD, Prof M M Santric-Milicevic PhD), School of Public Health and Health Management (Prof M M Santric-Milicevic PhD), University of Belgrade, Belgrade, Serbia; Department of Epidemiology (Prof M D Ilic PhD), University of Kragujevac, Kragujevac, Serbia; College of Pharmacy (M Imam PhD), Department of Electrical Engineering (I Malik PhD), Prince Sattam bin Abdulaziz University, Al Kharj, Saudi Arabia; Faculty of Pharmacy (L M Irham BPharm), University of Ahmad Dahlan, Yogyakarta, Indonesia; School of Pharmacy (M R Islam PhD), BRAC University, Dhaka, Bangladesh; Institute for Physical Activity and Nutrition (S Islam PhD), Department of Psychology (M A

Stokes PhD), Deakin University, Burwood, VIC, Australia; Department of Surveillance and Health Services Research (F Islami PhD), American Cancer Society, Atlanta, GA, USA; Clinical Laboratory (F Ismail PhD), Tobruk University, Tobruk, Libya; Department of Blood Transmitted Diseases (F Ismail PhD), National Center for Disease Control, Tobruk, Libya; Department of Clinical Pharmacy & Pharmacy Practice (Prof N Ismail PhD), Asian Institute of Medicine, Science and Technology, Kedah, Malaysia; Malaysian Academy of Pharmacy, Puchong, Malaysia (Prof N Ismail PhD); Department of Health Services Research (M Iwagami PhD), Research and Development Center for Health Services (Prof K Yamagishi MD), University of Tsukuba, Tsukuba, Japan; School of Health Systems and Public Health (C C D Iwu MPH), Department of Nuclear Medicine (P W Mahasha PhD), University of Pretoria, Pretoria, South Africa; Department of Biotechnology (M Iyer PhD, S Muthu MS), Karpagam Academy of Higher Education (Deemed to be University), Coimbatore, India; Department of Health Studies (K H Jacobsen PhD), University of Richmond, Richmond, VA, USA; Shiraz Neuroscience Research Center (M Jafarinia PhD, R Tavakoli Oliaee PhD), Health Policy Research Center (H Kasraei MD, Y Sarikhani PhD), Department of Epidemiology and Biostatistics (H Raeisi Shahraki PhD), Department of Biostatistics (E Sadeghi PhD), Non-communicable Disease Research Center (S G Sepanlou MD), Shiraz University of Medical Sciences, Shiraz, Iran; Department of Nephrology (K Jaggi MD), San Mateo Medical Center, San Mateo, CA, USA; Department of Nephrology (K Jaggi MD), Mills Peninsula Medical Center, Burlingame, CA, USA; College of Medicine and Medical Sciences (H Jahrami PhD), Arabian Gulf University, Manama, Bahrain; Department of Psychiatry (Z Saif MBA), Ministry of Health, Manama, Bahrain (H Jahrami PhD); Department of Leukemia (A Jain MD), The University of MD Anderson Cancer Center, Houston, TX, USA; Statistics Unit (N Jain MD), Riga Stradins University, Riga, Latvia; Department of Health and Safety (A A Jairoun PhD), Dubai Municipality, Dubai, United Arab Emirates; Centre for Community Medicine (A Jaiswal MD), Department of Psychiatry (Prof R Sagar MD), Department of Radiation Oncology (A Shankar MD), All India Institute of Medical Sciences, New Delhi, India; The World Academy of Sciences UNESCO, Trieste, Italy (Prof M Jakovljevic PhD); Shaanxi University of Technology, Hanzhong, China (Prof M Jakovljevic PhD); School of Pharmacy and Pharmacology (A Jatau PhD), Menzies Institute for Medical Research (F Pan PhD), University of Tasmania, Hobart, TAS, Australia; Department of Physiology (Prof S Javadov PhD), University of Puerto Rico Medical Sciences Campus, San Juan, Puerto Rico; Centre of Studies and Research (S Jayapal PhD), Ministry of Health, Muscat, Oman; Department of Biochemistry (Prof S Jayaram MD), Government Medical College, Mysuru, India; Department of Nutrition (A Jeyakumar PhD), University of Nevada Reno, Reno, NV, USA; Department of Cardiovascular Medicine (A K Jha MD), Saint Vincent Hospital, Worcester, MA, USA; Melbourne School of Population and Global Health (H Jiang PhD, L Reifels PhD), School of Health Sciences (A Meretoja MD), University of Melbourne, Melbourne, VIC, Australia; Department of Global Health (Y Jin PhD), China Center for Health Development Studies (Y Yao MD), School of Public Health (H Zhang MS), Peking University, Beijing, China; Institute of Molecular and Clinical Ophthalmology Basel, Basel, Switzerland (Prof J B Jonas MD); Department of Ophthalmology (Prof J B Jonas MD), Heidelberg University, Mannheim, Germany; Hungarian Health Management Association (T Palicz MD), Hungarian Health Management Association, Budapest, Hungary (T Joo PhD); Department of Gastroenterology and Hepatology (A Joseph MD), Department of Radiology (S Ramasamy MD), Stanford University, Stanford, CA, USA; Department of Economics (C E Joshua BSc), National Open University, Benin City, Nigeria; Department of Family Medicine and Public Health (J J Jozwiak PhD), University of Opole, Opole, Poland; Institute of Family Medicine and Public Health (M Jürisson PhD), University of Tartu, Tartu, Estonia; School of Public Health (Z Kabir PhD), University College Cork, Cork, Ireland; Department of Oral and Maxillofacial Pathology (V Kadashetti MDS), Department of Public

Health Dentistry (Prof K M Shivakumar PhD), Krishna Vishwa Vidyapeeth (Deemed to be University), Karad, India; Department of Dermatology (F Kaliyadan MD), King Faisal University, Hofuf, Saudi Arabia; Department of Endocrinology (S Kalra DM), Bharti Hospital Karnal, Karnal, India; Department of Noncommunicable Diseases (K Kamenov PhD), World Health Organization (WHO), Geneva, Switzerland; Department of Public Health (N Kamyari PhD), Abadan University of Medical Sciences, Abadan, Iran; School of Graduate Studies (T Kanagasabai PhD), Meharry Medical College, Nashville, TN, USA; Sydney Eye Hospital (H Kandel PhD), South Eastern Sydney Local Health District, Sydney, NSW, Australia; Cardiology Division (A R Kanmanthareddy MD), Creighton University, Omaha, NE, USA; College of Public Health (A R Kanmanthareddy MD), Department of Environmental, Agricultural and Occupational Health (J Taiba MPH), University of Nebraska Medical Center, Omaha, NE, USA; Faculty of Dentistry (K K Kanmodi MPH), University of Puthisastra, Phnom Penh, Cambodia; Office of the Executive Director (K K Kanmodi MPH), Campaign for Health and Neck Cancer Education (CHANCE) Programme (A A Salami BDS), Cephas Health Research Initiative Inc, Ibadan, Nigeria; The Hansjörg Wyss Department of Plastic and Reconstructive Surgery (R S Kantar MD), Nab'a Al-Hayat Foundation for Medical Sciences and Health Care, New York, NY, USA; Cleft Lip and Palate Surgery Division (R S Kantar MD), Global Smile Foundation, Norwood, MA, USA; School of Health Professions and Human Services (I M Karaye MD), Hofstra University, Hempstead, NY, USA; Department of Anesthesiology (I M Karaye MD), Montefiore Medical Center, Bronx, NY, USA; Surgery Research Unit (Prof J H Kauppila MD), University of Oulu, Oulu, Finland; International Research Center of Excellence (G A Kayode PhD), Institute of Human Virology Nigeria, Abuja, Nigeria; Julius Centre for Health Sciences and Primary Care (G A Kayode PhD), Copernicus Institute of Sustainable Development (G Koren PhD), Utrecht University, Utrecht, Netherlands; Department of Healthcare Services Management (L Keikavoosi-Arani PhD), Non-communicable Diseases Research Center (P Mardi MD), Alborz University of Medical Sciences, Karaj, Iran; Eye Unit (Prof J H Kempen MD), MyungSung Medical College, Addis Ababa, Ethiopia; Centre for Adolescent Health (J A Kerr PhD), Department of Critical Care and Neurosciences (Prof R G Weintraub MB), Murdoch Childrens Research Institute, Parkville, VIC, Australia; Department of Psychological Medicine (J A Kerr PhD), University of Otago, Christchurch, New Zealand; Department of Biomedical Informatics (K Keshtkar BSc), Arizona State University, Phoenix, AZ, USA; Department of Human Nutrition (E Kesse-Guyot PhD), National Research Institute for Agriculture, Food and Environment, Jouy-en-Josas, France; University Sorbonne Paris Nord (E Kesse-Guyot PhD), Department of Health, Medicine and Human Biology (M Touvier PhD), Sorbonne Paris Nord University, Bobigny, France; Amity Institute of Forensic Sciences (H Khajuria PhD, B P Nayak PhD, P Puri PhD), Amity Institute of Pharmacy (K Munjal PhD), Amity Institute of Public Health (M Shannawaz PhD), Amity University, Noida, India; College of Health Sciences (N Khalid PhD), Abu Dhabi University, Adu Dhabi, United Arab Emirates; Department of Biostatistics (Prof A Khalilian PhD), Mazandaran University of Medical Sciences, Mazandaran, Iran; Research Center for Hydatid Disease in Iran (F Khamesipour PhD), Kerman University of Medical Sciences, Kerman, Iran; Department of Botany (Prof I Khan PhD), Abdul Wali Khan University Mardan, Mardan, Pakistan; Primary Care Department (M A Khan MSc), NHS North West London, London, UK; College of Health, Wellbeing and Life Sciences (Prof K Khatab PhD), Sheffield Hallam University, Sheffield, UK; College of Arts and Sciences (Prof K Khatab PhD), Ohio University, Zanesville, OH, USA; Department of Basic Medical Sciences (M M Khatatbeh PhD), Yarmouk University, Irbid, Jordan; Department of Biochemistry (F Khidri PhD), Liaquat University Of Medical and Health Sciences, Jamshoro, Pakistan; Cardiovascular Disease Initiative (M Kim MD), Broad Institute of MIT and Harvard, Cambridge, MA, USA; Millennium Prevention, Westwood, MA, USA (R W Kimokoti MD); School of Health Sciences (Prof A Kisa PhD), Kristiania

University College, Oslo, Norway; Department of International Health and Sustainable Development (Prof A Kisa PhD), Tulane University, New Orleans, LA, USA; Department of Nursing and Health Promotion (S Kisa PhD), Faculty of Health Sciences (Prof A W Wolf PhD), Oslo Metropolitan University, Oslo, Norway; Department of Disease Burden (A S Knudsen PhD), GBD Collaborating Unit (Prof S E Vollset DrPH), Norwegian Institute of Public Health, Bergen, Norway; Global Healthcare Consulting, New Delhi, India (S Kochhar MD); Department of Neurology (H Koh PhD), Baylor College of Medicine, Houston, TX, USA; Department of General Practice – Family Medicine (Prof O Korzh DSc), Kharkiv National Medical University, Kharkiv, Ukraine; Independent Consultant, Jakarta, Indonesia (S Kosen MD); Kasturba Medical College, Mangalore (S Koulmane Laxminarayana MD), Manipal Academy of Higher Education, Udupi, India; Department of Anthropology (Prof K Krishan PhD), Institute of Forensic Science & Criminology (V Sharma PhD), Panjab University, Chandigarh, India; Department of Anesthesiology (V Krishnamoorthy MD), Department of Population Health Sciences (J B Lusk MD), Duke Global Health Institute (C Wu PhD), Center for the Study of Aging and Human Development (Y Yao MD), Duke University, Durham, NC, USA; Department of Demography (Prof B Kuate Defo PhD), Department of Social and Preventive Medicine (Prof B Kuate Defo PhD), University of Montreal, Montreal, QC, Canada; Department of Biochemistry (Prof M Kuddus PhD), College of Public Health & Health Informatics (R Kumar PhD), University of Hail, Hail, Saudi Arabia; Department of Pediatrics (I Kuitunen PhD), Kuopio University Hospital, Kuopio, Finland; Institute of Clinical Medicine (I Kuitunen PhD), University of Eastern Finland, Kuopio, Finland; Department of Medicine (V Kulkarni MS), Queensland Health, Brisbane, QLD, Australia; Department of Psychiatry (M Kumar PhD), University of Nairobi, Nairobi, Kenya; Department of Medicine (O P Kurmi PhD), Department of Health Research Methods, Evidence and Impact (E J Mills PhD), Department of Psychiatry and Behavioural Neurosciences (A T Olagunju MD), Health Information Research Unit (O O Olasupo PhD), McMaster University, Hamilton, ON, Canada; Department of Health Services Research and Management (D Kusuma DSc), City University of London, London, UK; Faculty of Public Health (D Kusuma DSc), University of Indonesia, Depok, Indonesia; Nuffield Department of Population Health (B Lacey PhD), Health Economics Research Centre (Prof J A B Rodriguez PhD), University of Oxford, Oxford, UK; National Institute for Health Research (NIHR) Oxford Biomedical Research Centre, Oxford, UK (B Lacey PhD); Institute for Social and Health Sciences (Prof L Laflamme PhD), University of South Africa, Pretoria, South Africa; Department of Health Policy and Strategy (Prof C Lahariya MD), Foundation for People-centric Health Systems, New Delhi, India; SD Gupta School of Public Health (Prof C Lahariya MD), Indian Institute of Health Management Research University, Jaipur, India; School of Digital Science (D T C Lai PhD), Institute of Applied Data Analytics (D T C Lai PhD), Faculty of Science (E Leong PhD), Universiti Brunei Darussalam (University of Brunei Darussalam), Bandar Seri Begawan, Brunei; Indian Council of Medical Research, New Delhi, India (D K Lal MD); Department of Public Health (Prof T Lallukka PhD), Department of Virology (F Zakham PhD), University of Helsinki, Helsinki, Finland (T J Meretoja MD); NEVES Society for Patient Safety, Budapest, Hungary (J Lám PhD); Division of Cancer Epidemiology and Genetics (Q Lan PhD), National Cancer Institute, Rockville, MD, USA; Unit of Genetics and Public Health (Prof I Landires MD), Institute of Medical Sciences, Las Tablas, Panama; Ministry of Health, Herrera, Panama (Prof I Landires MD); Department of Psychiatry and Psychotherapy (B Langguth PhD), University of Regensburg, Regensburg, Germany; Department of Behavioural Sciences and Learning (A Laplante-Lévesque PhD), Linköping University, Linköping, Sweden; Department of Medical Sciences (Prof A O Larsson PhD, Prof J Sundström PhD), Uppsala University, Uppsala, Sweden; Department of Clinical Chemistry and Pharmacology (Prof A O Larsson PhD), Uppsala University Hospital, Uppsala, Sweden; Department of Otorhinolaryngology (S Lasrado MS), Father Muller

Medical College, Mangalore, India; International Society Doctors for the Environment, Arezzo, Italy (P Lauriola MD); Faculty of Medicine (H Le MD, N Le MD), University of Medicine and Pharmacy at Ho Chi Minh City, Ho Chi Minh City, Vietnam (T D T Le MD); Cardiovascular Research Department (H Le MD), Department of Cardiovascular Research (N Le MD), Methodist Hospital, Merrillville, IN, USA; Health Economics Division (L K D Le PhD), Monash University, Burwood, VIC, Australia; Independent Consultant, Ho Chi Minh City, Vietnam (T D T Le MD); College of Optometry (J L Leasher OD), Nova Southeastern University, Fort Lauderdale, FL, USA; Department of Medical Science (M Lee PhD), Ajou University School of Medicine, Suwon, South Korea; Pattern Recognition and Machine Learning Lab (Prof S Lee PhD), Gachon University, Seongnam, South Korea; Department of Precision Medicine (Prof S W Lee MD), Sungkyunkwan University, Suwon-si, South Korea; The Department of Family Medicine (W Lee PhD), University of Texas, Galveston, TX, USA; Department of Preventive Medicine (Prof Y Lee PhD), Korea University, Seoul, South Korea; Center for Youth Substance Abuse Research (J Leung PhD), The University of Queensland, St. Lucia, QLD, Australia; Department of Health Promotion and Health Education (M Li PhD), National Taiwan Normal University, Taipei, Taiwan; National Clinical Research Center for Cardiovascular Diseases (Y Li PhD), Chinese Academy of Medical Sciences, Shenzhen, China; Department of Endocrinology and Metabolism (Y Li PhD), The First Hospital of China Medical University, Shenyang, China; Department of Medicine (L Lim MRCP), Faculty of Medicine (H Shah MS), University of Malaya, Kuala Lumpur, Malaysia; School of Public Health (Prof S Linn DrPH), University of Haifa, Haifa, Israel; School of Life Sciences (G Liu PhD), University of Technology Sydney, Ultimo, NSW, Australia; Center for Evidence-Based Medicine and Clinical Research (R Liu MD), The First Clinical College (M Peng MPH), School of Public Health and Management (Y Yu MS), Hubei University of Medicine, Shiyan, China; Institute for Health and Environment (W Liu PhD), Chongqing University of Science and Technology, Chongqing, China; Department of Molecular Epidemiology (E Llanaj PhD), German Institute of Human Nutrition Potsdam-Rehbrücke, Potsdam, Germany; German Center for Diabetes Research (DZD), München-Neuherberg, Germany (E Llanaj PhD); Department of Internal Medicine (C Lo MD), Kirk Kerkorian School of Medicine at UNLV, Las Vegas, NV, USA; Department of Physical Medicine and Nursing (R López-Bueno PhD), University of Zaragoza, Zaragoza, Spain; Department of Musculoskeletal disorders (R López-Bueno PhD), National Research Centre for the Working Environment, Copenhagen, Denmark; National Institutes of Health (A Loreche BS), University of the Philippines Manila, Manila, Philippines; School of Medicine and Public Health (A Loreche BS), Center for Research and Innovation (V F Pepito MSc), Ateneo De Manila University, Pasig City, Philippines; Department of Health Economics (L Lorenzovici MSc), Syreon Research Romania, Targu Mures, Romania; Department of Doctoral Studies (L Lorenzovici MSc), George Emil Palade University of Medicine, Pharmacy, Science, and Technology of Targu Mures, Targu Mures, Romania; School of Medicine (Prof G Lucchetti PhD), Federal University of Juiz de Fora, Juiz de Fora, Brazil; Department of General Surgery (Prof R Lunevicius DSc), Liverpool University Hospitals NHS Foundation Trust, Liverpool, UK; Department of Epidemiology (h lv BA), Chinese Center for Disease Control and Prevention, Shenyang, China; Centre for Public Health and Wellbeing (Z Ma PhD), University of the West of England, Bristol, UK; 2nd Department of Propaedeutic Surgery (N Machairas PhD), 3rd Department of Cardiology (M Spartalis PhD), University of Athens, Athens, Greece; Laboratório de Farmacognosia (LAQV) (Associated Laboratory for Green Chemistry (Á M Madureira-Carvalho PhD), Universidade do Porto (University of Porto), Porto, Portugal; Department of Human Nutrition Research (J A Magaña Gómez PhD), Autonomous University of Sinaloa, Culiacán, Mexico; Grants, Innovation and Product Development Unit (P W Mahasha PhD), Risk and Resilience in Mental Disorders Unit (Prof D J Stein MD), South African Medical Research Council, Cape Town, South

Africa (C A Nnaji MPH); Department of Public Health (M Maheri PhD), Social Determinants of Health Center (M Mirza-Aghazadeh-Attari MD), Urmia University of Medical Sciences, Urmia, Iran; Cellular and Molecular Biology Research Center (Prof S Mahjoub PhD), Department of Clinical Biochemistry (Prof S Mahjoub PhD, A Mosapour PhD), Social Determinants of Health Research Center (S Mouodi PhD), Babol University of Medical Sciences, Babol, Iran; Department of Clinical and Hospital Pharmacy (M A Mahmoud PhD), Taibah University, Al-Madinah Al-Munawarrah, Saudi Arabia; Cyprus International Institute for Environmental and Public Health (K C Makris PhD), Cyprus University of Technology, Limassol, Cyprus; Rama Medical College Hospital and Research Centre, Uttar Pradesh, India (K Malhotra MBBS); Smidt Heart Institute (Y Manla MD), Cedars-Sinai Medical Center, Los Angeles, CA, USA; Information and Communication Technology Research Pole (Lab-STICC) (Prof A Mansour PhD), ENSTA Bretagne, Brest, France; Laboratory of Public Health (Prof L G Mantovani DSc), Instituto Auxologico Italiano IRCCS (Italian Auxological Institute), Milan, Italy; Biomedical Engineering Research Center (CREB) (H Marateb PhD), Universitat Politècnica de Catalunya (Barcelona Tech - UPC), Barcelona, Spain; Department of Artificial Intelligence (H Marateb PhD), Smart University of Medical Sciences, Tehran, Iran; Department of Economics (Prof G Martinez PhD), Autonomous Technology Institute of Mexico, Mexico City, Mexico; Noncommunicable Diseases and Mental Health Department (R Martinez-Piedra BSc), Pan American Health Organization, Washington, DC, USA; Campus Fortaleza (F R Martins-Melo PhD), Federal Institute of Education, Science and Technology of Ceará, Fortaleza, Brazil; Department of Nutrition and Dietetics (M Martorell PhD), University of Concepcion, Concepción, Chile; Centre for Healthy Living (M Martorell PhD), University of Concepción, Concepción, Chile; Department of Pharmacy (S Maryam PharmD), Bahauddin Zakariya University, Multan, Pakistan; Faculty of Humanities and Health Sciences (Prof R R Marzo MD), Curtin University, Malaysia, Sarawak, Malaysia; Jeffrey Cheah School of Medicine and Health Sciences (Prof R R Marzo MD), School of Pharmacy (Y Wong PhD), Monash University, Subang Jaya, Malaysia; Department of Anatomy, Genetics and Biomedical Informatics (Y Mathangasinghe MD), Department of Surgery (D P Wickramasinghe MD), University of Colombo, Colombo, Sri Lanka; North West Lung Centre (A G Mathioudakis PhD), Manchester University NHS Foundation Trust, Manchester, UK; Department of Medicine (J Mattumpuram MD), University of Louisville, Louisville, KY, USA; Orthopedic Trauma Pathology Department (A Mazzotti PhD), IRCCS, Bologna, Italy; National Centre for Register-based Research (Prof J J McGrath MD), Aarhus University, Aarhus, Denmark; Department of Public Health (T Mekene Meto MPH), Arba Minch University, Arbaminch, Ethiopia; Peru Country Office (W Mendoza MD), United Nations Population Fund (UNFPA), Lima, Peru; Center for Translation Research and Implementation Science (G A Mensah MD), National Institutes of Health, Bethesda, MD, USA; Department of Medicine (G A Mensah MD), School of Public Health and Family Medicine (C A Nnaji MPH), Division of Cardiology (Prof M Ntsekhe PhD), Department of Paediatrics and Child Health (Prof H J Zar PhD), University of Cape Town, Cape Town, South Africa; International Dx Department (A A Mentis MD), BGI Genomics, Copenhagen, Denmark; Neurology Unit (A Meretoja MD), Breast Surgery Unit (T J Meretoja MD), Helsinki University Hospital, Helsinki, Finland; Department of Nursing (A M Mersha MSc), Arba Minch University, Arba Minch, Ethiopia; University Centre Varazdin (T Mestrovic PhD), University North, Varazdin, Croatia; Department of Pharmacology (Prof K D Mettananda PhD), Department of Paediatrics (Prof S Mettananda DPhil), University of Kelaniya, Ragama, Sri Lanka; Clinical Medicine Department (Prof K D Mettananda PhD), North Colombo Teadhing Hospital, Ragama, Sri Lanka; University Paediatrics Unit (Prof S Mettananda DPhil), Colombo North Teaching Hospital, Ragama, Sri Lanka; Department of Epidemiology (I Michalek PhD), National Cancer Registry (I Michalek PhD), Maria Sklodowska-Curie National Research Institute of Oncology,

Warsaw, Poland; Queensland Centre for Mental Health Research (QCMHR) (P A Miller PhD), The University of Queensland, Wacol, QLD, Australia; Pacific Institute for Research & Evaluation, Calverton, MD, USA (T R Miller PhD); Department of Medical Sciences (A Mirijello MD), IRCCS Home for the Relief of Suffering General Hospital (IRCCS Casa Sollievo della Sofferenza General Hospital), San Giovanni Rotondo, Italy; Internal Medicine Programme (Prof E M Mirrakhimov PhD), Kyrgyz State Medical Academy, Bishkek, Kyrgyzstan; Department of Atherosclerosis and Coronary Heart Disease (Prof E M Mirrakhimov PhD), National Center of Cardiology and Internal Disease, Bishkek, Kyrgyzstan; Office of the Minister (M K Mirutse MPH), Federal Ministry of Health, Addis Ababa, Ethiopia; National Data Management Center for Health (A Misganaw PhD), Ethiopian Public Health Institute, Addis Ababa, Ethiopia; Division of Cardiology (A K Mishra MD), St. Vincent College, Worcester, MA, USA; Department of Forensic Medicine and Toxicology (C Mittal MD), Dr. B. C. Roy Multi-Specialty Medical Research Centre, Kharagpur, India; Institute of Addiction Research (ISFF) (B Moazen MSc), Frankfurt University of Applied Sciences, Frankfurt am Main, Germany; College of Applied and Natural Science (J Mohamed MSc), University of Hargeisa, Hargeisa, Somalia; Department of Internal Medicine (M F H Mohamed MSc), Brown University, Providence, RI, USA; Molecular Biology Unit (N S Mohamed MSc), Bio-Statistical and Molecular Biology Department (N S Mohamed MSc), Sirius Training and Research Centre, Khartoum, Sudan; Department of Public Health (H Mohammed MPH), Dire Dawa University, Dire Dawa, Ethiopia; Department of Pharmaceutical Sciences (S Mohammed PhD), Notre Dame of Maryland University, Baltimore, MD, USA; Department of Pharmacy (S Mohammed PhD), Mizan-Tepi University, Mizan, Ethiopia; Health Systems and Policy Research Unit (S Mohammed PhD), Ahmadu Bello University, Zaria, Nigeria; Institute of Clinical Physiology (S Molinaro PhD), National Research Council, Pisa, Italy; Clinical Epidemiology and Public Health Research Unit (L Monasta DSc, L Ronfani PhD), Burlo Garofolo Institute for Maternal and Child Health, Trieste, Italy; Department of Biomedical and Dental Sciences and Morphofunctional Imaging (Prof S Mondello MD), Messina University, Messina, Italy; Faculty of Medicine (A Moodi Ghalibaf MD), Infectious Diseases Research Center (F Nikoomanesh PhD), Birjand University of Medical Sciences, Birjand, Iran; Department of Epidemiology and Biostatistics (Y Moradi PhD), Department of Microbiology (M Sedighi PhD), Kurdistan University of Medical Sciences, Sanandaj, Iran; Computer, Electrical, and Mathematical Sciences and Engineering Division (P Moraga PhD), King Abdullah University of Science and Technology, Thuwal, Saudi Arabia; International Laboratory for Air Quality and Health (Prof L Morawska PhD), School of Public Health and Social Work (M T N Tran PhD, N Wang PhD), Queensland University of Technology, Brisbane, QLD, Australia; Department of Public Health (Prof R S Moreira PhD), Oswaldo Cruz Foundation, Recife, Brazil; Department of Public Health (Prof R S Moreira PhD), Federal University of Pernambuco, Recife, Brazil; Department of Biology and Biological Engineering (J Morze PhD), Chalmers University of Technology, Gothenburg, Sweden; College of Medical Sciences (J Morze PhD), SGMK Copernicus University, Warsaw, Poland; Department of Clinical Biochemistry (A Mosapour PhD), Department of Parasitology and Entomology (L Zaki PhD), Tarbiat Modares University, Tehran, Iran; Department of Health Policy (Prof E Mossialos PhD), London School of Economics and Political Science, London, UK; Research Department (M Mrejen PhD), Instituto de Estudos para Políticas de Saúde (IEPS), São Paulo, Brazil; Unit of Pharmacotherapy, Epidemiology and Economy (S Mubarik PhD), University Medical Center Groningen (Prof M J Postma PhD), Department of Internal Medicine (P Vart PhD), University of Groningen, Groningen, Netherlands; Federal Institute for Population Research, Wiesbaden, Germany (Prof U O Mueller MD); Center for Population and Health, Wiesbaden, Germany (Prof U O Mueller MD); Department of Surgery (F Mulita PhD, G Verras MD), General University Hospital of Patras, Patras, Greece; Faculty of Medicine (F Mulita PhD), Department of

Internal Medicine (G Ntaios PhD), University of Thessaly, Larissa, Greece; Clinical Epidemiology Research Unit (E Murillo-Zamora PhD), Mexican Institute of Social Security, Villa de Alvarez, Mexico; Postgraduate in Medical Sciences (E Murillo-Zamora PhD), Universidad de Colima, Colima, Mexico; Department of Pediatrics & Pediatric Pulmonology (Prof G Mustafa MD), Institute of Mother & Child Care, Multan, Pakistan; Department of Research Methodology (S Muthu MS), Orthopaedic Research Group, Coimbatore, India; Department of Neuropsychiatry (W Myung PhD), Department of Food and Nutrition (A P Okekunle PhD), Seoul National University, Seoul, South Korea; Research and Analytics Department (A J Nagarajan MTech), Initiative for Financing Health and Human Development, Chennai, India; Department of Research and Analytics (A J Nagarajan MTech), Bioinsilico Technologies, Chennai, India; Faculty of Pharmacy (F Nainu PhD), Hasanuddin University, Makassar, Indonesia; Department of Pulmonary Medicine (S Nair MD), Government Medical College Trivandrum, Trivandrum, India; Health Action by People, Trivandrum, India (S Nair MD); Department of Medical Laboratory Analysis (H H Najmuldeen PhD), Cihan University Sulaymaniya, Sulaymaniyah, Iraq; Suraj Eye Institute, Nagpur, India (V Nangia MD); School of Pharmacy (A Naqvi PhD), University of Reading, Reading, UK; National Dental Research Institute Singapore (G G Nascimento PhD), Duke-NUS Medical School, Singapore, Singapore; Department of Nursing Education & Research (A J Nashwan MSc), Department of Geriatric and Long Term Care (B Sathian PhD), Hamad Medical Corporation, Doha, Qatar; School of Pharmacy (S O Nduaguba PhD), West Virginia University, Morgantown, WV, USA; Department of General Surgery (I Negoi PhD), Emergency University Hospital Bucharest, Bucharest, Romania; Department of Cardiology (R I Negoi PhD), Cardio-Aid, Bucharest, Romania; College of Medicine and Health Sciences (H B Netsere MS), Bahir Dar University, Gondar, Ethiopia; Department of Public Health (G Nguefack-Tsague PhD), University of Yaoundé I, Yaoundé, Cameroon; Department of Biological Sciences (J W Ngunjiri DrPH), University of Embu, Embu, Kenya; Department of Medical Engineering (D H Nguyen BS), University of South Florida, Tampa, FL, USA; Cardiovascular Research Department (H Q Nguyen MD), Methodist Hospital, Merrillville, IL, USA; International Islamic University Islamabad, Islamabad, Pakistan (R K Niazi PhD); Department of General Surgery (T K Nikolouzakis PhD), University Hospital of Heraklion, Heraklion, Crete, Greece; Laboratory of Toxicology (T K Nikolouzakis PhD), Department of Medicine (Prof A Tsatsakis DSc), University of Crete, Heraklion, Greece; Department of Biomedical and Molecular Sciences (A Nikpoor PhD), Queen's University, Kingston, ON, Canada; Center for Public Health (L A Nnyanzi PhD), Teesside University, Middlesbrough, UK; Faculty of Applied Sciences and Technology (E A Noman PhD), Universiti Tun Hussein Onn Malaysia, Johor, Malaysia; Department of Health Policy and Management (S Nomura PhD), Keio University, Tokyo, Japan; Department of Clinical Sciences (Prof B Norrving PhD), Lund University, Lund, Sweden; Department of Paediatrics (C A Nri-Ezedi MD), Nnamdi Azikiwe University, Awka, Nigeria; The Cardiac Clinic (Prof M Ntsekhe PhD), Groote Schuur Hospital, Cape Town, South Africa; Department of Public Health (D Nurrika PhD), Banten School of Health Science, South Tangerang, Indonesia; Ministry of Research, Technology and Higher Education (D Nurrika PhD), Higher Education Service Institutions (LL-DIKTI) Region IV, Bandung, Indonesia; Center of Excellence in Reproductive Health Innovation (CERHI) (C I Nzoputam MPH), University of Benin, Benin City, Nigeria; Department of Physiology (O J Nzoputam PhD), University of Benin, Edo, Nigeria; Department of Physiology (O J Nzoputam PhD), Benson Idahosa University, Benin City, Nigeria; Department of Applied Economics and Quantitative Analysis (Prof B Oancea PhD), University of Bucharest, Bucharest, Romania; Department of Medicine (M J O'Donnell PhD), National University of Ireland - Galway, Galway, Ireland; Independent Consultant, Sydney, NSW, Australia (S R Okeke PhD); School of Pharmacy (O C Okonji MSc), University of the Western Cape, Cape Town, South Africa; Department of Psychiatry (A T Olagunju MD), University of

Lagos, Lagos, Nigeria; Department of Nursing Science (M I Olatubi PhD), Bowen University, Iwo, Nigeria; Cardiology Department (G M M Oliveira PhD), Federal University of Rio de Janeiro, Rio de Janeiro, Brazil; Slum and Rural Health Initiative Research Academy (I I Olufadewa MHS), Slum and Rural Health Initiative, Ibadan, Nigeria; Centre for Healthy Start Initiative, Lagos, Nigeria (B O Olusanya PhD, J O Olusanya MBA); Department of Pharmacology and Toxicology (Prof H A Omar PhD), Beni-Suef University, Beni-Suef, Egypt; Surgery Department (G L Omer MD), Sulaimani University, Sulaimani, Iraq; ENT Department (G L Omer MD), Tor Vergata University of Rome, Rome, Italy; Department of Anatomic Pathology (A E E Omonisi FWACP), Ekiti State University, Ado-Ekiti, Nigeria; Department of Anatomic Pathology (A E E Omonisi FWACP), Ekiti State University Teaching Hospital, Ado Ekiti, Nigeria; Faculty of Psychology (S Onie PhD), Universitas Airlangga, Surabaya, Indonesia; Department of Pharmacotherapy and Pharmaceutical Care (M Ordak PhD), Department of Biochemistry and Pharmacogenomics (M Zielińska MPharm), Medical University of Warsaw, Warsaw, Poland; Sickle Cell Unit (V N Orish PhD), Ho Teaching Hospital, Ho Municipality, Ghana; Department of Medicine (Prof A Ortiz MD), Hospital Universitario de La Princesa (Princess University Hospital) (Prof J B Soriano MD), Universidad Autónoma de Madrid (Autonomous University of Madrid), Madrid, Spain; Department of Nephrology and Hypertension (Prof A Ortiz MD), The Institute for Health Research Foundation Jiménez Díaz University Hospital, Madrid, Spain; Department of Infectious Diseases (E Ortiz-Brizuela MSc), Instituto Nacional de Nutrición Salvador Zubirán (Salvador Zubiran National Institute of Medical Sciences and Nutrition), Mexico City, Mexico; Department of Epidemiology, Biostatistics and Occupational Health (E Ortiz-Brizuela MSc), McGill University, Montreal, QC, Canada; Department of Biology (W M S Osman PhD), Khalifa University, Abu Dhabi, United Arab Emirates; School of Medicine (U L Osuagwu PhD, Prof A M N Renzaho PhD), Translational Health Research Institute (K Rana PhD, Prof A M N Renzaho PhD), Western Sydney University, Campbelltown, NSW, Australia; Department of Optometry and Vision Science (U L Osuagwu PhD), University of KwaZulu-Natal, KwaZulu-Natal, South Africa; Laboratory of Public Health Indicators Analysis and Health Digitalization (N Otstavnov BA, S S Otstavnov PhD), Moscow Institute of Physics and Technology, Dolgoprudny, Russia; Department of Project Management (S S Otstavnov PhD), Department of Health Care Administration and Economics (Prof V Vlassov MD), National Research University Higher School of Economics, Moscow, Russia; Faculty of Medicine (Prof A Ouyahia PhD), University Ferhat Abbas of Setif, Setif, Algeria; Division of Infectious Diseases (Prof A Ouyahia PhD), University Hospital of Setif, Setif, Algeria; Department of General Surgery (G Ouyang MD), Central South University, ChangSha, China; Department of Respiratory Medicine (Prof M P P A DNB), Department of Oral and Maxillofacial Surgery (C S N PhD), Jagadguru Sri Shivarathreeswara University, Mysore, India; Department of Forensic Medicine and Toxicology (J Padubidri MD), Kasturba Medical College, Mangalore, Mangalore, India; Research Institute for Medicines (C Palladino PhD, Prof J Perdigão PhD), Universidade de Lisboa (University of Lisbon), Lisbon, Portugal; Division of Research and Development (S R Pandi-Perumal MSc), Lovely Professional University, Phagwara, India; National Research and Innovation Agency Republic of Indonesia (BRIN), Jakarta, Indonesia (H U Pangaribuan MSc); Department of Ophthalmology (G D Panos PhD), Nottingham University Hospitals Queen's Medical Centre Campus, Nottingham, UK; Division of Ophthalmology & Visual Sciences (G D Panos PhD), University of Nottingham, Nottingham, UK; Vision and Eye Research Institute (Prof S Pardhan PhD), Anglia Ruskin University, Cambridge, UK; Department of Medical Sciences (R Passera PhD), University of Torino, Torino, Italy; Department of Imaging (R Passera PhD), AOU Città della Salute e della Scienza di Torino, Torino, Italy; School of Dentistry (J Patel BSc), University of Leeds, Leeds, UK; Department of Poverty, Gender and Youth (S K Patel PhD), Population Council, New Delhi, India; College of Dental Medicine

(Prof S Patil PhD), Roseman University of Health Sciences, South Jordan, UT, USA; Centre of Molecular Medicine and Diagnostics (COMManD) (Prof S Patil PhD), Saveetha University, Chennai, India; Second Department of Internal Medicine (D Patoulias PhD), European Interbalkan Medical Center, Thessaloniki, Greece; Department of Internal Medicine (V Patthipati MD), Advent Health, Palm Coast, FL, USA; Department of Hospital Medicine (V Patthipati MD), Sound Physicians, Palm Coast, FL, USA; Clinical Research Department (P Pedersini MSc), IRCCS Fondazione Don Carlo Gnocchi, Milan, Italy; Outpatient Department (M Peng MPH), Taihe Hospital, Shiyan, China; Department of Neurology (U Pensato MD), IRCCS Humanitas Research Hospital, Milan, Italy; Mario Negri Institute for Pharmacological Research, Bergamo, Italy (N Perico MD, Prof G Remuzzi MD); Facultad de Medicina (F E Petermann-Rocha PhD), Universidad Diego Portales (Diego Portales University), Santiago, Chile; School of Cardiovascular and Metabolic Health (F E Petermann-Rocha PhD), University of Glasgow, Glasgow, UK; School of Medicine (W A Petri MD), University of Virginia, Charlottesville, VA, USA; School of Medicine (H Pham MD), Pham Ngoc Thach University of Medicine, Ho Chi Minh City, Vietnam; Shanghai Mental Health Center (Prof M R Phillips MD), Shanghai Jiao Tong University, Shanghai, China; Department of Psychiatry (Prof M R Phillips MD), Department of Neurology (Prof N Scarmeas PhD), Department of Health and Behavior Studies (Prof I D Sigfusdottir PhD), Columbia University, New York, NY, USA; Department of Pediatric Orthopedic Surgery (M Pigeolet MD), Hôpital Necker - Enfants Malades, Paris, France; Basic Medical Sciences Department (J D Pillay PhD), Durban University of Technology, Durban, South Africa; International Center of Medical Sciences Research, Islamabad, Pakistan (Z Z Piracha PhD); Department of Environmental Hygiene (D Plass DrPH), German Environment Agency, Dessau-Roßlau, Germany; Research School of Chemistry and Applied Biomedical Sciences (E Plotnikov PhD), Tomsk Polytechnic University, Tomsk, Russia; Mental Health Research Institute (E Plotnikov PhD), Tomsk National Research Medical Center of the Russian Academy of Sciences, Tomsk, Russia; Clinical Academic Department of Pediatrics (Prof D Poddighe PhD), University Medical Center (UMC), Astana, Kazakhstan; Center of Excellence in Higher Education for Pharmaceutical Care Innovation (Prof M J Postma PhD), Universitas Padjadjaran (Padjadjaran University), Bandung, Indonesia; Non-communicable Diseases Research Center (N Pourtaheri PhD), Bam University of Medical Sciences, Bam, Iran; Centro de Investigaciones Clinicas (Clinical Research Center) (S I Prada PhD), Fundación Valle del Lili (Valle del Lili Foundation), Cali, Colombia; Askok & Rita Patel Institute of Physiotherapy (V Prakash PhD), Charotar University of Science and Technology, Anand, India; Department of Clinical Research and Epidemiology (M Prasad MD), Institute of Liver and Biliary Sciences, New Delhi, New Delhi, India; Department of Biostatistics Epidemiology and Informatics (J Puvvula PhD), University of Pennsylvania, Philadelphia, PA, USA; Cihan University Sulaimaniya Research Center (N H Qasim PhD), Cihan University Sulaimaniya, Sulaimaniya, Iraq; Department of Biological Sciences (A S Qazi PhD), National University of Medical Sciences (NUMS), Rawalpindi, Pakistan; Department of Cardiology (G Qian MS), Third Military Medical University, Chongqing, China; Cardiovascular Research Center (M Rabiee Rad MD), Isfahan Cariovascular Research Institute, Isfahan, Iran; Department of Medical Oncology (Prof V Radhakrishnan MD), Cancer Institute (W.I.A), Chennai, India; UO Neurologia, Salute Pubblica e Disabilità (The Neurology, Public Health and Disability Unit) (A Raggi PhD), Fondazione IRCCS Istituto Neurologico Carlo Besta (IRCCS Foundation Carlo Besta Neurological Institute), Milan, Italy; Department of Community Medicine and Family Medicine (Prof P R Raghav MD), Department of Pharmacology (M Shamim MBBS), Department of Urology and Kidney Transplant (M Singh MCh Urology), All India Institute of Medical Sciences, Jodhpur, India; Department of Health Policy & Organization (M Rahim MA), Department of Health Services Administration (M Rahim MA), Department of Psychology (D C Schwebel PhD), School of Medicine (Prof

J A Singh MD), University of Alabama at Birmingham, Birmingham, AL, USA; Institute of Health and Wellbeing (M Rahman PhD), Federation University Australia, Berwick, VIC, Australia; Department of Nutrition Science (S Rahmawaty PhD), Muhammadiyah University of Surakarta, Surakarta, Indonesia; Department of Community Medicine (S Rajaa MD), Employees' State Insurance Model Hospital, Chennai, India; Department of Community Medicine (P Ramasubramani MD), Mahatma Gandhi Medical College and Research Institute, Puducherry, India; Research Department (C L Ranabhat PhD), Science, Technology, and Natural Resources Department (S Tandukar PhD), Policy Research Institute, Kathmandu, Nepal; Health and Public Policy Department (C L Ranabhat PhD), Global Center for Research and Development, Kathmandu, Nepal; Centre for Clinical Pharmacology (N Rancic PhD), University of Defence in Belgrade, Belgrade, Serbia; Centre for Clinical Pharmacology (N Rancic PhD), Medical College of Georgia at Augusta University, Belgrade, Serbia; Health Economics and Outcomes Research Department (A Rane MS), Agios Pharmaceuticals, Cambridge, MA, USA; Department of Pharmaceutical Economics and Policy (A Rane MS), Massachusetts College of Pharmacy and Health Sciences, Boston, MA, USA; Department of Oral Medicine and Radiology, Forensic Odontology (K Rao PhD), Nitte University, Mangalore, India; Department of Oral Pathology (S Rao MDS), Sharavathi Dental College and Hospital, Shimogga, India; Inovus Medical, St Helens, UK (D L Rawaf MRCS); Academic Public Health England (Prof S Rawaf MD), Public Health England, London, UK; Department of Biological Sciences (Prof E M M Redwan PhD), King Abdulaziz University, Jeddah, Egypt; Department of Protein Research (Prof E M M Redwan PhD), Research and Academic Institution, Alexandria, Egypt; Unisabana Center for Translational Science (L F Reyes PhD), Universidad de La Sabana (Savannah University), Chia, Colombia; Critical Care Department (L F Reyes PhD), Clinica Universidad De La Sabana (Savannah University Clinic), Chia, Colombia; Network of Immunity in Infection, Malignancy and Autoimmunity (NIIMA) (Prof N Rezaei PhD), Universal Scientific Education and Research Network (USERN), Tehran, Iran; Department of Epidemiology and Biostatistics (Prof M Rezaeian PhD), Rafsanjan University of Medical Sciences, Rafsanjan, Iran; Department of Surgery (J Rickard MD), University Teaching Hospital of Kigali, Kigali, Rwanda; 1H-TOXRUN - One Health Toxicology Research Unit (Prof C F Rodrigues PhD), Instituto Universitário de Ciências da Saúde (CESPU), Paredes, Portugal; Departamento de Farmacologia y toxicologia (Department of Pharmacology and Toxicology) (Prof J A B Rodriguez PhD), Universidad de Antioquia (University of Antioquia), Medellin, Colombia; Department of Clinical Research (L Roever PhD), Federal University of Uberlândia, Uberlândia, Brazil; Gilbert and Rose-Marie Chagoury School of Medicine (L Roever PhD), Lebanese American University, Beirut, Lebanon; Golestan Research Center of Gastroenterology and Hepatology (G Roshandel PhD), Golestan University of Medical Sciences, Gorgan, Iran; Technical Department (K Rotimi MSc), Malaria Consortium, Abuja, Nigeria; Department of Public Health Pharmacy (K Rotimi MSc), West African Postgraduate College of Pharmacists, Lagos, Nigeria; Department of Analytical and Applied Economics (Prof H S Rout PhD, C K Swain MPhil), UGC Centre of Advanced Study in Psychology (M Satpathy PhD), Utkal University, Bhubaneswar, India; Faculty of Medicine (B Roy PhD), Quest International University Perak, Ipoh, Malaysia; Department of Biochemistry and Food Analysis (N Roy PhD), Patuakhali Science and Technology University, Patuakhali, Bangladesh; Department of Labour (P Roy PhD), Directorate of Factories, Government of West Bengal, Kolkata, India; Centro de Investigación Palmira (Palmira Research Center) (E Rubagotti PhD), Corporación Colombiana de Investigación Agropecuaria AGROSAVIA (Colombian Agricultural Research Corporation), Bogota, Colombia; Department of Medical Pharmacology (M M Saber-Ayad MD), Public Health and Community Medicine Department (M R Salem MD), Cairo University, Giza, Egypt; Neuropsychiatric Institute (Prof P S Sachdev MD), Prince of Wales Hospital, Randwick, NSW, Australia; Department of Pharmaceutical

Chemistry (Prof M R Saeb PhD), Medical University of Gdańsk, Gdańsk, Poland; Multidisciplinary Laboratory Foundation University School of Health Sciences (FUSH) (Prof U Saeed PhD), Foundation University, Islamabad, Pakistan; International Center of Medical Sciences Research (ICMSR), Islamabad, Pakistan (Prof U Saeed PhD); Faculty of Medicine, Bioscience and Nursing (S Z Safi PhD), MAHSA University, Selangor, Malaysia; Interdisciplinary Research Centre in Biomedical Materials (IRCBM) (S Z Safi PhD), COMSATS Institute of Information Technology, Lahore, Pakistan; Department of Statistics (M R Sajid PhD), University of Gujrat, Gujrat, Pakistan; Institute for Employment Research, Nuremberg, Germany (J W Sakshaug PhD); Technology Management Department (Prof M Z Y Salem PhD), University College of Applied Sciences, Gaza, Palestine; School of Economics and Management (Prof M Z Y Salem PhD), University of Kassel, Kassel, Germany; Department of Pathology, Microbiology and Forensic Medicine (M Sallam PhD), Department of Clinical Laboratories and Forensic Medicine (M Sallam PhD), Independent Consultant, Amman, Jordan; Pharmacy Study Program (M A Sarasmita PharmD), Udayana University, Badung, Indonesia; Department of Public Health (Y Sarikhani PhD), Jahrom University of Medical Sciences, Jahrom, Iran; Department of Health and Society (Prof R Sarmiento-Suárez MPH), University of Applied and Environmental Sciences, Bogota, Colombia; National School of Public Health (Prof R Sarmiento-Suárez MPH), Carlos III Health Institute, Madrid, Spain; Department of Oral Pathology and Microbiology (Prof G S Sarode PhD, Prof S C Sarode PhD), Dr. D. Y. Patil University, Pune, India; Faculty of Health & Social Sciences (B Sathian PhD), Bournemouth University, Bournemouth, UK; Department of Medicine (A Sathyanarayan MD, M Sharath MBBS), Bangalore Medical College and Research Institute, Bangalore, India; Udyam-Global Association for Sustainable Development, Bhubaneswar, India (M Satpathy PhD); Department of Public Health Sciences (M Sawhney PhD), University of North Carolina at Charlotte, Charlotte, NC, USA; Department of Neurology (Prof N Scarmeas PhD), National and Kapodistrian University of Athens, Athens, Greece; Department of Health Sciences (I J C Schneider PhD), Federal University of Santa Catarina, Araranguá, Brazil; Clinic for Conservative Dentistry and Periodontology (Prof F Schwendicke PhD), University Hospital of the Ludwig-Maximilians-University Munich, Munich, Germany; Emergency Department (S Senthilkumaran MD), Manian Medical Centre, Erode, India; Department of Medicine and Surgery (Y Sethi MBBS), Government Doon Medical College, Dehradun, India; Department of Medicine (Prof S Setoguchi MD), Rutgers University, New Brunswick, NJ, USA; National Heart, Lung, and Blood Institute (A Seylani BS), National Institute of Health, Rockville, MD, USA; Department of Medicine (N S Shah MD), Northwestern University, Chicago, IL, USA; Department of Infectious Diseases and Microbiology (P A Shah MBBS), Rajiv Gandhi University of Health Sciences, Bangalore, India; HepatoPancreatoBiliary Surgery and Liver Transplant Department (P A Shah MBBS), Healthcare Global Limited Cancer Care Hospital, Bangalore, India; Independent Consultant, Karachi, Pakistan (M A Shaikh MD); Department of Pathology and Laboratory Medicine (S Sham MD), Northwell Health, New York, NY, USA; Department of Pathobiology (M Shamshirgaran PhD), Shahid Bahonar University of Kerman, Kerman, Iran; Facultad de Medicina (Faculty of Medicine) (J Sharifi-Rad PhD), Universidad del Azuay (University of Azuay), Cuenca, Ecuador; University School of Management and Entrepreneurship (R Sharma PhD), Delhi Technological University, Delhi, India; Department of Physiotherapy (S Sharma PhD), Kathmandu University, Dhulikhel, Nepal; Division of Microbiology and Biotechnology (R P Shastry PhD), Yenepoya Research Center, Mangalore, India; Department of Engineering (A Shavandi PhD), Free University of Brussels, Brussels, Belgium; Department of Bioengineering (A Shayan BS), Clemson University, Clemson, SC, USA; Department of Ophthalmology (M Shayan MD), Harvard Medical School, Boston, MA, USA; Tokyo Foundation for Policy Research, Tokyo, Japan (Prof K Shibuya MD); Department of Psychiatry (J E Shifa MSc), Madda Walabu

University, Shashemene, Ethiopia; Department of Mental Health (J E Shifa MSc), Ethiopian Mental Health Association, Addis Ababa, Ethiopia; Department of Public Health (D Shiferaw MPH), Dambi Dollo University, Dembi Dollo, Ethiopia; Department of Nursing (W S Shiferaw MSc), Department of Pediatrics and Child Health Nursing (S S Yehualashet MSc), Debre Berhan University, Debre Berhan, Ethiopia; National Institute of Infectious Diseases, Tokyo, Japan (M Shigematsu PhD); Finnish Institute of Occupational Health, Helsinki, Finland (R Shiri PhD); Department of Veterinary Public Health and Preventive Medicine (A Shittu MSc), Usmanu Danfodiyo University, Sokoto, Sokoto, Nigeria; Department of Clinical Immunology and Hematology (V Shivarov PhD), Sofiamed University Hospital, Sofia, Bulgaria; Department of Genetics (V Shivarov PhD), Sofia University "St. Kliment Ohridiski", Sofia, Bulgaria; School of Pharmacy (S Shrestha PharmD), Monash University, Selangor Darul Ehsan, Malaysia; The Cooper Institute, Dallas, TX, USA (K Shuval PhD); Department of Pediatrics and Child Health Nursing (M M Sibhat MSc), Dilla University, Dilla, Ethiopia; Unit of Basic Medical Sciences (E E Siddig MD), University of Khartoum, Khartoum, Sudan; Department of Medical Microbiology and Infectious Diseases (E E Siddig MD), Erasmus University, Rotterdam, Netherlands; Department of Psychology (Prof I D Sigfusdottir PhD), Reykjavik University, Reykjavik, Iceland; Center of Potential and Innovation of Natural Resources (Prof L M R Silva PhD), Polytechnic Institute of Guarda, Guarda, Portugal; Health Sciences Research Centre (Prof L M R Silva PhD), University of Beira Interior, Covilhã, Portugal; Faculty of Pharmacy (S Silva MSc), Coimbra Institute for Biomedical Imaging and Translational Research (S Silva MSc), University of Coimbra, Coimbra, Portugal; School of Health (Prof C R Simpson PhD), Victoria University of Wellington, Wellington, New Zealand; Department of Anatomy (A Singal PhD), Department of Radiodiagnosis (P Singh MD), All India Institute of Medical Sciences, Bathinda, India; Department of Dentistry (A Singh MD), All India Institute of Medical Sciences, Bhopal, India; School of Public Health & Zoonoses (B B Singh PhD), Guru Angad Dev Veterinary & Animal Sciences University, Ludhiana, India; Department of Pharmacology (H Singh DM Clinical Pharmacology), Government Medical College and Hospital, Chandigarh, India; Medicine Service (Prof J A Singh MD), US Department of Veterans Affairs (VA), Birmingham, AL, USA; Department of Physiotherapy and Occupational Therapy (Prof S T Skou PhD), Næstved-Slagelse-Ringsted Hospitals, Slagelse, Denmark; Division of Injury Prevention (Prof D A Sleet PhD), The Bizzell Group, Atlanta, GA, USA; Department of Systemic Pathology (R Solanki MD), Touro College of Osteopathic Medicine, Middletown, NY, USA; Department of Pathology (R Solanki MD), American University of the Caribbean School of Medicine, Cupecoy, Saint Martin; Department of Health Policy and Management (S Song PhD), University of Georgia College of Public Health, Athens, GA, USA; Centro de Investigación Biomédica en Red Enfermedades Respiratorias (Center for Biomedical Research in Respiratory Diseases Network) (CIBERES), Madrid, Spain (Prof J B Soriano MD); Hull York Medical School (I N Soyiri PhD), University of Hull, Hull City, UK; Division of Community Medicine (C T Sreeramareddy MD), International Medical University, Kuala Lumpur, Malaysia; Nutrition and Dietetics Department (A V Starodubova DSc), Federal Research Institute of Nutrition, Biotechnology and Food Safety, Moscow, Russia; Department of Internal Disease (A V Starodubova DSc), Pirogov Russian National Research Medical University, Moscow, Russia; Department of Medicine (P Steiropoulos MD), Democritus University of Thrace, Alexandroupolis, Greece; Occupational and Environmental Medicine Department (L Stockfelt PhD), Institute of Health and Care Sciences (Prof A W Wolf PhD), University of Gothenburg, Gothenburg, Sweden; School of Exercise and Nutrition Sciences (N S Subedi MPH), Deakin University, Melbourne, VIC, Australia; School of Medicine (V Subramaniyan PhD), Monash University, Sunway, Malaysia; Department of Geriatrics (C K Suemoto MD), University of São Paulo, Sao Paulo, Brazil; Center for Biotechnology and Microbiology (M Suleman PhD), University of Swat, Charbagh,

Pakistan; School of Life Sciences (M Suleman PhD), Xiamen University, China, Xiamen, China; National Institute of Epidemiology (R Suliankatchi Abdulkader MD), Indian Council of Medical Research, Chennai, India; Mental Health Research (A Sultana MD), Independent Consultant, Khulna, Bangladesh; Division of Global Mental Health (A Sultana MD), EviSyn Health, Khulna, Bangladesh; Cardiovascular Program (X Xu PhD), The George Institute for Global Health, Sydney, NSW, Australia (Prof J Sundström PhD); Department of Clinical Outcomes (Prof L Szarpak PhD), Maria Sklodowska-Curie Medical Academy, Warsaw, Poland; Department of Clinical Research and Development (Prof L Szarpak PhD), LUXMED Group, Warsaw, Poland; Department of Neurology (P Tabaee Damavandi MD), Neurocenter of Southern Switzerland (NSI), Lugano, Switzerland; Department of Medicine (Prof R Tabarés-Seisdedos PhD), University of Valencia, Valencia, Spain; Carlos III Health Institute (Prof R Tabarés-Seisdedos PhD), Biomedical Research Networking Center for Mental Health Network (CiberSAM), Madrid, Spain; Department of Basic Medical Sciences (S Tabatabaeizadeh PhD), Department of Internal Medicine (S Tabatabaeizadeh PhD), Islamic Azad University, Mashhad, Iran; School of Dentistry and Oral Health (S K Tadakamadla PhD), Griffith University, Gold Coast, QLD, Australia; Living Systems Institute (Y Taheri Abkenar PharmD), Department of Health and Community Sciences (A Udoh PhD), University of Exeter, Exeter, UK; Department of Biostatistics and Epidemiology (M Taheri Soodejani PhD), Shahid Sadoughi University of Medical Sciences, Yazd, Iran; National Centre for Epidemiology and Population Health (A Talukder MSc), Australian National University, Acton, ACT, Australia; Statistics Discipline (A Talukder MSc), Khulna University, Khulna, Bangladesh; Department of Dermato-Venereology (M Tampa PhD), Dr. Victor Babes Clinical Hospital of Infectious Diseases and Tropical Diseases, Bucharest, Romania; Department of Epidemiology (J L Tamuzi MSc), Stellenbosch University, Cape Town, South Africa; Department of Medicine (J L Tamuzi MSc), Northlands Medical Group, Omuthiya, Namibia; State Key Laboratory of Numerical Modeling for Atmospheric Sciences and Geophysical Fluid Dynamics (LASG) (H Tang PhD), Chinese Academy of Sciences, Beijing, China; Department of Urology (M Teimoori MD), Sabzevar University of Medical Sciences, Sabzevar, Iran; Department of Epidemiology and Biostatistics (M Teramoto MD), Department of Bioengineering and Therapeutic Sciences (Prof M S Zastrozhin PhD), University of California San Francisco, San Francisco, CA, USA; Department of Pharmacology (P Thangaraju MD), All India Institute of Medical Sciences, Raipur, India; Public Health Department (Prof K R Thankappan MD), Amrita Institute of Medical Sciences, Kochi, India; Faculty of Medicine (R Thayakaran PhD), University of Southampton, Southampton, UK; Department of Endocrinology, Diabetes and Metabolism (Prof N Thomas PhD), Christian Medical College and Hospital (CMC), Vellore, India; Department of Gastroenterology (N K Thomas MD), PSG Institute of Medical Sciences and Research, Coimbatore, India; Department of Psychiatry (C C Thum MB), Hospital Sultan Abdul Aziz Shah Universiti Putra Malaysia, Serdang, Malaysia; Faculty of Biomedical Engineering (A Tichopad PhD), Czech Technical University, Prague, Czech Republic; Faculty of Public Health (J H V Ticoalu MPH), Universitas Sam Ratulangi, Manado, Indonesia; Nuffield Department of Primary Care Health Sciences (T Tillawi MD), Oxford University, Oxford, UK; Public Health Department (T Y Tiruye PhD), Department of Human Nutrition and Food Sciences (E G Wassie MSc), Debre Markos University, Debre Markos, Ethiopia; Office of Global Relations (Prof R Tobe-Gai PhD), Nagasaki University, Nagasaki, Japan; Department of Medicine (Prof M Tonelli MD), Department of Oncology (L Yang PhD), University of Calgary, Calgary, AB, Canada; Institute of Public Health (R Topor-Madry PhD), Jagiellonian University Medical College, Kraków, Poland; Agency for Health Technology Assessment and Tariff System, Warsaw, Poland (R Topor-Madry PhD); Nutritional Epidemiology Research Team (EREN) (M Touvier PhD), National Institute for Health and Medical Research (INSERM), Paris, France; SRM College of Pharmacy (M R Tovani-Palone

PhD), SRM Institute of Science and Technology (SRMIST), Chennai, India; School of Medicine (J T Tran BS), Indiana University, Indianapolis, IN, USA; Health Informatics Department (M T N Tran PhD), Hanoi Medical University, Ha Noi, Vietnam; Department of Health (N M Tran MD), Children's Hospital 1, Ho Chi Minh City, Vietnam; Department of Surgical, Medical, Molecular Pathology and Critical Care Medicine (D Trico MD), University of Pisa, Pisa, Italy; Adult Learning Disability Service (S J Tromans PhD), Leicestershire Partnership National Health Service Trust, Leicester, UK; School of Medicine (T T Truyen MD), Nam Can Tho University, Can Tho, Vietnam; Department of Nutrition and Food Studies (S Tyrovolas PhD), George Mason University, Fairfax, VA, USA; Medical Genomics Research Department (M Umair PhD), King Abdullah International Medical Research Center, Riyadh, Saudi Arabia; Department of Life Sciences (M Umair PhD), University of Management and Technology, Lahore, Pakistan; Department of Paraclinical Sciences (S Umakanthan MD), The University of the West Indies, St. Augustine, Trinidad and Tobago; School of Government (E A Undurraga PhD), Pontificia Universidad Catolica de Chile (Pontifical Catholic University of Chile), Santiago, Chile; Department of Cardiovascular, Endocrine-metabolic Diseases and Aging (B Unim PhD), National Institute of Health, Rome, Italy; Institute of Health and Wellbeing (Prof C A Unsworth PhD), Federation University Australia, Churchill, VIC, Australia; Amity Institute of Biotechnology (E Upadhyay PhD), Amity University Rajasthan, Jaipur, India; Center for Neurodegenerative Diseases and the Aging Brain (D Urso MD), University of Bari, Tricase, Italy; College of Health and Sport Sciences (A G Vaithinathan MSc), University of Bahrain, Salmanya, Bahrain; Department of Cardiovascular Sciences (J Van den Eynde BSc), Katholieke Universiteit Leuven, Leuven, Belgium; Department of Public Health and Epidemiology (O Varga PhD), University of Debrecen, Debrecen, Hungary; Achutha Menon Centre for Health Science Studies (R P Varma MD), Sree Chitra Tirunal Institute for Medical Sciences and Technology, Trivandrum, India; UKK Institute, Tampere, Finland (Prof T J Vasankari MD); Faculty of Medicine and Health Technology (Prof T J Vasankari MD), Tampere University, Tampere, Finland; Institute of Public Health of Serbia, Belgrade, Serbia (M Vasic PhD); Department of Human Genetics & Molecular Biology (B Vellingiri PhD), Bharathiar University, Coimbatore, India; Raffles Neuroscience Centre (Prof N Venketasubramanian MBBS), Raffles Hospital, Singapore, Singapore; Department of Physiotherapy (J H Villafañe PhD), Universidad Europea de Madridn (European University of Madrid), Villaviciosa de Odón, Spain; Occupational Health Unit (Prof F S Violante MD), Sant'Orsola Malpighi Hospital, Bologna, Italy; Department of Medical Oncology (S R Volovat PhD), University of Medicine and Pharmacy "Grigore T Popa" Iasi, Iaşi, Romania; Department of Medical Oncology (S R Volovat PhD), Regional Institute of Oncology, Iaşi, Romania; Office of Research, Innovation, and Commercialization (ORIC) (Prof Y Waheed PhD), Shaheed Zulfiqar Ali Bhutto Medical University (SZABMU), Islamabad, Pakistan; Gilbert and Rose-Marie Chagoury School of Medicine (Prof Y Waheed PhD), Lebanese American University, Byblos, Lebanon; School of Public Health (F Wang PhD), Xuzhou Medical University, Xuzhou, China; Department of Neurosurgery (S Wang MD), Capital Medical University, Beijing, China; Department of Neurosurgery (S Wang MD), Beijing Tiantan Hospital, Beijing, China; Department of Parasitology (Prof K G Weerakoon PhD), Department of Community Medicine (N D Wickramasinghe MD), Rajarata University of Sri Lanka, Anuradhapura, Sri Lanka; Cardiology Department (Prof R G Weintraub MB), Royal Children's Hospital, Melbourne, VIC, Australia; Key Laboratory of Shaanxi Province for Craniofacial Precision Medicine Research (Y Wen PhD), Stomatological Hospital (College) of Xi'an Jiaotong University, Xi'an, China; Department of Physical Therapy (T Wiangkham PhD), Naresuan University, Phitsanulok, Thailand; Department of Nursing (A Wilandika MKep), Universitas Aisyiyah Bandung, Bandung, Indonesia; Department of Medical Statistics, Informatics and Health Economics (Prof P Willeit PhD), Medical University Innsbruck, Innsbruck, Austria; Department of Public

Health and Primary Care (Prof P Willeit PhD), University of Cambridge, Cambridge, UK; Department of Neurobiology, Care Sciences and Society (Prof A Wimo PhD), Karolinska Institute, Solna, Sweden; Department of Nutrition (D H Woldegebreal MPH), University of California Davis, Davis, CA, USA; Bone and Joint Research Group (Prof A D Woolf MBBS), Royal Cornwall Hospital, Truro, UK; Global Alliance for Musculoskeletal Health, Truro, UK (Prof A D Woolf MBBS); Global Health Research Center (C Wu PhD), Duke Kunshan University, Kunshan, China; Department of Food Science and Human Nutrition (Prof F Wu PhD), Michigan State University, East Lansing, MI, USA; School of Public Health (Shenzhen) (X Wu MPH), Sun Yat-sen University, Shenzhen, China; Division of Gastroenterology (Prof Z Wu PhD), Huazhong University of Science and Technology, Wuhan, China; Vanke School of Public Health (Y Xia MD), Tsinghua University, Beijing, China; School of Public Health (H Xiao PhD), Zhejiang University, Zhejiang, China; Department of Public Health Science (H Xiao PhD), Fred Hutchinson Cancer Research Center, Seattle, WA, USA; Australian Institute of Health Innovation (L Yadav PhD), Macquarie University, Macquarie Park, NSW, Australia; Department of Basic Medical Sciences (S Yaghoubi PhD), Neyshabur University of Medical Sciences, Neyshabur, Iran; Graduate School of Medicine (Prof K Yamagishi MD), Osaka University, Suita, Japan; Department of Cancer Epidemiology and Prevention Research (L Yang PhD), Alberta Health Services, Calgary, AB, Canada; Faculty of Medicine (Y Yano MD), Department of Public Health (Prof N Yonemoto PhD), Juntendo University, Tokyo, Japan; Research Center of Physiology (H Yaribeygi PhD), Semnan University of Medical Sciences, Semnan, Iran; Department of Family Medicine (S A Yesuf MSc), St. Peter's Specialized Hospital, Addis Ababa, Ethiopia; Independent Consultant, Addis Ababa, Ethiopia (S A Yesuf MSc); Biostatics, Epidemiology, and Science Computing Department (S Yezli PhD), King Faisal Specialist Hospital & Research Center, Riyadh, Saudi Arabia; Department of Health Management (A Yiğit PhD, V Yiğit PhD), Süleyman Demirel Üniversitesi (Süleyman Demirel University), Isparta, Turkiye; Pharmacy Department (Y Yismaw MSc), Alkan Health Science, Business and Technology College, Bahir Dar, Ethiopia; Department of Pediatrics (Prof D Yon MD), Kyung Hee University, Seoul, South Korea; Department of Biostatistics (Prof N Yonemoto PhD), University of Toyama School of Medicine, Toyama, Japan; Department of Health Policy and Management (Prof M Z Younis PhD), Jackson State University, Jackson, MS, USA; School of Business & Economics (Prof M Z Younis PhD), Universiti Putra Malaysia (University of Putra Malaysia), Kuala Lumpur, Malaysia; Department of Epidemiology and Biostatistics (Prof C Yu PhD), Wuhan University, Wuhan, China; Faculty of Medicine and Health Sciences (F Zakham PhD), Hodeidah University, Hodeidah, Yemen; Department of Pharmacology (B A Zaman MSc), University of Duhok, Duhok, Iraq; Hospital San Juan de Dios, Tarija, Bolivia (N Zamora MD); Department of Neuroscience (R Zand MD), Geisinger Health System, Danville, PA, USA; Department of Neurology (R Zand MD), University of Tennessee, Memphis, TN, USA; Unit on Child & Adolescent Health (Prof H J Zar PhD), Medical Research Council South Africa, Cape Town, South Africa; Addictology Department (Prof M S Zastrozhin PhD), Russian Medical Academy of Continuous Professional Education, Moscow, Russia; Department of Obstetrics and Gynecology (N Zhang BS), Frist Affiliated Hospital of Anhui Medical University, Hefei, China; School of Public Health (Y Zhang PhD), Hubei Province Key Laboratory of Occupational Hazard Identification and Control (Y Zhang PhD), Wuhan University of Science and Technology, Wuhan, China; College of Traditional Chinese Medicine (H Zhao MD), Hebei University, Baoding, China; School of Population Medicine and Public Health (P Zhong MSc), Peking Union Medical College, Beijing, China; Computational Bioscience Research Center (J Zhou PhD), King Abdullah University of Science and Technology, Jeddah, Saudi Arabia; Department of Nursing (M Zoladl PhD), Yasuj University of Medical Sciences, Yasuj, Iran; NIHR-Biomedical Research Centre (NIHR-BRC)

(Prof A Zumla PhD), University College London Hospitals, London, UK; School of Physics (S H Zyoud PhD), Universiti Sains Malaysia (University of Science Malaysia), Penang, Malaysia

Authors' Contributions

Managing the overall research enterprise

Simon I Hay, Ali H Mokdad, Christopher J L Murray, and Theo Vos.

Writing the first draft of the manuscript

Alize J Ferrari, Samuel M Ostroff, and Damian Francesco Santomauro.

Primary responsibility for applying analytical methods to produce estimates

Daniel T Araki, Michael Benjamin Arndt, Charlie Ashbaugh, Michael Brauer, Katrin Burkart, Sinclair Carr, Angela W Chen, Eunice Chung, Xiaochen Dai, Kara Estep, Lisa M Force, William M Gardner, Peter W Gething, Erin B Hamilton, Claire A Henson, Julia Hon, Kevin S Ikuta, Jonathan M Kocarnik, Hmwe Hmwe Kyu, Kate E LeGrand, Megan Lindstrom, Ana M Mantilla Herrera, Tomislav Mestrovic, Paul Anthony Miller, Madeline E Moberg, Robin M Mohr, Vincent Mougin, Olivia D Nesbit, Maja Pasovic, Spencer A Pease, David M Pigott, Natalie Pritchett, Christian Razo, Austin E Schumacher, Jamileh Shadid, Erica Leigh N Slepak, Reed J D Sorensen, Benjamin A Stark, Jaimie D Steinmetz, Theo Vos, Daniel J Weiss, Joanna L Whisnant, Harvey A Whiteford, Caroline Wilkerson, Demewoz H Woldegebreal, Sarah Wulf Hanson, Yvonne Yiru Xu, and Stephanie R M Zimsen.

Primary responsibility for seeking, cataloguing, extracting, or cleaning data; designing or coding figures and tables

Justin Byun, Alize J Ferrari, Johnathan M Hsu, Vincent C Iannucci, Audrey L Ihler, and Damian Francesco Santomauro.

Providing data or critical feedback on data sources

Yohannes Habtegiorgis Abate, Cristiana Abbafati, Hedayat Abbastabar, Auwal Abdullahi, Roberto Ariel Abeldaño Zuñiga, Richard Gyan Aboagye, Hassan Abolhassani, Lucas Guimarães Abreu, Niveen ME Abu-Rmeileh, Akindele Olupelumi Adebiyi, Abiola Victor Adepoju, Habeeb Omoponle Adewuyi, Saira Afzal, Antonella Agodi, Ali Ahmed, Muktar Beshir Ahmed, Mohammed Albashtawy, Robert W Aldridge, Kefyalew Addis Alene, Syed Shujait Shujait Ali, Syed Mohamed Aljunid, Nelson Alvis-Guzman, Nelson J Alvis-Zakzuk, Mohammad Sami Al-Wardat, Azmeraw T Amare, Edward Kwabena Ameyaw, Deanna Anderlini, Pedro Prata Andrade, Hossein Ansari, Saleha Anwar, Sumadi Lukman Anwar, Jalal Arabloo, Mosab Arafat, Benedetta Armocida, Michael Benjamin Arndt, Anton A Artamonov, Marvellous O Asika, Seyyed Shamsadin Athari, Prince Atorkey, Alok Atreya, Beatriz Paulina Ayala Quintanilla, Jose L Ayuso-Mateos, Ashish D Badiye, Abdulaziz T Bako, Senthilkumar Balakrishnan, Palash Chandra Banik, Martina Barchitta, Mainak Bardhan, Hiba Jawdat Barqawi, Amadou Barrow, Sandra Barteit, Sanjay Basu, Nebiyou Simegnew Bayileyegn, Michelle L Bell, Olorunjuwon Omolaja Bello, Apostolos Beloukas, Eduardo Bernabe, Robert S Bernstein, Akshaya Srikanth Bhagavathula, Dinesh Bhandari, Sonu Bhaskar, Vivek Bhat, Gurjit Kaur Bhatti, Jasvinder Singh Bhatti, Zulfiqar A Bhutta, Boris Bikbov, Virginia Bodolica, Aadam Olalekan Bodunrin, Milad Bonakdar Hashemi, Berrak Bora Basara, Hamed Borhany, Christopher Boxe, Oliver J Brady, Nicola Luigi Bragazzi, Dejana Braithwaite, Luisa C Brant, Traolach Brugha, Danilo Buonsenso, Katrin Burkart, Justin Byun, Florentino Luciano Caetano dos Santos, Chao Cao, Joao Mauricio

Castaldelli-Maia, Carlos A Castañeda-Orjuela, Francieli Cembranel, Rama Mohan Chandika, Vijay Kumar Chattu, Angela W Chen, Catherine S Chen, William C S Cho, Sungchul Choi, Bryan Chong, Rajiv Chowdhury, Dinh-Toi Chu, Eric Chung, Eunice Chung, Alyssa Columbus, Joao Conde, Paolo Angelo Cortesi, Ewerton Cousin, Michael H Criqui, Natália Cruz-Martins, Siyu Dai, Xiaochen Dai, Giovanni Damiani, Saswati Das, Claudio Alberto Dávila-Cervantes, Aklilu Tamire Debele, Nicole K DeCleene, Farah Deeba, Louisa Degenhardt, Andreas K Demetriades, Nikolaos Dervenis, Hardik Dineshbhai Desai, Rupak Desai, Samath Dhamminda Dharmaratne, Sameer Dhingra, Diana Dias da Silva, Daniel Diaz, Michael J Diaz, M Ashworth Dirac, Thao Huynh Phuong Do, Regina-Mae Villanueva Dominguez, Leila Doshmangir, Robert Kokou Dowou, Tim Robert Driscoll, Haneil Larson Dsouza, Bruce B Duncan, Andre Rodrigues Duraes, Senbagam Duraisamy, Paulina Agnieszka Dzianach, Ebrahim Eini, Michael Ekholuenetale, Temitope Cyrus Ekundayo, Iman El Sayed, Ryenchindorj Erkhembayar, Adeniyi Francis Fagbamigbe, Mohammad Fareed, Carla Sofia e Sá Farinha, Andre Faro, Ali Fatehizadeh, Seyed-Mohammad Fereshtehnejad, Alize J Ferrari, David Flood, Lisa M Force, Takeshi Fukumoto, João M Furtado, Peter Andras Gaal, Muktar A Gadanya, Santosh Gaihre, Yaseen Galali, Mandukhai Ganbat, Balasankar Ganesan, Mohd Ashraf Ganie, William M Gardner, Tilaye Gebru Gebi, Tesfay B B Gebremariam, Teferi Gebru Gebremeskel, Simona Roxana Georgescu, Peter W Gething, Elena Ghotbi, Alem Girmay, Laszlo Göbölös, Myron Anthony Godinho, Mahaveer Golechha, Pouya Goleij, Alessandra C Goulart, Ayman Grada, Shi-Yang Guan, Avirup Guha, Ishita Gupta, Rajeev Gupta, Vijai Kumar Gupta, Nils Haep, Nima Hafezi-Nejad, Abdul Hafiz, Rabih Halwani, Erin B Hamilton, Harapan Harapan, Josep Maria Haro, Johannes Haubold, Rasmus J Havmoeller, Simon I Hay, Jeffrey J Hebert, Claire A Henson, Nguyen Quoc Hoan, Julia Hon, Md Mahbub Hossain, Mehdi Hosseinzadeh, Javid Hussain, Nawfal R Hussein, Hong-Han Huynh, Olayinka Stephen Ilesanmi, Lalu Muhammad Irham, Sheikh Mohammed Shariful Islam, Nahlah Elkudssiah Ismail, Gaetano Isola, Mahalaxmi Iyer, Jalil Jaafari, Kathryn H Jacobsen, Morteza Jafarinia, Khushleen Jaggi, Nader Jahanmehr, Haitham Jahrami, Nityanand Jain, Mihajlo Jakovljevic, Tahereh Javaheri, Sathish Kumar Jayapal, Shubha Jayaram, Jost B Jonas, Tamas Joo, Abel Joseph, Charity Ehimwenma Joshua, Jacek Jerzy Jozwiak, Mikk Jürisson, Billingsley Kaambwa, Zubair Kabir, Vidya Kadashetti, Leila R Kalankesh, Sanjay Kalra, Naser Kamyari, Himal Kandel, Rami S Kantar, Norito Kawakami, Gbenga A Kayode, Cathleen Keller, John H Kempen, Emmanuelle Kesse-Guyot, Himanshu Khajuria, Nauman Khalid, Faham Khamesipour, Ikramullah Khan, Maseer Khan, Moien AB Khan, Khaled Khatab, Amir M Khater, Feriha Fatima Khidri, Min Seo Kim, Adnan Kisa, Sezer Kisa, Ann Kristin Skrindo Knudsen, Jonathan M Kocarnik, Gerbrand Koren, Oleksii Korzh, Soewarta Kosen, Sindhura Lakshmi Koulmane Laxminarayana, Kewal Krishan, Vijay Krishnamoorthy, Barthelemy Kuate Defo, Manasi Kumar, Dian Kusuma, Hmwe Hmwe Kyu, Muhammad Awwal Ladan, Chandrakant Lahariya, Dharmesh Kumar Lal, Anders O Larsson, Savita Lasrado, Huu-Hoai Le, Long Khanh Dao Le, Nhi Huu Hanh Le, Trang Diep Thanh Le, Janet L Leasher, Caterina Ledda, Munjae Lee, Sang-woong Lee, Seung Won Lee, Yo Han Lee, Kate E LeGrand, Janni Leung, Yichong Li, Lee-Ling Lim, Stephen S Lim, Megan Lindstrom, Gang Liu, Runben Liu, Shiwei Liu, Wei Liu, Xuefeng Liu, Erand Llanaj, Rubén López-Bueno, László Lorenzovici, Rafael Lozano, Jay B Lusk, Zheng Feei Ma, Azzam A Maghazachi, Azeem Majeed, Kashish Malhotra, Iram Malik, Deborah Carvalho Malta, Mohammad Ali Mansournia, Ana M Mantilla Herrera, Lorenzo Giovanni Mantovani, Parham Mardi, Daniela Martini, Francisco Rogerlândio Martins-Melo, Sharmeen Maryam, Roy Rillera Marzo, Alexander G Mathioudakis, Jishanth Mattumpuram, Mohsen Mazidi, Anna Laura W McKowen, Michael A McPhail, Entezar Mehrabi Nasab, Tesfahun Mekene Meto, Walter Mendoza, Ritesh G Menezes, Haftu Asmerom Meresa, Atte Meretoja, Sachith Mettananda, Irmina Maria Michalek, Paul Anthony Miller, Ted R Miller, Edward J Mills, Le Huu Nhat Minh, Erkin M Mirrakhimov, Awoke Misganaw,

Chaitanya Mittal, Babak Moazen, Madeline E Moberg, Soheil Mohammadi, Salahuddin Mohammed, Shafiu Mohammed, Ali H Mokdad, Sara Momtazmanesh, Lorenzo Monasta, Yousef Moradi, Maziar Moradi-Lakeh, Shane Douglas Morrison, Jakub Morze, Jonathan F Mosser, Vincent Mougin, Ahmed Msherghi, Sumaira Mubarik, Ulrich Otto Mueller, Francesk Mulita, Efrén Murillo-Zamora, Christopher J L Murray, Ghulam Mustafa, Ahamarshan Jayaraman Nagarajan, Ganesh R Naik, Sanjeev Nair, Aparna Ichalangod Narayana, Shumaila Nargus, Zuhair S Natto, Biswa Prakash Nayak, Ionut Negoi, Ruxandra Irina Negoi, Henok Biresaw Netsere, Josephine W Ngunjiri, Dang H Nguyen, Hien Quang Nguyen, Robina Khan Niazi, Taxiarchis Konstantinos Nikolouzakis, Chukwudi A Nnaji, Lawrence Achilles Nnyanzi, Shuhei Nomura, Bo Norrving, Mpiko Ntsekhe, Dieta Nurrika, Chimezie Igwegbe Nzoputam, Ogochukwu Janet Nzoputam, Bogdan Oancea, Ismail A Odetokun, Ayodipupo Sikiru Oguntade, James Odhiambo Oguta, Osaretin Christabel Okonji, Andrew T Olagunju, Matthew Idowu Olatubi, Bolajoko Olubukunola Olusanya, Jacob Olusegun Olusanya, Hany A Omar, Abidemi E Emmanuel Omonisi, Sandersan Onie, Obinna E Onwujekwe, Alberto Ortiz, Edgar Ortiz-Brizuela, Samuel M Ostroff, Adrian Otoiu, Stanislav S Otstavnov, Amel Ouyahia, Mayowa O Owolabi, Mahesh Padukudru P A, Jagadish Rao Padubidri, Seithikurippu R Pandi-Perumal, Leonidas D Panos, Anca Mihaela Pantea Stoian, Shahina Pardhan, Romil R Parikh, Maja Pasovic, Jay Patel, Sangram Kishor Patel, Shankargouda Patil, Shrikant Pawar, Spencer A Pease, Paolo Pedersini, Veincent Christian Filipino Pepito, Emmanuel K Peprah, Prince Peprah, Arokiasamy Perianayagam, William A Petri, Hoang Tran Pham, Manon Pigeolet, David M Pigott, Julian David Pillay, Zahra Zahid Piracha, Saeed Pirouzpanah, Dietrich Plass, Maarten J Postma, Naeimeh Pourtaheri, Manya Prasad, Elton Junio Sady Prates, Natalie Pritchett, Jagadeesh Puvvula, Ibrahim Qattea, Asma Saleem Qazi, Raghu Anekal Radhakrishnan, Hadi Raeisi Shahraki, Quinn Rafferty, Md Jillur Rahim, Mohammad Rahmanian, Shakthi Kumaran Ramasamy, Sheena Ramazanu, Chhabi Lal Ranabhat, Nemanja Rancic, Chythra R Rao, Sowmya J Rao, Salman Rawaf, Christian Razo, Andre M N Renzaho, Bhageerathy Reshmi, Nima Rezaei, Peyman Rezaei Hachesu, Célia Fortuna Rodrigues, Jefferson Antonio Buendia Rodriguez, Leonardo Roever, Luca Ronfani, Gholamreza Roshandel, Kunle Rotimi, Himanshu Sekhar Rout, Priyanka Roy, Enrico Rubagotti, Aly M A Saad, Siamak Sabour, Perminder S Sachdev, Basema Saddik, Adam Saddler, Bashdar Abuzed Sadee, Umar Saeed, Sher Zaman Safi, Rajesh Sagar, Zahra Saif, Mirza Rizwan Sajid, Afeez Abolarinwa Salami, Marwa Rashad Salem, Malik Sallam, Sara Samadzadeh, Abdallah M Samy, Francesca Sanna, Damian Francesco Santomauro, Itamar S Santos, Milena M Santric-Milicevic, Brijesh Sathian, Anudeep Sathyanarayan, Maheswar Satpathy, Monika Sawhney, Maria Inês Schmidt, David C Schwebel, Subramanian Senthilkumaran, Allen Seylani, Humaira Shah, Pritik A Shah, Masood Ali Shaikh, Sunder Sham, Muhammad Aaqib Shamim, Mohammad Anas Shamsi, Abhishek Shankar, Mohammed Shannawaz, Medha Sharath, Javad Sharifi-Rad, Rajesh Sharma, Vishal Sharma, Rajesh P Shastry, Maryam Shayan, Jemal Ebrahim Shifa, Aminu Shittu, K M Shivakumar, Sina Shool, Sunil Shrestha, Inga Dora Sigfusdottir, Luís Manuel Lopes Rodrigues Silva, Abhinav Singh, Harmanjit Singh, Jasvinder A Singh, Paramdeep Singh, Erica Leigh N Slepak, Reed J D Sorensen, Ireneous N Soyiri, Michael Spartalis, Chandrashekhar T Sreeramareddy, Benjamin A Stark, Caroline Stein, Timothy J Steiner, Jaimie D Steinmetz, Mark A Stokes, Muhammad Suleman, Rizwan Suliankatchi Abdulkader, Abida Sultana, Johan Sundström, Chandan Kumar Swain, Payam Tabaee Damavandi, Rafael Tabarés-Seisdedos, Shima Tabatabai, Mohammad Tabish, Santosh Kumar Tadakamadla, Yasaman Taheri Abkenar, Amir Taherkhani, Jabeen Taiba, Mircea Tampa, Ker-Kan Tan, Pugazhenthan Thangaraju, Nihal Thomas, Nikhil Kenny Thomas, Chern Choong Chern Thum, Tala Tillawi, Ruoyan Tobe-Gai, Roman Topor-Madry, Mathilde Touvier, Marcos Roberto Tovani-Palone, Jasmine T Tran, Mai Thi Ngoc Tran, Domenico Trico, Guesh Mebrahtom Tsegay, Munkhtuya Tumurkhuu, Muhammad Umair, Srikanth Umakanthan,

Tungki Pratama Umar, Bhaskaran Unnikrishnan, Era Upadhyay, Jibrin Sammani Usman, Jef Van den Eynde, Tommi Juhani Vasankari, Balachandar Vellingiri, Narayanaswamy Venketasubramanian, Georgios-Ioannis Verras, Vasily Vlassov, Simona Ruxandra Volovat, Avina Vongpradith, Theo Vos, Yasir Waheed, Shu Wang, Daniel J Weiss, Abrha Hailay Weldemariam, Joanna L Whisnant, Harvey A Whiteford, Taweewat Wiangkham, Dakshitha Praneeth Wickramasinghe, Angga Wilandika, Caroline Wilkerson, Peter Willeit, Demewoz H Woldegebreal, Yen Jun Wong, Felicia Wu, Zenghong Wu, Sarah Wulf Hanson, Yvonne Yiru Xu, Ali Yadollahpour, Kazumasa Yamagishi, Yao Yao, Habib Yaribeygi, Pengpeng Ye, Vahit Yiğit, Dong Keon Yon, Naohiro Yonemoto, Mustafa Z Younis, Chuanhua Yu, Yong Yu, Hadiza Yusuf, Ghazal G Z Zandieh, Mikhail Sergeevich Zastrozhin, Magdalena Zielińska, Stephanie R M Zimsen, Mohammad Zoladl, and Alimuddin Zumla.

Developing methods or computational machinery

Cristiana Abbafati, Saira Afzal, Muktar Beshir Ahmed, Mohammed Albashtawy, Daniel T Araki, Aleksandr Y Aravkin, Michael Benjamin Arndt, Marcel Ausloos, Akshaya Srikanth Bhagavathula, Aadam Olalekan Bodunrin, Milad Bonakdar Hashemi, Hamed Borhany, Michael Brauer, Justin Byun, Catherine S Chen, Eunice Chung, Rebecca M Cogen, Xiaochen Dai, Nicole K DeCleene, Hardik Dineshbhai Desai, M Ashworth Dirac, Paulina Agnieszka Dzianach, Michael Ekholuenetale, Adeniyi Francis Fagbamigbe, Ali Fatehizadeh, Alize J Ferrari, Luisa S Flor, Lisa M Force, Mandukhai Ganbat, Mohd Ashraf Ganie, William M Gardner, Tesfay B B Gebremariam, Peter W Gething, Alireza Ghajar, Alem Girmay, Ayman Grada, Shi-Yang Guan, Ishita Gupta, Erin B Hamilton, Mohammad Hasanian, Simon I Hay, Mehdi Hosseinzadeh, Chantal K Huynh, Hong-Han Huynh, Kevin S Ikuta, Gaetano Isola, Morteza Jafarinia, Tahereh Javaheri, Sathish Kumar Jayapal, Charity Ehimwenma Joshua, Sanjay Kalra, Salah Eddin Karimi, Cathleen Keller, Alireza Khalilian, Adnan Kisa, Jonathan M Kocarnik, Chandrakant Lahariya, Huu-Hoai Le, Nhi Huu Hanh Le, Sang-woong Lee, Megan Lindstrom, Kashish Malhotra, Andrea Maugeri, Michael A McPhail, Le Huu Nhat Minh, Madeline E Moberg, Salahuddin Mohammed, Shafiu Mohammed, Ali H Mokdad, Yousef Moradi, Jonathan F Mosser, Vincent Mougin, Francesk Mulita, Christopher J L Murray, Amir Nasrollahizadeh, Josephine W Ngunjiri, Dang H Nguyen, Hien Quang Nguyen, Bolajoko Olubukunola Olusanya, Jacob Olusegun Olusanya, Michal Ordak, Maja Pasovic, Spencer A Pease, Hoang Tran Pham, Saeed Pirouzpanah, Hadi Raeisi Shahraki, Quinn Rafferty, Chhabi Lal Ranabhat, Giridhara Rathnaiah Babu, Christian Razo, Robert C Reiner Jr, Peyman Rezaei Hachesu, Himanshu Sekhar Rout, Enrico Rubagotti, Adam Saddler, Umar Saeed, Abdallah M Samy, Francesca Sanna, Damian Francesco Santomauro, Maheswar Satpathy, Austin E Schumacher, Pritik A Shah, Mohammed Shannawaz, Vishal Sharma, Reed J D Sorensen, Michael Spartalis, Benjamin A Stark, Caitlyn Steiner, Timothy J Steiner, Jaimie D Steinmetz, Muhammad Suleman, Chandan Kumar Swain, Chern Choong Chern Thum, Tala Tillawi, Mai Thi Ngoc Tran, Muhammad Umair, Avina Vongpradith, Theo Vos, Daniel J Weiss, Joanna L Whisnant, Harvey A Whiteford, Demewoz H Woldegebreal, Yen Jun Wong, Zenghong Wu, Sarah Wulf Hanson, Yvonne Yiru Xu, Dong Keon Yon, Ghazal G Z Zandieh, and Stephanie R M Zimsen.

Providing critical feedback on methods or results

Amirali Aali, Yohannes Habtegiorgis Abate, Cristiana Abbafati, Hedayat Abbastabar, Samar Abd ElHafeez, Michael Abdelmasseh, Sherief Abd-Elsalam, Arash Abdollahi, Auwal Abdullahi, Roberto Ariel Abeldaño Zuñiga, Richard Gyan Aboagye, Hassan Abolhassani, Lucas Guimarães Abreu, Hasan Abualruz, Eman Abu-Gharbieh, Ilana N Ackerman, Isaac Yeboah Addo, Giovanni Addolorato, Abiola Victor Adepoju, Habeeb Omoponle Adewuyi, Shadi Afyouni, Saira Afzal, Sina Afzal, Antonella Agodi, Aqeel Ahmad, Danish Ahmad, Firdos Ahmad, Shahzaib Ahmad, Ali Ahmed, Luai A Ahmed, Muktar Beshir Ahmed,

Karolina Akinosoglou, Syed Mahfuz Al Hasan, Samer O Alalalmeh, Ziyad Al-Aly, Mohammed Albashtawy, Robert W Aldridge, Meseret Desalegn Alemu, Yihun Mulugeta Alemu, Kefyalew Addis Alene, Adel Ali Saeed Al-Gheethi, Maryam Alharrasi, Robert Kaba Alhassan, Mohammed Usman Ali, Rafat Ali, Syed Shujait Shujait Ali, Sheikh Mohammad Alif, Syed Mohamed Aljunid, Sabah Al-Marwani, Joseph Uy Almazan, Mahmoud A Alomari, Basem Al-Omari, Zaid Altaany, Nelson Alvis-Guzman, Nelson J Alvis-Zakzuk, Hassan Alwafi, Mohammad Sami Al-Wardat, Yaser Mohammed Al-Worafi, Safwat Aly, Karem H Alzoubi, Azmeraw T Amare, Prince M Amegbor, Edward Kwabena Ameyaw, Tarek Tawfik Amin, Alireza Amindarolzarbi, Sohrab Amiri, Dickson A Amugsi, Robert Ancuceanu, Deanna Anderlini, David B Anderson, Pedro Prata Andrade, Catalina Liliana Andrei, Hossein Ansari, Saleha Anwar, Sumadi Lukman Anwar, Razique Anwer, Philip Emeka Anyanwu, Juan Pablo Arab, Jalal Arabloo, Mosab Arafat, Mesay Arkew, Benedetta Armocida, Mahwish Arooj, Anton A Artamonov, Raphael Taiwo Aruleba, Ashokan Arumugam, Charlie Ashbaugh, Mubarek Yesse Ashemo, Muhammad Ashraf, Marvellous O Asika, Thomas Astell-Burt, Seyyed Shamsadin Athari, Prince Atorkey, Maha Moh'd Wahbi Atout, Alok Atreya, Avinash Aujayeb, Marcel Ausloos, Abolfazl Avan, Adedapo Wasiu Awotidebe, Kofi Awuviry-Newton, Beatriz Paulina Ayala Quintanilla, Sina Azadnajafabad, Rui M S Azevedo, Abraham Samuel Babu, Muhammad Badar, Ashish D Badiye, Soroush Baghdadi, Nasser Bagheri, Sulaiman Bah, Ruhai Bai, Jennifer L Baker, Shankar M Bakkannavar, Abdulaziz T Bako, Senthilkumar Balakrishnan, Palash Chandra Banik, Martina Barchitta, Hiba Jawdat Barqawi, Amadou Barrow, Sandra Barteit, Lingkan Barua, Somaye Bashiri Aliabadi, Afisu Basiru, Sanjay Basu, Saurav Basu, Prapthi Persis Bathini, Kavita Batra, Bernhard T Baune, Nebiyou Simegnew Bayileyegn, Babak Behnam, Amir Hossein Behnoush, Diana Fernanda Bejarano Ramirez, Michelle L Bell, Olorunjuwon Omolaja Bello, Apostolos Beloukas, Isabela M Bensenor, Eduardo Bernabe, Robert S Bernstein, Akshaya Srikanth Bhagavathula, Neeraj Bhala, Dinesh Bhandari, Ashish Bhargava, Sonu Bhaskar, Vivek Bhat, Gurjit Kaur Bhatti, Jasvinder Singh Bhatti, Manpreet S Bhatti, Rajbir Bhatti, Zulfiqar A Bhutta, Boris Bikbov, Jessica Devin Bishai, Veera R Bitra, Virginia Bodolica, Aadam Olalekan Bodunrin, Eyob Ketema Bogale, Milad Bonakdar Hashemi, Berrak Bora Basara, Hamed Borhany, Christopher Boxe, Oliver J Brady, Nicola Luigi Bragazzi, Dejana Braithwaite, Hermann Brenner, Julie Brown, Traolach Brugha, Norma B Bulamu, Danilo Buonsenso, Katrin Burkart, Richard A Burns, Reinhard Busse, Yasser Bustanji, Zahid A Butt, Justin Byun, Florentino Luciano Caetano dos Santos, Luis Alberto Cámera, Ismael R Campos-Nonato, Chao Cao, Angelo Capodici, Márcia Carvalho, Joao Mauricio Castaldelli-Maia, Carlos A Castañeda-Orjuela, Giulio Castelpietra, Alberico L Catapano, Luca Cegolon, Francieli Cembranel, Muthia Cenderadewi, Ester Cerin, Promit Ananyo Chakraborty, Raymond N C Chan, Rama Mohan Chandika, Eeshwar K Chandrasekar, Periklis Charalampous, Vijay Kumar Chattu, Victoria Chatzimavridou-Grigoriadou, An-Tian Chen, Haowei Chen, Esther T W Cheng, Odgerel Chimed-Ochir, Ritesh Chimoriya, William C S Cho, Sungchul Choi, Bryan Chong, Yuen Yu Chong, Sonali Gajanan Choudhari, Rajiv Chowdhury, Dinh-Toi Chu, Isaac Sunday Chukwu, Eric Chung, Eunice Chung, Muhammad Chutiyami, Alyssa Columbus, Joao Conde, Paolo Angelo Cortesi, Ewerton Cousin, Michael H Criqui, Natália Cruz-Martins, Omid Dadras, Siyu Dai, Xiaochen Dai, Zhaoli Dai, Maxwell Ayindenaba Dalaba, Giovanni Damiani, Jai K Das, Saswati Das, Mohsen Dashti, Claudio Alberto Dávila-Cervantes, Kairat Davletov, Diego De Leo, Aklilu Tamire Debele, Shayom Debopadhaya, Nicole K DeCleene, Farah Deeba, Louisa Degenhardt, Cristian Del Bo', Ivan Delgado-Enciso, Andreas K Demetriades, Nikolaos Dervenis, Hardik Dineshbhai Desai, Rupak Desai, Keshab Deuba, Kuldeep Dhama, Samath Dhamminda Dharmaratne, Sameer Dhingra, Diana Dias da Silva, Daniel Diaz, Michael J Diaz, Adriana Dima, Delaney D Ding, M Ashworth Dirac, Thao Huynh Phuong Do, Camila Bruneli do Prado, Deepa Dongarwar, Mario D'Oria, E Ray Dorsey, Leila Doshmangir, Robert Kokou Dowou, Haneil Larson Dsouza, Viola Dsouza, John

Dube, Samuel C Dumith, Senbagam Duraisamy, Oyewole Christopher Durojaiye, Paulina Agnieszka Dzianach, Arkadiusz Marian Dziedzic, Ejemai Eboreime, Alireza Ebrahimi, Hisham Atan Edinur, David Edvardsson, Terje Andreas Eikemo, Ebrahim Eini, Michael Ekholuenetale, Temitope Cyrus Ekundayo, Iman El Sayed, Noha Mousaad Elemam, Ghada Metwally Tawfik ElGohary, Muhammed Elhadi, Omar Abdelsadek Abdou Elmeligy, Gihan ELNahas, Mohammed Elshaer, Ibrahim Elsohaby, Luchuo Engelbert Bain, Babak Eshrati, Natalia Fabin, Adeniyi Francis Fagbamigbe, Luca Falzone, Mohammad Fareed, Carla Sofia e Sá Farinha, Andre Faro, Pegah Farrokhi, Ali Fatehizadeh, Valery L Feigin, Xiaoqi Feng, Seyed-Mohammad Fereshtehnejad, Alize J Ferrari, Paulo H Ferreira, Florian Fischer, Joanne Flavel, David Flood, Luisa S Flor, Nataliya A Foigt, Morenike Oluwatoyin Folayan, Lisa M Force, Daniela Fortuna, Matteo Foschi, Richard Charles Franklin, Alberto Freitas, Takeshi Fukumoto, João M Furtado, Peter Andras Gaal, Muktar A Gadanya, Abhay Motiramji Gaidhane, Santosh Gaihre, Yaseen Galali, Aravind P Gandhi, Balasankar Ganesan, Mohd Ashraf Ganie, Mohammad Arfat Ganiyani, William M Gardner, Tilaye Gebru Gebi, Miglas W Gebregergis, Mesfin Gebrehiwot, Tesfay B B Gebremariam, Teferi Gebru Gebremeskel, Simona Roxana Georgescu, Abera Getachew Obsa, Molla Getie, Keyghobad Ghadiri, Khalid Yaser Ghailan, Alireza Ghajar, Ghazal Ghasempour Dabaghi, Afsaneh Ghasemzadeh, Ramy Mohamed Ghazy, Elena Ghotbi, Ruth Margaret Gibson, Tiffany K Gill, Themba G Ginindza, Alem Girmay, James C Glasbey, Laszlo Göbölös, Myron Anthony Godinho, Salime Goharinezhad, Mohamad Goldust, Mahaveer Golechha, Philimon N Gona, Giuseppe Gorini, Alessandra C Goulart, Ayman Grada, Michal Grivna, Shi-Yang Guan, Mohammed Ibrahim Mohialdeen Gubari, Mesay Dechasa Gudeta, Avirup Guha, Stefano Guicciardi, Snigdha Gulati, David Gulisashvili, Damitha Asanga Gunawardane, Cui Guo, Anish Kumar Gupta, Bhawna Gupta, Ishita Gupta, Mohak Gupta, Veer Bala Gupta, Vijai Kumar Gupta, Vivek Kumar Gupta, Reyna Alma Gutiérrez, Farrokh Habibzadeh, Parham Habibzadeh, Rasool Haddadi, Najah R Hadi, Nils Haep, Nima Hafezi-Nejad, Abdul Hafiz, Aram Halimi, Sebastian Haller, Rabih Halwani, Erin B Hamilton, Md Nuruzzaman Haque, Harapan Harapan, Josep Maria Haro, Jan Hartvigsen, Ahmed I Hasaballah, Ikramul Hasan, Md Saquib Hasnain, Johannes Haubold, Rasmus J Havmoeller, Simon I Hay, Khezar Hayat, Omar E Hegazi, Golnaz Heidari, Bartosz Helfer, Mehdi Hemmati, Delia Hendrie, Kamal Hezam, Yuta Hiraike, Nguyen Quoc Hoan, Ramesh Holla, Md Mahbub Hossain, Hassan Hosseinzadeh, Mehdi Hosseinzadeh, Mihaela Hostiuc, Kiavash Hushmandi, Javid Hussain, Nawfal R Hussein, Hong-Han Huynh, Bing-Fang Hwang, Kevin S Ikuta, Olayinka Stephen Ilesanmi, Irena M Ilic, Milena D Ilic, Mohammad Tarique Imam, Mustapha Immurana, Lalu Muhammad Irham, Md Rabiul Islam, Sheikh Mohammed Shariful Islam, Farhad Islami, Faisal Ismail, Nahlah Elkudssiah Ismail, Gaetano Isola, Masao Iwagami, Chidozie C D Iwu, Mahalaxmi Iyer, Jalil Jaafari, Kathryn H Jacobsen, Farhad Jadidi-Niaragh, Morteza Jafarinia, Khushleen Jaggi, Nader Jahanmehr, Haitham Jahrami, Akhil Jain, Nityanand Jain, Ammar Abdulrahman Jairoun, Abhishek Jaiswal, Mihajlo Jakovljevic, Abubakar Ibrahim Jatau, Sabzali Javadov, Tahereh Javaheri, Sathish Kumar Jayapal, Shubha Jayaram, Sun Ha Jee, Jayakumar Jeganathan, Angeline Jeyakumar, Anil K Jha, Heng Jiang, Yinzi Jin, Jost B Jonas, Tamas Joo, Abel Joseph, Nitin Joseph, Charity Ehimwenma Joshua, Jacek Jerzy Jozwiak, Mikk Jürisson, Vaishali K, Billingsley Kaambwa, Ali Kabir, Zubair Kabir, Vidya Kadashetti, Leila R Kalankesh, Feroze Kaliyadan, Sanjay Kalra, Kaloyan Kamenov, Naser Kamyari, Thanigaivelan Kanagasabai, Himal Kandel, Arun R Kanmanthareddy, Kehinde Kazeem Kanmodi, Rami S Kantar, Ibraheem M Karaye, Asima Karim, Salah Eddin Karimi, Yeganeh Karimi, Norito Kawakami, Gbenga A Kayode, Foad Kazemi, Sina Kazemian, Leila Keikavoosi-Arani, Cathleen Keller, John H Kempen, Jessica A Kerr, Kamyab Keshtkar, Emmanuelle Kesse-Guyot, Mohammad Keykhaei, Himanshu Khajuria, Amirmohammad Khalaji, Asaad Khalid, Nauman Khalid, Alireza Khalilian, Faham Khamesipour, Asaduzzaman Khan, Ikramullah Khan, Maseer Khan, Moien AB Khan, Shaghayegh Khanmohammadi,

Khaled Khatab, Fatemeh Khatami, Moawiah Mohammad Khatatbeh, Amir M Khater, Feriha Fatima Khidri, Moein Khormali, Zahra Khorrami, Zemene Demelash Kifle, Min Seo Kim, Ruth W Kimokoti, Adnan Kisa, Sezer Kisa, Ann Kristin Skrindo Knudsen, Jonathan M Kocarnik, Sonali Kochhar, Hyun Yong Koh, Ali-Asghar Kolahi, Farzad Kompani, Gerbrand Koren, Oleksii Korzh, Sindhura Lakshmi Koulmane Laxminarayana, Kewal Krishan, Varun Krishna, Vijay Krishnamoorthy, Barthelemy Kuate Defo, Md Abdul Kuddus, Mohammed Kuddus, Ilari Kuitunen, Vishnutheertha Kulkarni, Nithin Kumar, Om P Kurmi, Dian Kusuma, Hmwe Hmwe Kyu, Carlo La Vecchia, Muhammad Awwal Ladan, Alessandra Lafranconi, Chandrakant Lahariya, Daphne Teck Ching Lai, Dharmesh Kumar Lal, Tea Lallukka, Judit Lám, Qing Lan, Tuo Lan, Iván Landires, Francesco Lanfranchi, Bagher Larijani, Savita Lasrado, Paolo Lauriola, Huu-Hoai Le, Long Khanh Dao Le, Nhi Huu Hanh Le, Trang Diep Thanh Le, Janet L Leasher, Caterina Ledda, Munjae Lee, Sang-woong Lee, Seung Won Lee, Wei-Chen Lee, Yo Han Lee, Kate E LeGrand, Jacopo Lenzi, Elvynna Leong, Ming-Chieh Li, Wei Li, Xiaopan Li, Yongze Li, Lee-Ling Lim, Stephen S Lim, Megan Lindstrom, Shai Linn, Gang Liu, Shiwei Liu, Wei Liu, Xiaofeng Liu, Xuefeng Liu, Erand Llanaj, Chun-Han Lo, Rubén López-Bueno, Arianna Maever Loreche, László Lorenzovici, Jailos Lubinda, Giancarlo Lucchetti, Jay B Lusk, Zheng Feei Ma, Nikolaos Machairas, Áurea M Madureira-Carvalho, Javier A Magaña Gómez, Azzam A Maghazachi, Preeti Maharjan, Phetole Walter Mahasha, Mina Maheri, Soleiman Mahjoub, Mansour Adam Mahmoud, Elham Mahmoudi, Azeem Majeed, Konstantinos Christos Makris, Elaheh Malakan Rad, Kashish Malhotra, Ahmad Azam Malik, Iram Malik, Deborah Carvalho Malta, Yosef Manla, Ali Mansour, Pejman Mansouri, Mohammad Ali Mansournia, Emmanuel Manu, Hamid Reza Marateb, Parham Mardi, Ramon Martinez-Piedra, Daniela Martini, Francisco Rogerlândio Martins-Melo, Miquel Martorell, Wolfgang Marx, Sharmeen Maryam, Roy Rillera Marzo, Yasith Mathangasinghe, Stephanie Mathieson, Alexander G Mathioudakis, Jishanth Mattumpuram, Andrea Maugeri, Mahsa Mayeli, Mohsen Mazidi, Antonio Mazzotti, John J McGrath, Martin McKee, Anna Laura W McKowen, Michael A McPhail, Kamran Mehrabani-Zeinabad, Entezar Mehrabi Nasab, Tesfahun Mekene Meto, Walter Mendoza, Ritesh G Menezes, Alexios-Fotios A Mentis, Sultan Ayoub Meo, Haftu Asmerom Meresa, Atte Meretoja, Tuomo J Meretoja, Abera M Mersha, Tomislav Mestrovic, Kukulege Chamila Dinushi Mettananda, Sachith Mettananda, Irmina Maria Michalek, Ted R Miller, Edward J Mills, Le Huu Nhat Minh, Antonio Mirijello, Erkin M Mirrakhimov, Mizan Kiros Mirutse, Mohammad Mirza-Aghazadeh-Attari, Maryam Mirzaei, Awoke Misganaw, Ajay Kumar Mishra, Chaitanya Mittal, Babak Moazen, Madeline E Moberg, Jama Mohamed, Mouhand F H Mohamed, Nouh Saad Mohamed, Esmaeil Mohammadi, Soheil Mohammadi, Salahuddin Mohammed, Shafiu Mohammed, Ali H Mokdad, Sabrina Molinaro, Sara Momtazmanesh, Lorenzo Monasta, Stefania Mondello, AmirAli Moodi Ghalibaf, Maryam Moradi, Yousef Moradi, Maziar Moradi-Lakeh, Paula Moraga, Lidia Morawska, Rafael Silveira Moreira, Negar Morovatdar, Jakub Morze, Jonathan F Mosser, Elias Mossialos, Rohith Motappa, Simin Mouodi, Ahmed Msherghi, Sumaira Mubarik, Ulrich Otto Mueller, Francesk Mulita, Kavita Munjal, Efrén Murillo-Zamora, Christopher J L Murray, Ghulam Mustafa, Sathish Muthu, Muhammad Muzaffar, Woojae Myung, Ahamarshan Jayaraman Nagarajan, Pirouz Naghavi, Ganesh R Naik, Firzan Nainu, Sanjeev Nair, Hastyar Hama Rashid Najmuldeen, Vinay Nangia, Atta Abbas Naqvi, Shumaila Nargus, Gustavo G Nascimento, Abdulqadir J Nashwan, Ali Nasrollahizadeh, Amir Nasrollahizadeh, Zuhair S Natto, Biswa Prakash Nayak, Vinod C Nayak, Hadush Negash, Ionut Negoi, Ruxandra Irina Negoi, Seyed Aria Nejadghaderi, Olivia D Nesbit, Henok Biresaw Netsere, Marie Ng, Georges Nguefack-Tsague, Josephine W Ngunjiri, Dang H Nguyen, Hien Quang Nguyen, Robina Khan Niazi, Taxiarchis Konstantinos Nikolouzakis, Ali Nikoobar, Amin Reza Nikpoor, Chukwudi A Nnaji, Lawrence Achilles Nnyanzi, Efaq Ali Noman, Shuhei Nomura, Bo Norrving, Chisom Adaobi Nri-Ezedi, George Ntaios, Mpiko Ntsekhe, Dieta Nurrika, Chimezie Igwegbe Nzoputam,

Ogochukwu Janet Nzoputam, Bogdan Oancea, Ismail A Odetokun, Martin James O'Donnell, Ayodipupo Sikiru Oguntade, James Odhiambo Oguta, Hassan Okati-Aliabad, Akinkunmi Paul Okekunle, Osaretin Christabel Okonji, Andrew T Olagunju, Omotola O Olasupo, Matthew Idowu Olatubi, Isaac Iyinoluwa Olufadewa, Bolajoko Olubukunola Olusanya, Jacob Olusegun Olusanya, Hany A Omar, Goran Latif Omer, Abidemi E Emmanuel Omonisi, Obinna E Onwujekwe, Michal Ordak, Doris V Ortega-Altamirano, Alberto Ortiz, Edgar Ortiz-Brizuela, Wael M S Osman, Samuel M Ostroff, Uchechukwu Levi Osuagwu, Adrian Otoiu, Nikita Otstavnov, Stanislav S Otstavnov, Amel Ouyahia, Mayowa O Owolabi, Mahesh Padukudru P A, Alicia Padron-Monedero, Jagadish Rao Padubidri, Tamás Palicz, Feng Pan, Seithikurippu R Pandi-Perumal, Helena Ullyartha Pangaribuan, Georgios D Panos, Leonidas D Panos, Anca Mihaela Pantea Stoian, Shahina Pardhan, Romil R Parikh, Ava Pashaei, Maja Pasovic, Roberto Passera, Jay Patel, Sangram Kishor Patel, Shankargouda Patil, Dimitrios Patoulias, Shrikant Pawar, Amy E Peden, Paolo Pedersini, Veincent Christian Filipino Pepito, Emmanuel K Peprah, Prince Peprah, João Perdigão, Maria Odete Pereira, Arokiasamy Perianayagam, Konrad Pesudovs, Fanny Emily Petermann-Rocha, William A Petri, Hoang Tran Pham, Anil K Philip, Michael R Phillips, Manon Pigeolet, David M Pigott, Julian David Pillay, Zahra Zahid Piracha, Saeed Pirouzpanah, Dietrich Plass, Evgenii Plotnikov, Dimitri Poddighe, Maarten J Postma, Naeimeh Pourtaheri, Sergio I Prada, Pranil Man Singh Pradhan, V Prakash, Manya Prasad, Elton Junio Sady Prates, Tina Priscilla, Natalie Pritchett, Pooja Puri, Jagadeesh Puvvula, Nameer Hashim Qasim, Ibrahim Qattea, Asma Saleem Qazi, Gangzhen Qian, Mehrdad Rabiee Rad, Raghu Anekal Radhakrishnan, Venkatraman Radhakrishnan, Hadi Raeisi Shahraki, Quinn Rafferty, Pankaja Raghav Raghav, Md Jillur Rahim, Md Mosfequr Rahman, Mohammad Hifz Ur Rahman, Mosiur Rahman, Muhammad Aziz Rahman, Shayan Rahmani, Mohammad Rahmanian, Setyaningrum Rahmawaty, Sathish Rajaa, Mahmoud Mohammed Ramadan, Shakthi Kumaran Ramasamy, Premkumar Ramasubramani, Kritika Rana, Chhabi Lal Ranabhat, Nemanja Rancic, Amey Rane, Chythra R Rao, Mithun Rao, Sowmya J Rao, Mohammad-Mahdi Rashidi, Giridhara Rathnaiah Babu, Santosh Kumar Rauniyar, David Laith Rawaf, Salman Rawaf, Christian Razo, Murali Mohan Rama Krishna Reddy, Elrashdy Moustafa Mohamed Redwan, Lennart Reifels, Robert C Reiner Jr, Andre M N Renzaho, Bhageerathy Reshmi, Luis Felipe Reyes, Nazila Rezaei, Negar Rezaei, Nima Rezaei, Mohsen Rezaeian, Jennifer Rickard, Célia Fortuna Rodrigues, Jefferson Antonio Buendia Rodriguez, Leonardo Roever, Gholamreza Roshandel, Kunle Rotimi, Himanshu Sekhar Rout, Nitai Roy, Enrico Rubagotti, Chandan S N, Aly M A Saad, Maha Mohamed Saber-Ayad, Siamak Sabour, Basema Saddik, Bashdar Abuzed Sadee, Erfan Sadeghi, Mohammad Reza Saeb, Umar Saeed, Sher Zaman Safi, Rajesh Sagar, Zahra Saif, Mirza Rizwan Sajid, Joseph W Sakshaug, Afeez Abolarinwa Salami, Luciane B Salaroli, Mohamed A Saleh, Marwa Rashad Salem, Malik Sallam, Sara Samadzadeh, Saad Samargandy, Yoseph Leonardo Samodra, Abdallah M Samy, Juan Sanabria, Damian Francesco Santomauro, Itamar S Santos, Milena M Santric-Milicevic, Yaser Sarikhani, Rodrigo Sarmiento-Suárez, Gargi Sachin Sarode, Sachin C Sarode, Arash Sarveazad, Brijesh Sathian, Anudeep Sathyanarayan, Maheswar Satpathy, Monika Sawhney, Nikolaos Scarmeas, Benedikt Michael Schaarschmidt, Ione Jayce Ceola Schneider, David C Schwebel, Falk Schwendicke, Mansour Sedighi, Sabyasachi Senapati, Subramanian Senthilkumaran, Sadaf G Sepanlou, Yashendra Sethi, Mahan Shafie, Humaira Shah, Nilay S Shah, Pritik A Shah, Ataollah Shahbandi, Samiah Shahid, Wajeehah Shahid, Masood Ali Shaikh, Alireza Shakeri, Ali S Shalash, Muhammad Aaqib Shamim, Mohammad Ali Shamshirgaran, Mohammad Anas Shamsi, Mohd Shanawaz, Abhishek Shankar, Mohammed Shannawaz, Medha Sharath, Amin Sharifan, Javad Sharifi-Rad, Rajesh Sharma, Saurab Sharma, Ujjawal Sharma, Vishal Sharma, Rajesh P Shastry, Amin Shavandi, Amir Mehdi Shayan, Maryam Shayan, Pavanchand H Shetty, Kenji Shibuya, Jemal Ebrahim Shifa, Desalegn Shiferaw, Wondimeneh Shibabaw Shiferaw, Mika Shigematsu, Rahman Shiri,

Nebiyu Aniley Shitaye, Aminu Shittu, K M Shivakumar, Velizar Shivarov, Sina Shool, Sunil Shrestha, Kerem Shuval, Migbar Mekonnen Sibhat, Emmanuel Edwar Siddig, Inga Dora Sigfusdottir, Diego Augusto Santos Silva, João Pedro Silva, Luís Manuel Lopes Rodrigues Silva, Soraia Silva, Anjali Singal, Abhinav Singh, Balbir Bagicha Singh, Harmanjit Singh, Jasvinder A Singh, Mahendra Singh, Paramdeep Singh, Søren T Skou, David A Sleet, Sameh S M Soliman, Suhang Song, Yimeng Song, Reed J D Sorensen, Joan B Soriano, Ireneous N Soyiri, Michael Spartalis, Chandrashekhar T Sreeramareddy, Benjamin A Stark, Antonina V Starodubova, Caroline Stein, Caitlyn Steiner, Timothy J Steiner, Jaimie D Steinmetz, Paschalis Steiropoulos, Leo Stockfelt, Mark A Stokes, Vetriselvan Subramaniyan, Muhammad Suleman, Rizwan Suliankatchi Abdulkader, Abida Sultana, Johan Sundström, Chandan Kumar Swain, Lukasz Szarpak, Payam Tabaee Damavandi, Rafael Tabarés-Seisdedos, Ozra Tabatabaei Malazy, Seyed-Amir Tabatabaeizadeh, Shima Tabatabai, Celine Tabche, Mohammad Tabish, Santosh Kumar Tadakamadla, Yasaman Taheri Abkenar, Moslem Taheri Soodejani, Jabeen Taiba, Iman M Talaat, Mircea Tampa, Jacques Lukenze Tamuzi, Ker-Kan Tan, Sarmila Tandukar, Haosu Tang, Razieh Tavakoli Oliaee, Seyed Mohammad Tavangar, Mohamad-Hani Temsah, Masayuki Teramoto, Pugazhenthan Thangaraju, Kavumpurathu Raman Thankappan, Rekha Thapar, Rasiah Thayakaran, Sathish Thirunavukkarasu, Nihal Thomas, Nikhil Kenny Thomas, Chern Choong Chern Thum, Jansje Henny Vera Ticoalu, Tala Tillawi, Tenaw Yimer Tiruye, Marcello Tonelli, Roman Topor-Madry, Mathilde Touvier, Marcos Roberto Tovani-Palone, Jasmine T Tran, Mai Thi Ngoc Tran, Nghia Minh Tran, Ngoc-Ha Tran, Domenico Trico, Samuel Joseph Tromans, Guesh Mebrahtom Tsegay, Evangelia Eirini Tsermpini, Munkhtuya Tumurkhuu, Stefanos Tyrovolas, Arit Udoh, Muhammad Umair, Srikanth Umakanthan, Tungki Pratama Umar, Eduardo A Undurraga, Bhaskaran Unnikrishnan, Carolyn Anne Unsworth, Era Upadhyay, Daniele Urso, Jibrin Sammani Usman, Seyed Mohammad Vahabi, Asokan Govindaraj Vaithinathan, Jef Van den Eynde, Orsolya Varga, Priya Vart, Milena Vasic, Siavash Vaziri, Balachandar Vellingiri, Narayanaswamy Venketasubramanian, Massimiliano Veroux, Georgios-Ioannis Verras, Dominique Vervoort, Jorge Hugo Villafañe, Francesco S Violante, Vasily Vlassov, Stein Emil Vollset, Simona Ruxandra Volovat, Avina Vongpradith, Theo Vos, Yasir Waheed, Cong Wang, Fang Wang, Ning Wang, Shu Wang, Yanzhong Wang, Yuan-Pang Wang, Paul Ward, Emebet Gashaw Wassie, Kosala Gayan Weerakoon, Abrha Hailay Weldemariam, Yi Feng Wen, Joanna L Whisnant, Harvey A Whiteford, Taweewat Wiangkham, Dakshitha Praneeth Wickramasinghe, Nuwan Darshana Wickramasinghe, Angga Wilandika, Peter Willeit, Anders Wimo, Axel Walter Wolf, Yen Jun Wong, Anthony D Woolf, Chenkai Wu, Felicia Wu, Sarah Wulf Hanson, Yanjie Xia, Hong Xiao, Xiaoyue Xu, Yvonne Yiru Xu, Ali Yadollahpour, Lin Yang, Yuichiro Yano, Yao Yao, Habib Yaribeygi, Mohammad Hosein Yazdanpanah, Pengpeng Ye, Sisay Shewasinad Yehualashet, Subah Abderehim Yesuf, Saber Yezli, Arzu Yiğit, Vahit Yiğit, Zeamanuel Anteneh Yigzaw, Yazachew Yismaw, Dong Keon Yon, Naohiro Yonemoto, Mustafa Z Younis, Chuanhua Yu, Yong Yu, Hadiza Yusuf, Mondal Hasan Zahid, Fathiah Zakham, Leila Zaki, Nazar Zaki, Burhan Abdullah Zaman, Nelson Zamora, Ramin Zand, Ghazal G Z Zandieh, Armin Zarrintan, Mikhail Sergeevich Zastrozhin, Haijun Zhang, Ning Zhang, Yunquan Zhang, Chenwen Zhong, Juexiao Zhou, Zhaohua Zhu, Magdalena Zielińska, Stephanie R M Zimsen, Mohammad Zoladl, Alimuddin Zumla, and Samer H Zyoud.

Drafting the work or revising it critically for important intellectual content

Amirali Aali, Yohannes Habtegiorgis Abate, Cristiana Abbafati, Samar Abd ElHafeez, Michael Abdelmasseh, Sherief Abd-Elsalam, Arash Abdollahi, Auwal Abdullahi, Kedir Hussein Abegaz, Roberto Ariel Abeldaño Zuñiga, Hassan Abolhassani, Lucas Guimarães Abreu, Hasan Abualruz, Eman Abu-Gharbieh, Niveen ME Abu-Rmeileh, Ilana N Ackerman, Isaac Yeboah Addo, Giovanni Addolorato, Akindele Olupelumi Adebiyi, Abiola Victor Adepoju, Shadi Afyouni, Saira Afzal, Antonella Agodi, Danish

Ahmad, Firdos Ahmad, Luai A Ahmed, Muktar Beshir Ahmed, Marjan Ajami, Mohammed Ahmed Akkaif, Samer O Alalalmeh, Mohammed Albashtawy, Kefyalew Addis Alene, Maryam Alharrasi, Robert Kaba Alhassan, Mohammed Usman Ali, Rafat Ali, Syed Shujait Shujait Ali, Sheikh Mohammad Alif, Joseph Uy Almazan, Nelson Alvis-Guzman, Nelson J Alvis-Zakzuk, Hassan Alwafi, Mohammad Sami Al-Wardat, Yaser Mohammed Al-Worafi, Safwat Aly, Karem H Alzoubi, Azmeraw T Amare, Prince M Amegbor, Tarek Tawfik Amin, Alireza Amindarolzarbi, Sohrab Amiri, Dickson A Amugsi, Robert Ancuceanu, Deanna Anderlini, David B Anderson, Pedro Prata Andrade, Catalina Liliana Andrei, Catherine M Antony, Saleha Anwar, Razique Anwer, Juan Pablo Arab, Jalal Arabloo, Mesay Arkew, Benedetta Armocida, Mahwish Arooj, Raphael Taiwo Aruleba, Ashokan Arumugam, Muhammad Ashraf, Marvellous O Asika, Elaheh Askari, Seyyed Shamsadin Athari, Prince Atorkey, Maha Moh'd Wahbi Atout, Alok Atreya, Avinash Aujayeb, Marcel Ausloos, Abolfazl Avan, Adedapo Wasiu Awotidebe, Kofi Awuviry-Newton, Beatriz Paulina Ayala Quintanilla, Sina Azadnajafabad, Rui M S Azevedo, Abraham Samuel Babu, Muhammad Badar, Ashish D Badiye, Soroush Baghdadi, Sulaiman Bah, Jennifer L Baker, Abdulaziz T Bako, Senthilkumar Balakrishnan, Kiran Bam, Martina Barchitta, Mainak Bardhan, Erfan Bardideh, Suzanne Lyn Barker-Collo, Hiba Jawdat Barqawi, Amadou Barrow, Somaye Bashiri Aliabadi, Afisu Basiru, Sanjay Basu, Prapthi Persis Bathini, Bernhard T Baune, Babak Behnam, Amir Hossein Behnoush, Maryam Beiranvand, Diana Fernanda Bejarano Ramirez, Michelle L Bell, Olorunjuwon Omolaja Bello, Apostolos Beloukas, Isabela M Bensenor, Zombor Berezvai, Eduardo Bernabe, Paulo J G Bettencourt, Akshaya Srikanth Bhagavathula, Neeraj Bhala, Dinesh Bhandari, Ashish Bhargava, Sonu Bhaskar, Vivek Bhat, Gurjit Kaur Bhatti, Jasvinder Singh Bhatti, Manpreet S Bhatti, Rajbir Bhatti, Boris Bikbov, Jessica Devin Bishai, Catherine Bisignano, Veera R Bitra, Tone Bjørge, Virginia Bodolica, Milad Bonakdar Hashemi, Aime Bonny, Hamed Borhany, Christopher Boxe, Oliver J Brady, Nicola Luigi Bragazzi, Dejana Braithwaite, Luisa C Brant, Susanne Breitner, Hermann Brenner, Julie Brown, Traolach Brugha, Norma B Bulamu, Danilo Buonsenso, Richard A Burns, Yasser Bustanji, Justin Byun, Florentino Luciano Caetano dos Santos, Daniela Calina, Ismael R Campos-Nonato, Angelo Capodici, Sinclair Carr, Giulia Carreras, Andrea Carugno, Márcia Carvalho, Joao Mauricio Castaldelli-Maia, Carlos A Castañeda-Orjuela, Giulio Castelpietra, Alberico L Catapano, Maria Sofia Cattaruzza, Arthur Caye, Luca Cegolon, Francieli Cembranel, Muthia Cenderadewi, Ester Cerin, Jeffrey Shi Kai Chan, Rama Mohan Chandika, Eeshwar K Chandrasekar, Vijay Kumar Chattu, Victoria Chatzimavridou-Grigoriadou, An-Tian Chen, Haowei Chen, Ritesh Chimoriya, Patrick R Ching, William C S Cho, Sungchul Choi, Bryan Chong, Yuen Yu Chong, Rajiv Chowdhury, Steffan Wittrup McPhee Christensen, Dinh-Toi Chu, Eric Chung, Muhammad Chutiyami, Mareli M Claassens, Alyssa Columbus, Joao Conde, Ewerton Cousin, Michael H Criqui, Natália Cruz-Martins, Siyu Dai, Zhaoli Dai, Maxwell Ayindenaba Dalaba, Giovanni Damiani, Mohsen Dashti, Claudio Alberto Dávila-Cervantes, Aklilu Tamire Debele, Louisa Degenhardt, Ivan Delgado-Enciso, Andreas K Demetriades, Edgar Denova-Gutiérrez, Nikolaos Dervenis, Hardik Dineshbhai Desai, Rupak Desai, Samath Dhamminda Dharmaratne, Sameer Dhingra, Diana Dias da Silva, Daniel Diaz, Luis Antonio Diaz, Michael J Diaz, Adriana Dima, Delaney D Ding, M Ashworth Dirac, Camila Bruneli do Prado, Sushil Dohare, Wanyue Dong, Deepa Dongarwar, Mario D'Oria, E Ray Dorsey, Leila Doshmangir, Robert Kokou Dowou, Tim Robert Driscoll, Haneil Larson Dsouza, John Dube, Samuel C Dumith, Bruce B Duncan, Senbagam Duraisamy, Oyewole Christopher Durojaiye, Arkadiusz Marian Dziedzic, Ejemai Eboreime, David Edvardsson, Ebrahim Eini, Michael Ekholuenetale, Iman El Sayed, Maha El Tantawi, Iffat Elbarazi, Noha Mousaad Elemam, Ghada Metwally Tawfik ElGohary, Muhammed Elhadi, Omar Abdelsadek Abdou Elmeligy, Gihan ELNahas, Mohammed Elshaer, Ibrahim Elsohaby, Ryenchindorj Erkhembayar, Natalia Fabin, Adeniyi Francis Fagbamigbe, Luca Falzone, Carla Sofia e Sá Farinha, MoezAlIslam Ezzat Mahmoud

Faris, Andre Faro, Ali Fatehizadeh, Nelsensius Klau Fauk, Valery L Feigin, Seyed-Mohammad Fereshtehnejad, Abdullah Hamid Feroze, Alize J Ferrari, Nuno Ferreira, Florian Fischer, Joanne Flavel, David Flood, Nataliya A Foigt, Morenike Oluwatoyin Folayan, Lisa M Force, Daniela Fortuna, Matteo Foschi, Alberto Freitas, Takeshi Fukumoto, Peter Andras Gaal, Muktar A Gadanya, Santosh Gaihre, Yaseen Galali, Aravind P Gandhi, Balasankar Ganesan, Mohd Ashraf Ganie, Mohammad Arfat Ganiyani, Tilaye Gebru Gebi, Miglas W Gebregergis, Yibeltal Yismaw Gela, Simona Roxana Georgescu, Abera Getachew Obsa, Molla Getie, Fataneh Ghadirian, Alireza Ghajar, MohammadReza Ghasemi, Ghazal Ghasempour Dabaghi, Afsaneh Ghasemzadeh, Ramy Mohamed Ghazy, Ali Gholamrezanezhad, Mahsa Ghorbani, Elena Ghotbi, Ruth Margaret Gibson, Tiffany K Gill, Alem Girmay, James C Glasbey, Laszlo Göbölös, Myron Anthony Godinho, Mohamad Goldust, Philimon N Gona, Alessandra C Goulart, Ayman Grada, Michal Grivna, Shi-Yang Guan, Giovanni Guarducci, Mohammed Ibrahim Mohialdeen Gubari, Mesay Dechasa Gudeta, Avirup Guha, Stefano Guicciardi, Snigdha Gulati, David Gulisashvili, Damitha Asanga Gunawardane, Cui Guo, Bhawna Gupta, Ishita Gupta, Mohak Gupta, Rajeev Gupta, Veer Bala Gupta, Vivek Kumar Gupta, Reyna Alma Gutiérrez, Farrokh Habibzadeh, Parham Habibzadeh, Najah R Hadi, Nils Haep, Nima Hafezi-Nejad, Abdul Hafiz, Hailey Hagins, Esam S Halboub, Aram Halimi, Rabih Halwani, Graeme J Hankey, Md Abdul Hannan, Harapan Harapan, Josep Maria Haro, Jan Hartvigsen, Ahmed I Hasaballah, Md Saquib Hasnain, Amr Hassan, Johannes Haubold, Rasmus J Havmoeller, Simon I Hay, Khezar Hayat, Jeffrey J Hebert, Omar E Hegazi, Golnaz Heidari, Bartosz Helfer, Mehdi Hemmati, Kamal Hezam, Yuta Hiraike, Nguyen Quoc Hoan, Ramesh Holla, Md Mahbub Hossain, Sorin Hostiuc, Johnathan M Hsu, Junjie Huang, Fernando N Hugo, Kiavash Hushmandi, Javid Hussain, Hong-Han Huynh, Vincent C Iannucci, Audrey L Ihler, Adalia I Ikiroma, Olayinka Stephen Ilesanmi, Irena M Ilic, Milena D Ilic, Mustapha Immurana, Lalu Muhammad Irham, Md Rabiul Islam, Sheikh Mohammed Shariful Islam, Farhad Islami, Faisal Ismail, Nahlah Elkudssiah Ismail, Gaetano Isola, Chidozie C D Iwu, Mahalaxmi Iyer, Kathryn H Jacobsen, Morteza Jafarinia, Khushleen Jaggi, Kasra Jahankhani, Nader Jahanmehr, Haitham Jahrami, Akhil Jain, Abhishek Jaiswal, Mihajlo Jakovljevic, Abubakar Ibrahim Jatau, Sathish Kumar Jayapal, Shubha Jayaram, Jost B Jonas, Tamas Joo, Abel Joseph, Nitin Joseph, Charity Ehimwenma Joshua, Jacek Jerzy Jozwiak, Mikk Jürisson, Ali Kabir, Vidya Kadashetti, Rizwan Kalani, Feroze Kaliyadan, Sanjay Kalra, Thanigaivelan Kanagasabai, Himal Kandel, Arun R Kanmanthareddy, Kehinde Kazeem Kanmodi, Rami S Kantar, Asima Karim, Yeganeh Karimi, Hengameh Kasraei, Joonas H Kauppila, Gbenga A Kayode, Foad Kazemi, Cathleen Keller, John H Kempen, Jessica A Kerr, Emmanuelle Kesse-Guyot, Himanshu Khajuria, Amirmohammad Khalaji, Asaad Khalid, Nauman Khalid, Ikramullah Khan, Maseer Khan, Moien AB Khan, Shaghayegh Khanmohammadi, Khaled Khatab, Moawiah Mohammad Khatatbeh, Hamid Reza Khayat Kashani, Feriha Fatima Khidri, Elaheh Khodadoust, Min Seo Kim, Adnan Kisa, Sezer Kisa, Ann Kristin Skrindo Knudsen, Sonali Kochhar, Hyun Yong Koh, Farzad Kompani, Oleksii Korzh, Sindhura Lakshmi Koulmane Laxminarayana, Kewal Krishan, Varun Krishna, Barthelemy Kuate Defo, Md Abdul Kuddus, Mohammed Kuddus, Ilari Kuitunen, Vishnutheertha Kulkarni, Rakesh Kumar, Om P Kurmi, Dian Kusuma, Carlo La Vecchia, Ben Lacey, Muhammad Awwal Ladan, Lucie Laflamme, Chandrakant Lahariya, Ratilal Lalloo, Tea Lallukka, Judit Lám, Iván Landires, Berthold Langguth, Ariane Laplante-Lévesque, Bagher Larijani, Anders O Larsson, Savita Lasrado, Huu-Hoai Le, Nhi Huu Hanh Le, Caterina Ledda, Paul H Lee, Jacopo Lenzi, Elvynna Leong, Wei Li, Lee-Ling Lim, Runben Liu, Wei Liu, Xuefeng Liu, Erand Llanaj, Chun-Han Lo, Rubén López-Bueno, László Lorenzovici, Giancarlo Lucchetti, Raimundas Lunevicius, Jay B Lusk, hengliang lv, Zheng Feei Ma, Nikolaos Machairas, Áurea M Madureira-Carvalho, Javier A Magaña Gómez, Preeti Maharjan, Mina Maheri, Soleiman Mahjoub, Mansour Adam Mahmoud, Elham Mahmoudi, Konstantinos Christos Makris, Elaheh Malakan Rad, Kashish Malhotra, Ahmad Azam

Malik, Deborah Carvalho Malta, Lorenzo Giovanni Mantovani, Emmanuel Manu, Hamid Reza Marateb, Parham Mardi, Gabriel Martinez, Ramon Martinez-Piedra, Daniela Martini, Francisco Rogerlândio Martins-Melo, Miquel Martorell, Wolfgang Marx, Sharmeen Maryam, Roy Rillera Marzo, Yasith Mathangasinghe, Stephanie Mathieson, Alexander G Mathioudakis, Jishanth Mattumpuram, Andrea Maugeri, Mahsa Mayeli, Antonio Mazzotti, John J McGrath, Anna Laura W McKowen, Kamran Mehrabani-Zeinabad, Entezar Mehrabi Nasab, Walter Mendoza, Ritesh G Menezes, George A Mensah, Alexios-Fotios A Mentis, Sultan Ayoub Meo, Haftu Asmerom Meresa, Atte Meretoja, Tuomo J Meretoja, Tomislav Mestrovic, Kukulege Chamila Dinushi Mettananda, Sachith Mettananda, Irmina Maria Michalek, Ted R Miller, Edward J Mills, Le Huu Nhat Minh, Antonio Mirijello, Mohammad Mirza-Aghazadeh-Attari, Roya Mirzaei, Awoke Misganaw, Philip B Mitchell, Chaitanya Mittal, Babak Moazen, Mouhand F H Mohamed, Nouh Saad Mohamed, Esmaeil Mohammadi, Hussen Mohammed, Salahuddin Mohammed, Shafiu Mohammed, Robin M Mohr, Ali H Mokdad, Sabrina Molinaro, Sara Momtazmanesh, Lorenzo Monasta, Stefania Mondello, AmirAli Moodi Ghalibaf, Maryam Moradi, Maziar Moradi-Lakeh, Paula Moraga, Rafael Silveira Moreira, Shane Douglas Morrison, Jakub Morze, Abbas Mosapour, Rohith Motappa, Simin Mouodi, Matías Mrejen, Ahmed Msherghi, Ulrich Otto Mueller, Francesk Mulita, BV Murlimanju, Christopher J L Murray, Ghulam Mustafa, Sathish Muthu, Muhammad Muzaffar, Ahamarshan Jayaraman Nagarajan, Sanjeev Nair, Aparna Ichalangod Narayana, Shumaila Nargus, Gustavo G Nascimento, Abdulqadir J Nashwan, Ali Nasrollahizadeh, Amir Nasrollahizadeh, Zuhair S Natto, Biswa Prakash Nayak, Vinod C Nayak, Sabina Onyinye Nduaguba, Hadush Negash, Ionut Negoi, Ruxandra Irina Negoi, Seyed Aria Nejadghaderi, Georges Nguefack-Tsague, Josephine W Ngunjiri, Dang H Nguyen, Hien Quang Nguyen, Robina Khan Niazi, Taxiarchis Konstantinos Nikolouzakis, Fatemeh Nikoomanesh, Amin Reza Nikpoor, Lawrence Achilles Nnyanzi, Bo Norrving, Chisom Adaobi Nri-Ezedi, George Ntaios, Mpiko Ntsekhe, Chimezie Igwegbe Nzoputam, Ogochukwu Janet Nzoputam, Bogdan Oancea, Ismail A Odetokun, Martin James O'Donnell, Ayodipupo Sikiru Oguntade, Sylvester Reuben Okeke, Akinkunmi Paul Okekunle, Osaretin Christabel Okonji, Andrew T Olagunju, Matthew Idowu Olatubi, Gláucia Maria Moraes Oliveira, Bolajoko Olubukunola Olusanya, Jacob Olusegun Olusanya, Hany A Omar, Goran Latif Omer, Abidemi E Emmanuel Omonisi, Sandersan Onie, Obinna E Onwujekwe, Michal Ordak, Verner N Orish, Doris V Ortega-Altamirano, Alberto Ortiz, Edgar Ortiz-Brizuela, Wael M S Osman, Samuel M Ostroff, Uchechukwu Levi Osuagwu, Adrian Otoiu, Nikita Otstavnov, Amel Ouyahia, Guoqing Ouyang, Mayowa O Owolabi, Mahesh Padukudru P A, Alicia Padron-Monedero, Jagadish Rao Padubidri, Tamás Palicz, Claudia Palladino, Feng Pan, Helena Ullyartha Pangaribuan, Leonidas D Panos, Anca Mihaela Pantea Stoian, Shahina Pardhan, Romil R Parikh, Ava Pashaei, Maja Pasovic, Roberto Passera, Jay Patel, Shankargouda Patil, Dimitrios Patoulias, Venkata Suresh Patthipati, Shrikant Pawar, Hamidreza Pazoki Toroudi, Amy E Peden, Paolo Pedersini, Minjin Peng, Umberto Pensato, João Perdigão, Arokiasamy Perianayagam, Norberto Perico, Konrad Pesudovs, Fanny Emily Petermann-Rocha, Hoang Tran Pham, Anil K Philip, Michael R Phillips, Julian David Pillay, Zahra Zahid Piracha, Saeed Pirouzpanah, Dietrich Plass, Suzanne Polinder, Maarten J Postma, Pranil Man Singh Pradhan, Manya Prasad, Elton Junio Sady Prates, Tina Priscilla, Nameer Hashim Qasim, Asma Saleem Qazi, Mehrdad Rabiee Rad, Raghu Anekal Radhakrishnan, Venkatraman Radhakrishnan, Hadi Raeisi Shahraki, Alberto Raggi, Pankaja Raghav Raghav, Mohammad Hifz Ur Rahman, Shayan Rahmani, Mohammad Rahmanian, Sathish Rajaa, Mahmoud Mohammed Ramadan, Shakthi Kumaran Ramasamy, Premkumar Ramasubramani, Kritika Rana, Chhabi Lal Ranabhat, Nemanja Rancic, Chythra R Rao, Kumuda Rao, Sowmya J Rao, Giridhara Rathnaiah Babu, Santosh Kumar Rauniyar, David Laith Rawaf, Salman Rawaf, Elrashdy Moustafa Mohamed Redwan, Giuseppe Remuzzi, Andre M N Renzaho, Bhageerathy Reshmi, Luis Felipe Reyes,

Nazila Rezaei, Nima Rezaei, Peyman Rezaei Hachesu, Jennifer Rickard, Jefferson Antonio Buendia Rodriguez, Leonardo Roever, Luca Ronfani, Kunle Rotimi, Himanshu Sekhar Rout, Bedanta Roy, Nitai Roy, Priyanka Roy, Enrico Rubagotti, Chandan S N, Aly M A Saad, Maha Mohamed Saber-Ayad, Simona Sacco, Perminder S Sachdev, Basema Saddik, Adam Saddler, Bashdar Abuzed Sadee, Masoumeh Sadeghi, Umar Saeed, Rajesh Sagar, Dominic Sagoe, Zahra Saif, Mirza Rizwan Sajid, Joseph W Sakshaug, Nasir Salam, Afeez Abolarinwa Salami, Marwa Rashad Salem, Mohammed Z Y Salem, Malik Sallam, Sara Samadzadeh, Saad Samargandy, Abdallah M Samy, Juan Sanabria, Damian Francesco Santomauro, Itamar S Santos, Milena M Santric-Milicevic, Made Ary Sarasmita, Yaser Sarikhani, Rodrigo Sarmiento-Suárez, Gargi Sachin Sarode, Sachin C Sarode, Arash Sarveazad, Anudeep Sathyanarayan, Maheswar Satpathy, Monika Sawhney, Nikolaos Scarmeas, Benedikt Michael Schaarschmidt, Maria Inês Schmidt, Ione Jayce Ceola Schneider, David C Schwebel, Sabyasachi Senapati, Sadaf G Sepanlou, Yashendra Sethi, Soko Setoguchi, Allen Seylani, Jamileh Shadid, Mahan Shafie, Nilay S Shah, Pritik A Shah, Samiah Shahid, Moyad Jamal Shahwan, Alireza Shakeri, Ali S Shalash, Muhammad Aaqib Shamim, Mohammad Anas Shamsi, Mohd Shanawaz, Abhishek Shankar, Mohammed Shannawaz, Medha Sharath, Amin Sharifan, Javad Sharifi-Rad, Manoj Sharma, Rajesh Sharma, Saurab Sharma, Ujjawal Sharma, Rajesh P Shastry, Amin Shavandi, Amr Mohamed Elsayed Shehabeldine, Pavanchand H Shetty, Kenji Shibuya, Jemal Ebrahim Shifa, Mika Shigematsu, Nebiyu Aniley Shitaye, Aminu Shittu, K M Shivakumar, Sina Shool, Kerem Shuval, Emmanuel Edwar Siddig, Diego Augusto Santos Silva, João Pedro Silva, Soraia Silva, Colin R Simpson, Abhinav Singh, Balbir Bagicha Singh, Harmanjit Singh, Jasvinder A Singh, Paramdeep Singh, Søren T Skou, Ranjan Solanki, Sameh S M Soliman, Suhang Song, Joan B Soriano, Ireneous N Soyiri, Michael Spartalis, Chandrashekhar T Sreeramareddy, Antonina V Starodubova, Dan J Stein, Timothy J Steiner, Paschalis Steiropoulos, Leo Stockfelt, Mark A Stokes, Narayan Subedi Subedi, Vetriselvan Subramaniyan, Claudia Kimie Suemoto, Muhammad Suleman, Abida Sultana, Johan Sundström, Chandan Kumar Swain, Lukasz Szarpak, Payam Tabaee Damavandi, Rafael Tabarés-Seisdedos, Ozra Tabatabaei Malazy, Seyed-Amir Tabatabaeizadeh, Celine Tabche, Santosh Kumar Tadakamadla, Yasaman Taheri Abkenar, Iman M Talaat, Ashis Talukder, Mircea Tampa, Jacques Lukenze Tamuzi, Ker-Kan Tan, Razieh Tavakoli Oliaee, Seyed Mohammad Tavangar, Mojtaba Teimoori, Mohamad-Hani Temsah, Masayuki Teramoto, Pugazhenthan Thangaraju, Sathish Thirunavukkarasu, Nihal Thomas, Chern Choong Chern Thum, Ales Tichopad, Tala Tillawi, Tenaw Yimer Tiruye, Marcello Tonelli, Roman Topor-Madry, Mathilde Touvier, Marcos Roberto Tovani-Palone, Jasmine T Tran, Mai Thi Ngoc Tran, Nghia Minh Tran, Ngoc-Ha Tran, Domenico Trico, Samuel Joseph Tromans, Thien Tan Tri Tai Truyen, Aristidis Tsatsakis, Evangelia Eirini Tsermpini, Stefanos Tyrovolas, Arit Udoh, Muhammad Umair, Tungki Pratama Umar, Eduardo A Undurraga, Brigid Unim, Bhaskaran Unnikrishnan, Carolyn Anne Unsworth, Era Upadhyay, Daniele Urso, Jibrin Sammani Usman, Asokan Govindaraj Vaithinathan, Jef Van den Eynde, Orsolya Varga, Ravi Prasad Varma, Priya Vart, Tommi Juhani Vasankari, Balachandar Vellingiri, Narayanaswamy Venketasubramanian, Massimiliano Veroux, Georgios-Ioannis Verras, Dominique Vervoort, Jorge Hugo Villafañe, Francesco S Violante, Vasily Vlassov, Stein Emil Vollset, Simona Ruxandra Volovat, Cong Wang, Fang Wang, Shu Wang, Yanzhong Wang, Yuan-Pang Wang, Paul Ward, Emebet Gashaw Wassie, Marcia R Weaver, Robert G Weintraub, Yi Feng Wen, Joanna L Whisnant, Taweewat Wiangkham, Dakshitha Praneeth Wickramasinghe, Nuwan Darshana Wickramasinghe, Peter Willeit, Anders Wimo, Axel Walter Wolf, Yen Jun Wong, Xinsheng Wu, Sarah Wulf Hanson, Hong Xiao, Lalit Yadav, Ali Yadollahpour, Sajad Yaghoubi, Kazumasa Yamagishi, Lin Yang, Mohammad Hosein Yazdanpanah, Sisay Shewasinad Yehualashet, Saber Yezli, Arzu Yiğit, Vahit Yiğit, Naohiro Yonemoto, Hadiza Yusuf, Mondal Hasan Zahid, Burhan Abdullah Zaman, Nelson Zamora, Ramin Zand, Ghazal G Z Zandieh, Heather J Zar, Armin

Zarrintan, Mikhail Sergeevich Zastrozhin, Haijun Zhang, Hanqing Zhao, Chenwen Zhong, Panliang Zhong, Zhaohua Zhu, Makan Ziafati, Magdalena Zielińska, Mohammad Zoladl, Alimuddin Zumla, and Samer H Zyoud.

Managing the estimation or publications process

Saira Afzal, Mohammed Albashtawy, Catherine M Antony, Charlie Ashbaugh, Marcel Ausloos, Mainak Bardhan, Milad Bonakdar Hashemi, Hamed Borhany, Michael Brauer, Justin Byun, Francieli Cembranel, Catherine S Chen, Dinh-Toi Chu, Giovanni Damiani, Robert Kokou Dowou, Iman El Sayed, Kara Estep, Ali Fatehizadeh, Alize J Ferrari, Lisa M Force, Mohd Ashraf Ganie, Alem Girmay, Ayman Grada, Shi-Yang Guan, Hailey Hagins, Erin B Hamilton, Simon I Hay, Hong-Han Huynh, Morteza Jafarinia, Haitham Jahrami, Abel Joseph, Sanjay Kalra, Rami S Kantar, Molly B Kassel, Jonathan M Kocarnik, Chandrakant Lahariya, Huu-Hoai Le, Nhi Huu Hanh Le, Kate E LeGrand, Ana M Mantilla Herrera, Roy Rillera Marzo, Anna Laura W McKowen, Paul Anthony Miller, Le Huu Nhat Minh, Madeline E Moberg, Salahuddin Mohammed, Robin M Mohr, Ali H Mokdad, Maryam Moradi, Jonathan F Mosser, Ahmed Msherghi, Christopher J L Murray, Samuel M Ostroff, Mahesh Padukudru P A, Maja Pasovic, Hoang Tran Pham, David M Pigott, Nemanja Rancic, Aly M A Saad, Bashdar Abuzed Sadee, Rajesh Sagar, Zahra Saif, Abdallah M Samy, Damian Francesco Santomauro, Maheswar Satpathy, Allen Seylani, Muhammad Aaqib Shamim, Vishal Sharma, Amr Mohamed Elsayed Shehabeldine, Zahra Shokati Eshkiki, Michael Spartalis, Caitlyn Steiner, Lukasz Szarpak, Pugazhenthan Thangaraju, Anna E Torre, Mai Thi Ngoc Tran, Theo Vos, Katherine M Wells, Harvey A Whiteford, Ghazal G Z Zandieh, and Mikhail Sergeevich Zastrozhin.
