# Supplementary material for: Global incidence, prevalence, years lived with disability (YLDs), disability-adjusted life-years (DALYs), and healthy life expectancy (HALE) for 371 diseases and injuries in 204 countries and territories and 811 subnational locations, 1990–2021: a systematic analysis for the Global Burden of Disease Study 2021
Source: Lancet. 2024 May 18;403(10440):2133–61. doi: 10.1016/S0140-6736(24)00757-8 (PMC11122111; doi:10.1016/S0140-6736(24)00757-8)
Supplement: Supplementary appendix 1 [file mmc1.pdf]

Appendix 1: methods appendix to "Global incidence, prevalence, years lived with disability (YLDs), disability-adjusted life-years (DALYs), and healthy life expectancy (HALE) for 371 diseases and injuries in 204 countries and territories and 811 subnational locations, 1990–2021: A systematic analysis for the Global Burden of Disease Study 2021"

Table of Contents

Section 1: GBD OVERVIEW 6

Section 1.1: Geographic locations of the analysis 6

Section 1.2: Time period of the analysis 6

Section 1.3: GBD cause list 7

Section 1.4: Statement of GATHER compliance 7

Section 1.5 GBD results overview 8

Section 1.6 Data input sources overview 8

Section 1.7 Funding sources 8

Section 1.8: Abbreviations 8

Section 2: NON-FATAL OUTCOME ESTIMATION 10

Section 2.1: Data sources, identification, and extraction 10

Section 2.1.1: Systematic reviews 10

Section 2.1.2: Survey data preparation 10

Section 2.1.3: Disease registries 11

Section 2.1.4: Case notifications 11

Section 2.2: Clinical input data and methods summary 11

Section 2.2.1: Mapping diagnoses to GBD diseases and injuries 11

Section 2.2.3: Inpatient hospital admissions 13

Section 2.2.4: Outpatient encounter data 16

Section 2.2.5: Estimation of the inpatient utilization envelope 17

Section 2.3 Data Adjustments 21

Section 2.3.1: Crosswalking 21

Section 2.3.2: Bias adjustment for alternative case definitions and study methods 22

Section 2.3.3: Example bias adjustment calculation 23

Section 2.3.4 Network Analysis 24

Section 2.3.5 Age sex splitting 25

Section 2.4: Spatiotemporal Gaussian process
regression (ST-GPR) modelling 25
Section 2.4.1 Estimating mean functions 24
Section 2.4.1: Estimating error variance 26
Section 2.4.2: Estimating the covariance function 29
Section 2.4.3: Prediction using GPR 29
Section 2.4.4: Subnational scaling and aggregation 30
Section 2.5: MR-BRT meta-regression modelling 31
Section 2.5.1 MR-BRT Overview 31
Section 2.5.2 MR-BRT Formula 30
Section 2.5.3 MR-BRT Features 32
Section 2.6: DisMod-MR 2.1 estimation 33
Section 2.6.1: Estimation of sequelae and causes 33
Section 2.6.2: DisMod-MR 2.1 description 33
Section 2.6.3: DisMod-MR 2.1 likelihood estimation 36
Section 2.7: Impairment and underlying cause estimation 38
Section 2.7.1: Impairment squeeze 38
Section 2.9: Disability weights 42
Section 2.9.1: GBD 2010 Disability Weights
Measurement Study 42
Section 2.9.2: GBD 2013 European Disability Weights
Measurement Study 44
Section 2.10: Comorbidity correction (COMO) 45
Section 2.11: YLD computation, uncertainty, and residual YLDs 47
Section 2.11.1: Residual YLDs 47
Section 2.12: Birth prevalence 47
Section 3: SDI 48
Section 4: ESTIMATION PROCESS FOR DALYS 50
Section 5: HALE 51

Section 6: NON-FATAL CAUSE-SPECIFIC MODELLING DESCRIPTIONS 51

Section 7: APPENDIX TABLES & FIGURES 1228

Section 8: AUTHOR'S CONTRIBUTION 1465

List of Tables & Figures

Appendix Figures:

Figure 1: GBD 2021 Claims Data Processing

Figure 2: GBD 2021 Inpatient Hospital Data Processing

Figure 3: GBD 2021 Outpatient data extraction process

Figure 4: Overview process of estimation of hospital envelope

Figure 5: Age-pattern used to age-split wide age bin data

Figure 6: Performance statistics comparing randomly and differentially culled datasets.

Figure 7: Performance statistics comparing datasets with specific measures held out vs. randomly or differentially culled datasets

Figure 8: SF-12 composite scores and disability weights for 60 health states with fitted loess regression

Figure 9: DALY burden estimation for GBD 2021

Appendix Tables:

Table 1: GBD 2021 location hierarchy with levels

Table 2: GBD 2021 cause hierarchy with level

Table 3: GATHER checklist of information that should be included in reports of global health estimates, with description of compliance and location of information for the Global Burden of Diseases, Injuries, and Risk Factors study 2021

Table 4: Sex-splitting Adjustment Factor

Table 5: Estimated coefficients of the inpatient envelope model.

Table 6: GBD 2021 sequelae, health states, health state lay descriptions, and disability weights

Table 7: GBD 2021 methods of estimating years lived with disability (YLDs) for 34 residual categories

Table 8: List of GBD 2021 non-fatal causes with prevalence at birth

Table 9: GBD 2021 Socio-Demographic Index groupings by location

Table 10: GBD 2021 Socio-Demographic Index R-squared values with lags up to 10 years

Table 11: GBD 2021 Socio-Demographic Index quintiles - or basically same as SDI values just listed in order of SDI value rather than by location groupings

Table 12: List of International Classification of Diseases (ICD) codes mapped to non-fatal causes and injuries in the GBD 2021

Section 1: GBD Overview

Section 1.1: Geographic locations of the analysis

We produced estimates for 204 countries and territories that were grouped into 21 regions and seven super regions (table 1). - The seven super-regions are central Europe, eastern Europe, and central Asia; high income; Latin America and the Caribbean; north Africa and the Middle East; south Asia; southeast Asia, east Asia, and Oceania; and sub-Saharan Africa. In GBD 2021 we continue to analyse at subnational levels countries that were added in previous cycles including Brazil, China, Ethiopia, India, Indonesia, Iran, Italy, Japan, Kenya, Mexico, New Zealand, Nigeria, Norway, Pakistan, Russia, the Philippines, Poland, South Africa, Sweden, the UK, and the USA. All analyses are at the first level of administrative organisation within each country except for New Zealand (by Māori ethnicity), Sweden (by Stockholm and non-Stockholm), the UK (by local government authorities), and the Philippines (by provinces). To meet data use requirements, in this publication we present subnational estimates for Brazil, India, Indonesia, Japan, Kenya, Mexico, Sweden, the UK, and the USA); given space constraints, these results are presented in Appendix 2 instead of the main text. Subnational estimates for China are included in maps but are not reported in appendix tables. Subnational estimates for other countries will be released in separate publications.

At the most detailed spatial resolution, we generated estimates for 983 unique locations. As was done in GBD 2019, in GBD 2021 we continue to use the set of locations defined as standard locations and non-standard locations. Standard GBD locations are defined as the set of all subnationals belonging to countries where data quality is high and with populations over 200 million, in addition to all other countries. Standard locations include the subnationals for China, India, the USA, and Brazil, but not Indonesia; data for China, India, the USA, and Brazil are also included at the country level. All other countries with subnational estimates are defined as non-standard locations.

Section 1.2: Time period of the analysis

We estimated numbers and rates of incidence, prevalence, years lived with disability (YLDs), and disability-adjusted life-years (DALYs) for the years 1990–2021; we estimated deaths and years of life lost (YLLs) for 1980–2021.

Section 1.3: GBD cause list

The GBD cause and sequelae list is organized hierarchically (see table 2) to accommodate different purposes and needs of various users.

The first two levels aggregate causes into general groupings. At Level 1 there are three cause groups: communicable, maternal, neonatal, and nutritional diseases (Group 1 diseases); non-communicable diseases (Group 2); and injuries (Group 3). These Level 1 aggregates are subdivided at Level 2 of the hierarchy into 22 cause groupings (eg, neonatal disorders, neurological disorders, and transport injuries). The disaggregation into Levels 3 and 4 contains the finest level of detail for causes captured in GBD 2021. The greatest detail available for some causes, such as anxiety disorders or rheumatoid arthritis, is at Level 3 of the hierarchy, while other specific causes are at Level 4 of the hierarchy with an aggregate category at Level 3 (for example, depressive disorders at Level 3, which encompasses major depressive disorders and dysthymia at Level 4). Sequelae of diseases and injuries are organised at Levels 5 and 6 of the hierarchy. In GBD, sequelae are defined as distinct, mutually exclusive categories of health consequences that can be directly attributed to a cause. For example, both neuropathy and blindness due to diabetic retinopathy are sequelae of diabetes; stroke and ischaemic heart disease are not, as these consequences cannot be categorically ascribed to diabetes in an individual despite good evidence for increased risk of these outcomes. The finest detail for all sequelae estimated in GBD is at Level 6 and is aggregated into summary sequelae categories (Level 5) for causes with large numbers of sequelae. Examples include the grouping of the infectious disease episodes and long-term sequelae of meningitis. For GBD 2021 there are 3499 mutually exclusive and collectively exhaustive sequela, 2089 cause sequelae and 1410 injuries sequelae, and thus our YLD estimates at each level of the hierarchy sum to the total of the level above. Prevalence and incidence aggregation is estimated at the level of individuals who may have more than one sequela or disease and therefore are not additive.

The GBD cause list continues to evolve to reflect the policy relevance, and public health and medical care importance of the causes of major losses of health. The cause and sequelae list expanded based on input from the Scientific Council and GBD collaborator network. For GBD 2021, the causes of death cause listhas increased to 288 causes, from the 286 causes in GBD 2019. The non-fatal cause list has expanded from 364 causes in GBD 2019 to 365 causes in GBD 2021. The total number of fatal and non-fatal causes combined for GBD 2021 is 371. As in GBD 2019, we made no estimates for YLDs for just five causes, either because no disability is possible (as is the case with sudden infant death syndrome); because disability may occur rarely but at levels too low for accurate estimation given the data (as for aortic aneurysm); or because the disability is captured by the complicating causes that led to that cause of death (as for indirect maternal deaths, late maternal deaths, and maternal deaths aggravated by HIV/AIDS).

Section 1.4: Statement of GATHER compliance

This study complies with GATHER recommendations. We have documented the steps in our analytical procedures and detailed the data sources used. See table 3for the GATHER checklist. The GATHER recommendations can be found at the GATHER website under GATHER Statement.

Section 1.5 GBD results overview

Results from GBD 2021 are available through an interactive data downloading tool on the Global Health Data Exchange (GHDx). The GHDx is the world's most comprehensive catalogue of surveys, censuses, vital statistics, and other health-related data. Results are measured in terabytes.

The latest version of the data download tool, available here: http://ghdx.healthdata.org/GBD-results-tool, contains core summary results for GBD 2021. These results include deaths, years of life lost (YLLs), YLDs, disability-adjusted life-years (DALYs), prevalence, incidence, and rate of change. The GHDx includes data for causes, risks, cause-risk attribution, aetiologies, and impairments.

Data above a certain size cannot be viewed online but can be downloaded. Depending on the size of the download, users may need to enter an email address; a download location will be sent to them when the files are prepared.

All GBD 2021 online data visualisations are available at http://www.healthdata.org/gbd/data-visualizations, which provides results for all GBD health metrics.

Section 1.6 Data input sources overview

GBD 2021 synthesises a large and growing number of data input sources including surveys, censuses, vital statistics, and other health-related data sources. The data from these sources are used to estimate morbidity; illness, and injury; and attributable risk for 204 countries and territories from 1990 to 2021; mortality deaths are estimated from 1980 to 2021. The input sources are accessible through an interactive citation tool available in the GHDx.

Citations for specific GBD components, causes and risks, and locations can be found through the Data Input Sources Tool in GHDx: http://ghdx.healthdata.org/gbd/2020/data-input-sources. This tool allows users to view and access GHDx records for input sources and export a comma-separated value (CSV) file that includes metadata, citations, and information about where the data were used in GBD. As required by GATHER, additional metadata for input sources are available through the citation tool as well.

Section 1.7 Funding sources

This publication and the research it presents was funded by the Bill & Melinda Gates Foundation; Queensland Department of Health, Australia; the National Health and Medical Research Council, Australia; Public Health England; the Norwegian Institute of Public Health; St. Jude Children's Research Hospital; the Cardiovascular Medical Research and Education Fund; the National Institute on Ageing of the National Institutes of Health (award P30AG047845); and the National Institute of Mental Health of the National Institutes of Health (award R01MH110163). The funders of the study had no role in study design, data collection, data analysis, data interpretation, or writing of the report. All authors had full access to all data in the study and had final responsibility for the decision to submit for publication.

Section 1.8: Abbreviations

ARC – annualized rate of change ASFR- age-specific fertility rate
ACMR all-cause mortality rate

BMI Body Mass Index
CMNN Communicable, maternal, neonatal, and nutritional diseases
CoD causes of death
CODEm Cause of Death Ensemble modelling
COMO comorbidity correction
COPD Chronic obstructive pulmonary disease
CSMR cause-specific mortality rates
CV coefficient of variation
DALYs disability-adjusted life-years
DisMod-AT disease model-Bayesian age-time
DisMod-MR disease model-Bayesian meta-regression
DW – disability weight
EDU15+ education for those 15 years old and older
EMR excess mortality rate
GATHER Guidelines for Accurate and Transparent Health Estimates Reporting
GBD Global Burden of Diseases, Injuries, and Risk Factors Study
GHDx Global Health Data Exchange
GPR Gaussian process regression
HALE healthy life expectancy
HAT human African trypanosomiasis
ICD- International Classification of Diseases
ICG- ICD groups
IFD in-facility delivery proportion
IHME Institute for Health Metrics and Evaluation
LASSO least absolute shrinkage and selection operator
LDI lag-distributed income
LOESS locally estimated scatterplot smoothing
MAD median absolute deviation
MCCD Medical Certification of Causes of Death
MEPS Medical Expenditure Panel Survey
MICS Multiple Indicators Survey
MR-BRT Meta-regression—Bayesian, regularised, trimmed
NESARC National Epidemiologic Survey on Alcohol and Related Conditions
NSMHWB Australian National Survey of Mental Health and Wellbeing
NTDs – neglected tropical diseases
RSME root mean square error
SARS-CoV 2 Severe acute respiratory syndrome coronavirus 2
SD Standard deviation
SID HCUP State Inpatient Database
SDI Social Demographic Index
ST-GPR spatiotemporal Gaussian process regression
TFR total fertility rate
TFU25 younger than 25 years old (fertility rate)
UI uncertainty interval
UK United Kingdom
UI uncertainty interval
USA United States of America
WHO World Health Organization

YLDs years lived with disability
YLLs years of life lost

Section 2: Non-fatal outcome estimation

The GBD 2021 non-fatal estimation process describes the steps necessary to estimate incidence, prevalence, and YLDs for disease and injury sequelae in GBD 2021. Conceptually, the estimation effort is divided into eight major components: (1) compiling data sources through data identification and extraction; (2) data adjustments; (3) estimation of prevalence and incidence by cause and sequelae by using DisMod-MR 2.1, or alternative modelling strategies for select cause groups; (4) estimation by impairment; (5) severity distributions; (6) incorporation of disability weights (DWs); (7) comorbidity adjustment; and (8) the estimation of YLDs by sequelae and causes. Section 6 contains additional detail specific to each non-fatal disease, impairment, and injury, and their sequelae. Non-fatal modelling strategies vary significantly between causes.

Section 2.1: Data sources, identification, and extraction

Section 2.1.1: Systematic reviews

For GBD 2021, updated systematic reviews were conducted for 77- causes and risk factors. For other disease sequelae, only a small fraction of the existing data appears in the published literature, and other sources predominate, such as survey data, disease registers, notification data, or hospital inpatient data. As was done in past rounds of GBD, data were systematically screened from household surveys archived in the GHDx (http://ghdx.healthdata.org/), including Demographic and Health Surveys, Multiple Indicator Cluster Surveys (MICS), Living Standards Measurement Surveys, and Reproductive Health Surveys. Other national health surveys were identified on the basis of survey series that had yielded usable data for past rounds of GBD, sources suggested to us by in-country GBD collaborators, and surveys identified in major multinational survey data catalogues such as the International Household Survey Network and the WHO Central Data Catalog, as well as through country Ministry of Health and Central Statistical Office websites. Case notifications reported to the WHO were updated through 2020. Citations for all data sources used for non-fatal estimation in GBD 2021 are provided in searchable form through a web tool (http://ghdx.healthdata.org/). A description of the search terms used for cause-specific systematic reviews are detailed by cause in Section 6

Section 2.1.2: Survey data preparation

For GBD 2021, survey data for which we have access to the unit record data constitute a substantial part of the underlying data used in the estimation process. During extraction, we concentrated on demographic variables (eg, location, sex, age), survey design variables (eg,

sampling strategy and sampling weights), and the variables used to define the population estimate (eg, prevalence or a proportion) and a measure of uncertainty (standard error, confidence interval or sample size, and number of cases).

Section 2.1.3: Disease registries

For GBD 2021 non-fatal estimation, disease registries were an important source for a select number of conditions such as cancers, end-stage renal disease, and congenital disorders. Registry data is particularly key in the estimation of neoplasms when we consider the increasing attention to non-communicable diseases, particularly cancers, in low and middle-income areas of the world. The GHDx source tool (http://ghdx.healthdata.org/data-type/disease-registry) provides a comprehensive list of registry data used in GBD estimation processes.

Section 2.1.4: Case notifications

Case notifications, active screening, intervention coverage studies, and surveillance contributed to estimates of infectious diseases. If data were available, we extracted it from survey and administrative microdata; otherwise, data were extracted from published literature and reports. For many infectious diseases and neglected tropical diseases (NTDs), we used cases for which notification was made by countries to the WHO and other global monitoring entities. The causes for which we used WHO case notification data included tuberculosis, measles, yellow fever, rabies, dengue, cholera, whooping cough, human African trypanosomiasis (HAT), meningitis, all sexually transmitted infections, and other infectious diseases and NTDs, such as Ebola.

Section 2.2: Clinical input data and methods summary

Administrative claims, inpatient hospital, and outpatient data played a key role in the process of estimating many non-fatal causes and injuries in GBD 2021. Data sources were heterogeneous in granularity, comprehensiveness, and level of detail, and the methods described below were used to transform data to be comparable and complete across locations, ages, sexes, years, and causes.

Section 2.2.1: Mapping diagnoses to GBD diseases and injuries

Most clinical sources are coded using the International Classification of Diseases (ICD) system that we map to GBD-defined diagnosis groups. ICD-9 and ICD-10 codes are mapped to what are termed “ICD code groups” (ICGs) with a many-to-one relationship, which simplifies the disease categorization and reduces complexity. ICGs are then mapped to a disease or injury modelling entity used by GBD modelers.

Some ICD codes are not mapped to a clinical modelling entity as some causes in the GBD cause hierarchy do not use clinical data sources. These ICD codes are still included in the sum of all admissions for that location. We also designate whether each modelling entity is processed in terms of incidence or prevalence, depending on the nature of the disease and the expected pattern of treatment. Table 12 shows the ICD codes used for non-fatal modelling by GBD cause and injury.

Figure 1. GBD 2021 Claims Data Processing

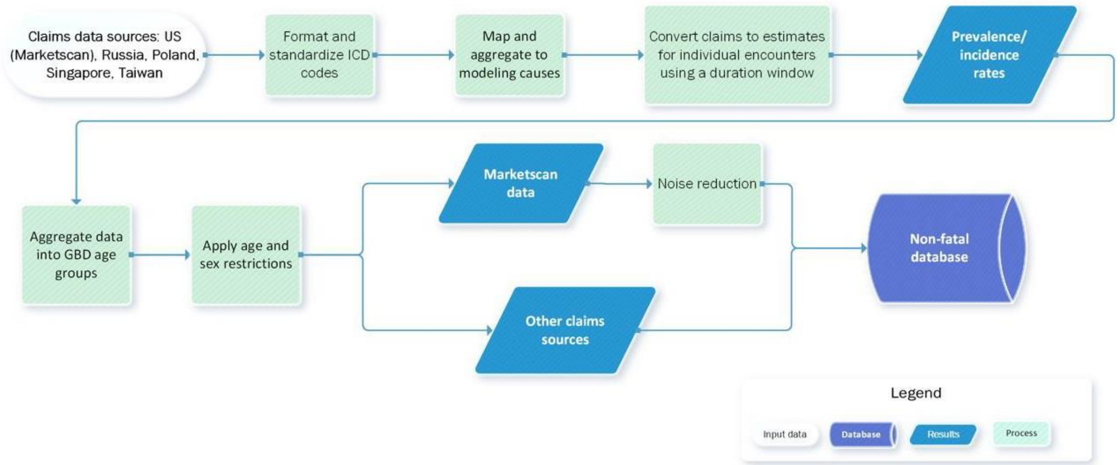

Marketscan claims

For GBD 2021, we accessed aggregate data derived from the Merative database of USA private health insurance and Medicare private supplemental insurance for the years 2000 and 2010-2017. The population covered in each year was 3.3 million in 2000, 40.4 million in 2010, 44.4 million in 2011, 40.8 million in 2012, 42.2 million in 2013, 36.4 million in 2014, 22.6 million in 2015, 22.4 million in 2016, and 20.8 million in 2017. For each of these individuals, claims representing every health service encounter were used and all episodes of care were linked to individuals by unique identifiers. For the GBD, we subset the population in the Marketscan database to individuals with a full year of insurance coverage or those who were born or died in the year of interest in order to ensure the sample includes all healthcare utilization for a given individual in that year.

We mapped ICD diagnoses in each source to GBD causes and injuries. GBD conditions are processed as "prevalence" or "incidence" based on the specification of the research team responsible for the cause. Prevalent conditions are identified as any primary or non-primary diagnosis on any inpatient or outpatient claim within the year of interest. To reduce noise from spurious coding practices, a minimum of two outpatient claims for the same individual are required in a calendar year to count as a prevalent case. Incidence of disease or injury was

calculated based on a duration window which varied by cause. Any individual who had multiple diagnoses for the same cause within the duration window are counted as a single incident case, and additional diagnoses outside of the duration window are treated as new incident cases.

After mapping to cause and identifying prevalent and incident cases by cause, we applied a noise reduction model to smooth trends over age and time.

Other claims data
Claims data from Poland, Singapore, and Taiwan (province of China) were also processed for GBD 2021. Anonymized, individual-level claims data from Poland were accessed through an existing collaboration and institutional partnership with the Agency for Health Technology and Tariff System (AOTMiT). The data is derived from the National Health Fund (Narodowy Fundusz Zdrowia) database in Poland and is representative of every service encounter and episode of care in the public health care system (close to 92% population coverage) from 2015 to 2018.

Tabulated inpatient-only claims data from Singapore for the years 1991-2017 were derived from the MediClaim database and provided by the Ministry of Health of Singapore. The MediClaim data processed for the GBD is inclusive of all inpatient admissions in the country's public and private hospital facilities, and for all patients covered under MediShield Life, MediSafe, and MediFund, with admissions aggregated at national level. Similarly, Taiwan (province of China) claims for the year 2016, derived from the National Health Insurance Research Database (NHIRD) and covering all residents in Taiwan under a universal single-payer health care system, was used. The NHIRD is representative of the whole population for Taiwan and covers both inpatient admissions and outpatient encounters.

Section 2.2.3: Inpatient hospital admissions

Figure 2. GBD 2021 Inpatient Hospital Data Processing

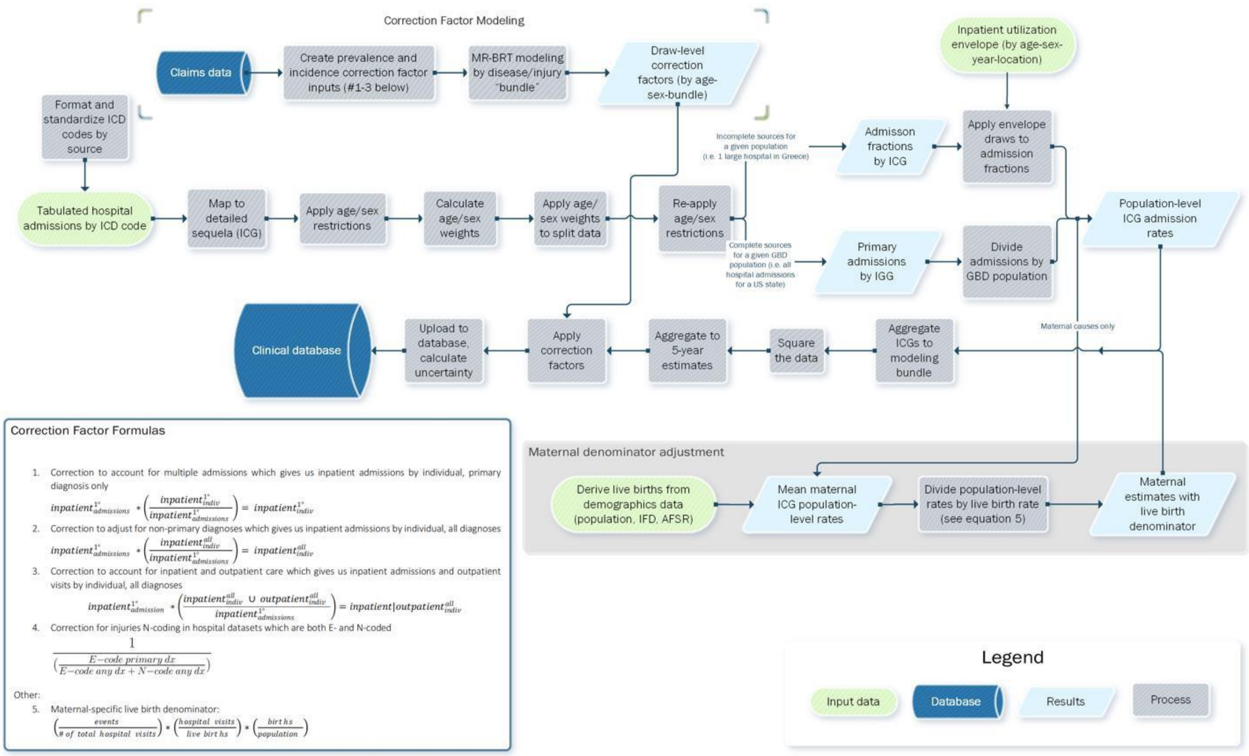

Age-sex splitting and processing individual-level data

Inpatient hospital data were extracted from 4,722 location-years in 49 countries. ICD coding was standardized across sources and versions of ICD. Counts of admissions with a primary diagnosis of each cause were extracted from all sources and stepped through the inpatient data processing methods. For inpatient data, a case of disease was defined as an overnight inpatient admission with a primary diagnosis of that cause. We tabulated the incident or prevalent admissions for each source according to the disease or injury. Admissions were then aggregated to create cause fractions, defined as the number of admissions for a given disease/injury divided by total admissions for that age, sex, and year. Secondary diagnostic detail was included in estimation through corrections as described below.

In GBD 2021, 13 inpatient sources with high percentages of live birth diagnosis codes (i.e. Z37.0) in the 0-6 day age group were either removed or swapped from the primary diagnosis position for the subsequent diagnosis in sources with multiple diagnoses.

Deriving population-level estimation

Section 2.2.5 of the appendix describes the modelling process for the inpatient utilisation envelope, an estimate of inpatient admissions per capita for all GBD locations, years, ages, and both sexes. Inpatient sources were assessed for whether or not they capture a complete and representative GBD population, meaning that we would expect all hospital admissions for a given location and year to be present in the data source. Sources that meet this criteria did not

use the inpatient utilisation envelope to derive population-level estimations and used GBD population estimates instead. Changes from GBD 2019 to GBD 2021 in the estimation of the inpatient utilisation envelope are outlined in the dedicated section below.

Corrections

We performed three adjustments on inpatient hospital data to synthesize all inpatient sources to the same definition of care and to account for cases that were not captured in inpatient sources. Data were first adjusted to account for multiple admissions for a single case of disease and then adjusted to account for cases of any disease that were non-primary diagnoses recorded for an admission. Finally, admissions were scaled by the ratio of outpatient cases observed for any inpatient case of disease to account for additional cases that did not warrant an inpatient admission. Combined with the uncorrected incidence and prevalence rates from the inpatient sources (with no scalar applied), this process resulted in four versions of inpatient estimates: (1) un-corrected inpatient admissions by episode, primary diagnosis; (2) inpatient admissions by individual, primary diagnosis only; (3) inpatient hospital admissions, accounting for all diagnoses; and (4) an estimate of inpatient admissions and outpatient visits by individual, accounting for all diagnoses. Estimate 4 was applied to all causes except those where outpatient care or non-primary diagnosis were not used in the modeling strategy given the nature of the disease. Adjustment ratios were calculated using all clinical sources that had patient-level data and primary and non-primary diagnoses.

Sources of this data include Marketscan and Taiwan claims data as described above; claims and inpatient data from Poland, the Philippines, New Zealand, and the HCUP State Inpatient Database (SID). Only Marketscan, Poland, and Taiwan claims data included a link between inpatient and outpatient care to be used in the fourth estimate described. Ratios from these sources were modelled over age and sex using a mixed-effects model in MR-BRT for each cause. If data for any ratio did not exist for the youngest or oldest age groups, we assumed a uniform tail on the model from the nearest age group with data. All models were conducted in log-space in order to bound the model to be greater than one for any age, sex, and cause. We used the following equations for each of the three scalars:

1) Correction to account for multiple admissions, which gives us inpatient admissions by individual, primary diagnosis only
    a. $inpatient^{1°}_{admin} * \left(\frac{inpatient^{1°}_{indiv}}{inpatient^{1°}_{admin}}\right) = inpatient^{1°}_{indiv}$
2) Correction to adjust for non-primary diagnoses, which gives us inpatient admissions by individual, all diagnoses
    a. $inpatient^{1°}_{admin} * \left(\frac{inpatient^{all}_{indiv}}{inpatient^{1°}_{admin}}\right) = inpatient^{all}_{indiv}$

3) Correction to account for inpatient and outpatient care, which gives us inpatient admissions and outpatient visits by individual for all diagnoses
    a. $inpatient^{1°}_{admission} * (\frac{inpatient^{all}_{indiv} \cup outpatient^{all}_{indiv}}{inpatient^{1°}_{admissions}}) = inpatient|outpatient^{all}_{indiv}$

Denominators for maternal conditions were adjusted using in-facility delivery proportion (IFD) and age-specific fertility rate (ASFR) covariates to include only those at risk for maternal conditions. After this adjustment, the denominator represents people who gave birth in that year.

Inpatient sources that use the inpatient utilisation envelope:

$$(\frac{condition\ specific\ admissions}{all\ cause\ admissions}) * (\frac{estimated\ envelope\ admissions}{GBD\ pop * (IFD * ASFR)})$$

Inpatient sources that do not use the inpatient utilization envelope:

$$\frac{condition\ specific\ admissions}{(GBD\ pop * birth\ adjustment)}$$

Clinical estimates for injuries use a separate correction factor from those described above, which adjusts sources with an insufficient proportion of ecodes (external causes of injury) among all injuries ICD codes. We aggregate total ecodes, in the primary Dx position, by source-GBD location-year, and divide by ecodes and ncodes (nature of injury codes), in any diagnosis position, for the same demographic. Source-GBD location-years that have a proportion less than .15 are dropped. For example, Japan-Yamanashi-2010 has a proportion of .018, interpreted as 1.8% of injuries codes in that demographic are ecodes, and would be removed.

The injuries correction is created directly from this proportion:

$$\frac{1}{ecode\ primary\ Dx\ admissions/(ecode{+}ncode\ codes, all\ diagnoses)}$$

A final adjustment was applied to each of the above estimates. The HAQ Index was used to account for differences in access and quality of health care across time and space. The HAQ Index adjustment was applied by dividing the above estimates by a scalar ranging from 0 to 100, where 0 represents the first percentile of observed access and quality and 100 the 99th percentile.

Section 2.2.4: Outpatient encounter data

Figure 3. GBD 2021 Outpatient data extraction process

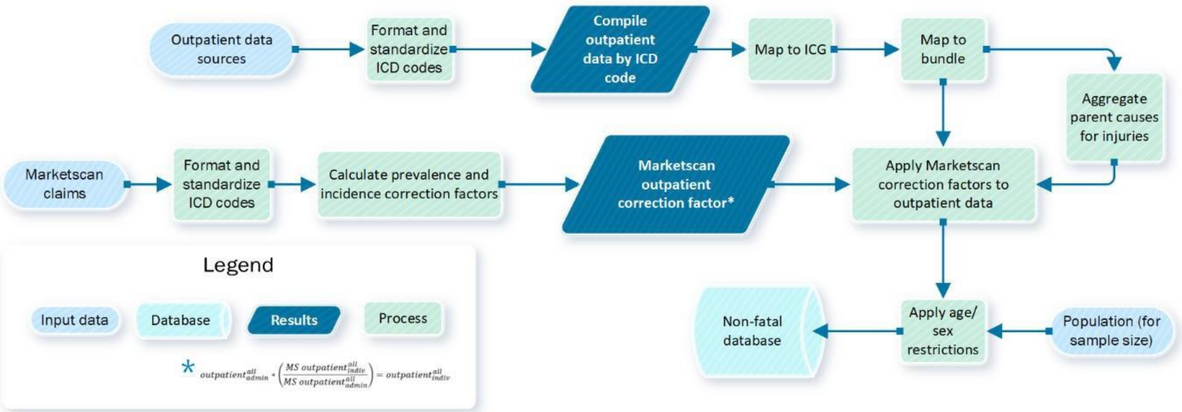

Outpatient encounter data, that could not be linked to inpatient admissions, were processed from the USA and Sweden for 109 location-years. No changes were made in the processing of outpatient data from GBD 2019, except for updates to the ICD mapping.

As with the inpatient hospital data, a scalar was calculated by using Marketscan outpatient claims data to adjust for multiple visits per individual within one year (for prevalent conditions) and within a cause-specific duration (for incident causes).

Calculating uncertainty

Uncertainty in claims estimates was calculated using Wilson's approximation, utilizing sample size derived from enrollment data (i.e. Marketscan) or GBD population estimates (i.e. Poland), depending on the source. Uncertainty in outpatient estimates was also calculated using Wilson's approximation and GBD population. Uncertainty for inpatient sources that are not complete for the population and use the inpatient utilization envelope came from the upper and lower uncertainty intervals of 1000 bootstrapped samples of the envelope and correction factor models. Inpatient sources that are complete for the population derived uncertainty from Wilson's approximation and GBD population.

Wilson's approximation:

$$\sigma^2 = \frac{\frac{cf\,(1-cf)}{n} + \frac{1.96^2}{4n^2}}{\left(1 + \frac{1.96^2}{n}\right)^2}$$

where cf is the cause fraction and n is the sample size.

Section 2.2.5: Estimation of the inpatient utilization envelope

This process utilises administrative data, reported tabulations, and survey microdata to estimate the rates of inpatient admissions per capita for every location and demographic group in the GBD hierarchy.

Figure 4. Overview process of estimation of hospital envelope

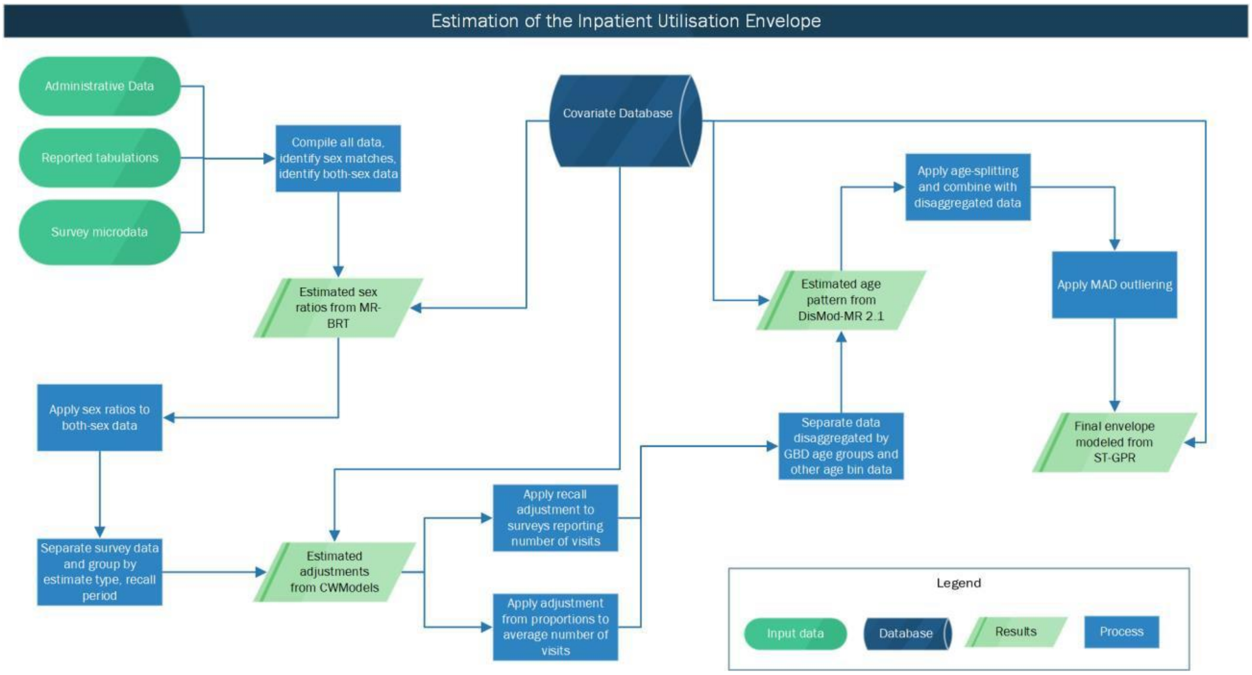

Case definition

We defined a hospital admission as admission into a formal health care facility for, at least, an overnight stay. However, we excluded admissions to long-term care facilities (>120 days), nursing care facilities, and facilities staffed by traditional or spiritual healers.

Input data

We searched the GHDx for population surveys, administrative records, and censuses from January 1990 to September 2019. We applied the following keyword filters: “Health care use” OR “Length of stay” AND “Hospitals” OR “Health care services”. We applied no language restrictions to our search and required all returned records to contain either microdata or tabulated reports. We searched the returned records’ metadata for measures of inpatient care. For inclusion, we required all measures to be nationally or subnationally representative. Additionally, we consulted with experts and GBD collaborators to gather data sources that were not within the GHDx. We included 2064 sources for GBD 2021, adding 400 new sources relative to GBD 2019.

Data processing

From data sources for which microdata were available, we extracted and binned the data based on gender and age groups of 0-11 months, 12-23 months, 2 to 4 years, 5-9 years, 10-14 years, and similar increments of years up to 95 years and older. Data was occasionally binned into wider age groups where less detailed age data was available, or where samples were sufficiently small.

Our input data contained a limited number of both-sex data points. We used the MR-BRT modelling tool (see Section 2.5 for details on MR-BRT) to model the ratio of female to male admissions based on

matched sex-specific data. The results of this model were used to split both-sex data points into sex-specific data. The estimated adjustment factor from the MR-BRT analysis is presented below. This factor can be interpreted as the observed ratio between female and male utilisation.

Table 4. Sex-splitting Adjustment Factor

Data input	Beta coefficient, log (95% UI)	Adjustment factor
Sex	-0.056 (-0.559, 0.449)	0.946

We classified each of the accepted data sources into four data types: (1) proportion of survey respondents who were admitted into the hospital in the last 30 days; (2) proportion of survey respondents who were admitted to the hospital in the last year; (3) average number of admissions (utilisation rate) reported by survey respondents in the last year; and (4) average number of visits reported by annual administrative records. We assigned measures reported by annual administrative records as our reference group because these data types were free from recall bias and most closely matched our case definition.

We crosswalked each of the three non-reference (survey) data types to the reference (administrative record) data type via adjustment factors derived from MR-BRT meta-regressions. For each non-reference data type and each sex, we looked for overlap between the non-reference data type and the reference data type based on location, year, age group, and sex. The MR-BRT analyses were performed between each alternative data type and the reference with a spline on age and the covariates of hospital beds per 1000 and lag-distributed income (LDI) to account for non-systematic differences between the data types.

After crosswalking all non-reference data to the reference data type, we used DisMod-MR 2.1 model with all data disaggregated by age to estimate countries' age-pattern. This age pattern was then applied the estimated age-pattern to split aggregated age data into the most granular age groups that are necessary for ST-GPR. The age pattern used to split aggregated age data is shown below.

Figure 5. Age-pattern used to age-split wide age bin data

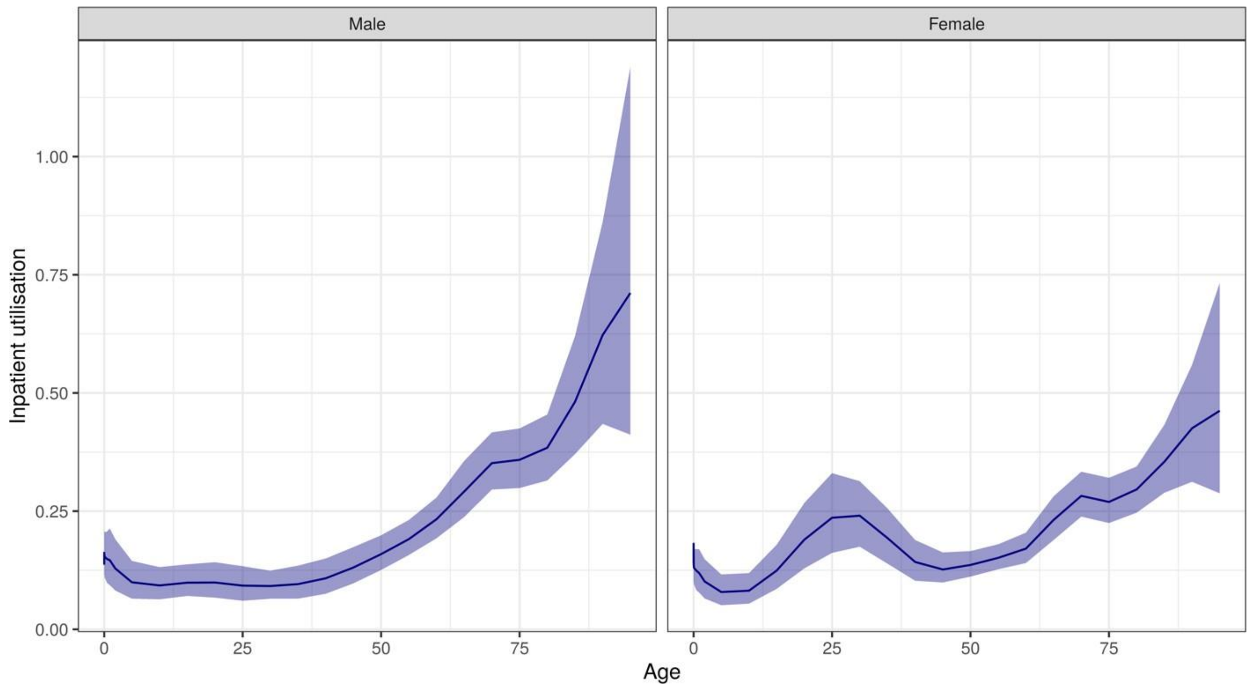

Before modelling, we applied a systematic outliering processes to identify data points that differed substantially from the trend. To do this, we calculated the median absolute deviation (MAD) from the age-standardized mean utilisation for each sex-location-year-source combination. Points that were more than three MADs above or below median utilisation were marked as outliers.

Modelling strategy

The input data were modelled using ST-GPR to allow for smoothing over age, time, and location to produce estimates of utilisation for every age, sex, location, year combination in the GBD. We included three covariates to help explain variation in geographies with little to no data and included random effects on location in the modelling specifications. We used the covariates of the natural log of hospital beds per 1000, natural log of health expenditure per capita, and the HAQ Index for every location. Coefficients for the covariates are presented in the table that follows.

Table 5. Estimated coefficients of the inpatient envelope model.

Covariate	Sex	Coefficient (95% UI)	Exponentiated Coefficient (95% UI)
Log hospital beds per 1000	Male	0.60 (0.57, 0.63)	1.82 (1.77, 1.88)
	Female	0.50 (0.46, 0.53)	1.64 (1.59, 1.70)
HAQ Index	Male	-0.000039 (-0.0012, 0.0012)	1.00 (0.99, 1.00)
	Female	0.00080 (-0.00045, 0.0021)	1.00 (1.00, 1.00)

Log health expenditure per capita	Male	0.21<br>(0.19, 0.23)	1.24<br>(1.21, 1.26)
	Female	0.22<br>(0.20, 0.23)	1.24<br>(1.22, 1.26)

Changes from GBD 2019 to GBD 2021

Relative to GBD 2019, there were a number of changes to the inpatient utilisation envelope modelling strategy. First was the addition of new input data, both from survey series and new years of administrative data. Second, the crosswalk analysis was done using MR-BRT, where it was previously done via penalised spline regressions. Third was the incorporation of the MAD outliering technique, to help systematically identify implausible estimates of utilisation in the input data prior to modelling. Fourth, we no longer used in-facility delivery estimates as input data for the youngest age group, relying instead on input data from administrative sources and surveys. Finally, we no longer used the all-cause mortality covariate in the ST-GPR model. All together, these changes resulted in more robust estimates of inpatient utilisation across GBD demographics.

Section 2.3 Data Adjustments

Section 2.3.1: Crosswalking

Crosswalking refers to the process of adjusting data for known biases. An observation is considered biased if it differs in a consistent way from the standard GBD definition of the modeled parameter. Examples include self-reported rather than doctor-diagnosed measures of disease incidence, or diagnostic tests with a lower sensitivity or specificity compared to the gold standard diagnostic method. If the difference between an alternative measurement method and the GBD definition is consistent and systematic, we can model it as a function of covariates and use this model to predict the degree of adjustment needed for a given alternative or non-standard observation. The result of crosswalking is that GBD models can incorporate data from a wider range of sources.

Specifically, crosswalking involves:

1. Finding pairs of alternative and reference (e.g. self-reported and measured) observations that match on relevant criteria (e.g. age, sex, location and year);
2. Taking the difference between these observations in log or logit space, to ensure that the crosswalk adjustment remains bounded correctly;
3. Running a meta-regression model that estimates this difference potentially as a function of covariates;
4. Predicting how much each alternative data point in the original dataset should be adjusted; and

5. Applying the adjustment.

Section 2.3.2: Bias adjustment for alternative case definitions and study methods

In GBD 2021 we continued the practice started in GBD 2019 of crosswalking non-fatal and risk exposure data to account for alternative case definitions or study methods. The adjustments were applied prior to entering data into our main analytical tools of DisMod-MR 2.1 and ST-GPR, ensuring that all data inputs were expressed on a consistent scale. We also used this approach to convert data presented for both sexes to a male and female equivalent. The starting point was to explicitly state the reference case definition and study method and identify alternative definitions and study characteristics that fall within our inclusion criteria.

We compiled data from both within-study comparisons (ie, data that used alternative and reference definitions in the same population) and between-study comparisons (ie, data that used an alternative definition in one population and a reference definition in another population that overlap in location, time, age, and sex) of different case definitions. For between-study comparisons, we allowed a maximum calendar year difference between studies of five years. Where validation studies (ie, those carried out at the introduction of a new set of diagnostic criteria comparing to previous criteria) were available, we extracted data on the comparison of alternative to reference. For quantities of interest with multiple alternative definitions/methods we also looked for pairs comparing two alternatives.

If both between and within study pairs were available, we examined whether there was a systematic difference between these. If there was a significant difference, we made judgement call as to whether within-study or between study data comparisons were most appropriate. In general, this was the within-study data. However, there were important measurement or conceptual reasons for choosing between-study data. For example, for crosswalks between self-reported height and weight compared to measured height and weight, between-study comparisons may be preferable if respondents knew they would be measured and, therefore, were less likely to misreport their height and weight.

To quantify the degree of bias for an alternative data source, we calculated the difference between matched pairs of alternative and reference observations and used this quantity as the dependent variable in a mixed effect meta-regression model. The model could include any number of covariates to capture how bias might vary as a function of other variables, like age or sex. Predictions from the model were then used to convert alternative observations to their equivalent reference values. For GBD 2021, we developed an open source Python package to facilitate the process of modeling and applying bias adjustments (ihmeuw-msca , 2023)

To choose covariates for the model, we examined whether there were systematic differences in the adjustments by key demographics (age, sex, geographic location, year) and other potential factors that may lead to variation in the degree of bias adjustment. We did this when there was a strong rationale, eg, biological plausibility, for variation by such characteristics. After fitting

the model, for predicted adjustment factors that were not statistically significant, we still applied the adjustments if there was a conceptual reason to believe that the alternative definition is biased. This expands the variance of data points using a non-standard case definition or study method, effectively reducing their influence in subsequent modeling steps.

Section 2.3.3: Example bias adjustment calculation

As an example, we provide mathematical notation for a bias adjustment to a data source that measures prevalence using a non-standard case definition. We have pairs of alternative and reference observations (denoted i) that match on age, sex, location, and time period combination (denoted j). The degree of bias varies as a function of age and sex. Because the parameter of interest is prevalence, which is bounded by 0 and 1, we calculate the logit-scale difference between alternative and reference observations in a given matched pair:

$$y_{i,j} = logit(p^{alt}_{i,j}) - logit(p^{ref}_{i,j}).$$

In preparing the data for this calculation, if the values of either the reference or alternative were zero, we aggregated values across age groups until both values had non-zero observations. We used the delta method to compute the standard error of the reference and alternative measures in logit space. The standard error of the logit-scale difference was computed as the square root of the sum of the variances of each data point in a pair.

If the parameter had instead been bounded by only 0, like incidence, we would have calculated the log-scale difference. From simulations we found that the two methods provide almost identical results for quantities that after adjustment do not exceed a value of 0.5 (eg, prevalence or proportion). The logit-scale difference method much better dealt with higher values and avoided prevalence or proportions to exceed one.

As a next step in this hypothetical example, we modeled the differences as the dependent variable in a mixed-effects meta-regression model with age and sex as covariates:

$$y_{i,j} = \beta_0 + \beta_1 \; age_i + \beta_2 \; sex_i + u_j + \epsilon_i$$
$$u_j \sim N(0, \gamma)$$
$$\epsilon_i \sim N(0, \sigma_i^2)$$

We then used the linear predictor of this model to predict the degree of bias adjustment needed for the various age and sex combinations among the alternative observations:

$$\hat{\iota}_{a,s} = f(age, sex) = \beta_0 + \beta_1 \, age + \beta_2 \, sex.$$

To adjust a particular alternative observation $p^{alt}_{a,s}$ we subtracted the adjustment factor in logit space, and the inverse logit transformation was applied to the result to convert back to natural units:

$$p^{adjusted}_{a,s} = logit^{-1}(logit(p^{alt}_{a,s}) - \delta_{a,s}).$$

The uncertainty for the adjusted logit-scale prevalence includes:

- uncertainty of the original observation in logit space,
- uncertainty from the posterior distribution of the predicted adjustment, and
- random intercepts in the meta-regression model (denoted γ above).

The variances from the three components were summed and then transformed into natural unit space using the delta method.

Section 2.3.4 Network Analysis

When there were multiple alternative case definitions or study methods, we used network analysis to leverage the additional information provided by indirect comparisons. For example, if A is the reference and B and C are two alternatives, the comparison of C versus A would be considered a direct comparison to the reference. This case was the subject of the previous section. In contrast, the combination of A versus B and B versus C provides an indirect comparison of the alternative C against the reference A. Or in other words, the inclusion of B-versus-C comparisons in the dataset provides additional information with which to estimate the difference between C and A.

Implementing a network analysis requires careful construction of the design matrix, or the dataset we pass to the mixed effects meta-regression model. Continuing the example with reference A and alternatives B and C, the design matrix for a network analysis with no covariates is created as follows:

- Create *k* dummy variables where *k* are all definitions/methods other than A (eg, *k* = B, C)
- Code dummy *k* as
    - 1 if the first term of the logit-scale difference is *k*;
    - -1 if *k* is second term of the logit-scale difference;
    - 0 otherwise

For example:

Study	Comparison	DummyB	DummyC
1	logit(B)-logit(A)	1	0
2	logit(B)-logit(A)	1	0
3	logit(C)-logit(A)	0	1
4	logit(C)-logit(A)	0	1
5	logit(C)-logit(B)	-1	1
6	logit(C)-logit(B)	-1	1

The coding structure outlined above assumes that all case definitions are mutually exclusive. In some cases, however, individual case definitions are composed of different sub-components or dimensions. For example, case definitions may vary by the type of symptoms that a respondent experiences as well as the recall period over which those symptoms are experienced. In the presence of sparse data, it may be difficult to find both direct and indirect comparisons of all individual case definitions. In these cases, an alternative approach is to assume different dimensions of case definitions have a multiplicative effect. In other words, the effect of recall period has the same relative effect across different categories of symptoms reported by respondents. To implement this coding scheme:

- Create *k* dummy variable columns for each case definition dimension.
- For each dummy variable k:
    - Add 1 if *k* is a component of the first term in the logit-scale difference.
    - Subtract 1 if *k* is a component of the second term in the logit-scale difference.

Network analysis is a feature of the open source Python package for conducting bias adjustments (ihmeuw-msca , 2023) mentioned earlier. The package abstracts away the need to create the design matrix manually as in this example and can incorporate an arbitrary number of alternative definitions and covariates.

Section 2.3.5 Age sex splitting

Before modelling, we ran a Dismod-MR 2.1 model with data disaggregated by age to estimate countries' age-pattern and then applied the estimated age-pattern to split aggregated all-age data into the 5-year age groups preferred for ST-GPR modelling. This procedure was done by calculating a constant, k, which was the ratio of the aggregated all-age data point, $\mu_{all\ age}$, to the all-age estimated utilisation rate from the DisMod-MR 2.1 model, $\hat{\mu}$

$$k = \frac{\mu_{all\ age}}{\hat{\mu}}$$

The constant, k, was then multiplied by age-specific utilisation rates from the DisMod-MR 2.1 model. Observation-specific uncertainty and uncertainty from the estimated age-pattern were both propagated into the uncertainty for a given post-splitting data point. The split data were then incorporated into the final DisMod-MR 2.1 model.

Section 2.4: Spatiotemporal Gaussian process regression (ST-GPR) modelling

The input data were modelled by using ST-GPR to allow for smoothing over age, time, and location in locations that were missing complete datasets.

The flowchart showing the analytic steps can be found elsewhere (Collaborators, 2020) The approach is a stochastic modelling technique that is designed to detect signals amidst noisy data. It also serves as a powerful tool for interpolating non-linear trends (Vasudevan S, 2009) (CE, 2005). Unlike classical linear models that assume that the trend underlying data follows a definitive functional form, GPR assumes that the specific trend of interest follows a Gaussian process, which is defined by a mean function $m(\cdot)$ and a covariance function $Cov(\cdot)$. For example, let $p_{c,a,s,t}$ be the prevalence, in normal, log, or logit space, observed in country c, for age group a, and sex s at time t:

$$(p_{c,a,s,t}) = g_{c,a,s}(t) + \epsilon_{c,a,s,t}$$

where

$$\epsilon_{c,a,s,t} \sim Normal(0, \sigma_p^2),$$

$$g_{c,a,s}(t) \sim GP\ (m_{c,a,s}(t), Cov\ (g_{c,a,s}(t))).$$

The derivation of the mean and covariance functions, $m_{c,a,s}(t)$ and $Cov\ (g_{c,a,s}(t))$, along with a more detailed description of the error variance (σ_p^2), is described below.

Section 2.4.1 Estimating mean functions

We estimated mean functions by using a two-step approach. To be more specific, $m_{c,a,s}(t)$ can be expressed, depending on the prevalence transformation, as:

$$log(p_{c,a,s}(t)) = X_{c,a,s}\beta + h(r_{c,a,s,t})$$

$$logit(p_{c,a,s}(t)) = X_{c,a,s}\beta + h(r_{c,a,s,t})$$

$$p_{c,a,s}(t) = X_{c,a,s}\beta + h(r_{c,a,s,t})$$

where $X\beta$ is the summation of the components of a hierarchical mixed-effects linear regression, including the intercept and the product of covariates with their corresponding fixed-effect coefficients. Some models were run as hierarchical mixed-effects linear regressions with random effects on the levels of the location hierarchy. For most mixed-effects models, random effects were only used in the fit, not in the prediction. The second part of the equation, $h(r_{c,a,s,t})$, is a smoothing function for the residuals, $r_{c,a,s,t}$, derived from the linear model.[44] Cause-specific methods details can be found in appendix sections 6.

Although the linear component captures general trends over time, much of the data variability may still not be adequately accounted for. To address this, we fit a locally weighted polynomial regression (locally estimated scatterplot smoothing, or LOESS) function $h(r_{c,a,s,t})$ to systematically estimate this residual variability by borrowing strength across time, age, and space patterns (the spatiotemporal component of ST-GPR) (Ng M, 2014) (Ng M) The time adjustment parameter, defined by λ , aims to borrow strength from neighboring time points (ie, the prevalence in this year is highly correlated with prevalence in the previous year but less so

further back in time). The age-adjustment parameter, defined by ω, borrows strength from data in neighboring age groups. The space-adjustment parameter, defined by ξ, aims to borrow strength across the hierarchy of geographical locations. The spatial and temporal weights are combined into a single space-time weight to allow the amount of spatial weight given to a particular point $r_{c,a,s,t}$ to fluctuate given the data availability at each time *t* and location-level *l* in the location hierarchy.

Let $w_{c,a,s,t}$ be the final weight assigned to observation $r_{c,a,s,t}$ with reference to a focal observation r_{c_0,a_0,s_0,t_0}. We first generated a temporal weight $t.w_{c,a,s,t}$ for smoothing over time, which was based on the scaled distance along the time dimension of the two observations (Ng M) :

$$t.w_{c,a,s,t} = \frac{1}{e^{\lambda|t-t_0|}}$$

Next, we generated a spatial weight to smooth over geography. Specifically, we defined a geospatial relationship by categorizing data based on the GBD location hierarchy (table 1). *zeta* acts as a scalar on a given datapoint given its proximity to the target location:

$$t.w_{c,a,s,t} = \zeta^{|c-c_0|}$$

For example, estimating a country, would use the following weighting scheme:

- Country data: $\zeta^0 = 1$
- Regional data not from the country being estimated: ζ^1
- Data from other regions in the same super region: ξ^2
- Global data from other super regions: ζ^3

Under the spatial weighting specification, typical values of ζ range from [0.001, 0.2], where ζ can be interpreted as the amount to downweight regional datapoints compared to country datapoints for a given estimating country. For example, for a given datapoint $r_{c,a,s,t}$ and ζ = 0.01, a datapoint not within country *c* but within the same region *r* as $r_{c,a,s,t}$ would be assigned $\frac{1}{100}$ the weight of a datapoint within the country.

The spatial and temporal weights were then multiplied and summed across each level of the location hierarchy and normalised for each time period *t* . This procedure allowed the space-time weight to implicitly take into account the amount of data available at the country vs. region vs. super-region level and attribute spatial weight accordingly.

Given a normalisation constant,

$$K_i = \sum_{c\epsilon C} s.w_{c,t} * t.w_{c.t} + \sum_{c\epsilon R} s.w_{c,t} * t.w_{c.t} + \sum_{c\epsilon SR} s.w_{c,t} * t.w_{c.t}$$

the final space-time weight would then equal

$$w'_{c,a,s,t} = \frac{s.w_{c,t} * t.w_{c,t}}{K_i}$$

Finally, we calculated the weight $w''_{c,a,s,t}$ to smooth over age, which is based on a distance along the age dimension of two observations. For a point between the age a of the observation $r_{c,a,s,t}$ and a focal observation r_{c_0,a_0,s_0,t_0}, the weight is defined as follows:

$$w''_{c,a,s,t} = \frac{1}{e^{\omega|a-a_0|}}$$

The final weights were then computed by simply multiplying the space-time weights and age weights and normalising so all weights for a given time period *t* sum to 1. A full derivation of weights for each category, assuming the location being estimated was a country, follows:

1) If the observation $r_{c,t}$ belongs to the same country c_0 of the focal observation r_{c_0,t_0}:

$$w_{c,a,s,t} = \frac{(w'_{c,a,s,t} \; w''_{c,a,s,t})}{\sum_{c=c_0} (w_{c,a,s,t} \; w_{c,a,s,t})} \qquad \forall c = c_0$$

2) If the observation $r_{c,t}$ belongs to a different country than the focal observation r_{c_0,t_0}, but both belong to the same region *R*:

$$w_{c,a,s,t} = \frac{(w'_{c,a,s,t} \; w''_{c,a,s,t})}{\sum_{c\neq c_0} (w_{c,a,s,t} \; w_{c,a,s,t})} \qquad \forall c \neq c_0 \cap R[c] = R[c_0]$$

3) If the observation $r_{c,t}$ belongs to the same super region *SR* but to both a different country c_0 and a different region $R[c_0]$ than the focal observation r_{c_0,t_0}:

$$w_{c,a,s,t} = \frac{(w'_{c,a,s,t} \; w''_{c,a,s,t})}{\sum_{c\neq c_0} (w_{c,a,s,t} \; w_{c,a,s,t})} \qquad \forall c \neq c_0 \cap R[c] \neq R[c_0] \cap SR[c] = SR[c_0]$$

4) If the observation $r_{c,t}$ is from a different super region than the focal observation r_{c_0,t_0} (Ie, all other data currently not receiving a weight):

$$w_{c,a,s,t} = \frac{(w'_{c,a,s,t} \; w''_{c,a,s,t})}{\sum_{c\neq c_0} (w_{c,a,s,t} \; w_{c,a,s,t})} \qquad \forall c \neq c_0 \cap R[c] \neq R[c_0] \cap SR[c] \neq SR[c_0]$$

Observations could be downweighted by a factor of 0.1, usually because they were not geographically representative at the unit of estimation. Details of reasons for downweighting can be found in cause-specific modeling summaries. The final weights were then normalised such that the sum of weights across age, time, and geographic hierarchy for a reference group was 1.

Section 2.4.1: Estimating error variance

σ_p^2 represents the error variance in normal or transformed space including the sampling variance of the estimates and prediction error from any crosswalks performed. First, variance was systematically imputed if the data extraction did not include any measure of uncertainty. When some sample sizes for data were available, missing sample sizes were imputed as the 5th percentile of available sample sizes. Missing variances were then calculated as $\sigma_p^2 = \frac{p*(1-p)}{n}$ for proportions or were predicted from the mean by using a regression for continuous values. When sample sizes were entirely missing and could not be imputed, the 95th percentile of available variances at the most granular geographic level (ie, first country, then region, etc.) were used to impute missing variances. For proportions where *p*n* or *(1-p)*n* is <20, variance was replaced by using the Wilson Interval Score method.

Next, if prevalence was modelled as a log transformation, the error variance was transformed into log-space by using the delta method approximation as follows:

$$\sigma_p^2 \cong \frac{\sigma_{p'}^2}{p_{c,a,s,t}^2}$$

where $\sigma_{p'}^2$ represents the error variance in normal space. If prevalence was modelled as a logit transformation, the error variance was transformed into logit-space by using the delta method approximation as follows:

$$\sigma_p^2 \cong \frac{\sigma_{p'}^2}{(p_{c,a,s,t} * (1 - p_{c,a,s,t}))^2}$$

Finally, prior to GPR, an approximation of non-sampling variance was added to the error variance. Calculations of non-sampling variance were done on normal-space variances. Non-sampling variance was calculated as the variance of inverse-variance weighted residuals from the space-time estimate at a given location-level hierarchy. If there were <10 data points at a given level of the location hierarchy, the non-sampling variance was replaced with that of the next highest geography level with >10 data points.

Section 2.4.2: Estimating the covariance function

The final input into GPR is the covariance function, which defines the shape and distribution of the trends. Here, we have chosen the Matern-Euclidian covariance function, which offers the flexibility to model a wide spectrum of trends with varying degrees of smoothness. The function is defined as follows:

$$M(t, t') = \sigma^2 \frac{2^{1-v}}{\Gamma(v)} \left(\frac{d(t, t')\sqrt{2v}}{l} \right)^v K_v \left(\frac{d(t, t')\sqrt{2v}}{l} \right)$$

where $d(\cdot)$ is a distance function; σ^2, v, l, and K_v are hyperparameters of the covariance function—specifically σ^2 is the marginal variance, v is the smoothness parameter that defines the differentiability of the function, l is the length scale, which roughly defines the distance between which two points become uncorrelated, and K_v is the Bessel function. We approximated σ^2 by taking the normalised median absolute deviation $MADN(r_c')$ of the difference, which is the normalised absolute deviation of the difference of the first-stage linear regression estimate from the second-stage spatiotemporal smoothing step for each country. We then took the mean of these country-level MADN estimates for all countries with 10+ country-years of data to ensure that differences between first- and second-stage estimates had sufficient data to truly convey meaningful information on model uncertainty. We used the parameter specification $v = 2$ for all models. The scale parameter l used for each cause is reported in appendix sections 3.4 and 4.12.

Section 2.4.3: Prediction using GPR

We integrated over $g_{c,t}(t_*)$ to predict a full time series for country c, age a, sex s, and prediction time t_* as follows:

$$p_{c,a,s}(t_*) \sim N\left(m_{c,a,s,t}(t_*), \sigma_p^2 I + Cov\left(g_{c,a,s,t}(t_*)\right)\right)$$

Random draws of 1000 samples were obtained from the distributions above for every country for a given indicator. The final estimated mean for each country was the mean of the draws. In addition, 95% UIs were calculated by taking the 2.5 and 97.5 percentile of the sample distribution. The linear modelling process was implemented by using the lmer4 package in R, and the ST-GPR analysis was implemented through the PyMC2 package in Python.

Section 2.4.4: Subnational scaling and aggregation

To ensure internal consistency of the estimates between countries and their respective subnational locations, national estimates were either created by population-weighted aggregation or subnational estimates were adjusted by population-weighted scaling to the national estimates, depending on the data coverage of a given country compared to that of its subnational locations. For example, if data coverage was better at the national level than at its corresponding subnational locations for a given country and cause across age, sex, and time, estimates were rescaled to be consistent with the national level. Conversely, if data coverage was better at the subnational level, estimates for its parent country were generated through population-weighted aggregation of subnational estimates.

Estimates can also be scaled within logit space. Scaling in logit space ensures that subnational estimates of proportion models do not exceed one after being rescaled to the national estimate.

Section 2.5: MR-BRT meta-regression modelling

Section 2.5.1 MR-BRT Overview

MR-BRT is a meta-regression modeling tool developed at IHME. In contrast to other types of regression, meta-regression incorporates uncertainty in the dependent variable; each observation comes with its own standard error. This characteristic is important when the input data are results of scientific studies that are reported with uncertainty. Observations with greater uncertainty are given less weight in the model. To describe variation in the parameter of interest, MR-BRT can incorporate both fixed and random effects. Fixed effects include binary and continuous covariates as in a traditional regression model. Random effects describe group-level variation and are often used to characterize differences between studies beyond what is captured by measured covariates.

Section 2.5.2 MR-BRT Formula

Formally, a linear mixed effects meta-regression as implemented in MR-BRT can be described as:

$$y_{ij} = \beta_0 + \beta_1 x_1 + \cdots + \beta_n x_n + u_j + \epsilon_{ij}.$$

The variable y_{ij} refers to the value of observation i in study j; it is typically expressed in log or logit space to ensure that model predictions remain within logical constraints, for example that relative risks cannot be negative. The terms $\beta_0 + \beta_1 x_1 + \cdots + \beta_n x_n$comprise the linear predictor, including both the intercept and the effects of any number of covariates. The term u_j is a random intercept corresponding to study j. The full set of random intercepts is assumed to follow a Normal distribution where γ is the variance of between-study heterogeneity. Random

effects can be estimated for continuous covariates as well, in which case they are called random slopes. The term ϵ_{ij} refers to the stochastic error corresponding to observation i in study j, and the set of values are assumed to follow a Normal distribution in which observation-specific standard errors are known prior to modeling. This linear mixed effects formulation of the model covers most features MR-BRT.Features that involve nonlinear optimization techniques like the ratio model (described below) extend this framework and are described formally elsewhere (Zheng, 2021).

Section 2.5.3 MR-BRT Features

MR-BRT – as suggested by its full name "Meta-Regression with Bayesian priors, Regularization and Trimming" – comes equipped with several capabilities that expand upon the classical mixed effects meta-regression model:

- Bayesian priors can be applied to any estimated coefficient, enabling information from outside the dataset to be considered in the process of fitting the model. A Uniform prior sets hard bounds on the allowed values of an estimated coefficient. A Gaussian prior acts as a suggestion for the estimated value of a coefficient, with the standard deviation of the specified Gaussian distribution determining the strength of the prior.
- LASSO variable selection, also known as L1 regularization, can be implemented by specifying Laplace priors with mean 0 on the β coefficients. Similarly, ridge regression, also known as L2 regularization, can be implemented by specifying Gaussian priors with mean 0 on the β coefficients.
- Trimming is a method for identifying and removing the effects of outliers. Users define the proportion of points to be excluded and the algorithm determines which ones to exclude. Because the trimming algorithm is an integrated part of the model's likelihood function, MR-BRT identifies outliers and estimates the β coefficients simultaneously during the fitting process.
- A spline term may be used to describe the nonlinear effect of a covariate. MR-BRT implements a B-spline, or basis spline. Users have control over the flexibility of the estimated curve by specifying the number of knots, location of knots, spline degree (i.e. cubic or quadratic), linearity in the tail segments, convexity, concavity, or a monotonicity constraint requiring the spline to be non-decreasing or non-increasing.
- Pairs of exposure intervals may be used as an independent variable using a method known as the "ratio model". This feature is most often used when the epidemiological literature reports relative risks corresponding to a reference exposure range (e.g. BMI = [18,22)) and an alternative exposure range (e.g. BMI = [30,35)). It is usually used in conjunction with a spline to capture the nonlinear effect of the exposure. The ratio model works by integrating over the span of each interval and taking the ratio as part of the likelihood function (Zheng, 2021).

The source code for MR-BRT is publicly available on GitHub as the Python package mrtool (ihmeuw-msca , 2023) The mrtool package builds upon the open source mixed effects package LimeTr (https://github.com/zhengp0/limetr). For a full technical description of MR-BRT and the underlying mathematics (Zheng, 2021)

Section 2.6: DisMod-MR 2.1 estimation

Section 2.6.1: Estimation of sequelae and causes

The most extensively used estimation method is the Bayesian meta-regression method DisMod-MR 2.1. For some causes, such as HIV/AIDS or measles, disease-specific natural history models have been used for which the underlying three-state model in DisMod-MR 2.1 (susceptible, cases, dead) is insufficient to capture the complexity of a disease process. For some diseases with a range of sequelae differentiated by severity, such as COPD or diabetes mellitus, DisMod-MR 2.1 was used to meta-analyse the data on overall prevalence with separate DisMod-MR 2.1 models of the proportions of cases with different severity levels or sequelae. Likewise, DisMod-MR 2.1 was used to meta-analyse data on the proportions of liver cancer and cirrhosis due to underlying aetiologies such as hepatitis B, hepatitis C, and alcohol use disorders.

Section 2.6.2: DisMod-MR 2.1 description

Until GBD 2010, non-fatal estimates in burden of disease assessments were based on a single data source on prevalence, incidence, remission, or a mortality risk selected by the researcher as most relevant to a particular location and time. For GBD 2010, we set a more ambitious goal: to evaluate all available information on a disease that passes a minimum quality standard. That required a different analytical tool that would be able to pool disparate information presented for varying age groupings and from data sources by using different case definitions. The DisMod-MR 1.0 tool used in GBD 2010 evaluated and pooled all available data, adjusted data for systematic bias associated with case ascertainment methods that varied from the reference and produced estimates by world regions with UIs by using Bayesian statistical methods. For GBD 2013, the improved DisMod-MR 2.0 increased computational speed, which allowed computations to be consistent between all disease parameters at the country rather than the region level. The hundred-fold increase in speed of DisMod-MR 2.0 was partly due to a more efficient rewrite of the code in C++, but also due to switching to a model specification of log rates rather than a negative binomial model used in DisMod-MR 1.0. In cross-validation tests, the log rates specification worked as well as or better than the negative binomial specification.[39] The sequence of estimation occurs at five levels: global, super-region, region, country and, where applicable, subnational location. The super-region priors are generated at the global level with mixed-effects, non-linear regression by using all available data; the super-region fit, in turn, informs the region fit, and so on down the cascade. Analysts can choose to branch the cascade in terms of time and sex at different levels depending on data density. The default used in most models is to branch by sex after the global fit but to retain all years of data until the lowest level in the cascade is reached.

The computational engine is limited to three levels of random effects; we differentiate estimates at the super-region, region, and country level. In GBD 2013, the subnational units of China, the United Kingdom and Mexico were treated as “countries” to enable a random effect to be estimated for every location with contributing data. However, the lack of a hierarchy between country and subnational units meant that the fit to country data contributed as much to the estimation of a subnational unit as the fits for all other countries in the region. We found inconsistency between the country fit and the aggregation of subnational estimates when the country’s epidemiology varied from the average of the region. Adding an additional level of random effects required a prohibitively comprehensive rewrite of the underlying DisMod-MR engine. Instead, we added a fifth layer to the cascade, with subnational estimation informed by the country fit and country covariates, plus an adjustment based on the average of the residuals between the subnational location’s available data and its prior. This technique mimicked the impact of a random effect on estimates among subnationals.

In GBD 2015, we also improved how country covariates differentiate non-fatal estimates for diseases with sparse data. The coefficients for country covariates are re-estimated at each level of the cascade. For a given location, country coefficients are calculated by using both data and prior information available for that location. In the absence of data, the coefficient of its parent location is used to utilise the predictive power of our covariates in data-sparse situations.
For GBD 2016, the computational engine (DisMod-MR 2.1) remained substantively unchanged from GBD 2015. We updated the age prediction sets to include age groups 80–84 years, 85–89 years, 90–94 years, and 95 years and older to comply with changes across all functional areas of the GBD.

In GBD 2017, we continued to use DisMod-MR 2.1 because no substantial changes were made. Updates to computation include extending the terminal prediction year to 2017 and additional subnational units in Ethiopia, Iran, New Zealand, Norway, and the Russian Federation.
In GBD 2019 and 2021, no substantial changes were made to DisMod-MR 2.1, but we made more substantial changes to how we use the tool. First, we added the years 2019, 2020, and 2021 as additional years of estimation. Second, we also included the option again to have random effects on cause-specific mortality rates (CSMR) and EMR. This functionality had been dropped a couple of GBD rounds earlier. Third, as we did all our adjustments for alternative case definitions and study methods as well as adjustments to combined-sex data points prior to entering data into DisMod-MR 2.1, we no longer used the functionality in DisMod-MR 2.1 to estimate coefficients for study and sex covariates. Fourth, based on simulation testing conducted in GBD 2019 we found that coverage improved, and errors reduced when passing down priors with a wider setting of minimum coefficient of variation (which determines the uncertainty around priors and hence how 'informative' the priors are) than had generally been used in past GBD iterations. We settled on a default value of 0.8 where in the past values of 0.4 or less had been more commonly used. We made some exceptions for highly prevalent conditions where a lower minimum coefficient of variation (CV) setting achieved the task of making priors less informative, but not completely uninformative.

In GBD 2017 and 2019 GBD rounds we calculated priors on excess mortality and entered these as data points by matching sex-specific prevalence data with an age width of 20 or less with the corresponding CSMR for the same location and year. Forstability, we excluded calculation of EMR for prevalence data points of less than 1 in a million. EMR is simply calculated as CSMR divided by prevalence. As with previous GBD years, for diseases with an average duration of less than a year (as indicated by a setting of remission greater than one), we ran an initial global model to get an equivalent prevalence and used the following formula to calculate EMR:

$$EMR = {CSMR * (remission + (ACMR - CSMR) + EMR\_pred)} / {incidence}$$

where,

ACMR is the all-cause mortality rate
EMR_pred is the EMR fit from an initial global DisMod model

Despite using the log of LDI or the HAQ Index as a covariate with a prior that the coefficient had to be negative, we found many disease models with an implausible distribution of mortality to prevalence (or incidence) ratios implying lower case fatality in locations with lower HAQ Index than in countries with higher HAQ Index. This likely signals an inconsistency between fatal and non-fatal data inputs. For GBD 2019, we decided to run regressions on EMR data (calculated as described above) first using MR-BRT with HAQ Index as a predictor. In general, we tend to think that CSMR estimates are more robust than non-fatal data because of much greater data availability and a lesser task in adjusting cause death data for garbage coding than the complex task of adjusting non-fatal data sources for alternative case definitions and study methods. To indicate that we would reduce the random effects on EMR and the minimum coefficient of variation for priors on EMR being created at each next level down the cascade. However, there were exceptions. For drug use disorders, the risk of overdose deaths is less a function of a country's quality of health services but driven more by the availability of harm reduction strategies, such as opioid substitution therapy, and the availability of highly potent opioids such as fentanyl, which have been an important contributor to the large increase in overdose deaths in the USA in the last decade. We settled on a model for opioid use disorder with wider random effects and higher minimum coefficient of variation to give less emphasis on CSMR when enforcing consistency with prevalence data. In a next round, we will work to find covariates that are more relevant to drug overdose deaths such as a grading of harm reduction strategies by country and over time. In the case of COPD, we noted that following the data on CSMR and EMR led to large increases in prevalence estimates in east Asia, Oceania and, to a lesser extent, south Asia. In the oldest age groups, prevalence estimates would be higher than the prevalence data for these locations and reach a level of close to 80% in the oldest age groups. In these locations, we will pay attention to how garbage codes are being redistributed onto COPD in the next round of GBD.

Section 2.6.3: DisMod-MR 2.1 likelihood estimation

Analysts have the choice of using a Gaussian, log-Gaussian, Laplace, or Log-Laplace likelihood function in DisMod-MR 2.1. The default log-Gaussian equation for the data likelihood is

$$-log[p(y_j|\Phi)] = \log(\sqrt{2}\pi) + \log(\delta_j + s_j) + \frac{1}{2}\left(\frac{\log(a_j + \eta_j) - \log(m_j + \eta_j)}{\delta_j + s_j}\right)^2$$

Where,

y_j is a "measurement value" (ie, data point)

Φ denotes all model random variables

η_j is the offset value, *eta*, for a particular "integrand" (prevalence, incidence, remission, excess mortality rate, with-condition mortality rate, cause-specific mortality rate, relative risk, or standardised mortality ratio)

a_j is the adjusted measurement for data point j, defined by

$$a_j = e^{(-u_j - c_j)} y_j$$

Where:

u_j is the total "area effect" (ie, the sum of the random effects at three levels of the cascade: super-region, region and country) and

c_j is the total covariate effect (ie, the mean combined fixed effects for sex, study level, and country level covariates), defined by

$$c_j = \sum_{k=0}^{K[I(j)]-1} \beta_{I(j),k} \hat{X}_{k,j}$$

with SD

$$s_j = \sum_{l=0}^{L[I(j)]-1} \zeta_{I(j),l} \hat{Z}_{k,j}$$

Where:

k denotes the mean value of each data point in relation to a covariate (also called x-covariate)

I(j) denotes a data point for a particular integrand, *j*

$\beta_{I(j),k}$ is the multiplier of the kth x-covariate for the ith integrand

$\hat{X}_{k,j}$ is the covariate value corresponding to the data point *j* for covariate *k*;

l denotes the SD of each data point in relation to a covariate (also called z-covariate)

$\zeta_{I(j),k}$ is the multiplier of the lth z-covariate for the ith integrand

δ_j is the SD for adjusted measurement *j*, defined by:

$$\delta_j = log[y_j + e^{(-u_j - c_j)}\eta_j + c_j] - log[y_j + e^{(-u_j - c_j)}\eta_j]$$

Where:

m_j denotes the model for the jth measurement, not counting effects or measurement noise, and defined by:

$$m_j = \frac{1}{B(j)-A(j)} \int_{A(j)}^{B(j)} I_j \text{ (a) da}$$

Where:

A(j) is the lower bound of the age range for a data point
B(j) is the upper bound of the age range for a data point
I_j denotes the function of age corresponding to the integrand for data point *j*

Section 2.7: Impairment and underlying cause estimation

For GBD 2021, as in GBD 2019, GBD 2017 and GBD 2016, we estimated the country-age-sex-year prevalence of nine impairments. Impairments in GBD are conditions or specific domains of functional health loss that are spread across many GBD causes as sequelae and for which there are better data to estimate the occurrence of the overall impairment than for each sequela based on the underlying cause. These impairments included anaemia, epilepsy, hearing loss, heart failure, intellectual disability, infertility, vision loss, Guillain-Barré syndrome, and pelvic inflammatory disease. Overall impairment prevalence was estimated by using DisMod-MR 2.1. We constrained cause-specific estimates of impairments, as in the 19 causes of blindness, to sum to the total prevalence estimated for that impairment. Anaemia, epilepsy, hearing loss, heart failure, and intellectual disability were estimated at different levels of severity. Estimates were made separately for primary infertility (those unable to conceive), secondary infertility (those having trouble conceiving again), and whether the impairment affected men and/or women. In the case of epilepsy, we determined the proportions with idiopathic and secondary epilepsy as well as the proportions with severe and less severe epilepsy by using mixed effects regressions. The sparse data for the proportion of seizure-free, treated epilepsy were pooled in a random effects meta-analysis. DisMod-MR 2.1 models produced country-, age-, sex-, and year-specific severity levels of hearing loss and vision loss. Because of limited information on the severity levels of intellectual disability, we assumed a similar distribution of severity globally based on random effects meta-analysis of IQ-specific data for the overall impairment. This assumption was supplemented by cause-specific severity distributions for chromosomal causes and iodine deficiency; the severity of intellectual disability included in the long-term sequelae of causes including neonatal disorders, meningitis, encephalitis, neonatal tetanus, and malaria was estimated in combined health states of multiple impairments such as motor impairment, blindness, and/or seizures (R, 2015).We changed the name of the intellectual disability impairment to specify that estimates reflect cases arising during the developmental period, which we have defined as ages under 20 years. The severity of heart failure was derived from our Medical Expenditure Panel Surveys (MEPS) analysis and therefore was not specific for country, year, age, or sex.

A detailed description of the methods of each impairment can be found at the end of Section 4.12 of this appendix.

Section 2.7.1: Impairment squeeze

For the impairments epilepsy, intellectual disability, and blindness, mentioned above in Step 4, we often have better information regarding the total prevalence of the impairment rather than the prevalence of said impairment due to its various causes. For example, we have more data and a better idea of the total number of blind individuals (which we refer to herein as the blindness "envelope") in the world than we do the number of individuals who are blind due to a specific cause like retinopathy of prematurity or cataract. We achieve this consistency by either squeezing or inflating the individual sequela prevalence values so that their sums fit into each appropriate envelope. Blindness, epilepsy, and/or intellectual disability appear in various combinations with motor impairment levels as sequelae for a number of neonatal disorders and infectious diseases like malaria and neonatal tetanus ("Moderate motor impairment with blindness and epilepsy due to neonatal tetanus", for example). This presents an extra challenge because any squeeze or inflation of one of the impairments making up a sequela affects the others.

We set rules on how to do these adjustments sequentially. First, when the envelope of an impairment is smaller than the sum of all contributing causes, we redistribute the excess prevalent cases of combined impairment sequelae onto the sequelae that only have motor impairment (at a mild, moderate, or severe level) within the same cause grouping. Second, we apply the adjustments in a particular order such that we always fit at least one of the envelopes exactly where the other one or two envelopes may be exceeded by some amount. We first enforce a fit to the epilepsy impairment envelope, then intellectual disability, and last, blindness. Thus, the epilepsy envelope always matches exactly, whereas the intellectual disability and blindness envelopes may occasionally be exceeded on a draw-by-draw basis.

Section 2.8: Severity distributionSequelae were defined in terms of severity for 236 causes. We generally followed the same approach for estimating the distribution of severity we used in GBD 2019. In cases in which severity was related to a particular impairment, such as mild, moderate, and severe heart failure due to ischaemic heart disease or pulmonary arterial hypertension, the analysis was driven by impairment estimation methods. Severity levels for causes such as chronic kidney disease, epilepsy and COPD were modelled using DisMod-MR 2.1 or ST-GPR, whereas we performed meta-analyses to estimate the allocation of severity for causes such as rheumatoid arthritis, and multiple sclerosis. For dementia, we changed from using meta-analysis of three age categories to a more flexible model in MR-BRT using a spline on age. That allowed us to increase the number of studies informing severity from 7 to 67. For gallbladder and biliary diseases, we performed a meta-analysis of six community-based studies of the proportion of cases of gallbladder disease identified by ultrasonography who are symptomatic. In previous rounds, inpatient admission for gall bladder and biliary disease as a primary diagnosis were taken to represent symptomatic cases.

For many causes, we continue to have inadequate data on severity from surveys or the epidemiological literature. For those diseases, we made use of three population surveys: the MEPS 2000–2014, the [US] National Epidemiological Survey on Alcohol and Related Conditions (NESARC) 2000–2001 and 2004–2005, and the Australian National Survey of Mental Health and

Wellbeing of Adults (NSMHWB) 1997 (Medical Expenditure Panel Survey Home, n.d.) (Mental Health and Wellbeing: Profile of Adults, Australia, 1998) Each dataset contained individual-level measurements of functional health status made by using the 12-Item Short Form Health Survey (SF-12) as well as diagnostic information on the causes affecting each individual.
To use the data collected by measuring the distribution of severity with the SF-12, the individual SF-12 summary scores were mapped to an equivalent DW. A convenience sample of respondents was asked to complete SF-12 for the hypothetical individual living in a health state described by using a selection of 60 of the 235 health states with their lay descriptions from the GBD DW surveys reflecting the full range of severity. Each of these health states has a measured DW associated with it on a zero to one scale. We collected 2783 usable responses in total.

The final relationship between SF-12 score and DW is depicted in figure 8:

Figure 8. SF-12 composite scores and disability weights for 60 health states with fitted loess regression

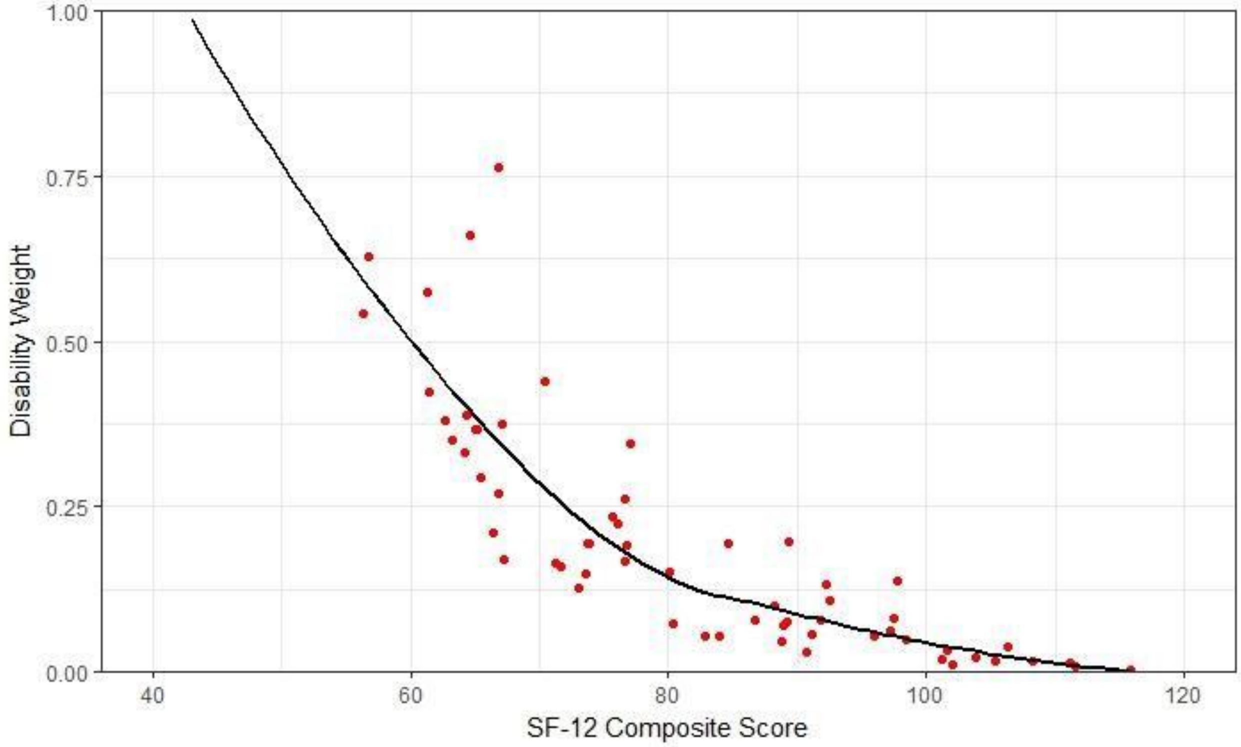

To generate a smooth mapping from SF-12 combined scores to the GBD DW space, we used locally estimated scatterplot smoothing regression on the random effects for each health state. DWs were capped to remain between 0 and 1. All SF-12 survey data were thus transformed into DW space.
The second stage of the analysis was to build models predicting the transformed SF-12 scores as a function of the number of causes suffered by each individual. First, variable selection was performed by using least absolute shrinkage and selection operator (LASSO) regression to

penalize the regression coefficients of highly correlated causes. The tuning parameter, λ, controls the strength of the least-squares penalty. When λ=0, LASSO regression returns the same results as ordinary least-squares regression. Higher values of λ impose a stronger penalty and constrain a greater number of model parameters to 0. A ten-fold cross-validation was used to find the value of the λ that minimized the mean cross-validated error. This process resulted in a λ value of 0.0013 and eliminated 10 causes from the analysis. Transformed SF-12 scores into the DW scale for the remaining 190 causes were then modelled for each measure *m* of each individual *i* over *n* total causes in the survey as follows:

$$logit(DW)_{im} = \beta_0 + \beta_1 Condition1_{im} + \cdots + \beta_n Condition\ n_{im}$$

This equation effectively assumes that comorbid causes act to change SF-12 scores in a multiplicative fashion rather than an additive fashion.
To estimate the comorbidity-corrected effect of each cause (ie, in isolation) on total disability, we compared the predicted DW without the cause of interest (counterfactual DW) with the predicted DW including the cause of interest. Following the multiplicative comorbidity equation, the joint effect can be written

$$Condition\ specificDW = 1 - \frac{1 - predictedDW_m}{1 - counterfactualDW_m}$$

The mean of this cause-specific effect over all observations is the population marginal effect of a cause.

Using the model above, we estimate a counterfactual DW – the total individual DW excluding the effect of the cause of interest. We compared the observed distribution of functional health status with this counterfactual distribution to determine the marginal effect of the cause of interest. In other words, we estimated the health state for each individual and for each cause as the cumulative individual weight minus the effects of all comorbid causes.

$$Health\ state\ DW = 1 - \frac{1 - individual\ cumulativeDW_m}{1 - counterfactualDW_m}$$

The estimation strategy for health state-specific severity distributions for which there are multiple severity categories involved binning individuals' weights into severity cut-offs (eg, mild, moderate, and severe) for which DWs were derived. These bins were defined by using results from the GBD Disability Weights Studies (JA, 2015) for causes that had multiple health states defined. Cut-offs for the severity group were the midpoints between DWs of the health state and cases distributed into severity bins accordingly. For example, individuals with a health state DW above the mid-point between the mild DW and the moderate DW for a particular condition

would be assigned the moderate sequela. Cases were considered asymptomatic if the counterfactual weight was equal to or greater than the individual cumulative weight. The proportion of cases of a condition assigned to each level of severity for that condition was then used as the severity distribution of the condition for prevalence estimates to be apportioned accordingly into severity-specific prevalence estimates.

Section 2.9: Disability weights

To compute YLDs for a particular health outcome in a given population, the number of people living with that outcome is multiplied by a disability weight (DW) that represents the magnitude of health loss associated with the outcome. DWs are measured on a scale from 0 to 1; 0 implies a state equivalent to full health, and 1, a state equivalent to death.

Section 2.9.1: GBD 2010 Disability Weights Measurement Study

For GBD 2010, a primary data collection effort focused on measuring health loss rather than welfare loss by using a standardised approach of simple comparison questions directed to the general public across diverse communities.

Multi-country household surveys were conducted between Oct 28, 2009 and June 23, 2010 in five countries (Bangladesh, Indonesia, Peru, Tanzania, and the USA) selected to provide diversity across culture, language, and socioeconomic status.

Personal face-to-face computer-assisted interviews were conducted for all household surveys except for the survey in the US, which was conducted by computer-assisted telephone interview. Households were randomly selected by using a multistage stratified sampling design for which the probability of selection was proportional to the population size. In all cases, samples were designed to be representative of a given geographical area and, in the USA, to provide national representation.

For every contacted household, an adult respondent age 18 years or older was randomly selected by the survey program by means of the Kish approach. For face-to-face interviews, as many as three visits were made to selected households to establish contact. When a respondent was identified, as many as three return visits were made to do the survey at a time when the respondent was available. For the US telephone surveys, repeated calls were made up to seven times.

A web-based survey was posted at a dedicated URL between July 26, 2010 and May 16, 2011. The survey was initially available in English and subsequently available in Spanish and Mandarin. Recruitment of respondents occurred through several channels, such as news items and editorials in scientific journals, announcements at scientific meetings, postings on websites of institutions participating in the GBD, and social networking and communication mobilisation channels as well as direct contact with individuals and groups with known global health

interests by tapping into the professional networks of the study investigators and their colleagues. Participants in the web-based survey were required to be ages 18 or older. Household surveys obtained oral informed consent from all participants; written informed consent was obtained from participants in the web survey. Ethical review board approval was obtained from each household survey site and the University of Washington, Seattle, WA.

Standardised survey instruments were developed to obtain comparative assessments of the full array of disease and injury sequelae, parsimoniously captured in 220 unique health states. Lay descriptions of health states formed the basis for all comparisons. These descriptions used simple, non-clinical vocabulary that emphasised the major functional consequences and symptoms associated with each health state. Development of these descriptions involved an iterative process of detailed consultation with experts participating in the GBD 2010 study; the goals was to capture the most relevant details of each health state while avoiding ambiguity and ensuring consistency. When possible, health states were grounded in standard clinical classifications systems. For example, the Canadian Cardiovascular Society grading scale was referenced for descriptions of stages of angina (L., 2002), and the New York Heart Association functional classification was referenced for severity of heart failure (M., 1994). Pilot testing indicated that the lay descriptions in face-to-face interviews should not exceed 30 words.

A paired comparison question formed the basis of all surveys. The questions in the survey were framed with the following statement, “A person’s health may limit how well parts of his body or mind work. As a result, some people are not able to do all of the things in life that others may do, and some people are more severely limited than others. I am going to ask you a series of questions about different health problems. In each question, I will describe two different people…” Descriptions of two hypothetical people, each with a particular health state, were presented to respondents who were then asked which person they regarded as healthier. Health pairs in all surveys were selected by a randomizing computer algorithm. In the five household surveys, paired comparisons were presented for a subset of 108 health states pertaining to chronic conditions. The framing of chronic and acute conditions is different as they were presented as causing life-long or temporary health loss. We chose to only field health states that could be framed as lasting a lifetime in the household surveys as we hypothesized that presenting differently framed comparisons would be difficult to convey in face-to-face interviews. In the web survey, we considered this more feasible because respondents could read and refer to the framing of the question for each pair-wise comparison. All 220 health states were thus evaluated in the web survey.

In addition, the web survey included questions relating to population health and health programs specifically—such as “Imagine two different health programs. The first program prevented 1000 people from getting an illness that causes rapid death. The second program prevented 2000 people from getting an illness that is not fatal but causes lifelong health

problems resulting in moderate to severe disability. Which program would you say produced the greater overall health benefits?” This information was used to anchor the results from the pair-wise comparisons on the 0–1 DW scale.

Section 2.9.2: GBD 2013 European disability weights measurement study

The GBD 2010 DWs were critically dependent on the ways that outcomes were described to survey respondents. Descriptions for health states were designed to balance validity and parsimony, and this approach necessarily meant that some details of different health states had to be omitted. Because lay descriptions were developed collaboratively through individual expert groups organised around a particular set of health issues, some amount of variability in language and detail inevitably occurred. Criticisms and suggestions for improvement came from a number of commentators on the GBD 2010 DWs measurement study (E., 2013) (Taylor HR, 2013) (Voigt K, 2014)

GBD 2013 expanded the list of disease and injury causes and sequelae mapped to 235 unique health states. Additional data for the European Disability Weights Measurement Study were collected between September 23, 2013, and November 11, 2013, in Hungary, Italy, the Netherlands, and Sweden. The initiation of these surveys was connected to a project sponsored by the European Centre for Disease Prevention and Control (M, 2012)The four selected countries were chosen to be representative of the four regions of Europe (east, south, middle, and north) in terms of age, sex, and education of the respondents. Respondents were recruited from standing internet panels in each country on the basis of quota sampling with reference to age, sex, and education in such a way as to maintain the population representativeness of these characteristics. Eligible participants were 18–65 years old and were preselected in the Netherlands, where the age, sex, and education of respondents were already known, or in the other three countries, invited to participate via a web-link and then selected on the basis of their individual characteristics.

The protocol for the European DWs measurement study followed the protocol that was developed and implemented in the GBD 2010 DWs measurement study. Lay descriptions for some health states that lacked mention of an important symptom or for which consistency of wording across different levels of severity had been noted were reworded. The European DWs measurement study included 255 health states, of which 183 were used in the analyses of GBD 2013. Those 183 consisted of 135 of the 220 health states that were included in the European DWs measurement study with unmodified lay descriptions and 30 from GBD 2010 for which alternative lay descriptions were included. DWs were estimated for additional sequelae that were incorporated into GBD 2013 but had not been included in GBD 2010.

Finding high correlation in resulting DW values between the country surveys and the web survey, we analysed the results of all surveys together. We ran probit regression analyses on

the answers to the pair-wise comparison questions by using dummies for each health state with a value of 1 for the first state in a pair, –1 for the second state in a pair, and 0 for all states other than the pair. This method formalizes the intuition that if two health states in a pair produce similar health loss, the answers are likely to be evenly split; a pair of health states with very different health loss get many more responses favouring one over the other. The statistical methods infer the distances between values attached to different health states based on the frequencies of responses to the paired comparisons.

A second analytic step is needed to anchor the resulting estimates onto the 0–1 DWs scale, where 0 equals no loss of health, with 1 meant to represent loss equivalent to death. We anchored results from the probit regression analysis onto the 0–1 scale by using population health equivalence data from the GBD 2010 web survey by using a linear regression of the probit coefficients from the analysis of paired comparisons on the logit-transformed DW estimates derived from interval regression of the population health equivalence responses. Using numerical integration, we then estimated mean values for DWs on the natural 0–1 scale. Uncertainty was estimated by bootstrapping with 1,000 samples. For a complete listing of the lay descriptions and values for the 440 health states (including combined health states) used in GBD 2021, please refer to Table 6. For a complete overview of disability weights applied to the Global Burden of Disease Study (al, 2015)

Section 2.10: Comorbidity correction (COMO)

The final stage in the estimation of YLDs is a micro-simulation, which adjusts for comorbidity. We refer to this micro-simulation process as "COMO" (for comorbidity correction). For GBD 2019 and 2021, we estimated the co-occurrence of different diseases by simulating 20,000 individuals in each location-age-sex-year combination as exposed to the independent probability of having any of the sequelae included in GBD based on prevalence. We tested the contribution of dependent and independent comorbidity in the US MEPS data and found that independent comorbidity was the dominant factor even though well-known examples of dependent comorbidity exist, such as clustering of conditions like diabetes and stroke or anxiety and alcohol use disorders. Age was the main predictor of comorbidity such that age-specific micro-simulations accommodated most of the required comorbidity correction (Vos T, 2012)

The two components necessary for the computation of YLDs and are the two inputs into COMO: 1) prevalence of each disease sequela and 2) DWs. The prevalence values of causes are primarily produced by using DisMod-MR 2.1 and, for causes with multiple sequelae, subsequently apportioned into sequela-specific prevalence based on available estimates of the severity distribution. The estimation of DWs and severity distributions have been described earlier in this appendix.

The micro-simulation, as performed for each age-sex-location-year, can best be represented as a four-step process. First, simulated individuals (simulants) are exposed to independent

probabilities of having each sequela, where the probability is equal to the prevalence estimate. For each simulant, the probability of having a disease sequela is equal to the estimated prevalence . Each simulant is determined to have or not have the disease sequelae based on a draw from a binomial distribution. From this simulation, simulants end up with any number of sequelae, from 0 up to the theoretical maximum given their demographics. Second, the DW for each simulant is estimated on the basis of the disease sequelae that they have acquired. The formula for the cumulative DW for a simulant is one minus the multiplicative sum of one minus each DW present

$$Simulant\ DW_l = 1 - \prod_{k=i}^{j}(1 - DW_k)$$

Where:

DW_k is the DW for the kth disease sequela that the simulant *l* has acquired.

Once the simulant DW is computed, the DW attributable to each sequela for the simulant is calculated by using the following formula:

$$ADW_{lk} = \frac{DW_k}{\sum_{k=i}^{k=j} DW_k} * Simulant\ DW_l$$

Where:

ADW_{lk} is the attributable DW for disease sequela *k* in simulant *l*

DW_k is the DW for disease sequela *k*

Simulant DW_l is the DW for simulant *l* from the combination of all sequelae that they have acquired.

This formula apportions the overall simulant DW to each condition in proportion to the DW of each condition in isolation.

Finally, YLDs per capita in an age-sex-country-year are computed by taking the sum of the attributable DWs for a disease sequela across simulants.

$$YLD\ Rate_k = \frac{\sum_{l=1}^{n} ADW_{lk}}{n}$$

The actual number of YLDs from disease sequela *k* in an age-sex-location-year is then computed as the YLD rate *k* times the appropriate age-sex-location-year population.

By repeating the simulation process for each age-sex-country-year 500 times, the uncertainty in the prevalence of each disease sequela and the DW is propagated into the final comorbidity corrected YLD results. We selected 20,000 simulants for each age-sex-location-year group on the basis of simulation testing, which has shown that results are stable for YLDs at this number of simulants even in the younger age groups when prevalence is relatively low. Mean results for

YLDs that reflect 10 million simulants (20,000 simulants multiplied by 500 iterations to capture uncertainty) are very stable in each age-sex-location-year. For any given location-year-age-sex group, acause aggregate prevalence values were calculated as $1- \prod(1\text{-prevalence})$

Section 2.11: YLD computation, uncertainty, and residual YLDs

For GBD 2021, we computed YLDs by sequela as prevalence multiplied by the DW for the health state associated with that sequela. The uncertainty ranges reported around YLDs incorporate uncertainty in prevalence and uncertainty in the DW. To do this, we take the 500 samples of comorbidity-corrected YLDs and 500 samples of the DW to generate 500 samples of the YLD distribution. We assume no correlation in the uncertainty in prevalence and DWs. The 95% uncertainty interval is reported as the 25th and 975th values of the distribution. UIs for YLDs at different points in time (1990, 1995, 2000, 2005, 2010, 2015, 2020 and 2021) for a given disease or sequela are correlated because of the shared uncertainty in the DW and DW draws are not year specific. For this reason, changes in YLDs over time can be significant even if the UIs of the two estimates of YLDs largely overlap And prevalence UIs are used to determine significance of change in YLDs over time since DW draws are year agnostic.

Section 2.11.1: Residual YLDs

Despite expanding our list of causes and sequelae in successive GBD iterations, many diseases remain for which we do not explicitly estimate disease prevalence and YLDs. Less common diseases and their sequelae were included in 34 residual categories (table 7). For 22 of these residual categories, epidemiological data on incidence or prevalence were available, so these were modelled accordingly. For 13 residual categories, epidemiological data on incidence and prevalence were not available, but sufficient CoD data allowed for CoD estimates. For these residual categories, we estimated YLDs by multiplying their YLL estimates by the ratio of YLDs to YLLs from the Level 3 causes in the same disease category that were explicitly modelled. This scaling was done for each country-sex-year. This approach made the simplifying assumption that the residual diseases caused disability proportionate to the ratio of disability to mortality in explicitly modelled diseases. We did not include causes with large disability but no or little mortality in estimating these ratios. For example, we estimated the YLDs from other neurological disorders from the YLD to YLL ratios for dementia, multiple sclerosis, and Parkinson's disease but did not include the YLDs from headaches and epilepsy in the ratio. Detailed information on how YLDs for residual causes were estimated are available in their respective cause writeups in section 6.

Section 2.12: Birth prevalence

A number of conditions are present at birth, and quantifying them is important in fully describing the epidemiology of diseases within populations. These include many conditions included in the GBD cause group of neonatal disorders, infections that are transmitted from

mother to child either transplacentally or during birth, and congenital birth defects arising either *de novo* or from maternal exposures. Although these conditions were included in the underlying models informing previous GBD iterations, we developed a system for reporting them for the first time in GBD 2017; a list of these causes is reported in table 8. Mathematically (ie, in the models), conditions present at birth are equivalent to "birth prevalence." However, we report these as "incidence" in recognition of the way that GBD defines incidence as a new case of a disease or injury entering the population. To process these results for publication in GBD, we used a three-step process. First, the number of cases at birth was calculated as birth prevalence rate multiplied by number of live births for each location, sex, and year. Second, the number of cases present at birth were summed with incident cases during the early neonatal period (calculated as the 0-to-6-days incidence rate times the 0-to-6-days population), and the early neonatal incidence rate was recalculated by re-dividing by the 0-to-6-days population. Third, incidence rates for aggregate age groups were re-calculated by using the revised incidence figures for the early neonatal period.
Causes included in reporting are all of those for which birth prevalence has been estimated in GBD 2021 as part of existing modelling processes. Although extensive, this list should not be considered exhaustive of all of the conditions that can be present at birth. Future efforts in GBD will focus on identifying and comprehensively including all conditions present at birth, including revision of model frameworks as necessary. These efforts will also be facilitated by continuing improvements in the resolution of epidemiologic estimates of disease burden during pregnancy. These efforts are also expected to facilitate subsequent analyses derived from GBD that evaluate how maternal interventions, including pregnancy surveillance, can influence patterns of neonatal, infant, and child health.

Section 3: SDI

Section 3.1: SDI definition

The Socio-demographic Index (SDI) is a composite indicator of background social and economic conditions that influence health outcomes in each location. In short, it is the geometric mean of 0 to 1 indices of total fertility rate (TFR) for those younger than 25 years old (TFU25), mean education for those 15 years old and older (EDU15+), and lag-distributed income (LDI) per capita. For GBD 2021 after calculating SDI, values were multiplied by 100 for a scale of 0 to 100.

Section 3.2: Development of revised SDI indicator

SDI was originally constructed for GBD 2015 by using the Human Development Index (HDI) methodology, wherein a 0 to 1 index value was determined for each of the original three covariate inputs (TFR in ages 15 to 49 years, EDU15+, and LDI per capita) by using the observed minima and maxima over the estimation period to set the scales (H, 2016)

In response to feedback from collaborators and the evolution of the GBD, we have refined the indicator with each GBD cycle. Beginning in GBD 2017, along with our expanded estimation of age-specific fertility, we replaced TFR with TFU25 as one of the three component indices. The TFU25 provides a better measure of women's status in society because it focuses on ages at which childbearing disrupts the pursuit of education and entrance into the workforce. In addition, we observed that in highly developed countries, the TFU25 has tended to decline consistently over time despite rebounds in TFR driven by increasing fertility at older ages. The concordance correlation coefficient between SDI based on the GBD 2016 method and the updated method for GBD 2017 was 0.981.

During GBD 2016, we moved from using relative index scales to using absolute scales to enhance the stability of SDI interpretation over time because we noticed that the measure was highly sensitive to the addition of subnational units that tended to stretch the empirical minima and maxima.[21] We selected the minima and maxima of the scales by examining the relationships each of the inputs had with life expectancy at birth and under-5 mortality and by identifying points of limiting returns at both high and low values if they occurred before theoretical limits (eg, a TFU25 of 0) were reached.

Thus, for each covariate input, an index score of 0 represents the minimum level of each covariate input past which selected health outcomes can get no worse, and an index score of 1 represents the maximum level of each covariate input past which selected health outcomes cease to improve. As a composite, a location with an SDI of 0 would have a theoretical minimum level of sociodemographic development relevant to these health outcomes, and a location with an SDI of 1 (before multiplying by 100 for reporting) would have a theoretical maximum level of sociodemographic development relevant to these health outcomes.

We computed the index scores underlying SDI as follows:

$$I_{Cly} = \max\left(\frac{C_{ly} - C_{low}}{C_{high} - C_{low}}, 0.005\right)$$

Where:

I_{cly} is the index for covariate *C*, location *l*, and year *y* and is equal to the difference between the value of that covariate in that location-year and the lower bound of the covariate divided by the difference between the upper and lower bounds for that covariate

If the values of input covariates fell outside the upper or lower bounds, they were mapped to the respective upper or lower bounds. We also note that the index value for TFU25 was computed as $1 - I_{TFU25ly}$ because lower TFU25s correspond to higher levels of development and thus higher index scores. For GBD 2021, we expanded the computation of SDI to 1075 national and subnational locations spanning the time period 1950–2021.

The composite SDI is the geometric mean of these three indices for a given location-year. The cut-off values used to determine quintiles for analysis were then computed by using country-level estimates of SDI for the year 2019, excluding countries with populations less than 1 million.

For GBD 2021, final SDI values were multiplied by 100 for reporting, in order to improve understanding of and broader engagement with the values. As such, GBD 2021 SDI is calculated as it was in 2019, but multiplied by 100 at the end (see example calculation below). Final reporting values are on a 0 to 100 scale.

Example calculation

We present the equation used to calculate SDI for a hypothetical country in the year 2010:

$$TFU25 = 1.09;\ Mean\ educ\ yrs\ pc = 8.23;\ lnLDI = 9.60$$

$$I_{TFU25} = 1 - \frac{1.09 - 0}{3 - 0} = 0.637$$

$$I_{Educ} = \frac{8.23 - 0}{17 - 0} = 0.484$$

$$I_{\ln LDI} = \frac{9.60 - 5.52}{11.00 - 5.52} = 0.744$$

$$SDI = \sqrt[3]{I_{TFU25} \cdot I_{Educ} \cdot I_{\ln LDI}} = \sqrt{.637 \cdot .484 \cdot .744} = 0.611$$

$$I_{lnLDI} = \frac{9.58 - 5.52}{11.00 - 5.52} = 0.741$$

$$SDI = \sqrt[3]{I_{TFU25} \cdot I_{Educ} \cdot I_{\ln LDI}} = \sqrt{.855 \cdot .543 \cdot .741} = 0.701$$

$$\text{GBD 2019 reporting } SDI = 0.701 * 100 = 70.1$$

Section 4: Estimation process for DALYs

To estimate DALYs for GBD 2021, we started by estimating cause-specific mortality and non-fatal health loss. For each year for which YLDs have been estimated, we computed DALYs by adding YLLs and YLDs for each age-sex-location (Figure A). Uncertainty in YLLs was assumed to be independent of uncertainty in YLDs. We calculated 500 draws for DALYs by summing the first draw of the 500 draws for YLLs and YLDs and then repeating for each subsequent draw. 95% UIs were computed by using the 25th and 975th ordered draw of the DALY uncertainty distribution. We calculated DALYs as the sum of YLLs and YLDs for each cause, location, age group, sex, and year.

Figure 9. DALY burden estimation for GBD 2021

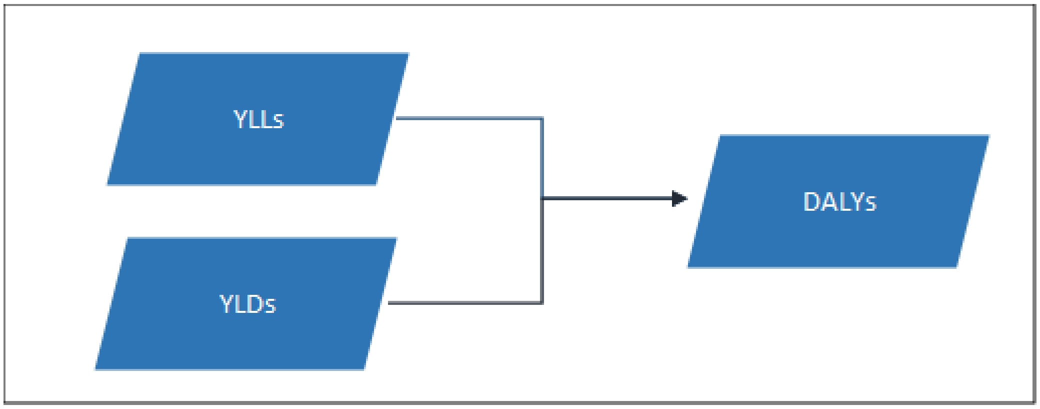

Section 5: HALE

The first step to calculating healthy life expectancy for a population (defined by sex, country, and year) was to compute average health of individuals for every age group in that population. We combined information about prevalences for all sequelae and their associated disability weights, and accounted for comorbidity with a Monte Carlo simulation approach. We made the assumption that comorbidities were independent within each age group. We created simulations where individuals were exposed to each sequela with a probability equal to the estimated prevalence of that sequela in each age group. This created a simulated population where the frequencies of many possible multi-morbidities were consistent with the underlying estimates of prevalence. We define 1 minus the disability weight as the positive health associated with each sequela. The combined health for a simulated individual was the product of these positive health values for all relevant sequelae in the presence of multiple sequelae.

Average health values are computed as 1 minute the YLD per person in a population, which are then used to compute health adjusted person years. We incorporated average health values into the life table using Sullivan's method. First, we multiplied values in the nLx (average person-years lived within an age interval starting at age x) column of the life table by the corresponding average health value in that interval. We recalculated the rest of the life table using the adjusted nLx values. Sullivan's method began with an adjusted estimate of health adjusted life years within the terminal age interval (equal to nLx multiplied by the average health value for the terminal age group) and subsequent calculations we produced estimates by iterating through younger age intervals, summing the health-adjusted person-years with all age intervals above the current age interval to generate health adjusted person years lived above a certain age (adjusted Tx) for each age group. After calculating adjusted Tx for all age groups, HALE was calculated by dividing the adjusted Tx for each age group by the proportion of hypothetical birth cohort still alive at age x (al K. H., 2018).

Section 6: Non-fatal cause-specific modelling descriptions

GBD 2021 non-fatal appendix write-ups in order:

1. Acne vulgaris
2. Acute glomerulonephritis
3. Acute hepatitis
4. African trypanosomiasis
5. Alcohol use disorders (fetal)
6. Alcohol use disorders
7. Alopecia areata
8. Alzheimer's disease and other dementias
9. Amphetamine use disorders
10. Anaemia
11. Anorexia nervosa
12. Anxiety disorders
13. Appendicitis
14. Ascariasis
15. Asthma
16. Atrial fibrillation and flutter
17. Attention-deficit/hyperactivity disorder
18. Autism spectrum disorders
19. Benign prostatic hyperplasia
20. Bipolar disorder
21. Blindness and vision impairment
22. Bulimia nervosa
23. Cannabis use disorders
24. Cellulitis
25. Chagas disease
26. Chronic kidney disease
27. Chronic obstructive pulmonary disease
28. Cirrhosis and other chronic liver diseases
29. Cocaine use disorders
30. Conduct disorder
31. Congenital birth defects
32. COVID-19 Adjustments
33. COVID- 19
34. Cutaneous and mucocutaneous leishmaniasis
35. Cystic echinococcosis
36. Cysticercosis
37. Decubitus ulcer
38. Dengue
39. Dermatitis
40. Developmental intellectual disability
41. Diabetes mellitus
42. Diarrhoeal diseases
43. Diphtheria

44. Dysthymia
45. Ebola virus disease
46. Encephalitis
47. Endocarditis
48. Endocrine, metabolic, blood, and immune disorders
49. Epilepsy
50. Fistula
51. Food-borne trematodiases
52. Fungal skin diseases
53. Gallbladder and biliary diseases
54. Gastritis and duodenitis
55. Gastro-oesophageal reflux disease
56. Gout
57. Guillain-Barré syndrome
58. Guinea worm disease
59. Gynaecological diseases
60. Haemoglobinopathies and haemolytic anaemias
61. Headache disorders
62. Hearing loss
63. Heart failure
64. HIV/AIDS
65. Hookworm disease
66. Infertility
67. Inflammatory bowel disease
68. Inguinal, femoral, and abdominal hernia
69. Injuries
70. Interstitial lung disease and pulmonary sarcoidosis
71. Invasive non-typhoidal Salmonella (iNTS)
72. Ischaemic heart disease
73. Leprosy
74. Low back pain
75. Lower respiratory infections
76. Lymphatic filariasis
77. Major depressive disorder
78. Malaria
79. Maternal disorders
80. Measles
81. Meningitis
82. Motor neuron disease
83. Multiple sclerosis
84. Myocarditis
85. NAFLD
86. Neck pain
87. Neonatal preterm birth
88. Neoplasms
89. Non-rheumatic valvular heart disease
90. Nutritional deficiencies
91. Onchocerciasis

92. Opioid use disorders
93. Oral disorders
94. Osteoarthritis
95. Other cardiovascular and circulatory diseases
96. Other chronic respiratory diseases
97. Other digestive diseases
98. Other drug use disorders
99. Other intestinal infectious diseases
100. Other mental disorders
101. Other musculoskeletal disorders
102. Other neglected tropical diseases
103. Other neurological disorders
104. Other sense organ diseases
105. Other skin and subcutaneous diseases
106. Other unspecified infectious diseases
107. Other urinary diseases
108. Otitis media
109. Pancreatitis
110. Paralytic ileus and intestinal obstruction
111. Parkinson's disease
112. Pelvic inflammatory disease
113. Peptic ulcer disease
114. Peripheral artery disease
115. Pneumoconiosis
116. Pruritus
117. Psoriasis
118. Pulmonary arterial hypertension
119. Pyoderma
120. Rabies
121. Rheumatic heart disease
122. Rheumatoid arthritis
123. Scabies
124. Schistosomiasis
125. Schizophrenia
126. Sexual violence
127. Sexually transmitted infections excluding HIV
128. Stroke
129. Syphilis
130. Tetanus
131. Trichuriasis
132. Tuberculosis
133. Typhoid and paratyphoid
134. Upper respiratory infections
135. Urinary tract infection and interstitial nephritis
136. Urolithiasis
137. Urticaria
138. Varicella and herpes zoster
139. Vascular intestinal disorders

140. Viral skin diseases
141. Visceral leishmaniasis
142. Whooping cough (pertussis)
143. Yellow fever
144. Zika virus disease

References:

Peng Zheng, Ryan Barber, Reed J. D. Sorensen, Christopher J. L

al, K. H. (2018). Global, regional, and national disability-adjusted life-years (DALYs) for 359 diseases and injuries and healthy life expectancy (HALE) for 195 countries and territories, 1990–2017: a systematic analysis for the Global Burden of Disease Study 2017. *The Lancet* , 392: 1859–922.

al, S. e. (2015). Disability weights for the Global Burden of Disease 2013 study. *The Lancet Global Health*.

CE, R. (2005). *Gaussian Processes for Machine Learning.* Boston, MA: The MIT Press.

Collaborators, G. 2. (2020). GBD 2019 Risk Factors Collaborators. Global burden of 87 risk factors in 204 countries and territories, 1990–2019: a systematic analysis for the Global Burden of Disease Study 2019. . *The Lancet*.

E., N. (2013). Disability weights in the Global Burden of Disease 2010: Unclear meaning and overstatement of international agreement. *Health Policy*, 111: 99–104.

H, W. (2016). Global, regional, and national life expectancy, all-cause mortality, and cause-specific mortality for 249 causes of death, 1980–2015: a systematic analysis for the Global Burden of Disease Study 2015. *The Lancet*, 388: 1459–544.

JA, S. (2015). Disability weights for the Global Burden of Disease 2013 study. *Lancet Glob Health* , 3: e712–23.

L., C. (2002). The Canadian Cardiovascular Society grading of angina pectoris revisited 30 years later. *Can J Cardiol*, 18(4):371-9.

M, K. (2012). New Methodology for Estimating the Burden of Infectious Diseases in Europe. *PLOS Med*, 9: e1001205.

M. , D. (1994). *Nomenclature and criteria for diagnosis of diseases of the heart and great vessels, 9th ed.* Boston, MA: Little Brown & Co,. Retrieved from https://trove.nla.gov.au/version/13288061

Medical Expenditure Panel Survey Home. (n.d.). Retrieved from https://meps.ahrq.gov/mepsweb/

Mental Health and Wellbeing: Profile of Adults, Australia. (1998, March 12). Retrieved from http://www.abs.gov.au/ausstats/abs@.nsf/ProductsbyReleaseDate/D5A0AC778746378FCA2574EA00122887?OpenDocument

Ng M, e. a. (2014). Global, regional, and national prevalence of overweight and obesity in children and adults during 1980–2013: a systematic analysis for the Global Burden of Disease Study 2013. *The Lancet* , 384: 766–81.

Ng M, e. a. (2014). Smoking Prevalence and Cigarette Consumption in 187 Countries, 1980-2012. . *JAMA*, 311: 183–92.

NIAAA Publications . (n.d.). Retrieved from http://pubs.niaaa.nih.gov/publications/arh29-2/74-78.htm

R, B. (2015). Estimating distributions of health state severity for the global burden of disease study. *Popul Health Metr* , 13: 31.

Taylor HR, e. a. (2013). Disability weights for vision disorders in Global Burden of Disease study. *The Lancet*, 381: 23.

Vasudevan S, e. a. (2009). Gaussian Process modeling of large scale terrain. *IEEE International Conference on Robotics and Automation.* , 1047–53.

Voigt K, K. N. (2014). Disability weights in the global burden of disease 2010 study: two steps forward, one step back? . *Bull World Health Organ*, 92: 226–.

Vos T, e. a. (2012). Years lived with disability (YLDs) for 1160 sequelae of 289 diseases and injuries 1990–2010: a systematic analysis for the Global Burden of Disease Study 2010. . *The Lancet 2012*, 380: 2163–96.

Zheng, P. (2021). Trimmed Constrained Mixed Effects Models: Formulations and Algorithms. *Journal of Computational and Graphical Statistics*, 544-556, DOI: 10.1080/106.

. Murray & Aleksandr Y. Aravkin (2021) Trimmed Constrained Mixed Effects Models: Formulations and Algorithms, Journal of Computational and Graphical Statistics, 30:3, 544-556, DOI: 10.1080/10618600.2020.1868303

Burstein R, Fleming T, Haagsma J, Salomon JA, Vos T, Murray CJL. Estimating distributions of health state severity for the global burden of disease study. *Popul Health Metr* 2015; **13**: 31.

Medical Expenditure Panel Survey Home. https://meps.ahrq.gov/mepsweb/ (accessed Nov 9, 2023).

4326.0 - Mental Health and Wellbeing: Profile of Adults, Australia, 1997. 1998; http://www.abs.gov.au/ausstats/abs@.nsf/ProductsbyReleaseDate/D5A0AC778746378FCA2574EA00122887?OpenDocument (accessed Nov 9, 2023).

Salomon JA, Haagsma JA, Davis A, *et al.* Disability weights for the Global Burden of Disease 2013 study. *Lancet Glob Health* 2015; **3**: e712–23.

Campeau L. The Canadian Cardiovascular Society grading of angina pectoris revisited 30 years later.

Kretzschmar M, Mangen M-JJ, Pinheiro P, *et al.* New Methodology for Estimating the Burden of Infectious Diseases in Europe. *PLOS Med* 2012; **9**: e1001205.

Wang H, Naghavi M, Allen C, *et al.* Global, regional, and national life expectancy, all-cause mortality, and cause-specific mortality for 249 causes of death, 1980–2015: a systematic analysis for the Global Burden of Disease Study 2015. *The Lancet* 2016; **388**: 1459–544.

Acne vulgaris

Flowchart for acne vulgaris

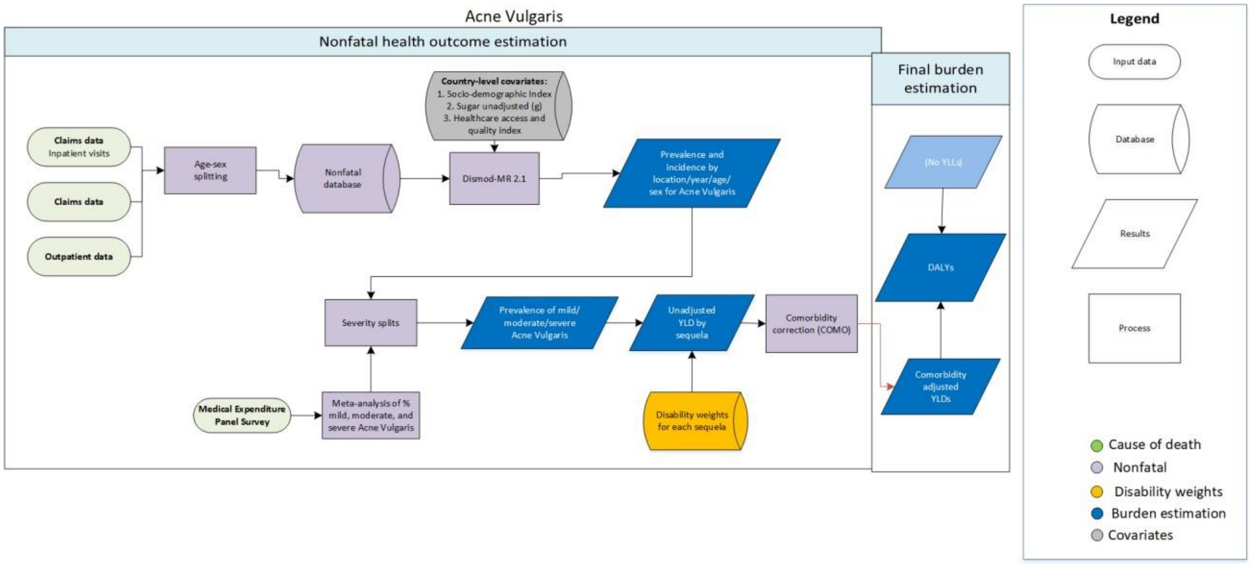

Input data and methodological summary for acne vulgaris

Case definition

Acne vulgaris is defined as a chronic inflammatory disease of the pilosebaceous unit associated with an increase in sebum secretion (ICD-10: L70, excluding L70.4). Acne vulgaris was included in the GBD 2021 cause group of skin and subcutaneous conditions.

Quantity of interest	Reference or Alternative	Definition
Acne vulgaris	Reference	Acne vulgaris diagnosed with a physical exam or ICD-10 coded claims data.
Acne vulgaris	Alternative	Acne vulgaris diagnosed without a physical exam. Includes outpatient and claims data prior to the year 2010.

Input data

In the GBD 2010 study, a systematic review of the literature was conducted using PubMed and Google Scholar to capture epidemiological data for acne vulgaris. The inclusion criteria stipulated that studies (1) must be published between 1980 and 2012; (2) must provide data on the incidence or prevalence of acne vulgaris; (3) must use samples representative of the general population (ie, samples derived from the experimental arm of clinical trials or based in dermatology clinics were excluded); (4) must use a sample size larger than 100; and (5) must provide sufficient information on study method and sample characteristics to assess the quality of the study. For GBD 2016, the GBD 2010 search strategy was replicated in PubMed to capture epidemiological studies published between 2013 and 2016. An additional literature search was carried out for GBD 2017 for USA data to better inform the DisMod crosswalk from USA claims data to literature data and capture any studies missed in previous literature searches. This

literature search also replicated the GBD 2010 search strategy and captured studies published between 1980 and 2017.

USA claims data from 2000 and 2010–2016 are included in this model, along with Poland claims data from 2015–2017, Taiwan (province of China) claims data from 2016, and outpatient data from Norway. USA outpatient data were not used due to implausibly high adjusted values.

Table 1: Data inputs for acne vulgaris morbidity modelling by parameter

**Cause/impairment name**	**Measure**	**Countries with data**	**New sources**	**Total sources**
Acne vulgaris	All measures	34	3	108
Acne vulgaris	Prevalence	34	3	93
Acne vulgaris	Proportion	1	0	15

Table 2: MR-BRT crosswalk adjustment factors for acne vulgaris

Data input	Reference or alternative case definition	Gamma	Beta coefficient, logit* (95% CI)	Adjustment factor**
Literature with physical exam and USA MarketScan 2010–2014	Reference	0.35	---	---
No physical exam	Alternative		1.47 (0.78 to 2.17)	0.81
USA MarketScan 2000	Alternative		–0.13 (–0.81 to 0.56)	0.47
Outpatient	Alternative		–2.49 (–3.19 to –1.79)	0.08

MR-BRT crosswalk adjustments can be interpreted as the factor the alternative case definition is adjusted by to reflect what it would have been had it been measured using the reference case definition. If the log/logit beta coefficient is negative, then the alternative is adjusted up to the reference. If the log/logit beta coefficient is positive, then the alternative is adjusted down to the reference.
****The adjustment factor column is the exponentiated beta coefficient. For log beta coefficients, this is the relative rate between the two case definitions. For logit beta coefficients, this is the relative odds between the two case definitions.***

Modelling strategy

DisMod-MR 2.1 was used to estimate prevalence by age, sex, year, and country for acne vulgaris.

Since our available data only contained information on prevalence, we specified additional expert priors to further inform analyses. We assumed zero excess mortality and remission from 0.38 to 0.6, implying a duration of approximately two to three years. This was in line with the available epidemiological data, expert opinion, and previous GBD work. A value prior of zero was set for incidence between the ages of 0 and 6, and 61 and 100. We used a time window of five years to determine which datapoints were used for a particular year of fit.

In GBD 2020, we replaced our within-DisMod crosswalks with crosswalks completed using the MR-BRT modelling tool. We adjusted outpatient data, along with data that were not based on physical exams

toward the level of other prevalence datapoints which were more representative of the general population. In addition, Socio-demographic Index, sugar consumption, and the Healthcare Access and Quality index were used as country-level covariates to guide estimates for countries with few or no data.

We have made no substantive changes in the modelling strategy from GBD 2019.

Table 3. Severity distribution, details on the severity levels for acne vulgaris and the associated disability weight (DW) with that severity

Sequela	Severity level	Lay description	DW (95% CI)
Mild acne vulgaris	Disfigurement, level 1	The individual has a slight, visible physical deformity that others notice, which causes some worry and discomfort.	0.011 (0.005–0.021)
Moderate acne vulgaris	Disfigurement, level 2	The individual has a visible physical deformity that causes others to stare and comment. As a result, the person is worried and has trouble sleeping and concentrating.	0.067 (0.044–0.096)
Severe acne vulgaris	Disfigurement, level 3	The individual has an obvious physical deformity that makes others uncomfortable, which causes the person to avoid social contact, feel worried, sleep poorly, and think about suicide.	0.405 (0.275–0.546)

Table 4. Covariates. Summary of covariates used in the acne vulgaris DisMod-MR meta-regression model

Covariate	Type	Parameter	Exponentiated beta (95% uncertainty interval)
Socio-demographic Index	Country-level	Prevalence	2.65 (2.57–2.71)
Sugar, unadjusted (g)	Country-level	Prevalence	1.00 (1.00–1.00)
Healthcare Access and Quality Index	Country-level	Prevalence	1.00 (1.00–1.00)

Acute glomerulonephritis

Flowchart

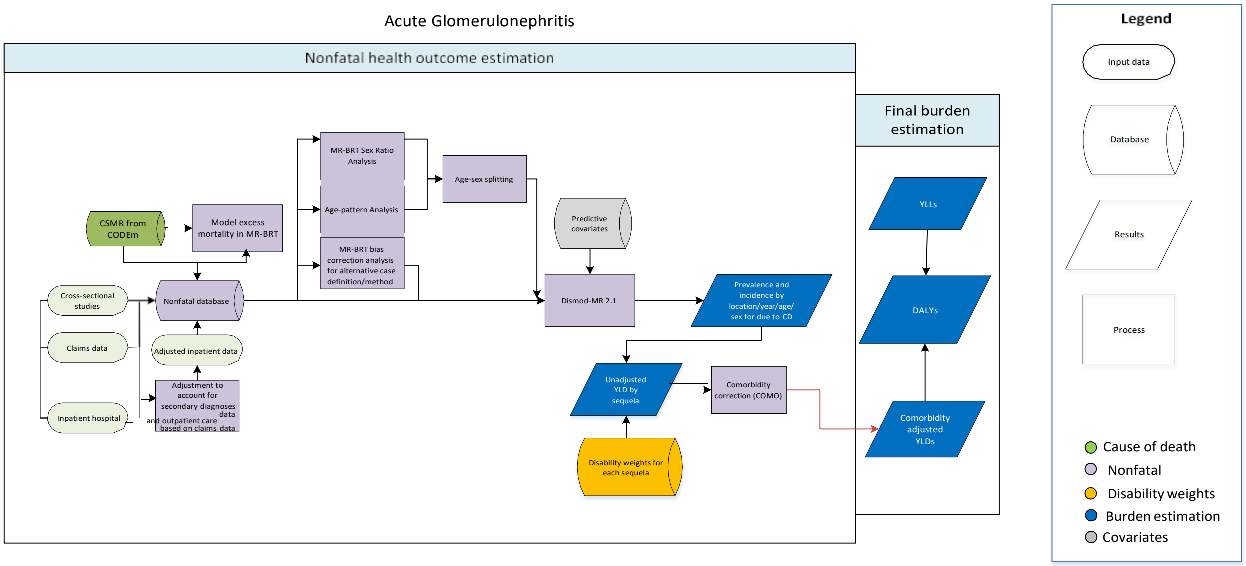

Input data and methodological summary for acute glomerulonephritis

Case definition

Acute glomerulonephritis (AG) is an acute episode of glomerular injury accompanied by inflammation, generally presenting with haematuria, oedema, hypertension, and acute kidney injury. ICD codes for AG include N00, N00.0, N00.1, N00.2, N00.3, N00.4, N00.5, N00.6, N00.7, N00.8, N00.9, N01, N01.1, N01.2, N01.3, N01.4, N01.5, N01.6, N01.7, N01.8, and N01.9.

In GBD 2017, our reference case definition for AG was limited to post-infectious AG; data sources that included other aetiologies of AG were adjusted to this reference standard. Similar to GBD 2019, in GBD 2021, the reference case definition was based on ICD diagnosis in administrative data and thus was not specific to a single aetiology.

Input data and data processing

Input data

A systematic literature review was first conducted in 2010, and again in 2013, extracting a total of 14 articles. In addition to claims and hospital discharge data used in GBD 2019, in GBD 2021, we newly added additional years of data from USA claims (year 2017) and Poland claims (year 2018), as well as hospital discharges in Greece, Armenia, Chile, Ecuador, Argentina, Italy, Brazil, and Spain.

Table 1. Data inputs for acute glomerulonephritis morbidity modelling by parameter.

	**Countries with data**	**New sources**	**Total sources**
Incidence	49	40	328

Inputs to our non-fatal modelling also included cause-specific mortality rate (CSMR) estimates taken from our fatal modelling process (see CoD cause-specific modelling description for AG in this appendix) and excess mortality rates (EMR) estimates modelled outside of DisMod-MR (see the EMR data processing section below).

Incidence data processing

Hospital discharge data provide observations about encounters, generally with only the primary diagnostic code for the encounter. Claims data, on the other hand, link claims for all inpatient and outpatient encounters for a single individual and provide primary and secondary diagnoses for all encounters.

In GBD 2017, an individual was extracted from claims data as an incident case if that individual had one or more inpatient encounters with an appropriate ICD code as any diagnosis. Hospital discharges with an appropriate ICD code as primary diagnosis were extracted and adjusted for readmissions.

In both GBD 2019 and GBD 2021, however, we employed data processing methods to capture cases that were diagnosed and/or treated in both inpatient and outpatient settings. Specifically, incident cases were extracted from claims data if an individual had at least one inpatient or outpatient encounter with an appropriate ICD code as any diagnosis; repeat encounters within 90 days, regardless of setting, were assumed to represent care for the same episode. Hospital discharge data were processed by extracting discharges with an appropriate ICD code as primary diagnosis and adjusting using correction factors (ie, correction factor 3) derived from claims data. Specifically, we modelled from the ratio of inpatient claims with AG as primary diagnosis to total incident cases of AG seen in claims data.

In addition to the improved case ascertainment of AG, the methods for bias adjustment were updated in GBD 2019 to allow a more direct comparison between different case definitions and/or study designs. In past GBD cycles, we used data from published studies that employed rigorous case definitions for post-infectious AG as our reference standard and adjusted clinical administrative data toward this reference standard by marking administrative data with binary covariates, and estimating a fixed effect for this covariate in our DisMod-MR meta-regression modelling process. This amounts to adjusting data using an ecological comparison and is vulnerable to compositional bias; if data from different location-years were collected using different methods or case definitions, true spatiotemporal differences in epidemiology can be erroneously adjusted, and differences truly due to differences in methods can be erroneously estimated as differences in underlying epidemiology. In GBD 2019, we avoided this risk by making pre-modelling bias adjustments and dropping data types that could not be rigorously adjusted. This was done by conducting a meta-regression of the relationship between datapoints matched with regard to year, age, sex, and location, but differing with regard to one or more study design characteristic. Data from studies that ascertained cases of post-infectious AG based on serological, histological, and/or imaging findings were scarce, and we were not able to find overlapping datapoints from administrative data sources to estimate adjustment factors. As a result, these data were excluded and a new case definition was adopted: diagnosis of AG of any aetiology as indicated by ICD code in a clinical encounter.

As first done in GBD 2019, USA claims data (extracted and processed as described above) were adjusted to account for selection bias due to commercial insurance using MR-BRT analysis. The table below shows these bias correction factors. Beta coefficients and adjustment factors incorporate study heterogeneity (gamma).

Table 2. MR-BRT crosswalk adjustment factors for acute glomerulonephritis

Data input	Reference or alternative data collection	Gamma	Beta coefficient, log (95% CI)	Adjustment factor*
Hospital + non-USA claims	Ref	0.33	---	---
USA claims from	Alt		1.83	6.21

year 2000			(–0.11 to 3.77)	(0.89–43.18)

USA claims from years 2010–2017	Alt		1.83 (0.96–2.70)	6.23 (2.61–14.89)

**Adjustment factor is the transformed beta coefficient in normal space and can be interpreted as the factor by which the alternative case definition is adjusted to reflect what it would have been if measured as the reference.*

Datapoints with an age-standardised incidence rate greater than 1.5 median absolute deviations from the median of the age-standardised incidence rate for all inpatient and non-USA claims data were marked as outliers and excluded from analysis. Hospital discharge data from Latvia, Meghalaya, Jordan, Qatar, Iran, and Turkey, and claims data from Poland were also marked as outliers because their estimates were implausibly high when compared to regional, super-regional, and global rates.

EMR input processing

In GBD 2017, EMR inputs were produced by matching prevalence datapoints with their corresponding CSMR values within the same age, sex, year, and location (by dividing CSMR by prevalence). For short-duration conditions (remission >1), the corresponding prevalence was derived by running an initial model and then applying the same CSMR/prevalence method. However, this method of producing EMR inputs demonstrated a rather unrealistic pattern of EMR compared to an expected pattern of decreasing EMR with greater access to quality health care. Such unexpected patterns often signal inconsistencies between CSMR estimates and the measures of prevalence and/or incidence. Thus, in an effort to provide greater guidance on the expected pattern of EMR, in GBD 2019, EMR data produced per above in GBD 2017 were modelled by age, sex, and Healthcare Access and Quality (HAQ) Index using MR-BRT, with a prior on HAQ Index having a negative coefficient. In GBD 2021, we employed the same MR-BRT method to predict EMR for each location, year, sex, and for ages 0, 10, 20….100; these predictions were used as inputs to our non-fatal model, below.

Modelling strategy

DisMod-MR model

Similar to previous rounds, we ran a DisMod-MR 2.1 model to produce estimates by age, sex, year, and location. Inputs to DisMod-MR for AG include incidence, CSMR, and EMR inputs processed as described above. Prior settings in the DisMod-MR model included setting remission of three to four weeks. It was assumed that no one was born with AG. The minimum coefficient of variation at the regional, super-regional, and global-level was set at 0.8. The HAQ Index was included as a predictive covariate on EMR. The beta and exponentiated values of this predictive covariate (which can be interpreted as an odds ratio) are shown in the table below.

Table 3. Covariates. Summary of covariates used in the acute glomerulonephritis DisMod-MR meta-regression model

Covariate	Parameter	Exponentiated beta (95% uncertainty interval)
HAQ Index	Excess mortality rate	0.97 (0.97–0.97)

Severity split and disability weight

The basis of the GBD disability weight assessment is lay descriptions of sequelae highlighting major functional consequences and symptoms. Disability weighting (DW) for AG associates with systemic

symptoms of fever, aches, weakness, and some difficulty with daily activities. The lay description and disability weight for acute glomerulonephritis are shown below.

Table 4. Severity distribution, details on the severity levels for acute glomerulonephritis in GBD 2021 and the associated DW with that severity.

Severity level	Lay description	DW (95% CI)
Moderate	Has a fever and aches, and feels weak, which causes some difficulty with daily activities.	0.051 (0.032–0.074)

Acute hepatitis: A, B, C, and E

Acute hepatitis A

Flowchart

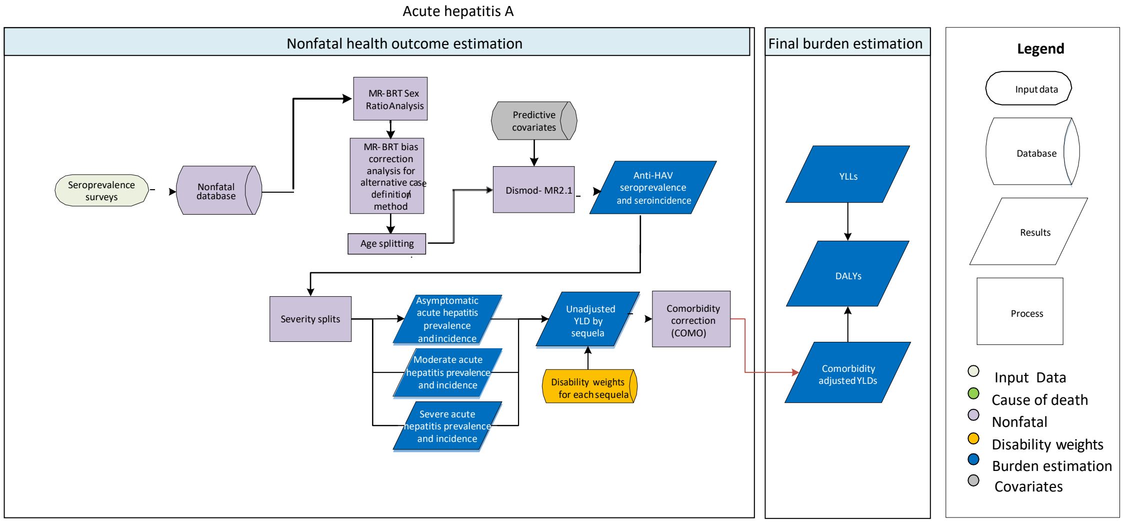

Input data and methodological summary for hepatitis A

Case definition

We define acute hepatitis A as an infection with the hepatitis A virus resulting in anti-HAV IgG seroconversion, regardless of symptoms.

Input data

Seroprevalence data inputs

We use anti-hepatitis A virus (HAV) seroprevalence data from population-based studies and surveys to estimate seroprevalence and seroincidence. The last systematic review was performed as part of GBD 2013. Additional data sources provided by collaborators were included in GBD 2019. No data changes were made as part of GBD 2021.

Table 1: Prevalence data inputs for anti-HAV seroprevalence modelling

Measure	Countries with data	Total sources
Prevalence	117	472

Data processing

Because we produce sex-specific estimates, we adjusted data that reported on both sexes into male and female sex-specific estimates. We identified studies that reported on sex-specific data and calculated the log ratio of female to male prevalence from studies that report sex-specific prevalence, modelling these log ratios in meta-regression—Bayesian, regularised, trimmed (MR-BRT), a regression tool developed at IHME. We then used the modelled sex ratio to adjust "both"-sex data values to expected "male" and "female" values. We calculated the male values as $val_{male} = val_{both} * \frac{pop_{both}}{(pop_{male}+ratio*pop_{female})}$. We calculated female values $val_{female} = ratio * val_{male}$.

Table 2: MR-BRT sex ratios for hepatitis A

Cause	Beta coefficient, log (95% UI)
Hepatitis A	0.008 (–0.027 to 0.042)

**MR-BRT crosswalk adjustments can be interpreted as the factor the alternative case definition is adjusted by to reflect what it would have been had it been measured using the reference case definition. If the log/logit beta coefficient is negative, then the alternative is adjusted up to the reference. If the log/logit beta coefficient is positive, then the alternative is adjusted down to the reference.*

We took as our reference population the general population of unvaccinated individuals living in a certain location in a certain year. Adjustment factors were estimated and applied prior to modelling to those prevalence data collected using non-reference study populations of pregnant women, blood donors, and a mix of vaccinated and unvaccinated individuals. Data were matched (by year, age, sex, location) for reference population and alternative populations, and their systematic differences were modelled using MR-BRT.

The process of adjusting for biases in non-reference data using MR-BRT with the logit-transformation method is described below:

1. Identify datapoints with overlapping year, age, sex, and location between reference and non-reference population data.
2. Logit transform overlapping datapoints of alternative and reference types.
3. Convert overlapping datapoints into a difference in logit space using the following equation: $logit(alternative) - logit(reference)$.
4. Use the delta method to compute standard errors of overlapping datapoints in logit space, then calculate standard error of logit difference using the following equation: $\sqrt{(variance\ of\ alternative) + (variance\ of\ reference)}$.
5. Using MR-BRT, conduct a random effects meta-regression to obtain the pooled logit difference of alternative to reference.
6. Apply the pooled logit difference to all datapoints of alternative case definitions using the following equation:
$$new_{estimate} = inverse.\,logit((logit(alternative)) - (pooled\ logit\ difference)).$$
7. Calculate new standard errors using the delta method, accounting for gamma (between-study heterogeneity).

Table 3: MR-BRT crosswalk factors for anti-HAV seroprevalence non-representative populations

Data input	Reference or alternative case definition	Gamma	Beta coefficient, logit (95% CI)
General population	Reference	0.87	---
Blood donors	Alternative		0.85 (–0.95 to 2.58)
Pregnant women	Alternative		1.31 (–1.18 to 3.80)

**MR-BRT crosswalk adjustments can be interpreted as the factor the alternative case definition is adjusted by to reflect what it would have been had it been measured using the reference case definition. If the log/logit beta coefficient is negative, then the alternative is adjusted up to the reference. If the log/logit beta coefficient is positive, then the alternative is adjusted down to the reference.*

Table 4: MR-BRT crosswalk factors for anti-HAV seroprevalence vaccination status

Data input	Reference or alternative case definition	Gamma	Beta coefficient, logit (95% CI)
Unvaccinated study sample	Reference	1.01	---
Study sample included both vaccinated and unvaccinated individuals	Alternative		0.59 (–1.41 to 2.61)

**MR-BRT crosswalk adjustments can be interpreted as the factor the alternative case definition is adjusted by to reflect what it would have been had it been measured using the reference case definition. If the log/logit beta coefficient is negative, then the alternative is adjusted up to the reference. If the log/logit beta coefficient is positive, then the alternative is adjusted down to the reference.*

We adjusted broad age-group data into 5-year age bins using an estimated age pattern, a continued strategy from GBD 2019. Data in which the age range was greater than 25 years was categorised as broad age range data. We assumed the age-distribution in the study sample was the same as the estimated population in GBD 2019. We also assumed that the ratios of age-specific prevalence to full age-prevalence was the same as the seroprevalence model from GBD 2019.

Modelling strategy

DisMod model of anti-HAV IgG seroprevalence

No changes were made to the modelling strategy in GBD 2021. We model the seroprevalence of anti-HAV IgG using a DisMod-MR 2.1 model. Remission and excess mortality value priors of zero were used, and an incidence value prior range between 0 and 0.5 was used. Additionally, a relative-risk-weight summary exposure variable for diarrhea risk factors was included as a predictive covariate in the DisMod model to inform estimates for location-years with little or no primary data, with the coefficient in the fitted model shown below.

Table 5: Summary of country-level covariates used in the anti-HAV seroprevalence DisMod-MR 2.1 model

Covariate	Parameter	Exponentiated beta (95% CI)
Log-transformed age-standardised SEV* scalar: diarrhoea	Prevalence	1.29 (1.27–1.32)

*Summary exposure value

Acute hepatitis A incidence and severity distribution estimation

Given its reasonably stable force of infection among susceptible people across age groups, we derive seroincidence from the seroprevalence estimates using the following formula:

$$incid = \frac{-\ln(1 - prev)}{\text{age}_{\text{mid}}} * (1 - prev)$$

We calculate acute symptomatic infections by multiplying incidence of seroconversion by the probability of an acute infection being symptomatic. The probability of symptomatic infection comes from Armstrong and Bell[1] and is shown in the figure below (where probability of symptomatic infection is represented as "probability of jaundice"). The probability increases with age from ~1% in the first year of life to ~85% in adulthood. The probability function is:

$$Prob\ (symptomatic) = 0.852 * (1 - e^{-0.01244\ *\ age^{1.903}})$$

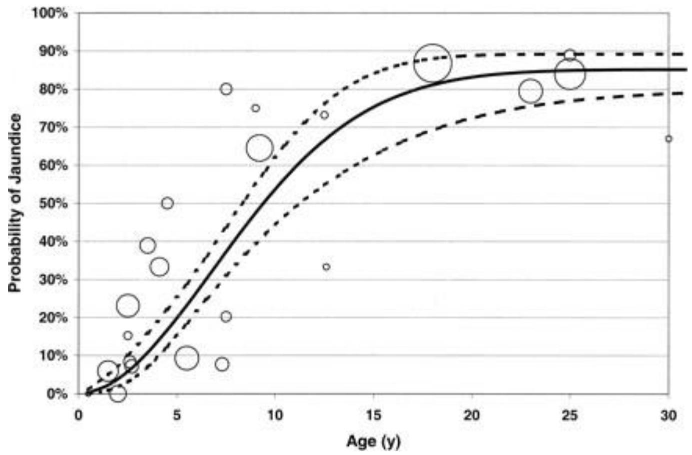

The remainder of acute infections are assumed to be asymptomatic.

We then base severity splits for moderate and severe on expert opinion that the probability of severe infection follows a beta distribution with mean 0.6% (the below table reports percentiles of this distribution). We assume the rest of symptomatic infections are moderate.

Table 5: Severity distribution of acute hepatitis A

0 percentile	25 percentile	50 percentile	75 percentile	100 percentile
0.0024	0.0054	0.006	0.007	0.01

Health states and disability weights

The table below illustrates the sequelae associated with acute hepatitis A, as well as the lay descriptions and associated disability weights.

Table 6: Disability weights

Sequela	Description	Disability weight (95% CI)
Moderate	Has a fever and aches, and feels weak, which causes some difficulty with daily activities.	0.051 (0.032–0.074)
Severe	Has a high fever and pain, and feels very weak, which causes great difficulty with daily activities.	0.133 (0.088–0.19)
Asymptomatic	Infection with no apparent illness.	NA

Acute hepatitis B

Flowchart

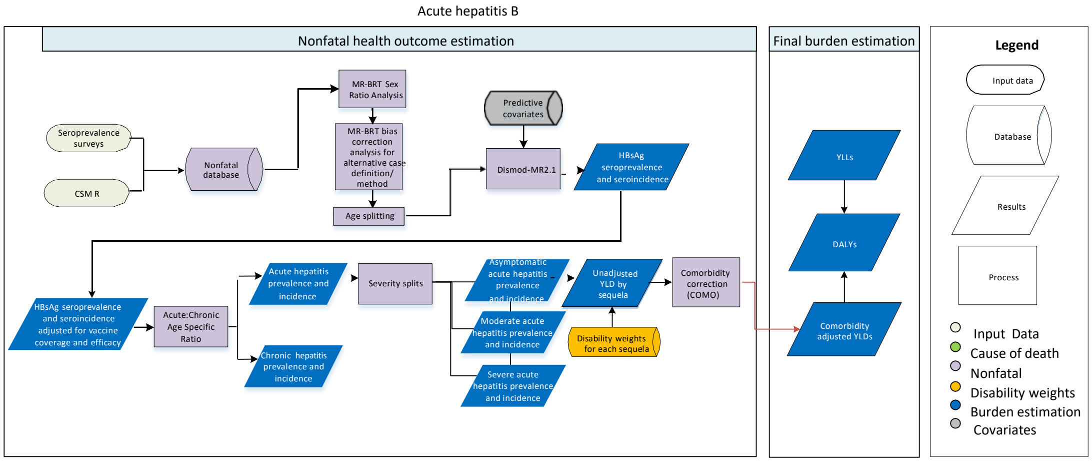

Input data and methodological summary for hepatitis B

Case definition

We define acute hepatitis B as the period corresponding to initial infection with the hepatitis B virus, regardless of symptoms.

Input data

Seroprevalence data inputs

We use hepatitis B surface antigen (HBsAg) seroprevalence data from population-based studies and surveys. The last systematic review conducted by IHME was performed as part of GBD 2013. This round, we completed an effort started in GBD2019 to align data sources with those identified in Schweitzer and colleagues.[2]

Figure 1: PRISMA diagram of HBsAg sources

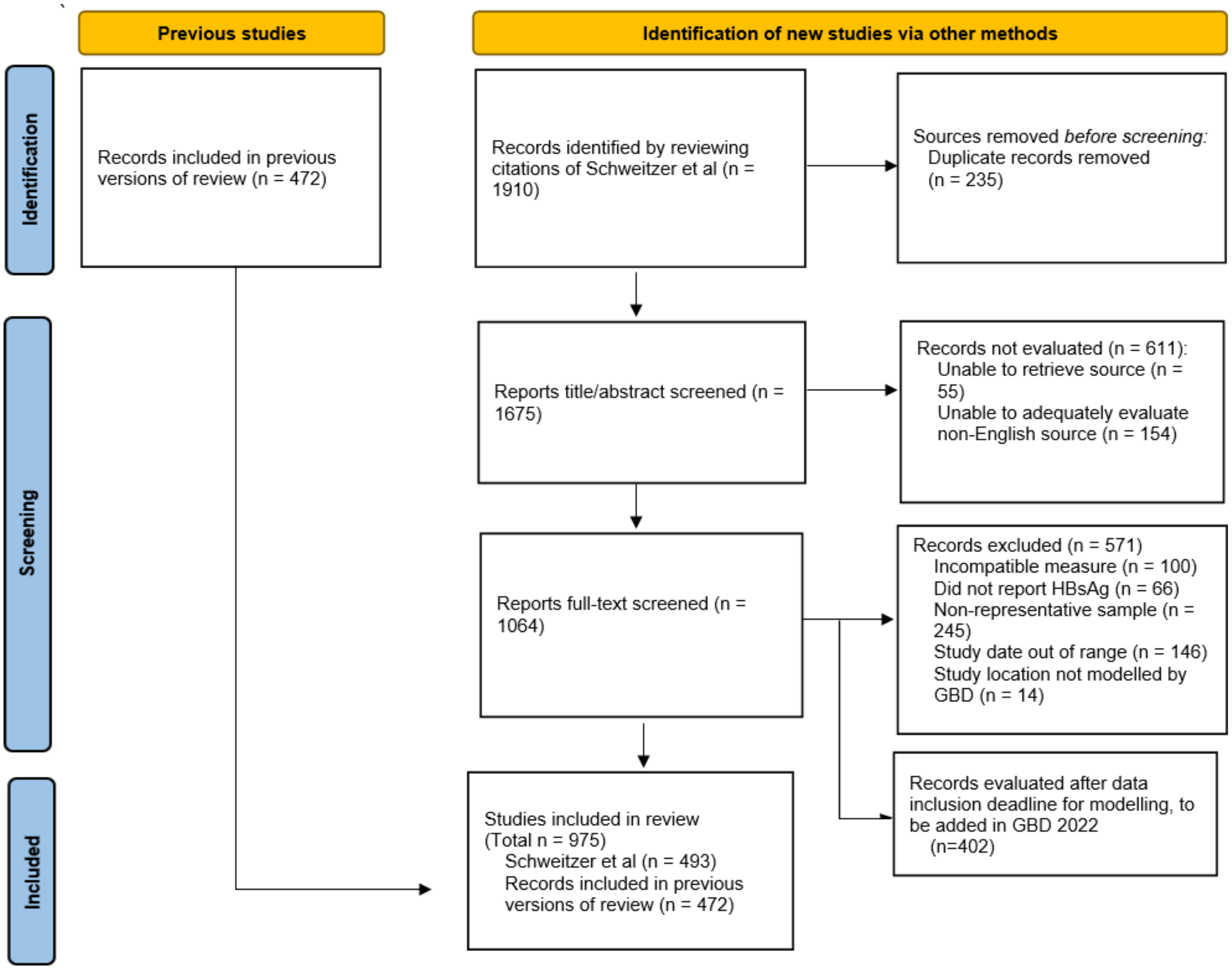

Table 1: Prevalence data inputs for HBsAg seroprevalence modelling

Measure	Countries with data	New sources	Total sources
**Prevalence**	124	491	948

Cause-specific mortality rate (CSMR)inputs

We included CSMR inputs to our DisMod compartmental model for estimating seroprevalence of HBsAg. To obtain these, we summed CoDCorrected-CSMR estimates for acute hepatitis B, cirrhosis due to hepatitis B, and liver cancer due to hepatitis B from the GBD fatal estimation processes.

Data processing

Seroprevalence data processing

We modelled HBsAg seroprevalence using a multi-step approach. First, we subset our database to only data from unvaccinated populations and performed pre-modelling bias adjustments. Then, we fit a "counterfactual" HBsAg seroprevalence model, using only these data from unvaccinated populations in a DisMod-MR 2.1 compartmental model to obtain estimates of what the incidence and prevalence of chronic carriage would be in a steady state without vaccine intervention. Next, we modified those results

using estimates of hepatitis B vaccine coverage and efficacy to obtain final estimates incidence and

prevalence of chronic hepatitis B carriage. Finally, we used natural history studies to infer what the total incidence of acute hepatitis B was from the incidence of chronic carriage. These processes are described in more detail below.

The rationale for this approach is as follows: Prior to GBD 2019, the DisMod-MR 2.1 model of hepatitis B surface antigen positivity, using all available data, tended to follow the data from unvaccinated populations, and poorly fit prevalence data from vaccinated populations at younger ages. DisMod-MR 2.1 assumes that diseases are steady state and employs data from a pre-set time window for the estimates for a given year. For example, estimates for the year 2000 can be set to utilise data from 1990 to 2010, 1995 to 2005, 1998 to 2002, and so on. Despite attempts to narrow the time window to between two- and five-year intervals, the model still did not capture rapid changes in seroprevalence that have resulted from vaccine uptake and cohort effects. In GBD 2019, we changed the modelling strategy to a counterfactual model to estimate what seroprevalence would be in the absence of vaccination efforts, and then adjusted by removing seroprevalent cases based on infant vaccine coverage and efficacy. The result of this process fit data from vaccinated cohorts better than DisMod models that were fit using all data, and so we continued this approach in GBD 2021.

Seroprevalence studies were excluded if all or at least 50% of a normal distribution of study participants were born after the location-specific year of hepatitis B three-dose vaccine introduction. Data collected from vaccinated populations were retained in the database to verify that subsequent modelling steps adequately accounted for the effect of vaccine programmes. Prior to modelling, we performed several data adjustments to correct for non-reference data-collection methods, including sex-splitting, crosswalking, and age-splitting.

Because we produce sex-specific estimates, we adjusted data that reported on both sexes into male and female sex-specific estimates. We identified studies that reported on sex-specific data and calculated the log ratio of female-to-male prevalence from studies that report sex-specific prevalence, modelling these log ratios in MR-BRT. We then used the modelled sex ratio to adjust “both”-sex data values to expected “male” and “female” values. We calculated the male values as $val_{male} = val_{both} * \frac{pop_{both}}{(pop_{male} + ratio * pop_{female})}$. We calculated female values $val_{female} = ratio * val_{male}$.

Table 2: MR-BRT sex ratios for HBsAg

Model	Beta coefficient, log (95% CI)	Gamma
HBsAg seroprevalence	–0.359 (–0.383 to –0.335)	0.0013

**MR-BRT crosswalk adjustments can be interpreted as the factor the alternative case definition is adjusted by to reflect what it would have been had it been measured using the reference case definition. If the log/logit beta coefficient is negative, then the alternative is adjusted up to the reference. If the log/logit beta coefficient is positive, then the alternative is adjusted down to the reference.*

Adjustment factors were estimated and applied prior to modelling to those prevalence data collected using non-reference study populations of pregnant women and blood donors. Data were matched (by year, age, sex, location) for general population and alternative populations, and their systematic differences were modelled using MR-BRT.

The process of adjusting for biases in non-reference data using MR-BRT with the logit-transformation method is described below:

1. Identify datapoints with overlapping year, age, sex, and location between reference and non-reference population data.

2. Convert overlapping datapoints into a difference in logit space using the following equation: $logit(alternative) - logit(reference)$.
3. Use the delta method to compute standard errors of overlapping datapoints in logit space, then calculate standard error of logit difference using the following equation: $\sqrt{(variance\ of\ alternative) + (variance\ of\ reference)}$.
4. Using MR-BRT, conduct a random effects meta-regression to obtain the pooled logit difference of alternative to reference.
5. Apply the pooled logit difference to all datapoints of alternative case definitions using the following equation: $new_{estimate} = inverse.\,logit((logit(alternative)) - (pooled\ logit\ difference))$.
6. Calculate new standard errors using the delta method, accounting for gamma (between-study heterogeneity).

Table 3: MR-BRT crosswalk factors for HBsAg seroprevalence non-representative populations

Data input	Reference or alternative population	Gamma	Beta coefficient, logit (95% CI)
General population	Reference	0.359	---
Blood donors	Alternative		–0.099 (–1.327 to 1.129)
Pregnant women	Alternative		0.0097 (–1.263 to 1.283)

**MR-BRT crosswalk adjustments can be interpreted as the factor the alternative case definition is adjusted by to reflect what it would have been had it been measured using the reference case definition. If the log/logit beta coefficient is negative, then the alternative is adjusted up to the reference. If the log/logit beta coefficient is positive, then the alternative is adjusted down to the reference.*

We adjusted broad age-group data into five-year age bins using an estimated age pattern. Data in which the age range was greater than 25 years was categorised as broad age range data. We assumed the age-distribution in the study sample was the same as the estimated population in GBD 2019. We also assumed that the ratios of age-specific prevalence to full age prevalence was the same as the seroprevalence model from GBD 2019.

Modelling strategy

Counterfactual seroprevalence model

We estimated HBsAg seroprevalence using DisMod-MR 2.1. We used the processed data described previously to generate location-age-sex-year-specific estimates. In addition to HBsAg seroprevalence and CSMR data, we included predictive covariates in the model to improve estimation in quantities of interest where data are absent or scarce. We included remission priors between 0 and 0.02, excess mortality prior between 0 and 0.1, and incidence priors between 0 and 0.05 for all ages. The summary of covariates using in the counterfactual HBsAg seroprevalence DisMod-MR 2.1 model are listed below.

Table 4: Summary of predictive covariates used in the HBsAg seroprevalence DisMod-MR 2.1 model

Covariate	Parameter	Exponentiated beta (95% CI)
Log-transformed age-standardised SEV scalar: hepatitis B	Prevalence	1.04 (1.00–1.16)
Socio-demographic Index	Prevalence	0.15 (0.14–0.16)
Healthcare Access And Quality Index	Excess mortality rate	1.00 (1.00–1.00)

Adjustment for vaccination effects

After the completion of the counterfactual DisMod model, a post-hoc adjustment was performed to modify estimates of HBsAg seropositivity based on GBD produced location-year specific hepatitis B three-dose vaccine coverage and vaccine efficacy. The proportion of coverage by location and year was multiplied by vaccine efficacy of 95% to get the proportion of the population protected. Protected individuals were subtracted from the HBsAg cases estimated in the counterfactual DisMod model to get final estimates of HBsAg prevalence and incidence.

An example of the counterfactual DisMod-MR 2.1 estimates and vaccine-adjusted estimates in comparison to included and excluded datapoints is shown in Figure 2.

Figure 2. Comparisons of counterfactual and vaccine-adjusted estimates to included and excluded datapoints

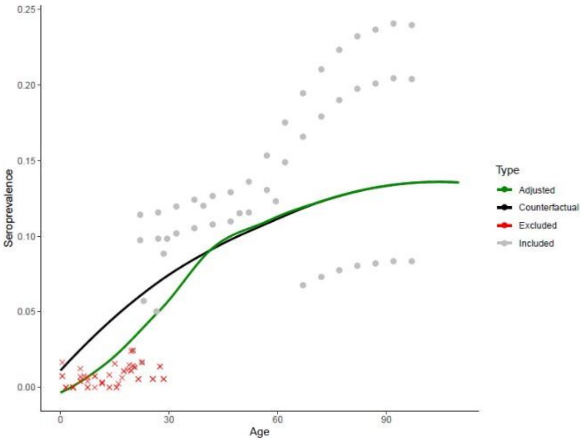

These final estimates of HBsAg seroprevalence serve as inputs to models for several entities, as described in the methods appendix sections on the estimation of the fatal and non-fatal burden of cirrhosis and other chronic liver diseases and liver cancer. The remainder of this section only discusses how HBsAg seroprevalence estimates are used to estimate acute hepatitis B infection.

Acute hepatitis B incidence and severity distribution estimation

The incidence obtained from the DisMod model of HBsAg is regarded as the incidence of chronic carriage. This is converted to the total incidence of hepatitis B infection by dividing age-specific estimates of the incidence of chronic carriage by age-specific estimates of the probability of infection resulting in carriage based on Edmunds and colleagues:[3]

$$P(carrier \mid age \ \leq 6 \ months) = \ 0.885$$

$$P(carrier \mid 6 \ months \leq age < 25 \ years) = e^{-0.645 \ \times age^{0.455}}$$

$$P(carrier \mid age \geq 25 \ years) = e^{-0.645 \ \times 25^{0.455}} = 0.061$$

We then split symptomatic cases into moderate (73%) and severe (27%) based on data from McMahon and colleagues.[4] We then assigned the moderate and severe cases the following health states and disability weights.

Health states and disability weights

The table below illustrates the sequelae associated with acute hepatitis B, as well as the lay descriptions and associated disability weights.

Table 5: Disability weights

Sequela	Description	Disability weight
Moderate	Has a fever and aches, and feels weak, which causes some difficulty with daily activities.	0.051 (0.032–0.074)
Severe	Has a high fever and pain, and feels very weak, which causes great difficulty with daily activities.	0.133 (0.088–0.19)
Asymptomatic	Infection with no apparent illness.	NA

Acute hepatitis C

Flow chart

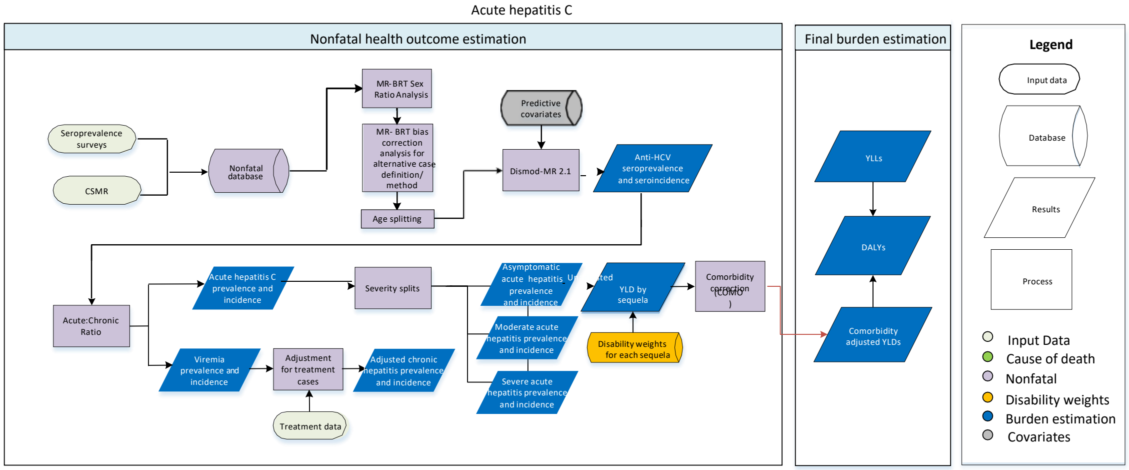

Input data and methodological summary for hepatitis C

Case definition

We define acute hepatitis C as the period corresponding to initial infection with the hepatitis C virus, resulting in anti-HCV IgG seroconversion, regardless of symptoms.

Input data and processing

Seroprevalence data

To estimate morbidity for hepatitis C, we use anti-HCV seroprevalence data from population-based studies and surveys to estimate seroincidence and seroprevalence. The last systematic review performed by IHME was part of GBD 2013. We added new data sources from collaborator inclusions in subsequent rounds, most substantially in GBD 2019 when we added sources collated by the Center for Disease Analysis and identified in the systematic review by Blach 2016.[5]

CSMR inputs

We also use CSMR estimates for acute hepatitis C, cirrhosis, and other chronic liver diseases due to hepatitis C, and liver cancer due to hepatitis C from the GBD causes of death modelling process as inputs in our DisMod compartmental model of anti-HCV seropositivity.

Data on the ratio of anti-HCV seroprevalence to HCV viremia

In GBD 2019, we identified from our seroprevalence database 42 studies that reported on the prevalence of anti-HCV antibody and HCV-RNA in the same individuals, which we used as inputs to a meta-analysis of the ratio of seroprevalence to viraemia in untreated populations.

Treatment data

Additionally, we use hepatitis C treatment volumes to account for curative efforts. In GBD 2019, we included information on treatment for Egypt, Japan, and Australia. With collaboration from the Coalition for Global Hepatitis Elimination, we expanded our treatment database in GBD 2021. This round we added

data from 31 countries. These include Romania, Portugal, Latvia, Iceland, Slovenia, Spain, England, France,

Bulgaria, Croatia, Hungary, Wales, Rwanda, South Africa, Argentina, Brazil, Morocco, Pakistan, Georgia, Ukraine, Indonesia, Mongolia, Canada, China, Ghana, India, Ireland, Malaysia, Mexico, and Russia. We extracted data on age, sex, and treatment type (ie, direct-acting antivirals, interferon, triple drug) when available.

Table 1: Data inputs for hepatitis C modelling by parameter

Measure	Countries with data	New sources	Total sources
Prevalence	107	193	493
Other	30	46	83

Data processing

Seroprevalence data processing

Because we produce sex-specific estimates, we adjusted data that reported on both sexes into male and female sex-specific estimates. We identified studies that reported on sex-specific data and calculated the log ratio of female-to-male prevalence from studies that report sex-specific prevalence, modelling these log ratios in MR-BRT. We then used the modelled sex ratio to adjust "both"-sex data values to expected "male" and "female" values. We calculated the male values as $val_{male} = val_{both} * \frac{pop_{both}}{(pop_{male} + ratio * pop_{female})}$. We calculated female values $val_{female} = ratio * val_{male}$.

Table 2: MR-BRT sex ratio for anti-HCV

Model	Beta coefficient, log (95% CI)	Gamma
Anti-HCV seroprevalence	–0.04306 (–0.376 to 0.29)	0.028

Adjustment factors were estimated and applied prior to modelling to those prevalence data collected using non-reference study populations of blood donors. Data were matched (by year, age, sex, location) for general population and alternative populations, and their systematic differences were modelled using MR-BRT.

The process of adjusting for biases in non-reference data using MR-BRT with the logit-transformation method is described below:

1. Identify datapoints with overlapping year, age, sex, and location reference and non-reference population data.
2. Logit transform overlapping datapoints of alternative and reference types.
3. Convert overlapping datapoints into a difference in logit space using the following equation: $logit(alternative) - logit(reference)$.
4. Use the delta method to compute standard errors of overlapping datapoints in logit space, then calculate standard error of logit difference using the following equation: $\sqrt{(variance\ of\ alternative) + (variance\ of\ reference)}$.
5. Using MR-BRT, conduct a random effects meta-regression to obtain the pooled logit difference of alternative to reference.
6. Apply the pooled logit difference to all datapoints of alternative case definitions using the following equation: $new_{estimate} = inverse.logit((logit(alternative)) - (pooled\ logit\ difference))$.

7. Calculate new standard errors using the delta method, accounting for gamma (between-study heterogeneity).

Table 3: MR-BRT crosswalk factors for anti-HCV seroprevalence non-representative populations

Data input	Reference or alternative case definition	Gamma	Beta coefficient, logit (95% CI)
General population	Reference	0.187	---
Blood donors	Alternative		–0.603 (–1.52 to 0.315)

We adjusted broad age-group data into five-year age bins using an estimated age pattern, a continued strategy from GBD 2019. Data in which the age range was greater than 25 years was categorised as broad age range data. We assumed the age-distribution in the study sample was the same as the estimated population in GBD 2019. We also assumed that the ratios of age-specific prevalence to full age prevalence was the same as the seroprevalence model from GBD 2019.

Modelling strategy

DisMod model of anti-HCV seropositivity

We estimated anti-HCV seropositivity using DisMod-MR 2.1. We used the processed seroprevalence data described previously to generate location-age-sex-year-specific estimates. In addition to anti-HCV seroprevalence and CSMR data, we included predictive covariates in the model to improve estimation in quantities of interest where data are absent or scarce. We included remission priors of 0, and no incidence values for all ages. The summary of covariates used in the anti-HCV positivity DisMod-MR 2.1 model are listed below.

Table 4: Summary of covariates used in the anti-HCV seroprevalence DisMod-MR 2.1 model

Covariate	Parameter	Exponentiated beta (95% CI)
Log-transformed age-standardised SEV scalar: hepatitis C	Prevalence	2.46 (2.46–2.47)
Socio-demographic Index	Prevalence	0.14 (0.14–0.14)

Estimating the incidence and point-prevalence of acute hepatitis C infection

The incidence of anti-HCV IgG seroconversion from DisMod-MR was treated as the incidence of acute hepatitis C infection. The incident infections were divided into asymptomatic (75%), moderate (24%), and severe (1%) states based on expert opinion and assigned the following health states and disability weights. We assumed duration of six weeks based on content experts.

Table 5: Disability weights for acute hepatitis C

Sequela	Description	Disability weight
Moderate	Has a fever and aches, and feels weak, which causes some difficulty with daily activities.	0.051 (0.032–0.074)
Severe	Has a high fever and pain, and feels very weak, which causes great difficulty with daily activities.	0.133 (0.088–0.19)
Asymptomatic	Infection with no apparent illness.	NA

Estimating the prevalence of chronic hepatitis C (viremia)

Beyond estimating burden due to acute hepatitis C, the DisMod model of anti-HCV seroprevalence was used to estimate prevalence of chronic infection (viraemia), which serves as an input to multiple estimation processes described in separate sections of this appendix (fatal and non-fatal burden of cirrhosis and other liver disease and liver cancer). This was done in three steps: first, we estimated the ratio of anti-HCV seroprevalence to viraemia; second, we converted seroprevalence estimates from DisMod to estimates of viraemia by multiplying by this ratio estimate at the draw level; and third, we reduced the number of prevalent cases based on the number of cases treated by national programmes.

We used 42 studies that reported on the prevalence of anti-HCV antibody and HCV-RNA to produce a pooled estimate of proportion viraemic among the seropositive. This was used to correct outputs of our model of anti-HCV seropositivity to estimate viraemia. We examined the estimated coefficient based on super-region, particularly looking to see if there is a difference in the ratio of anti-HCV to HCV-RNA positivity in sub-Saharan Africa as suggested by expert collaborators. However, no difference was identified, and we used the same conversion factor globally. Below is a graph of the pooled estimated logit difference and logit difference and standard error of input studies.

Figure 1. Forest plot of studies that report both anti-HCV and HCV-RNA estimates

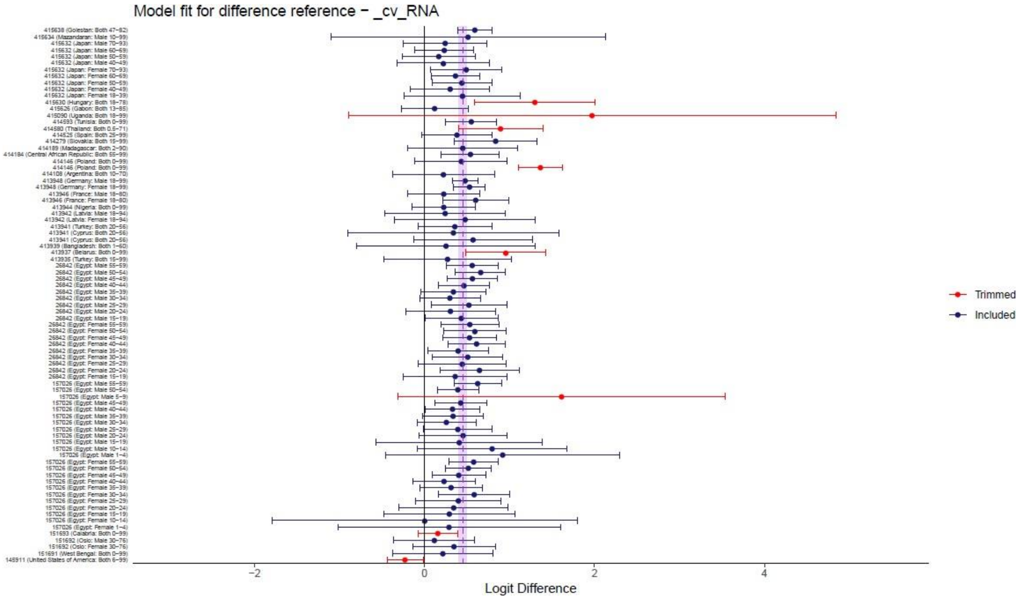

Treatment adjustment

We accounted for virus-clearing treatments of hepatitis C in locations where we had access to information on national treatment data. We processed treatment data by splitting both-sex data into sex-specific data and broad age-range data into five-year age bins using the age and sex distributions in our estimates of chronic HCV cases. (This implies equal access to treatment for all ages and both sexes whenever we have treatment data that do not report these characteristics.) We multiplied the number of cases treated by treatment efficacy (assumed 70% for pegylated interferon and 95% for direct-acting antiviral treatments). We subtracted the number of individuals treated in a year from the initial viraemic cases estimated in the steps above. The cumulative effect of treatment from year to year was the summation of treatment in the current year of treatment, as well as previous years and age groups, to ensure all years of treatment were reflected in final estimates. For locations where the treatment data did not include the final year of estimation, we assumed the proportion of cases treated in the last year of observed data was sustained until 2019 and assumed a decrease in treatment coverage in 2020 and 2021, as described below.

We identified treatment data from seven locations (Australia, Brazil, Canada, England, Georgia, Mexico, and Rwanda) that provided information on the number of people treated in 2020, 2021, or both. We used treatment data from these locations to estimate service disruption due to COVID-19. From these, we calculated an average reduction in treatment services of 19.6% in 2020 compared to 2019 and of 20.1% in 2021 compared to 2020.

In the locations for which we had treatment data from 2020 and 2021, the number of people treated in the COVID-19 pandemic years was calculated using its own treatment data; for locations where we had treatment data for 2020 but not 2021, we assumed the proportion of cases treated in 2020 was sustained in 2021. For the countries for which we had some years of treatment data prior to 2020, but not in the COVID-19 years, we applied the average treatment reduction rates (%) between 2019–2020 and 2020–2021 that were calculated based on the seven locations, as described above.

Acute hepatitis E

Flowchart

Acute hepatitis E

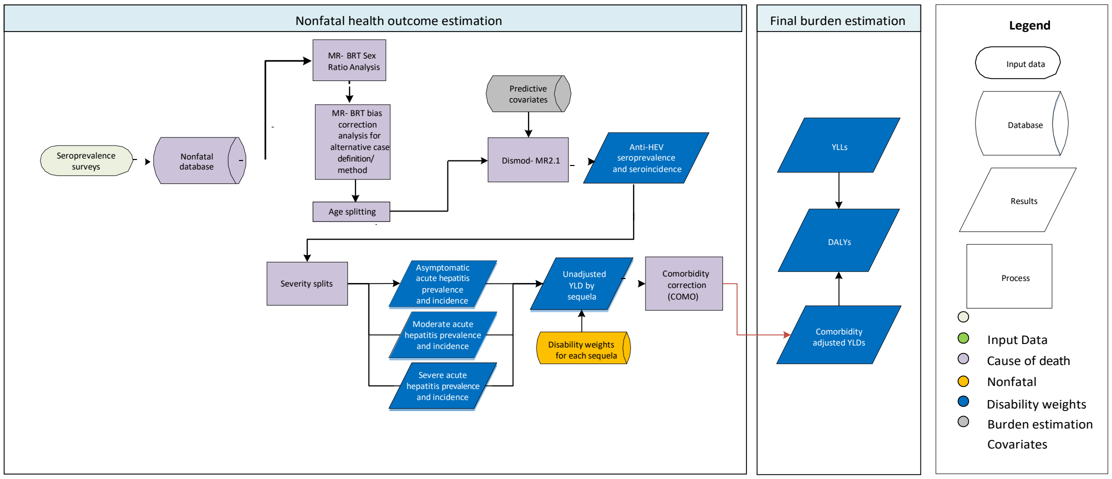

Input data and methodological summary for hepatitis E

Case definition

We define acute hepatitis E as an infection with the hepatitis E virus resulting in anti-HEV IgG seroconversion, regardless of symptoms.

Input data

We use anti-HEV seroprevalence data from population-based studies and surveys to estimate incidence of infection. The last systematic review was performed as part of GBD 2013.

Table 1: Data inputs for anti-HEV seroprevalence modelling

Measure	Countries with data	Total sources
Prevalence	44	81

Data processing

Seroprevalence data processing

Because we produce sex-specific estimates, we adjusted data that reported on both sexes into male and female sex-specific estimates. We identified studies that reported on sex-specific data and calculated the log ratio of female-to-male prevalence from studies that report sex-specific prevalence, modelling these log ratios in MR-BRT. We then used the modelled sex ratio to adjust "both"-sex data values to expected "male" and "female" values. We calculated the male values as $val_{male} = val_{both} * \frac{pop_{both}}{(pop_{male}+ratio*pop_{female})}$. We calculated female values $val_{female} = ratio * val_{male}$.

Table 2: MR-BRT sex ratio for anti-HEV

Model	Beta coefficient, log (95% CI)
Anti-HEV seroprevalence	–0.014 (–0.434 to 0.406)

Adjustment factors were estimated and applied prior to modelling to those prevalence data collected using non-reference study populations of blood donors. Data were matched (by year, age, sex, location) for general population and alternative populations, and their systematic differences were modelled using MR-BRT. There were insufficient matched studies of anti-HEV seroprevalence in alternative and reference populations from the same year-age-sex-location combinations to estimate an adjustment factor in MR-BRT. Thus, we combined matched pairs of studies of anti-HEV and matched pairs of studies of anti-HAV, to estimate an adjustment factor for all viral hepatitis with faecal-oral transmission and applied these adjustments to anti-HEV data collected by non-reference methods.

The process of adjusting for biases in non-reference data using MR-BRT with the logit-transformation method is described below:

1. Identify datapoints with overlapping year, age, sex, and location reference and non-reference population data.
2. Logit transform overlapping datapoints of alternative and reference types.
3. Convert overlapping datapoints into a difference in logit space using the following equation: $logit(alternative) - logit(reference)$.
4. Use the delta method to compute standard errors of overlapping datapoints in logit space, then calculate standard error of logit difference using the following equation: $\sqrt{(variance\ of\ alternative) + (variance\ of\ reference)}$.
5. Using MR-BRT, conduct a random effects meta-regression to obtain the pooled logit difference of alternative to reference.
6. Apply the pooled logit difference to all datapoints of alternative case definitions using the following equation: $new_{estimate} = inverse.\,logit((logit(alternative)) - (pooled\ logit\ difference))$.
7. Calculate new standard errors using the delta method, accounting for gamma (between-study heterogeneity).

Table 3: MR-BRT crosswalk factors for anti-HEV seroprevalence non-representative populations

Data input	Reference or alternative case definition	Gamma	Beta coefficient, logit (95% CI)
General population	Reference	0.88	---
Blood donors	Alternative		0.90 (–0.84 to 2.66)

We adjusted broad age-group data into five-year age bins using an estimated age pattern, a continued strategy from GBD 2019. Data in which the age range was greater than 25 years was categorised as broad age-range data. We assumed the age distribution in the study sample was the same as the estimated population in GBD 2017. We also assumed that the ratios of age-specific prevalence to full-age prevalence was the same as the seroprevalence model from GBD 2017.

Modelling strategy

DisMod model

We incorporated predictive covariates in the DisMod-MR-2.1 model to improve estimates in location-years with few or no data. The following tables provide an overview of the predictive covariates used in the anti-HEV seroprevalence model.

Table 4: Summary of covariates used in the anti-HEV seroprevalence DisMod-MR 2.1 model

Covariate	Parameter	Exponentiated beta (95% CI)
Proportion of the population living in the classic monsoon region (low-income countries)	Prevalence	1.21 (1.01–1.58)
Log-transformed SEV scalar: diarrhoea	Prevalence	1.07 (1.01–1.15)

Acute hepatitis E incidence estimation

The incidence of anti-HEV IgG seroconversion from DisMod-MR was treated as the incidence of acute hepatitis E infection.

Severity splits and disability weights

The probability of acute symptomatic infection was derived from total acute infection using the algorithm adapted from Edmonds and colleagues' 1993 publication.[3] Based on information published by Rein and colleagues,[7] we assume that the probability of symptomatic infection increases with age from ~1% in the first year of life to ~60% in adulthood.

The table below illustrates the sequelae associated with acute hepatitis E, along with their descriptions and disability weights.

Table 5: Disability weights for acute hepatitis E

Sequela	Description	Disability weight
Moderate	Has a fever and aches, and feels weak, which causes some difficulty with daily activities.	0.051 (0.032–0.074)
Severe	Has a high fever and pain, and feels very weak, which causes great difficulty with daily activities.	0.133 (0.088–0.19)
Asymptomatic	Infection with no apparent illness.	NA

References

1. Armstrong GL, Bell BP. Hepatitis A Virus Infections in the United States: Model-Based Estimates and Implications for Childhood Immunization. *Pediatrics*. 2002 May 1;109(5):839–45.
2. Schweitzer, A., Horn, J., Mikolajczyk, R. T., Krause, G., & Ott, J. J. Estimations of worldwide prevalence of chronic hepatitis B virus infection: a systematic review of data published between 1965 and 2013. *Lancet*. 2015; 386(10003), 1546–1555
3. Edmunds WJ, Medley GF, Nokes DJ, Hall AJ, Whittle HC. The influence of age on the development of the hepatitis B carrier state. *Proc Biol Sci*. 1993 Aug 23;253(1337):197–201.
4. McMahon BJ, Alward WL, Hall DB, Heyward WL, Bender TR, Francis DP, et al. Acute hepatitis B virus infection: relation of age to the clinical expression of disease and subsequent development of the carrier state. *J Infect Dis*. 1985 Apr;151(4):599–603).
5. Blach S et al. Global prevalence and genotype distribution of hepatitis C virus infection in 2015: a modelling study. *The Lancet Gastroenterology & Hepatology*. 2017; 2(3):161-176.
6. Guadagnino, Vincenzo, et al. Prevalence, risk factors, and genotype distribution of hepatitis C virus infection in the general population: a community-based survey in southern Italy. *Hepatology* 26.4 (1997): 1006-1011.

7. Rein DB, Stevens GA, Theaker J, Wittenborn JS, Wiersma ST. The global burden of hepatitis E virus genotypes 1 and 2 in 2005. *Hepatology*. 2012 Apr 1;55(4):988–97.

Human African trypanosomiasis (HAT)

Flowchart

African trypanosomiasis

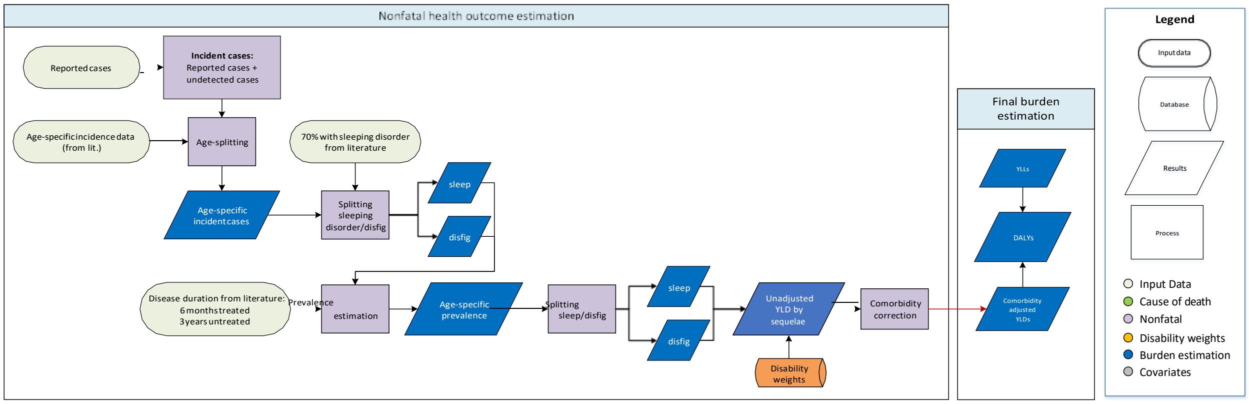

Input data and methodological summary

Case definition

Human African trypanosomiasis, also referred to as "sleeping sickness", is a protozoan infection transmitted by tsetse flies that causes headache, fever, and joint pain, then progresses to neurologic involvement including sleep, movement, speech, and psychiatric disorders; seizures; coma; and (if untreated) death. It is caused by the parasite *Trypanosoma brucei* with two subspecies, namely T.b. rhodesience (makes up less than 5% of total HAT cases) and T.b. gambiense. Cases are diagnosed through laboratory methods which rest on finding the parasite in body fluid or tissue by microscopy. In highly endemic or epidemic areas where the likelihood of false positives in serological tests is deemed lower, a seropositive individual is considered affected even in the absence of parasitological confirmation. The ICD-10 codes for HAT are B56.0, B56.1 and B56.9.

Human African trypanosomiasis

**Quantity of interest**	**Reference or alternative**	**Definition**
Human African trypanosomiasis (incidence)	Reference	Parasitological confirmation via microscopy. In highly endemic or epidemic areas, seropositive cases in absence of parasitological confirmation are included.
Human African trypanosomiasis (prevalence)	Reference	Parasitological confirmation via laboratory methods (microscopy). A seropositive individual is considered affected in absence of parasitological confirmation in highly endemic or epidemic areas.

Input data

Model inputs

Data sources for GBD 2021:

1) Annual case totals 1980–2018: National-level annual case totals from 1990–2018 were obtained from the publicly available data via WHO, available here: http://apps.who.int/gho/data/node.main.A1635?lang=en

   Subnational data:

   Kenya: Kenyan subnational estimates are attributed to Busia County. Identification of subnational locations for Kenyan case data were obtained via studies published in the peer-reviewed literature[1] and review of maps published from via the WHO HAT Atlas[2]: http://www.who.int/entity/trypanosomiasis_african/country/Kenya_whole_0014.jpg?ua=1.

2) Age/sex data: Data on the age and sex distribution of HAT cases were extracted from the peer-reviewed literature via a systematic review of sources identified in PubMed using the following search string:

   ((African trypanosomiasis[Title/Abstract] AND (incidence[Title/Abstract] OR burden[Title/Abstract] OR prevalence[Title/Abstract] OR community[Title/Abstract])) AND ("1990"[Date – Publication] : "2017"[Date – Publication]))

   This yielded 219 studies, of which only three met the inclusion criteria and were extracted[3-5]. The inclusion criteria were:

   1. Studies representative of the national population
   2. Population-based studies
   3. Studies with primary data on incidence
   4. Studies of human African trypanosomiasis (excluded studies on animal African trypanosomiasis)

3) Population at-risk estimates: 1980–2015 population at-risk estimates from GBD 2010 ArcGIS analysis using geocoded case notifications for 2000 to 2009[2] and population Count Grid estimates from Gridded Population of the World.

4) Screening coverage: Data on active versus passive screening coverage were obtained from a Weekly Epidemiological Report[6] identifying the population screened from 1997 to 2004 at the national level.

5) Geographic restrictions: Data file of all GBD locations, defining location as either endemic or non-endemic for HAT. Estimates are not produced for non-endemic countries, nor are they generated for countries with a history of HAT transmission but no data reported by WHO from 1990 to 2018.

Table 1 presents the total number of data sources used in this model.

Table 1. Total data source counts

Measure	Total sources	Countries with data
All measures	2970	35
Prevalence	1	1
Incidence	985	33
Proportion	1044	29
Population	940	29

Modelling strategy

Geographic restrictions
For countries historically considered endemic for HAT, but which have no reported case data or estimate of the population at risk, estimates are not produced. These countries include Botswana, Ethiopia, Guinea-Bissau, and Rwanda.

Among countries where population at-risk data are available, if no cases were reported to WHO, we assume the incidence of HAT is zero for those years and generate model estimates accordingly.

Modelling steps
Non-fatal estimates for HAT were generated as follows:

1. The incidence of reported HAT cases among the population at-risk was calculated as the total number of reported cases divided by the population at-risk estimates generated by the GBD working group for the period 1980–2015. Population at-risk estimates for 2016–2021 were generated by assuming an annual 2% rate of population growth.

2. To estimate the number of cases that were likely undetected by country and year, a multi-level mixed-effects linear regression of log-transformed incidence rate (ratio of reported HAT cases to population at risk) on log-transformed screening coverage (ratio of number screened for HAT to population at risk), with country random effects, was performed. Gaps were then filled using interpolation between years and extrapolation from 2019 to 2021 for reported cases. This model generates a beta-coefficient which is used to estimate the case detection rate (see step 4).

   For country-years in which no screening coverage data were reported:
   - Among countries with data reported, 1997–2004, the proportion of the at-risk population screened from 1997 was used retrospectively for the period 1980–1996 and the screening coverage from 2004 was carried forward from 2005–2021.
   - For countries with no screening data reported, the mean screening coverage for the region was used to impute a value over time.

3. Assuming the same proportion in treated (reported) and untreated (undetected) cases, the incidence estimates were then split into the two sequelae, skin disfigurement and sleeping disorder. This was done by generating 1,000 draws of the splitting proportion for the sequelae

(70%–74% with sleeping disorder) based on a study that reported presence of symptoms at admission of patients in treatment centers[7]. Draws were generated from a beta distribution with alpha parameter = 1884 and beta parameter = 649.

4. To compute prevalence of HAT, 1,000 draws of total duration of symptoms in untreated cases were generated from a normal distribution with mean = [ln(3) – 0.5 * sigma^2], and standard deviation = sigma, where sigma = [ln(4.39)-ln(1.92))/(invnormal(0.975)*2)]: these parameters were based on a study of *T.b. gambiense*[7] which estimated an average duration of three years to untreated cases. An estimated duration of six months was applied to cases that received treatment, based on findings from a paper about *T.b. rhodesiense* in Uganda[8].

5. Prevalence was then estimated from the incident cases before applying age pattern. Prevalence of treated and untreated cases were summed up, assuming that untreated cases have been prevalent up to their death for a certain duration[9]. For untreated cases, it was assumed that half the duration is spent with sleeping disorder (severe motor and cognitive impairment) and disfigurement[7]. Treated (ie, reported) cases are assumed to have been prevalent for 0.5 years, and for the fraction of treated cases that present with sleeping disorder, it was assumed that this is present for half the total duration and that the rest of the duration is spent suffering from disfiguring skin disease. Among reported cases assumed to be detected prior to stage 2 infection, we do not attribute any of the duration of morbidity to sleeping disorder.

6. Finally, an age pattern was applied to the prevalence estimates using the incidence studies from Sudan[5], DRC[3], and Uganda[4]. The age pattern in GBD 2019 employed a cubic spline to account for the higher risk of infection among working-age adults.

Severity splits/sequelae

The basis of the GBD disability weight (DW) survey assessments are lay descriptions of sequelae highlighting major functional consequences and symptoms. The lay descriptions and disability weights for HAT sequelae due to HAT are shown below in Table 2.

Table 2. Health states for human African trypanosomiasis

Sequela	Lay description	DW (95% CI)
Skin disfigurement, level 1	Has a slight, visible physical deformity that is sometimes sore or itchy. Others notice the deformity, which causes some worry and discomfort.	0.027 (0.015–0.042)
Motor plus cognitive impairments, severe	Cannot move around without help, and cannot lift or hold objects, get dressed or sit upright. The person also has very low intelligence, speaks few words, and needs constant supervision and help with all daily activities	0.542 (0.37–0.702)

Changes from GBD 2019 to GBD 2021

We have made no substantive changes in the modelling strategy from GBD 2019.

We did not apply any adjustments for the COVID-19 pandemic to Human African trypanosomiasis due to a lack of available data quantifying the impacts of the pandemic on NTD epidemiology.

References

1. Rutto JJ, Osano O, Thuranira EG, Kurgat RK, Odenyo VA. Socio-economic and cultural determinants of human african trypanosomiasis at the Kenya - Uganda transboundary. *PLoS Negl Trop Dis* 2013; 7(4): e2186.

2. Simarro PP, Cecchi G, Paone M, et al. The Atlas of human African trypanosomiasis: a contribution to global mapping of neglected tropical diseases. *Int J Health Geogr* 2010; 9: 57.

3. Lutumba P, Makieya E, Shaw A, Meheus F, Boelaert M. Human African trypanosomiasis in a rural community, Democratic Republic of Congo. *Emerg Infect Dis* 2007; 13(2): 248-54.

4. Fevre EM, Odiit M, Coleman PG, Woolhouse ME, Welburn SC. Estimating the burden of rhodesiense sleeping sickness during an outbreak in Serere, eastern Uganda. *BMC Public Health* 2008; 8: 96.

5. Moore A, Richer M, Enrile M, Losio E, Roberts J, LevyD. Resurgence of sleeping sickness in Tambura County, Sudan. *Am J Trop Med Hyg* 1999; 61(2): 315-8.

6. World Health Organization. Human African trypanosomiasis (sleeping sickness): epidemiological update. *Weekly epidemiological record* 2006; February 24(8): 69-80.

7. Blum J, Schmid C, Burri C. Clinical aspects of 2541 patients with second stage human African trypanosomiasis. *Acta Trop* 2006; 97(1): 55-64.

8. Odiit M, Kansiime F, Enyaru JC. Duration of symptoms and case fatality of sleeping sickness caused by Trypanosoma brucei rhodesiense in Tororo, Uganda. *East Afr Med J* 1997; 74(12): 792-5.

9. Checchi F, Filipe JA, Haydon DT, Chandramohan D, Chappuis F. Estimates of the duration of the early and late stage of gambiense sleeping sickness. *BMC Infect Dis* 2008; 8: 16.

Fetal alcohol syndrome

Flowchart

Fetal alcohol syndrome (FAS)

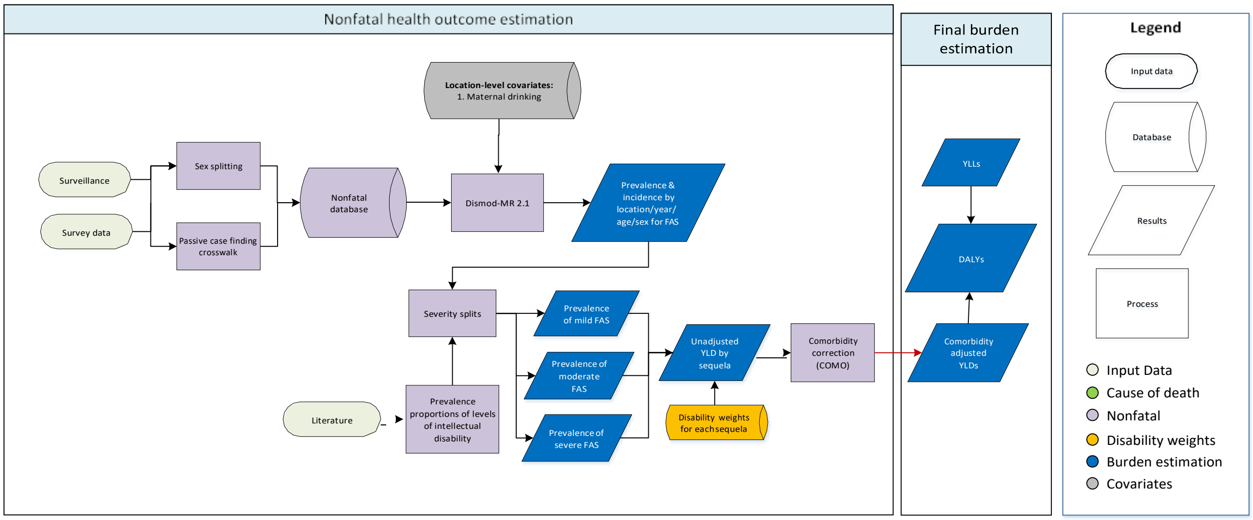

Input data and methodological summary

Case definition

Fetal alcohol syndrome (FAS; ICD-10: Q86.0) is a disorder caused by maternal drinking during pregnancy and is the most severe form of fetal alcohol spectrum disorder (FASD). In GBD, only FAS cases were included in the model. Other manifestations of FASD including partial fetal alcohol syndrome, alcohol-related neurodevelopmental disorder, and alcohol-related birth defects were not included. FAS is characterised by maternal alcohol exposure which results in certain patterns of facial anomalies such as short palpebral fissures and abnormalities in the premaxillary zone (eg, flat upper lip, flattened philtrum, and flat midface), growth retardation (eg, decelerating weight over time not due to nutrition), and central nervous system neurodevelopmental abnormalities (eg, decreased cranial size at birth) in the offspring.[1] Cases were defined according to diagnostic guidelines set by the USA Institute of Medicine, the British Paediatric Association, and other recognised bodies in the area.

Input data

Model inputs

A series of systematic literature reviews were conducted to capture studies reporting the prevalence, incidence, remission, and excess mortality of FAS. The reviews incorporated searches of peer-reviewed literature via electronic databases and consultation with experts. In order for a study to be included, it must use recognised classifications of FAS (eg, the USA Institute of Medicine) and provide sufficient details on study methodology and sample characteristics to determine study quality. No limitation was set on the language of publication. Data from the European Surveillance of Congenital Anomalies (EUROCAT) were also included and updated where relevant. This methodology was utilised in GBD 2015. Updates to systematic reviews are performed on an ongoing schedule across all GBD causes.

Table 1: Data Inputs fetal alcohol syndrome morbidity modelling by parameter.

Parameter	Countries with data	New sources	Total sources

Prevalence	32	0	169
Standardised mortality ratio	4	0	5
Other	4	0	7

Data reported for both sexes were split using a global sex ratio estimated using MR- BRT[2] (Meta-regression = Bayesian, Regularised, Trimmed, described in appendix 1, section 4.4.1 of the reference).

Table 2: MR-BRT Sex Splitting Adjustment Factors for fetal alcohol syndrome

Data input	Gamma	Beta Coefficient, Log (95% UI)	Adjustment factor*
Female: Male	0	-0.28 (-0.67, 0.11)	0.76

Adjustment factor is the transformed beta coefficient in normal space, and can be interpreted as the factor by which the alternative case definition is adjusted to reflect the ratio by which both-sex datapoints were split.

Bias correction

Prevalence data collected using both passive and active case finding methodologies was included in this model. As passive case finding methods are likely to underestimate the true prevalence of fetal alcohol syndrome, a crosswalk was applied to increase the uncertainty around those datapoints. The expected difference in reported prevalence was modelled using MR-BRT. To adjust the passive case data, a logit difference model was used in which the beta coefficient was subtracted from the logit transformed prevalence data, the inverse logit of which was used in the model. Table 2 summarises the MR-BRT crosswalk coefficients.

Table 3: MR-BRT Crosswalk Adjustment Factors for fetal alcohol syndrome

Data input	Reference or alternative case definition	Gamma	Beta Coefficient, Logit (95% UI)*
Active case finding	Ref	1.87	---
Passive case finding	Alt		-0.03 (-3.60, 3.51)

MR-BRT crosswalk adjustments can be interpreted as the factor the alternative case definition is adjusted by to reflect what it would have been had it been measured using the reference case definition. If the log/logit beta coefficient is negative, then the alternative is adjusted up to the reference. If the log/logit beta coefficient is positive, then the alternative is adjusted down to the reference.

Severity split inputs and disability weights

There were no data available which gave prevalence of FAS by severity. As such, severity splits for FAS were calculated by matching FAS severity to categories of IQ in children for which prevalence data are available. Severe FAS was matched to an IQ of less than 50, moderate FAS to an IQ of 50 to 69, mild FAS to an IQ of 74 to 84, and asymptomatic FAS to an IQ of 85 or higher. Prevalence data for these IQ levels were then used to calculate severity splits for FAS.

Table 4. Severity distribution, details on the severity levels for fetal alcohol syndrome in GBD 2019 and the

associated disability weight (DW) with that severity.

Severity level	Lay description	DW (95% CI)
Mild	Is a little slow in developing physically and mentally, which causes some difficulty in learning but no other difficulties in daily activities.	0.016 (0.008–0.03)
Moderate	Is slow in developing physically and mentally, which causes some difficulty in daily activities.	0.056 (0.035–0.083)
Severe	Is very slow in developing physically and mentally, which causes great difficulty in daily activities.	0.179 (0.119–0.257)

Modelling strategy

We have made no substantive changes in the modelling strategy from GBD 2019. The GBD 2021 modelling strategy utilised DisMod-MR 2. 1[3] (Disease Model – Bayesian Meta-regression, described in appendix 1, section 4.5) to estimate prevalence by age, sex, year, and location. Prevalence was set to begin from birth. Incidence was set to zero given cases cannot manifest after birth (despite the fact they may not be diagnosed immediately at birth). Remission was also set to zero. Estimates from known high-drinking populations (eg, indigenous populations) were not considered representative of the general population and were excluded. A country-level covariate was included representing the log proportion of pregnant women who drink during their pregnancy, estimated from a meta-analysis.[4] The table below illustrates the covariate, parameter, beta and exponentiated beta values for the model.

Table 5. Covariates. Summary of covariates used in the fetal alcohol syndrome DisMod-MR meta-regression model

Covariate	Type	Parameter	Exponentiated beta (95% Uncertainty Interval)
Maternal drinking	Country	Prevalence	1.07 (1.00 — 1.22)

References

1. Stratton K, Howe C, Battaglia F, editors. Fetal alcohol syndrome. Diagnosis, epidemiology, prevention, and treatment. Washington DC: National Academy Press; 1996.
2. Murray CJL, Aravkin AY, Zheng P, et al. Global burden of 87 risk factors in 204 countries and territories, 1990–2019: a systematic analysis for the Global Burden of Disease Study 2019. The Lancet 2020; 396: 1223–49. doi: https://doi.org/10.1016/S0140-6736(20)30752-2
3. Vos T, Lim SS, Abbafati C, et al. Global burden of 369 diseases and injuries in 204 countries and territories, 1990–2019: a systematic analysis for the Global Burden of Disease Study 2019. The Lancet 2020; 396: 1204–22. doi: https://doi.org/10.1016/S0140-6736(20)30925-9
4. Popova S, Lange S, Probst C, Gmel G, Rehm J. Estimation of national, regional, and global prevalence of alcohol use during pregnancy and fetal alcohol syndrome: a systematic review and meta-analysis. The Lancet Global Health 2017.

Alcohol use disorders

Flowchart

Alcohol use disorders

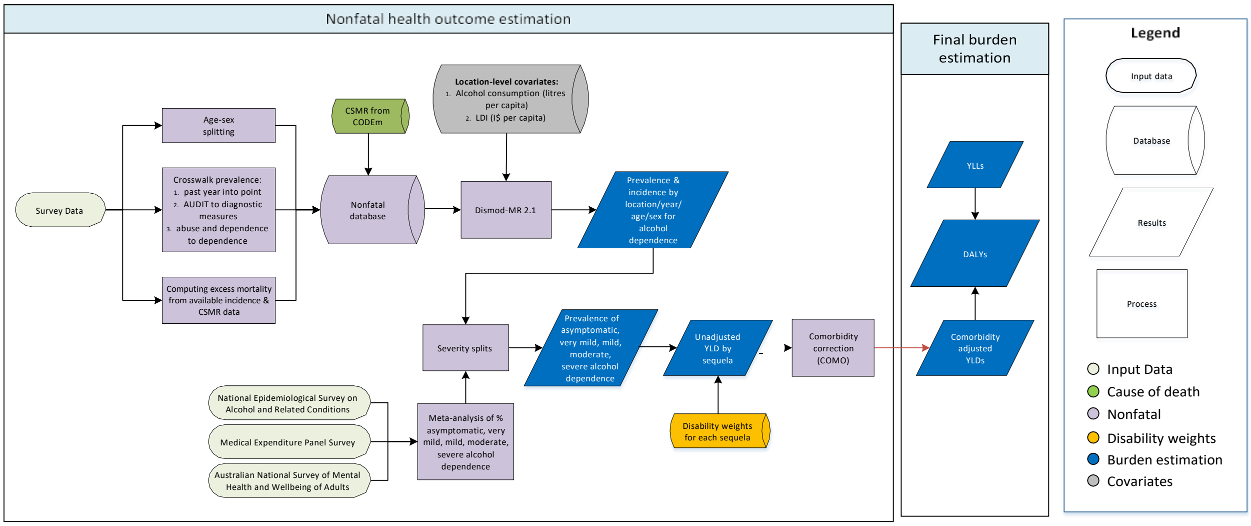

Case definition

Alcohol dependence is a substance-related disorder involving a dysfunctional pattern of alcohol use. According to the Diagnostic and Statistical Manual of Mental Disorders (DSM-IV) criteria for alcohol dependence, at least three out of seven of the following criteria must be manifested during a 12-month period:

- Tolerance
- Withdrawal symptoms or clinically defined alcohol withdrawal syndrome
- Use in larger amounts or for longer periods than intended
- Persistent desire or unsuccessful efforts to cut down on alcohol use
- Time is spent obtaining alcohol or recovering from effects
- Social, occupational, and recreational pursuits are given up or reduced because of alcohol use
- Use is continued despite knowledge of alcohol-related harm (physical or psychological)

The DSM-IV codes for alcohol dependence is 303.90, and the corresponding International Classification of Diseases (ICD-10) codes are F10.1 and F10.2.[1,2]

Input data

Model inputs

In GBD 2013 and GBD 2016, systematic reviews of literature were conducted to capture studies of

prevalence, incidence, remission, duration, and excess mortality associated with alcohol dependence. In

summary, the search was conducted in three stages involving searches of the peer-reviewed literature (via Medline, Embase, and PubMed), the grey literature, and expert consultation. Updates to systematic reviews are performed on an ongoing schedule across all GBD causes.

The last systematic review was conducted for GBD 2016 for papers published through [end date of review if you have it]. The inclusion criteria stipulated that (1) “caseness” must be based on clinical threshold as established by the DSM and ICD; (2) sufficient information must be provided on study method and sample characteristics to assess the quality of the study; and (3) study samples must be representative of the general population (ie, inpatient or pharmacological treatment samples [accepted for estimates of mortality], case studies, and veterans or refugee samples were excluded).

Table 1: Data Inputs for alcohol dependence morbidity modelling by parameter.

Parameter	Countries with data	New sources	Total sources
Prevalence	58	0	378
Incidence	3	0	3
Remission	3	0	3
Other	15	0	63

Prevalence estimates were split by age and sex where necessary. First, studies that reported prevalence for both sexes were split using a global sex ratio estimated using MR-BRT[3] (Meta-regression = Bayesian, Regularised, Trimmed, described in appendix 1, section 4.4.1 of the reference). Second, where studies reported estimates across age groups spanning 20 years or more, these were split into five-year age groups using the global age pattern estimated by DisMod-MR 2.1[4] (Disease Model – Bayesian Meta-regression, described in appendix 1, section 4.5).

Table 2: MR-BRT Sex Splitting Adjustment Factors for alcohol dependence

Data input	Gamma	Beta Coefficient, Log (95% UI)	Adjustment factor*
Female: Male	0.33	-0.69 (-1.35, -0.04)	0.50
Age < 20		0.12 (0.07, 0.18)	1.13

Adjustment factor is the transformed beta coefficient in normal space, and can be interpreted as the factor by which the alternative case definition is adjusted to reflect the ratio by which both-sex datapoints were split.

Bias correction

Due to insufficient data on alcohol dependence in some regions, three crosswalks were performed using MR-BRT to allow for the inclusion of data that did not meet our reference definitions in the epidemiological modelling of alcohol dependence. The first crosswalk converted estimates of alcohol use disorders (alcohol abuse + alcohol dependence) to reflect what they would be if the data represented estimates of alcohol dependence. Similarly, the second crosswalk was performed using MR-BRT to adjust past-year prevalence estimates of alcohol dependence toward the level they would have been had the

study measured point prevalence, as the latter is less susceptible to recall bias. The third crosswalk adjusted estimates of prevalence according to the Alcohol Use Disorder Identification Test (AUDIT) to what they would be had prevalence been determined based on diagnostic measures. For this final crosswalk, a systemic review was performed in GBD 2019 to identify AUDIT validation studies using the following search string:

```
(("audit"[tiab] AND "alcohol"[tiab]) OR "alcohol use disorders identification test"[tiab]) AND
("validation"[tiab] or "validity"[tiab]) NOT (animals[MeSH] NOT humans[MeSH])
```

Out of 303 total studies screened, 38 studies were found to report prevalence of alcohol dependence according to the AUDIT as well as according to physician diagnosis, or reported specificity and sensitivity to allow for the calculation of prevalence. These studies were used to generate crosswalk parameters using MR-BRT. All three crosswalks utilised a logit difference model, which has been described elsewhere. Briefly, alternative definition datapoints were logit transformed, and the MR-BRT beta was subtracted from them, after which they were transformed back into normal space.

Table 3: MR-BRT Crosswalk Adjustment Factors for alcohol dependence

Data input	Reference or alternative case definition	Gamma	Beta Coefficient, Logit (95% UI)
Point prevalence	Ref	0.68	---
Past- year prevalence	Alt		0.81 (-0.58 – 2.14)
Prevalence according to diagnostic measures	Ref	0.76	---
Prevalence according to AUDIT	Alt		1.09 (-0.40 – 2.63)
Alcohol dependence prevalence	Ref	0.57	---
Alcohol dependence and abuse prevalence	Alt		1.04 (-0.03 – 2.19)

MR-BRT crosswalk adjustments can be interpreted as the factor the alternative case definition is adjusted by to reflect what it would have been had it been measured using the reference case definition. If the log/logit beta coefficient is negative, then the alternative is adjusted up to the reference. If the log/logit beta coefficient is positive, then the alternative is adjusted down to the reference.

Severity split inputs and disability weights

The basis of the GBD disability weight survey assessments are lay descriptions of sequelae highlighting major functional consequences and symptoms. The lay descriptions and disability weights for alcohol dependence severity levels are shown below.

Table 4. Severity distribution, details on the severity levels for alcohol dependence in GBD 2019 and the associated disability weight (DW) with that severity.

Severity level	Lay description	DW (95% CI)
Very mild	Drinks alcohol daily and has difficulty controlling the urge to drink. When sober, the person functions normally.	0.123 (0.082–0.177)
Mild	Drinks a lot of alcohol and sometimes has difficulty controlling the urge to drink. While intoxicated, the person has difficulty performing daily activities.	0.235 (0.16–0.327)

Moderate	Drinks a lot, gets drunk almost every week and has great difficulty controlling the urge to drink. Drinking and recovering cause great difficulty in daily activities, sleep loss, and fatigue.	0.373 (0.248–0.508)
Severe	Gets drunk almost every day and is unable to control the urge to drink. Drinking and recovering replace most daily activities. The person has difficulty thinking, remembering and communicating, and feels constant pain and fatigue.	0.57 (0.396–0.732)

asymptomatic cases carried no disability weight

Severity splits used in GBD 2021 were consistent with those used in GBD 2017. The United States' Medical Expenditure Panel Survey (MEPS, conducted in annual waves since 1996)[5], the US National Epidemiological Survey on Alcohol and Related Conditions (NESARC, conducted in two waves from 2001–2002 and 2004–2005)[6], and the Australian National Survey of Mental Health and Wellbeing of Adults (NSMHWB, conducted in 1997)[7] were used to estimate the proportion of alcohol dependence cases in the asymptomatic 40.9% (38.4%–43.3%); very mild 46.9% (43.7%–50.0%); mild 4.0% (1.8%–5.8%); moderate 3.4% (2.3%–4.5%); and severe 4.8% (3.0%–7.0%) disease categories.

Modelling strategy

We have made no substantive changes in the modelling strategy from GBD 2019. The GBD 2021 epidemiological modelling strategy for alcohol dependence made use of DisMod-MR 2.1 to estimate prevalence by age, sex, year, and location. Standardised mortality ratio and relative risk data were excluded in the modelling process. Instead we pulled in cause-specific mortality rate (CSMR) data from our CODEm and CODcorrect analyses and matched it with prevalence datapoints for the same geography and study year to estimate priors on excess mortality rates (by dividing CSMR by prevalence). We assumed no incidence and mortality before age 10. An upper limit of 0.6 was placed on remission (in line with data from the US National Epidemiological Survey on Alcohol and Related Conditions (NESARC) as well as a declining trend with age to restrict DisMod-MR 2.1 from straying too far from the data inputs.

Three country-level covariates were included in the DisMod-MR 2.1 model. The LDI covariate represents a moving average of gross domestic product (GDP) over time. LDI was also applied to excess mortality data with a negative relationship assumed. Alcohol consumption was also represented by a covariate representing this in terms of litres of alcohol per capita. We also have an age-standardised, sex-specific summary exposure value for alcohol use.

Table 4. Covariates. Summary of covariates used in the alcohol dependence DisMod-MR meta-regression model

Covariate	Type	Parameter	Exponentiated beta (95% Uncertainty Interval)
Litres of alcohol consumed per capita	Country	Prevalence	1.01 (1.00 – 1.04)
LDI (I$ per capita)	Country	Excess mortality rate	0.90 (0.90 – 0.90)
Age-standardised, sex-specific SEV: Alcohol Use	Country	Prevalence	3.26 (2.91 – 3.48)

References

1. American Psychiatric Association. Diagnostic and Statistical Manual of Mental Disorders (DSM-IV-TR). 4th, Text Revision ed. Washington DC: American Psychiatric Association; 2000.

2. World Health Organization. The ICD-10 Classification of Mental and Behavioural Disorders. Clinical descriptions and diagnostic guidelines. Geneva: World Health Organization; 1992.

3. Murray CJL, Aravkin AY, Zheng P, et al. Global burden of 87 risk factors in 204 countries and territories, 1990–2019: a systematic analysis for the Global Burden of Disease Study 2019. The Lancet 2020; 396: 1223–49. doi: https://doi.org/10.1016/S0140-6736(20)30752-2

4. Vos T, Lim SS, Abbafati C, et al. Global burden of 369 diseases and injuries in 204 countries and territories, 1990–2019: a systematic analysis for the Global Burden of Disease Study 2019. The Lancet 2020; 396: 1204–22. doi: https://doi.org/10.1016/S0140-6736(20)30925-9

5. Agency for Healthcare Research and Quality. United States Medical Expenditure Panel Survey. Rockville, United States: Agency for Healthcare Research and Quality.

6. Introduction to the National Epidemiologic Survey on Alcohol and Related Conditions [http://pubs.niaaa.nih.gov/publications/arh29-2/74-78.htm]. Access date 1 December 2014.

7. Australian Bureau of Statistics. National Survey of Mental Health and Wellbeing of Adults 1997. Canberra: Australian Bureau of Statistics.

Anorexia nervosa

Flowchart

Anorexia nervosa

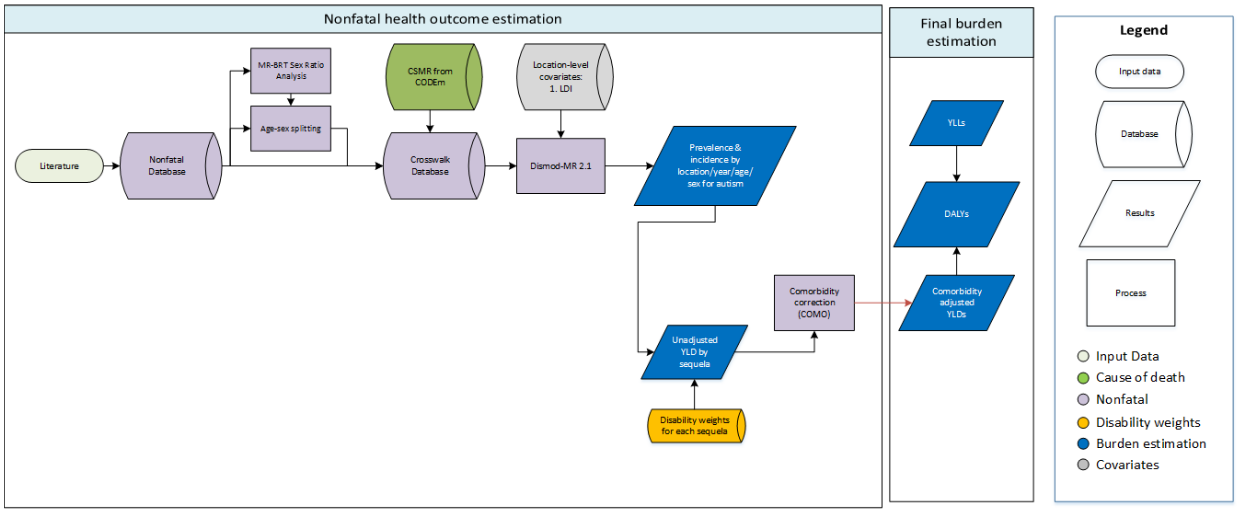

Input data and methodological summary for anorexia nervosa

Case definition

According to the Diagnostic and Statistical Manual of Mental Disorders fourth edition, text revision (DSM-

IV-TR),[1] anorexia nervosa (AN) is an eating disorder characterised by:

a) Refusal to maintain body weight at or above a minimally normal weight for age and height (eg, weight loss leading to maintenance of body weight less than 85% of that expected; or failure to make expected weight gain during period of growth, leading to body weight less than 85% of that expected).
b) Intense fear of gaining weight or becoming fat, even though underweight (expanded to include any behaviour that interferes with weight gain in DSM-5).[2]
c) Disturbance in the way in which one's body weight or shape is experienced, undue influence of body weight or shape on self-evaluation, or denial of the seriousness of the current low body weight.
d) In postmenarcheal females, amenorrhoea, ie, the absence of at least three consecutive menstrual cycles (this criterion was removed in DSM-5).[2]

Included in the GBD study were cases meeting diagnostic criteria according to DSM[1] or the International Classification of Diseases (ICD).[3] These were identified by the following codes: 307.1 (DSM-IV-TR) and F50.0-50.1 (ICD-10). Different versions of DSM (DSM-III, DSM-III-R, DSM-IV, DSM-IV-TR, DSM-5, and DSM-5-TR) and ICD (ICD-9, ICD-10 and ICD-11) were accepted.

Input data

Systematic literature reviews were conducted to capture studies reporting the prevalence, incidence, remission, and excess mortality of AN. These were conducted in three stages involving electronic searches of the peer-reviewed literature (ie, via PsycInfo, Embase, and PubMed), the grey literature, and expert consultation. For mental disorders, we update our GBD electronic database searches on a rolling basis. . An electronic search was not required for GBD 2021. The next update will be conducted in the next round of GBD. The grey literature searches and expert consultation were conducted for GBD 2021.

The GBD inclusion criteria stipulated that: 1) the publication year must be from 1980 onward; 2) "caseness" must be based on clinical threshold as established by the DSM or ICD; 3) sufficient information must be provided on study method and sample characteristics to assess the quality of the study; and 4) study samples must be representative of the general population (ie, inpatient or pharmacological treatment samples, case studies, veterans, or refugee samples were excluded). No limitation was set on the language of publication. Methods used for this systematic review have been reported in greater detail elsewhere.[4] Table 1 summarises data inputs by parameter for AN.

Table 1: Data Inputs for AN morbidity modelling by parameter.

Parameter	Countries with data	New sources	Total sources
Incidence	6	0	6
Prevalence	27	0	65
Remission	11	0	21
Other	9	4	23

Age-sex splitting

The extracted data underwent three types of age-sex splitting processes:

1. Where possible, estimates were further split by sex and age based on the available data. For instance, if studies reported prevalence for broad age groups by sex (eg, prevalence in 15–65 year-old-males and females separately), and also by specific age groups but for both sexes combined (eg, prevalence in 15–30-year-olds, then in 31–65-year-olds, for males and females combined);

age-specific estimates were split by sex using the reported sex-ratio and bounds of uncertainty.

1. A meta-regression—Bayesian, regularised, trimmed (MR-BRT) analysis was used to split the remaining both-sex estimates in the dataset. For each parameter, sex-specific estimates were matched by location, age, and year. A MR-BRT network meta-analysis was then used to estimate pooled sex ratios and bounds of uncertainty. These were then used to split the both-sex estimates in the dataset. The male-to-female prevalence ratio estimated was 0.32 (95% uncertainty interval [UI]: 0.12–0.52).
2. Studies reporting prevalence estimates across age groups spanning 25 years or more, were split into five-year age groups using the prevalence age pattern estimated by DisMod-MR 2.1. The DisMod-MR model used to estimate the age pattern did not contain any previously age split data.

Bias corrections/crosswalks

We tested for a number of potential sources for bias in prevalence between studies (eg, use of ICD vs. DSM criteria, past-year vs. point recall). However, none of the crosswalks had a statistically significant impact on prevalence and so no bias corrections were applied to these estimates.

Modelling strategy

We have made no substantive changes in the modelling strategy from GBD 2019.

After the above data processes were applied, DisMod MR 2.1 was used to model the epidemiological data for AN. Adjustments to model priors or the dataset were made where appropriate. Where outliers were identified in the data, we reassessed the study's methodology and quality before a decision was made to exclude or include the data.

We assumed no incidence prior to age 5 or from 50 years onward. These settings are in line with those placed on the corresponding cause of death model for AN. A cap of 0.6 was placed on remission in order to obtain a more plausible fit of the model. We used the function in DisMod-MR to pull in cause-specific mortality rate (CSMR) data from our CODEm and CoDCorrect analyses. As such, other mortality data (standardised mortality ratios and relative risks) were excluded. We also used these CSMR data to estimate priors on excess mortality rates (EMR) by matching them with prevalence datapoints for the same geography and study year and dividing CSMR by prevalence. A country-level covariate, lagged distributed income (LDI), was included. This covariate represents a moving average of gross domestic product (GDP) over time. The limits placed on this covariate meant that prevalence was assumed to increase with rising GDP. LDI was also applied to excess mortality data in order to better inform regional distribution. A summary of location-level covariates and exponentiated values for AN are shown in Table 2.

Table 2: Summary of covariates used in the AN DisMod-MR meta-regression model

Covariate	Type	Parameter	Exponentiated beta (95% UI)
LDI ($ per capita)	Location-level	Prevalence	1.48 (1.26—1.64)
LDI ($ per capita)	Location-level	Excess mortality	0.78 (0.66—0.90)

Disability weight

The GBD disability weight survey assessments include lay descriptions of sequelae highlighting major functional consequences and symptoms. No severity splits were applied to AN. The lay description and disability weight for AN are shown in Table 3.

Table 3: Lay description for AN in GBD 2021 and the associated disability weight

Lay description	Disability weight (95% UI)

Feels an overwhelming need to starve and exercises excessively to lose weight. The person is very thin, weak, and anxious.	0.224 (0.150–0.312)

There were no significant changes in GBD 2021 results for AN compared to GBD 2019. While we continue to improve on the data and methods used to estimate the burden of mental disorders, some challenges need to be acknowledged. Firstly, we still have a large number of locations with no high quality raw data available. Secondly, it is difficult to quantify and remove all variation due to measurement error in our epidemiological estimates. While we have improved the methodology used to account for known sources of bias, in some cases, we still have very few datapoints to inform these adjustments. Thirdly, there is a paucity of research on the risk factors of mental disorders which can be used as predictive covariates in our epidemiological models.

References

1. American Psychiatric Association. Diagnostic and Statistical Manual of Mental Disorders (DSM-IV-TR). 4th, Text Revision ed. Washington DC: American Psychiatric Association; 2000.
2. American Psychiatric Association. Diagnostic and Statistical Manual of Mental Disorders, Fifth Edition. Arlington, VA: American Psychiatric Association; 2013.
3. World Health Organization. ICD-10 Classification of Mental and Behavioural Disorders: Clinical descriptions and diagnostic guidelines. Geneva: World Health Organization; 1992.
4. Ferrari AJ, Charlson FJ, Norman RE, et al. Burden of depressive disorders by country, sex, age, and year: findings from the global burden of disease study 2010. *PLoS Med* 2013; **10**(11): e1001547.

Anxiety disorders

Flowchart

Anxiety disorders

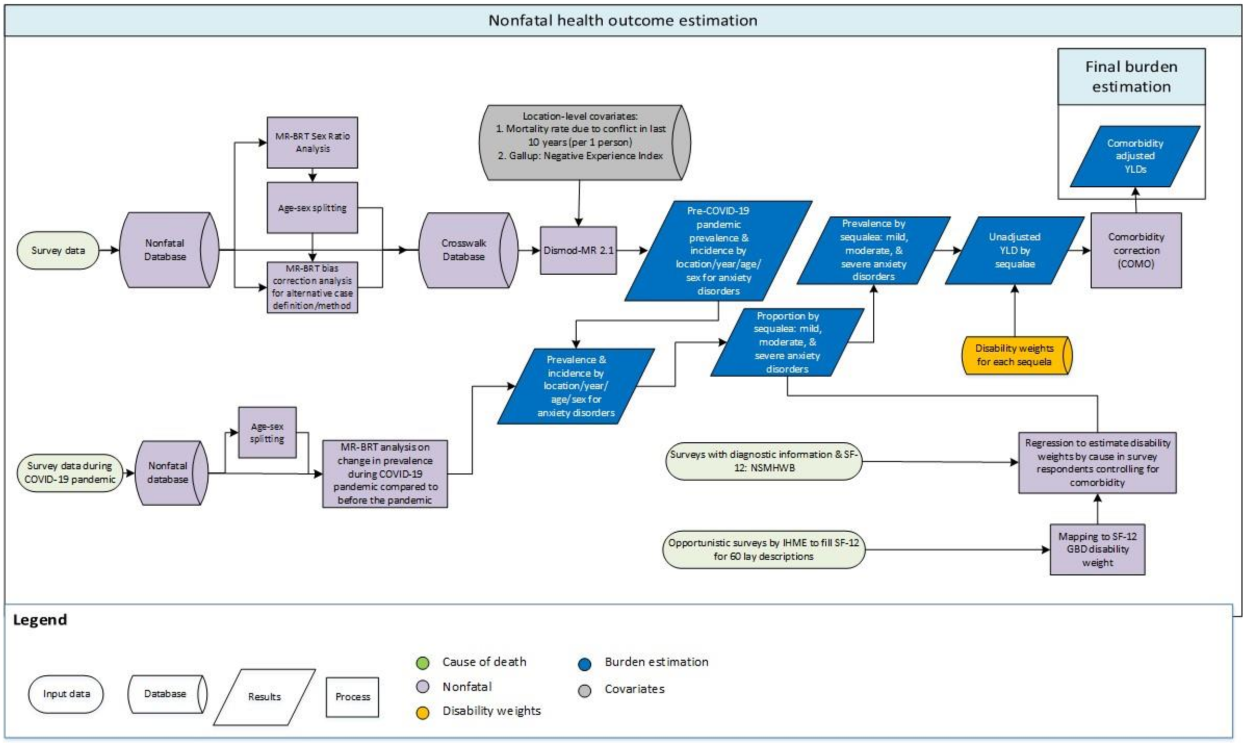

Input data and methodological summary for anxiety disorders

Case definition

Anxiety disorders are characterised by experiences of intense fear and distress, typically in combination with other physiological symptoms. We aimed to capture all cases of anxiety disorders reaching diagnostic threshold defined by the Diagnostic and Statistical Manual of Mental Disorders (DSM) or the World Health Organization (WHO) International Classification of Diseases (ICD).[1,2] The specific anxiety disorders included were panic disorder, agoraphobia, specific phobia, social phobia, obsessive-compulsive disorder (OCD), post-traumatic stress disorder (PTSD), generalised anxiety disorder (GAD) including overanxious disorder in childhood, separation anxiety disorder (SAD), and anxiety disorder "not otherwise specified" (NOS). These were identified by the following codes: DSM-IV-TR: 300.0-300.3, 208.3, 309.21, 309.81; ICD-10: F40-42, F43.0, F43.1, F93.0-93.2, F93.8. Excluded were anxiety disorders due to a general medical condition and substance-induced anxiety disorder. Different versions of DSM (DSM-III, DSM-III-R, DSM-IV, DSM-IV-TR, DSM-5, and DSM-5-TR) and ICD (ICD-9, ICD-10, and ICD-11) were accepted.

Anxiety disorders were modelled as a single cause for "any" anxiety disorder to avoid the double-counting of individuals meeting criteria for more than one anxiety disorder. Epidemiological estimates reporting an outcome for "any" or "total" anxiety disorders were included in analyses, if they reported on at least three anxiety disorders. This has been further explained in previous publications.[3,4]

Input data

The epidemiological systematic literature review for anxiety disorders was conducted in three stages involving electronic searches of the peer-reviewed literature (ie, via PsycInfo, Embase, and PubMed), the grey literature, and expert consultation. For mental disorders, we update our GBD electronic database

searches on a rolling basis. An electronic search was not required for GBD 2021. The next update will be

conducted in the next round of GBD. The grey literature searches and expert consultation were conducted for GBD 2021.

The GBD inclusion criteria stipulated that: 1) the publication year must be from 1980 onward; 2) "caseness" must be based on clinical threshold as established by the DSM or ICD; 3) sufficient information must be provided on study method and sample characteristics to assess the quality of the study; 4) a minimum of three (or two if occurring during childhood) anxiety disorder subtypes must be included within the overall estimate; and 5) study sample must be representative of the general population (ie, inpatient or pharmacological treatment samples, case studies, veterans, or refugee samples were excluded). No limitation was set on the language of publication. Methods used in this systematic review have been reported in greater detail elsewhere.[3,4] Table 1 summarises data inputs by parameter for anxiety disorders.

Table 1: Data inputs for anxiety disorders morbidity modelling by parameter

Parameter	Countries with data	New sources	Total sources
**Incidence**	2	2	3
**Prevalence**	60	51	227
**Remission**	3	0	3
**Other**	0	0	0

Age-sex splitting

The extracted data underwent three types of age-sex splitting processes:

3. Where possible, estimates were further split by sex and age based on the available data. For instance, if studies reported prevalence for broad age groups by sex (eg, prevalence in 15–65-year-old males and females separately), and also by specific age groups but for both sexes combined (eg, prevalence in 15–30-year-olds, then in 31–65-year-olds, for males and females combined); age-specific estimates were split by sex using the reported sex-ratio and bounds of uncertainty.
4. A meta-regression–Bayesian, regularised, trimmed (MR-BRT) analysis was used to split the remaining both-sex estimates in the dataset. For each parameter, sex-specific estimates were matched by location, age, and year. A MR-BRT network meta-analysis was then used to estimate pooled sex ratios and bounds of uncertainty. These were then used to split the both-sex estimates in the dataset. The male-to-female prevalence ratio estimated was 0.54 (95% uncertainty interval [UI]: 0.4–0.66).
5. Studies reporting prevalence estimates across age groups spanning 25 years or more were split into five-year age groups using the prevalence age pattern estimated by DisMod-MR 2.1. The DisMod-MR model used to estimate the age pattern did not contain any previously age-split data.

Bias corrections/crosswalks

Estimates with known biases were adjusted/crosswalked accordingly prior to DisMod-MR 2.1. For each crosswalk of interest, pairs of the reference and the alternative estimates were matched by age, sex, location, and year. This was done for both within-study (where possible) and between-study pairs. These pairs were then used as inputs in a MR-BRT network meta-analysis. The MR-BRT analysis produced a

pooled ratio between the reference estimates and alternative estimates, which was used to adjust all

alternative estimates in the dataset. For anxiety disorders, a past-year recall ratio was used to adjust all past-year recall estimates towards the level they would have been if the estimate had capture point/past-month prevalence. The latter prevalence period is less affected by recall bias. See Table 2 for adjustment factors used for anxiety disorders. The estimated UIs around the adjustment ratio incorporate Gamma which represents the between-study variance across all input data in the model. This added uncertainty widens the UIs for crosswalks with significant fixed effects.

Table 2: MR-BRT crosswalk adjustment factors for anxiety disorders

**Data input**	**Reference or alternative case definition**	**Gamma**	**Beta Coefficient, Log (95% UI)***	**Adjustment factor****
Population survey	Reference: past-month or point prevalence	0.23		
Population survey	Alternative: past-year prevalence		0.45 (–0.02 to 0.90)	1.57 (0.98–2.46)

**MR-BRT crosswalk adjustments can be interpreted as the factor the alternative case definition is adjusted by to reflect what it would have been had it been measured using the reference case definition. If the log/logit beta coefficient is negative, then the alternative is adjusted up to the reference. If the log/logit beta coefficient is positive, then the alternative is adjusted down to the reference.*
***The adjustment factor column is the exponentiated beta coefficient. For log beta coefficients, this is the relative rate between the two case definitions. For logit beta coefficients, this is the relative odds between the two case definitions.*

Impact of the COVID-19 pandemic

The emergence of the COVID-19 pandemic in 2020 has raised many questions around the resulting impacts on mental health. In GBD 2021, we sought to quantify the impact of COVID-19 on the prevalence and burden of major depressive disorder and anxiety disorders for the years 2020 and 2021.

We first conducted a systematic literature review to identify studies reporting on major depressive disorder or anxiety disorder prevalence during the COVID-19 pandemic published between 1 January 2020 and 29 January 2021. The search was conducted in three stages involving electronic searches of the peer-reviewed literature (ie, via PubMed), the grey literature (ie, via COVID-19: living map of the evidence by Eppi-centre, The DEPRESSD Project, WHO-COVID-19, COVID-minds, MedRxiv, and PsyArXiv), and expert consultation. The following search terms were used to develop search strings across all databases searched: 'mental health', 'mental disorders', 'anxiety disorder', 'depressive disorder', 'anxiety', 'depress*', 'psycholog' and 'novel coronavirus', 'covid', 'covid-19', 'nCoV', '2019nCoV', 'coronavirus', 'coronavi*', 'SARS-COV-2' 'SARSCoV2', 'outbreak', 'epidemic', 'pandemic', and 'prevalence', 'impact', 'outcome', 'effect', 'percentage'.[5]

We conducted an update to the systematic literature review in two stages. First, in July 2022, we conducted a review of reviews by searching for systematic reviews in PubMed published since 1 January 2021. Next, in August 2022, we conducted electronic searches of the peer-reviewed literature (ie, via PubMed), the grey literature (ie, via COVID-19: living map of the evidence by Eppi-centre, WHO-COVID-19, and COVID-minds), and expert consultation. Studies reporting data during 2021 and 2022 were prioritised in this update.

The inclusion criteria used closely mirrored the criteria used more broadly within the GBD to ensure

consistency in measurement. Studies had to report the prevalence of anxiety disorders during the

COVID-19 pandemic and have a pre-pandemic baseline. Longitudinal studies using samples that were representative of the general population were preferred, but cross-sectional studies conducted during the COVID-19 pandemic were also accepted if comparable pre-COVID-19 prevalence data could be identified. Studies reporting probable anxiety disorders using established screening measures (eg, the General Anxiety Disorder-7) were included due to lack of available data using diagnostic thresholds for anxiety disorders. Additionally, studies using screening measures of psychological distress or both symptoms of depression and anxiety combined (eg, the Kessler-6) were included and were controlled for in analyses.

The first search generated 5683 records, and the update generated 5569 (after duplicates were removed). The title/abstract screening across both searches reduced the number of relevant records to 2544 studies, of which 38 met criteria for inclusion for anxiety disorders.

Modelling strategy

The modelling strategy used in GBD 2021 was the same as GBD 2019, with the addition of COVID-19 adjustment. The COVID-19 adjustment was applied to the modelled prevalence estimates for 2020 and 2021, after the standard epidemiological modelling analysis to estimate prevalence by age, sex, location, and year had been undertaken.

DisMod-MR 2.1 was used to model the (pre-COVID-19) epidemiological data for anxiety disorders. Adjustments to model priors or the dataset were made where appropriate. Where outliers were identified in the data, we reassessed the study's methodology and quality before a decision was made to exclude or include the data.

Data across all epidemiological parameters were initially included in the modelling process. The incidence studies reported estimates which were very low relative to the prevalence data. As prevalence studies contributed much greater world coverage than incidence studies, we excluded the incidence data, relying instead on data from the other parameters. We assumed no incidence and prevalence before age 2 and after age 95. This minimum age of onset was corroborated with expert feedback and existing literature on anxiety disorders. Remission was set to a maximum of 0.2, consistent with the datapoints available.

The following location-level covariates were used to inform the estimation of prevalence:

1. The mean war mortality rate in the previous ten years: This covariate identified, for each GBD location, the mean mortality rate in the previous ten years due to war and terrorism. It was used given existing evidence that shows a positive association between conflict status and the prevalence of anxiety disorders.[6,7]
2. The Gallup negative experience index: The Gallup initiative conducts comprehensive and comparable national surveys across a wide range of countries worldwide.[8] This index measured respondents' past-day experiences of physical pain, worry, sadness, stress, and anger. The Gallup covariate was included as a means to test for a correlation between negative emotions at a location level and anxiety disorder prevalence. Data from the Gallup negative experience index was modelled using the spatiotemporal Gaussian process regression (ST-GPR) to produce estimates for all years and locations required by DisMod-MR. The log of the modelled output was used as the covariate in DisMod-MR due to skewedness of the data. The relationship detected was in the expected direction (ie, the higher the negative emotion, the higher the prevalence rate).

A summary of covariates and exponentiated values for anxiety disorders are shown in Table 3.

Table 3. Summary of covariates used in the anxiety disorders DisMod-MR meta-regression model

Covariate	Type	Parameter	Exponentiated beta (95% UI)
Mean war mortality rate in the previous 10 years	Location-level	Prevalence	1.67 (1.08–2.57)
Gallup: negative experience index	Location-level	Prevalence	1.61 (1.19–2.17)

Impact of the COVID-19 pandemic

Prevalence data from the COVID-19 systematic review were first analysed separately to the above DisMod-MR 2.1 analysis in order to investigate the change in prevalence of anxiety disorders during the COVID-19 pandemic.[5] The logit difference between pre-pandemic prevalence and prevalence during the pandemic was calculated for all eligible input data. A model to estimate the adjustment to prevalence was developed via a two-step process. In step one, an indicator model was run to develop an index for the impact of COVID-19. We then conducted a meta-regression via MR-BRT to predict the logit difference in prevalence from changes in human mobility (as captured by anonymous cell phone mobility data) and the IHME daily COVID-19 mortality rate, controlling for studies that compared mid-pandemic prevalence from a market research and quote sampling methodology against a prevalence from a random sample. We used the coefficients for these two indicators to calculate a single COVID-19 impact indicator for anxiety disorders. In step two, we developed a final model via backward elimination to regress the COVID-19 impact indicator and interactions between this indicator and age and sex. Bias covariates were also treated as interactions against the indicator except for studies that compared mid-pandemic prevalence from a market research and quota sampling methodology against a prevalence from a random sample, which were controlled for via a binary covariate on the change in logit prevalence. The least significant covariate was iteratively removed until no improvement was seen in the Akaike information criterion (See Table 4). This model was then used to predict the logit change in prevalence for every day of the years 2020 and 2021 by age, sex, and location. The 2020 and 2021 age-specific, sex-specific, and location-specific anxiety disorder prevalence estimated by DisMod-MR 2.1 (informed by prevalence data prior to 2020) was then adjusted by the predicted logit change from the MR-BRT model for every day of the years 2020 and 2021. Annual point prevalence estimates for 2020 and 2021 were then calculated as the average daily prevalence for the year.

Table 4. Meta-regression coefficients on the change in anxiety disorder logit prevalence over the course of the COVID-19 pandemic

Covariate	Coefficient	Uncertainty interval	***p***
COVID-19 impact indicator	0.765	0.547 to 0.982	0
Human mobility*	–0.200	–0.293 to –0.059	-
COVID-19 mortality rate*†	–67.200	–94.302 to –41.281	-
Mean or midpoint age	–0.018	–0.021 to –0.015	0
Proportion female	0.103	–0.013 to 0.22	0.080
Combined depressive and anxiety disorder symptoms	0.541	0.264 to 0.817	<0.001
Market research and quota sampling vs. market research and quota sampling	–1.842	–2.985 to –0.698	0.002
Market research and quota sampling vs. random sampling	–0.752	–1.163 to –0.342	<0.001

* Coefficients were estimated using the coefficient of the COVID-19 impact index multiplied by the coefficient of the COVID-19 impact indicators from the indicator model. †Square-root transformed before analysis to correct for positive skew.

Severity splits and disability weights

The GBD disability weight survey assessments include lay descriptions of sequelae highlighting major functional consequences and symptoms. The lay descriptions and disability weights for anxiety disorder severity levels are shown in Table 5. To determine the proportion of people with anxiety disorders within each of the severity levels, we used data from the Australian National Survey of Mental Health and Wellbeing of Adults (NSMHWB, conducted in 1997).[9] The proportion of anxiety disorder cases falling within each level of severity was: asymptomatic 16.8% (14.2–19.5), mild 42.4% (32.9–50.2), moderate 24.8% (18.9–31.0) and severe 16.1% (10.2–22.9). The same severity distribution and disability weights were applied to the pre-COVID-19 and post-COVID-19 prevalent cases of anxiety disorders.

Table 5. Severity distribution, details on the severity levels for anxiety disorders, and the associated disability weight with that severity

Severity level	Lay description	Disability weight (95% UI)
Mild	Feels mildly anxious and worried, which makes it slightly difficult to concentrate, remember things, and sleep. The person tires easily but is able to perform daily activities.	0.03 (0.018–0.046)
Moderate	Feels anxious and worried, which makes it difficult to concentrate, remember things, and sleep. The person tires easily and finds it difficult to perform daily activities.	0.133 (0.091–0.186)
Severe	Constantly feels very anxious and worried, which makes it difficult to concentrate, remember things, and sleep. The person has lost pleasure in life and thinks about suicide.	0.523 (0.362–0.677)

The addition of COVID-19 adjustment has meant that the prevalence of anxiety disorders increased in GBD 2021 compared to GBD 2019. The pandemic has created an environment where many determinants of mental health are also impacted. Social restrictions, lockdowns, school and business closures, loss of livelihood, and decreases in economic growth all have the potential to significantly impact mental health. In GBD 2021, we responded to this by incorporating a method to estimate the impact of COVID-19 on the prevalence and burden of anxiety disorders. That said, several limitations to this work need to be acknowledged. Data coverage was limited to high-income countries, with location-specific predictions relying on two COVID-19 indicators in the model – human mobility and IHME-estimated daily COVID-19 mortality. Our analysis relied on data from symptom scales capturing probable cases of anxiety disorders as very few diagnostic mental health surveys have been conducted during the COVID-19 pandemic. Our estimation of the impact of COVID-19 on mental disorders is still underway, with further improvements to be made as new epidemiological studies are published, and as we progress through various stages of the pandemic.

More broadly, across our entire epidemiological modelling process, it is also important to acknowledge that our case definition for anxiety disorders will need to be revised to better capture changes to the latest DSM/ICD criteria. Epidemiological estimates reporting an outcome for “any” or “total” anxiety disorders were included in GBD 2021 if they reported on at least three anxiety disorders. Future iterations of GBD will revisit the unique contribution of specific anxiety disorders. Secondly, we still have a large number of locations with no high-quality raw data available. Thirdly, it is difficult to quantify and

remove all variation due to measurement error in our epidemiological estimates. While we have improved the methodology used to account for known sources of bias, in some cases, we still have very few datapoints to inform these adjustments. Fourthly, there is a paucity of research on the risk factors of mental disorders which can be used as predictive covariates in our epidemiological models.

References

1. American Psychiatric Association. *Diagnostic and Statistical Manual of Mental Disorders (DSM-IV-TR)*. Fourth Edition, Text Revision ed. Washington DC: American Psychiatric Association; 2000.
2. World Health Organization. *The ICD-10 Classification of Mental and Behavioural Disorders. Clinical descriptions and diagnostic guidelines*. Geneva: World Health Organization; 1992.
3. Baxter AJ, Scott KM, Vos T, Whiteford HA. Global prevalence of anxiety disorders: a systematic review and meta-regression. *Psychological Medicine* 2013; **43**(05): 897-910.
4. Baxter AJ, Vos T, Scott KM, Ferrari AJ, Whiteford HA. The global burden of anxiety disorders in 2010. *Psychological Medicine* 2014; **44**(11): 2363-74.
5. Santomauro DF, Whiteford HA, Ferrari AJ. Depression and anxiety during COVID-19 - Authors' reply. *Lancet* 2022; **399**(10324): 518-9.
6. Karam E, Bou GM. Psychosocial consequences of war among civilian populations. *Current Opinion in Psychiatry* 2013; **16**(413–419).
7. Steel Z, Chey T, Silove D, Marnane C, Bryant RA, van Ommeren M. Association of torture and other potentially traumatic events with mental health outcomes among populations exposed to mass conflict and displacement: a systematic review and meta-analysis. *JAMA* 2009; **302**(5): 537-49.
8. Gallup G. The Gallup Poll: Public Opinion 2003: Rowman & Littlefield; 2004.
9. Australian Bureau of Statistics. National Survey of Mental Health and Wellbeing of Adults 1997. Canberra: Australian Bureau of Statistics.

Appendicitis

Flowchart

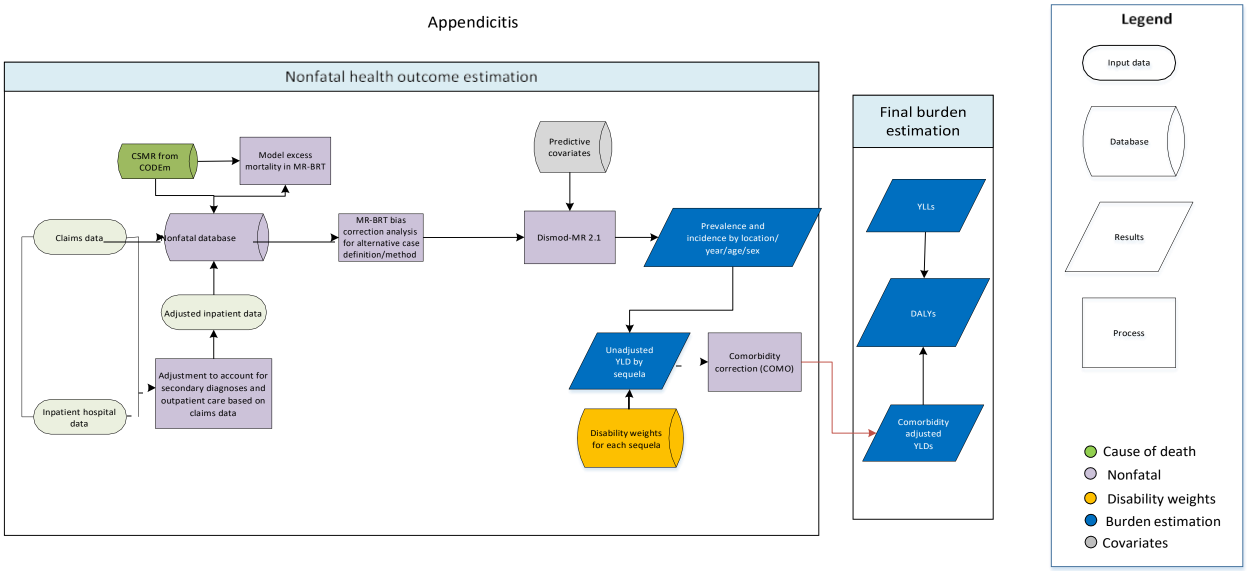

Input data and methodological summary for appendicitis

Case definition

Appendicitis is an inflammation of the appendix generally presenting with nausea, vomiting, and sharp pain in the right lower abdomen. Appendicitis carries risk of severe complications, including sepsis and death, and is usually treated surgically. ICD-10 codes included are K35-K35.3, K35.8, K35.80, K35.89, K35.9, K36, K36.0, K37, K37.0, K37.9, and K38.3.

Input data and data processing

Inputs

Like GBD 2019, the model included incidence data from hospital discharges and claims. In GBD 2021, we newly added additional years of data from USA claims (year 2017) and Poland claims (year 2018), as well as hospital discharges in Greece, Armenia, Chile, Ecuador, Argentina, Italy, Brazil, and Spain.

Table 1. Data Inputs for appendicitis morbidity modelling by parameter

	**Countries with data**	**New sources**	**Total sources**
Incidence	50	35	330

Inputs to our non-fatal modelling also included cause-specific mortality rate (CSMR) estimates taken from our fatal modelling process (see CoD cause-specific modelling description for appendicitis in this appendix) and excess mortality rates (EMR) estimates modelled outside of DisMod (see the EMR data processing section below).

Incidence data processing

Hospital discharge data provide observations about encounters, generally with only the primary diagnostic code for the encounter. Claims data, on the other hand, link claims for all inpatient and outpatient encounters for a single individual, and provide primary and secondary diagnoses for all encounters.

In GBD 2017, an individual was extracted from claims data as an incident case if that individual had one or more inpatient encounters with an appropriate ICD code as any diagnosis. Hospital discharges with an appropriate ICD code as primary diagnosis were extracted and adjusted for readmissions.

In both GBD 2019 and GBD 2021, however, we employed data processing methods to capture cases that were diagnosed and/or treated in both inpatient and outpatient settings. Specifically, an individual was extracted from claims data as an incident case if that individual had at least one inpatient or outpatient encounter with an appropriate ICD code as any diagnosis within one year. Hospital discharge data were processed by extracting discharges with an appropriate ICD code as primary diagnosis and adjusting using correction factors (ie, correction factor 3) derived from claims data. Specifically, we modelled from the ratio of inpatient claims with appendicitis as primary diagnosis to total incident cases of appendicitis seen in claims data. In GBD 2021, we updated the method of estimating these correction factors by assigning three frequency-placed knots, instead of two, in the age-spline parameter of MR-BRT analysis. Other processing methods remained the same as in GBD 2019.

As first done in GBD 2019, USA claims data (extracted and processed as described above) were adjusted to account for selection bias due to commercial insurance, using MR-BRT analysis. In contrast to GBD 2019, we used age as an additional covariate to estimate bias adjustment factors.

The process of adjusting for biases in non-reference data using MR-BRT with the logit-transformation method is described below:

1. Identify datapoints with overlapping year, age, sex, and location between commercial claims (non-reference data) and population-representative hospital discharges (reference data).
2. Logit transform overlapping datapoints of alternative and reference types.
3. Convert overlapping datapoints into a difference in logit space using the following equation: $logit(alternative) - logit(reference)$.
4. Use the delta method to compute standard errors of overlapping datapoints in logit space, then calculate standard error of logit difference using the following equation: $\sqrt{(variance\ of\ alternative) + (variance\ of\ reference)}$.
5. Using MR-BRT, conduct a random effects meta-regression to obtain the pooled logit difference of alternative to reference.
6. Apply the pooled logit difference to all datapoints of alternative case definitions using the following equation: $new_{estimate} = inverse.\ logit((logit(alternative)) - (pooled\ logit\ difference))$.
7. Calculate new standard errors using the delta method, accounting for gamma (between-study heterogeneity).

The table below shows bias correction factors estimated using MR-BRT.

Table 2. MR-BRT crosswalk adjustment factors for appendicitis

Data input	Reference or alternative data collection	Gamma	Covariate	Beta coefficient, logit (95% CI)	Adjustment factor*
Hospital + non-USA claims	Ref	0.002		---	---
USA claims from year 2000	Alt		Age (continuous from 0 to 95+)	0.01 (–0.08 to 0.10)	1.01 (0.92 to 1.10)
			Sex (female to male)	0.11 (–0.07 to 0.28)	1.11 (0.94 to 1.33)
			Intercept	–0.76 (–1.01 to –0.52	0.47 (0.37 to 0.60)
USA claims from years 2010–2017	Alt		Age (continuous from 0 to 95+)	0.004 (–0.06 to 0.07)	1.00 (0.94 to 1.08)
			Sex (female to male)	0.14 (–0.30 to 0.58)	1.15 (0.74 to 1.78)
			Intercept	–0.825 (1.40 to –0.25)	0.44 (0.25 to 0.78)

**MR-BRT crosswalk adjustments can be interpreted as the factor the alternative case definition is adjusted by to reflect what it would have been had it been measured using the reference case definition. If the log/logit beta coefficient is negative, then the alternative is adjusted up to the reference. If the log/logit beta coefficient is positive, then the alternative is adjusted down to the reference.*

***The adjustment factor column is the exponentiated beta coefficient. For log beta coefficients, this is the relative rate between the two case definitions. For logit beta coefficients, this is the relative odds between the two case definitions.*

Datapoints with an age-standardised incidence rate greater than two median absolute deviations from the median of the age-standardised incidence rate for all data were marked as outliers and excluded from analysis.

EMR processing

In GBD 2017, EMR inputs were produced by matching prevalence datapoints with their corresponding CSMR values within the same age, sex, year, and location (by dividing CSMR by prevalence). For short-duration conditions (remission >1), the corresponding prevalence was derived by running an initial model and then applying the same CSMR/prevalence method. However, this method of producing EMR inputs demonstrated a rather unrealistic pattern of EMR compared to an expected pattern of decreasing EMR with greater access to quality health care. Such unexpected patterns often signal inconsistencies between CSMR estimates and the measures of prevalence and/or incidence. Thus, in an effort to provide greater guidance on the expected pattern of EMR, in GBD 2019, EMR data produced per above in GBD 2017 were modelled by age, sex and Healthcare Access and Quality (HAQ) Index using MR-BRT, with a prior on HAQ Index having a negative coefficient. In GBD 2021, we employed the same MR-BRT method to predict EMR for each location, year, sex, and for ages 0, 10, 20….100. These predictions were used as inputs to our non-fatal model, below.

Modelling strategy

DisMod model

Similar to previous rounds, we ran a DisMod-MR 2.1 model to produce estimates by age, sex, year, and location. Inputs to DisMod for appendicitis include incidence, CSMR, and EMR inputs processed as described above. A prior value was set on remission so that all cases remit within two weeks. The minimum coefficient of variation at the regional, super-regional, and global level was set at 0.8. We included HAQ Index as a predictive covariate on EMR with a mean and standard deviation produced from the MR-BRT model described above. The fibre (g per day) consumption covariate was included as a predictive covariate on incidence. Betas and exponentiated values (which can be interpreted as odds ratios) of predictive covariates are shown in the table below.

Table 3. Covariates. Summary of covariates used in the appendicitis DisMod-MR meta-regression model

Covariate	Parameter	Exponentiated beta (95% uncertainty interval)
Fibre, unadjusted (g)	Incidence	0.99 (0.99–1.00)
Healthcare Access and Quality Index	Excess mortality rate	0.95 (0.95–0.95)

Severity split & disability weight

The basis of the GBD disability weight survey assessments are lay descriptions of sequelae highlighting major functional consequences and symptoms. The lay descriptions and disability weights for appendicitis are shown below.

Table 4. Severity distribution, details on the severity levels for appendicitis in GBD 2021 and the associated disability weight (DW) with that severity

Severity level	Lay description	DW (95% CI)
Severe	This person has severe pain in the belly and feels nauseated. The person is anxious and unable to carry out daily activities.	0.324 (0.219–0.442)

Ascariasis

Flowchart

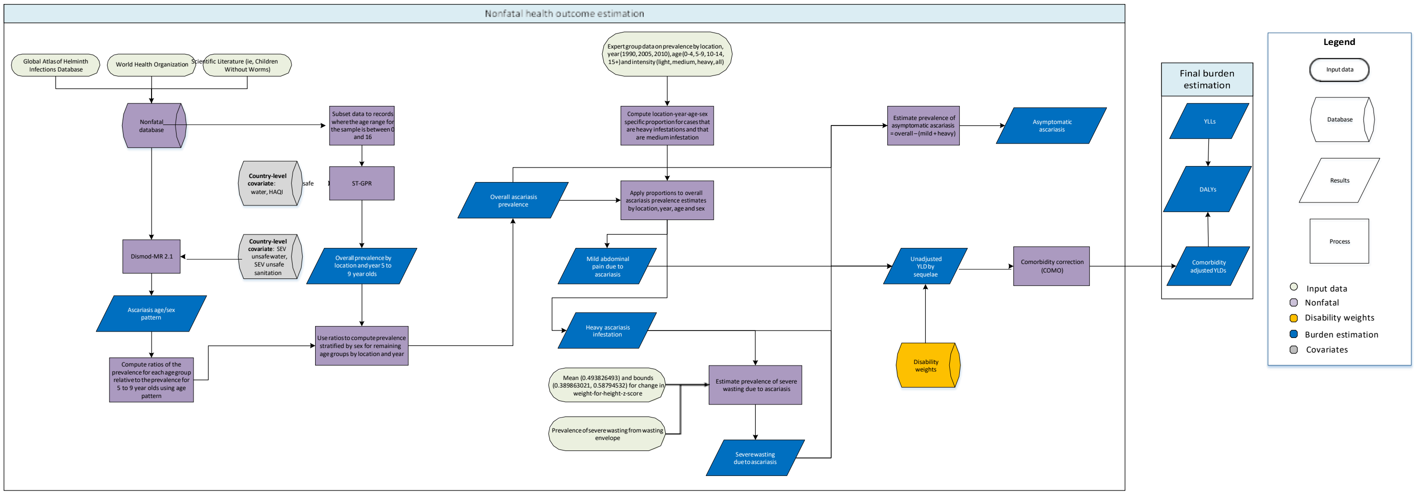

Input data and methodological summary for ascariasis

Case definition

Ascariasis is a helminthic disease caused by the parasitic roundworm *Ascaris lumbricoides* that can cause abdominal pain, nausea, vomiting, diarrhea, and malnutrition and complications such as intestinal obstruction or hepatobiliary and pancreatic disease. It is one of the three intestinal nematode infections, or soil-transmitted helminthiasis (STH), modelled in GBD. Diagnosis is made by examination of stool by microscope or PCR, with or without concentration procedures. The ICD-10 codes for ascariasis are B77-B77.9.

We used the following case definition for GBD 2021:

Quantity of interest	Reference or alternative	Definition

Ascariasis	Reference	Diagnosis made by examination of stool using Kato-Katz technique, resulting in positive for intestinal helminth eggs of type A. lumbricoides.

Input data

The primary input data for this model was from the Global Atlas of Helminth Infections (GAHI) database and the Expanded Special Project for the Elimination of Neglected Tropical Diseases (ESPEN). The GAHI and ESPEN databases include surveys and studies conducted to measure the prevalence of STH[1]. Each record in the database contained metadata (ie, location, year, age range, sex) of each study sample and the prevalence of ascariasis in that sample.

We supplemented the GAHI and ESPEN data with survey-data collected in a literature review performed by Children Without Worms (2006-2016), which included countries outside of Sub-Saharan Africa, , and additional data provided by the World Health Organization (WHO). For all input data, we excluded data points where the age range of the sample was unknown and retained only those surveys utilizing the Kato-Katz diagnostic method.

Table 1: Data inputs for Ascariasis morbidity modelling by parameter.

Measure	Countries with data	New sources	Total sources
All measures	140	52	218
Prevalence	83	52	217
Proportion	134	0	1

Geographic restrictions

We conducted a literature review (last updated for GBD 2017) to determine the geographic extent of the disease and classify locations based on whether the disease is absent or present in each year. Locations that were geographically restricted in any given year did not have estimates made for them. Of note, we did not attempt a complete systematic review, since a single high-quality source could offer sufficient evidence of presence. Evidence of absence or presence was not available for every location for each year. Assumptions made for missing years took into consideration the epidemiological characteristics of the disease.

If evidence indicated disease presence for two non-consecutive years, we assumed presence for all years between the two. If evidence indicated disease absence for two non-consecutive years, we assumed absence for all years between the two. If evidence indicated a change in status (ie, from absent to present, or present to absent) between two non-consecutive years, then we conducted targeted searches to ascertain the relevant year of introduction or elimination for that location. In the cases where presence or absence information was missing for the start or end years of our study interval without evidence of any introduction or elimination events within the interval, we applied the status of the first and last presence/absence observations, respectively, to all years between the interval bound and the observation year. Table 2 shows the search strings and associated yield for each of the databases queried.

Table 2. Geographic restriction search strings

Database	Search string	Yield

PubMed	(Ascariasis[Title/Abstract] OR Ascaris[Title/Abstract] OR "A. lumbricoides"[Title/Abstract] OR Ascaris[MeSH] OR Trichuris[Title/Abstract] OR Trichuriasis[Title/Abstract] OR "Whip Worm"[Title/Abstract] OR "T. trichura"[Title/Abstract] OR Trichuris[MeSH] OR Hookworm[Title/Abstract] OR "A. duodenale"[Title/Abstract] OR "Ancylostoma duodenale"[Title/Abstract] OR ancylostomiasis[Title/Abstract] OR "N. americanus"[Title/Abstract] OR "Necator americanus"[Title/Abstract] OR necatoriasis[Title/Abstract] OR Ancylostoma [MeSH] OR Necator[MeSH]) AND (prevalence[Title/Abstract] OR incidence[Title/Abstract] OR epidemiology[Title/Abstract] OR surveillance[Title/Abstract]) NOT(Animals[MeSH] NOT Humans[MeSH])	2,376
Web of Science	(Ascariasis OR Ascaris OR A. lumbricoides OR Trichuris OR Trichuriasis OR Whip Worm OR T. trichura OR Hookworm OR A. duodenale OR Ancylostoma duodenale OR anclyostomiasis OR N. americanus OR Necator americanus OR necatoriasis) AND TOPIC:(prevalence OR incidence OR epidemiology OR surveillance) NOTTOPIC: ((Animals NOT Humans))<br>Timespan: 1980-2016. Indexes: SCI-EXPANDED, SSCI, A&HCI, ESCI.	2,266
SCOPUS	TITLE-ABS_KEY (ascariasis OR ascaris OR a. lumbricoides OR trichuris OR trichuriasis OR whip worm OR t. trichura OR hookworm OR a. duodenale OR ancylostoma duodenale OR anclyostomiasis OR n. americanus OR necator americanus OR necatoriasis) AND PUBYEAR>1979	29

These papers classified location-years for all locations and years present in the literature. We only utilised papers that are explicitly concerned with ascariasis. Additionally, systematic literature reviews, meta-analyses, national health statistics publications, and collaborator input supported classification of location-years not present in the literature review wherever possible.

Modeling strategy

DisMod-MR

In the estimation of overall morbidity due to ascariasis, we implemented a three-stage modelling framework. The first stage of the modelling process was using a DisMod Bayesian Meta-Regression model (DisMod-MR), to generate a global age-sex curve to disaggregate all-age, both-sex prevalence data. DisMod-MR is an integrated meta-regression framework that allows multiple datasets to be used within a singular analysis regardless of age-binning, sources, and geographies. As a result, a variety of differently aggregated information combine to generate a consensus output. Our final model contained all processed GAHI data as input informed by two country-level covariates (ie, SEV for unsafe water and unsafe sanitation).

Table 3a. Covariates. Summary of covariates used in the ascariasis DisMod-MR model

Covariate	Type	Parameter	Exponentiated beta (95% UI)
SEV unsafe water	Country-level	Proportion	4.45 (4.39 — 4.48)
SEV unsafe sanitation	Country-level	Proportion	4.45 (4.38 — 4.48)

Figure 1: Global age-specific proportion estimates for males (left) and females (right) for the year 2020. Proportion (prevalence) is on the y-axis, and age in years on the x-axis.

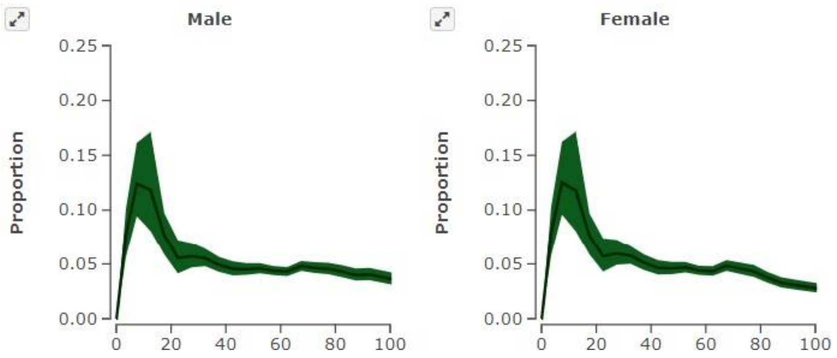

Figure 1 shows the age-specific variation in the proportion of prevalence, differentiated by sex. When considered as a global aggregate, we see that reported male and female prevalence are very similar. We use the age-specific proportions to adjust the output of the spatiotemporal Gaussian process regression (ST-GPR – see below for method) to predict prevalence for each age group.

ST-GPR

We then utilise a ST-GPR to generate a complete time series of estimates for each location where there are no geographical restrictions. ST-GPR attempts to model non-linear trends utilising a Gaussian process to fit a trend. We ran an age-restricted ST-GPR model, using all data with age bins between 0 and 16 because these data fall within the peak in prevalence across all age groups, the majority of data fall within these age ranges, and these data provide sufficient statistical power for our model. This left us with 292 site-years of input data. The following were the model specifications:

$$Prevalence = Proportion\ Safe\ Water + Healthcare\ Access\ and\ Quality\ Index + (1|level\ 2) + (1|level\ 3)$$

Levels 2 and 3 refer to GBD location hierarchies, or random effects for region and location. Notably, the covariates for the model were Healthcare Access and Quality (HAQ) Index and safe water or proportion of population with access to improved water sources. Improved water sources are defined by the Joint Monitoring Programme.[2] The following hyperparameters were used: st_lambda = 0.35, st_omega = 2, st_zeta = 0.01, gpr_scale = 15. We selected these hyperparameters as they provided more weight to country-level data rather than region-level data when estimating the prevalence for a given location-year, ensuring that the Gaussian process regressions follow country-specific data rather than region-specific data when estimating a time series for a location.

Table 3b. Covariates: summary of covariates used in the ascariasis ST-GPR model

Covariate	Beta coefficient, log (95% UI)	Standard error	Exponentiated beta (95% UI)
Improved water	-0.346 (-1.602 to 2.294)	0.994	0.707 (0.101–4.964)
HAQ Index	-0.055 (-0.102 to -0.008)	0.024	0.946 (0.903–0.992)

Imputations

The final stage of the overall prevalence modelling process is to impute the remaining age groups by borrowing information from the DisMod-MR global age-sex pattern and ST-GPR time series by first assuming the estimates from ST-GPR are representative of the 5–9-year-old age group. Each additional

age group is assigned a ratio representing how much larger or smaller the prevalence is compared to the

prevalence of the reference group (5–9-year-olds) using the DisMod-MR global age-sex pattern. The following is the computation for each age group:

$$Ratio = \frac{prevalence_{[age\ start] to\ [age\ end]}}{prevalence_{5\ to\ 9}}$$

With a ratio for every age group by sex, we multiplied the ratio by the ST-GPR location-year estimates to impute estimates for the remaining age groups.

Health states/sequelae

The Table 4 shows the list of sequelae due to ascariasis and the associated disability weights (DW). Prevalence of medium infection and heavy infection mapped to *mild abdominopelvic problems* and *heavy infestation of ascariasis,* respectively. Light infection or asymptomatic were not attributed any disability.

Table 4. Severity distribution, details on the severity levels for ascariasis, and the associated DW with that severity

Sequela	Lay description	DW (95% CI)
Mild abdominopelvic problems	Has some pain in the belly that causes nausea but does not interfere with daily activities	0.011 (0.005–0.021)
Heavy infestation	Has cramping pain and a bloated feeling in the belly	0.027 (0.015–0.043)
Severe wasting	Is extremely skinny and has no energy	0.128 (0.082–0.183)
Asymptomatic ascariasis	N/A	N/A

Following computations of location-year-age-sex-specific prevalence of ascariasis, we leverage information from the 2010 Expert Group (EG) data to conduct sequelae splits. The 2010 EG data provided estimates for heavy infestation, mild abdominopelvic problems, and asymptomatic ascariasis by location and for 1990, 2005, and 2010. These three values add up to *all cases* of ascariasis. Thus, for heavy infestation and mild abdominopelvic problems, we computed the proportion of cases that belong to our sequelae of interest over *all cases* of ascariasis. The following is the equation utilised to calculate heavy infestation and mild abdominopelvic problems:

$$Proportion_{sequelae} = \frac{prevalence_{sequelae}}{prevalence_{all\,cases}}$$

This calculates proportions for every location, year, and age group available. The 2010 EG data only had four age groups (0–4, 5–9, 10–14, 15+ years), so we applied the 15+ age group proportion for all remaining age groups. In addition, for the years 1995 and 2000, we applied the 1990 proportions, and for years 2015, 2019, and 2020-2021, we applied the 2010 proportions. Using these location-year-age-specific proportions, we multiplied the total ascariasis estimates to compute heavy infestation and mild abdominopelvic prevalence. To estimate the prevalence of asymptomatic ascariasis, prevalence of mild and heavy infestation were each subtracted from the overall ascariasis prevalence.

The final step in the modelling process was to estimate the prevalence of severe wasting due to ascariasis in age groups 1–5 months, 6–11 months, 12–23 months, and 2–4 years. This was done separately using 1000 draws of prevalence of heavy infestation due to ascariasis and the wasting envelope prevalence. The initial step in determining prevalence of severe wasting due to ascariasis was generating 1000 draws of

change in weight-for-height z-score per heavy prevalent case from a random normal distribution with

mean = 0.493826493 and standard deviation = 0.04972834 (calculated from upper and lower bounds of the mean estimate). The mean and upper and lower bounds were calculated using findings from a meta-analysis[3] (implemented in GBD 2013). The prevalence of severe wasting due to ascariasis was then obtained as a function of change in weight-for-height z-score. The following are the computations:

$$Prevalence_{wasting\ due\ to\ ascariasis} = wasting - \Phi(\Phi^{-1}(\text{wasting}) - \text{z score} * \text{heavy infestation})$$

Where Φ is the standard normal cumulative distribution function and Φ^{-1} is the inverse standard normal cumulative distribution function.

Changes from GBD 2019

The major change from GBD 2019 was in specifying new covariates for the ST-GPR global prevalence model, specifically in removing the WHO STH mass drug administration (MDA) covariate due to noise in the data causing sharp fluctuations in estimates. In future modelling, we plan to re-incorporate MDA coverage either as a covariate and/or by relating treatment to the distribution of severity after developing methods to account for noise in the underlying data.

There were also data changes between the rounds. New data inputs from WHO and ESPEN were added to the model. In addition, nationally tagged data in Nigeria and the Philippines were re-tagged to appropriate subnational locations.

We did not apply any adjustments to ascariasis for the COVID-19 pandemic due to a lack of available data quantifying the impacts of the pandemic on neglected tropical disease epidemiology.

Limitations

As we attempt to improve the modelling processes for ascariasis, we recognise several limitations. We only include studies where Kato-Katz identifies infected individuals. Future updates to the model will include a systematic review for within-study comparisons of diagnostic performance to facilitate a diagnostic crosswalk model.

A secondary limitation to our data is that several included studies are not nationally representative, and therefore at a location level, the data are highly heterogeneous . Numerous studies within the database come from districts or townships, and in some cases, the studies were done in areas where prevalence is known to be high.

Furthermore, we made a large assumption that the global age-sex distributions were applicable to all locations. While we believe that prevalence should peak among adolescents and slowly decline afterward, there is likely variation across regions and locations. Given that our data are among children or all ages, it is very difficult to build an age trend at granular location levels. Thus, we allowed DisMod-MR to disaggregate our heterogeneous data in an effort to provide sensible age-sex curves.

We believe that more work will improve our sequelae split methods. Since the 2010 EG data do not provide all estimation years and age groups, several assumptions had to be made. Thus, we will explore conducting literature searches to provide novel datapoints for sequelae estimations.

References

1. London School of Hygiene and Tropical Medicine. Global Atlas of Helminth Infections – Soil Transmitted Helminths. London, United Kingdom: London School of Hygiene and Tropical Medicine.

2. "Improved and Unimproved Water Sources and Sanitation Facilities." *WHO / UNICEF Joint Monitoring Programme: Wat/san Categories*. The WHO/UNICEF, n.d. Web. 08 June 2016.
3. Hall A, Hewitt G, Tuffrey V, de Silva N. A review and meta-analysis of the impact of intestinal worms on child growth and nutrition. *Maternal and Child Nutrition*. 2008. 4: 118-236.

Asthma

Flowchart

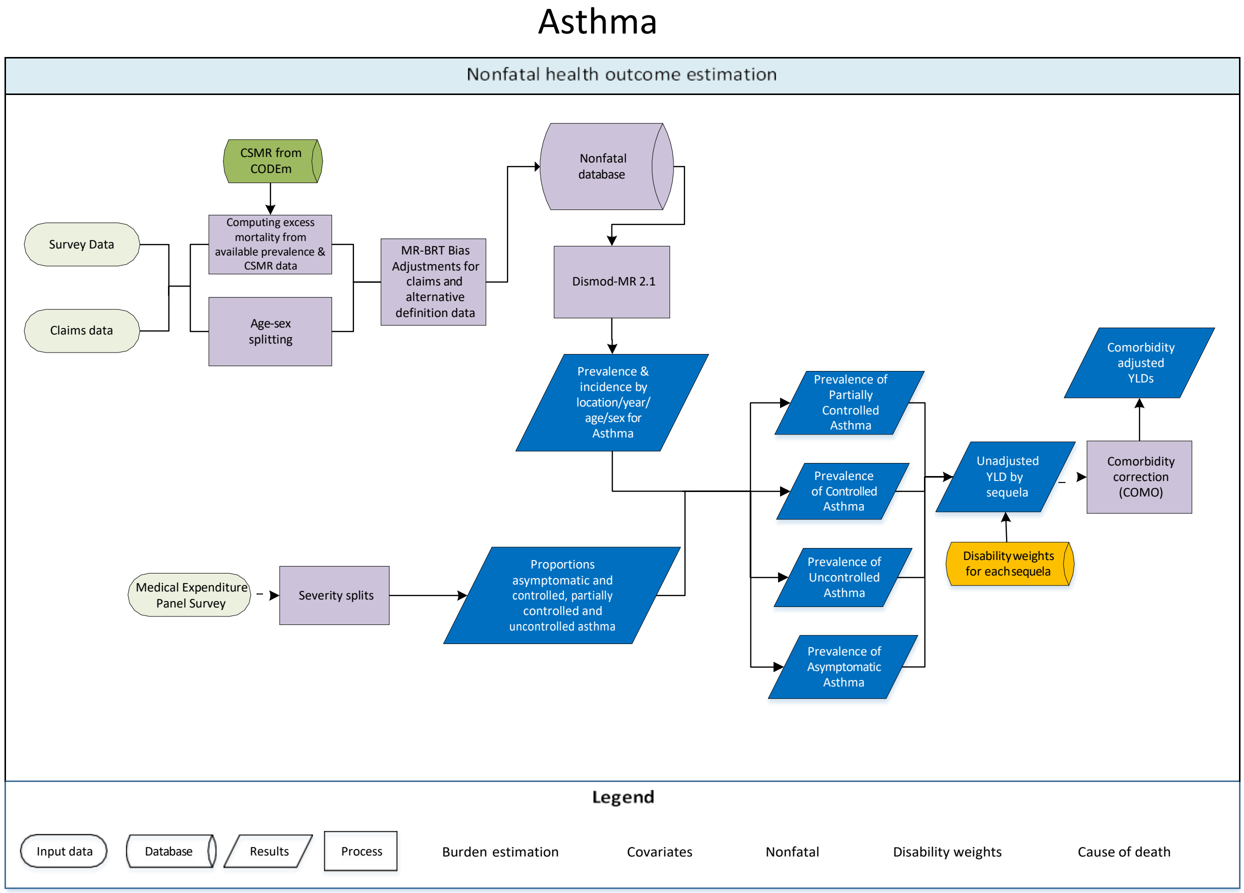

Case definition

Asthma is a chronic lung disease marked by spasms in the bronchi usually resulting from an allergic reaction or hypersensitivity and causing difficulty in breathing. We define asthma as a doctor's diagnosis and wheezing in the past year. The relevant ICD-10 codes are J45 and J46. ICD-9 code is 493.

Alternative case definitions include the following:

- Self-reported asthma in the past year
- Self-reported asthma ever
- Only a doctor's diagnosis in the past year
- Only wheezing in the past year

Input data

The last full systemic review of the literature on asthma was done for GBD 2016. The following search string was used in PubMed and filtered by studies of humans published between January 2012 and November 2016.

(Asthma[Title/Abstract] AND prevalence[Title/Abstract] AND "Cross-Sectional Studies"[MeSH Terms])

Data in literature matching our case definitions were extracted. Those that had definitions outside our alternative case definitions were not included. We also added new data for Wave 7 of the English Longitudinal Study of Ageing (ELSA). Surveys carried out as part of the International Study of Asthma and Allergies in Childhood (ISAAC) collaboration are the most important source of prevalence data in children.

Data inputs for asthma

Parameter	Countries with data	New sources	Total sources
Prevalence	136	15	389
Incidence	8	4	15
Remission	16	0	28
Other	4	0	22

Data processing

Age and sex split

In some cases, data are reported by only age or only sex, but not both. For example, a study may have included the prevalence of males and females with asthma and then separately reported the prevalence of both sexes combined in smaller age bins (eg, 40–45 years, 46–50, etc.) that have asthma. In these cases, we perform an age-sex split by utilising proportions within the study to disaggregate the data.

When data are not disaggregated into male and female categories, we instead perform a sex-split on the data by applying sex proportions from outside studies. The sex split analysis was carried out using MR-BRT[2] (meta-regression—Bayesian, regularised, trimmed, described in appendix 1, section 4.4.1 of the reference) and included a cubic spline on age to reflect the higher prevalence of asthma in males at young ages, which then transitions to a higher prevalence of asthma in females during the teenage years. When data are aggregated into age categories larger than 25 years, we split the data into smaller age bins based on the global age pattern from an initial DisMod model that only included input data with age ranges under 25 years.

Bias adjustments

In GBD 2021, we adjusted alternative case definition data or study designs using MR-BRT.

We made a series of adjustments to data that do not completely match our case definition, doctor's diagnosis and wheezing in the past year. The estimation of asthma in a population varies slightly by the case definition used (wheezing and diagnosis, only wheezing, etc.). Similarly, claims data are subject to biases. An analysis for GBD 2017 showed that claims data were systemically lower than asthma survey data, probably reflecting selection bias with regard to socioeconomic status. Adjustments are made to these data to correct these biases.

The adjustment is a logit-transformation method in MR-BRT. The general process is described below:

8. Identify datapoints with overlapping year, age, sex, and location between reference and alternative definitions.
9. Logit transform overlapping datapoints of alternative and reference case definitions.
10. Convert overlapping datapoints into a difference in logit space using the following equation: $logit(altnerative) - logit(reference)$
11. Use the delta method to compute standard errors of overlapping datapoints in logit space, then calculate standard error of logit difference using the following equation: $\sqrt{(variance\ of\ alternative) + (variance\ of\ reference)}$
12. Using MR-BRT, conduct a random effects meta-regression to obtain the pooled logit difference of alternative to reference.
13. Apply the pooled logit difference to all datapoints of alternative case definitions using the following equation:
$$new_{estimate} = inverse.logit((logit(alternative)) - (pooled\ logit\ difference))$$
14. Calculate new standard errors using the delta method, accounting for gamma (between-study heterogeneity).

Data derived from claims from commercial health insurance in the United States were also adjusted using a factor estimated in MR-BRT. To account for this, we estimated a MarketScan 2000 coefficient and a separate MarketScan coefficient for the remaining years of MarketScan data by comparing the national values in these datasets to national asthma estimates from the USA National Health and Nutrition Examination Survey and National Health Interview Surveys.

The coefficients for bias adjustments are shown:

MR-BRT crosswalk adjustment factors

Data input	Status	Gamma	Beta coefficient, logit* (95% UI)	Adjustment factor**
Wheezing + doctor's diagnosis	Ref	0.26	---	---
Only wheezing	Alt		1.09 (0.61, 1.59)	0.75 (.65, 0.83)
Only diagnosis	Alt		0.99 (0.50, 1.48)	0.73 (0.62, 0.82)
Self-reported currently have asthma	Alt		.01 (-0.48, 0.56)	0.50 (0.38, 0.64)
Self-reported ever having asthma	Alt		0.66 (0.11, 1.20)	0.66 (0.53, 0.77)
MarketScan 2000	Alt	0.00	-1.35 (-1.37, -1.33)	0.21 (0.20, 0.21)
MarketScan 2010 - 2016	Alt	0.60	-1.60 (-2.71, -0.43)	.17 (.06, .41)

**MR-BRT crosswalk adjustments can be interpreted as the factor the alternative case definition is adjusted by to reflect what it would have been had it been measured using the reference case definition. If the log/logit beta coefficient is negative, then the alternative is adjusted up to the reference. If the log/logit beta coefficient is positive, then the alternative is adjusted down to the reference.*

***The adjustment factor column is the exponentiated beta coefficient. For log beta coefficients, this is the relative rate between the two case definitions. For logit beta coefficients, this is the relative odds between the two case definitions.*

Modelling strategy

We use DisMod-MR 2.1[1] (disease model—Bayesian meta-regression, described in appendix 1, section 4.5) as the main modelling tool for asthma. Prior settings include a maximum remission of 0.3 (reflecting the upper bound of the highest observed data) and no incidence between the ages of 0 and 0.5 year, as a diagnosis cannot be made in young infants.

Predictive covariates

To assist estimation, particularly in locations with few or no data, we included covariates in our DisMod model that are associated with measures of asthma epidemiology in prior studies and for which estimates of those covariates are available for all GBD year-age-sex-location combinations. Specifically, we use log LDI and the asthma summary exposure variable (SEV), a scalar that combines exposure of all GBD risks that influence asthma.

Covariate	Measure	Beta	Exponentiated
Healthcare Access and Quality Index	EMR	-0.023<br>(-0.023 to -0.022)	0.98<br>(0.98–0.98)
Log SEV scalar: asthma	prevalence	0.75<br>(0.75–0.76)	2.13<br>(2.12–2.14)
Log LDI (I$ per capita)	excess mortality rate	-0.5<br>(-0.5 to -0.5)	0.61<br>(0.61–0.61)

Severity split inputs

Lay descriptions and disability weights for the asthma health states are shown in the table below. The distribution between the three health states is derived from an analysis of the USA Medical Expenditure Panel Surveys (MEPS). The methods are described in full in a separate section of this appendix. Briefly, MEPS is an ongoing survey of health service encounters with as its main objective to collect data on health expenditure. Panels are recruited every year and followed up for a period of two years. Diagnostic information provided by respondents on the reasons for any health care contact are coded into three-digit ICD-9 codes by professional coders.

Twice over the two-year follow-up period, respondents are asked to fill in 12-Item Short Form Surveys (SF-12). From convenience samples asking respondents to fill in SF-12 for 60 of the GBD health states, IHME has created a mapping from SF-12 scores to GBD disability weights (DW). We perform a regression with indicator variables for all GBD causes that we can identify from the ICD codes in MEPS to derive for each individual with a diagnosis the amount of disability that can be attributed to that condition after controlling for any comorbid conditions. Anyone with a diagnosis of asthma in whom the disability assigned to asthma is negative or zero we assume is asymptomatic (at the time of asking SF-12 questions relating to their health status in the past four weeks). Non-zero values we bin into the three health states assuming a split between these at the midpoint between DW values. The table below gives the proportions in MEPS in each of the health states and an asymptomatic state.

Severity level	Lay description	DW (95% CI)	Severity distribution

Asymptomatic			36.2% (35.0–37.3%)
Controlled	This person has wheezing and cough once a month, which does not cause difficulty with daily activities.	0.015 (0.007–0.026)	19.9% (13.6–27.8%)
Partially controlled	This person has wheezing and cough once a week, which causes some difficulty with daily activities.	0.036 (0.022–0.055)	20.6% (15.1–25.8%)
Uncontrolled	This person has wheezing, cough, and shortness of breath more than twice a week, which causes difficulty with daily activities and sometimes wakes the person at night.	0.133 (0.086–0.192)	23.3% (18.7–30.3%)

References

1. Vos T, Lim SS, Abbafati C, et al. Global burden of 369 diseases and injuries in 204 countries and territories, 1990–2019: a systematic analysis for the Global Burden of Disease Study 2019. The Lancet 2020; 396: 1204–22. doi: https://doi.org/10.1016/S0140-6736(20)30925-9
2. Murray CJL, Aravkin AY, Zheng P, et al. Global burden of 87 risk factors in 204 countries and territories, 1990–2019: a systematic analysis for the Global Burden of Disease Study 2019. The Lancet 2020; 396: 1223–49. doi: https://doi.org/10.1016/S0140-6736(20)30752-2

Atrial fibrillation and flutter

Flowchart

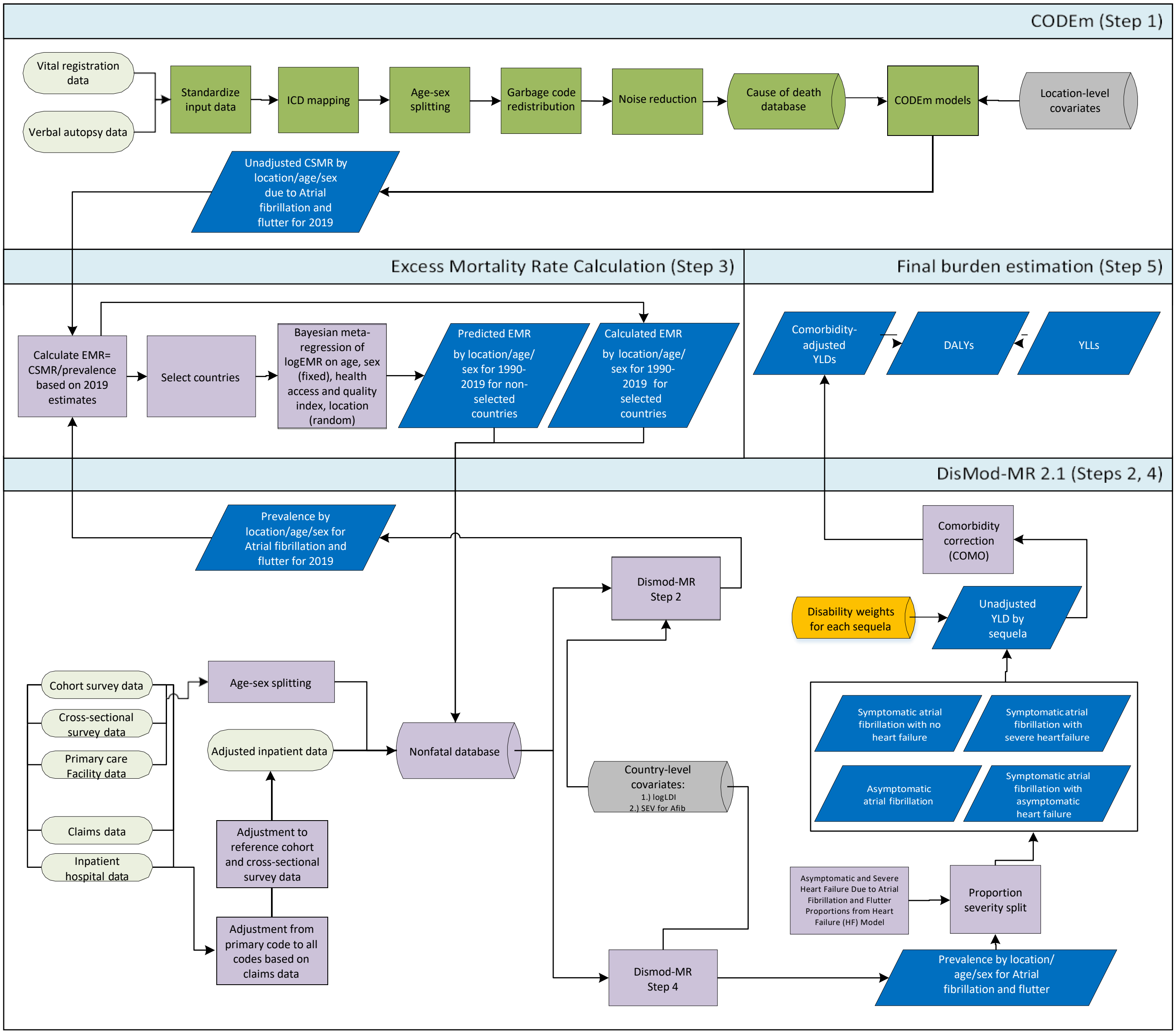

CODEm (Step 1)
Vital registration data
Verbal autopsy data
Standardize input data
ICD mapping
Age-sex splitting
Garbage code redistribution
Noise reduction
Cause of death database
CODEm models
Location-level covariates
Unadjusted CSMR by location/age/sex due to Atrial fibrillation and flutter for 2019
Excess Mortality Rate Calculation (Step 3)
Final burden estimation (Step 5)
Calculate EMR= CSMR/prevalence based on 2019 estimates
Select countries
Bayesian meta-regression of logEMR on age, sex (fixed), health access and quality index, location (random)
Predicted EMR by location/age/sex for 1990-2019 for non-selected countries
Calculated EMR by location/age/sex for 1990-2019 for selected countries
Comorbidity-adjusted YLDs
DALYs
YLLs
DisMod-MR 2.1 (Steps 2, 4)
Prevalence by location/age/sex for Atrial fibrillation and flutter for 2019
Comorbidity correction (COMO)
Dismod-MR Step 2
Disability weights for each sequela
Unadjusted YLD by sequela
Cohort survey data
Cross-sectional survey data
Primary care Facility data
Claims data
Inpatient hospital data
Age-sex splitting
Adjusted inpatient data
Nonfatal database
Adjustment to reference cohort and cross-sectional survey data
Adjustment from primary code to all codes based on claims data
Country-level covariates:
1.) logLDI
2.) SEV for Afib
Symptomatic atrial fibrillation with no heart failure
Symptomatic atrial fibrillation with severe heartfailure
Asymptomatic atrial fibrillation
Symptomatic atrial fibrillation with asymptomatic heart failure
Asymptomatic and Severe Heart Failure Due to Atrial Fibrillation and Flutter Proportions from Heart Failure (HF) Model
Proportion severity split
Dismod-MR Step 4
Prevalence by location/age/sex for Atrial fibrillation and flutter

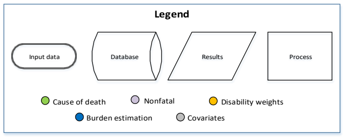

Legend
Input data
Database
Results
Process
Cause of death
Nonfatal
Disability weights
Burden estimation
Covariates

Input data and methodological summary

Case definition

Atrial fibrillation is a supraventricular arrhythmia due to disorganised depolarisation of the atrium. Atrial flutter is a macro-reentrant supraventricular arrhythmia, usually involving the cavo-tricuspid isthmus. Diagnosis requires an electrocardiogram (ECG) demonstrating 1) irregularly irregular RR intervals (in the absence of complete AV block); 2) no distinct P waves on the surface ECG, and 3) an atrial cycle length (when visible) that is usually variable and less than 200 milliseconds.

The International Classification of Disease (ICD) codes used for inclusion of hospital and claims are I48-I48.9 for ICD-10 and 427.3 for ICD-9.

Input data

Model inputs

Table 1 shows the source counts for atrial fibrillation and flutter in GBD 2021.

Measure	Total sources	Countries with data
All measures	300	50
Prevalence	260	45
Incidence	21	12
Excess mortality rate	5	5
With condition mortality rate	23	15
Relative risk	1	1
Standardised mortality ratio	2	2

We performed a systematic review for GBD 2021, our first update since GBD 2015. We searched PubMed, Embase, and the virtual health library databases for sources. The dates of the search were 1/1/2015–12/31/2019. The search strings used in the systematic review are shown below:

PubMed: ("atrial fibrillation"[TIAB] OR "atrial flutter"[TIAB]) AND (incidence[TIAB] OR prevalence[TIAB] OR "case fatality"[TIAB] OR "excess mortality"[TIAB]) AND ("2015/01/01"[PDAT]: "2019/12/31"[PDAT]) NOT dog NOT animal NOT mice NOT goat NOT pig

Embase: ('atrial fibrillation':ab,ti OR 'atrial flutter':ab,ti) AND (incidence:ab,ti OR prevalence:ab,ti OR 'case fatality':ab,ti OR epidemiology:ab,ti OR 'excess mortality':ab,ti) NOT (rats:ab,ti OR mice:ab,ti OR dogs:ab,ti OR apes:ab,ti OR fish:ab,ti OR monkeys:ab,ti) AND [2015-2019]/py AND ('article'/it OR 'article in press'/it OR 'conference paper'/it OR 'review'/it)`

Virtual Health Library: (tw:("atrial fibrillation") OR tw:("atrial flutter")) AND (tw:(incidence) OR tw:(prevalence) OR tw:(mortality) OR tw:("case fatality") OR tw:(epidemiology)) AND NOT (tw:(rats) OR tw:(mice) OR tw:(dogs) OR tw:(apes) OR tw:(fish) OR tw:(monkeys)) AND (year_cluster:[2015 TO 2019])

Figure 1 shows a PRISMA diagram that displays the text review and extraction process.

Figure 1: PRISMA diagram

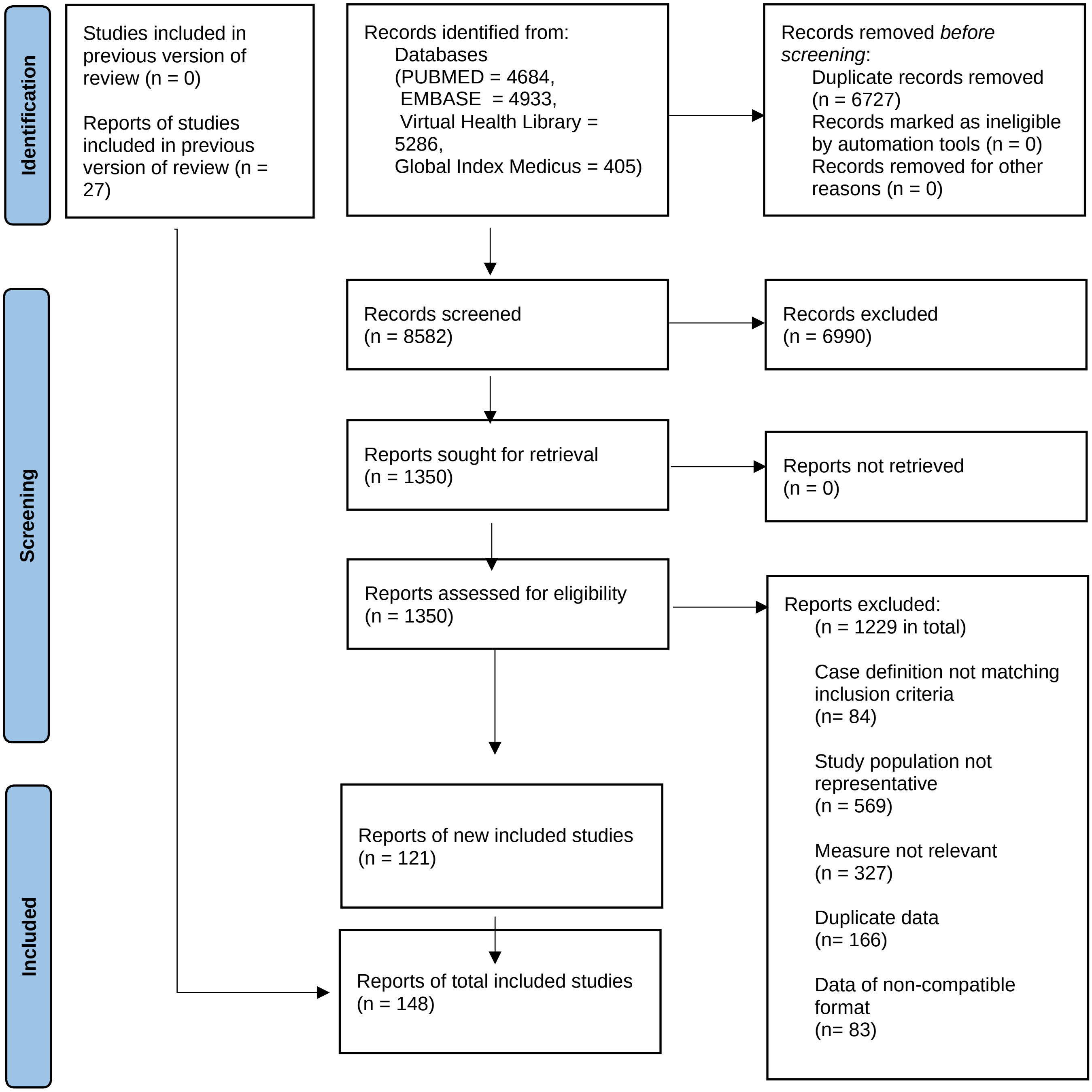

Apart from hospital and claims data points on prevalence, no non-literature-based data were included. We included hospital data corrected for readmission, primary to any diagnosis, and inpatient to outpatient utilisation ratios using adjustment factors calculated from USA claims data. We excluded hospital data in certain geographies (eg, the Philippines, China, India, Tibet, Kenya, Chile, Ecuador, Mexico, Botswana, Nepal, Brazil, and Japan) where the data were implausibly low. We also excluded all outpatient administrative data as the values for all locations were implausibly low, for example sources where the prevalence was consistently zero across all ages/sexes.

The reference case definition for atrial fibrillation was diagnosis based on an ECG reading. We adjusted datapoints which used an alternate definition, specifically claims and inpatient hospital data, using MR-BRT crosswalking standard GBD procedures; more details can be found in the non-fatal appendix crosswalking section. Table 2 shows the adjustment factors produced by the crosswalking procedure. The coefficients in Table 2 below were used to calculate adjustment factors for alternative definitions. The formula for computing adjustment factors is given in equation 1 below. We also included a cubic spline on a standardised age variable (age-scaled) and a categorical sex variable to the crosswalking procedure to adjust for the possibly of bias. The cubic spline can be seen in figure 2 below.

We also split prevalence datapoints where the age range was greater than 25 years. Age splitting was based on the global sex-specific age pattern from a DisMod model that included only prevalence datapoints where the age range was less than 25 years. This was done to allow sources that had wider age ranges to be included in the analysis. These wide-age data sources previously caused issues in fitting known increasing prevalence for atrial fibrillation and flutter with age.

Equation 1: Calculation of adjustment factors:

$$Estimated\ Reference\ Def = invlogit(logit(Alternative\ Def) - [\sum_{s=0}^{b} Beta_{Alternative\ Def, spline\ basis_s} * Spline\ basis_s(age\_scaled)] - Beta * I(Sex))$$

$I(.)$ = Indicator function, b = Number of spline bases used

Age splines for adjustment factors:

We fit a cubic spline to the standardised age variable (named age_scaled, calculated as $age\ scaled = \frac{(mean\ age\ of\ study - mean(age\ of\ all\ studies))}{standard\ deviation(age\ of\ all\ studies)}$). We selected knots for the cubic spline on age scaled based on visual inspection of the spline fit to observed ratios used in computing adjustment factors. Figure 2 below shows the fit of this spline on the standardised age variable for males and females with the observed logit difference between alternative and reference definitions on the vertical axis.

Cause – covariate	Knot placement (age_scaled)	Knot placement (age in years)
Atrial fibrillation and flutter – age_scaled	–1.99, –0.65, 0.22, 0.51, 1.85	47.1, 63.4, 73.9, 77.4, 93.8

Table 2: MR-BRT crosswalk adjustment factors for atrial fibrillation and flutter

Data input	Reference or alternative case definition	Gamma	Beta coefficient, logit (95% CI)
Literature using ECG reading	Ref	0.09	-
Inpatient clinical informatics data, intercept	Alt		–0.55 (–0.70 to –0.40)
Inpatient clinical informatics data, spline_0	Alt		0.03 (–0.07 to 0.12)
Inpatient clinical informatics data, spline_1	Alt		0.53 (0.46 to 0.60)
Inpatient clinical informatics data, spline_2	Alt		–0.35 (–0.38 to –0.32)

Inpatient clinical informatics data, spline_3	Alt		0.32<br>(0.27 to 0.37)
Inpatient clinical informatics data, spline_4	Alt		–0.92<br>(–0.98 to –0.86)
Inpatient clinical informatics data, spline_5	Alt		0.61<br>(0.56 to 0.66)
Inpatient clinical informatics data, male	Alt		–0.30<br>(–0.31 to –0.29)

Figure 2: Age-scaled spline for adjustment of inpatient clinical informatics prevalence data

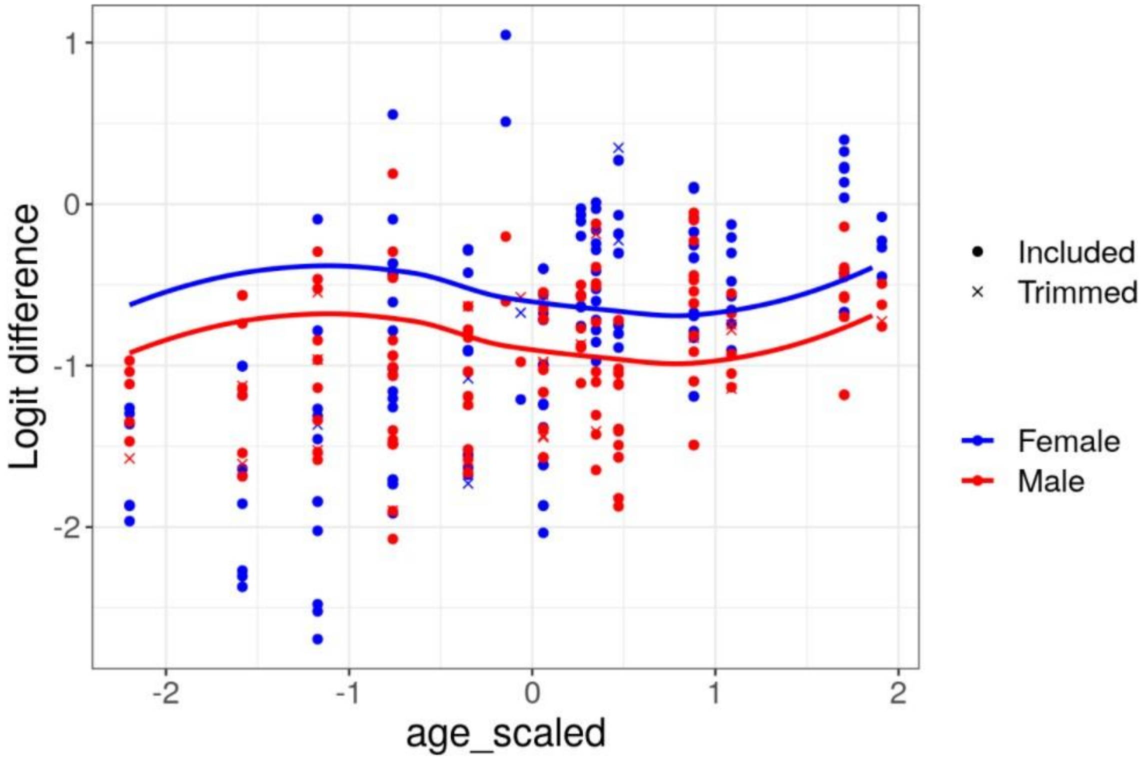

Modelling strategy

In order to address changes in coding practices for atrial fibrillation that resulted in an implausible trend of increasing death-certificate-based mortality rates, we used a prevalence-based modelling approach that combined DisMod-MR and CODEm models to generate estimates for atrial fibrillation and flutter. This approach, first used in GBD 2015, allowed us to generate more accurate estimates using observed prevalence and incidence rates along with modelled excess mortality rates generated from prevalence and cause-specific mortality estimates.

The modelling steps are illustrated in the above flowchart. Effect sizes for covariates included in both the DisMod-MR 2.1 and CODEm models can be found in the tables below.
In Step 1, we estimated deaths for atrial fibrillation using a standard CODEm approach. We used vital registration data (VR) for the CODEm models. We outliered ICD-8 and ICD-9 vital registration data that were discontinuous from other data in the time series and created an unlikely time trend. We also outliered datapoints that were implausibly low in multiple age groups. More details on this modelling

strategy and the list of outliered locations can be seen in the appendix section regarding cause of death modelling of atrial fibrillation.

In Step 2, we estimated prevalence rates in DisMod-MR using data from published reports of cross-sectional and cohort surveys, as well as primary care facility data. We also used claims data covering inpatient and outpatient visits for the USA along with inpatient hospital data from 196 locations in 24 countries. For GBD 2021, inpatient hospital data were adjusted using age- and sex-specific information for: 1) readmission within one year; 2) primary diagnosis code to secondary codes; and 3) the ratio of inpatient to outpatient visits. These clinical informatics data were then further adjusted using MR-BRT to account for misclassification compared with reference data that used ECG to identify atrial fibrillation and flutter. We set priors of no remission and capped excess mortality at 0.4 for all ages. We included the Healthcare Access and Quality (HAQ) Index as a country-level, fixed-effect covariate on excess mortality and the log-transformed, age-standardised summary exposure value (SEV) scalar for atrial fibrillation and flutter as a country-level, fixed-effect covariate on prevalence.

In Step 3, we calculated the excess mortality rate (EMR) for the year 2019 (defined as the cause-specific mortality rate [CSMR] estimated from CODEm divided by the prevalence rate from DisMod-MR). We then selected 21 countries based on four conditions: 1) ranking of 4 or 5 stars in the system for assessing the quality of VR data; 2) prevalence data available from the literature were included in the DisMod-MR estimation; 3) prevalence rate ≥0.005; and 4) CSMR ≥0.00002. For GBD 2021, we updated these locations to account for the addition of new prevalence data. This gave us data from six additional locations to use in the regression including New Zealand, Ireland, Israel, Norway, Portugal, and Brazil. Using information from these countries as input data, we ran a MR-BRT model of logEMR on sex, a cubic spline of age, and HAQ Index. Specifics on the MR-BRT framework can be found elsewhere in the appendix. We then predicted year-, age-, and sex-specific EMR using the results of this regression for all non-selected countries. Countries included in the regression were assigned their directly calculated values. These EMR datapoints were assigned to the time period 1990–2019 and uploaded into the non-fatal database in order to be used in modelling. Figures 3 illustrates the resulting estimated excess mortality rates described, and how estimates vary by levels of HAQ Index. Note that as access to quality health care decreases, the predicted excess mortality rate increases.

*Figure 3: Modleled excess mortality rate relationship with age, sex, and Healthcare Access and Quality Index. *HAQ Index divided into tertiles.*

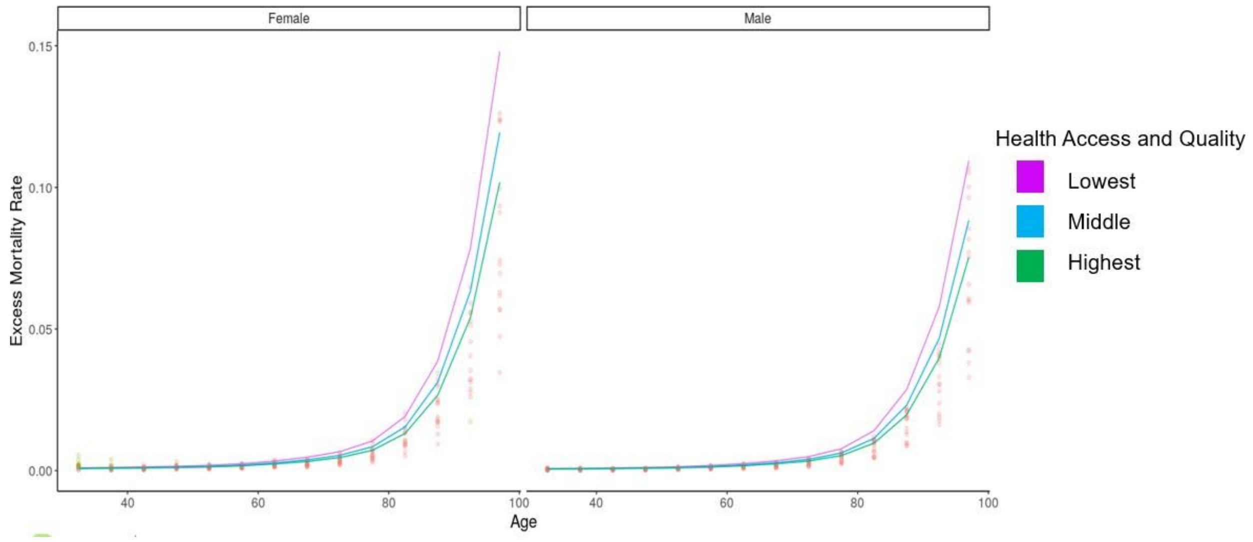

In Step 4, we reran DisMod-MR using the input data described in Step 2 along with the EMR estimated in Step 3. We included HAQ Index as a fixed-effect, country-level covariate on excess mortality and the log-transformed, age-standardised SEV scalar for atrial fibrillation and flutter as a fixed-effect, country-level covariate on prevalence. We included a value prior of 0 for remission for all ages and set a value prior of 0 for excess mortality and incidence for ages 0–30.

The prevalence from the DisMod-MR model in Step 4 was used as the finalised output for upload to the comorbidity adjustment and further processing into YLDs and DALYs.

The tables below include the study covariates, parameters, betas, and exponentiated betas.

Table 4a. Covariates. Summary of covariates used in the atrial fibrillation and flutter step 2 DisMod-MR meta-regression model

**Covariate**	**Parameter**	**Beta**	**Exponentiated beta**
Log-transformed age-standardised SEV scalar: A fib	Prevalence	0.75 (0.75 to 0.75)	2.12 (2.12 to 2.12)
Healthcare Access and Quality Index	Excess mortality rate	–0.16 (–0.18 to –0.14)	0.85 (0.84 to 0.87)

Table 4b. Covariates. Summary of covariates used in the atrial fibrillation and flutter step 4 DisMod-MR meta-regression model

Covariate	Parameter	Beta	Exponentiated beta
Log-transformed age-standardised SEV scalar: A fib	Prevalence	0.75 (0.75 to 0.76)	2.12 (2.12 to 2.14)
Healthcare Access and Quality Index	Excess mortality rate	–0.015 (–0.015 to –0.015)	0.98 (0.98 to 0.99)

Models were evaluated based on expert opinion, comparison with results from previous rounds of GBD, and model fit. No substantive changes were made to the modelling strategy for GBD 2021.

Severity splits & disability weights

Atrial fibrillation is split into symptomatic and asymptomatic based on standard GBD proportion information. For GBD 2021, we included heart failure due to atrial fibrillation and flutter sequela into the severity distribution. For details on the heart failure estimation process, see the heart failure section in the appendix. The table below includes lay descriptions and disability weights for the severity levels of atrial fibrillation:

Table 3. Severity distribution, details on the severity levels for atrial fibrillation and flutter in GBD 2021 and the associated disability weight (DW) with that severity

Severity level	Lay description	DW (95% CI)
Asymptomatic atrial fibrillation	No symptoms	N/A
Symptomatic atrial fibrillation, without heart failure	Has periods of rapid and irregular heartbeats and occasional fainting.	0.224 (0.151–0.312)
Symptomatic atrial fibrillation, with asymptomatic heart failure	Has periods of rapid and irregular heartbeats and occasional fainting, has been diagnosed with clinical heart failure, a chronic disease that requires medication every day and causes some worry but minimal interference with daily activities.	0.012 (0.006–0.023)
Symptomatic atrial fibrillation, with mild heart failure	Has periods of rapid and irregular heartbeats and occasional fainting, is short of breath and easily tires with moderate physical activity, such as walking uphill or more than a quarter-mile on level ground. The person feels comfortable at rest or during activities requiring less effort.	0.042 (0.026–0.062)
Symptomatic atrial fibrillation, with moderate heart failure	Has periods of rapid and irregular heartbeats and occasional fainting, is short of breath and easily tires with minimal physical activity, such as walking only a short distance. The person feels comfortable at rest but avoids moderate activity.	0.072 (0.047–0.103)
Symptomatic atrial fibrillation, with severe heart failure	Has periods of rapid and irregular heartbeats and occasional fainting, Is short of breath and feels tired when at rest. The person avoids any physical activity, for fear of worsening the breathing problems.	0.179 (0.122–0.312)

Attention-deficit/hyperactivity disorder

Flowchart

Attention-deficit/hyperactivity disorder

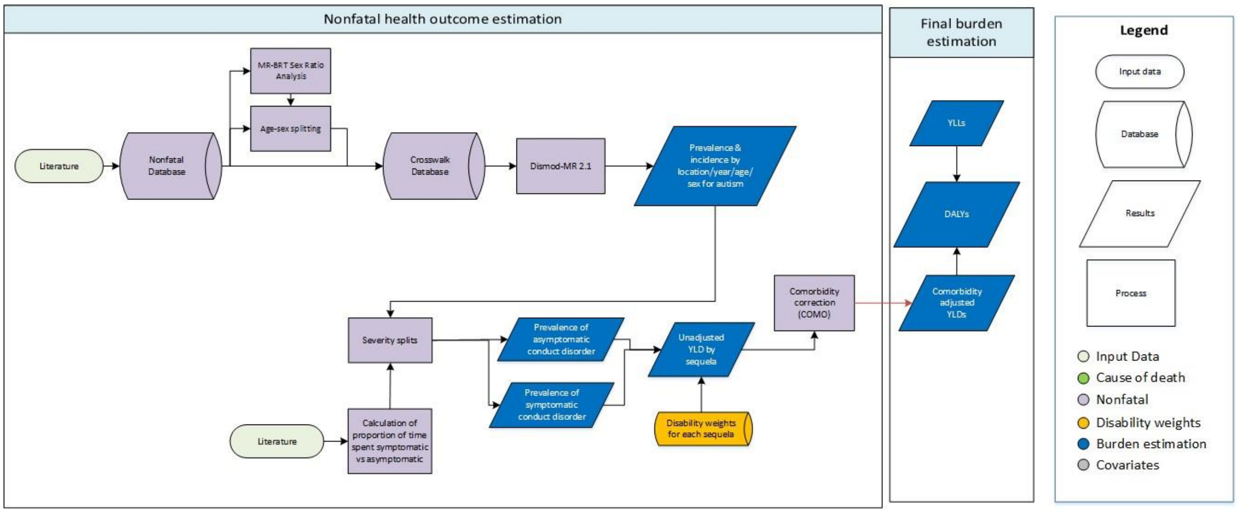

Input data and methodological summary for attention-deficit/hyperactivity disorder

Case definition

Attention-deficit/hyperactivity disorder (ADHD) is an externalising disorder characterised by persistent inattention and/or hyperactivity-impulsivity. As per criteria set by the Diagnostic and Statistical Manual of Mental Disorders Fourth Edition, Text Revision (DSM-IV-TR),[1] diagnosis requires six or more symptoms of inattention or hyperactivity-impulsivity to have persisted for at least six months in two or more settings causing significant impairment to functioning, with at least some impairing symptoms being present prior to 7 years of age (12 years of age in DSM-5).[2] Recognised symptoms include:

Inattention:

- often fails to give close attention to details or makes careless mistakes in schoolwork, work, or other activities
- often has difficulty sustaining attention in tasks or play activities
- often does not seem to listen when spoken to directly
- often does not follow through on instructions and fails to finish schoolwork, chores, or duties in the workplace (not due to oppositional behaviour or failure to understand instructions)
- often has difficulty organising tasks and activities
- often avoids, dislikes, or is reluctant to engage in tasks that require sustained mental effort (such as schoolwork or homework)
- often loses things necessary for tasks or activities (eg, toys, school assignments, pencils, books, or tools)
- is often easily distracted by extraneous stimuli
- is often forgetful in daily activities

Hyperactivity:

- often fidgets with hands or feet or squirms in seat
- often leaves seat in classroom or in other situations in which remaining seated is expected
- often runs about or climbs excessively in situations in which it is inappropriate (in adolescents or adults, may be limited to subjective feelings of restlessness)
- often has difficulty playing or engaging in leisure activities quietly

- is often “on the go” or often acts as if “driven by a motor”

- often talks excessively

Impulsivity:

- often blurts out answers before questions have been completed
- often has difficulty awaiting turn
- often interrupts or intrudes on others (eg, butts into conversations or games)

Included in the GBD study were cases meeting diagnostic criteria according to DSM[1] or the International Classification of Diseases (ICD)[3] (called "hyperkinetic disorder" in ICD). These were identified by the following codes: 314.0, 314.01 (DSM-IV-TR) and F90 (ICD-10). Different versions of DSM (DSM-III, DSM-III-R, DSM-IV, DSM-IV-TR, DSM-5, and DSM-5-TR) and ICD (ICD-9, ICD-10, and ICD-11) were accepted.

Input data

The epidemiological systematic literature review for ADHD was conducted in three stages involving electronic searches of the peer-reviewed literature (ie, via PsycInfo, Embase, and PubMed), the grey literature, and expert consultation. For mental disorders, we update our GBD electronic database searches on a rolling basis. An electronic search was not required for GBD 2021. The next update will be conducted in the next round of GBD. The grey literature searches and expert consultation were conducted for GBD 2021.

The GBD inclusion criteria stipulated that: 1) the publication year must be from 1980 onward; 2) "caseness" must be based on clinical threshold as established by the DSM or ICD; 3) sufficient information must be provided on study method and sample characteristics to assess the quality of the study; 4) study sample must be representative of the general population (ie, inpatient or pharmacological treatment samples, case studies, veterans, or refugee samples were excluded). No limitation was set on the language of publication. Methods used in previous systematic reviews have been reported in greater detail elsewhere.[4] Table 1 summarises data inputs by parameter for ADHD.

Table 1: Data Inputs for ADHD morbidity modelling by parameter

Parameter	Countries with data	New sources	Total sources
Incidence	1	0	2
Prevalence	49	0	172
Remission	6	0	14
Other	2	0	3

Age-sex splitting

The extracted data underwent two types of age-sex splitting processes:

1. Where possible, estimates were further split by sex and age based on the available data. For instance, if studies reported prevalence for broad age groups by sex (eg, prevalence in 15–65-year-old males and females separately), and also by specific age groups but for both sexes combined (eg, prevalence in 15–30-year-olds, then in 31–65-year-olds, for males and females combined); age-specific estimates were split by sex using the reported sex-ratio and bounds of uncertainty.
2. A meta-regression—Bayesian, regularised, trimmed (MR-BRT) analysis was used to split the remaining both-sex estimates in the dataset. For each parameter, sex-specific estimates were matched by location, age, and year. A MR-BRT network meta-analysis was then used to estimate pooled sex ratios and bounds of uncertainty. These were then used to split the both-sex estimates

in the dataset. The male-to-female prevalence ratio was estimated as 2.38 (95% uncertainty interval [UI]: 1.24–3.51).

Bias corrections/crosswalks

No crosswalks were applied to the estimates for ADHD.

Modelling strategy

We have made no substantive changes in the modelling strategy from GBD 2019.

After the above data processes were applied, DisMod-MR 2.1 was used to model the epidemiological data for ADHD. Adjustments to model priors or the dataset were made where appropriate. Where outliers were identified in the data, we re-assessed the study's methodology and quality before a decision was made to exclude or include the data.

Data across all epidemiological parameters were initially included in the modelling process. We assumed no incidence prior to 3 years of age or onward from 12 years of age. The minimum age of onset was set in consultation with experts and based on current literature, while the upper age limit on incidence was set in line with the latest DSM-5 criteria. Remission was set to zero prior to 12 years, in line with the restriction on incidence. Excess mortality was set to zero given only three estimates were found for this parameter and there was insufficient data to suggest an elevated risk of mortality in those with ADHD.

Severity splits and disability weight

The GBD disability weight survey assessments include lay descriptions of sequelae highlighting major functional consequences and symptoms. The lay description and disability weight for ADHD is shown in Table 2. A severity split for the proportion of time spent symptomatic versus asymptomatic was based on data from the Great Smoky Mountains Study which assessed the levels of disability found in children and adolescents with mental disorders.[5] Of those with ADHD, 48% reported disability, while 20% of individuals with no diagnosis reported disability at the time of survey. Using these as estimates of the proportion of time with disability in the "average case," the proportion of disability in children without a diagnosis was subtracted from the proportion with disability for ADHD, giving an adjusted proportion of 28%. Detailed descriptions of this methodology have been published elsewhere.[6]

Table 2. Lay description for ADHD in GBD 2021 and the associated disability weight

**Lay description**	**Disability weight (95% UI)**
Is hyperactive and has difficulty concentrating, remembering things, and completing tasks.	0.045 (0.028–0.066)

There were no significant changes in GBD 2021 results for ADHD compared to GBD 2019. While we continue to improve on the data and methods used in GBD, some challenges need to be acknowledged. Firstly, we still have a large number of locations with no high quality raw data available. Secondly, it is difficult to quantify and remove all variations due to measurement error in our prevalence estimates. While we have improved the methodology used to account for known sources of bias (eg, survey methods or case definitions), we still have very few datapoints to inform such adjustments. Thirdly, there is a paucity of research on the risk factors of mental disorders which can be used as predictive covariates in our epidemiological models.

References

1. American Psychiatric Association. Diagnostic and Statistical Manual of Mental Disorders (DSM-IV-TR). 4th, Text Revision ed. Washington DC: American Psychiatric Association; 2000.
2. American Psychiatric Association. Diagnostic and Statistical Manual of Mental Disorders, Fifth Edition. Arlington, VA: American Psychiatric Association; 2013.
3. World Health Organization. ICD-10 Classification of Mental and Behavioural Disorders: Clinical descriptions and diagnostic guidelines. Geneva: World Health Organization; 1992.
4. Erskine HE, Ferrari AJ, Nelson P, et al. Research Review: Epidemiological modelling of attention-deficit/hyperactivity disorder and conduct disorder for the Global Burden of Disease Study 2010. *Journal of Child Psychology and Psychiatry* 2013; **54**(12): 1263-74.
5. Ezpeleta L, Keeler G, Erkanli A, Costello EJ, Angold A. Epidemiology of Psychiatric Disability in Childhood and Adolescence. *J Child Psychol Psychiatry* 2001; **42**(7): 901-14.
6. Erskine HE, Ferrari AJ, Polanczyk GV, et al. The global burden of conduct disorder and attention-deficit/hyperactivity disorder in 2010. *J Child Psychol Psychiatry* 2014; **55**(4): 328-36.

Autism spectrum disorders

Flowchart

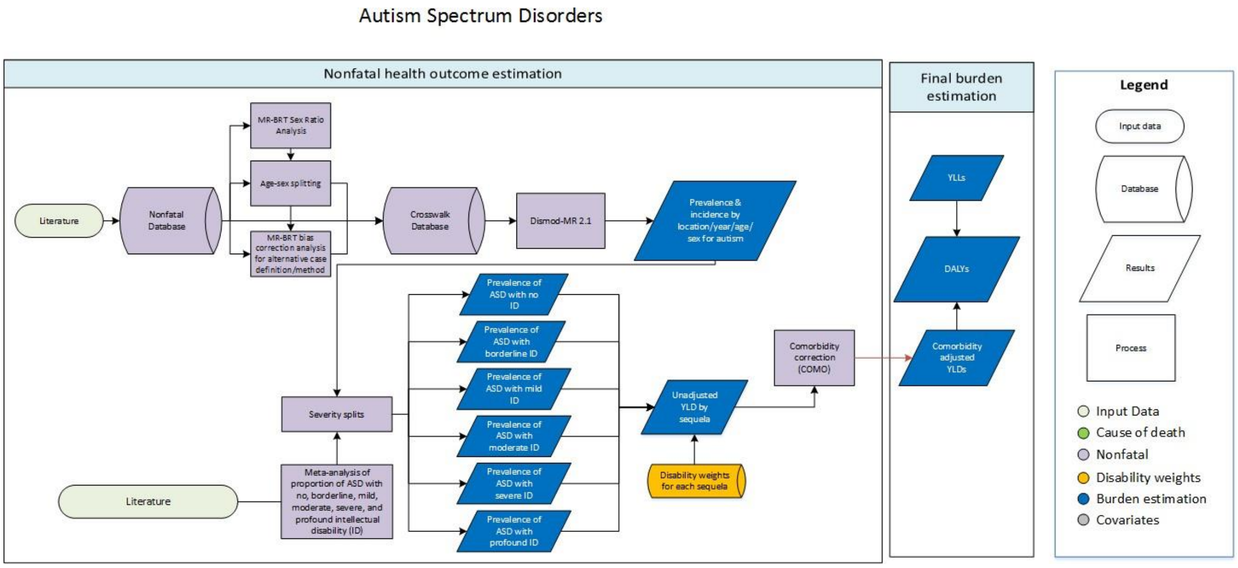

Input data and methodological summary for autism spectrum disorders

Case definition

Autism spectrum disorders (ASD) – also known as pervasive developmental disorders – are a group of neurodevelopmental disorders with onset occurring in early childhood. ASD is characterised by pervasive impairment in several areas of development, including social interaction and communication skills, along with restricted and repetitive patterns of behaviours and/or interests.

ASD was an umbrella for five sub-disorders according to the Diagnostic and Statistical Manual of Mental Disorders fourth edition, text revision (DSM-IV-TR):[1] autistic disorder (299.00), pervasive developmental disorder, pervasive developmental disorder not otherwise specified (299.80), Rett's disorder (299.8), Asperger's disorder (299.8), and childhood disintegrative disorder (299.10). ASD is still an umbrella for

eight sub-disorders according to the International Statistical Classification of Diseases and Related

Health Problems 10th Revision (ICD-10):[2] childhood autism (F84.0), atypical autism (F84.1), Rett's syndrome (F84.2), other childhood disintegrative disorder (F84.3), overactive disorder associated with mental retardation and stereotyped movements (F84.4), Asperger syndrome (F84.5), other pervasive developmental disorders (F84.8), and pervasive disorder unspecified (F84.9). However, it has been amalgamated into a single disorder in the Diagnostic and Statistical Manual for Mental Disorders 5th edition (DSM-5).[3] A diagnosis of ASD according to the DSM-5 requires the following criteria to be met:

Persistent deficits in social communication and social interaction across multiple contexts, as manifested by all of the following, currently or by history:

1. *Deficits in social-emotional reciprocity, ranging, for example, from abnormal social approach and failure of normal back-and-forth conversation; to reduced sharing of interests, emotions, or affect; to failure to initiate or respond to social interactions.*
2. *Deficits in nonverbal communicative behaviours used for social interaction, ranging, for example, from poorly integrated verbal and nonverbal communication; to abnormalities in eye contact and body language or deficits in understanding and use of gestures; to a total lack of facial expressions and nonverbal communication.*
3. *Deficits in developing, maintaining, and understanding relationships, ranging, for example, from difficulties adjusting behaviour to suit various social contexts; to difficulties in sharing imaginative play or in making friends; to absence of interest in peers.*

Restricted, repetitive patterns of behaviour, interests, or activities, as manifested by at least two of the following, currently or by history:

1. *Stereotyped or repetitive motor movements, use of objects, or speech (eg, simple motor stereotypies, lining up toys or flipping objects, echolalia, idiosyncratic phrases).*
2. *Insistence on sameness, inflexible adherence to routines, or ritualised patterns of verbal or nonverbal behaviour (eg, extreme distress at small changes, difficulties with transitions, rigid thinking patterns, greeting rituals, need to take same route or eat same food every day).*
3. *Highly restricted, fixated interests that are abnormal in intensity or focus (eg, strong attachment to or preoccupation with unusual objects, excessively circumscribed or perseverative interests).*
4. *Hyper- or hyporeactivity to sensory input or unusual interest in sensory aspects of the environment (eg, apparent indifference to pain/temperature, adverse response to specific sounds or textures, excessive smelling or touching of objects, visual fascination with lights or movement).*

The symptoms must be present in the early developmental period, cause clinically significant impairment, and not be better explained by intellectual impairment or global developmental delay.

Different versions of DSM (DSM-III, DSM-III-R, DSM-IV, DSM-IV-TR, DSM-5, and DSM-5-TR) and ICD (ICD-9, ICD-10, and ICD-11) were accepted.

Input data

The epidemiological systematic literature review for ASD was first conducted for GBD 2017 and updated in April 2021 for GBD 2021. It was conducted in three stages involving electronic searches of the peer-reviewed literature (ie, via PsycInfo, Embase, and PubMed), the grey literature, and expert consultation. For mental disorders, we update our GBD electronic database searches on a rolling basis.

The GBD inclusion criteria stipulated that: 1) the diagnostic criteria must be from 1980 onward; 2) "caseness" must be based on clinical threshold as established by the DSM, ICD, Chinese Classification of Mental Disorders (CCMD), or diagnosed by a clinician using established tools; 3) sufficient information must be provided on study method and sample characteristics to assess the quality of the study; and 4) study samples must be representative of the general population (ie, case studies, veterans, or refugee samples were excluded). No limitation was set on the language of publication.

To improve data coverage for ASD, estimates of the prevalence of the DSM-IV-TR sub-disorder autistic disorder (299.00), ICD-10 childhood autism (F84.0), and their DSM-III, DSM-III-R, DSM-IV, ICD-9, and CCMD equivalents were also included with an adjustment so that they reflected what these estimates would be if the data represented ASD. Administrative prevalence estimates were included with bias corrections in the epidemiological modelling of ASD in GBD 2019. However, there was not enough data to explore the interaction between administrative estimates and time or health-care access quality. This potentially inflated prevalence in locations with good health-care access quality where the majority of ASD cases are diagnosed, and underestimated prevalence in locations where health-care access quality is poor and the majority of ASD cases are missed. Administrative prevalence estimates were therefore excluded in GBD 2021.

Table 1 summarises data inputs by parameter for ASD.

Table 1: Data inputs for ASD morbidity modelling by parameter

**Parameter**	**Countries with data**	**New sources**	**Total sources**
Incidence	0	0	0
Prevalence	33	24	105
Remission	0	0	0
Excess mortality	6	5	6

Age-sex splitting

The extracted data underwent two types of age-sex splitting processes:

3. Where possible, estimates were further split by sex and age based on the available data. For instance, if studies reported prevalence for broad age groups by sex (eg, prevalence in 15–65-year-old males and females separately), and also by specific age groups but for both sexes combined (eg, prevalence in 15–30-year-olds, then in 31–65-year-olds, for males and females combined); age-specific estimates were split by sex using the reported sex-ratio and bounds of uncertainty.
4. A meta-regression—Bayesian, regularised, trimmed (MR-BRT) analysis was used to split the remaining both-sex estimates in the dataset. For each parameter, sex-specific estimates were matched by location, age, and year, and a MR-BRT network meta-analysis was used to estimate pooled sex ratios and bounds of uncertainty. These were then used to split the both-sex estimates in the dataset. The male-to-female prevalence ratio was 2.59 (95% uncertainty interval [UI]: 2.45–2.75). The male-to-female excess mortality rate ratio was 0.73 (0.62–0.86).

Bias corrections/crosswalks

Estimates with known biases were adjusted/crosswalked accordingly prior to DisMod-MR 2.1. Within the ASD epidemiological dataset, within-study estimates were paired by age, sex, location, and year, between the reference and alternative estimates. Pairs were also made between the different

alternative estimates where applicable. The ratios between these estimates were then used as inputs in a MR-BRT network meta-analysis. This analysis produced pooled ratios between the reference estimates and alternative estimates. These ratios (see Table 2) were used to adjust all alternative estimates in the dataset. The estimated UIs around the adjustment ratio incorporate Gamma which represents the between-study variance across all input data in the model. This added uncertainty widens the UIs for crosswalks with significant fixed effects. ASD had two alternative definitions to crosswalk:

1. Estimates of autism (rather than of ASD).
2. General population survey without additional case-finding. These are studies that conduct household or school surveys but do not conduct additional active case-finding (such as reviewing special education records) to find cases likely to be missed by survey methodology.

Table 2: MR-BRT crosswalk adjustment factors for ASD

Data input	Reference or alternative case definition	Gamma	Beta coefficient, log (95% UI)*	Adjustment factor**
Population survey	Reference: estimate represents ASD from general population surveys, with additional case-finding or total population screening	0.01		
Population survey	Alternative: estimate represents autism (rather than ASD)		-0.81 (-0.93 – -0.69)	0.44 (0.39–0.50)
Population survey	Alternative: general population survey without additional case-finding		-0.35 (-0.50–0.20)	0.70 (0.61–0.82)

**MR-BRT crosswalk adjustments can be interpreted as the factor the alternative case definition is adjusted by to reflect what it would have been had it been measured using the reference case definition. If the log/logit beta coefficient is negative, then the alternative is adjusted up to the reference. If the log/logit beta coefficient is positive, then the alternative is adjusted down to the reference.*

***The adjustment factor column is the exponentiated beta coefficient. For log beta coefficients, this is the relative rate between the two case definitions. For logit beta coefficients, this is the relative odds between the two case definitions.*

Modelling strategy

After the above data processes were applied, DisMod-MR 2.1 was used to model the epidemiological data for ASD. Adjustments to model priors or the dataset were made where appropriate. Where outliers were identified in the data, we reassessed the study's methodology and quality before a decision was made to exclude or include the data. We assumed all incidence of ASD occurred at birth. Remission was set to 0 after expert consultation revealed we would not expect remission for ASD. A maximum excess mortality rate of 0.3 was set to align with the largest observed confidence interval for an excess mortality rate estimate for ASD. The Healthcare access quality index was also used as a country-level covariate on excess mortality with a +/- 0.03 prior on the coefficient (coefficient = -0.026 (-0.03 — -0.02).

Severity splits and disability weights

ASD is one of the causes that contribute to the intellectual disability (ID) envelope. As such, a gradation of ASD by level of intellectual disability was required. Meta-analyses were conducted using data from 19

studies that used reference sampling methodology and reported information on the IQ level of those with ASD in order to calculate the severity splits by six sequelae: ASD with 1) no ID, 2) borderline ID, 3) mild ID, 4) moderate ID, 5) severe ID, and 6) profound ID.

The disability weights for each sequela of ASD were calculated using the disability weights for the health states autism; Asperger's syndrome and other ASD; borderline ID; mild ID; moderate ID; severe ID; and profound ID. These disability weights and their lay descriptions are presented in Table 3.

Table 3: Severity distribution, details on the severity levels for ASD and the associated disability weight with that severity

**Health state**	**Lay description**	**Disability weight (95% UI)**
Autism	Has severe problems interacting with others and difficulty understanding simple questions or directions. The person has great difficulty with basic daily activities and becomes distressed by any change in routine.	0.262 (0.176–0.365)
Asperger's syndrome and other ASDs	Has difficulty interacting with other people and is slow to understand or respond to questions. The person is often preoccupied with one thing and has some difficulty with basic daily activities.	0.104 (0.071–0.147)
ID, borderline	Is slow in learning at school. As an adult, the person has some difficulty doing complex or unfamiliar tasks but otherwise functions independently.	0.011 (0.005–0.020)
ID, mild	Has low intelligence and is slow in learning at school. As an adult, the person can live independently, but often needs help to raise children and can only work at simple supervised jobs.	0.043 (0.026–0.064)
ID, moderate	Has low intelligence, and is slow in learning to speak and to do even simple tasks. As an adult, the person requires a lot of support to live independently and raise children. The person can only work at the simplest supervised jobs.	0.100 (0.066–0.142)
ID, severe	Has very low intelligence and cannot speak more than a few words, needs constant supervision and help with most daily activities, and can do only the simplest tasks.	0.160 (0.107–0.226)
ID, profound	Has very low intelligence, has almost no language, and does not understand even the most basic requests or instructions. The person requires constant supervision and help for all activities.	0.200 (0.133–0.283)

To estimate the disability weights for each sequela of ASD, the following steps were conducted, with

each step pulling 1000 draws of each input:

1. A pooled disability weight for ASD was estimated:
$$DW_{ASD} = DW_{Autism} \times P_{Autism} + DW_{Asperger} \times (1 - P_{Autism})$$
Where *DW* is disability weight and *P* is the proportion of ASD cases estimated to meet DSM-IV criteria for the autism sub-type (derived from a meta-analysis of 19 studies, 0.41 [0.36 – 0.47]).
2. The disability weight for ASD and each remaining level of ID was estimated:
$$DW_{ASD+ID} = 1 - (1 - DW_{ASD}) \times (1 - DW_{ID})$$

The severity proportions from the meta-analysis used in the above process and the resulting disability weights for each sequela are presented in Table 4.

Table 4: Severity proportions and disability weights of ASD sequelae

**Sequela**	**Severity proportion (95% UI)**	**Disability weight (95% UI)**
ASD without ID	0.446 (0.395–0.496)	0.169 (0.114–0.236)
ASD with borderline ID	0.197 (0.159–0.235)	0.178 (0.123–0.244)
ASD with mild ID	0.149 (0.110–0.191)	0.205 (0.149–0.273)
ASD with moderate ID	0.139 (0.101–0.182)	0.252 (0.192–0.318)
ASD with severe ID	0.056 (0.034–0.084)	0.302 (0.236–0.373)
ASD with profound ID	0.014 (0.006–0.026)	0.336 (0.261–0.418)

Changes between GBD 2019 and GBD 2021

There were two changes implemented to the GBD 2021 modelling strategy for ASD which increased its prevalence and burden compared to what was estimated for GBD 2019. In GBD 2019, estimates derived from administrative prevalence were included in the epidemiological modelling of ASD with a bias correction. However, this process was reviewed in GBD 2021 following feedback that the inclusion of these estimates still led to an underestimation of prevalence despite the bias correction. Following further discussion and feedback from expert collaborators, these data were excluded. This led to a significant increase in the estimated prevalence of ASD for GBD 2021.

An additional change was implemented to the method used to estimate the disability weights for each sequelae of ASD. The sequelae for ASD represent ASD across six levels of intellectual disability (none, borderline, mild, moderate, severe, and profound), and so the disability weights for ASD are a combination of the disability weight of ASD without intellectual disability and the disability weight for the respective level of intellectual disability. In GBD 2019, we assumed the disability weight for ASD (disability weights for *autism* and *Asperger's syndrome and other ASDs* weighted for their respective proportions, 0.169 [0.114 – 236]) also included the disability of intellectual disability. This meant the disability of intellectual disability had to be quantified and removed from the disability weight for ASD prior to the estimation of sequela-specific disability weights (0.143 [0.081 – 0.225] in GBD 2019). However, a revisit of the lay descriptions for the disability weights (Table 3) illustrated minimal overlap in the descriptions between the ASD lay descriptions and the intellectual disability lay descriptions. We therefore opted not to apply the above correction for GBD 2021, and instead treat the disability weight for ASD as mutually exclusive to that of intellectual disability and not containing any overlapping disability.

While we continue to improve on the data and methods used to estimate the burden of mental disorders, other challenges need to be acknowledged. Firstly, we still have a large number of locations

with no high quality raw data available. Secondly, it is difficult to quantify and remove all variation due to measurement error in our epidemiological estimates. While we have attempted to account for known sources of bias, in some cases, we still have very few datapoints to inform these adjustments and to explore other interactions/bias adjustments. Thirdly, there is a paucity of research on the risk factors of mental disorders which can be used as predictive covariates in our epidemiological models.

References

1. American Psychiatric Association. Diagnostic and statistical manual of mental disorders : DSM-IV-TR. 4th ed., text revision. ed. Washington, DC: American Psychiatric Association; 2000.
2. World Health Organization. The ICD-10 classification of mental and behavioural disorders. Clinical descriptions and diagnostic guidelines. Geneva: World Health Organization; 1992.
3. American Psychiatric Association. Diagnostic and statistical manual of mental disorders : DSM-5. 4th , Text Revision ed. ed. Washington, DC: American Psychiatric Association Publishing; 2013.

Benign prostatic hyperplasia

Flowchart

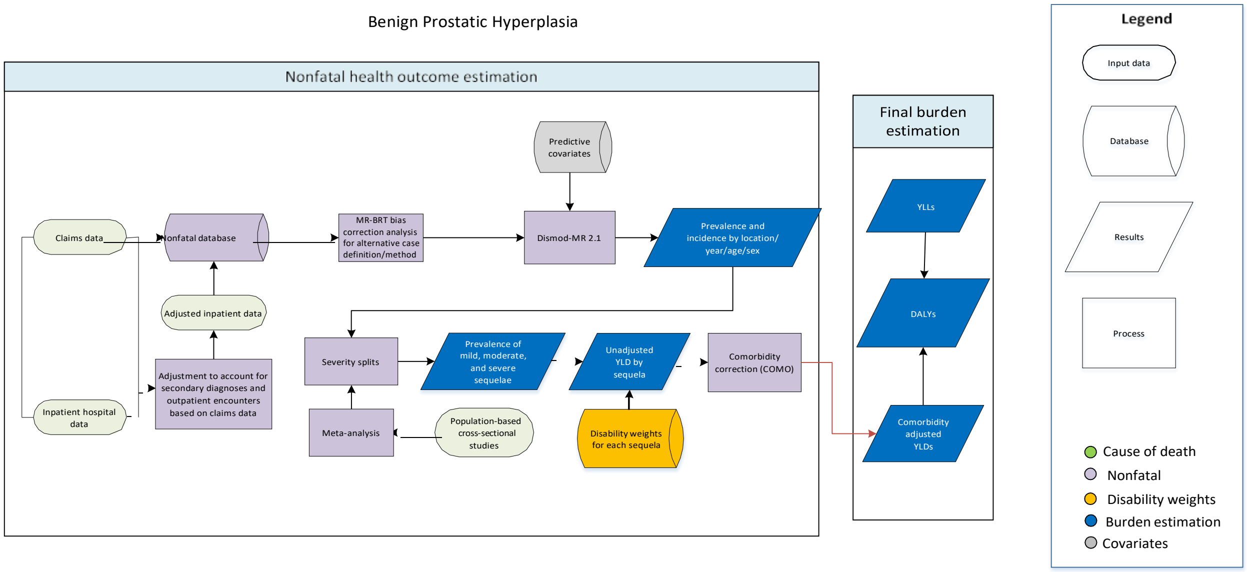

Input data and methodological summary for benign prostatic hyperplasia

Case definition

Benign prostatic hyperplasia (BPH) is defined as a chronic, non-cancerous proliferation of prostatic tissue regardless of symptoms. The ICD codes for BPH include N40, N40.0, N40.1, N40.2, N40.3, and N40.9.

Input data and data processing

Input data

Like GBD 2019, the model included prevalence data from hospital discharges and claims. In GBD 2021, we newly added additional years of data from USA claims (year 2017) and Poland claims (year 2018), as well as hospital discharges in Greece, Armenia, Chile, Ecuador, Argentina, Italy, Brazil, and Spain.

Table 1. Data Inputs for benign prostatic hyperplasia morbidity modelling by parameter

	Countries with data	New sources	Total sources
Prevalence	50	34	329

Data processing

Hospital discharge data provide observations about encounters, generally with only the primary diagnostic code for the encounter. Claims data, on the other hand, link claims for all inpatient and outpatient encounters for a single individual and provide primary and secondary diagnoses for all encounters.

An individual was extracted from claims data as a prevalent case if that individual had at least one inpatient or two outpatient encounters with an appropriate ICD code as any diagnosis within one year. Hospital discharge data were processed by extracting discharges with an appropriate ICD code as primary diagnosis and adjusting using correction factors (ie, correction factor 3) derived from claims data. Specifically, we modelled from the ratio of inpatient claims with BPH as primary diagnosis to total prevalent cases of BPH seen in claims data.

As first done in GBD 2019, USA claims data (extracted and processed as described above) were adjusted to account for selection bias due to commercial insurance, using MR-BRT (meta-regression—Bayesian, regularised, trimmed) analysis. The process of adjusting for biases in non-reference data using MR-BRT with the logit-transformation method is described below:

1. Identify datapoints with overlapping year, age, sex, and location between commercial claims (non-reference data) and population-representative hospital discharges (reference data).
2. Logit-transform overlapping datapoints of alternative and reference types.
3. Convert overlapping datapoints into a difference in logit space using the following equation: $logit(alternative) - logit(reference)$.
4. Use the delta method to compute standard errors of overlapping datapoints in logit space, then calculate standard error of logit difference using the following equation: $\sqrt{(variance\ of\ alternative) + (variance\ of\ reference)}$.
5. Using MR-BRT, conduct a random effects meta-regression to obtain the pooled logit difference of alternative to reference.
6. Apply the pooled logit difference to all datapoints of alternative case definitions using the following equation: $new_{estimate} = inverse.logit((logit(alternative)) - (pooled\ logit\ difference))$.
7. Calculate new standard errors using the delta method, accounting for gamma (between-study heterogeneity).

The table below shows bias correction factors estimated using MR-BRT.

Table 2. MR-BRT crosswalk adjustment factors for benign prostatic hyperplasia

Data input	Reference or alternative data collection	Gamma	Beta coefficient, logit (95% CI)	Adjustment factor*
Hospital + non-USA claims	Ref	0.000025	---	---
USA claims from year 2000	Alt		–0.87 (–0.94, –0.79)	0.29 (0.28, 0.31)

USA claims from year 2010–2017	Alt		–0.28 (–0.36, –0.21)	0.43 (0.41, 0.45)

**MR-BRT crosswalk adjustments can be interpreted as the factor the alternative case definition is adjusted by to reflect what it would have been had it been measured using the reference case definition. If the log/logit beta coefficient is negative, then the alternative is adjusted up to the reference. If the log/logit beta coefficient is positive, then the alternative is adjusted down to the reference.*

***The adjustment factor column is the exponentiated beta coefficient. For log beta coefficients, this is the relative rate between the two case definitions. For logit beta coefficients, this is the relative odds between the two case definitions.*

Datapoints with an age-standardised prevalence rate greater than two median absolute deviations from the median of the age-standardised prevalence rate for all inpatient and non-USA claims data were marked as outliers and excluded from analysis.

Modelling strategy

Similar to GBD 2019, we ran a DisMod-MR 2.1 model to produce estimates by age, sex, year, and country. Settings in the DisMod-MR model included a prior value of zero incidence and remission for ages less than 40 years. We set an upper bound on remission after age 40 years to 0.1, corresponding to a maximum disease duration of 10 years. We also assumed that there was no excess mortality related to BPH. The minimum coefficient of variation at the regional, super-regional, and global level was changed from 0.4 to 0.8 in GBD 2019 to improve model fit against input data, and this setting was carried forward in GBD 2021.

Similar to GBD 2019, we included the age-standardised prevalence of diabetes as a predictive covariate to inform prevalence, which was a better predictor than the mean BMI that was used in GBD 2017. The beta and exponentiated values of this covariate (which can be interpreted as an odds ratio) are shown in the table below.

Table 3. Covariates. Summary of covariates used in the benign prostatic hyperplasia DisMod-MR meta-regression model

**Covariate**	**Parameter**	**Exponentiated beta (95% uncertainty interval)**
Age-standardised prevalence of diabetes	Prevalence	11.30 (7.89–15.38)

Severity split & disability weight

The basis of the GBD disability weight survey assessments are lay descriptions of sequelae highlighting major functional consequences and symptoms of a given cause. BPH is split into symptomatic and asymptomatic types. There is no disability weight (DW) assigned to asymptomatic cases of BPH. The DW associated with symptomatic BPH, such as urinary frequency, that is sometimes associated with pain – as seen in the table below, which offers further information.

Table 4. Severity distribution, details on the severity levels for benign prostatic hyperplasia in GBD 2021 and the associated disability weight (DW) with that severity

Severity level	Lay description	DW (95% CI)
Asymptomatic	N/A	0

Symptomatic	Feels the urge to urinate frequently, but when passing urine, it comes out slowly and sometimes is painful.	0.067 (0.043–0.097)

In GBD 2021, we employed the severity distribution for BPH that was first developed and applied in GBD 2019. This severity distribution was derived from the International Prostate Symptom Score (I-PSS) reported in four population-based studies in Japan, USA, France, and Scotland.[1] I-PSS is a widely used validated questionnaire that is developed to assess severity of lower urinary tract symptoms (LUTS) related to BPH. The questionnaire consists of seven questions on incomplete emptying, frequency, intermittency, urgency, weak stream, straining, and nocturia, and one question on quality of life. Four studies recruited a representative sample of men between ages 40 and 79 in Japan, USA, and Scotland, and ages 50–84 in France. I-PSS was either self-administered in the presence of a research assistant or through face-to-face interviews. We modelled cumulative distribution of the I-PSS scores in the survey participants using MR-BRT to estimate the mean proportion of individuals with symptomatic LUTS. The severity distribution can be found in the table below.

Severity	Distribution
Asymptomatic BPH	0.673 (0.655–0.692)
Symptomatic BPH	0.327 (0.245–0.436)

References

Sagnier P-P, Girman CJ, Garraway M, Kumamoto Y, Lieber MM, Richard F, et al. International Comparison of the Community Prevalence of Symptoms of Prostatism in Four Countries. EUR. 1996;29:15–20.

Bipolar disorder

Flowchart

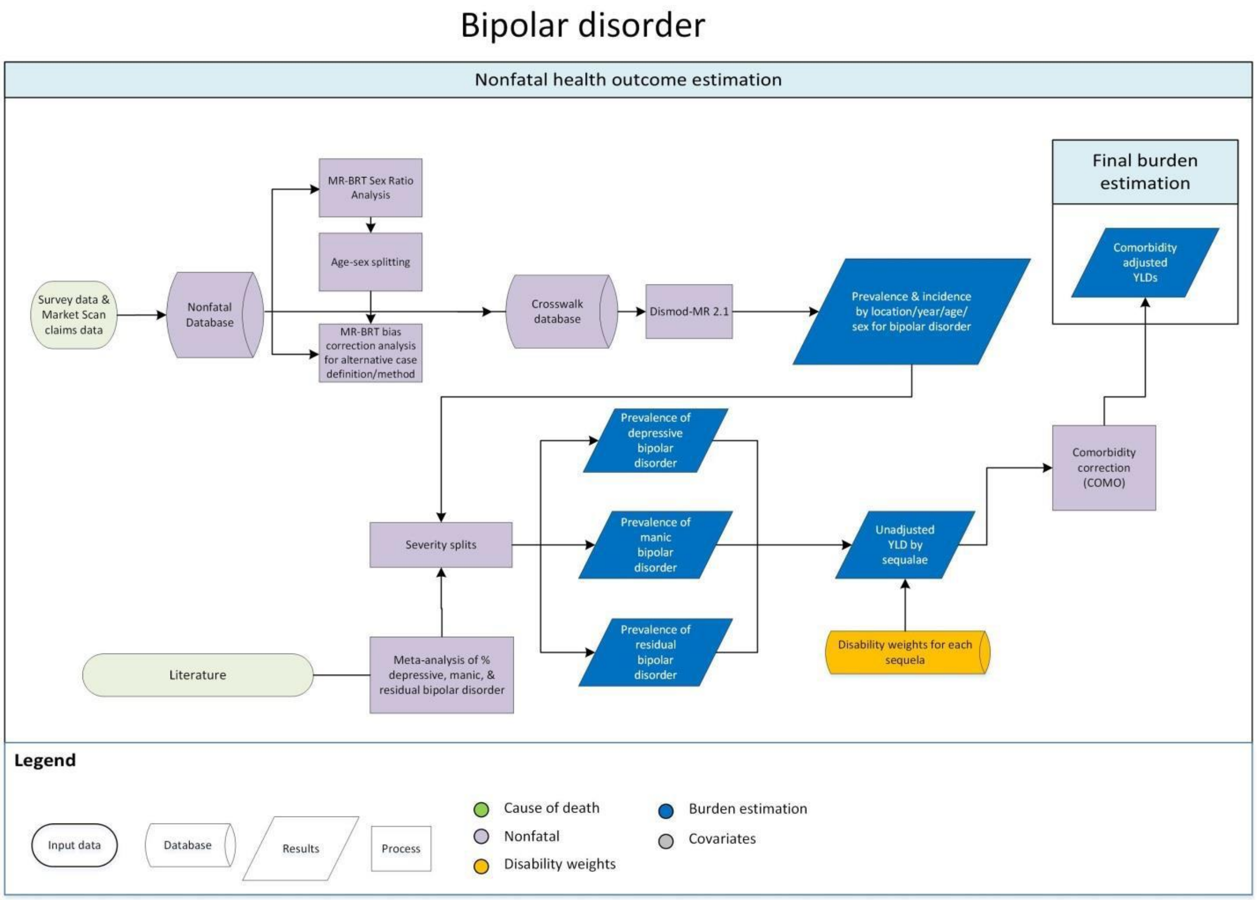

Input data and methodological summary for bipolar disorder

Case definition

Bipolar disorder is a serious mood disorder with little or no complete remission. Included in GBD disease modelling were cases meeting diagnostic criteria for bipolar disorder according to the Diagnostic and Statistical Manual of Mental Disorders (DSM), or the equivalent diagnosis in the International Classification of Diseases (ICD).[1,2] These are identified by the following codes: DSM-IV-TR: 296.0–296.7, 296.89, 301.13; ICD-10: F30.0-F30.9, F31.0–F31.6, F31.8–F31.9, F34.0. Excluded were bipolar disorder due to a general medical condition or substance-induced cases. Different versions of DSM (DSM-III, DSM-III-R, DSM-IV, DSM-IV-TR, DSM-5, and DSM-5-TR) and ICD (ICD-9, ICD-10, and ICD-11) were accepted.
A diagnosis of bipolar disorder involves the experience of one or more manic, hypomanic, and/or major depressive episodes.

According to DSM-IV-TR, a manic episode involves the experience of elevated, expansive, or irritable mood lasting for at least one week. During this period, at least three (or four if mood is only irritable) of the following symptoms must also be experienced: i) inflated self-esteem or grandiosity, ii) decreased need for sleep, iii) more talkative, iv) flight of ideas or experiences that thoughts are racing, v) distractibility, vi) increase in goal-directed activity, and vii) excessive involvement in pleasurable activities with high potential for painful consequences.

A hypomanic episode involves the experience of elevated, expansive, or irritable mood lasting for at least four days. During this period, at least three (or four if mood is only irritable) of the symptoms previously listed for a manic episode must also be experienced.

A major depressive episode involves the experience of depressed mood almost all day, every day, for at least two weeks. A total of five out of nine criteria must be met to make a diagnosis and at least one of the five criteria should either be “depressed mood” for most of every day or “loss of interest in nearly all activities” for most of every day. The other seven criteria are: i) change in eating, appetite, or weight, ii) excessive sleeping or insomnia, iii) agitated or slow motor activity, iv) fatigue, v) feeling worthless or inappropriately guilty, vi) trouble concentrating, and vii) repeated thoughts about death.

Different subtypes of bipolar disorder can be diagnosed depending on the combination of symptoms experienced. Bipolar I disorder is characterised by at least one manic episode, which can also alternate with a major depressive episode. Bipolar II disorder is characterised by depressive and hypomanic episodes. Cyclothymia is characterised by subsyndromal hypomanic episodes alternating with major depressive episodes. Bipolar disorder not otherwise specified is characterised by clinically significant symptoms of bipolar disorder which do not meet criteria for the other diagnoses.[2,3] In GBD 2021 we estimated burden for the entire spectrum of bipolar disorder simultaneously, rather than individually for each subtype of the disorder. At a minimum, epidemiological studies needed to report on bipolar I and bipolar II to be included in analyses.

Input data

The epidemiological systematic literature review for bipolar disorder was conducted in three stages involving electronic searches of the peer-reviewed literature (ie, via PsycInfo, Embase, and PubMed), the grey literature, and expert consultation. For mental disorders, we update our GBD electronic database searches on a rolling basis. An electronic search was not required for GBD 2021. The next update will be conducted in the next round of GBD. The grey literature searches and expert consultation were conducted for GBD 2021.

The GBD inclusion criteria stipulated that: 1) the publication year must be from 1980 onward; 2) “caseness” must be based on clinical threshold as established by the DSM or ICD; 3) sufficient information must be provided on study method and sample characteristics to assess the quality of the study; 4) study samples must be representative of the general population (ie, inpatient or pharmacological treatment samples, case studies, veterans, or refugee samples were excluded); and 5) as previously explained, we estimated the burden for the entire spectrum of bipolar disorder rather than individually for each subtype of the disorder. Combined estimates of all subtypes of bipolar disorder were required. Studies reporting separate estimates for bipolar I, bipolar II, cyclothymia, and/or bipolar not otherwise specified were accepted if sufficient information was available to sum the disorder-specific estimates. No limitation was set on the language of publication. Table 1 below summarises data inputs by parameter for bipolar disorder.

Table 1: Data inputs for bipolar disorder morbidity modelling by parameter

Parameter	Countries with data	New sources	Total sources
Incidence	2	0	3
Prevalence	41	3	116
Remission	0	0	0

Other	19	0	42

Age-sex splitting

The extracted data underwent three types of age-sex splitting processes:

5. Where possible, estimates were further split by sex and age based on the available data. For instance, if studies reported prevalence for broad age groups by sex (eg, prevalence in 15–65-year-old males and females separately), and also by specific age groups but for both sexes combined (eg, prevalence in 15–30-year-olds, then in 31–65-year-olds, for males and females combined); age-specific estimates were split by sex using the reported sex-ratio and bounds of uncertainty.
6. A meta-regression—Bayesian, regularised, trimmed (MR-BRT) analysis was used to split the remaining both-sex estimates in the dataset. For each parameter, sex-specific estimates were matched by location, age, and year, and a MR-BRT network meta-analysis was used to estimate pooled sex ratios and bounds of uncertainty. These were then used to split the both-sex estimates in the dataset. The male-to-female prevalence ratio estimated for prevalence estimates was 0.87 (95% uncertainty interval [UI]: 0.54–1.20).
7. Studies reporting prevalence estimates across age groups spanning 25 years or more, were split into five-year age groups using the prevalence age-pattern estimated by DisMod-MR 2.1. The DisMod-MR model used to estimate the age pattern did not contain any previously age split data.

Bias corrections/crosswalks

Estimates with known biases were adjusted/crosswalked accordingly prior to DisMod-MR 2.1. For each crosswalk of interest, pairs of the reference and the alternative estimates were matched by age, sex, location, and year. This was done for both within-study (where possible) and between-study pairs. Pairs were also made between the different alternative estimates. The ratios between these estimates were then used as inputs in a MR-BRT network meta-analysis. This analysis produced pooled ratios between the reference estimates and alternative estimates, which were used to adjust all alternative estimates in the dataset. Two adjustment ratios were used for bipolar disorder (See Table 2).

1. A point/past-month recall ratio adjusted point/past-month prevalence estimates toward the level they would have been if the study had captured past-year prevalence. We set past-year prevalence as the desirable level due to the episodic nature of bipolar disorder. Estimates of point prevalence surveying symptoms experienced in the past 30 days or less may fail to diagnose cases of bipolar disorder in a residual state, thereby underestimating prevalence.
2. A lifetime recall ratio adjusted all datapoints derived from lifetime prevalence towards the level they would have been if the study had captured past-year prevalence. Lifetime estimates were included as they are useful to capture potentially missed cases in the residual state.

The estimated UIs around the adjustment ratio incorporate Gamma which represents the between study variance across all input data in the model. This added uncertainty widens the UIs for crosswalks with significant fixed effects.

Table 2: MR-BRT crosswalk adjustment factors for bipolar disorder

Data input	Reference or alternative case definition	Gamma	Beta Coefficient, Log (95% UI)*	Adjustment factor**

Population survey	Reference: past-year prevalence of bipolar disorder	0.23		
Population survey	Alternative: point or past-month prevalence		-0.36 (-0.81–0.09)	0.70 (0.44–1.10)
Population survey	Alternative: lifetime prevalence		0.46 (-0.01–0.91)	1.58 (0.99–2.48)

**MR-BRT crosswalk adjustments can be interpreted as the factor the alternative case definition is adjusted by to reflect what it would have been had it been measured using the reference case definition. If the log/logit beta coefficient is negative, then the alternative is adjusted up to the reference. If the log/logit beta coefficient is positive, then the alternative is adjusted down to the reference.*

***The adjustment factor column is the exponentiated beta coefficient. For log beta coefficients, this is the relative rate between the two case definitions. For logit beta coefficients, this is the relative odds between the two case definitions.*

MarketScan data

We made use of United States (US) MarketScan data in our prevalence dataset. These were prevalence data for bipolar disorder derived from claims information in a database of private and public insurance schemes. Given the sparseness of the bipolar disorder prevalence dataset, this allowed us to incorporate detailed prevalence estimates by state, sex, and age in our modelling. Evaluation of the age-pattern of MarketScan data revealed that it was consistent to what can be observed in population-representative survey estimates; however, given that this data source only captures a subset of the population, the actual levels of prevalence, and the sex difference in prevalence, were not comparable and had to be adjusted accordingly.

We compared each year of MarketScan estimates against corresponding prevalence data from the National Comorbidity Survey Replication (NCS-R), a survey representative of the general US population. The resulting prevalence ratios were used to adjust all MarketScan estimates before they were entered into the bipolar disorder model. The NCS-R: MarketScan ratios are presented in Table 3 below.

Table 3. MarketScan adjustment factors

**MarketScan year**	**Males (95% UI)**	**Females (95% UI)**
2000	3.39 (2.22–4.57)	2.56 (1.74–3.38)
2010	2.17 (1.42–2.92)	1.51 (1.02–1.99)
2011	2.10 (1.38–2.83)	1.49 (1.01–1.97)
2012	2.11 (1.38–2.83)	1.45 (0.99–1.92)
2013	2.09 (1.37–2.82)	1.46 (0.99–1.92)
2014	2.05 (1.34–2.75)	1.37 (0.93–1.81)

Modelling strategy

We have made no substantive changes in the modelling strategy from GBD 2019.

After the above data processes were applied, DisMod MR 2.1 was used to model the epidemiological data for bipolar disorder. Adjustments to model priors or the dataset were made where appropriate. Where outliers were identified in the data, we reassessed the study’s methodology and quality before a

decision was made to exclude or include the data.

Data across all epidemiological parameters were initially included in the modelling process. The two studies on incidence reported 0% and 0.1% incidence of bipolar disorder and were low relative to the prevalence data. They were excluded from the final model where incidence was estimated using data from other parameters. We assumed no incidence and prevalence before age 10. This minimum age of onset was corroborated with expert feedback and was consistent with the available data. Remission was set to a maximum of 0.05 in agreement with literature and expert advice suggesting no or very little complete remission from bipolar disorder.[5,6]

Severity splits and disability weights

The GBD disability weight survey assessments include lay descriptions of sequelae highlighting major functional consequences and symptoms. The lay descriptions and disability weights for bipolar disorder severity levels are shown in Table 4. Information on the distribution of manic, depressive, and residual states of bipolar disorder was obtained from a systematic review of the literature[4] capturing data published between 1980 and 2012, and an update we conducted for GBD 2019 capturing data up to February 2018. Severity splits used in GBD 2021 were consistent with those used in GBD 2019.

Overall, 26 studies provided information on the proportion of bipolar disorder cases in a manic, depressive, or residual state. A MR-BRT analysis was used to explore between-study heterogeneity and to estimate the pooled proportion of cases falling within each bipolar health state. Two covariates were used in the analysis. The first was a sampling type covariate where the reference was population representative data or data from surveys of in- and out-patients combined. Alternatives for this covariate included data from inpatient only samples, and outpatient only samples. The second covariate was for bipolar subtypes where the reference was surveys screening for overall bipolar disorder (i.e., bipolar I, bipolar II, and/or bipolar not otherwise specified) and the alternative included studies that reported data for bipolar I only (n=6). An income covariate was tested (i.e., studies representative of high-income countries [n= 21] vs low- and middle-income countries [n=5]) but it was not statistically significant and was not included in the final analysis. The proportion of bipolar disorder cases falling within each state were as follows: manic 18.7% (9.1%–30.7%), depressive 31.7% (15.6%–48.1%), and residual 49.5% (24.9%–74.1%).

Table 4. Severity distribution, details on the severity levels for bipolar disorder and the associated disability weight with that severity

**Severity level**	**Lay description**	**DW (95% UI)**
Manic	Is hyperactive, hears and believes things that are not real, and engages in impulsive and aggressive behaviour that endangers the person and others.	0.492 (0.341–0.646)
Depressive*	Has constant sadness and has lost interest in usual activities. The person has some difficulty in daily life, sleeps badly, has trouble concentrating, and sometimes thinks about harming himself (or herself).	0.396 (0.267–0.531)
Residual	Has mild mood swings, irritability, and some difficulty with daily activities.	0.032 (0.018–0.051)

*Note. *Equivalent to the disability weight estimated for moderate major depressive disorder*

There were no significant changes in GBD 2021 results for bipolar disorder compared to GBD 2019. While we continue to improve on the data and methods used to estimate the burden of mental disorders, some

challenges need to be acknowledged. Firstly, we still have a large number of locations with no high quality

raw data available. Secondly, it is difficult to quantify and remove all variation due to measurement error in our epidemiological estimates. While we have improved the methodology used to account for known sources of bias, in some cases, we still have very few datapoints to inform these adjustments. Thirdly, there is a paucity of research on the risk factors of mental disorders which can be used as predictive covariates in our epidemiological models.

References

1. American Psychiatric Association. Diagnostic and Statistical Manual of Mental Disorders (DSM-IV-TR). Fourth Edition, Text Revision ed. Washington DC: American Psychiatric Association; 2000.
2. World Health Organization. The ICD-10 Classification of Mental and Behavioural Disorders. Clinical descriptions and diagnostic guidelines. Geneva: World Health Organization; 1992.
3. American Psychiatric Association. Diagnostic and Statistical Manual of Mental Disorders (DSM). Washington: American Psychiatric Association, 1952.
4. Ferrari AJ, Saha S, McGrath JJ, et al. Health states for schizophrenia and bipolar disorder within the Global Burden of Disease 2010 Study. *Population health metrics* 2012; **10**(1): 16.
5. Angst J, Sellaro R. Historical perspectives and natural history of bipolar disorder. *Biological Psychiatry* 2000; **48**(6): 445–57.
6. Colom F, Vieta E. The road to DSM-V. Bipolar disorder episode and course specifiers. *Psychopathology* 2009; **42**(4): 209–18.

Blindness and vision loss

Flowcharts

Vision loss

Nonfatal health outcome estimation

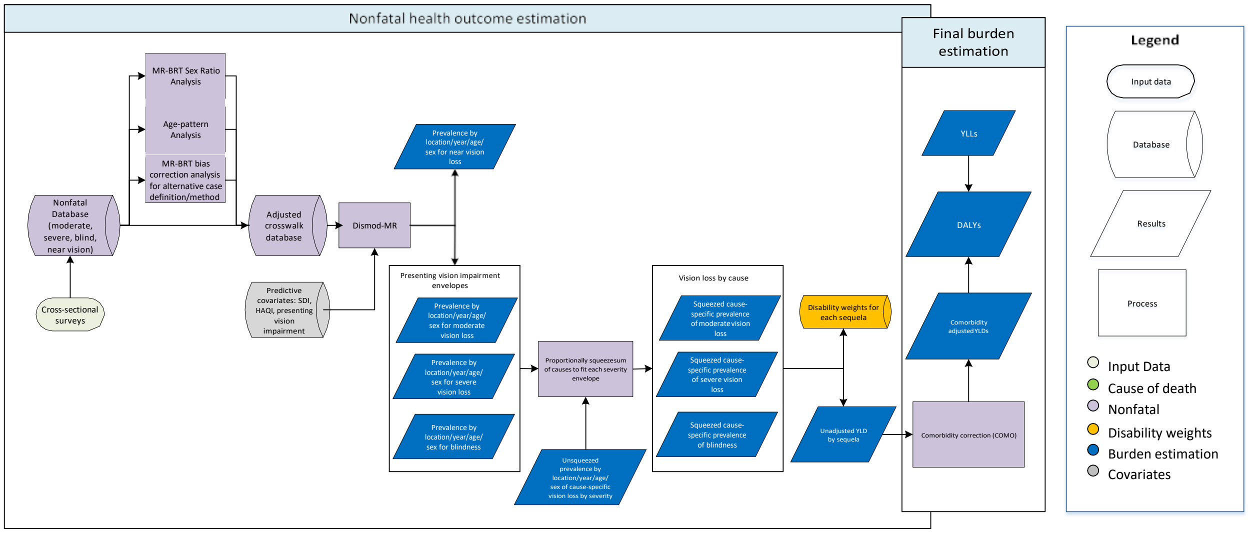

Cause-specific vision loss: cataract, glaucoma, macular degeneration, other vision loss

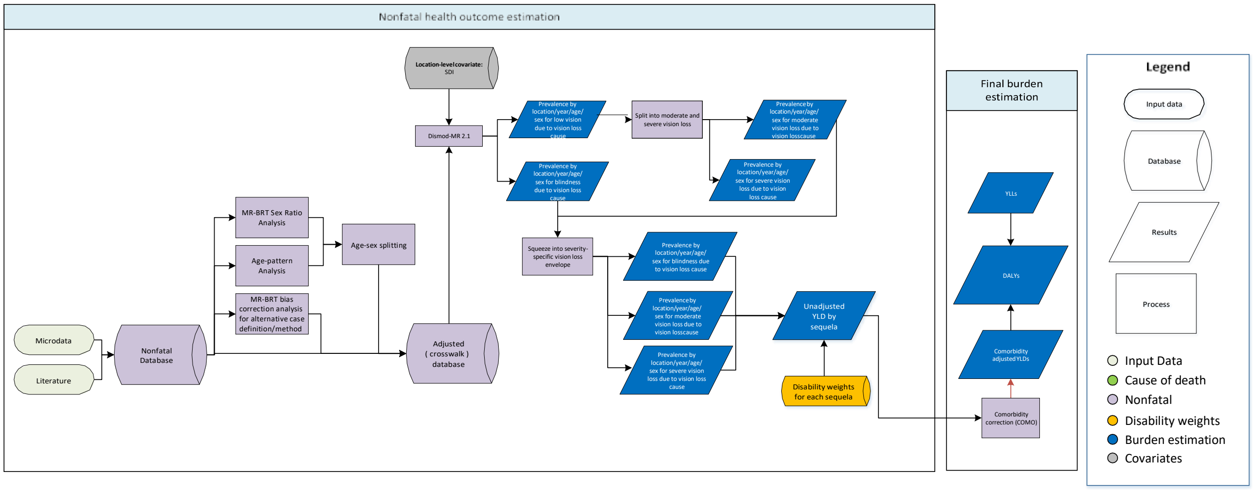

Cause-specific vision loss: diabetic retinopathy

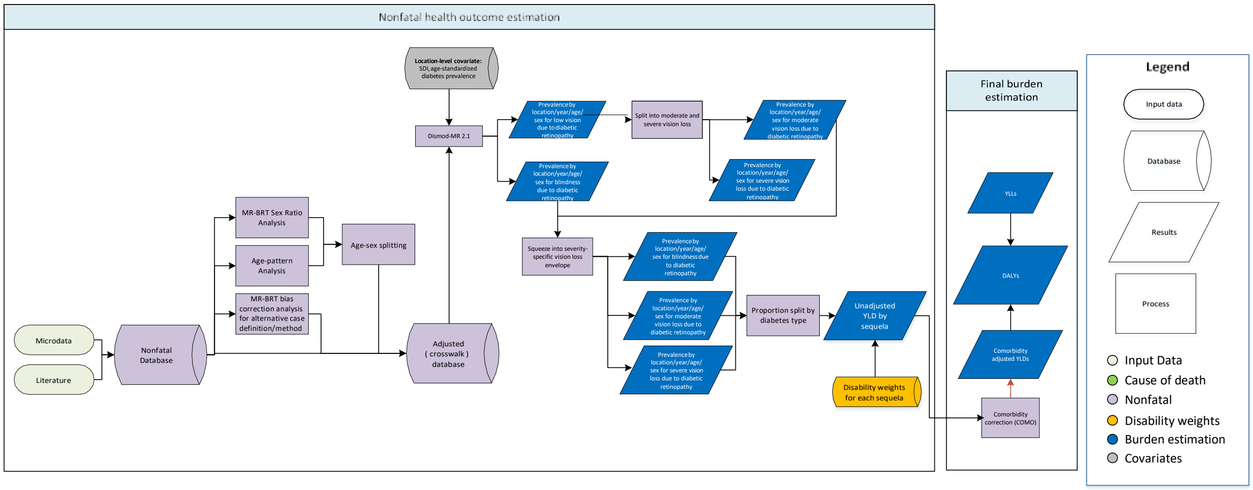

Cause-specific vision loss: trachoma

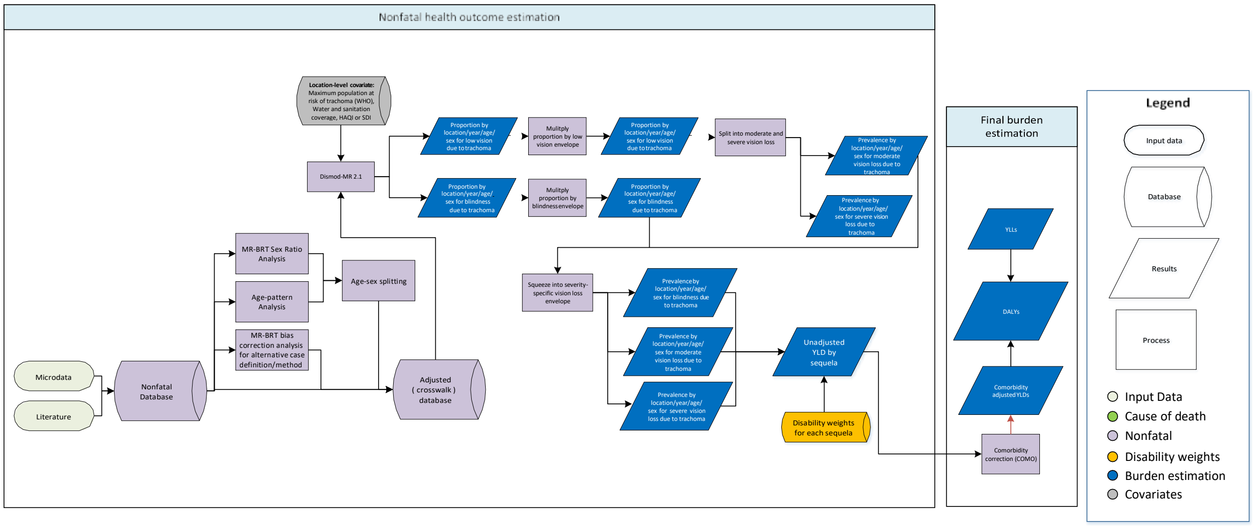

Case definition

Total vision loss and cause-specific vision loss are estimated for the following severities (Table 1). Severity of vision loss is determined using a measured visual acuity test such as a Snellen chart or LogMAR chart.

Table 1. Severity of vision loss assigned based on range of visual acuity for distance vision loss and presbyopia

Vision loss severity	Case definition
Blindness	Distance visual acuity of <3/60 or <10% visual field around central fixation
Severe vision loss	Distance visual acuity of ≥3/60 and <6/60
Moderate vision loss	Distance visual acuity of ≥6/60 and <6/18
Near vision loss (uncorrected presbyopia)	Near visual acuity of <6/12 distance equivalent

Near vision loss describes the progressive inability to focus on near objects as individuals age (presbyopia). This impairs the ability to read. The majority of presbyopia can be corrected by the use of reading glasses, contact lenses, or refractive surgery.

We model vision loss due to the following causes: uncorrected refractive error, cataract, glaucoma, macular degeneration, diabetic retinopathy, trachoma, vitamin A deficiency, retinopathy of prematurity, meningitis, encephalitis, onchocerciasis, and a residual category of other vision loss. Vision loss due to vitamin A deficiency, retinopathy of prematurity, meningitis, encephalitis, and onchocerciasis are modelled as part of their underlying cause as described in their respective sections. (Table 2)

Table 2. Causes of vision loss

Condition	Case definition
Cataract	Clouding of the lens of the eye due to protein buildup that impairs vision. Cataracts can be addressed via surgical lens replacement.
Diabetic retinopathy	Damage to the retina caused by damaged blood vessels that can leak blood into the retina and cause scarring of the retina.
Glaucoma	A condition with increased intraocular pressure which can lead to damage of the optic nerve.
Age-related macular degeneration	Age-related deterioration of the macula, the part of the retina responsible for central vision, leading to central vision loss.
Uncorrected refractive error	Blurry vision due to the lens's inability to focus. The blurriness caused by refractive error can be addressed through the use of contact lenses, glasses, or refractive surgery. Uncorrected refractive error is the difference in acuity between presenting vision (whatever corrective lens the individual uses) and best-corrected vision.
Trachoma	Results from a conjunctival bacterial infection (*Chlamydia trachomatis*) that produces inflammation and inversion of the eyelids and eyelashes scratching and scarring the cornea, and eventually leading to trichiasis and impaired vision from corneal scarring.

Input data

Model inputs

Data on overall vision loss come from surveys measuring visual acuity in representative population-based studies, either from publications in peer-reviewed and grey literature or surveys for which we had the unit record data. Data were excluded if no test was used of visual acuity that can be converted to the Snellen scale, and if a study did not assess "presenting" or "best-corrected" vision. Presenting vision is the visual acuity as measured with the glasses used by an individual. Best-corrected vision is with the best possible correction for refractive error, regardless of the strength of glasses used by an individual. A subset of these studies that reported vision loss by cause were used to estimate the prevalence of vision loss due to cataract, glaucoma, macular degeneration, diabetic retinopathy, and other causes.

For GBD 2019 and GBD 2021, we added literature sources from a systematic review conducted by collaborators in the Vision Loss Expert Group (VLEG) where all screened abstracts were sent to regional expert groups to assess data quality for inclusion. Many members of VLEG are also GBD collaborators and for GBD 2019, VLEG and GBD estimates were the same. This systematic review was conducted using the search engines MEDLINE, Embase, WHOLIS, SciELO, Open Grey and other grey literature searches commissioned by VLEG from York Health Economics Consortium, UK, an organisation that has supported the VLEG by independently conducting these searches in the past. These searches covered the time

period of 1980–2018. In total, since 2010, VLEG has provided data extracted from 137 studies, of which 67 came from the most recent systematic review update (2014-2018). Data from 95 of these literature sources that matched GBD inclusion criteria were newly added to vision models. The Vision Loss Expert Group also provided additional data provided by principle investigators for existing studies, 51 new RAAB surveys, and 5-year disaggregated data for 151 RAAB surveys (previously only data for combined ages 50–99 were available), which better informed the age pattern for vision loss in this year's estimates.

Several adjustments were made to data extracted from the original data sources.

1) Where studies only reported "both" sex data, a meta-regression in MR-BRT[1] (meta-regression—Bayesian, regularised, trimmed) was used to split these datapoints into sex-specific datapoints.
2) Where studies reported visual acuity spanning multiple thresholds (eg, <6/60, rather than separate severe and blind estimates), we applied a logit-difference adjustment meta-regression, using data from studies reporting vision loss by both severity levels.
3) Some studies reported best-corrected vision loss, but not presenting vision loss. We crosswalked these datapoints using a logit-difference meta-regression. This gave us predicted presenting vision loss datapoints for studies not explicitly reporting presenting vision loss.
4) Where datapoints spanned more than 25 years of age, we age-split using an algorithm that applies the age pattern of the super-region (from a DisMod-MR model that only contains data with age groups that span fewer than 25 years) to split the data to five-year age groups. Dismod-MR 2.1 is the tool used to produce year-age-sex-location specific prevalence estimates[1] (disease model—Bayesian meta-regression, details on this method can be found in appendix 1, section 4.5 of the citation)

Whereas other vision loss aetiologies are modelled based on prevalence data, vision loss due to trachoma is modelled as a proportion of the overall best-corrected vision loss envelope, a strategy that was chosen based on the nature of available data.

The total source count used in GBD 2021 modelling is listed in the table below:

Table 3. Total vision loss for each severity

Measure	Total sources	Countries with data
All measures	500	113
Prevalence	500	113

Table 4. Vision loss for the modelled causes of vision loss

Measure	Total sources	Countries with data
All measures	403	104
Prevalence	385	102
Proportion	25	18

Modelling strategy

We modelled the prevalence of vision loss in two steps. In the first step, we estimated the total prevalence estimates of presenting vision loss: moderate vision loss, severe vision loss, blindness, and near vision loss (presbyopia). We directly derived prevalence of near vision loss from this step, whereas the remaining three models that reflect different severity levels of distance vision loss continued to the next step.

1) Estimate severity-specific vision loss (the “envelopes”)

First, we ran five DisMod-MR 2.1 models to estimate the total prevalence estimates of vision loss: moderate presenting vision loss, severe presenting vision loss, presenting blindness, and uncorrected presbyopia.

Non-reference case definitions were adjusted to reference (full visual acuity exam, presenting vision) using MR-BRT (meta-regression—Bayesian, regularised, trimmed). Betas and exponentiated values, which can be interpreted as an odds ratio, are shown in the tables below for each adjustment for alternative case definitions. The best-corrected adjustment factor indicates whether the test measured visual acuity with the level of correction the patient presents with or the ophthalmologist provides additional correction via pinhole or lens correction. Rapid-assessment corrects for potential biases in cause-specific vision loss from studies using expedited visual acuity measurement. The severity covariate splits mixed severity data (moderate/severe, severe/blindness) into severity-specific data. Gamma captures the between study heterogeneity, and the adjustment factor is the inverse-logit transformed beta coefficient where <0.5 represents that the alternative case definition is adjusted upward and >0.5 represents that the alternative case definition is adjusted downward.

Table 5. MR-BRT crosswalk adjustment factors for vision loss models

MR-BRT crosswalk adjustment factors for moderate vision loss envelope

Data input	Reference or alternative case definition	Gamma	Beta Coefficient, Logit (95% CI)*	Adjustment factor**
Presenting visual acuity, does not use rapid methodology	Ref	0.59	---	---
Best-corrected visual acuity	Alt		-1.11 (-2.27 – 0.06)	0.33
Uses rapid methodology	Alt		-0.06 (-1.23 – 1.11)	0.94

MR-BRT crosswalk adjustment factors for severe vision loss envelope

Data input	Reference or alternative case definition	Gamma	Beta Coefficient, Logit (95% CI)*	Adjustment factor**
Presenting visual acuity, does not use rapid methodology	Ref	0.69	---	---
Best-corrected visual acuity	Alt		-0.94 (-2.30 – 0.42)	0.39
Uses rapid methodology	Alt		0.11 (-1.25 – 1.48)	1.12

MR-BRT crosswalk adjustment factors for blindness envelope

Data input	Reference or alternative case definition	Gamma	Beta Coefficient, Logit (95% CI)*	Adjustment factor**
Presenting visual acuity, does not use rapid methodology	Ref	0.02	---	---
Best-corrected visual acuity	Alt		-0.15 (-0.19 – -0.15)	0.86
Uses rapid methodology	Alt		0.07 (-0.03 – 0.34)	1.07

MR-BRT crosswalk adjustment factors for cause-specific low vision models

Data input	Reference or alternative case definition	Gamma	Beta Coefficient, Logit (95% CI)*	Adjustment factor**
Does not use rapid methodology	Ref	0.70	---	---
Uses rapid methodology	Alt		0.12 (-0.03 – 0.34)	01.13

MR-BRT crosswalk adjustment factors for cause-specific blindness models

Data input	Reference or alternative case definition	Gamma	Beta Coefficient, Logit (95% CI)*	Adjustment factor**
Does not use rapid methodology	Ref		---	---
Uses rapid methodology	Alt		0.06 (-0.03 – 0.15)	01.06

MR-BRT crosswalk adjustments can be interpreted as the factor the alternative case definition is adjusted by to reflect what it would have been had it been measured using the reference case definition. If the log/logit beta coefficient is negative, then the alternative is adjusted up to the reference. If the log/logit beta coefficient is positive, then the alternative is adjusted down to the reference.

****The adjustment factor column is the exponentiated beta coefficient. For log beta coefficients, this is the relative rate between the two case definitions. For logit beta coefficients, this is the relative odds between the two case definitions.***

Mixed severity data (either mixed moderate and severe vision loss, or mixed severe vision loss and blindness) was split into severity-specific vision loss using a meta-regression in MR-BRT with a cubic spline on age. The below plots show the underlying data input in each regression, and the model fit over age. These plots demonstrate that the ratio of moderate to severe vision loss decreases with age, and the ratio of blindness to severe vision loss increases slightly with age.

Figure 1. MR-BRT crosswalk adjustment for mixed severity vision loss data

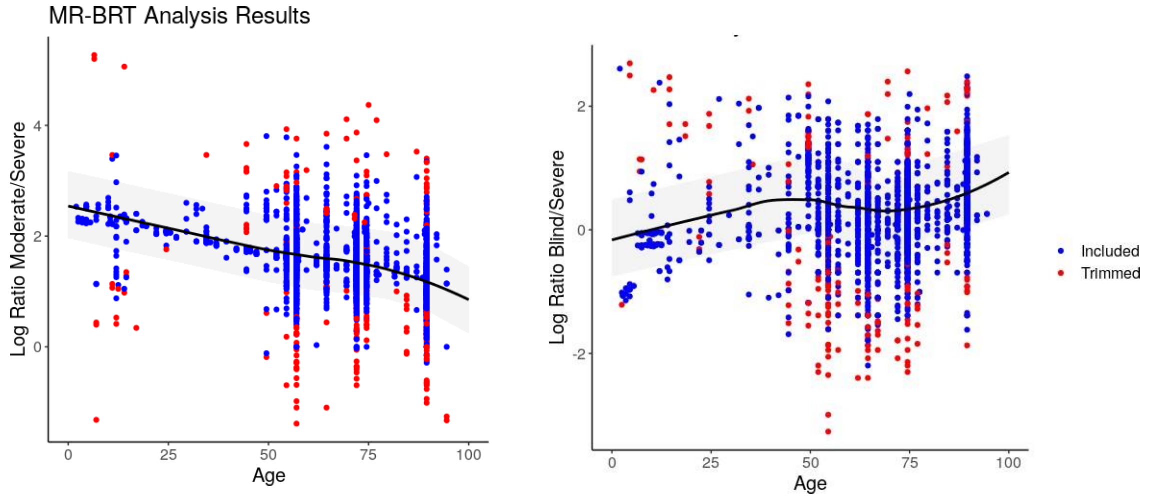

Socio-demographic Index (SDI) and Healthcare Access and Quality (HAQ) Index were used as location covariates as a proxy measure of access to eye care such as cataract surgery. All predictors are listed below for each vision model. The exponentiated beta can be interpreted as an odds ratio. For example, in row 1 below, an exponentiated beta of 0.44 for SDI means that for every 1 unit change in SDI (measured on a scale from 0 to 1), moderate vision loss is lower by a factor of 0.44.

Table 6. Summary of predictive covariates used in vision DisMod-MR meta-regression models

Cause	Covariate	Type	Coefficient	Exponentiated beta (95% Uncertainty Interval)
Moderate vision loss envelope	Socio-demographic Index	Prevalence	-0.2 (-0.35 – -0.05)	0.82 (0.70 – 0.95)
Severe vision loss envelope	Socio-demographic Index	Prevalence	-1.12 (-1.39 – -0.87)	0.32 (0.25 – 0.42)
Blindness envelope	Socio-demographic Index	Prevalence	-1.41 (-1.73 – -1.07)	0.25 (0.18 – 0.34)
Blindness envelope	Healthcare Access and Quality Index	Prevalence	-0.02 (-0.02 – -0.01)	0.98 (0.98 – 0.99)
Moderate vision loss due to uncorrected refractive error	Socio-demographic Index	Prevalence	-1.45 (-1.50 – -1.38)	0.23 (0.22 – 0.25)
Severe vision loss due to uncorrected refractive error	Socio-demographic Index	Prevalence	-1.91 (-2.00 – -1.74)	0.15 (0.14 – 0.18)
Blindness due to uncorrected refractive error	Socio-demographic Index	Prevalence	-1.98 (-2.00 – -1.95)	0.14 (0.14 – 0.14)
Vision loss due to other vision loss	Socio-demographic Index	Prevalence	-1.00	0.37 (0.37-0.37)
Blindness due to other vision loss	Socio-demographic Index	Prevalence	-1.00	0.37 (0.37-0.37)
Vision loss due to macular degeneration	Socio-demographic Index	Prevalence	-0.94	0.39 (0.37 – 0.45)
Blindness due to macular degeneration	Socio-demographic Index	Prevalence	-0.91	0.40 (0.37 – 0.48)
Vision loss due to glaucoma	Socio-demographic Index	Prevalence	-0.99	0.37 (0.37 – 0.38)

Blindness due to glaucoma	Socio-demographic Index	Prevalence	-1.97	0.14 (0.14 – 0.15)

Vision loss due to cataract	Socio-demographic Index	Prevalence	-0.66	0.52 (0.40 – 0.66)
Blindness due to cataract	Socio-demographic Index	Prevalence	-2.96	0.052 (0.05 – 0.05)
Vision loss due to diabetes mellitus	Socio-demographic Index	Prevalence	-1.7	0.18 (0.14 – 0.29)
Vision loss due to diabetes mellitus	Diabetes age-standard prevalence (proportion)	Prevalence	0.72	2.05 (1.56 – 2.70)
Blindness due to diabetes mellitus	Socio-demographic Index	Prevalence	-1.77	0.17 (0.14 – 0.24)
Blindness due to diabetes mellitus	Diabetes age-standard prevalence (proportion)	Prevalence	3.95	52.12 (48.23 – 54.49)
Vision loss due to trachoma	Socio-demographic Index	Proportion	-5.99	0.003 (0.003 – 0.003)
Blindness due to trachoma	Healthcare Access and Quality Index	Proportion	-1.98	0.14 (0.11 – 0.17)
Blindness due to trachoma	Max trachoma population at risk	Proportion	-0.66	0.51 (0.30 – 0.82)
Blindness due to trachoma	Improved water source (proportion access)	Proportion	-2.19	0.11 (0.07 – 0.18)

2 Estimate cause-specific vision loss

In the second step, we estimated the prevalence of vision loss due to multiple causes: refractive error, cataract, glaucoma, macular degeneration, diabetic retinopathy, retinopathy due to prematurity, trachoma, vitamin A deficiency, onchocerciasis, meningitis, and other causes not classified elsewhere. Vision loss due to retinopathy of prematurity, vitamin A deficiency, onchocerciasis, meningitis, tetanus, and neonatal conditions was modelled as part of these underlying causes; see their respective write-ups for more information.

For each of cataract, glaucoma, macular degeneration, diabetic retinopathy, and other vision loss, we ran two DisMod-MR 2.1 models: one for the combined category of moderate and severe vision loss due to the cause, and one for blindness due to the cause. Moderate and severe vision loss were modelled together because input data were mostly available for the aggregate. Refractive error was modelled in three models, one for each severity.

We used the following age restrictions based on input from the Vision Loss Expert Group:

Table 7. Age restriction in cause-specific DisMod-MR models.

Cause	Minimum age
Cataracts	20
Glaucoma	45
Macular degeneration	45
Diabetic retinopathy	20
Trachoma	15
Other vision loss	0

Vision loss due to trachoma was modelled as a proportion of the envelope, with separate proportion models for (severe and moderate) vision loss and blindness. We estimated the proportions of low vision and blindness due to trachoma using DisMod-MR 2.1 models. Our model included fixed effects on the maximum population at risk for trachoma (proportion) reported by WHO, the proportion of the population with access to sanitation, and HAQ Index. Finally, we applied geographic and age restrictions to ensure that we estimate zero proportions in non-endemic locations (see neglected tropical disease appendices for more information) and among those younger than 15 year of age (as scarring of the cornea due to trachoma takes decades to develop).

We split the moderate plus severe vision loss estimates for each cause into moderate and severe using the ratio of presenting moderate and severe vision loss envelopes. Onchocerciasis and retinopathy of prematurity are the two exceptions because moderate and severe vision loss are modelled as part of the estimation process of these causes.

We scaled the cause-specific vision loss prevalence to the total prevalence of the vision loss envelopes for each of the three severity levels. The final result is prevalence of vision loss due to each cause by severity.

Table 8. Health states and disability weights.

Health state name	Health state description	Disability weight
Distance vision, severe loss	This person has severe vision loss, which causes difficulty in daily activities, some emotional impact (for example, worry), and some difficulty going outside the home without assistance.	0.184 (0.125–0.259)
Distance vision, moderate loss	This person has vision problems that make it difficult to recognise faces or objects across a room.	0.031 (0.019–0.049)
Distance vision blindness	This person is completely blind, which causes great difficulty in some daily activities, worry and anxiety, and great difficulty going outside the home without assistance.	0.187 (0.124–0.26)
Near Vision Loss	This person has difficulty seeing things that are nearer than 3 feet if uncorrected by reading glasses, but has no difficulty with seeing things at a distance.	0.011 (0.005–0.02)

Bulimia nervosa

Flowchart

Bulimia nervosa

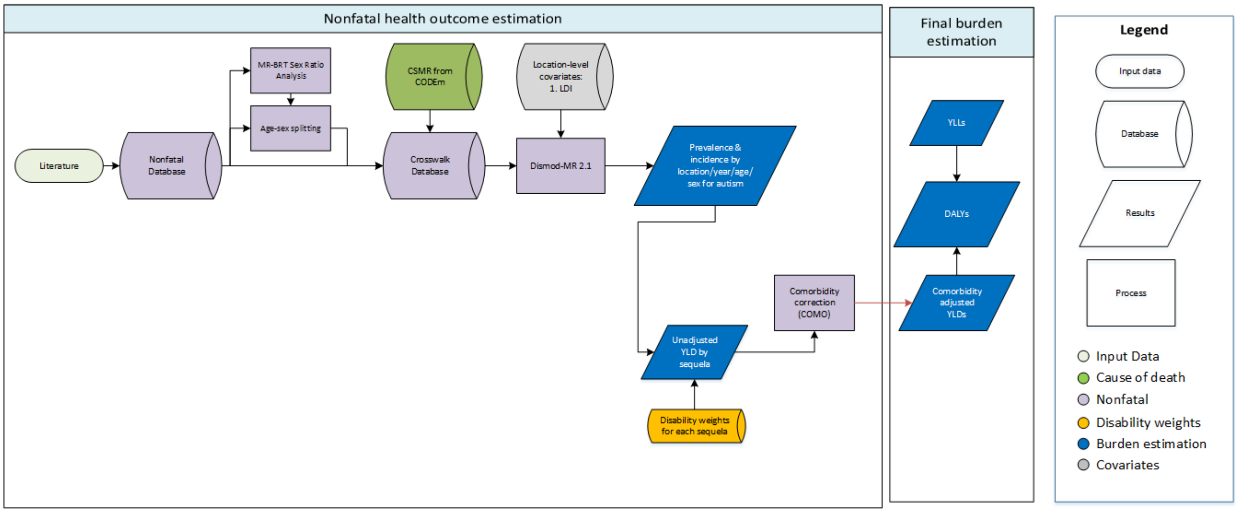

Input data and methodological summary for bulimia nervosa

Case definition

According to the Diagnostic and Statistical Manual of Mental Disorders fourth edition, text revision (DSM-IV-TR),[1] bulimia nervosa (BN) is an eating disorder characterised by:

a) Recurrent episodes of binge eating. An episode of binge eating is characterised by both of the following:
    1) eating, in a discrete period of time (eg, within any two-hour period), an amount of food that is definitely larger than most people would eat during a similar period of time and under similar circumstances
    2) a sense of lack of control over eating during the episode (eg, a feeling that one cannot stop eating or control what or how much one is eating)
b) Recurrent inappropriate compensatory behaviour in order to prevent weight gain, such as self-induced vomiting; misuse of laxatives, diuretics, enemas, or other medications; fasting; or excessive exercise.
c) The binge eating and inappropriate compensatory behaviours both occur, on average, at least twice a week for three months (changed to once a week for three months in DSM-5).[2]
d) Self-evaluation is unduly influenced by body shape and weight.
e) The disturbance does not occur exclusively during episodes of anorexia nervosa.

Included in GBD were cases meeting diagnostic criteria according to DSM[1] or the International Classification of Diseases (ICD).[3] These were identified by the following codes: 307.51 (DSM-IV-TR) and F50.2 (ICD-10). Different versions of DSM (DSM-III, DSM-III-R, DSM-IV, DSM-IV-TR, DSM-5, and DSM-5-TR) and ICD (ICD-9, ICD-10, and ICD-11) were accepted.

Input data

Systematic literature reviews were conducted to capture studies reporting the prevalence, incidence, remission, and excess mortality of BN. These were conducted in three stages involving electronic searches of the peer-reviewed literature (ie, via PsycInfo, Embase, and PubMed), the grey literature, and expert consultation. For mental disorders, we update our GBD electronic database searches on a rolling basis. An electronic search was not required for GBD 2021. The next update will be conducted in the next round of

GBD. The grey literature searches and expert consultation were conducted for GBD 2021.

The GBD inclusion criteria stipulated that: 1) the publication year must be from 1980 onward; 2) "caseness" must be based on clinical threshold as established by the DSM or ICD; 3) sufficient information must be provided on study method and sample characteristics to assess the quality of the study; and 4) study samples must be representative of the general population (ie, inpatient or pharmacological treatment samples, case studies, veterans, or refugee samples were excluded). No limitation was set on the language of publication. Methods used for this systematic review have been reported in greater detail elsewhere.[4] Table 1 summarises data inputs by parameter for BN.

Table 1: Data Inputs for BN morbidity modelling by parameter

Parameter	Countries with data	New sources	Total sources
Incidence	4	0	4
Prevalence	31	0	66
Remission	7	5	15
Other	7	6	12

Age-sex splitting

The extracted data underwent three types of age-sex splitting processes:

8. Where possible, estimates were further split by sex and age based on the available data. For instance, if studies reported prevalence for broad age groups by sex (eg, prevalence in 15–65-year-old males and females separately), and also by specific age groups but for both sexes combined (eg, prevalence in 15–30-year-olds, then in 31–65-year-olds, for males and females combined); age-specific estimates were split by sex using the reported sex-ratio and bounds of uncertainty.
9. A meta-regression—Bayesian, regularised, trimmed (MR-BRT) analysis was used to split the remaining both-sex estimates in the dataset. For each parameter, sex-specific estimates were matched by location, age, and year. A MR-BRT network meta-analysis was then used to estimate pooled sex ratios and bounds of uncertainty. These were then used to split the both-sex estimates in the dataset. The male-to-female prevalence ratio estimated was 0.39 (95% uncertainty interval [UI]: 0.28-0.50).
10. Studies reporting prevalence estimates across age groups spanning 25 years or more, were split into five-year age groups using the prevalence age pattern estimated by DisMod-MR 2.1. The DisMod-MR model used to estimate the age pattern did not contain any previously age split data.

Bias corrections/crosswalks

We tested for a number of potential sources for bias in prevalence between studies (eg, use of ICD criteria vs DSM criteria). However, none of the crosswalks had a statistically significant impact on prevalence and so no bias corrections were applied to these estimates.

Modelling strategy

After the above data processes were applied, DisMod MR 2.1 was used to model the epidemiological data for BN. Adjustments to model priors or the dataset were made where appropriate. Where outliers were identified in the data, we reassessed the study's methodology and quality before a decision was made to exclude or include the data.

We assumed no incidence prior to 10 years of age or onward from 40 years of age. In GBD 2021 a decision was made to remove BN as a cause of death due to the very limited data available to inform this model. There was also no clear epidemiological evidence from our systematic review of the literature to suggest that BN is associated with a statistically significant risk of death. Instead, excess-mortality was set to 0 in our analysis.

A country-level covariate, lagged distributed income (LDI), was also included. This covariate represents a moving average of gross domestic product (GDP) over time. The limits placed on this covariate meant that prevalence was assumed to increase with rising GDP. A summary of location-level covariates and exponentiated values for BN are shown in Table 2.

Table 2: Summary of covariates used in the BN DisMod-MR meta-regression model

Covariate	Type	Parameter	Exponentiated beta (95% UI)
LDI ($ per capita)	Location-level	Prevalence	1.50 (1.32—1.64)

Disability weight

The GBD disability weight survey assessments include lay descriptions of sequelae highlighting major functional consequences and symptoms. No severity splits were applied to BN. The lay description and disability weight for BN are shown in Table 3 below.

Table 3: Lay description for BN in GBD 2021 and the associated disability weight

Lay description	Disability weight (95% UI)
Has uncontrolled overeating followed by guilt, starving, and vomiting to lose weight.	0.223 (0.149–0.311)

We saw an increase in prevalence in GBD 2021 compared to GBD 2019 as a result of setting excess mortality to 0 in our analysis. Data on the elevated risk of mortality in those with BN are limited, making any excess-mortality or cause of death analysis difficult within the GBD framework.

While we continue to improve on the data and methods used to estimate the burden of mental disorders, some other challenges need to be acknowledged. Firstly, we still have a large number of locations with no high quality raw data available. Secondly, it is difficult to quantify and remove all variation due to measurement error in our epidemiological estimates. While we have improved the methodology used to account for known sources of bias, in some cases, we still have very few datapoints to inform these adjustments. Thirdly, there is a paucity of research on the risk factors of mental disorders which can be used as predictive covariates in our epidemiological models.

References

1. American Psychiatric Association. Diagnostic and Statistical Manual of Mental Disorders (DSM-IV-TR). 4th, Text Revision ed. Washington DC: American Psychiatric Association; 2000.
2. American Psychiatric Association. Diagnostic and Statistical Manual of Mental Disorders, Fifth Edition. Arlington, VA: American Psychiatric Association; 2013.
3. World Health Organization. ICD-10 Classification of Mental and Behavioural Disorders: Clinical descriptions and diagnostic guidelines. Geneva: World Health Organization; 1992.
4. Ferrari AJ, Charlson FJ, Norman RE, et al. Burden of depressive disorders by country, sex, age,

and year: findings from the global burden of disease study 2010. *PLoS Med* 2013; **10**(11): e1001547.

Cannabis use disorders

Flowchart

Cannabis dependence

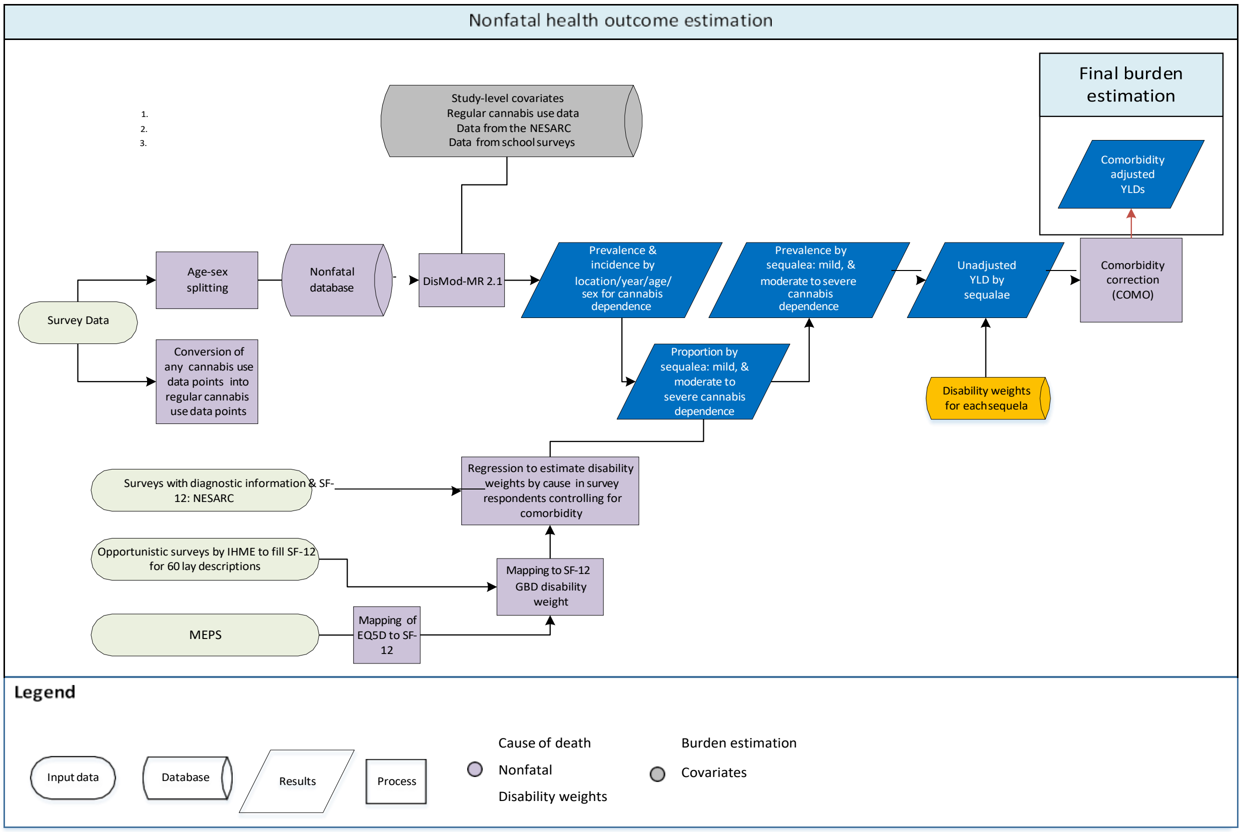

Input data and methodological summary for cannabis use disorders

Case definition

Cannabis dependence is a substance-related disorder involving a dysfunctional pattern of cannabis use. Included in the Global Burden of Disease (GBD) modelling were cases meeting diagnostic criteria for cannabis dependence according to the Diagnostic and Statistical Manual of Mental Disorders (DSM), or the equivalent diagnosis in the International Classification of Diseases (ICD). These were identified by the following codes: DSM:304.30, ICD:F12.2; excluding those cases due to a general medical condition.[1,2]

According to DSM-IV-TR criteria, cannabis dependence involves a maladaptive pattern of cannabis use, leading to clinically significant impairment or distress. At least three of the following symptoms must be experienced within the same 12-month period:

- Tolerance, characterised by either
    - a need for increased amounts of the substance to achieve intoxication; or
    - markedly diminished effect with continued use of the same amount of the substance;

- Withdrawal, characterised by either
    - Withdrawal symptoms characteristic to cannabis dependence; or
    - the same (or similar) substance is taken to avoid withdrawal symptoms;
- substance taken in progressively larger amounts or for longer periods;
- persistent desire or unsuccessful efforts to reduce substance use;
- disproportionate time dedicated to obtaining the substance;
- other important activities are given up because of the substance use; and
- substance use is continued despite knowledge of physical or psychological problems occurring as a result of the substance.

Input data

For GBD 2010, a systematic review of the literature was conducted to capture studies of prevalence, incidence, duration, and excess mortality associated with cannabis dependence. In summary, the search was conducted in three stages involving electronic searches of the peer-reviewed literature (via PsycInfo, Embase and PubMed), the grey literature, and expert consultation. The agreed-upon approach for mental and substance use disorders was to conduct electronic database searches on a rolling basis. All three stages of GBD 2010's literature review were repeated for GBD 2013 and GBD 2016. In GBD 2017, stages two and three of the literature review were conducted. Additionally, two targeted systematic reviews were conducted to further supplement the cannabis dependence dataset. The first review captured studies reporting on the epidemiology of cannabis dependence within Maori versus non-Maori populations (as opposed to New Zealand more broadly), given the inclusion of these two sub-groups in GBD 2017. The second review searched for studies on the epidemiology of cannabis dependence in China using primarily the China National Knowledge Infrastructure database. The focus was to search for studies published in Chinese journals that would not typically be captured in mainstream databases such as PsycInfo, Embase, and PubMed.

The inclusion criteria stipulated that: (1) the publication year must be from 1980 onward; (2) "caseness" must be based on clinical threshold as established by the DSM or ICD; (3) sufficient information must be provided on study method and sample characteristics to assess the quality of the study; and (4) study samples must be representative of the general population (ie, inpatient or pharmacological treatment samples, case studies, veterans or refugee samples were excluded). No limitation was set on the language of publication. Methods used for this systematic review have been reported in greater detail elsewhere.[3-6]

Table 1: Data inputs for cannabis use disorders morbidity modelling by parameter

Measure	Total sources	Countries with data
All measures	806	121
Prevalence	802	121
Remission	3	3

Age and sex splitting

Reported estimates of prevalence were split by age and sex where possible. First, if studies reported prevalence for broad age groups by sex (eg, prevalence in 15- to 65-year-old males and females separately), and also by specific age groups for both sexes combined (eg, prevalence in 15- to 30-year-olds, then in 31- to 65-year-olds, for males and females combined), age-specific estimates were split by sex using the reported sex ratio and bounds of uncertainty. Second, prevalence data for both sexes that could not be split using a within-study ratio were split using a sex ratio derived from a meta-analysis of existing sex-specific data using our meta-regression—Bayesian, regularised, trimmed tool[7] (MR-BRT). Details on MR-BRT can be found in appendix 1, section 4.4.1 of the reference article.

The female to male ratio was 0.49 (0.33 to 0.70) for ages 20 and above and 0.61 (0.42 to 0.88) for ages below 20. Finally, after the application of bias adjustments, where studies reported estimates across age groups spanning 25 years or more, these were split into five-year age groups using the super-region-specific prevalence age pattern estimated by our disease model—Bayesian meta-regression tool[8] (DisMod-MR 2.1) on all data prior to age-splitting. Information on DisMod-MR 2.1 can be found in appendix 1, section 4.5 of the reference article.

Data adjustment

Due to insufficient data in the optimal case definition of cannabis dependence, the prevalence dataset included datapoints originally reporting any cannabis use, regular (ie, weekly) cannabis use, and cannabis dependence. Adjusting any cannabis use and regular cannabis use to cannabis dependence involved a two-step process. In the first stage, estimates of any cannabis use were converted to estimates of regular cannabis use. In GBD 2021, we retained the GBD 2019 adjustment coefficient for this first stage. Briefly, a ratio of any use to regular use was calculated by comparing similar regular use and any use estimates in the dataset. To allow for meaningful comparisons, paired regular use and use estimates needed to be similar in terms of the country they were from, year, age group, sex, and prevalence type. Once a dataset was set up with paired regular use and use estimates, MetaXL (a meta-analysis add-in for Microsoft Excel) was used to estimate a ratio of use to regular use whereby use estimates were found to be 2.9 (2.5–3.3) times higher than regular use estimates. This ratio was used to adjust all use estimates in the dataset downward, toward the level they would have been had the study reported regular cannabis use.

In GBD 2019, we focused on updating the second stage of the adjustment, in which regular use estimates were converted to cannabis dependence estimates, using a logit-difference coefficient calculated using MR-BRT. In this second stage we also adjusted for bias in school-based surveys compared to household surveys among youth. We found an age pattern to the relationship between regular use and dependence, and therefore ran separate models for youth (under age 25) and adults (over age 25). A network analysis allowing for both direct and indirect comparisons was preferred for adjusting youth data for the two study-level covariates (regular use and school-based surveys); therefore, two separate MR-BRT models were run on cannabis data, one on adults and one on youth. Compared to GBD 2017, adjustments calculated using a logit-difference approach in MR-BRT resulted in slightly higher post-adjustment prevalence estimates among both youth and adults. No changes were made in GBD 2021.

Table 2: MR-BRT crosswalk adjustment factors for cannabis use disorder, youth

Data input	Reference or alternative case definition	Gamma	Beta coefficient, logit (95% UI)*
Cannabis dependence, household-based	Ref	0.32	---
Cannabis dependence, school-based	Alt		0.33 (-0.30 to 0.94)
Cannabis regular use, household-based	Alt		0.73 (0.12 to 1.34)
Cannabis regular use, school-based	Alt		1.08 (0.44 to 1.70)

MR-BRT crosswalk adjustments can be interpreted as the factor the alternative case definition is adjusted by to reflect what it would have been had it been measured using the reference case definition. If the log/logit beta coefficient is negative, then the alternative is adjusted up to the reference. If the log/logit beta coefficient is positive, then the alternative is adjusted down to the reference.

Table 3: MR-BRT crosswalk adjustment factors for cannabis use disorder, adults

Data input	Reference or alternative case definition	Gamma	Beta coefficient, logit (95% UI)*
Cannabis dependence	Ref	0.28	---
Cannabis regular use	Alt		1.31 (0.77 to 1.86)

MR-BRT crosswalk adjustments can be interpreted as the factor the alternative case definition is adjusted by to reflect what it would have been had it been measured using the reference case definition. If the log/logit beta coefficient is negative, then the alternative is adjusted up to the reference. If the log/logit beta coefficient is positive, then the alternative is adjusted down to the reference.

Modelling strategy

Prior settings in DisMod included assuming no incidence before age 13. This minimum age of onset was corroborated with expert feedback and existing literature on cannabis dependence. We also assumed no incidence after age 70 as supported by data from various sources including the European Monitoring Centre for Drugs and Drug Addiction.[9] An upper limit of 0.25 was placed on remission, consistent with limits in the dataset. These settings were retained for GBD 2021. In GBD 2021, as in GBD 2019, no country-level covariates were used in predictions.

Severity and disability

The basis of the GBD disability weight survey assessments are lay descriptions of health states highlighting major functional consequences and symptoms. The lay descriptions and disability weights for cannabis dependence severity levels are shown below.

Table 4. Severity distribution, details on the severity levels for cannabis use disorders in GBD 2021 and the associated disability weight (DW) with that severity.

Severity level	Lay description	DW (95% CI)
Mild	Uses marijuana at least once a week and has some difficulty controlling the habit. When not using, the person functions normally.	0.039 (0.024–0.06)
Moderate to severe	Uses marijuana daily and has difficulty controlling the habit. The person sometimes has mood swings, anxiety, and hallucinations, and has some difficulty in daily activities.	0.266 (0.178–0.364)

The US National Epidemiological Survey on Alcohol and Related Conditions (NESARC, conducted in two waves from 2001 to 2002 and 2004 to 2005)[10] was used to estimate the proportion of cannabis dependence cases asymptomatic (58%, 51%–63%), mild (36%, 31%–42%), and moderate to severe (6%, 4%–8%). NESARC is a direct household survey. As such, it is expected to underestimate moderate to severe cases of drug dependence; however, there are very few sources of usable drug severity data.

References

1. American Psychiatric Association. Diagnostic and Statistical Manual of Mental Disorders (DSM-IV-TR). 4th, Text Revision ed. Washington DC: American Psychiatric Association; 2000.
2. World Health Organization. The ICD-10 Classification of Mental and Behavioural Disorders. Clinical descriptions and diagnostic guidelines. Geneva: World Health Organization; 1992.
3. Calabria B, Degenhardt L, Briegleb C, et al. Systematic review of prospective studies investigating "remission" from amphetamine, cannabis, cocaine or opioid dependence. Addictive Behaviors 2010; 35(8): 741-9.
4. Calabria B, Degenhardt L, Hall W, Lynskey M. Does cannabis use increase the risk of death? Systematic review of epidemiological evidence on adverse effects of cannabis use. Drug Alcohol Rev 2010; 29(3): 318-30.
5. Calabria B, Degenhardt L, Nelson P, et al. What do we know about the extent of cannabis use and dependence? Results of a global systematic review. Sydney: National Drug and Alcohol Research Centre, University of NSW, 2010.
6. Degenhardt L, Ferrari AJ, Calabria B, et al. The global epidemiology and contribution of cannabis use and dependence to the global burden of disease: results from the GBD 2010 study. PloS one 2013; 8(10): e76635.
7. Murray CJL, Aravkin AY, Zheng P, et al. Global burden of 87 risk factors in 204 countries and territories, 1990–2019: a systematic analysis for the Global Burden of Disease Study 2019. The Lancet 2020; 396: 1223–49. doi: https://doi.org/10.1016/S0140-6736(20)30752-2

8. Vos T, Lim SS, Abbafati C, et al. Global burden of 369 diseases and injuries in 204 countries and territories, 1990–2019: a systematic analysis for the Global Burden of Disease Study 2019. The Lancet 2020; 396: 1204–22. doi: https://doi.org/10.1016/S0140-6736(20)30925-9
9. Introduction to the National Epidemiologic Survey on Alcohol and Related Conditions [http://pubs.niaaa.nih.gov/publications/arh29-2/74-78.htm]. Access date 1 December 2014.
10. European Montioring Centre for Drugs and Drug Addiction. Lisbon, Portugal; 2014.

Cellulitis

Flowchart for cellulitis

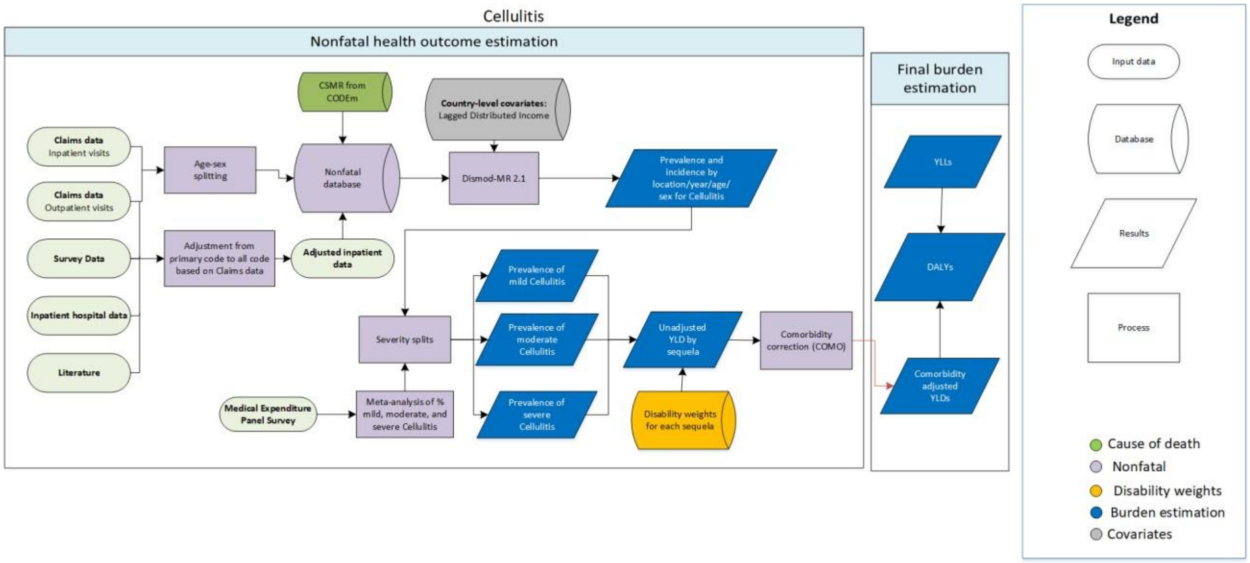

Input data and methodological summary for cellulitis

Case definition

Cellulitis was included in the GBD 2019 cause group of skin and subcutaneous conditions. Cellulitis is a skin disease marked by a bacterial infection that affects and spreads through the skin and soft tissues. (ICD-10: L03)

**Quantity of interest**	**Reference or Alternative**	**Definition**
Cellulitis	Reference	Cellulitis as determined by a physical exam.
Cellulitis	Alternative	Cellulitis as indicated by hospital admission and claims data.
Cellulitis	Alternative	Self-reported cellulitis.

Input data

In the GBD 2010 study, a systematic review of the literature was conducted using PubMed and Google Scholar to capture epidemiological data for cellulitis. Due to lack of published data on the epidemiology of

cellulitis, the literature search also included relevant incidence data from national inpatient or outpatient records in Europe, North America, and Latin America. When years in the national data from the hospital records overlapped, inpatient and outpatient data were summed together in an effort to better estimate the population incidence of cellulitis. The final dataset also includes USA claims data, Taiwan (province of China) claims data, Poland claims data, hospital inpatient data, and cause-specific mortality rates for cellulitis estimated by CODEm.

The inclusion criteria stipulated that studies (1) must be published between 1980 and 2012; (2) must provide data on the incidence or prevalence of cellulitis; (3) must use samples representative of the general population (ie, samples derived from the experimental arm of clinical trials or based in dermatology clinics were excluded); (4) must use a sample size larger than 100; and (5) must provide sufficient information on study method and sample characteristics to assess the quality of the study. For GBD 2013, the GBD 2010 search strategy was replicated to capture epidemiological studies published between 2012 and 2013. Data were outliered or excluded if we found them unreasonable when compared to regional, super-regional, and global rates.

Table 1: Data inputs for cellulitis morbidity modelling by parameter

**Cause/impairment name**	**Measure**	**Countries with data**	**New sources**	**Total sources**
Cellulitis	All measures	48	30	334
Cellulitis	Incidence	48	30	319
Cellulitis	Proportion	1	0	15

Table 2: MR-BRT crosswalk adjustment factors for cellulitis

Data input	Reference or alternative case definition	Gamma	Beta coefficient, logit* (95% CI)	Adjustment factor**
Literature with physical exam	Reference	1.13	---	---
USA MarketScan 2000	Alternative		0.09 (–2.86 to 3.03)	0.52
USA MarketScan 2010	Alternative		0.14 (–2.88 to 3.15)	0.53
USA MarketScan 2011	Alternative		0.15 (–2.86 to 3.16)	0.54
USA MarketScan 2012	Alternative		0.18 (–2.83 to 3.19)	0.54
USA MarketScan 2013	Alternative		0.10 (–2.91 to 3.12)	0.53
USA MarketScan 2014	Alternative		0.10 (–2.91 to 3.11)	0.53
USA MarketScan 2015	Alternative		–0.02 (–3.03 to 2.99)	0.50
USA MarketScan 2016	Alternative		–0.20 (–3.21 to 2.82)	0.45

USA MarketScan 2017	Alternative		–0.15 (–3.17 to 2.86)	0.46
Taiwan claims	Alternative		0.45 (–2.57 to 3.47)	0.61
Inpatient data	Alternative		–0.83 (–3.83 to 2.16)	0.30

***MR-BRT crosswalk adjustments can be interpreted as the factor the alternative case definition is adjusted by to reflect what it would have been had it been measured using the reference case definition. If the log/logit beta coefficient is negative, then the alternative is adjusted up to the reference. If the log/logit beta coefficient is positive, then the alternative is adjusted down to the reference.**

****The adjustment factor column is the exponentiated beta coefficient. For log beta coefficients, this is the relative rate between the two case definitions. For logit beta coefficients, this is the relative odds between the two case definitions.**

Modelling strategy

DisMod-MR 2.1, a Bayesian meta-regression tool, was used to estimate cellulitis prevalence by age, sex, year, and geography (subnational [select countries], country, region, super-region). Cellulitis was modelled with remission set between 12 and 30, implying a duration of 12 days to one month. This was in line with the available epidemiological data, expert opinion, and previous GBD work. The cellulitis dataset was sufficiently large to make use of a relatively short time window of five years to determine which datapoints were used for a particular year of fit.

In GBD 2020, we replaced our within-DisMod crosswalks with crosswalks completed using the MR-BRT modelling tool. We adjusted inpatient data, along with USA claims data and Taiwan claims data toward the level of other incidence datapoints which were more representative of the general population. In addition, log-transformed lagged-distributed income (LDI) was used as a country-level covariate to guide estimates for locations with few or no data. LDI was restricted to a range of –0.5 to –0.1. We restricted location random effects to (–0.5, 0.5) across all seven GBD super-regions.

In previous rounds, priors on excess mortality rate (EMR) were estimated in DisMod by matching prevalence datapoints with their corresponding CSMR values within the same age, sex, year, location (by dividing CSMR by prevalence). For short-duration conditions (remission >1), the corresponding prevalence was derived by running an initial model and then applying the same CSMR/prevalence method. However, for many causes, DisMod estimated a rather unrealistic pattern of EMR compared to an expected pattern of decreasing EMR with greater access to quality health care. Such unexpected patterns often signal inconsistencies between CSMR estimates and the measures of prevalence and/or incidence. In effort to provide greater guidance to DisMod on the expected pattern of EMR, EMR data generated in the previous round were modelled using the MR-BRT approach by age and sex with a prior on Healthcare Access and Quality (HAQ) Index having a negative coefficient. Results from MR-BRT were then predicted for each location, year, sex, and for ages 0, 10, 20 ….100.

The table below indicates the covariates, parameters, and exponentiated beta values used in GBD 2021.

We have made no substantive changes in the modelling strategy from GBD 2019.

Table 3. Severity distribution, details on the severity levels for cellulitis and the associated disability weight (DW) with that severity.

Sequela	Severity level	Lay description	DW (95% CI)

Mild cellulitis	Infectious disease, acute episode, mild	This person has a low fever and mild discomfort, but no difficulty with daily activities.	0.006 (0.002–0.012)
Moderate cellulitis	Infectious disease, acute episode, moderate	This person has a fever and aches, and feels weak, which causes some difficulty with daily activities.	0.051 (0.032–0.074)
Severe cellulitis	Infectious disease, acute episode, severe	This person has a high fever and pain, and feels very weak, which causes great difficulty with daily activities.	0.133 (0.088–0.19)

Table 4. Covariates. Summary of covariates used in the Cellulitis DisMod-MR meta-regression model

Covariate	Type	Parameter	Exponentiated beta (95% uncertainty interval)
LDI (I$ per capita)	Country-level	Excess mortality rate	0.70 (0.70–0.70)

Chagas disease

Flowchart

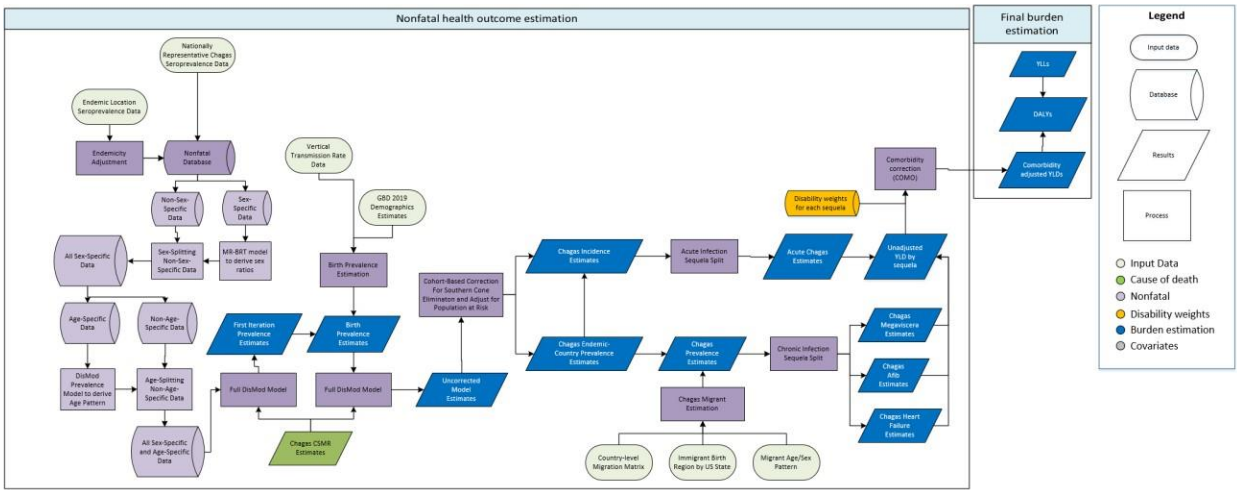

Case definition

Chagas disease is a protozoan infection caused by Trypanosoma cruzi, transmitted primarily by triatomine insects. Acute infection can cause fever, rash, headache, swollen lymph nodes, and pain, while chronic infection can lead to cardiac and gastrointestinal disorders. It includes all ICD-10 codes

under the heading B57 (Chagas disease), with codes B57.0-B75.1 corresponding to the acute phase,

B57.2 corresponding to chronic cardiovascular sequelae, and B57.3 corresponding to chronic digestive sequelae.

Chagas disease

Quantity of interest	Reference or alternative	Definition
Chagas disease	Reference	Prevalence determined through diagnosis of acute and chronic infections with the protozoa Trypanosoma cruzi. It includes all ICD-10 codes under the heading B57 (Chagas disease).

Input data

Model inputs

Table 1: Source Counts

Measure	Total sources	Countries with data
All measures	84	21
Prevalence	81	20
Proportion	3	1
Population	1	1

For GBD 2021 estimation, we used seroprevalence data to model Chagas prevalence. We used a MR-BRT (meta-regression—Bayesian, regularised, trimmed) model with our sex-specific data to derive an estimate of the ratio of the male prevalence of Chagas disease to female prevalence of Chagas disease to split non-sex-specific data. Then, a DisMod-MR 2.1 Bayesian meta-regression model using the age-specific input data was run to derive an age pattern to apply to split the all-age data.

Table 2: MR-BRT crosswalk adjustment factors for Chagas disease

Data input	Reference or alternative case definition	Gamma	Beta Coefficient, Log (95% CI)*	Adjustment factor**
Female data	Ref	0.37	---	---
Male data	Alt		0.07 (-0.65, 0.79)	1.07

**MR-BRT crosswalk adjustments can be interpreted as the factor the alternative case definition is adjusted by to reflect what it would have been had it been measured using the reference case definition. If the log/logit beta coefficient is negative, then the alternative is adjusted up to the reference. If the log/logit beta coefficient is positive, then the alternative is adjusted down to the reference.*

***The adjustment factor column is the exponentiated beta coefficient. For log beta coefficients, this is the relative rate between the two case definitions. For logit beta coefficients, this is the relative odds between the two case definitions.*

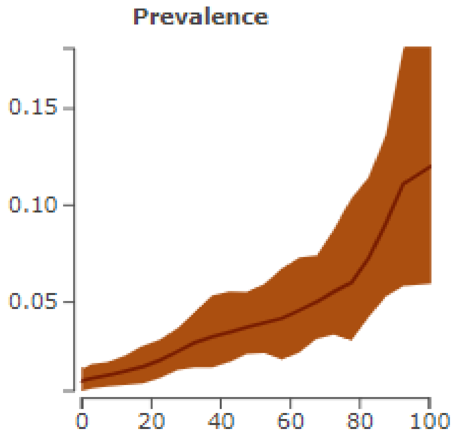

Figure 1: Latin America-specific age pattern for Chagas disease used to split all-age data into age-specific datapoints for further modelling.

We also use CSMR estimates in the modelling process, which will be addressed in further detail below.

Modelling strategy

We modelled Chagas disease using a full DisMod-MR 2.1 Bayesian meta-regression model incorporating seroprevalence data, as above, and CSMR estimates. We assume no remission. We eliminate all new infections, except those via vertical transmission, in Chile and Uruguay for years after the interruption of vector-based transmission (Abad-Franch F, Diotaiuti L, Gurgel-Gonçalves R, Gürtler RE. Certifying the interruption of Chagas disease transmission by native vectors: cui bono? Mem Inst Oswaldo Cruz 2013;108:251–4.; Coura JR. Chagas disease: control, elimination and eradication. Is it possible? Mem Inst Oswaldo Cruz 2013;108:962–7.). We then adjust these estimates for population at-risk as estimated by the Pan-American Health Organization in 2005 (Pan-American Health Organization (PAHO), World Health Organization (WHO). Quantitative Estimation of Chagas in the Americas). For non-endemic countries, we estimate the prevalence of imported chronic infections based on migration. For each non-endemic country, we estimate the total number of people infected with Chagas as the sum of the number of immigrants from each endemic country multiplied by the corresponding prevalence of Chagas in that endemic country.

We estimate five sequelae: symptomatic acute infection from incidence; and megaviscera, heart failure, atrial fibrillation, and chronic asymptomatic infection from prevalence. We assume that 5% of acute infections will be symptomatic (Teixeira AR, Nitz N, Guimaro MC, Gomes C, Santos-Buch CA. Chagas disease. Postgrad Med J 2006;82:788–98.). The proportion of chronic infections resulting in a given sequela varies by sex and age: the prevalence of megaviscera among those infected with Chagas ranges from 0% in children to nearly 10% among older adults (Coura JR, Naranjo MA, Willcox HP. Chagas' disease in the Brazilian Amazon: II. A serological survey. Rev Inst Med Trop São Paulo 1995; 37:103–7.); the prevalence of atrial fibrillation attributable to Chagas ranges from 0% among children to approximately 10% in men over 80 years of age (Ribeiro AL, Marcolino MS, Prineas RJ, Lima-Costa MF. Electrocardiographic abnormalities in elderly Chagas disease patients: 10-year follow-up of the Bambuí Cohort Study of Aging. J Am Heart Assoc 2014;3:e000632.); and the prevalence of heart failure attributable to Chagas among those who are infected ranges from 0% among young children, to a

maximum of 23% among men over 80 years of age (Sabino EC, Ribeiro AL, Salemi VM, et al., for the

National Heart, Lung, and Blood Institute Retrovirus Epidemiology Donor Study-II (REDS-II), International Component. Ten-year incidence of Chagas cardiomyopathy among asymptomatic Trypanosoma cruzi-seropositive former blood donors. Circulation 2013;127:1105–15.).

Severity splits and disability weights

The table below illustrates the sequelae, lay descriptions, and disability weights (DWs) for Chagas disease.

Table 3. Sequelae, lay description and DWs

Sequelae	Description	Disability weight
Atrial fibrillation and flutter due to Chagas disease	Has periods of rapid and irregular heartbeats and occasional fainting.	0.224 (0.151–0.312)
Mild heart failure due to Chagas disease	Is short of breath and easily tires with moderate physical activity, such as walking uphill or more than a quarter-mile on level ground. The person feels comfortable at rest or during activities requiring less effort.	0.041 (0.026–0.062)
Moderate heart failure due to Chagas disease	Is short of breath and easily tires with minimal physical activity, such as walking only a short distance. The person feels comfortable at rest but avoids moderate activity.	0.072 (0.047–0.103)
Severe heart failure due to Chagas disease	Is short of breath and feels tired when at rest. The person avoids any physical activity, for fear of worsening the breathing problems.	0.179 (0.122–0.251)
Mild chronic digestive disease due to Chagas disease	Has some pain in the belly that causes nausea but does not interfere with daily activities.	0.011 (0.005–0.021)
Moderate chronic digestive disease due to Chagas disease	Has pain in the belly and feels nauseated. The person has difficulties with daily activities.	0.114 (0.078–0.159)
Acute Chagas disease	Has a fever and aches, and feels weak, which causes some difficulty with daily activities.	0.051 (0.032–0.074)
Asymptomatic Chagas disease	Latent Chagas infection (ie, chronic infection with no apparent symptoms)	NA

Changes from GBD 2019 to GBD 2021

We have made no substantive changes in the modelling strategy from GBD 2019.

We did not apply any adjustments for the COVID pandemic to Chagas disease due to a lack of available data quantifying the impacts of the pandemic on NTD epidemiology.

Chronic kidney disease

Flowchart

Chronic Kidney Disease (CKD)

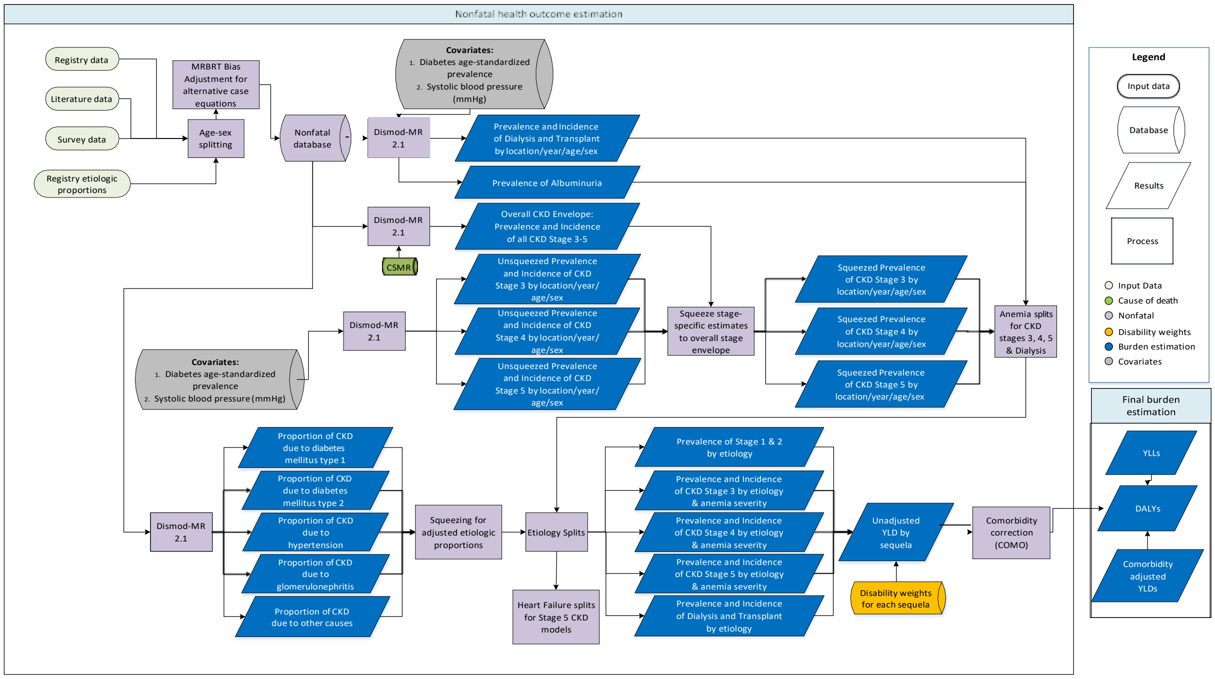

Case definition

Chronic kidney disease (CKD) is defined as a permanent loss of kidney function as indicated by estimated glomerular filtration rate (eGFR) and urinary albumin to creatinine ratio (ACR). The CKD-Epi eGFR equation is considered our gold standard for those 18 years or older and the Schwartz equation is our gold standard for those younger than 18. These equations can be found in Table 1. The GBD study considers six stages of CKD as defined by degree of loss of kidney function or receipt of kidney replacement therapy. These definitions of the six stages can be found in Table 2. The ICD-10 codes associated with CKD include N18.1-N18.9. Moreover, the clinical case definition for CKD is the following: A chronic, progressive condition of the kidney, lasting 3 months or more, with a loss in its key function to filtrate blood to produce urine.

Table 1. CKD eGFR Equations

Equation	Formula
CKD-EPI 2009	eGFR = $141 \times \min(S_{Cr}/\kappa, 1)^{\alpha} \times \max(S_{Cr}/\kappa, 1)^{-1.209} \times 0.993^{Age} \times 1.018$ [if female] $\times 1.159$ [if Black] κ is 0.7 for females and 0.9 for males α is -0.329 for females and -0.411 for males, where min indicates the minimum of Scr/κ or 1, and max indicates the maximum of Scr/κ or 1

Schwartz	eGFR = 0.413 x (height/Scr) if height is expressed in centimetres<br>OR<br>41.3 x (height/Scr) if height is expressed in metres

Table 2. GBD Case Definitions of CKD

Quantity of interest	Measure	Reference or alternative	Definition
Stages 1&2 chronic kidney disease	Prevalence	Reference	Albumin to creatinine ratio (ACR) of >=30 mg/g and estimated glomerular filtration rate (eGFR) > 60 mL/min/1.73m2 as estimated using the CKD-EPI equation for individuals age >18 and the Schwartz equation for those <18.
Stage 3 chronic kidney disease	Prevalence	Reference	Estimated glomerular filtration rate (eGFR) 30-60 mL/min/1.73m2 as estimated using the CKD-EPI equation for individuals age >18 and the Schwartz equation for those <18 not on renal replacement therapy.
Stage 3 chronic kidney disease	Prevalence	Alternative	Estimated glomerular filtration rate (eGFR) 30-60 mL/min/1.73m2 as estimated using the MDRD equation (or modifications thereof) for individuals age >18 not on renal replacement therapy.
Stage 3 chronic kidney disease	Prevalence	Alternative	Estimated glomerular filtration rate (eGFR) 30-60 mL/min/1.73m2 as estimated using the Cockcroft-Gault equation (standardised for body surface area) for individuals age >18 not on renal replacement therapy.
Stage 4 chronic kidney disease	Prevalence	Reference	Estimated glomerular filtration rate (eGFR) 15-30 mL/min/1.73m2 as estimated using the CKD-EPI equation for individuals age >18 and the Schwartz equation for those <18 not on renal replacement therapy.
Stage 4 chronic kidney disease	Prevalence	Alternative	Estimated glomerular filtration rate (eGFR) 15-30 mL/min/1.73m2 as estimated using the MDRD equation (or modifications thereof) for individuals age >18 not on renal replacement therapy.
Stage 4 chronic kidney disease	Prevalence	Alternative	Estimated glomerular filtration rate (eGFR) 15-30 mL/min/1.73m2 as estimated using the Cockcroft-Gault equation (standardised for body surface area) for individuals age >18 not on renal replacement therapy.
Stage 5 chronic kidney disease	Prevalence	Reference	Estimated glomerular filtration rate (eGFR) <15 mL/min/1.73m2 as estimated using the CKD-EPI equation for individuals age >18 and the Schwartz equation for those <18 not on renal replacement therapy.
Stage 5 chronic kidney disease	Prevalence	Alternative	Estimated glomerular filtration rate (eGFR) <15 mL/min/1.73m2 as estimated using the MDRD equation (or modifications thereof) for individuals age >18 not on renal replacement therapy.
Stage 5 chronic kidney disease	Prevalence	Alternative	Estimated glomerular filtration rate (eGFR) <15 mL/min/1.73m2 as estimated using the Cockcroft-Gault equation (standardised for body surface area) for individuals age >18 not on renal replacement therapy.
End-stage renal disease after transplant	Incidence	Reference	Received a kidney transplant due to end-stage renal disease. Includes all kidney transplants due to ESRD, not just preemptive transplants.

End-stage renal disease after transplant	Prevalence	Reference	Ever received kidney transplant due to end-stage renal disease. Includes all kidney transplants due to ESRD, not just preemptive transplant.
End-stage renal disease on dialysis	Incidence	Reference	A new case of dialysis (haemodialysis or peritoneal dialysis) treatment for a period > 90 days.
End-stage renal disease on dialysis	Prevalence	Reference	On dialysis (haemodialysis or peritoneal dialysis) for > 90 days.

Input data

Model inputs

Throughout GBD 2021, we opportunistically updated scientific literature extractions from the Global Health Data Exchange (GHDx). Literature extractions were augmented by identification of population-based surveys that measured kidney function. Additionally, the following listed below are new data that were added this round:

1. We re-extracted ERA-EDTA from 1998-2017. We did this because in GBD 2019, aetiology models only had ERA-EDTA for 1998, 2000, 2003, 2005, 2008, 2010, 2012, 2013 and the maintenance dialysis and transplant models only had "Both" sex ERA-EDTA data for 1998-2015. The re-extractions were only added to the models that were missing data for that location, year and measure. Otherwise, in general, for maintenance dialysis and kidney transplantation data are largely obtained from kidney registry reports.
2. We added back in the China National Health Survey data that was excluded in GBD 2019. The survey was originally excluded due to small sample sizes but after an evaluation of our current methods, we decided that once we aggregated the age groups to 20-year bins the age pattern was reasonable and usable.

The exclusion criteria for extraction are:

1. Studies clearly not representative of the national population
2. Studies that did not provide primary data on epidemiological parameters, eg, a commentary piece.
3. Studies of a specific aetiology of CKD only to avoid bias in studies selectively reporting a single aetiology.

For an additional breakdown of the data inputs by parameters and aetiology, see Tables 3a and 3b.

Table 3a. Data Inputs for CKD morbidity modelling by parameter.

Parameter	Countries with data	New sources	Total sources
Incidence	90	221	1400
Prevalence	120	228	1490
Excess Mortality Rate	13	11	45
With-condition mortality rate	3	0	3

Table 3b. Data Inputs by CKD aetiologies morbidity modelling.

Aetiology model	Parameter	Countries with data	New sources	Total sources
CKD due to Hypertension	Proportion	60	35	389
CKD due to diabetes mellitus type 1	Proportion	43	178	218
CKD due to diabetes mellitus type 2	Proportion	43	178	218
CKD due to glomerulonephritis	Proportion	60	39	392
CKD due to Other	Proportion	60	39	393

Data processing

Age-sex and sex split

In order to obtain an appropriate age -pattern with which to age-split input data, we first ran a disease model—Bayesian meta-regression[1] (DisMod-MR 2.1) for all datapoints with an age range less than 30 years. In GBD 2019, the estimated age pattern was created using all data with an age range less than 50. We changed this age range to less than 30 for GBD 20210 because, for example, a 40-year range is uninformative for the overall age pattern. However, after re-evaluation, we determined that since DisMod-MR 2.1 depends on rates and not case numbers, these less than one case numbers can be handled reasonably within DisMod-MR 2.1. Thus, we removed the data restriction and allowed these datapoints to be age split.

We then used age -pattern by super-region to age-split dialysis and transplant input data, thereby allowing for variation in the age -pattern by location. After age-splitting, we ran a model on all processed data, including age-split data and age-specific data, to obtain final estimates of dialysis incidence and prevalence by location, year, age, and sex. For dialysis, remission data for dialysis were calculated as the ratio of the incidence of kidney transplantation to prevalence of dialysis at the gender-, age-, and country-matched level. A similar process occurs for Stage 3 and Stage 4 remission where, remission data for the respective stages are calculated as the ratio of the incidence of the later stage to the prevalence of the stage of interest at the gender-, age-, and country-matched level. For the other stage models (Stage 1-2, Stage 3, Stage 4 and Stage 5), these models' age splits and age patterns are determined by DisMod-MR 2.1.

In some cases, data are reported by only age or only sex, but not both. For example, a study may have included the proportion of males and females with stage 3 CKD and then separately reported the proportion of both sexes by smaller age bins (e.g. 40 – 44, 45-–49) that have stage 3 CKD. In these cases, we perform an age-sex split by utilizing proportions within the study to disaggregate the data.
For our models with more data (CKD Stage 3-5, CKD Stage 3, CKD Stage 4, Dialysis and Transplant) we applied a sex split by super-region. For our models that are data sparse (CKD Stage 1–-2 and Stage 5), we perform a sex -split on the data by applying a global sex proportions instead of super-region-specific split.

Excess mortality data
The EMR strategy from GBD 2019 utilized cause specific mortality rate (CSMR) along with prevalence to inform the DisMod-MR 2.1 EMR estimates. This method assumes that CSMR represents excess deaths due to a cause. So, for CKD, CSMR captures deaths due to CKD as the primary cause of death. Upon further examination, since CKD increases the risk of death due to many comorbidities (heart disease, diabetes, etc.), the CSMR does not accurately represent all excess deaths due to CKD. Thus, we removed this modelled EMR approach in GBD 2021 and reverted to using DisMod MR 2.1 estimated excess mortality. Overall, this led to lower prevalence and incidence curves.

Bias adjustments
We have made no substantive changes in the bias adjustment strategy from GBD 2019 for GBD 2021. We utilised a meta-regression—Bayesian, regularised, trimmed model[1] (MR-BRT) outside of DisMod-MR 2.1 to directly compare the differences between different case definitions and/or study designs.

Glomerular filtration rate (GFR) can be estimated using a variety of equations that lead to different prevalence estimates. Our CKD reference equation is the CKD-Epi Creatinine equation. We also included data estimated with the Modification of Diet in Renal Disease (MDRD) and the Cockcroft-Gault (CG) equation. For those under the age 18, the Schwartz equation was used as the reference. We adjusted data using MDRD and CG equations through a MR-BRT model to account for different estimates that result from these different equations. The adjustment is a logit-transformation method in MR-BRT. The general process is described below:

1. Identify datapoints with overlapping year, age, sex, and location between reference and alternative definitions.
2. Logit transforms overlapping datapoints of alternative and reference case definitions.
3. Convert overlapping datapoints into a difference in logit space using the following equation:

$$logit(alternative) - logit(reference)$$

4. Use the delta method to compute standard errors of overlapping datapoints in logit space, then calculate standard error of logit difference using the following equation:

$$\sqrt{(variance\ of\ alternative) + (variance\ of\ \ reference)}$$

5. Using MR-BRT, conduct a random effects meta-regression to obtain the pooled logit difference of alternative to reference.
6. Apply the pooled logit difference to all datapoints of alternative case definitions using the following equation:

$$newestimate = inverse.logit(logit(alternative) - (pooled\ logit\ difference))$$

7. Calculate new standard errors using the delta method, accounting for gamma (between-study heterogeneity)

Table 4 shows the adjustment factors used to adjust the data:

Table 4. MR-BRT Crosswalk Adjustment Factors for CKD

Data input	Reference or alternative case definition	*Gamma*	*Beta Coefficient, Logit (95% CI) **

Stage 3, Stage 4, Stge 5, Stage 3-5 CKD-EPI	Ref	---	---
Stage 3 CG	Alt	*0.25*	0.24 (-0.28 - 0.76)
Stage 3 MDRD	Alt	*0.03*	0.49 (0.34 - 0.64)
Stage 4 CG	Alt	*0*	0.09 (-0.05 - 0.24)
Stage 4 MDRD	Alt	*0*	-0.07 (-0.19 - 0.04)
Stage 5 CG	Alt	*0*	-0.18 (-0.45 - 0.09)
Stage 5 MDRD	Alt	*0*	-0.06 (-0.28 - 0.18)
Stage 3-5 CG	Alt	*0.26*	0.23 (-0.29 - 0.75)
Stage 3-5 MDRD	Alt	*0.03*	0.47 (0.32- 0.62)

**MR-BRT crosswalk adjustments can be interpreted as the factor the alternative case definition is adjusted by to reflect what it would have been had it been measured using the reference case definition. If the log/logit beta coefficient is negative, then the alternative is adjusted up to the reference. If the log/logit beta coefficient is positive, then the alternative is adjusted down to the reference.*

Modelling strategy

CKD stage models
The modelling strategy for GBD 2021 is quite like GBD 2019. Most of our updates from the previous round are described above under *Data processing: age-sex and sex split*.

In general, we run separate DisMod-MR 2.1 models for each stage of CKD and an aggregate CKD stage 3-5 model to produce estimates by age, sex, year, and country. The stage 3-5 model is used as an envelope to ensure that the stage level models are consistent. CKD stage models 3, 4 and 5 were then rescaled to the aggregate stage 3-5 model for every age, sex, year, and country.

Progression of CKD
Because DisMod-MR 2.1 does not incorporate disease progression in its compartmental model, we used "remission" as a proxy for progression, where a surviving prevalent case ceases to be a case in this stage. As CKD is a progressive disease, we assume there is no true remission, which allows us to apply this parameter substitution. To account for the progression of individuals from stage 3 to 4 and from 4 to 5, we back-calculated progression to later stages of CKD. This was done by calculating the ratio of the incidence of the next stage with the prevalence of the previous stage. For inclusion in DisMod-MR 2.1 models, these custom input data were calculated as:

$$remission_s = \frac{incidence_{s+1}}{prevalence_s}, where\ s\ is\ stage$$

Furthermore, remission was set to 0 for Stage 5 and the excess mortality parameter was used to account for progression to end-stage kidney disease and mortality due to CKD Stage 5 collectively (even though

‘technically’ this is not correct for those who go onto dialysis, this was a decision to facilitate

modelling). Bounds on excess mortality were informed using a meta-analysis of survival analyses[2] of individuals with untreated CKD Stage 5.

CKD covariate selection
Based on collaborator feedback and our understanding of the epidemiology of CKD, the following covariates in Table 5 were selected for each stage model.

Table 5. Covariates. Summary of covariates used in the CKD DisMod-MR 2.1

Model	Covariate	Type	Parameter	Exponentiated beta (95% Uncertainty Interval)
CKD Stage 3-5	Diabetes Age-Standardised Prevalence (proportion)	Country-level	Prevalence	1.22 (1.01 — 1.76)
	Systolic Blood Pressure (mm Hg)	Country-level	Prevalence	1.07 (1.00 — 1.20)
	Healthcare Access and Quality Index	Country-level	Excess mortality rate	1.01 (1.01 — 1.01)
CKD Stage 3	Diabetes Age-Standardised Prevalence (proportion)	Country-level	Prevalence	1.03 (1.00 — 1.08)
	Systolic Blood Pressure (mm Hg)	Country-level	Prevalence	1.14 (1.01 — 1.58)
CKD Stage 4	Diabetes Age-Standardised Prevalence (proportion)	Country-level	Prevalence	1.19 (1.01 — 1.62)
	Systolic Blood Pressure (mm Hg)	Country-level	Prevalence	2.30 (1.76 — 2.69)
CKD Stage 5	Diabetes Age-Standardised Prevalence (proportion)	Country-level	Prevalence	2.06 (1.82 — 2.32)
	Systolic Blood Pressure (mm Hg)	Country-level	Prevalence	4.08 (3.39 — 4.46)
Dialysis	Healthcare access and quality index	Country-level	Incidence hazard	1.01 (1.01 — 1.02)
Transplant	Healthcare access and quality index	Country-level	Incidence hazard	1.01 (1.01 — 1.01)

CKD aetiology proportion models

To model aetiology proportions of CKD, we utilised two separate types of data.

The first are data from end-stage kidney registries used to estimate the proportion of each aetiology for those on dialysis or with kidney transplants. The results from all five aetiology-specific models were adjusted so that estimates across the etiologies sum to 100%. These adjusted proportions were then applied to the DisMod-MR 2.1 models for end-stage renal disease dialysis and transplant to obtain estimates of each of the aetiologies by location, year, age, and sex.

The second data comes from the Geisinger Health System in Pennsylvania[3]. These data contain age-sex-stage-specific aetiology proportions that allowed differential etiologic composition of CKD stages (stages 1&2, 3, 4, and 5). For everyone with CKD, we scanned their history of recorded International Classification of Diseases (ICD) codes to identify ICD codes for primary kidney diseases (See Table 6). Individuals with CKD but with no history of a primary kidney disease ICD code were classified as having CKD of unknown aetiology. We ran a multinomial logistic regression including sex and a non-linear term

for age to predict the probability of each aetiology by age and sex for each stage of CKD (1/2, 3, and 4/5

combined). For each stage, aetiology, age, and sex, we converted this probability into the proportion of CKD due to the given aetiology and applied these proportions to the prevalence of CKD for the same stage, age, and sex category to estimate the prevalence of each stage of CKD by aetiology, age, and sex.

Table 6. International Classification of Disease Codes Used for GBD aetiology mapping, list of the ICD codes used to identify the CKD aetiology attribution.

CKD Aetiology	ICD 9 Codes	ICD 10 Codes
Type 1 diabetes	250.41, 250.43	E10.2, E10.21, E10.22, E10.29
Type 2 diabetes	250.40, 250.42	E11.2, E11.21, E11.22, E11.29
Glomerulonephritis	581, 581.0, 581.1, 581.2, 581.3, 581.8, 581.81, 581.89, 581.9, 582, 582.0, 582.1, 582.2, 582.4, 582.8, 582.81, 582.89, 582.9, 583, 583.0, 583.1, 583.2, 583.4, 583.6, 583.7, 583.8, 583.81, 583.89, 583.9	N02, N02.0, N02.1, N02.2, N02.3, N02.4, N02.5, N02.6, N02.7, N02.8, N02.9, N03, N03.0, N03.1, N03.2, N03.3, N03.4, N03.5, N03.6, N03.7, N03.8, N03.9, N04, N04.0, N04.1, N04.2, N04.3, N04.4, N04.5, N04.6, N04.7, N04.8, N04.9, N05, N05.0, N05.1, N05.2, N05.3, N05.4, N05.5, N05.6, N05.7, N05.8, N05.9, N06, N06.0, N06.1, N06.2, N06.3, N06.4, N06.5, N06.6, N06.7, N06.8, N06.9
Hypertension	403, 403.0, 403.00, 403.01, 403.1, 403.10, 403.11, 403.6, 403.9, 403.90, 403.91, 404, 404.0, 404.00, 404.01, 404.02, 404.03, 404.1, 404.10, 404.11, 404.12, 404.13, 404.9, 404.90, 404.91, 404.92, 404.93	I12, I12.0, I12.1, I12.2, I12.9, I13, I13.0, I13.1, I13.10, I13.11, I13.2, I13.9
Other	589, 589.0, 589.1, 589.9, 753.0, 753.1, 753.10, 753.11, 753.12, 753.13, 753.14, 753.15, 753.16, 753.17, 753.19, 753.2, 753.20, 753.21, 753.22, 753.23, 753.29, 753.3, 283.11, 710.0, 753.0, 753.21, 753.22, 753.29	N07, N07.0, N07.1, N07.2, N07.3, N07.4, N07.5, N07.6, N07.7, N07.8, N07.9, N08, N08.0, N08.1, N08.2, N08.3, N08.4, N08.5, N08.8, N15.0, Q61, Q61.0, Q61.00, Q61.01, Q61.02, Q61.1, Q61.11, Q61.19, Q61.2, Q61.3, Q61.4, Q61.5, Q61.8, Q61.9, Q62, Q62.0, Q62.1, Q62.10, Q62.11, Q62.12, Q62.2, Q62.3, Q62.31, Q62.32, Q62.39, Q62.4, Q62.5, Q62.6, Q62.60, Q62.61, Q62.62, Q62.63, Q62.69, Q62.7, Q62.8, D59.3, M31.31, M32.14, M32.15, N11.9, N13.70, N13.8, Q60.2, Q63.8, N14.0, N14.1, N14.3, N25.89, N26.9, N28.0

CKD diabetes corrections and adjustments for aetiology models
In order to make use of all available data, we modelled the proportion of CKD due to overall DM, DM type 1, and DM type 2. Proportion of CKD due to DM type 1 and DM type 2 were then scaled to sum to the proportion of overall DM at the gender, age, and country-matched level.

To maintain consistency between GBD estimates of type 1 diabetes prevalence estimates and CKD due to

type 1 diabetes prevalence estimates and generalise the results of the Geisinger analysis to all locations,

we performed a location-specific correction for the proportion of CKD due to type 1 and type 2 diabetes. Type 1 diabetes makes up a larger proportion of total diabetes in the United States than it does in other locations. For each diabetic subtype (e) for a given location (l), age (a), and sex (g) the ratio of subtype-specific diabetes prevalence to total diabetes prevalence (r) was calculated as:

$$r_{e,l,a,g} = \frac{prevalence_{e,l,a,g}}{prevalence_{dm1,l,a,g} + prevalence_{dm2,l,a,g}}$$

This ratio is used to adjust the proportion of CKD due to a given diabetic subtype (p) for a given CKD stage (s), l, a, and g by scaling the predicted proportion of CKD due to that subtype (k) by the ratio of total DM due to e in l to the ratio of total DM due to e in the United States (USA).

$$p_{s,e,l,a,g} = k_{\ s,a,g} \times \frac{r_{e,l,a,s}}{r_{e,USA,a,s}}$$

The stage-specific approach utilised to estimate the prevalence of CKD stages is limited using data from a single geographic region.

Anaemia causal attribution

The age- and sex-specific anaemia prevalence for CKD was analysed as part of overall anaemia causal attribution for GBD 2021. The details of the anaemia analysis are described separately in the "Anaemia Impairment" section. Briefly, after estimating total anaemia, a series of counterfactual distributions were generated based on the age- and sex-specific prevalence of each anaemia-causing condition and the quantitative effect that the condition has on haemoglobin concentration in the blood, a so-called "haemoglobin shift," that was derived by meta-analysing cohort studies, observational studies, or trials comparing the haemotologic status of those with as compared to without the disease. Due to limited data on haemoglobin shift, all were assumed to be invariant over age, sex, location, and year.

Severity splits and disability weights

Estimates of prevalence and incidence are split using CKD aetiology proportion models, resulting in CKD estimates by stage and aetiology. Then a portion of each aetiology split for CKD stages 3, 4, and 5 is attributed a disability weight associated with mild, moderate, or severe anaemia. For GBD 2021, each aetiology split for Stage 5 is attributed a disability weight associated with mild, moderate, or severe heart failure.

Table 7. Severity distribution, details on the severity levels for CKD and the associated disability weight (DW) with that severity.

Severity level	Lay description	DW (95% CI)
Albuminuria	Asymptomatic	--
CKD stage 3 without anaemia	Asymptomatic	--
CKD stage 3 with mild anaemia	Feels slightly tired and weak at times, but this does not interfere with normal daily activities.	0.004 (0.001–0.008)
CKD stage 3 with moderate anaemia	Feels moderate fatigue, weakness, and shortness of breath after exercise, making daily activities more difficult.	0.052 (0.034–0.076)
CKD stage 3 with severe anaemia	Feels very weak, tired, and short of breath, and has problems with activities that require physical effort or deep concentration.	0.149 (0.101–0.21)

CKD stage 4 without anaemia	Tires easily, has nausea, reduced appetite, and difficulty sleeping.	0.104 (0.07–0.147)
CKD stage 4 with mild anaemia	Combined disability weight	0.108 (0.072–0.151)
CKD stage 4 with moderate anaemia	Combined disability weight	0.15 (0.103–0.207)
CKD stage 4 with severe anaemia	Combined disability weight	0.237 (0.165–0.324)
CKD stage 5 without anaemia	Has lost a lot of weight and has constant pain. The person has no appetite, feels nauseated, and needs to spend most of the day in bed.	0.569 (0.389–0.727)
CKD stage 5 with mild anaemia	Combined disability weight	0.570 (0.391–0.727)
CKD stage 5 with moderate anaemia	Combined disability weight	0.591 (0.414–0.743)
CKD stage 5 with severe anaemia	Combined disability weight	0.631 (0.456–0.782)
End-stage kidney disease, on dialysis	Is tired and has itching, cramps, headache, joint pains, and shortness of breath. The person needs intensive medical care every other day lasting about half a day.	0.571 (0.397–0.725)
End-stage renal disease, on dialysis and mild anemia	Combined disability weight	0.573 (0.403-0.726)
End-stage renal disease, on dialysis and moderate anemia	Combined disability weight	0.593 (0.424-0.742)
End-stage renal disease, on dialysis and severe anemia	Combined disability weight	0.633 (0.462-0.781)
End-stage kidney disease, with kidney transplant	Sometimes feels tired and down, and has some difficulty with daily activities.	0.024 (0.014–0.039)
Stage 5 due to type 1 diabetes mellitus, with asymptomatic heart failure	Has lost a lot of weight and has constant pain. The person has no appetite, feels nauseous, and needs to spend most of the day in bed.	0.148 (0.100-0.205)
Stage 5 due to type 1 diabetes mellitus, with mild heart failure	Combined disability weight	0.141 (0.097-0.195)
Stage 5 due to type 1 diabetes mellitus, with moderate heart failure	Combined disability weight	0.168 (0.115-0.230)
Stage 5 due to type 1 diabetes mellitus, with severe heart failure	Combined disability weight	0.264 (0.186-0.358)
Stage 5 due to type 2 diabetes mellitus, with asymptomatic heart failure	Has lost a lot of weight and has constant pain. The person has no appetite, feels nauseous, and needs to spend most of the day in bed.	0.148 (0.100-0.205)
Stage 5 due to type 2 diabetes mellitus, with mild heart failure	Combined disability weight	0.141 (0.097-0.195)
Stage 5 due to type 2 diabetes mellitus, with moderate heart failure	Combined disability weight	0.168 (0.115-0.230)

Stage 5 due to type 2 diabetes mellitus, with severe heart failure	Combined disability weight	0.264 (0.186-0.358)
Stage 5 due to hypertension, with asymptomatic heart failure	Has lost a lot of weight and has constant pain. The person has no appetite, feels nauseous, and needs to spend most of the day in bed.	0.148 (0.1-0.205)
Stage 5 due to hypertension, with mild heart failure	Combined disability weight	0.141 (0.097-0.195)
Stage 5 due to hypertension, with moderate heart failure	Combined disability weight	0.168 (0.115-0.230)
Stage 5 due to hypertension, with severe heart failure	Combined disability weight	0.264 (0.186-0.358)
Stage 5 due to glomerulonephritis, with asymptomatic heart failure	Combined disability weight	0.148 (0.1-0.205)
Stage 5 due to glomerulonephritis, with mild heart failure	Combined disability weight	0.141 (0.097-0.195)
Stage 5 due to glomerulonephritis, with moderate heart failure	Combined disability weight	0.168 (0.115-0.230)
Stage 5 due to glomerulonephritis, with severe heart failure	Combined disability weight	0.264 (0.186-0.358)
Stage 5 due to other and unspecified causes, with asymptomatic heart failure	Has lost a lot of weight and has constant pain. The person has no appetite, feels nauseous, and needs to spend most of the day in bed.	0.148 (0.100-0.205)
Stage 5 due to other and unspecified causes, with mild heart failure	Combined disability weight	0.141 (0.097-0.195)
Stage 5 due to other and unspecified causes, with moderate heart failure	Combined disability weight	0.168 (0.115-0.230)
Stage 5 due to other and unspecified causes, with severe heart failure	Combined disability weight	0.264 (0.186-0.358)

Note: the DWs for CKD 4 and 5 stages with anaemia are derived from a multiplicative function combining the CKD stage DW and the corresponding severity of anaemia DW

Citations

1. Vos T, Lim SS, Abbafati C, *et al.* Global burden of 369 diseases and injuries in 204 countries and territories, 1990–2019: a systematic analysis for the Global Burden of Disease Study 2019. *The Lancet* 2020; 396: 1204–22. doi: https://doi.org/10.1016/S0140-6736(20)30925-9 . Details found in appendix 1, section 4.4.1 and 4.5

2. Murtagh FEM, Marsh JE, Donohoe P, Ekbal NJ, Sheerin NS, Harris FE. Dialysis or not? A comparative survival study of patients over 75 years with chronic kidney disease stage 5. *Nephrol Dial Transplant*. 2007;

22(7): 1955–62.

3. Chang AR, Kirchner HL, Sang Y, Grams ME, Coresh J, Geisinger Health System, Johns Hopkins University (United States), Chronic Kidney Disease Prognosis Consortium (CKD-PC). United States- Pennsylvania Geisinger Health System Chronic Kidney Disease Etiology Proportions 1997-2017, Analyzed by CKD-PC. [Unpublished.]

Chronic obstructive pulmonary disease (COPD)

Flowchart

Chronic Obstructive Pulmonary Disease (COPD)

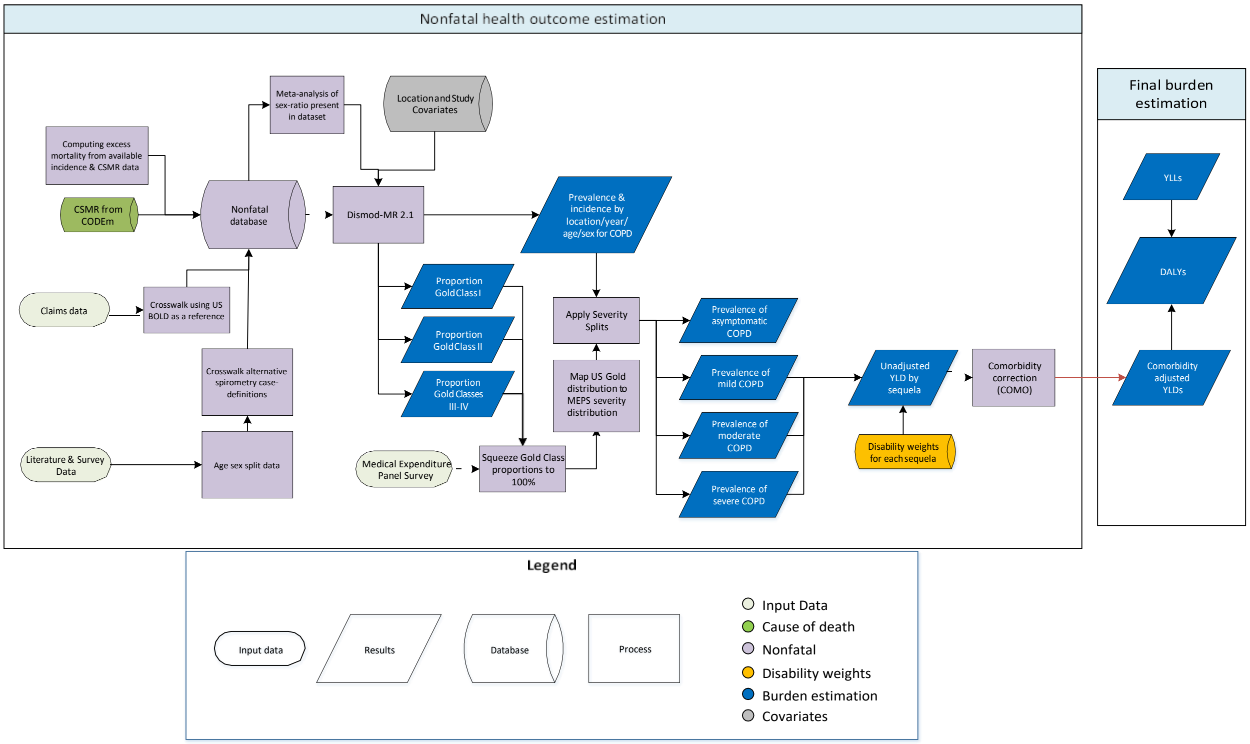

Clinical definition

Chronic inflammatory lung disease that causes obstructed airflow and breathing problems. It includes emphysema and chronic bronchitis.

Case definition

COPD is defined as in the Global Initiative for Chronic Obstructive Lung Disease (GOLD) classification: a measurement of <0.7 FEV_1/FVC (one second of forceful exhalation/total forced expiration) on spirometry after bronchodilation. The severity grading of COPD follows this GOLD class definition.

GOLD CLASS	FEV_1 Score
I: Mild	>=80% of normal
II: Moderate	50-79% of normal
IV: Severe	<50% of normal

ICD-10 codes associated with COPD include J41, J42, J43, J44, and J47. The corresponding ICD-9 codes are

491-492, and 496. J40 & 490 (Bronchitis, not specified as acute or chronic) and J47 & 494 (Bronchiectasis) were removed from COPD mapping in GBD 2017.

Alternative case definitions that differ from the GOLD post-bronchodilation definition are as follows: GOLD pre-bronchodilation, lower limit of normal (LLN) post-bronchodilation, LLN pre-bronchodilation, and European Respiratory Society (ERS) guidelines. These are all different methods of evaluating whether an individual has COPD.

Input data

Last systematic review was completed for GBD 2017, we updated the systematic review from previous iterations. The full search term was:

(chronic obstructive pulmonary disease[Title/Abstract] AND (prevalence[Title/Abstract] OR incidence[Title/Abstract] OR mortality[Title/Abstract] OR death[Title/Abstract]) AND "Cross-Sectional Studies"[MeSH Terms]) AND (("2016/07/01"[PDAT] : "2017/09/08"[PDAT]) AND "humans"[MeSH Terms])

COPD has the following data sources

- Prevalence, incidence, and remission data from literature
- Hospital claims data
- Proportion data of GOLD class severities
- Burden of Obstructive Lung Disease (BOLD) Study data

Prevalence, incidence, and remission data relating to COPD are extracted from literature provided by collaborators or found with a systematic review. All data include spirometry-based measures. Other data come from hospital claims data for non-fatal estimation and vital registrations for cause of death.

GOLD class proportions are extracted from literature when the severity is available. Our models estimate three separate severities:

- Mild COPD: GOLD class I
- Moderate COPD: Gold class II
- Severe COPD: Gold class III & IV

These severities are used in the modelling process to split COPD by severities.

The Burden of Obstructive Lung Disease (BOLD) data are specifically notable because of their use in bias adjustments described in the data processing section.

While no systematic review of the literature was completed for GBD 2021, additional data were included from key relevant survey series, GBD collaborators and an opportunistic search using previous systematic review search string in Pubmed was conducted.

New data added this round include the English Longitudinal Study of Aging (ELSA), Korea National Health and Nutrition Examination Survey, BOLD related publications, and several sources from scientific literature.

Claims data, specifically from the United States, have been included since GBD 2019. Additional information on the claims data collection and pre-corrections are provided elsewhere. Briefly, we determined USA national and state-level estimates of COPD prevalence from a database of individual-level ICD-coded health service encounters. Persons with any inpatient claim or at least two outpatient claims associated with COPD were marked as a prevalent case for that year.

Data inputs for chronic obstructive pulmonary disease

Parameters	Countries with data	New sources	Total sources
Prevalence	54	42	160
Incidence	7	18	7
Remission	0	0	0
Other	32	0	36

Data processing

Age and sex split

In some cases, data are reported by only age or only sex, but not both. For example, a study may have included the prevalence of males and females with COPD and then separately reported the prevalence for both sexes in smaller age bins (eg, age 40–45, 46–50, etc.) that have COPD. In these cases, we perform an age-sex split by utilising proportions within the study to disaggregate the data.

When data are not disaggregated into male and female categories for a given data source, we instead perform a sex-split on the data by applying sex proportions from other studies that do have male- and female-specific data. When data are aggregated into age categories larger than 25 years, we split into smaller age bins based on super-regional age patterns in the 2017 COPD model.

Modelled excess mortality data

In GBD 2021, we continued modelling excess mortality rate (EMR) data outside of DisMod-MR 2.1[1] (disease model—Bayesian meta-regression, described in appendix 1, section 4.5) following GBD 2019.

Prior to GBD 2019, priors on EMR were estimated in DisMod by matching prevalence datapoints with their corresponding CSMR values within the same age, sex, year, location (by dividing CSMR by prevalence).

However, for many causes, DisMod estimated a rather unrealistic pattern of EMR compared to an expected pattern of decreasing EMR with greater access to quality health care. Such unexpected patterns often signal inconsistencies between CSMR estimates and the measures of prevalence and/or incidence.

In an effort to provide greater guidance to DisMod on the expected pattern of EMR, EMR data generated in the previous round were used as inputs for modeling in MR-BRT[2] (meta-regression—Bayesian, regularised, trimmed, described in appendix 1, section 4.4.1 of the reference) with age, sex, and Healthcare Access and Quality (HAQ) Index included as covariates. Results from MR-BRT were then predicted for each location year, sex, and for ages 0, 10, 20 ….100.

This method led to improvements in the consistency of EMR relative to health-care access. We also included HAQ Index as a country-level covariate in DisMod to inform EMR with the mean and standard deviation produced from MR-BRT analysis.

Bias adjustments

In GBD 2021, we ran the same bias adjustment methods used in GBD 2019, by utilising a MR-BRT model outside of DisMod to allow a more direct comparison between different case definitions and/or study

designs.

We made a series of adjustments to data that do not completely match our case definition. Different diagnosis often leads to different estimates of COPD. Similarly, claims data are subject to biases. Claims data are often systemically lower than survey data, probably due to selection bias with regard to socioeconomic status. Adjustments are made to these data to correct these biases.

The adjustment is a logit-transformation method in MR-BRT. The general process is described below:

15. Identify datapoints with overlapping year, age, sex, and location between reference and alternative definitions.
16. Logit transform overlapping datapoints of alternative and reference case definitions
17. Convert overlapping datapoints into a difference in logit space using the following equation: $logit(altnerative) - logit(reference)$
18. Use the delta method to compute standard errors of overlapping datapoints in logit space, then calculate standard error of logit difference using the following equation: $\sqrt{(variance\ of\ alternative) + (variance\ of\ reference)}$
19. Using MR-BRT, conduct a random effects meta-regression to obtain the pooled logit difference of alternative to reference
20. Apply the pooled logit difference to all data points of alternative case definitions using the following equation:
$$new_{estimate} = inverse.logit((logit(alternative)) - (pooled\ logit\ difference))$$
21. Calculate new standard errors using the delta method, accounting for gamma (between-study heterogeneity)

Data derived from claims from commercial health insurance in the United States were also adjusted using a factor estimated in MR-BRT. Claims data, notably USA MarketScan, were adjusted in relation to the BOLD study data. In this case, the BOLD data serve as the reference definition while the MarketScan data are the alternative definition.

MR-BRT crosswalk adjustment factors

Data input	Status	Gamma	Beta coefficient, logit* (95% UI)	Adjustment factor**
GOLD post	Ref	0.25	---	---
GOLD pre	Alt		0.50 (-0.02 - 1.07)	0.62 (0.49 - 0.74)
ERS	Alt		0.70 (0.11 - 1.31)	0.67 (0.53 - 0.79)
LLN pre	Alt	0.08	0.10 (0.01 - 0.19)	0.52 (0.50 - 0.55)
LLN post			-0.34 (-0.50 - -0.19)	0.42 (0.38 -0.45)
BOLD	Ref	.19	---	---
MarketScan	Alt		-1.93 (-2.35 - -1.50)	0.13 (0.08 - 0.18)

MR-BRT crosswalk adjustments can be interpreted as the factor the alternative case definition is adjusted by to reflect what it would have been had it been measured using the reference case definition. If the log/logit beta coefficient is negative, then the alternative is adjusted up to the reference. If the log/logit beta coefficient is positive, then the alternative is adjusted down to the reference.

***The adjustment factor column is the exponentiated beta coefficient. For log beta coefficients, this is the relative rate between the two case definitions. For logit beta coefficients, this is the relative odds between the two case*

definitions.

Modelling strategy

The estimation of COPD burden has two distinct steps.

1. Estimate prevalence and incidence using a DisMod-MR 2.1 model
2. Estimate proportion of COPD severities using GOLD class groupings in DisMod-MR 2.1

After these two steps, the COPD prevalence and incidence are split by age, sex, and location for each severity level.

Step 1: Main COPD model – estimate prevalence and incidence using DisMod-MR 2.1

Model settings
We set remission to 0 because individuals do not recover once they have COPD. The symptoms are only managed. Incidence ceiling is set at 0.0002 before age 15 and a ceiling at 0.0005 before age 30 to avoid a kick-up of estimates in age ranges with few or no primary data.

Each model includes a series of country-level covariates that describe spatiotemporal patterns.

- COPD standardised exposure variables (SEV) aggregates multiple risk factors into a single variable.
- Healthcare Access and Quality (HAQ) Index on EMR to capture country-level variation of EMR, assuming a negative coefficient (ie, lower mortality with rising GDP and HAQ). The priors of HAQ Index came from the EMR MR-BRT prediction.
- The proportion of elevation over 1500m was included as a country-level covariate on EMR because of its significance in COPD cause of death models.

Model coefficients for COPD

Model	Variable name	Measure	Beta	Exponentiated
COPD	Elevation over 1500m (proportion)	excess mortality rate	0.60<br>(0.14 — 0.95)	1.81<br>(1.15 — 2.58)
COPD	Healthcare Access and Quality Index	excess mortality rate	-0.022<br>(-0.023 — -0.022)	0.98<br>(0.98 — 0.98)
COPD	Log age-standardised SEV scalar: COPD	prevalence	0.91<br>(0.90 — 0.92)	2.47<br>(2.46 — 2.50)

Step 2: GOLD class models to estimate proportions of severities
The GOLD class models use data from surveys that specified prevalence by GOLD class after expressing the values as a proportion of all COPD cases. For GBD 2016, we used fixed effects from the SEV scalar and the log of lag-distributed income (LDI) per capita to assist estimation. For GBD 2017, we dropped these covariates because they did not produce significant coefficients, and we also did not use them for GBD 2019 or GBD 2021. We also restricted random effects to +/-0.5 to control implausible geographical variation.

Severity splits

The three GOLD class groupings reflect a grading based on a physiological measurement rather than a direct measurement of disease severity. In order to map the epidemiological findings by GOLD class into the three COPD health states for which we have disability weights (DW), we used the 2001–2011 Medical

Expenditure Panel Survey (MEPS) data from the USA. Specifically, we convert the GOLD class designations

estimated for the USA in 2005 (the midpoint of MEPS years of analyses) into GBD classifications of asymptomatic, mild, moderate, and severe COPD.

The table below shows the three health states of COPD and the corresponding lay descriptions and disability weights. The graph shows the average proportion by GOLD class (after scaling to 100%) across all ages for USA in 2005. We also show the proportion of MEPS respondents reporting any health service contact in the past year for COPD with a DW value attributable to COPD of 0, mild range (0 to midpoint between DWs for mild and moderate), moderate range (midpoint of DW values mild and moderate to midpoint of DW values for moderate and severe) and severe range (midpoint between DW values moderate and severe or higher). The DW value for COPD was derived from a regression with indicator variables for all health states reported by MEPS respondents and their reported overall level of disability derived from a conversion of 12-Item Short Form Surveys (SF-12) answers to GBD DW values. This analysis gave the severity distribution for each GBD cause reported in MEPS after correcting for any comorbid causes individual respondents reported during a year.

Description of health states

Health state	Lay description	DW (95% CI)
Mild COPD	This person has cough and shortness of breath after heavy physical activity, but is able to walk long distances and climb stairs.	0.019 (0.011–0.033)
Moderate COPD	This person has cough, wheezing, and shortness of breath, even after light physical activity. The person feels tired and can walk only short distances or climb only a few stairs.	0.225 (0.153–0.31)
Severe COPD	This person has cough, wheezing, and shortness of breath all the time. The person has great difficulty walking even short distances or climbing any stairs, feels tired when at rest, and is anxious.	0.408 (0.273–0.556)

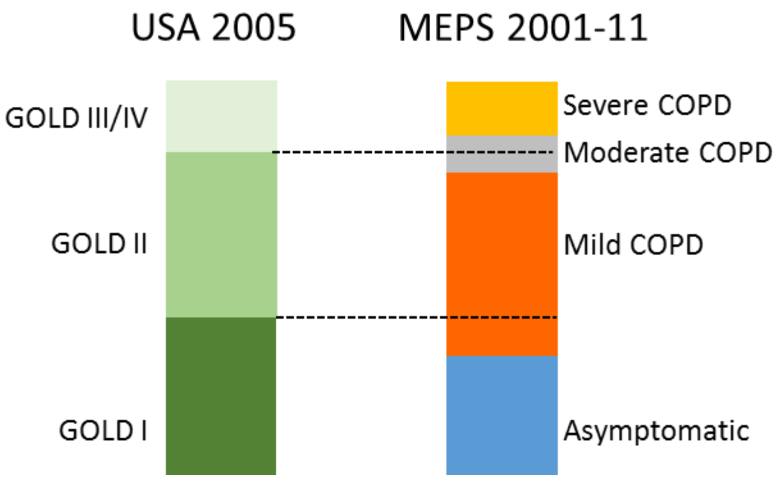

The algorithm to translate GOLD class to COPD DW categories first assigns GOLD III&IV to severe COPD and what remains to moderate. Next, GOLD class I is assigned to the asymptomatic category first and what remains goes to mild COPD. This algorithm is repeated for each age and sex category and for all 1000 draws from the DisMod models of GOLD classes and the MEPS analyses. We end up with proportions of each of the GOLD class categories that map onto GBD COPD health states with uncertainty

bounds determined by the 25th and 975th values of the 1000 draws. These values are then applied to the

estimates of the proportion of cases by GOLD class category, after scaling to 100%, by location, year, age, and sex. This assumes that the relationship between GOLD class and GBD COPD health states in the United States applies everywhere.

References

1. Vos T, Lim SS, Abbafati C, et al. Global burden of 369 diseases and injuries in 204 countries and territories, 1990–2019: a systematic analysis for the Global Burden of Disease Study 2019. The Lancet 2020; 396: 1204–22. doi: https://doi.org/10.1016/S0140-6736(20)30925-9
2. Murray CJL, Aravkin AY, Zheng P, et al. Global burden of 87 risk factors in 204 countries and territories, 1990–2019: a systematic analysis for the Global Burden of Disease Study 2019. The Lancet 2020; 396: 1223–49. doi: https://doi.org/10.1016/S0140-6736(20)30752-2

Cirrhosis and other chronic liver diseases

Flowchart

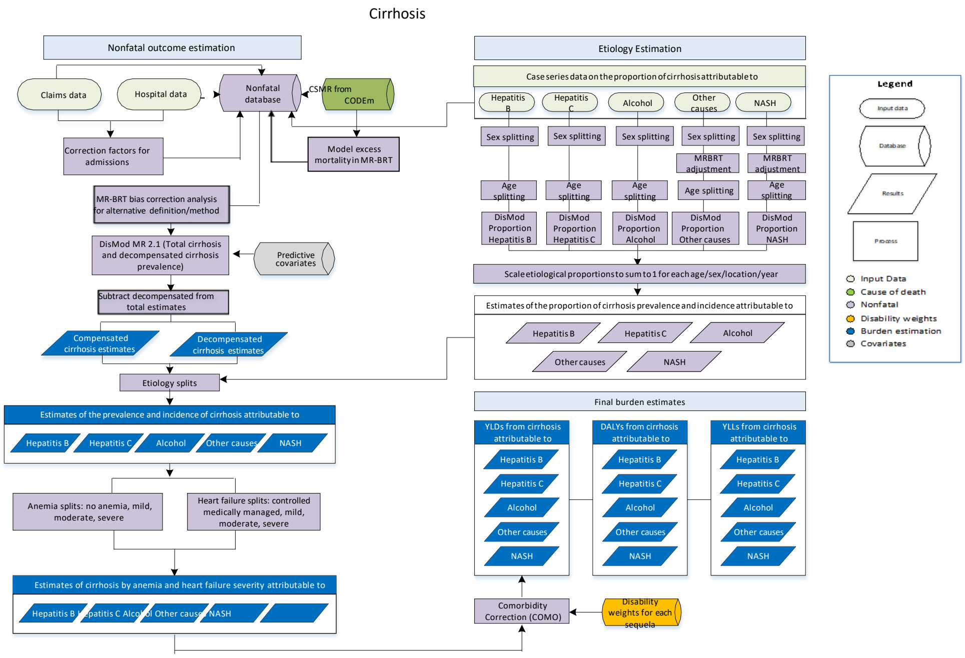

Input data and methodological summary for cirrhosis

Case definition

This cause encompasses both cirrhosis and a number of other chronic liver diseases that, left unchecked, may progress to cirrhosis. Cirrhosis is chronic, progressive replacement of healthy liver tissue by scarring, including cases where the liver is still able to functionally compensate for lost tissue, and decompensated cases, where liver function has become impaired and complications develop (such as ascites, jaundice, gastrointestinal bleeding, renal failure, or encephalopathy). This cause also includes nonalcoholic fatty

liver disease (without cirrhosis), chronic hepatitis B infection (without cirrhosis), and chronic hepatitis C infection (without cirrhosis).

This Level 3 cause includes five Level 4 causes:

- Cirrhosis and other chronic liver diseases due to alcohol use: cirrhosis (ie, scarring of liver) due to alcohol use, regardless of whether there is functional liver impairment and symptoms.
- Chronic hepatitis B including cirrhosis: Encompasses all chronic infection with hepatitis B virus, including cases that have developed cirrhosis (ie, scarring of the liver) and those who have not.
- Chronic hepatitis C including cirrhosis: Encompasses all chronic infection with hepatitis C virus, including cases that have developed cirrhosis (ie, scarring of the liver) and those who have not, due to chronic infection by hepatitis C virus.
- Nonalcoholic fatty liver disease including cirrhosis: Encompasses the spectrum of nonalcoholic fatty liver disease including fat deposition without cirrhosis and cirrhosis (ie, scarring of the liver) that can result from longstanding and progressive fat deposition and inflammation.
- Cirrhosis and other chronic liver diseases due to other causes: Cirrhosis (ie, scarring of liver) due to other causes such as (but not limited to): Wilson's disease, cryptogenic, PBC primary biliary cholangitis, hemochromatosis, and autoimmune disease, and cryptogenic cases, regardless of whether there is functional liver impairment and symptoms.

Input data and processing

Input data for total cirrhosis and decompensated cirrhosis

We modelled the incidence and prevalence of total cirrhosis and of decompensated cirrhosis using hospital discharge data and claims data. A limitation of hospital data is that individuals cannot be identified in the administrative records. As such, one person can have multiple hospital encounters for the same condition in a year, leading to overestimation of non-fatal burden. We resolved this issue using patient-level sources that do track individual hospital encounters for the same reason and correct for readmissions. Another concern is that hospital data do not reflect outpatient cases. We used MarketScan data, which contain both inpatient and outpatient data, to generate scalars to adjust data sources that only report inpatient primary admissions to inpatient and outpatient all diagnoses.

The total cirrhosis model uses claims data for both inpatient and outpatient care and inpatient discharge data adjusted to total cases diagnosed in inpatient and outpatient encounters. The decompensated model uses claims data only for inpatient care, and hospital discharge data adjusted only to account for readmissions. (See sections of this appendix for details of hospital and claims data processing.)

Table 7: Prevalence data inputs for total and decompensated cirrhosis

Measure	Countries with data	New sources	Total sources
Prevalence	55	35	380
Other	43	15	105

Additional inputs to the non-fatal models of total and decompensated cirrhosis include cause-specific mortality rates (CSMR) produced for every year, age, sex, and location in the CoDCorrect process (please see CoD cause-specific modeling description for cirrhosis in this appendix) and excess mortality rates (EMR) inputs generated using MR-BRT (see the EMR data processing section below).

Input data for cirrhosis aetiologic proportions

Additionally, we use data from cirrhosis case-series that report the proportion of cirrhosis cases attributed to alcohol, hepatitis B, hepatitis C, NASH, and other causes. In GBD 2021, 11 new case-series studies were added from a literature review in PubMed using the search string below. Given time limitations, we expedited the search by looking for results that reported the terms “cirrhosis” and “cases” from the search hits. Studies that did not have these terms in the title/abstract were deferred to GBD 2022 for screening.

(((((((hepatitis b[Title/Abstract] OR "hepatitis b antibod*"[Title/Abstract] OR "hepatitis b antigens"[Title/Abstract] OR hbsag[Title/Abstract])) OR (hepatitis c[Title/Abstract] OR "hepatitis c antibod*"[Title/Abstract] OR "hepatitis c antigens"[Title/Abstract] OR "anti-hcv"[Title/Abstract] OR HCV-RNA[Title/Abstract]))))) AND (alcohol* OR "alcoholic disorders" OR cirrhosis))) AND (NAFLD OR "non-alcoholic fatty liver disease" OR NAFL)

The inclusion criteria for case-series data stipulated that:

- The publication year must be from 1980 onward.
- Sufficient information must be provided on study method and sample characteristics to assess the quality of the study.
- The sample had to be a representative sample of those with* decompensated cirrhosis without HCC, compensated cirrhosis without HCC, deaths due to cirrhosis without HCC (reference standard populations).
  ** Note: We included case-series that studied various diagnoses and stages in our inclusion criteria but will disaggregate proportions in future rounds to identify variation in aetiologies by diagnosis or stage*.
- The cirrhosis cases should be identified by admin data, chart review, non-invasive test, liver biopsy or other diagnostic for which a valid adjustment can be made.
- The diagnosis of the aetiology can include the following reference definitions or an alternative definition that could produce a crosswalk.
  - Hepatitis B: confirmed via HBsAg
  - Hepatitis C: confirmed via anti-HCV OR HCV-RNA
  - Alcohol: reliable history of significant alcohol intake, clinical examination and laboratory features suggestive of significant alcohol intake
  - NAFLD: intake of less than 20 g of ethanol per day AND appropriate exclusion of other liver diseases

The exclusion criteria were as follows:

- If a study evaluated only a subset of aetiologies, the study must have exhaustively categorised all cirrhotic persons in the sample into a specific aetiology or an "other" or "unknown" category, but cannot have excluded cirrhotic persons from the denominator for not having one of the aetiologies under study.
- If the study reported on "multiple aetiologies" without specifying co-occurrence of aetiologies, the study can be included but those cases should be removed from both the numerator and the denominator in study extraction.
- Administrative records with no report on methods used to determine aetiology of cirrhosis.

Figure 1: Aetiological proportion data sources

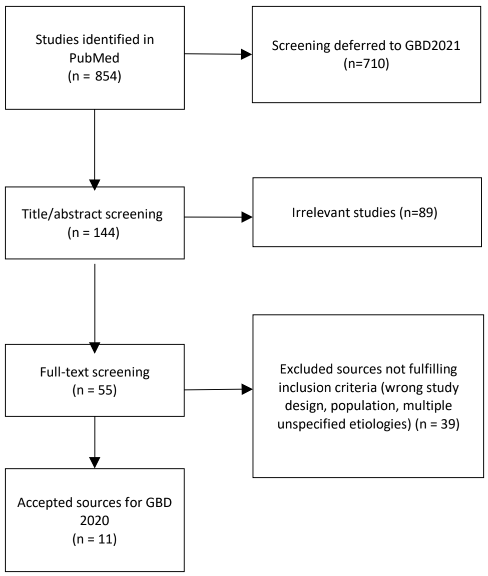

Table 8: Data inputs for cirrhosis aetiological proportion modelling

Model	Measure	Total sources	New sources	Countries with data	Number of regions	Number of super-regions
Cirrhosis and other chronic liver diseases due to hepatitis B, proportion	Proportion	99	15	40	18	7
Cirrhosis and other chronic liver diseases due to hepatitis C, proportion	Proportion	98	13	40	17	7
Cirrhosis and other chronic liver diseases due to alcohol, proportion	Proportion	64	10	27	13	6
Proportion of cirrhosis due to other causes	Proportion	44	13	23	13	7
Proportion of cirrhosis due to NASH	Proportion	35	11	19	10	5

Prevalence input processing

Adjustment factors were estimated and applied prior to modelling to those prevalence data collected using non-reference case definitions or study designs. Data with different study design characteristics were matched (by year, age, sex, location) for reference and alternative definitions, and their systematic differences were modelled using meta-regression—Bayesian, regularised, trimmed (MR-BRT), a regression tool developed at IHME. Specifically, adjustments were made for data from MarketScan, a database of claims data for commercial insurance in the USA, which may be biased because commercially insured individuals may have differential health-care-seeking behaviours compared to those in the general population. We conducted an analysis in MR-BRT with a spline on age to adjust these commercial claims data to hospital data differentially by age. The analysis was conducted between MarketScan data in 2000 compared to hospital data in 2000, and then all other years of MarketScan data compared to other years of hospital data. The process of adjusting for biases in non-reference data using MR-BRT with the logit-transformation method is described below:

22. Identify datapoints with overlapping year, age, sex, and location between non-reference data and reference data.
23. Logit transform overlapping datapoints of alternative and reference types.
24. Convert overlapping datapoints into a difference in logit space using the following equation: $logit(alternative) - logit(reference)$.
25. Use the delta method to compute standard errors of overlapping datapoints in logit space, then calculate standard error of logit difference using the following equation: $\sqrt{(variance\ of\ alternative) + (variance\ of\ reference)}$.
26. Using MR-BRT, conduct a random effects meta-regression to obtain the pooled logit difference of alternative to reference.

27. Apply the pooled logit difference to all data points of alternative case definitions using the following equation:

$$new_{estimate} = inverse.logit((logit(alternative)) - (pooled\ logit\ difference)).$$

28. Calculate new standard errors using the delta method, accounting for gamma (between-study heterogeneity).

The estimated adjustment factors and their uncertainty are shown in the table below, followed by figures showing examples of adjustment of non-refence definition to the reference definition after data processing in MR-BRT.

Table 9. MR-BRT crosswalk adjustment factors for total cirrhosis prevalence data

Data input	Reference or alternative data collection	Gamma	Covariate	Beta coefficient, logit (95% CI)
Hospital + non-USA claims	Ref	0.002		---
USA claims from year 2000	Alt		Age	–0.016 (–0.018 to –0.013)
			Sex	0.206 (0.132 to 0.281)
			Intercept	0.764 (0.584 to 0.943)
USA claims from years 2010–2017	Alt		Age	0.0004 (–0.083 to 0.084)
			Sex	0.272 (0.188 to 0.357)
			Intercept	0.472 (0.378 to 0.566)

**MR-BRT crosswalk adjustments can be interpreted as the factor the alternative case definition is adjusted by to reflect what it would have been had it been measured using the reference case definition. If the log/logit beta coefficient is negative, then the alternative is adjusted up to the reference. If the log/logit beta coefficient is positive, then the alternative is adjusted down to the reference.*

Table 4. MR-BRT crosswalk adjustment factors for decompensated cirrhosis prevalence data

Data input	Reference or alternative data collection	Gamma	Covariate	Beta coefficient, logit (95% CI)
Hospital + non-USA claims	Ref	0.002		---
USA claims from year 2000	Alt		Age	0.012 (–0.071 to 0.095)
			Sex	0.24 (0.056 to 0.425)
			Intercept	–0.739 (–1.186 to –0.291)
USA claims from years 2010–2017	Alt		Age	0.012 (–0.071 to 0.095)
			Sex	0.287 (0.201 to 0.374)
			Intercept	0.038 (–0.071 to 0.147)

**MR-BRT crosswalk adjustments can be interpreted as the factor the alternative case definition is adjusted by to reflect what it would have been had it been measured using the reference case definition. If the log/logit beta coefficient is negative, then the alternative is adjusted up to the reference. If the log/logit beta coefficient is positive, then the alternative is adjusted down to the reference.*

Figure 2: Adjust for total cirrhosis (left) and decompensated cirrhosis (right) prevalence data MarketScan years after 2000

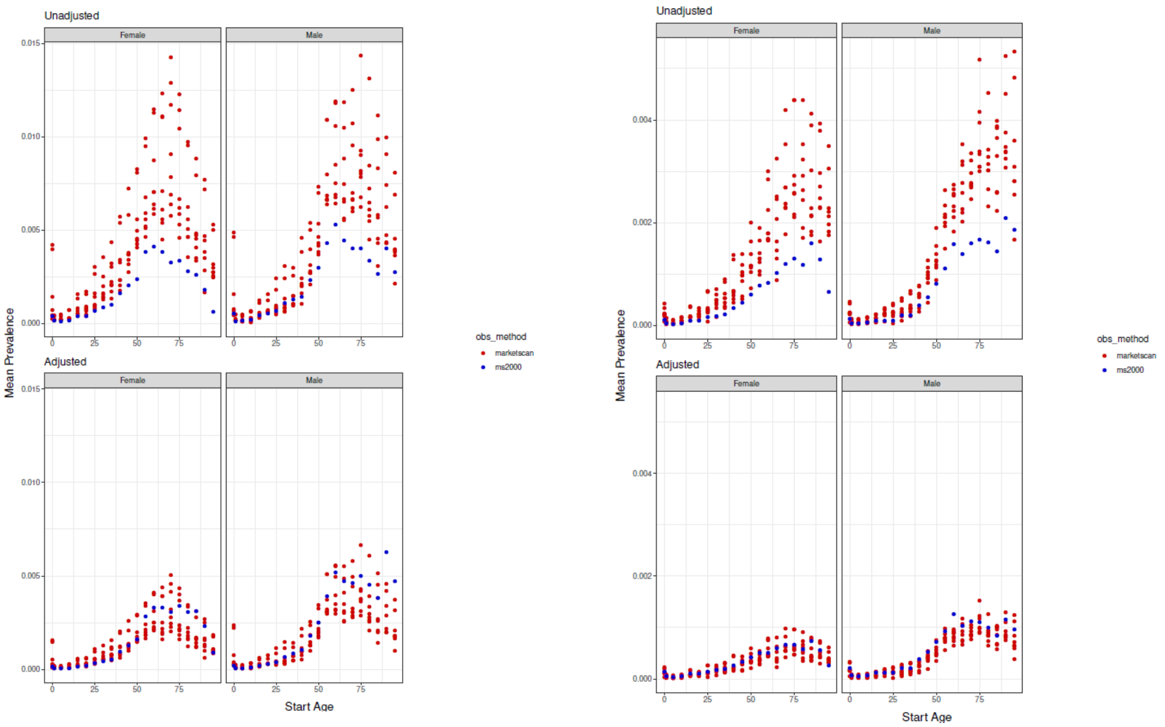

Datapoints with an age-standardised prevalence rate greater than two median absolute deviations from the median of the age-standardised prevalence rate for all data were marked as outliers and excluded from analysis.

EMR input processing

In GBD 2017, EMR inputs were produced by matching prevalence datapoints with their corresponding CSMR values within the same age, sex, year, and location (by dividing CSMR by prevalence). For short-duration conditions (remission >1), the corresponding prevalence was derived by running an initial model and then applying the same CSMR/prevalence method. However, this method of producing EMR inputs demonstrated a rather unrealistic pattern of EMR compared to an expected pattern of decreasing EMR with greater access to quality health care. Such unexpected patterns often signal inconsistencies between CSMR estimates and the measures of prevalence and/or incidence. Thus, in an effort to provide greater guidance on the expected pattern of EMR, in GBD 2019, EMR data produced per above in GBD 2017 were modelled by age, sex and Healthcare Access and Quality (HAQ) Index using MR-BRT, with a prior on HAQ Index having a negative coefficient. In GBD 2021, we employed the same MR-BRT method to predict EMR for each location, year, sex, and for ages 0, 10, 20….100; these predictions were used as inputs to our non-fatal model, below.

Aetiological proportion input processing

Prior to modelling, we performed several adjustments to case-series data sources to correct for non-reference data collection methods, including “multi-aetiology splitting”, “sex-splitting”, and “age-splitting”.

Some studies reported cases in which multiple risk factors of cirrhosis were identified. However, we do not have enough data on multiple aetiology data to estimate combinations of aetologies in distinct models. Instead, we reassigned these multi-aetiology cases to single aetiologies prior to modelling the five distinct aetologies using intra-study proportions. For example, a study might report 100 cases of cirrhosis total, of which 40 cases are due to hepatitis B, 20 due to alcohol, 30 due to NAFLD, and 10 due to hepatitis B and alcohol. We must redistribute cases due to both hepatitis B and alcohol proportionate to cases of each aetiology separately, without adjusting cirrhosis due to NAFLD data. We redistribute the 10 cases of hepatitis B and alcohol by a ratio of 40:20, resulting in 47 cases of hepatitis B and 23 cases due to alcohol.

Figure 3: Pre- and post-adjustment of multi-aetiology casesError! Reference source not found. figure below shows the proportion of each aetiology before and after adjustment of multi-aetiology cases to single aetiologies.

Figure 3: Pre- and post-adjustment of multi-aetiology cases

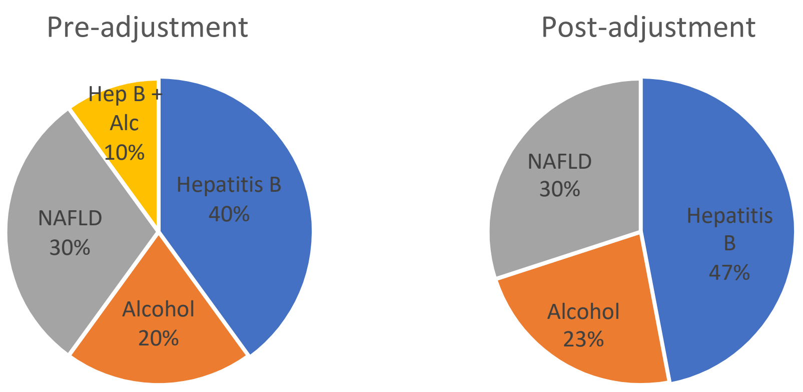

Because we produce sex-specific estimates, we adjusted data that reported on both sexes into male and female sex-specific estimates. We identified studies that reported on sex-specific data and calculated the log ratio of female to male prevalence from studies that report sex-specific prevalence, modelling these log ratios in MR-BRT. We used cause-specific studies to estimate the sex ratio for aetiological proportions of cirrhosis in order to reflect the underlying epidemiology of the disease in a given population. We then used the modelled sex ratio to adjust "both"-sex data values to expected "male" and "female" values. We calculated the male values as $val_{male} = val_{both} * \frac{pop_{both}}{(pop_{male}+ratio*pop_{female})}$. We calculated female values $val_{female} = ratio * val_{male}$. The table below lists out the estimated sex ratio by proportion aetiology, and the figures show funnel plots. We split datapoints where the age range was greater than 25 years using the super-region age patterns informed by the datapoints with fine age groups (ie, ages 5-9, 10-14, and 15-20…).

Table 10: MR-BRT sex ratios for cirrhosis aetiology proportions

Cirrhosis etiology proportion	Beta coefficient, log (95% CI)	Gamma	Interpretation
Cirrhosis due to hepatitis B	–0.26 (–0.39 to –0.13)	0.018	Higher for males
Cirrhosis due to hepatitis C	0.14 (–0.04 to 0.33)	0.089	Higher for females
Cirrhosis due to alcohol	–1.02 (–1.42 to –0.62)	0.312	Higher for males
Cirrhosis due to other causes	0.642 (0.41 to 0.87)	0.063	Higher for females
Cirrhosis due to NASH	0.80 (0.65 to 0.95)	0.005	Higher for females

**MR-BRT crosswalk adjustments can be interpreted as the factor the alternative case definition is adjusted by to reflect what it would have been had it been measured using the reference case definition. If the log/logit beta coefficient is negative, then the alternative is adjusted up to the reference. If the log/logit beta coefficient is positive, then the alternative is adjusted down to the reference.*

Epidemiological studies and hepatologists have indicated that cryptogenic cases of cirrhosis may be un-identified cases of cirrhosis due to NASH. In GBD 2017, if studies reported on crytogenic cases without reports of NASH, we considered NASH. In GBD 2019 and GBD 2021, we analysed case-series studies that reported both NASH and cryptogenic cases, modelling the proportion due to NASH (out of NASH plus cryptogenic) in MR-BRT. We then identified the case-series in our database that reported cryptogenic, but not NASH, as an aetiology of cirrhosis, and extracted a proportion due to NASH and a proportion due to other causes based on the proportion modelled in MR-BRT.

Table 11: Cryptogenic-NASH adjustment factor in MR-BRT

Data input	Beta coefficient, logit (95% CI)	Gamma
Proportion of cryptogenic cases out of cryptogenic cases plus NASH cases reported in the same study	0.465 (0.231–0.698)	0.111

Data inputs for estimating the incidence and prevalence of chronic hepatitis B infection, the incidence and prevalence of chronic hepatitis C infection, and the incidence and prevalence of non-alcoholic fatty liver disease are described in the sections titled “Acute Heaptitis A, B, C and E” and “Nonalcoholic fatty liver disease without cirrhosis” of the “Non-fatal cause-specific modelling descriptions” section of this appendix.

Modelling strategy

Overview

We modelled the prevalence and incidence of total cirrhosis and the prevalence and incidence of decompensated cirrhosis using clinical informatics prevalence data, CSMR and EMR, processed as described above, assuming no remission, in full compartmental models in DisMod-MR 2.1. The summary of covariates and the exponentiated betas of the total cirrhosis and decompensated cirrhosis DisMod-MR 2.1 models are listed in tables below. To estimate the prevalence of cirrhosis due to alcohol, hepatitis B, hepatitis C, NASH, and other causes, we developed five single-parameter models of five aetiological proportions using DisMod-MR 2.1, and used the results of these models to split the parent total cirrhosis and decompensated cirrhosis prevalence estimates.

Total and decompensated cirrhosis DisMod models

Table 12: Summary of covariates used in the total cirrhosis DisMod-MR 2.1 model

Covariate	Parameter	Exponentiated beta (95% uncertainty interval)
Vaccine-adjusted HBsAg seroprevalence, age-standardised	Prevalence	43.4 (31.5–53.7)
Chronic hepatitis C, age-standardised	Prevalence	1.23 (1.01–1.70)

Alcohol drinker proportion, age-standardised	Prevalence	1.26 (1.20–1.32)
Litres of alcohol consumed per capita	Prevalence	1.00 (1.00–1.00)
Prevalence of obesity	Prevalence	1.01 (1.00–1.03)
Healthcare Access and Quality Index	Excess mortality rate	0.99 (0.99–0.99)

Table 13: Summary of covariates used in the decompensated cirrhosis DisMod-MR 2.1 model

Covariate	Parameter	Exponentiated beta (95% uncertainty interval)
Vaccine-adjusted HBsAg seroprevalence, age-standardised	Prevalence	52.8 (50.0–54.5)
Chronic hepatitis C, age-standardised	Prevalence	1.14 (1.01–1.37)
Litres of alcohol consumed per capita	Prevalence	1.00 (1.00–1.00)
Alcohol drinker proportion, age-standardised	Prevalence	1.33 (1.27–1.41)
Prevalence of obesity	Prevalence	1.00 (1.00–1.01)
Healthcare Access and Quality Index	Excess mortality rate	0.98 (0.98–0.98)

Aetological proportion DisMod models

Data for aetiological proportion models are scant, and estimates are strengthened by using predictive covariates. As in previous rounds, DisMod models for the proportion of cirrhosis due to each aetiology use the following predictive covariates: the prevalence of the precursor states that can give rise to each aetiology of cirrhosis (prevalence of hepatitis B, hepatitis C, alcohol consumption, etc.) and the most recent estimate of the proportion of liver cancer cases due to each aetiology, all with bounds limiting to positive associations. (See liver cancer appendix section for details on estimation of aetiological proportions for liver cancer.) The summary of covariates and the exponentiated betas of each aetiological proportion model are listed in tables below (Table 14 to Table 18).

Table 14: Covariates used in the proportion of cirrhosis due to hepatitis B DisMod-MR meta-regression model

Covariate	Exponentiated beta (95% uncertainty interval)
Vaccine-adjusted HBsAg seroprevalence, age-standardised	1.64 (1.06–2.57)
Proportion of liver cancer due to hepatitis B, age-standardised	1.28 (1.02–1.72)
Hepatitis B vaccine coverage (proportion), aged through time	0.55 (0.38–0.88)
Proportion of cirrhosis due to alcohol	0.46 (0.37–0.65)
Proportion of cirrhosis due to hepatitis C	0.65 (0.45–0.93)
Proportion of cirrhosis due to other causes	0.68 (0.47–0.94)
Proportion of cirrhosis due to NASH	0.59 (0.39–0.94)

Table 15: Covariates used in the proportion of cirrhosis due to hepatitis C DisMod-MR meta-regression model

Covariate	Exponentiated beta<br>(95% uncertainty interval)

Chronic hepatitis C, age-standardised	1.79 (1.10–2.63)
Proportion of liver cancer due to hepatitis C, age-standardised	1.86 (1.19–2.63)
Proportion of cirrhosis due to alcohol	0.41 (0.37–0.50)
Proportion of cirrhosis due to hepatitis B	0.53 (0.38–0.80)
Proportion of cirrhosis due to other causes	0.94 (0.82–1.00)
Proportion of cirrhosis due to NASH	0.63 (0.41–0.94)

Table 16: Covariates used in the proportion of cirrhosis due to alcohol DisMod-MR meta-regression model

Covariate	Exponentiated beta (95% uncertainty interval)
Litres of alcohol consumed per capita	1.01 (1.00–1.03)
Alcohol drinker proportion, age-standardised	1.58 (1.14–2.19)
Proportion of liver cancer due to alcohol, age-standardised	1.39 (1.02–2.17)

Table 17: Covariates used in the proportion of cirrhosis due to other causes DisMod-MR meta-regression model

Covariate	Exponentiated beta (95% uncertainty interval)
Proportion of liver cancer due to other causes, age-standardised	1.91 (1.22–2.64)

Table 18: Covariates used in the proportion of cirrhosis due to NASH DisMod-MR meta-regression model

Covariate	Exponentiated beta (95% uncertainty interval)
Mean BMI	1.27 (1.06–1.54)
Prevalence of obesity	2.19 (1.06–5.45)
NAFLD/NASH prevalence	3.22 (1.27–6.78)
Proportion of liver cancer due to NASH, age-standardised	2.30 (1.07–5.75)

Compensated cirrhosis prevalence estimation

Final decompensated cirrhosis prevalence estimates at the 1000-draw level were subtracted from the final total cirrhosis prevalence estimates at the 1000-draw level to generate 1000 draws of compensated cirrhosis prevalence estimates (which provides an estimated mean with 95% uncertainty interval).

Aetiology-specific cirrhosis prevalence estimation

We used the five aetiological proportion estimates to split the compensated and decompensated cirrhosis prevalence estimates. Proportions were rescaled to sum to one at the draw level and then multiplied by the estimates of the prevalence of decompensated cirrhosis and compensated cirrhosis.

Cause-level incidence estimation

In GBD, we consider cirrhosis to develop through one of five aetiological pathways: heavy alcohol use, chronic infection with hepatitis B or C, non-alcoholic steatohepatitis, and a residual category of multiple other causes. In order to develop cirrhosis, we assume that people must first have been at risk of developing cirrhosis through one of these five pathways. Nonetheless, the cirrhosis and other chronic liver disease estimates variably include precursor states depending on aetiology (see below), and incidence estimates reported in GBD reflect the incidence of the earliest stage of chronic liver disease estimated for that aetiology.

The incidence estimates for cirrhosis and chronic liver disease corresponding to each aetiology of cirrhosis were, therefore, calculated as follows:

- For alcohol use and other causes, cause-level incidence estimates are estimates of the incidence of compensated cirrhosis due to alcohol use and compensated cirrhosis due to other causes. Since all cases of cirrhosis must start as compensated and progress to decompensated, the incidence estimates from the DisMod-MR 2.1 compartmental model of total cirrhosis were treated as the incidence of compensated cirrhosis, and these were multiplied by the aetiological proportions for alcohol and for other causes at the draw-level to estimate incidence of compensated cirrhosis due to alcohol use and compensated cirrhosis due to other causes.
- For cirrhosis and other chronic liver diseases due to chronic infection with hepatitis B, cause-level incidence estimates are estimates of the incidence of chronic hepatitis B infection (see “Acute Hepatitis A, B, C, and E” section of this appendix).
- For cirrhosis and other chronic liver diseases due to chronic infection with hepatitis C, cause-level incidence estimates are estimates of the incidence of chronic hepatitis C infection (see “Acute Hepatitis A, B, C, and E” section of this appendix).
- For cirrhosis and other chronic liver diseases due to non-alcoholic steatohepatitis, cause-level incidence estimates are the non-alcoholic fatty liver incidence estimates (see “Nonalcoholic fatty liver disease without cirrhosis” section of this appendix).

Sequelae and disability weights

We estimated the proportion of individuals with decompensated cirrhosis that had different severity levels of anaemia: no anaemia, mild anaemia, moderate anaemia, and severe anaemia. After estimation of decompensated cirrhosis due to each aetiology, we further split estimates to reflect these anaemia severity proportions. See the “Anaemia impairment (envelope and causal attribution)” section of this appendix for details. We also estimated the proportion of individuals with heart feailure that had decompensated cirrhosis. The different severity levels of heart failure include controlled and medically managed, mild, moderate, and severe. See the “Heart failure estimation” section of this appendix for details. Decompensated cirrhosis with and without anaemia and heart failure were assigned the following health states and disability weights.

Table 19: Disability weights for decompensated cirrhosis

Health state	Lay description	Disability weight (95% CI)

Decompensated cirrhosis of the liver	Has a swollen belly and swollen legs. The person feels weakness, fatigue and loss of appetite.	0.178 (0.113–0.243)
Decompensated cirrhosis of the liver and mild anaemia	Feels slightly tired and weak at times, but this does not interfere with normal daily activities.	0.181 (0.116–0.246)
Decompensated cirrhosis of the liver and moderate anaemia	Feels moderate fatigue, weakness, and shortness of breath after exercise, making daily activities more difficult.	0.220 (0.146–0.295)
Decompensated cirrhosis of the liver and severe anaemia	Feels very weak, tired and short of breath, and has problems with activities that require physical effort or deep concentration.	0.300 (0.202–0.397)
Decompensated cirrhosis of the liver, controlled, medically managed heart failure	Has been diagnosed with clinical heart failure, a chronic disease that requires medication every day and causes some worry but minimal interference with daily activities.	0.218 (0.154–0.298)
Decompensated cirrhosis of the liver, mild heart failure	Is short of breath and easily tires with moderate physical activity, such as walking uphill or more than a quarter-mile on level ground. The person feels comfortable at rest or during activities requiring less effort.	0.212 (0.150–0.290)
Decompensated cirrhosis of the liver, moderate heart failure	Is short of breath and easily tires with minimal physical activity, such as walking only a short distance. The person feels comfortable at rest but avoids moderate activity.	0.237 (0.167–0.320)
Decompensated cirrhosis of the liver, severe heart failure	Is short of breath and feels tired when at rest. The person avoids any physical activity, for fear of worsening the breathing problems.	0.324 (0.233–0.436)

Dividing the above symptomatic cirrhosis outcomes by aetiology, and combining with asymptomatic states, the total set of sequelae included in non-fatal estimation of cirrhosis and other chronic liver diseases are as shown in the table below.

Table 14: Comprehensive sequelae for non-fatal estimation of cirrhosis and other chronic liver diseases

Level	Cause name
Level 3	Cirrhosis and other chronic liver diseases

Level 4	Cirrhosis and other chronic liver diseases due to alcohol*<br><br>- Compensated cirrhosis due to alcohol<br>- Decompensated cirrhosis due to alcohol (*3 levels of anaemia, 4 levels of heart failure, neither*)<br>- Decompensated cirrhosis due to alcohol, without anaemia or heart failure<br>- Decompensated cirrhosis due to alcohol, with mild anaemia<br>- Decompensated cirrhosis due to alcohol, with moderate anaemia<br>- Decompensated cirrhosis due to alcohol, with severe anaemia<br>- Decompensated cirrhosis due to alcohol, with medically managed heart failure

	- Decompensated cirrhosis due to alcohol, with mild heart failure<br>- Decompensated cirrhosis due to alcohol, with moderate heart failure<br>- Decompensated cirrhosis due to alcohol, with severe heart failure
Level 4	Chronic hepatitis B including cirrhosis**<br><br>- Compensated cirrhosis due to hepatitis B<br>- Decompensated cirrhosis due to hepatitis B (*3 levels of anaemia, 4 levels of heart failure, neither*)<br>- Decompensated cirrhosis due to hepatitis B, without anaemia or heart failure<br>- Decompensated cirrhosis due to hepatitis B, with mild anaemia<br>- Decompensated cirrhosis due to hepatitis B, with moderate anaemia<br>- Decompensated cirrhosis due to hepatitis B, with severe anaemia<br>- Decompensated cirrhosis due to hepatitis B, with medically managed heart failure<br>- Decompensated cirrhosis due to hepatitis B, with mild heart failure<br>- Decompensated cirrhosis due to hepatitis B, with moderate heart failure<br>- Decompensated cirrhosis due to hepatitis B, with severe heart failure<br>- Chronic hepatitis B without cirrhosis
Level 4	Chronic hepatitis C including cirrhosis**<br><br>- Compensated cirrhosis due to hepatitis C<br>- Decompensated cirrhosis due to hepatitis C (*3 levels of anaemia, 4 levels of heart failure, neither*)<br>- Decompensated cirrhosis due to hepatitis C, without anaemia or heart failure<br>- Decompensated cirrhosis due to hepatitis C, with mild anaemia<br>- Decompensated cirrhosis due to hepatitis C, with moderate anaemia<br>- Decompensated cirrhosis due to hepatitis C, with severe anaemia<br>- Decompensated cirrhosis due to hepatitis C, with medically managed heart failure<br>- Decompensated cirrhosis due to hepatitis C, with mild heart failure<br>- Decompensated cirrhosis due to hepatitis C, with moderate heart failure<br>- Decompensated cirrhosis due to hepatitis C, with severe heart failure<br>- Chronic hepatitis C without cirrhosis
Level 4	Nonalcoholic fatty liver disease including cirrhosis**<br><br>- Compensated cirrhosis due to NASH<br>- Decompensated cirrhosis due to NASH (*3 levels of anaemia, 4 levels of heart failure, neither*)<br>- Decompensated cirrhosis due to NASH, without anaemia or heart failure<br>- Decompensated cirrhosis due to NASH, with mild anaemia<br>- Decompensated cirrhosis due to NASH, with moderate anaemia<br>- Decompensated cirrhosis due to NASH, with severe anaemia<br>- Decompensated cirrhosis due to NASH, with medically managed heart failure<br>- Decompensated cirrhosis due to NASH, with mild heart failure<br>- Decompensated cirrhosis due to NASH, with moderate heart failure<br>- Decompensated cirrhosis due to NASH, with severe heart failure<br>- NAFL/NASH (without cirrhosis)

Level 4	Cirrhosis and other chronic liver diseases due to other causes*<br><br>- Compensated cirrhosis due to other causes<br>- Decompensated cirrhosis due to other causes (*3 levels of anaemia, 4 levels of heart failure, neither*)<br>- Decompensated cirrhosis due to other causes, without anaemia or heart failure<br>- Decompensated cirrhosis due to other causes, with mild anaemia

	- Decompensated cirrhosis due to other causes, with moderate anaemia<br>- Decompensated cirrhosis due to other causes, with severe anaemia<br>- Decompensated cirrhosis due to other causes, with medically managed heart failure<br>- Decompensated cirrhosis due to other causes, with mild heart failure<br>- Decompensated cirrhosis due to other causes, with moderate heart failure<br>- Decompensated cirrhosis due to other causes, with severe heart failure

*Because these causes do not include estimates of pre-cirrhotic precursor states, they represent the prevalence and incidence of cirrhosis due to alcohol and cirrhosis due to other causes, respectively.
**Because these causes include estimates of pre-cirrhotic precursor states, they represent the prevalence and incidence of chronic hepatitis B infection, chronic hepatitis C infection, and NALFD, respectively, including those who have developed cirrhosis and those who have not.

Cocaine use disorders

Flowchart

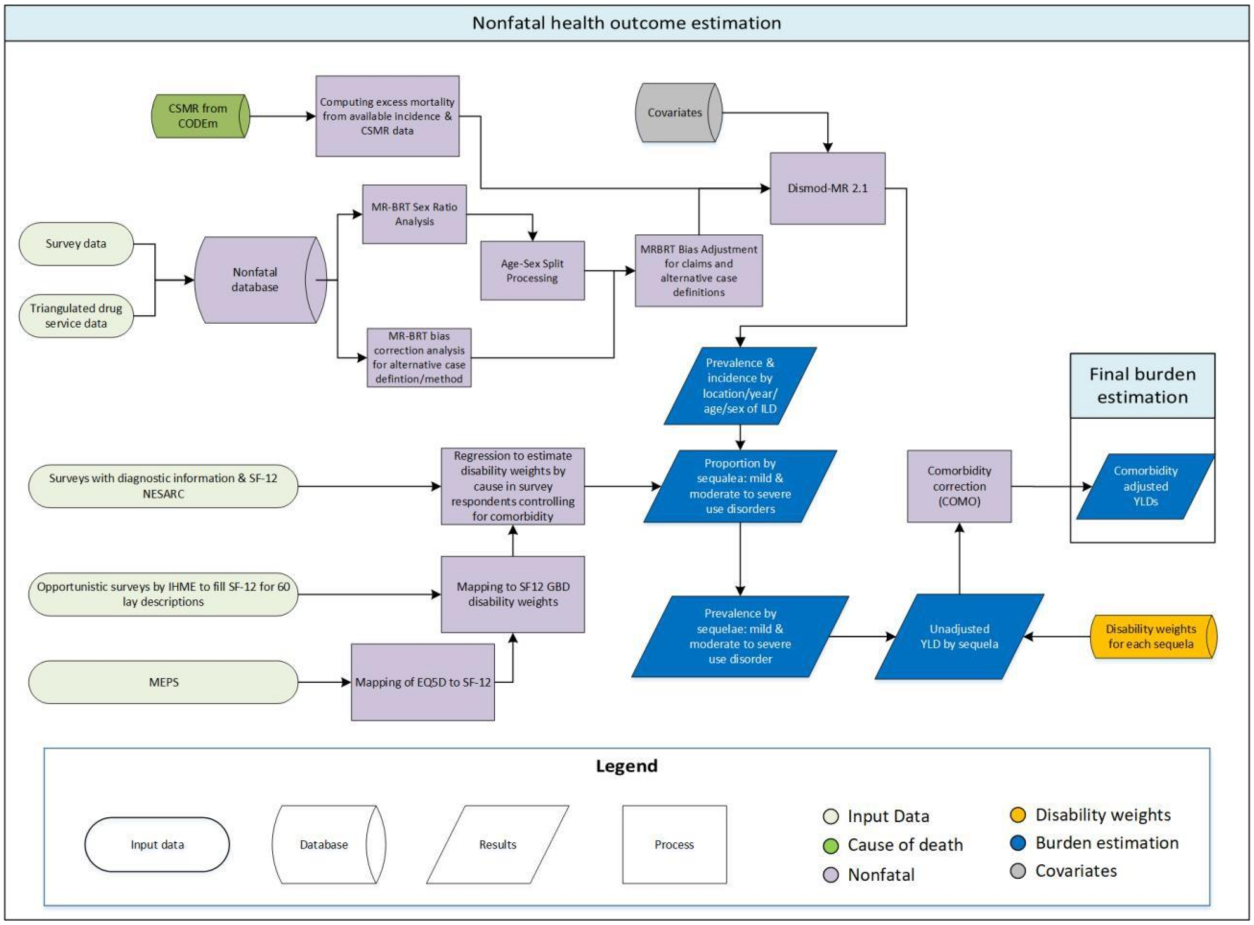

Input data and methodological summary for cocaine use disorders

Case definition

Cocaine dependence is a substance-related disorder involving a dysfunctional pattern of cocaine use. Included in the Global Burden of Disease (GBD) disease modelling were cases meeting the Diagnostic and Statistical Manual of Mental Disorders (DSM-IV-TR) or the International Classification of Diseases (ICD-10) diagnostic criteria for cocaine dependence (DSM: 304.20; ICD: F14.2), excluding those cases due to a general medical condition.[1,2] According to DSM-IV TR criteria, dependence involves a maladaptive pattern of substance use leading to clinically significant impairment or distress. At least three of the following symptoms must be experienced within the same 12-month period:

- Tolerance, characterised by either
  - a need for increased amounts of the substance to achieve intoxication; or
  - markedly diminished effect with continued use of the same amount of the substance;
- Withdrawal, characterised by either
  - withdrawal symptoms characteristic to dependence; or
  - the same (or similar) substance is taken to avoid withdrawal symptoms;
- Substance taken in progressively larger amounts or for a longer period;
- Persistent desire or unsuccessful efforts to reduce substance use;
- Disproportionate time dedicated to obtaining the substance;
- Other important activities are given up because of the substance use; and
- Substance use is continued despite knowledge of physical or psychological problems occurring as a result of the substance.

Input data

There were no major changes to input data for GBD 2021.

For GBD 2010, a systematic review of the literature was conducted in to capture studies of prevalence, incidence, remission, and excess mortality associated with cocaine dependence. In summary, the search was conducted in three stages involving searches of the peer-reviewed literature (via Medline, Embase, and PubMed), the grey literature, and expert consultation. The agreed-upon approach for mental and substance use disorders was to conduct electronic database searches on a rolling basis. All three stages of GBD 2010's literature review were repeated for GBD 2013 to capture additional data published up to 2013. For GBD 2015, stages 2 and 3 of the literature review were updated, and in GBD 2016, the peer-reviewed database search (stage 1) was conducted via Medline, Embase, and PsycINFO to capture studies published from 2013 to 2016. GBD 2017 included additional sources identified by GBD experts and microdata where available. Additionally, in GBD 2017, two targeted systematic reviews were conducted to further supplement the dataset. The first review captured studies within Maori versus non-Maori populations (as opposed to New Zealand more broadly), given the inclusion of these two sub-groups in GBD 2017. The second review utilised the China National Knowledge Infrastructure database to find studies that would not typically be captured in PubMed, Embase, and PsycINFO.

The inclusion criteria stipulated that 1) the publication year must be from 1980 onward; 2) "caseness" must be based on clinical threshold as established by the DSM or ICD; 3) sufficient information must be provided on study method and sample characteristics to assess the quality of the study; and (4) study samples must be representative of the general population (ie, inpatient or pharmacological treatment samples, case studies, veterans or refugee samples were excluded). No limitation was set on the

language of publication. Methods used for this systematic review have been reported in greater detail elsewhere.[3,4]

Table 1: Data Inputs for Cocaine Use Disorders Morbidity Modelling by Parameter

Measure	Total sources	Countries with data
All measures	365	68
Prevalence	353	68
Remission	3	2
Relative risk	2	2
Standardised mortality ratio	3	3
With-condition mortality rate	3	2

Age and sex splitting

Reported estimates of prevalence were split by age and sex where possible. First, if studies reported prevalence for broad age groups by sex (eg, prevalence in 15- to 65-year-old males and females separately), and also by specific age groups for both sexes combined (eg, prevalence in 15- to 30-year-olds, then in 31- to 65-year-olds, for males and females combined), age-specific estimates were split by sex using the reported sex ratio and bounds of uncertainty. Second, prevalence data for both sexes that could not be split using a within-study ratio were split using a sex ratio derived from a meta-analysis of existing sex-specific data using our meta-regression—Bayesian, regularised, trimmed tool[5] (MR-BRT). Details on MR-BRT can be found in appendix 1, section 4.4.1 of the reference article.

The female to male ratio was 0.50 (0.39 to 0.66) for ages 20 and above, and 0.68 (0.51 to 0.89) for ages below 20. Finally, after the application of bias adjustments, where studies reported estimates across age groups spanning 25 years or more, these were split into five-year age groups using the super-region-specific prevalence age pattern estimated by our disease model—Bayesian meta-regression tool[6] (DisMod-MR 2.1) on all data prior to age-splitting. Information on DisMod-MR 2.1 can be found in appendix 1, section 4.5 of the reference article.

Data adjustment

Due to insufficient data in the optimal case definition of cocaine dependence, the prevalence dataset included datapoints of both use and dependence estimated using "direct" or "indirect" survey methods. "Direct" methods of measuring cocaine dependence predominantly involve surveys of the general population that ask if respondents use or are dependent on cocaine. Surveys tend to underestimate the prevalence of the most harmful and stigmatised forms of illicit drug use in ways that probably vary between countries and cultures.[7] "Indirect" methods are considered superior; they use different sources of data to indirectly estimate the total number of drug users (methods include "multiplier methods," back-projection and capture-recapture methods). Due to the lack of data available on cocaine

dependence from indirect survey methods (considered to be the gold standard for GBD purposes),

estimates of use and/or estimates from direct survey methods were also included in the modelling. We marked studies reporting on the prevalence of cocaine dependence obtained via direct methods as well as those reporting on the prevalence of cocaine use obtained via direct methods and derived adjustment factors using MR-BRT. Due to limited overlapping data and roughly similar patterns of use, we combined amphetamine and cocaine data to derive a single adjustment factor. Betas coefficients, in logit space are shown in the table below:

Table 2: MR-BRT Crosswalk Adjustment Factors for Cocaine and Amphetamine Use Disorders

Data input	Reference or alternative case definition	Gamma	Beta Coefficient, Logit (95% UI)*
Cocaine dependence – indirect method	Ref	0.62	---
Cocaine use – indirect method	Alt		1.07 (-0.11 to 2.35)
Cocaine dependence – direct method	Alt		-0.54 (-1.73 to 0.76)
Cocaine use – direct method	Alt		0.54 (-0.65 to 1.81)

**MR-BRT crosswalk adjustments can be interpreted as the factor the alternative case definition is adjusted by to reflect what it would have been had it been measured using the reference case definition. If the log/logit beta coefficient is negative, then the alternative is adjusted up to the reference. If the log/logit beta coefficient is positive, then the alternative is adjusted down to the reference.*

Subsequently, we adjusted for recall period to adjust from one-year recall to point prevalence, again using combined cocaine and amphetamine data. Beta coefficients from MR-BRT are shown in the table below:

Table 3: MR-BRT Crosswalk Adjustment Factors for Cocaine and Amphetamine Use Disorders

Data input	Reference or alternative case definition	Gamma	Beta Coefficient, Logit (95% UI)*
Cocaine dependence point prevalence	Ref	0	---
Cocaine dependence 1-year recall	Alt		0.71 (0.63 to 0.79)

**MR-BRT crosswalk adjustments can be interpreted as the factor the alternative case definition is adjusted by to reflect what it would have been had it been measured using the reference case definition. If the log/logit beta coefficient is negative, then the alternative is adjusted up to the reference. If the log/logit beta coefficient is positive, then the alternative is adjusted down to the reference.*

Modelling strategy

There were no major modelling changes in GBD 2021.

Prior settings in DisMod-MR 2.1 included assuming no incidence, remission, and excess mortality before age 15, and an upper limit of 0.2 on remission. The minimum age of onset was corroborated with expert feedback and existing literature from various sources including the European Monitoring Centre for Drugs and Drug Addiction.[6] These settings were retained for GBD 2021.

As in GBD 2019, LDI was included as a country covariate on EMR with bounds set at -0.5 and -0.1.

Table 4. Covariates. Summary of covariates used in the cocaine use disorders DisMod-MR meta-regression model

Covariate	Parameter	Beta, log (95% Uncertainty Interval)	Exponentiated beta (95% Uncertainty Interval)
LDI ($ per capita)	Excess mortality rate	-0.1 (-0.1 to -0.1)	0.90 (0.90 to 0.90)

Severity and disability

The basis of the GBD disability weight survey assessments are lay descriptions of sequelae highlighting major functional consequences and symptoms. The lay descriptions and disability weights for cocaine dependence severity levels are shown below.

Table 5. Severity distribution, details on the severity levels for cocaine use disorders in GBD 2021 and the associated disability weight (DW) with that severity.

Severity level	Lay description	DW (95% CI)
Mild	Uses cocaine at least once a week and has some difficulty controlling the habit. When not using, the person functions normally.	0.116 (0.074–0.165)
Moderate to severe	Uses cocaine and has difficulty controlling the habit. The person sometimes has mood swings, anxiety, paranoia, hallucinations and sleep problems, and has some difficulty in daily activities.	0.479 (0.324–0.634)

The proportion of people with cocaine dependence within each of the severity levels were determined based on available data from US National Epidemiological Survey on Alcohol and Related Conditions (NESARC), conducted in two waves from 2001 to 2002 and 2004 to 2005.[7] NESARC is a direct household survey. As such, it is expected to underestimate moderate to severe cases of drug dependence. The estimated distribution of cocaine dependent cases by severity were asymptomatic (50%, 37%–64%), mild (25%, 18%–33%), and moderate/severe (25%, 17%–33%).

References

1. American Psychiatric Association. Diagnostic and Statistical Manual of Mental Disorders (DSM-IV-TR). 4th, Text Revision ed Washington DC: American Psychiatric Association. 2000.

2. World Health Organization. The ICD-10 Classification of Mental and Behavioural Disorders. Clinical descriptions and diagnostic guidelines Geneva: World Health Organization. 1992.

3. Degenhardt L, Bucello C, Calabria B, Nelson P, Roberts A, Hall W, et al. What data are available on the extent of illicit drug use and dependence globally? Results of four systematic reviews. Drug and alcohol dependence. 2011.

4. Calabria B, Degenhardt L, Briegleb C, Vos T, Hall W, Lynskey M, et al. Systematic review of prospective studies investigating "remission" from amphetamine, cannabis, cocaine or opioid dependence. Addictive Behaviors. 2010.

5. Murray CJL, Aravkin AY, Zheng P, et al. Global burden of 87 risk factors in 204 countries and territories, 1990–2019: a systematic analysis for the Global Burden of Disease Study 2019. The Lancet 2020; 396: 1223–49. doi: https://doi.org/10.1016/S0140-6736(20)30752-2

6. Vos T, Lim SS, Abbafati C, et al. Global burden of 369 diseases and injuries in 204 countries and territories, 1990–2019: a systematic analysis for the Global Burden of Disease Study 2019. The Lancet 2020; 396: 1204–22. doi: https://doi.org/10.1016/S0140-6736(20)30925-9

7. Reuter P, Trautmann F. A Report on Global Illicit Drugs Markets 1998-2007. Utrecht. 2009.

8. European Montioring Centre for Drugs and Drug Addiction. Lisbon, Portugal 2014.

9. Grant BF, Dawson DA. National Institute on Alcohol Abuse and Alcoholism. Alcohol Health & Research World. 2006; 29(2): p. 74.

Conduct disorder

Flowchart

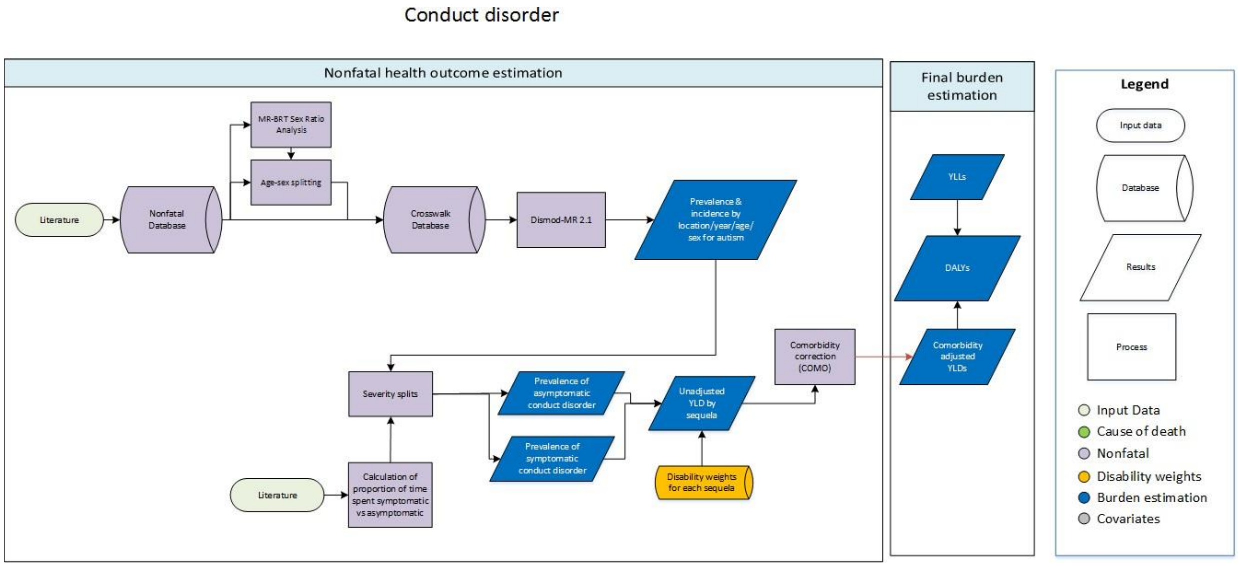

Input data and methodological summary for conduct disorder

Case definition

Conduct disorder (CD) is an externalising behaviour disorder characterised by a pattern of antisocial behaviour that violates the basic rights of others or major age-appropriate societal norms. As per criteria set by the Diagnostic and Statistical Manual of Mental Disorders fourth edition, text revision (DSM-IV-TR),[1] diagnosis requires three or more of the following symptoms to be present in the past 12 months (with at least one present in the last six months) and cause significant impairment in functioning. Symptoms include:

Aggression to people and animals

- often bullies, threatens, or intimidates others
- often initiates physical fights
- has used a weapon that can cause serious physical harm to others (eg, a bat, brick, broken bottle, knife, gun)
- has been physically cruel to people
- has been physically cruel to animals
- has stolen while confronting a victim (eg, mugging, purse snatching, extortion, armed robbery)
- has forced someone into sexual activity

Destruction of property

- has deliberately engaged in fire setting with the intention of causing serious damage
- has deliberately destroyed others' property (other than by fire setting)

Deceitfulness or theft

- has broken into someone else's house, building, or car
- often lies to obtain goods or favors or to avoid obligations (ie, "cons" others)

- has stolen items of nontrivial value without confronting a victim (eg, shoplifting, but without breaking and entering; forgery)

Serious violations of rules

- often stays out at night despite parental prohibitions, beginning before age 13 years
- has run away from home overnight at least twice while living in parental or parental surrogate home (or once without returning for a lengthy period)
- is often truant from school, beginning before age 13 years

CD is considered a disorder of childhood but can be diagnosed in adults who display such behaviours yet do not meet the criteria for antisocial personality disorder. However, there are almost no studies measuring adult CD as existing studies in this area tend to measure adult antisocial behaviour rather than adult CD.[2] As such, only childhood CD (ie, cases prior to 18 years of age) was modelled in GBD.

Included in GBD were cases meeting diagnostic criteria according to DSM[1] or the International Classification of Diseases (ICD).[3] These were identified by the following codes: 312.81-312.89 (DSM-IV-TR) and F91 (ICD-10). Different versions of DSM (DSM-III, DSM-III-R, DSM-IV, DSM-IV-TR, DSM-5, and DSM-5-TR) and ICD (ICD-9, ICD-10, and ICD-11) were accepted.

Input data

The epidemiological systematic literature review for CD was conducted in three stages involving electronic searches of the peer-reviewed literature (ie, via PsycInfo, Embase, and PubMed), the grey literature, and expert consultation. For mental disorders, we update our GBD electronic database searches on a rolling basis. An electronic search was not required for GBD 2021. The next update will be conducted in the next round of GBD. The grey literature searches and expert consultation were conducted for GBD 2021.

The GBD inclusion criteria stipulated that: 1) the publication year must be from 1980 onward; 2) "caseness" must be based on clinical threshold as established by the DSM or ICD; 3) sufficient information must be provided on study method and sample characteristics to assess the quality of the study; and 4) study sample must be representative of the general population (ie, inpatient or pharmacological treatment samples, case studies, veterans, or refugee samples were excluded). No limitation was set on the language of publications. Methods used in previous systematic reviews have been reported in greater detail elsewhere.[2] Table 1 below summarises data inputs by parameter for conduct disorders.

Table 1: Data Inputs for CD morbidity modelling by parameter

Parameter	Countries with data	New sources	Total sources
Incidence	1	0	1
Prevalence	23	0	46
Remission	3	1	3
Other	1	0	2

Age-sex splitting

The extracted data underwent two types of age-sex splitting processes:

11. Where possible, estimates were further split by sex and age based on the available data. For instance, if studies reported prevalence for broad age groups by sex (eg, prevalence in 15–65-year-old males and females separately), and also by specific age groups but for both sexes combined (eg, prevalence in 15–30-year-olds, then in 31–65-year-olds, for males and females

combined); age-specific estimates were split by sex using the reported sex-ratio and bounds of uncertainty.
12. A meta-regression—Bayesian, regularised, trimmed (MR-BRT) analysis was used to split the remaining both-sex estimates in the dataset. For each parameter, sex-specific estimates were matched by location, age, and year. A MR-BRT network meta-analysis was then used to estimate pooled sex ratios and bounds of uncertainty. The male-to-female ratio estimated was 2.38 (95% uncertainty interval [UI]: 0.68–4.07).

Bias corrections/crosswalks

No crosswalks were applied to the estimates for CD.

Modelling strategy

We have made no substantive changes in the modelling strategy from GBD 2019.

After the above data processes were applied, DisMod MR 2.1 was used to model the epidemiological data for CD. Adjustments to model priors or the dataset were made where appropriate. Where outliers were identified in the data, we reassessed the study's methodology and quality before a decision was made to exclude or include the data.

Data across all epidemiological parameters were initially included in the modelling process. We assumed no incidence or prevalence prior to 5 years of age or after 18 years of age. The minimum age of onset was set in consultation with experts while the upper age limit was set in line with DSM criteria. Excess mortality was set to zero given the absence of data demonstrating an association between CD and an increased risk of death. Remission and incidence were capped between ages 4 and 17 years in order to gain a more plausible output.

Severity splits and disability weight

The GBD disability weight survey assessments include lay descriptions of sequelae highlighting major functional consequences and symptoms. The lay description and disability weight for CD is shown in Table 2. A severity split for the proportion of time spent symptomatic versus asymptomatic was based on data from the Great Smoky Mountains Study which assessed the levels of disability found in children and adolescents with mental disorders.[4] Of those with CD 72% reported disability, while 20% of individuals with no diagnosis reported disability at the time of survey. Using these as estimates of the proportion of time with disability in the "average case", the proportion of disability in children without a diagnosis was subtracted from the proportion with disability for CD, giving an adjusted proportion of 52%. Detailed descriptions of this methodology have been published elsewhere.[5] The lay description and disability weight for CD is shown in Table 2.

Table 2. Lay description for CD in GBD 2021 and the associated disability weight

**Lay description**	**Disability weight (95% UI)**
Has frequent behaviour problems, which are sometimes violent. The person often has difficulty interacting with other people and feels irritable.	0.241 (0.159–0.341)

There were no significant changes in GBD 2021 results for dysthymia compared to GBD 2019. While we continue to improve on the data and methods used to estimate the burden of mental disorders, some

challenges need to be acknowledged. Firstly, we still have a large number of locations with no high-quality

raw data available. Secondly, it is difficult to quantify and remove all variation due to measurement error in our epidemiological estimates. While we have improved the methodology used to account for known sources of bias, in some cases, we still have very few datapoints to inform these adjustments. Thirdly, there is a paucity of research on the risk factors of mental disorders which can be used as predictive covariates in our epidemiological models.

References

1. American Psychiatric Association. Diagnostic and Statistical Manual of Mental Disorders (DSM-IV-TR). 4th, Text Revision ed. Washington DC: American Psychiatric Association; 2000.
2. Erskine HE, Ferrari AJ, Nelson P, et al. Research Review: Epidemiological modelling of attention-deficit/hyperactivity disorder and conduct disorder for the Global Burden of Disease Study 2010. *Journal of Child Psychology and Psychiatry* 2013; **54**(12): 1263-74.
3. World Health Organization. ICD-10 Classification of Mental and Behavioural Disorders: Clinical descriptions and diagnostic guidelines. Geneva: World Health Organization; 1992.
4. Ezpeleta L, Keeler G, Erkanli A, Costello EJ, Angold A. Epidemiology of Psychiatric Disability in Childhood and Adolescence. *J Child Psychol Psychiatry* 2001; **42**(7): 901-14.
5. Erskine HE, Ferrari AJ, Polanczyk GV, et al. The global burden of conduct disorder and attention-deficit/hyperactivity disorder in 2010. *Journal of Child Psychology and Psychiatry* 2014; **55**(4): 328-36.

Estimating COVID-19 impact on select infectious syndromes

COVID-19 has strained health-care systems around the world and limited capacity to deliver routine immunisations, priming populations for outbreaks of infectious disease. Conversely, physical distancing measures, masking, and school closures have the potential to interrupt usual transmission patterns of other infectious diseases, as they do for COVID-19. Considering these competing ways in which COVID-19 could influence other diseases, in combination with many countries reporting greatly reduced incidence of influenza and measles, we sought to capture the impact of COVID-19 in our estimates of other infectious diseases for 2020, 2021, and 2022.

Data

We reviewed national-level case notification data from ministry of health websites, media reports, and published literature for measles, pertussis, diphtheria, tetanus, varicella, diarrhoeal disease, influenza, respiratory syncytial virus, and infections due to *S. pneumoniae*, *H. influenzae*, and *N. meningitidis* to look for evidence of disruption. For measles and influenza, we relied on case notifications reported directly by countries to WHO regional offices; these causes had the most complete geographical and temporal coverage. Because of this completeness in reporting, we utilised them as indicator causes for further modelling, as described below.

Modelling

We began by evaluating each cause for evidence of disruption. For each cause, to determine whether a disruption occurred in 2020, we conducted a random effect meta-analysis with restricted maximum likelihood estimation using the metafor package in R. Each point was the ratio of cases observed in 2020 to the cases observed over the average of 2017–2019. Given the relative completeness of measles and influenza data, we developed a primary model for these causes and then, for causes other than measles and influenza, evaluated whether the reduction modelled for measles or influenza

could be applied directly to the other cause. To do this, we examined the change in case notifications

between 2020 and previous years for a cause relative to the change in case notifications between 2020 and previous years for measles and influenza. When determining whether to adjust each cause, we considered the size and statistical significance of the observed effect, the consistency and quality of the available data, and epidemiological plausibility. At the time of estimation, these factors supported adjustment of only pertussis and RSV, using estimates of disruption derived from the influenza disruption model results (see below). As we receive more data, we plan to re-examine additional causes and aetiologies to apply disruption if warranted.

We developed a four-step modelling process to calculate disruption ratios from the COVID-19 pandemic for measles and influenza. First, we interpolated the number of reported cases of influenza and measles in 2020–2022, by month. We leveraged the RegMod framework, a Poisson model that estimates the underlying rate of infection in each month as a function of a seasonal pattern and an underlying temporal trend. The temporal trend was reflected as a piecewise linear spline with knots at the start of each year. We placed the last knot of the underlying time trend in January 2021 for measles and influenza. We used monthly data through March 2022 to fit the model, starting in January 2010 for influenza and January 2011 for measles. The RegMod model results are 1000 sets of estimates of the number of reported cases in each month and inputs to the next phase of modelling. We excluded RegMod results from any country missing at least six months of data in any year within 2017–2021 to reduce the risk of outbreaks occurring and subsiding during the periods of missing data.

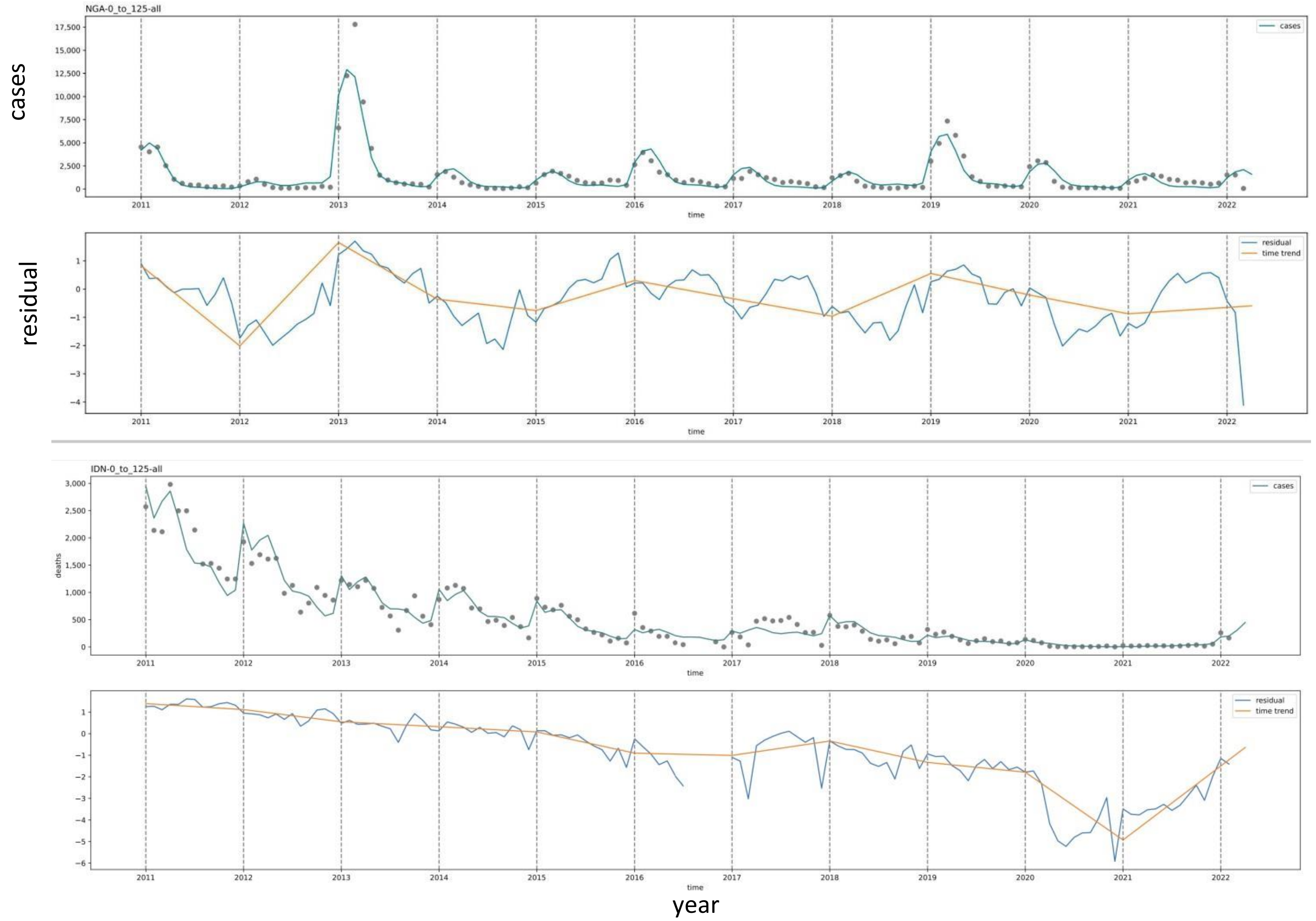

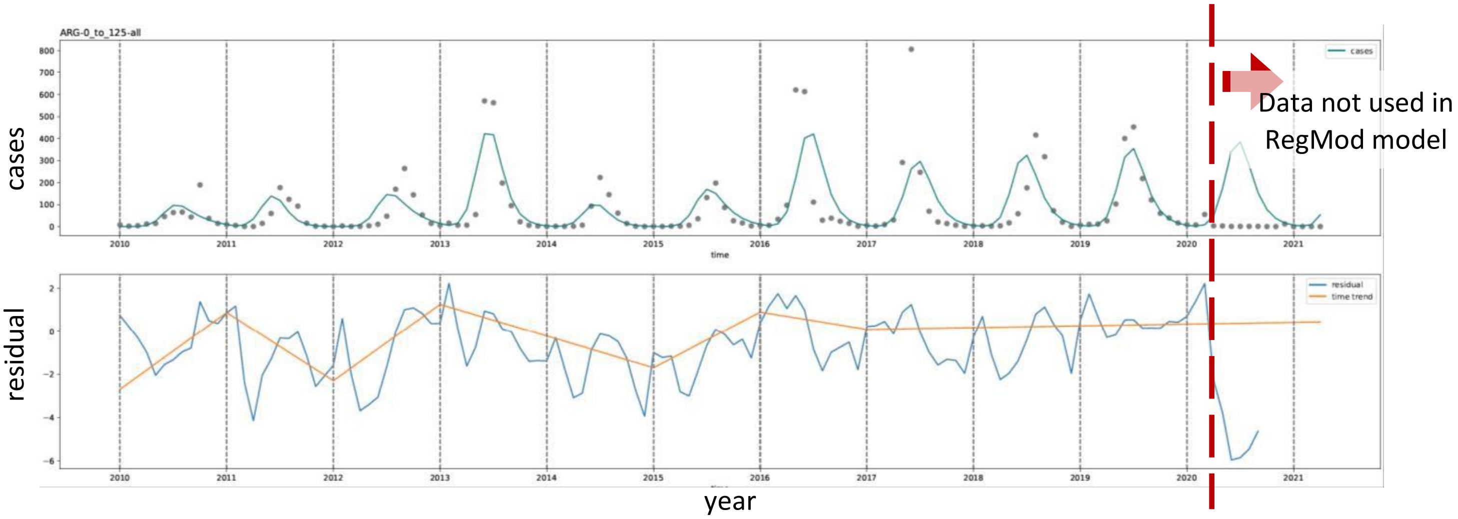

Figure 1: RegMod example for measles in Nigeria (top) and influenza in Indonesia (bottom). For each country-cause, the top panel represents cases over time; points are the observed number of reported cases, and line is the interpolated number of reported cases from the RegMod model. The bottom panel represents the residual over time and the time trend.

In the second step of the modelling process, we calculated the underreporting ratio (URR) implied before the COVID-19 pandemic by comparing RegMod results to GBD COVID-free counterfactual estimates of the true number of cases separately for influenza and measles. For influenza, we used a reference period of 2017–2019 when calculating the URR; for measles, we used 2015–2019. We used a longer period for measles because of greater year-to-year variation in the long-term time trends in cases.

Third, we estimated the COVID-free counterfactual number of reported cases we would have expected during the pandemic years. From the URR and the GBD COVID-free counterfactual estimates, we estimated the COVID-free counterfactual number of reported cases by multiplying the GBD COVID-free estimates by the URR. Lastly, we calculated a disruption ratio by dividing the interpolated number of reported cases from RegMod by the counterfactual COVID-free number of reported cases. We did not calculate disruption values for measles in 2022 due to limited input data availability. This value was calculated by year in all cases except for measles in 2020, where it was calculated by month.

RegMod estimates were produced at the monthly time scale, requiring the conversion of annual estimates of counterfactual reported cases to monthly estimates to allow for the monthly calculation necessary. To account for seasonality, we calculated a seasonality weight for each month for measles. For each month from January 2017 to December 2019, we divided the RegMod measles case estimates from that location-month by the average monthly cases across months in that year. This gave a set of seasonality weights for each location-month, for each year. We then averaged each month's seasonality weight across the three years to yield a three-year average seasonality weight for each location-month. Our monthly counterfactual estimates were produced by dividing the location's annual measles cases by 12 and multiplying by the seasonality weight. For locations without a full time-series of data, we used the average seasonality weight from locations with similar latitude. Disruption ratios were set to 1 for

January, February, and March 2020 to remove the influence of outbreaks observed in early 2020 in the absence of COVID-19 on the overall disruption ratio. We then converted our monthly disruption estimates for measles in 2020 to annual disruption estimates by calculating a seasonality-weighted average of the monthly disruption estimates for each location.

We then modelled the natural log of this standardised disruption ratio (r) for each month (m), disease (d), and country (c) as a function of mask use and mobility disruptions, using a three-stage hierarchical, regularised, trimmed Bayesian meta-regression model.

Table 1. Equations for meta-regression model for measles and influenza

Step 2: Disease incidence disruption versus mobility and mask use	
Global model	$\log(r_{m,d,c}) \sim \beta_{1,d}\, spline(mob_{m,c}) + \beta_{2,d} mask_{m,c}$
Super-region model	$\log(r_{m,d,c}) \sim \beta_{1,d,sr}\, spline(mob_{m,c}) + \beta_{2,d,sr} mask_{m,c}$<br>$\beta_{1,sr,c} \sim N(\beta_{1,d}, \theta_1 \sigma_{1,d}^2)$<br>$\beta_{2,sr,d} \sim N(\beta_{2,d}, \theta_3 \sigma^2_{d}), -3 \le \beta_{2,sr,d} \le 0$
Country model	$\log(r_{m,d,c}) \sim \beta_{1,d,c}\, spline(mob_{m,c}) + \beta_{2,d,c} mask_{m,c}$<br>$\beta_{1,d,c} \sim N(\beta_{1,sr,d}, \theta_2 \sigma_{1,sr,d}^2)$<br>$\beta_{2,d,c} \sim N(\beta_{2,sr,d}, \theta_3 \sigma^2_{d}), -3 \le \beta_{2,d,c} \le 0$

The fitted mask use beta and fitted spline on mobility were passed as Gaussian priors from one level of the cascade to the subsequent level, with a scalar multiplier, θ, on the standard error from the previous level to control the influence of the prior on the next level of the cascade. For mask use, we used θ_3 = 5 for all levels of the cascade. The spline on mobility used θ_1 = 1 between the global and super-region model but passed the spline as a weaker prior from the super-region to country models with θ_2 = 15, allowing for additional country-specific variation in the shape of the relationship between mobility disruption and disease disruption where supported by the data. The beta on mask use was further constrained to be between –3 and 0 at the super-region and country model level. From this model, we obtained 1000 sets of estimates of the disruption ratio for every location-month in 2020, using covariate information and borrowing information from across countries in the region to improve our estimates. The 1000 draws were generated using asymptotic statistics and a lognormal distribution. Table 2 shows the fitted global model coefficients for the mobility splines and mask use for both measles and influenza.

Table 2. Global, cause-specific meta-regression model coefficients for measles and influenza

Global model coefficient	Measles (95% UI)	Influenza (95% UI)
Mobility B-spline coefficient 1	–0.10 (–0.29, 0.09)	3.75e-10 (–0.68, 0.68)
Mobility B-spline coefficient 2	–0.28 (–0.48, –0.09)	–0.026 (–0.48, 0.43)
Mobility B-spline coefficient 3	–0.48 (–0.65, –0.31)	–0.14 (–0.49, 0.21)
Mobility B-spline coefficient 4	–0.59 (–0.82, –0.37)	–0.33 (–0.66, –0.01)
Mobility B-spline coefficient 5	–0.62 (–0.77, –0.48)	–0.51 (–0.79, –0.24)
Mobility B-spline coefficient 6	–0.62 (–0.75, –0.49)	–0.62 (–0.85, –0.38)
Mask use	–1.92 (–2.06, –1.78)	–3.67 (–3.92, –3.40)

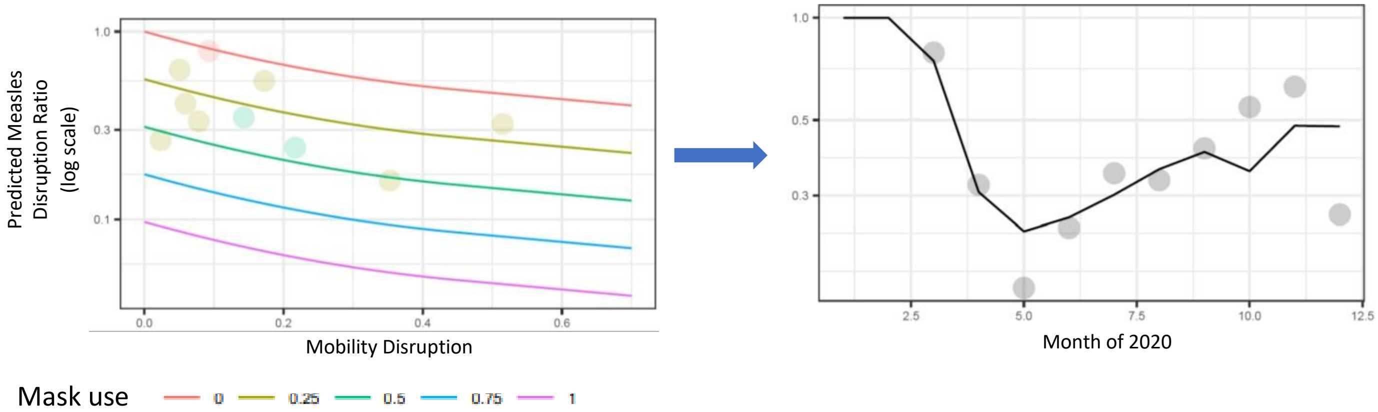

These annual disruption estimates were location-specific. For measles and pertussis, the scalars were age- and sex-agnostic. Because the aetiological fraction of LRI due to RSV and influenza varies by age and sex, these scalars were location-, age-, and sex-specific.

Measles adjustment

For locations in the Latin America and the Caribbean, high-income, and central Europe, eastern Europe, and central Asia super-regions and any locations outside these super-regions with WHO-verified measles elimination, as well as select locations with known strong measles surveillance systems (China and Jordan), we used measles case notifications directly for our burden estimates. These locations are considered “trusted,” and this practice is consistent with our measles incidence estimation framework in years without COVID-19. For all other locations, we scaled measles incidence and prevalence estimates generated using our standard measles estimation approach (described elsewhere in this appendix) with counterfactual estimates of vaccine coverage in the absence of COVID-19 as the vaccine coverage covariate by the measles disruption scalar. At the time of this analysis, there were insufficient data to estimate whether and to what degree COVID-19 may have affected measles case-fatality rates. We therefore used a substantial assumption that COVID-19 did not affect case-fatality rate. Maintaining our usual natural history model framework for measles, fatal estimates were scaled to match the scaling applied to incidence and prevalence. Additional data and analyses will be required in the future to better assess the potential impact of the COVID-19 pandemic on case-fatality rates, including for measles.

LRI adjustment

We conducted a meta-analysis to compare location-specific disruptions for RSV to measles and influenza and found that the disruption in RSV cases in 2020 was analogous to that observed for influenza. For each location/age/sex for which LRI is estimated, influenza and RSV cases were scaled using the annualised ratios as calculated for influenza. Hib-attributed, pneumococcus-attributed, and unattributed cases of LRI were not scaled at this time.

Pertussis adjustment

We conducted a meta-analysis to compare location-specific disruptions for pertussis to measles and influenza and found that the disruption in pertussis cases in 2020 was analogous to that observed for influenza. All locations’ incidence and prevalence estimates for 2020–2022 were scaled using the annualised ratios as calculated for influenza.

Limitations

A key limitation of this framework is that it relies exclusively on case notification data from

national and multinational surveillance networks. It cannot separate the effects of true decreases in

disease incidence from the effects of decreased reporting. Currently, we cannot adjust for the assumption that case notifications reflect true decreases in disease incidence because we do not have any data without changes in reporting, or data on reporting patterns themselves; however, we hope to address this in the future. In addition, we have only adjusted estimates for influenza, measles, RSV, and pertussis in this release due to a scarcity of data. New research also suggests substantial decreases in other LRI and meningitis-causing pathogens, specifically *N. meningitidis*, *S. pneumoniae*, and *H. influenzae*; we plan to incorporate this source, and continue our data seeking, to improve our adjustments for additional diseases in later releases. For years past 2020, additional data and revisions to this modelling framework will be needed to allow for more flexibility in capturing disease resurgence.

For fatal estimates, once created, scalars are applied to an intermediate set of CoDCorrect results (prior to adding shocks) to create a set of positive or negative shocks using the formula below.

$$\text{Shock} = (\text{cc\_draw} * (\text{scalar\_draw} - 1))$$

For non-fatal estimates, once created, scalars are applied to select disease estimates via the central GBD processes EPIC and Burdenator. Further information on those processes can be found elsewhere in the GBD 2021 appendices.

Congenital birth defects

Overview and cause list

This write-up covers the following causes: congenital heart defects, neural tube defects, cleft lip and cleft palate, congenital anomalies of the urogenital system, congenital anomalies of the gastrointestinal tract, musculoskeletal congenital anomalies, congenital chromosomal birth defects (Down syndrome, Turner syndrome, Klinefelter syndrome, and other chromosomal abnormalities, genetic syndromes, and micro-deletions). This appendix will first describe the input data sources and aspects of the modelling strategy that are common to all sub-types of congenital anomalies. We will then provide a description of the case definitions, ICD-10 codes, and health states associated with each of the component congenital causes, as well as the specific modelling strategies employed in each congenital cause, including the model settings, study-level and country-level covariates, and other modelling decisions made.

We have estimated the prevalence and associated disability of the following categories of congenital birth defects (those in bold are GBD causes):

1. **Neural tube defects**
   a. Anencephaly
   b. Encephalocele
   c. Spina bifida
2. **Congenital heart defects**
   a. Single ventricle and single ventricle pathway defects
   b. Complex congenital heart defects excluding single ventricle and single ventricle pathway defects
   c. Malformations of great vessels, congenital valvular heart

disease, and patent ductus arteriosus

   d. Ventricular septal defect and atrial septal defect
   e. Other congenital cardiovascular anomalies

3. **Orofacial clefts: Cleft lip and cleft palate**
4. **Total chromosomal congenital birth defects**
   a. **Down syndrome**
   b. **Turner syndrome**
   c. **Klinefelter syndrome**

   - **d. Other chromosomal abnormalities, genetic syndromes, and micro-deletions**
      - i. Edwards syndrome and Patau syndrome
      - ii. Other chromosomal abnormalities, genetic syndromes, and micro-deletions

5. **Congenital anomalies of the urogenital system**
   - a. Congenital urinary anomalies
   - b. Congenital genital anomalies
6. **Congenital anomalies of the digestive system**
   - a. Congenital diaphragmatic hernia
   - b. Congenital malformations of the abdominal wall
   - c. Congenital atresia and/or stenosis of the gastrointestinal tract
   - d. Other congenital malformations of the gastrointestinal tract
7. **Musculoskeletal congenital anomalies**
   - a. Polydactyly and syndactyly
   - b. Limb reduction defects
   - c. Other musculoskeletal congenital anomalies
8. **Other congenital anomalies:** all birth defects (excluding minor anomalies) not contained in the other categories

Overall flowchart

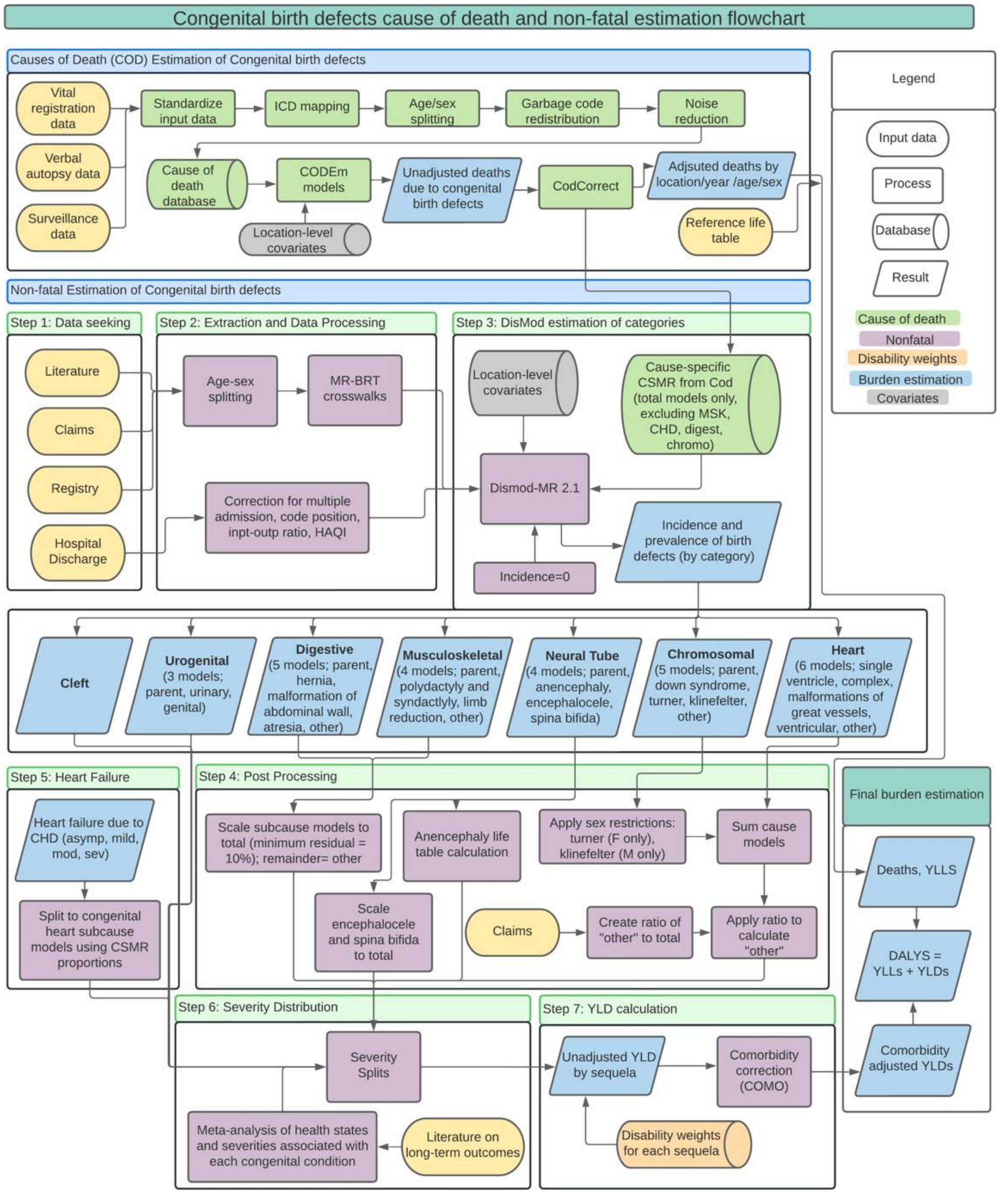

Registry flow chart

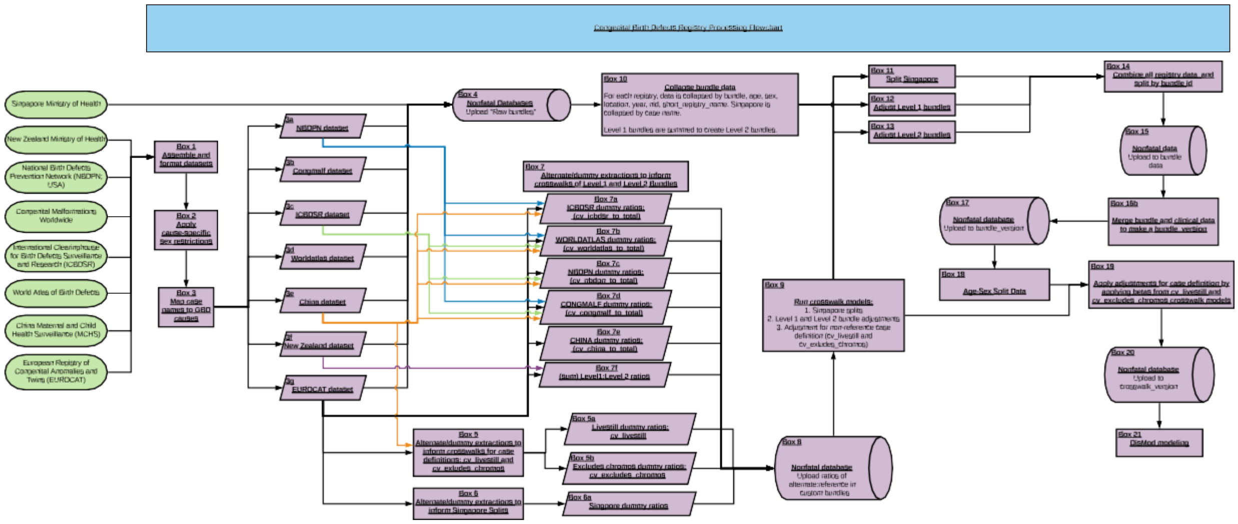

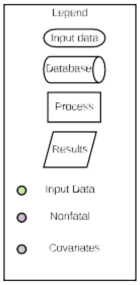

Level 1 (subcause) and Level 2 (parent) bundle list

Congenital muskuloskeletal anomalies
Level 2 bundle:
- 602 - Total muscoskeletal congenital anomalies

Level 1 bundles:
- 604 - Congenital msk - subcause - Limb reduction deficits
- 606 - Congenital msk - subcause - Polydactyly and syndactyly
- 3778 - Congenital msk - subcause - clubfoot
- 3779 - Congenital msk - subcause - hip dysplasia
- 2972 - Other musculoskeletal congenital anomalies

Neural tube defects
Level 2 bundle:
- 608 - Total neural tube defects

Level 1 bundles:
- 610 - Congenital neural - subcause - Anencephaly
- 612 - Congenital neural - subcause - Encephalocele
- 614 - Congenital neural - subcause - Spina Bifida

Congenital muskuloskeletal anomalies
Level 2 bundle:
- 620 - Total congenital digestive anomalies

Level 1 bundles:
- 622 - Congenital digestive - subcause - Congenital diaphragmatic hernia
- 624 - Congenital digestive - subcause - Congenital malformations of the abdominal wall
- 626 - Congenital digestive - subcause - Congenital atresia and/or stenosis of the digestive tract
- 2975 - Other digestive congenital anomalies

Congenital heart disease
Level 2 bundle:
- 628 - Total congenital heart defects

Level 1 bundles:
- 630 - Congenital heart - subcause - Single ventricle and single ventricle pathway heart defects
- 632 - Congenital heart - subcause - Critical malformations of great vessels, congenital valvular heart disease and patent ductus arteriosis
- 634 - Congenital heart - subcause - Ventricular septal defect and atrial septal defect
- 636 - Congenital heart - subcause - Severe congenital heart defects excluding single ventricle and single ventricle pathway
- 2978 - Other congenital cardiovascular defects

Congenital chromosomal anomalies
Level 2 bundle:
- 3029 - Total chromosomal congenital anomalies

Level 1 bundles:
- 436 - Congenital chromo -subcause - Down syndrome
- 437 - Congenital chromo - subcause - Turner syndrome
- 438 - Congenital chromo - subcause - Klinefelter syndrome
- 638 - Congenital chromo - subcause - Edward Syndrome and Patau Syndrome
- 439 - Other chromosomal anomalies, genetic syndrome and micro deletions

Congenital urogenital anomalies
Level 1 bundles:
616 - Congenital urinary anomalies
618 - Congenital genital anomalies

Orofacial clefts
Level 1 bundle:
435 - Orofacial clefts

Other Level 1 Bundles not currently directly used in congenital birth defects modeling:
3188 - Other congenital birth defects
248 - Congenital cataract
3065 - Congenital hearing loss

Case definition

The GBD case definition of congenital anomalies includes any condition present at birth that is a result of abnormalities of embryonic development, excluding those that are directly the result of infections or substance abuse (eg, fetal alcohol syndrome, congenital syphilis), modelled elsewhere in GBD, and excludes minor anomalies as they are defined by European Surveillance of Congenital Anomalies (EUROCAT).

Input data

Several types of data sources are used in the estimation of congenital anomalies: literature prevalence; with-condition mortality and excess mortality data; birth prevalence and neonatal with-condition mortality data from a number of international birth defects registries and surveillance systems; inpatient hospital and MarketScan claims data (a trusted data source) prepared internally by the GBD research team; and cause-specific mortality estimates produced by the causes of death analysis.

First, we extracted data from a number of international birth defects registries. The International Clearinghouse for Birth Defects Surveillance and Research (ICBDSR) reports birth prevalence from a number of international member registries. The World Atlas Report also published birth prevalence estimates from these international registries prior to the publication of ICBDSR reports. EUROCAT reports the birth prevalence of anomalies for a variety of locations in western Europe as reported by participating member registries. China's Maternal and Child Health Surveillance survey (MCHS) reports birth prevalence and early neonatal mortality data for all subnational locations of China. The National Birth Defects Prevention Network (NBDPN) reports birth prevalence estimates as compiled by a number of subnational registries within the USA. The Birth Defects Registry of India (BDRI) reports congenital anomalies from participating hospitals within India.

Second, we used inpatient hospital and claims data (from the USA, Taiwan [province of China], and Singapore) for all congenital anomalies causes and sub-cause models. These data were prepared centrally by the Clinical Informatics research team and are described in detail in the Clinical Informatics section of this appendix. Four rounds of data bias correction were employed in the processing of clinical data. This included 1) adjustment for readmission, 2) correction of primary diagnoses to all diagnoses, 3) adjustment for inpatient-to-outpatient ratio, and 4) adjustment based on Healthcare Access and Quality Index. Of note, in GBD 2017 we used congenital birth defects data only using the first two corrections, but changed in GBD 2019 and 2021 to use clinical data that had all four corrections applied. This change was facilitated by improvements in analysis of corrections by the Clinical Informatics team and was a change made across GBD. Of note, we also changed the mapping of club foot and hip dysplasia in GBD 2019. Previously they were mapped to "limb reduction defects", but in preparation for disaggregated models (which is planned for the next time they are estimated in GBD), they are now included only in the total for musculoskeletal birth defects.

Third, we included data from a systematic review of the available literature for all types of congenital birth defects that was completed in GBD 2015 by constructing search strings designed to capture information on the prevalence, associated mortality, and long-term health outcomes associated with each sub-category of congenital anomalies. All results were screened – first abstracts, then full-text screenings – to ensure the availability of required information and the representativeness of the reported population, and the exclusion of duplicate data also reported as part of the birth registry data inputs.

Table 1: Data inputs for modelling prevalence of congenital causes

Cause	Countries with data	New sources	Total sources
Congenital birth defects (all measures)	105	124	1908
Prevalence	100	124	1745
With-condition mortality rate	33	0	130
Proportion	25	0	52
Neural tube defects (all measures)	90	88	1546
Prevalence	90	88	1535
With-condition mortality rate	2	0	5
Proportion	3	0	8
Congenital heart anomalies (all measures)	94	112	1661
Prevalence	90	112	1550
With-condition mortality rate	26	0	108
Orofacial clefts (all measures)	89	87	1496
Prevalence	89	87	1494
With-condition mortality rate	1	0	3
Down syndrome (all measures)	77	86	1535
Prevalence	74	86	1507
With-condition mortality rate	9	0	8
Proportion	21	0	21
Turner syndrome (all measures)	49	86	849
Prevalence	49	86	845
encephalocele	1	0	1
Proportion	1	0	3
Klinefelter syndrome (all measures)	45	74	837
Prevalence	45	74	834
Proportion	1	0	3
Other chromosomal abnormalities (all measures)	71	89	1350
Prevalence	69	89	1327
Proportion	22	0	23
Congenital musculoskeletal and limb anomalies (all measures)	90	119	1539
Prevalence	90	119	1536
With-condition mortality rate	2	0	6
Proportion	1	0	2
Urogenital congenital anomalies (all measures)	95	88	1569
Prevalence	95	88	1560
With-condition mortality rate	2	0	3
Proportion	4	0	7
Digestive congenital anomalies (all measures)	78	88	1598
Prevalence	78	88	1578
With-condition mortality rate	8	0	18
Proportion	5	0	7

Data processing

Age-sex splitting

Any data that were not sex-specific or did not fit entirely within GBD age groups were age- and sex-split to fit these groups prior to modelling using empirical age- and sex-patterns derived from previous DisMod-MR 2.1 models of the same condition. This is a change from GBD 2017, when age- and sex-splitting of data was not completed prior to modelling, which had a substantial effect on the magnitude of estimates in those causes for which cause-specific mortality rate (CSMR) data were used in modelling. This is described further below.

Crosswalks in MR-BRT

A number of the input data sources used for the estimation of congenital birth defects are known to have biases leading to under-reporting or over-reporting relative to the true prevalence of congenital anomalies among livebirths and all subsequent age groups. We used meta-regression—Bayesian, regularised, trimmed (MR-BRT) to develop statistical models that were used to adjust non-reference data. The alternate definitions that were crosswalked are described below. The specifics of each MR-BRT crosswalk are described below (for "registry to total" crosswalks) and in the corresponding cause-specific sections (for live/stillbirth and exclusion of chromosomal conditions crosswalks).

Live/stillbirths: Where necessary, we used a crosswalk to adjust for the inclusion of stillbirths in the reported birth prevalence estimates in literature and registry data sources, as stillbirths are not included in our case definition of prevalence among livebirths. Each of these crosswalks used a log-transformed neonatal mortality rate as a dose-response (spline/linear) covariate in the crosswalks.

Exclusion of chromosomal conditions: Some sources report birth defects in isolation (ie, excluding any persons who have a coexisting genetic or chromosomal disorder). Our reference definition is the inclusion of chromosomal diagnoses. These splines did not consider any additional covariates.

Registry to total: For a subset of congenital causes, particularly the congenital heart defects, we noted substantial differences in the lists of case definitions being reported to the various congenital registries. For each type of congenital birth defects, registries with the most complete list of reported case definitions – ie, the highest case ascertainment – were used as reference registries and were considered the gold standard for that type of congenital birth defect, or modellable entity. For each modellable entity, we used registry-specific crosswalks to adjust non-gold-standard registries to match the case ascertainment seen in the gold-standard registry. No splines were used in these crosswalks.

Table 2: Crosswalks for data from *Congenital Malformations Worldwide*[1]

Reference registry	Modellable entity name	Gamma	Beta coefficient, log (95% CI)	Adjustment factor*
EUROCAT, China	Total musculoskeletal congenital anomalies	0.043446	–1.659 (–1.752, –1.567)	0.1902 (0.1734, 0.2088)
EUROCAT, China	Total neural tube defects	0	–8.862e-05 (–0.01755, 0.01737)	0.9999 (0.9826, 1.018)
New Zealand, ICBDMS, EUROCAT	Congenital urinary anomalies	0.410011	–0.8497 (–0.9172, –0.7822)	0.4275 (0.3996, 0.4574)
EUROCAT, ICBDMS, NBDPN	Total congenital digestive anomalies	0.007148	–0.1384 (–0.1517, –0.1252)	0.8707 (0.8593, 0.8823)
EUROCAT, ICBDMS	Congenital malformations of the abdominal wall	0.00356	–0.06254 (–0.0806, –0.04448)	0.9394 (0.9226, 0.9565)
NBDPN	Congenital atresia and/or stenosis of the digestive tract	0	–6.641e-21 (–0.01697, 0.01697)	1 (0.9832, 1.017)
EUROCAT	Total congenital heart defects	0.043003	–1.716 (–1.806, –1.626)	0.1798 (0.1643, 0.1968)

[1] Congenital Malformations Worldwide: A Report from the International Clearinghouse for Birth Defects Monitoring Systems | GHDx. http://internal-ghdx.healthdata.org/record/congenital-malformations-worldwide-report-international-clearinghouse-birth-defects (accessed April 21,

2021).

EUROCAT, NBDPN	Single ventricle and single ventricle pathway heart defects	0.039983	–0.4149 (–0.4979, –0.3318)	0.6604 (0.6078, 0.7176)
EUROCAT, NBDPN	Severe congenital heart defects excluding single ventricle and single ventricle pathway	0.011641	–0.6259 (–0.6711, –0.5807)	0.5348 (0.5112, 0.5595)
EUROCAT, China	Total chromosomal congenital anomalies	0.046543	–0.6162 (–0.7063, –0.5262)	0.54 (0.4935, 0.5909)

Table 3: Crosswalks for data from *International Clearinghouse for Birth Defects Monitoring System (ICBDMS)*[1]

Reference registry	Modellable entity name	Gamma	Beta coefficient, log (95% CI)	Adjustment factor*
EUROCAT, China	Total musculoskeletal congenital anomalies	0.076511	–1.393 (–1.506, –1.279)	0.2484 (0.2217, 0.2784)
EUROCAT, New Zealand, China	Polydactyly and syndactyly	0.008397	–0.3163 (–0.3577, –0.2749)	0.7288 (0.6993, 0.7596)
EUROCAT, China	Total neural tube defects	0	–8.862e-05 (–0.01755, 0.01737)	0.9999 (0.9826, 1.018)
EUROCAT	Total congenital heart defects	0.03719	–1.426 (–1.51, –1.342)	0.2402 (0.2209, 0.2612)
EUROCAT, NBDPN	Single ventricle and single ventricle pathway heart defects	0.039983	–0.4149 (–0.4979, –0.3318)	0.6604 (0.6078, 0.7176)
EUROCAT, NBDPN	Critical malformations of great vessels, congenital valvular heart disease, and patent ductus arteriosus	0.028993	–0.5232 (–0.5936, –0.4529)	0.5926 (0.5523, 0.6358)
EUROCAT, NBDPN	Severe congenital heart defects excluding single ventricle and single ventricle pathway	0.025623	–0.3316 (–0.3952, –0.268)	0.7178 (0.6735, 0.7649)
EUROCAT, China	Total chromosomal congenital anomalies	0.046543	–0.6162 (–0.7063, –0.5262)	0.54 (0.4935, 0.5909)

Table 4: Crosswalks for data from *NBDPN*[2]

Reference registry	Modellable entity name	Gamma	Beta coefficient, log (95% CI)	Adjustment factor*
EUROCAT, China	Total musculoskeletal congenital anomalies	0.018925	–1.008 (–1.065, –0.9506)	0.3649 (0.3446, 0.3865)
EUROCAT, China	Total neural tube defects	0	–8.862e-05 (–0.01755, 0.01737)	0.9999 (0.9826, 1.018)
EUROCAT, ICBDMS	Congenital malformations of the abdominal wall	0.00356	–0.06254 (–0.0806, –0.04448)	0.9394 (0.9226, 0.9565)
EUROCAT	Total congenital heart defects	0.000931	0.07634 (0.06148, 0.09121)	1.079 (1.063, 1.096)
EUROCAT, China	Total chromosomal congenital anomalies	0.046895	–0.5862 (–0.6766, –0.4957)	0.5565 (0.5083, 0.6091)

Table 5: Crosswalks for data from *New Zealand Birth defects registry*[3]

[1] International Clearinghouse for Birth Defects Surveillance and Research | GHDx. http://internal-ghdx.healthdata.org/series/international-clearinghouse-birth-defects-surveillance-and-research (accessed April 21, 2021).

[2] United States National Birth Defects Prevention Network (NBDPN) | GHDx. http://internal-ghdx.healthdata.org/series/united-states-national-birth-defects-prevention-network-nbdpn (accessed April 21, 2021).

[3] New Zealand Birth Defects Registry (NZBDR) | GHDx. http://internal-ghdx.healthdata.org/series/new-zealand-birth-defects-registry-nzbdr (accessed April 21,

2021).

Reference registry	Modellable entity name	Gamma	Beta coefficient, log (95% CI)	Adjustment factor*
EUROCAT, China	Total musculoskeletal congenital anomalies	0.023344	–0.7577 (–0.815, –0.7003)	0.4688 (0.4426, 0.4964)
EUROCAT, China	Total neural tube defects	0.020533	–0.7704 (–0.8242, –0.7165)	0.4628 (0.4386, 0.4884)
EUROCAT	Total congenital heart defects	0.010986	–0.5204 (–0.5605, –0.4803)	0.5943 (0.5709, 0.6186)
EUROCAT, NBDPN	Single ventricle and single ventricle pathway heart defects	0.010111	–0.682 (–0.7232, –0.6407)	0.5056 (0.4852, 0.5269)
EUROCAT, NBDPN	Critical malformations of great vessels, congenital valvular heart disease, and patent ductus arteriosus	0.01217	–0.7667 (–0.8119, –0.7215)	0.4645 (0.444, 0.486)
EUROCAT, NBDPN	Severe congenital heart defects excluding single ventricle and single ventricle pathway	0.01263	–0.9006 (–0.9483, –0.853)	0.4063 (0.3874, 0.4261)
EUROCAT, China	Total chromosomal congenital anomalies	0.030019	–0.6302 (–0.6956, –0.5648)	0.5325 (0.4988, 0.5685)

Table 6: Crosswalks for data from *Singapore Birth defects registry*[1]

Reference registry	Modellable entity name	Gamma	Beta coefficient, log (95% CI)	Adjustment factor*
EUROCAT	Down syndrome	0.043064	0.6031 (0.5145, 0.6918)	1.828 (1.673, 1.997)
EUROCAT	Total musculoskeletal congenital anomalies	0.000336	0.05078 (0.04121, 0.06034)	1.052 (1.042, 1.062)
EUROCAT	Limb reduction deficits	0.03955	1.609 (1.518, 1.7)	4.999 (4.565, 5.475)
EUROCAT	Polydactyly and syndactyly	0.014893	1.282 (1.229, 1.334)	3.603 (3.419, 3.796)
EUROCAT	Total neural tube defects	0.032992	0.9013 (0.8212, 0.9813)	2.463 (2.273, 2.668)
EUROCAT	Anencephaly	0.045988	1.471 (1.377, 1.565)	4.354 (3.964, 4.782)
EUROCAT	Encephalocele	0.040592	1.506 (1.417, 1.594)	4.508 (4.126, 4.925)
EUROCAT	Spina bifida	0.025375	1.068 (0.9968, 1.14)	2.911 (2.71, 3.127)
EUROCAT	Congenital diaphragmatic hernia	0.047244	2.253 (2.16, 2.345)	9.512 (8.675, 10.43)
EUROCAT	Congenital malformations of the abdominal wall	0.006371	1.001 (0.9622, 1.039)	2.72 (2.618, 2.827)
EUROCAT	Congenital atresia and/or stenosis of the digestive tract	0.009226	0.5386 (0.4939, 0.5833)	1.714 (1.639, 1.792)
EUROCAT	Single ventricle and single ventricle pathway heart defects	0.045698	2.017 (1.924, 2.11)	7.519 (6.852, 8.252)
EUROCAT	Critical malformations of great vessels, congenital valvular heart disease, and patent ductus arteriosus	0.022149	1.243 (1.178, 1.308)	3.466 (3.247, 3.699)
EUROCAT	Ventricular septal defect and atrial septal defect	0.002317	0.1981 (0.1757, 0.2205)	1.219 (1.192, 1.247)
EUROCAT	Severe congenital heart defects excluding single ventricle and single ventricle pathway	0.036296	1.417 (1.334, 1.5)	4.125 (3.797, 4.482)
EUROCAT	Edward syndrome and Patau syndrome	0.046072	1.525 (1.434, 1.617)	4.597 (4.193, 5.04)

[1] Immigration and Checkpoints Authority (ICA) (Singapore). Singapore Registry of Births and Deaths - Live Births By Birth Order. Singapore, Singapore: Singapore Department of Statistics.

Table 7: Crosswalks for data from *World Atlas of Birth Defects*[1]

Reference registry	Modellable entity name	Gamma	Beta coefficient, log (95% CI)	Adjustment factor*
EUROCAT	Total musculoskeletal congenital anomalies	0.02394	–1.394 (–1.461, –1.328)	0.248 (0.2321, 0.265)
EUROCAT, China	Total neural tube defects	0	–0.1143 (–0.1327, –0.0959)	0.892 (0.8758, 0.9086)
New Zealand, ICBDMS, EUROCAT	Congenital urinary anomalies	0.142379	–0.1339 (–0.1754, –0.09251)	0.8746 (0.8391, 0.9116)
EUROCAT, ICBDMS	Congenital malformations of the abdominal wall	0.00356	–0.06254 (–0.0806, –0.04448)	0.9394 (0.9226, 0.9565)
EUROCAT	Total congenital heart defects	0.03719	–1.426 (–1.51, –1.342)	0.2402 (0.2209, 0.2612)
EUROCAT, NBDPN	Single ventricle and single ventricle pathway heart defects	0.039983	–0.4149 (–0.4979, –0.3318)	0.6604 (0.6078, 0.7176)
EUROCAT, NBDPN	Critical malformations of great vessels, congenital valvular heart disease, and patent ductus arteriosus	0.028993	–0.5232 (–0.5936, –0.4529)	0.5926 (0.5523, 0.6358)
EUROCAT, NBDPN	Severe congenital heart defects excluding single ventricle and single ventricle pathway	0.006351	–0.4172 (–0.4526, –0.3818)	0.6589 (0.636, 0.6826)
EUROCAT, China	Total chromosomal congenital anomalies	0.046543	–0.6162 (–0.7063, –0.5262)	0.54 (0.4935, 0.5909)

Determining outliers and data thresholds

Under-reporting of congenital birth defects is common and can vary by source, location, year, sex, and age. In order to have an empirical, systematic approach to outliering of data, we adapted the non-zero floor approach used by the GBD cause-specific mortality analysis. After all age-sex splitting and crosswalking was complete, the first step was to calculate median absolute deviation (MAD) for the age group of birth, where registry and literature data were combined with all clinical data for the early neonatal age group (0–6 days). The thresholds chosen were –0.5 MAD and +3 MAD, with any data outside of these bounds being identified as outliers. This was determined based on the right-skewed distribution observed in most of the congenital data and the expert prior that under-reporting is far more prevalent than over-reporting – and therefore the bias is asymmetric. In any case where the lower MAD bound was negative, we used a threshold of 0.

For most models, we calculated the MADs using only the EUROCAT data, which we found to be the most reliable source for prevalence of congenital disorders. Exceptions were neural tube defects (all data sources), Urinary birth defects (EUROCAT and USA claims data), musculoskeletal defects (only USA claims data), and chromosomal anomalies, which differed by condition given the high volume of zeroes in the data. For Down syndrome, we used all data. For Edward syndrome and Patau syndrome, we used all non-zero EUROCAT data. For Turner and Klinefelter syndromes, we used EUROCAT data and logged mean absolute deviation and exponentiated this to determine bounds for these data.

To evaluate data for older age groups, we employed two approaches. First, we outliered data from any location-year-source that was outliered for the first stage MAD algorithm. Second, using all clinical and literature data, we developed a model with fixed effects by age to estimated implied MAD bounds for each non-zero age group and again applied the same thresholds of –0.5 MAD and +3 MAD.

[1] European Surveillance of Congenital Anomalies (EUROCAT), International Centre on Birth Defects, World Health Organization (WHO). World Atlas of Birth Defects. 2nd ed. Geneva, Switzerland: World Health Organization (WHO), 2003.

Modelling strategy

Overview

All available input data were utilised in a series DisMod-MR 2.1 models to estimate the prevalence of each category of congenital anomalies across the full life course for each location/age/sex combination. Incidence was set to 0 for all congenital models, as congenital conditions occur at the time of birth and by GBD case definition, congenital cases do not occur after birth. Remission was allowed only in the models of a select subset of causes for which surgical intervention or spontaneous remission can completely eliminate the disability due to that congenital condition. Cause-specific priors and slope priors were used to guide biologically plausible DisMod-MR 2.1 estimates of excess mortality and remission where applicable.

For most of the congenital birth defects causes, we ran DisMod-MR 2.1 models of all defects combined (termed “parent” models). This allowed us to use data on all anomalies within each cause as well as to leverage CSMR results from the GBD cause of death (CoD) analysis. When CSMR data were used as an input, DisMod-MR 2.1 pairs each CSMR datum with a matching prevalence datapoint by age, sex, location, and year. After matching, CSMR is divided by prevalence to calculate an implied excess mortality rate (EMR) datum. All EMR data are then used in driving the model. Of note, EMR data are not calculated when prevalence data are of broader than GBD age groups or are for both sexes combined.

We used CSMR as input to all of the models except congenital heart disease, chromosomal anomalies, digestive anomalies, musculoskeletal birth defects, and urogenital congenital anomalies. For congenital heart defects, the reason is that excess mortality would be underestimated in older ages if CSMR results were used because despite continuing higher rates of mortality through adolescence and adulthood, many of these deaths are not coded as being due to congenital heart disease. Similarly, musculoskeletal and gastrointestinal anomalies estimates for CSMR in older children, adolescents, and adults are much lower than would be suggested by cohort and cross-sectional studies of survival, as few of these deaths are coded as being due to the congenital birth defect present. Finally, for urogenital congenital anomalies, in addition to our modelling urinary and genital anomalies separately, the mechanism of death in older ages will typically be via development of chronic kidney disease, and these deaths are classified in GBD as being due to chronic kidney disease due to other conditions. Details are in each cause-specific section below.

Location-level covariates

Location-level covariates were used in each of the congenital DisMod-MR 2.1 models based on published information about the risk factors for these birth defects. Folic acid availability was used as a covariate on prevalence for all neural tube defects models and a subset of the congenital musculoskeletal anomalies models. A folic acid fortification covariate was used in the neural tube defects and cleft models, which was modelled based on data from the Global Fortification Data Exchange. The legality of abortion was used as a covariate on prevalence for conditions in which prenatal diagnosis is commonly available and the prognosis is severe enough to cause a high rate of termination of pregnancy following prenatal diagnosis: these include all chromosomal conditions and a subset of the congenital heart defects. Maternal consumption of alcohol during pregnancy as a proportion of all pregnancies was used as a covariate on prevalence for all congenital heart defects. The proportion of livebirths by mothers age 35+ was used as a covariate on all chromosomal models. Across many of the congenital models, the Healthcare Access and Quality Index covariate was used to guide the global pattern of with-condition mortality and excess mortality, as was the natural log of the lag-distributed income per capita (LN-LDI). For most of the severe congenital conditions, the mortality

associated with the condition is highly dependent on access to adequate surgical interventions and other medical care during the first hours, weeks, and years of life.

Post-model processing

For those causes with a parent model (neural tube defects), we then squeezed the sum of the specific sub-cause prevalence estimates to these total prevalence estimates to ensure internal consistency of our cause-level and sub-cause estimates. The prevalence of other heart, musculoskeletal, and gastrointestinal anomalies was derived by reducing the total envelope model for each cause by its sub-causes to derive the difference that was attributable to other anomalies in that category.

Assigning health states and sequelae for long-term outcomes

To determine the distribution of health outcomes associated with the congenital causes, we performed a review of available literature on the long-term health outcomes of survivors in cohorts born with each type of congenital malformation. For conditions requiring surgical intervention shortly after birth to ensure survival, the health states included in the disability weight calculations correspond to the post-surgery outcomes reported in cohorts of individuals born with these life-threatening congenital conditions. Where data were available from multiple cohorts, we pooled these cohorts together to calculate the proportion of individuals with each health state. Where data on the joint distribution of the long-term health outcomes was not available, we assumed independence of each long-term health outcome. Combined disability weights were calculated for all necessary combinations of existing disability weights.

Neural tube defects

Neural tube defects (parent)

To ensure internal consistency of the estimates of each sub-type of neural tube defects, we developed a model of the total prevalence of neural tube defects and used these location, year, sex, and age-specific prevalence estimates to scale the estimates of anencephaly, encephalocele, and spina bifida prevalence. This modelling strategy allowed us to incorporate the cause-specific mortality estimates from the GBD CoD analysis and allowed us to use literature data where the prevalence and mortality estimates were reported for the total of all neural tube defects only.

Crosswalks

The MR-BRT crosswalk results are shown below.

Table 1: MR-BRT crosswalk betas for alternate definitions (reference = livebirths including those with chromosomal anomalies)

Crosswalk	Beta	Standard error
Excluding chromosomal diagnoses adjustment	–0.038	0.028

Figure 1: MR-BRT crosswalk of alternate definition (livebirths and stillbirths included) with spline on log-transformed neonatal mortality rate

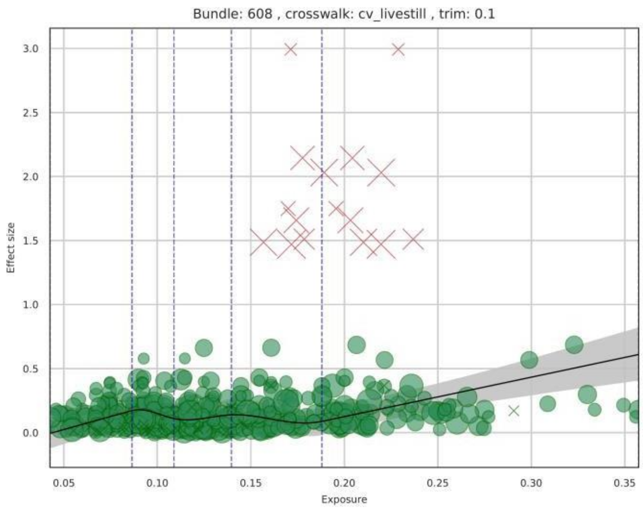

Modelling strategy

The DisMod-MR 2.1 model of total neural tube defects used cause-specific mortality (CSMR) estimates from the GBD CoD analysis for neural tube defects. This model had a minimum excess mortality of 0.5 for the first week of age and a minimum excess mortality of 0.0003 for ages 1–100 years as the risk of death due to neural tube defects is greatest shortly after birth. The model also used an increased smoothness (maximum xi=3) on EMR in order to allow high excess mortality in the early neonatal age group. Random effects on prevalence were limited to 0–0.75 to limit geographical variation in the estimated birth prevalence, and all min coefficient of variation (cv) settings were 0.8.

Table 2. Location-level covariate effects

Covariate name	Measure	Beta value	Exponentiated value
Folic acid unadjusted (µg)	Prevalence	–0.0005 (–0.00064 to –0.00036)	1.00 (1.00–1.00)
Composite fortification standard and folic acid inclusion	Prevalence	–0.31 (–0.35 to –0.27)	0.73 (0.70–0.76)
Healthcare Access and Quality Index	EMR	–0.041 (–0.041 to –0.04)	0.96 (0.96–0.96)

Anencephaly

Case definition and associated health states

Anencephaly is the absence of a major portion of the brain, skull, and scalp. Anencephaly corresponds to the ICD-10 codes Q00.0 and Q00.2. All infants with anencephaly are assigned the health state of severe motor and cognitive impairment.

Crosswalks

The MR-BRT crosswalk results are shown below.

Table 1: MR-BRT crosswalk betas for alternate definitions (reference = livebirths including those with chromosomal anomalies)

Crosswalk	Beta	Standard error
Excluding chromosomal diagnoses adjustment	–0.030	0.163

Figure 1: MR-BRT crosswalk of alternate definition (livebirths and stillbirths included) with spline on log-

transformed neonatal mortality rate

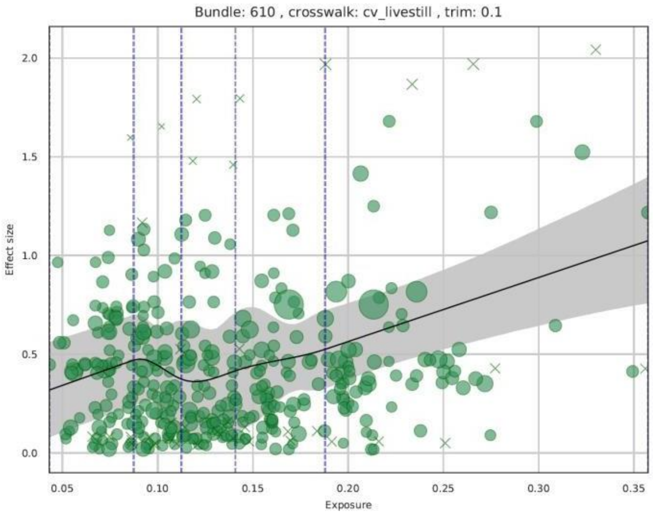

Modelling strategy

The life expectancy for infants born with anencephaly is on the order of hours or days; none of these infants survives past the neonatal age period. Because of the extremely high excess mortality associated with this condition and the short age range over which the prevalence varies, we used a custom modelling process to estimate the prevalence of anencephaly. We first used DisMod-MR 2.1 to model the prevalence of anencephaly at birth for every location, year, age, and sex combination. We then used literature data on outcomes from the largest available cohort of infants born with anencephaly,[1,2] using the precise time of death information from this cohort to create a life table that applied the high EMRs to all cases of anencephaly at birth.

We applied these mortality rates to both sexes and all locations, generating the time lived by infants with anencephaly during the early and late neonatal age groups by location, year, and sex. We then used GBD 2019 mortality estimates to calculate the time lived by all infants during the early and late neonatal age groups by location, year, and sex, and used these two values to calculate the prevalence of anencephaly in the early and late neonatal age groups; after one month of age, all available literature indicates that no infants born with anencephaly are still alive.

The DisMod-MR 2.1 model for the birth prevalence of anencephaly has random effects on prevalence limited to ±0.5. As this model was designed to estimate only the prevalence at birth, incidence, remission, and excess mortality were set to zero for all ages, and the only age mesh points were 0 and 100 years of age.

Table 2. Location-level covariate effects

Covariate name	Measure	Beta value	Exponentiated value
Legality of abortion	Prevalence	–0.0006 (–0.0016 to –0.00003)	1.00 (1.00–1.00)
Folic acid unadjusted (μg)	Prevalence	–0.000096 (–0.00035 to –0.0000042)	1.00 (1.00–1.00)
Composite fortification standard and folic acid inclusion	Prevalence	–0.42 (–0.5 to –0.34)	0.66 (0.61–0.71)

[1] Jaquier M. Anencephaly Online Survey. Anencephaly.info [Internet]. 2006.

[2] Jaquier M, Klein A, Boltshauser E. Spontaneous pregnancy outcome after prenatal diagnosis of anencephaly. *BJOG*. 2006; 113(8): 951-3.

Encephalocele

Case definition and associated health states

Encephalocele is characterised by sac-like protrusions of the brain and meninges through openings in the skull. Encephalocele corresponds to the ICD-10 codes Q01.2, Q01.8, and Q01.9. Our case definitions of spina bifida and encephalocele do not consider surgical intervention for either condition as remission.

Cases of spina bifida and encephalocele are split into every combination of mild, moderate, and severe motor impairment, all severities of intellectual disability, and urinary incontinence. These proportions were calculated using a pooled analysis of available literature on the long-term outcomes in cohorts of individuals born with each subtype of neural tube defects. The distribution of health states associated with encephalocele[1,2,3] was derived separately from the distribution of health states associated with spina bifida,[4,5] although these two categories of neural tube defects are associated with the same list of long-term outcome sequelae.

Crosswalks

The MR-BRT crosswalk results are shown below.

Table 1: MR-BRT crosswalk betas for alternate definitions (reference = livebirths including those with chromosomal anomalies)

Crosswalk	Beta	Standard error
Excluding chromosomal diagnoses adjustment	–0.068	0.074

Figure 1: MR-BRT crosswalk of alternate definition (livebirths and stillbirths included) with spline on log-transformed neonatal mortality rate

[1] Lanton AP. The Characteristics of Patients with Encephaloceles. *Z Kinderchir*. 1990; 45(Suppl 1): 18-9.

[2] Da Silva SL, Jeelani Y, Dang H, Krieger MD, McComb JG. Risk factors for hydrocephalus and neurological deficit in children born with an encephalocele. *J Neurosurg Pediatr*. 2015; 15(4): 392–8.

[3] Lo BWY, Kulkarni AV, Rutka JT, Jea A, Drake JM, Lamberti-Pasculli M, Dirks PB, Thabane L. Clinical predictors of developmental outcome in patients with cephaloceles. *J Neurosurg Pediatr*. 2008; 2(4): 254–7.

[4] Moeini Naghani I, Hashemi Zonouz T, Shahjouei S, Homayoun AA, Nejat F, El Khashab M. Congenital cardiac anomalies in myelomeningocele patients. *Acta Med Acad*. 2014; 43(2): 160–4.

[5] Oakeshott P, Hunt GM, Poulton A, Reid F. Open spina bifida: birth findings predict long-term outcome. *Arch Dis Child*. 2012; 97(5): 474–6.

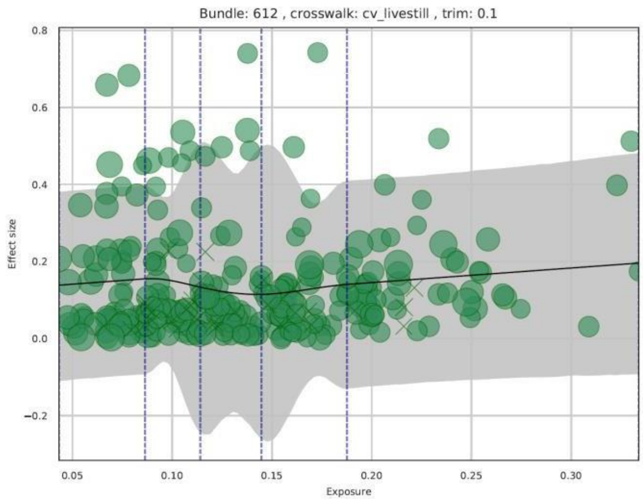

Modelling strategy

The DisMod-MR 2.1 model for encephalocele had a minimum excess mortality prior of 0.2 for the first week of age and a minimum excess mortality prior of 0.0003 for ages 1–54. Excess mortality was restricted to 0–0.1 thereafter, as we believe that those with encephalocele would no longer be dying of this condition past age 55. The model also used an increased smoothness on EMR (maximum xi=3). Random effects on prevalence were limited to ±0.5, as we expect limited geographical variation in the birth prevalence of encephalocele.

Table 2. Location-level covariate effects

Covariate name	Measure	Beta value	Exponentiated value
Legality of abortion	Prevalence	–0.004 (–0.0053 to –0.0027)	1.00 (0.99–1.00)
Folic acid unadjusted (μg)	Prevalence	–0.00054 (–0.00091 to –0.00016)	1.00 (1.00–1.00)
Composite fortification standard and folic acid inclusion	Prevalence	–0.29 (–0.34 to –0.25)	0.75 (0.71–0.78)
Healthcare Access and Quality Index	EMR	–0.025 (–0.049 to –0.001)	0.98 (0.95–1.00)

Spina bifida

Case definition and associated health states

Spina bifida occurs when part of the spinal cord and/or meninges are uncovered by skin. Spina bifida occulta, a much less severe form of spina bifida, in which the defect in vertebral column remains covered by skin, is excluded from the GBD case definition of spina bifida. Spina bifida corresponds to the ICD-10 codes Q05.0, Q05.4, Q05.6, Q05.7, Q05.8, and Q05.9. Our case definitions of spina bifida and encephalocele do not consider surgical intervention for either condition as remission.

Cases of spina bifida and encephalocele are split into every combination of mild, moderate, and severe motor impairment, all severities of intellectual disability, and urinary incontinence. These proportions were calculated using a pooled analysis of available literature on the long-term outcomes in cohorts of individuals born with each subtype of neural tube defects. The distribution of health states associated

with encephalocele[1,2,3] was derived separately from the distribution of health states associated with spina bifida,[4,5] although these two categories of neural tube defects are associated with the same list of long-term outcome sequelae.

Crosswalks

The MR-BRT crosswalk results are shown below.

Table 1: MR-BRT crosswalk betas for alternate definitions (reference = livebirths including those with chromosomal anomalies)

Crosswalk	Beta	Standard error
Excluding chromosomal diagnoses adjustment	–0.0386	0.034

Figure 1: MR-BRT crosswalk of alternate definition (livebirths and stillbirths included) with spline on log-transformed neonatal mortality rate

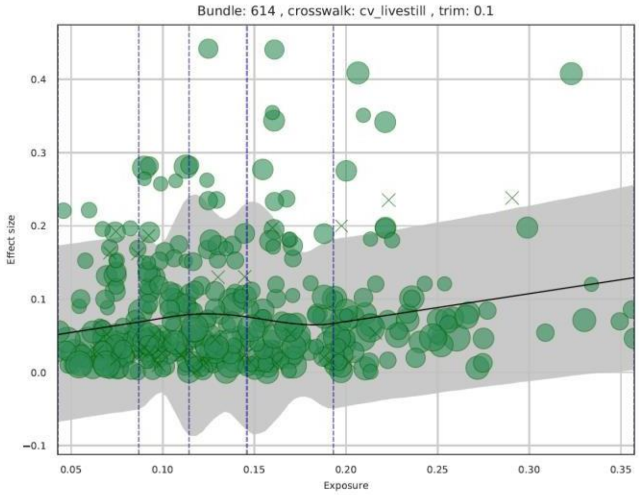

Modelling strategy

---

[1] Lanton AP. The Characteristics of Patients with Encephaloceles. *Z Kinderchir*. 1990; 45(Suppl 1): 18-9.

[2] Da Silva SL, Jeelani Y, Dang H, Krieger MD, McComb JG. Risk factors for hydrocephalus and neurological deficit in children born with an encephalocele. *J Neurosurg Pediatr*. 2015; 15(4): 392–8.

[3] Lo BWY, Kulkarni AV, Rutka JT, Jea A, Drake JM, Lamberti-Pasculli M, Dirks PB, Thabane L. Clinical predictors of developmental outcome in patients with cephaloceles. *J Neurosurg Pediatr*. 2008; 2(4): 254–7.

[4] Moeini Naghani I, Hashemi Zonouz T, Shahjouei S, Homayoun AA, Nejat F, El Khashab M. Congenital cardiac anomalies in myelomeningocele patients. *Acta Med Acad*. 2014; 43(2): 160–4.

[5] Oakeshott P, Hunt GM, Poulton A, Reid F. Open spina bifida: birth findings predict long-term outcome. *Arch Dis Child*. 2012; 97(5): 474–6.

The DisMod-MR 2.1 model for spina bifida had a minimum excess mortality of 0.2 for the first week of age, and a minimum of 0.0002 for ages 1+, and a maximum smoothness on EMR of xi=3. Random effects on prevalence were also limited to ±0.5.

Table 2. Location-level covariate effects

Covariate name	Measure	Beta value	Exponentiated value
Legality of abortion	Prevalence	–0.0078 (–0.0087 to –0.0069)	0.99 (0.99–0.99)
Folic acid unadjusted (μg)	Prevalence	–0.00017 (–0.00045 to –0.0000069)	1.00 (1.00–1.00)
Composite fortification standard and folic acid inclusion	Prevalence	–0.054 (–0.098 to –0.013)	0.95 (0.91–0.99)
Healthcare Access and Quality Index	EMR	–0.025 (–0.049 to –0.00086)	0.98 (0.95–1.00)
Healthcare Access and Quality Index	With-condition mortality rate	–0.052 (–0.064 to –0.041)	0.95 (0.94–0.96)

Post-model processing

Prevalence of spina bifida and encephalocele were summed and scaled to match the total for neural tube defects parent model by location, age group, sex, and year. Age-specific anencephaly prevalence was calculated separately as described above.

Congenital heart anomalies

Summary and associated health states

There are many distinct types of congenital heart anomalies with a range of anatomical patterns, severities, and requirements for medical treatment. For the purpose of estimating non-fatal outcomes, in GBD 2017, congenital heart anomalies were split into five sub-categories based on both the anatomical characteristics and the treatment requirements of each condition:

1. Single ventricle and single ventricle pathway defects
2. Complex congenital heart defects excluding single ventricle and single ventricle pathway defects
3. Malformations of great vessels, congenital valvular heart disease, and patent ductus arteriosus
4. Ventricular septal defect and atrial septal defect
5. Other congenital cardiovascular anomalies

We also began development of a model of total congenital heart anomalies, but this was not used in scaling the sub-causes for GBD 2019. Instead, we used claims data to calculate a ratio of other-to-total, and this was applied to the sum of the other four sub-causes for each location, age group, sex, and year.

Every case of congenital heart defects was associated with a health state of congenital heart disease, except for a proportion of ventricular and atrial septal defects (VSD/ASD) that are considered asymptomatic. All congenital heart defects cases were split into a proportion without intellectual disability and a proportion with every severity from borderline to profound intellectual disability. The proportion of congenital heart anomalies cases experiencing each severity of intellectual disability were calculated using available literature sources on the prevalence and severity of intellectual disability in

congenital heart defect populations.[1,2,3] The proportion of VSD/ASD cases attributed to the asymptomatic category was derived from literature sources on the long-term outcomes of patients diagnosed with septal defects at birth.[4,5,6] GBD estimates of congenital heart failure were assigned to the congenital heart defect categories according to the proportion of total congenital heart cause-specific mortality assigned to each category of congenital heart defects.

Total congenital heart anomalies

Crosswalks

The MR-BRT crosswalk results are shown below.

Table 1: MR-BRT crosswalk betas for alternate definitions (reference = livebirths including those with chromosomal anomalies)

Crosswalk	Beta	Standard error
Excluding chromosomal diagnoses adjustment	–0.099	0.0010

Figure 1: Funnel plot of MR-BRT crosswalk betas for alternate definitions (reference = livebirths including those with chromosomal anomalies)

[1] Riehle-Colarusso T, Autry A, Razzaghi H, Boyle CA, Mahle WT, Van Naarden Braun K, Correa A. Congenital Heart Defects and Receipt of Special Education Services. *Pediatrics*. 2015; 136(3): 496–504.

[2] Menting ME, Cuypers JAAE, Opić P, Utens EMWJ, Witsenburg M, van den Bosch AE, van Domburg RT, Meijboom FJ, Boersma E, Bogers AJJC, Roos-Hesselink JW. The unnatural history of the ventricular septal defect: outcome up to 40 years after surgical closure. *J Am Coll Cardiol*. 2015; 65(18): 1941–51.

[3] Gaynor JW, Stopp C, Wypij D, Andropoulos DB, Atallah J, Atz AM, Beca J, Donofrio MT, Duncan K, Ghanayem NS, Goldberg CS, Hövels-Gürich H, Ichida F, Jacobs JP, Justo R, Latal B, Li JS, Mahle WT, McQuillen PS, Menon SC, Pemberton VL, Pike NA, Pizarro C, Shekerdemian LS, Synnes A, Williams I, Bellinger DC, Newburger JW, International Cardiac Collaborative on Neurodevelopment (ICCON) Investigators. Neurodevelopmental outcomes after cardiac surgery in infancy. *Pediatrics*. 2015; 135(5): 816–25.

[4] Wren C, O'Sullivan JJ. Survival with congenital heart disease and need for follow up in adult life. *Heart*. 2001; 85(4): 438–43.

[5] Gabriel HM, Heger M, Innerhofer P, Zehetgruber M, Mundigler G, Wimmer M, Maurer G, Baumgartner H. Long-term outcome of patients with ventricular septal defect considered not to require surgical closure during childhood. *J Am Coll Cardiol*. 2002; 39(6): 1066–71.

[6] Neumayer U, Stone S, Somerville J. Small ventricular septal defects in adults. *Eur Heart J.* 1998; 19(10): 1573–82.

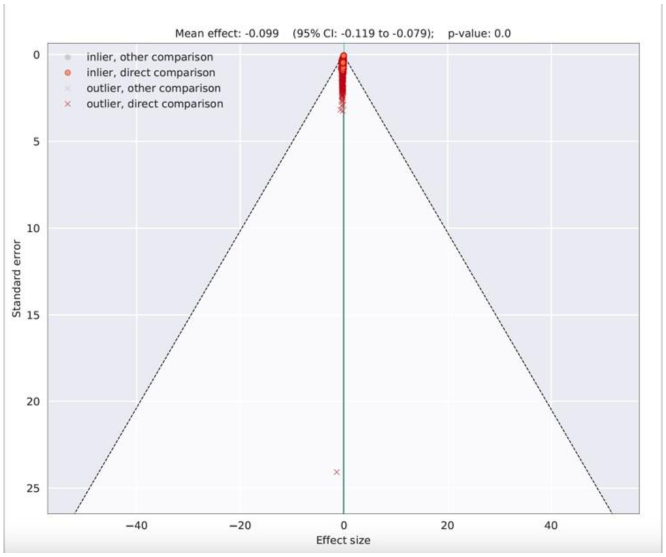

Figure 2: MR-BRT crosswalk of alternate definition (livebirths and stillbirths included) with the log-transformed neonatal mortality rate

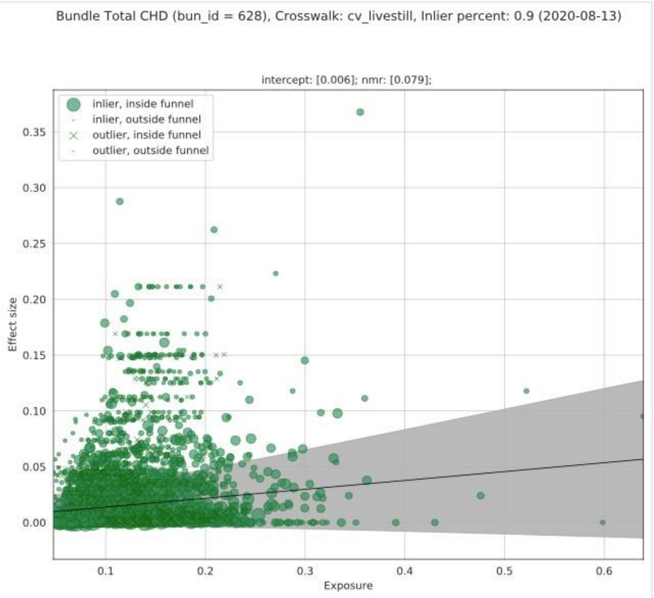

Modelling strategy

In the DisMod-MR 2.1 model of total congenital heart anomalies, random effects on prevalence were limited to ±0.5 to limit geographical variation in the estimates of birth prevalence. The minimum EMR for the neonatal age range was set to 0.1. The smoothness on EMR was increased to xi=3.0 to allow high excess mortality in the neonatal age groups and lower EMRs in older ages.

Table 2. Location-level covariate effects

Covariate name	Measure	Beta value	Exponentiated value
Maternal alcohol consumption during pregnancy (proportion)	Prevalence	0.011 (0.00036 to 0.040)	1.01 (1.00–1.04)
Healthcare Access and Quality Index	Prevalence	0.00020 (0.000047 to 0.00035)	1.00 (1.00–1.00)
Healthcare Access and Quality Index	EMR	–0.025 (–0.048 to –0.0013)	0.98 (0.95–1.00)

Single ventricle and single ventricle pathway defects

Case definition

Single ventricle and single ventricle pathway defects include tricuspid atresia, hypoplastic left heart syndrome, mitral valve atresia, single left ventricle, double outlet right ventricle, and pulmonary atresia; the corresponding ICD-10 codes are Q20.1, Q20.2, Q20.4, Q22.4, Q22.6, and Q23.4. Each of the single ventricle and single ventricle pathway conditions requires surgical intervention shortly after birth to ensure infant survival.

Crosswalks

The MR-BRT crosswalk results are shown below.

Table 1: MR-BRT crosswalk betas for alternate definitions (reference = livebirths including those with chromosomal anomalies)

Crosswalk	Beta	Standard error
Excluding chromosomal diagnoses adjustment	–0.071	0.023

Figure 1: Funnel plot of MR-BRT crosswalk betas for alternate definitions (reference = livebirths including those with chromosomal anomalies)

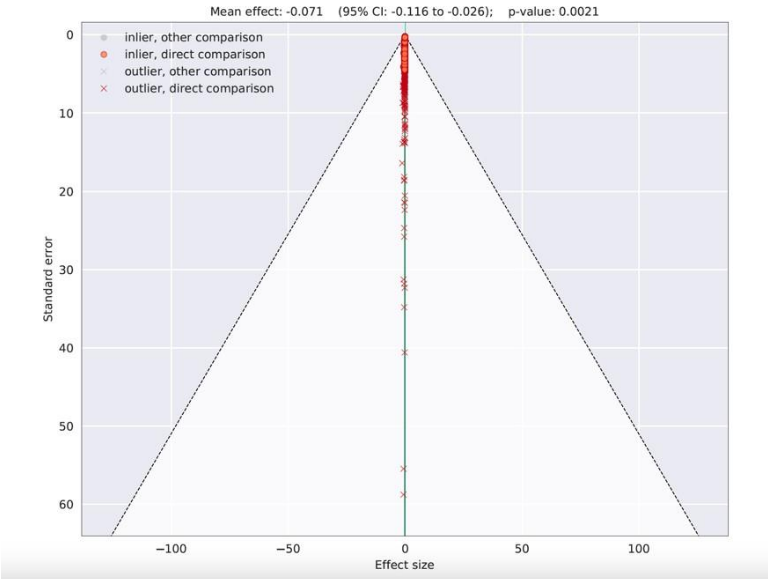

Figure 2: MR-BRT crosswalk of alternate definition (livebirths and stillbirths included) with the log-transformed neonatal mortality rate

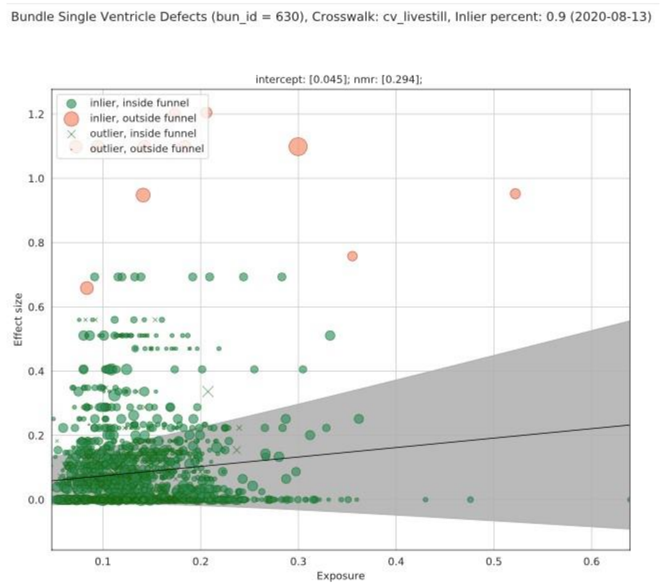

Modelling strategy

In the DisMod-MR 2.1 model of single ventricle and single ventricle pathway heart defects, random effects on prevalence were limited to ±0.5 to limit the estimated geographical variation in birth prevalence. A minimum EMR of 8 was set for the early neonatal period to capture the high mortality risk, based on expert priors and a review of available literature on the mortality risk among infants born with single ventricle and single ventricle pathway heart defects. The smoothness on EMR was set to 5.0 in order to fit steep changes in the EMR during the first weeks of life, as the risk of death due to these congenital heart anomalies is greatest shortly after birth and diminishes over the life course.

Table 2. Location-level covariate effects

Covariate name	Measure	Beta value	Exponentiated value
Maternal alcohol consumption during pregnancy (proportion)	Prevalence	0.061 (0.0038 to 0.14)	1.06 (1.00–1.15)
Healthcare Access and Quality Index	EMR	–0.05 (–0.098 to –0.0024)	0.95 (0.91–1.00)

Complex congenital heart defects excluding single ventricle and single ventricle pathway defects

Case definition

Complex congenital heart defects excluding single ventricle and single ventricle pathway defects include common arterial trunk, common truncus, discordant ventriculo-arterial connection, transposition of great vessels, atrioventricular septal defect, endocardial cushion defect, tetralogy of Fallot, aortopulmonary septal defect, pulmonary valve atresia, congenital stenosis of aortic valve, and total anomalous pulmonary venous connection. This category of severe congenital heart defects includes ICD-10 codes Q20.0, Q20.3, Q21.2, Q21.3, Q21.4, Q22.0, Q23.0, and Q26.2.

Crosswalks

The MR-BRT crosswalk results are shown below.

Table 1: MR-BRT crosswalk betas for alternate definitions (reference = livebirths including those with chromosomal anomalies)

Crosswalk	Beta	Standard error
Excluding chromosomal diagnoses adjustment	–0.191	0.017

Figure 1: Funnel plot of MR-BRT crosswalk betas for alternate definitions (reference = livebirths including those with chromosomal anomalies)

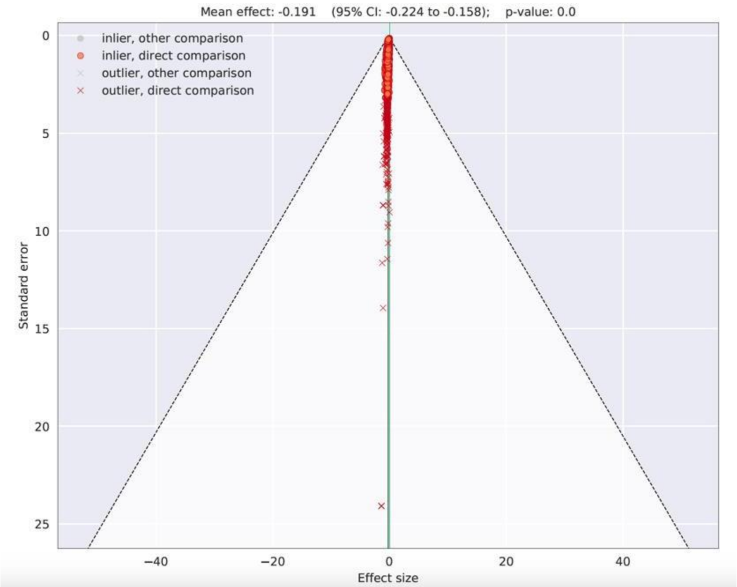

Figure 2: MR-BRT crosswalk of alternate definition (livebirths and stillbirths included) with the log-transformed neonatal mortality rate

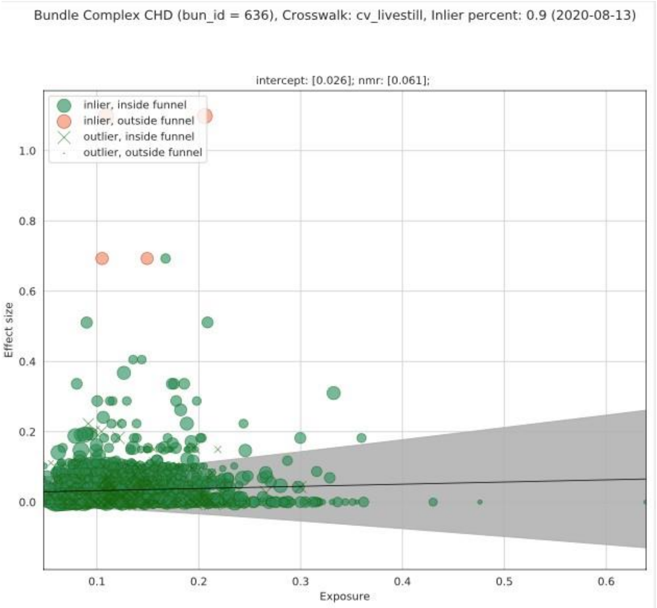

Modelling strategy

In the DisMod-MR 2.1 model of congenital heart defects excluding single ventricle and single ventricle pathway defects, random effects on prevalence were limited to ±0.5. A minimum EMR of 1.0 for the early neonatal period was enforced to capture the high risk of mortality associated with these conditions. The smoothness on EMR was set to i = 3.0 to allow the model to fit steep changes in the

mortality rate of these conditions in the neonatal age period.

Table 2. Location-level covariate effects

Covariate name	Measure	Beta value	Exponentiated value
Maternal alcohol consumption during pregnancy (proportion)	Prevalence	0.25 (0.032 to 0.49)	1.29 (1.03–1.63)
Healthcare Access and Quality Index	EMR	–0.025 (–0.049 to –0.0011)	0.97 (0.95–1.00)

Malformations of great vessels, congenital valvular heart disease, and patent ductus arteriosus

Case definition

The malformations of vessels and valves in this sub-cause category include Ebstein's anomaly, congenital pulmonary valve stenosis, pulmonary valve insufficiency, other malformations of the pulmonary valve, malformations of the tricuspid valve, tricuspid atresia or stenosis, insufficiency of the aortic valve, mitral stenosis or insufficiency, and other malformations of aortic and mitral valves. Patent ductus arteriosus cases are only included among infants of >37 weeks gestational age, as premature infants often have minor patent ductus arteriosus that closes shortly after birth. The ICD-10 codes corresponding to the critical malformations of great vessels category include Q22.1, Q22.2, Q22.3, Q22.5, Q22.8, Q22.9, Q23.1, Q23.2, Q23.3, Q23.8, Q23, Q25.1, Q25.2, Q25.3, Q25.4, Q25.5, and Q25.0. The majority of these conditions require medical attention within the first few weeks of life.

Crosswalks

The MR-BRT crosswalk results are shown below.

Table 1: MR-BRT crosswalk betas for alternate definitions (reference = livebirths including those with chromosomal anomalies)

Crosswalk	Beta	Standard error
Excluding chromosomal diagnoses adjustment	–0.074	0.0096

Figure 1: Funnel plot of MR-BRT crosswalk betas for alternate definitions (reference = livebirths including those with chromosomal anomalies)

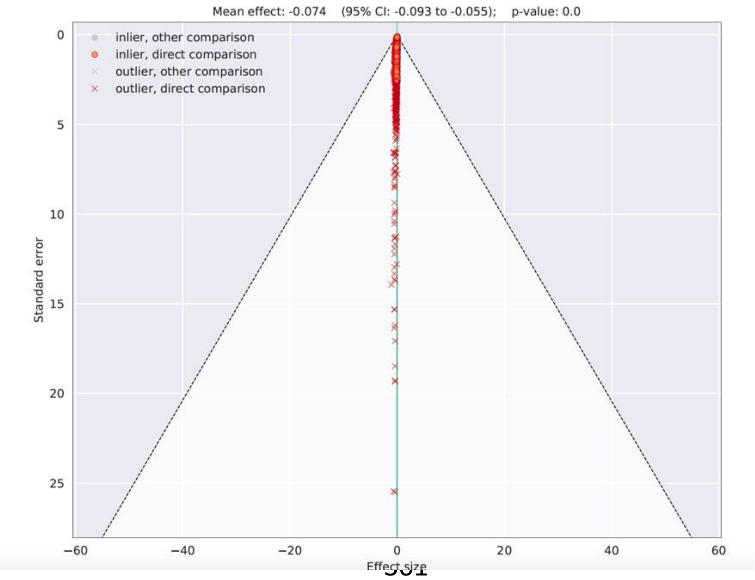

Figure 2: MR-BRT crosswalk of alternate definition (livebirths and stillbirths included) with the log-transformed neonatal mortality rate

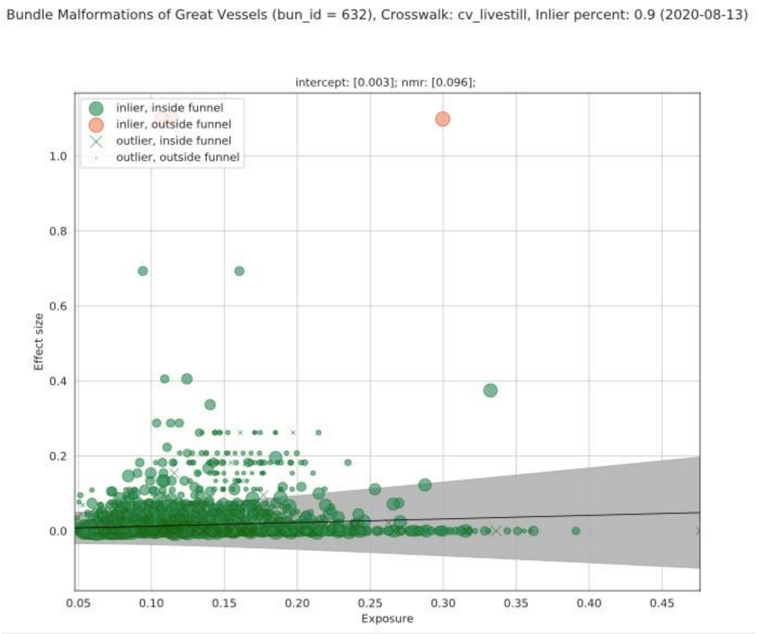

Modelling strategy

In the DisMod-MR 2.1 model of critical malformations of great vessels, congenital valvular heart disease, and patent ductus arteriosus, random effects on prevalence were limited to 0–0.5. A minimum EMR of 1.0 was set for the early neonatal period to capture the high mortality risk associated with these conditions. The smoothness on excess mortality was increased to xi=3.0 to fit steep changes in the mortality associated with these conditions during and after the neonatal period, as the risk of death due to congenital heart anomalies is highest shortly after birth.

Table 2. Location-level covariate effects

Covariate name	Measure	Beta value	Exponentiated value
Maternal alcohol consumption during pregnancy (proportion)	Prevalence	0.091 (0.0027 to 0.23)	1.10 (1.00–1.26)
Healthcare Access and Quality Index	EMR	–0.025 (–0.049 to –0.001)	0.98 (0.95–1.00)

Ventricular septal defects and atrial septal defects

Case definition

Ventricular septal defects and atrial septal defects includes holes in the walls separating the chambers of the heart. Many of these septal defects close spontaneously, while other require surgical care. The ICD-10 codes corresponding to ventricular septal defect and atrial septal defect are Q21.0 and Q21.1, respectively.

Crosswalks

The MR-BRT crosswalk results are shown below.

Table 1: MR-BRT crosswalk betas for alternate definitions (reference = livebirths including those with chromosomal anomalies)

Crosswalk	Beta	Standard error
Excluding chromosomal diagnoses adjustment	–0.089	0.0093

Figure 1: Funnel plot of MR-BRT crosswalk betas for alternate definitions (reference = livebirths including those with chromosomal anomalies)

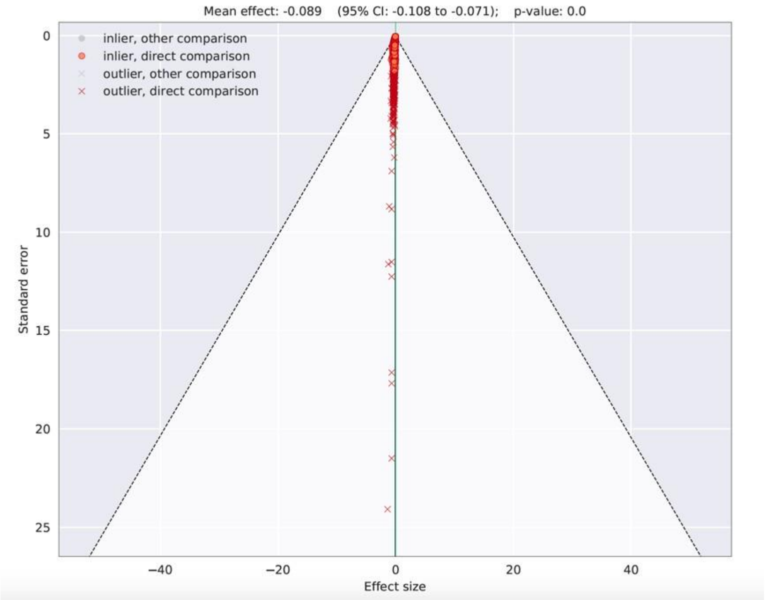

Figure 2: MR-BRT crosswalk of alternate definition (livebirths and stillbirths included) with the log-transformed neonatal mortality rate

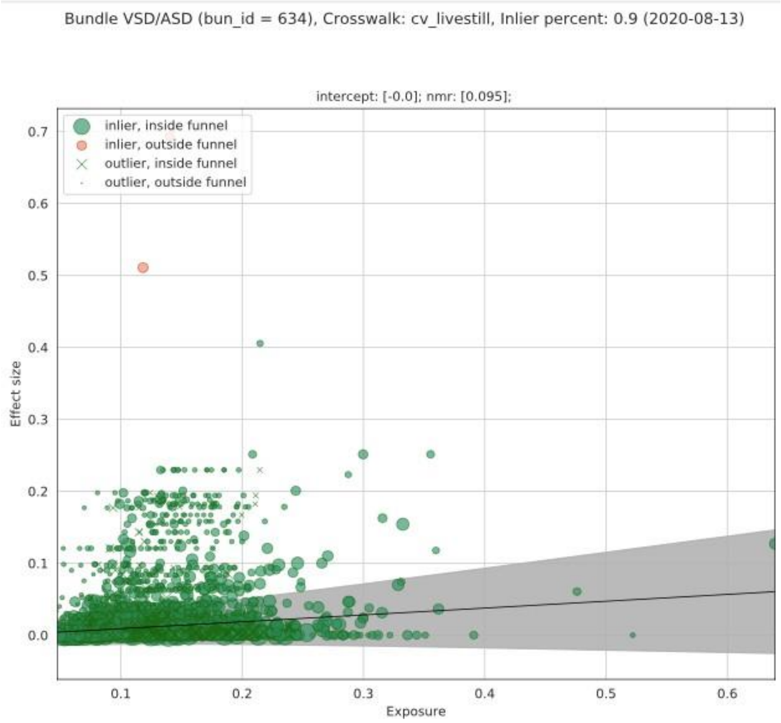

Modelling strategy

In the DisMod-MR 2.1 model of ventricular septal defects and atrial septal defects (VSD/ASD), remission was set 0.05–0.2 for ages 0–5, 0–0.05 for ages 5–10, and 0 for all subsequent ages. Random effects on prevalence were limited to ±0.3 to limit the random geographical variation in the estimated birth prevalence. No minimum EMR was set in this model, as VSD/ASD cases are not associated with EMRs as high as the other sub-types of congenital heart defects. The smoothness on EMR was set to xi=3.0, and a decreasing slope prior was set on remission for all ages, with remission set to 0 past age 10.

Table 2. Location-level covariate effects

Covariate name	Measure	Beta value	Exponentiated value
Maternal alcohol consumption during pregnancy (proportion)	Prevalence	0.0040 (0.00013 to 0.013)	1.00 (1.00–1.01)
Healthcare Access and Quality Index	EMR	–0.026 (–0.05 to –0.002)	0.97 (0.95–1.00)

Other congenital cardiovascular birth defects

Case definition

The fifth and final sub-cause category of congenital heart defects is other congenital cardiovascular anomalies, which correspond to ICD-10 codes Q27, Q27.1, Q27.2, Q27.3, Q27.30, Q27.31, Q27.32, Q27.33, Q27.34, Q27.39, Q27.4, Q27.8, Q27.9, Q28, Q28.0, Q28.1, Q28.2, Q28.3, Q28.8, and Q28.9.

Modelling strategy

Other congenital cardiovascular anomalies are modelled by applying the ratio of other congenital heart anomalies to total congenital heart anomalies as it is reflected in MarketScan data, to the sum of the sub-causes of congenital cardiovascular anomalies. The result is prevalence of other congenital cardiovascular anomalies by age/year/sex/location. Specifically, we use claims data to calculate the proportion of cases that are due to the other causes. To do that, we sum the cases for the specified congenital sub-causes and the other category sub-causes. We divide the number of other sub-cause cases by the total number of cases to obtain the proportion. In order to have a valid proportion, we only use datapoints for which we have the combination of age, sex, location, and year for all sub-causes. We then calculate the prevalence of other: p_other = (p_sum_subcauses / 1-prop_other) - p_sub_subcauses.

Orofacial clefts

Case definition and associated health states

Orofacial clefts include isolated cleft lip, isolated cleft palate, and combined cleft lip and cleft palate. Cleft lip is an opening in the upper lip that may extend into the nose, and with cleft palate, the roof of the mouth contains an opening into the nose. Both conditions are the result of the tissues of the face not joining properly during development. The GBD case definition of orofacial clefts includes isolated cleft palate, which corresponds to ICD-10 codes Q35.2, Q35.3, Q35.5, Q35.6, Q35.7, Q35.8, and Q35.9, and cleft palate with or without cleft lip, which corresponds to ICD-10 codes Q36.0, Q36.1, Q36.9, Q37.1, Q37.5, Q37.8, and Q37.9. Craniofacial clefts that do not include the oropharynx are excluded.

These conditions can be successfully treated by surgery, which is typically done during the first few months or years of life but may occasionally be completed later in life. The sequelae associated with orofacial clefts are disfigurement level 1, disfigurement level 2, and disfigurement level 2 with speech

problems. Additionally, a proportion of the population with orofacial clefts is considered to be asymptomatic. In the absence of data, we assumed the proportion of each is equal.

Crosswalks

The MR-BRT crosswalk results are shown below.

Table 1: MR-BRT crosswalk betas for alternate definitions (reference = livebirths including those with chromosomal anomalies)

Crosswalk	Beta	Standard error
Excluding chromosomal diagnoses adjustment	–0.055	0.012

Figure 1: MR-BRT crosswalk of alternate definition (livebirths and stillbirths included) with spline on log-transformed neonatal mortality rate

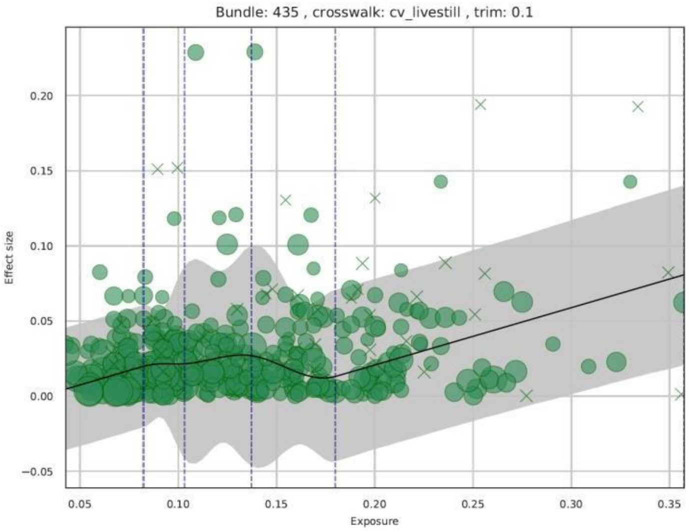

Modelling strategy

The DisMod-MR 2.1 model of orofacial clefts had random effects on prevalence limited to ±0.8, as we expected limited variation in birth prevalence of orofacial clefts. The model settings allow increased smoothness on both EMR and remission (maximum xi=5.0) to fit steep changes in the rates mortality and remission during the first few years of life.

Incidence was set to zero for all ages. Remission was set to zero for the first three months of life, as cleft lip and/or palate are rarely corrected in the first few months of life. A maximum remission of 0.8 was set for ages 3 months to 2 years, the age range in which cleft repair is most commonly performed, allowing up to 75% of cleft cases to be repaired between 3 months and 2 years of age. Remission was bounded from 0 to 0.07 for ages 2–5 years, 0–0.004 for ages 5–20 years, then bounded from 0–0.002 for ages 20–50 years, and set at 0 for ages 50+ years. These limits on remission reflect our priors that up to 20% of remaining cleft cases are repaired between 2 and 5 years of age, another 5% may be repaired between 5 and 20 years of age, and a maximum 5% of remaining cases are surgically repaired between ages 20 and 50 years.

Priors on EMR were set at a maximum of 2.5 for the early neonatal period, 0.01 for ages 5–10, and 0.000001 for ages 10+. These limits on excess mortality reflect our priors that up to 5% of individuals with orofacial clefts die in the first week of life, up to 5% die in the following three weeks, up to 20% die in the next 11 months, another maximum of 20% before 5 years of ages, and a maximum of 5% of the remaining individuals die between ages 5 and 10 years.

Table 2. Location-level covariate effects

Covariate name	Measure	Beta value	Exponentiated value
Healthcare Access and Quality Index	Prevalence	–0.000097 (–0.00019 to –0.000015)	1.00 (1.00–1.00)
Folic acid unadjusted (µg)	Prevalence	–0.00016 (–0.00026 to –0.000071)	1.00 (1.00–1.00)
Composite fortification standard and folic acid inclusion	Prevalence	–0.0077 (–0.014 to –0.0015)	0.99 (0.99–1.00)
LN-LDI (I$ per capita)	EMR	–0.75 (–0.75 to –0.75)	0.47 (0.47–0.47)

Chromosomal anomalies

In addition to Down syndrome, Turner syndrome, and Klinefelter syndrome, hundreds of different types of chromosomal abnormalities and other genetic syndromes have been identified, described, and categorised. Commonalties between genetic syndromes include the predisposition of affected persons to have dysmorphic body features, congenital heart disease, endocrine problems, and neurodevelopmental abnormalities that can lead to intellectual disability. Many of those with chromosomal abnormalities can be readily recognised or suspected by such features. While each has hallmark physical features and diagnostic criteria, most also require sophisticated laboratory facilities to confirm diagnosis; therefore, especially in lower-resource settings, a large number of cases are diagnosed as having "unspecified chromosomal abnormalities" – an ICD code that corresponds to the GBD cause of "other chromosomal abnormalities." Additionally, most congenital birth defects registries have only limited scope as they only track a subset of genetic syndromes.

Total chromosomal anomalies

Crosswalks

The MR-BRT crosswalk results are shown below.

Figure 1: MR-BRT crosswalk of alternate definition (livebirths and stillbirths included) with spline on log-transformed neonatal mortality rate

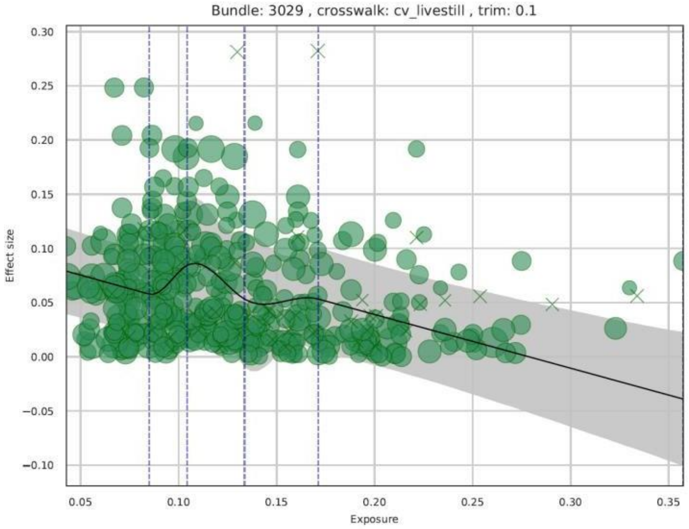

Modelling strategy

To maximise the data basis for estimating chromosomal abnormalities and genetic syndromes, we completed an analysis of all chromosomal abnormalities together, leveraging cause-specific mortality results from the GBD CoD analysis (for Down syndrome plus "other chromosomal abnormalities"), all prevalence data from registries, and clinical administrative data (hospital and claims). This model estimates total chromosomal abnormalities in DisMod-MR 2.1 and served as the basis for scaling the remaining specific causes (Down, Klinefelter, Turner, Edward/Patau) and estimating the remainder.

Table 2. Location-level covariate effects

Covariate name	Measure	Beta value	Exponentiated value
Legality of abortion	Prevalence	–0.0054 (–0.007 to –0.004)	0.99 (0.99–1.00)
Livebirths 35+ (proportion)	Prevalence	0.24 (0.22 to 0.26)	1.27 (1.24–1.30)
Healthcare Access and Quality Index	EMR	–0.02 (–0.022 to –0.018)	0.98 (0.98–0.98)

Down syndrome

Case definition and associated health states

Down syndrome, also known as Trisomy 21, is the presence of a third copy of chromosome 21, typically caused by non-disjunction during the production of gametes. Down syndrome is associated with several specific physical characteristics, including decreased muscle tone, flat facial features, an upward slant to the eyes, abnormally shaped ears, a single deep crease across the centre of the palm, folded skin on the inner corners of the eyes, and ability to extend joints beyond the usual, among others. The GBD case definition of Down syndrome includes ICD-10 codes Q90.0, Q90.1, Q90.2, and Q90.9.

Individuals with Down syndrome may have several combinations of sequelae. Those included in the GBD sequelae list are intellectual disability, congenital heart disease, and dementia. The joint distribution of intellectual disability, congenital heart disease, and dementia associated with cases of Down syndrome was derived from a review of literature on long-term outcomes in cohorts of Down syndrome individuals. To calculate the severity distribution of intellectual disability due to Down syndrome, we used literature values for the IQ distribution of individuals with Down syndrome[1] and calculated the area under the curve. We obtained age-specific proportions of individuals with Down syndrome and dementia, and thus global age patterns were modelled to calculate the proportion of the population with each combination of sequelae for each of the following age ranges: 0–44 years, 45–49 years, 50–54 years, 55–69 years, 70–79 years, and 80+ years.

Crosswalks

The MR-BRT crosswalk results are shown below.

Figure 1: MR-BRT crosswalk of alternate definition (livebirths and stillbirths included) with spline on log-transformed neonatal mortality rate

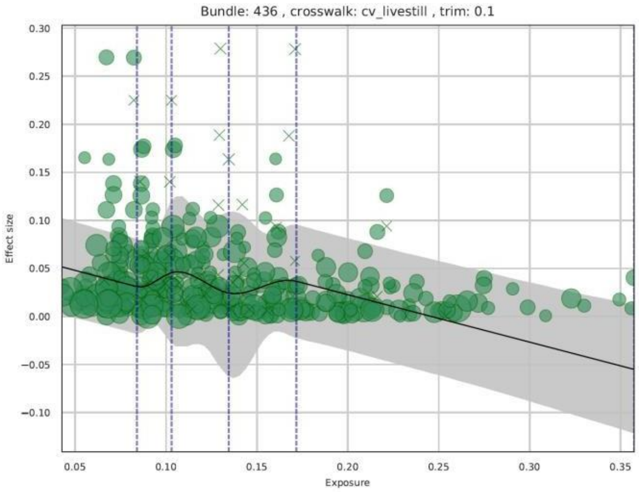

Modelling strategy

The DisMod-MR 2.1 model of Down syndrome excluded all data with a prevalence of zero as outliers, as we expect that these low values are indicative of under-reporting in the data sources. The DisMod-MR

[1] Epstein CJ. Down syndrome (trisomy 21). In: Scriver CR, Beaudet AL, Sly WS, Valle D, eds. *The Metabolic Basis of Inherited Disease*. 7th ed. New York, United States: McGraw Hill Inc., 1995.

2.1 model used CSMR data from the corresponding Down syndrome model in the GBD CoD analysis, and converted these data to EMR estimates where matching prevalence data are available. Random effects EMR were limited to ±0.1, and on prevalence to ±0.2, to limit the geographical variation in birth prevalence allowed in the model. The maximum smoothness on EMR was increased to x=3.0 to fit the observed steep decline in the mortality risk associated with Down syndrome after the neonatal age range.

Of note, the use of cause-specific mortality data in the non-fatal model of Down syndrome is a substantial change in the modelling strategy as compared to the previous iterations of the GBD, and results in much better-informed excess mortality estimates driving the Down syndrome prevalence estimates across the life course.

Table 2. Location-level covariate effects

Covariate name	Measure	Beta value	Exponentiated value
Legality of abortion	Prevalence	–0.0047 (–0.0055 to –0.004)	1.00 (0.99–1.00)
Livebirths 35+ (proportion)	Prevalence	0.0014 (0.000039 to 0.0040)	1.00 (1.00–1.00)
Healthcare Access and Quality Index	EMR	–0.043 (–0.044 to –0.043)	0.96 (0.96–0.96)

Turner syndrome

Case definitions and associated health states

Turner syndrome, also known as 45 XO, is a condition in which a female is partly or completely missing an X chromosome. Turner syndrome can lead to a variety of medical and developmental problems, including short height, failure to commence puberty, infertility, heart defects, learning disabilities, and difficulty with social adjustment. The GBD case definition of Turner syndrome includes ICD-10 codes Q96.0, Q96.3, and Q96.9. The sequelae associated with Turner syndrome are congenital heart disease, infertility, and the combination of both congenital heart disease and infertility; additionally, a subset of individuals with Turner syndrome are asymptomatic. The distribution of these sequelae was determined by a review of existing literature on the long-term health consequences of Turner syndrome.

Crosswalks

The MR-BRT crosswalk results are shown below.

Figure 1: MR-BRT crosswalk of alternate definition (livebirths and stillbirths included) with spline on log-transformed neonatal mortality rate

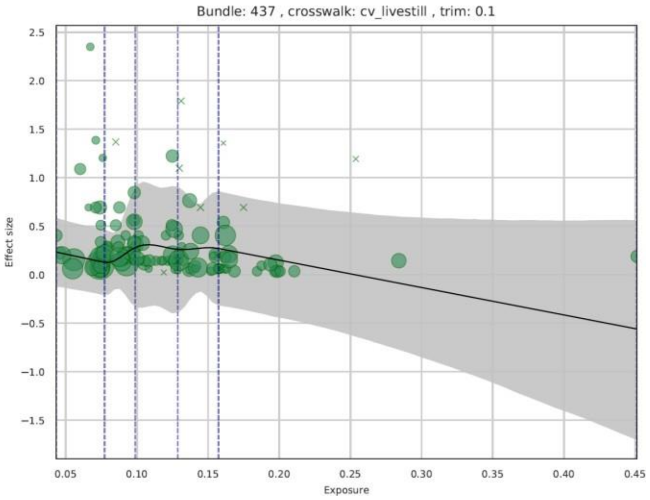

Modelling strategy

One of the known limitations to the use of birth prevalence data on Turner syndrome is that individuals with Turner syndrome are commonly diagnosed later in life rather than prenatally or at birth. Thus, we implemented a correction factor to account for under-diagnosis in all birth registry data sources, using available literature on the trends in age pattern of Turner syndrome diagnosis over time.[1] Although improvements in diagnosis have occurred over time, only between 15% and 30% of all diagnosed Turner syndrome cases are diagnosed before 1 year of age. Additionally, the reported denominators from all birth registries – the number of livebirths in each registry catchment area – were adjusted to include only female births using the GBD fertility estimates of the age, year, and location-specific proportion of total livebirths that are female. Furthermore, all prevalence data with values of zero were excluded as outliers, as these low values indicate severe under-reporting in the input data. These modelling strategy changes address known causes of under-reporting of Turner syndrome in the previous iterations of the GBD and led to higher estimates than reported previously.

The DisMod-MR 2.1 model of Turner syndrome had an EMR capped at 0.1 (slightly higher than the highest available literature estimate of EMR). The model did not have a slope prior set on EMR as the risk of mortality associated with Turner syndrome is not specific to the neonatal ages. This model also allows an increased maximum smoothness on EMR (maximum xi=3.0) and random effects on prevalence limited to ±0.5 to limit random geographical variation in the estimated birth prevalence of Turner syndrome.

Table 2. Location-level covariate effects

Covariate name	Measure	Beta value	Exponentiated value
Livebirths 35+ (proportion)	Prevalence	–0.15 (–0.28 to –0.02)	0.96 (0.76–0.98)
Healthcare Access and Quality Index	EMR	–0.025 (–0.049 to 0)	0.98 (0.95–1.00)

Klinefelter syndrome

Case definitions and associated health states

[1] Massa G, Verlinde F, De Schepper J, Thomas M, Bourguignon JP, Craen M, de Zegher F, Francois I, Du Caju M, Maes M, Heinrichs C, in collaboration with the Belgian Study Group for Paediatric Endocrinology. Trends in age at diagnosis of Turner syndrome. *Arch Dis Child*. 2005; 90(3): 267-8.

Klinefelter syndrome, also known as 47 XXY, is a condition in which a male is born with an extra X chromosome in all or some of his cells. We also include other genotypes with supernumerary X chromosomes, eg, XXXY, XXXXY, etc. The primary feature of Klinefelter syndrome is sterility, but it can cause a variety of other conditions, including weaker muscles, increased height, poor coordination abilities, smaller genitals, breast growth, and reduced sexual drive as a result of lower testosterone levels. The GBD case definition of Klinefelter syndrome includes ICD-10 codes Q98.0, Q98.5, and Q99.8. The sequelae associated with Klinefelter syndrome are borderline intellectual disability, mild intellectual disability, primary infertility, the combination of borderline intellectual disability and infertility, and the combination of mild intellectual disability and infertility. In addition, a subset of individuals with Klinefelter syndrome are asymptomatic. The distribution of these sequelae was determined by a review of existing literature on the long-term health consequences of Turner syndrome.

Crosswalks

The MR-BRT crosswalk results are shown below.

Figure 1: MR-BRT crosswalk of alternate definition (livebirths and stillbirths included) with spline on log-transformed neonatal mortality rate

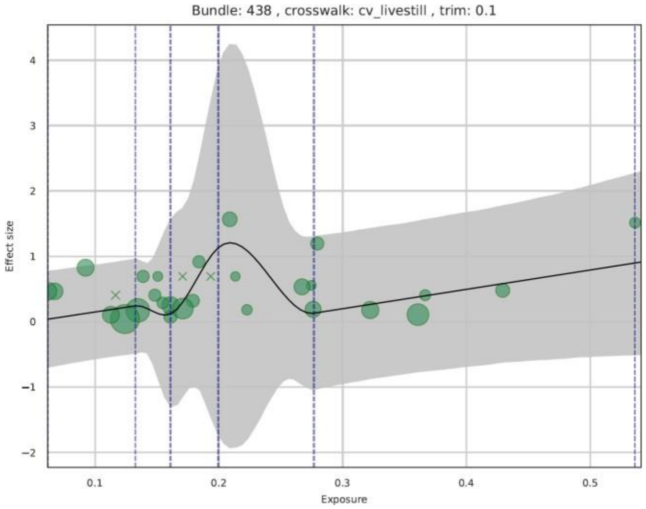

Modelling strategy

As discussed above for Turner syndrome, one limitation to the use of birth registry data for the estimation of Klinefelter syndrome is that many individuals with Klinefelter syndrome are not diagnosed prenatally or at birth. To correct this systematic under-reporting in the birth registry data, we applied a correction factor to all birth registry input data using available literature on the age pattern of Klinefelter syndrome diagnosis.[1] We also adjusted the both-sex livebirth denominators provided in registry data using location-, age-, and year-specific proportions of all livebirths that were male. Furthermore, all prevalence data with values of zero were excluded as outliers, as these low values indicate severe under-reporting in the input data. These modelling strategy changes address known causes of under-reporting in the previous iterations of the GBD and resulted in higher estimates of Klinefelter syndrome than were reported previously.

The DisMod-MR 2.1 model of Klinefelter syndrome had an EMR maximum limit of 0.015, allowing the model to fit estimates of excess mortality up to slightly higher than the highest reported literature values. The model did not have a slope prior set on excess mortality and allowed an increased smoothness on EMR.

Table 2. Location-level covariate effects

Covariate name	Measure	Beta value	Exponentiated value
Legality of abortion	Prevalence	–0.00027 (–0.00092 to –0.000004)	1.00 (1.00–1.00)
Livebirths 35+ (proportion)	Prevalence	0.23 (0.13 to 0.30)	1.26 (1.14–1.35)
Healthcare Access and Quality Index	EMR	–0.025 (–0.049 to –0.0019)	0.98 (0.95–1.00)

Edward and Patau syndromes

Case definitions and associated health states

Edwards syndrome, also known as Trisomy 18, is the condition in which infants are born with a third copy of chromosome 18. Patau syndrome, also known as Trisomy 13, is the condition in which infants

[1] Bojesen A, Juul S, Gravholt CH. Prenatal and Postnatal Prevalence of Klinefelter Syndrome: A National Registry Study. *J Clin Endocrinol Metab*. 2003; 88(2): 622-6.

are born with a third copy of chromosome 13. The GBD estimates the combined prevalence of these two conditions in a single model as they present similarly and are associated with similar rates of excess mortality. Infants with Edwards syndrome typically have low birthweights and a range of associated conditions including a small head and jaw, limb abnormalities, and severe intellectual disability. Infants with Patau syndrome have a range of associated defects including musculoskeletal anomalies, developmental abnormalities of the nervous system such as microcephaly, congenital heart defects, and severe intellectual disability. The ICD-10 code for Edwards syndrome is Q91.3, and the ICD-10 code for Patau syndrome is Q91.7. In GBD 2017, all cases of Edwards and Patau syndrome were assigned the sequela of severe motor and cognitive impairment, and a proportion of these cases are also associated with congenital heart disease. The proportion of cases with associated congenital heart disease was 0.775, derived by pooling estimates from available literature on the health states associated with the two trisomies.[1] [2] This continues to be the case for GBD 2021.

Crosswalks

The MR-BRT crosswalk results are shown below.

Figure 1: MR-BRT crosswalk of alternate definition (livebirths and stillbirths included) with spline on log-transformed neonatal mortality rate

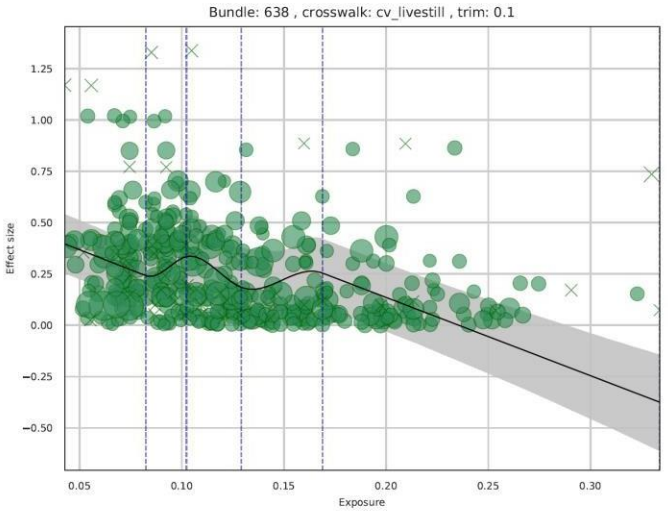

Modelling strategy

In the DisMod-MR 2.1 model of Edwards Syndrome and Patau Syndrome, random effects on prevalence were limited to ±0.5, reflecting the expectation of limited geographic variation in the birth prevalence of Edwards Syndrome and Patau Syndrome. A decreasing slope prior was set on EMR for ages 0–1, and an increasing slope prior was set on EMR for all ages 1+, as individuals with these trisomies generally die within the first few years of life. The model allowed a maximum smoothness of xi=3.0 in order to fit high excess mortality in the early age groups.

[1] Petry P, Polli JB, Mattos VF, Rosa RCM, Zen PRG, Graziadio C, Paskulin GA, Rosa RFM. Clinical features and prognosis of a sample of patients with trisomy 13 (Patau syndrome) from Brazil. *Am J Med Genet A*. 2013; 161A(6): 1278–83.

[2] Polli JB, Groff D de P, Petry P, Mattos VF, Rosa RCM, Zen PRG, Graziadio C, Paskulin GA, Rosa RFM. Trisomy 13 (Patau syndrome) and congenital heart defects. *Am J Med Genet A*. 2014; 164A(1): 272–5.

Table 2. Location-level covariate effects

Covariate name	Measure	Beta value	Exponentiated value
Legality of abortion	Prevalence	–0.004 (–0.0058 to –0.0022)	1.00 (0.99–1.00)
Livebirths 35+ (proportion)	Prevalence	0.034 (0.0013 to 0.091)	1.03 (1.00–1.10)
Healthcare Access and Quality Index	EMR	–0.025 (–0.049 to –0.0023)	0.98 (0.95–1.00)

All input data with birth prevalence values of zero were excluded as outliers, as these values represent under-reporting and low case ascertainment in the input data rather than a true lack of these chromosomal conditions in the corresponding locations.

Other chromosomal abnormalities, genetic syndromes, and micro-deletions

Case definitions and associated health states

Unbalanced chromosomal rearrangements are genetic anomalies that typically occur due to meiotic non-disjunction, when homologous chromosomes do not separate normally in nuclear division during gamete formation. The GBD case definition of other chromosomal rearrangements includes 47 XXX (Triple X syndrome), other meiotic nondisjunction events, other female sex chromosome abnormalities, and other unspecified chromosomal abnormalities. The GBD case definition corresponds to the ICD-10 codes Q92.0, Q97.0, Q97.8, and Q99.9. Excluded from this definition are the chromosomal abnormalities of Down syndrome, Turner syndrome, Klinefelter syndrome, Edward syndrome, and Patau syndrome, which are each modelled separately. The sequelae associated with other chromosomal rearrangements include intellectual disability, intellectual disability with dementia, intellectual disability with congenital heart disease and dementia, and intellectual disability with congenital heart disease. Additionally, a proportion of the individuals with unbalanced chromosomal rearrangements are asymptomatic. In the absence of available literature on the long-term health outcomes among individuals with other chromosomal conditions, the severity distributions associated with Down syndrome were used for the sequelae associated with other chromosomal anomalies.

Post-model processing

Other chromosomal anomalies were calculated based on reducing the model of total chromosomal anomalies by each of the chromosomal sub-causes, and the remaining prevalence was attributed to other chromosomal anomalies. Specifically, we use claims data to calculate the proportion of cases that are due to the other causes. To do that, we sum the cases for the specific sub-causes and the other sub-causes cases. We divide the number of other sub-cause cases by the total number of cases to obtain the proportion. In order to have a valid proportion, we only use datapoints for which we have the combination of age, sex, location, and year for all sub-causes. We then calculate the prevalence of other: p_other = (p_sum_subcauses / 1-prop_other) - p_sub_subcauses.

Musculoskeletal congenital anomalies

The GBD definition of musculoskeletal congenital anomalies includes any anomalies of the muscles or skeletal system present at birth that are not caused by a defined chromosomal syndrome. Within the range of congenital musculoskeletal anomalies, we explicitly model three sub-categories: polydactyly and syndactyly, limb reduction defects, and all other congenital musculoskeletal anomalies.

Total musculoskeletal birth defects

Crosswalks

The MR-BRT crosswalk results are shown below.

Table 1: MR-BRT crosswalk betas for alternate definitions (reference = livebirths including those with chromosomal anomalies)

Crosswalk	Beta	Standard error
Excluding chromosomal diagnoses adjustment	–0.053	0.007

Figure 1: MR-BRT crosswalk of alternate definition (livebirths and stillbirths included) with spline on log-transformed neonatal mortality rate

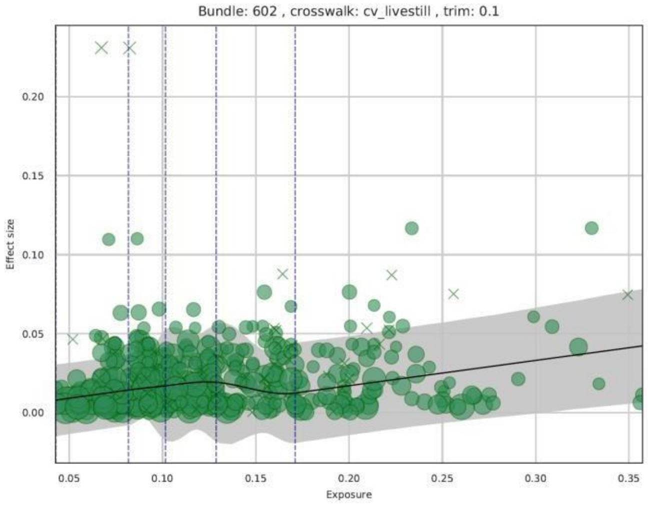

Modelling strategy

The DisMod-MR 2.1 model of total musculoskeletal anomalies used cause-specific mortality estimates from the corresponding model in the GBD CoD analysis, and converted these data to excess mortality estimates where corresponding prevalence data were available. Random effects on prevalence were limited to ±1.0 to limit geographical variation in the birth prevalence of congenital musculoskeletal anomalies. Smoothness on EMR was increased to xi=3.0 to allow the model to fit a steep decrease in EMR after the earliest age groups.

Table 2. Location-level covariate effects

Covariate name	Measure	Beta value	Exponentiated value
Legality of abortion	Prevalence	–0.00012 (–0.00032 to –0.0000069)	1.00 (1.00–1.00)
Healthcare Access and Quality Index	EMR	–0.074 (–0.15 to –0.0042)	0.93 (0.86–1.00)
Age-standardised summary exposure value (SEV) for household air pollution	Prevalence	0.0063 (0.00033 to 0.016)	1.01 (1.00–1.02)
Age-standardised SEV for smoking	Prevalence	0.024 (0.0013 to 0.063)	1.02 (1.00–1.07)

Limb reduction defects

Case definitions and associated health states

Limb reduction defects are the condition where a part or all of the arm or limb of a fetus fails to form during development, so that the limb is either reduced from its normal size or missing entirely. The GBD case definition of limb reduction defects corresponds with ICD-10 codes Q71 (all three-digit codes under Q71), Q72 (all three-digit codes), Q73.0, Q73.1, and Q73.8. Of note, club foot and hip dysplasia are no longer included in this category as of GBD 2019.

All cases of limb reduction defects are associated with level 2 disfigurement. A proportion of limb reduction defect cases are associated with no motor impairment, mild motor impairment with and without pain, and moderate motor impairment with and without pain. The distribution of health states

associated with congenital limb reduction was derived from an analysis of available literature on the long-term outcomes among individuals with congenital limb reductions.[1] [2]

Crosswalks

The MR-BRT crosswalk results are shown below.

Table 1: MR-BRT crosswalk betas for alternate definitions (reference = livebirths including those with chromosomal anomalies)

Crosswalk	Beta	Standard error
Excluding chromosomal diagnoses adjustment	–0.042	0.034

Figure 1: MR-BRT crosswalk of alternate definition (livebirths and stillbirths included) with spline on log-transformed neonatal mortality rate

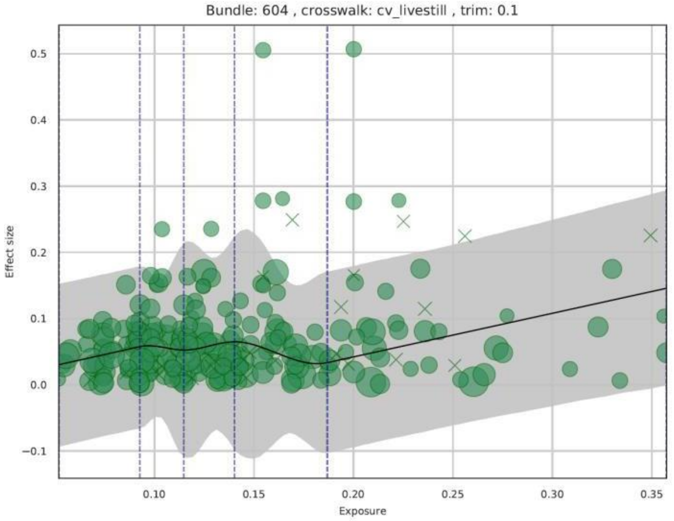

Modelling strategy

In the DisMod-MR 2.1 model of limb reduction defects, random effects on prevalence were limited to ±0.75 to limit geographical variation in the estimated birth prevalence. The EMR was set to a maximum of 0.02 for all ages to reflect the relatively low mortality risk of congenital limb anomalies. Remission for the first 3 months of life was restricted to 0–0.02, while for 3 months to 2 years it was allowed to go up to a maximum of 1. From ages 2–5 years, remission was restricted to 0–0.1, and for all ages after 5 years old remission was bound between 0–0.004.

Table 2. Location-level covariate effects

Covariate name	Measure	Beta value	Exponentiated value

---

[1] Johansen H, Østlie K, Andersen LØ, Rand-Hendriksen S. Adults with congenital limb deficiency in Norway: demographic and clinical features, pain and the use of health care and welfare services. A cross-sectional study. *Disabil Rehabil*. 2015; 37(22): 2076–82.

[2] Johansen H, Dammann B, Oinæs Andersen L, Andresen I-L. Children with congenital limb deficiency in Norway: issues related to school life and health-related quality of life. A cross-sectional study. *Disabil Rehabil*. 2016; 38(18): 1803–10.

Legality of abortion	Prevalence	–0.00039 (–0.00094 to –0.000029)	1.00 (1.00–1.00)
Healthcare Access and Quality Index	EMR	–0.025 (–0.049 to –0.00051)	0.98 (0.95–1.00)
Age-standardised SEV for household air pollution	Prevalence	1.39 (1.25 to 1.52)	4.00 (3.50–4.57)

Polydactyly and syndactyly

Case definitions and associated health states

Polydactyly is the condition of being born with at least one extra digit on either the hand or the foot, while syndactyly is absence of at least one digit. Our case definition of polydactyly corresponds to ICD-10 code Q69, and syndactyly corresponds to Q70. The sequela associated with all cases of polydactyly and syndactyly is level 1 disfigurement.

All cases of polydactyly and syndactyly are assigned the health state of level 1 disfigurement. Remission is allowed in the model of polydactyly and syndactyly, as individuals born with these conditions may have them surgically corrected and are then no longer considered within our case definition.

Crosswalks

The MR-BRT crosswalk results are shown below.

Table 1: MR-BRT crosswalk betas for alternate definitions (reference = livebirths including those with chromosomal anomalies)

Crosswalk	Beta	Standard error
Excluding chromosomal diagnoses adjustment	–0.05	0.011

Figure 1: MR-BRT crosswalk of alternate definition (livebirths and stillbirths included) with spline on log-transformed neonatal mortality rate

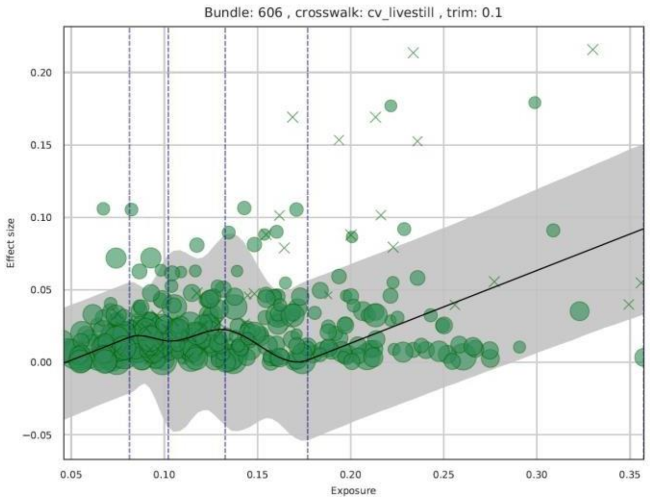

Modelling strategy

The DisMod-MR 2.1 model of polydactyly and syndactyly limited random effects on prevalence to ±0.75, as we expected limited geographical variation in the birth prevalence estimates. Excess mortality priors were set to 0 for ages 0–54 and had a max of 0.1 for ages 55+, as it is not expected that someone would die of these conditions at an early age. The remission rate was bounded from 0 to 0.02 for the first 3 months of life, as surgical correction of polydactyly or syndactyly rarely occurs in the first few months of

life. Remission was bounded between 0 and 5 for ages 3 months to 2 years, and between 0 and 0.5 for

ages 2–5 years, the ages during which surgical correction is most likely to occur, then set to a maximum of 0.02 after 5 years of age. The smoothness on remission was set to xi=1.5 in order to facilitate steep changes in remission rates during the first few years of life.

Table 2. Location-level covariate effects

Covariate name	Measure	Beta value	Exponentiated value
LDI (I$ per capita)	Remission	1.01 (0.54–1.47)	2.74 (1.72–4.36)

Other congenital musculoskeletal defects

Case definitions and associated health states

The other congenital musculoskeletal anomalies included within the total estimate of congenital musculoskeletal anomalies includes clubfoot, skeletal dysplasias, congenital deformities of the spine, congenital dysplasia of the hip, and other congenital musculoskeletal anomalies. This “other” category corresponds to ICD-10 codes Q65, Q65.0, Q65.00, Q65.01, Q65.02, Q65.1, Q65.2, Q65.8, Q65.81, Q65.82, Q65.89, Q65.9, Q66, Q66.0, Q66.1, Q68, Q68.1, Q68.2, Q68.6, Q68.8, Q74, Q74.1, Q74.2, Q74.3, Q74.9, Q75, Q75.0, Q75.5, Q75.9, Q79.8, Q79.9, Q76, Q76.1, Q76.2, Q76.3, Q76.4, Q76.41, Q76.411, Q76.412, Q76.413, Q76.414, Q76.415, Q76.419, Q76.42, Q76.425, Q76.426, Q76.427, Q76.428, Q76.429, Q76.49, Q76.8, Q76.9, Q77, Q77.0, Q77.1, Q77.2, Q77.3, Q77.4, Q77.5, Q77.6, Q77.7, Q77.8, Q77.9, Q78, Q78.0, Q78.1, Q78.2, Q78.3, Q78.4, Q78.5, Q78.6, Q78.8, and Q78.9.

In the absence of comprehensive literature on the long-term outcomes associated with the category of other congenital musculoskeletal anomalies, prevalence estimates of other congenital musculoskeletal anomalies were assigned health states using the proportions derived for limb reduction defects.

Post-model processing

Other congenital musculoskeletal anomalies are modelled by applying the ratio of other congenital digestive anomalies to total congenital digestive anomalies as it is reflected in MarketScan data, to the sum of the sub-causes of congenital musculoskeletal anomalies. The result is prevalence of other congenital musculoskeletal anomalies by age/year/sex/location. Specifically, we use claims data to calculate the proportion of cases that are due to the other causes. To do that, we sum the cases for the specific sub-causes and the other sub-cause cases. We divide the number of other sub-cause cases by total number of cases to obtain the proportion. In order to have a valid proportion, we only use datapoints for which we have the combination of age, sex, location, and year for all sub-causes. We then calculate the prevalence of other: p_other = (p_sum_subcauses / 1-prop_other) - p_sub_subcauses.

Urogenital congenital anomalies

The GBD case definition of urogenital congenital anomalies includes anomalies of the genitals and the urinary system that are present at birth. While some types of urogenital congenital anomalies encompass both the urinary and genital systems, we have assigned each congenital condition as a malformation of either the urinary or the genital system in a mutually exclusive fashion and model anomalies of the urinary and genital systems separately.

Congenital urogenital anomalies were modelled as two distinct categories, with distinct model specifications: urinary congenital anomalies and genital congenital anomalies.

Congenital urinary anomalies

Case definitions and associated health states

Urinary anomalies include congenital malformation of the collecting system, ureter, bladder, and kidney, as well as bladder exstrophy and epispadias. The ICD-10 codes included in the category of urinary anomalies are Q64.0, Q64.1, Q60-Q61, and Q62-Q63.

The total prevalence of congenital urinary anomalies was split into proportions with and without each of the following health states: urinary incontinence, impotence, recurrent urinary tract infections and other recurring abdominal issues, and atypical genitalia (corresponding to disfigurement, level 1 in the GBD Disability Weights Study). The distribution of these long-term outcomes was derived from a review of available literature on the long-term outcomes experienced cohorts of individuals born with a range of congenital urogenital anomalies.[1,2,3,4,5,6]

Crosswalks

The MR-BRT crosswalk results are shown below.

Table 1: MR-BRT crosswalk betas for alternate definitions (reference = livebirths including those with chromosomal anomalies)

Crosswalk	Beta	Standard error
Excluding chromosomal diagnoses adjustment	–0.032	0.008

Figure 1: MR-BRT crosswalk of alternate definition (livebirths and stillbirths included) with spline on log-transformed neonatal mortality rate

[1] Ching CB, Wood HM, Ross JH, Gao T, Angermeier KW. The Cleveland Clinic experience with adult hypospadias patients undergoing repair: their presentation and a new classification system. *BJU Int*. 2011; 107(7): 1142–6.

[2] Davies MC, Liao L-M, Wilcox DT, Woodhouse CRJ, Creighton SM. Anorectal malformations: what happens in adulthood?. *BJU Int*. 2010; 106(3): 398–404.

[3] Rintala RJ. Congenital cloaca: Long-term follow-up results with emphasis on outcomes beyond childhood. *Semin Pediatr Surg*. 2016; 25(2): 112–6.

[4] Sircili MHP, e Silva FA de Q, Costa EMF, Brito VN, Arnhold IJP, Dénes FT, Inacio M, de Mendonca BB. Long-term surgical outcome of masculinizing genitoplasty in large cohort of patients with disorders of sex development. *J Urol*. 2010; 184(3): 1122–7.

[5] van der Zwan YG, Callens N, van Kuppenveld J, Kwak K, Drop SLS, Kortmann B, Dessens AB, Wolffenbuttel KP, Dutch Study Group on DSD. Long-term outcomes in males with disorders of sex development. *J Urol*. 2013; 190(3): 1038–42.

[6] Warne SA, Wilcox DT, Creighton S, Ransley PG. Long-term gynecological outcome of patients with persistent cloaca. *J Urol*. 2003; 170(4 Pt 2): 1493–6.

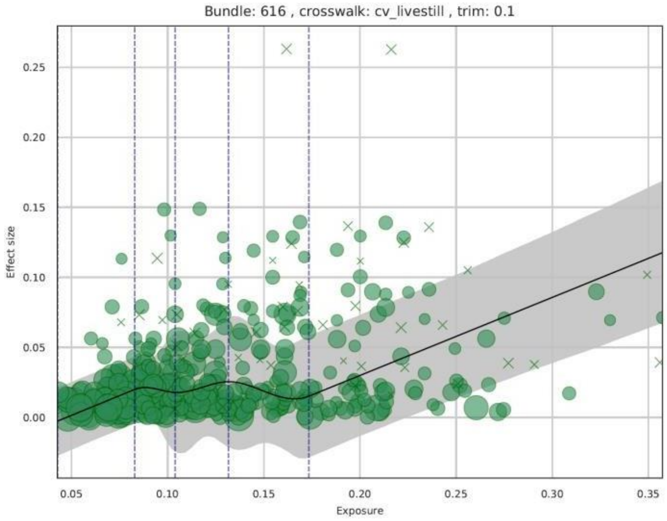

Modelling strategy

In the DisMod-MR 2.1 model of congenital urinary anomalies, random effects on prevalence were limited to ±0.5, and random effects on with-condition mortality were limited to ±1.0. The maximum EMR was set to 0.1 for all ages. The smoothness on EMR was set to xi=3 to fit changes in the EMR during the neonatal period. CSMR was also pulled in from our CoD model of congenital urogenital anomalies. As we assume no death due to congenital genital anomalies, this model represents deaths associated with exclusively congenital urinary anomalies.

Table 2. Location-level covariate effects

Covariate name	Measure	Beta value	Exponentiated value
Age-standardised SEV for ambient particulate matter	Prevalence	0.013 (0.00036 to 0.046)	1.01 (1.00–1.05)
Age-standardised SEV for high fasting plasma glucose	Prevalence	2.80 (2.61 to 2.97)	16.39 (13.61–19.57)
Healthcare Access and Quality Index	EMR	–0.029 (–0.03 to –0.027)	0.97 (0.97–0.97)

Congenital genital anomalies

Case definitions and associated health states

Genital anomalies include hypospadias, ambiguous or indeterminate sex, other congenital abnormalities of the male genitalia, and a variety of female genital malformations. ICD-10 codes Q50–Q52, Q54, Q56, and Q55 (excluding Q55.20–Q55.21) are included in the case definition of congenital genital anomalies. Undescended testicles are excluded from the case definition of genital anomalies, as this is not considered a severe condition.

Cases of congenital genital anomalies was split into proportions with and without primary infertility, impotence, recurrent urinary tract infections and other recurring abdominal issues, and atypical genitalia. Estimates were produced for the prevalence of every possible combination of those long-term sequelae, assuming independence between the outcomes. The distribution of these long-term

outcomes was derived from a review of available literature on the long-term outcomes experienced cohorts of individuals born with a range of congenital urogenital anomalies.[1] [2] [3] [4] [5] [6] [7] [8] [9] [10]

---

[1] Ching CB, Wood HM, Ross JH, Gao T, Angermeier KW. The Cleveland Clinic experience with adult hypospadias patients undergoing repair: their presentation and a new classification system. *BJU Int*. 2011; 107(7): 1142–6.

[2] Davies MC, Liao L-M, Wilcox DT, Woodhouse CRJ, Creighton SM. Anorectal malformations: what happens in adulthood?. *BJU Int*. 2010; 106(3): 398–404.

[3] Rintala RJ. Congenital cloaca: Long-term follow-up results with emphasis on outcomes beyond childhood. *Semin Pediatr Surg*. 2016; 25(2): 112–6.

[4] Sircili MHP, e Silva FA de Q, Costa EMF, Brito VN, Arnhold IJP, Dénes FT, Inacio M, de Mendonca BB. Long-term surgical outcome of masculinizing genitoplasty in large cohort of patients with disorders of sex development. *J Urol*. 2010; 184(3): 1122–7.

[5] van der Zwan YG, Callens N, van Kuppenveld J, Kwak K, Drop SLS, Kortmann B, Dessens AB, Wolffenbuttel KP, Dutch Study Group on DSD. Long-term outcomes in males with disorders of sex development. *J Urol*. 2013; 190(3): 1038–42.

[6] Warne SA, Wilcox DT, Creighton S, Ransley PG. Long-term gynecological outcome of patients with persistent cloaca. *J Urol*. 2003; 170(4 Pt 2): 1493–6.

[7] Rintala RJ. Congenital cloaca: Long-term follow-up results with emphasis on outcomes beyond childhood. *Semin Pediatr Surg*. 2016; 25(2): 112–6.

[8] Sircili MHP, e Silva FA de Q, Costa EMF, Brito VN, Arnhold IJP, Dénes FT, Inacio M, de Mendonca BB. Long-term surgical outcome of masculinizing genitoplasty in large cohort of patients with disorders of sex development. *J Urol*. 2010; 184(3): 1122–7.

[9] van der Zwan YG, Callens N, van Kuppenveld J, Kwak K, Drop SLS, Kortmann B, Dessens AB, Wolffenbuttel KP, Dutch Study Group on DSD. Long-term outcomes in males with disorders of sex development. *J Urol*. 2013; 190(3): 1038–42.

[10] Warne SA, Wilcox DT, Creighton S, Ransley PG. Long-term gynecological outcome of patients with persistent cloaca. *J Urol*. 2003; 170(4 Pt 2): 1493–6.

Crosswalks

The MR-BRT crosswalk results are shown below.

Table 1: MR-BRT crosswalk betas for alternate definitions (reference = livebirths including those with chromosomal anomalies)

Crosswalk	Beta	Standard error
Excluding chromosomal diagnoses adjustment	–0.019	0.011

Figure 1: MR-BRT crosswalk of alternate definition (livebirths and stillbirths included) with spline on log-transformed neonatal mortality rate

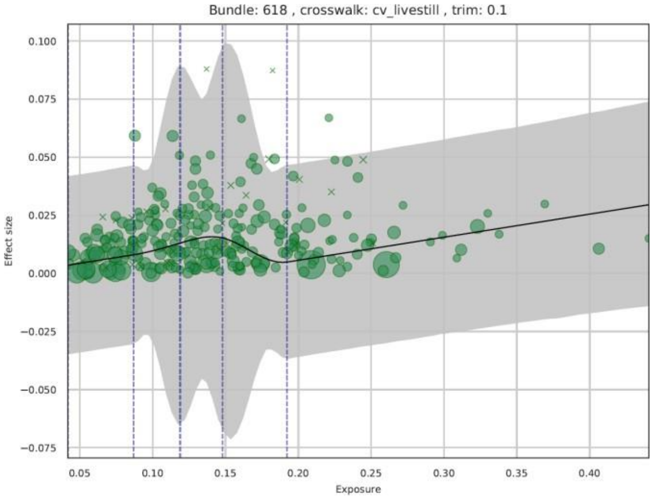

Modelling strategy

In the DisMod-MR 2.1 model of congenital genital anomalies, random effects on prevalence were limited to ±0.75 to limit random geographical variation in the estimates of birth prevalence. Excess mortality was set to 0 for all ages, as we do not believe that individuals are dying due to genital anomalies. This is consistent with our CoD analysis, in which the only causes reflected in our urogenital mortality estimates are congenital urinary conditions.

Table 2. Location-level covariate effects

Covariate name	Measure	Beta value	Exponentiated value
Age-standardised SEV for ambient particulates	Prevalence	0.050 (0.0018–0.14)	1.05 (1.00–1.15)
Age-standardised SEV for high fasting plasma glucose	Prevalence	0.25 (0.042–0.48)	1.29 (1.04–1.62)

Congenital anomalies of the digestive system

Case definitions

Congenital anomalies of the digestive system include any anomalies of the gastrointestinal tract present at birth as the result of abnormal embryonic development. As with the other congenital causes, this variety of digestive system abnormalities is split into four sub-cause categories.

Total digestive congenital anomalies

To ensure internal consistency in the estimates of each sub-type of congenital digestive anomalies, we generated a model to estimate the total prevalence and associated mortality due to all congenital digestive anomalies, then fit the estimates of each sub-type of congenital digestive anomalies proportionally to the envelope of this total model. The prevalence estimates of other congenital digestive anomalies were derived by reducing the total envelope model for each cause by its sub-causes to derive the difference that was attributable to other anomalies in that category. This modelling strategy allowed us to utilise the GBD CoD estimates as input to the total congenital digestive anomalies estimates and allowed us to incorporate literature data that reported only the total prevalence of all digestive anomalies.

Crosswalks

The MR-BRT crosswalk results are shown below.

Table 1: MR-BRT crosswalk betas for alternate definitions (reference = livebirths including those with chromosomal anomalies)

Crosswalk	Beta	Standard error
Excluding chromosomal diagnoses adjustment	–0.078	0.011

Figure 1: MR-BRT crosswalk of alternate definition (livebirths and stillbirths included) with spline on log-transformed neonatal mortality rate

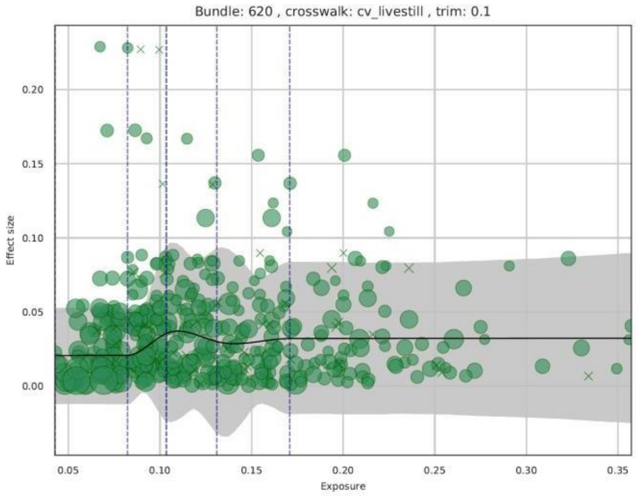

Modelling strategy

The DisMod-MR 2.1 model of total congenital digestive anomalies used cause-specific mortality estimates from the corresponding GBD CoD model of congenital digestive anomalies, and these data were converted to excess mortality estimates where corresponding cause-specific mortality estimates were available. The model had random effects on prevalence limited to ±0.5 and random effects on excess mortality limited to ±0.1. The model also had a slope prior on remission to decrease with age and have an overall all-ages maximum of 1.0. The smoothness on EMR was increased to xi=3.0 in order to fit steep changes in EMR during the neonatal age period.

Table 2. Location-level covariate effects

Covariate name	Measure	Beta value	Exponentiated value
Age-standardised SEV for smoking	Prevalence	0.052 (0.0021 to 0.20)	1.05 (1.00–1.22)
Age-standardised SEV for high BMI	Prevalence	0.99 (0.96 to 1.00)	2.69 (2.62–2.72)
Litres of alcohol consumed per capita	Prevalence	0.0031 (0.00013 to 0.0080)	1.00 (1.00–1.01)

Healthcare Access and Quality Index	EMR	–0.027 (–0.028 to –0.026)	0.97 (0.97–0.97)

Congenital diaphragmatic hernia

Case definitions and associated health states

Congenital diaphragmatic hernia, a life-threatening malformation of the diaphragm that allows the abdominal organs to push into the chest cavity and obstructs proper formation of the lungs, is modelled separately from all other congenital malformations of the digestive system. Congenital diaphragmatic hernia corresponds to ICD-10 code Q79.0.

The health outcomes associated with congenital diaphragmatic hernia include every combination of disfigurement, chronic abdominal pain, mild chronic respiratory problems, breathlessness, mild intellectual disability, and a proportion of patients who are asymptomatic. The distribution of these long-term health outcomes was derived from a pooled analysis of available literature on the long-term outcomes in surviving patients born with congenital diaphragmatic hernias.[1,2,3]

Crosswalks

The MR-BRT crosswalk results are shown below.

Table 1: MR-BRT crosswalk betas for alternate definitions (reference = livebirths including those with chromosomal anomalies)

Crosswalk	Beta	Standard error
Excluding chromosomal diagnoses adjustment	–0.063	0.035

Figure 1: MR-BRT crosswalk of alternate definition (livebirths and stillbirths included) with spline on log-transformed neonatal mortality rate

[1] Crankson SJ, Al Jadaan SA, Namshan MA, Al-Rabeeah AA, Oda O. The immediate and long-term outcomes of newborns with congenital diaphragmatic hernia. *Pediatr Surg Int*. 2006; 22(4): 335-40.

[2] Öst E, Joelsson MÖ, Burgos CM, Frenckner B. Self-assessed physical health among children with congenital diaphragmatic hernia. *Pediatr Surg Int*. 2016; 32(5): 493–503.

[3] Rocha GM, Bianchi RF, Severo M, Rodrigues MM, Baptista MJ, Correia-Pinto J, Guimarães HA. Congenital Diaphragmatic Hernia. The Post-neonatal period. Part II. *Eur J Pediatr Surg*. 2008; 18(5): 307–12.

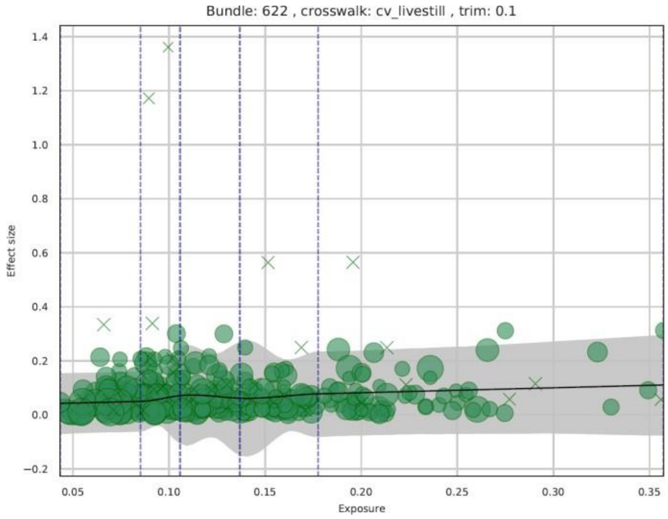

Modelling strategy

In the DisMod-MR 2.1 model of congenital diaphragmatic hernia, random effects on prevalence were set to ±0.5. The maximum excess mortality for the early neonatal age period was set to 10.0, and to 0.05 for all subsequent ages. A decreasing slope prior on remission rate was set for all ages and smoothness on EMR was increased to xi=3.0 in order to fit steep changes in EMR during the first weeks of life.

Table 2. Location-level covariate effects

Covariate name	Measure	Beta value	Exponentiated value
Age-standardised SEV for smoking	Prevalence	0.48 (0.17 to 0.79)	1.62 (1.18–2.21)
Age-standardised SEV for high BMI	Prevalence	0.065 (0.0047 to 0.15)	1.07 (1.00–1.16)
Litres of alcohol consumed per capita	Prevalence	0.00057 (0.000023 to 0.0016)	1.00 (1.00–1.00)
Healthcare Access and Quality Index	EMR	–0.025 (–0.049 to –0.0012)	0.98 (0.95–1.00)

Congenital malformations of the abdominal wall

Case definitions and associated health states

All congenital malformations of the abdominal wall are modelled together as a distinct sub-category. The primary diagnoses in this category are gastroschisis, omphalocele, and prune belly syndrome, corresponding to ICD-10 codes Q79.3, Q79.2, and Q79.4, respectively.

The health outcomes associated with congenital malformations of the abdominal wall include every combination of constipation, chronic abdominal pain, and disfigurement and concern about scars. The distribution of these outcomes was calculated from a pooled analysis of literature sources on the long-term outcomes among surviving individuals born with congenital malformations of the abdominal wall.[1,2]

[1] van Eijck FC, Wijnen RMH, van Goor H. The incidence and morbidity of adhesions after treatment of neonates with gastroschisis and omphalocele: a 30-year review. *J Pediatr Surg*. 2008; 43(3): 479–83.

[2] Harris EL, Minutillo C, Hart S, Warner TM, Ravikumara M, Nathan EA, Dickinson JE. The long term physical consequences of gastroschisis. *J Pediatr Surg*. 2014; 49(10): 1466–70.

Crosswalks

The MR-BRT crosswalk results are shown below.

Table 1: MR-BRT crosswalk betas for alternate definitions (reference = livebirths including those with chromosomal anomalies)

Crosswalk	Beta	Standard error
Excluding chromosomal diagnoses adjustment	–0.069	0.025

Figure 1: MR-BRT crosswalk of alternate definition (livebirths and stillbirths included) with spline on log-transformed neonatal mortality rate

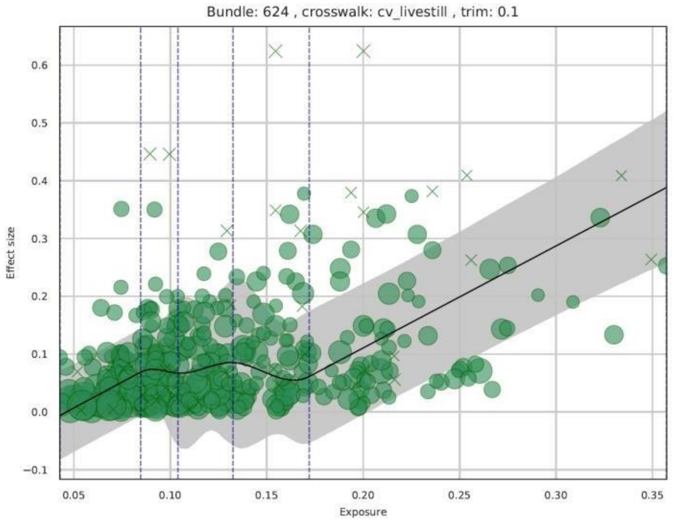

Modelling strategy

The DisMod-MR 2.1 model of congenital malformations of the abdominal wall had random effects on prevalence limited to ±0.5. The minimum EMR was set to 0.5 with a maximum EMR of 10.0, for the early neonatal period. For ages 0.5–100 years, excess mortality max was set to 0.05. A decreasing slope prior on remission was set for all ages, and the smoothness on EMR was set to xi=3.0, allowing the model to fit a steep decrease in the EMR after the neonatal age period.

Table 2. Location-level covariate effects

Covariate name	Measure	Beta value	Exponentiated value
Age-standardised SEV for smoking	Prevalence	0.047 (0.0023 to 0.13)	1.05 (1.00–1.13)
Age-standardised SEV for high BMI	Prevalence	0.038 (0.0018 to 0.10)	1.04 (1.00–1.11)
Litres of alcohol consumed per capita	Prevalence	0.00064 (0.000018 to 0.0017)	1.00 (1.00–1.00)
Healthcare Access and Quality Index	EMR	–0.025 (–0.049 to 0)	0.98 (0.95–1.00)

Congenital atresia and/or stenosis of the digestive tract

Case definitions and associated health states

All variations of atresia and/or stenosis of the digestive tract are modelled together as the third distinct sub-category of digestive congenital anomalies. This includes biliary atresia, oesophageal atresia and/or stenosis with and without trachea-oesophageal fistula, and atresia and stenosis of the small intestine,

large intestine, rectum, and anus. The ICD-10 codes included in the atresia and stenosis sub-cause category are Q42.0, Q42.1, Q42.2, Q42.3, Q42.4, Q42.8, Q42.9, Q42.8, Q42.9, Q42.0, Q42.1, Q42.2, Q42.3, Q42.4, Q41 (Q41.0, Q41.1, Q41.2, Q41.8, Q41.9), Q44.2, Q39.0, Q39.1, and Q39.2.

The outcomes associated with congenital atresia and/or stenosis of the abdominal tract include every combination of dysphagia, acid reflux, chronic abdominal pain and/or nausea, and chronic respiratory problems; the distribution of these long-term outcomes was also derived from available long-term follow-up studies.[1 2]

Crosswalks

The MR-BRT crosswalk results are shown below.

Table 1: MR-BRT crosswalk betas for alternate definitions (reference = livebirths including those with chromosomal anomalies)

Crosswalk	Beta	Standard error
Excluding chromosomal diagnoses adjustment	–0.093	0.016

Figure 1: MR-BRT crosswalk of alternate definition (livebirths and stillbirths included) with spline on log-transformed neonatal mortality rate

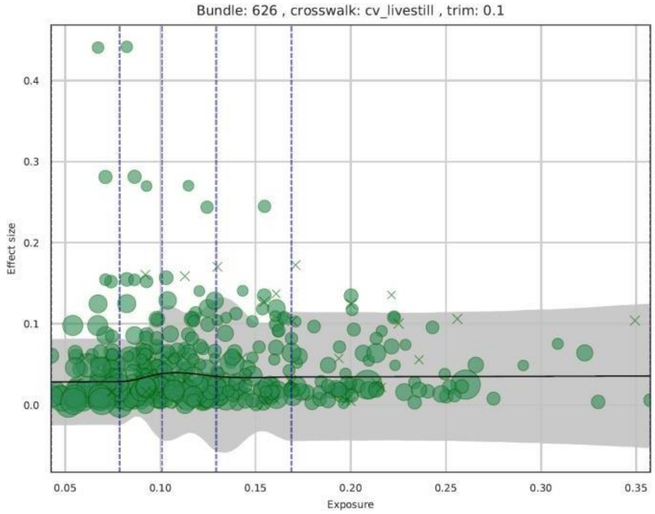

Modelling strategy

In the DisMod-MR 2.1 model of congenital atresia and/or stenosis of the digestive tract, random effects on prevalence were set to ±0.5, and random effects on with-condition mortality were set to ±1.0. A decreasing slope prior on remission was set for all ages, as remission is most likely just after birth. The

---

[1] Dingemann C, Meyer A, Kircher G, Boemers TM, Vaske B, Till H, Ure BM. Long-term health-related quality of life after complex and/or complicated esophageal atresia in adults and children registered in a German patient support group. *J Pediatr Surg*. 2014; 49(4): 631–8.

[2] Lilja HE, Wester T. Outcome in neonates with esophageal atresia treated over the last 20 years. *Pediatr Surg Int*. 2008; 24(5): 531–6.

smoothness on EMR was increased to xi=3.0 in order to fit steep changes in EMR during the first weeks of life, with value priors set to 2–15 for the early neonatal period and 0 for ages 70–100.

Table 2. Location-level covariate effects

Covariate name	Measure	Beta value	Exponentiated value
Age-standardised SEV for smoking	Prevalence	0.076 (0.0039 to 0.19)	1.08 (1.00–1.21)
Age-standardised SEV for high BMI	Prevalence	0.99 (0.96 to 1.00)	2.68 (2.62–2.72)
Litres of alcohol consumed per capita	Prevalence	0.0028 (0.00029 to 0.0059)	1.00 (1.00–1.01)
Healthcare Access and Quality Index	EMR	–0.025 (–0.049 to –0.00079)	0.98 (0.95–1.00)

Other congenital digestive anomalies

Case definitions and associated health states

Other congenital malformations and diseases of the digestive system includes ICD-10 codes Q38 (Q38.0, Q38.3, Q38.4, Q38.6, Q38.7, Q38.8), Q39 (Q39.3, Q39.4, Q39.5, Q39.6, Q39.8, Q39.9), Q40(Q40.0, Q40.1, Q40.2, Q40.3, Q40.8, Q40.9), Q43 (Q43.1, Q43.2, Q43.3, Q43.4, Q43.5, Q43.6, Q43.7, Q43.8, Q43.9), Q44 (Q44.0, Q44.1, Q44.3, Q44.4, Q44.5, Q44.6, Q44.7), Q45 (Q45.0, Q45.1, Q45.2, Q45.3, Q45.8, Q45.9), Q79.1, and Q79.5 (Q79.51, Q79.59). Inguinal hernias present at birth are excluded from the case definition of gastrointestinal congenital anomalies and are modelled separately as part of the estimation of inguinal hernias.

The distribution of health outcomes associated with other congenital anomalies of the gastrointestinal tract was considered to be the same as the health outcomes associated with atresia and/or stenosis of the abdominal tract.

Post-model processing

Other congenital digestive anomalies are calculated by summing all of the sub-causes of congenital digestive anomalies and subtracting this sum from the total congenital digestive model (by age/sex/year/location). This residual is the prevalence of other congenital digestive anomalies. If this residual is less than 10% of the total congenital digestive anomalies model, the other sub-causes are squeezed down and other congenital digestive anomalies becomes 10% of the total congenital digestive anomalies model.

Other congenital anomalies

In addition, of the specific types of congenital anomalies outlined in the preceding pages, there are a number of other types of defects that may be present at birth. These other congenital defects include anomalies of the ears, eyes, face, and neck; respiratory malformation and diseases; skin disorders; phakomatoses; and other neurological disorders that are not included in the case definition of neural tube defects. Estimates of the YLDs attributable to these other congenital anomalies are derived from a YLL:YLD ratio. This ratio was calculated for all congenital birth defects combined, but excluding congenital heart defects, as the location-age-sex-year-specific ratio of YLLs from the CoD estimates to YLDs from the non-fatal analyses described above. This ratio was then applied to the YLLs estimates for other congenital anomalies to derive estimated YLDs for other congenital anomalies.

COVID-19

Flowcharts

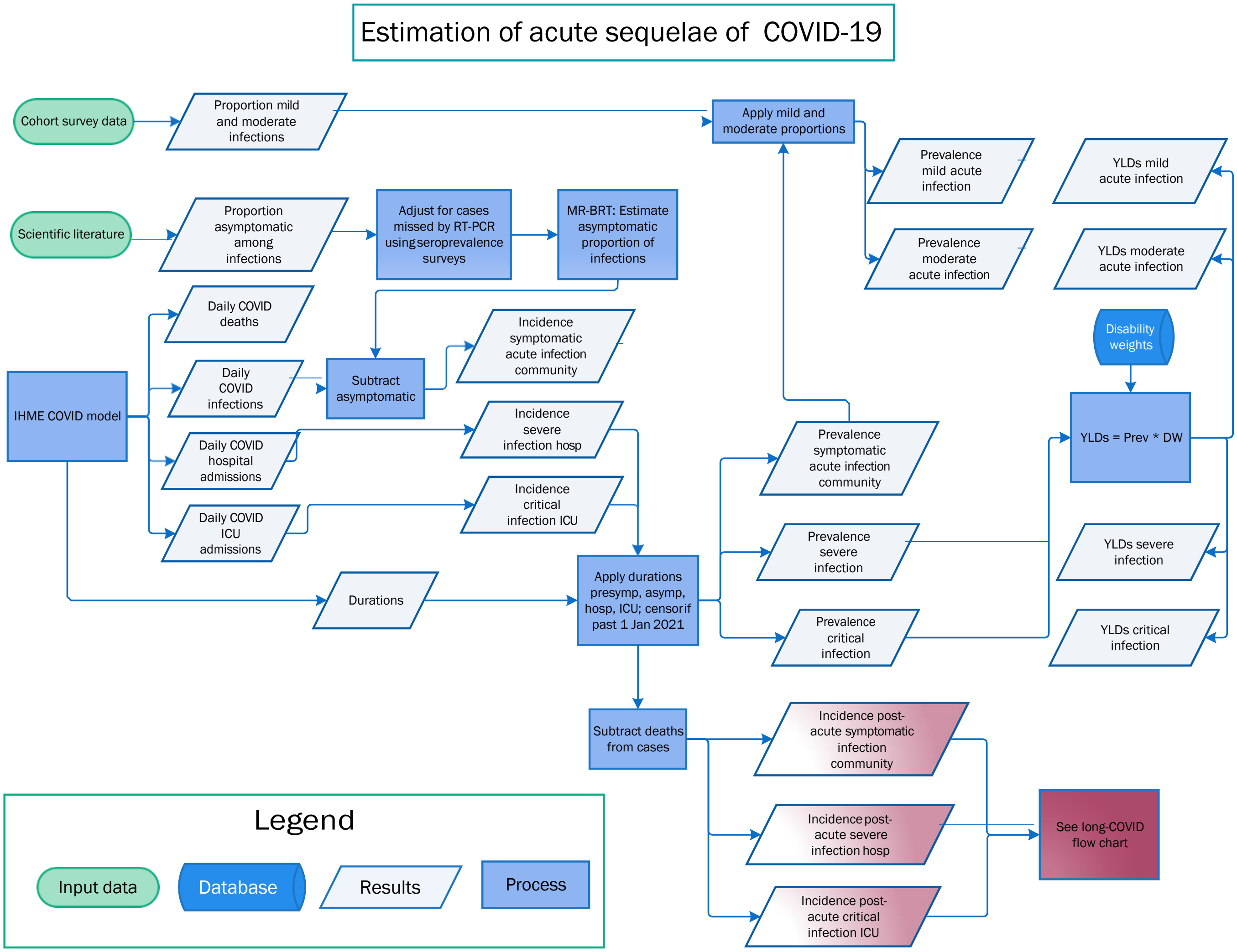

Input data and methodological summary for COVID-19

The analysis is split into two large components: estimating the acute sequelae of COVID-19 and estimating the post-acute sequelae among survivors of COVID-19.

First, estimates of daily infections, hospital admissions, ICU admissions, and deaths due to severe acute respiratory syndrome coronavirus 2 (SARS-CoV-2) infection were taken from the COVID-19 model of the Institute for Health Metrics and Evaluation (IHME).[12,13] Infections were multiplied by the pooled estimate of the proportion of infections without symptoms, and deaths were subtracted from the estimate of

symptomatic cases and to get estimates by age, sex, and country of symptomatic survivors of COVID-19 infection. Then, infections were followed through the disease course to obtain surviving cases of mild/moderate non-hospitalised, severe hospitalised, and critical ICU cases of COVID-19 at risk for post-acute symptoms.

Second, post-acute sequelae were estimated. The proportions of symptomatic survivors with one or more of three symptom clusters of long COVID (with fatigue, cognitive problems, and shortness of breath as the key symptoms) were extracted from international cohort studies and two USA medical record databases. Data from four cohort studies with individual case records available that did not report on excess risk of long COVID symptom clusters in comparison to controls or self-reported health status prior to COVID-19 were adjusted by the ratio of excess to total symptoms from six studies that reported both. Then, the proportions with long COVID symptom clusters by follow-up time since the end of the acute infection were estimated using a Bayesian meta-regression tool, separately for hospitalised and non-hospitalised cases. Subsequently, estimates from studies providing distributions of symptom cluster overlap and severity gradients of cognitive and respiratory problems were pooled. Finally, the global estimates of symptomatic COVID-19 survivors were multiplied by the proportions experiencing one or more of the symptom clusters at three months post-infection.

Case definition

Quantity of interest	Reference or alternative	Definition
Asymptomatic		An asymptomatic case is defined as a person infected with detectable viral load of SARS-CoV-2 but without symptoms.
Community		Community cases of COVID-19 are defined as symptomatic, non-hospitalised, mild/moderate cases of COVID-19.<br>$$inc_{comm} = infections * (1 - prop\ asymp) - hosp\ admissions$$<br>where $\boldsymbol{hosp\ admissions}$ represents the hospital admissions corresponding to infections from 12 days prior, a lag defined in the IHME COVID model from which we derive cases and hospitalisations.
Proportion of deaths in long-term care		Community deaths are defined as deaths due to COVID-19 that occur outside the hospital in long-term care facilities.
Hospitalised cases		Hospitalised cases of COVID-19 are defined as cases of COVID-19 needing hospitalisation but not ICU care, regardless of access or utilisation of care. These cases are calculated from hospital admissions by subtracting corresponding ICU admissions from three days later, the lag assumed in the overall COVID model, as well as severe cases who died outside a hospital in LTC.

ICU cases		ICU cases of COVID-19 are defined as cases of COVID-19 needing ICU care due to critical acute symptoms, regardless of access or utilisation of care.

Modelling strategy

Data inputs

Reported cases data

Data on reported cases primarily come from Johns Hopkins University,[1] supplemented by location-specific datasets extracted either directly from ministries of health, departments of public health, or other third parties. Adjustments to the time series are periodically required, either to account for interruptions in daily reporting due to, for instance, major public holidays, or more systematic issues, such as reporting backlogs of cases accumulated in laboratory processing, or adjustments due to changes in case definitions. A catalog of these corrections is available through the associated GHDx (Global Health Data Exchange; a repository of population health data sources maintained at the Institute for Health Metrics and Evaluation) record.

Hospital admissions data

Data on reported daily admissions, or cumulative hospitalisations, are typically sourced from ministries of health, or multi-jurisdiction agencies such as the USA Department of Human and Health Services, or the European Centres for Disease Control. Adjustments to the time series are periodically required, either to account for interruptions in daily reporting due to, for instance, major public holidays, or more systematic issues, such as changes in COVID case definitions. A catalog of these corrections is available through the associated GHDx record.

Reported deaths data

Data on reported daily deaths primarily come from Johns Hopkins University,[1] supplemented by location-specific datasets extracted either directly from ministries of health, departments of public health, or other third parties. Adjustments to the time series are periodically required, either to account for interruptions in daily reporting due to, for instance, major public holidays, or more systematic issues, such as reporting backlogs of deaths accumulated in vital registration system processing, or adjustments due to changes in case definitions and reconciliation of death certificates. A catalog of these corrections is available through the associated GHDx record.

Full lists of data sources used for cases, hospitalisations, and deaths can be referenced in the appendices of the manuscript by COVID-19 Cumulative Infection Collaborators.[2]

Modelling overview

Our approach can be divided into six steps, which are applied by use of an ensemble model framework.[2] First, we developed a dataset of reported COVID-19 cases, total COVID-19 deaths, and hospitalisations (where available), corrected for known biases such as lags in reporting. Second, we identified representative SARS-CoV-2 seroprevalence surveys that could be used to create a database of cumulative

infections and adjusted them for waning antibody sensitivity, vaccinations, and reinfection from escape variants. Third, using adjusted seroprevalence survey data matched to cases, hospitalisations, and deaths, we created an empirical database of IDRs, infection–hospitalisation ratios (IHRs), and IFRs. Fourth, for locations without seroprevalence surveys and to estimate a complete time series for each location, we developed statistical models to predict the IDR, IHR, and IFR by location and day, as a function of a wide range of covariates. Fifth, three series of estimates of daily infections (cases divided by IDR, hospitalisations divided by IHR, and deaths divided by IFR) were combined into a more robust estimate of daily infections. Sixth, we used the combined time series of daily infections to estimate cumulative infections and the cumulative proportion of the population with one or more infections, and calculate posterior estimates of cumulative IDR, IHR, and IFR.

1. Input data corrections

We make several types of corrections to reported data to take into account common challenges that have emerged during the course of the pandemic. First, for some locations, hospital time series do not have complete time coverage. We impute the missing part of the hospital series using the relationship between hospitalisation and cases and deaths.

Second, we track lags in the reporting for cases, hospitalisation, and deaths for each location. Significant reporting lags could easily lead to incorrect inference about the trend in infections. Including data with reporting lags can lead to false estimates of declining transmission in SEIR models. To avoid that, in locations where we identify major reporting lags, we drop the more recently reported data from the analysis. We have found that reporting lags differ by location and for cases, hospitalisation, and deaths.

2. Adjusting seroprevalence data for vaccination, re-infection due to escape variants, and declining antibody test sensitivity as a function of time since infection

Adjusting for vaccinations

Methods for estimating vaccination rates are described by the COVID-19 Forecasting Team.[3] Seroprevalence studies that use anti-spike tests have been shown to identify the vast majority of individuals tested who have received a vaccine.[4] In order to prevent this from influencing our estimates of cumulative infections, we must determine the proportion of the population that is likely to have been vaccinated but not infected. The formula for this adjustment is:

$$p_{true} = 1 - \frac{1 - p_{obs}}{1 - v \times 0.8}$$

where true sero-prevalence, p_{true} is based on observed sero-prevalence, p_{obs} assuming 80% of vaccinated individuals, v would test positive.[2]

Adjusting for reinfection from escape variants

Methods for estimating variant prevalence are described by the COVID-19 Forecasting Team.[3] In settings with escape variants present, seroprevalence surveys provide an estimate of the cumulative number of individuals with one or more infections. To compute the IFR, IHR, and IDR, we need an estimate of cumulative infections, including re-infections. We estimated the number of cumulative infections from seroprevalence surveys, based on the prevalence of escape variants (Beta, Gamma, and Delta) and an assumed level of cross-variant immunity of 30% to 70% between the escape variants, ancestral variants,

and other variants, such as Alpha, that do not show immune escape. This estimate was derived from an empirical analysis of variant scale-up using our SEIR model. The formula for the correction for escape variant prevalence is:

$$I_t^a = \frac{\sum_{d=1}^{t} i_d^o (1 - p_d^e)}{population}$$

$$U_t = I_t^a (1 - c)$$

$$I_t^{a,e} = \frac{\sum_{d=1}^{t} U_d i_d^o p_d^e}{population}$$

$$S_t = \frac{I_t^o}{I_t^o - I_t^{a,e}}$$

where cumulative ancestral-type infections at time t, I_t^a, is a function of daily observed infections, i_d^o, and daily escape variant prevalence, p_d^e; unprotected population fraction at time t, U_t, is the percentage of individuals exposed to ancestral-strain COVID not protected by cross-variant immunity, c; and ancestral-type infections re-infected with escape-variant COVID at time t, $I_t^{a,e}$, is then the product of unprotected exposed, observed infections, and escape variant prevalence. The adjustment scalar at time t, S_t, was then applied to seroprevalence data in order to account for repeat infections.

Adjusting for sero-reversion

Published studies[5–7] following cohorts of patients with positive viral tests show declining antibody test sensitivity as a function of time since infection. They have shown that different commercial tests have different rates of declining sensitivity, which may be related to the isotype or antigen target. To correct each reported sero-prevalence survey for under-reporting due to declining sensitivity, we used information on the specific test used in each survey, the pattern of declining sensitivity over time, and information on the time pattern of infections. For studies that used assays for which we do not have data on sensitivity decay, we used the average sensitivity curve among the assays we did have after matching on antigen target and isotype. As with the correction for multiple infections, we used an initial approximation of infections in the form of deaths divided by a naïve IFR estimated based on seroprevalence without accounting for sensitivity decay. Independently for each seroprevalence observation, we determined how many past infections would have tested positive based on the number of days between exposure and the midpoint of the serology study dates, factoring in the sensitivity curve matched to the data based on antibody test. We then scaled the seroprevalence data by the ratio of total estimated infections to the cumulative sum of presumed positives.

3. Modelling deaths, hospitalisations, and confirmed cases per infection

3a. Bayesian regression cascade

Models for IFR, IHR, and IDR were fit using MRTool, an open-source Bayesian meta-regression library developed at IHME. We have implemented a "cascading" framework wherein after a global model is fit using all available data, subsequent models are fit using only data pertaining to subsets of a geographical hierarchy with levels for super-region, region, country, and subnational (where possible). We used an adapted version of the Global Burden of Disease location hierarchy in this algorithm. In each of these models, the mean and standard deviation of the coefficients estimated in the "parent" location model were passed on to "child" location models as Gaussian priors. For example, a model for the high-income super-region was fit using data from all locations within that super-region and was also informed by all available data through the priors that were derived from the global model coefficients. Similarly, a model for western Europe used data directly from countries within that region and was also informed by the high-income model through the priors. Taking this a step further down the "cascade," the model for Belgium used only country-specific data and was also informed by the western European parent model through the priors that it used. Locations without seroprevalence data used the parameters estimated from the model of the nearest parent location for prediction.

3b. Estimating the infection-fatality rate

Using seroprevalence surveys where we could match to deaths due to COVID-19, we obtained 2073 direct measurements of the infection-fatality ratio (IFR). Because age is such an important determinant of the IFR, we first analysed the age pattern of the IFR and used that to analyse the broader set of all-age IFR measurements using indirect age-standardisation methods.[2]

For a subset of locations with age-specific data on seroprevalence and reported COVID-19 deaths, we estimate the age-specific IFR directly. We found that the IFR generally increased nearly 10% for each year of age. At the youngest ages, the relationship appeared to be J-shaped, where the IFR decreased from age 0 to 10 and then started increasing steadily with each year of age. Because of the strong relationship with age, we use age-standardised IFR data in subsequent all-age analyses. Because many seroprevalence surveys only provided all-age seroprevalence, we used indirect standardisation methods to generate age-standardised rates. Indirect standardisation computes the ratio of observed IFR to the IFR that is expected based on each location's population age structure and the global age pattern of the IFR.

Patient-level data from registries of USA hospital patients, USA claims data, and Brazil hospitalisations for COVID-19 all show that the hospital-fatality ratio decreased from March 2020 through to late fall and then increased in many settings. The increase in the hospital-fatality ratio may have been due to changes in the tendency to admit moderately severely ill patients to hospital when there was more demand on available hospital beds. These patient-level studies on the hospital-fatality ratio strongly suggest that the prevalence of obesity is an important predictor of the hospital-fatality ratio.

We estimated the logit-transformed age-standardised IFR as a function of time and age-standardised obesity by location. The age-standardisation was reversed when predicting out from the model. Time indexing of IFR data was based on the average date of death for each observation. We used the patient-level data on the hospital-fatality ratio to inform the prior on the obesity coefficient. We also incorporate the conclusion from that analysis that the IFR was declining from March until sometime in the summer or fall. For each location, we tested if the IFR stopped declining in each month from May to November by

running separate linear spline regressions with one knot fixed to the first day of each of those months, where the IFR was allowed to decline in the period preceding the knot and was held constant following that date. We selected the date of inflection for each location based on the best fit to seroprevalence data in the nearest location in the geographical hierarchy with at least one observation later than July 1, 2020, in order to ensure that evaluation was informed by data beyond the nascent stages of the pandemic. Lastly, we accounted for changes in the all-age IFR caused by differential vaccination rates by age, as well as the presence of more lethal variants.

3c. Infection-detection ratio

We have identified 2074 seroprevalence surveys that are representative of the general population in the settings where they were conducted or sampled from populations that can be considered representative, such as blood donors.[2] For each survey, the seroprevalence estimates adjusted for vaccination, waning antibody sensitivity, and re-infection rates were used to estimate cumulative infections. These were then matched with cumulative reported cases to generate an empirical estimate of the average infection-detection ratio over the interval from the beginning of the pandemic to the date of the seroprevalence survey data collection. For the calculation of the IDR, the appropriate lags have been used to match cumulative infections estimated from the seroprevalence survey to cumulative cases to reflect both the average time from infection to getting diagnosed as a case and the lag between infection and becoming antibody-positive. The estimate of the IDR is time-localised to the average date of infection based on the model estimate and daily cases.

We evaluated a number of covariates to predict the IDR (modelled as logit IDR). In the model, we used the log of the infection-weighted average testing capacity at the time of the surveillance observation, where testing capacity was defined as the maximum testing rate at a given date. We then predicted the daily IDR using the observed daily testing capacity. Because even in the beginning of the pandemic when testing rates were low, severely ill patients would have gone to hospital and many would have been diagnosed, we set location-specific floor values for the IDR. To estimate the value for the floor, we used an iterative selection algorithm that tested values between 0.01% and 10% and selected the value that yielded the best fit to the available seroprevalence data.

3d. Infection-hospitalisation rate

By matching seroprevalence surveys to cumulative hospitalisations, we get 1033 direct measurements of the infection-hospitalisation rate (IHR). For a subset of locations with age-specific data on seroprevalence and hospitalisations, we have direct measurements of age-specific IHR. There was a marked relationship where the IHR generally increased nearly 5% per each single year of age. Because of the strong relationship with age, we used age-standardised IHR data in subsequent modelling steps. Many seroprevalence surveys only provide estimates of all-age seroprevalence, so we have used indirect standardisation methods to generate age-standardised rates.

We explored several covariates, including the prevalence of obesity and other comorbidities, but did not find any predictive relationships, so we use an intercept-only model to estimate logit age-standardised IHR. The predicted age-standardised IHR for each location is then converted to an estimate of the all-age IHR that reflects local population age structure, reversing the procedure for indirect age-standardisation. As with the IFR, we account for changes in the all-age IHR caused by differential vaccination rates by age and the presence of escape variants.

4. Smoothed time series of cases, hospitalisations, and deaths

Reporting patterns for cases and deaths exhibit substantial variation according to the day of the week. We also observe characteristic patterns of lagged and then catch-up reporting around holiday periods – such as the last week of December, Easter, and Thanksgiving in the USA. We fit a smooth function to these in two steps. First, we primed the smoother by taking a centred seven-day rolling average of each daily reported measure, allowing every datapoint to be informed by reporting from each day of the week. We then fit a cubic spline to the natural log of those data with a knot every seven days.

5. Daily infections

For each smoothed time series of cases, hospitalisations, and deaths, we generated an estimate of daily infections by dividing by the IDR, IHR, and IFR, respectively. Each estimated sequence of daily infections was shifted in time to take into account the natural history from infection to case identification, hospitalisation, and death. Specifically, we assumed that on average, the time from infection to becoming a diagnosed case was 10–13 days based on individual record of the time from exposure to lab-confirmation.[8] For death, we assumed a lag of 22–28 days from patient-level data in the USA.[9] There may be variation in the lag between infection and various outcomes across locations and over time, but in this analysis, we assumed these lags did not vary.

The approach we used to combine the series into a single composite estimate of daily infections was designed to deal with the compositional bias problem caused by varying temporal coverage among cases, hospitalisations, and deaths, or due to different lags in the time between infection and those events. The unit of the analysis in the initial stage of synthesising these measures was the first difference in log daily values. We incorporated these data into a random knots spline regression using MRTool without the cascading framework, wherein we provided a number of knots and a number of unique knot combinations to an algorithm that ran a model with each combination and made a weighted composite estimate from the sub-models based on in-sample performance. We specified one knot per 28 days of data and tested 100 random knot combinations of a quadratic spline. We then converted the estimate into ln(daily) values by taking the cumulative sum and found the initial value of the composite time series by fitting a model to the average ln(daily) residual of the three original curves with respect to the composite.

To incorporate uncertainty in our infections estimate based on the consistency of our three inputs, as well as measurement error in those data, we performed additional steps to create samples of our infections curve reflective of that error. We first converted the observed daily cases, hospitalisations, and deaths into "observed" infections by dividing them by the estimated time series of IDR, IHR, and IFR, respectively. We then used the log of these values to compute the residuals with respect to the mean infections curve we have estimated in the previous step and calculated the robust standard deviation. With that, we independently sampled 1000 infections for each day, which gave us 1000 uncorrelated time series of ln(daily infections) that were representative of the noise in the raw data. We then refitted curves to these noisy series using our random knots spline model; in this step, we used a cubic spline based on one randomly sampled knot combination per time series draw, again based on one knot per 28 days of data, to produce 1000 smooth past infections curves.

6. Cumulative infections

We then used daily infections to estimate cumulative infections and the cumulative proportion of the population with one or more infections, and we then calculated posterior estimates of cumulative IDR,

IHR, and IFR using cumulative infections and the corrected data on reported cases, hospitalisations, and deaths.

Acute sequelae of SARS-CoV-2 infection

Asymptomatic cases

Case definitions

An asymptomatic case is defined as a person infected with detectable viral load of SARS-CoV-2 but without symptoms.

Data

Data sources were obtained from a published systematic literature review which contains the proportion of confirmed positive COVID cases through antibody testing that were asymptomatic, from studies across the world.[10]

We have two primary inclusion criteria: 1) antibody screening studies; and 2) randomly selected sample to increase representativeness. Of the 18 antibody screening studies included in the review, six met our inclusion and exclusion criteria (Error! Not a valid bookmark self-reference.2).

Table 2. Input data of proportion asymptomatic among COVID infections

Author	Location	Sample
Ward et al.[11]	China	17 576
Pollán et al.[12]	Hubei	3053
Da Silva et al.[13]	Shandong	1167
Feehan et al.[14]	Bahrain	311
Hippich et al.[15]	Hubei	47
Mahajan et al.[16]	Guangdong	23

[Step 1]

The standard error of each datapoint was calculated using the following equation for a binomial distribution.

$$Standard\ error = \sqrt{\frac{proportion_{asymp} * (1 - proportion_{asymp})}{sample\ size}}$$

[Step 2]

Methods

First, we pooled the studies using a simple random effects model with the MR-BRT tool in logit space to constrain the estimate between 0 and 1 (Figure 5Error! Reference source not found.). The delta method w as used to convert the standard error into logit space for the meta-analysis.

Figure 5. Pooled estimate of proportion asymptomatic among SARS-CoV-2 infections

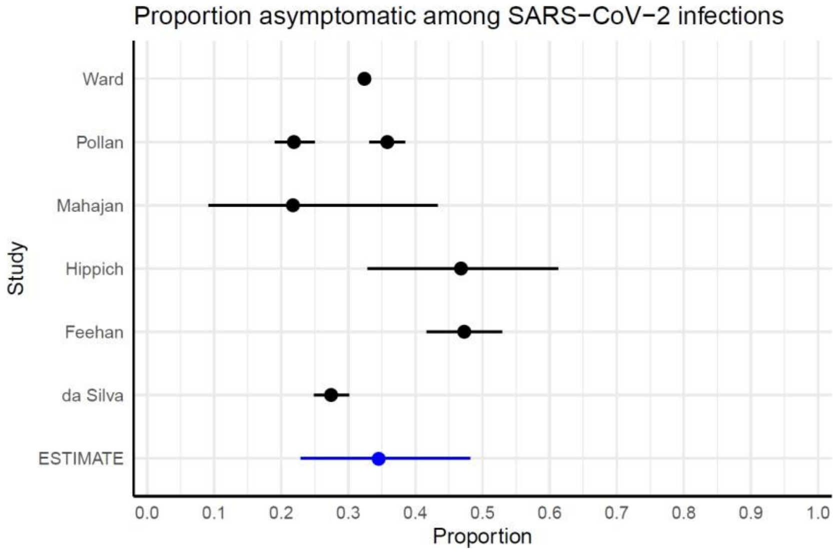

The data are high-quality but heterogeneous in the observed proportions asymptomatic, ranging from 22% to 47% asymptomatic. This could be due to differential rather than consistent antibody testing capture of SARS-CoV-2 infections in different settings, true variation in the proportion asymptomatic due to different underlying risk factors in the study populations, or differential symptom recall by the patients in these studies.

Cases at risk for long COVID

Asymptomatic cases are assumed to not be at risk for long COVID, due to lack of data. Five cohorts included asymptomatic cases: the UW Coronavirus Cohort (HAARVI), Faroe Islands, Zurich SARS-CoV-2 Cohort, Rome ISARIC pediatrics, and Rome ISARIC adults cohorts, with 9, 22, 182, 27, and 26 cases, respectively, that were asymptomatic during the acute COVID episode. Long COVID, according to our definition, was not identified among asymptomatic cases that were followed in HAARVI and Rome ISARIC cohorts. In the Faroe Islands cohort, three patients who did not report any symptoms during the acute phase developed long COVID symptoms, and in the Zurich SARS-CoV-2 Cohort of 182 asymptomatic infections, five developed at least one long COVID symptom cluster at one or three or six months' follow-up. The two cohorts did not explicitly measure a difference in symptoms compared to before COVID infection. From the available information, we cannot preclude that there is some risk of long COVID among asymptomatic cases, but the number of cases in the available studies is very small and we prefer to be cautious and exclude them from our calculations until stronger evidence is available.

Community cases

Case definition

Community cases of COVID-19 are defined as symptomatic, non-hospitalised, mild/moderate cases of COVID-19.

$$inc_{comm} = infections * (1 - prop\ asymp) - hosp\ admissions$$

where $hosp\ admissions$ represents the hospital admissions corresponding to infections from 12 days prior, a lag defined in the IHME COVID model from which we derive cases and hospitalisations.

Proportion of deaths in long-term care

Case definition

Community deaths are defined as deaths due to COVID-19 that occur outside the hospital in long-term care facilities.

Data

Data sources were obtained from online reports in the Netherlands, Belgium, France, and all USA states which contain the proportion of COVID-19 deaths which occurred in LTC.[17–20]

The standard error of each datapoint was calculated using the following equation for a binomial distribution.

$$Standard\ error = \sqrt{\frac{proportion_{LTC} * (1 - proportion_{LTC})}{sample\ size}}$$

Methods

We pooled the studies using a simple random effects model with the MR-BRT tool in logit space to constrain the estimate between 0 and 1, trimming 10% of the datapoints (Figure 6).

Figure 6. Pooled estimate of proportion of COVID-19 deaths that occurred in long-term care facilities

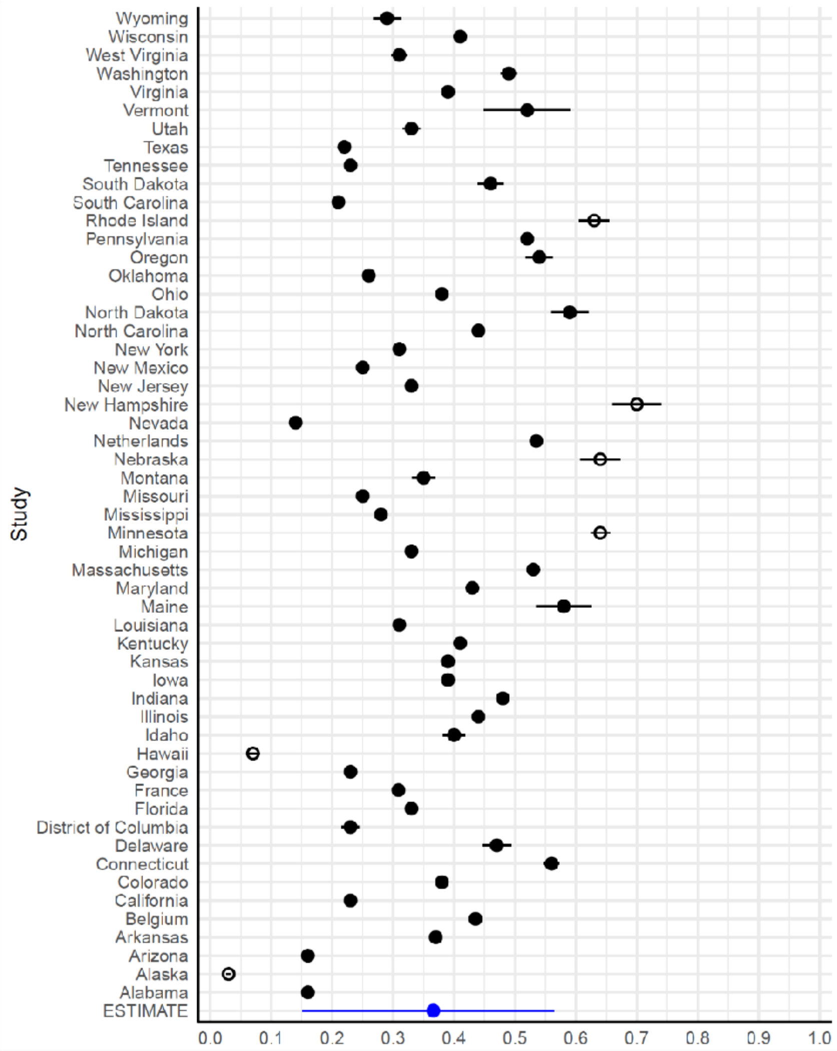

The resulting estimated proportion of deaths that occurred in long-term care facilities was 36.2% (95% UI 14.4–57.0). We accounted for all estimated deaths from the COVID SEIR model by multiplying this proportion by deaths to obtain community deaths, multiplying hospitalised non-ICU and ICU admissions by age-specific case-fatality ratios (described below in "Proportion deaths among hospitalised and ICU cases") to obtain hospitalised and ICU deaths, and proportionally scaled these three counts of deaths to the total number of deaths by age/sex/location/day.

This analysis assumes that among COVID-19 cases who die, their probability of dying in LTC facilities does not differ by age. There are currently insufficient data to evaluate the validity of this assumption.

Hospitalised cases

Case definition

Hospitalised cases of COVID-19 are defined as cases of COVID-19 needing hospitalisation but not ICU care, regardless of access or utilisation of care. These cases are calculated from hospital admissions by

subtracting corresponding ICU admissions from three days later, the lag assumed in the overall COVID model, as well as severe cases who died outside a hospital in LTC.

Proportion deaths among hospitalised and ICU cases

Data

Age-specific data on COVID deaths among hospitalised and/or ICU patients proved extremely difficult to find, and we found only one comprehensive source with this level of detail from the Netherlands COVID-19 ICU online dashboard.[21]

Methods

Case fatality among hospitalised and ICU patients was extracted and fit with a sixth-order polynomial to most closely follow the curves of the data so that case-fatality estimates could be extracted for every five-year age group (Figure 7). The value for case fatality for age group 5–9 was extrapolated back to age 0 due to lack of data at the very young ages.

Figure 7. Case-fatality ratios among hospitalised and ICU COVID-19 patients by age

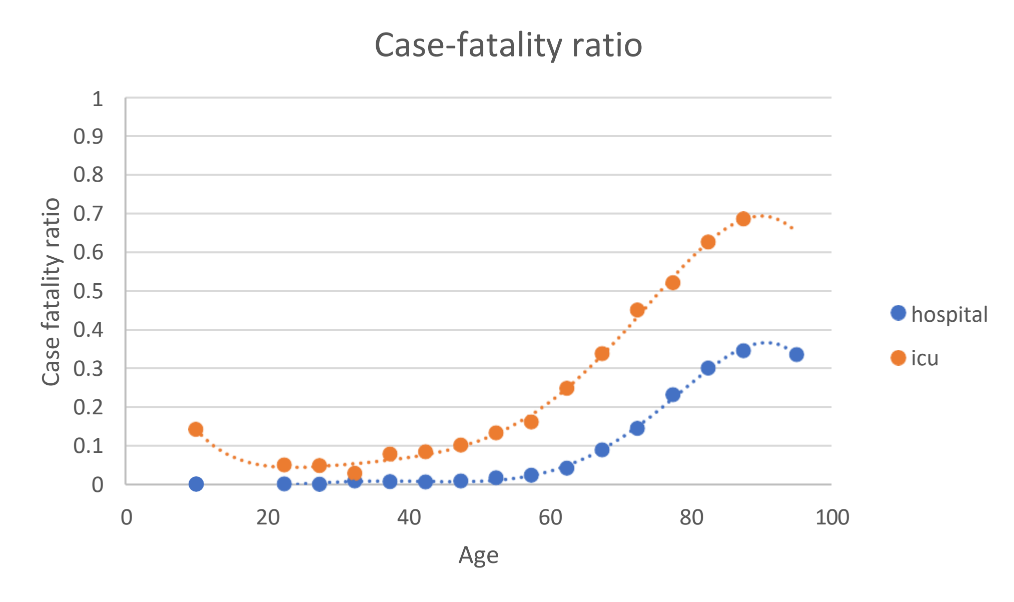

ICU cases

Case definition

ICU cases of COVID-19 are defined as cases of COVID-19 needing ICU care due to critical acute symptoms, regardless of access or utilisation of care.

Cutaneous and mucocutaneous leishmaniasis

Flowchart

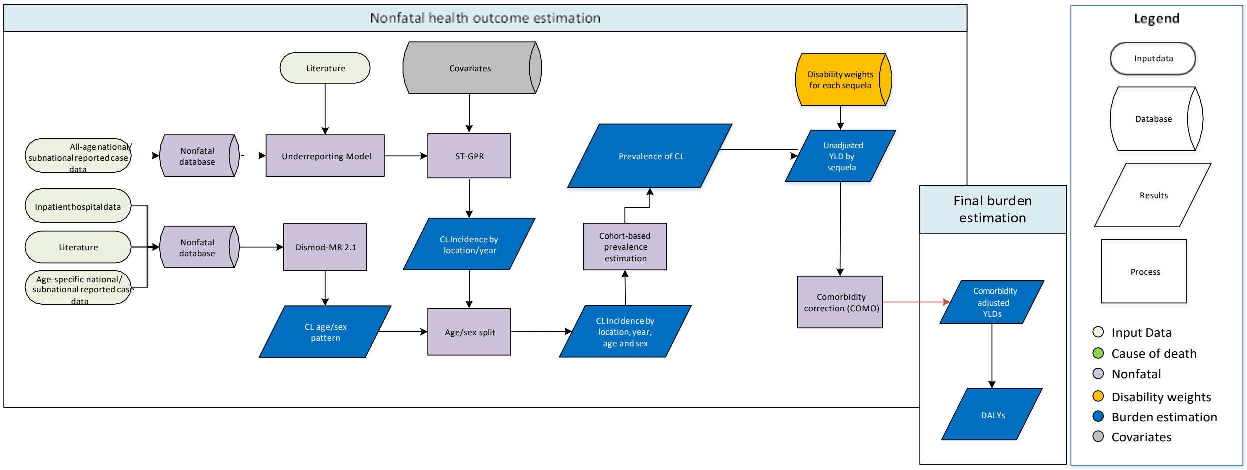

Input data and methodological summary

Case definition

Cutaneous and mucocutaneous leishmaniasis (CL) is a group of syndromes caused by the *Leishmania* parasite, transmitted through the bite of phlebotomine sandflies. The most common form is cutaneous leishmaniasis, and those infected typically present with a well-demarcated skin lesion at the site of the sandfly bite. This initial lesion is usually painless and may enlarge and ulcerate, developing a scab or crust. Mucocutaneous leishmaniasis is less common and begins with a primary cutaneous ulcer progressing to partial or complete destruction of nasopharyngeal tissues. The ICD-9 codes related to CL are 085.4 and 085.5, and the ICD-10 codes are B55.1 and B55.2.

We used the following case definition for GBD 2021:

Quantity of interest	Reference or Alternative	Definition
Cutaneous and mucocutaneous leishmaniasis	Reference	A person identified as a case through a case notification system or by clinical diagnosis of skin lesions, with or without laboratory confirmation.

Description of general methodology

The non-fatal estimation process for cutaneous leishmaniasis builds from incident case notification data representative of the GBD geographical location, which are adjusted for under-reporting. The upscaled all-age, both sex, case counts are modelled using spatiotemporal Gaussian process regression (ST-GPR) in order to impute for missing location-year combinations as well as to account for further biases and inaccuracies in reporting. Datasets that disaggregate CL cases by age and sex are modelled using DisMod-MR to produce location-specific age-sex splits, which are applied to all-age, both-sex envelope estimates resulting from ST-GPR. These incidence estimates are used to derive prevalence measures, as well as compute the resulting years lived with disability values.

Input data – case notification time series

Current estimation for the all-age, both-sex incidence envelope is based upon location-representative information rather than site-specific epidemiological measures due to the absence of global foci maps allowing for upscaling of geographically precise information. The primary input data are case notification time-series reported by National Control Programs, Ministries of Health, and the World Health Organization (WHO). This is supplemented by systematic literature review (last updated for GBD 2015) to identify alternate sources of data for years missing information. For countries with subnational estimates, in-country collaborators have compiled information for respective programmes, or identified key resources, again supplemented by literature reviews. Where possible, information disaggregating location-level statistics by age and sex were extracted.

Table 1: Data inputs for CL morbidity modelling by parameter

Measure	Countries with data	New sources	Total sources
All measures	55	4	662
Prevalence	2	4	4
Incidence	54	0	658

Input data – under-reporting adjustment

Case count data were translated into estimates of true case counts by using under-reporting scalars as identified by Alvar et al. (2012). This analysis provided estimates of plausible incidence ranges of CL based on published data and expert judgment of the magnitude of under-reporting. The incidence ranges were determined at the country and/or region level and were based on reported estimates multiplied by probable under-reporting factors.

Method – geographical restrictions

There are strong climatic and biogeographical constraints on the geographical distribution of CL, resulting in a focal, rather than global distribution. As a result, it is necessary to identify locations burdened by the disease through space and time as distinct from countries where CL is absent. Tags were assigned to each location-year based upon the outcome of a search of IHME databases, as well as location-specific searches of PubMed. Each location-year is tagged as follows:

- Present – where a specific citation of either an autochthonous laboratory-confirmed case (ie, a case with PCR, serological, or parasitological diagnosis), reported case (ie, a case noted as CL, but with no supporting diagnostic), or supporting evidence (ie, confirmed infection in animal reservoirs or sandfly vectors)
- Protocol Present – for a given location-year, where no specific citation is used, butis present for another year in the same location, it is assumed that CL is present given that eradication of the pathogen has not been achieved
- Absent – where PubMed location-specific searches returned zero relevant results, in locations scoring -25 or lower as evaluated by Pigott and colleagues (2014) [the threshold for “absence” in that study], locations were tagged as Absent
- Protocol Absent – as with Absent, locations with zero relevant PubMed results, but with greater than -25 as evaluated by Pigott and colleagues (2014), were tagged as Protocol Absent

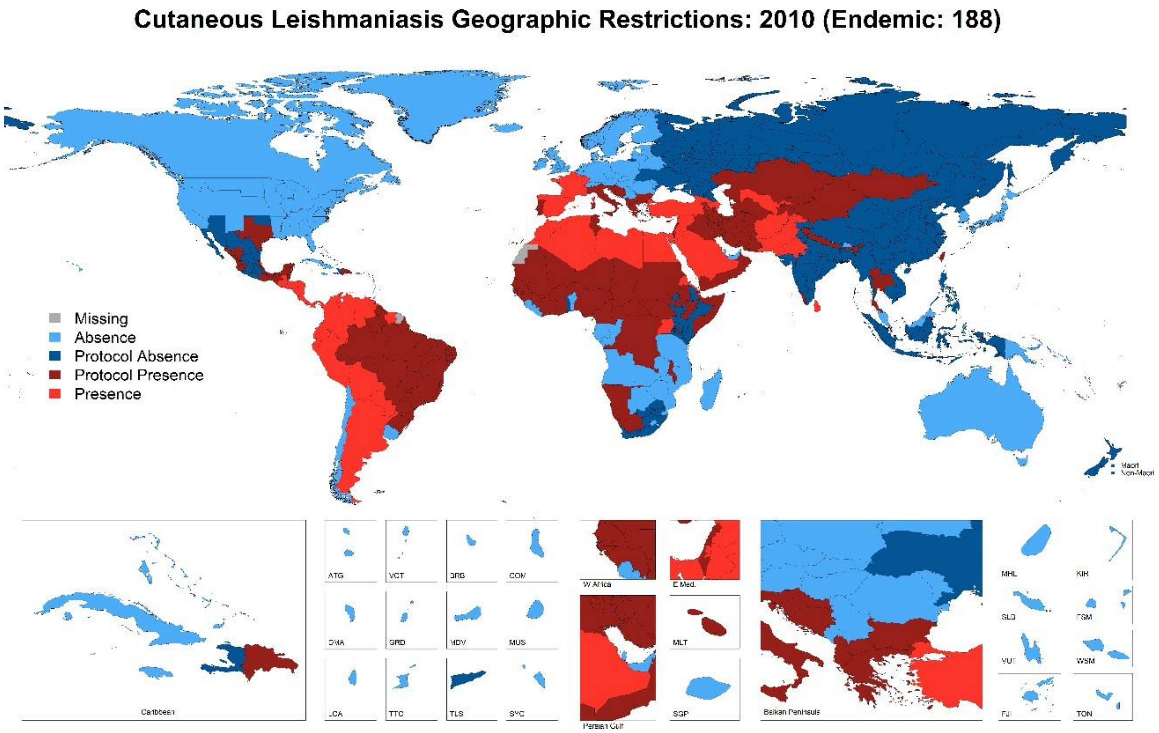

Figure 1: Cutaneous Leishmaniasis geographical restrictions for the year 2010. GBD locations tagged as present are coloured in red, dark red represents protocol presence, dark blue represents protocol absence, and absence is represented by light blue. Locations missing tags are presented in grey.

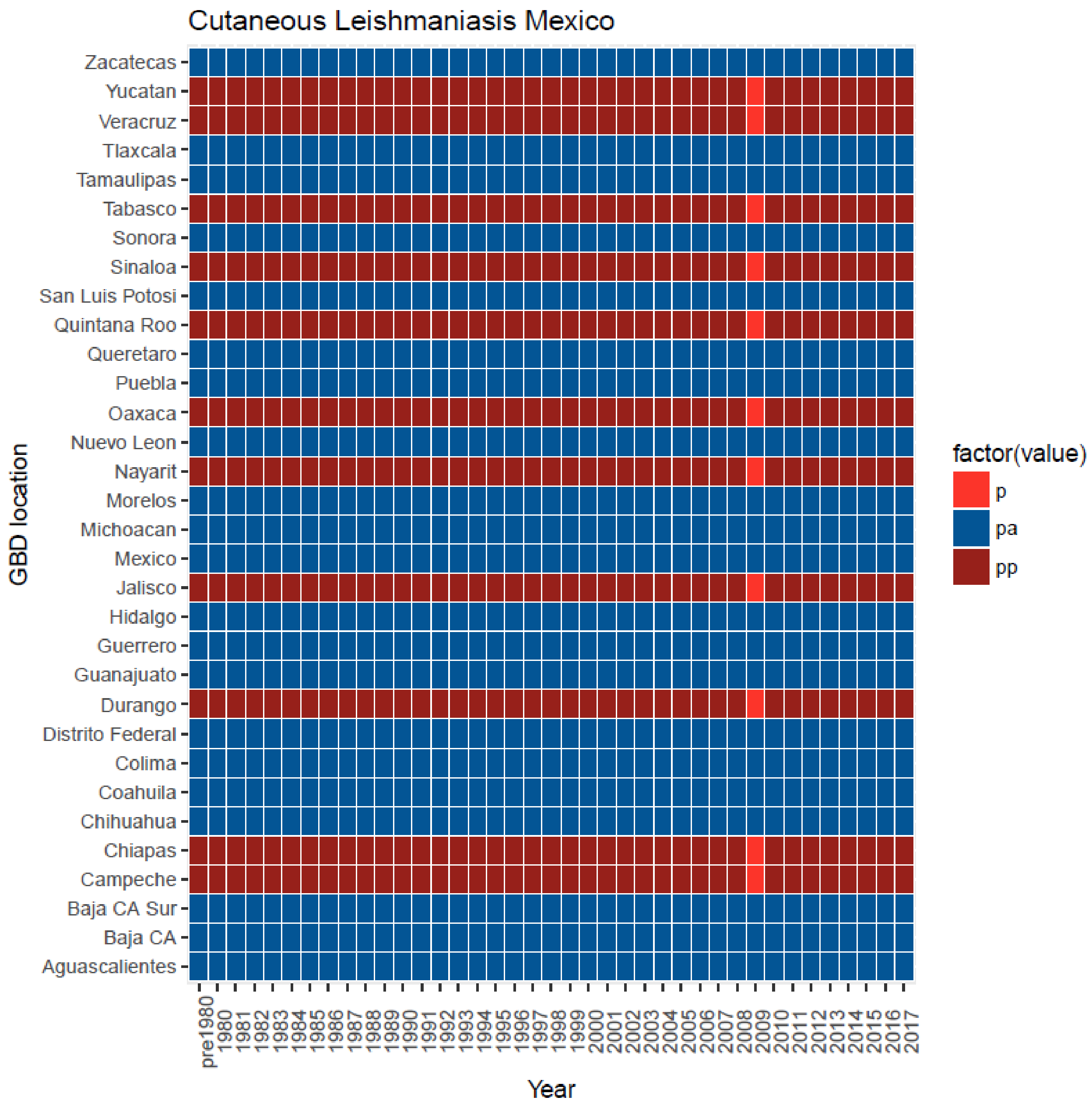

Figure 2: Cutaneous Leishmaniasis geographical restrictions for Mexican subnationals. Locations tagged as present are coloured in red, dark red represents protocol presence, and dark blue represents protocol absence.

Method – ST-GPR

The summarised values were modelled using ST-GPR to produce a complete time series of estimates for each location-year tagged “Present” or “Protocol Present”. In short, ST-GPR attempts to model non-linear trends utilising a Gaussian process to fit a trend, rather than a definitive functional form. The following were the model specifications:

$$Incidence = Leishmaniasis\ Endemicity + Sanitation\ Proportion + (1|level\ 1) + (1|level\ 2) + (1|level\ 3)$$

Levels 1, 2, and 3 refer to GBD location hierarchies, treated as nested random effects by super-region, region, and country, respectively. The following hyperparameters were used: st-lambda = 0.5, st-omega = 1.0, st-zeta = 0.01 and gpr-scale = 1. The table below lists coefficients of the covariates.

Table 2a: Covariates. Summary of covariates used in the CL ST-GPR model

Covariate	Beta coefficient, logit (95% UI)	Standard error	Exponentiated beta (95% UI)
Leishmaniasis endemicity	1.57 (1.35–1.79)	0.11	4.81 (3.87–5.96)
Sanitation proportion	-1.34 (-1.93 to -0.75)	0.30	0.26 (0.15–0.47)

Method – DisMod-MR

DisMod-MR was used to generate location- and sex-specific age curves to disaggregate all-age, both-sex incidence data. DisMod-MR is an integrated meta-regression framework that allows multiple datasets to be integrated into a singular analysis regardless of age-binning, sources, and geographies. This allows evaluation of a variety of differently aggregated information to generate a consensus output.

Table 2b: Covariates. Summary of covariates used in the CL DisMod-MR model

Covariate	Type	Parameter	Exponentiated beta (95% UI)
Socio-demographic Index	Country-level	Incidence	0.32 (0.29–0.36)
Healthcare Access and Quality Index	Country-level	Incidence	1.20 (1.09–1.36)

Method – YLD estimation (incorporating duration and disability weighting) / COMO

Following standard GBD estimation protocols, incidence estimates were used to calculate disease prevalence (by multiplication with duration), disaggregated by disease sequelae. One health state is assigned to Cutaneous Leishmaniasis, [Table 3]. Duration value of initial acute infection was set to six months (Reithinger et al. 2007). Prevalence of long-term sequelae was based upon the proportion of cases that would result in facial scarring. The average proportion of sores that occurred on the face was calculated based upon a sample-weighted average of the proportion from four studies conducted in North Africa and the Middle East [4-7]. This proportion was 0.476. Of these people, only those who did not have appropriate access to health care were assigned long-term sequelae, estimated via the Healthcare Access and Quality Index. CL incidence, multiplied by proportion of people with facial sores, times the proportion of people without adequate health-care access in each location-year, was used to obtain incidence of people with long-term sequelae, with cohorts streamed through time.

Table 3. Severity distribution, details on the severity levels for CL and the associated disability weight (DW) with that severity

Sequela	Health state lay description	DW (95% CI)	Duration
Cutaneous and mucocutaneous leishmaniasis	"has a slight, visible physical deformity that others notice, which causes some worry and discomfort"	0.011 (0.005–0.021)	6 months<br>(46.7% * HAQ Index) lifelong

Central processing generates the final estimates, including comorbidity simulations.

Changes from GBD 2019

There were no substantive changes implemented in GBD 2021. We did not apply any adjustments for the COVID pandemic to CL due to a lack of available data quantifying the impacts of the pandemic on NTD epidemiology.

Limitations

As with any modelling process, a number of limitations are known, which will be the focus of additional effort in upcoming GBD cycles and engagement with collaborators. Given the focus on location-representative estimates, the existing model is focused on national case counts. This excludes a large resource of published literature and grey literature focused on site-specific surveillance or surveys. While some pathogens have integrated subnational approaches as a building block for national estimates (eg, schistosomiasis) this has yet to be implemented for cutaneous leishmaniasis. Regardless of contribution to the global incidence model, these data can be used to inform age-sex splits, as well as a variety of other key parameters, particularly duration parameters, which are currently lacking uncertainty.

References

1. Alvar J, Vélez ID, Bern C, *et al.* Leishmaniasis Worldwide and Global Estimates of Its Incidence. *PLoS One* 2012; 7.
2. Pigott DM, Bhatt S, Golding N, *et al.* Global distribution maps of the leishmaniases. *eLife* 2014; 3. DOI:10.7554/eLife.02851.
3. Reithinger R, Dujardin J-C, Louzir H, Pirmez C, Alexander B, Brooker S. Cutaneous
4. leishmaniasis. *The Lancet Infectious Diseases* 2007; 7: 581–96.
5. Momeni AZ, Aminjavaheri M. Clinical picture of cutaneous leishmaniasis in Isfahan, Iran. *Int J Dermatol* 1994; 33: 260–5.
6. Gurel MS, Ulukanligil M, Ozbilge H. Cutaneous leishmaniasis in Sanliurfa: epidemiologic and clinical features of the last four years (1997-2000). *Int J Dermatol* 2002; 41: 32–7.
7. Sharifi I, Fekri AR, Aflatonian MR, Nadim A, Nikian Y, Kamesipour A. Cutaneous leishmaniasis in primary school children in the south-eastern Iranian city of Bam, 1994-95. *Bull World Health Organ* 1998; 76: 289–93.
8. Mujtaba G, Khalid M. Cutaneous leishmaniasis in Multan, Pakistan. *Int J Dermatol* 1998; 37: 843–5.

Cystic echinococcosis

Flowchart

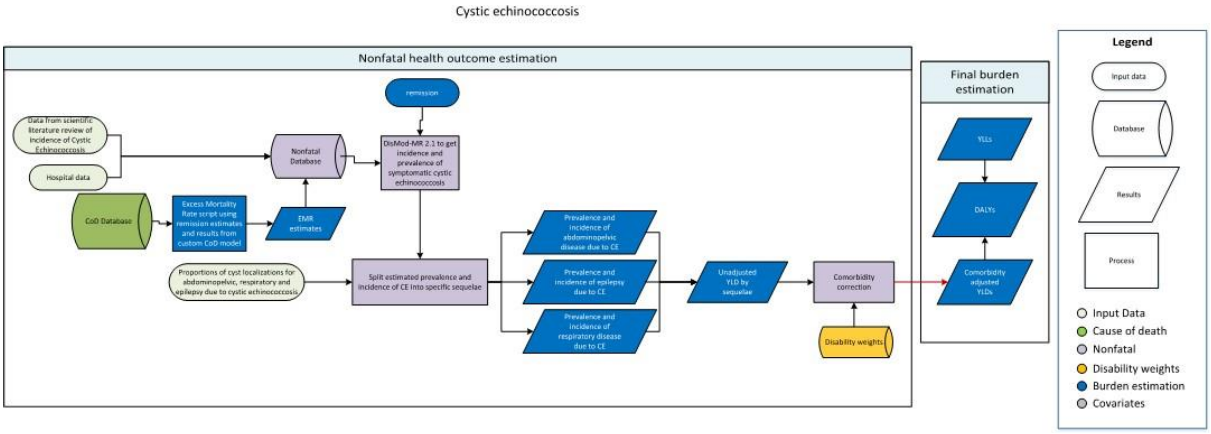

Input data and methodological summary

Case definition

Cystic echinococcosis is an infection with Echinococcus tapeworms, which are transmitted primarily via consumption of food, water, or soil contaminated with animal feces. Larval growth causes cyst formation in different parts of the body, especially the liver, lungs, and central nervous system; symptoms include abdominal pain, respiratory distress, and neurologic symptoms including seizures. Diagnosis is made by clinical findings, imaging, serology, and tissue pathology. The ICD-9 and ICD-10 codes for echinococcosis are 122.0-122.9 and B67-B67.9, respectively.

Cystic echinococcosis

**Quantity of interest**	**Reference or Alternative**	**Definition**
Cystic echinococcosis	Reference	Diagnosis of cystic echinococcosis based on imaging findings or clinical findings, based on ICD-9 codes 122.0-122.9 or ICD-10 codes B67-B67.9.

Input data

Table 1: Source counts

Measure	Total sources	Countries with data
All measures	353	62
Incidence	353	62

Systematic literature review

The non-fatal estimation for cystic echinococcosis (CE) focused on estimating incidence and prevalence of CE and its sequelae. A systematic review of literature was conducted in PubMed for GBD 2015 using the following search string:

> ("echinococcosis"[Title/Abstract] OR "hydatid disease"[Title/Abstract] OR "hydatidosis"[Title/Abstract] OR "echinococcal disease"[Title/Abstract] OR "Echinococcus

granulosus infection"[Title/Abstract]) AND (“1990”[Date – Publication] : “2015”[Date – Publication]) AND (epidemiology OR incidence OR prevalence).

This yielded 1,619 studies of which 279 were included during the title/abstract screening. Following the full-text screening, 77 studies (32 incidence, 43 prevalence, and 2 both) were included and extracted – studies were excluded because of one or more of the following reasons:

5. study not population-based
6. study does not have primary data on prevalence and/or incidence
7. study not in humans
8. study on sub-populations
9. review study

Since we were interested in modelling symptomatic CE cases, we only used data on incidence of patients diagnosed by imaging techniques (mainly ultrasonography). Therefore, we excluded prevalence data, which were mostly from serological studies. Data from these extracted studies were combined with data from studies extracted during GBD 2013.

Hospital data

Hospital data prepared by the GBD team were used as additional input into our models. These data were adjusted to account for multiple hospital episodes of a single case and non-primary diagnoses.

Geographic restrictions

We conducted a literature review to determine the geographic extent of the disease and classify locations based on whether the disease is absent or present in each year. Locations that were geographically restricted in any given year did not have estimates made. Of note, we did not attempt a complete systematic review, since a single high-quality source could offer sufficient evidence of presence. Evidence of absence or presence was not available for every location for each year, and so assumptions were made for missing years by taking into consideration the epidemiological characteristics of the disease.

If evidence indicated disease presence for two non-consecutive years, we assumed presence for all years between the two. If evidence indicated disease absence for two non-consecutive years, we assumed absence for all years between the two. If evidence indicated a change in status (ie, from absent to present, or present to absent) between two non-consecutive years, then we conducted targeted searches to ascertain the relevant year of introduction or elimination for that location. In the cases where presence or absence information was missing for the start or end years of our study interval (1990–2019) without evidence of any introduction or elimination events within the interval, we applied the status of the first and last presence/absence observations respectively to all years between the interval bound and the observation year. For cystic echinococcosis, we performed targeted searches to classify location-years in PubMed and Google Scholar. Geographic restrictions were populated by reviewing sources referenced by Deplazes and colleagues along with ad hoc searches in PubMed for evidence of active transmission of cystic echinococcosis in respective countries [1].

Modelling strategy

The morbidity model for cystic echinococcosis involved a multi-step process. First, incidence data reported for both sexes was first split into sex-specific inputs. To sex-split our both-sex datapoints, we used sex-specific inputs in a Bayesian meta-regression (MR-BRT) model to derive a ratio of female cystic echinococcosis incidence to both-sex incidence (from scientific literature data). The resultant log ratio was applied to both-sex datapoints to calculate out females, and males were calculated via subtraction. The beta coefficients of the adjustment are presented in Table 2.

Table 2: MR-BRT crosswalk adjustment factors for cystic echinococcosis

Data input	Reference or alternative case definition	Gamma	Beta Coefficient, Log (95% UI)*	Adjustment factor**
Females	Ref	0.32	---	---
Both sex	Alt		0.15 (-1.03; 1.33)	1.16

**MR-BRT crosswalk adjustments can be interpreted as the factor the alternative case definition is adjusted by to reflect what it would have been had it been measured using the reference case definition. If the log/logit beta coefficient is negative, then the alternative is adjusted up to the reference. If the log/logit beta coefficient is positive, then the alternative is adjusted down to the reference.*

***The adjustment factor column is the exponentiated beta coefficient. For log beta coefficients, this is the relative rate between the two case definitions. For logit beta coefficients, this is the relative odds between the two case definitions.*

We then split all-age case data into age-specific observations using a global age pattern derived from a DisMod Bayesian Meta-Regression model (DisMod-MR). The age pattern was developed using a single-parameter incidence model in DisMod-MR. Uncertainty was propagated throughout the sex- and age-splitting processes, such that final sex- and age-specific incidence estimates reflect the uncertainty of the original data.

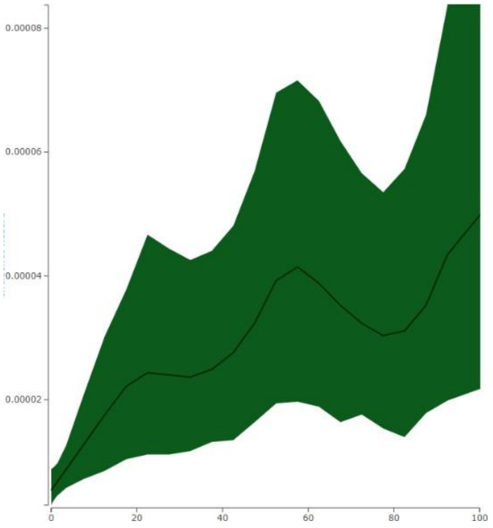

Figure 1. Global age pattern of cystic echinococcosis incidence produced by DisMod-MR

Then, DisMod-MR was used to model incidence and prevalence of symptomatic cystic echinococcosis using incidence data from systematic reviews in GBD 2013 and 2015 and hospital data, excess mortality rate (EMR) estimates, and an assumed remission of 0.15–0.25 per case per year (duration 2–6.7 years, average 5 years). We used urbanicity, echinococcosis endemicity, and proportion of population involved in agricultural activities as country-level covariates. To estimate the EMR used in the final DisMod-MR model, we first fit an initial DisMod-MR model using the input data, covariates (excluding EMR), and allowing remission to range from 0 to 1. We then obtained predictions of EMR from this initial DisMod-MR model, which were in turn as input data for a meta-regression—Bayesian, regularised, trimmed (MR-BRT) model. The MR-BRT model estimated EMR for all national level locations, using the Healthcare Access and Quality (HAQ) Index, age, and sex as covariates. This approach produced predictions of remission and EMR that were 3.5 times higher than values predicted from previous models at the global level. We therefore adjusted all predicted EMR values, dividing by 3.5 in order to return the overall scale of EMR to that predicted by previous models, while also leveraging the relationships between HAQ Index and EMR estimated by the MR-BRT model. Last, the predicted EMR values from the MR-BRT model, after rescaling, were used as inputs into our final DisMod-MR model. This approach helps to ensure that the excess mortality trends implied by the final DisMod-MR model better match expected patterns across different levels of HAQ.

Geographic restrictions were applied to set incidence and prevalence to zero in location-years where the disease was not endemic.

Table 3. DisMod-MR model covariates

Covariate	Type	Parameter	Exponentiated beta
Sex	Study-level	Incidence	1.00 (0.96; 1.04)
Urbanicity	Country-level	Incidence	1.00 (1.00–1.00)
Echinococcosis endemicity	Country-level	Incidence	20.08 (20.07; 20.09)
Proportion of population involved in agricultural activities	Country-level	Incidence	1.20 (1.01; 1.44)
Sex	Study-level	Excess mortality rate	1.60 (1.59; 1.61)

After producing all-case prevalence draws, 1,000 draws of proportions for abdominal, respiratory, and epileptic symptoms among echinococcosis cases adding up to 1 were generated. Uncertainty in the splitting proportions was captured by drawing them from a Dirichlet distribution, informed by published data on cysts localisation [2]. On average, the proportions of abdominal, respiratory, and epileptic symptoms due to echinococcosis were 0.5, 0.47, and 0.03, respectively. These proportions were used to split the prevalence and incidence from DisMod-MR into the three sequelae.

Model evaluation was done by separately assessing the fit of the DisMod-MR model and checking the estimates produced after estimating incidence and prevalence of sequelae due to cystic echinococcosis. Plots of time trends of incidence and prevalence across locations and age were used to evaluate the

results. In addition, maps of the global distribution of incidence and prevalence were assessed across time.

Sequelae due to cystic echinococcosis

The table below shows the sequelae due to echinococcosis and their associated disability weights.

Table 4. Sequelae, lay descriptions, and disability weights (DWs)

Sequela	Lay description	DW (95% CI)
Chronic respiratory disease	"has cough and shortness of breath after heavy physical activity, but is able to walk long distances and climb stairs."	0.019 (0.011–0.033)
Abdominal problems	"has pain in the belly and feels nauseated. The person has difficulties with daily activities."	0.114 (0.078–0.159)
Epilepsy	(Combined DW)	NA

Changes from GBD 2019 to GBD 2021

The major change that we implemented this cycle was to calculate the EMR for all country-level locations using the MR-BRT model, in previous cycle the EMR was calculated using a custom model.

We did not apply any adjustments for the COVID pandemic to cystic echinococcosis due to a lack of available data quantifying the impacts of the pandemic on NTD epidemiology.

References

1. Deplazes P, Rinaldi L, Alvarez Rojas CA, Torgerson PR, Harandi MF, Romig T, Antolova D, Schrurer JM, Lahmar S, Cringoli G, Magambo J, Thompson RC, Jenkins EJ. Global Distribution of Alveolar and Cystic Echinococcosis. Advanced Parasitology. 2017. 95: 315-493.
2. Raether W, Hänel H. Epidemiology, clinical manifestations and diagnosis of zoonotic cestode infections: an update. Parasitology Research. 2003. 91:412-438.

Cysticercosis

Flowchart

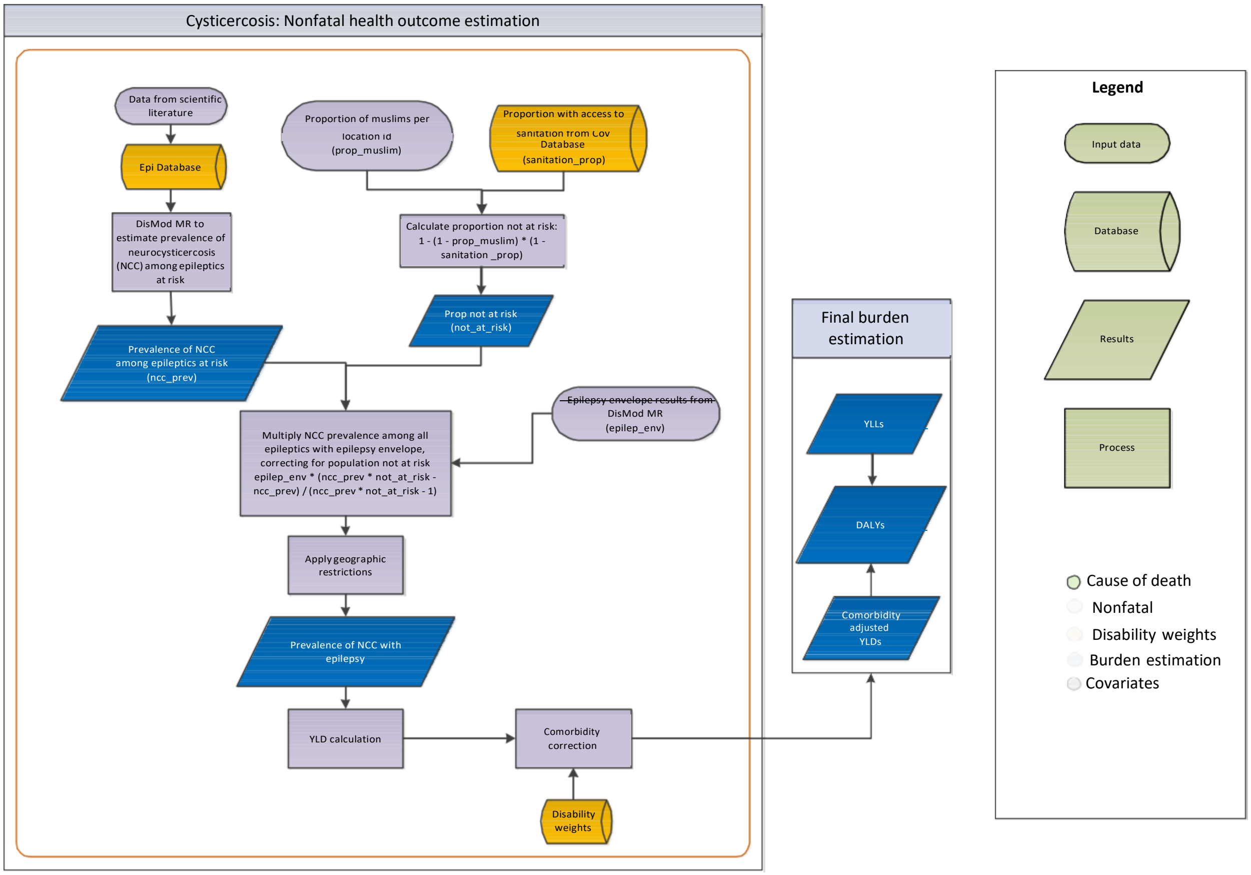

Input data and methodological summary

Case definition

Cysticercosis is a parasitic disease caused by the pig tapeworm Taenia solium, transmitted via ingestion of contaminated food or water. Parasite development to larvae in the human central nervous system can cause neurologic symptoms including epilepsy. Diagnosis is made by magnetic resonance imaging (MRI) or computerised tomography (CT) brain scans to identify cysts. The ICD-10 codes for cysticercosis are B69-B69.9.

Cysticercosis

Quantity of interest	Reference or alternative	Definition
Cysticercosis	Reference	An epilepsy patient with either (a) T.Solium identified in excised cyticersi from tissues by microscopic examination or (b) identification of cysticerci by CT scan, MRI or X-ray and positive result on CDC immunoblot assay.

Cysticercosis	Alternative	An epilepsy patient with calcified cystic lesions in the brain identified by CT scan, MRI, or X-ray; or positive result on CDC immunoblot assay. [A "probable" case.]

Input data

Systematic literature review

The non-fatal estimation for cysticercosis focused on estimating prevalence of NCC among epileptics at risk as well as the prevalence of NCC with epilepsy. A systematic review of literature was conducted in PubMed for GBD 2015 using the following search string:

> ("cysticercosis"[Title/Abstract] OR "neurocysticercosis"[Title/Abstract] OR "cysticerciasis"[Title/Abstract] OR "Taenia solium"[Title/Abstract]) AND (“1990”[Date – Publication] : “2015”[Date – Publication]) AND (epidemiology OR prevalence)).

This yielded 1,038 studies, of which 166 were included during the title/abstract screening. Following the full-text screening, 17 studies were included and extracted – studies were excluded because of one or more of the following reasons:

10. study not in epileptics
11. study not population-based
12. study does not have primary data on prevalence of NCC among epileptics at risk
13. study not in humans (some studies were on cysticercosis in pigs)
14. study on comorbidities with NCC (other than epilepsy)
15. study on sub-population, (eg, patients with neurological disorders)
16. review study

Table 1 presents a summary of source counts for this model.

Table 1. Total data source counts

Measure	Total sources	Countries with data
All measures	30	16
Prevalence	30	16

Data processing

Input data were classified as either probable or definite diagnosis. We extracted 16 within-study comparisons to crosswalk the data using definite diagnosis as a reference using MR-BRT (meta-regression—Bayesian, regularised, trimmed) (Table 2).

Table 2. MR-BRT crosswalk adjustment factors

Data input	Reference or alternative case definition	Gamma	Beta Coefficient, Log (95% CI)*	Adjustment factor**

Definite	Ref	0.62	---	---
Probable	Alt		0.59 (0.22, 0.96)	0.55

**MR-BRT crosswalk adjustments can be interpreted as the factor the alternative case definition is adjusted by to reflect what it would have been had it been measured using the reference case definition. If the log/logit beta coefficient is negative, then the alternative is adjusted up to the reference. If the log/logit beta coefficient is positive, then the alternative is adjusted down to the reference.*

***The adjustment factor column is the exponentiated beta coefficient. For log beta coefficients, this is the relative rate between the two case definitions. For logit beta coefficients, this is the relative odds between the two case definitions.*

Covariates

Data were ascertained from the PEW Research Center [1] on the proportion of the population that is Muslim and incorporated as a continuous covariate with a range between 0 and 1.

Epilepsy envelope

The modelling process incorporates 1,000 draws of epilepsy envelope prevalence from the GBD 2021 epilepsy DisMod-MR model – details on this modelling process can be found elsewhere.

Modelling strategy

DisMod-MR was used to model the prevalence of NCC among epileptics at risk. In the model, pigs raised in extensive agricultural systems per capita, SDI, and religion (binary, >50% Muslim) were used as country-level covariates (Table 3).

Table 3. DisMod-MR model covariates

Covariate	Type	Parameter	Exponentiated beta
Religion (binary, > 50% Muslim)	Country-level	Prevalence	0.22 (0.16, 0.33)
Socio-demographic Index	Country-level	Prevalence	0.14 (0.14, 0.16)
Pigs raised in extensive agricultural systems per capita	Country-level	Prevalence	3.07 (1.34, 6.38)

After running DisMod-MR, we adjusted the fraction of people with epilepsy attributable to cysticercosis in endemic countries for the population at risk based on the proportion of the population without access to sanitation and the proportion of the population that is Muslim. The following is the computation for estimating NCC prevalence among epileptics at risk:

$$Prevalence_{NCC\ prevalence} = Prevalence_{epilepsy} * \frac{NM - N}{NM - 1}$$

Where prevalence = prevalence of all-cause epilepsy in total population, N = proportion of NCC among epileptics at risk (non-Muslims without access to sanitation), and M = proportion of population not at risk of contracting NCC. It was assumed that the prevalence of epilepsy due to causes other than NCC is

the same regardless of whether a population is at risk or not. It was also assumed that Muslims and non-Muslims have equal access to sanitation. Geographic restrictions were applied to set prevalence to zero in non-endemic locations.

Model evaluation was done by separately assessing the fit of the DisMod-MR model and checking the estimates produced after estimating prevalence of NCC with epilepsy. Plots of time trends of prevalence across locations and age were used to evaluate the results. In addition, maps of the global distribution of prevalence of NCC among epileptics at risk and prevalence of NCC with epilepsy were also assessed across time.

Changes from GBD 2019 to GBD 2021

We have made no substantive changes in the modelling strategy from GBD 2019.

We did not apply any adjustments for the COVID pandemic to cysticercosis due to a lack of available data quantifying the impacts of the pandemic on NTD epidemiology.

References:

1. "Table: Muslim Population by Country Pew Research Center, Washington, D.C." (July 7, 2017). http://www.pewforum.org/2011/01/27/table-muslim-population-by-country/

Decubitus ulcer

Flowchart for decubitus ulcer

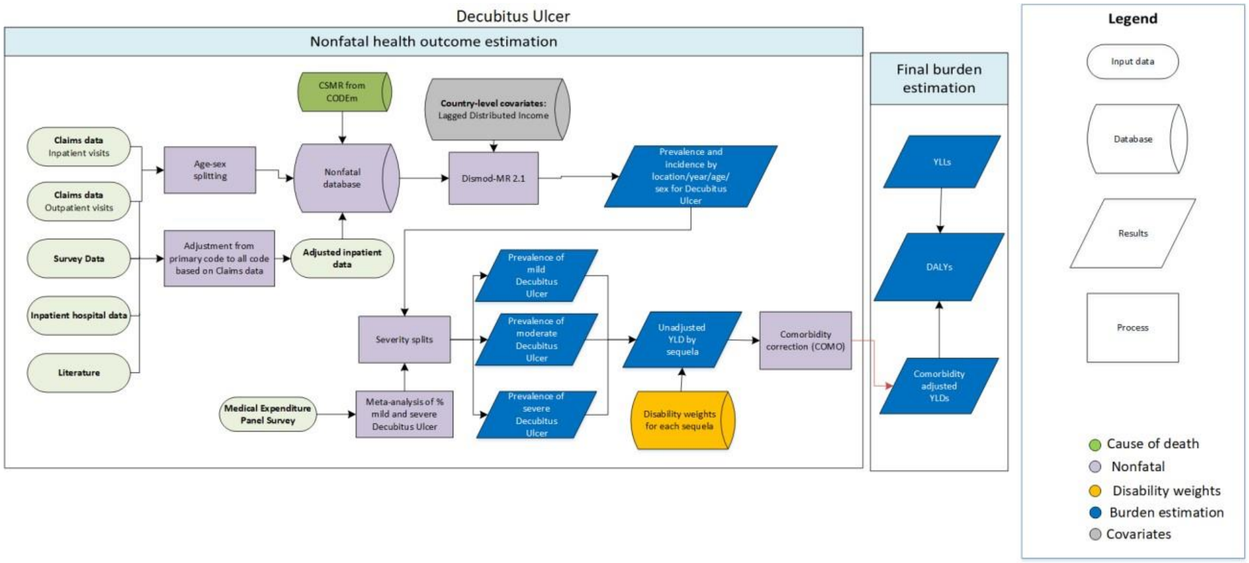

Input data and methodological summary for decubitus ulcer

Case definition

Decubitus ulcer was included in the GBD 2021 cause group of skin and subcutaneous conditions. Decubitus ulcer is defined as an injury to the skin and underlying tissue resulting from an obstruction of blood flow due to pressure on the skin. Also known as pressure ulcer/sore (ICD-10: L89).

**Quantity of interest**	**Reference or Alternative**	**Definition**
Decubitus ulcer	Reference	Decubitus ulcer as determined by clinical diagnosis and claims data since 2010.
Decubitus ulcer	Alternative	Decubitus ulcer as indicated by hospital admission and claims data before 2000.

Input data

In the GBD 2010 study, a systematic review of the literature was conducted using PubMed and Google Scholar to capture epidemiological data for decubitus ulcer. The inclusion criteria stipulated that studies (1) must be published between 1980 and 2012; (2) must provide data on the incidence or prevalence of decubitus ulcer; (3) must use samples representative of the general population (ie, samples derived from the experimental arm of clinical trials or based in dermatology clinics were excluded); (4) must use a sample size larger than 100; and (5) must provide sufficient information on study method and sample characteristics to assess the quality of the study. The data from literature were sparse but contained both prevalence and incidence estimates. For GBD 2013, the GBD 2010 search strategy was replicated to capture epidemiological studies published between 2012 and 2013. The available data were from high-income countries. Hospital inpatient, USA claims data from 2000 and 2010–2016, Taiwan (province of China) claims data for 2016, and Poland claims data for 2015–2017 were also used for GBD 2020. The final dataset also included cause-specific mortality rates for decubitus ulcer estimated by CODEm. Data were outliered or excluded if we found them unreasonable when compared to regional, super-regional, and global rates.

Table 1: Data inputs for decubitus ulcer morbidity modelling by parameter

**Cause/impairment name**	**Measure**	**Countries with data**	**New sources**	**Total sources**
Decubitus ulcer	All measures	47	35	345
Decubitus ulcer	Incidence	47	35	330
Decubitus ulcer	Proportion	1	0	15

Table 2: MR-BRT crosswalk adjustment factors for decubitus ulcer

Data input	Reference or alternative case definition	Gamma	Beta coefficient, logit* (95% CI)	Adjustment factor**
Literature with physical exam	Reference	0.10	---	---
USA MarketScan 2000	Alternative		–0.29 (–0.96 to –0.38)	0.43

Inpatient data	Alternative		–0.22 (–0.87 to 0.42)	0.44

**MR-BRT crosswalk adjustments can be interpreted as the factor the alternative case definition is adjusted by to reflect what it would have been had it been measured using the reference case definition. If the log/logit beta coefficient is negative, then the alternative is adjusted up to the reference. If the log/logit beta coefficient is positive, then the alternative is adjusted down to the reference.*
***The adjustment factor column is the exponentiated beta coefficient. For log beta coefficients, this is the relative rate between the two case definitions. For logit beta coefficients, this is the relative odds between the two case definitions.*

Modelling strategy

DisMod-MR 2.1 was used to estimate prevalence by age, sex, year, and geography (subnational, country, region, super-region) for decubitus ulcer. Per expert advice, remission was set from 3 to 4, implying a duration of three to four months. This was based on the assumption that remission does not change with treatment. These values were also in line with the available epidemiological data, expert opinion, and previous GBD work. The decubitus ulcer dataset was sufficiently large to make use of a relatively short time window of five years to determine which datapoints were used for a particular year of fit.

In GBD 2021, we replaced our within-DisMod crosswalks with crosswalks completed using the MR-BRT modeling tool. We adjusted inpatient data, along with USA MarketScan data 2000 and inpatient data toward the level of other incidence datapoints which were more representative of the general population. In addition, log-transformed lagged distributed income (LDI) was used as a country-level covariate to guide estimates for locations with few or no data. LDI was restricted to a range of –0.5 to –0.1. We restricted location random effects to (–0.5, 0.5) across all seven GBD super-regions.

In previous rounds, priors on excess mortality rate (EMR) were estimated in DisMod by matching prevalence datapoints with their corresponding CSMR values within the same age, sex, year, location (by dividing CSMR by prevalence). For short duration conditions (remission >1), the corresponding prevalence was derived by running an initial model and then applying the same CSMR/prevalence method. However, for many causes, DisMod estimated a rather unrealistic pattern of EMR compared to an expected pattern of decreasing EMR with greater access to quality health care. Such unexpected patterns often signal inconsistencies between CSMR estimates and the measures of prevalence and/or incidence. In effort to provide greater guidance to DisMod on the expected pattern of EMR, EMR data generated in the previous round were modelled using the MR-BRT approach by age and sex with a prior on Healthcare Access and Quality (HAQ) Index having a negative coefficient. Results from MR-BRT were then predicted for each location year, sex and for ages 0, 10, 20 ….100.

We have made no substantive changes in the modelling strategy from GBD 2019.

Table 3. Severity distribution, details on the severity levels for decubitus ulcer and the associated disability weight (DW) with that severity

Sequela	Severity level	Lay description	DW (95% CI)

Mild decubitus ulcer	Disfigurement, level 1 with itch/pain	The person has a slight, visible physical deformity that is sometimes sore or itchy. Others notice the deformity, which causes some worry and discomfort.	0.027 (0.015–0.042)
Moderate decubitus ulcer	Disfigurement, level 2, with itch/pain	The person has a visible physical deformity that is sore and itchy. Other people stare and comment, which causes the person to worry. The person has trouble sleeping and concentrating.	0.188 (0.124–0.267)
Severe decubitus ulcer	Disfigurement, level 3, with itch/pain	The person has an obvious physical deformity that is very painful and itchy. The physical deformity makes others uncomfortable, which causes the person to avoid social contact, feel worried, sleep poorly, and think about suicide.	0.576 (0.401–0.731)

Table 4. Covariates. Summary of covariates used in the decubitus ulcer DisMod-MR meta-regression model

Covariate	Type	Parameter	Exponentiated beta (95% uncertainty interval)
LDI (I$ per capita)	Country-level	Excess mortality rate	0.90 (0.90–0.90)

Dengue

Flowchart

Dengue – GBD 2021

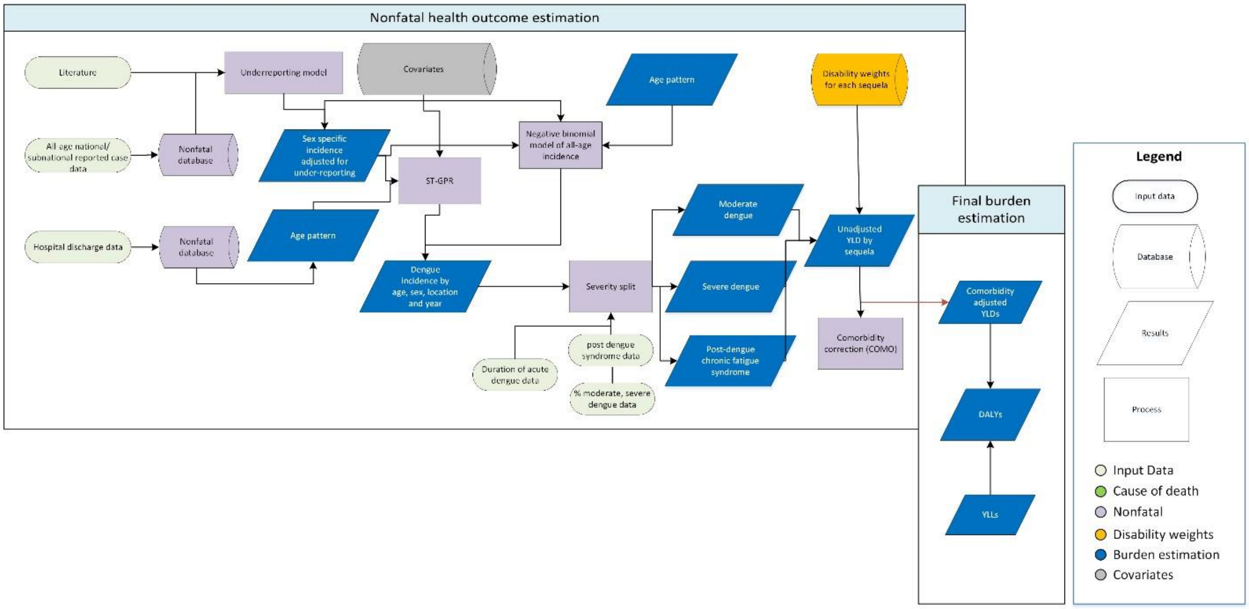

Case definition

Dengue is mosquito-borne viral infection that causes febrile illness and, in severe cases, jaundice, haemorrhage, and death. It includes all ICD-10 codes under the heading A90 (Dengue fever [classical dengue]) and A91 (Dengue haemorrhagic fever).

Dengue

**Quantity of interest**	**Reference or alternative**	**Definition**
Dengue	Reference	Case of dengue confirmed by any of: virus isolation in cell culture; detection of viral nucleic acid by PCR; NS1 antigen detection by ELISA or rapid test; serological detection of IgM or IgG antibodies by ELISA or rapid test or haemagglutination inhibition. (Based on WHO definition.)
Dengue	Reference	Cases of dengue notified to public health agencies.
Dengue	Reference	WHO definition of a probable case of dengue through clinical diagnosis based on combination of (a) residency in or travel to dengue-endemic area and (b) fever and (c) two of the following criteria: nausea or vomiting; rash; aches and pains; tourniquet test positive; leukopenia; any warning sign requiring strict observation and medical intervention, including abdominal pain or tenderness, persistent vomiting, fluid accumulation, mucosal bleed, lethargy or restlessness, liver enlargment >2 cm; increase in HCT concurrent with rapid decrease in platelet count. (Based on WHO definition.)

Input data

Model inputs

For GBD 2021, we modelled dengue incidence based on reported cases. We last updated these data sources for GBD 2019, during which time data-seeking targeted specific geographies (India, Indonesia, Pakistan, Brazil, and China) for subnational case details, along with years updates for years 2016–2018. Age-specific data were collated separately to enable disaggregation of all-age and both-sex case data into age and sex-specific inputs prior to modelling. A systematic literature review was conducted to identify studies that compared incidence of dengue among passive and active case detection systems to estimate a correction factor to adjust for under-reporting. Scientific literature sources were used for assumptions related to severity.

Table 1 presents the total number of data sources used in the non-fatal estimation.

Table 1. Total data source counts

Measure	Total sources	Number of countries
All measures	1879	131
Incidence	1775	131
Duration	2	0
Proportion	367	43
Continuous	17	15

Data processing

Correction for under-reporting

Since dengue disease is often under-reported due to health system capacity or misdiagnosed as other febrile illnesses, we conducted a systematic literature review to identify sources that compared incidence rates reported via active versus passive surveillance.

We searched PubMed for dengue underreporting with the following search terms (without date restrictions) on 24 May 2019:

> (("active"[Title/Abstract] AND "passive"[Title/Abstract]) OR "case detection"[Text Word] OR "under reporting"[Text Word] OR "coverage"[Text Word]) AND dengue[MeSH Terms]

The search returned 143 results (see Figure 2), published between 1982 and 2019. We added four sources previously extracted, and 46 more discovered by other means (generally from reference lists of meta-analyses or other sources with composite results). In screening titles and abstracts, we excluded 111 sources. The remaining 80 were subject to full-text screening for extraction. Of these, 64 were excluded as not meeting extraction criteria; 17 sources were extracted. In our final analysis we included a total of 52 comparisons, from 7 sources using enhanced surveillance compared to passive surveillance, to generate an adjustment factor to correct for under-reporting. The under-reporting adjustment factors were estimated using MR-BRT (meta-regression—Bayesian, regularised, trimmed) and included Socio-demographic Index (SDI) and reported incidence rate, trimming 10% of the input data. The uncertainty from the MR-BRT meta-regression was applied to the adjustment. Table 2 presents the correction factors for under-reporting.

Table 2: MR-BRT crosswalk adjustment factors for under-reporting due to dengue

Data input	Gamma	Beta Coefficient*, Log (95% CI)	Adjustment factor**
Intercept	0.89	-0.43 (-2.43; 1.63)	0.65
Population Density (over 1000 ppl/sqkm, proportion)		-3.86 (-6.10; -1.62)	0.02

**MR-BRT crosswalk adjustments can be interpreted as the factor the alternative case definition is adjusted by to reflect what it would have been had it been measured using the reference case definition. If the log/logit beta coefficient is negative, then the alternative is adjusted up to the reference. If the log/logit beta coefficient is positive, then the alternative is adjusted down to the reference.*

***The adjustment factor column is the exponentiated beta coefficient. For log beta coefficients, this is the relative rate between the two case definitions. For logit beta coefficients, this is the relative odds between the two case definitions.*

Dengue incidence data reported for both sexes was first split into sex-specific inputs. To sex-split our both-sex datapoints, we used sex-specific inputs in a Bayesian meta-regression (MR-BRT) model to derive a ratio of female dengue incidence to both-sex incidence (sci-lit data). The resultant log ratio was applied to both-sex datapoints to calculate out females, and males were calculated via subtraction. The beta coefficients of the adjustment are presented in Table 3.

Table 3. Ratio of males: females estimated using MR-BRT

Data input	Reference or alternative case definition	Gamma	Beta Coefficient, Log (variance)	Adjustment factor
Intercept	Females (ref)	0.14	0.0121 (0.0018)	1.012195

**MR-BRT crosswalk adjustments can be interpreted as the factor the alternative case definition is adjusted by to reflect what it would have been had it been measured using the reference case definition. If the log/logit beta coefficient is negative, then the alternative is adjusted up to the reference. If the log/logit beta coefficient is positive, then the alternative is adjusted down to the reference.*

***The adjustment factor column is the exponentiated beta coefficient. For log beta coefficients, this is the relative rate between the two case definitions. For logit beta coefficients, this is the relative odds between the two case definitions.*

Modelling strategy

Once the data were adjusted for under-reporting and sex-split, a hybrid approach was used to generate incidence estimates using two models: (1) a spaciotemporal Gaussian process regression (ST-GPR) and a (2) negative binomial regression using fixed effects to model all-incidence. These two models were hybridised (500 draws from each approach were combined to generate 1,000 draws of incidence).

ST-GPR

We ran an all ages ST-GPR model for incidence including the settings listed in Table 4. The covariates used were the population-weighted probability of dengue infection (based on data from Messina et al 2019[1]), GBD-location level cause-specific mortality rate (CSMR), population density, proportion of the population living between 0 and 15 absolute degrees latitude, and Healthcare Access and Quality (HAQ) Index. ST-GPR was used to model incidence, excluding inputs for which zero cases were reported (under the assumption that in dengue-endemic settings zero reported cases would be implausible).

Table 4. ST-GPR Model settings

Parameter	Value
Lambda	0.5
Omega	1
Zeta	.01
Scale	1
Amplitude	1

Initial model testing showed that inclusion of data from the 2009 Cabo Verde dengue outbreak resulted in implausibly high values for West African locations, largely due to the limited number of data inputs for this modelling region (34 total inputs). Data from south Asia and Cabo Verde were additionally excluded – the values in the underlying data suggested substantial under-reporting and, in initial testing, led the model to predict no cases. The all-age estimates were then disaggregated by age using an overall age pattern derived from the age-specific hospital discharge data inputs. This age pattern was modelled using a negative binomial regression with cubic spline variables for age group.

Negative binomial regression

A negative binomial regression was implemented with the CSMR and population-weighted probability of dengue transmission[1] as predictors to model total incidence of dengue disease. Input data were adjusted for under-reporting using the MR-BRT method described above. The fixed and random effects from this model were used to generate estimates of all-age, both-sex incidence for all locations except south Asia. South Asia locations were estimated off the fixed effects only, because of the limited data from this region generated implausibly low random effects. Then all south Asia locations, except Bangladesh (where we have more reliable data), were inflated by random draws from the under-reporting correction. After this, the all ages and both sexes incidence estimates were disaggregated by age and sex using an overall age pattern derived from the same age-specific hospital discharge data inputs. This age pattern was modelled using a negative binomial regression with cubic spline variables for age group.

Severity splits and disability weights

The resulting incidence estimates were then split into moderate (94.5%) and severe (5.5%) sequelae, based on the proportion of reported cases that were severe using data from WHO and PAHO reports and scientific literature. Prevalence of moderate dengue was calculated assuming a duration of 6 days and prevalence of severe dengue estimated using an assumption of duration of 14 days based on Whitehead et al 2007[2]. We assume that 8.4% of symptomatic infections will produce post-acute chronic fatigue lasting an average of six months based on Teixeira et al 2010[3]. (.). Disability weights are presented in Table 5.

Table 5. Severity distribution.

Severity level	Lay description	DW (95% CI)
Moderate	Has a fever and aches, and feels weak, which causes some difficulty with daily activities.	0.051 (0.032, 0.074)
Severe	Has a high fever and pain, and feels very weak, which causes great difficulty with daily activities.	0.133 (0.088-0.19)
Post-dengue chronic fatigue syndrome	Is always tired and easily upset. The person feels pain all over the body and is depressed.	0.219 (0.148-0.308)

Figure 2. PRISMA chart for systematic review for under-reporting of dengue

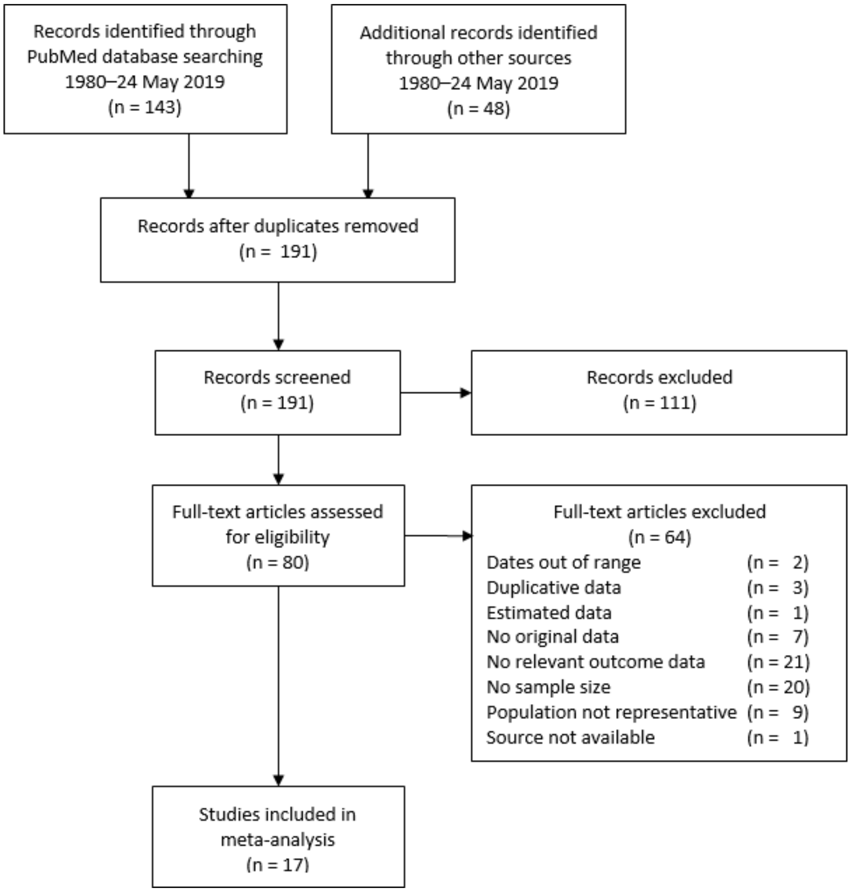

Changes from GBD 2019 to GBD 2021

The major change from GBD2019 is that we used all-age input data for running the ST-GPR model, and then we then age-split using a single age pattern. Last cycle we age-split the input data before using the ST-GPR data.

We did not apply any adjustments for the COVID-19 pandemic to dengue due to a lack of available data quantifying the impacts of the pandemic on NTD epidemiology.

References

1 - Messina JP, Brady OJ, Golding N, Kraemer MU, Wint GW, Ray SE, Pigott DM, Shearer FM, Johnson K, Earl L, Marczak LB. The current and future global distribution and population at risk of dengue. Nature microbiology. 2019 Sep;4(9):1508-15.

2 - Whitehead SS, Blaney JE, Durbin AP, Murphy BR. Prospects for a dengue virus vaccine. Nature Reviews Microbiology. 2007 Jul;5(7):518-28.

3 - Teixeira L de AS, Lopes JSM, Martins AG da C, Campos FAB, Miranzi S de SC, Nascentes GAN. Persistence of dengue symptoms in patients in Uberaba, Minas Gerais State, Brazil. *Cad Saúde Pública* 2010; **26**: 624–30

Dermatitis

Flowchart for atopic dermatitis, contact dermatitis, & seborrhoeic dermatitis

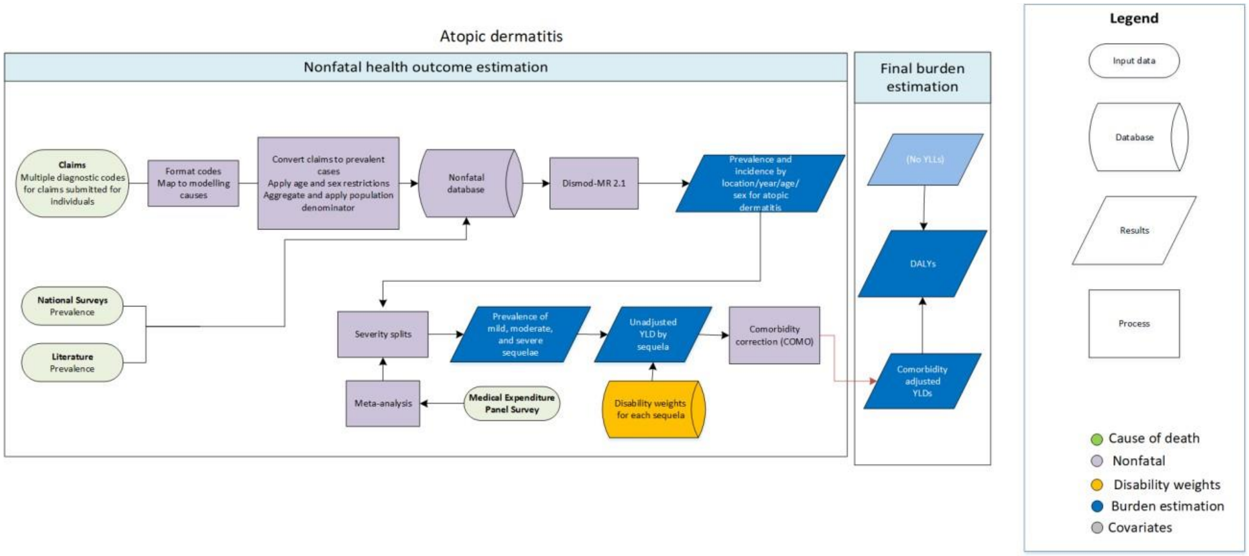

Contact dermatitis

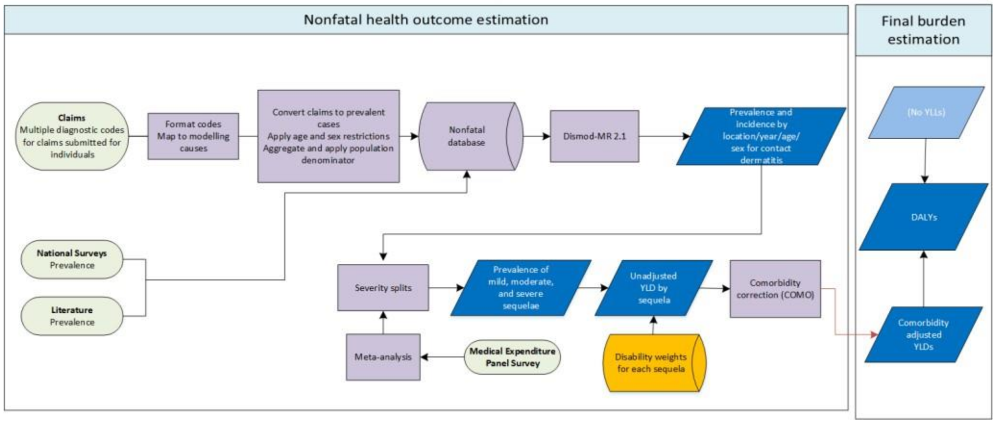

Seborrhoeic dermatitis

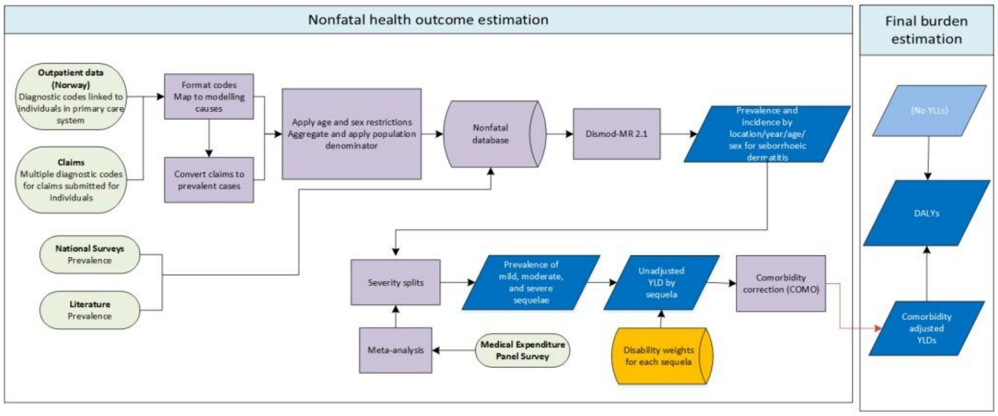

Input data and methodological summary for dermatitis

Case definition

Dermatitis, or eczema, refers to inflammation of the dermal layer of the skin, with disruption of the epidermal barrier. This inflammation leads to rashes that are commonly red, scaly, or flaky.

Atopic dermatitis is defined as relapsing inflammation of the dermal layer of the skin with disruption of the epidermal barrier (dermatitis). Associated with elevated serum immunoglobulin E and some degree of immune dysregulation, it can be localised or widespread (ICD-10: L20).

Contact dermatitis is defined as localised inflammation of the dermal layer of skin with disruption of the epidermal barrier (dermatitis) caused by direct contact with allergens or irritants (ICD: 10: L22-26).

Seborrhoeic dermatitis is defined as inflammation of the dermal layer of the skin with disruption of the epidermal barrier (dermatitis) affecting the sebaceous-gland-rich areas of skin (ICD-10: L21).

Quantity of interest	Reference or Alternative	Definition
Atopic dermatitis	Reference	Atopic dermatitis determined by a physical examination.
Atopic dermatitis	Alternative	Atopic dermatitis that is self-reported or determined without a physical exam.

Contact dermatitis	Reference	Contact dermatitis determined by a physical examination and USA MarketScan data from 2010–2014.
Contact dermatitis	Alternative	Contact dermatitis self-reported, recorded in claims before 2010, or identified in the Medical Expenditure Panel Survey (MEPS).
Seborrhoeic dermatitis	Reference	Seborrhoeic dermatitis determined by a physical examination and USA MarketScan data from 2010–2014.
Seborrhoeic dermatitis	Alternative	Seborrhoeic dermatitis recorded in ICPC, MEPS, or USA MarketScan data before 2010.

Input data

Data for dermatitis came from scientific literature and claims submitted for individuals to USA commercial insurance. The atopic and contact dermatitis model additionally incorporated data from a claims database in Russia and the atopic dermatitis model incorporated claims data from Poland. A literature review was conducted in GBD 2016 for studies of the incidence and prevalence of dermatitis, the details of which are described in the appendix to GBD 2016, and the results of this review were used in GBD 2020. Inpatient data were regarded as inappropriate for this chronic, non-fatal condition that is primarily cared for in non-acute settings. Data from the Medical Expenditure Panel Survey (MEPS) in the USA in 2000–2009 (2) were included to inform the age pattern of the prevalence output. Data from the NHANES study and the NHIS study (both from the USA) were not extracted, as questions regarding dermatitis were too broad (ie, asked whether a respondent had experienced eczema or any other rash). The data for dermatitis were expanded based on recommendations of research articles and reviews by the skin expert group.

Data from outpatient encounters in the USA and Sweden were considered for inclusion but were found to violate established age patterns and regional trends and were excluded. Additional data were marked as outliers and excluded if we found them unreasonable when compared to regional, super-regional, and global rates. See descriptions of individual modelling approaches for more information.

Table 1: Data inputs for dermatitis, atopic dermatitis, contact dermatitis, and seborrhoeic dermatitis morbidity modelling by parameter

**Cause/Impairment Name**	**Measure**	**Countries with data**	**New Sources**	**Total sources**
Dermatitis	All measures	114	3	344
Dermatitis	Prevalence	114	3	341
Dermatitis	Incidence	2	0	2
Dermatitis	Proportion	1	0	15
Atopic dermatitis	All measures	113	3	316
Atopic dermatitis	Prevalence	113	3	316
Atopic dermatitis	Incidence	1	0	1
Contact dermatitis	All measures	17	3	69
Contact dermatitis	Prevalence	17	3	66
Contact dermatitis	Incidence	1	0	1
Contact dermatitis	Proportion	1	0	15

Seborrhoeic dermatitis	All measures	23	3	73
Seborrhoeic dermatitis	Prevalence	23	3	70
Seborrhoeic dermatitis	Incidence	1	0	1
Seborrhoeic dermatitis	Proportion	1	0	15

Table 2: MR-BRT crosswalk adjustment factors for dermatitis, atopic dermatitis, contact dermatitis, and seborrhoeic dermatitis

Cause	Data input	Reference or alternative case definition	Gamma	Beta coefficient, logit (95% CI)*	Adjustment factor**
Atopic dermatitis	Literature with physical exam and Poland claims	Reference	1.03	---	---
	Administrative data	Alternative		–1.04 (–3.07 to 0.98)	0.26
	Medical Expenditure Panel Survey (MEPS)	Alternative		–0.56 (–2.59 to 1.47)	0.36
	No physical exam	Alternative		0.25 (–1.78 to 2.28)	0.56
	USA MarketScan 2000	Alternative		–1.78 (–3.81 to 0.25)	0.14
Contact Dermatitis	Literature with physical exam and USA MarketScan 2010–2014	Reference	0.29	--	--
	Self-report	Alternative		0.40 (–0.19 to 1.00)	0.60
	MEPS	Alternative		–0.72 (–1.30 to –0.14)	0.33
	Recall 1 year	Alternative		0.40 (–0.19 to 1.00)	0.60
	USA MarketScan 2000	Alternative		–0.14 (–0.71 to 0.44)	0.47
Seborrhoeic dermatitis	Literature with physical exam and USA MarketScan 2010-2014	Reference	0.30	--	--
	ICPC	Alternative		–2.97 (–3.60 to –2.35)	0.05
	MEPS	Alternative		–2.69 (–3.29 to –2.10)	0.06
	USA MarketScan 2000	Alternative		–0.56 (–1.16 to 0.04)	0.36

**MR-BRT crosswalk adjustments can be interpreted as the factor the alternative case definition is adjusted by to reflect what it would have been had it been measured using the reference case definition. If the log/logit beta coefficient is negative, then the alternative is adjusted up to the reference. If the log/logit beta coefficient is positive, then the alternative is adjusted down to the reference.*
***The adjustment factor column is the exponentiated beta coefficient. For log beta coefficients, this is the relative rate between the two case definitions. For logit beta coefficients, this is the relative odds between the two case definitions.*

Modelling strategy

DisMod-MR 2.1 was used to estimate prevalence by age, sex, year, and geography (subnational, country, region, super-region) for atopic dermatitis, contact dermatitis, and seborrhoeic dermatitis. Separate models were run for each cause. We have made no substantive changes in the modelling strategy from GBD 2019.

Model parameters

Atopic dermatitis

Since our available data mostly contained information on prevalence, we specified additional expert priors to further inform analyses. The prior value on excess mortality was set to zero, and the prior value on remission was bounded to 0–0.2 (equivalent to five years to life time duration). Since GBD 2019, we replaced our within-DisMod crosswalks with crosswalks completed using the MR-BRT modelling tool. We adjusted administrative data, along with data from the Medical Expenditure Panel Survey, USA MarketScan 2000 data, and data that were not based on physical exams toward the level of other datapoints which were more representative of the general population. To improve regional and global estimates, the minimum coefficient of variation was set at 0.4 and location random effects for Paraguay, Sweden, and England were restricted to [–0.25, 0.25], [–0.25, 0.25], and [–0.5, 0.5], respectively. A time window of ten years was used to determine which datapoints were used for a particular year of fit.

Contact dermatitis

Similar to atopic dermatitis, mostly prevalence data were available for contact dermatitis. Per expert advice, the remission parameter was set from 0.1 to 4, excess mortality was set to zero, and incidence was set to zero prior to age 6. Since GBD 2019, we replaced our within-DisMod crosswalks with crosswalks completed using the MR-BRT modelling tool. We adjusted data with a recall period of 1 year, along with data from the Medical Expenditure Panel Survey, USA MarketScan 2000 data, and data that were not based on physical exams toward the level of other datapoints which were more representative of the general population. In order to improve model estimates, location random effects were added for all super-regions [–0.25, 0.25]. A time window of 25 years was used to determine which datapoints were used for a particular year of fit.

Seborrhoeic dermatitis:

As with contact dermatitis, the available data were mostly prevalence estimates. Per expert advice, settings were placed on incidence as follows: 0–4 years = 0–0.1, and 60–100 = 0–0.01. Excess mortality was set to zero while a setting of 0.1–12 was placed on remission, implying a duration of one month to ten years. Since GBD 2019, we replaced our within-DisMod crosswalks with crosswalks completed using

the MR-BRT modelling tool. We adjusted RA diagnosis from administrative data, along with data from the Medical Expenditure Panel Survey (MEPS), USA MarketScan 2000 data, and Norway outpatient data (ICPC) toward the level of other datapoints which were more representative of the general population. To improve the model estimate, a location random effect was added to the central Europe, eastern Europe, and central Asia super-region [–1, 1] in order to prioritise the Poland data. A time window of 25 years was used to determine which datapoints were used for a particular year of it.

Table 3. Severity distribution, details on the severity levels for atopic dermatitis, contact dermatitis, and seborrhoeic dermatitis and the associated disability weight (DW) with that severity.

Sequela	Severity level	Lay description	DW (95% CI)
Mild atopic dermatitis	Disfigurement, level 1 with itch/pain	The person has a slight, visible physical deformity that is sometimes sore or itchy. Others notice the deformity, which causes some worry and discomfort.	0.027 (0.015–0.042)
Moderate atopic dermatitis	Disfigurement, level 2, with itch/pain	The person has a visible physical deformity that is sore and itchy. Other people stare and comment, which causes the person to worry. The person has trouble sleeping and concentrating.	0.188 (0.124–0.267)
Severe atopic dermatitis	Disfigurement, level 3, with itch/pain	The person has an obvious physical deformity that is very painful and itchy. The physical deformity makes others uncomfortable, which causes the person to avoid social contact, feel worried, sleep poorly, and think about suicide.	0.576 (0.401–0.731)
Mild contact dermatitis	Disfigurement, level 1 with itch/pain	The person has a slight, visible physical deformity that is sometimes sore or itchy. Others notice the deformity, which causes some worry and discomfort.	0.027 (0.015–0.042)

Moderate contact dermatitis	Disfigurement, level 2, with itch/pain	The person has a visible physical deformity that is sore and itchy. Other people stare and comment, which causes the person to worry. The person has trouble sleeping and concentrating.	0.188 (0.124–0.267)
Symptomatic seborrhoeic dermatitis	Disfigurement, level 1 with itch/pain	The person has a slight, visible physical deformity that is sometimes sore or itchy. Others notice the deformity, which causes some worry and discomfort.	0.027 (0.015–0.042)

Developmental intellectual disability

Flowchart

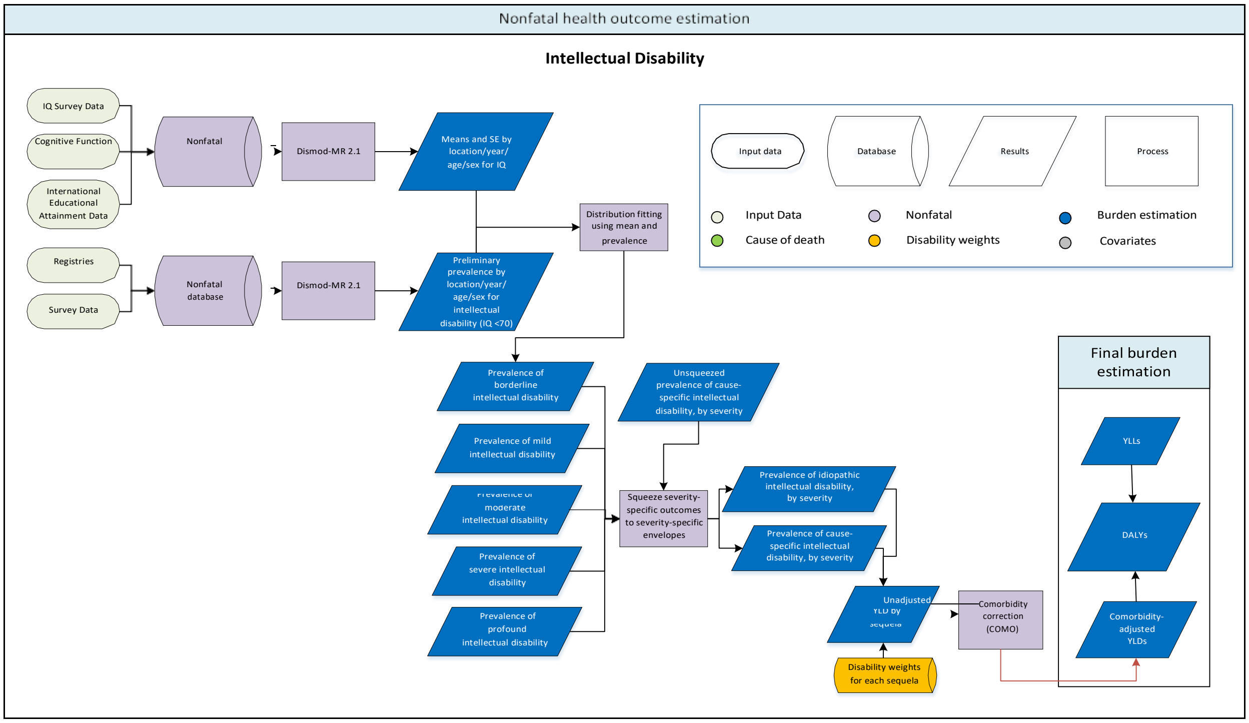

Case definition

Developmental intellectual disability (ID) is a condition characterised by significant limitations in both intellectual functioning and adaptive behavior. . Consistent with the American Association on Intellectual and Developmental Disabilities, we define developmental intellectual disability as a condition originating before age 18 (as such, it does not include impairment due to stroke, Alzheimer's disease, or other conditions that affect older populations). We model the severities shown in Table 1, as measured by score on intelligence quotient (IQ) tests, which are standardised to have a mean of 100. Commonly used IQ tests include: Wechsler Preschool and Primary Scale of Intelligence (WIPPSI), Wechsler Intelligence Scale for Children (WISC), and Wechsler Adult Intelligence Scale (WAIS).

Table 1. ID severity definitions

Severity of intellectual disability	IQ score
Profound	0 to 19
Severe	20 to 34
Moderate	35 to 49
Mild	50 to 69
Borderline	70 to 85

Input data

Model inputs

The prevalence of intellectual disability (IQ score <70) is estimated from a systematic review of publications since January 1, 1990, using the following search string: *(((intellectual disability[MeSH Terms]) AND prevalence[Title/Abstract]) AND ('1990'[Date - Publication] : '3000'[Date - Publication]))*. We included studies that estimated the general population prevalence of intellectual disability. We excluded studies that did not use a case definition based on intelligence quotient (IQ) and studies that investigated non-representative groups, such as hospital patients or people of a specific ethnicity. This systematic review was last updated for GBD 2016. Table 2 shows a summary of the input data used.

Table 2. Input data

Measure	Total sources	Countries with data
All measures	58	31
Prevalence	58	31

Data processing

In GBD 2021, we used MR-BRT to split our both-sex data points into sex-specific data. Table 3 has the model coefficient used in sex-splitting.

Table 3. MR-BRT coefficient values (raw and exponentiated)

Sex-split coefficient (95% CI)	Exponentiated sex-split coefficient (95% CI)
-0.27 (-0.78 to 0.24)	0.77 (0.46 to 1.27)

Because we code males as “1” and females as “2”, this coefficient means that the observed prevalence of ID is slightly higher in males than in females (ie, prevalence in females is 0.77 times prevalence in males). To split our both-sex data, we first used the coefficient to get a population-weighted adjustment factor. We then multiplied that adjustment factor by the both-sex data points to get expected prevalence in males, and finally multiplied the coefficient by the expected male prevalence to get expected prevalence in females. In our final modelling dataset, we exclusively used the sex-specific and sex-split data (ie, no both-sex data were included in the model).

Severity splits – disability weights

Table 4. Intellectual disability severity disability weights

Health state	Description	Disability weight
Borderline intellectual functioning	This person is slow in learning at school. As an adult, the person has some difficulty doing complex or unfamiliar tasks but otherwise functions independently.	0.011 (0.005–0.02)
Intellectual disability/mental retardation, mild	This person has low intelligence and is slow in learning at school. As an adult, the person can live independently, but often needs help to raise children and can only work at simple supervised jobs.	0.043 (0.026–0.064)
Intellectual disability/mental retardation, moderate	This person has low intelligence, and is slow in learning to speak and to do even simple tasks. As an adult, the person requires a lot of support to live independently and raise children. The person can only work at the simplest supervised jobs.	0.1 (0.066–0.142)
Intellectual disability/mental retardation, severe	This person has very low intelligence and cannot speak more than a few words, needs constant supervision and help with most daily activities, and can do only the simplest tasks.	0.16 (0.107–0.226)
Intellectual disability/mental retardation, profound	This person has very low intelligence, has almost no language, and does not understand even the most basic requests or instructions. The person requires constant supervision and help for all activities.	0.2 (0.133–0.283)

Modelling strategy

We modelled the prevalence of ID, both aetiology-specific IDs and idiopathic ID, over multiple steps.

First, we ran a DisMod-MR 2.1 model to estimate the total prevalence of intellectual disability of level IQ <70. We included lagged distributed income and child underweight summary exposure value (SEV) in the model as predictive covariates. Table 5 shows raw and exponentiated model coefficients for the covariates used in the estimation process for the DisMod model. Exponentiated coefficients can be interpreted as odds ratios.

Table 5. Model coefficient values (raw and exponentiated)

Covariate	Parameter	Coefficient (95% CI)	Exponentiated coefficient (95% CI)

Lagged distributed income (LDI) per capita	Prevalence	-0.29 (-0.38 to -0.2)	0.75 (0.68 to 0.82)
Age- and sex-specific SEV for child underweight	Prevalence	1.45 (0.17 to 2.81)	4.27 (1.18 to 16.53)
Sex	Prevalence	0.25 (0.18 to 0.32)	1.28 (1.19 to 1.37)

Second, we split the total prevalence of idiopathic ID into four severity levels: mild (IQ 50-69), moderate (IQ 35-49), severe (IQ 20-34), and profound (IQ below 20). We pooled a subset of studies that distinguished intellectual disability by these severity levels. We used cumulative severity levels (ie, IQ <50, IQ <35, and IQ <20) to maximise the number of sources. We estimated these cumulative severities' proportion of the <70 envelope via random effects meta-analyses stratified by two levels of income status (high-income versus low- and middle-income). These proportions were used to estimate discrete severities from the overall intellectual disability (IQ <70) prevalence. We estimated the final severity level, borderline disability (IQ 70-84), via another random-effects meta-analysis of the ratio of IQ 70-84 to IQ <70. The uncertainty of the pooled fractions and ratios was propagated throughout our calculations using 1000 draws from a normal distribution with mean and standard error estimated by the meta-analysis. The results of the meta-analysis are shown in Table 6.

Table 6. Proportion of intellectual disability cases by severity

Severity	Mean	Standard error
None	0.161	0.034
Borderline	0.161	0.034
Mild	0.375	0.037
Moderate	0.190	0.031
Severe	0.090	0.177
Profound	0.024	0.134

Third, we estimated prevalence of each aetiology-specific intellectual disability using models of the following parent causes. Since we model only developmental intellectual disability, causes that affect older populations such as stroke and Alzheimer's disease are not included in the causal attribution process.

Parent causes included in causal attribution:

- Neonatal preterm birth complications (<28w, 28-32w, 32-36w)
- Neonatal encephalopathy due to birth asphyxia and trauma
- Congenital birth defects (diaphragmatic hernia, cardiovascular anomalies)
- Haemolytic disease and other neonatal jaundice
- Meningitis (pneumococcal, *H influenzae* type B, meningococcal, other bacterial)
- Encephalitis
- Malaria
- Neonatal tetanus
- Neonatal sepsis and other neonatal infections

- Iodine deficiency
- African trypanosomiasis
- Down syndrome
- Klinefelter syndrome
- Chromosomal abnormalities (unbalanced rearrangements, Down syndrome, Edwards syndrome, Patau syndrome, other chromosomal abnormalities)
- Neural tube defects (eg, spina bifida, encephalocele)
- Hypertensive disorders of pregnancy (eclampsia, preeclampsia)
- Autism spectrum disorders (ASD)
- Fetal alcohol syndrome

We calculated the prevalence of idiopathic ID by subtracting all severity- and aetiology-specific ID from the severity-specific envelope assuming the residuals to represent idiopathic disability. If the residual was less than 5% of the severity-specific envelope, the prevalence of all aetiology-specific ID was proportionally squeezed to fit within 95% of the envelope, leaving 5% for idiopathic ID.

As we estimated the prevalence of individual aetiology-specific ID by models from the respective parent causes, the squeezing may have resulted in a distorted balance of prevalence estimates within their parent causes. With the aim to maintain consistencies of prevalence within each of the parent causes, we added the difference between the original and the squeezed prevalence estimates to the "motor impairment" sequela if the squeezed sequela represented "motor and cognitive impairment." For autism, we obtained the fraction of cases that result in ID from literature (0.29; 95% CI 0.27–0.30) and applied this fraction to the subtraction and squeezing processes. We assumed that all ID cases due to iodine deficiency (cretinism) would result in either severe or profound disability, and that Klinefelter syndrome cases that result in ID would have either borderline or mild severity. Lastly, in GBD 2013, all aetiology-specific models were squeezed into the overall (IQ <70) envelope, while in all subsequent rounds (including GBD 2021), we squeezed each model into its discrete severity envelope.

Diabetes mellitus

Diabetes mellitus prevalence is estimated for overall diabetes mellitus, diabetes mellitus type 1, and diabetes mellitus type 2 in GBD 2021.

Flowchart

Figure 1: *Calculating prevalence of diabetes mellitus (total, type 1, and type 2)*

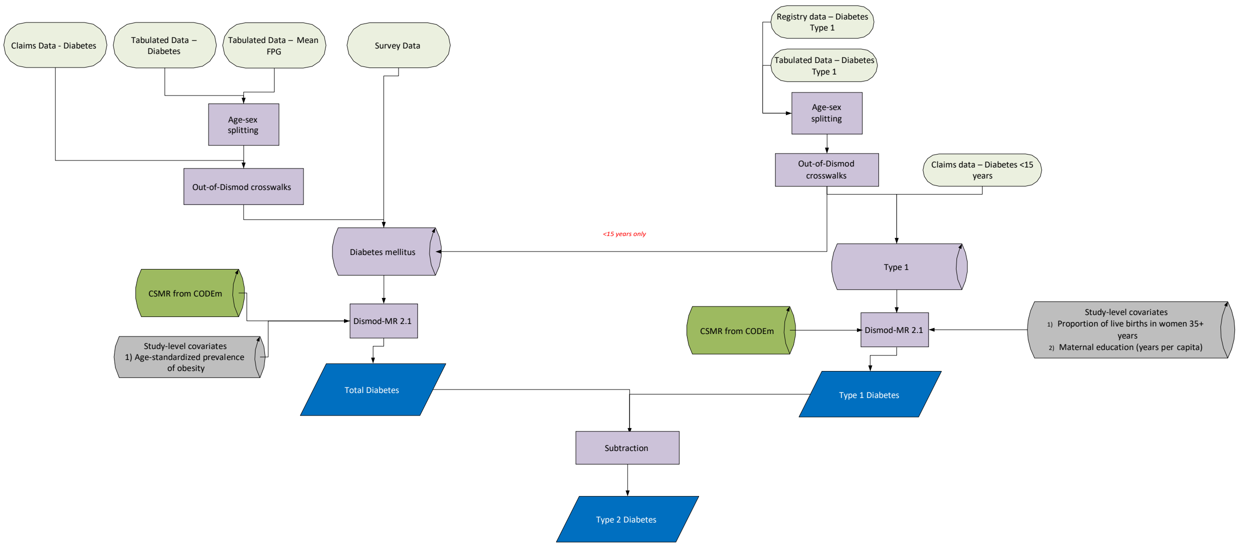

Figure 2: *Calculating type-specific diabetes sequelae*

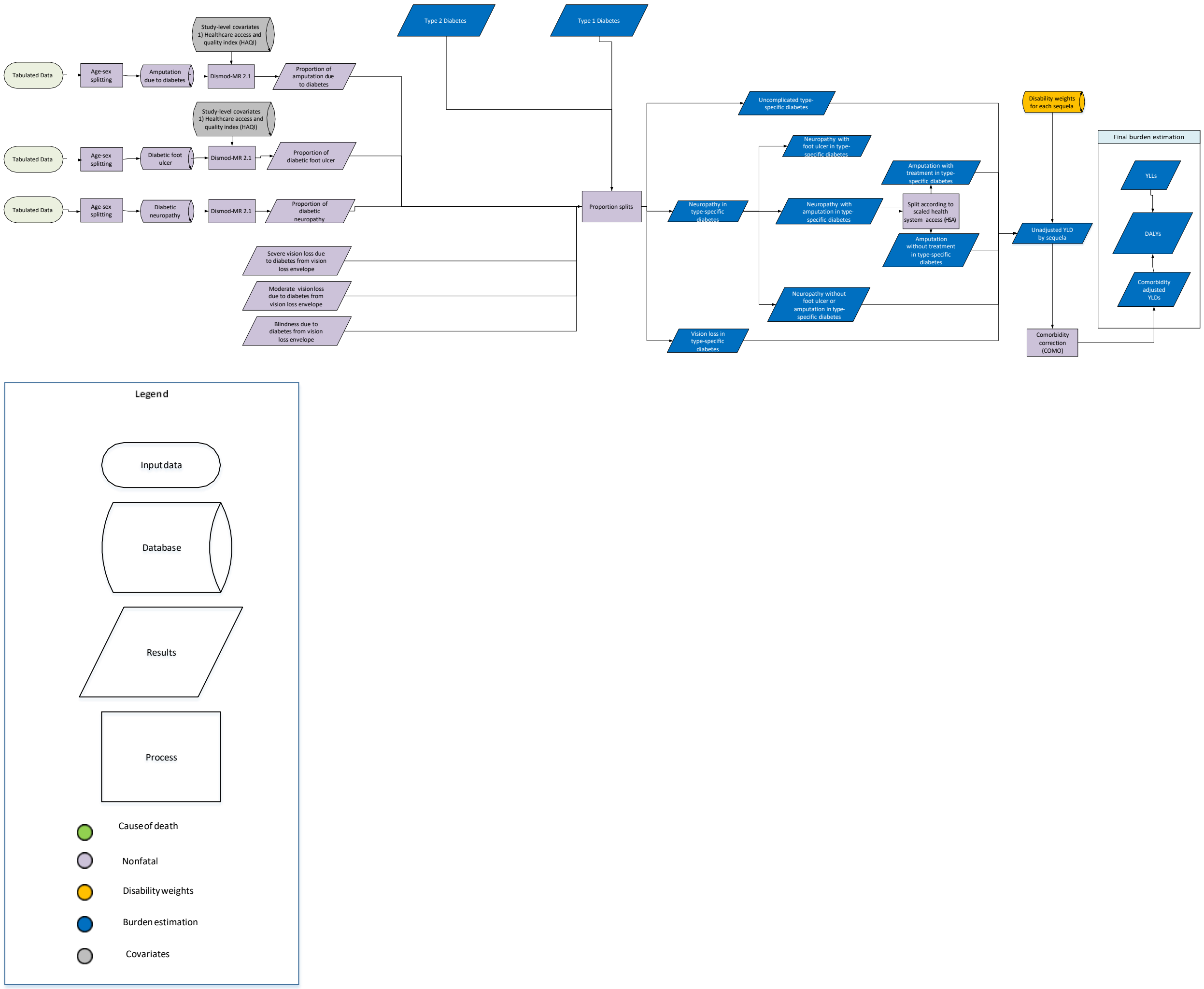

Case definitions

Clinical, reference, and alternative case definitions and diagnostic criteria are presented in the table below.

Table 1: *Case definitions for diabetes mellitus*

Quantity of interest	Clinical, reference, or alternative	Definition
Diabetes mellitus	Clinical	A metabolic disorder in which the body does not produce enough or does not respond normally to insulin, causing chronic high blood sugar (glucose) levels, which over time leads to serious damage to the heart, blood vessels, eyes, kidneys, and nerves.
Diabetes mellitus	Reference	Fasting plasma glucose (FPG) greater than or equal to 126 mg/dl (7 mmol/l) or current treatment (insulin or anti-diabetic drugs).
Diabetes mellitus	Alternative	FPG greater than a threshold not equal to 126 mg/dl (7mmol/L) or current treatment (insulin or anti-diabetic drugs).
Diabetes mellitus	Alternative	Blood sugar measured using glycated haemoglobin (HbA1c) at a given threshold or current treatment (insulin or anti-diabetic drugs).
Diabetes mellitus	Alternative	Blood sugar measured using oral glucose tolerance test (OGTT) at a given threshold or current treatment (insulin or anti-diabetic drugs).
Diabetes mellitus	Alternative	Blood sugar measured using post-prandial glucose test (PPG) at a given threshold or current treatment (insulin or anti-diabetic drugs).
Diabetes mellitus	Alternative	Any combination and thresholds of FPG/HbA1c/OGTT/PPG or current treatment (insulin or anti-diabetic drugs).
Diabetes mellitus	Alternative	Any combination and thresholds of FPG/HbA1c/OGTT/PPG (no treatment).
Diabetes mellitus	Alternative	Diabetes as reported in USA claims.
Diabetes mellitus	Alternative	Diabetes as reported in Taiwan (province of China) claims.
Diabetes mellitus	Alternative	Mean FPG in a representative population.
Diabetes mellitus type 1	Clinical	A metabolic disorder in which the body produces little to no insulin due to autoimmune destruction of pancreatic β-cells, causing chronic high blood sugar (glucose) levels which over time leads to serious damage to the heart, blood vessels, eyes, kidneys, and nerves.
Diabetes mellitus type 1	Reference	Cases of physician-diagnosed type 1 diabetes, or type 1 diabetes cases in a diabetic registry or hospital, or any case of diabetes in persons <15 years who are on insulin.
Diabetes mellitus type 1	Alternative	Cases of type 1 diabetes determined by c-peptide, islet cell autoantibodies (ICA), glutamic acid decarboxylase autoantibodies (GADA).
Diabetes mellitus type 1	Alternative	Cases of type 1 diabetes found using pharmacy data, diabetic camps, or another alternative data collection system that is not a registry.
Diabetes mellitus type 2	Clinical	A metabolic disorder in which the body does not respond normally to insulin, causing chronic high blood sugar (glucose) levels, which over time leads to serious damage to the heart, blood vessels, eyes, kidneys, and nerves.
Neuropathy	Reference	People with diabetes mellitus who have diabetic neuropathy determined by microfilament test.
Neuropathy	Alternative	People with diabetes mellitus who have diabetic neuropathy determined by a test that is not a microfilament test.
Diabetic foot	Reference	People with diabetes mellitus who have diabetic foot (ulcer).
Amputations due to diabetes mellitus	Reference	People with diabetes mellitus who have a lower limb amputation.

Amputations due to diabetes mellitus	Alternative	People with diabetes mellitus who have a specific part of the lower limb amputated (eg, toes, feet, below ankle).
Low vision/blindness due to diabetic retinopathy	Clinical	Vision loss due to damage to the retina among persons with diabetes that is caused by damaged blood vessels that can leak blood into the retina and cause scarring.
Low vision due to diabetic retinopathy	Reference	Low vision (presenting visual acuity of <6/18 ≥3/60 in the better eye using the Snellen chart) from damage to the retina caused by damaged blood vessels due to diabetes. Presenting vision is measured using any corrective lenses currently in use.
Low vision due to diabetic retinopathy	Alternative	Low vision (presenting visual acuity of <6/18 ≥3/60 in the better eye using the Snellen chart) from damage to the retina caused by damaged blood vessels due to diabetes, as measured by Rapid Assessment of Avoidable Blindness (RAAB) surveys.
Blindness due to diabetic retinopathy	Reference	Blindness (acuity in the better eye of <3/60 or <10% visual field around central fixation point) from damage to the retina caused by damaged blood vessels that can leak blood into the retina and cause scarring. Presenting vision is measured using any corrective lenses currently in use.
Blindness due to diabetic retinopathy	Alternative	Blindness (acuity in the better eye of <3/60 or <10% visual field around central fixation point) from damage to the retina caused by damaged blood vessels that can leak blood into the retina and cause scarring as measured by Rapid Assessment of Avoidable Blindness (RAAB) surveys.

Diabetes mellitus, diabetes mellitus type 1, diabetes mellitus type 2

Data seeking

Collaborator-provided sources that were either shared directly with us or were identified through searching the Global Health Data Exchange (GHDx) were reviewed for inclusion.

- 115 new sources were included in the overall diabetes mellitus model for GBD 2021.
    - Of these new sources, four were also included in the diabetes mellitus type 1 model for GBD 2021 as they were specific to type 1 diabetes mellitus.

No systematic review was conducted for the overall diabetes mellitus model for GBD 2021; the most recent systematic review was conducted for GBD 2019. In place of a systematic review, an "audit" of the current data in the total diabetes mellitus model was undertaken. The audit process involved returning to each data source to re-evaluate inclusion into the model, and to recheck data extractions for those sources that remain eligible for inclusion. GBD 2019 sources (excluding those specific to type 1 diabetes mellitus) and 111 new GBD 2021 sources were included in the audit (the four new sources specific to type 1 diabetes mellitus were not included).

Figure 3: *Diagram of data sources in the GBD 2021 diabetes mellitus model*

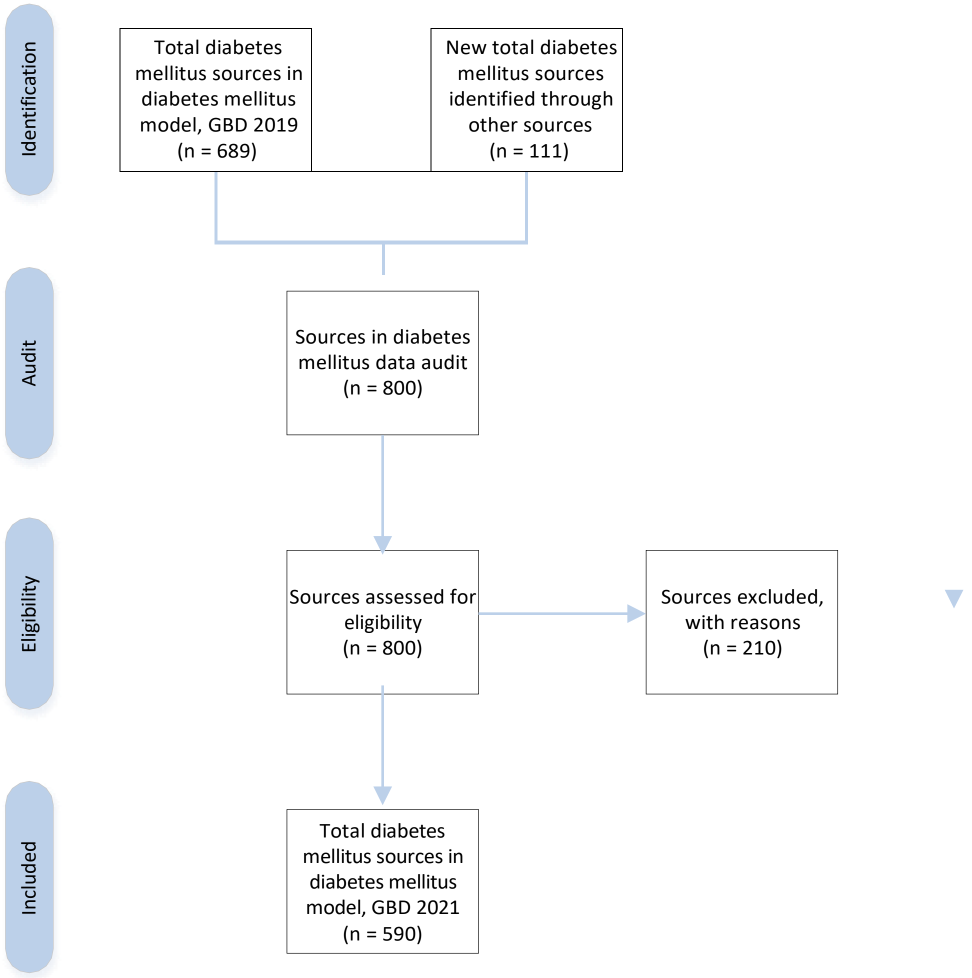

Main exclusion reasons include duplicative studies, not population representative, and self-report of diabetes status.

Source counts

Overall diabetes mellitus

Source counts for diabetes mellitus include sources directly used in the overall diabetes mellitus model, sources used in the diabetes mellitus type 1 model, and sources used in the diabetic sequelae models.

Table 2: *Data inputs for diabetes mellitus morbidity modelling by parameter*

	**Countries with data**	**New sources**	**Total sources**
Incidence	77	6	217
Prevalence	165	121	920
Remission	0	0	0
Other	45	0	85

Diabetes mellitus type 1

Source counts for diabetes mellitus type 1 include sources directly used in the diabetes mellitus type 1 model, and sources used in the diabetic sequelae models. The majority of the new sources in the table below can be attributed to the new source counting strategy in GBD 2021 of including sequelae source counts in the type 1 counts.

Table 3: *Data inputs for diabetes mellitus type 1 morbidity modelling by parameter*

	**Countries with data**	**New sources**	**Total sources**
Incidence	74	36	199
Prevalence	91	253	285
Remission	0	0	0
Other	45	81	85

Data inputs

Overall diabetes mellitus

Purpose

To incorporate all available population-representative data of diabetes, we accepted other measures of blood sugar (glycated haemoglobin A1c, oral glucose tolerance test, post-prandial glucose test) in addition to fasting plasma glucose to define diabetes. Studies that used random plasma glucose to define diabetes or self-reporting of diabetes status were not accepted.

Data

1. Data inputs came from four types of sources:
    - Estimates of diabetes in a representative population
    - Estimates of mean FPG in a representative population
    - Individual-level data of blood sugar from surveys
    - Insurance claims data from USA and Taiwan (province of China)

When a study reported both mean FPG and prevalence of diabetes, we used the prevalence of diabetes. Where possible, individual-level data from a cohort superseded any data described in a published paper. Individual-level data were collapsed and aggregated to produce estimates for each age group, sex, location, and year a survey was conducted.

2. Covariates
    - Age-standardised prevalence of obesity

Diabetes mellitus type 1

Purpose

To incorporate all available population-representative data of diabetes type 1, we accepted data that reported diabetes type 1, juvenile-onset diabetes, and insulin-dependent diabetes among children.

Data

1. Data inputs came from two types of sources:
    - Published estimates of type 1 diabetes mellitus in a representative population
    - Diabetic registries

2. Covariates
    - Proportion of livebirths in women 35+ years
    - Maternal education (years per capita)

Diabetes mellitus type 2

We found that the diagnostic criteria in the methodological sections of papers that report estimates of type 2 diabetes mellitus are not sufficiently specific for GBD. Thus, we calculated estimates of diabetes mellitus type 2 by subtracting the estimates of diabetes mellitus type 1 from estimates of overall diabetes mellitus for each age, sex, and location from 1990 to 2021.

Data processing

Overall diabetes mellitus

We performed several processing steps to the data to address sampling and measurement inconsistencies that will ensure the data are comparable across data sources and between high fasting plasma glucose modelling efforts.

1. *Small sample size*: Data with a sample size of ten or less were outliered prior to modelling.
2. *Mean FPG processing*: We used an ensemble distribution to estimate the prevalence of diabetes based on mean FPG for sources where data on prevalence of diabetes were not available but there were data on mean FPG. Essentially, we constructed a distribution based on unit-level data available in 31 different countries. Then we predicted out the prevalence of diabetes by age and sex. This provides the conversion of mean FPG to prevalence of diabetes defined as FPG greater than or equal to 126 mg/dL (7 mmol/L). Because this definition is not consistent with our reference case definition (which also includes those on treatment), we then apply an adjustment to adjust these datapoints to the reference case definition. For information on how these adjustments are made, please see the section, "Age splitting and bias adjustments" below.
3. *Age splitting and bias adjustments*: Reported estimates of prevalence were split by age and sex where possible. First, if studies reported prevalence for broad age groups by sex and by specific age groups but for both sexes combined, age-specific estimates were split by sex using the sex ratio from within the study. Second, input data reporting prevalence for both sexes that could not be split using a within-study ratio were split using a sex ratio derived from a meta-analysis of existing sex-specific data using meta-regression—Bayesian, regularised, trimmed (MR-BRT).[1] The female to male ratio for diabetes was 0.85 (0.61–1.09). Finally, after the application of bias adjustments, where studies reported estimates across age groups spanning 25 years or more, these were split into five-year age groups using the prevalence age pattern estimated by disease model—Bayesian meta-regression (DisMod-MR 2.1)[2] from a model that contained the subset of data with age range less than 25 years. Additional information on DisMod-MR 2.1 can be found in appendix 1, section 4.5 of the reference article.

Figure 4: *Age pattern used to split data with age range >25 years*

a. Female b. Male

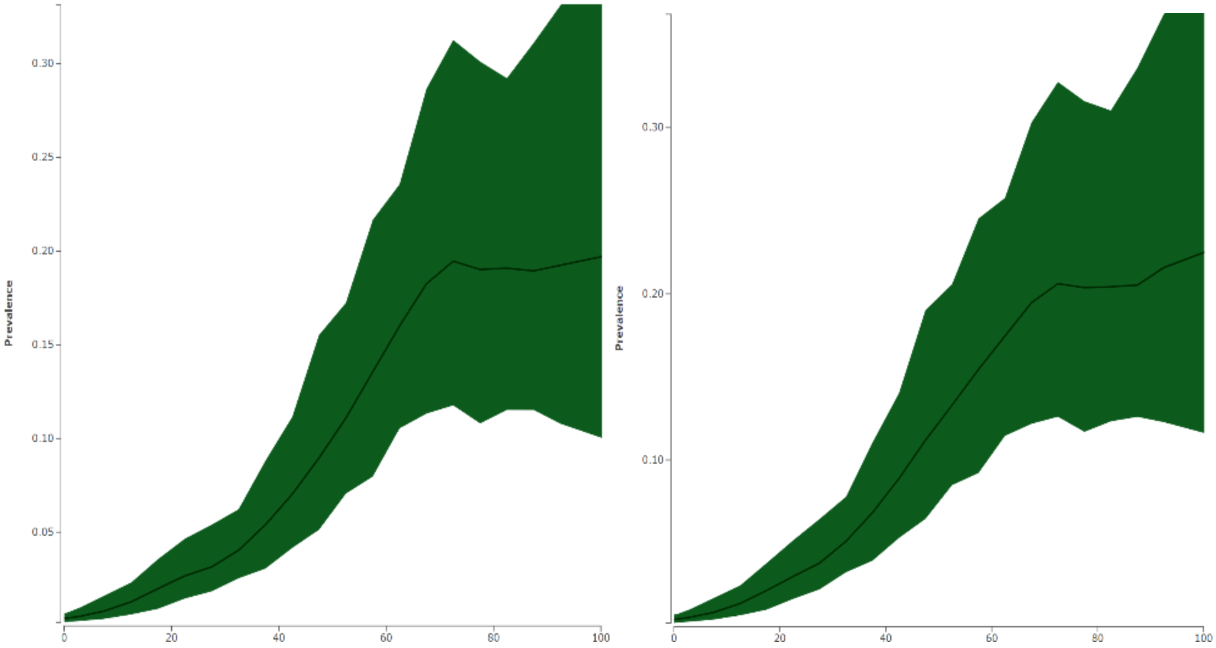

We also adjusted estimates from alternative case definitions to the reference case definition. Ratios were constructed between alternative case definitions and the reference case definition using data from surveys that measured glucose level based on different glucose tests on a single person or between survey and the insurance claims data. However, we assume that claims data in persons <15 years are type 1 diabetes and that 100% of people with diabetes are captured in this age group. Thus, we only adjust the claims data in persons >15 years. We used MR-BRT analysis to adjust for bias due to commercial insurance or use of alternative case definitions. We performed this analysis in logit space due to the high prevalence of diabetes (from simulations we learned that for prevalence greater than 50%, the log ratio method is biased).

The process of adjusting for non-reference data using MR-BRT with the logit-transformation method is described below:

1. Identify datapoints with overlapping year, age, sex, and location between alternative case definition and reference case definition.
2. Logit transform overlapping datapoints of alternative and reference case definitions.
3. Convert overlapping datapoints into a difference in logit space using the following equation: logit(alternative) – logit(reference).
4. Use the delta method to compute standard errors of overlapping datapoints in logit space, then calculate standard error of logit difference using the following equation:

√((variance of alternative) + (variance of reference)).

5. Using MR-BRT, conduct a random effects meta-regression to obtain the pooled logit difference of alternative to reference.
6. Apply the pooled logit difference to all datapoints of alternative case definitions using the following equation:
   New estimate = inverse.logit((logit(alternative)) – (pooled logit difference)).
7. Calculate new standard errors using the delta method, accounting for gamma (between-study heterogeneity).

Table 4: *MR-BRT crosswalk adjustment factors for diabetes mellitus*

**Data input**	**Reference or alternative case definition**	**Gamma**	**Beta coefficient, logit (95% UI)***	**Adjustment factor (95% UI)****
FPG >126 mg/dl (7 mmol/L) or Tx	Ref	--	---	---
HbA1c >6.5%	Alt	0.41	–0.30 (–1.11 to 0.51)	0.74 (0.33–1.66)
HbA1c >6.4% or Tx	Alt	0.31	0.06 (–0.56 to 0.67)	1.06 (0.57–1.96)
HbA1c >6%	Alt	0.57	0.70 (–0.43 to 1.82)	2.01 (0.65–6.20)
HbA1c >6.5% or Tx	Alt	0.29	–0.08 (–0.65 to 0.49)	0.92 (0.52–1.63)
FPG >100 mg/dl (5.6 mmol/L) or Tx	Alt	0.28	1.61 (1.06 to 2.15)	4.98 (2.89–8.58)
FPG >100 mg/dl (5.6 mmol/L)	Alt	0.27	1.55 (1.01 to 2.09)	4.72 (2.76–8.08)
FPG >110 mg/dl (6.1 mmol/L) or Tx	Alt	0.13	0.69 (0.44 to 0.93)	1.99 (1.55–2.54)
FPG >110 mg/dl (6.1 mmol/L)	Alt	0.16	0.59 (0.27 to 0.90)	1.8 (1.31–2.47)
FPG >115 mg/dl (6.4 mmol/L) or Tx	Alt	0.08	0.38 (0.22 to 0.53)	1.46 (1.25–1.70)
FPG >120 mg/dl (6.7 mmol/L)	Alt	0.13	–0.003 (–0.26 to 0.25)	0.997 (0.77–1.29)
FPG >121 mg/dl (6.7 mmol/L)	Alt	0.11	–0.04 (–0.26 to 0.18)	0.96 (0.77–1.20)
FPG >126 mg/dl (7 mmol/L)	Alt	0.14	–0.25 (–0.51 to 0.02)	0.78 (0.60–1.02)
FPG >140 mg/dl (7.8 mmol/L) or Tx	Alt	0.10	–0.27 (–0.48 to –0.07)	0.76 (0.62–0.93)
FPG >144 mg/dl (8 mmol/L) or Tx	Alt	0.12	–0.33 (–0.56 to –0.09)	0.72 (0.57–0.91)
OGTT >180 mg/dl (10 mmol/L) or Tx	Alt	0.17	0.82 (0.45 to 1.19)	2.28 (1.57–3.30)
OGTT >200 mg/dl (11.1 mmol/L)	Alt	0.17	0.41 (0.04 to 0.77)	1.5 (1.04–2.15)

OGTT >200 mg/dl (11.1 mmol/L) or Tx	Alt	0.17	0.41 (0.04 to 0.78)	1.5 (1.04–2.18)
FPG >110 mg/dl (6.1 mmol/L) or OGTT >200 mg/dl (11.1 mmol/L)	Alt	0.24	1.59 (1.08 to 2.11)	4.92 (2.94–8.24)
FPG >126 mg/dl (7 mmol/L) or OGTT >200 mg/dl (11.1 mmol/L)	Alt	0.10	0.62 (0.40 to 0.83)	1.85 (1.49–2.30)
FPG >126 mg/dl (7 mmol/L) or OGTT >200 mg/dl (11.1 mmol/L) or Tx	Alt	0.10	0.62 (0.40 to 0.85)	1.86 (1.49–2.33)
FPG >126 mg/dl (7 mmol/L) or OGTT >220 mg/dl (12.2 mmol/L)	Alt	0.07	0.36 (0.20 to 0.53)	1.44 (1.22–1.70)
FPG >144 mg/dl (8 mmol/L) or OGTT >200 mg/dl (11.1 mmol/L) or Tx	Alt	0.17	0.43 (0.06 to 0.80)	1.53 (1.06–2.22)
FPG >140 mg/dl (7.8 mmol/L) or OGTT >200 mg/dl (11.1 mmol/L) or Tx	Alt	0.18	0.43 (0.06 to 0.81)	1.54 (1.06–2.24)
FPG >140 mg/dl (7.8 mmol/L) or OGTT >200 mg/dl (11.1 mmol/L)	Alt	0.17	0.43 (0.06 to 0.80)	1.53 (1.06–2.22)
FPG >126 mg/dl (7 mmol/L) or OGTT >200 mg/dl (11.1 mmol/L) or HbA1c >6.1%	Alt	0.48	1.30 (0.30 to 2.30)	3.67 (1.35–10.00)
USA claims	Alt	0.15	–0.62 (–0.92 to –0.31)	0.54 (0.40–0.73)
Taiwan claims	Alt	0.38	0.15 (–0.63 to 0.93)	1.16 (0.53–2.53)

**MR-BRT crosswalk adjustments can be interpreted as the factor the alternative case definition is adjusted by to reflect what it would have been had it been measured using the reference case definition. If the log/logit beta coefficient is negative, then the alternative is adjusted up to the reference. If the log/logit beta coefficient is positive, then the alternative is adjusted down to the reference.*
***The adjustment factor column is the exponentiated beta coefficient. For log beta coefficients, this is the relative rate between the two case definitions. For logit beta coefficients, this is the relative odds between the two case definitions.*

Diabetes mellitus type 1

Based on the assumption that claims data in persons <15 years are type 1 diabetes and that 100% of people with diabetes are captured in this age group, we make no adjustments to data in these ages. Claims data are reported as prevalence.

There are a number of different sources and ascertainment methods that were used to identify people with type 1 diabetes. The majority of data that are reported in the literature are from a diabetic registry, hospital discharge data review, physician interview, or insulin use. We assumed that there is no systematic bias between these sources and consider sources identified through these methods as reference. For the other sources that use alternative ascertainment techniques (eg, pharmacy reports, diabetic camps, school reports), there was not sufficient data to perform an analysis on each individual type, and the model had relatively few datapoints in locations where these approaches were used. Therefore, we collapsed all alternative sources and treated the estimates from these sources as defined as an alternative case definition.

Table 5: *MR-BRT crosswalk adjustment factors for diabetes mellitus type 1*

**Data input**	**Reference or alternative case definition**	**Beta coefficient, logit (95% UI)***	**Adjustment factor (95% UI)****
Cases of physician-diagnosed type 1 diabetes, or type 1 diabetes cases in a diabetic registry or hospital, or any case of diabetes in persons <15 years who are on insulin	Ref	---	---
Ascertainment through pharmacy, schools, diabetic camps	Alt	–0.11 (–0.22 to 0.10)	0.90 (0.80 to 1.10)

**MR-BRT crosswalk adjustments can be interpreted as the factor the alternative case definition is adjusted by to reflect what it would have been had it been measured using the reference case definition. If the log/logit beta coefficient is negative, then the alternative is adjusted up to the reference. If the log/logit beta coefficient is positive, then the alternative is adjusted down to the reference.*
***The adjustment factor column is the exponentiated beta coefficient. For log beta coefficients, this is the relative rate between the two case definitions. For logit beta coefficients, this is the relative odds between the two case definitions.*

Modelling strategy

Overall diabetes mellitus

For GBD 2021, we estimated diabetes mellitus using DisMod-MR 2.1, which produces estimates of the prevalence of diabetes for each age, sex, geographical location, and year. We used data that reported prevalence and incidence for diabetes mellitus. After modelling, we replaced the total diabetes estimates for less than 15 years with the estimates from the type 1 diabetes mellitus model for each age, sex, location, and year for this age range. This was to ensure that the <15 years estimates for total diabetes mellitus and type 1 diabetes mellitus were equivalent, because we assume type 2 diabetes mellitus cannot occur before 15 years.

<u>Model parameters and estimates</u>

- We set a value prior of 0 for remission for ages 0 to 14
- We set a value prior of a maximum value of 0.01 for remission for ages 15 to 100
- We set a value prior of a maximum value of 0.15 for excess mortality for all ages
- We set a value prior of 0 for incidence for ages 0 to 1
- We set a value prior of a maximum value of 0.0008 for incidence for ages 1 to 15
- We set a value prior of a maximum value of 0.1 for incidence for ages 15 to 100

Table 6: *Summary of covariates used in the diabetes mellitus DisMod-MR model*

Covariate	Type	Parameter	Exponentiated beta (95% uncertainty interval)
Prevalence of obesity (age-standardised)	Country-level	Prevalence	1.47 (1.32–1.63)
Year	Country-level	Prevalence	1.04 (1.04–1.04)

Diabetes mellitus type 1

For GBD 2021, we estimated type 1 diabetes mellitus using DisMod-MR 2.1. We used data that reported incidence, standardised mortality ratio, and prevalence data in claims data for persons <15 years for diabetes mellitus type 1. We decided to not include reported type 1 diabetes prevalence in non-claims sources because we found that their estimates of prevalence and incidence were inconsistent. We decided to trust the incidence data and thus had to exclude the prevalence data from the model. Similarly, we did not include prevalence of diabetes type 1 in people >15 years from claims sources because of poor reporting on type of diabetes.

Model parameters and estimates

- We set a value prior of 0 for remission for all ages
- We set a value prior of a maximum value of 0.002 for excess mortality for ages 0 to 19
- We set a value prior of a maximum value of 0 for incidence for ages 0 to 1
- We set a value prior of a maximum value of 0.0006 for incidence for ages 1 to 20
- We set a value prior of a maximum value of 0.00033 for incidence for ages 65 to 100

Table 7: *Summary of covariates used in the diabetes mellitus type 1 DisMod-MR model*

Covariate	Type	Parameter	Exponentiated beta (95% uncertainty interval)
Proportion of livebirths in women 35+ years	Country-level	Incidence	14.66 (11.28–19.14)
Maternal education (years per capita)	Country-level	Incidence	1.09 (1.08–1.10)
Healthcare Access and Quality Index	Country-level	Excess mortality rate	0.98 (0.98–0.98)

Diabetes outcomes

Data seeking

1. No systematic review was conducted for the diabetes mellitus outcomes for GBD 2021. Previous systematic reviews for diabetic neuropathy, diabetic foot ulcer, and amputation due to diabetes mellitus were undertaken for GBD 2017.

Data inputs

Diabetic neuropathy

Data

1. Data inputs came from
   - Estimates of neuropathy in a representative population of people with diabetes
2. Covariates
   - None

Diabetic foot ulcer

1. Data inputs came from
   - Estimates of foot ulcer in a representative population of people with diabetes
2. Covariates
   - Healthcare Access and Quality Index

Amputation due to diabetes

1. Data inputs came from
   - Estimates of amputation in a representative population of people with diabetes
2. Covariates
   - Healthcare Access and Quality Index

Data processing

Diabetic neuropathy, diabetic foot ulcer, and amputation due to diabetes

All input data and sources were reviewed for GBD 2019. We found that nearly all sources reported estimates in age ranges that exceed 50 years. We identified a single study for each outcome that reported estimates in age bins of <25 years. We applied this age pattern to the remaining datapoints.

Due to a lack of data in the diabetic outcome models, no adjustments were undertaken for alternative case definitions, and therefore all case definitions were treated as reference.

Modelling strategy

For GBD 2021, we estimated amputation due to diabetes mellitus, diabetic neuropathy, and diabetic foot for diabetes mellitus type 1 and diabetes mellitus type 2 using DisMod-MR 2.1. We then multiply all

proportion draws from neuropathy/foot/amputation models by the parent diabetes model so that all estimates are in the same population-space.

While we do not directly model vision loss, we use the estimates of blindness and moderate and severe vision loss to estimate the proportion of the population with diabetes who have these conditions. The vision loss estimates are derived as part of the vision loss impairment analyses based on data ascribing vision loss to underlying causes in population-based surveys. Further details on these analyses can be found in the appendix section for vision loss estimation. The diabetes process takes these estimates into account when estimating diabetic outcomes.

First, we ensure that the sum of the prevalence for neuropathy due to diabetes mellitus, moderate vision loss due to diabetes mellitus, severe vision loss due to diabetes mellitus, and blindness due to diabetes mellitus does not exceed 90% of the prevalence of all diabetes mellitus. If the sum exceeds 90%, then we rescale the individual outcomes to 90%. This treats vision loss and neuropathy as mutually exclusive categories by assuming a patient will not have both simultaneously. From here, we calculate uncomplicated diabetes as the remainder of diabetes cases exclusive of neuropathy and vision loss.

We perform the same check to ensure that the prevalence of amputation due to diabetes mellitus and prevalence of foot ulcer due to diabetes mellitus does not exceed 90% of the prevalence of neuropathy due to diabetes mellitus. This treats foot ulcer and amputation as mutually exclusive categories by assuming a patient will not have both simultaneously.

In addition, we estimate the prevalence of amputation due to diabetes by splitting into with and without treatment using scaled health systems access (HSA) values. For diabetic amputation, we calculated a distribution of treated versus untreated amputation, defined as receiving a prosthesis or not. We first rescaled the IHME estimates to be between 0 and 0.9, under the assumption that 10% of amputees will not receive a prosthetic, even in high-income countries. We based this assumption on the retrospective study by Moore et al, which found that about 80% of patients following major lower extremity amputation were fitted with prostheses in the authors' institutions from 1978 to 1986 in the USA.[3] We then performed a population-weighted average of this country-specific value to obtain a proxy for the proportion of amputees who receive a prosthetic, by super-region. Because these are rough estimates based on large assumptions, we applied confidence intervals of +/- 50% of the value to reflect our uncertainty.

<u>Model parameters and estimates</u>

Diabetic neuropathy

- We set a value prior on the proportion of 0 from ages 0 to 1

Diabetic foot ulcer

- We set a value prior on the proportion of 0 from ages 0 to 10

Amputation due to diabetes

- We set a value prior of 0 for incidence for ages 0 to 15
- We set a value prior of 0 for remission for all ages

Table 9: *Summary of covariates used in the diabetic foot ulcer DisMod-MR model*

Covariate	Type	Parameter	Exponentiated beta (95% uncertainty interval)
Healthcare Access and Quality Index	Country-level	Proportion	0.99 (0.99–1.00)

Table 10: *Summary of covariates used in the amputations due to diabetes DisMod-MR model*

Covariate	Type	Parameter	Exponentiated beta (95% uncertainty interval)
Healthcare Access and Quality Index	Country-level	Prevalence	1.00 (0.99–1.02)

Severity distributions

We derived the disability weights for each sequela from the GBD disability weight survey. The table below illustrates the severity levels, lay descriptions, and associated disability weights applicable for outcomes related to diabetes mellitus type 1 and diabetes mellitus type 2:

Table 11: *Details on the severity levels for diabetes mellitus and the associated disability weight (DW) with that severity*

Severity level	Lay description	DW (95% CI)
Uncomplicated diabetes mellitus	Has a chronic disease that requires medication every day and causes some worry, but minimal interference with daily activities	0.049 (0.031–0.072)
Diabetic neuropathy	Has pain, tingling, and numbness in the arms, legs, hands, and feet. The person sometimes gets cramps and muscle weakness.	0.133 (0.089–0.187)
Diabetic neuropathy with diabetic foot	Has a sore on the foot that is swollen and causes some difficulty in walking.	[a]
Diabetic neuropathy with treated amputation	Has lost part of one leg, leaving pain and tingling in the stump. The person has an artificial leg that helps in moving around.	[a]

Diabetic neuropathy with untreated amputation	Has lost part of one leg, leaving pain and tingling in the stump. The person does not have an artificial leg, has frequent sores, and uses crutches.	[a]
Moderate vision loss due to diabetes mellitus	Has vision problems that make it difficult to recognise faces or objects across a room.	0.031 (0.019–0.049)
Severe vision loss due to diabetes mellitus	Has severe vision loss, which causes difficulty in daily activities, some emotional impact (for example, worry), and some difficulty going outside the home without assistance.	0.184 (0.125–0.259)
Blindness due to diabetes mellitus	Is completely blind, which causes great difficulty in some daily activities, worry and anxiety, and great difficulty going outside the home without assistance.	0.187 (0.124–0.26)

[a] The disability weights are produced from a combination of two health states: neuropathy and diabetic foot/amputation

Comparison to other published estimates

We identified two groups who also make global estimates of diabetes, the International Diabetes Federation (IDF) and the NCD Risk Factor Collaboration (NCD-RisC). The IDF publishes annual updates to their estimates, with the most recent estimates published in the tenth atlas (https://www.diabetesatlas.org/en/), and NCD-Risc published estimates in the paper "Worldwide trends in diabetes since 1980: a pooled analysis of 751 population-based studies with 4·4 million participants."

Below is a table comparing the global number of diabetes reported by GBD 2021, IDF 10th Atlas, and NCD-Risc for the closest years that align with 1990, 2010, and 2021.

Organisation	Source	1990	2010	2021
IHME	GBD 2021	148 million	319 million	489 million
International Diabetes Federation	IDF 10th Atlas	151 million (2000)	285 million (2010)	537 million (2021)

NCD Risk Factor Collaboration	Figure 7	148 million (1990)	350 million (2010)	422 million (2014)

There are several methodological and analytical differences between each group's approach which explains differences in the number of cases. The table below summarises the main differences.

Organisation	Age	Case definition	Analysis
IHME	All ages	FPG ≥7 mmol/L (126 mg/dL) or currently on treatment (insulin or drugs)	Bayesian hierarchical meta-regression
International Diabetes Federation	20–79 years	FPG ≥7 mmol/L (126 mg/dL) or OGTT ≥11.1 mmol/L (200 mg/dL) or HbA1c ≥6.5% or random plasma glucose ≥11.1 mmol/L (200 mg/dL) or self-report diabetes status	Generalised linear regression model
NCD Risk Factor Collaboration	≥18 years	FPG ≥7 mmol/L (126 mg/dL) or self-report diabetes status	Bayesian hierarchical model

References

[1]Zheng, P., Barber, R., Sorensen, R. J., Murray, C. J., & Aravkin, A. Y. (2021). Trimmed constrained mixed effects models: formulations and algorithms. *Journal of Computational and Graphical Statistics*, 1-13. https://www.tandfonline.com/doi/full/10.1080/10618600.2020.1868303

[2]GBD 2019 Diseases and Injuries Collaborators. Global burden of 369 diseases and injuries in 204 countries and territories, 1990–2019: a systematic analysis for the Global Burden of Disease Study 2019. *The Lancet* 2020; 396: 1204–22. doi: https://doi.org/10.1016/S0140-6736(20)30925-9

[3]Moore TJ, Barron J, Hutchinson F 3rd, Golden C, Ellis C, Humphries D. Prosthetic usage following major lower extremity amputation. *Clinical Orthopaedics and Related Research.* 1989 Jan;(238):219-24.

Diarrhoeal diseases

Flowchart

Diarrheal diseases

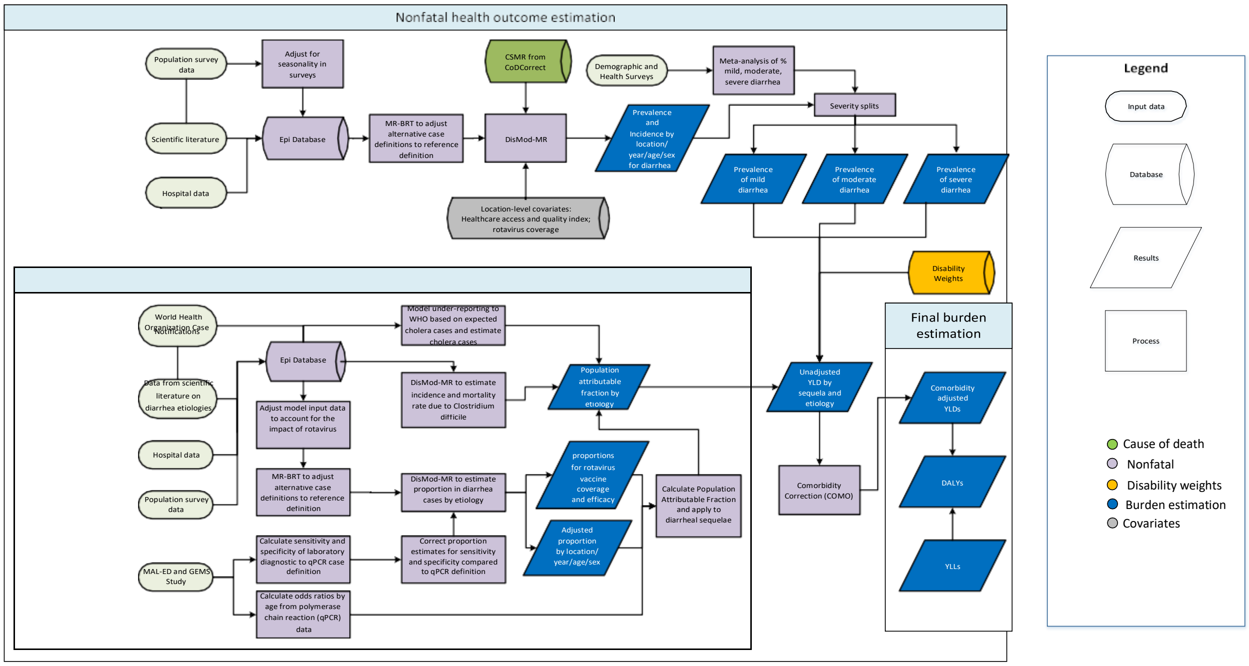

Case definition

We defined diarrhoeal disease episodes as three or more loose stools in a 24-hour period. In the diarrhoea models, self-reported prevalence is the reference category for all data adjustments. Hospital input data use ICD-9 codes 001-009.9 and ICD-10 codes A00-A09.

The case definitions accepted for diarrhoea are shown below.

Quantity of interest	Reference, alternative, or clinical	Definition
Incidence or prevalence of diarrhoea	Reference	Three or more abnormally loose stools in a 24-hour period. Self-reported or parental report for children.
Incidence or prevalence of diarrhoea	Clinical	The passage of three or more loose or liquid stools per day (or more frequent passage than is normal for the individual).
Incidence of inpatient diarrhoea episodes	Alternative	Incidence of diarrhoea episodes that become inpatients reported in health care data.
Incidence of diarrhoea episodes in clinical claims data	Alternative	Incidence of diarrhoea episodes reported in claims data.
Incidence of hospitalised diarrhoea episodes reported in literature	Alternative	Incidence of diarrhoea episodes that become inpatients reported in literature data.

Incidence of medically attended diarrhoea reported in literature	Alternative	Incidence of diarrhoeal episodes that are treated at a health care provider including primary care and outpatient facilities. Reported in literature only.

Input data

Model inputs

We used three main types of data in the diarrhoea non-fatal burden estimation: hospital data, population-based surveys, and data from scientific literature.

The first type of data is the incidence of diarrhoea in hospital settings, including inpatient, outpatient, and claims data. These data were identified using the ICD-9 codes 001-009.9 and ICD-10 codes A00-A09, and we adjusted prior to modelling for multiple admissions and multiple diagnoses. To be consistent with the population-based survey data, adjusted hospital data were transformed from incidence to prevalence using the following equation:

$$Prevalence = Incidence * \frac{duration(days)}{365}$$

The second type of data are from population-representative surveys, such as the Demographic and Health Surveys and the Multiple Indicator Cluster Surveys. We converted the prevalence of maternal-reported two-week period from surveys to point prevalence in one-year age groups using this equation:

$$Point\ Prevalence = Period\ Prevalence * \frac{Duration}{(Recall\ Period + Duration - 1)}$$

Where the mean duration was the duration in days, an average of 4.3 days (4.2–4.4) in both equations.[1]

Survey data were adjusted for seasonality. Surveys are frequently conducted over several months. To account for seasonal variation in diarrhoea prevalence, we fit a mixed-effects generalised additive model for each GBD region with a forced periodicity and a random intercept by country. The ratio between the monthly model-fit diarrhoea prevalence and the corresponding regional diarrhoea prevalence is a scalar to adjust survey data by month and geography.

The third type of data are from scientific literature. Inclusion criteria include diarrhoea as the case definition, studies with a sample size of at least 100, and a study duration of at least one year to avoid bias in the seasonal timing of diarrhoea. We excluded studies that reported on diarrhoeal outbreaks exclusively and studies that combined acute gastroenteritis with and without diarrhoea. We included all literature data sources used in GBD 2019 and conducted an updated review of literature for GBD 2021 covering the period 2/7/2019 to 1/3/2020 for diarrhoea prevalence, incidence, and all diarrhoea aetiologies.

Aetiologies

We extracted data on all aetiologies except *C. difficile* from scientific literature that reported the proportion of diarrhoea cases that tested positive for each pathogen. We applied the same inclusion and exclusion criteria described above for diarrhoea.

We searched articles using a PubMed search term that combined non-specific and aetiology-specific diarrhoea using the following search string:

(diarrhoea[title/abstract] OR diarrhea[title/abstract]) AND (2019/02/07:2020/12/31[PDat]) AND (incidence[title/abstract] OR prevalence[title/abstract] OR epidemiology[title/abstract] OR salmonella[title/abstract] OR aeromona[title/abstract] OR shigell*[title/abstract] OR enteropathogenic[title/abstract] OR enterotoxigenic[title/abstract] OR campylobacter[title/abstract] OR amoebiasis[title/abstract] OR entamoeb*[title/abstract] OR cryptosporid*[title/abstract] OR rotavirus[title/abstract] OR norovirus[title/abstract] OR adenovirus[title/abstract] OR etiology[title/abstract]) NOT (appendicitis[title/abstract] OR esophag*[title/abstract] OR surger*[title/abstract] OR gastritis[title/abstract] OR liver[title/abstract] OR case report[title] OR case-report[title] OR therapy[title] OR treatment[title] Crohn[title/abstract] OR "inflammatory bowel"[title/abstract] OR irritable[title/abstract] OR travel*[title] OR Outbreak[title] OR Review[ptyp] OR vomiting[title/abstract) NOT (animals[MeSH] NOT humans[MeSH])*

We identified 924 studies, of which 60 met our inclusion criteria. We extracted data for location, sex, year, and age.

Figure 4. Diarrhoeal disease aetiology systematic review PRISMA diagram

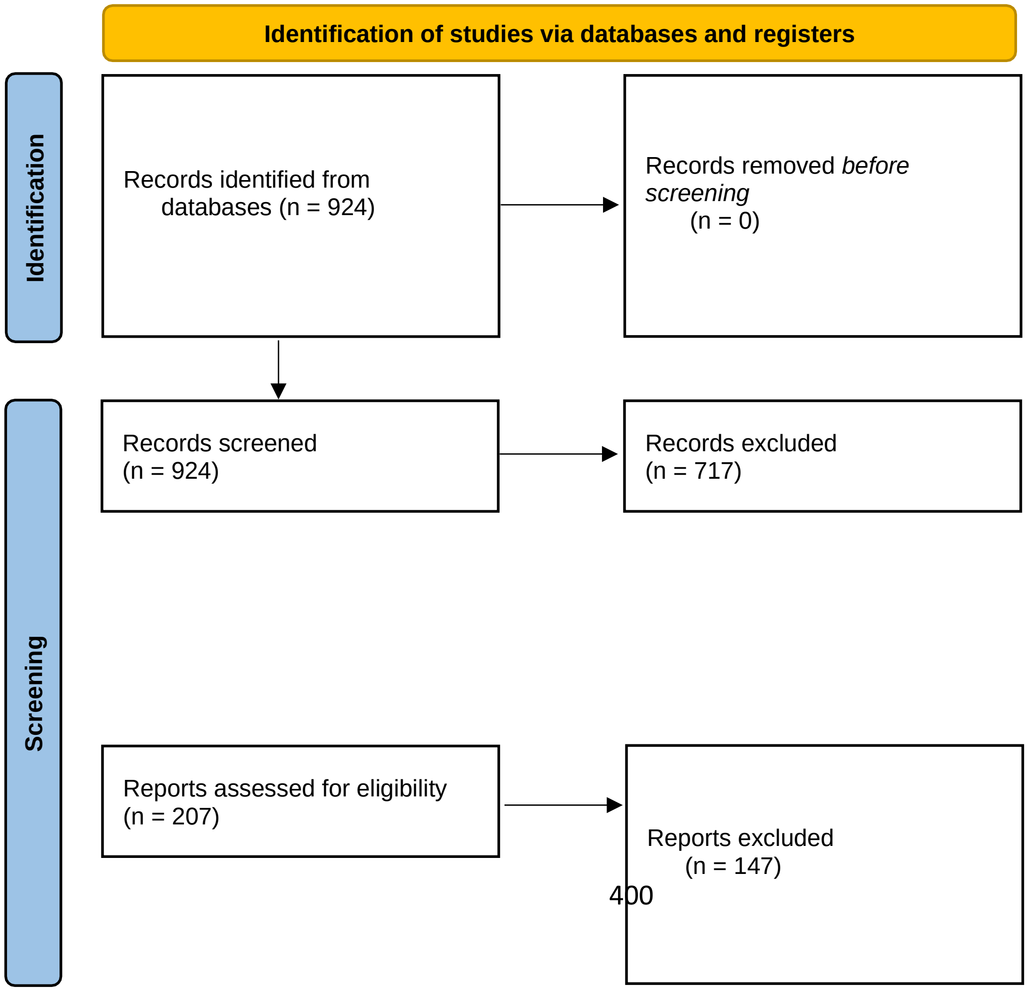

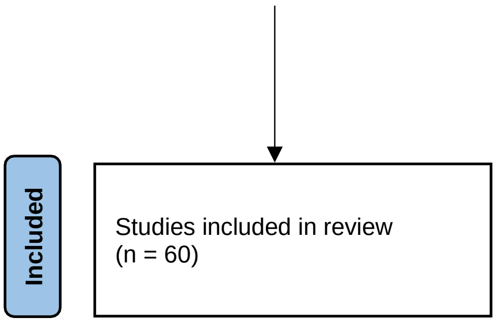

Similarly, we used the following search string to supplement incidence data on *C. difficile:*

> "clostridium difficile" AND diarrhea[title/abstract] AND (epidemiolog* OR incidence OR prevalence) AND (("2019/02/07"[PDat] : "2020/12/31"[PDat])) NOT (animals[MeSH] NOT humans[MeSH])

We identified 57 studies, of which one met our inclusion criteria. We extracted datapoints for location, sex, year, and age.

Figure 5. C. difficile *systematic review PRISMA diagram*

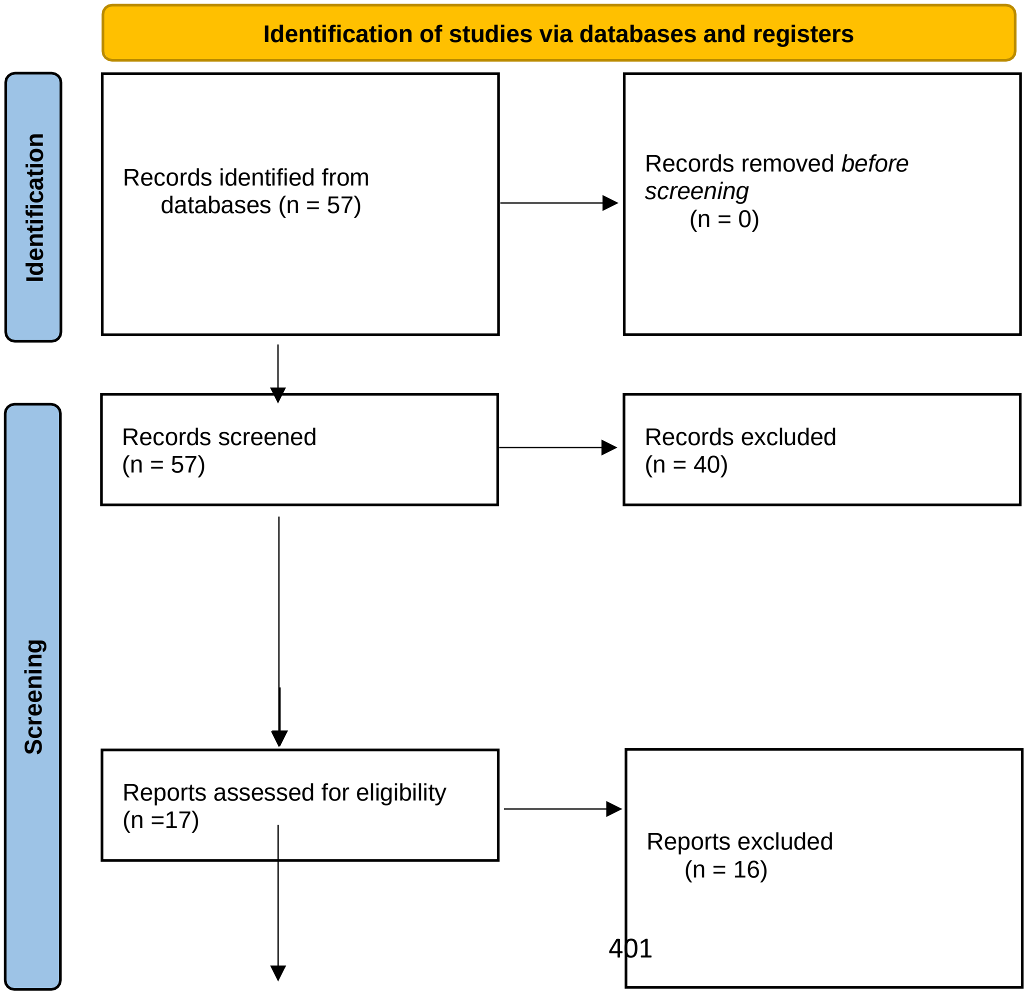

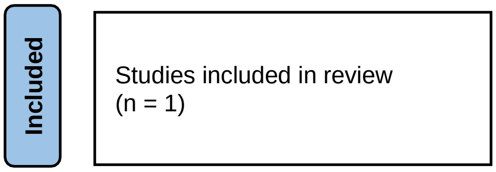

Additionally, we searched specifically for data sources detailing rotavirus coverage and vaccine efficacy using the following search string:

(((rotavirus[title/abstract] AND vaccine[title/abstract] AND (efficacy[title/abstract] OR effectiveness[title/abstract]) AND (2019/02/07[PDAT] : 2020/12/31[PDAT])))) NOT Review[Publication Type] NOT (animals[MeSH] NOT humans[MeSH])

We identified 63 studies via PubMed and an additional 27 studies through manual reference search. Of the 90 studies identified, six met our inclusion criteria.

Figure 6. Rotavirus vaccine efficacy systematic review PRISMA diagram

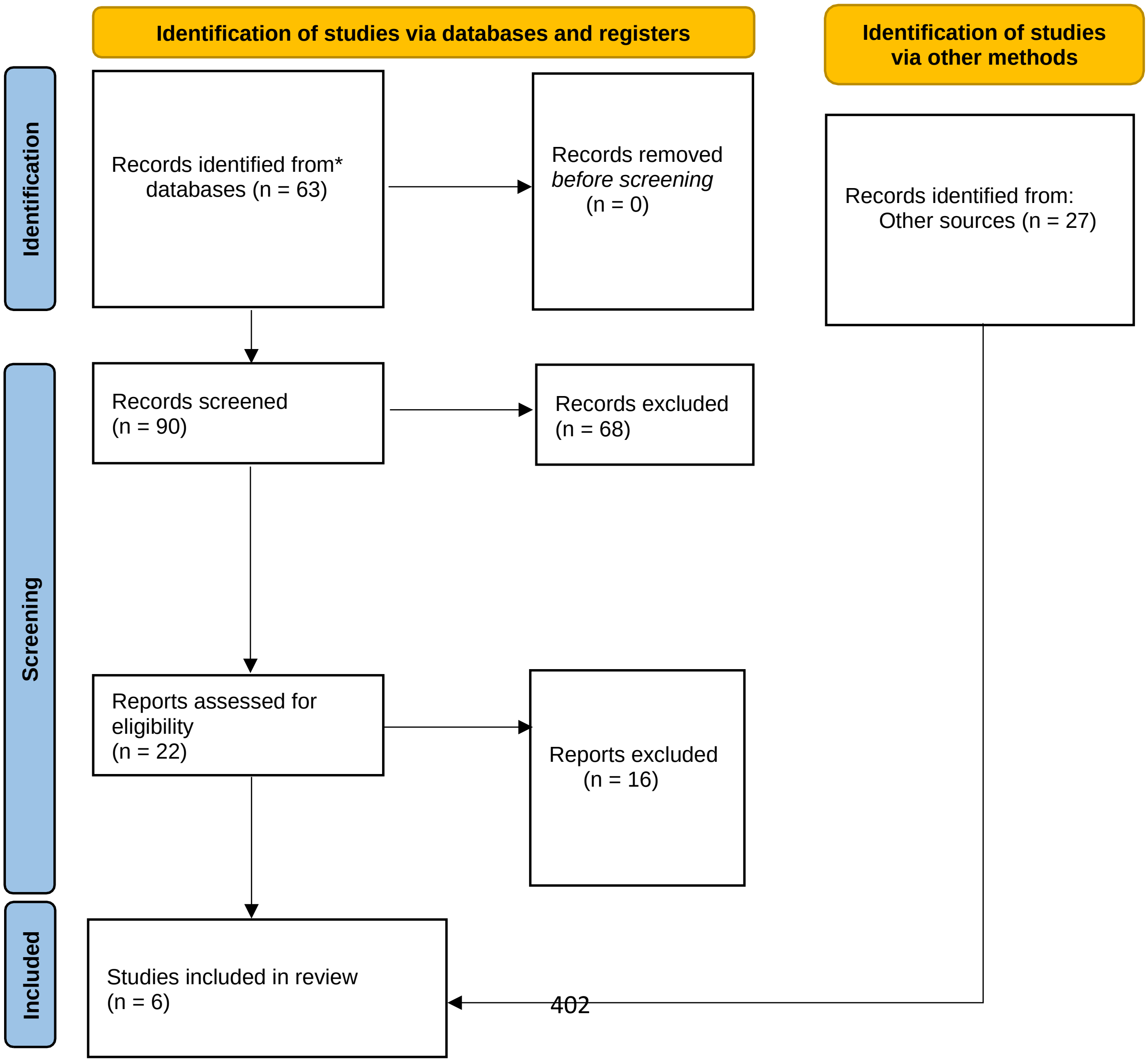

We used the Global Enteric Multicenter Study (GEMS), a seven-site, case-control study of moderate-to-severe diarrhoea in children under 5 years,[2] and the MAL-ED study,[3] a multi-site birth cohort, to calculate odds ratios for the diarrhoeal pathogens. We analysed raw data for a systematic reanalysis, representative of the distribution of cases and controls by age and site that were tested for the presence of pathogen using quantitative polymerase chain reaction (qPCR).[4]

Data that did not use qPCR for detection were adjusted for sensitivity and specificity prior to modelling in order to standardise data regardless of detection method. Adjusting these data prior to modelling allowed us to adjust only data that did not use qPCR, as well as better control for values at extreme bounds and capture uncertainty in modelling.

Newly identified sources were added to studies and sources identified in previous rounds of the GBD, resulting in 1694 total unique sources for diarrhoeal diseases, representing data from 205 countries (Table 1).

Table 1. Unique source counts for diarrhoeal diseases by measure

Measure	Total sources	Countries with data
All measures	1694	287
Prevalence	1242	171
Other	452	116

Data crosswalks

One of the GBD core principles is to use all available data to inform our estimates. To account for differences between studies, we conducted a meta-regression of the ratio of reference to non-reference data using the meta-regression—Bayesian, regularised, trimmed (MR-BRT) tool. When possible, crosswalks based on data matched within studies on age, sex, and location are used. When not possible, ratios between alternative and reference case definitions/methods were based on data matched between studies, nearby in age, year, with exact matches on sex and location. We adjusted inpatient data and claims data up to the level of self-reported data (our reference case definition) (table 2). Additionally, age was shown to be a predictor of this adjustment for claims data. To accommodate any non-linear association between age and the crosswalk ratios, we incorporated splines on age midpoint as shown in table 2.

Table 2. Diarrhoeal disease crosswalk coefficients

Data input	Reference or alternative case definition	Gamma	Crosswalk covariate	Beta coefficient, logit (95% UI)
Self-reported diarrhoea	ref	--	--	--

Clinical, inpatient	alt	2.07	intercept	6.51 (6.43–6.58)
Claims, MarketScan	alt	0	age_mid_0	5.37 (5.20–5.53)
Claims, MarketScan	alt		age_mid_1	7.75 (6.74–8.76)
Claims, MarketScan	alt		age_mid_2	7.56 (7.01–8.10)
Claims, MarketScan	alt		age_mid_3	6.71 (6.23–7.19)
Claims, MarketScan	alt		age_mid_4	5.32 (4.89–5.76)
Literature, inpatient	alt	1.91	intercept	2.00 (–0.66 to 4.66)
Literature, hospital-based	alt	0.16	intercept	0.29 (0.05–0.54)

Age-sex splits

Data were age and sex split based on population and a modelled age-curve generated using age-specific data as inputs in MR-BRT to better estimate the distribution of non-age-specific data.

Severity split inputs

Diarrhoeal diseases have three severity levels: mild, moderate, and severe (Table 3). The proportion of diarrhoea cases that are assigned to each comes from a systematic review of diarrhoea severity.[1] Mild cases are the proportion of diarrhoea cases that did not seek medical care (64.8%); moderate cases are the proportion that sought medical care but did not have severe dehydration or bloody stool (28.9%); and severe cases are the proportion that sought medical care with severe dehydration or bloody stool (6.9%). These proportions are based on the frequency of dehydration and bloody stool among community-based studies reported in the systematic review.

Table 3. Severity splits, details on the severity levels for diarrhoea in GBD 2019 and the associated disability weight (DW) with that severity.

Severity level	Lay description	Disability weight (95% CI)	Proportion
Mild	Has diarrhoea defined as 3 or more loose stools in a 24-hour period with no dehydration.	0.074 (0.049–0.104)	64.8%
Moderate	Has diarrhoea defined as 3 or more loose stools in a 24-hour period with painful cramps and feeling thirsty and any dehydration.	0.188 (0.125–0.264)	28.9%
Severe	Has diarrhoea defined as 3 or more loose stools in a 24-hour period with painful cramps and is very thirsty or feels nauseated or tired and/or severely dehydrated.	0.247 (0.164–0.348)	6.9%

Modelling strategy

Diarrhoea incidence and prevalence

The non-fatal diarrhoeal disease burden is modelled in DisMod-MR 2.1, a Bayesian meta-regression modelling framework. DisMod-MR produces estimates of the incidence, prevalence, and remission of diarrhoea for each age, sex, geographical location, and year. We defined remission, or the time to recovery, as five days average. The reference category for our input data is community-based diarrhoea episodes such as data from population-representative surveys or community cohorts. As described in the data crosswalks section above, input data that are from a different population, such as hospital inpatient groups, are adjusted before modelling by determining a meta-regression ratio of non-reference to reference data values, so that they are consistent with the reference category. Country-level covariates are used to inform the model (Table 4).

Table 4. Covariates. Summary of covariates used in the diarrhoea DisMod-MR meta-regression model

Covariate	Type	Parameter	Exponentiated beta (95% uncertainty interval)
Rotavirus vaccine coverage	Country-level	Prevalence	0.99 (0.99–0.99)
Socio-demographic Index	Country-level	Prevalence	0.14 (0.14–0.14)
Healthcare Access and Quality Index	Country-level	Excess mortality	0.97 (0.97–0.97)

Aetiologies

We estimated diarrhoeal disease aetiologies independently from overall diarrhoea envelope using a counterfactual strategy for enteric adenovirus, *Aeromonas*, *Entamoeba histolytica* (amoebiasis), *Campylobacter*, *Cryptosporidium*, typical EPEC, enterotoxigenic *Escherichia coli* (ETEC), norovirus, non-typhoidal *Salmonella* infections, rotavirus, and *Shigella*. *Vibrio cholerae* and *C. difficile* were modelled separately **(Table 5)**.

Table 5. Inpatient to community crosswalk coefficients for diarrhoeal disease aetiologies, not including *Vibrio cholerae* or *C. difficile*

Aetiology	Data input	Reference or alternative case definition	Gamma	Crosswalk covariate	Beta coefficient, logit (95% UI)
All	community-based samples	ref	--	--	--
Adenovirus	Clinical, inpatient	alt	0.14	intercept	0.36 (0.15 to 0.56)
Aeromonas	Clinical, inpatient	alt	0.14	intercept	0.19 (–0.09 to 0.46)

Amoebiasis	Clinical, inpatient	alt	0.85	intercept	0.17 (–0.43 to 0.78)
Campylobacter	Clinical, inpatient	alt	0.26	intercept	–0.11 (–0.33 to 0.12)
Cryptosporidium	Clinical, inpatient	alt	0.05	intercept	0.26 (0.12 to 0.39)
EPEC	Clinical, inpatient	alt	0.03	intercept	0.13 (0.002 to 0.27)
ETEC	Clinical, inpatient	alt	0.04	intercept	0.23 (0.08 to 0.37)
Norovirus	Clinical, inpatient	alt	0.04	intercept	0.06 (–0.06 to 0.18)
Rotavirus	Clinical, inpatient	alt	0.35	intercept	0.71 (0.54 to 0.89)
Salmonella	Clinical, inpatient	alt	0.15	intercept	0.49 (0.21 to 0.76)
Shigellosis	Clinical, inpatient	alt	0.38	intercept	0.39 (0.13 to 0.66)

Diarrhoeal aetiologies are attributed to diarrhoeal cases using a counterfactual approach. We calculated a population attributable fraction (PAF) from the proportion of diarrhoea cases that are positive for each aetiology. The PAF represents the relative reduction in diarrhoea burden if there was no exposure to a given aetiology. As diarrhoea can be caused by multiple pathogens and the pathogens may co-infect, PAFs can overlap and are not scaled to sum to 100%. We calculated the PAF from the proportion of diarrhoea cases that are positive for each aetiology. We used the following formula to estimate PAF:[5]

$$PAF = Proportion * (1 - \frac{1}{OR})$$

Where *Proportion* is the proportion of diarrhoea cases positive for an aetiology and *OR* is the odds ratio of diarrhoea given the presence of the pathogen.

We dichotomised the continuous qPCR test result using the value of the cycle threshold (Ct) that most accurately discriminated between cases and controls. The Ct values range from 0 to 35 cycles representing the relative concentration of the target gene in the stool sample. A low value indicates a higher concentration of the pathogen, while a value of 35 indicates the absence of the target in the sample. We used the lower Ct value when we had multiple Ct values for the cut-point. The case definition for each pathogen is a Ct value that is below the established cutoff point **(Table 6)**.

Table 6. Single to multi-pathogen study crosswalk coefficients for diarrhoeal disease aetiologies, not including *Vibrio cholerae* or *C. difficile*

Aetiology	Data input	Reference or alternative case definition	Gamma	Crosswalk covariate	Beta coefficient, logit (95% UI)
All	Multi-pathogen studies	ref	--	--	--
Adenovirus	Single pathogen	alt	0.00	intercept	1.06 (0.89 to 1.23)
Aeromonas	Single pathogen	alt	N/A	intercept	N/A
Amoebiasis	Single pathogen	alt	N/A	intercept	N/A
Campylobacter	Single pathogen	alt	N/A	intercept	N/A
Cryptosporidium	Single pathogen	alt	N/A	intercept	N/A
EPEC	Single pathogen	alt	0.00	intercept	0.09 (–1.08 to 1.26)
ETEC	Single pathogen	alt	0.00	intercept	0.25 (0.08 to 0.42)
Norovirus	Single pathogen	alt	N/A	intercept	N/A
Rotavirus	Single pathogen	alt	0.48	intercept	0.41 (0.17 to 0.65)
Salmonella	Single pathogen	alt	0.00	intercept	0.98 (0.88 to 1.07)
Shigellosis	Single pathogen	alt	4.96	intercept	2.98 (–0.19 to 6.16)

We used a generalised linear mixed effects logistic regression model to calculate the odds ratio for under 1 year and 1–2 years old for each of our pathogens from the MAL-ED study. The MAL-ED study was used exclusively because the samples tested from that study are from community-based samples, which we determined were more representative of non-fatal diarrhoea than the GEMS samples, which tested only

moderate-to-severe diarrhoea. The odds ratio for 1–2 years was applied to all GBD age groups over 5 years. There were three pathogen-age odds ratios that were not statistically significant: *Aeromonas* and amoebiasis in under 1 year and *Campylobacter* in 1–2 years. If the odds ratio was not statistically significant, we transformed the odds ratios only for those aetiologies in log-space such that exponentiated values could not be below 1. The transformation was:

$$Odds\ ratio = exp(log(OR) - 1)) + 1$$

We modelled the proportion data using the Bayesian meta-regression tool DisMod-MR to estimate the proportion of positive diarrhoea cases for each separate aetiology by location/year/age/sex and to adjust for the covariates. We used the estimated sensitivity and specificity of the original laboratory diagnostic test results from the pooled GEMS and MAL-ED qPCR stool samples compared to the qPCR test result to adjust our proportion before we modelled the proportions:[6]

$$Proportion_{True} = \frac{(Proportion_{Observed} + Specificity - 1)}{(Sensitivity + Specificity - 1)}$$

We used this correction to account for the fact that the proportions we used are based on a new test that is not consistent with the laboratory-based case definition (qPCR versus GEMS conventional laboratory testing for pathogens).[7] Because differences in the type of PCR used in the original (non-reference qPCR diagnostic) between GEMS and MAL-ED in detecting norovirus, we combined the sensitivity and specificity results for norovirus such that 50% of the draws were coming from GEMS test results exclusively, and 50% of the draws were coming from MAL-ED test results exclusively. Additionally, because the original laboratory diagnostic technique used for *Campylobacter* in MAL-ED was one not commonly used, we only used GEMS to determine the sensitivity and specificity of bacterial culture compared to qPCR in detecting *Campylobacter.*[8]

Our literature review extracted the proportion of any enteropathogenic *Escherichia coli* (EPEC) without differentiating between typical (tEPEC) and atypical (aEPEC). In order to be consistent with the odds ratios that we obtained, we adjusted our proportion estimates of any EPEC to typical EPEC only. This adjustment was informed by a subset of our literature review that reported both atypical and typical EPEC. We estimated a ratio by super-region of tEPEC to any EPEC and adjusted our proportion estimates accordingly. We found that the majority of EPEC diarrhoea cases were positive for atypical EPEC, consistent with other published work.[9] We applied the same approach to differentiate between heat-stable toxin (ST) and heat-labile toxin-producing (LT) ETEC. For the first time, GBD 2019 split these serotypes so that estimates in GBD 2019 represent the diarrhoeal disease burden attributable to ST-ETEC. This was based on work showing that ST-ETEC was much more pathogenic than LT-ETEC. As our proportion data were extracted for any ETEC, we determined a proportion of all ETEC that produced ST from the GEMS and MAL-ED studies and applied that ratio to our input data so that they represented ST-ETEC only. We re-estimated the sensitivity and specificity values as well as the odds ratios for our new definition of ST-ETEC.

For *Vibrio cholerae* (cholera), we used the literature review to estimate the expected number of cholera cases for each country-year using the incidence of diarrhoea (estimated using DisMod-MR) and the proportion of diarrhoea cases that are positive for cholera. We assigned cholera PAF using odds ratios

from the qPCR results to estimate a number of cholera-attributable cases. We compared this expected number of cholera cases to the number reported to WHO at the country-year level.[10] We modelled the under-reporting fraction to correct the cholera case notification data for all countries using health system access and the diarrhoea SEV scalar to predict total cholera cases. We used the age-specific proportion of positive cholera samples in DisMod-MR and our incidence estimates to predict the number of cholera cases for each age/sex/year/location. Finally, we modelled the case fatality ratio of cholera using DisMod-MR and to estimate the number of cholera deaths.

For *C. difficile*, we modelled incidence data identified via systematic review and excess mortality estimates in DisMod-MR 2.1. Excess mortality rates (EMRs) were computed based on case-fatality rates by age from hospital data and duration using the following equation: EMR = –ln(1 – CFR)/duration. Duration was assumed to be 1.0 month (0.3–1.7).

For rotavirus, we explicitly accounted for rotavirus vaccine efficacy when estimating attributable fraction, as in GBD 2019. The impact of the rotavirus vaccine is dependent on modelled vaccine coverage for a location-year and on the rotavirus vaccine efficacy (VE). Numerous studies demonstrate a difference in VE by national income and development.[11] We also determined via LASSO (least absolute shrinkage and selection operator) that Socio-demographic Index (SDI) was the best predictor of rotavirus VE. We used a meta-regression with SDI as covariate to predict the rotavirus VE by location and year.

For GBD 2019, we explicitly incorporated the results from our analysis of VE to produce more robust estimates of the proportion of diarrhoea that has rotavirus over time and space. We assumed that the impact of the vaccine can be represented as 1 minus the product of the estimated vaccine coverage and VE.

$$Vaccine\ impact = 1 - vaccine\ coverage * vaccine\ efficacy$$

Both of these values vary in time and space but not by age. To avoid discontinuities in our DisMod model, we adjusted the input proportion data to remove the impact of the rotavirus vaccine by dividing the observed proportion by the vaccine impact.

$$Rotavirus\ proportion_{Adjusted} = \frac{Rotavirus\ proportion}{1 - Cov_{RotaV} * VE_{Modeled}}$$

The result from DisMod is the modelled proportion of diarrhoea positive for rotavirus in the absence of the vaccine. This modelled value is then multiplied by the impact of the rotavirus vaccine to determine the estimated proportion of diarrhoea positive for rotavirus in the presence of the vaccine. Our modified attributable fraction is then:

$$DisModPAF = Modeled\ Proportion\ (from\ DisMod) * (1 - \frac{1}{OR})$$

The last step is to account for the expected impact of the rotavirus vaccine. We do this using the equation below:

$$PAF_{Rota} = DisModPAF * \frac{(1 - Cov_{RotaV} * VE_{Modeled})}{(1 - DisModPAF * Cov_{RotaV} * VE_{Modeled})}$$

Where the final attributable fraction for rotavirus is the product of the PAF estimated in DisMod-MR and the expected reduction in that PAF given modelled vaccine coverage and modelled VE by location-year,

and this value is only applied to children 28 days to 5 years old. The product of the rotavirus attributable fraction and the number of deaths or cases of diarrhoea is the number of deaths and cases caused by rotavirus.

References

1 Lamberti LM, Fischer Walker CL, Black RE. Systematic review of diarrhea duration and severity in children and adults in low- and middle-income countries. *BMC Public Health* 2012; **12**: 276.

2 Kotloff KL, Nataro JP, Blackwelder WC, *et al.* Burden and aetiology of diarrhoeal disease in infants and young children in developing countries (the Global Enteric Multicenter Study, GEMS): a prospective, case-control study. *Lancet* 2013; **382**: 209–22.

3 Platts-Mills J, Liu J, Rogawski E. Aetiology, burden and clinical characteristics of diarrhoea in children in low-resource settings using quantitative molecular diagnostics: results from the MAL-ED cohort study. *Lancet Glob Health* 2018; Accepted.

4 Liu J, Gratz J, Amour C, *et al.* A laboratory-developed TaqMan Array Card for simultaneous detection of 19 enteropathogens. *J Clin Microbiol* 2013; **51**: 472–80.

5 Miettinen OS. Proportion of disease caused or prevented by a given exposure, trait or intervention. *Am J Epidemiol* 1974; **99**: 325–32.

6 Reiczigel J, Földi J, Ozsvári L. Exact confidence limits for prevalence of a disease with an imperfect diagnostic test. *Epidemiol Infect* 2010; **138**: 1674–8.

7 Platts-Mills JA, Operario DJ, Houpt ER. Molecular diagnosis of diarrhea: current status and future potential. *Curr Infect Dis Rep* 2012; **14**: 41–6.

8 Platts-Mills JA, Liu J, Gratz J, *et al.* Detection of Campylobacter in stool and determination of significance by culture, enzyme immunoassay, and PCR in developing countries. *J Clin Microbiol* 2014; **52**: 1074–80.

9 Ochoa TJ, Barletta F, Contreras C, Mercado E. New insights into the epidemiology of enteropathogenic Escherichia coli infection. *Trans R Soc Trop Med Hyg* 2008; **102**: 852–6.

10 World Health Organization. Global Health Observatory data repository: Cholera. 2016. http://apps.who.int/gho/data/node.main.174?lang=en (accessed Aug 25, 2016).

11 Lamberti LM, Ashraf S, Walker CLF, Black RE. A Systematic Review of the Effect of Rotavirus Vaccination on Diarrhea Outcomes Among Children Younger Than 5 Years. *Pediatr Infect Dis J* 2016; **35**: 992–8.

Diphtheria

Model flowchart

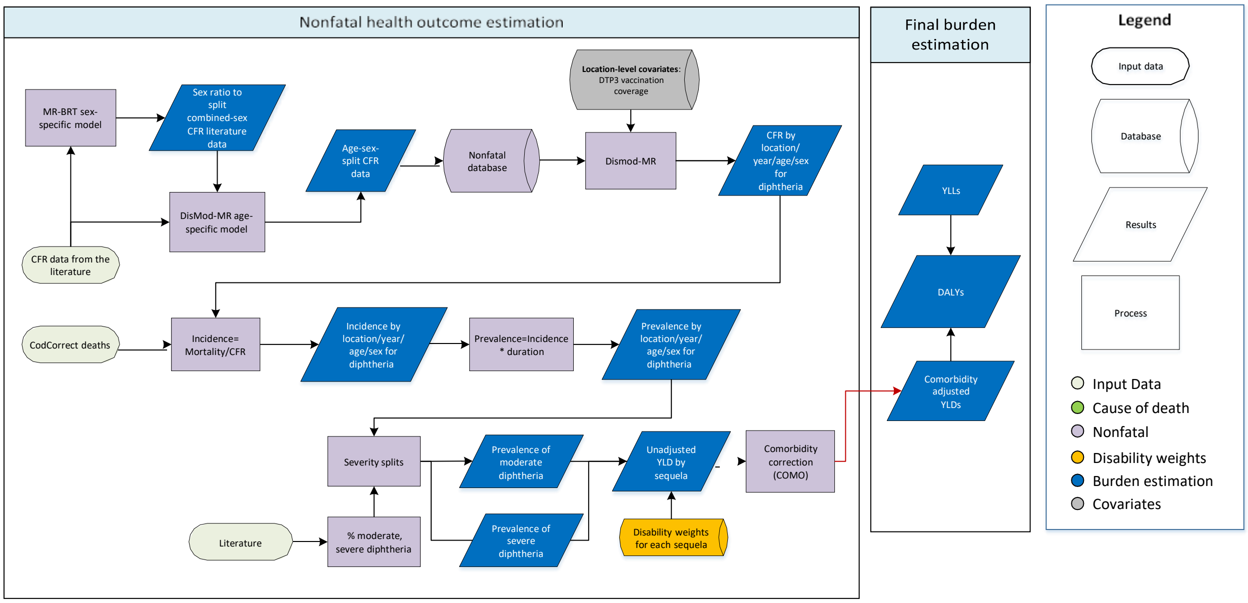

Case definition

Diphtheria is a disease of the respiratory tract caused by the bacterial pathogen Corynebacterium diphtheriae. Typical manifestations include fever, purulent discharge, and pseudomembrane formation along the respiratory tree, primarily the upper track. Toxigenic strains of C. diphtheriae may lead to cardiac and neurological disease. . For diphtheria, ICD-10 codes are A36- A36.9, Z22.2, Z23.6, and ICD-9 codes are 032-032.9, V02.4, V03.5, and V74.3.

Diphtheria

**Quantity of interest**	**Reference or Alternative**	**Definition**
Diphtheria case fatality rate	Reference	Ratio of fatal cases of diphtheria over total confirmed cases of diphtheria in the sample

Input data

Model inputs

The non-fatal diphtheria model has two primary inputs. The first is literature data obtained from systematic reviews of diphtheria case fatality ratio (CFR). The second is GBD mortality estimates of diphtheria, calculated per country by either Cause of Death Ensemble modelling (CODEm) or a negative binomial regression modelling method.

The diphtheria CFR systematic review was most recently updated in GBD 2019. New data were added to existing sources from earlier GBD cycles' systematic reviews, conducted approximately every three years. For GBD 2019, the search terms used in PubMed were: *((((diphtheria[MeSH Terms] OR diphtheria) AND (mortality[MeSH Terms] OR mortality OR "case fatality rate" OR "case fatality ratio" OR "case fatality"))) AND ("2016"[Date - Publication] : "2019"[Date - Publication]))*. Data were excluded if they were missing information about diphtheria cases and deaths or referred to diphtheria outbreaks in camps of refugees, internally displaced people, or ethnic minority groups. Table 1 summarises the literature-extracted non-fatal input data used in the diphtheria model.

Table 1: Data Inputs for diphtheria morbidity modelling by parameter

	**Countries with data**	**New sources**	**Total sources**
Incidence	0	0	0
Prevalence	0	0	0
Remission	0	0	0
Other	22	1	31

Input data processing

All extracted diphtheria CFR data that were not sex- and age-specific (ie, the data that were reflective of both sexes combined and/or age ranges greater than 20 years) were split into sex- and age-specific groups prior to use in modelling. Scant age- and sex-specific diphtheria CFR data are currently available, which precludes the estimation of location- or year-specific age and sex patterns. Instead, a global sex ratio and age pattern were generated using all available literature and clinical sex- and age-specific diphtheria CFR data. This pattern and ratio were then used to split all non-age- or sex-specific CFR data prior to inclusion in the final CFR model while propagating uncertainty from the splitting process. In GBD 2021, we switched from modelling the ratio of CFR in males to CFR in females to modelling the ratio of CFR in females to CFR in males to align with standard GBD sex-splitting practices.

The ratio used to make the sex splits was calculated using MR-BRT, a Bayesian meta-regression tool, and updated for GBD 2021. For studies that included CFR data that were separately age- and sex-specific, the within-study sex ratio, rather than the global sex ratio, was used to split the age-specific data. Few diphtheria CFR data sources matching inclusion criteria had sufficient, paired sex information to create a standard male to female ratio. To supplement these sources, paired, sex-specific, non-zero CFRs from hospital claims data from the Philippines and nine Brazil states were used only during generation of the ratio. The female/male sex adjustment factor calculated for use in GBD 2021 modelling was 0.849. The male/female adjustment factor that was calculated during modelling in GBD 2019 was 1.31 (0.88 to 1.99), equivalent to a mean female/male ratio of 0.76. The MR-BRT sex-ratio model with 10% trimming was updated in GBD 2021 to ensure agreement in outliers between the sex-ratio model and the DisMod CFR model. The resulting sex-ratio model suggests smaller differences in diphtheria CFR between males and females than were estimated in GBD 2019.

Table 2: MR-BRT sex-splitting adjustment factor for diphtheria CFR

Data input	Reference or alternative case definition	Beta coefficient, log (95% CI)	Adjustment factor*
Sex (female/male)	N/A	-0.164 (-0.455 to 0.128)	0.849

**MR-BRT crosswalk adjustments can be interpreted as the factor the alternative case definition is adjusted by to reflect what it would have been had it been measured using the reference case definition. If the log/logit beta coefficient is negative, then the alternative is adjusted up to the reference. If the log/logit beta coefficient is positive, then the alternative is adjusted down to the reference.*
***The adjustment factor column is the exponentiated beta coefficient. For log beta coefficients, this is the relative rate between the two case definitions. For logit beta coefficients, this is the relative odds between the two case definitions.*

For diphtheria CFR data representing an age range wider than 20 years, the extracted CFR values were split proportionally to follow a global age pattern generated from available age-specific diphtheria CFR data. To generate this global age pattern, diphtheria CFR data representing age groups less than 20 years in width were used to fit a DisMod-MR model with the GBD Healthcare Access and Quality (HAQ) Index as a location-level covariate. Then, the final global age pattern output – produced by DisMod for each sex for ages from early neonatal to 95+ years and updated to include the new GBD 2021 under-5 age groups – was used to split the death counts in the remaining data sources.

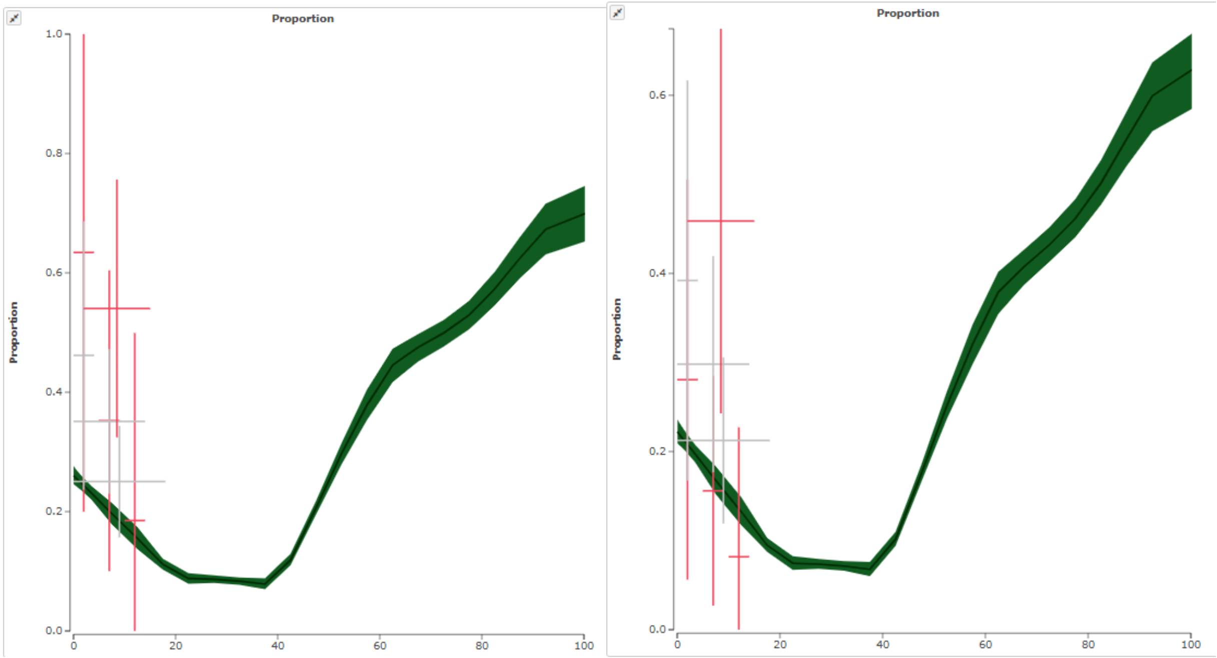

Figure 1. CFR Global age pattern for diphtheria CFR (L: male, R: female)

Modelling strategy

We used DisMod-MR to produce location-, year-, age-, and sex-specific diphtheria CFR estimates from our available sex- and age-specific input data. In the model, we used the HAQ Index as a location-level covariate, enforcing a directional prior so locations with higher HAQ Index are predicted to have lower CFR.

Table 4 displays the raw and exponentiated magnitudes of covariate influence, which can be interpreted as odds ratios. The change in input data outliering drives differences in covariate influence when compared to GBD 2019, which in turn drives differences in CFR estimates. Specifically, we estimate lower regional CFR in sub-Saharan Africa and south Asia and higher regional CFR in Latin America and the Caribbean when compared to GBD 2019.

Incidence was calculated as mortality rate divided by case fatality ratio. The diphtheria mortality rate was produced in GBD 2021, modelled using CODEm or a negative binomial regression and data from the cause of death database with the five-year rolling mean DTP3 coverage covariate, age dummy variables, and HAQ Index as key predictors (see diphtheria in cause of death appendix). Then, prevalence was calculated as the product of incidence and diphtheria case duration (mean of 27.5 days, based on a meta-analysis of duration data from the literature). For all countries, we produced estimates for all age groups between post-neonatal and 59 years. These calculations were completed in 1000-draw space to encompass and propagate uncertainty throughout the modelling process. Draw-level estimates were then summarised as means of draws and 95% uncertainty intervals (2.5th and 97.5th percentiles of all draws).

Severity split and disability weights

Our estimated, non-fatal diphtheria cases are split by severity following distributions summarised from literature reviews. 70% (95% CI: 66.5–73.5) of cases are presumed moderate, and the remaining 30% (95% CI: 26.5–33.5) severe. Table 3 provides severity level descriptions in addition to these weights.

Table 3. Severity distribution, details on the severity levels for diphtheria in GBD 2021 and the associated disability weight (DW) with that severity

Severity level	Lay description	DW (95% CI)
Moderate diphtheria	Has a fever and aches, and feels weak, which causes some difficulty with daily activities.	0.051 (0.032–0.074)
Severe diphtheria	Has a high fever and pain, and feels very weak, which causes great difficulty with daily activities.	0.133 (0.088–0.19)

Table 4. Covariates. Summary of covariates used in the diphtheria CFR DisMod-MR meta-regression model

Covariate	Type	Parameter	Exponentiated beta (95% uncertainty interval)
Healthcare Access and Quality (HAQ) Index	Country-level	Case fatality ratio	0.95 (0.86–1.00)

We made no additional substantive changes in the modelling strategy from GBD 2019.

Dysthymia

Flowchart

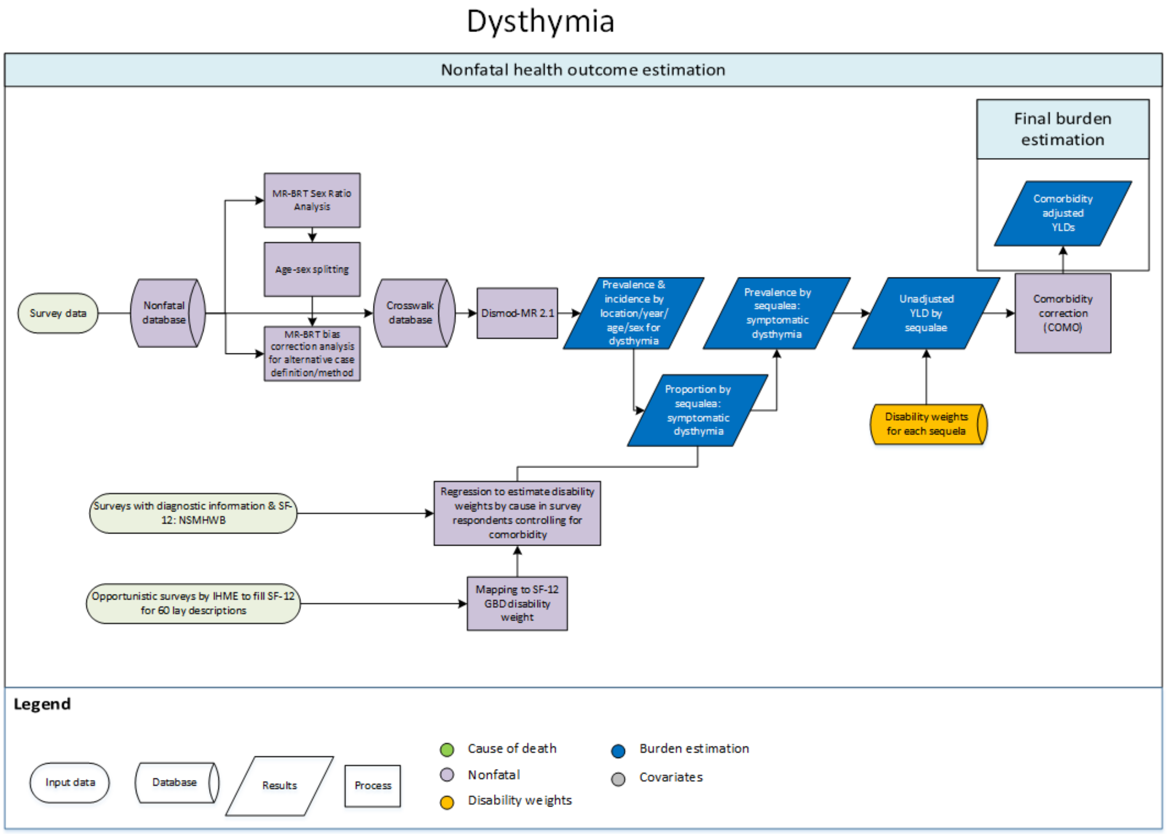

Input data and methodological summary for dysthymia

Case definition

Dysthymia is a mood disorder consisting of chronic depression, demonstrating less severe but longer-lasting symptoms than major depressive disorder. Included in GBD disease modelling were cases meeting diagnostic criteria for dysthymia according to the Diagnostic and Statistical Manual of Mental Disorders (DSM), or the equivalent diagnosis in the International Classification of Diseases (ICD).[1,2] These were identified by the following codes: DSM-IV-TR: 300.4, ICD-10: F34.1; excluding those cases due to a general medical condition or substance-induced cases.[1,2] Different versions of DSM (DSM-III, DSM-III-R, DSM-IV, DSM-IV-TR, DSM-5, and DSM-5-TR) and ICD (ICD-9, ICD-10, and ICD-11) were accepted.

According to DSM-IV TR criteria, dysthymia involves the experience of chronically depressed mood for most of the day, more days than not, for at least two years (or at least one year in children and adolescents). During this period, at least two of the following symptoms must also be experienced:

- poor appetite or overeating
- insomnia or hypersomnia
- low energy or fatigue
- low self-esteem

- poor concentration or indecisiveness
- feelings of hopelessness

Input data

The epidemiological systematic literature review for dysthymia was conducted in three stages involving electronic searches of the peer-reviewed literature (ie, via PsycInfo, Embase, and PubMed), the grey literature, and expert consultation. For mental disorders, we update our GBD electronic database searches on a rolling basis. An electronic search was not required for GBD 2021. The next update will be conducted in the next round of GBD. The grey literature searches and expert consultation were conducted for GBD 2021.

The GBD inclusion criteria stipulated that 1) the publication year must be from 1980 onward; 2) "caseness" must be based on clinical threshold as established by the DSM or ICD; 3) sufficient information must be provided on study method and sample characteristics to assess the quality of the study; and 4) study samples must be representative of the general population (ie, inpatient or pharmacological treatment samples, case studies, veterans, or refugee samples were excluded). No limitation was set on the language of publication. Methods used for this systematic review have been reported in greater detail elsewhere.[3,4] Table 1 summarises data inputs by parameter for dysthymia.

Table 1: Data Inputs for dysthymia morbidity modelling by parameter

Parameter	Countries with data	New sources	Total sources
Incidence	1	1	2
Prevalence	37	1	105
Remission	2	0	2
Other	1	0	1

Age-sex splitting

The extracted data underwent three types of age-sex splitting processes:

13. Where possible, estimates were further split by sex and age based on the available data. For instance, if studies reported prevalence for broad age groups by sex (eg, prevalence in 15–65-year-old males and females separately), and also by specific age groups but for both sexes combined (eg, prevalence in 15–30-year-olds, then in 31–65-year-olds, for males and females combined); age-specific estimates were split by sex using the reported sex-ratio and bounds of uncertainty.
14. A meta-regression—Bayesian, regularised, trimmed (MR-BRT) analysis was used to split the remaining both-sex estimates in the dataset. For each parameter, sex-specific estimates were matched by location, mid-age, and year. A MR-BRT network meta-analysis was then used to estimate pooled sex ratios. Given evidence to suggest that the sex-ratio in depressive disorders varies with age,[5-7] we also tested for an age interaction in the model. We found that the sex difference in dysthymia decreased significantly with age ie, prevalence in males (compared to females) increased significantly with increasing age. The global sex-ratio (at the mean mid-age of data informing the sex-ratio model) was estimated as 0.62 (95% uncertainty interval [UI]: 0.49–0.84) while Figure 1 shows the estimated male-to-female prevalence ratio by age. Age-specific sex ratios were used to split both-sex estimates in the dataset.

Figure 1. Sex ratios by age for dysthymia

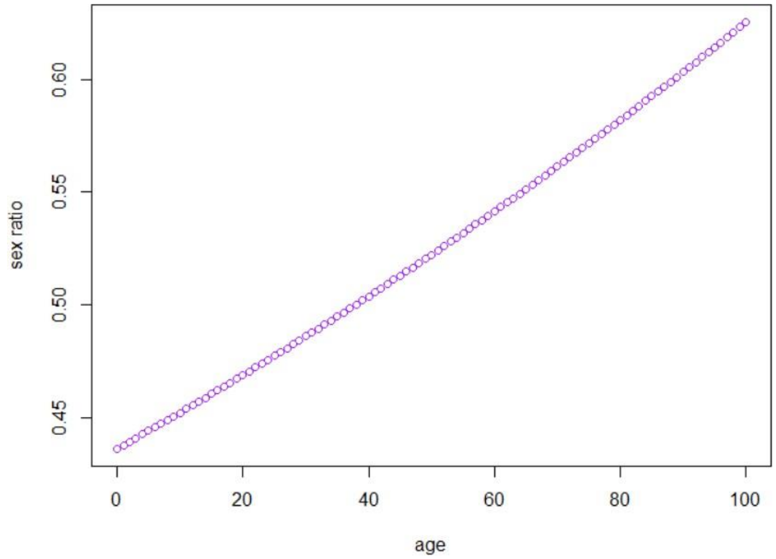

15. Studies reporting prevalence estimates across age groups spanning 25 years or more were split into five-year age groups using the prevalence age pattern estimated by DisMod-MR 2.1. The DisMod-MR model used to estimate the age pattern did not contain any previously age split data.

Bias corrections/crosswalks

Estimates with known biases were adjusted/crosswalked accordingly prior to DisMod-MR 2.1. For each crosswalk of interest, pairs of the reference and the alternative estimates were matched by age, sex, location, and year. This was done for both within-study (where possible) and between-study pairs. These pairs were then used as inputs in a MR-BRT network meta-analysis. The MR-BRT analysis produced a pooled ratio between the reference estimates and alternative estimates, which was used to adjust all alternative estimates in the dataset. For dysthymia a lay-interviewer ratio (see Table 2) was used to adjust all prevalence estimates derived from trained lay-interviewers towards the level they would have been if the estimate was derived from clinically trained interviewers (ie, psychologist or psychiatrist). We consider interviews conducted by clinicians to be more sensitive to detecting cases of dysthymia, particularly in locations where predominantly westernised mental health case definitions and instruments are yet to be fully validated. The estimated UIs around the adjustment ratio incorporate Gamma which represents the between study variance across all input data in the model. This added uncertainty widens the UIs for crosswalks with significant fixed effects.

Table 2: MR-BRT crosswalk adjustment factors for dysthymia

**Data input**	**Reference or alternative case definition**	**Gamma**	**Beta Coefficient, Log (95% UI)***	**Adjustment factor****
Population survey	Reference: clinical diagnosis	0.43		

Population survey	Alternative: lay-interviewer diagnosis		-0.21 (-1.07–0.62)	0.81 (0.34–1.86)

**MR-BRT crosswalk adjustments can be interpreted as the factor the alternative case definition is adjusted by to reflect what it would have been had it been measured using the reference case definition. If the log/logit beta coefficient is negative, then the alternative is adjusted up to the reference. If the log/logit beta coefficient is positive, then the alternative is adjusted down to the reference.*
***The adjustment factor column is the exponentiated beta coefficient. For log beta coefficients, this is the relative rate between the two case definitions. For logit beta coefficients, this is the relative odds between the two case definitions.*

Modelling Strategy

We have made no substantive changes in the modelling strategy from GBD 2019.

After the above data processes were applied, DisMod MR 2.1 was used to model the epidemiological data for dysthymia. Adjustments to model priors or the dataset were made where appropriate. Where outliers were identified in the data, we reassessed the study's methodology and quality before a decision was made to exclude or include the data.

Data across all epidemiological parameters were initially included in the modelling process. The incidence studies reported estimates which were very low relative to the prevalence data. As prevalence studies contributed much greater world coverage than incidence studies, we excluded the incidence data, relying instead on data from the other parameters. We assumed no incidence and prevalence before age 3. This minimum age of onset was corroborated with expert feedback and was consistent with the available data. Excess-mortality was set to 0 as there is no epidemiological evidence to suggest that dysthymia is associated with a statistically significant risk of mortality.[3,4]

Severity splits and disability weights

The GBD disability weight survey assessments include lay descriptions of sequelae highlighting major functional consequences and symptoms. The lay description and disability weight for a symptomatic state of dysthymia is shown in Table 3. Given the milder and more stable presentation of dysthymia, it was assigned the same disability weight as that for mild major depressive disorder. To determine the proportion of people with symptomatic and asymptomatic dysthymia, the US National Epidemiological Survey on Alcohol and Related Conditions (NESARC, conducted in two waves from 2001–2002 and 2004–2005)[8] and the Australian National Survey of Mental Health and Wellbeing of Adults (NSMHWB, conducted in 1997)[9] were used. The proportion of dysthymia cases falling within each severity level were as follows: asymptomatic 29% (23%–36%), and symptomatic 71% (64%–77%).

Table 3. Lay description for dysthymia in GBD 2021 and the associated disability weight

**Severity level**	**Lay description**	**Disability weight (95% UI)**
Symptomatic dysthymia	Feels persistent sadness and has lost interest in usual activities. The person sometimes sleeps badly, feels tired, or has trouble concentrating but still manages to function in daily life with extra effort.	0.145 (0.099–0.209)

There were no significant changes in GBD 2021 results for dysthymia compared to GBD 2019. While we continue to improve on the data and methods used to estimate the burden of mental disorders, some challenges need to be acknowledged. Firstly, we still have a large number of locations with no high quality raw data available. Secondly, it is difficult to quantify and remove all variation due to measurement error in our epidemiological estimates. While we have improved the methodology used to account for known sources of bias, in some cases, we still have very few datapoints to inform these adjustments. Thirdly, there is a paucity of research on the risk factors of mental disorders which can be used as predictive covariates in our epidemiological models.

References

1. American Psychiatric Association. Diagnostic and Statistical Manual of Mental Disorders (DSM). Washington: American Psychiatric Association, 1952.

2. World Health Organization. The ICD-10 Classification of Mental and Behavioural Disorders. Clinical descriptions and diagnostic guidelines. Geneva: World Health Organization; 1992.

3. Charlson FJ, Ferrari AJ, Flaxman AD, Whiteford HA. The epidemiological modelling of dysthymia: application for the Global Burden of Disease Study 2010. *J Affect Disord* 2013; **151**(1): 111-20.

4. Ferrari AJ, Charlson FJ, Norman RE, et al. Burden of depressive disorders by country, sex, age, and year: findings from the global burden of disease study 2010. *PLoS Med* 2013; **10**(11): e1001547.

5. Patten SB, Wang JL, Williams JV, et al. Descriptive epidemiology of major depression in Canada. *The Canadian Journal of Psychiatry* 2006; **51**(2): 84-90.

6. Patten SB, Williams JV, Lavorato DH, Wang JL, Bulloch AG, Sajobi T. The association between major depression prevalence and sex becomes weaker with age. *Social psychiatry and psychiatric epidemiology* 2016; **51**(2): 203-10.

7. Salk RH, Hyde JS, Abramson LY. Gender differences in depression in representative national samples: meta-analyses of diagnoses and symptoms. *Psychological bulletin* 2017; **143**(8): 783.

8. Introduction to the National Epidemiologic Survey on Alcohol and Related Conditions [http://pubs.niaaa.nih.gov/publications/arh29-2/74-78.htm]. Access date 1 December 2014.

9. Australian Bureau of Statistics. National Survey of Mental Health and Wellbeing of Adults 1997. Canberra: Australian Bureau of Statistics.

Ebola virus disease

Flowchart

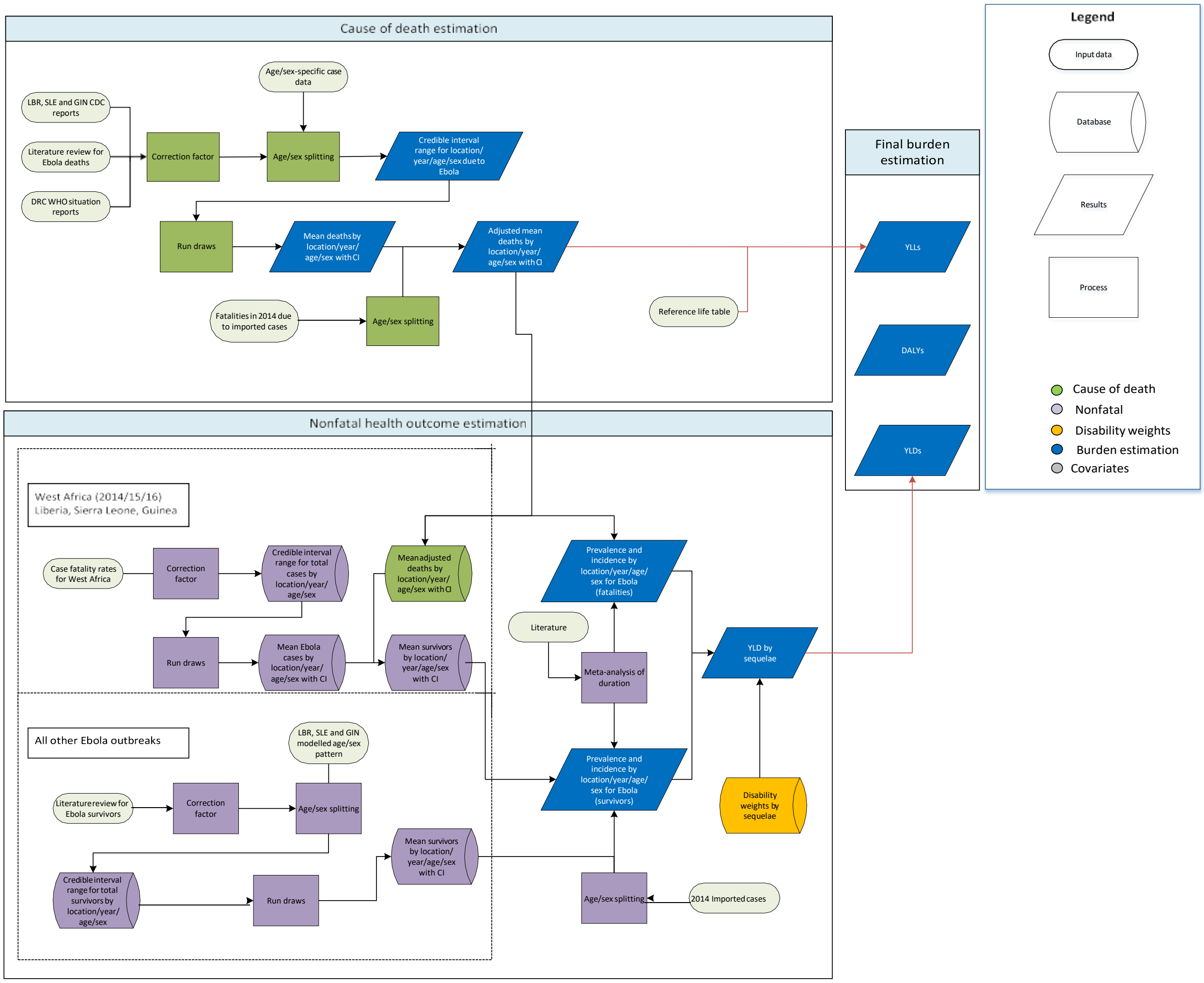

Input data and methodological summary for Ebola virus disease

Background and case definition

Ebola virus is a relatively rare viral pathogen linked with high case-fatality rates in both humans and non-human primates. The disease is zoonotic, and while bats have been implicated as reservoirs, definitive host species are yet to be identified. Once a human becomes infected after viral transmission from animal sources either directly or indirectly, secondary human-to-human transmission is possible, primarily through exchange of infectious bodily fluids and secretions. Clinical cases typically present initially as a febrile illness, similar to a number of different pathogens, which can subsequently be followed by haemorrhagic complications and death. Historically, there have been a number of outbreaks, usually no more than a few hundred cases, typically constrained to one country, focused in central Africa. The West African outbreak, however, which started in Guinea in 2013, claimed more lives than all previous outbreaks combined and spread across the region seeding additional outbreaks. The ICD code for Ebola is A98.4, but no data used in the modelling reference this code (ie, all the data are from literature extractions). Data for Ebola virus disease were only included if the case was identified as either “probable” or “confirmed” as per World Health Organization (WHO) definitions.

We used the following case definitions for GBD 2021:

Quantity of interest	Reference or Alternative	Definition
Ebola	Reference	Prevalence determined using cases identified as either “probable” or “confirmed” A confirmed case is any suspected or probable case with a positive laboratory result through either detection of virus RNA via reverse transcriptase-polymerase chain reaction, or by detection of IgM antibodies directed against Ebola. A probable case is any suspected case evaluated by a clinician or any deceased suspected case with an epidemiological link to a confirmed case.

Input data

Table 1: Source counts

Measure	Countries with data	New sources	Total sources
All measures	16	8	52
Causes of death	11	8	37
Duration	0	0	6
Continuous	0	0	1
Population	16	3	38

Model inputs

Two distinct sequelae were assigned to Ebola virus disease (EVD) to be incorporated into the YLD estimation process: (i) sequela associated with the initial symptomatic phase of the infection (associated with all cases of EVD) and (ii) sequela characterising the long-term post-EVD consequences of infection. As such, data were required to ascertain both the number of deaths as well as those surviving from each outbreak.

Data on fatal cases inherited from the GBD 2017 mortality estimation process were converted into incidence of cases of Ebola (with fatal outcomes) by cross-referencing locational annualised population estimates.

In order to calculate the numbers of survivors from each outbreak, two data sources were referenced, one based upon modelled estimates of the main three countries in the West African Ebola outbreak (namely Sierra Leone, Liberia, and Guinea), supplemented by WHO Situation Reports covering the clusters of 2016 cases and literature references covering all other subsequent outbreaks.

Age-sex patterns derived from the age- and sex-specific input data were applied to total envelope estimates as reported by WHO and Centers for Disease Control and Prevention (CDC). Raw number of survivors were estimated by subtracting total deaths from total cases.

For all other outbreaks, numbers of survivors were directly evaluated based upon numbers published in a previous review[1,2] and consulting original documents describing these outbreaks. This initial review was also updated to include the outbreaks that occurred in the Democratic Republic of the Congo (DRC) in

2014[3], cases in 2016 and 2017, the 2018 DRC Equateur Province outbreak[4], the 2018-2020 DRC Ituri, North Kivu and South Kivu Provinces outbreak[5,6,7], including cases in Uganda[8], the outbreak in 2020 in the DRC Equateur Province[9], and the outbreaks in 2021 in Guinea and DRC North Kivu Province[10]. This resulted in datasets describing each outbreak with variable degrees of detail: some fully describing the age and sex breakdown of all survivors [e.g. Rosello et al.[11]] and others simply providing the final total. Only confirmed or probable cases were included as per the case definition. Outbreaks that spanned multiple years, in the absence of sufficient data providing an accurate breakdown, were apportioned between the years by evenly assigning a uniform number of survivors to each month of the outbreak's duration. An additional search was conducted to identify imported cases from the West African outbreak during 2014 and 2015.

Modelling strategy

Data on cases (both survivors and fatalities) resulting from imported cases from 2014 and 2015 were used as specific count data as it was assumed to be an accurate representation of the cases and outbreaks in these countries, all of which were on high alert for importation of cases.[14,15]

All other input data were processed prior to inclusion in GBD to account for any potential under-reporting of deaths. A meta-analysis of existing under-reporting studies from the literature was performed using a random effects model with a DerSimonian-Laird estimator. A variety of sources were included, capturing a number of different estimation processes, all identified by literature review. The figure below shows the different effect sizes of the different studies, as well as the resulting GBD 2016 (used in GBD 2021) correction factor, with the GBD 2015 correction factor for reference. The correction factor ranged from 1.5147 to 2.5720 with a mean of 2.0433.

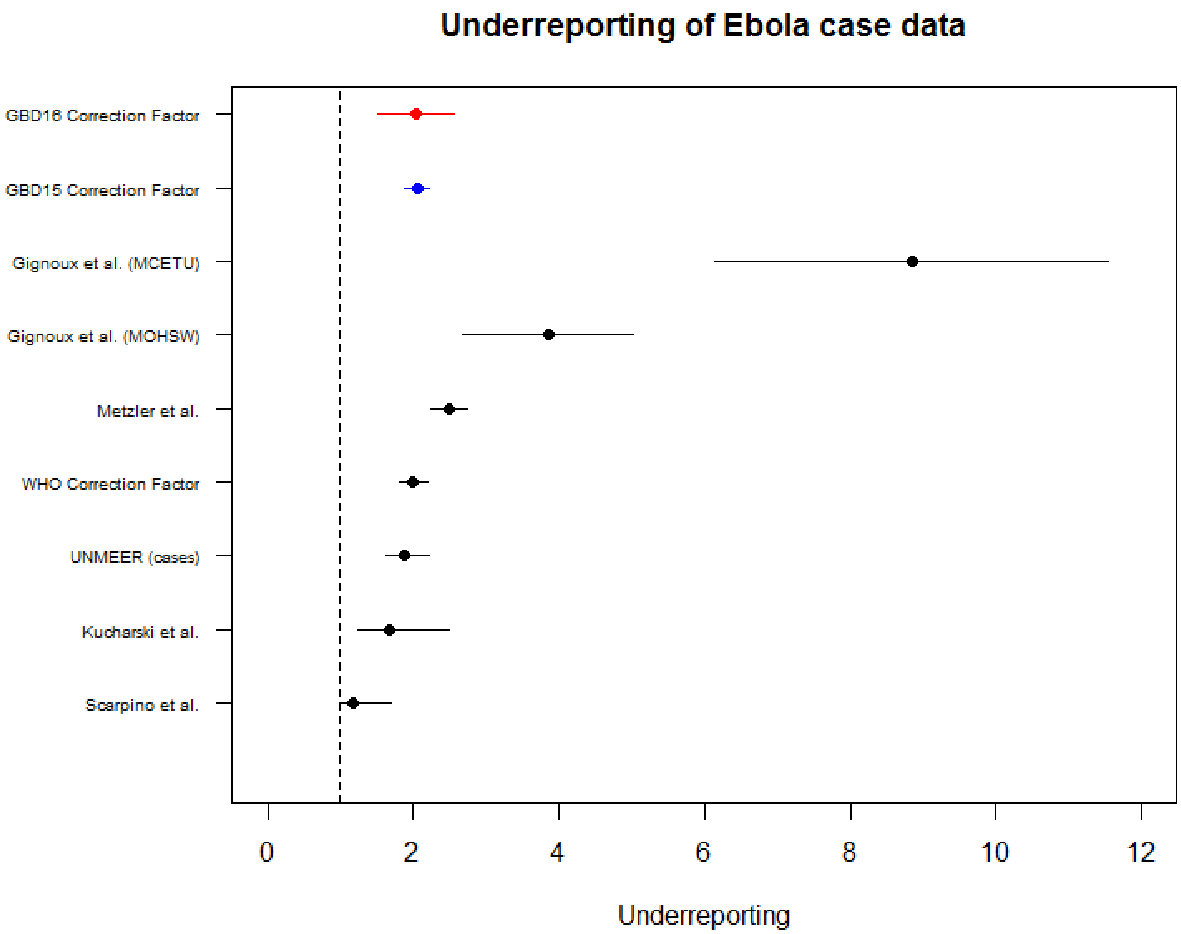

In order to capture this potential variation, all input data were multiplied by the lower and upper limit of this estimated correction factor; these numbers then provided the lower and upper bounds from which draw values were taken. For outbreaks where no data were supplied for age and/or sex, the pattern observed in the West African outbreak (for which there were the most comprehensive data) was used to apportion these total values.

1000 draws were taken from a normal distribution fitted between these lower and upper bound values, which generated mean estimates stratified by age, sex, location, and year along with credible intervals for these numbers. For the West African outbreak, this generated total case numbers, from which the estimated number of deaths was subtracted in order to provide an estimate for the total number of survivors. For all other outbreaks, this data processing directly estimated the total number of survivors from each outbreak. These count data were converted into prevalence estimates by cross-referencing estimates of population size.

In order to estimate the duration of the sequelae categories, previous modelled assessments of the West African outbreak were consulted.[1,2] The duration of initial infection for patients was calculated as the total time period between onset of symptoms to death or to discharge from hospital (8.2 days [7.9–8.4] and 15.1 days [14.6–15.6], respectively). These time periods were assumed to be appropriate for

characterising all other outbreaks. This time period was then assigned a disability weight corresponding to "infectious disease, acute episode, severe."

For long-term sequelae estimation, the proportion of survivors still suffering post-acute consequences was modelled using an exponential function with proportions of survivors still reporting poor health states (derived from a number of survivor studies[16-26]) reported over different time periods. The average duration of post-Ebola sequelae was then calculated as 0.9042 years (0.3673–1.4268).

The final combination of YLDs associated with prevalent initial onset of disease and prevalent post-EVD consequences was then calculated to provide an overall YLD estimate stratified by age, sex, location, and year. Estimates were provided for the years 1990, 1995, 2000, 2005, 2010, 2015, 2019, 2020, and 2021 as per non-fatal GBD estimation protocols.

Health states/sequelae

The table below shows the list of sequelae due to Ebola and the associated disability weights (DW). It was not possible to create bespoke disability weights for the more specific sequelae often associated with Ebola virus disease (eg, haemorrhaging or ocular complications in survivors), and thus existing disability weights were co-opted. General high fevers and weakness characterise the majority of presenting cases,[12] with long-term complications generally related to weakness and arthralgia.[13]

Table 2. Severity distribution, details on the severity levels for EVD and the associated disability weight (DW) with that severity

Sequelae	Description	DW (95% CI)
Infectious disease, acute episode, severe	Has a high fever and pain and feels very weak, which causes great difficulty with daily activities	0.133 (0.088–0.19)
Infectious disease, post-acute consequences (fatigue, emotional lability, insomnia)	Is always tired and easily upset. The person feels pain all over the body and is depressed	0.219 (0.148–0.308)

Changes from GBD 2019

There were no substantive changes implemented in GBD 2021. We did not apply any adjustments for the COVID pandemic to EVD due to a lack of available data quantifying the impacts of the pandemic on NTD epidemiology.

Limitations

Data on Ebola outbreaks prior to 2014 are sparse, and as a result many values derived from the West African outbreak were assumed to be valid for historical outbreaks as well. This may mask significant differences that exist between these outbreaks, some of which were caused by different species of Ebola virus. In order to minimise this problem, we chose to implement a data-driven approach – for those outbreaks where sufficiently detailed historical data could be obtained, this was used in preference to any assumed age/sex breakdown.

Haemorrhagic manifestations are currently not considered as an explicit health state for disability weighting, and as a result, the current classification (of infectious disease, acute episode, severe) may be an underestimate. In contrast, the post-Ebola disease sequelae disability weighting may overestimate this burden, particularly when applied over a long period of time. In both instances, however, these disability weightings represent the most relevant linkages in the absence of bespoke values being generated.

Due to so few historical survivors of Ebola virus disease, only a handful of studies have tracked the long-term sequelae among cohorts of survivors beyond a two-year period. Given the large number of survivors from the West African outbreak, it is likely that future parameterisation of this component will become much better data-driven. The current log-linear regression model extends for a period of 20 years and therefore could prove to be an overestimate of duration. In addition, ocular manifestations are not currently considered within the sequelae envelope – future iterations will consider health states identified by ongoing cohort analyses of Ebola survivors. Comments from collaborators in previous cycles have highlighted ocular conditions for inclusion; however, definitive evidence of a linkage with Ebola remains inconclusive. A study (conducted in West Africa) comparing Ebola survivors with background prevalence rates of many of the symptoms reported in survivors (eg, uveitis), suggested no difference in rates of these ophthalmic complications.[27] Understanding which of the many observed clinical outcomes in patients are caused by the virus, as opposed to incidentally co-morbid, is a necessary prerequisite for inclusion in the GBD.

References

1 Pigott DM, Golding N, Mylne A, *et al.* Mapping the zoonotic niche of Ebola virus disease in Africa. *Elife* 2014; **3**: e04395.

2 Mylne A, Brady OJ, Huang Z, *et al.* A comprehensive database of the geographic spread of past human Ebola outbreaks. *Sci Data* 2014; **1**: 140042.

3 Maganga GD, Kapetshi J, Berthet N, *et al.* Ebola virus disease in the Democratic Republic of Congo. *N Engl J Med* 2014; **371**: 2083–91.

4 World Health Organization (WHO). WHO Ebola Situation Report 2018 - Number 17. 2018.

5 World Health Organization (WHO). WHO Ebola Situation Report 2019- Number 69. 2019.

6 World Health Organization (WHO). WHO Ebola Situation Report 2019- Number 73. 2019.

7 World Health Organization (WHO). WHO Ebola Situation Report 2019- Number 98. 2020.

8 World Health Organization (WHO). WHO Ebola Situation Report 2019 - Number 45. 2019.

9 2020 Democratic Republic of the Congo, Equateur Province | Democratic Republic of Congo | Outbreaks | Ebola (Ebola Virus Disease) | CDC. 2020; published online Nov 19. https://www-cdc-gov.offcampus.lib.washington.edu/vhf/ebola/outbreaks/drc/2020-june.html (accessed January 11, 2021).

10 Centers for Disease Control and Prevention (CDC). Ebola (Ebola Virus Disease). 2021 Democratic Republic of the Congo, North Kivu Province & Guinea, N'Zérékoré prefecture. 2021. https://www.cdc.gov/vhf/ebola/history/chronology.html (accessed July 27, 2022).

11 Rosello A, Mossoko M, Flasche S, *et al.* Ebola virus disease in the Democratic Republic of the Congo, 1976-2014. *Elife* 2015; **4**. DOI:10.7554/eLife.09015.

12 Schieffelin JS, Shaffer JG, Goba A, *et al.* Clinical Illness and Outcomes in Patients with Ebola in Sierra Leone. *N Engl J Med* 2014; **371**: 2092–100.

13 Tiffany A, Vetter P, Mattia J, *et al.* Ebola Virus Disease Complications as Experienced by Survivors in Sierra Leone. *Clin Infect Dis* 2016; **62**: 1360–6.

14 Fasina FO, Shittu A, Lazarus D, *et al.* Transmission dynamics and control of Ebola virus disease outbreak in Nigeria, July to September 2014. *Euro Surveill* 2014; **19**: 20920.

15 Althaus CL, Low N, Musa EO, Shuaib F, Gsteiger S. Ebola virus disease outbreak in Nigeria: Transmission dynamics and rapid control. *Epidemics* 2015; **11**: 80–4.

16 UNMEER. Sierra Leone: Ebola emergency Weekly Situation Report No. 7. 2014 https://www.humanitarianresponse.info/system/files/documents/files/UNMEER_NERC_SitRep_07Dec.pdf.

17 Clark D V, Kibuuka H, Millard M, *et al.* Long-term sequelae after Ebola virus disease in Bundibugyo, Uganda: a retrospective cohort study. *Lancet Infect Dis* 2015; **15**: 905–12.

18 Qureshi AI, Chughtai M, Loua TO, *et al.* Study of Ebola Virus Disease Survivors in Guinea. *Clin Infect Dis* 2015; **61**: 1035–42.

19 Rowe AK, Bertolli J, Khan AS, *et al.* Clinical, virologic, and immunologic follow-up of convalescent Ebola hemorrhagic fever patients and their household contacts, Kikwit, Democratic Republic of the Congo. Commission de Lutte contre les Epidémies à Kikwit. *J Infect Dis* 1999; **179 Suppl**: S28-35.

20 Bwaka MA, Bonnet MJ, Calain P, *et al.* Ebola hemorrhagic fever in Kikwit, Democratic Republic of the Congo: clinical observations in 103 patients. *J Infect Dis* 1999; **179 Suppl**: S1-7.

21 Mohammed H, Vandy AO, Stretch R, *et al.* Sequelae and Other Conditions in Ebola Virus Disease Survivors, Sierra Leone, 2015. *Emerg Infect Dis* 2017; **23**: 66–73.

22 Nanyonga M, Saidu J, Ramsay A, Shindo N, Bausch DG. Sequelae of Ebola Virus Disease, Kenema District, Sierra Leone. *Clin Infect Dis* 2016; **62**: 125–6.

23 Mattia JG, Vandy MJ, Chang JC, *et al.* Early clinical sequelae of Ebola virus disease in Sierra Leone: a cross-sectional study. *Lancet Infect Dis* 2016; **16**: 331–8.

24 Epstein L, Wong KK, Kallen AJ, Uyeki TM. Post-Ebola Signs and Symptoms in U.S. Survivors. *N Engl J Med* 2015; **373**: 2484–6.

25 Etard J-F, Sow MS, Leroy S, *et al.* Multidisciplinary assessment of post-Ebola sequelae in Guinea (Postebogui): an observational cohort study. *Lancet Infect Dis* 2017. DOI:10.1016/S1473-3099(16)30516-3.

26 Scott JT, Sesay FR, Massaquoi TA, Idriss BR, Sahr F, Semple MG. Post-Ebola Syndrome, Sierra Leone. *Emerg Infect Dis* 2016; **22**: 641–6.

27 Steptoe, PJ, Scott JT, Baxter, JM, *et al.* Novel retinal lesion in Ebola survivors, Sierra Leone, 2016. *Emerg Infect Dis* 2017; **23**: 1102-9

Encephalitis

Flowchart

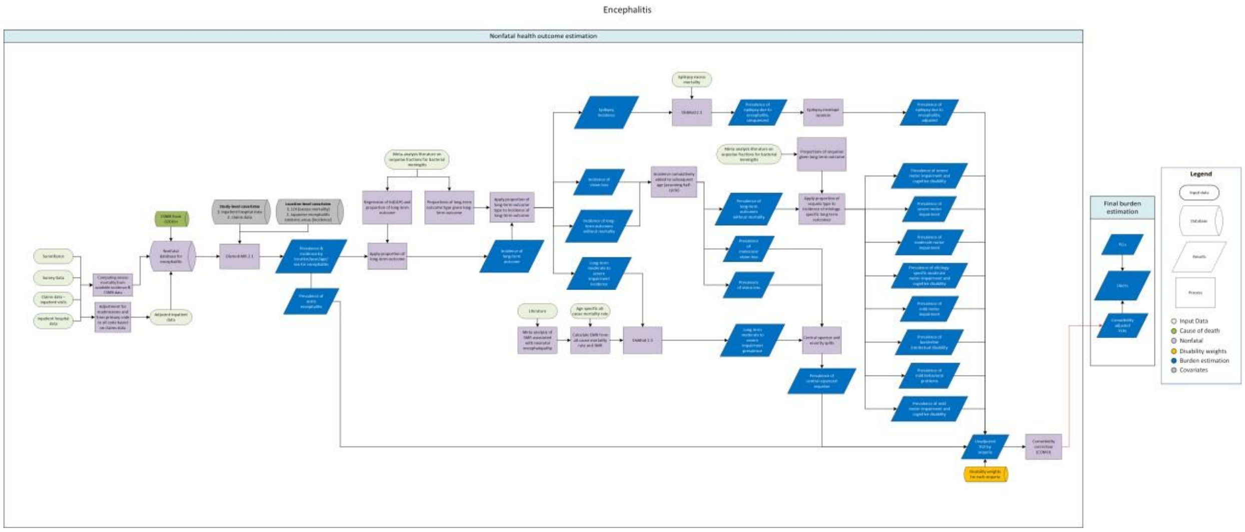

Case definition

Encephalitis is a disease caused by an acute inflammation of the brain. Symptoms of encephalitis can include flu-like symptoms like headache, fever, drowsiness, and fatigue, and at times, seizures, hallucinations, or stroke. Included in the GBD modelling were cases meeting ICD-10 diagnostic criteria for encephalitis (A83-A86.4, B94.1, F07.1, G04-G05.8).

The case definitions accepted for encephalitis are shown below.

**Quantity of interest**	**Reference or alternative**	**Definition**
Incidence of encephalitis	Reference	Encephalitis from inpatient data.
Incidence of encephalitis	Alternative	Encephalitis from USA private claims data.
Incidence of encephalitis	Alternative	Cases detected by epidemiological surveillance.

Input data

Model inputs

In the GBD 2021 study, a systematic review of literature was conducted to capture studies of incidence for encephalitis. These data sources included hospital data and literature. The inclusion criteria stipulated that: (1) the publication year must be between 1980 and 2020; (2) sufficient information must be provided on study method and sample characteristics to assess the quality of the study; and (3) study samples must be representative of the general population. No limitation was set on the language of publication.

We performed an updated systematic literature review for GBD 2021 to capture studies of incidence through the present year. The PubMed search terms were: ("encephalitis"[MeSH Terms] OR "encephalitis"[Title/Abstract] OR motor cognitive impairments[Title/Abstract]) AND

("incidence"[Title/Abstract] OR "incidence"[MeSH Terms]) AND (2019/07/01[Date – Publication] : 3000[Date – Publication]) NOT ("animals"[MeSH Terms] NOT "humans"[MeSH Terms])

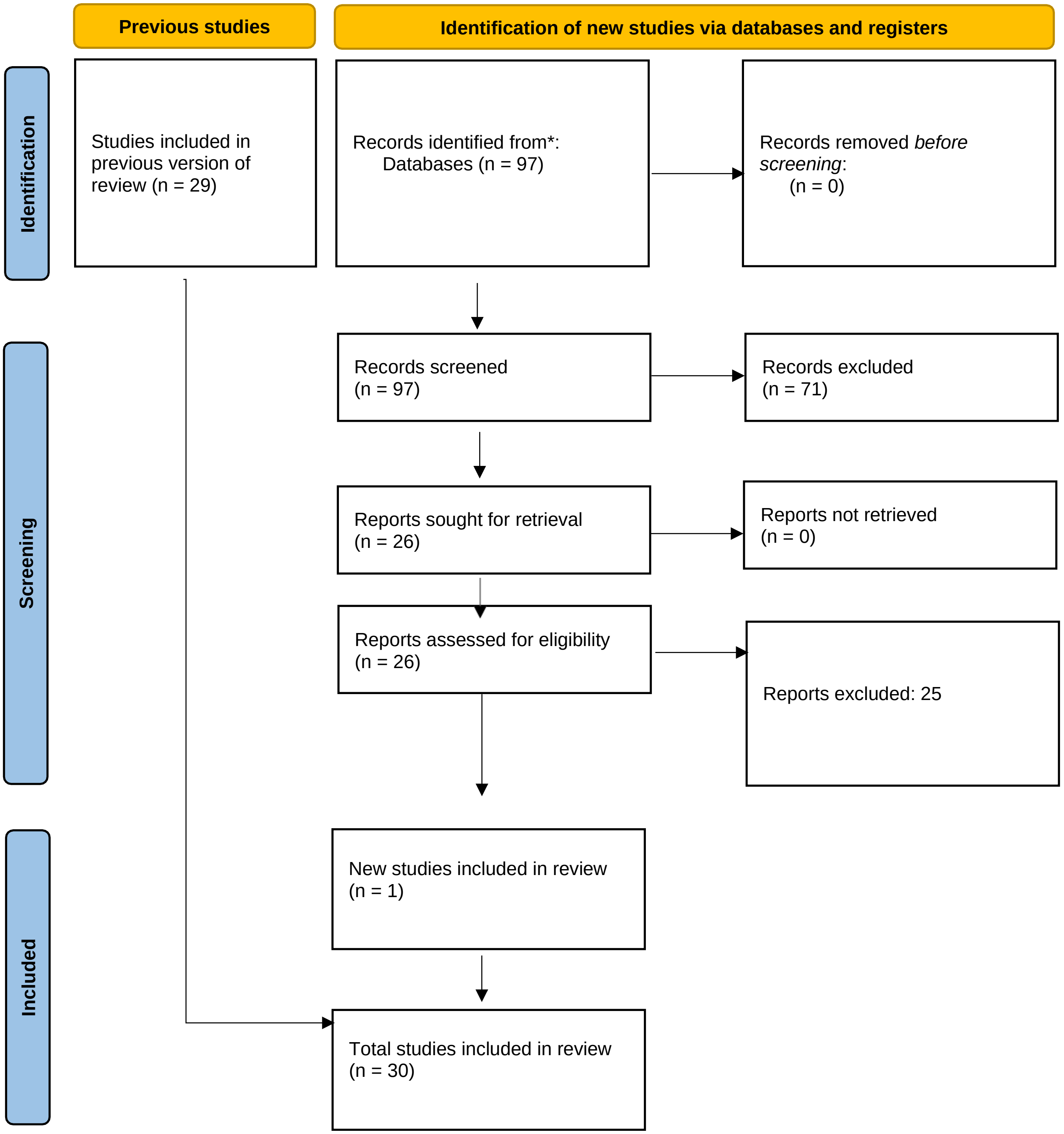

Figure 1 PRISMA diagram for encephalitis 2021 systematic review

Additional sources we included were inpatient hospital data and CF2 corrected inpatient claims data, primary diagnosis and inpatient only. A meta-analysis by Edmond and colleagues (1) informed sequelae and severity splits, while an internal meta-analysis informed mortality estimates for long-term moderate-to-severe impairments.

Table 1: Data inputs for encephalitis morbidity modelling by parameter.

	**Countries with data**	**New sources**	**Total sources**
Incidence	57	64	389
Prevalence	0	0	0
Remission	0	0	0
Other	0	0	1

Data were outliered or excluded if we found they differed significantly when compared to regional, super-regional, and global rates.

Bias corrections

Hospital data were flagged with a covariate for inpatient hospital data and were used as the reference category. Claims data were flagged with year-specific covariates. Surveillance data were flagged with covariates specific to the type of surveillance (eg, active versus passive and sentinel-based versus population-based). Both claims and surveillance data were crosswalked up to the reference category.

Table 2: MR-BRT crosswalk adjustment factors for encephalitis

Data input	Reference or alternative case definition	Gamma	Basis function on age midpoint	B-spline coefficient, logit (95% UI)*	Adjustment factor **
Inpatient hospital (CF2)	Ref		---	---	---
Claims, inpatient only	Alt	0.00	age_mid_0	2.54 (1.94 to 3.15)	12.70
			age_mid_1	2.83 (2.45 to 3.20)	16.83
			age_mid_2	–0.10 (–0.69 to 0.49)	0.90
			age_mid_3	1.50 (1.12 to 1.88)	4.50
			age_mid_4	1.12 (0.93 to 1.31)	3.07
Claims, inpatient only, year 2000	Alt	0.00	age_mid_0	1.65 (–3.67 to 6.98)	5.23
			age_mid_1	1.71 (–1.46 to 4.88)	5.54
			age_mid_2	0.36 (–4.06 to 4.78)	1.43
			age_mid_3	0.49 (–2.02 to 3.00)	1.63
			age_mid_4	1.01 (0.05 to 1.96)	2.73
Surveillance	Alt	0.77	---	–4.00 (–5.71 to –2.28)	0.02

**MR-BRT crosswalk adjustments can be interpreted as the factor the alternative case definition is adjusted by to reflect what it would have been had it been measured using the reference case definition. If the log/logit beta coefficient is negative, then the alternative is adjusted up to the reference. If the log/logit beta coefficient is positive, then the alternative is adjusted down to the reference.*

***The adjustment factor column is the exponentiated beta coefficient. For log beta coefficients, this is the relative rate between the two case definitions. For logit beta coefficients, this is the relative odds between the two case definitions.*

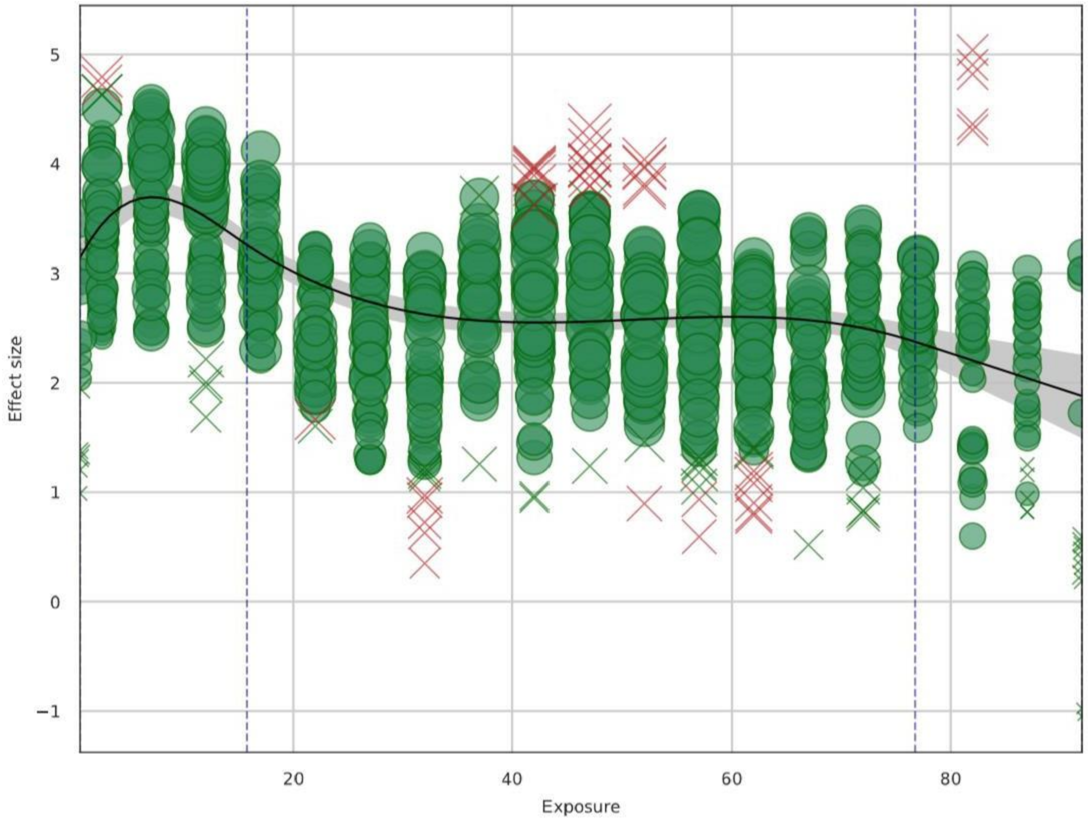

Figure 2a Cubic spline on age midpoint for MarketScan claims crosswalk (exposure is age midpoint, effect size is the adjustment factor in logit space)

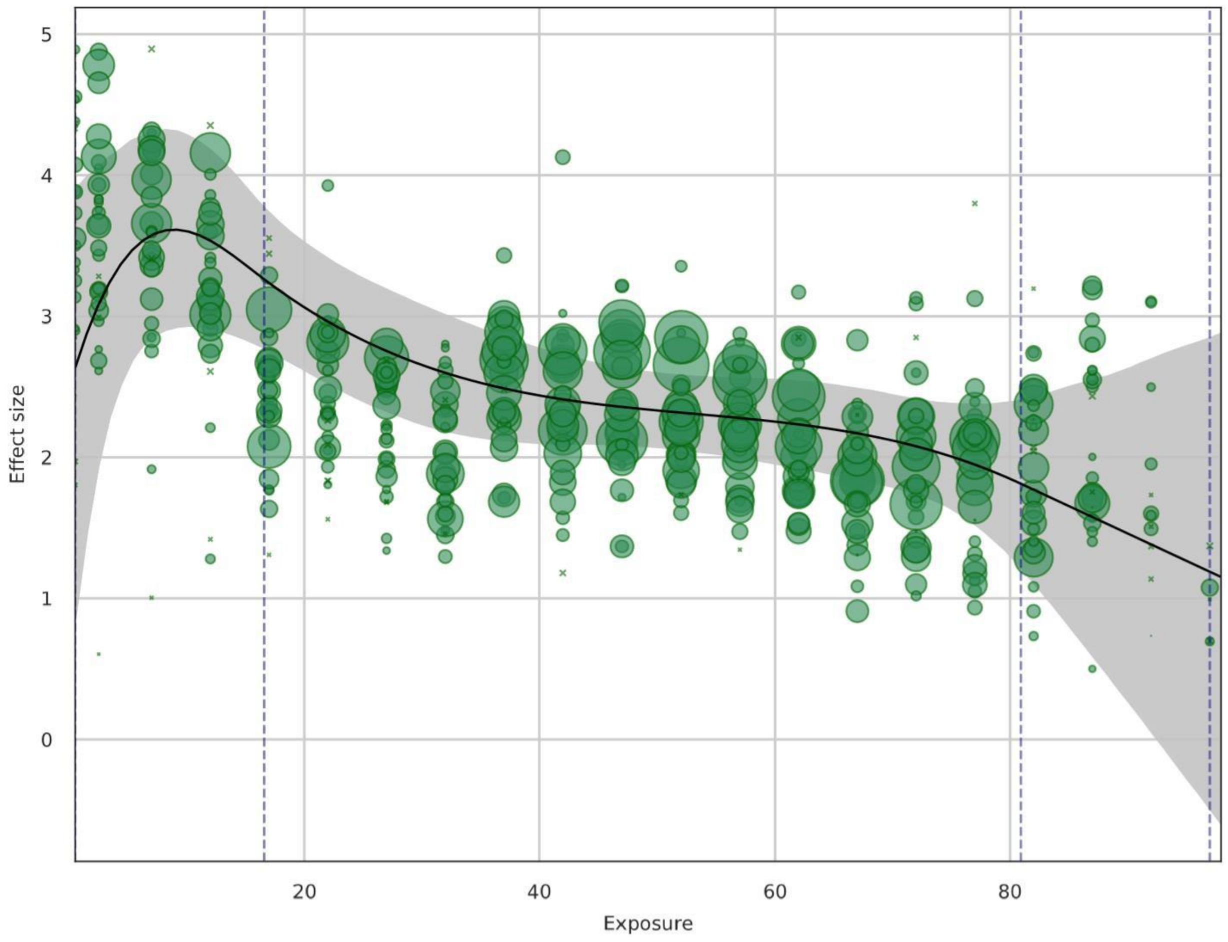

Figure 2b Cubic spline on age midpoint for MarketScan 2000 claims crosswalk (exposure is age midpoint, effect size is the adjustment factor in logit space)

Modelling strategy

Non-fatal outcomes were modelled using a combination of custom models and DisMod-MR 2.1. First, the overall incidence and prevalence of encephalitis were modelled to estimate the short-term morbidity due to acute infection. This DisMod model had a set duration (1/remission) of three weeks. We also imposed caps on excess mortality for ages 10–50. USA claims data were grouped into year-specific covariates based on quality and were crosswalked to the reference data, which we extracted from literature and inpatient hospital data. We used the function in DisMod-MR 2.1 to pull in cause-specific mortality rate (CSMR) data from our CODEm and CoDCorrect analyses and match with incidence datapoints for the same geography. We calculated excess mortality rate to estimate priors for EMR by dividing CSMR by prevalence, calculated from remission and incidence. To help inform trends where we lack data, we applied a binary country-level covariate at the subnational and country level that indicates if the location is in a Japanese encephalitis-endemic area (2). We also applied a lag-distributed income covariate to excess mortality. Betas and exponentiated values (which can be interpreted as an odds ratio)

are shown in the tables below for study-level covariates and country-level covariates. In GBD 2019, we updated the Japanese encephalitis covariate to include all Philippine subnationals and all Pakistan subnationals. We outliered incidence input datapoints with zero cases that were dragging down final estimates. We also improved our time efficiency and estimation accuracy by using an ordinary differential equations solver (ODE solver) in place of traditional DisMod-MR.

Table 3. Covariates. Summary of covariates used in the encephalitis DisMod-MR meta-regression model

Covariate	Type	Parameter	Exponentiated beta (95% uncertainty interval)
Japanese encephalitis endemic area	Country-level covariate	Incidence	1.10 (1.10–1.10)
LDI (log transformed)	Country-level covariate	Excess mortality	1.00 (1.00–1.00)

In addition to short-term sequelae as a result of acute encephalitis, we also modelled the long-term outcomes from encephalitis.

Sequelae splits

We first split the long-term sequelae among survivors of acute infection. We calculated the acute phase survivors by applying the excess mortality (calculated by the acute encephalitis DisMod model) to the incidence of each aetiology (excess mortality was converted to case fatality rate by $e^{(\text{-excess mortality x 1/(excess mortality + remission))}}$. The survivors were then subject to long-term sequelae by applying the post-discharge proportions of health consequences calculated by a meta-analysis by Edmond and colleagues (2). We calculated the ratio of acute encephalitis cases that result in a major long-term impairment, and the ratio of minor impairments to major impairments, based off a regression of log-transformed GDP and ratio values from Edmond and colleagues. This regression was done differently from last year when we used GNI. The regression is shown below:

$$y = -0.33590 \ln(GDP) + 1.15230$$

We assumed a similar pattern of health outcomes for encephalitis infection survivors as with other bacterial meningitis survivors (except hearing loss, as we could not find evidence of hearing loss as a consequence of encephalitis infection). We used these two ratios to calculate the proportions of survivors who contract a long-term minor impairment and those who contract a long-term major impairment. The proportion with major impairments were further split (again using pooled proportions from Edmond and colleagues) into specific major impairments, which were grouped into vision loss, moderate to severe cognitive impairments, and epilepsy.

The calculated incidence of long-term sequelae was then converted to prevalence by two different approaches. For the sequelae not associated with excess mortality, which were vision loss, intellectual disability, motor impairment, and behavioural problems, the incidence of each age was cumulatively added up to the subsequent age (assuming half-cycle) to construct prevalence at each age. If the sequela is associated with excess mortality (epilepsy and moderate-to-severe cognitive impairments), the calculated incidence was used as an input to the ODE solver, together with the corresponding mortality parameters (excess mortality data from the epilepsy envelope DisMod model, and standardised

mortality ratio data from a neonatal encephalopathy meta-analysis, converted to excess mortality using all-cause mortality estimates) to estimate the prevalence. Vision loss and epilepsy estimates were squeezed and severity split centrally.

Disability weights

The basis of the GBD disability weight survey assessments is lay descriptions of sequelae highlighting major functional consequences and symptoms. The lay descriptions and disability weights for sequelae associated with encephalitis are shown below.

Table 4. Severity distribution, details on the severity levels for encephalitis in GBD 2019 and the associated disability weight (DW) with that severity.

Severity split	Lay description	DW (95% CI)
Mild behaviour problems	This person is hyperactive and has difficulty concentrating, remembering things, and completing tasks.	0.045 (0.028–0.066)
Moderate motor impairment	This person has some difficulty in moving around, and difficulty in lifting and holding objects, dressing, and sitting upright, but is able to walk without help.	0.061 (0.040–0.089)
Moderate motor plus cognitive impairments	This person has some difficulty in moving around, holding objects, dressing, and sitting upright, but can walk without help. This person has low intelligence and is slow in learning to speak and to do simple tasks.	0.20 (0.13–0.29)
Long-term mild motor impairment	This person has some difficulty in moving around but is able to walk without help.	0.01 (0.005–0.019)
Borderline intellectual disability	This person is slow in learning at school. As an adult, the person has some difficulty doing complex or unfamiliar tasks but otherwise functions independently.	0.011 (0.005–0.020)
Severe motor impairment	This person is unable to move around without help, and is not able to lift or hold objects, get dressed, or sit upright.	0.40 (0.27–0.55)
Epilepsy	(combined DW)	NA
Blindness	Is completely blind, which causes great difficulty in some daily activities, worry and anxiety, and great difficulty going outside the home without assistance.	0.19 (0.12–0.26)
Acute encephalitis	This person has a high fever and pain, and feels very weak, which causes great difficulty with daily activities.	0.13 (0.088–0.19)
Mild intellectual disability	This person has low intelligence and is slow in learning at school. As an adult, the person can live independently but often needs help to raise children and can only work at simple supervised jobs.	0.043 (0.026–0.064)
Monocular distance vision loss	This person is blind in one eye and has difficulty judging distances.	0.017 (0.009–0.029)

Mild motor plus cognitive impairments	This person has some difficulty in moving around but is able to walk without help. The person is slow in learning at school. As an adult, the person has some difficulty doing complex or unfamiliar tasks but otherwise functions independently.	0.031 (0.018–0.050)
Severe motor plus cognitive impairments	This person cannot move around without help, and cannot lift or hold objects, get dressed, or sit upright. The person also has very low intelligence, speaks few words, and needs constant supervision and help with all daily activities.	0.54 (0.37–0.70)
Moderate vision impairment due to encephalitis	This person has vision problems that make it difficult to recognize faces or objects across a room.	0.031 (0.019–0.049)
Severe vision impairment due to encephalitis	This person has severe vision loss, which causes difficulty in daily activities, some emotional impact (for example worry), and some difficulty going outside the home without assistance.	0.18 (0.13–0.26)

References

1. Edmond, K. *et al.* Global and regional risk of disabling sequelae from bacterial meningitis: a systematic review and meta-analysis. *The Lancet Infectious Diseases* **10,** 317–328 (2010).
2. Centers for Disease Control (CDC). CDC health information for international travel 2016: the yellow book. New York City, United States: Oxford University Press, USA, 2016.

Acute endocarditis

Flowchart

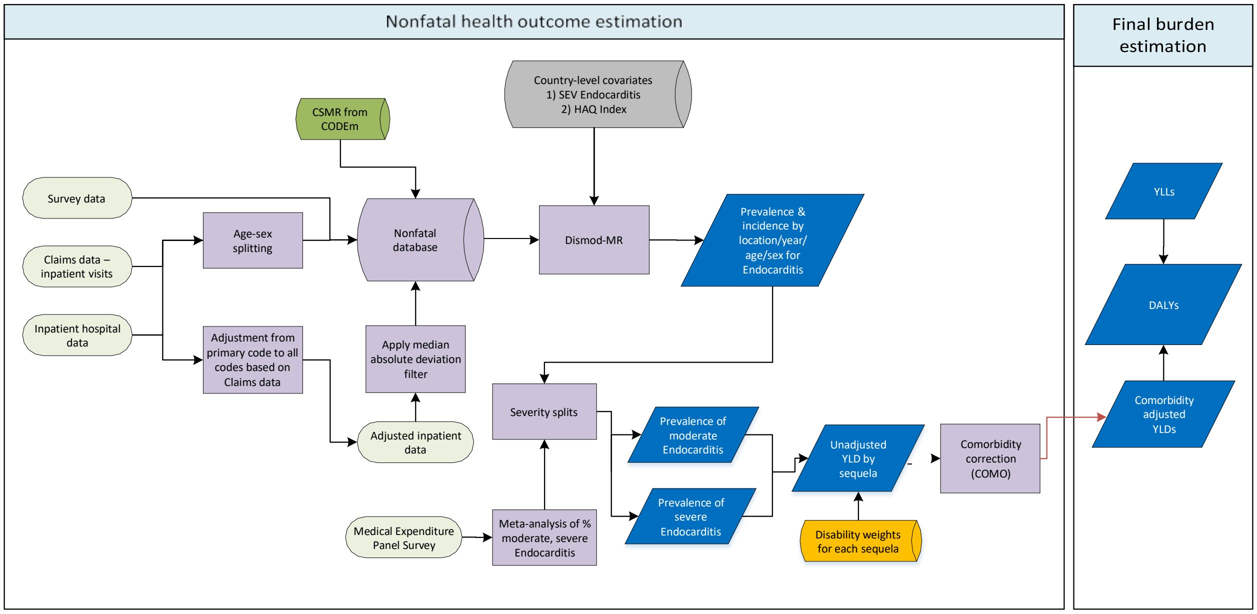

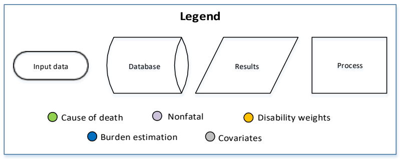

Input data and methodological appendix

Case definition

Acute endocarditis is a bacterial or fungal infection of the heart, with a vegetation adherent to a heart valve or chordae. The standard for clinical diagnosis of infective endocarditis is through the Duke Criteria, which include confirmation through clinical criteria, specific blood tests, and cardiovascular imaging.

Table 1: ICD codes used for inclusion of hospital and claims data

Cause	ICD-9	ICD-10
Endocarditis	421-421.9	I33-I33.9, I38-I39.9

Input data

Model inputs

Table 2: Source counts for acute endocarditis

Measure	Total sources	Countries with data
All measures	337	44
Incidence	336	44
Excess mortality rate	1	1

We did not perform a systematic review for GBD 2021. A systematic review was last performed for GBD 2015. The following search terms were used: ((‘endocarditis’[MeSH Terms] OR ‘endocarditis’[All Fields]) AND ‘epidemiology’[Subheading]) OR ((‘endocarditis’[MeSH Terms] OR ‘endocarditis’[All Fields]) AND ((‘epidemiology’[Subheading] OR ‘epidemiology’[All Fields] OR ‘incidence’[All Fields] OR ‘incidence’[MeSH Terms]) OR (‘epidemiology’[Subheading] OR ‘epidemiology’[All Fields] OR ‘prevalence’[All Fields] OR ‘prevalence’[MeSH Terms]) OR ‘case fatality’[All Fields])) OR ((‘endocardium’[MeSH Terms] OR ‘endocardium’[All Fields]) AND inflammation[TIAB] AND ‘epidemiology’[Subheading]) OR ((‘endocardium’[MeSH Terms] OR ‘endocardium’[All Fields]) AND inflammation[TIAB] AND ((‘epidemiology’[Subheading] OR ‘epidemiology’[All Fields] OR ‘incidence’[All Fields] OR ‘incidence’[MeSH Terms]) OR (‘epidemiology’[Subheading] OR ‘epidemiology’[All Fields] OR ‘prevalence’[All Fields] OR ‘prevalence’[MeSH Terms]) OR ‘case fatality’[All Fields]))

- Dates included in search: 1/1/2013–3/16/2015
- Number of initial hits: 1246
- Number of sources included: 6

We did not include any non-literature-based data types, apart from the hospital and claims data described elsewhere. We excluded all outpatient data, as they were implausibly low when compared with inpatient data and claims data from the same locations. We used hospital data corrected for readmission and primary to any diagnosis based on the correction factors generated by the clinical informatics team.

More information on how correction factors were made for this adjustment can be found in the "Claims data" section of the non-fatal appendix. Diagnosis of endocarditis requires a combination of information provided by imaging and blood cultures;[2] in low- and middle-income settings, this can pose a challenge in diagnosis, for example with regard to organism non-identification, resulting in unclear incidence of the disease.[3] For several locations with hospital data, there were either an implausible number of zero counts of incident cases of endocarditis for all ages and sexes, or stochastic age patterns across the age range. To address this, we excluded any inpatient hospital datapoints which were more than two-fold higher or 0.5-fold lower than the median absolute deviation[1] value for high-income North America, central Europe, and western Europe for that age-sex group. No data adjustments were done for acute endocarditis in GBD 2021.

Severity split inputs

The proportion of moderate and severe for acute endocarditis were determined by the standard approach for severity splitting for GBD 2021 that used the Medical Expenditure Panel Survey (MEPS) to map endocarditis ICD codes (see table 1) to quality of life metrics to quantify disability. More information on methodology on the proportion split using MEPS can be found in the appendix section 4.7: Severity distribution. The table below includes the severity level, lay descriptions, and disability weights (DWs) associated with acute endocarditis.

Table 3. Severity distribution, details on the severity levels for acute endocarditis in GBD 2021 and the associated disability weights

Severity level	Lay description	DW (95% CI)
Moderate	Has a fever and aches, and feels weak, which causes some difficulty with daily activities.	0.051 (0.032–0.074)
Severe	Has a high fever and pain, and feels very weak, which causes great difficulty with daily activities.	0.133 (0.088–0.19)

Modelling strategy

For GBD 2020, we estimated the incidence and prevalence of acute endocarditis using a DisMod-MR 2.1 Bayesian meta-regression model; the long-term prevalence of heart failure due to endocarditis is estimated as part of the heart failure modelling process. We set a minimum of 11 and maximum of 13 as value priors on remission to establish an average duration of one month. Country-level covariates used included the age-standardised endocarditis summary exposure variable scalar (SEV scalar) on incidence and Healthcare Access and Quality Index on excess mortality. The table below gives the parameters, betas, and exponentiated betas for study-level and country-level covariates used in the model.

Table 4. Covariates. Summary of covariates used in the acute endocarditis DisMod-MR meta-regression model

Covariate	Parameter	Beta	Exponentiated beta (95% uncertainty interval)
Healthcare Access and Quality Index	Excess mortality rate	–0.10 (–0.10 to –0.10)	0.90 (0.90 to 0.90)
Log-transformed age-standardised SEV scalar: endocarditis	Incidence	0.76 (0.75 to 0.79)	2.14 (2.12 to 2.20)

We evaluated models by comparing model fits with the data and with results from previous GBD estimation cycles. Apart from updates to the clinical informatics data included in the model, there have been no substantive updates for the acute endocarditis estimation process since GBD 2017.

References:

[1] Huber, P.J. (2011). Robust Statistics. In: Lovric, M. (eds) *International Encyclopedia of Statistical Science*. Springer, Berlin, Heidelberg. https://doi.org/10.1007/978-3-642-04898-2_594

[2] Cuervo G, Escrihuela-Vidal F, Gudiol C, Carratalà J. Current Challenges in the Management of Infective Endocarditis. *Front Med* (Lausanne). 2021 Feb 22;8:641243. doi: 10.3389/fmed.2021.641243. PMID: 33693021; PMCID: PMC7937698.

[3] Njuguna B, Gardner A, Karwa R, Delahaye F. Infective Endocarditis in Low- and Middle-Income Countries. *Cardiol Clin*. 2017 Feb;35(1):153-163. doi: 10.1016/j.ccl.2016.08.011. PMID: 27886786.

Endocrine, metabolic, blood, and immune disorders

Flowchart

Endocrine, Metabolic, Blood, Immune Disorders (EMBID)

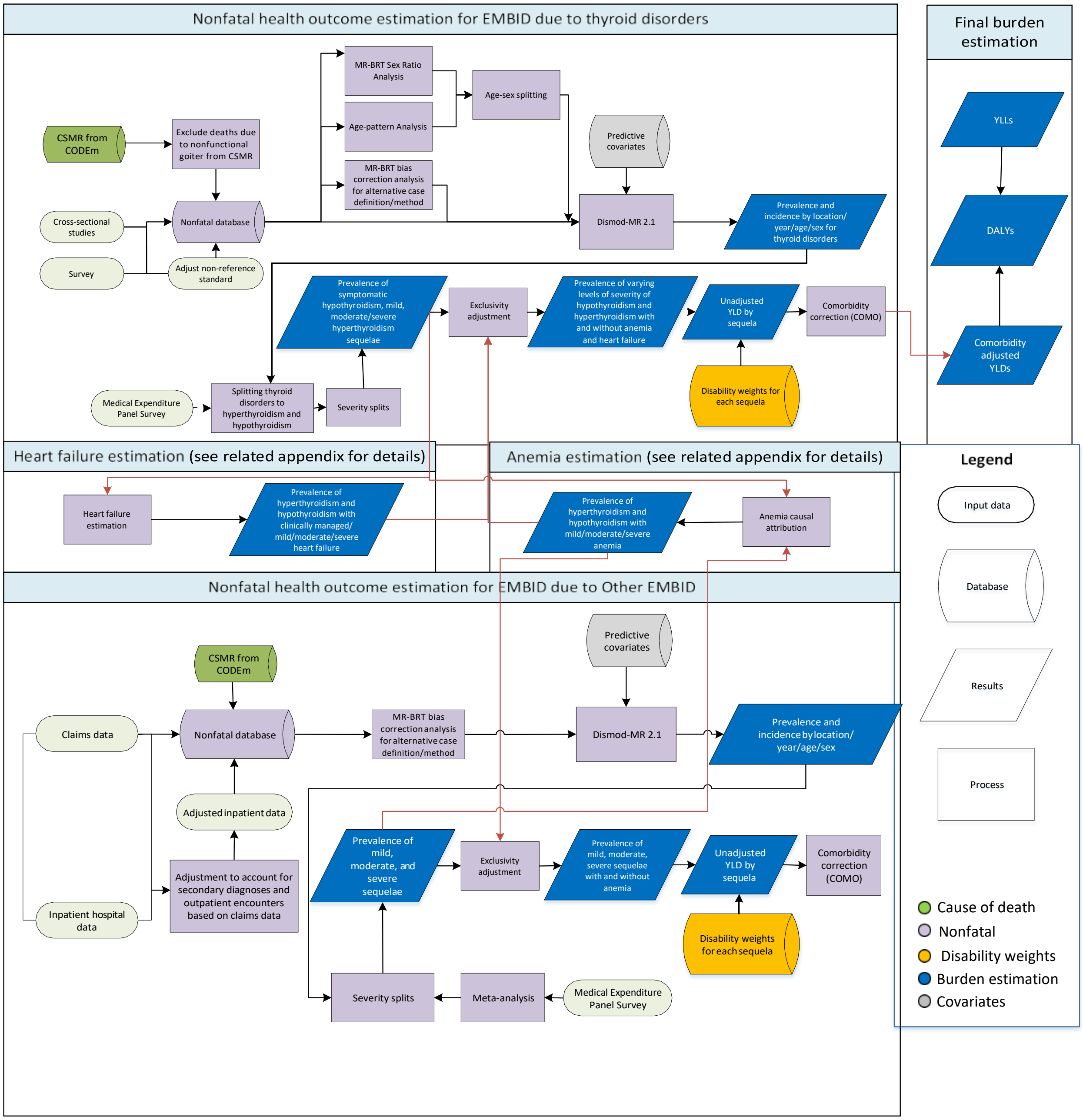

Input data and methodological summary for endocrine, metabolic, blood, and immune disorders

Case definitions

Endocrine, metabolic, blood, and immune disorders (EMBID) is a residual cause consisting of conditions that do not map to other causes within the diabetes, urogenital, blood, and endocrine disease hierarchy. In GBD 2021, we introduced two Level 4 causes under total EMBID: 1) thyroid disorders and 2) other EMBID excluding thyroid disorders (referred as "other EMBID" throughout this report).

Thyroid disorders are defined as having abnormal thyroid function, evidenced by abnormal levels of thyroid-stimulating hormone and thyroxine, or by being on treatment for thyroid dysfunction. Specifically, abnormal levels of thyroid stimulating hormone and thyroxine are defined as:

- Overproduction of thyroid hormones: serum thyroid-stimulating hormone (TSH) concentration of 0.5 mIU/L or less; confirmed with high serum free T4 result (by laboratory standard)
- Underproduction of thyroid hormones: serum TSH concentration of 5 mIU/L or greater; confirmed with low serum free T4 result (by laboratory standard)

Other EMBID includes other metabolic, immune, or blood disorders (excluding haemoglobinopathies, haemolytic anaemias, and iron deficiency). From the ICD chapter on endocrine, metabolic, and immune disorders (the E chapter), GBD's definition of other EMBID excludes the codes for nutritional deficiencies, diabetes, and anaemia, which are modelled as separate causes or impairments; as well as those for obesity and hypercholesterolaemia, which are modelled as risk factors.

ICD-10 codes for other EMBID include: D64.4, D64.8, D68-D68.6, D68.8-D68.9, D69.6, D73- D73.5, D73.8-D73.9, D74.0, D74.8-D74.9, D75-D75.2, D75.8-D75.9, D76-D76.3, D80-D80.9, D81-D81.9, D82-D82.4, D82.8-D82.9, D83-D83.2, D83.8-D83.9, D84-D84.1, D84.8-D84.9, D86.8, D89-D89.2, D89.8-D89.9, E04-E04.2, E04.8-E04.9, E16.. 1-E16.4, E16.8-E16.9, E20-E20.1, E20.8-E20.9, E21-E21.5, E22-E22.2, E22.8-E22.9, E23.0, E23.2-E23.3, E23.6-E23.7, E24-E24.1, E24.3, E24.9, E25.0, E25.8-E25.9, E26-, E26.8-E26.9, E27-E27.2, E27.4-E27.5, E27.8-E27.9, E28-E28.1, E28.3, E28.8-E28.9, E29-E29.1, E29.8-E29.9, E30-E30.1, E30.8-E30.9, E31-E31.2, E31.8-E31.9, E32-E32.1, E32.8-E32.9, E34-E34.5, E34.8-E34.9, E67-E67.3, E67.8, E70-E70.5, E70.8-E70.9, E71-E71.5, E72-E72.5, E72.8-E72.9, E73-E73.1, E73.8-E73.9, E74-E74.4, E74.8-E74.9, E75-E75.6, E76-E76.3, E76.8-E76.9, E77-E77.1, E77.8-E77.9, E79-E79.2, E79.8-E79.9, E80-E80.7, E84-E84.9, E88-E88.9.

In GBD 2021, we removed D70-D70.4, D70.8-D70.9, D72-D72.1, D72.8-D72.9, D75.1, E26.1 E83-E83.9, E85-E85.9, E88.3, E71.43, D69-D69.4, and D69-D69.8 from EMBID.

Overall strategy

We utilised two databases for EMBID as inputs to two separate, complete compartmental DisMod-MR models: thyroid disorders and other EMBID. The model outputs of thyroid disorders and other EMBID were separately adjusted for varying levels of sequelae and comorbidity, which were combined at the end of the modelling pipeline to produce the final non-fatal estimates of total EMBID.

All input data used for thyroid disorders and other EMBID are summarised below.

Table 1. Total data inputs for endocrine, metabolic, blood, and immune disorders morbidity modelling by parameter

Measure	Countries with data	New sources	Total sources

All measures	50	54	363
Prevalence	50	51	344
Incidence	4	4	4
Other	1	0	15

Thyroid disorders

Input data and data processing

Input data

For thyroid disorders, we extracted prevalence and incidence data from peer-reviewed publications identified via systematic literature reviews conducted by Madariaga and colleagues in 2014[1] and Taylor and colleagues in 2018.[2] We also included microdata from the USA National Health and Nutrition Examination Survey (NHANES) from years 2001–2002, 2007–2008, and 2009–2010.

The non-fatal model of thyroid disorders also included cause-specific mortality rate (CSMR) estimates taken from our fatal modelling process (see CoD cause-specific modelling description for thyroid disorders in this appendix). Prior to using these CSMR estimates in the non-fatal model, we conducted an adjustment to remove deaths due to non-functional goitres (see the CSMR data processing section below).

Prevalence and incidence data processing

We first adjusted data from the studies that used non-reference TSH cut-offs to diagnose thyroid disorders. To do this, we leveraged TSH distributions found in the National Health Surveys from Chile and USA. Specifically, we calculated the prevalence estimates meeting the alternative (non-reference) TSH cut-offs and the reference TSH cut-offs. Then we calculated the logit difference of the non-reference prevalence estimates and the reference prevalence estimates. These logit differences were used as an input to MR-BRT (meta-regression—Bayesian, regularised, trimmed) to estimate the adjustment factors, which were then applied to all datapoints meeting the alternative TSH cut-offs.

We also made a systematic bias adjustment on the datapoints that only included previously undiagnosed populations. For this, we first identified studies that reported data for both reference (ie, including those currently on treatment) and non-reference (ie, excluding those currently on treatment) (Figure 1). We then calculated the logit difference between the two datapoints for each study and used as an input to MR-BRT the same manner described above. The adjustment factor was then applied to all datapoints of non-reference standard.

---

[1] Garmendia Madariaga A, Santos Palacios S, Guillén-Grima F, Galofré JC. The incidence and prevalence of thyroid dysfunction in Europe: a meta-analysis. J Clin Endocrinol Metab. 2014 Mar;99(3):923-31. doi: 10.1210/jc.2013-2409. Epub 2014 Jan 1. PMID: 24423323.

[2] Taylor PN, Albrecht D, Scholz A, Gutierrez-Buey G, Lazarus JH, Dayan CM, Okosieme OE. Global epidemiology of hyperthyroidism and hypothyroidism. Nat Rev Endocrinol. 2018 May;14(5):301-316. doi: 10.1038/nrendo.2018.18. Epub 2018 Mar 23. PMID: 29569622.

The process of adjusting for non-reference data using MR-BRT with the logit-transformation method is described below:

29. Identify datapoints with overlapping year, age, sex, and location between non-reference and reference data within the same study.
30. Logit transform overlapping datapoints of alternative and reference case definitions.
31. Convert overlapping datapoints into a difference in logit space using the following equation: $logit(altnerative) - logit(reference)$.
32. Use the delta method to compute standard errors of overlapping datapoints in logit space, then calculate standard error of logit difference using the following equation: $\sqrt{(variance\ of\ alternative) + (variance\ of\ reference)}$.
33. Using MR-BRT, conduct a random effects meta-regression to obtain the pooled logit difference of alternative to reference.
34. Apply the pooled logit difference to all datapoints of non-reference case definitions using the following equation:

$$new_{estimate} = inverse.logit((logit(alternative)) - (pooled\ logit\ difference)).$$

35. Calculate new standard errors using the delta method, accounting for gamma (between-study heterogeneity).

Figure 1. Studies reported both reference and non-reference (alternative) data; used in MR-BRT

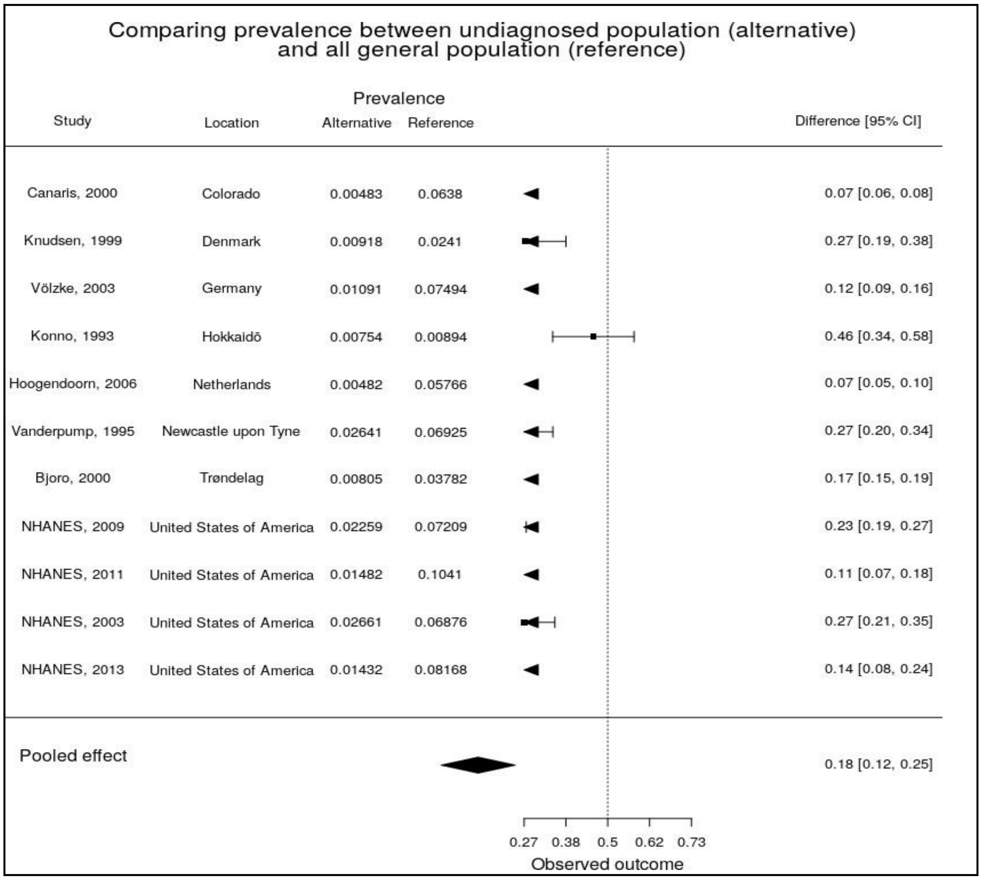

Table 2. MR-BRT crosswalk adjustment factors for thyroid disorders

Data input	Reference or alternative case definition	Gamma	Beta coefficient, logit (95% CI)	Adjustment factor*
General population	Ref	0	--	--
Excluding those currently on treatment	Alt		–1.45 (–1.78 to –1.12)	0.234 (0.17 to 0.33)

**MR-BRT crosswalk adjustments can be interpreted as the factor the alternative case definition is adjusted by to reflect what it would have been had it been measured using the reference case definition. If the log/logit beta coefficient is negative, then the alternative is adjusted up to the reference. If the log/logit beta coefficient is positive, then the alternative is adjusted down to the reference.*
***The adjustment factor column is the exponentiated beta coefficient. For log beta coefficients, this is the relative rate between the two case definitions. For logit beta coefficients, this is the relative odds between the two case definitions.*

We split datapoints where the age range was greater than 25 years using the global age pattern informed by the datapoints with fine age groups (ie, ages 5–9, 10–14, and 15–20…). We also split data reported for both sexes using the pooled sex ratio estimated from studies that reported prevalence or incidence in males and females separately. For prevalence, the ratios of female to male cases derived from MR-BRT analysis were 2.71 (95% UI 1.88–3.91). For incidence, we only had sex-specific datapoints and thus did not require separate sex-splitting data process.

CSMR data processing

Unlike the fatal model, our non-fatal model excludes non-functional goitres. To maintain consistency between the fatal and non-fatal input data, we conducted a custom modelling process to estimate CSMR of thyroid disorders excluding non-functional goitre. Specifically, this was done by modelling the proportion of nonfunctional goitre deaths from the total thyroid disorders deaths by year and super-region using cause of death data extracted from data rich locations (see CoD cause-specific modelling description in this appendix).

Modelling approach of thyroid disorders

DisMod-MR model

We ran a DisMod-MR 2.1 model to produce estimates by age, sex, year, and country. Inputs to DisMod-MR were prevalence, incidence, and CSMR estimates described above. We set a maximum disease duration of two years and assumed that no one was born with thyroid disorders. The minimum coefficient of variation at the regional, super-regional, and global level was 0.8.

We included Healthcare Access and Quality Index and proportion of households using iodised salt (adjusted) as predictive covariates to inform excess mortality and incidence. The beta and exponentiated values of these covariates (which can be interpreted as an odds ratio) are shown in the table below.

Table 3. Covariates. Summary of covariates used in the thyroid disorders DisMod-MR meta-regression model

Covariate	Parameter	Exponentiated beta (95% uncertainty interval)
Healthcare Access and Quality Index	Excess mortality rate	1.00<br>(1.00–1.00)
Proportion of households using iodised salt (adjusted)	Incidence	0.14<br>(0.14–0.16)

Symptom severity could vary between hyperthyroidism and hypothyroidism. To better account for disease burden, we used data from the Medical Expenditure Panel Survey (MEPS) to split the estimates of thyroid disorders into hyperthyroidism and hypothyroidism:

Disease	ICD-9 codes used in MEPS	Frequency	Proportion
Hyperthyroidism	242, 245	1138	9.8%
Hypothyroidism	243, 244, 246	10,417	90.2%

Severity split & disability weight

The basis of the GBD disability weight survey assessments is lay descriptions of sequelae highlighting major functional consequences and symptoms. Hyperthyroidism and hypothyroidism are assigned different levels of severity. Specifically, hypothyroidism is split into asymptomatic and symptomatic categories. Hyperthyroidism is split into asymptomatic, mild, and moderate/severe categories. The lay descriptions and disability weights for thyroid disorders are shown below.

Table 4. Severity distribution, details on the severity levels for endocrine, metabolic, blood, and immune disorders and the associated disability weight (DW) with that severity.

Disease	Severity level	Lay description	DW (95% CI)
Hypothyroidism	Asymptomatic	--	--
	Symptomatic	Has low energy and feels cold	0.019<br>(0.010–0.032)
Hyperthyroidism	Asymptomatic	--	--
	Mild	Has a chronic disease that requires medication every day and causes some worry but minimal interference with daily activities	0.049<br>(0.031–0.072)
	Moderate/severe	Feels nervous, has palpitations, sweats a lot, and has difficulty sleeping	0.145<br>(0.095–0.202)

The severity distributions of hypothyroidism and hyperthyroidism were derived from analysis of the Medical Expenditure Panel Surveys (MEPS). MEPS is an overlapping panel survey of the non-institutionalised USA population that collects data on respondents' health service interactions. Panels are initiated every year. Each panel is two years long and consists of five rounds. In 2000, MEPS began using 12-Item Short Form Surveys (SF-12) to collect data on functional health status. The SF-12 survey is administered twice per panel (about once per year).

To translate SF-12 scores into GBD disability weights, 62 lay descriptions for conditions representing the full range of disability weight values (from most mild to most severe) were selected. A convenience sample of respondents was then asked to complete an SF-12 form for an individual with the health state described in the lay descriptions of these conditions. Composite mental and physical SF-12 score was regressed on GBD disability weight to derive the relationship between disability weight and SF-12 score. Individual respondent scores were then regressed on reported conditions to obtain a comorbidity-corrected condition-specific disability weight. The distribution of these condition-specific weights was used to derive the proportion of individuals with the conditions that fall within each GBD severity category.

Disease	Severity	Distribution
Hypothyroidism	Asymptomatic hypothyroidism	0.436<br>(0.432–0.441)
	Symptomatic hypothyroidism	0.564<br>(0.559–0.568)
Hyperthyroidism	Asymptomatic hyperthyroidism	0.417<br>(0.410–0.423)
	Mild hyperthyroidism	0.418<br>(0.360–0.447)
	Moderate/severe hyperthyroidism	0.165<br>(0.138–0.222)

Other endocrine, metabolic, blood, immune disorders

Input data and data processing

Input data

The other EMBID model included prevalence data from hospital discharges and claims. In GBD 2021, we newly added additional years of data from USA claims (year 2017) and Poland claims (year 2018), as well as hospital discharges in Greece, Armenia, Chile, Ecuador, Argentina, Italy, Brazil, and Spain.

In addition to prevalence data, inputs to our non-fatal modelling also included cause-specific mortality rate (CSMR) estimates taken from our fatal modelling process (see CoD cause-specific modelling description for other EMBID in this appendix).

Prevalence data processing

Hospital discharge data provide observations about encounters, generally with only the primary diagnostic code for the encounter. Claims data, on the other hand, link claims for all inpatient and outpatient encounters for a single individual, and provide primary and secondary diagnoses for all encounters.

An individual was extracted from claims data as an incident case if that individual had at least one inpatient or outpatient encounter with an appropriate ICD code as any diagnosis within one year. Hospital discharge data were processed by extracting discharges with an appropriate ICD code as

primary diagnosis and adjusting using correction factors (ie, correction factor 3) derived from claims data. Specifically, we modelled from the ratio of inpatient claims with other EMBID as primary diagnosis to total incident cases of other EMBID seen in claims data.

The USA claims data (extracted and processed as described above) were adjusted to account for selection bias due to commercial insurance, using the same MR-BRT analysis described above.

Table 5. MR-BRT crosswalk adjustment factors for other endocrine, metabolic, blood, immune disorders

Data input	Reference or alternative data collection	Gamma	Covariate	Beta coefficient, logit (95% CI)	Adjustment factor*
Hospital + non-USA claims	Ref	0.002		---	---
USA claims from year 2000	Alt		Age (continuous from 0 to 95+)	–0.04 (–0.06 to –0.2)	0.97 (0.95 to 0.98)
			Sex (female to male)	–0.33 (–0.37 to –0.28)	0.72 (0.69 to 0.75)
			Intercept	3.90 (3.81 to 3.99)	49.27 (44.98 to 53.97)
USA claims from years 2010–2017	Alt		Age (continuous from 0 to 95+)	–0.03 (–0.05 to –0.01)	0.97 (0.95 to 0.99)
			Sex (female to male)	–0.53 (–0.56 to 0.52)	0.59 (0.57 to 0.60)
			Intercept	4.97 (4.89 to 5.04)	143.34 (132.75 to 154.78)

**MR-BRT crosswalk adjustments can be interpreted as the factor the alternative case definition is adjusted by to reflect what it would have been had it been measured using the reference case definition. If the log/logit beta coefficient is negative, then the alternative is adjusted up to the reference. If the log/logit beta coefficient is positive, then the alternative is adjusted down to the reference.*
***The adjustment factor column is the exponentiated beta coefficient. For log beta coefficients, this is the relative rate between the two case definitions. For logit beta coefficients, this is the relative odds between the two case definitions.*

Datapoints with an age-standardised prevalence rate greater than two median absolute deviations from the median of the age-standardised prevalence rate for all inpatient and non-USA claims data were marked as outliers and excluded from analysis.

Modelling of other EMBID

DisMod-MR model

We ran a DisMod-MR 2.1 model to produce estimates by age, sex, year, and country. Input data were prevalence and CSMR estimates described above. Prior settings in the DisMod-MR model included

setting maximum remission of four years. We also assumed that no one was born with other EMBID. The minimum coefficient of variation at the regional, super-regional, and global level was set at 0.8.

We included lagged distributed income (LDI) as a predictive covariate to inform excess mortality, with a lower bound of –0.5 and an upper bound of –0.1. The beta and exponentiated values of this covariate (which can be interpreted as an odds ratio) are shown in the table below.

Table 6. Covariates. Summary of covariates used in the other endocrine, metabolic, blood, and immune disorders DisMod-MR meta-regression model

Covariate	Parameter	Exponentiated beta (95% uncertainty interval)
LDI (I$ per capita)	Excess mortality rate	0.81<br>(0.81–0.83)

Severity split & disability weight

Other EMBID is split into four levels of severity: asymptomatic, mild, moderate, and severe. The lay descriptions and disability weights for other EMBID are shown below.

Table 7. Severity distribution, details on the severity levels for endocrine, metabolic, blood, and immune disorders and the associated disability weight (DW) with that severity.

Disease	Severity level	Lay description	DW (95% CI)
Other EMBID	Asymptomatic	--	--
	Mild	Feels slightly tired and weak at times, but this does not interfere with normal daily activities	0.004<br>(0.001–0.008)
	Moderate	Has a chronic disease that requires medication every day and causes some worry but minimal interference with daily activities	0.049<br>(0.031–0.072)
	Severe	Easily bruises and sometimes bleeds from the gums and nose; feels weak and has some difficulty with daily activities	0.159<br>(0.106–0.226)

The severity distributions of other EMBID were derived from analysis of the Medical Expenditure Panel Surveys (MEPS) the same manner as hyperthyroidism and hypothyroidism as described above.

Disease	Severity	Distribution
Other EMBID	Asymptomatic other endocrine, metabolic, blood, and immune disorders	0.431<br>(0.427–0.436)
	Mild other endocrine, metabolic, blood, and immune disorders	0.202<br>(0.148–0.262)
	Moderate other endocrine, metabolic, blood, and immune disorders	0.189<br>(0.149–0.226)

	Severe other endocrine, metabolic, blood, and immune disorders	0.178 (0.148–0.220)

Epilepsy impairment envelope

Flowchart

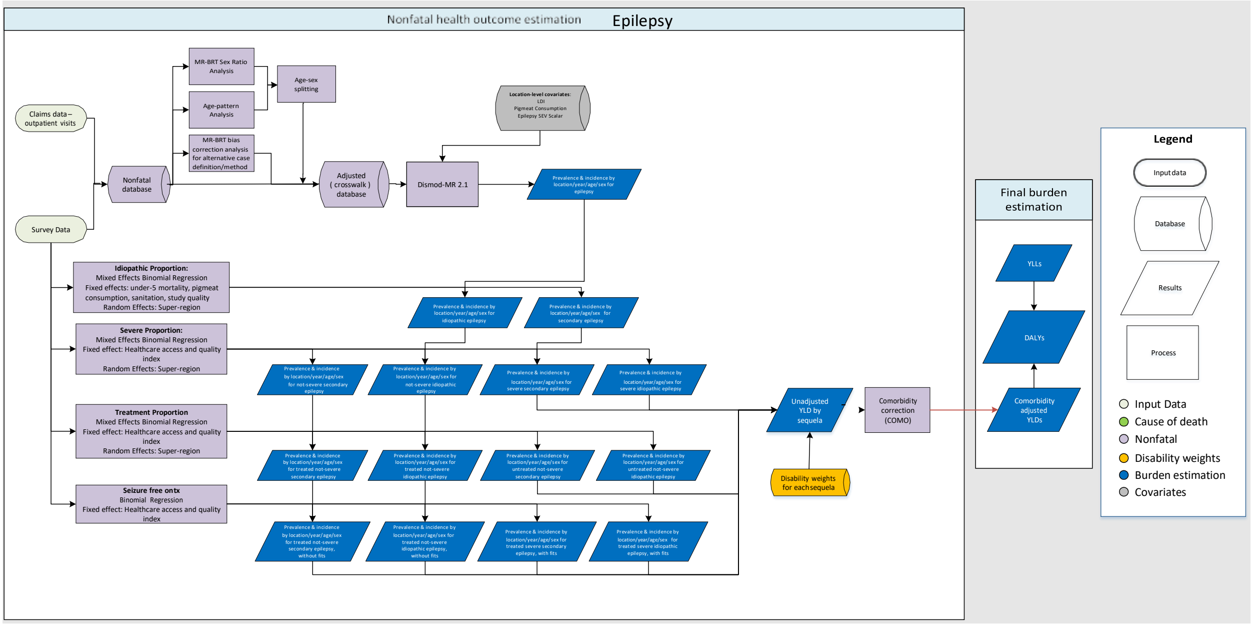

Case definition

Epilepsy is a condition characterized by recurrent epileptic seizures due to abnormal electrical activity in the brain with underlying causes including stroke, traumatic brain injury, neonatal insult to the brain, and others, including unknown origin. Since GBD 2013, we have used the following definitions from the "Guidelines for Epidemiologic Studies on Epilepsy": 1) Epilepsy: a condition characterised by recurrent (two or more) epileptic seizures, unprovoked by any immediate identified cause, and 2) "Active" epilepsy: a prevalent case of active epilepsy is defined as a person with epilepsy who has had at least one epileptic seizure in the previous five years, regardless of antiepileptic drug (AED) treatment. We also use the following ICD-10 codes for epilepsy: G40 (Neuro, epilepsy, total) and G41 (Neuro, epilepsy, status epilepticus). We define severe epilepsy as having seizures one or more times per month.

Input data and processing

Data inputs

The primary data inputs for the epilepsy modelling strategy were measurements of prevalence, incidence, remission rate, excess mortality rate, relative risk of mortality, standardised mortality ratio, or with-condition mortality rate for all epilepsy, regardless of cause, severity, or treatment status.

For GBD 2021, we conducted a systematic review covering 01/10/2016 to 01/28/2020 using the following search string:

(2016/10/01[PDAT] : 3000[PDAT]) AND ("epilepsy"[MeSH Terms] OR "epilepsy, partial, motor"[MeSH Terms] OR "epilepsy, benign neonatal"[MeSH Terms] OR "epilepsy, reflex"[MeSH Terms] OR "myoclonic epilepsy, juvenile"[MeSH Terms] OR "epilepsy, frontal lobe"[MeSH Terms] OR "epilepsy, complex partial"[MeSH Terms] OR "epilepsy, post-traumatic"[MeSH Terms] OR "epilepsy, temporal lobe"[MeSH Terms] OR "epilepsy, absence"[MeSH Terms] OR "epilepsy, tonic-clonic"[MeSH Terms] OR "epilepsies, myoclonic"[MeSH Terms] OR "epilepsies, partial"[MeSH Terms] OR epilep*[Title/Abstract]) AND (inciden*[Title/Abstract] OR prevalen*[Title/Abstract]) NOT (animals[MeSH] NOT umans[MeSH])

We included representative, population-based surveys that reported on prevalence, incidence, remission rate, excess mortality rate, relative risk of mortality, standardised mortality ratio, or with-condition mortality rate. We excluded studies with no clearly defined sample (eg, among clinic attenders or patient organisation members with non-specific or non-representative catchment area).

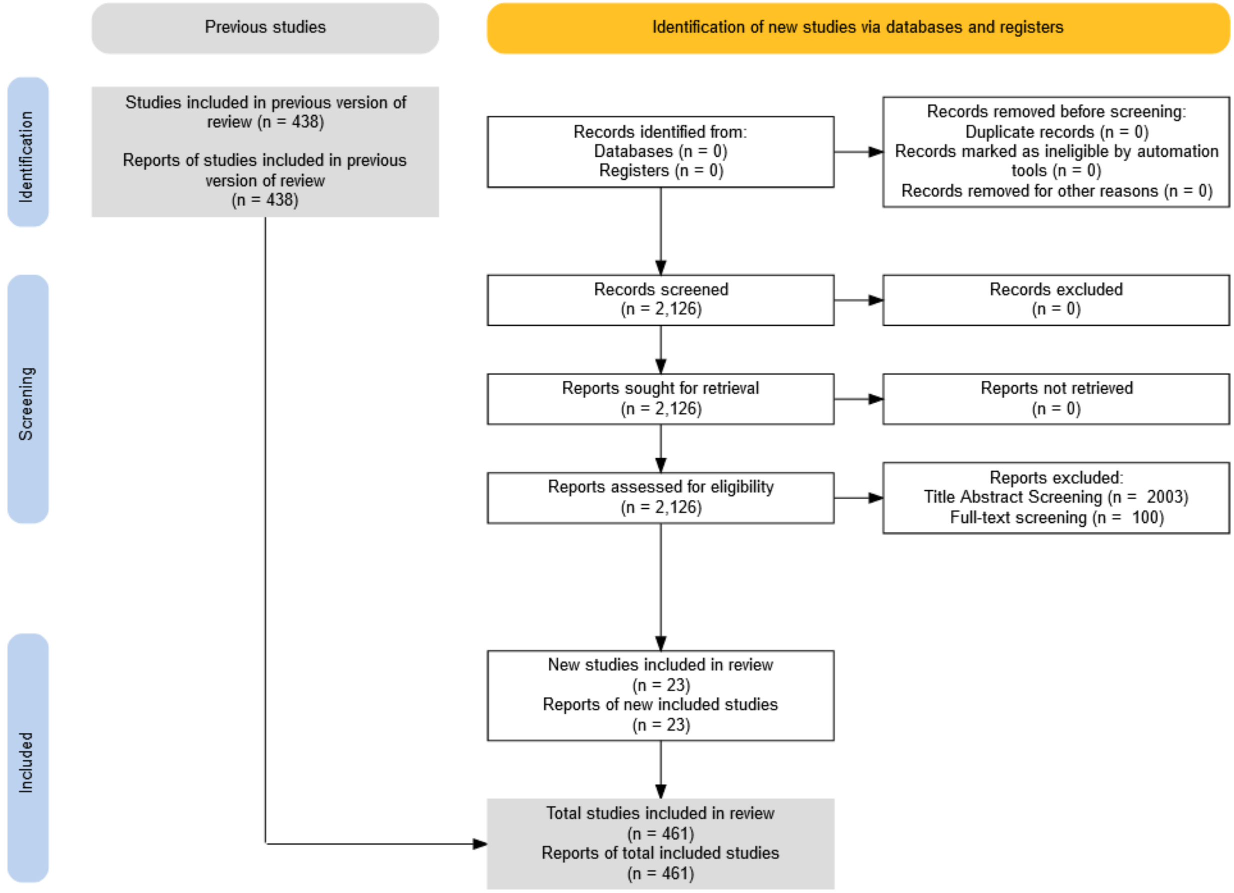

For epilepsy modelling in GBD 2021, we used the following clinical data sources: Poland claims data from 2018, and Taiwan claims data from 2016. While we have previously used USA MarketScan claims data from the years 2000, and 2010 through 2017, with the addition of the USA MarketScan claims data for

2018 we found that there was so much MarketScan claims data in comparison to the smaller population studies that MarketScan was having an unduly large impact on the model. As such we decided to drop all USA MarketScan claims data as we trust the smaller population studies more.

Additional data inputs include data on the proportion of epilepsy that is primary or idiopathic, the proportion of epilepsy that is severe (one or more fits per month), the proportion of epilepsy that is untreated (the treatment gap), and the proportion of treated epilepsy that is treated without fits (no fits reported in the preceding year).

The number of sources used for all epilepsy, and for idiopathic epilepsy specifically, are listed below:

Epilepsy impairment:

Measure	Total sources	Countries with data
All measures	491	93
Prevalence	373	87
Incidence	89	38
Remission	3	3
Other	174	56

Data processing

For GBD 2021, raw data with large age ranges were split into 5-year age groups using the age pattern generated from a Dismod-MR 2.1[1] (disease model—Bayesian meta-regression, details on this method can be found in appendix 1, section 4.5 of the citation) model with input data of only less than 25 years age range. Standard GBD sex splitting methods were used for studies with only “both”-sex datapoints. We modelled the ratio of female/male prevalence in MR-BRT (meta-regression—Bayesian, regularised, trimmed) and calculated male prevalence:

$$prev_{male} = prev_{both} * \frac{pop_{both}}{(pop_{male} + ratio * pop_{female})}$$

And then calculated female prevalence:

$$prev_{female} = ratio * prev_{male}$$

For epilepsy, the modelled female/male ratio demonstrated a higher prevalence in males and was used to proportionally split “both”-sex datapoints into male and female datapoints (as seen in the figure below).

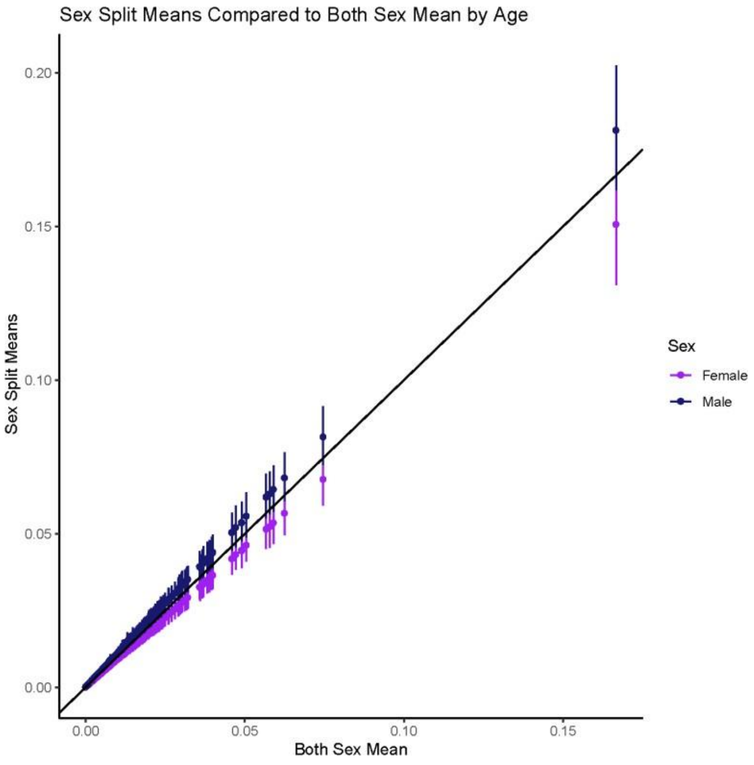

For GBD 2021, adjustment factors for all study-level covariates were determined using matched data (by year, age, sex, location) for reference and alternative case definitions in a logit ratio meta-regression. Studies that asked for lifetime recall were crosswalked to the reference definition for epilepsy (see case definition).

The table below shows adjustment factors estimated using MR-BRT.

MR-BRT crosswalk adjustment factors for epilepsy impairment envelope

Data input	Reference or alternative case definition	Gamma	Beta coefficient, log (95% UI)*	Adjustment factor**
	Ref	N/A	N/A	N/A
Recall lifetime	Alt	0.39	0.26 (–0.75 to 1.27)	1.3

**MR-BRT crosswalk adjustments can be interpreted as the factor the alternative case definition is adjusted by to reflect what it would have been had it been measured using the reference case definition. If the log/logit beta coefficient is negative, then the alternative is adjusted up to the reference. If the log/logit beta coefficient is positive, then the alternative is adjusted down to the reference.*

***The adjustment factor column is the exponentiated beta coefficient. For log beta coefficients, this is the relative rate between the two case definitions. For logit beta coefficients, this is the relative odds between the two case definitions.*

Modelling strategy

We modelled the prevalence of epilepsy in two steps: first, we created an epilepsy impairment envelope. Second, we split the envelope into primary (or idiopathic) and secondary epilepsies. Each of these was subdivided into "severe" (on average one or more fits per month) and "non-severe." Non-severe cases were subdivided into "treated" and "untreated." Finally, "treated" cases were divided into "treated cases with fits" (between one and 11 fits on average in the preceding year) and "treated cases without fits" (no fits reported in the preceding year).

In the first step, we used DisMod-MR 2.1 for the epilepsy impairment envelope to model a consistent fit between incidence, prevalence, remission, and fatal data.

We also included the SEV epilepsy scalar, which summarises the epilepsy risk exposure level from all epilepsy risk factors for each country, as a predictive covariate on prevalence. We included cause-specific mortality rate (CSMR) estimates from the epilepsy mortality model as input data to the DisMod-MR model. Where age-specific prevalence data were available, we calculated excess mortality rate (EMR) from prevalence and CSMR. We included the log of the lag-distributed income (LDI) as a covariate on EMR to account for lower mortality in developed countries. We included Bayesian priors on remission to account for the scarcity of remission data. We set bounds on remission from 0 to 0.25 from age 0–60 and 0 to 0.05 from age 61–100. The table below indicates the covariates used in the estimation process, as well as parameters, betas, and exponentiated betas.

Covariates. Summary of covariates used in the epilepsy impairment envelope DisMod-MR meta-regression model.

Covariate	Type	Beta coefficient, log difference (95% UI)*	Adjustment factor**
Log-transformed age-standardised SEV scalar: Idiopathic epilepsy	Prevalence	0.98 (0.82 to 1.14)	2.67 (2.27 to 3.14)
LDI ($ per capita)	Excess mortality rate	–0.55 (–0.97 to –0.12)	0.58 (0.38 to 0.88)

In the second step, we used mixed-effects generalised linear models (binomial family) run in GBD 2021 to predict the proportion of idiopathic epilepsy, the proportion of severe epilepsy, the proportion of treated epilepsy, and the proportion of epilepsy that is treated without fits.

Because not all the data on the proportion of idiopathic epilepsy used optimal case finding methods (using CT scans or MRIs in addition to EEGs in order to diagnose secondary epilepsy), we first ran an initial linear regression model with a covariate on study quality. We then used the beta from this model to crosswalk studies with non-optimal case finding methods to those with adequate methods. The adjusted data were then used in the regression for the proportion of epilepsy that is idiopathic, with a fixed effect on SDI as well as a random effect on super-region.

We used similar models to predict the proportion of severe epilepsy and treatment gap based on the reported proportions extracted from the systematic review. To predict the proportion of severe epilepsy and the treatment gap, we used mixed-effects models with a fixed effect on the log of Healthcare Access and Quality (HAQ) Index and a random effect on super-region.

For the regression to determine the proportion of treated epilepsy cases that had not had a fit in the last year, there was a much smaller dataset, and therefore we could not use a random effect in the model. Therefore, we used generalised linear model (binomial family) to generate predictions for the proportion of treated epilepsy that was seizure-free with a fixed effect on the log of HAQ Index.

Severity splits & disability weights
The table below illustrates the severity levels, descriptions, and disability weights associated with epilepsy. These are calculated using regressions from literature (ie, frequency of seizures).

Severity level	Lay description	Disability weights (95% CI)
severe (seizures ≥ once per month)	This person has sudden seizures one or more times each month, with violent muscle contractions and stiffness, loss of consciousness, and loss of urine or bowel control. Between seizures the person has memory loss and difficulty concentrating.	0.552 (0.375–0.71)
less severe (seizures < once per month)	This person has sudden seizures two to five times a year, with violent muscle contractions and stiffness, loss of consciousness, and loss of urine or bowel control.	0.263 (0.173–0.367)
Treated without fits	This person has a chronic disease that requires medication every day and causes some worry but minimal interference with daily activities.	0.49 (0.031–0.072)

[1] Vos T, Lim SS, Abbafati C, *et al.* Global burden of 369 diseases and injuries in 204 countries and territories, 1990–2019: a systematic analysis for the Global Burden of Disease Study 2019. *The Lancet* 2020; 396: 1204–22. doi: https://doi.org/10.1016/S0140-6736(20)30925-9

Fistula (impairment)

Flowchart

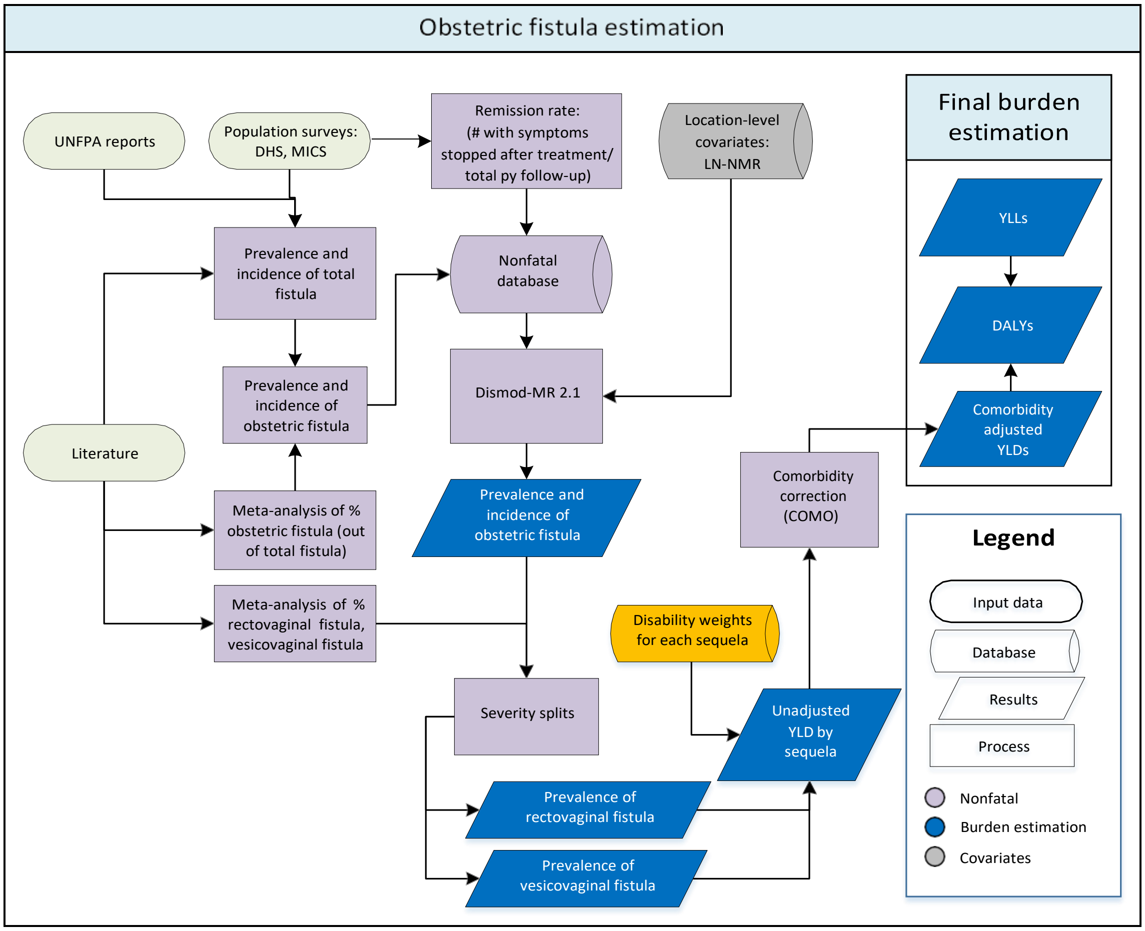

Case definition

This is estimated as a component of maternal obstructed labour. Obstetric fistula is an abnormal opening between the vagina and the bladder or rectum with involuntary escape of urine, flatus, and/or faeces following childbirth.

Input data

A systematic review was last conducted for GBD 2015, at which time no additional studies were identified. The PubMed search terms for this search, which were a repeat of those used in GBD 2010 and GBD 2013 were: (('obstetric fistula'[All Fields] OR 'vesicovaginal fistula'[All Fields]) OR 'rectovaginal fistula'[All Fields]) AND ('2013'[PDAT] : '2015'[PDAT]) AND 'humans'[MeSH Terms].

The exclusion criteria were studies that did not provide primary data on epidemiological parameters, eg, commentaries, case series, and reviews. The table below shows the number of literature studies included in GBD 2021, as well as the number of countries or subnational units and GBD world regions represented. In addition to using data from published studies, we also included data from UNFPA reports and nationally representative Demographic and Health Surveys and Multiple Indicator Cluster Surveys.

The table below shows the number of total sources used in the estimation of obstetric fistula:

Cause/impairment name	Measure	Total sources*	Countries with data*
Maternal obstructed labour and uterine rupture	All measures	397	76
Maternal obstructed labour and uterine rupture	Prevalence	33	26
Maternal obstructed labour and uterine rupture	Incidence	349	59
Maternal obstructed labour and uterine rupture	Other	13	6

*These counts include the data sources used in estimating obstructed labour acute event, as well as obstetric fistula. The count of prevalence sources is exclusive to fistula, where the other measures are combined with obstructed labour.

Starting in GBD 2019, we began age-splitting all input data where the age range was wider than a single GBD age group using weights derived from our best GBD 2019 decomposition 1 model results. Weights were determined by dividing the result for a specific age by the result for the aggregate age specified in a given input datapoint. Age-specific values were then calculated by multiplying the aggregate input datapoint by these age specific weights.

Modelling strategy

For GBD 2021, obstetric fistula was modelled using DisMod-MR 2.1. We used neonatal mortality rate as a country-level covariate. We assume obstetric fistula is restricted to sub-Saharan Africa, south Asia, Yemen, Afghanistan, and Sudan. Remission was calculated, using the cure data from 11 Demographic and Health surveys, by dividing the number of cured obstetric fistula cases by total person-years of follow-up of all cases (cured, uncured, and untreated). The person-year of follow-up for uncured or untreated fistula cases was calculated as the time interval (in years) between the last birth and the date of interview. For cured cases, we assumed that the person-year of follow-up was half the time interval (in years) between the last birth and the date of interview.

Betas and exponentiated values (which can be interpreted as an odds ratio) are shown in the table below:

Modellable entity	Covariate name	Measure	Beta value	Exponentiated beta value
Obstetric fistula	Neonatal mortality rate modelled 2 (per 1000)	Remission	–0.48 (–0.94 to –0.013)	0.62 (0.39 to 0.99)
	Neonatal mortality rate modelled 2 (per 1000)	Prevalence	1.85 (1.66 to 1.99)	6.37 (5.24 to 7.31)
	Neonatal mortality rate modelled 2 (per 1000)	Incidence hazard	0.82 (0.45 to 1.28)	2.28 (1.57 to 3.60)

The following severity distributions were assigned based on a meta-analysis of published studies[1-4] and Pakistan Demographic and Health Survey (2006–2007): vesicovaginal fistula (90.8%, 95% CI: 85.0–95.4); rectovaginal fistula (9.2%, 95% CI: 4.6–15.0). The lay descriptions and disability weights for severity levels derived from the GBD disability weights study are shown below.

Table 1: Health states for fistula impairment severity distribution

Severity level	Lay description	DW (95% CI)

Vesicovaginal fistula	Has an abnormal opening between the bladder and the vagina, which makes them unable to control urinating. The person is anxious and depressed.	0.342 (0.227–0.478)
Rectovaginal fistula	Has an abnormal opening between their vagina and rectum causing flatulence and faeces to escape through the vagina. The person gets infections in their vagina and has pain when urinating.	0.501 (0.339–0.657)

References

1. Danso KA, Martey J, Wall LL, Elkins TE. The epidemiology of genitourinary fistulae in Kumasi, Ghana, 1977–1992. *International Urogynecology Journal*. 1996;7(3):117-120.

2. Wall LL, Karshima JA, Kirschner C, Arrowsmith SD. The obstetric vesicovaginal fistula: characteristics of 899 patients from Jos, Nigeria. *American Journal of Obstetrics and Gynecology*. 2004;190(4):1011-1016.

3. Das R, Sengupta S. Vesico-vaginal fistula of obstetric origin. *Journal of Obstetrics and Gynaecology of India*. 1969;19:383-389.

4. Mahfouz NP. Urinary and Faecal Fistulae. *BJOG*. 1938;45(3):405-424.

Foodborne trematodiases

Clonorchiasis

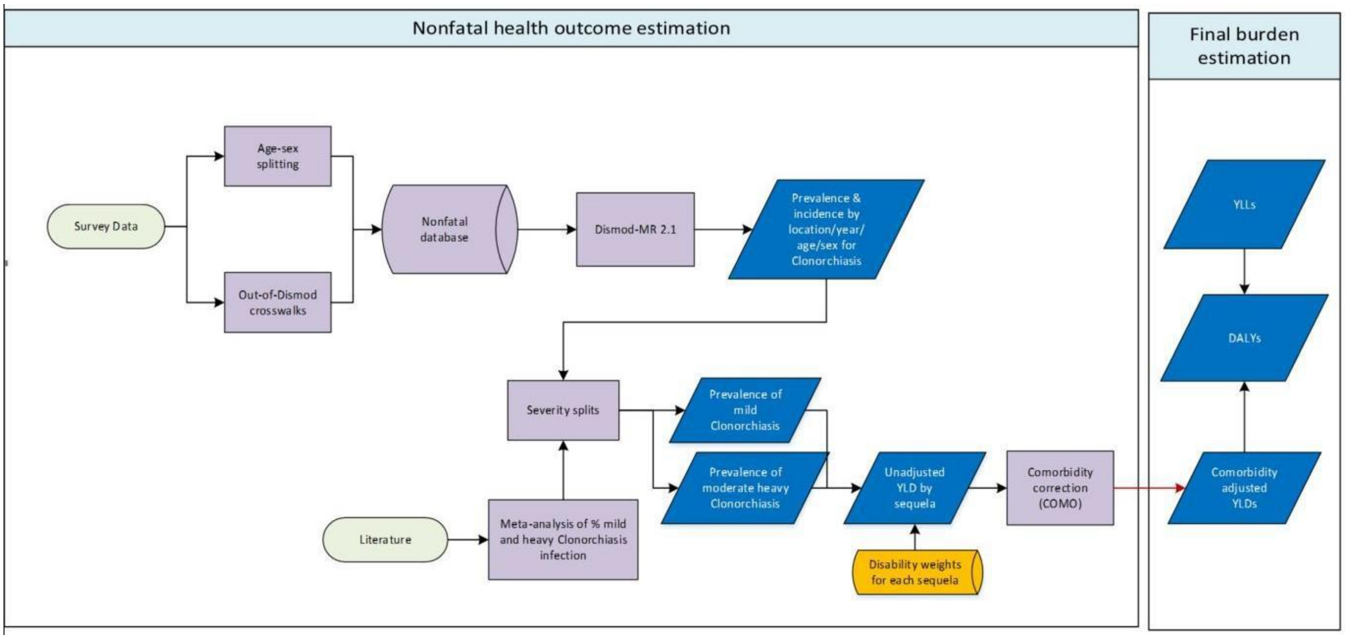

Fascioliasis

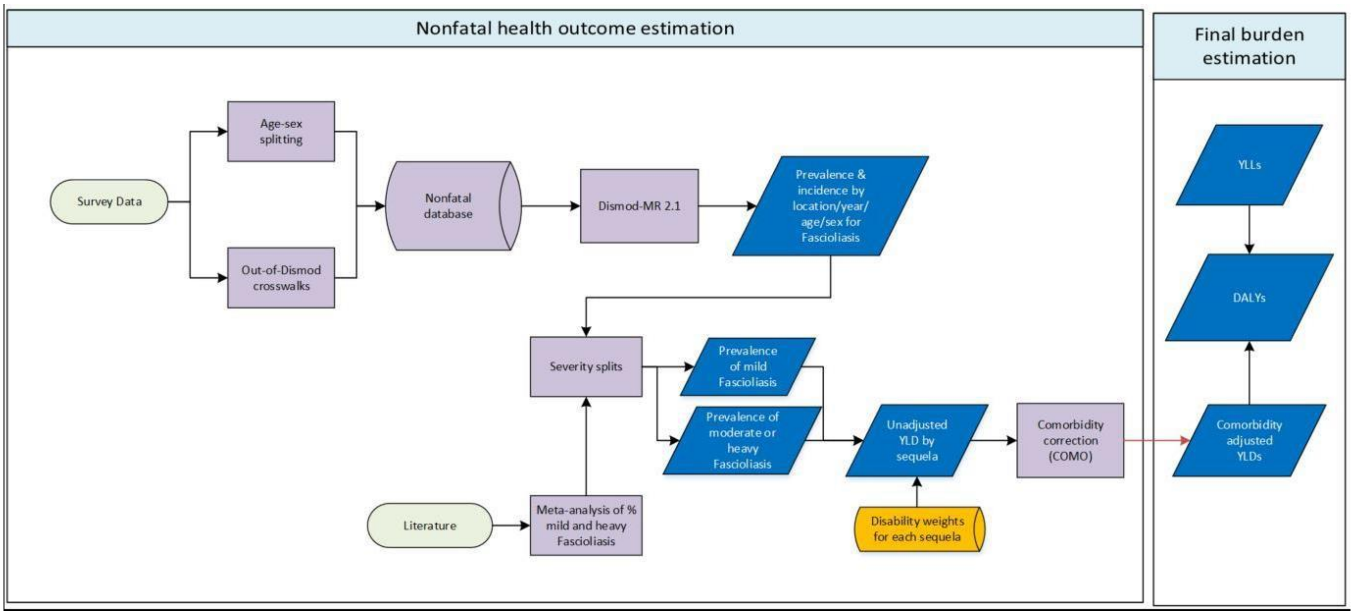

Intestinal fluke

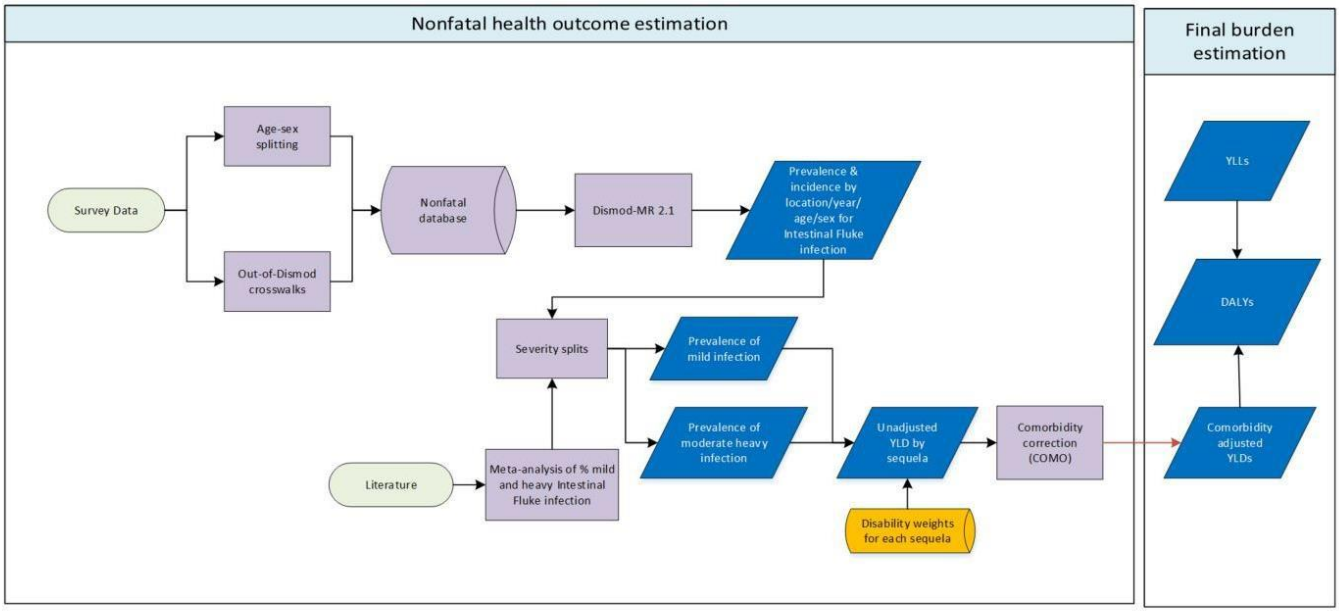

Opisthorchiasis

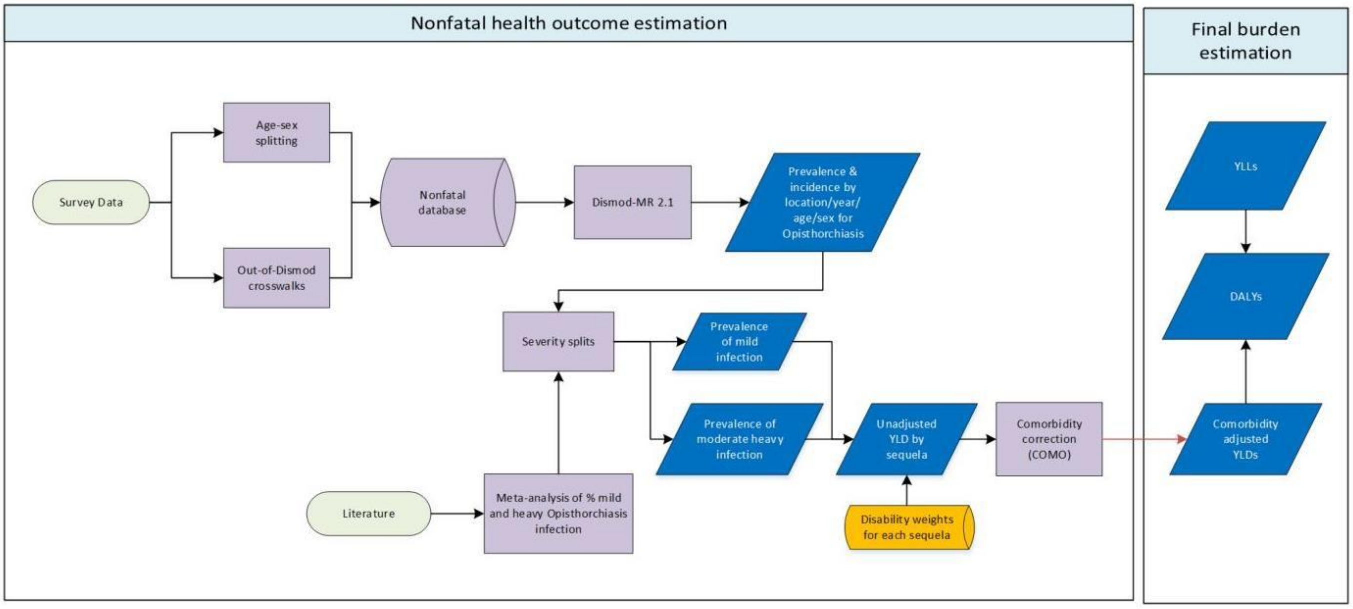

Paragonimiasis

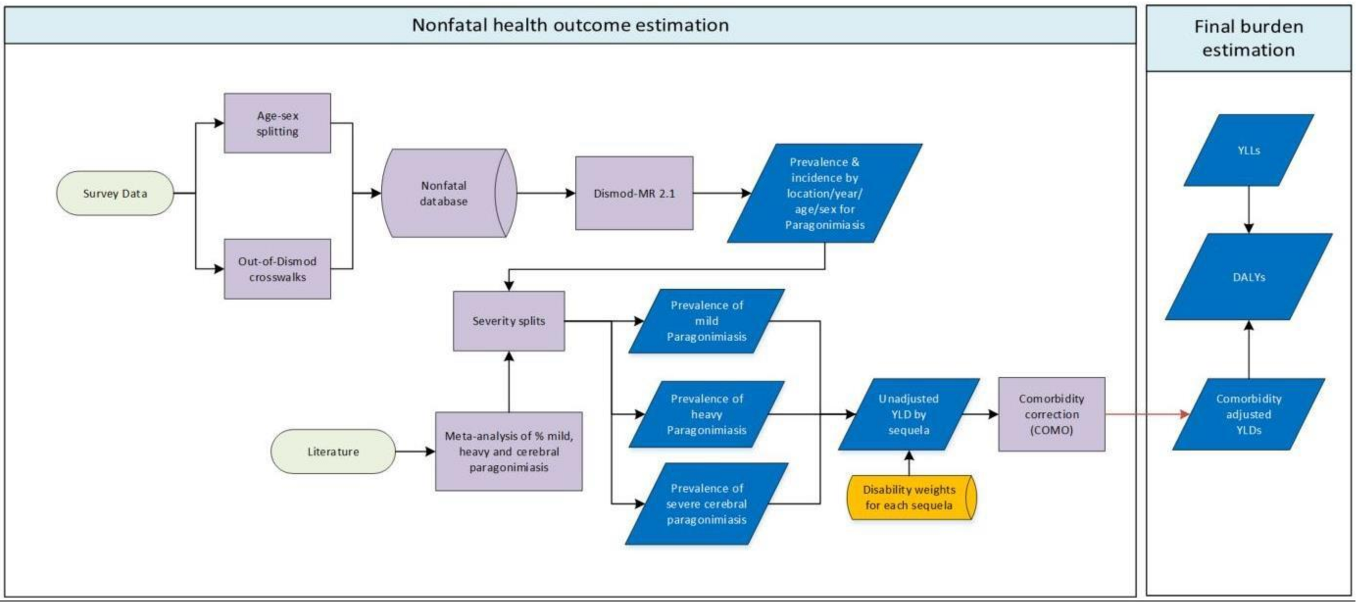

Input data and methodological summary

Case definition

The foodborne trematodiases (FBT) are a group of diseases that result from infection with parasitic worms of the class Trematoda, also known as flukes, via consumption of contaminated food. Infection of the liver, gallbladder, lungs, or brain can result in abdominal pain, chronic respiratory symptoms, neurologic symptoms including epilepsy, and cholangiocarcinoma (bile duct cancer). In the ICD-10, FBT are listed under code B66 [1].

FBT is subdivided into six types of FBT (see Table 1):

- Clonorchiasis

Clonorchiasis is a parasitic disease that results from infection with the liver fluke Clonorchis sinensis, transmitted primarily via consumption of raw or undercooked fish. In addition to acute infectious symptoms, longer-term complications can result from inflammation of the liver, gallbladder, and pancreas and biliary obstruction. Clinical manifestations include abdominal pain, nausea, vomiting, weight loss, fatigue, jaundice, and cholangiocarcinoma (bile duct cancer).

- Fascioliasis
  Fascioliasis is a parasitic disease that results from infection with the liver flukes Fasciola hepatica or Fasciola gigantica, transmitted primarily via consumption of contaminated raw water plants such as watercress. Acute clinical manifestations include abdominal pain, nausea, vomiting, fever, and rash, while chronic manifestations include jaundice, hepatomegaly, and weight loss, along with inflammation of the liver, gallbladder, and/or pancreas.
- Intestinal fluke
  Intestinal flukes are a diverse set of parasites including Fasciolopsis buski, Metagonimus yokogawai, Heterophyes heterophyes, Echinostoma species, and others, which can cause disease after infection of the intestinal tract. They are most commonly acquired via consumption of water plants, fish, and/or crustaceans. Many infected individuals are asymptomatic, but clinical manifestations can include abdominal pain, diarrhea, vomiting, or weight loss.
- Opisthorchiasis
  Opisthorchiasis is a parasitic disease that results from infection with the liver flukes Opisthorchis felineus or Opisthorchis viverrini, transmitted primarily via consumption of raw or undercooked fish. In addition to acute infectious symptoms, longer-term complications can result from inflammation of the liver, gallbladder, and pancreas and biliary obstruction. Clinical manifestations include abdominal pain, nausea, vomiting, weight loss, fatigue, jaundice, and cholangiocarcinoma (bile duct cancer).
- Paragonimiasis (normal and cerebral infections)
  Paragonimiasis is a parasitic disease that results from infection with lung flukes of the genus Paragonimus, most commonly Paragonimus westermani, transmitted via consumption of contaminated food - most commonly raw or undercooked crabs, crayfish, or snails. Acute infection can result in fever, abdominal pain, rash, chest pain, and cough; late infection can cause hemoptysis (cough with bloody sputum). Less common clinical manifestations result from spread of the parasite outside of the lungs, including to the central nervous system (causing meningitis or encephalitis, resulting in headache, fever, vomiting, and/or seizures), the intestines (causing nausea, vomiting and/or diarrhea), the kidneys (causing bloody urine), or the skin (causing skin nodules).

Table 1. Subtypes of FBT

	Species of FBT	Category	Carcinogen
1	Chlonorchiasis	Liver fluke	Associated with choliangiocarcinoma
2	Opisthorchiasis<br>(*O viverrini & O felineus*)	Liver fluke	Associated with choliangiocarcinoma<br>(*O viverrini*)
3	Fascioliasis	Liver fluke	No available evidence

4	Intestinal flukes (*Fasciolopsis buski, Metagonimus yokogawai, Heterophyes heterophyes, Echinostoma species,* and others)	Intestinal fluke	No available evidence
5	Paragonimiasis	Lung fluke	

Case definitions used for estimation of non-fatal health burden of FBTs

**Quantity of interest**	**Reference or alternative**	**Definition**
Chlonorchiasis	Reference	Prevalence of Chlonorchiasis fluke infections identified by the presence of eggs in microscopic examination of stool or serological tests.
Opisthorchiasis	Reference	Prevalence of Opisthorchiasis fluke infections identified by the presence of eggs in microscopic examination of stool.
Fascioliasis	Reference	Prevalence of Fascioliasis fluke infections identified by the presence of eggs in microscopic examination of stool or serological tests.
Intestinal fluke	Reference	Prevalence of Intestinal fluke infections identified by the presence of either adult worms or eggs in the examination of stool.
Normal and cerebral Paragonimiasis	Reference	Prevalence of Paragonimiasis fluke infections identified by the presence of eggs in microscopic examination of stool or serological tests.

Thresholds for heavy infection and duration by species of FBT

The majority of people infected with FBTs are asymptomatic. When symptoms do occur, they are often non-specific. Among the clinical symptomatic group, severity is associated with worm burden, typically measured by faecal egg counts, and the duration of infection. The thresholds for heavy infection and duration by species of FBT are shown in Table 2. The clinical presentation of FBT depends on the target organs (liver, lung, or intestines). Clonorchiasis and opisthorchiasis patients may suffer from loss of appetite, fullness, indigestion, diarrhoea, pain in the right upper quadrant, lassitude, weight loss, ascites, and oedema.[2, 3] Cholangitis, obstructive jaundice, intra-abdominal mass, cholecystitis, and gallbladder or intrahepatic stones may occur as complications.[3, 4]

Table 2. Thresholds for heavy infection and duration by species of FBT

	Species of FBT	Case thresholds for heavy infection	Duration
1	Chlonorchiasis	10,000 eggs per g of faeces	lifelong
2	Opisthorchiasis	10,000 eggs per g of faeces	lifelong
3	Fascioliasis	1,000 eggs per g of faeces	lifelong
4	Intestinal fluke	1,000 eggs per g of faeces	lifelong
5	Paragonimiasis	100 eggs per 5 ml sputum	lifelong

6	Cerebral paragonimiasis	Any infection of the brain with flukes and/or eggs of *Paragonimus* spp.	lifelong

Input data

Table 3: Source counts

Measure	Total sources	Countries with data
All measures	57	18
Prevalence	56	18
Proportion	1	0

Model inputs

For GBD 2010, the data came from an expert group analysis, which used the results of a systematic literature review performed by Furst and colleagues as a starting point.[5] Furst and colleagues searched PubMed, WHOLIS, FAOBIB, Embase, CAB Abstracts, Literatura Latino Americana e do Caribe em Ciências de Saùde (LILACS), ISI Web of Science, BIOSIS preview, Science Direct, African Journals OnLine (AJOL), and the System for Information on Grey Literature in Europe (SIGLE), period Jan 1, 1980, to Dec 31, 2008. The initial number of studies identified through the literature review was ~34,000 references. The literature review included extracted data from 181 studies. For GBD 2013 and GBD 2015, the search strategy was replicated to capture epidemiological studies published between 2008 and 2015.

Input data for the assessment of the total national number of infected people

Only studies that used countrywide surveys to estimate the national prevalence rates were included (or for China, province-wide surveys). We included only national studies because FBT shows a highly focal spatial distribution and local cross-sectional surveys would profoundly under- or overestimate true national prevalence. Infection is highly related to food habits, and there are highly varying differences between national and subnational prevalence rates. This search was last updated for GBD 2015; the final dataset contained 29 prevalence studies from 17 countries. We used raw data from the selected studies as input for DisMod-MR.

Prevalence of intestinal fluke infection

Intestinal fluke infections can be caused by several different pathogens, such as *Metagonimus* spp., *Echinostoma* spp., and *Neodiplostomatidae*.[6] When assessing the prevalence of intestinal fluke infection, we added the identified prevalence for each parasite species in order to obtain the overall prevalence of intestinal fluke infections. This approach may lead to a certain overestimation of the true prevalence, because people may be co-infected with more than one intestinal fluke species. There is not sufficient evidence about the proportion of co-infections to effectively account for this in our modelling process, but the resulting overestimation of the true prevalence may be offset by the assumptions made in our modelling approach and the many challenges in generating the underlying epidemiological parameters (eg, diagnostic inaccuracy in the detection of infections with the more than 50 intestinal

fluke species). Also of note, the transmission sources of intestinal fluke infections are species-specific and therefore vary. For instance, *Fasciolopsis buski* is usually transmitted by eating raw water plants with the infective parasite stage attached to the water plants, whereas *Neodiplostomatidae* are transmitted by eating undercooked and infested frogs, snakes, and tadpoles. Because of these different transmission pathways, the rate of co-infection might in fact be smaller than expected.

Input data to differentiate between asymptomatic and heavy infections

We estimated the proportion of heavily infected among all infected in all available national and regional cross-sectional surveys. It is expected that heavy infection increases with age, and there are data available on heavy infection by age group. We therefore decided to include age-dependent rates of heavy infection for clonorchiasis, opisthorchiasis, and intestinal fluke infection. For (cerebral) paragonimiasis and fascioliasis there were not sufficient age-dependent data on high-intensity FBT infection for this approach, and we therefore used an estimate of the rate of heavy infection that did not vary by age.

Data pre-processing

We used a MR-BRT (meta-regression—Bayesian, regularised, trimmed) model with our sex-specific data to derive an estimate of the ratio of the male prevalence of all-species FBT infection to female prevalence of all-species FBT infection to split non-sex-specific data. Then, a DisMod-MR 2.1 Bayesian meta-regression model using the age-specific input data was run to derive an age pattern to apply to split the all-age data.

Table 4: MR-BRT crosswalk adjustment factors for all-species FBT infection

Data input	Reference or alternative case definition	Gamma	Beta Coefficient, Log (95% CI)*	Adjustment factor**
Female data	Ref	0.82	---	---
Male data	Alt		0.48 (-1.16 – 2.12)	1.62

**MR-BRT crosswalk adjustments can be interpreted as the factor the alternative case definition is adjusted by to reflect what it would have been had it been measured using the reference case definition. If the log/logit beta coefficient is negative, then the alternative is adjusted up to the reference. If the log/logit beta coefficient is positive, then the alternative is adjusted down to the reference.*

***The adjustment factor column is the exponentiated beta coefficient. For log beta coefficients, this is the relative rate between the two case definitions. For logit beta coefficients, this is the relative odds between the two case definitions.*

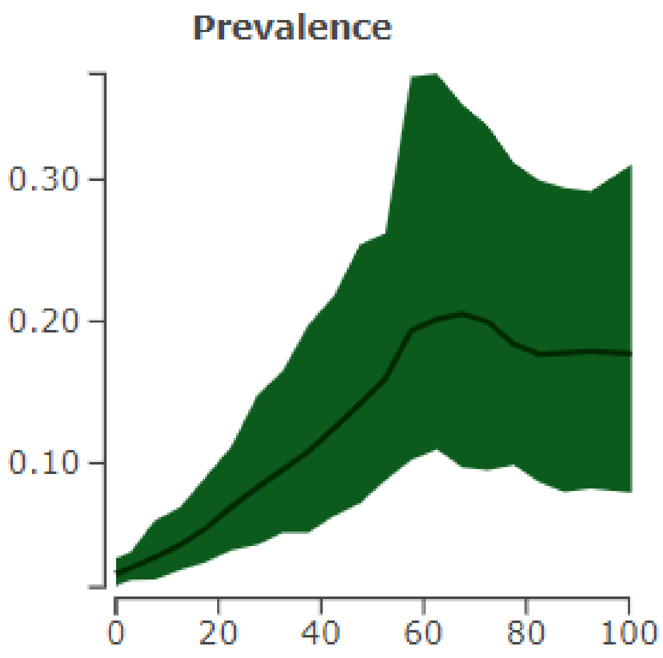

Figure 1: Global age pattern for all-species FBT infection used to split all-age data into age-specific datapoints for further modelling.

Modelling strategy

We used a three-step process for the disease modelling of FBT. In the first step we used DisMod-MR 2.0 to estimate the prevalence of FBT by age, sex, year, and country. In the second we differentiated between asymptomatic and heavy infections. MetaXL (a meta-analysis add-in for Microsoft Excel) was used to estimate the proportion of heavily infected among all infected by age group for clonorchiasis, opisthorchiasis, and intestinal fluke infection (see Table 4 and 5). These proportions were used to estimate the prevalence of heavy FBT infection. The third step consisted of deselecting countries that have no autochtonous case reports of FBTs.

Table 5. Percentage of high-intensity infection by age group and type of FBT (based on eight FBT prevalence studies)

Age category	Clonorchiasis			Opisthorchiasis			Intestinal fluke infection		
	Mean	Low	High	Mean	Low	High	Mean	Low	High
0-9	30%	17%	44%	10%	0%	29%	8%	3%	14%
10-19	15%	0%	43%	15%	0%	69%	11%	8%	14%
20-29	18%	10%	29%	16%	0%	52%	18%	15%	21%
30-39	17%	5%	34%	21%	0%	56%	22%	17%	28%
40-49	22%	13%	32%	28%	1%	68%	22%	13%	32%
50-59	18%	0%	49%	29%	0%	75%	17%	9%	28%
60+	32%	18%	47%	25%	0%	64%	15%	8%	23%

Table 6. Percentage of high-intensity infection by type of FBT (based on four FBT prevalence studies)

Type of FBT	Mean	Low	High
Paragonimiasis	23%	0%	59%
Fascioliasis	19%	3%	41%

Cerebral paragonimiasis

It was assumed that 0.8% of paragonimiasis cases have cerebral involvement. This proportion was used to estimate the prevalence of cerebral paragonimiasis. This proportion is based on one study. The data are from Oh SJ. The rate of cerebral involvement in paragonimiasis: an epidemiologic study. *Jpn J Parasitol* 1969;18:211-14. The study was performed in Paju, South Korea. This is an area with 6,738 inhabitants, and according to the survey, it was estimated that 29.6% of all individuals would react to intradermal test (an immunological reaction indicating previous or current contact with the parasite). 25% of all "positive reactors" may have eggs in their sputum (active infection with the parasite currently present in the human host). If these rates are applied to the community as a whole, the number of patients with active paragonimiasis would be at least 498 (=6,738*0.296*0.250). Furthermore, four cases of cerebral paragonimiasis were found in this community. Therefore, four out of 498 individuals with active paragonimus infection suffered from cerebral infection (=0.80%; 95% confidence interval 0.019%–1.587%).

Severity splits and disability weights

For GBD 2021, FBT was not split into health states with different severities, except of paragonimiasis. The table below shows the GBD 2021 disability weights that were used to calculate the burden of FBT in years lived with disability (YLDs).

Table 7. Disability weights that were used to calculate FBT YLDs

Sequelae	Severity description	Health state name	Disability weight
Asymptomatic clonorchiasis	Clonorchiasis, currently without symptoms	N/A	0.000 (0.000–0.000)
Heavy clonorchiasis	Abdominal pain and nausea reported as moderate	Abdominopelvic problem, moderate	0.114 (0.078–0.159)
Asymptomatic opisthorchiasis	Opisthorchiasis, currently without symptoms	N/A	0.000 (0.000–0.000)
Heavy opisthorchiasis	Abdominal pain and nausea reported as moderate	Abdominopelvic problem, moderate	0.114 (0.078–0.159)
Asymptomatic fascioliasis	Fascioliasis, currently without symptoms	N/A	0.000 (0.000–0.000)
Heavy fascioliasis	Abdominal pain and nausea reported as moderate	Abdominopelvic problem, moderate	0.114 (0.078–0.159)

Asymptomatic intestinal fluke infection	Intestinal fluke infection, currently without symptoms	N/A	0.000 (0.000–0.000)
Heavy intestinal fluke infection	Abdominal pain and nausea reported as moderate	Abdominopelvic problem, moderate	0.114 (0.078–0.159)
Mild paragonimiasis due to foodborne trematodiases	COPD and other chronic respiratory problems, mild	Has cough and shortness of breath after heavy physical activity, but is able to walk long distances and climb stairs.	0.019 (0.011-0.033)
Moderate paragonimiasis due to foodborne trematodiases	COPD and other chronic respiratory problems, moderate	Has cough, wheezing and shortness of breath, even after light physical activity. The person feels tired and can walk only short distances or climb only a few stairs.	0.225 (0.153-0.31)
Severe paragonimiasis due to foodborne trematodiases	COPD and other chronic respiratory problems, severe	Has cough, wheezing and shortness of breath all the time. The person has great difficulty walking even short distances or climbing any stairs, feels tired when at rest, and is anxious.	0.408 (0.273-0.556)
Cerebral paragonimiasis	Epilepsy	(combined DW)	--

Note. N/A: not applicable

Changes from GBD 2019 to GBD 2021

There were no major changes to our modelling approach between GBD 2019 and GBD 2021.
We did not apply any adjustments for the COVID-19 pandemic to foodborne trematodiases due to a lack of available data quantifying the impacts of the pandemic on NTD epidemiology.

References

1. WHO. *International Statistical Classification of Diseases and Related Health Problems. 10th Revision. Version for 2007.* 2007 [cited 2009 October 14, 2009]; Available from: http://apps.who.int/classifications/apps/icd/icd10online/.
2. Rim, H.J., *Clonorchiasis: an update.* J Helminthol, 2005. **79**(3): p. 269-81.
3. Pungpak, S., et al., *Clinical features in severe opisthorchiasis viverrini.* Southeast Asian J Trop Med Public Health, 1985. **16**(3): p. 405-9.
4. Rim, H.J., *The current pathobiology and chemotherapy of clonorchiasis.* Korean J Parasitol, 1986. **24**(Suppl.): p. 1-141.
5. Furst, T., J. Keiser, and J. Utzinger, *Global burden of human food-borne trematodiasis: a systematic review and meta-analysis.* Lancet Infect Dis, 2012. **12**(3): p. 210-21.

6. Furst, T., et al., *Manifestation, diagnosis, and management of foodborne trematodiasis.* BMJ, 2012. **344**: p. e4093.

Fungal skin diseases

Flowchart for tinea capitis and other fungal skin diseases

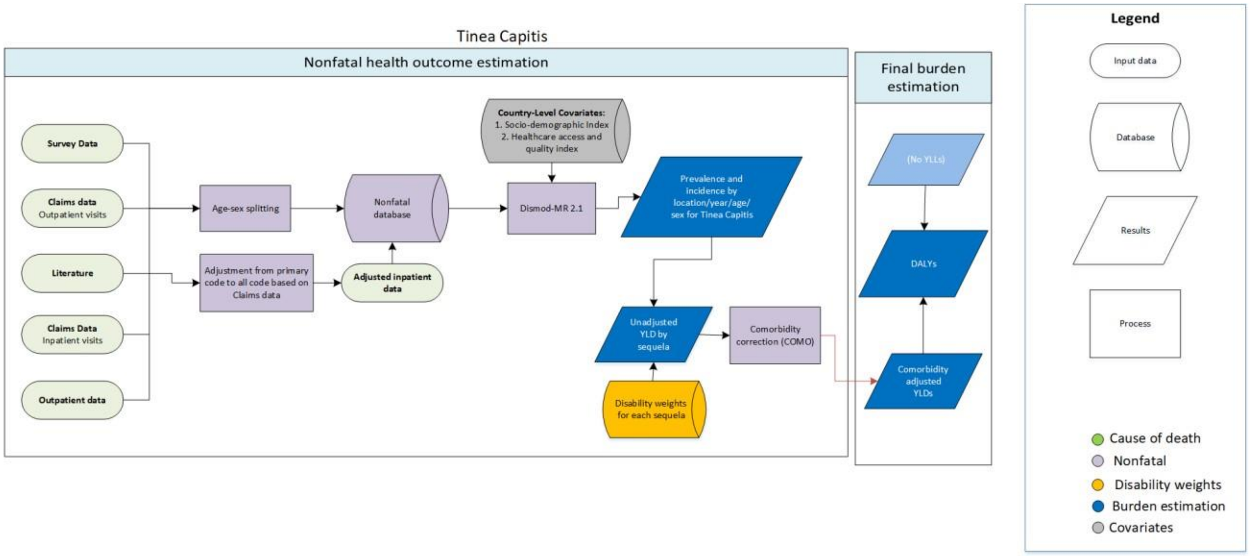

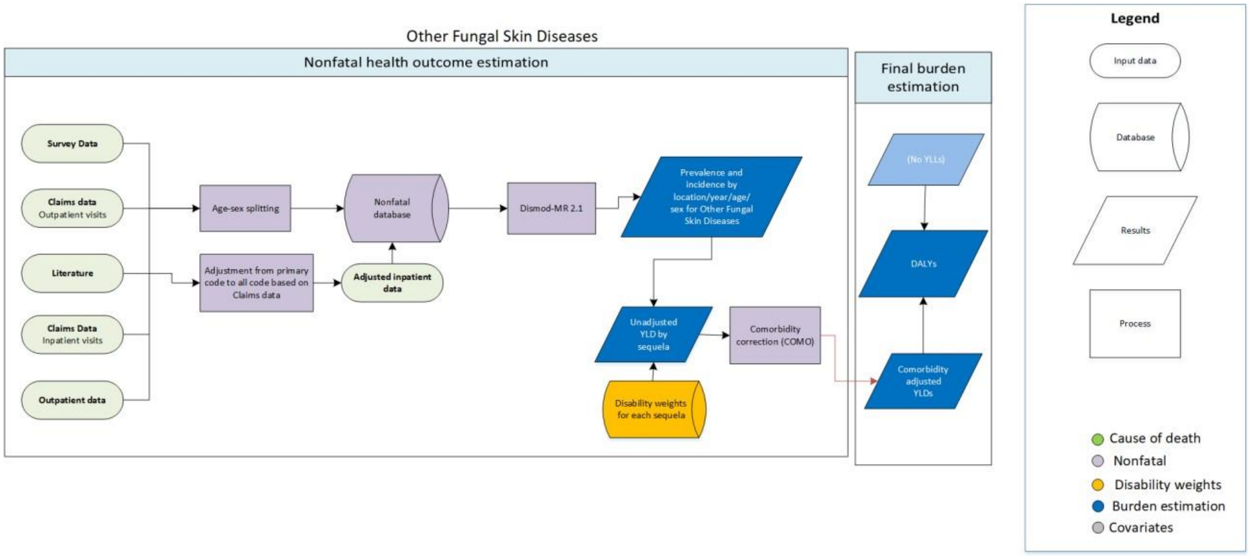

Input data and methodological summary for fungal skin diseases

Case definition

Fungal diseases were included in the GBD 2020 cause group of skin and subcutaneous conditions and consisted of tinea capitis and a residual group of “any” other fungal disease. Similar to GBD 2017, tinea

capitis was modelled separately from the other fungal skin diseases. This was done to better accommodate differences in burden between tinea capitis and other subtypes of fungal skin diseases.

The residual group of "any" other fungal skin disease included any fungal skin disease that was specifically not tinea capitis or onychomycosis (ie, fungal nail infection). The ICD-10 (1) list of other fungal skin diseases includes tinea manuum (ICD-10: B35.2), or hand ringworm; tinea pedis (ICD-10: B35.3), or athlete's foot; tinea corporis (ICD-10:B35.4), or ringworm of the body; tinea imbricata (ICD-10:B35.5), a superficial fungal infection limited to parts of Asia and Central America; tinea cruris (ICD-10:B35.6), also known as dhobi itch, groin ringworm, or jock itch. In GBD 2016, we added dermatophytosis (ICD-10:B35.9).

**Quantity of interest**	**Reference or Alternative**	**Definition**
Fungal skin diseases	Reference	Fungal skin diseases confirmed by a physical exam or recorded in claims data since 2010.
Fungal skin diseases	Alternative	Self-reported case of fungal skin disease or a case of fungal skin disease that is recorded in MEPS, claims data before 2010, or diagnosed without a physical exam.

Input data

For GBD 2010, a systematic review of the literature using PubMed and Google Scholar was conducted to capture epidemiological data for fungal skin diseases. The literature search also included any relevant data from the Medical Expenditure Panel Survey (MEPS) in the USA in 2000–2009. The inclusion criteria stipulated that studies (1) must be published between 1980 and 2012; (2) must provide data on the incidence or prevalence of fungal skin diseases; (3) must use samples representative of the general population (ie, samples derived from the experimental arm of clinical trials or based in dermatology clinics were excluded); (4) must use a sample size larger than 100; and (5) must provide sufficient information on study method and sample characteristics to assess the quality of the study. For GBD 2013, the GBD 2010 search strategy was replicated to capture epidemiological studies published between 2012 and 2013. For GBD 2017, the GBD 2010 search strategy was replicated in PubMed to capture epidemiological studies published between 2013 and 2017. Data were outliered or excluded if we found them unreasonable when compared to regional, super-regional, and global rates.

In addition, data from USA claims for 2000 and 2010 through 2016 by state were included for both tinea capitis and other fungal skin diseases, and Poland claims data and USA outpatient data were included for tinea capitis. For tinea capitis, we compared the rates in the outpatient data from Norway, Sweden, Canada, and the USA and found implausibly large differences with the rates from the claims data.

Table 1: Data inputs for fungal skin diseases morbidity modelling by parameter

**Cause/impairment name**	**Measure**	**Countries with data**	**New sources**	**Total sources**
Fungal skin diseases	All measures	31	3	137
Fungal skin diseases	Prevalence	31	3	137

Table 2: MR-BRT crosswalk adjustment factors for fungal skin diseases

Cause	Data input	Reference or alternative case definition	Gamma	Beta coefficient, logit* (95% CI)	Adjustment factor**
Tinea capitis	Literature with physical exam and USA MarketScan	Reference	0.24	---	---
	Outpatient	Alternative		2.04 (1.47 to 2.61)	0.88
Other fungal skin diseases	Literature with physical exam and USA MarketScan	Reference	0.13	---	---
	MEPS	Alternative		–0.93 (–1.19 to –0.67)	0.28
	USA MarketScan 2000	Alternative		0.02 (–0.24 to 0.28)	0.51
	No physical exam	Alternative		0.34 (0.06 to 0.62)	0.58

MR-BRT crosswalk adjustments can be interpreted as the factor the alternative case definition is adjusted by to reflect what it would have been had it been measured using the reference case definition. If the log/logit beta coefficient is negative, then the alternative is adjusted up to the reference. If the log/logit beta coefficient is positive, then the alternative is adjusted down to the reference.

*****The adjustment factor column is the exponentiated beta coefficient. For log beta coefficients, this is the relative rate between the two case definitions. For logit beta coefficients, this is the relative odds between the two case definitions.***

Modelling strategy

DisMod-MR 2.1, a Bayesian meta-regression tool, was used to estimate tinea capitis and other fungal skin diseases prevalence by age, sex, year, and geography (subnational, country, region, super-region). Separate models were run for tinea capitis and other fungal skin diseases.

Tinea capitis. To help inform the distribution of tinea capitis across the lifespan, excess mortality was set at zero, remission was set at 0.5 to 4, and incidence was set at 0 to 0.02 between 20 and 100 years. This was in agreement with the available prevalence data and expert advice. We made use of a relatively long time window of 20 years to determine which datapoints were used for a particular year of fit. This means that for the year 2000, for instance, DisMod-MR 2.1 incorporated all datapoints ranging from 1980 to present to estimate prevalence. Since GBD 2019, we replaced our within-DisMod crosswalks with crosswalks completed using the MR-BRT modelling tool. We adjusted USA outpatient data toward the level of other prevalence datapoints, which were more representative of the general population. We limited random effects for sub-Saharan Africa (–1,1), north Africa and the Middle East (–1, 1), southeast Asia, east Asia, and Oceania (–1, 1), and western Europe (–0.1, 1) to improve model estimates. In addition, Socio-

demographic Index and the Healthcare Access and Quality Index were used as country-level covariates to guide estimates for countries with few or no data.

Other fungal skin diseases. The modelling strategy was similar to that for tinea capitis, with remission set between 0.33 and 4. In GBD 2021, we replaced our within-DisMod crosswalks with crosswalks completed using the MR-BRT modelling tool. We adjusted Medical Expenditure Panel Survey (MEPS) datapoints, USA MarketScan data from 2000, and literature data that were not based on a physical exam toward the level of other prevalence datapoints which were more representative of the general population. We limited random effects for Nigeria (–0.5, 0.5) and Ethiopia (–0.5, 0.5) to improve model estimates.

We have made no substantive changes in the modelling strategy from GBD 2019.

Table 3. Severity distribution, details on the severity levels for fungal skin diseases and the associated disability weight (DW) with that severity

Severity level	Lay description	DW (95% CI)
Infectious disease, acute episode, mild	The person has a low fever and mild discomfort but no difficulty with daily activities.	0.006 (0.002–0.012)

Table 4. Covariates. Summary of covariates used in the fungal skin diseases DisMod-MR meta-regression model

Cause	Covariate	Type	Parameter	Exponentiated beta (95% uncertainty interval)
Tinea capitis	Socio-demographic Index	Country-level	Prevalence	5.41 (5.16–5.69)
	Healthcare Access and Quality Index	Country-level	Prevalence	0.96 (0.96–0.96)

Gallbladder and biliary diseases

Flowchart

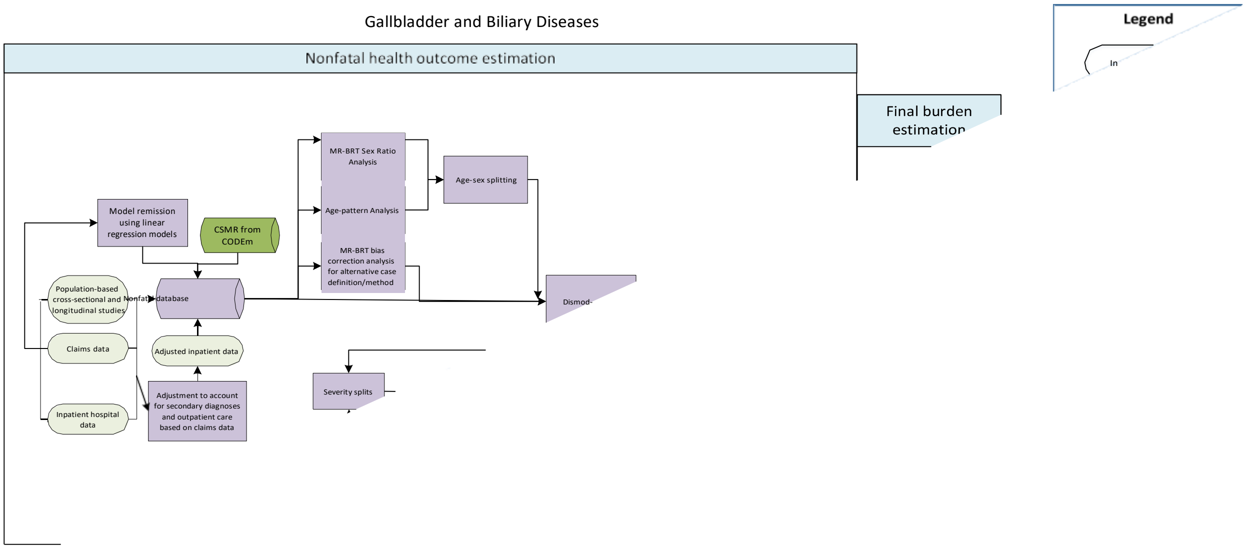

Input data and methodological summary for gallbladder and biliary diseases

Case definition

Gallbladder and biliary diseases encompass gallstones, cholecystitis, cholangitis, and other non-cancer diseases of the gallbladder and biliary tract, including those with and without symptoms. Gallstones are crystalline masses formed abnormally in the gallbladder or bile ducts from bile pigments, cholesterol, or calcium salts, which can cause abdominal pain. Cholecystitis is an inflammation of the gallbladder, and cholangitis is an inflammation of the bile duct, which can result from obstruction by gallstones and cause severe abdominal pain, nausea, vomiting, fever, and jaundice.

ICD-10 codes for gallstone and biliary diseases included in GBD are K80, K81, K82, and K83. The procedure codes used to identify remission of gallbladder and biliary diseases are 47400-47480, 47490-47544, 47550-47556, 47562-47579, 47600-47715, 47720-47900, 47999-47999.

Overall strategy

In GBD 2017, two databases were created for gallbladder and biliary diseases to separately model total (symptomatic + asymptomatic cases) and symptomatic cases. In GBD 2019, the DisMod-MR model for symptomatic cases was dropped, and we only modelled total cases of gallbladder and biliary diseases in DisMod-MR; an updated severity distribution was then applied as described below. This GBD 2019 approach was carried forward in GBD 2021.

Input data and data processing

Inputs

A systematic review was conducted to identify data on the prevalence of total gallbladder and biliary disease for GBD 2016. The search string used was ((gall bladder disease[Title/Abstract] OR cholecyst*[Title/Abstract] AND prevalence[Title/Abstract] AND ("2010/01/01"[Date - Publication] : "2016/11/01"[Date - Publication])) NOT(animals[MeSH] NOT humans[MeSH])). We excluded studies that were not representative of a general population described by year, age, sex, and location (ie, studies of clinically defined subpopulations such as *H. pylori* cohorts or patients presenting with pain) and without sufficient information on study and sampling methods. We also excluded reviews.

In addition to data from the search-string-based review, input data for the total model included clinical administrative data from the GBD clinical informatics datasets, which were extracted as prevalence. In GBD 2021, we newly added additional years of data from USA claims (year 2017) and Poland claims (year 2018), as well as hospital discharges in Greece, Armenia, Chile, Ecuador, Argentina, Italy, Brazil, and Spain.

Table 1. Data inputs for gallbladder and biliary diseases morbidity modelling by parameter

	**Countries with data**	**New sources**	**Total sources**
Prevalence	57	56	373
Other	1	0	15

Inputs to our non-fatal modelling also included cause-specific mortality rate (CSMR) estimates taken from our fatal modelling process (see CoD cause-specific modelling description for gallbladder and biliary diseases in this appendix) and remission estimates modelled outside of DisMod-MR (see the remission data processing section below).

Prevalence input processing

Hospital discharge data provide observations about encounters, generally with only the primary diagnostic code for the encounter. Claims data, on the other hand, link claims for all inpatient and outpatient encounters for a single individual and provide primary and secondary diagnoses for all encounters.

The data processing approach was largely similar to GBD 2019. Specifically, we extracted prevalent cases for the total gallbladder and biliary disease database from claims data in the same manner as in GBD 2019—extracting prevalent cases from claims data if an individual had one inpatient or two outpatient encounters with a gallbladder and biliary disease ICD code as any diagnosis. Hospital discharge data were processed by extracting discharges with an appropriate ICD code as primary diagnosis and adjusting using correction factors (ie, correction factor 3) derived from claims data. Specifically, we modelled from the ratio of inpatient claims with gallbladder and biliary diseases as primary diagnosis to total prevalent cases of gallbladder and biliary diseases seen in claims data.

In GBD 2017, the total model utilised ICD-code-based clinical administrative data as the reference standard. In GBD 2019 and GBD 2021, we improved our reference case definition, employing data from studies in which general population samples were screened for both symptomatic and asymptomatic cases of gallbladder and biliary diseases using ultrasonography. Claims and hospital discharge data were adjusted toward this new reference standard to account for systematic differences prior to modelling in

DisMod-MR. The USA claims data from the year 2000 and from the years 2010–2017 were separately adjusted to account for selection bias due to commercial insurance.

The process of adjusting for biases in non-reference data using MR-BRT (meta-regression—Bayesian, regularised, trimmed) with the logit-transformation method is described below:

1. Identify datapoints with overlapping year, age, sex, and location between non-reference data and reference data.
2. Logit-transform overlapping datapoints of alternative and reference types.
3. Convert overlapping datapoints into a difference in logit space using the following equation: $logit(alternative) - logit(reference)$.
4. Use the delta method to compute standard errors of overlapping datapoints in logit space, then calculate standard error of logit difference using the following equation: $\sqrt{(variance\ of\ alternative) + (variance\ of\ reference)}$.
5. Using MR-BRT, conduct a random effects meta-regression to obtain the pooled logit difference of alternative to reference.
6. Apply the pooled logit difference to all datapoints of alternative case definitions using the following equation: $new_{estimate} = inverse.\,logit((logit(alternative)) - (pooled\ logit\ difference))$.
7. Calculate new standard errors using the delta method, accounting for gamma (between-study heterogeneity).

The table below shows bias correction factors estimated using MR-BRT.

Table 2. MR-BRT crosswalk adjustment factors for gallbladder and biliary diseases

Data input	Reference or alternative case definition	Gamma	Beta coefficient, logit (95% CI)	Adjustment factor*
Ultrasound-based diagnosis	Ref	0.008	---	---
Hospital + non-USA claims	Alt		–3.01 (–3.11 to –2.92)	0.05 (0.045 to 0.054)
USA claims from year 2000	Alt		–2.07 (–2.27 to –1.87)	0.13 (0.10 to 0.16)
USA claims from years 2010-2017	Alt		–2.40 (–2.61 to 2.20)	0.09 (0.07 to 0.11)

**MR-BRT crosswalk adjustments can be interpreted as the factor the alternative case definition is adjusted by to reflect what it would have been had it been measured using the reference case definition. If the log/logit beta coefficient is negative, then the alternative is adjusted up to the reference. If the log/logit beta coefficient is positive, then the alternative is adjusted down to the reference.*

***The adjustment factor column is the exponentiated beta coefficient. For log beta coefficients, this is the relative rate between the two case definitions. For logit beta coefficients, this is the relative odds between the two case definitions.*

We split datapoints where the age range was greater than 25 years using the global age pattern informed by the datapoints with fine age groups (ie, ages 5–9, 10–14, and 15–20…). We also split data reported for both sexes using the pooled sex-ratio estimated from studies that reported prevalence in males and

females separately. The ratio of female to male cases derived from MR-BRT analysis was 1.69 (95% UI: 1.07–2.68).

Datapoints with an age-standardised prevalence greater than two median absolute deviations from the median of the age-standardised prevalence for all inpatient and non-USA claims data were marked as outliers and excluded from analysis.

Remission processing

As first done in GBD 2019, we used remission data from the USA claims, defined as a number of people with procedure codes among all people with diagnosis of gallbladder and biliary diseases, and regressed against Healthcare Access and Quality (HAQ) Index and sex. This was to better inform DisMod-MR on the increasing pattern of remission with greater access to quality health care. In GBD 2021, we updated this by including an additional covariate, age, to capture age variations in remission. The results from the regression model were then used to predict remission estimates for each location, year, and sex for ages 0, 10, 20…100.

Figure 1. Predicted remission in function of age, sex, and HAQ Index

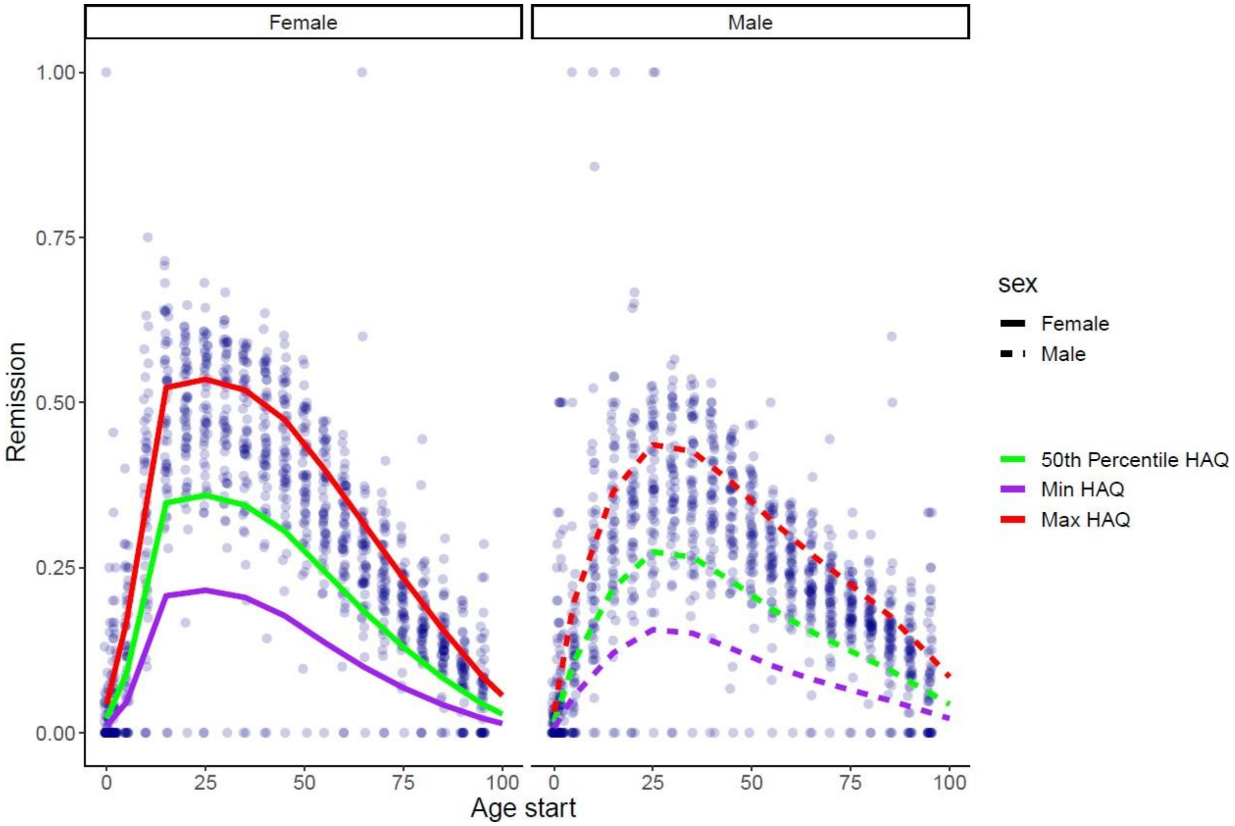

EMR processing

Similar to previous rounds, EMR inputs were produced inside DisMod-MR by matching prevalence datapoints with their corresponding CSMR values within the same age, sex, year, and location (by dividing CSMR by prevalence).

Modelling strategy

Modelling

We ran a DisMod-MR 2.1 model to produce estimates by age, sex, year, and country. Inputs to DisMod-MR for total gallbladder and biliary diseases include incidence, remission, and CSMR and EMR inputs processed as described above. The minimum coefficient of variation at the regional, super-regional, and global level was set at 0.8.

In previous rounds, we had applied a lag-distributed income covariate to EMR, log-transformed and forced negative with an upper bound of 0 and a lower bound of –1. The effect of the lag-distributed income covariate on EMR was found to be negligible (effect: 1.00 [95% UI: 1.00–1.00]). Therefore, we dropped this covariate from our model in GBD 2021. No other predictive covariates were added in the model.

Severity split and disability weight

The basis of the GBD disability weight survey assessments are lay descriptions of sequelae highlighting major functional consequences and symptoms. As in GBD 2019, cases from the total model were divided into asymptomatic and symptomatic groups using proportions found in a review of six studies of the natural history of gallbladder and biliary diseases and modelled in MR-BRT. Symptomatic cases of gallbladder and biliary diseases were then divided according to severity distributions derived from data from the Medical Expenditure Panel Survey (MEPS) to assign them to mild, moderate, andsevere sequelae. Asymptomatic cases were assigned no disability. The lay descriptions and disability weights for gallbladder and biliary diseases are shown below.

Table 3. Severity distribution, details on the severity levels for gallbladder and biliary diseases in GBD 2021 and the associated disability weight (DW) with that severity

Severity level	Lay description	DW (95% CI)
Asymptomatic	--	0
Mild	This person has some pain in the belly that causes nausea but does not interfere with daily activities.	0.011 (0.005–0.021)
Moderate	This person has pain in the belly and feels nauseous. The person has difficulties with daily activities.	0.114 (0.080–0.159)
Severe	This person has severe pain in the belly and feels nauseated. The person is anxious and unable to carry out daily activities.	0.324 (0.219–0.442)

The severity distribution of gallbladder and biliary disease was derived from analysis of the MEPS. MEPS is an overlapping panel survey of the non-institutionalised USA population that collects data on respondents' health service interactions. Panels are initiated every year. Each panel is two years long and consists of five rounds. In 2000, MEPS began using 12-Item Short Form Surveys (SF-12) to collect data on functional health status. The SF-12 survey is administered twice per panel (about once per year).

In order to translate SF-12 scores into GBD disability weights, 62 lay descriptions for conditions representing the full range of disability weight values (from mildest to most severe) were selected. A convenience sample of respondents was then asked to complete an SF-12 form for an individual with the health state described in the lay descriptions of these conditions. Composite mental and physical SF-12 score was regressed on GBD disability weight to derive the relationship between disability weight and SF-12 score. Individual respondent scores were then regressed on reported conditions to obtain a comorbidity-corrected condition-specific disability weight. The distribution of these condition-specific weights was used to derive the proportion of individuals with the conditions that fall within each GBD severity category.

Severity	Distribution
Asymptomatic	0.739 (0.566 – 0.966)
Mild acute urolithiasis	0.153 (0.122 – 0.183)
Moderate acute urolithiasis	0.065 (0.042 – 0.086)
Severe acute urolithiasis	0.043 (0.031 – 0.054)

Gastritis and duodenitis

Flowchart

Gastritis and duodenitis

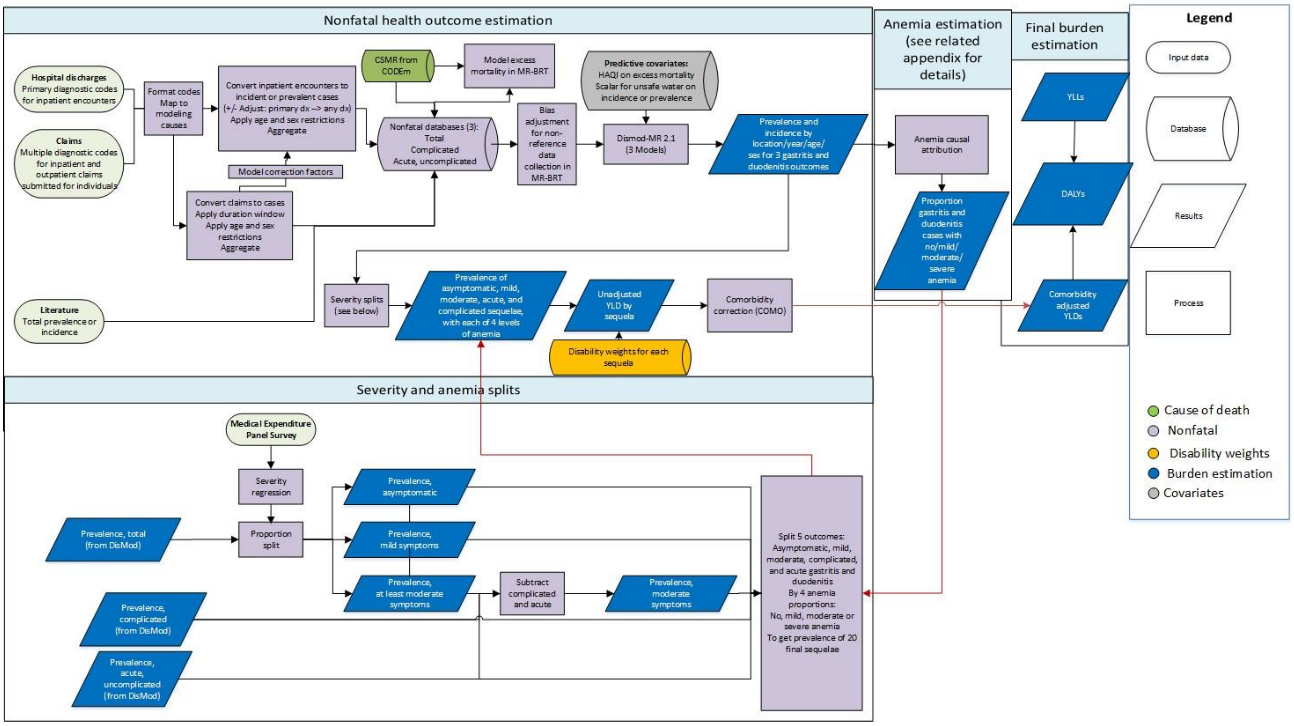

Case definition

Although gastritis and duodenitis refer to inflammation of the mucosal lining of the stomach and duodenum, respectively, we adopt the common practice of using these terms to describe gastropathy and duodenopathy, meaning any form of injury to the mucosal lining of the stomach and duodenum, be it inflammatory (such as due to infection or autoimmune disease) or otherwise (such as due to nonsteroidal anti-inflammatory medications), regardless of symptoms.

In practice and in the GBD, both inflammatory and non-inflammatory mucosal damage are classified together as gastritis and duodenitis. These entities exclude cases of damage that extends through the muscularis mucosa, which defines peptic ulcer disease, which is estimated and described separately.

Gold-standard diagnosis of gastritis and duodenitis is by biopsy, although endoscopic visualisation and a number of biochemical and microbiological tests have good predictive value. Gastritis and duodenitis can acutely produce severe symptoms, or have a subtle onset and evolve into a chronic illness characterised by asymptomatic periods and periods of abdominal pain, bloating, nausea, and early satiety. Complications such as haemorrhage may develop. Chronic gastritis is associated with elevated risk of gastric cancer.

In GBD 2021, gastritis and duodenitis were defined by diagnostic codes as described below. The ICD-10 code for gastritis and duodenitis is K29. ICD-10 codes for complicated gastritis and duodenitis are K29.01, K29.21, K29.31, K29.41, K29.51, K29.61, K29.71, K29.81, K29.91. ICD-10 codes for acute gastritis are K29.0, K 29.00, K29.1, K29.2, and K29.20. Equivalent ICD-9 codes were used where appropriate.

Overall strategy

As in GBD 2017 and GBD 2019, the GBD 2021 non-fatal estimation strategy for gastritis and duodenitis consisted of:

- Estimating the prevalence of total gastritis and duodenitis
- Dividing the total prevalent cases into asymptomatic, mild, and at least moderate severity levels
- Separately estimating the prevalence of gastritis and duodenitis with complication
- Separately estimating the prevalence of gastritis and duodenitis, acute, without complication (but with sufficient severity to require hospitalisation)
- Subtracting prevalent cases of gastritis and duodenitis with complication and gastritis and duodenitis, acute, without complication (but with sufficient severity to require hospitalisation) from prevalent cases of gastritis and duodenitis of at least moderate severity
-

Input data and data processing

Data sources

As in previous rounds, our GBD 2021 gastritis and duodenitis models relied primarily on data from hospital discharges and claims. In GBD 2021, we newly added additional years of data from USA claims (year 2017) and Poland claims (year 2018), as well as hospital discharges in Greece, Armenia, Chile, Ecuador, Argentina, Italy, Brazil, and Spain.

Additional sources of data for gastritis and duodenitis included peer-reviewed publications identified via systematic reviews of the literature conducted using recognised search engines (PubMed, Embase) for previous rounds of GBD, most recently GBD 2016. In brief, to be included, studies from all sources needed to:

1) Report a standard epidemiological measure (incidence, prevalence, case-fatality ratio, standardised mortality rate, etc.) of gastritis, duodenitis, or both.
2) Provide sufficient information on study methods and sample characteristics to assess its quality and make appropriate adjustments.
3) Use a gold-standard case definition based onendoscopy and biopsy, or use a well-defined alternative case definition that could be adjusted toward a reference standard.
4) Be conducted in a representative sample of a general population defined only by year, age, sex, and location, or be conducted in a representative sample of a well-defined sub-population for which valid adjustments could be made, or ascertain all cases for a defined catchment area for which GBD population estimates are available.

As in GBD 2019, the GBD 2021 gastritis and duodenitis modelling strategy used three separate databases: total gastritis and duodenitis, gastritis and duodenitis with complication (such as haemorrhage), and gastritis and duodenitis, acute, without complication (but with sufficient severity to require hospitalisation). The total gastritis and duodenitis dataset included data from hospital discharges and claims coded with any gastritis or duodenitis ICD code, as well as data from peer-reviewed publications. The gastritis and duodenitis with complication dataset included hospital discharges and inpatient claims with ICD codes specifying the occurrence of complications. The gastritis and duodenitis, uncomplicated, acute dataset included only hospital discharges and inpatient claims with ICD codes specifying that a complication did not occur.

Data inputs for gastritis and duodenitis morbidity modelling by parameter

Measure	Total sources	New sources	Countries with data
All measures	369	34	49
Prevalence	327	34	48
Incidence	297	34	28
Other	15	0	1

Inputs to our non-fatal modelling also included cause-specific mortality rate (CSMR) estimates taken from our fatal modelling process (see CoD cause-specific modelling description for appendicitis in this appendix) and excess mortality rates (EMR) estimates modelled outside of DisMod (see the EMR data processing section below).

Prevalence and incidence data processing

The extraction and processing of prevalence and incidence data for gastritis and duodenitis were identical in GBD 2021 and GBD 2019. The preponderance of these data came from claims and hospital discharges. Hospital discharge data provide observations about encounters, generally with only the primary diagnostic code for the encounter. Claims data, on the other hand, link claims for all inpatient and outpatient encounters for a single individual and provide primary and secondary diagnoses for all encounters.

For the total gastritis and duodenitis database, an individual was extracted from claims data as a prevalent case if they had any gastritis and duodenitis ICD code as any diagnosis in one or more inpatient encounters or two or more outpatient encounters. Hospital discharges were extracted if an appropriate ICD code appeared as the primary discharge diagnosis, and the discharges were then adjusted using a correction factor estimated from claims data. Specifically, the correction factor (known as cf3) was modelled as the ratio of inpatient claims with an appropriate primary diagnostic code to all prevalent cases (inpatient and outpatient) in claims data, using MR-BRT.

For the gastritis and duodenitis with complication dataset and the gastritis and duodenitis, uncomplicated, acute dataset, individuals were extracted from claims as incident cases if they had an inpatient claim with an appropriate ICD code as any diagnosis. These incident cases were extracted linking multiple encounters for an individual and assuming multiple claims within a 60-day window represented a single incident case, and multiple claims separated by more than 60 days represented separate episodes of illness and, thus, additional incident cases. Hospital discharges were extracted if an appropriate ICD code appeared as the primary diagnosis, and the discharges were then adjusted using a correction factor estimated from claims data. Specifically, the correction factor (known as cf2), was modelled as the ratio of inpatient claims with an appropriate primary diagnostic code to all incident (inpatient) cases in claims data, using MR-BRT.

Details of the extraction, utilisation envelope, and correction factor models used to process hospital discharge and claims data for gastritis and duodenitis are found in the "Claims, inpatient hospital and outpatient data" section of the appendix to the GBD 2019 Diseases & Injuries report.[1]

Epidemiological measurements from peer-reviewed publications were manually extracted for the most granular age-sex groups reported, with a measure of uncertainty and information on the study design.

Extracted measures were marked with dichotomous variables to indicate alternative (non-reference) case definitions, study populations, or other study design features. Where a single study reported measures using more than one case definition, multiple measurements were extracted to create paired data for modelling adjustment factors.

For total gastritis and duodenitis, we sought to use a gold-standard case definition of endoscopy without clinical indication, and to develop adjustments for alternative case definitions of endoscopy with clinical indication, serology (pepsinogen), diagnostic code in administrative data (such as hospital discharges or claims), and self-reported diagnosis (current or with 12-month recall). Unfortunately, only a single study in our database used endoscopy to survey for gastritis in a general population selected without regard to symptoms, two used endoscopy performed only in symptomatic persons, eight used serology, and four used self-report; among these, a total of three matches in year, age, sex, and location were observed between the studies, and no matches were observed between any of these data types and data from administrative sources. Thus, valid adjustments toward the gold-standard definition could not be estimated, we dropped the endoscopy-based data, and we adopted diagnostic code in administrative data as our reference case definition.

A pre-modelling adjustment was made to account for the fact that claims data from the USA only cover a commercially insured sub-population. Commercial claims data were available for all 51 USA subnational locations and matched hospital discharge data covering the general population for one or more years were available for 24 USA subnational locations. Thus, 24 sets of paired data were used as inputs to a model of the difference in logit prevalence between alternative (commercially biased) and reference (general population) data in MR-BRT. The estimated mean logit differences were applied to USA claims data as bias correction prior to modelling in DisMod-MR 2.1 (below).

The process of adjusting alternative data types using MR-BRT with the logit-transformation method is described below:

36. Identify datapoints with overlapping year, age, sex, and location between commercial claims (alternative data) and hospital discharges (reference data).
37. Logit-transform overlapping datapoints of alternative and reference types.
38. Convert overlapping datapoints into a difference in logit space using the following equation: $logit(alternative) - logit(reference)$.
39. Use the delta method to compute standard errors of overlapping datapoints in logit space, then calculate standard error of logit difference using the following equation: $\sqrt{(variance\ of\ alternative) + (variance\ of\ reference)}$.
40. Using MR-BRT, conduct a random effects meta-regression to obtain the pooled logit difference of alternative to reference.
41. Apply the pooled logit difference to all datapoints of alternative case definitions using the following equation: $new_{estimate} = inverse.\ logit((logit(alternative)) - (pooled\ logit\ difference))$.
42. Calculate new standard errors using the delta method, accounting for gamma (between-study heterogeneity).

The table below shows bias correction factors estimated using MR-BRT.

MR-BRT crosswalk adjustment factors for total gastritis and duodenitis

Data input	Reference or alternative data collection	Gamma	Beta coefficient, logit difference (95% CI)	Adjustment factor*
Hospital + non-USA claims	Reference	0.83	---	---
USA claims from year 2000	Alternative		–0.44 (–2.7 to 1.9)	0.39 (0.066 to 0.87)
USA claims from years 2010–2016	Alternative		–0.030 (–1.7 to 1.7)	0.49 (0.15 to 0.85)

For gastritis and duodenitis with complication, and gastritis and duodenitis, uncomplicated, acute, only administrative data were available. Pre-modelling adjustments were made to data from commercial claims, using an approach similar to that described above for total peptic ulcer disease data.

MR-BRT crosswalk adjustment factors for gastritis and duodenitis with complication

Data input	Reference or alternative data collection	Gamma	Beta coefficient, logit difference (95% CI)	Adjustment factor*
Hospital + non-USA claims	Reference	0.16	---	---
USA claims from year 2000	Alternative		–0.42 (–0.89 to 0.054)	0.40 (0.29 to 0.51)
USA claims from years 2010–2016	Alternative		–0.24 (–0.57 to 0.093)	0.44 (0.36 to 0.52)

MR-BRT crosswalk adjustment factors for gastritis and duodenitis, uncomplicated, acute

Data input	Reference or alternative data collection	Gamma	Beta coefficient, logit difference (95% CI)	Adjustment factor*
Hospital + non-USA claims	Reference	0.21	---	---
USA claims from year 2000	Alternative		0.29 (–0.29 to 0.87)	0.57 (0.43 to 0.71)
USA claims from years 2010–2016	Alternative		–0.072 (–0.51 to 0.36)	0.48 (0.37 to 0.59)

**Adjustment factor is the inverse-logit-transformed beta coefficient; <0.5 represents that alternative is adjusted upward; >0.5 represents that alternative is adjusted downward.*

After adjustment, for each source-location-year-sex combination, age-standardised mean was calculated, and the data series was excluded if this was zero or was greater than two times the median absolute deviation above or below the median for the database.

EMR processing
EMR inputs have evolved in recent rounds of GBD. In GBD 2017, EMR inputs were produced by matching total gastritis and duodenitis prevalence datapoints with their corresponding CSMR values within the

same age, sex, year, and location (by dividing CSMR by prevalence). However, this method of producing EMR inputs demonstrated a rather unrealistic pattern of EMR compared to an expected pattern of decreasing EMR with greater access to quality health care. (Such unexpected patterns often signal inconsistencies between CSMR estimates and the measures of prevalence and/or incidence.) Thus, in an effort to provide greater guidance on the expected pattern of EMR, in GBD 2019, EMR data produced as above in GBD 2017 were modelled by age, sex, and Healthcare Access and Quality (HAQ) Index using MR-BRT, with a prior on HAQ Index having a negative coefficient. We then predicted EMR for each country, year, sex, and for ages 0, 10, 20….100. These predictions were used as inputs to our total gastritis and duodenitis DisMod model in GBD 2019 and GBD 2021.

Modelling strategy

Total gastritis and duodenitis, symptomatic and asymptomatic

Similar to previous rounds, we ran a DisMod-MR 2.1 model to produce estimates by age, sex, year, and location. Inputs to DisMod for total gastritis and duodenitis included prevalence, CSMR, and EMR inputs processed as described above, and expert priors for other epidemiological measures.

Prior value of remission was bounded from 0 to 1 (a minimum duration of one year). The minimum coefficient of variation at the regional, super-regional, and global level was set at 0.8, and the time window of data to include for fitting was five years. We included HAQ Index as a predictive covariate on EMR with a mean and standard deviation produced from the MR-BRT model described above. Predictive covariates for alcohol consumption and access to safe water were applied to prevalence, which we forced positive with a lower bound of zero on the priors. Betas and exponentiated values (which can be interpreted as an odds ratio) are shown in the tables below for all predictive covariates in the DisMod model.

DisMod-MR 2.1 model covariates for total gastritis and duodenitis

Covariate	Parameter	beta	Exponentiated beta
Litres of alcohol per capita	Prevalence	0.00 (0.00 to 0.00)	1.00 (1.00 to 1.00)
Scaled exposure variable for unsafe water	Prevalence	0.75 (0.75 to 0.75)	2.12 (2.12 to 2.13)
Healthcare Access and Quality Index	Excess mortality	–0.033 (–0.033 to –0.032)	0.97 (0.97 to 0.97)

Complicated gastritis and duodenitis

The DisMod model for complicated gastritis and duodenitis included incidence data as described above. The prior value of incidence was bounded to 0 to 0.3, the prior value of EMR was bounded to 0.1 to 10, and the prior value of remission was bounded to 6 to 13 cases of remission per person-year (disease duration 4 to 8.7 weeks). A location-level covariate for HAQ Index was applied to EMR, and location-level covariates for the log-transformed age-standardised death rate due to gastritis and duodenitis and unsafe water access were applied to incidence. Random effects for all super-regions except for the high-income super-region were bounded to –0.25 to 0.25. Betas and exponentiated values (which can be interpreted as odds ratios) are shown in the table below for all covariates.

DisMod-MR 2.1 model covariates for gastritis and duodenitis with complication

Covariate	Parameter	beta	Exponentiated beta
Natural log of age-standardised death rate	Incidence	0.00 (0.00 to 0.00)	1.00 (1.00–1.00)
Scaled exposure variable for unsafe water access	Incidence	0.00 (0.00 to 0.00)	1.00 (1.00–1.00)
Healthcare Access and Quality Index	Excess mortality rate	–0.5 (–0.99 to –0.017)	0.61 (0.37–0.98)

Acute gastritis and duodenitis, without complication

The DisMod model for acute, uncomplicated gastritis and duodenitis included incidence data as described above. The prior value on incidence was set to 0 through age 5 years, the range of prior values on EMR was bounded to 0 to 0.1, and the range of prior values on remission was bounded to 6 to 13 cases per person-year. Location-level covariates were applied for log-transformed, lag-distributed income (on excess mortality rate), log-transformed age-standardised death rate due to gastritis and duodenitis (on incidence), and per capita alcohol consumption (on incidence). Betas and exponentiated values (which can be interpreted as odds ratios) are shown for these covariates in the tables below.

DisMod-MR 2.1 model covariates for gastritis and duodenitis, uncomplicated, acute

Covariate	Parameter	beta	Exponentiated beta
Log-transformed lag-distributed income	Excess mortality rate	–0.5 (–0.97 to –0.00)	0.61 (0.38–1.00)
Natural log of age-standardised death rate	Incidence	0.00 (0.00 to 0.00)	1.00 (1.00–1.00)

Severity split & disability weight

The basis of the GBD disability weight survey assessments are lay descriptions of health states highlighting major functional consequences and symptoms.

Prevalence draws from the total gastritis and duodenitis model were divided into asymptomatic, mild, and at least moderate severity levels using proportions derived from the Medical Expenditure Panel Survey (MEPS). It must be noted that the MEPS analysis uses quality-of-life data from individuals who had a health care encounter for gastritis and duodenitis within the preceding 12 months and were interviewed about their quality of life in the preceding four weeks, so the asymptomatic proportion represents those with diagnosed disease who were asymptomatic in a given period of time, not those always asymptomatic who may have gastritis and duodenitis on lab tests or endoscopy if examined for study or screening purposes. After dividing the total prevalence draws by these three proportions, the complicated and uncomplicated acute prevalence draws were subtracted from the at least moderate draws.

The asymptomatic, mild, and remaining moderate prevalent cases were then assigned the following lay descriptions and disability weights.

Severity level	Lay description	DW (95% CI)

Diagnosed gastritis and duodenitis, not in a symptomatic episode	--	0
Mild gastritis and duodenitis episode	This person has some pain in the belly that causes nausea but does not interfere with daily activities.	0.011 (0.005–0.021)
Moderate gastritis and duodenitis episode	This person has pain in the belly and feels nauseous. The person has difficulties with daily activities.	0.114 (0.080–0.159)

Gastritis and duodenitis, with complication, and gastritis and duodenitis, uncomplicated, acute, were then assigned the following lay descriptions and disability weights.

Severity level	Lay description	DW (95% CI)
Gastritis and duodenitis, with complication	This person vomits blood and feels nauseous.	0.325 (0.209–0.462)
Gastritis and duodenitis, acute, uncomplicated	This person has severe pain in the belly and feels nauseous. The person is anxious and unable to carry out daily activities.	0.324 (0.220–0.442)

These five final health states were then combined with health states for anaemia. Methods for causal attribution of anaemia due to gastritis and duodenitis can be found in the "Impairment and underlying cause estimation" and the "Non-fatal cause-specific modelling description" titled "Anaemia".

1. Vos T, Lim SS, Abbafati C, et al. Global burden of 369 diseases and injuries in 204 countries and territories, 1990–2019: a systematic analysis for the Global Burden of Disease Study 2019. *The Lancet*. 2020;396(10258):1204-1222. doi:10.1016/S0140-6736(20)30925-9

Gastro-oesophageal reflux disease

Flowchart

Gastroesophageal reflux disease

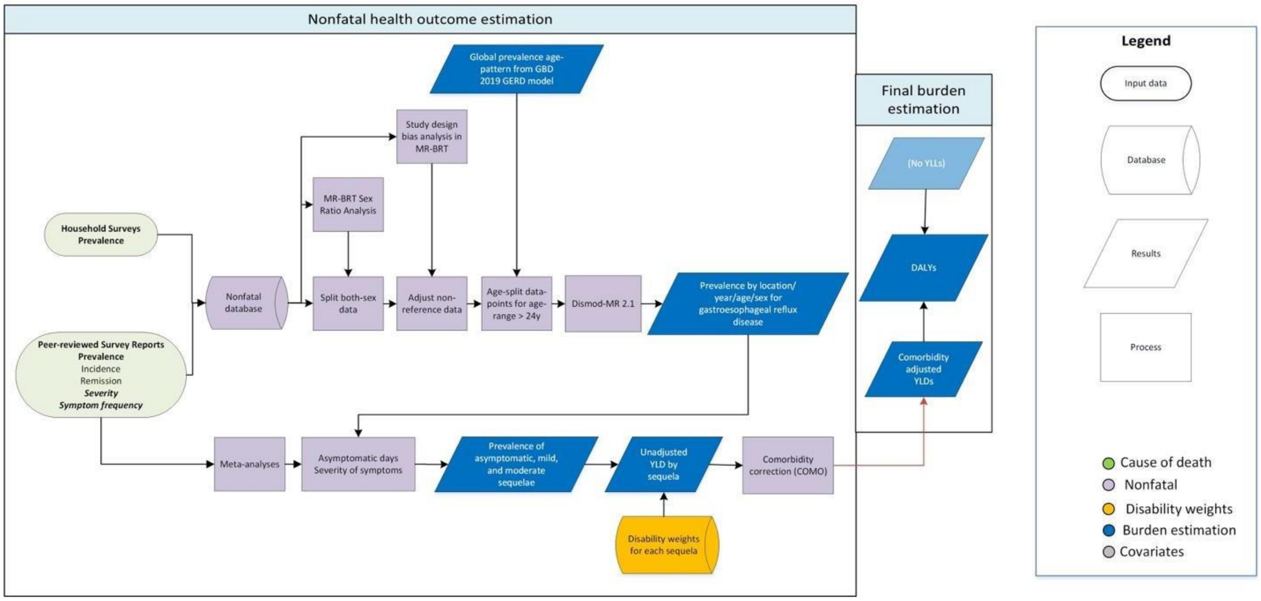

Case definition

Gastro-oesophageal reflux disease (GORD) is a chronic condition that results when the reflux of stomach contents causes troublesome symptoms, complications, or both. The cardinal symptoms of typical GORD are heartburn (a burning feeling behind the breastbone) and regurgitation (the unpleasant sensation of material moving upward from the stomach toward the mouth).

In GBD, the occurrence of heartburn, regurgitation, or both, at least once weekly over a 12-month recall period is employed as the reference case definition.

Individuals who experience oesophageal complications (ulceration, metaplasia, etc.) without symptoms, whose sole symptom of gastro-oesophageal reflux is chest pain without typical reflux symptoms, or who experience reflux primarily as a trigger or exacerbating factor in respiratory or head and neck diseases (chronic cough, dental erosion, etc.) were not included. This strategy avoids double-counting disability already attributed to other underlying diseases modelled in GBD. Likewise, we regarded newborn reflux as a separate disease, which is modelled elsewhere and excluded from this analysis.

Input data and processing

Data inputs and processing for GORD in GBD 2021 are unchanged from GBD 2019.

Data inputs

Data inputs for estimating the prevalence of GORD in GBD 2021 came from a systematic review conducted for GBD 2017. In brief, peer-reviewed publications reporting epidemiological measures of GORD were identified via a search-string-based review in PubMed, citations of those articles identified by search-string, and suggestions from the GBD Collaborator Network. Two household surveys – the USA National Health Interview Surveys in 2007 and 2012 – were identified from the Global Health Data Exchange as asking participants about the occurrence of typical reflux symptoms, and were also included. In brief, data from all sources had to:

1) Report a standard epidemiological measure (incidence, prevalence, case–fatality ratio, standardised mortality rate, etc.) of GORD or provide individual-level data from which one could be calculated.
2) Provide sufficient information on study methods and sample characteristics to assess its quality and make appropriate adjustments.
3) Use our reference case-definition, or use a well-defined alternative case-definition that could be adjusted toward our reference standard.
4) Be conducted in a representative sample of a general population defined only by year, age, sex, and location, or be conducted in a representative sample of a well-defined sub-population for which valid adjustments could be made, or ascertain all cases for a defined catchment area for which GBD population estimates are available.
5) Provide information on uncertainty (sample size, standard deviation, or confidence interval) and follow-up time.
6) Be written in a language that the modelling team could read (English, French, Portuguese, or Spanish).

In our search, all studies reporting incidence or remission of GORD provided insufficient information on person-time of observation and were excluded, so only prevalence data were included. Data from claims data extracted and prepared for GBD as described in the "Claims, inpatient hospital and outpatient data" section of the appendix to the GBD 2019 Diseases & Injuries report[1] were used to develop adjustment factors for published studies from the search-string-based review that ascertained cases based on diagnostic codes in administrative data but were not used in the primary analysis of GORD prevalence.

Prevalence measurements from peer-reviewed publications were manually extracted for the most granular age-sex groups reported, with a measure of uncertainty and information on the study design. Prevalence estimates were extracted from individual-level data from two household surveys using questionnaire text, skip-pattern, and weights for complex sampling strategies provided in the documentation from original study investigators. Extracted measurements were marked with dichotomous variables to indicate alternative (non-reference) case definitions, study populations, or other study design features. Where a single study measured using more than one case definition, multiple measurements were extracted to create paired data for modelling adjustment factors.

Data inputs for GORD morbidity modelling by parameter

Measure	Total sources	Countries with data
Prevalence	110	37

Data processing

For studies that reported prevalence by age for both sexes combined, and prevalence by sex for all ages combined, we calculated the sex-ratio of cases in that study and applied it to the age-specific prevalence measures to estimate age-sex-specific prevalence.

To estimate sex-specific prevalence from studies that reported prevalence only for both sexes combined, we modelled the log sex ratio in MR-BRT using all sex-specific prevalence measurements from all other studies in the database: 0.24 (–0.23 to 0.70) and combined this with the GBD sex-specific population estimates for the relevant age group. These were applied by calculating male prevalence:

$$prev_{male} = prev_{both} * \frac{pop_{both}}{(pop_{male} + ratio * pop_{female})}$$

and then calculating female prevalence:

$$prev_{female} = ratio * prev_{male}$$

For GORD, 27 studies used our reference case definition. The remaining studies had one or more non-reference study design feature thought to systematically bias prevalence measurements: questionnaire only asked subjects about heartburn, questionnaire only asked subjects about regurgitation, case definition required subjects to have additional symptoms to qualify as having GORD (such as sleep disruption or sour taste in mouth), case definition allowed subjects to qualify as having GORD due to having symptoms other than heartburn and regurgitation, recall period was less than 12 months, case definition required more than weekly symptoms, case definition included those with less than weekly symptoms, case definition used a scoring system that integrated information on number, frequency, and duration of symptoms, or cases were identified based on diagnostic code in administrative data. These were modelled as independent effects in a network meta-analysis in MR-BRT, using 82 studies. Adjustments were modelled as difference in logit prevalence between alternative and reference data. The estimated mean logit differences were applied to non-reference data types as bias correction prior to modelling in DisMod-MR 2.1 (below).

The process of adjusting non-reference data using MR-BRT with the logit-transformation method is described below:

1. Mark all datapoints with dichotomous variables for all study design characteristics to be adjusted.
2. Identify datapoints with overlapping year, age, sex, and location that differ with regard to one or more study design characteristics.
3. Logit-transform prevalence estimates for all overlapping datapoints.
4. For all pair-wise combinations of overlapping datapoints, calculate the difference between prevalence estimates in logit space.
5. Use the delta method to compute standard errors of overlapping datapoints in logit space, then calculate standard error of logit difference.
6. Using MR-BRT, conduct a random effects meta-regression to estimate the logit difference of alternative to reference study designs, with covariates for each study design variable and no intercept.
7. Logit-transform the prevalence estimates for all data (not just points that overlap).

8. Transform the logit prevalence of each non-reference datapoint by subtracting the coefficients from MR-BRT for all applicable study design variables.
9. Calculate new standard errors using the delta method, accounting for gamma (between-study heterogeneity).

The table below shows bias correction factors for study design characteristics estimated using MR-BRT.

MR-BRT crosswalk adjustment factors for gastro-oesophageal reflux disease

Data input	Reference or alternative data collection	Gamma	Beta coefficient, logit difference (95% CI)	Adjustment factor*
Heartburn and/or regurgitation at least weekly for 12 months	Reference	0.61	---	---
Only asked about heartburn	Alternative		–0.61 (–2.1 to 0.92)	0.35 (0.11 to 0.72)
Only asked about regurgitation	Alternative		–0.26 (–1.8 to 1.3)	0.43 (0.14 to 0.78)
Required additional symptoms to meet case definition	Alternative		0.25 (–1.3 to 1.8)	0.56 (0.23 to 0.86)
Could meet case definition with other symptom options	Alternative		0.58 (–0.96 to 2.1)	0.64 (0.28 to 0.89)
Shorter recall period	Alternative		0.26 (–1.3 to 1.8)	0.56 (0.22 to 0.86)
Required greater minimum symptom frequency to meet case definition	Alternative		–1.2 (–2.7 to 0.35)	0.23 (0.063 to 0.59)
Had lower symptom frequency requirement to meet case definition	Alternative		0.89 (–0.63 to 2.4)	0.71 (0.35 to 0.92)
Used diagnostic score integrating multiple domains	Alternative		–0.027 (–1.6 to 1.5)	0.49 (0.17 to 0.82)
Diagnostic code in administrative data	Alternative		–1.7 (–3.2 to –0.13)	0.16 (0.039 to 0.47)

**Adjustment factor is the inverse-logit-transformed beta coefficient; <0.5 represents that alternative is adjusted upward; >0.5 represents that alternative is adjusted downward.*

Data sources that used non-reference study designs were dropped for which valid adjustments could not be developed: sampling of populations defined by profession (4), convenience sampling from waiting rooms (3), case definition limited to endoscopically confirmed erosive oesophagitis (1), and self-reported diagnosis without symptom-based questions (1).

Subsequently, datapoints for samples spanning 25 years of age or more were disaggregated by applying the age-pattern observed in the global fit for the GBD 2017 GORD model.

Specific datapoints from some sources from subnational locations were excluded if relatively high values in young age groups led to overestimation of the entire age range.

Modelling strategy

Compartmental DisMod model
A full compartmental model of GORD epidemiology was run using DisMod-MR 2.1. Adjusted prevalence data as processed for GBD 2019 and described above were the inputs. Parameter settings were unchanged from GBD 2019. Excess mortality rate was assumed a priori to be 0, and remission prior was set to 0.2 to 0.5 cases per person-year. Incidence was forced to 0 from birth to age 5 years, and after this age, prior was set to 0 to 0.2 cases per person-year. In previous rounds, we trialed covariates for mean body-mass index, prevalence of obesity, and per capita alcohol consumption, but these were not predictive, so were removed from the model.

Severity split & disability weight
Severity distributions and disability weights for GORD are unchanged from GBD 2019.

Throughout the literature, the severity of GORD is often divided into three or four categories, using definitions such as those in the table below. In GBD 2017, we reviewed the studies in our prevalence database, above, and, if provided, extracted counts of cases of each severity as reported. These cases were then mapped to one of two GBD GORD severities (also shown in the table below). These categories were mapped to GBD health states, which are associated with disability weights. The basis of the GBD disability weight survey assessments are lay descriptions of health states highlighting major functional consequences and symptoms, also shown below.

Sample mapping of reported GORD severity levels to GBD GORD severity levels

Literature severity levels	GBD severity level	Lay description
Mild: can be ignored	Mild/moderate	Often has a burning sensation in the back of the chest after eating
Moderate: cannot be ignored but does not affect lifestyle	Mild/moderate	Often has a burning sensation in the back of the chest after eating
Severe: affects lifestyle	Severe (abdom_mod)	Has pain in the belly* and feels nauseous. Has difficulty with daily activities.
Very severe: has marked effect on lifestyle	Severe (abdom_mod)	Has pain in the belly* and feels nauseous. Has difficulty with daily activities.

*We acknowledge that gastro-oesophageal reflux symptoms are felt in the chest, not the belly, but opine that a health state that incorporates other gastrointestinal symptoms and indicates interference with daily activities, such as difficulty eating and sleeping, better represents more severe gastro-oesophageal reflux disease than a health state that describes only post-prandial heartburn.

The proportion of cases in each of the GBD GORD severities was then estimated using the metafor package (version 2.0-0) in R (version 3.4). Inputs to this meta-analysis are shown below. In GBD 2017, all

studies with severity information for sample of cases defined by at least weekly symptoms were included, whether the defining symptoms were heartburn, regurgitation, either, or both, and regardless of recall period or duration; thus 15 studies were included. In GBD 2019, we limited the severity meta-analysis to only those studies that used the reference case definition of heartburn and/or regurgitation at least weekly for 12 months; thus, only four studies were included.

Meta-analysis of proportion severe/very severe for GORD

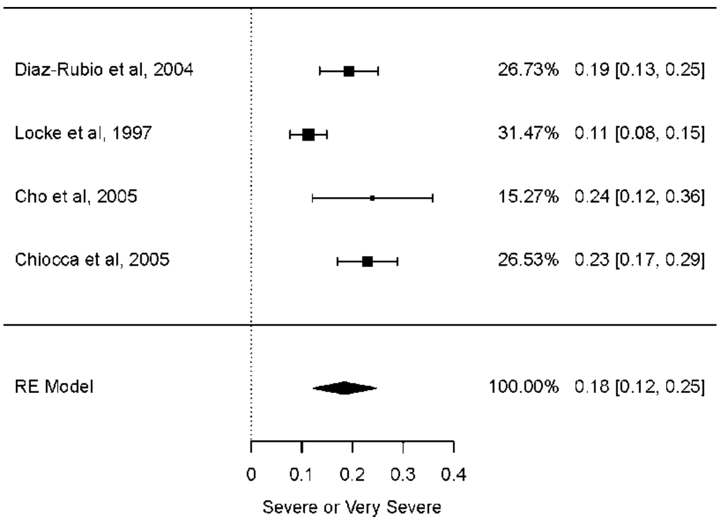

Many studies in the literature also report the frequency of GORD symptoms as the proportions of cases in each of a set of mutually exclusive and collectively exhaustive frequency categories. Examples include: 1–6 days/week and daily; 1 day/week, 2–6 days/week and daily; 1–3 days/week, 4–6 days/week, and daily; etc. For each study, for each frequency category, 1000 proportion draws were generated using a beta distribution with case counts in and out of the frequency category as shape parameters. We then assume that the number of days symptomatic within a category are uniformly distributed. We combine proportion draws and this assumption about mean days symptomatic in each category to produce draws of the mean number of days/week symptomatic across all cases in a study. Means and standard deviations of these draws are displayed in the forest plot below.

Meta-analysis of days/week spent symptomatic for GORD

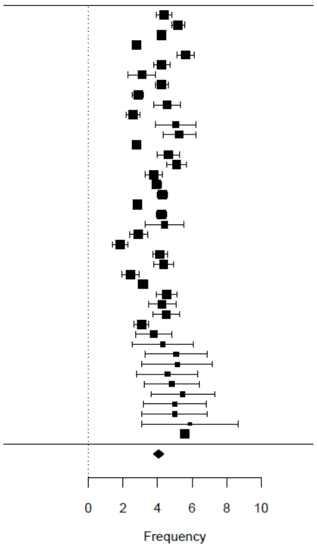

These inputs were then combined in a meta-analysis, and final mean and standard deviation were divided by seven to estimate the proportion of cases symptomatic on a given day, with uncertainty.

Severity and frequency categories were combined to generate four categories, as shown below.

GBD severity-frequency category	Proportion	Proportion	DW (95% CI)
Mild/moderate GORD, asymptomatic days	0.72 (0.71–0.74)	0.42 (0.38–0.46)	None
Mild/moderate GORD, symptomatic days		0.58 (0.54–0.62)	0.027 (0.015–0.046)
Severe GORD, asymptomatic days	0.28 (0.26–0.29)	0.42 (0.38–0.46)	None
Severe GORD, symptomatic days		0.58 (0.54–0.62)	0.114 (0.080–0.159)

Gout

Flowchart

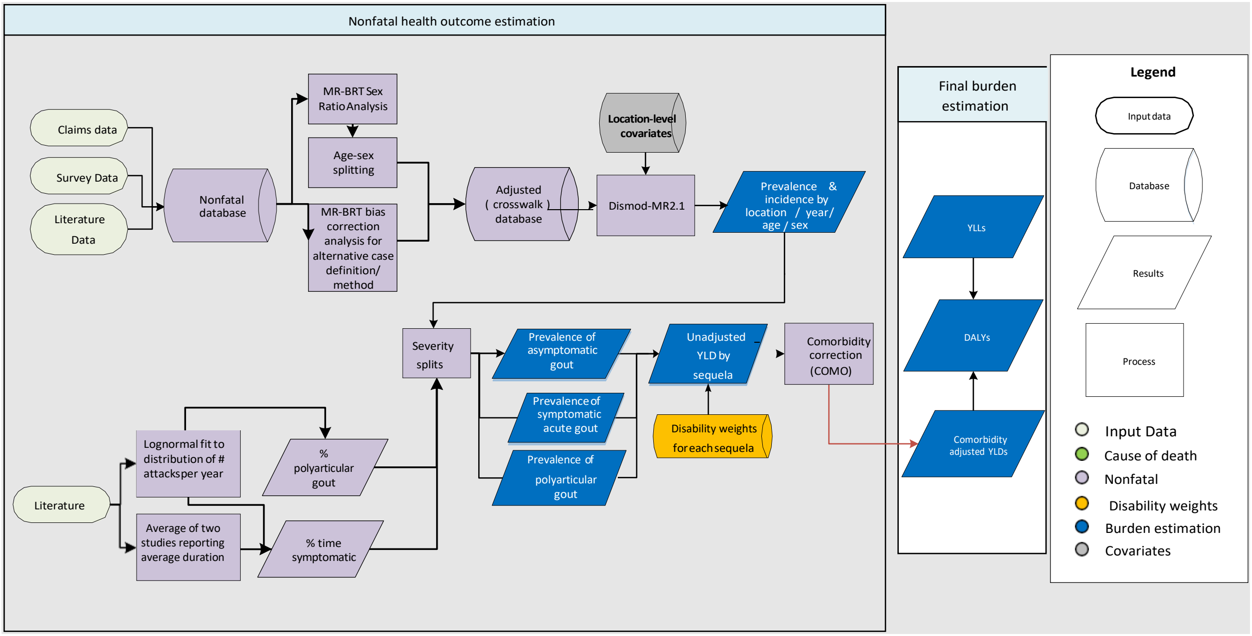

Input data and methodological summary for gout

Case definition

Gout is a rheumatic disease that is characterised by deposition of monosodium urate (MSU) crystals in the synovial fluid of joints and in other tissues, causing inflammation. The crystal formation is caused by elevated urate levels in extracellular fluids. Case definitions are found in the table below. The ICD-10 code for gout is M10 and the ICD9 code is 274.

Table 1. Case definitions for gout modelling

Reference or alternative	Definition
Reference	American College of Rheumatology 1977 (ARA 1977 or Wallace Criteria) survey criteria requiring the presence of MSU crystals in joint fluid or the presence of a tophus proven to contain MSU crystals and at least six of 12 gout symptoms or findings (>1 attack of acute arthritis, development of maximal inflammation within a day, attack of monarticular arthritis, observation of joint erythema, pain or swelling in the first MTP joint, unilaterally attack involving the first MTP joint, unilateral attack involving tarsal joint, suspected tophus, hyperuricemia, asymmetrical swelling within a joint on X-ray and negative culture of joint fluid for microorganisms during attack of joint inflammation) to make a diagnosis
Alternative	Self-reported diagnosis of gout
Alternative	USA claims data
Alternative	Taiwan claims data
Alternative	Physician diagnosis of gout, criteria unspecified
Alternative	Physician diagnosis of gout using diagnostic criteria other than the ARA 1977

Input data

The last systematic review was conducted in GBD 2013 for studies published between 1980 to 2009 using the following search terms on MEDLINE, EMBASE, CINAHL, CAB Abstracts, WHO Library (WHOLIS), and OpenSIGLE. For prevalence and incidence, the following search terms were used: (gout* OR hyperuricemia) AND (prevalen* OR inciden* OR cross-sectional OR cross sectional OR epidemiol* OR survey OR population-based OR population based OR population study OR population sample OR cohort OR follow-up OR follow up OR longitudinal OR regist*) AND (list of names of all GBD countries).

Exclusion criteria were:

- Sub-populations clearly not representative of the national population
- Not a population-based study
- Low sample size (less than 150)
- Review rather than original studies

For GBD 2019, 14 additional studies shared through the collaborator network were added. In addition, data from USA claims data for 2000 and 2010–2014 by state and Taiwan claims data from 2016 were included.

Table 2: Data inputs for gout

Measure	Total sources	Countries with data
All measures	131	35
Prevalence	114	34
Incidence	15	6
Other	11	4

Age and sex splitting

Reported estimates of prevalence were split by age and sex where possible. First, if studies reported prevalence for broad age groups by sex (eg, prevalence in 15- to 65-year-old males and females separately), and also by specific age groups for both sexes combined (eg, prevalence in 15- to 30-year-olds, then in 31- to 65-year-olds, for males and females combined), age-specific estimates were split by sex using the reported sex ratio and bounds of uncertainty. Second, prevalence data for both sexes that could not be split using a within-study ratio were split using a sex ratio derived from a meta-analysis of existing sex-specific data using MR-BRT (Meta-regression— Bayesian, regularised, trimmed). The female to male ratio was 0.33 (0.33 to 0.34). Finally, after the application of bias adjustments, where studies reported estimates across age groups spanning 25 years or more, these were split into five-year age groups using the prevalence age pattern estimated by DisMod-MR 2.1 (disease model—Bayesian meta-regression[1]) in GBD 2019.

Data adjustment

We used study covariates for studies relying on self-reported diagnoses and those identifying sources through a diagnostic code in administrative data, which include gout ICD codes as well as read codes

used in the UK health system. We used MR-BRT to adjust alternative case definition and claims data in the USA from the year 2000 and from 2010 onward and for Taiwan claims data to the reference case definition. Matched data was based off of age, sex, year, and location. The mean and standard error for the coefficients were calculated using the MR-BRT crosswalk adjustment method. Betas and exponentiated values (which can be interpreted as an odds ratio) for these covariates are shown in the table below:

Table 3: MR-BRT crosswalk adjustment factors for gout

Data input	Reference or alternative case definition	Gamma	Beta Coefficient, Log (95% CI)*	Adjustment factor**
Physician-diagnosed gout	Ref	0.55	---	---
Self-reported gout	Alt		0.33 (0.050 to 0.60)	1.39 (1.05 to 1.83)
Gout identified with administrative data	Alt		0.29 (0.29 to 0.30)	1.34 (1.34 to 1.35)
USA claims data – 2000	Alt		-1.88 (-2.84 to -0.92)	0.15 (0.058 to 0.40)
USA claims data – 2010–2016	Alt		-1.55 (-2.00 to -1.09)	0.22 (0.13 to 0.34)
Taiwan claims data – 2016	Alt		0.30 (0.27 to 0.33)	1.35 (1.31 to 1.40)

**MR-BRT crosswalk adjustments can be interpreted as the factor the alternative case definition is adjusted by to reflect what it would have been had it been measured using the reference case definition. If the log/logit beta coefficient is negative, then the alternative is adjusted up to the reference. If the log/logit beta coefficient is positive, then the alternative is adjusted down to the reference.*

***The adjustment factor column is the exponentiated beta coefficient. For log beta coefficients, this is the relative rate between the two case definitions. For logit beta coefficients, this is the relative odds between the two case definitions.*

Modelling strategy

Prior settings included assuming the excess mortality rate and remission of gout did not exceed 0.01 and 0.2, respectively, and that there was no incidence or prevalence of gout before the age of 15 years. We have made no substantive changes in the modelling strategy from GBD 2019. We included the summary exposure variable (SEV) scalar for gout which summarises exposure to risks estimated in GBD to impinge on gout, ie, low glomerular filtration rate, as a country covariate. We set bounds of 0.75 to 1.25 as the SEV is constructed in a way that if our risk estimates are accurate the value should be 1.

Table 4. Covariates. Summary of covariates used in the gout DisMod-MR meta-regression model

Covariate	Type	Parameter	Beta (95% Uncertainty Interval)	Exponentiated beta (95% Uncertainty Interval)
Log-transformed age-standardised SEV scalar: Gout	Country-level	Prevalence	1.25 (1.24 to 1.25)	3.48 (3.45 to 3.49)

Severity and disability

The basis of the GBD disability weight (DW) survey assessments are lay descriptions of sequelae highlighting major functional consequences and symptoms. The lay descriptions and disability weights for gout severity levels are shown below.

Table 5. Severity distribution, details on the severity levels for gout in GBD 2019 and the associated disability weight (DW) with that severity.

Severity level	Lay description	DW (95% CI)
Gout, acute	This person has severe pain and swelling in the leg, making it very difficult to get up and down, stand, walk, lift, and carry heavy things. The person has trouble sleeping because of the pain.	0.295 (0.196–0.409)
Polyarticular gout (same as for severe RA)	This person has severe, constant pain and deformity in most joints, causing difficulty moving around, getting up and down, eating, dressing, lifting, carrying, and using the hands. The person often feels sadness, anxiety, and extreme fatigue.	0.581 (0.403–0.739)
Asymptomatic gout	This person has a diagnosis of gout without pain or functional difficulties.	0

To calculate the severity distribution of gout, we used three studies on the distribution of the number of gout attacks per year and fitted a lognormal curve using a least squared differences method.[1,2,3] In the absence of data on the proportion of gout cases who have chronic polyarticular gout, we assumed the proportion was equal to those who would have 52 attacks a year (ie, weekly) or more as implied by the lognormal curve.

The average number of attacks was estimated from the lognormal fit: 5.66 (5.14–6.18). From two studies we derived an average duration of attacks of 6.1 (5.4–6.8) days by simple averaging. The resulting proportion of time symptomatic for acute gout was taken as the multiplication of these two estimates divided by the number of days in a year: 9.4% (8.0–10.9).

Figure 1: Distribution of cases by frequency

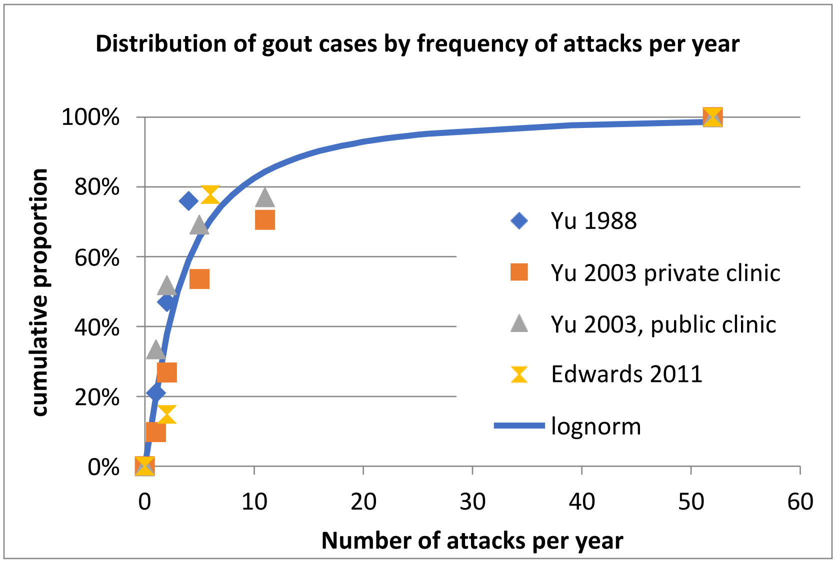

References

1. Edwards NL, Sundy JS, Forsythe A, Blume S, Pan F, Becker MA. Work productivity loss due to flares in patients with chronic gout refractory to conventional therapy. Journal of Medical Economics. 2011; 14(1).
2. Yu, KH, Luo SF, et al. Younger age of onset of gout in Taiwan. Rheumatology. 2003; 42(1): p. 166-170.
3. Yu TF, et al. Diversity of clinical features in gouty arthritis. Seminars in Arthritis and Rheumatism. 1984; 13(4): p. 360-368.

Dracunculiasis (Guinea worm)

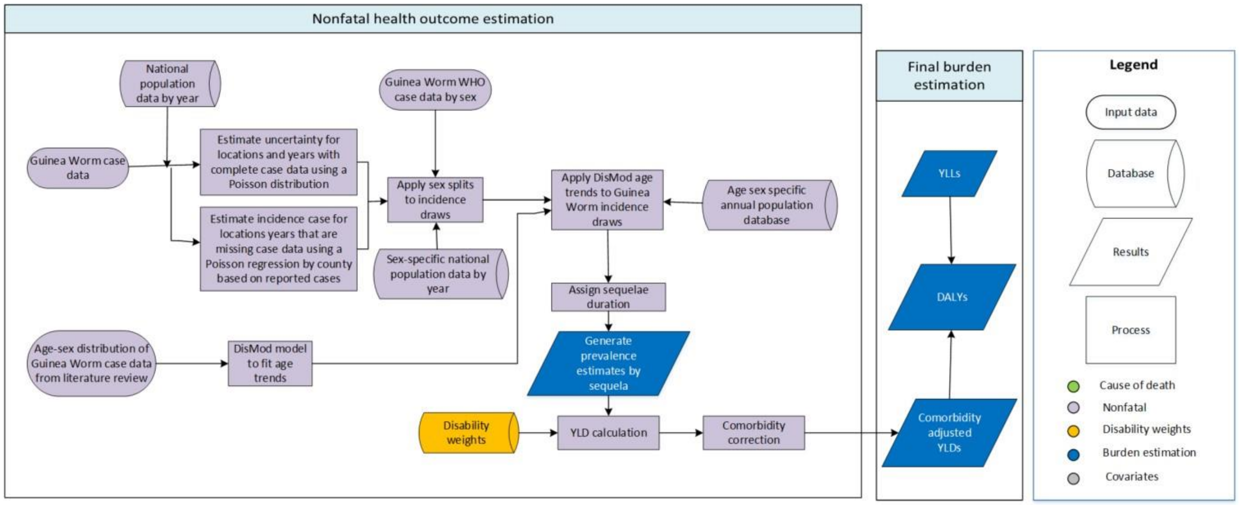

Background

Guinea-worm disease is caused by the parasitic worm *Dracunculus medinensis*. The transmission cycle begins when Guinea worm larvae are released in stagnant water (eg, ponds, lakes, open wells) where they are ingested by freshwater copepods (small crustaceans sometimes called water fleas) of the genus *Cyclops* [1]. When a person consumes water containing *Cyclops*, the copepods are dissolved by gastric acids and intestinal enzymes and the larvae are released. Larvae then migrate through the intestinal wall and travel to the connective tissues. The larvae mature and mate 60–90 days after infection; shortly thereafter, the male dies and the pregnant female worm continues to move through the victim's connective tissues. Approximately 10–14 months post-infection, the adult worm creates a painful burning blister on the skin that develops and enlarges over several days, usually from the feet or lower limbs. Blister formation may be preceded by a slight fever, itchy rash, nausea, vomiting, and diarrhoea. To relieve the pain associated with the worm's emergence, infected persons immerse the infected part of their body in local stagnant water sources, such as ponds. Upon entering the water, the female worm will expel her larvae and the cycle can begin again [1-4]. Worm removal is painful and can be complicated by secondary bacterial infection.

The global campaign to eradicate Guinea worm began in 1980, when the US Centers for Disease Control and Prevention (CDC) suggested that Guinea worm eradication would be an ideal indicator of the success of the International Drinking Water Supply and Sanitation Decade of 1981–1990; in 1981, Guinea worm eradication was adopted as a sub-goal of this United Nations advocacy effort [1, 5]. In 1986, the World Health Assembly adopted a resolution to eliminate Guinea worm disease, and since then, the Carter Center has led a coalition that includes ministries of health of endemic countries, CDC, the World Health Organization (WHO), the United Nations Children's Fund (UNICEF), thousands of village volunteers, and supervisory staff supported by numerous donors [5].

To break the cycle of transmission, ministries of health in endemic countries implement a suite of interventions: case detection and containment, provision of safe water sources, distribution of filter cloths and pipe filters, water source treatment with Abate® (a larvicide), and health education.

By design, the Guinea worm eradication programmatic infrastructure covers the entire at-risk population in endemic countries. Since case containment[6] is a key intervention designed to not only interrupt transmission but also monitor progress toward eradication, incident cases of Guinea worm disease are nationally representative. To implement case containment as an intervention, all cases of Guinea worm disease are identified. Containment is defined as detection within 24 hours of the worm's emergence; the patient did not contaminate any water source; the patient received proper wound care and health education on not entering any water source; a supervisor verified the case as dracunculiasis within seven days; and Abate® is used if there is any uncertainty about contamination of water sources or known contamination of water sources [7]. Case reporting occurs at the village level on a monthly basis; case data are then aggregated within the national Guinea Worm Eradication Program and reported to WHO. In settings where annual case reports are low (suggesting no transmission) or transmission has been interrupted, cash rewards are promoted to enhance surveillance activities.

Input data & methodological summary

Case definition

A Guinea worm case is defined as an individual with Guinea worm disease. A person is counted as a case only once in a calendar year, ie, when the first Guinea worm emerged from that person, although an individual may have more than one worm emerge at a time and/or more than one worm emerge during the year. These cases are confirmed through the Guinea worm eradication programme infrastructure by clinical exam and verification by local supervisors. All specimens from case-patients are sent to the CDC for laboratory evaluation and confirmation [7].

We used the following case definitions for GBD 2021:

Quantity of interest	Reference or alternative		Definition
Guinea worm	Reference		An incident case is defined as a person exhibiting a skin lesion with emergence of a Guinea worm at least once in a calendar year. National disease programmes confirm number of reported cases by clinical exam and verification by local supervisors. In recent years, specimens from case-patients are sent to the US Centers for Disease Control and Prevention (CDC) for laboratory evaluation and confirmation.

Input data

Data sources

1) Case data by geography, by year
2) Literature review of age/sex distribution
3) Literature review for sequelae (type, duration, and proportion)

Case data: Annual case data were reported by WHO in the Weekly Epidemiological Record for the period 1990–2017. For years or geographies for which WER reports were not published, the following sources were also used to extract case counts:

1) CDC's MMWR reports
2) 1990–1999 total country reports from Hopkins *et al*[8]
3) India subnational estimates: India MOH report (1984–1999)
4) The Carter Center's Guinea worm wrap-up: disaggregation of case totals for Sudan and South Sudan pre-2011 (independence) to ensure case totals from 1990–2010 are consistent with current national boundaries; 2019-2021 provisional case data.

The number of cases annually was compared to official total numbers published in WER 2016 to ensure accuracy of data entry.

Table 1 presents the total number of data sources used to generate burden estimates.

Table 1. Source counts

Measure	Countries with data	New sources	Total sources
All measures	22	10	446
Prevalence	4	0	7
Incidence	22	10	439

Subnational data

India: Subnational data for India were obtained from the Ministry of Health for the period 1984–1999; cases were reported by year and state.

Kenya: Subnational data from Kenya were requested from the MOH but not obtained. To split cases by subnational unit, the Carter Center Guinea Worm Wrap-Up was reviewed to identify districts with endemic villages. A national survey conducted 1993/1994 found cases in Turkana and West Pokot counties, but case totals were not reported by county. Indigenous transmission was interrupted in 1995, with imported cases reported until 2005. WER reports from 1999 to 2006 document that all imported cases from 1998 to 2005 occurred in Turkana County. All cases in Kenya are currently analysed in GBD as occurring in Turkana County as we are unable to disaggregate the data.

Ethiopia: Subnational data for the Ethiopian state of Gambella were obtained from country reports (Hopkins *et al*[8]) and WHO reports covering the period of 1990–2019.

Pakistan: Subnational data for endemic Pakistan provinces were obtained from country reports from Hopkins *et al*[8] for the period 1988–1994; cases were reported by year and state.

Nigeria: Subnational data for Nigeria were obtained from the Carter Center for the period 1987–2008; cases were reported by year and state.

Geographical restrictions

Only the following countries were identified as Guinea-worm endemic as of 1990[8]: Benin, Burkina Faso, Cameroon, Central African Republic, Chad, Côte d'Ivoire, Ethiopia, Ghana, India, Kenya, Mali, Mauritania, Niger, Nigeria, Pakistan, Senegal, Sudan, South Sudan, Togo, Uganda, and Yemen[8]. Any country not reporting Guinea worm as of 1990 is not included in the GBD model.

Geographical restrictions by year were also implemented to account for the period post-transmission to reflect the accomplishments of the Guinea worm eradication campaign. Geographical restriction for countries that were endemic in 1990 was defined based on data reported post-interruption of transmission. In the GBD analysis, Guinea worm disease was no longer modelled for the year that followed the last reported case (imported or indigenous) provided that the subsequent years through 2019 also had no case reports. To ensure that cases were attributed to burden in the country in which the case was detected, both indigenous and imported cases were included. For example, Kenya reported its last (imported) case in 2005, and as no other cases were reported through 2019, incidence from 2006

onward is zero. For Chad, there were no cases reported in 2001–2009; however, all other years had at least one case reported, and thus the model reflects the entire period of 1990–2021.

Accounting for possible under-reporting

Once national eradication programmes were initiated, national case searches were conducted to improve the accuracy of national case estimates. These searches were designed to enumerate prevalent Guinea worm disease cases and identify endemic villages to direct intervention and surveillance activities. For the majority of years included in the GBD analysis, the total number of Guinea worm cases reported is equivalent to a national census, as all cases are identified and reported. Nevertheless, not all endemic countries were able to initiate full national surveillance as of 1990.

The model does not account for the possibility that cases occurred in communities that were not included in routine surveillance or did not achieve 100% reporting coverage over time. However, any cases that may have been undetected would likely not have been a significant increase over annual totals given the comprehensive nature of Guinea worm disease surveillance activities. Nevertheless, there are years for which the annual case data are inconsistent with preceding/following annual case totals and could not be accounted for in our model. For example, Niger reported 500 cases in 1992, despite reporting 32,829 cases in 1991 and 25,346 cases in 1993. In those instances, the following datapoints were identified as outliers and excluded from analysis as follows:

Table 2. List of reported case data outliered in the analysis to account for possible under-reporting

Country	Year	Reported cases
Central African Republic	1996	9
Central African Republic	1997	5
Ethiopia	1992	303
Kenya (Turkana County)	1990	6
Uganda	1990	4,704
Uganda*	1992	126,369
Benin	1991	4,006
Benin	1992	4,315
Chad	1992	156
Côte d'Ivoire	1990	1,360
Mali	1990	884
Mauritania	1992	1,557
Niger	1992	500
Senegal	1990	38
Togo	1990	3,042
Togo	1991	5,118
South Sudan*	1996	116,844
Sudan	1994	132

*For these two datapoints, we do not dispute that over 100,000 cases of Guinea worm likely occurred. However, given the amount of missing data in the early time series for these two countries, inclusion of these resulted in implausibly high case predictions (over 1 million cases in Uganda in 1990 and over 1.5 million for South Sudan from 1990 to 1995).

Age/sex distribution

Generally, the risk of Guinea worm infection varies according to sex- or age-specific differences in access to safe drinking water. A study in Ethiopia found women were more likely to experience Guinea worm disease than men; in India, men experienced greater risk of infection [1]. Exposure to unsafe water sources varies largely on mobility patterns and type of water sources: communities in which infected water is carried in for consumption are more likely to see more Guinea worm disease in children and older adults [9]. Once interventions to control the spread of Guinea worm infection are implemented, the age and sex distribution likely changes to reflect variation in coverage and uptake of eradication interventions, such as larvicide of water sources and case-containment rates; age/sex case data are currently not available.

The evidence base available to describe risk of infection by age is as follows:

1) Studies from Nigeria:
    a. Adeyeba *et al* [10]: Guinea worm disease not common among children <1 year of age; increase in risk by age
    b. Kale *et al* [11]: More boys ages 5-9 years than girls were infected (11.9% v. 6.8%); Women ages 20-29 years had higher prevalence of infection than men (13.4% v. 4.7%); Overall, the prevalence in both men and women was highest in ages 10-14 years and 30 years or older.
    c. Greenwood *et al* [12]: The mean age of male cases was 25.8 years (95% CI: 23.9, 27.7) and 26.9 years for females (95% CI: 23.7, 30.1).
2) Other countries:
    a. Sudan [13]: No significant age trend among lower-endemicity villages; higher-endemicity villages (n=4) had higher prevalence in children and older adults. This study attributes the difference in age trends to community-level water source.
    b. Ghana [14]: The trend in age of first infection reported was similar for males and females, with more females experiencing first infection between 15 and 19 years and males between 20 and 24 years of age. The proportion of men with Guinea worm disease was much higher than among women 25-54 years of age. Adults >15 years of age were more likely to be infected than children.

The evidence base available to describe the risk of infection by gender is as follows:

1) Studies from Nigeria:
    a. Adeyeba *et al* [10]: No difference among males and females.
    b. Kale *et al* [11]: No overall gender difference comparing total males infected to total females infected, although gender differences for certain age groups (see notes above).
    c. Greenwood *et al* [12]: Two-thirds of cases reported among 47 villages from 1971 to 1974 were male.

WHO Weekly Epidemiological Record (WER) age reports: Age and sex data were reported by country for 2009 onward; these data capture the age distribution for Chad, Ethiopia, Ghana, Mali, and South Sudan. We excluded these data as the age/sex distribution is only described for children <15 years or adults, which does not permit fitting an age trend across multiple categories.

WER sex-specific data: Sex-specific differences in the burden of Guinea worm disease could reflect differing levels of access to eradication programme interventions, in addition to risk factors associated

with local transmission dynamics. Since the data reported from 2009 to 2015 are the only available nationally representative data, we used the overall sex difference to generate sex-specific incidence and prevalence, with females experiencing a slightly higher risk (53%) compared to males (47%):

Table 3. WHO Weekly Epidemiological Record total worm burden by gender, by year

Year	Female	Male	Total	% Fem	% Male
2009	1699	1490	3189	53%	47%
2010	976	821	1797	54%	46%
2011	524	534	1058	50%	50%
2012	273	269	542	50%	50%
2013	79	69	148	53%	47%
2014	63	63	126	50%	50%
2015	9	13	22	41%	59%
Total	3623	3259	6882	53%	47%

There is limited evidence to suggest that risk varies jointly by sex and age; however, evidence for this modification also suggests that such age- and sex-specific risks may vary by endemic community within a given geography (in some settings, women at higher risk, in others men, but not for all age strata). Without additional data sources in which cases are disaggregated by age and sex, this joint relationship is not modelled.

To model age-specific variation, we used data from seven studies with age-specific case data to generate an age-trend in a DisMod model. We further assumed no Guinea worm disease occurred in infants less than 1 year of age.

Severity splits/sequelae

Sequelae associated with Guinea worm relate to the wound at the site of the worm's emergence, which can include abscesses and chronic ulcerations. Joint and tissue damage can occur, as well as secondary infection in connective tissues [15]. During the worm's emergence, which takes approximately one month to exit the body, the ulcer is painful and itchy [1]. The wound is subject to secondary infection and scarring. Possible long-term consequences of Guinea worm infection include arthritis or other permanent damage to connective tissues; however, data on this are limited. In the Greenwood study, 41.7% of all cases experienced infection at the site of emergence, and the annual proportion of cases with definite arthritis ranged from 1.6% to 7.3% of all cases.

While an individual experiences Guinea worm disease, they are generally unable to work and have limited mobility at the time prior and during emergence and in the subsequent period in which they are healing. Although most worms emerge in the feet and lower legs, there are reports of worms exiting at other sites [15], which could cause other disability not accounted for here. A study in Nigeria found that 98% of worms emerged in the lower limbs [16]. The Greenwood study also observed that 88.4% emerged in the lower limbs. Therefore, for the purposes of estimating the burden of Guinea worm disease in GBD, all disability associated with Guinea worm disease is attributed to lower limb conditions, pain, and lack of mobility. Due to limited data, we cannot account for differential disability based on number of worms emerging at the same time.

The following evidence base was reviewed to determine the proportion of cases attributed to each sequela, as well as duration of sequelae.

Duration of disability and type of disability:

Studies from Nigeria:

1) Adeyeba *et al* [10]: 93.4% incapacitated for an average of 26 days.
2) Smith *et al* [17]: Average disability duration 12.7 weeks; 58% unable to leave the home for a mean duration of 4.2 weeks; duration of disability greater among those older than 50 years compared to those younger than 50 years.
3) Okoye *et al* [16]: 21% of cases were totally incapacitated due to their infection (not permanently disabled).
4) Kate *et al* [11]: A survey of 17 villages from 1971 to 1975 found that duration of disability was approximately 100 days.
5) Greenwood *et al* [12]: Weekly visits to 47 villages from 1971 to 1974 reported mean duration of illness ranging from 4.2 weeks to 7.2 weeks. 17.4% of cases had an active infection which persisted for 10 weeks or more.

Other countries:

6) Benin [18]: From two villages in highly endemic areas, estimated 39-59 days of disability experienced after worm emergence.
7) Ghana [19]: 28.2% experienced pain 12-18 months post-emergence; 5% unable to carry out at least one daily activity, 0.5% permanently impaired (ligament damage to thumb).
8) Ghana [14]: Complete disability experienced among males with Guinea worm disease lasted approximately 5 weeks among those untreated. Among cases provided supportive care (wound management), the duration of disability was 2.5 weeks.

For all cases, we assume each experiences pain and disfigurement (level 2), and musculoskeletal problems, lower limb (moderate) for a period of one month, followed by two months of pain and disfigurement (mild). We then assume that 30% of all cases will then experience disfigurement level 1 with itch/pain for an additional nine months (approximately a year of disability) to account for longer-term disability associated with recovery.

Table 4. Severity distribution, details on the severity levels for Guinea worm and the associated disability weight (DW) with that severity

Sequela	Lay description	DW (95% CI)
Disfigurement, level 2, with itch/pain	Has a visible physical deformity that is sore and itchy. Other people stare and comment, which causes the person to worry. The person has trouble sleeping and concentrating.	0.188 (0.125–0.267)
Disfigurement, level 1, with itch/pain	Has a slight, visible physical deformity that is sometimes sore or itchy. Others notice the deformity, which causes some worry and discomfort.	0.027 (0.015–0.042)
Musculoskeletal problems, lower limbs, moderate	Has moderate pain in the leg, which makes the person limp, and causes some difficulty walking, standing, lifting and carrying heavy things, getting up and down, and sleeping.	0.079 (0.054–0.11)

Modelling strategy

Total incidence

The incidence of Guinea worm disease is modelled in GBD using two approaches: for years and locations for which case data were reported, 1000 draws of incidence were estimated using a beta distribution of cases and total population minus cases. For years and locations for which case data were missing (largely the early 1990s) a Poisson regression of all case data was implemented per country, using the total population as the offset. The predicted incidence and standard error were used to generate a random distribution of 1000 incidence draws. Incidence is multiplied by duration of sequelae to calculate prevalence.

Figure 1. Overall comparison of model versus reported cases (excluding outliers)

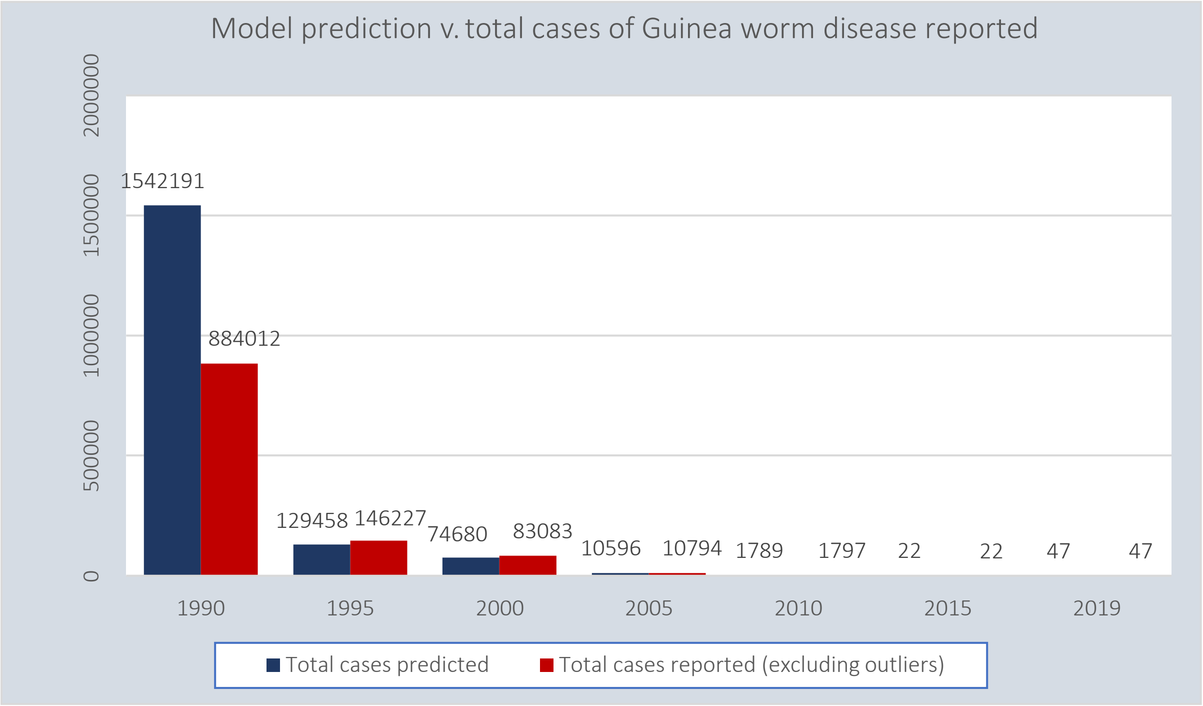

Sex-specific incidence

To account for the proportion of cases in females compared to males (53% to 47%), the incidence draws were multiplied by the sex proportion and the total population (to estimate number of cases by sex), then divided by the sex-specific total population for that year to calculate sex-specific incidence.

Age-specific incidence

In order to generate age-specific incidence, a literature search was conducted to identify national and subnational data sources in which age-specific prevalence was reported. The only nationally representative data available were WER reports from 2009 onward; however, age was only reported as less than 15 years of age or older than 15 years of age. In order to generate a trend over the life course, eight subnational data sources were identified. The prevalence of Guinea worm disease was extracted by age category reported in the original paper. An age trend was fit using DisMod-MR 2.1, with the following model settings:

Age mesh points: 0 0.01 5 10 15 20 25 30 35 40 45 50 55 60 65 70 75 80 85 90 100

Drill year: 2000; Drill location: Global; no birth prevalence; 30 year time window

The age data generated a single-age trend that we assumed applied to all geographies and all estimation periods from 1990 to 2021.

Figure 2. Age-specific prevalence model generated by DisMod

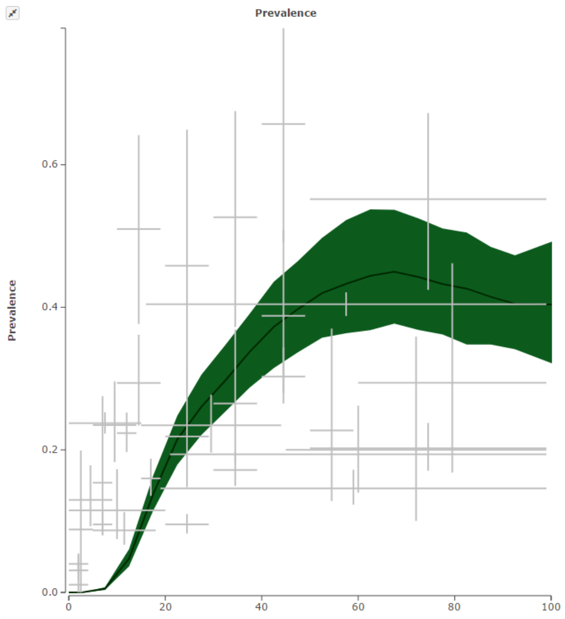

To apply this age prevalence curve to the sex-split incidence draws, 1000 draws of output were downloaded from DisMod and applied to the incidence data as follows:

j indexes the age strata

i indexes the draw (1 to 1000)

sex cases draw is the total number of cases for the sex stratum (all ages)

$$age\ cases_j = DisMod\ Draw_{i,j} * age\ population_j$$

$$age\ incidence\ draw_i = \frac{age\ cases_j\ (\frac{sex\ cases\ draw_i}{total\ cases})}{age\ population_j}$$

Under the assumption that Guinea worm disease occurs approximately one year post-infection, incidence among children aged less than 1 year was set to zero.

Sequelae splits

Prevalence of the sequelae listed in Table 3 was calculated by multiplying the age- and sex-specific incidence draw by the duration of the health state (in years).

1) Guinea worm pain associated with worm emergence (Level 2): all cases, 1 month
2) Guinea worm pain associated with worm emergence (Level 1): all cases, 2 months plus 30% of cases for an additional 9 months
3) Lower limb musculoskeletal problems: all cases, 1 month

References

1. Cairncross S, Muller R, Zagaria N. Dracunculiasis (Guinea worm disease) and the eradication initiative. *Clin Microbiol Rev* 2002; 15: 223–46.
2. Biswas G, Sankara DP, Agua-Agum J, Maiga A. Dracunculiasis (Guinea worm disease): eradication without a drug or a vaccine. *Philos Trans R Soc Lond B Biol Sci* 2013; 368: 20120146.
3. Ruiz-Tiben E, Hopkins DR. Dracunculiasis (Guinea worm disease) eradication. *Adv Parasitol* 2006; 61: 275–309.
4. Greenaway C. Dracunculiasis (guinea worm disease). *CMAJ* 2004; 170: 495–500.
5. Hopkins DR, Ruiz-Tiben E, Downs P, Withers PC, Roy S. Dracunculiasis eradication: neglected no longer. *Am J Trop Med Hyg* 2008; 79: 474–9.
6. Kappus KD, Hopkins DR, Ruiz-Tiben E, *et al.* A strategy to speed the eradication of dracunculiasis. *World Health Forum* 1991; 12: 220–5.
7. Prevention CfDCa. Guinea worm wrap-up Atlanta, GA: WHO Collborating center for Research, Training and Eradication of Dracunculiasis, CDC; 2015.
8. Hopkins DR, Ruiz-Tiben E, Diallo N, Withers PC, Jr., Maguire JH. Dracunculiasis eradication: and now, South Sudan. *The American journal of tropical medicine and hygiene* 2013; 89.
9. Watts SJ, Brieger WR, Yacoob M. Guinea worm: an in-depth study of what happens to mothers, families and communities. *Soc Sci Med* 1989; 29: 1043–9.
10. Adeyeba OA, Kale OO. Epidemiology of dracunculiasis and its socio-economic impact in a village in south-west Nigeria. *West Afr J Med* 1991; 10: 208–15.
11. Kale OO. The clinico-epidemiological profile of guinea worm in the Ibadan district of Nigeria. *Am J Trop Med Hyg* 1977; 26: 208–14.
12. Greenwood B, Greenwood A, Bradley A. Guinea worm infection in northern Nigeria: reflections on a disease approaching eradication. *Trop Med Int Health* 2017; 22: 558–66.
13. Tayeh A, Cairncross S. The impact of dracunculiasis on the nutritional status of children in South Kordofan, Sudan. *Ann Trop Paediatr*. 1996; 16:221-6.
14. Belcher DW, Wurapa FK, Ward WB, Lourie IM. Guinea worm in southern Ghana: its epidemiology and impact on agricultural productivity. *Am J Trop Med Hyg* 1975; 24: 243–9.
15. Muller R. Guinea worm disease: epidemiology, control, and treatment. *Bull World Health Organ* 1979; 57: 683–9.
16. Okoye SN, Onwuliri CO, Anosike JC. A survey of predilection sites and degree of disability associated with guineaworm (Dracunculus medinensis). *Int J Parasitol.* 1995; 25:1127-9.
17. Smith GS, Blum D, Huttly SR, Okeke N, Kirkwood BR, Feachem RG. Disability from dracunculiasis: effect on mobility. *Ann Trop Med Parasitol* 1989; 83: 151–8.
18. Chippaux JP, Banzou A, Agbede K. [Social and economic impact of dracunculosis: a longitudinal study carried out in 2 villages in Benin]. *Bull World Health Organ* 1992; 70: 73–8.
19. Hours M, Cairncross S. Long-term disability due to guinea worm disease. *Trans R Soc Trop Med Hyg* 1994; 88: 559–60.

Gynaecological conditions

For GBD 2021, we estimate the burden of gynaecological diseases including uterine fibroids, polycystic ovarian syndrome, endometriosis, genital prolapse, premenstrual syndrome, and other gynaecological diseases. ICD-10 codes for each cause included in the non-fatal estimation are listed in the table below.

Table 1: ICD-10 codes used in the non-fatal estimation for gynaecological diseases

Cause	ICD-10 code
Uterine fibroids	D25-D26.9, D28.2
Polycystic ovarian syndrome	E28.2
Endometriosis	N80-N80.9
Genital prolapse	N81-N81.9
Premenstrual syndrome	N94.3
Other gynaecologic diseases<br>- Menstrual disorders<br>- Other non-menstrual gynaecological disorders	 N91-N95.9<br>B37.3-B37.49, N61 – N64.9, N72, N75 – N77.8, N83 – N86, N88 – N90.9

Uterine fibroids

Flowchart

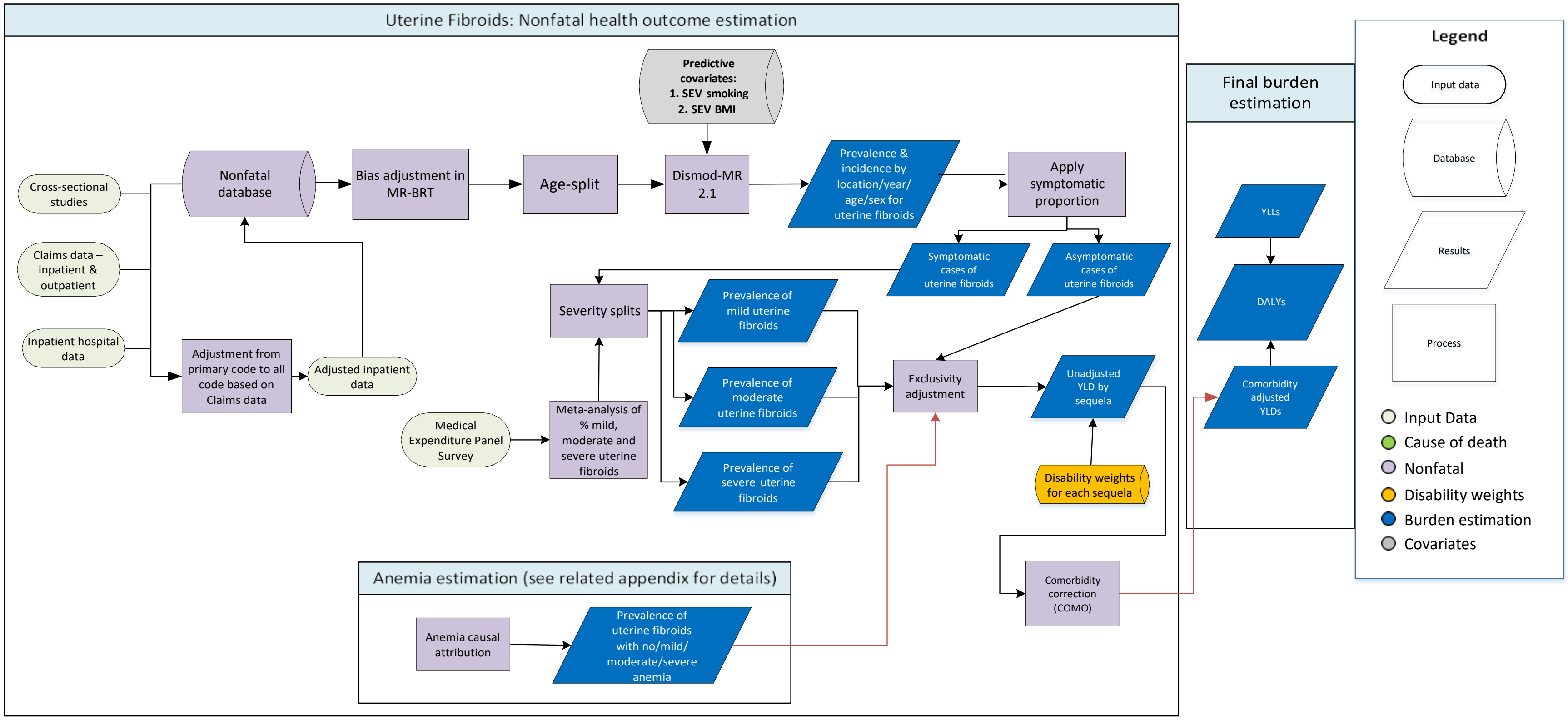

Case definition

Uterine fibroids, also called uterine myomas or leiomyomas, are non-cancerous tumours that develop from the muscle tissue of the uterus, regardless of symptoms. When present, symptoms include abdominal/pelvic pain, painful intercourse, infertility, and heavy vaginal bleeding, which can lead to anaemia.

Signs and symptoms of fibroids can be detected by clinical interview and pelvic exam, but the diagnosis should be confirmed by ultrasonography, hysterectomy, hysterosalpingography, sonohysterography, laparoscopy, or imaging tests such as MRI or CT scan. For GBD 2021, we use the definition proposed by the American College of Obstetricians and Gynecologists (ACOG) as the reference case definition, that is, cases of uterine fibroids diagnosed by pelvic exam followed by or with ultrasonography, hysteroscopy, hysterosalpingography, sonohysterography, or laparoscopy.[1] We also incorporate studies that ascertained cases by self-report, pelvic exam only, or via diagnostic codes in administrative data, by adjusting data toward our reference case definition, as described below.

Input data

The last systematic review for uterine fibroids was done in GBD 2010, when Ovid MEDLINE, EMBASE, CINAHL, CAB abstracts, WHOLIS, and ISGLE database were searched. An updated PubMed search is planned during the next GBD update of gynaecological disorders. The search strings used in the initial search were as follows:

> **PUBMED**: (“Leiomyoma”[Mesh] OR fibroid OR fibroids OR leiomyoma OR leiomyomas OR leimyoma OR leimyomas OR leyomyoma OR leyomyomas OR fibromyoma OR fibromyomas OR fibroma OR fibromas OR myoma OR myomas) AND (“Genitalia, Female”[Mesh] OR “Gynecology”[Mesh] OR “Uterus”[Mesh] OR genital OR genitals OR genitalia OR gynecology OR gynaecology OR gynecologic OR gynecological OR gynaecologic OR gynaecological OR uterine OR uterus OR hysterectomy) AND (“Prevalence”[Mesh] OR prevalence OR prevalences)

> **EMBASE**: (‘uterus myoma’/exp OR fibroid OR fibroids OR leiomyoma OR leiomyomas OR leimyoma OR leimyomas OR leyomyoma OR leyomyomas OR fibromyoma OR fibromyomas OR fibroma OR fibromas OR myoma OR myomas) AND (‘uterus’/exp OR ‘gynecology’/exp OR ‘female genital system’/exp OR genital OR genitals OR genitalia OR gynecology OR gynaecology OR gynecologic OR gynecological OR gynaecologic OR gynaecological OR uterine OR uterus OR hysterectomy) AND (prevalence/exp OR prevalence OR prevalences)

Exclusion criteria for the initial systematic review were reviews, studies that did not provide primary data on epidemiological parameters (eg, commentary), and clearly non-representative studies (eg, of only high-risk pregnant women).

In addition to data from the above systematic review, claims data from the USA (MarketScan), the Philippines, Taiwan (province of China), and Poland were included, along with hospital discharge data that were processed by extracting discharges with an appropriate ICD code as primary diagnosis and adjusting using a correction factor. Specifically, we used claims data to model the ratio of inpatient claims with uterine fibroids as primary diagnosis to total prevalent cases of uterine fibroids seen in both inpatient and outpatient settings (see the section on Non-fatal data sources, identification, and extraction for a description of GBD modelling of hospital utilisation and processing of inpatient and claims data in this appendix). The total number of data sources used for the non-fatal estimation of uterine fibroids is provided in the following table.

Table 2: Data inputs for uterine fibroids morbidity modelling by parameter

Measures	Total sources	New sources	Countries with data

Prevalence	339	37	53
Incidence	4	1	3

Data processing

The first step of data processing was age splitting. For any datum that did not entirely fit within a GBD age group, the observation was split to be multiple age-specific datapoints based on the age pattern predicted by GBD 2017 DisMod-MR 2.1 models.

With changes to the hospital and claims administrative data-processing algorithms implemented since GBD 2017, most notably the addition of a requirement that two outpatient visits coded to a cause are required for a person to count as "a case" of a given disease, the inpatient-to-outpatient corrected administrative data became much more variable. This is hypothesised to be due to differences in care-seeking and health-care provision patterns for women with uterine fibroids, including differences between countries in whether women who have procedures for fibroids are categorised as inpatients or outpatients. We therefore used only inpatient hospital and claims data.

As mentioned before, the ACOG case definition of uterine fibroids was set as the reference case definition; this definition encompasses both symptomatic and asymptomatic cases that can be detected by pelvic exam or ultrasonography, sonohysterography, laparoscopy, or imaging tests such as MRI and CT scans. We consider clinical diagnoses indicated by ICD codes in administrative data (inpatient hospital and claims only), self-report, and symptomatic-only cases to be alternative case definitions.

In accordance with GBD 2021 principles for data processing, to make data comparable, we began by evaluating the number of observations of each alternate definition that matched with a corresponding observation from the reference definition. Due to data scarcity, we only found "between-study" matches. That means, we matched observations of different studies by age group, location (at the regional level), and whether the midpoint of the study was within five years of the midpoint of the reference definition observations. All observations that matched were paired with one another and logit-transformed, and the difference of the logit mean values were calculated. The standard error of the logit difference was calculated using the delta method. We then modelled the logit difference using meta-regression—Bayesian, regularised, trimmed (MR-BRT), a meta-analytic tool developed for the Global Burden of Disease study. The process of adjusting for biases in non-reference data using MR-BRT with logit-transformation method is described below:

43. Identify datapoints with overlapping year, age, sex, and location between non-reference data and reference data.
44. Logit transform overlapping datapoints of alternative and reference types.
45. Convert overlapping datapoints into a difference in logit space using the following equation: $logit(alternative) - logit(reference)$.
46. Use the delta method to compute standard errors of overlapping datapoints in logit space, then calculate standard error of logit difference using the following equation: $\sqrt{(variance\ of\ alternative) + (variance\ of\ reference)}$.
47. Using MR-BRT, conduct a random effects meta-regression to obtain the pooled logit difference of alternative to reference.

48. Apply the pooled logit difference to all datapoints of alternative case definitions using the following equation:
$$new_{estimate} = inverse.\,logit((logit(alternative)) - (pooled\ logit\ difference)).$$
49. Calculate new standard errors using the delta method, accounting for gamma (between-study heterogeneity).

Adjustment of data inputs for uterine fibroids was conducted in two steps, using the above method serially. In the first step, we adjusted only clinical data to a common standard, by using claims data as the reference definition and inpatient hospital data as the alternative definition. In this model, we trimmed 10% of the data and added a quadratic spline on age, assuming non-linear tails. Our final model results for this crosswalk process are illustrated below.

Uterine fibroids MR-BRT crosswalk adjustments factors by age for hospital (alternate) to claims (reference) data.

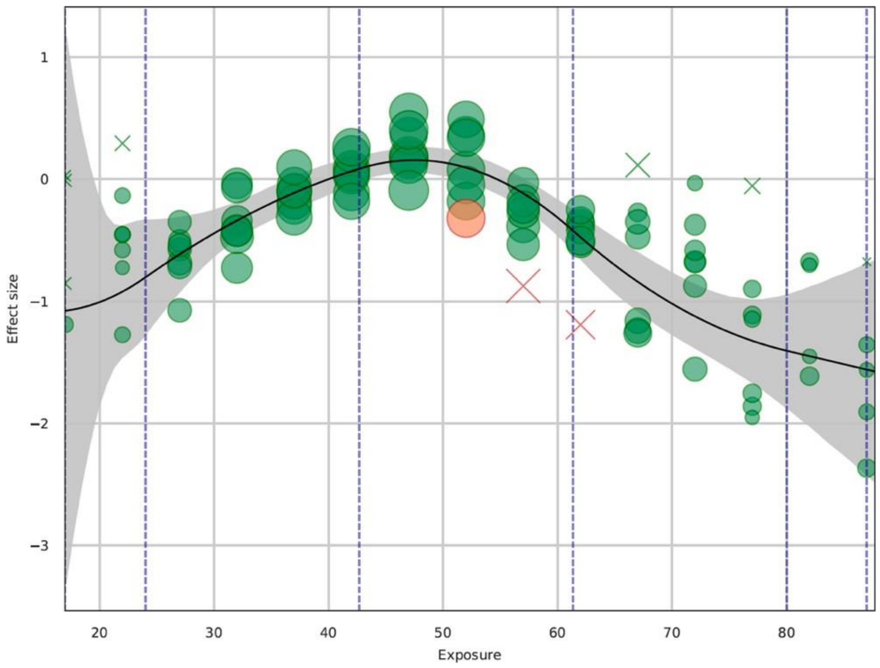

**Exposure on the x-axis is GBD age group and effect size is the logit-transformed difference of inpatient to claims data.*

According to this model, hospital data underestimated the number of uterine fibroid cases for most age groups. With ages 40–55 years, the inverse relationship is true.
Once the clinical data were adjusted, we performed a network MR-BRT considering the ACOG definition as the reference and clinical data (inpatient hospital and claims, adjusted per above), self-report, and symptomatic-only cases as alternative definitions. The adjustment factors for each of the included covariates in the models are summarised in the following table.

Table 3: MR-BRT crosswalk adjustment factors for uterine fibroids network model to standardise to ACOG definition

Data input	Reference or alternative case definition	Gamma	Beta coefficient, logit (95% CI)	Adjustment factor*
ACOG definition	Reference	1.13	---	---
Self-report	Alt		–2.991<br>(–3.41 to –2.54)	0.049<br>(0.031 to 0.07)
Symptomatic cases	Alt		–3.558<br>(–5.22 to –1.83)	0.028<br>(0.005 to 0.138)
Clinical data	Alt		–1.824<br>(–2.18 to –1.47)	0.014<br>(0.102 to 0.0187)

**MR-BRT crosswalk adjustments can be interpreted as the factor the alternative case definition is adjusted by to reflect what it would have been had it been measured using the reference case definition. If the log/logit beta coefficient is negative, then the alternative is adjusted up to the reference. If the log/logit beta coefficient is positive, then the alternative is adjusted down to the reference.*

***The adjustment factor column is the exponentiated beta coefficient. For log beta coefficients, this is the relative rate between the two case definitions. For logit beta coefficients, this is the relative odds between the two case definitions.*

Modelling strategy

After standardisation of prevalence data to the ACOG definition, we modelled incidence, prevalence, remission, and excess mortality due to uterine fibroids in DisMod-MR 2.1.

As in previous GBD iterations, incidence was set to zero prior to 10 years of age and after 49, and we assumed no excess mortality. We set the minimum coefficient of variation to 0.8 and the priors on the location random effects to +/- 0.5.

In GBD 2019, a large number of potentially predictive covariates were selected *a priori* based on a non-systematic literature review, including summary exposure value (SEV) for smoking, body-mass index, systolic blood pressure, physical activity, alcohol consumption, the age-standardised death rate (lnASDR) of sexually transmitted infections (STIs) from GBD 2019 COD analyses, prevalence of pelvic inflammatory disease from GBD 2019 non-fatal analyses, prevalence of contraception, and total fertility rate, and tested in preliminary DisMod models. From this list, two covariates were selected and used in the final GBD 2019 DisMod model. These same two covariates were used in GBD 2021, as shown in the following table:

Table 4: Summary of covariates used in the uterine fibroids DisMod-MR meta-regression model

Covariate name	Measure	Beta value	Exponentiated value
Age-standardised SEV for high body-mass index	Prevalence	0.14<br>(0.08–0.20)	1.15<br>(1.08–1.22)
Age-standardised SEV for smoking	Prevalence	0.091<br>(0.018–0.17)	1.09<br>(1.02–1.19)

The above modelling strategy is consistent with that employed in GBD 2019 but is a change from previous GBD cycles when only symptomatic fibroids were modelled in DisMod, using clinical data that included only inpatient encounters. The assumption at that time was that all inpatient admissions represented fibroids that were symptomatic enough to warrant medical care. Total fibroids in previous cycles were then calculated based on a single study that reported 50% of the total cases of uterine fibroids to be symptomatic.[2] Starting in GBD 2019 and continuing in GBD 2021, the use of MR-BRT analysis allowed us to quantify the bias introduced when measuring the prevalence of only symptomatic

uterine fibroid cases. This allowed us adjust data toward the reference case definition prior to DisMod modelling, and thus to model total fibroids cases directly.

Severity splits and disability weights

We split total cases of uterine fibroids into symptomatic and asymptomatic cases of fibroids using the beta coefficient obtained in the crosswalk during data processing. The coefficient suggests that most uterine fibroids cases (97%) are asymptomatic. This proportion seem to be consistent with other studies that suggest that the majority of women with uterine fibroids do not experience symptoms[3,4] but is a notably significant departure from the proportion identified prior to GBD 2019. The remaining symptomatic cases were all assumed to have severe symptoms such as abdominal discomfort, severe haemorrhage, and consequently, anaemia due to fibroids.

The age-specific anaemia prevalence for symptomatic cases of uterine fibroids was analysed as part of overall anaemia causal attribution for GBD 2021. The details of the anaemia analysis are described separately in the “anaemia impairment” section of this appendix. Briefly, after estimating total anaemia, a series of counterfactual distributions are generated based on the age- and sex-specific prevalence of each anaemia-causing condition and the quantitative effect that the condition has on haemoglobin concentration in the blood, a so-called “haemoglobin shift,” that was derived by meta-analysing cohort studies, observational studies, or trials comparing the haemotological status of those with as compared to without the disease. Due to limited data on haemoglobin shift, all were assumed to be invariant over age, sex, location, and year. It should be noted that anaemia alone is not ascribed to fibroids, but only in conjunction with mild abdominal pain with the assumption that more severe, symptomatic cases would be more likely to cause anaemia. Disability weights for each sequela are listed below for reference.

Table 5: Severity distribution, details on the severity levels for uterine fibroids, and the associated disability weight (DW) with that severity

Severity	Lay description	DW (95% CI)
Asymptomatic		--
Abdominopelvic problem, mild	Has some pain in the belly that causes nausea but does not interfere with daily activities.	0.011 (0.005–0.021)
Anaemia, mild	Feels slightly tired and weak at times, but this does not interfere with normal daily activities.	0.004 (0.001–0.008)
Anaemia, moderate	Feels moderate fatigue, weakness, and shortness of breath after exercise, making daily activities more difficult.	0.052 (0.034–0.076)
Anaemia, severe	Feels very weak, tired, and short of breath, and has problems with activities that require physical effort or deep concentration.	0.149 (0.101–0.21)

Endometriosis

Flowchart

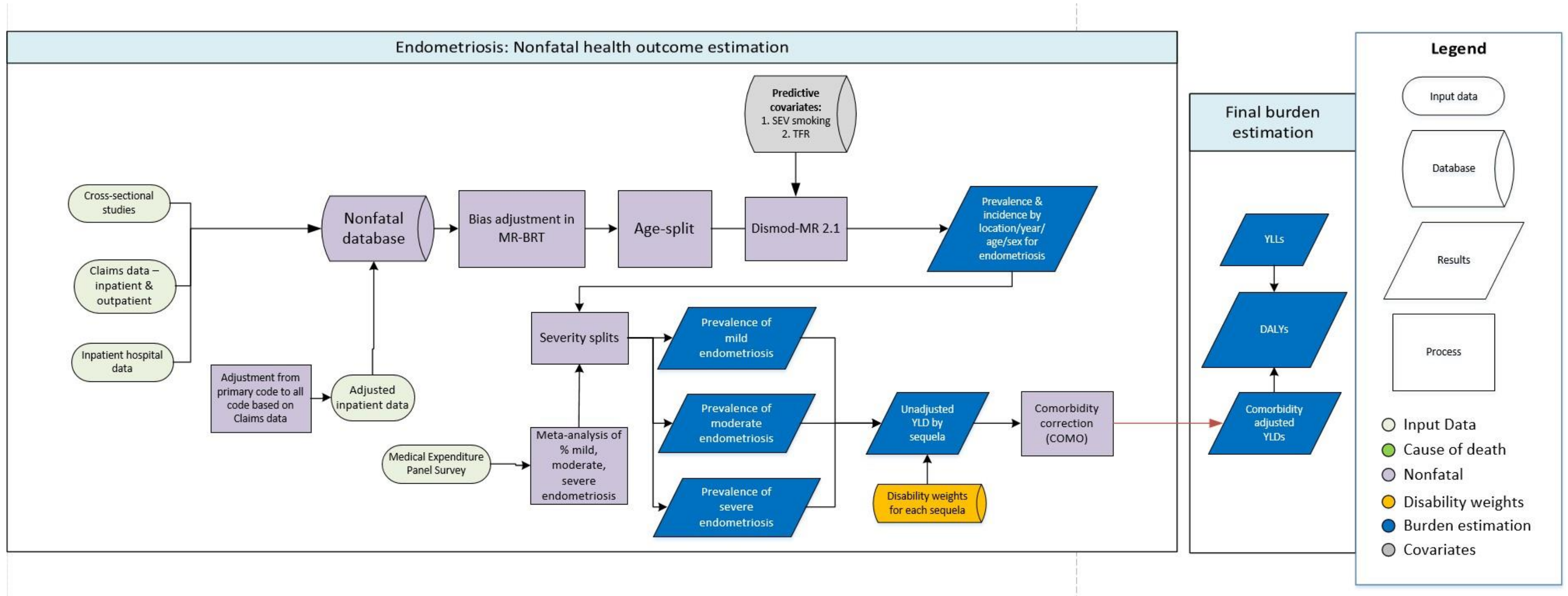

Case definition

Endometriosis is a gynaecological condition defined as growth of endometrial tissue outside the uterus regardless of symptoms. Common symptoms include chronic abdominal and pelvic pain, especially before and during a menstrual period and during sexual intercourse. Endometriosis can also lead to infertility. For GBD 2021, we define endometriosis cases according to the ACOG guidelines as cases diagnosed by pelvic exam confirmed by laparoscopy or laparotomy.[9]

Input data

A systematic review of endometriosis prevalence was conducted for GBD 2010. The review consisted of a PubMed search and a systematic review of endometriosis throughout the world. Ovid MEDLINE, EMBASE, CINAHL, CAB abstracts, WHOLIS, and ISGLE database were searched. The search strings for PubMed and EMBASE were as follows:

> **PUBMED:** ("Endometriosis"[Mesh] OR Endometriosis OR Endometrioses OR Endometrioma OR Endometriomas OR Adenomyosis) AND ("Incidence"[Mesh] OR Incidence OR Incidences OR "Prevalence"[Mesh] OR Prevalence OR Prevalences)
>
> **EMBASE:** ('endometriosis'/exp OR endometriosis OR endometrioses OR endometrioma OR endometriomas OR adenomyosis) AND ('incidence'/exp OR incidence OR incidences OR 'prevalence'/exp OR prevalence OR prevalences)

Exclusion criteria for the initial systematic review were reviews, studies that did not provide primary data on epidemiological parameters (eg, commentary), and clearly non-representative studies (eg, of only high-risk pregnant women).

In addition to data from the above systematic review, claims data from the USA (MarketScan), the Philippines, Taiwan (province of China), and Poland were included, along with hospital discharge data that were processed by extracting discharges with an appropriate ICD code as primary diagnosis and adjusting using a correction factor. Specifically, we used claims data to model the ratio of inpatient claims with endometriosis as primary diagnosis to total prevalent cases of endometriosis seen in both inpatient and outpatient settings (see the section on Non-fatal data sources, identification, and extraction for a description of GBD modelling of hospital utilisation and processing of inpatient and claims data in this appendix). The total number of data sources used for the non-fatal estimation of endometriosis is provided in the following table:

Table 6: Data inputs for endometriosis morbidity modelling by parameter

Measures	Total sources	New sources	Countries with data
Prevalence	335	35	51
Incidence	9	2	6

Data processing

Any datum referring to a sample of women with an age range that did not entirely fit within a GBD age group was split into age-specific datapoints based on the age pattern predicted by GBD 2017 DisMod-MR 2.1 models.

Once the data were age-split, we adjusted data collected using non-reference case definitions, study populations, or other data collection methods using an MR-BRT analysis. To do this, first we counted the number of observations of each alternate definition that matched with a corresponding observation

from the reference definition. We matched observations by age group and location (at the region level), and when the midpoint of the study was within five years of the midpoint of the reference definition observation.

All matched observations were paired with one another and logit-transformed, and the difference of the mean values were calculated in logit space. The standard error of the difference in logits was calculated using the delta method. As for uterine fibroids, adjustment of data inputs for endometriosis was conducted in two steps, using MR-BRT and following the general steps to GBD crosswalking described above. In the first step, we adjusted only clinical data to a common standard, by using claims data as the reference definition and inpatient hospital data as the alternative definition; for this, we used logit-transformed mean difference and standard errors for matched claims and hospital data as inputs to run a MR-BRT model with a cubic spline on age and four knots, trimming 10% of the data and assuming linear tails. Our final model results for this crosswalk process are illustrated in the next figure.

Endometriosis MR-BRT crosswalk adjustment factors by age for hospital (alternate) to claims (reference) data.

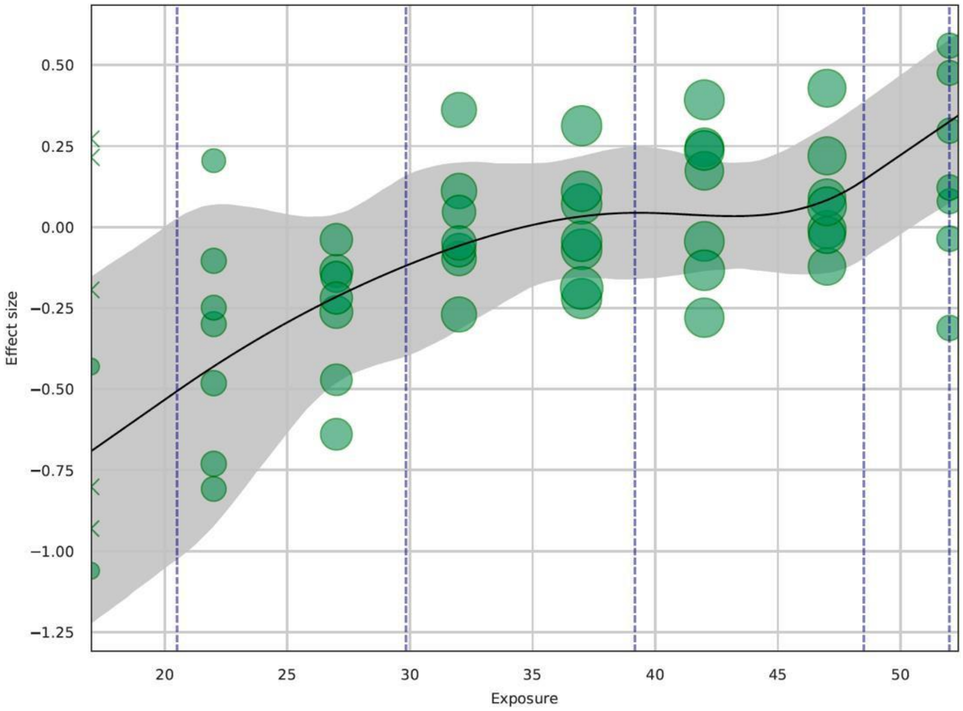

**Exposure on the x-axis is GBD age group and effect size is the logit-transformed difference of inpatient to claims data.*

According to this model, hospital data underestimated the number of endometriosis cases for the earlier age groups. After age 35, the inverse relationship is true.

After the first crosswalk, we performed a network MR-BRT analysis to adjust the data sources that use alternative definitions (clinical data and self-report endometriosis cases) considering the ACOG definition as the reference. The adjustment factors for each of the covariates included in the model are summarised in the following table.

Table 7: MR-BRT crosswalk adjustment factors for endometriosis to standardise to ACOG definition

Data input	Reference or alternative case definition	Gamma	Beta coefficient, logit (95% CI)	Adjustment factor*
ACOG definition	Reference	1.13	---	---
Self-report	Alt		0.15 (0.13 to 0.17)	0.54 (0.53 to 0.55)
Clinical data	Alt		–0.22 (-0.23 to -0.21)	0.44 (0.43 to 0.45)

**MR-BRT crosswalk adjustments can be interpreted as the factor the alternative case definition is adjusted by to reflect what it would have been had it been measured using the reference case definition. If the log/logit beta coefficient is negative, then the alternative is adjusted up to the reference. If the log/logit beta coefficient is positive, then the alternative is adjusted down to the reference.*

***The adjustment factor column is the exponentiated beta coefficient. For log beta coefficients, this is the relative rate between the two case definitions. For logit beta coefficients, this is the relative odds between the two case definitions.*

Modelling strategy

We used DisMod-MR 2.1, a Bayesian meta-regression epidemiological tool, to generate incidence, prevalence, and remission estimates for endometriosis by age, sex, year, and location.

As in previous GBD iterations, incidence was assumed to be zero except between the ages of 15 and 50 years. This is because a woman must enter puberty before she can get endometriosis, and the condition remits spontaneously after the onset of menopause. The Bayesian prior on remission was bounded from 0 to 0.2 before the age of 50 years and was set to be equal to 0.2 (1/remission = duration = 5 years) from the age of 51 years through the end of life. We also bound the excess mortality rate among the prevalent cases to a maximum of 3 deaths per 10,000 person-years and used the Healthcare Access and Quality (HAQ) Index as the lone predictive covariate on this parameter.

Prior to GBD 2019, no covariates were used to inform the prevalence estimates of endometriosis. For GBD 2019, a non-systematic literature review was conducted to identify possible predictive covariates, which identified the following: the summary exposure values (SEV) for smoking, high body-mass index, low physical activity, and alcohol consumption; the age-standardised death rate (lnASDR) of sexually transmitted infections (STIs); the prevalence of pelvic inflammatory diseases; prevalence of contraception; and total fertility rate (TFR). The covariates were tested in preliminary models in GBD 2019, and TFR and the risk-weighted prevalence of smoking were selected as covariates in the final model. These covariates were used again in GBD 2021, with corresponding beta coefficients and exponentiated values as shown in the following:

Table 8: Summary of covariates used in the endometriosis DisMod-MR meta-regression model

Covariate name	Measure	Beta value	Exponentiated value
Total fertility rate	Prevalence	0.29 (0.25–0.33)	1.33 (1.28–1.39)
Age-standardised SEV for smoking	Prevalence	0.19 (0.12–0.25)	1.21 (1.13–1.29)
Healthcare Access and Quality Index	Excess mortality rate	–0.01 (–0.019 to –0.00072)	0.99 (0.98–1.00)

Severity splits & disability weights

The basis of the GBD disability weight (DW) survey assessment are lay descriptions of sequelae highlighting major functional consequences and symptoms. The GBD 2010 systematic literature review identified three studies that were combined to inform the severity distribution of those with endometriosis. Only one study reported on the proportion of endometriosis cases with chronic abdominal pain,[11] and another was found to contain data on the distribution of pain severity.[12] Data from each study were combined to calculate a pooled proportion of 69.4% (95% CI 66.5–72.4) of women with endometriosis who have abdominal pain and, of those who suffer pain, 8.2% (7.3–9.1) with mild pain; 75.1% (73.6–76.5) with moderate pain; and 16.8% (15.5–18.0) with severe pain. No information was available on the proportion of time spent with pain. From the Australian Longitudinal Women's Health Study (ALWHS), we were able to derive an estimate of the proportion of women who have endometriosis and long-term infertility.[13] The excess risk of being permanently infertile with endometriosis (relative to no endometriosis) was calculated as the difference in risk of being infertile with and without endometriosis. This excess risk was 6.2% (4.3–8.3). Disability weights for each sequela are listed below for reference.

Table 9: Health states used in estimating YLDs due to endometriosis.

Severity	Lay description	DW (95% CI)
Abdominopelvic problem, mild	Has some pain in the belly that causes nausea but does not interfere with daily activities.	0.011 (0.005–0.021)
Abdominopelvic problem, moderate	Has pain in the belly and feels nauseous. The person has difficulties with daily activities.	0.114 (0.078–0.159)
Abdominopelvic problem, severe	Has severe pain in the belly and feels nauseous. The person is anxious and unable to carry out daily activities.	0.324 (0.219–0.442)
Infertility, primary	Wants to have a child and has a fertile partner, but the couple cannot conceive.	0.008 (0.003–0.015)
Infertility, secondary	Has at least one child and wants to have more children. The person has a fertile partner, but the couple cannot conceive.	0.005 (0.002–0.011)

Polycystic ovarian syndrome (PCOS)

Flowchart

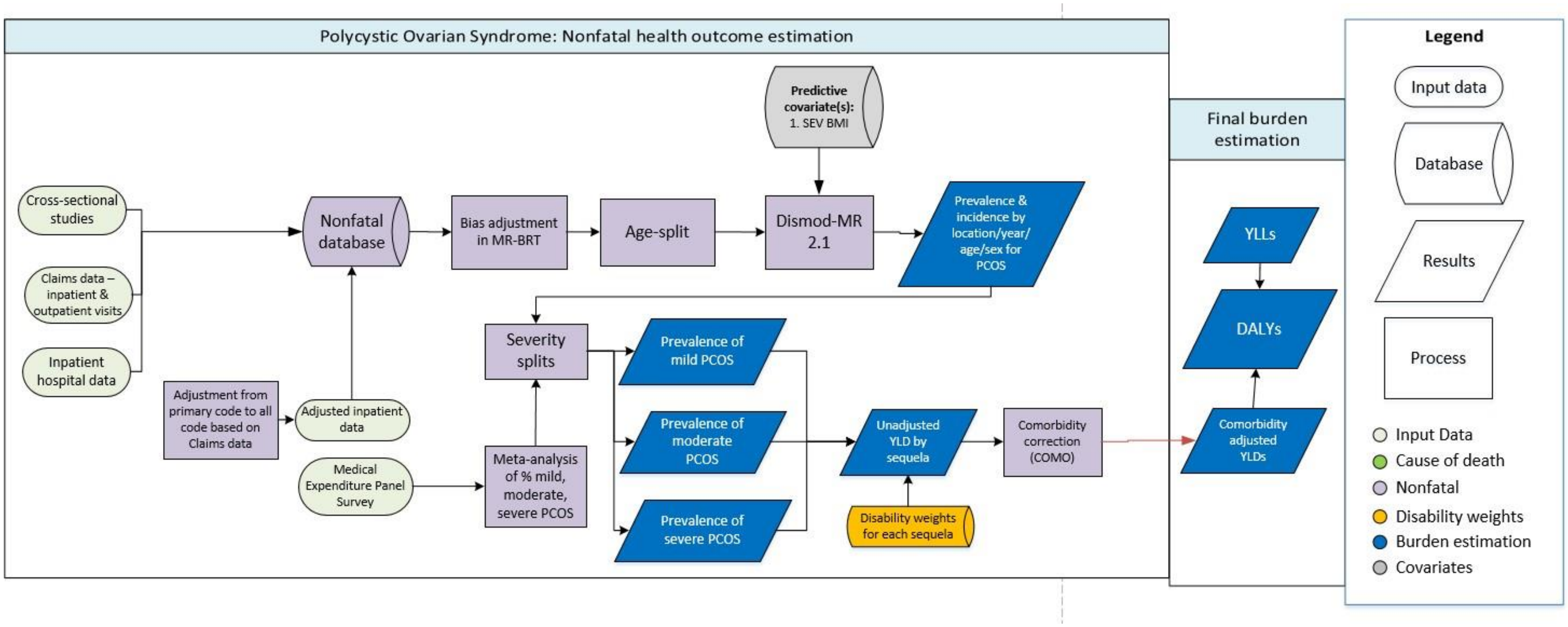

Case definition

Polycystic ovarian syndrome (PCOS) is an endocrinopathy characterised by hyperandrogenism, ovulatory dysfunction, and polycystic ovaries which can lead to infrequent menstruation, excess hair growth (hirsutism), acne, obesity, and infertility among women.[5,13] Women with PCOS often have enlarged ovaries that contain pockets of fluid.

There is no universally accepted definition of PCOS.[6] Expert-generated diagnostic criteria include the National Institutes of Health (NIH) diagnostic,[7] the Rotterdam criteria,[8] and the Androgen Excess Society (AES) definition.[9] All diagnostic approaches require the presence of more than one sign or symptom and recommend that secondary causes (such as congenital adrenal hyperplasia, hyperprolactinemia, and androgen-secreting neoplasms) should first be excluded.

In GBD 2019, we standardised the reference definition of all gynaecological diseases, including PCOS, to the ACOG definitions. According to ACOG, however, PCOS diagnosis can be accomplished using any of the three diagnostic approaches mentioned previously (NIH, Rotterdam, or AES).[5] As the Rotterdam and AES definitions have been criticised for including more mild phenotypes,[9] we continued using the NIH definition, which noted the disorder as having 1) hyperandrogenism and/or hyperandrogenemia, 2) oligo-ovulation, and 3) exclusion of known disorders, as our reference definition.[6]

Input data

For GBD 2021, we used the same data utilised in GBD 2019, which include peer-reviewed studies from a previous systematic review, plus clinical administrative data aggregated and processed annually by GBD.

For GBD 2010, a systematic review of PCOS throughout the world was conducted. Ovid MEDLINE, EMBASE, CINAHL, CAB abstracts, WHOLIS, and ISGLE and PUBMED database were searched. Search strings were as follows:

> **PUBMED:** (“Polycystic Ovary Syndrome”[Mesh] OR “Polycystic Ovary Syndrome” OR “Sclerocystic Ovary Syndrome” OR “Sclerocystic Ovarian Degeneration” OR “Stein-Leventhal Syndrome” OR “Stein Leventhal Syndrome” OR “Sclerocystic Ovaries” OR “Sclerocystic Ovary”) AND (“Incidence”[Mesh] OR Incidence OR Incidences OR “Prevalence”[Mesh] OR Prevalence OR Prevalences)
>
> **EMBASE:** (“ovary polycystic disease”/exp OR “cystic ovary” OR “micropolycystic ovary” OR “multiple follicle cyst” OR “ovary polycystic syndrome” OR “ovary, micropolycystic” OR “ovary, polycystic” OR “polycystic ovarian disease” OR “polycystic ovary” OR “polycystic ovary disease” OR “polycystic ovary syndrome”) AND (‘incidence’/exp OR incidence OR incidences OR ‘prevalence’/exp OR prevalence OR prevalences)

We excluded reviews and studies that did not provide primary data on epidemiological parameters (eg, commentary) and clearly non-representative studies (eg, of only high-risk pregnant women).

In GBD 2021, in addition to data from the GBD 2010 systematic review, claims data from the USA (MarketScan), the Philippines, Taiwan (province of China), and Poland were included, along with hospital discharge data that were processed by extracting discharges with an appropriate ICD code as primary diagnosis and adjusting using a correction factor. Specifically, we used claims data to model the ratio of inpatient claims with PCOS as primary diagnosis to total prevalent cases of PCOS seen in both inpatient and outpatient settings (see the section on Non-fatal data sources, identification, and extraction for a description of GBD modelling of hospital utilisation and processing of inpatient and claims data in this

appendix). The total number of data sources used for the non-fatal estimation of PCOS is provided in the following table.

Table 10: Data inputs for polycystic ovarian syndrome morbidity modelling by parameter

Measures	Total sources	Countries with data
Prevalence	255	30

Data processing

Prior to modelling, we performed age-splitting to ensure all data fit into specific GBD standard age groups. Briefly, the age-splitting algorithm uses population weights that are determined by dividing the result predicted by GBD 2017 DisMod-MR 2.1 models for a specific age group by the result for the aggregate age specified in a given input datapoint. Age-specific values were then calculated by multiplying the aggregate input datapoint by these age specific weights.

Because prevalence and incidence of PCOS among reproductive-aged women vary according to the diagnostic criteria, we used the NIH case definition as the reference definition and adjusted the data from alternative definitions using two MR-BRT models. Acceptable alternate definitions included the Rotterdam definition, AES definition, self-report, and clinical data. We started by evaluating the number of observations of each alternate definition that matched with a corresponding observation from the reference definition. Due to data scarcity, we only found "between-study" matches (observations of different studies matched by age group and location and when the midpoint of the study was within five years of the midpoint of the reference definition observation).

To perform the crosswalk, all observations that matched were paired with one another and logit-transformed and the difference of the logit mean values was calculated. The standard error of the logit difference was calculated using the delta method. Adjustment of data inputs for PCOS was conducted in two steps using the MR-BRT steps described in uterine fibroids. In the first step, we adjusted only clinical data to a common standard, by using claims data as the reference definition and inpatient hospital data as the alternative definition, entering the logit-transformed mean difference and standard error as inputs to run a MR-BRT model, trimming 10% of the data and assuming linear tails. Our final model results for this crosswalk process are illustrated in the next figure and table.

Table 11: MR-BRT crosswalk adjustment factors for polycystic ovarian syndrome to standardise between different clinical administrative data types

Data input	Reference or alternative case definition	Gamma	Beta coefficient, logit (95% CI)	Adjustment factor*
Claims data	Reference	0	---	---
Inpatient data	Alt		–1.52 (–2.05 to –0.95)	0.18 (0.11 to 0.28)

**MR-BRT crosswalk adjustments can be interpreted as the factor the alternative case definition is adjusted by to reflect what it would have been had it been measured using the reference case definition. If the log/logit beta coefficient is negative, then the alternative is adjusted up to the reference. If the log/logit beta coefficient is positive, then the alternative is adjusted down to the reference.*

***The adjustment factor column is the exponentiated beta coefficient. For log beta coefficients, this is the relative rate between the two case definitions. For logit beta coefficients, this is the relative odds between the two case definitions.*

For the second MR-BRT model, we used a network analysis to crosswalk the different diagnostic criteria including the NIH definition as the reference and the Rotterdam diagnostic criteria, the AES definition, self-report, and clinical data as alternative definitions. The adjustment factors for each of the included covariates in the models are summarised in the following table.

Table 12: MR-BRT crosswalk adjustment factors for polycystic ovarian syndrome to standardise between different diagnostic criteria

Data input	Reference or alternative case definition	Gamma	Beta coefficient, logit (95% CI)	Adjustment factor*
NIH definition	Reference	0.43	---	---
Rotterdam definition	Alt		0.22 (0.12 to 0.32)	0.55 (0.47 to 0.58)
AES definition	Alt		–0.006 (–0.10 to 0.09)	0.50 (0.47 to 0.52)
Self-report cases	Alt		–0.60 (–0.69 to –0.52)	0.35 (0.33 to 0.37)
Clinical data	Alt		–3.88 (–5.48 to –2.33)	0.02 (0.004 to 0.09)

**MR-BRT crosswalk adjustments can be interpreted as the factor the alternative case definition is adjusted by to reflect what it would have been had it been measured using the reference case definition. If the log/logit beta coefficient is negative, then the alternative is adjusted up to the reference. If the log/logit beta coefficient is positive, then the alternative is adjusted down to the reference.*
***The adjustment factor column is the exponentiated beta coefficient. For log beta coefficients, this is the relative rate between the two case definitions. For logit beta coefficients, this is the relative odds between the two case definitions.*

Modelling strategy

We modelled prevalence, incidence, and remission of PCOS using DisMod-MR 2.1. Incidence was set to zero prior to 10 years of age and after 55 years of age to reflect that women are only susceptible between menarche and menopause. Remission until age 54 was bounded to have a maximum value of 1 per 10 person-years. After age 55, no priors for remission were set. PCOS is not considered a cause of death, and therefore, excess mortality rate was set to 0. We set the minimum coefficient of variation (which helps determine the influence of Bayesian priors from the geographical cascade relative to local data) to 0.8 and set the parameter xi (which controls age smoothing) to have a maximum value of 3. In addition, a decreasing slope prior for incidence starting at age 16 was used to help the model to match the highest incidence observed in the data among younger ages (13–20 years). The time span of data used to fit for a particular year was set to five years.

In GBD 2019, a set of potentially associated factors were selected based on a non-systematic literature review and evaluated for predictive power in a series of test models; these included the summary exposure values (SEV) for smoking, high body-mass index, low physical activity, and alcohol consumption; the age-standardised death rate (lnASDR) of sexually transmitted infections (STIs) from the previous round's COD analyses; prevalence of pelvic inflammatory disease from the previous round's non-fatal analyses; prevalence of contraception; total fertility rate; and the Socio-demographic Index (SDI). Most of the covariates from this list were not associated with the prevalence of PCOS in test models. Because obesity plays an important role in the aetiology of the syndrome, we include the relative risk-weighted prevalence of high body-mass index as a predictive covariate to help drive the magnitude of prevalence estimates in areas of sparse or absent data (the coefficients are shown in the following table).

Table 13: Covariates used in the polycystic ovarian syndrome DisMod-MR meta-regression model

Covariate name	Measure	Beta value	Exponentiated value
Age-standardised SEV for high body-mass index	Prevalence	0.65 (0.51–0.77)	1.91 (1.67–2.15)

Severity splits

The basis of the GBD disability weight (DW) survey assessment are lay descriptions of sequelae highlighting major functional consequences and symptoms. Unfortunately, no health states specific to PCOS were included in the GBD disability weights survey. The main sequelae of PCOS are infertility and hyperandrogenism/hirsutism, the latter of which was approximated with the health state of "disfigurement, level 1." The NIH definition, which we designated as the reference case definition, considers that most cases of PCOS have hyperandrogenism and hirsutism, and therefore we assumed that a majority of PCOS would experience this sequela. Disability weights for each sequela are listed below for reference.

Table 14: Health states used in estimating YLDs due to polycystic ovarian syndrome

Severity	Lay description	DW (95% CI)
Disfigurement, level 1	Has a slight, visible physical deformity that others notice, which causes some worry and discomfort.	0.011 (0.005–0.021)
Infertility, primary	Wants to have a child and has a fertile partner, but the couple cannot conceive.	0.008 (0.003–0.015)
Infertility, secondary	Has at least one child and wants to have more children. The person has a fertile partner, but the couple cannot conceive.	0.005 (0.002–0.011)

Genital prolapse

Flowchart

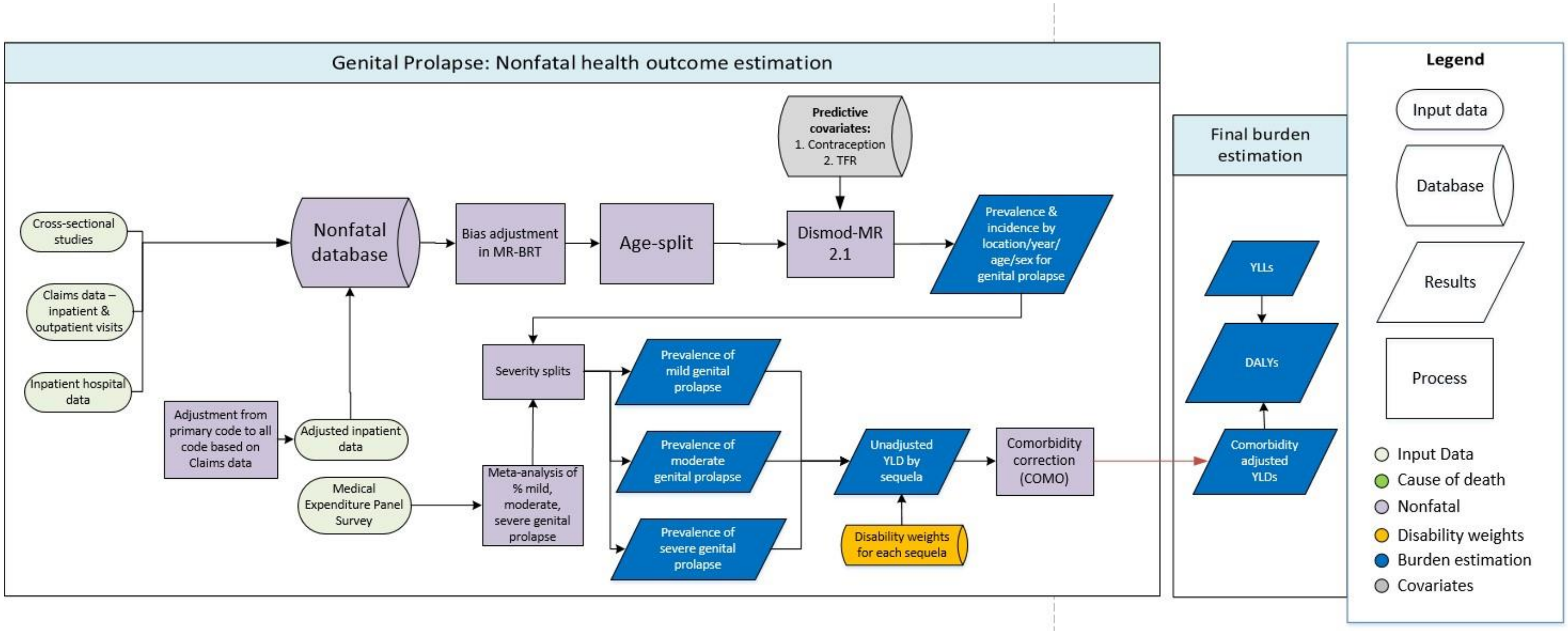

Case definition

As defined by ACOG, genital prolapse, also called pelvic organ prolapse, is the clinically relevant descent of one or more of the female pelvic structures, including the uterus, bladder, rectum, small or large bowl, or vagina.[15] Risk of prolapse increases with age and can be exacerbated by vaginal childbirth or physical strain. The ICD-10 code associated with genital prolapse is N81. In an effort to standardise the case definitions of all gynaecological diseases, in GBD 2019, we started using the ACOG definition of genital prolapse as the reference definition.[14] The ACOG definition states that mild descent of the pelvic organs should not be considered pathological unless women experience symptoms such as pressure with or without a bulge, sexual dysfunction, or if it is disrupting normal lower urinary tract or bowel function.[14]

Input data

Data sources used to inform the genital prolapse non-fatal estimates include data from peer-reviewed literature identified in a previous systematic review (mainly from population-level and community prevalence surveys), claims data, and hospital administrative data. The last comprehensive literature review was completed in GBD 2010, where we identified data on prevalence of genital prolapse using the following search strings:

> **PUBMED:** (("genital prolapse" OR "genital prolapses" OR "vaginal prolapse" OR "vaginal prolapses" OR "uterine prolapse" OR "uterine prolapses" OR "uterovaginal prolapse" OR "uterus prolapse" OR "pelvic organ prolapse" OR "urogenital prolapse" OR "vaginal vault prolapse" OR cystocele OR cystoceles OR “Vaginal enterocele” OR “urethrocele” OR “urethroceles”) AND (prevalence OR prevalences OR epidemiology OR incidence OR incidences)) OR ((“Uterine prolapse”[MeSH] OR “Pelvic organ prolapse”[MeSH] OR “cystocele”[MeSH]) AND (“Prevalence”[MeSH] OR “Epidemiology”[MeSH])

> **EMBASE:** (("genital prolapse" OR "genital prolapses" OR "vaginal prolapse" OR "vaginal prolapses" OR "uterine prolapse" OR "uterine prolapses" OR "uterovaginal prolapse" OR "uterus prolapse" OR "pelvic organ prolapse" OR "urogenital prolapse" OR "vaginal vault prolapse" OR cystocele OR cystoceles OR “Vaginal enterocele” OR “urethrocele” OR “urethroceles”) AND (‘incidence’/exp OR incidence OR incidences OR ‘prevalence'/exp OR prevalence OR prevalences)) OR ((‘Uterus prolapse’/exp, ‘Pelvic organ prolapse’/exp, ‘Cystocele’/exp, ‘Enterocele’/exp) AND (‘incidence’/exp OR incidence OR incidences OR ‘prevalence'/exp OR prevalence OR prevalences))

We excluded studies that did not provide primary data on epidemiological parameters (eg, reviews, commentary) and clearly non-representative studies.

In addition to data from the above systematic review, claims data from the USA (MarketScan), the Philippines, Taiwan (province of China), and Poland were included, along with hospital discharge data that were processed by extracting discharges with an appropriate ICD code as primary diagnosis and adjusting using a correction factor. Specifically, we used claims data to model the ratio of inpatient claims with genital prolapse as primary diagnosis to total prevalent cases of genital prolapse seen in both inpatient and outpatient settings (see the section on Non-fatal data sources, identification, and extraction for a description of GBD modelling of hospital utilisation and processing of inpatient and claims data in this appendix). The following table shows the total number of data sources consider in the non-fatal estimation process.

Table 15: Data inputs for genital prolapse morbidity modelling by parameter

Measures	Total sources	Countries with data
Prevalence	312	50

Data processing

In GBD 2021, the first step to process the data was age-sex splitting. For any datum that did not entirely fit within a GBD age group, the observation was split to be multiple age-specific datapoints based on the age pattern predicted by GBD 2017 DisMod-MR 2.1 models.

As the prevalence estimates on self-reported symptoms were markedly lower than the prevalence identified by medical examination, we used MR-BRT models to crosswalk the data collected from non-reference definitions including symptomatic cases, self-reported cases, and clinical data to the reference definition (cases of genital prolapse diagnosed by medical examination).

To perform the crosswalk, all observations that matched were paired with one another and logit-transformed, and the difference of the logit mean values was calculated. The standard error of the logit difference was calculated using the delta method. Adjustment of data inputs for genital prolapse was conducted in two steps, using the MR-BRT steps described in uterine fibroids. In the first step, we adjusted only clinical data to a common standard, by using claims data as the reference definition and inpatient hospital data as the alternative definition. We then used the logit-transformed mean difference and standard error as inputs to run a MR-BRT model with a cubic spline on age and four knots, trimming 10% of the data and assuming linear tails. Our final model results for this crosswalk process are illustrated in the next figure.

Genital prolapse MR-BRT crosswalk adjustments factors by age for hospital (alternate) and claims (reference) data.

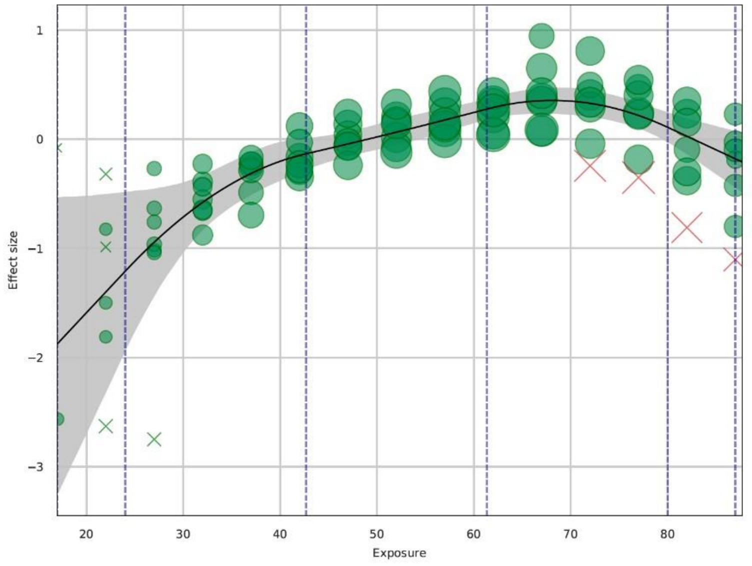

**Exposure on the x-axis is GBD age group and effect size is the logit-transformed difference of inpatient to claims data. MR-BRT model ran with a quadratic spline on age, linear tails and trimming 10% of the data.*

According to this model, hospital data underestimated the number of genital prolapse cases for younger ages and up to age 50 and after age 80. Between ages 50 to 80, the inverse relationship is true.

In the second step, clinical data (as adjusted in the first step) were included as an alternative definition along with symptomatic and self-reported cases in a network MR-BRT model, where the reference definition was cases diagnosed using the ACOG definition. The adjustment factors for each of the covariates included in the model are summarised in the following table.

Table 16: MR-BRT crosswalk adjustment factors for genital prolapse network model to standardise to ACOG definition

Data input	Reference or alternative case definition	Gamma	Beta coefficient, logit (95% CI)	Adjustment factor*
ACOG definition	Reference	0.51	---	---
Self-report	Alt		–3.48 (–4.55 to –2.43)	0.03 (0.01 to 0.08)
Symptomatic cases	Alt		–2.24 (–3.33 to –1.13)	0.10 (0.03 to 0.24)
Clinical data	Alt		–5.58 (–5.77 to –5.38)	0.004 (0.003 to 0.005)

**MR-BRT crosswalk adjustments can be interpreted as the factor the alternative case definition is adjusted by to reflect what it would have been had it been measured using the reference case definition. If the log/logit beta coefficient is negative, then the alternative is adjusted up to the reference. If the log/logit beta coefficient is positive, then the alternative is adjusted down to the reference.*

***The adjustment factor column is the exponentiated beta coefficient. For log beta coefficients, this is the relative rate between the two case definitions. For logit beta coefficients, this is the relative odds between the two case definitions.*

Modelling strategy

We used DisMod-MR 2.1 to estimate the prevalence, incidence, and remission of genital prolapse. As in previous GBD iterations, incidence was set to zero prior to 15 years of age. We set the minimum coefficient of variation to 0.8 and the time span of data used to fit for a particular year to five years. To ensure that the age pattern of the estimates was consistent with the age pattern observed in the literature and because it is highly unlikely that young women would experience genital prolapse, we marked as outliers and excluded all data that reported prevalence values higher than 5% for women under 25 years.

In GBD 2019, we also conducted a non-systematic literature review to find the main predictors of genital prolapse that could inform DisMod-MR 2.1 estimates. We tested the association between the prevalence of genital prolapse and the summary exposure values (SEV) for smoking, high body-mass index, and low physical activity; the prevalence of contraception; and total fertility rate (TFR). No significant statistical association was found between the prevalence of prolapse and most of the aforementioned covariates. In the final model, we used log-transformed total fertility rate and the prevalence of contraception as predictive covariates as multiparity is a recognised risk factor for prolapse. These two covariates were used again in GBD 2021. The following table illustrates covariates, measures, parameters, beta, and exponentiated beta values of the final model that was selected based on a combination of qualitative and quantitative goodness of fit to input data, plausibility of geographical and temporal trends, and consistency of age pattern.

Table 17: Covariates used in the genital prolapse DisMod-MR meta-regression model

Covariate name	Type	Measure	Beta value	Exponentiated value
Contraception (modern) prevalence (proportion by age)	Country covariate	Prevalence	2.02 (1.50–2.78)	7.53 (4.50–16.19)
Total fertility rate	Country covariate	Prevalence	0.83 (0.76–0.90)	2.29 (2.15–2.46)

Severity splits

The basis of the GBD disability weight (DW) survey assessment are lay descriptions of sequelae highlighting major functional consequences and symptoms. To determine the proportion of people within each domain of disability, several studies from the systematic review were identified to contain information on the proportion of women with symptoms. These data were pooled and applied to prevalence estimates. This included two studies with information on the proportion of women with prolapse who experience a bulging sensation (pooled proportion = 11.7% [95% CI 6.8–19.4]),[16,17] three that reported on the proportion with stress incontinence (pooled proportion = 52.8% [40.1–65.1]),[18–20] and one that reported on the frequency (measured as proportion of the year) of incontinence symptoms (pooled proportion = 7.9% [4.6–13.6]).[20] Percentages were combined to calculate the proportion of women who fall into both stress incontinence and bulging sensation categories. The lay descriptions and disability weights for genital prolapse are shown below.

Table 18: Severity distribution, details on the severity levels for genital prolapse, and the associated disability weight (DW) with that severity

Severity	Lay description	DW (95% CI)
Stress incontinence	Loses small amounts of urine without meaning to when coughing, sneezing, laughing, or during physical exercise.	0.02 (0.011–0.035)
Abdominopelvic problem, mild	Has some pain in the belly that causes nausea but does not interfere with daily activities.	0.011 (0.005–0.021)

Premenstrual syndrome (PMS)

Flowchart

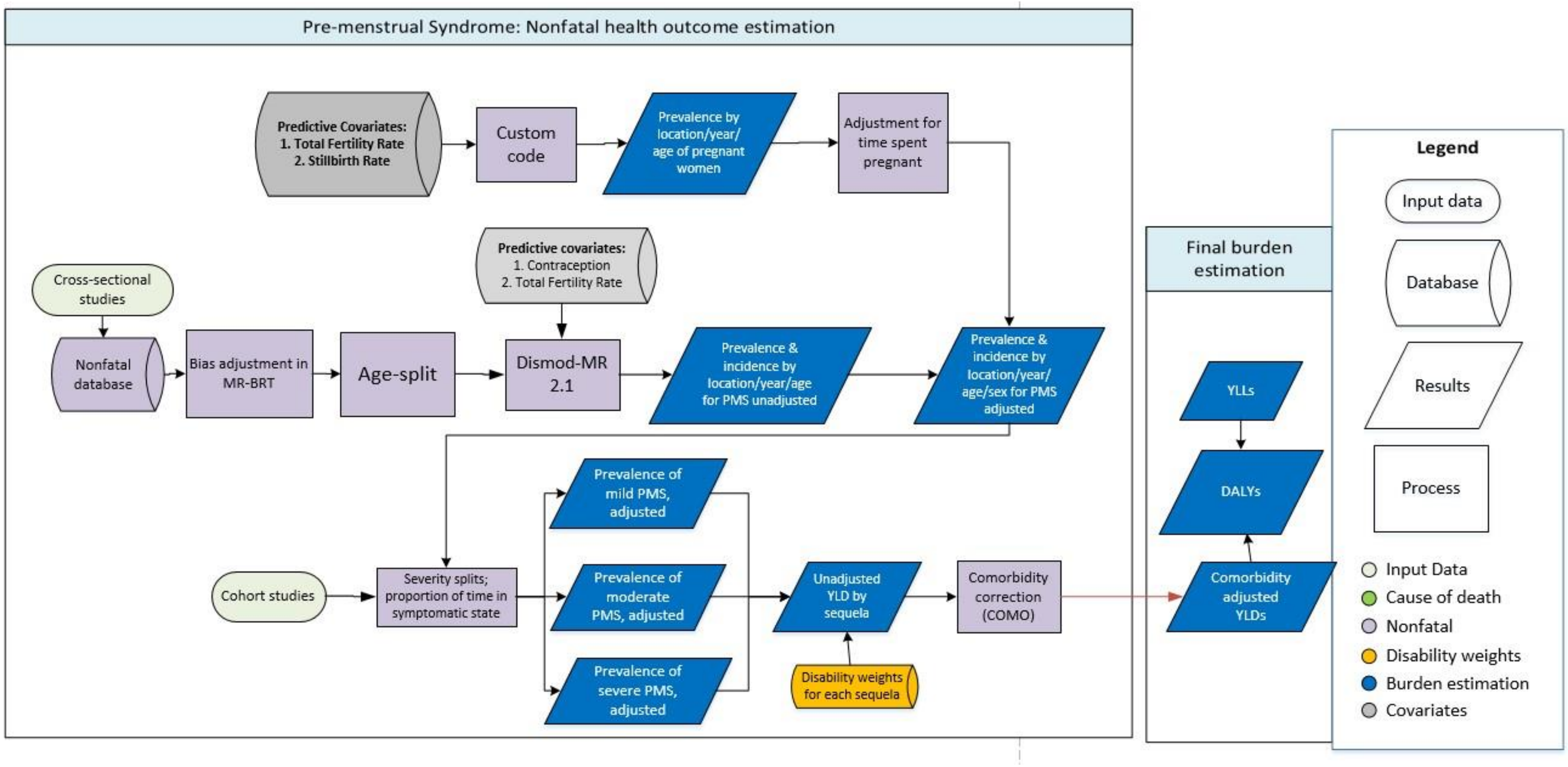

Case definition

Premenstrual syndrome (PMS) refers to psychological and physical symptoms that occur during the luteal phase of the menstrual cycle. Symptoms vary in nature and severity, but include tenderness, bloating, irritability, fatigue, abdominal pain, and altered mental states. PMS ceases when a woman is pregnant and when she reaches menopause. Lacking definitive and universally accepted diagnostic criteria for PMS, in GBD 2019, we started using the diagnostic criteria proposed by the American College of Obstetricians and Gynecologists (ACOG) as the reference definition. The ACOG definition of PMS requires at least one emotional or physical symptom to be experienced by women during the five days before menses and remit within four days of onset of menses, with no recurrence at least until day 13 of the cycle, in each of three prior menstrual cycles. Additionally, identifiable dysfunction in social or economic performance and prospective confirmation for two cycles are required.

Input data

A comprehensive literature review was completed in GBD 2010, where we identified data on prevalence of PMS using the following search strings:

> **PUBMED:** "Premenstrual Syndrome"[Mesh] OR (premenstrual AND syndrome) OR (premenstrual AND syndrome) OR (premenstrual AND tension) OR (premenstrual AND tensions) OR (premenstrual AND stress) OR "premenstrual dysphoric disorder" OR "premenstrual dysphoric disorders" OR (menstrual AND distress) AND (("Incidence"[Mesh] OR incidence OR incidences OR onset OR occurrence) OR ("Prevalence"[Mesh] OR prevalence OR prevalences)
>
> **EMBASE:** 'premenstrual syndrome'/exp OR 'premenstrual dysphoric disorder'/exp OR (premenstrual AND syndrome) OR (premenstrual AND syndromes) OR (premenstrual AND tension) OR (premenstrual AND tensions) OR (premenstrual AND stress) OR "premenstrual dysphoric disorder" OR "premenstrual dysphoric disorders" OR (menstrual AND distress) AND (('incidence'/exp OR incidence OR incidences OR onset OR occurrence) OR (prevalence/exp OR prevalence OR prevalences)

Exclusion criteria for the initial systematic review were studies that did not provide primary data on epidemiological parameters (eg, commentary) and reviews.

Administrative data (claims and hospital discharge data aggregated and processed for GBD and coded with ICD-10 code N94.3) were considered for inclusion in modelling PMS, but were ultimately not incorporated, as we believe that the likelihood that women with PMS would seek care in the medical system would be more variable across time and space than the true epidemiological variation.

Table 19: Data inputs for premenstrual syndrome morbidity modelling by parameter

Measures	Total sources	Countries with data
Prevalence	46	23

Data processing

We performed age-splitting to ensure all data fit into GBD standard age groups. In other words, for any datum that did not entirely fit within a GBD age group, the observation was split to be multiple age-specific datapoints based on the age pattern predicted by GBD 2017 DisMod-MR 2.1 models.

Case definitions for PMS vary widely, including varying constellations of symptoms and varying requirements for when symptoms occur relative to menses and over how many cycles; ascertainment methods and recall periods also vary in published studies. We use as our reference definition the ACOG criteria, which state that the patient reports at least one of each of the following affective and somatic

symptoms during the five days before their menses and these appear in three consecutive cycles: depression, angry outbursts, irritability, anxiety, confusion, social withdrawal; breast tenderness, abdominal bloating, headache, or swelling of extremities. Alternative ("non-reference") data include those that use the WHO/ICD-10 definition of having at least one premenstrual symptom during period of assessment, those that use the Premenstrual Symptoms Screening Tool (PSST) definition, those that limit their measurement to cases of premenstrual syndrome described as "moderate or severe", those that employ other definitions of PMS that are not frequently used, and those that report period-prevalence.

To adjust the non-reference data, we followed the general steps to crosswalking on GBD, as described above. Specifically, we first evaluated the number of observations of each alternate type that matched with a corresponding observation of the reference type. Due to data scarcity, we found only "between-study" matches. That means we matched observations of different studies from the same region where the midpoint of the age-range for the observation was within 20 years of the midpoint of the reference definition observation. Using the same logic, we found all the matches among all possible combinations of alternative data types. All observations that matched were paired with one another and logit transformed, and the difference of the mean values was calculated in logit space. The standard error of the logit difference was calculated using the delta method, and these were entered into a meta-regression—Bayesian, regularised, trimmed (MR-BRT) network model, trimming 10% of the data. The adjustment factors for each of the included non-reference data characteristics in the models are summarised in the following table.

Table 20: MR-BRT crosswalk adjustment factors for premenstrual syndrome to standardise to ACOG definition

Data input	Reference or alternative case definition	Gamma	Beta coefficient, logit (95% CI)	Adjustment factor*
ACOG definition	Reference	1.03	---	---
WHO/ICD-10 definition	Alt		2.08 (1.99 to 2.17)	0.89 (0.87 to 0.90)
Premenstrual syndrome screening tool	Alt		–1.47 (–1.32 to –1.17)	0.19 (0.21 to 0.24)
Other definitions	Alt		–0.42 (–0.33 to –0.05)	0.39 (0.41 to 0.49)
Moderate and severe cases only	Alt		–0.38 (–0.45 to –0.30)	0.41 (0.39 to 0.42)
Period prevalence studies	Alt		–0.60 (–0.67 to –0.52)	0.35 (0.33 to 0.37)

**MR-BRT crosswalk adjustments can be interpreted as the factor the alternative case definition is adjusted by to reflect what it would have been had it been measured using the reference case definition. If the log/logit beta coefficient is negative, then the alternative is adjusted up to the reference. If the log/logit beta coefficient is positive, then the alternative is adjusted down to the reference.*

***The adjustment factor column is the exponentiated beta coefficient. For log beta coefficients, this is the relative rate between the two case definitions. For logit beta coefficients, this is the relative odds between the two case definitions.*

Modelling strategy

After the data adjustments, we used DisMod-MR-2.1 to estimate the prevalence, incidence, and remission of PMS. As in previous GBD iterations, incidence was set to zero prior to 15 years of age and after 49 years for GBD 2021. This is because a woman is by definition only susceptible between menarche and menopause. We assumed no excess mortality from PMS and further assumed that the

duration of the condition is between 3.3 and 5 years (remission rate = 0.2–0.3 per person-year). As in GBD 2019, we set the minimum coefficient of variation to 0.8 and the time span of data used to fit a particular year to 5 years.

In GBD 2019, potential predictive covariates for PMS were selected a priori based on a non-systematic literature review and tested in preliminary models; these included the summary exposure values (SEV) for smoking, high body-mass index, high sodium intake, alcohol consumption, and low physical activity. The final GBD 2019 model included risk-weighted prevalence of BMI, which was selected based on a combination of qualitative and quantitative goodness of fit to input data, plausibility of geographical and temporal trends and consistency of age pattern. The same predictive covariate was employed in GBD 2021. The following table shows the coefficients for the covariates used in the PMS model.

Table 21: Summary of covariates used in the DisMod-MR meta-regression model for premenstrual syndrome

Covariate name	Type	Measure	Beta value	Exponentiated value
Age-standardised SEV for high body-mass index	Country covariate	Prevalence	–0.19 (–0.55 to 0.11)	0.83 (0.58 to 1.11)

Post-modelling adjustment

Studies on the prevalence of PMS consistently excluded women who were not regularly menstruating. To re-parameterise our estimates to reflect the prevalence in the entire population of women aged 10–54 years, we divided DisMod estimates of PMS by the prevalence of pregnancy; in GBD 2019 and 2021, the prevalence of pregnancy estimated for the purpose combined GBD estimates of age-specific fertility rate (ASFR) and the stillbirth ratio (SBR). The equation used to compute the prevalence of pregnancy was as follows:

$$\textit{Prevalence of pregnancy} = (ASFR + (SBR * ASFR)) * 46/52$$

Where ASFR is the age-specific fertility rate, SBR is the stillbirth ratio (stillbirths per livebirth), and 46/52 is the proportion of the year spent pregnant (40 weeks) and postpartum (6 weeks). This is in contrast to GBD 2017 and earlier, when prevalence of pregnancy was estimated via a DisMod model using the UNPOP fertility estimates as input data.

Severity splits and disability weights

The basis of the GBD disability weight (DW) survey assessment are lay descriptions of sequelae highlighting major functional consequences and symptoms. Unfortunately, no specific disability weights for PMS were estimated during the GBD Disability Weight Measurement Survey. Instead, we identified two health states – abdominopelvic problem (mild) and major depression (mild) – as the closest approximations of the symptoms associated with PMS. To determine the proportion of people within each of these severity levels, five studies were consulted. Three of the prevalence studies in the systematic review provided information on the proportion of PMS cases who feel depressed.[21,23] The pooled proportion was 74.2% (95% CI 69.6–78.3). Two other studies addressed the proportion of women with PMS who experience abdominal pain.[24,25] The pooled proportion was 41.1% (31.7–51.3). The lay descriptions and disability weights for premenstrual syndrome are shown below.

Table 22: Severity distribution, details on the severity levels for premenstrual syndrome and the associated disability weight (DW) with that severity

Severity	Lay description	DW (95% CI)
Major depressive disorder, mild episode	Feels persistent sadness and has lost interest in usual activities. The person sometimes sleeps badly, feels tired, or has trouble concentrating but still manages to function in daily life with extra effort.	0.145 (0.099–0.209)
Abdominopelvic problem, mild	Has some pain in the belly that causes nausea but does not interfere with daily activities.	0.011 (0.005–0.021)

Other gynaecological conditions – non-menstrual disorders

Flowchart

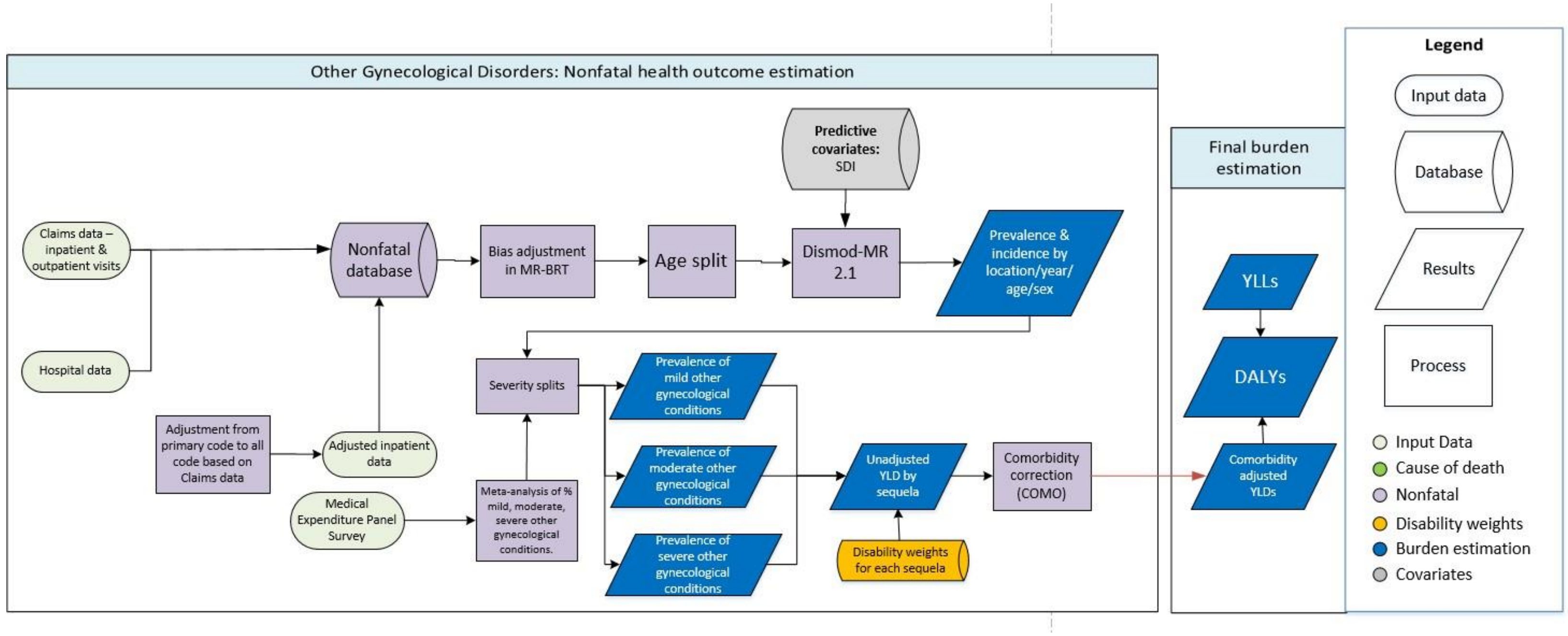

Case definition

Other gynecological diseases encompass all gynaecological disorders that are not menstruation- or bleeding-related and do not fall under the heading of any of the other gynaecological causes in the GBD. Specifically, other gynaecological disorders include breast disorders; inflammatory disease of cervix uteri; diseases of Bartholin's gland; other inflammation of vagina and vulva; vulvovaginal ulceration and inflammation in diseases classified elsewhere; non-inflammatory disorders of ovary, fallopian tube, and broad ligament; other non-inflammatory disorders of the uterus, cervix, vagina, vulva, and perineum; and menopausal and other perimenopausal disorders.

Input data

Data inputs included claims data from the USA (MarketScan), the Philippines, Taiwan (province of China), and Poland, along with hospital discharge data that were processed by extracting discharges with an appropriate ICD code as primary diagnosis and adjusting using a correction factor. We used claims data to model the ratio of inpatient claims with uterine fibroids as primary diagnosis to total prevalent cases of uterine fibroids seen in both inpatient and outpatient settings (see the section on Non-fatal data sources, identification, and extraction for a description of GBD modelling of hospital utilisation and processing of inpatient and claims data in this appendix). The total number of data sources used in the non-fatal estimation process are shown in the following table.

Table 23: Data inputs for other gynaecological (including other menstrual- and non-menstrual-related disorders) morbidity modelling by parameter

Measures	Total sources	Countries with data
Prevalence	300	46
Other	15	1

Data processing

A detailed explanation of the clinical data processing is described elsewhere in the appendix. In accordance with GBD 2021 principles for data processing, to make hospital inpatient data and claims data comparable, we began by evaluating the number of observations from hospital inpatient data (alternate definition) that matched with a corresponding observation from claims data (reference definition). We matched the observations by age group and location. All observations that matched were paired with one another and logit transformed, the difference of the mean logit values of each was calculated. The standard error of the logit difference was calculated using the delta method. To perform the crosswalk, we used a meta-regression—Bayesian, regularised, trimmed (MR-BRT). In this model we trimmed 10% of the data and added a cubic spline on age, assuming linear tails. Our final model results for this crosswalk process are illustrated in the following figure.

MR-BRT crosswalk adjustments factors by age for hospital (alternate) and claims (reference) data.

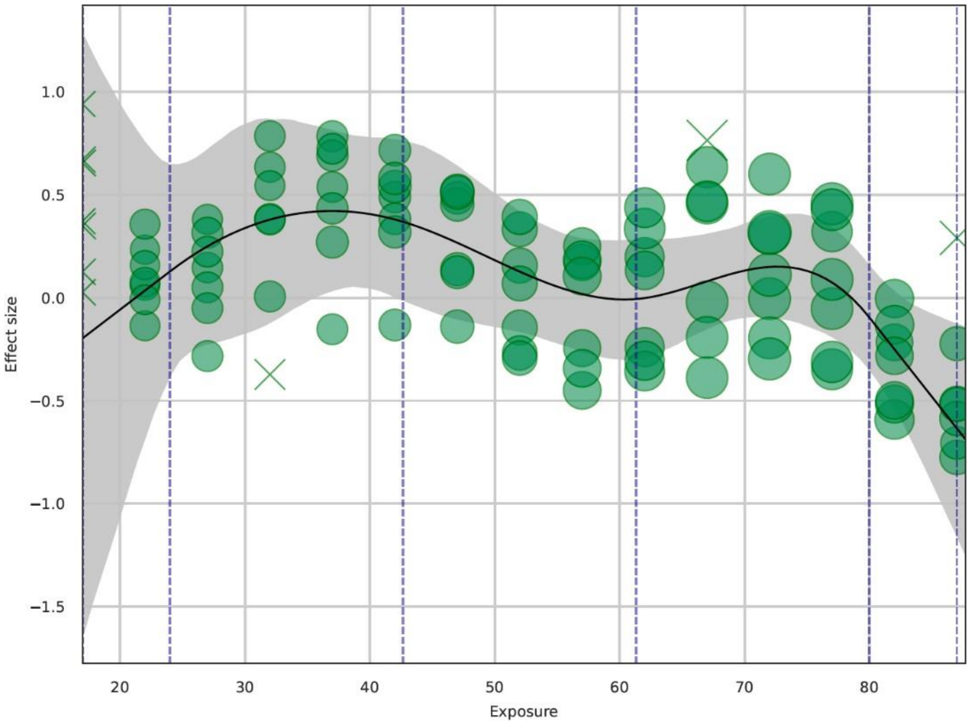

**Exposure on the x-axis is GBD age group and effect size is the logit-transformed difference of inpatient to claims data.*

According to this model, hospital data overestimated the number of other gynaecological diseases for most of the age groups. Before age 20 and after age 75, the inverse relationship is true.

Modelling strategy

We used DisMod-MR 2.1 to estimate the burden of other gynaecological diseases. Incidence was set to zero prior to 15 years of age, and we assumed no excess mortality from other gynaecological conditions over the same age range. We set the minimum coefficient of variation to 0.8 and the time span of data used to fit a particular year to 5 years.

In GBD 2019, we evaluated the association between the prevalence of these conditions and potential predictive covariates in a series of test models; these included the summary exposure values (SEV) for smoking, high body-mass index, sodium intake, alcohol consumption, and low physical activity; Socio-demographic Index (SDI); total fertility rate; use of contraception; prevalence of pelvic inflammatory diseases; and the age-standardised rate of sexually transmitted infections. However, none of the prior mentioned variables, except SDI, were associated with the prevalence of these conditions. Thus, the final GBD 2019 model included SDI as the only predictor and was selected based on a combination of qualitative and quantitative goodness of fit to input data, plausibility of geographical and temporal trends, and consistency of age pattern. This predictive covariate was employed again in GBD 2021, with the following coefficient:

Table 24: Summary of covariates used in the DisMod-MR meta-regression model for other gynaecological diseases

Covariate name	Measure	Beta value	Exponentiated value
Socio-demographic Index	Prevalence	–0.97 (–0.99 to 0.92)	0.38 (0.37–0.041)

Severity splits & disability weights

The basis of the GBD disability weight (DW) survey assessment are lay descriptions of sequelae highlighting major functional consequences and symptoms. To determine the proportion of women with other gynaecological conditions who fall into each severity level of abdominopelvic problem, data from the Medical Expenditure Panel Survey (MEPS) were used. MEPS is an overlapping panel survey of the non-institutionalised USA population that collects data on respondents' health service interactions. Panels are initiated every year. Each panel is two years long and consists of five rounds. In 2000, MEPS began using 12-Item Short Form Surveys (SF-12) to collect data on functional health status. The SF-12 survey is administered twice per panel (about once per year). The lay descriptions and disability weights for other gynaecological conditions are shown in the following table.

Table 25: Severity distribution, details on the severity levels for other gynaecological diseases, and the associated disability weight (DW) with that severity

Severity	Lay description	DW (95% CI)
Abdominopelvic problem, mild	Has some pain in the belly that causes nausea but does not interfere with daily activities.	0.011 (0.005–0.021)
Abdominopelvic problem, moderate	Has pain in the belly and feels nauseous. The person has difficulties with daily activities.	0.114 (0.078–0.159)
Abdominopelvic problem, severe	Has severe pain in the belly and feels nauseous. The person is anxious and unable to carry out daily activities.	0.324 (0.219–0.442)

Other gynaecological conditions – menstrual disorders

Flowchart

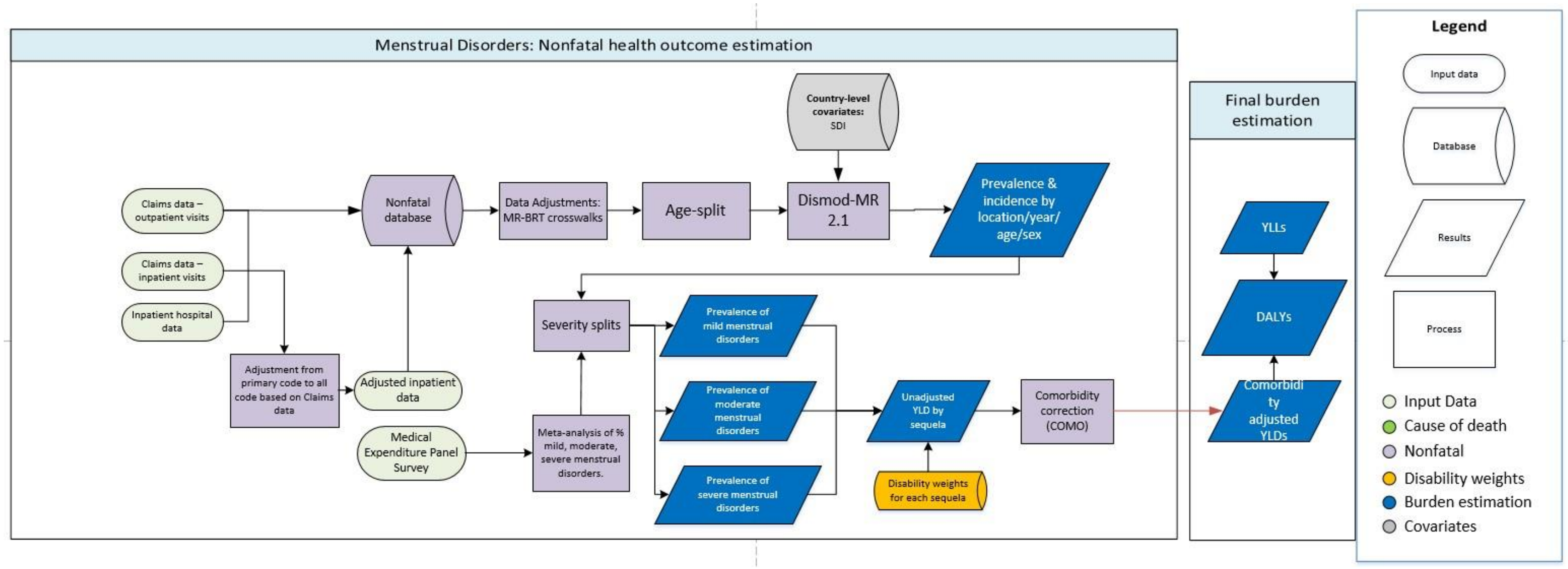

Case definition

Menstrual disorders encompasses all gynaecological disorders that are menstruation- or bleeding-related that do not fall under the heading of any of the other named gynaecological causes in the GBD. Specifically, menstrual disorders include absent, scanty, and rare menstruation, pain, and other conditions associated with female genital organs and menstrual cycle as defined by the ICD.

Input data

We used claims data from the USA (MarketScan), Philippines, Taiwan (province of China), and Poland, along with hospital discharge data that were processed by extracting discharges with an appropriate ICD code as primary diagnosis and adjusting using a correction factor. Specifically, we used claims data to model the ratio of inpatient claims with menstrual disorder as primary diagnosis to total prevalent cases of menstrual disorders seen in both inpatient and outpatient settings (see the section on Non-fatal data sources, identification, and extraction for a description of GBD modelling of hospital utilisation and processing of inpatient and claims data in this appendix). The total number of data sources used for the non-fatal estimation of menstrual disorders is provided in the following table.

Table 26: Data inputs for menstrual disorders morbidity modelling by parameter

Measures	Total sources	Countries with data
Prevalence	294	45

Data processing

In accordance with GBD 2021 principles for data processing, to make hospital inpatient data and claims data comparable, we began by evaluating the number of observations from hospital inpatient data (alternate definition) that matched with a corresponding observation from claims data (reference definition). We matched the observations by age group, location, and when the midpoint of the study was within five years of the midpoint of the reference definition observation. All observations that matched were paired with one another and logit transformed the difference of the logit mean values was calculated. The standard error of the logit difference was calculated using the delta method. To perform the crosswalk, we used a meta-regression—Bayesian, regularised, trimmed (MR-BRT). In this model we trimmed 10% of the data and added a cubic spline on age, assuming linear tails. Our final model results for this crosswalk process are illustrated in the following figure.

MR-BRT crosswalk adjustments factors by age for hospital (alternate) and claims (reference) data.

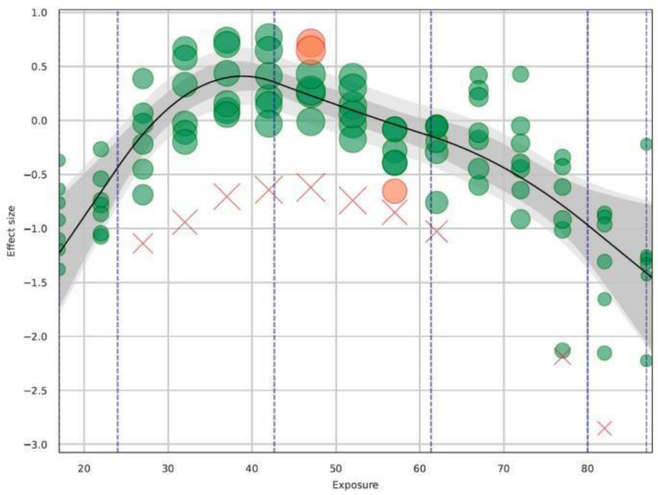

**Exposure on the x-axis is GBD age group and effect size is the logit-transformed difference of inpatient to claims data.*

According to this model, hospital data underestimated the number of menstrual gynaecological disorders for ages 15 to 30 years and after age 55. Between ages 30 and 54 years, the inverse relationship is true.

Modelling strategy

We used DisMod-MR 2.1 to estimate the burden of menstrual disorders. Incidence was set to zero prior to 10 years of age and after 55 years. We assume no excess mortality from menstrual disorders. We set the minimum coefficient of variation to 0.8 and the time span of data used to fit a particular year to 5 years.

In GBD 2021, we evaluated the association between the prevalence of these conditions and potential predictive covariates including the summary exposure values (SEV) for smoking, high body-mass index, sodium intake, alcohol consumption, and low physical activity, along with Socio-demographic Index (SDI), total fertility rate, use of contraception, the prevalence of pelvic inflammatory diseases, and the age-standardised rate of sexually transmitted infections. However, none of the prior mentioned variables, except SDI, were associated with the prevalence of these conditions. From the list of covariates, we included the prevalence of PID and the summary exposure value for high body-mass index as prevalence predictors in the final model, which was selected based on a combination of qualitative and quantitative goodness of fit to input data, plausibility of geographical and temporal trends, and consistency of age pattern.

Table 27: Summary of covariates used in the DisMod-MR meta-regression model for menstrual disorders

Covariate name	Measure	Beta value	Exponentiated value
Pelvic inflammatory disease age-standardised prevalence	Prevalence	0.53 (0.073 to 0.96)	1.70 (1.08 to 2.62)
Age-standardised SEV for high body-mass index	Prevalence	–0.96 (–1 to –0.86)	0.38 (0.37 to 0.42)

Severity splits & disability weights

Anaemia causal attribution analysis used prevalence of menstrual disorders and information on the quantitative effect of menstrual disorders on haemoglobin levels to estimate the proportion of overall anaemia by severity that is due to menstrual disorders. The details of the anaemia analysis are described separately in the "Anaemia impairment" section. Briefly, after estimating total anaemia, a series of counterfactual distributions are generated based on the age- and sex-specific prevalence of each anaemia-causing condition and the quantitative effect that the condition has on haemglobin concentration in the blood, a so-called "haemoglobin shift," that was derived by meta-analysing cohort studies, observational studies, or trials comparing the haemotological status of those with as compared to without the disease. Due to limited data on haemologbin shift, all were assumed to be invariant over age, sex, location, and year.

Table 28: Severity distribution, details on the severity levels for menstrual disorders, and the associated disability weight (DW) with that severity

Severity	Lay description	DW (95% CI)
Anaemia, mild	feels slightly tired and weak at times, but this does not interfere with normal daily activities.	0.004 (0.001–0.008)
Anaemia, moderate	feels moderate fatigue, weakness, and shortness of breath after exercise, making daily activities more difficult.	0.052 (0.034–0.076)
Anaemia, severe	feels very weak, tired, and short of breath, and has problems with activities that require physical effort or deep concentration.	0.149 (0.10–0.21)

References

1 Uterine Fibroids - ACOG. https://www.acog.org/Patients/FAQs/Uterine-Fibroids?IsMobileSet=false (accessed Oct 18, 2019).

2 Divakar H. Asymptomatic uterine fibroids. *Best Pract Res Clin Obstet Gynaecol* 2008; **22**: 643–54.

3 Okolo S. Incidence, aetiology and epidemiology of uterine fibroids. *Best Pract Res Clin Obstet Gynaecol* 2008; **22**: 571–88.

4 Schwartz SM, Marshall LM, Baird DD. Epidemiologic contributions to understanding the etiology of uterine leiomyomata. *Environ Health Perspect* 2000; **108 Suppl 5**: 821–7.

5 Polycystic Ovary Syndrome: ACOG Practice Bulletin, Number 194. *Obstet Gynecol* 2018; **131**: e157.

6 Franks S. Controversy in clinical endocrinology: diagnosis of polycystic ovarian syndrome: in defense of the Rotterdam criteria. *J Clin Endocrinol Metab* 2006; **91**: 786–9.

7 Dunaif A, Givens JR, Haseltine FP, Merriam GR, eds. Polycystic ovary syndrome. Oxford, UK:Blackwell; 59 – 69

8 Rotterdam ESHRE/ASRM-Sponsored PCOS Consensus Workshop Group. Revised 2003 consensus on diagnostic criteria and long-term health risks related to polycystic ovary syndrome. *Fertil Steril* 2004; **81**: 19–25.

9 Azziz R, Carmina E, Dewailly D, *et al.* The Androgen Excess and PCOS Society criteria for the polycystic ovary syndrome: the complete task force report. *Fertil Steril* 2009; **91**: 456–88.

10 Endometriosis - ACOG. https://www.acog.org/Patients/FAQs/Endometriosis?IsMobileSet=false (accessed Oct 21, 2019).

11 Sinaii N, Plumb K, Cotton L, *et al.* Differences in characteristics among 1,000 women with endometriosis based on extent of disease. *Fertil Steril* 2008; **89**: 538–45.

12 Sinaii N, Cleary SD, Ballweg ML, Nieman LK, Stratton P. High rates of autoimmune and endocrine disorders, fibromyalgia, chronic fatigue syndrome and atopic diseases among women with endometriosis: a survey analysis. *Hum Reprod Oxf Engl* 2002; **17**: 2715–24.

13 Loxton D, Dobson A, Byles J, Tooth L. Australian Longitudinal Study on Women's Health (ALSWH). http://www.alswh.org.au/.

14 Committee on Practice Bulletins-Gynecology, American Urogynecologic Society. Practice Bulletin No. 185: Pelvic Organ Prolapse. *Obstet Gynecol* 2017; **130**: e234–50.

15 Slieker-ten Hove MCP, Pool-Goudzwaard AL, Eijkemans MJC, Steegers-Theunissen RPM, Burger CW, Vierhout ME. Symptomatic pelvic organ prolapse and possible risk factors in a general population. *Am J Obstet Gynecol* 2009; **200**: 184.e1-7.

16 Scherf C, Morison L, Fiander A, Ekpo G, Walraven G. Epidemiology of pelvic organ prolapse in rural Gambia, West Africa. *BJOG Int J Obstet Gynaecol* 2002; **109**: 431–6.

17 Lawrence JM, Lukacz ES, Nager CW, Hsu J-WY, Luber KM. Prevalence and co-occurrence of pelvic floor disorders in community-dwelling women. *Obstet Gynecol* 2008; **111**: 678–85.

18 Gomman HM, Nossier SA, Fotohi EM, Kholeif AE. Prevalence and factors associated with genital prolapse: a hospital-based study in Alexandria (Part I). *J Egypt Public Health Assoc* 2001; **76**: 313–35.

19 Chuenchompoonut V, Bunyavejchevin S, Wisawasukmongchol W, Taechakraichana N. Prevalence of genital prolapse in Thai menopausal women (using new standardization classification). *J Med Assoc Thail Chotmaihet Thangphaet* 2005; **88**: 1–4.

20 Townsend MK, Danforth KN, Lifford KL, *et al.* Incidence and remission of urinary incontinence in middle-aged women. *Am J Obstet Gynecol* 2007; **197**: 167.e1-5.

21 Nisar N, Zehra N, Haider G, Munir AA, Sohoo NA. Frequency, intensity and impact of premenstrual syndrome in medical students. *J Coll Physicians Surg--Pak JCPSP* 2008; **18**: 481–4.

22 Tabassum S, Afridi B, Aman Z, Tabassum W, Durrani R. Premenstrual syndrome: frequency and severity in young college girls. *JPMA J Pak Med Assoc* 2005; **55**: 546–9.

23 Steiner M, Macdougall M, Brown E. The premenstrual symptoms screening tool (PSST) for clinicians. *Arch Womens Ment Health* 2003; **6**: 203–9.

24 Choi D, Lee D-Y, Lehert P, Lee IS, Kim SH, Dennerstein L. The impact of premenstrual symptoms on activities of daily life in Korean women. *J Psychosom Obstet Gynaecol* 2010; **31**: 10–5.

25 Deuster PA, Adera T, South-Paul J. Biological, social, and behavioral factors associated with premenstrual syndrome. *Arch Fam Med* 1999; **8**: 122–8.

Haemoglobinopathies and haemolytic anaemias

This document describes the non-fatal disease burden modelling process for GBD 2021 for each of sickle cell disorders, thalassaemias, glucose-6-phosphate dehydrogenase (G6PD) deficiency, sickle cell trait, thalassaemia trait, hemizygous G6PD deficiency, and other haemoglobinopathies and haemolytic anaemias.

Flowchart

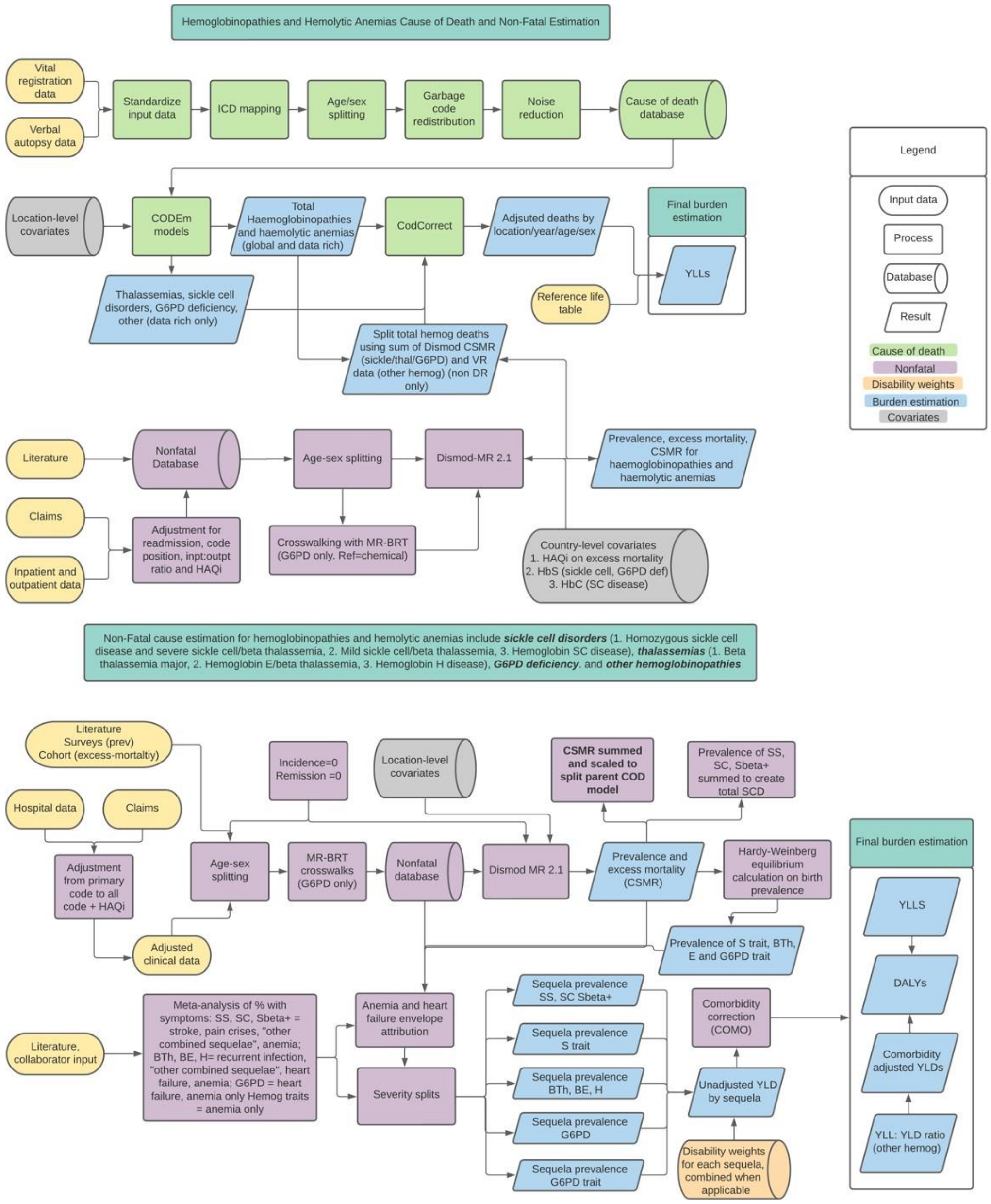

Case definition and overview

Haemoglobinopathies and haemolytic anaemias span four GBD causes: thalassaemias, sickle cell disorders, G6PD deficiency, and other haemoglobinopathies and haemolytic anaemias. Case definitions for each ofthe types of thalassaemias and sickle cell were based on genotype. G6PD deficiency is an X-linked recessive genetic disease, and our reference definition was based on quantitative decline in G6PD activity during reagent (ie, chemical) testing; genotype or other testing was an acceptable alternate definition and adjusted as described below. Sickle cell trait, thalassaemia trait, and hemizygous G6PD deficiency were all similarly defined by genotype. They were estimated from the component disease models' estimates of birth prevalence assuming Hardy-Weinberg equilibrium. YLDs due to other haemoglobinopathies and haemolytic anaemias were estimated assuming the YLD-to-YLL ratio foreach

age, sex, location, and year was similar to that of the aggregate of sickle cell, thalassaemias, and G6PD deficiency. The primary conditions in this group are aplastic anaemias.

Several unique combinations of genetic mutations lead to distinct phenotypes with different natural history, which has led us to estimate several distinct subtypes of thalassaemias and sickle cell disorders. The three thalassaemia models included 1) beta-thalassaemia major, 2) haemoglobin E/beta-thalassaemia, and 3) haemoglobin H disease (genotype = - - / - alpha). Sickle cell models included 1) homozygous sickle cell and severe sickle cell/beta-thalassaemia where the latter genotype had either a severe version of the sickle gene (assumed to always be the case if unspecified and west of the Arabian peninsula) or a nonsense (as opposed to reduced activity) mutation at the other beta haemoglobin gene locus; 2) haemoglobin sickle cell disease; and 3) "mild" sickle cell-beta-thalassaemia. G6PD deficiency was estimated in a single model.

Input data

Three sources of data were used for DisMod-MR 2.1 models: literature (generally from community prevalence surveys, birth screening, and cohort studies), claims data, and ICD-9 & ICD-10 hospital discharge data that were adjusted for ICD code position, readmission, inpatient-to-outpatient ratio, and location-specific Healthcare Access and Quality (HAQ) Index. We added data from select geographies identified by GBD Collaborators for GBD 2021. Of note, there were no hospital data available for haemoglobin E/beta-thalassaemia, haemoglobin H disease, or G6PD deficiency. Our last comprehensive literature review was completed in GBD 2017, where we identified data on prevalence, excess mortality rate, or with-condition mortality rate. Age-specific survival probabilities from cohort studies were converted to corresponding with-condition mortality rates.

A systematic literature review was last completed for GBD 2016 using the following search strings in PubMed:

> (G6PD[Title/Abstract] OR G6PD deficiency[Title/Abstract] OR glucose-6 phosphate dehydrogenase[Title/Abstract] OR glucose-6-phosphate dehydrogenase deficiency[Title/Abstract] AND (survival[Title/Abstract] OR mortality[Title/Abstract] OR prevalence[Title/Abstract] OR incidence[Title/Abstract]) AND (2013/01/01[PDat] : 2016/12/31[PDat])) AND "humans"[MeSH Terms]
>
> (sickle cell[Title/Abstract] AND (mortality[Title/Abstract] OR survival[Title/Abstract] OR prevalence[Title/Abstract] OR incidence[Title/Abstract]) AND (2013/04/01[PDat] : 2016/12/31[PDat])) AND "humans"[MeSH Terms]
>
> (thalassemias [Title/Abstract] AND (prevalence[Title/Abstract] OR incidence[Title/Abstract] OR survival[Title/Abstract] OR mortality[Title/Abstract]) AND (2013/01/01[PDat] : 2016/12/31[PDat])) AND "humans"[MeSH Terms]

Inclusion criteria were community or facility-based surveys of prevalence of condition where either genetic testing was completed or the search was completed on July 5, 2016, and supplemented similar searches that were completed for GBD 2010 and GBD 2013. The G6PD deficiency search yielded 120 results, of which 57 were selected for full text review and 32 were extracted. The sickle cell search yielded 488 results, of which 49 were selected for full text review and 22 were extracted. The thalassaemias search yielded 27 results, 10 had full text review, and four were extracted.

We extracted prevalence data from population-level and community surveys as well as with-condition mortality and excess mortality data from cohort studies. Age-specific survival proportions were converted to with-condition mortality rates as needed. We also included data from hospital and claims data for a subset of haemoglobinopathy models, including beta-thalassaemia major, haemoglobin E/beta-thalassaemia, homozygous sickle cell and severe sickle cell/beta-thalassaemia, haemoglobin SC disease, and mild sickle cell/beta-thalassaemia.

Processing of clinical administrative data (ie, hospital and claims) were based on ICD-9 and ICD-10 codes as listed in Table 1. The extraction and processing of hospital and claims data is described separately.

Figure 1. PRISMA diagram of GBD 2016 literature review

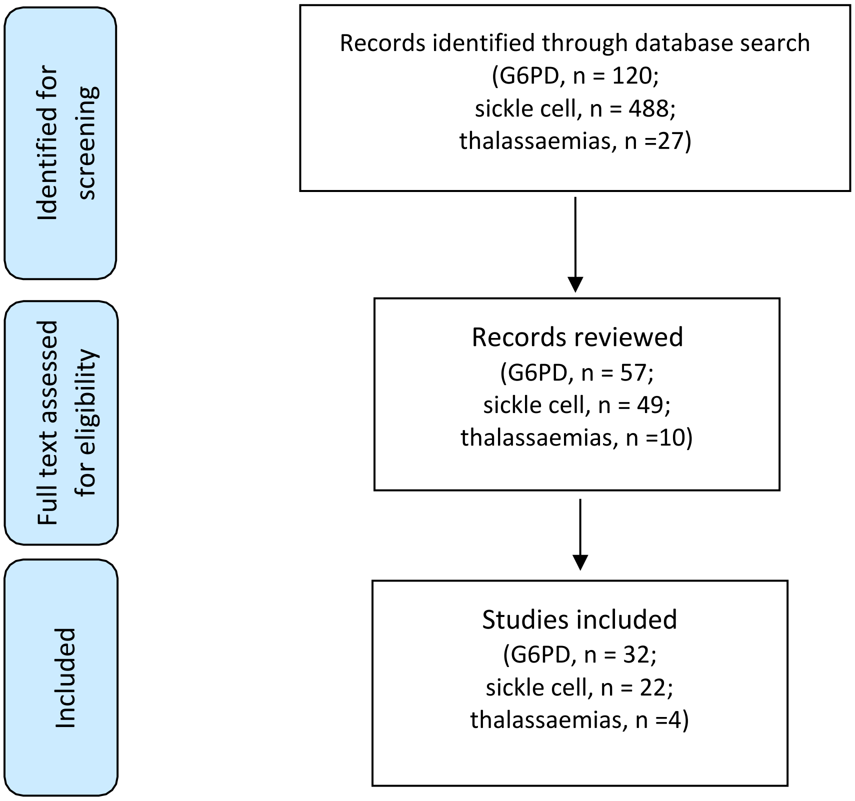

Table 1. Data inputs for modelling prevalence of haemoglobinopathies and haemolytic anaemias

Condition	New sources	Total sources	Countries with data
Haemoglobinopathies and haemolytic anaemias (all measures)	94	790	138
Prevalence	66	752	138
Excess mortality rate	20	25	13
With-condition mortality rate	11	16	11
Other	0	9	6
Thalassaemias (all measures)	69	354	94
Prevalence	63	348	94
Excess mortality rate	5	6	6
With-condition mortality rate	1	1	1
Sickle cell disorders (all measures)	88	556	117
Prevalence	66	533	117
Excess mortality rate	15	19	9
With-condition mortality rate	10	15	10
G6PD deficiency (all measures)	0	185	69
Prevalence	0	176	68
Other	0	9	6

Table 2. International classification of diseases codes for haemoglobinopathies and haemolytic anaemias in GBD 2021 cause of death analysis

Condition	ICD-10 code	ICD-9 code
Thalassaemias	D56	282.4
Sickle cell disorders	D57	282.5–282.6
G6PD deficiency	D55	282.2–282.3
Other haemoglobinopathies and haemolytic anaemias	D58–D64.8	282.0–282.1, 282.7–285.8

Data processing

Data processing strategies did not change from GBD 2019 such that we conducted age-sex splitting and crosswalking in the same methods detailed as follows; however, we did update processes to account for GBD 2021 age and location hierarchies.

The first step of the process was age-sex splitting. For any datum that did not entirely fit within a GBD age group or was for both sexes combined, the observation was split to be multiple age-sex-specific datapoints based on the age and sex pattern predicted by GBD 2019 DisMod-MR 2.1 models. It is our intention to update this age-sex splitting with each cycle of GBD. For thalassaemias and sickle cell disorders, this was the only processing completed.

For G6PD deficiency, we crosswalked all data to the reference definition of chemical test. In accordance with GBD 2021 principles for data processing, we began by evaluating the number of observations of each alternate definition that matched with a corresponding observation from the reference definition. A match was considered "within" study if it was from the same data source and an exact match for age, sex, location, and year. A match was considered "between" study if it was from the same GBD location, GBD age group, sex, and the midpoint of the study was within five years of the midpoint of the reference definition observation. Because the prevalence of G6PD deficiency itself can vary between studies, and the difference between reagent and chemical testing is expected to be a largely constant phenomenon, we restricted the crosswalk only to be based on within-study matches. There were no matches for diagnostics that were not based on either genetic or reagent testing. All of these data were therefore dropped from the model. The total number of datapoints and matches is shown in the table below.

Table 3. Datapoints and matches between alternate and reference definitions

	Reference (cv_dx_chemical)	Alternate #1 (cv_dx_genetic)	Alternate #2 (cv_dx_other)
Number of datapoints	6370	2578	9
Within-study matches to reference	--	397	0

The ratio of prevalence from alternate:reference was calculated, log-transformed, the standard error of the ratio calculated using the delta method, and all were analysed using meta-regression—Bayesian, regularised, trimmed (MR-BRT) a meta-regression tool developed for GBD 2019. We tested the relationships as a function of sex, age, and the variability as a function of location (grouped into super-regions). Only sex remained a significant predictor, so it was the only additional factor included in the

final crosswalk model. We trimmed 10% of the data from the MR-BRT model. Our covariate betas for each of the included covariates in the model are summarised in the table below.

Table 4. MR-BRT crosswalk adjustment factors for haemoglobinopathies and haemolytic anaemias

Data input	Reference or alternative case definition	Gamma	Beta coefficient, log (95% CI)	Adjustment factor*
Chemical test	Reference	0.06	---	---
Genetic test	Alternative		0.291 (–0.175 to 0.755)	1.33 (0.84–2.13)
Sex	Alternative		–0.027	

**MR-BRT crosswalk adjustments can be interpreted as the factor the alternative case definition is adjusted by to reflect what it would have been had it been measured using the reference case definition. If the log beta coefficient is negative, then the alternative is adjusted up to the reference. If the log beta coefficient is positive, then the alternative is adjusted down to the reference. The adjustment factor column is the exponentiated beta coefficient and can be interpreted as the relative rate between the two case definitions.*

Modelling strategy

Substantive changes were not made since GBD 2019 besides the addition of summing the three sickle cell prevalence sub-causes to create a fourth, total sickle cell disorders model. Covariates for the prevalence of haemoglobin S (HbS) and haemoglobin C (HbC) to the sickle cell and G6PD deficiency models bS and HbC rasters were summarised into GBD geographies from Malaria Atlas Project publications on them and assumed to be invariant over time and age. We estimated the non-fatal burden of haemoglobinopathies in four parts.

1. DisMod-MR 2.1 modelling of disease

First, we used the datasets described above to estimate prevalence for each age-sex-location-year in the GBD 2019 location hierarchy using DisMod-MR 2.1. Natural-log-transformed lag-distributed income per capita was used as a covariate on excess mortality for most models. HbS and HbC were used for each of the subtypes of sickle cell disorders and for G6PD deficiency, where the effect size and predictive power were expectedly much smaller. HAQ Index was also used as a covariate for excess mortality rate in the homozygous sickle cell and severe sickle cell/beta-thalassaemia model. A full table of all the location-level covariates and their effect sizes are shown below.

In consultation with GBD researchers and collaborators, final models were selected on a combination of qualitative and quantitative goodness of fit to input data, plausibility of geographical and temporal trends, consistency of age pattern, and, when available, comparison with other published studies on haemoglobinopathy epidemiology. Directionality, magnitude, and plausibility of study-level and country-level covariates was also considered in the process of model development. Of note, due to the nature of statistical modelling, final results do not always cover the values reported in input data.

Table 5. Covariate, parameter, beta, and exponentiated beta values for each model

**Model**	**Covariate**	**Parameter**	**Beta**	**Exponentiated beta**
Beta-thalassaemia major	UHC	EMR	–0.026 (–0.047 to –0.0037)	0.97 (0.95–1.00)

Haemoglobin E/beta-thlassaemia	UHC	EMR	–0.025 (–0.05 to –0.0024)	0.98 (0.95–1.00)
Haemoglobin E/beta-thlassaemia	Year	Prev	0.020 (0.018 to 0.020)	1.02 (1.02 to 1.02)
Haemoglobin H disease	Year	Prev	–0.018 (–0.019 to –0.017)	0.98 (0.98 to 0.98)
Homozygous sickle cell and severe sickle cell/beta-thal	(HbS)^2	Prev	49.94 (49.90 to 50.00)	5.02e+21 (4.69e+21 to 5.18e+21)
Homozygous sickle cell and severe sickle cell/beta-thal	UHC	EMR	–0.028 (–0.048 to –0.0036)	0.97 (0.95 to 1.00)
Haemoglobin SC disease	HbS	Prev	19.99 (19.98 to 20.00)	4.82e+8 (4.76e+8 to 4.85e+8)
Haemoglobin SC disease	HbC	Prev	10.00 (9.99 to 10.00)	2.19e+4 (2.18e+4 to 2.20e+4)
Haemoglobin SC disease	UHC	EMR	–0.024 (–0.046 to –0.0038)	0.98 (0.96 to 1.00)
Mild sickle cell/beta-thalassaemia	HbS	Prev	19.99 (19.97 to 20.00)	4.80e+8 (4.71e+8 to 4.85e+8)
Mild sickle cell/beta-thalassaemia	UHC	EMR	–0.025 (–0.048 to –0.0029)	0.98 (0.95 to 1.00)
G6PD deficiency	|Latitude|	Prev	–0.003 (–0.0045 to –0.0016)	1.00 (1.00 to 1.00)
G6PD deficiency	HbC	Prev	0.068 (0.0031 to 0.17)	1.07 (1.00 to 1.19)
G6PD deficiency	HbS	Prev	0.12 (0.0043 to 0.40)	1.13 (1.00 to 1.50)

Abbreviations: UHC=universal health coverage. EMR=excess mortality rate. Prev=prevalence. HbS=haemoglobin S trait prevalence. HbC=haemoglobin C trait prevalence.

2. Hardy-Weinberg equilibrium to estimate carrier prevalence

Second, we calculated prevalence of haemoglobinopathy traits (sickle cell trait, haemoglobin E trait, haemoglobin beta trait, hemizygous G6PD) by back-calculating from birth prevalence estimates from corresponding DisMod-MR 2.1 models, assuming Hardy-Weinberg equilibrium and no excess mortality. Because G6PD deficiency is an X-linked disease, hemizygous G6PD can only occur in females.

3. Severity distributions and sequelae of disease

With the exception of anaemia, only homozygous individuals were considered to experience disability. Estimated sequelae of thalassaemias included anaemia (described separately), heart failure (described separately), and periodic severe infection. Another series of common, but not universal, sequelae also occur in those with thalassaemias, including splenomegaly, skeletal deformity, delayed growth/puberty, diabetes, hypothyroidism, and leg ulcers. Given sparse data on the occurrence of these sequelae, they were approximated with a health state named "other combined sequelae of thalassaemia," for which we used the disability weight corresponding to a health state of "generic uncomplicated disease, anxiety about diagnosis and daily medication" which, of note, was also used to approximate the disability for those with cancer in remission. For sickle cell disorders, we similarly estimated YLDs for anaemia (described separately), stroke, and pain crises separately and approximated the myriad additional complications of sickle cell disease with the health state "other combined sequelae of sickle cell

disease.” The only sequelae estimated for G6PD deficiency were anaemia (described separately) and heart failure (described separately). Notably, however, G6PD deficiency is considered to be asymptomatic for a vast majority of those with the condition, with only a very small subset of around 1 in 1 million having chronic haemolysis (Class I disease) and approximately 1% having periodic haemolytic episodes (Class II disease) with exposure to environmental, pharmaceutical, or food products. Females heterozygous for G6PD deficiency exhibit chimerism, as one X chromosome becomes dominant in each of the red blood cells, so we estimated half as many heterozygous females will be symptomatic as homozygous females. Table 6 has all the disabling health states that were included in calculation of YLDs for haemoglobinopathies and haemolytic anaemias.

4. Anaemia causal attribution

The age- and sex-specific anaemia prevalence for each of the haemoglobinopathies, as well as the estimates of anaemia due to carrier/trait state, were analysed as part of overall anaemia causal attribution for GBD 2021. The details of the anaemia analysis are described separately in the “Anaemia Impairment” section. Briefly, after estimating total anaemia, a series of counterfactual distributions are generated based on the prevalence of each anaemia-causing condition and the quantitative effect that the condition has on haemoglobin concentration in the blood, a so-called “haemoglobin shift,” that was derived by meta-analysing cohort studies, observational studies, or trials comparing the haemotological status of those with as compared to without the disease. Due to limited data on haemologbin shift, all were assumed to be invariant over age, sex, location, and year.

5. YLL:YLD ratio for other haemoglobinopathies and haemolytic anaemias

Finally, we found the YLD-to-YLL ratio for all haemoglobinopathies and then applied it to YLLs estimated for other haemoglobinopathies and haemolytic anaemias in our cause-specific mortality analysis. Quantitative crosswalk results for each model are shown below.

Table 6. Health states for haemoglobinopathies and haemolytic anaemias

Severity level	Lay description	DW (95% CI)	Cause
Mild anaemia	Feels slightly tired and weak at times, but this does not interfere with normal daily activities.	0.004 (0.001–0.008)	All
Moderate anaemia	Feels moderate fatigue, weakness, and shortness of breath after exercise, making daily activities more difficult.	0.052 (0.034–0.076)	All
Severe anaemia	Feels very weak, tired, and short of breath, and has problems with activities that require physical effort or deep concentration.	0.149 (0.101–0.209)	All
Severe abdominopelvic problem (proxy for vaso-occlusive crisis)	Has severe pain in the belly and feels nauseous. The person is anxious and unable to carry out daily activities.	0.324 (0.22–0.442)	Sickle cell disorders
Stroke, long-term consequences, moderate plus cognition problems	Has some difficulty in moving around, in using the hands for lifting and holding things, dressing and grooming, and in speaking. The person is often forgetful and confused.	0.316 (0.206–0.437)	Sickle cell disorders
Combined sequelae of disease (approximation of all other sequelae)	Has a chronic disease that requires medication every day and causes some worry but minimal interference with daily activities.	0.049 (0.031–0.072)	Sickle cell disorders, thalassaemias
Medically managed heart failure	--		Thalassaemias
Mild heart failure	Is short of breath and easily tires with moderate physical activity, such as walking uphill or more than a quarter-mile on level ground. The person feels comfortable at rest or during activities requiring less effort.	0.041 (0.026–0.062)	Thalassaemias
Moderate heart failure	Is short of breath and easily tires with minimal physical activity, such as walking only a short distance. The person feels comfortable at rest but avoids moderate activity.	0.072 (0.047–0.103)	Thalassaemias
Severe heart failure	Is short of breath and feels tired when at rest. The person avoids any physical activity for fear of worsening the breathing problems.	0.179 (0.122–0.251)	Thalassaemias
Severe infection	Has a high fever and pain, and feels very weak, which causes great difficulty with daily activities.	0.133 (0.088–0.19)	Thalassaemias

Headaches

Flowchart

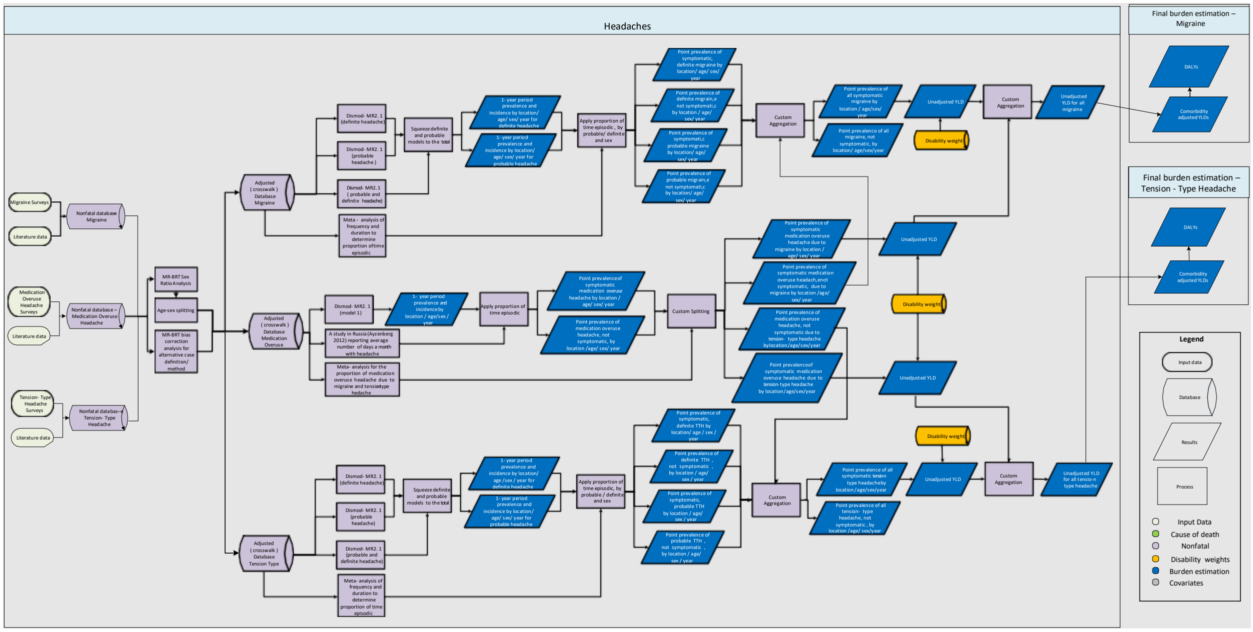

Input Data and Methodological Summary for Headaches

Case definition

Migraine

Migraine is a disabling primary headache disorder, typically characterised by recurrent moderate or severe unilateral pulsatile headaches. The two major types are migraine without aura and migraine with aura (transient neurological symptoms). In GBD, we do not distinguish between migraine with and without aura as most epidemiological studies report on overall migraine only. The reference diagnostic criteria for migraine are from the International Classification of Headache Disorders (ICHD)-3, which describes five criteria:

1. At least five attacks fulfilling criteria 2–5
2. Headache attacks lasting 4–72 hours (untreated or unsuccessfully treated)
3. Headache has at least two of the following four characteristics:
    a. Unilateral location
    b. Pulsating quality
    c. Moderate or severe pain intensity
    d. Aggravation by or causing avoidance of routine physical activity
4. During headache at least one of the following:
    a. Nausea and/or vomiting
    b. Photophobia and phonophobia
5. Not better accounted for by another ICHD-3 diagnosis

Definite migraine is a headache that satisfies all the criteria outlined above, while probable migraine satisfies all of the above criteria except one. Studies that have looked at the reasons for cases with probable headache not fulfilling criteria for definite diagnosis have suggested that most often it is the duration criterion that is left unfilled.[1, 2, 3, 4, 5] Before GBD 2017 we did not distinguish between probable and definite migraine. Since GBD 2017 we accounted for the varying case definitions used by different sources.

Tension-type headache

Tension-type headache (TTH) is characterised by a dull, non-pulsatile, diffuse, band-like (or vice-like) pain of mild to moderate intensity in the head or neck. The reference diagnostic criteria for tension-type headache are from the ICHD-3, which describes five criteria:

1. At least 10 attacks fulfilling criteria 2–5
2. Lasing from 30 minutes to 7 days
3. At least two of the following four characteristics:
    a. Bilateral location
    b. Pressing or tightening (non-pulsating) quality
    c. Mild or moderate intensity
    d. Not aggravated by routine physical activity such as walking or climbing stairs
4. Both of the following:
    a. No nausea or vomiting
    b. No more than one of photophobia or phonophobia
5. Not better accounted for by another ICHD-3 diagnosis

Definite tension-type headache is a headache that satisfies all criteria outlined above, while probable tension-type headache satisfies all of the above criteria except one. Before GBD 2017 we did not distinguish between probable and definite tension-type headache. Since GBD 2017 we have accounted for varying case definitions used by different sources.

Medication overuse headache

Both migraine and tension-type headache can give rise to medication overuse headache (MOH), with the following International Classification of Headache Disorders (ICHD-3) diagnostic criteria:

1. Headache occurring ≥15 days/month in a patient with a pre-existing headache disorder
2. Regular overuse for >3 months of one or more drugs that can be taken for acute and/or symptomatic treatment of headache
3. Not better accounted for by another ICHD-3 diagnosis.

ICHD-3 explicitly states that, when a person fulfils criteria for both migraine and MOH, both diagnoses should be given. However, our GBD headache collaborators, Steiner and Stovner, indicated that in survey practice, a screening question on chronic headache is used first, followed by questions to determine if medication overuse is present. This means the diagnoses of migraine and MOH become mutually exclusive (obviating any potential problem of double-counting).

Input data

Migraine

We last conducted a systematic review of migraine for GBD 2017, which covered papers published through September 2017. The search string for this review was (((((("migraine disorders"[MeSH Terms] OR migraine[All Fields]) AND ((prevalence[Title/Abstract] OR incidence[Title/Abstract] OR remission[Title/Abstract] OR epidemiology[Title/Abstract]))))))).

Inclusion criteria of the systematic reviews were:

- Representative, population-based surveys
- Reporting of prevalence of migraine headache

In GBD 2017 we decided to exclude medical claims data as the adjustment needed to make the claims data comparable to population representative surveys was unstable.

Tension-type headache

We last conducted a systematic review of TTH for GBD 2017, which covered papers published through September 2017. The search string for this review was ((((("headache"[MeSH Terms]) OR ("headache"[Title/Abstract] AND "tension"[Title/Abstract])) AND ("epidemiology"[Title/Abstract] OR "prevalence"[Title/Abstract] OR "incidence"[Title/Abstract] OR "remission"[Title/Abstract])))).

Inclusion criteria of the systematic reviews were:

- Representative, population-based surveys
- Reporting of prevalence of TTH headache

In GDB 2017 we decided to exclude medical claims data, as the adjustment needed to make the claims data comparable to population representative surveys was unstable.

Medication overuse headache

We last conducted a systematic review of MOH for GBD 2017, which covered papers published through September 2017. The search string for this review was (("headache"[MeSH Terms] OR "headache"[Title/Abstract]) AND ("pharmaceutical preparations"[MeSH Terms] OR "pharmaceutical preparations"[Title/Abstract] OR "medication"[Title/Abstract]) AND ("epidemiology"[Title/Abstract] OR "prevalence"[Title/Abstract] OR "incidence"[Title/Abstract] OR "remission"[Title/Abstract])).

Inclusion criteria of the systematic reviews were:

- Representative, population-based surveys
- Reporting of prevalence of MOH headache

Table 1: Data inputs

Cause Name	Measure	Total sources	Countries with data
Migraine	All measures	144	50
Migraine	Prevalence	135	49
Migraine	Incidence	4	4
Migraine	Remission	7	5
Tension-type headache	All measures	93	39
Tension-type headache	Prevalence	87	38
Tension-type headache	Incidence	0	0
Tension-type headache	Remission	7	4
Headache disorders	All measures	151	50
Headache disorders	Prevalence	142	49
Headache disorders	Incidence	4	4
Headache disorders	Remission	7	5

Note: Sources for medication-overuse headaches are accounted for within migraine, tension-type headache and overall headache disorder source counts

Age and sex splitting

Reported estimates of prevalence were split by age and sex where possible.

First, if studies reported prevalence for broad age groups by sex (eg, prevalence in 15- to 65-year-old males and females separately), and also by specific age groups but for both sexes combined (eg, prevalence in 15- to 30-year-olds, then in 31- to 65-year-olds, for males and females combined), age-specific estimates were split by sex using the reported sex ratio and bounds of uncertainty.

Second, prevalence data for both sexes that could not be split using a within-study ratio were split using a sex ratio derived from a meta-analysis of existing sex-specific data using a MR-BRT (meta-regression—Bayesian, regularised, trimmed) model[1] (Additional information can be found in appendix 1, section 4.4.1 of the cited paper). The female to male ratio was 1.90 (1.85 to 1.96).

Finally, after the application of bias adjustments, if studies reported estimates across age groups spanning 25 years or more, these were split into five-year age groups using the prevalence age pattern estimated by the best DisMod-MR 2.1[6] (disease model—Bayesian meta-regression, details on this method can be found in appendix 1, section 4.5 of the citation) for each headache type from GBD 2019.

Data adjustment (Bias adjustments)

We used a list of binary adjustment criteria which are a modified version of quality indicators of epidemiological studies on headache (Steiner TJ, Stovner LJ et al [2013]. Improving quality in population surveys of headache prevalence, burden, and cost: key methodological considerations. J Headache Pain, 14: 87) and shown in the table below.

Table 2: Study covariates

Study covariate	Notation	
	Less desirable (1)	Reference (zero)
Other than one-year recall period	Point prevalence	One-year prevalence
Not representative	Selected population	General population or community-based sample from whole country OR general population or community-based sample from defined region within a country, or school-based (for children)
Low-quality sampling method	Not stated OR no (or failed) attempt to secure representativeness	Total defined population, or random sample corrected for population demographics OR random sample uncorrected for population demographics
Poor response	Not stated, or <70%	70–100%
Low-quality survey method and type of interviewer	Not stated OR self-administered (unsupervised) questionnaire OR telephone or face-to-face interview by untrained or unspecified interviewer(s)	Face-to-face interview with headache expert or trained interviewer

Low-quality validation of diagnostic instrument	Instrument not specified or not validated OR validated, but sensitivity and/or specificity <70% OR validated only in screen-positive sub-sample, or in clinic or unspecified sample, but sensitivity and specificity ^{3}70%	Validated in target population or similar, and sensitivity and specificity ^{3}70%, or all diagnoses made in face-to-face or telephone interviews by headache expert
Low-quality diagnostic criteria	Not stated OR stated, other than ICHD OR ICHD (or reasonable modification)	ICHD (or reasonable modification)
Headache type assumed	Probable/definite headache has been assumed based on descriptions and not stated explicitly	Didn't have to assume headache type

Studies based on lifetime recall of headaches were not included because of the concern of significant recall bias. For migraine and tension-type headache, **we additionally tagged studies where the type of headache (probable/definite) was not explicitly mentioned in the report but the type was determined based on the diagnostic criteria stated to the best of our understanding.** This covariate is called Headache type assumed.

The mean and standard error for the coefficients were calculated using the MR-BRT adjustment method. All study covariates were initially evaluated independently for each of the three types of headache. However, covariate values varied not only in magnitude but in direction across the three headache types. **Because we assume that the same study covariate should adjust data at least in the same direction for all headache types, the final study covariates were evaluated taking all migraine, tension-type, and medication overuse headache data into account**. Studies conducted in a school setting were retained in the models but were no longer adjusted in this round of the GBD, as we were unable to find matches to inform a reliable crosswalk. These studies were not excluded because the headache models are relatively data sparse. Betas and inverse-logit values for these covariates are shown in the table below:

Table 3: MR-BRT Crosswalk Adjustment Factors for Headaches

Data input	Reference or alternative case definition	Gamma	Beta Coefficient, Logit (95% UI)*	Adjustment factor**
Other than one-year recall	Alt	1.20	-0.89 (-0.97 to -0.80)	0.30 (0.28 to 0.31)
Not representative	Alt		-0.39 (-0.45 to -0.33)	0.40 (0.39 to 0.42)
Low-quality sampling method	Alt		0.73 (0.66 to 0.79)	0.67 (0.66 to 0.69)
Poor response	Alt		-0.45 (-0.53 to -0.36)	0.40 (0.37 to 0.41)
Low-quality survey method	Alt		-0.22 (-0.31 to -0.13)	0.45 (0.42 to 0.47)
Low-quality diagnostic instrument	Alt		0.15 (0.13 to 0.19)	0.54 (0.53 to 0.55)

Low-quality diagnostic criteria	Alt		-0.37 (-0.43 to -0.32)	0.41 (0.39 to 0.42)
Headache type assumed	Alt		0.37 (0.33 to 0.42)	0.59 (0.58 to 0.60)

**MR-BRT crosswalk adjustments can be interpreted as the factor the alternative case definition is adjusted by to reflect what it would have been had it been measured using the reference case definition. If the log/logit beta coefficient is negative, then the alternative is adjusted up to the reference. If the log/logit beta coefficient is positive, then the alternative is adjusted down to the reference.*

**The adjustment factor column is the exponentiated beta coefficient. For log beta coefficients, this is the relative rate between the two case definitions. For logit beta coefficients, this is the relative odds between the two case definitions.

Modelling strategy

As in GBD 2019, standard DisMod-MR settings across all headache models included setting excess mortality to 0, and assuming that there was no incidence or prevalence before the age of 5 years.

Migraine

We made no substantive changes in the modelling strategy of migraine from GBD 2019. As in the last round, we ran separate DisMod-MR models for definite migraine, probable migraine, and the total migraine category and set an upper bound on remission of 0.1 across all models. After running the separate models, we then scaled the results of probable and definite headache to the total headache envelope to ensure consistency.

Because some data sources, especially earlier data from before ICHD became the standard (the initial criteria were published in 1988), largely report on definite migraine, we also adjusted studies that reported only on definite migraine to the total migraine category in order to better inform that model. All data that reported on both definite and total migraine were used in regression models by sex in order to derive an age- and sex-specific adjustment. The adjustment is shown in the graphs below.

Male Female

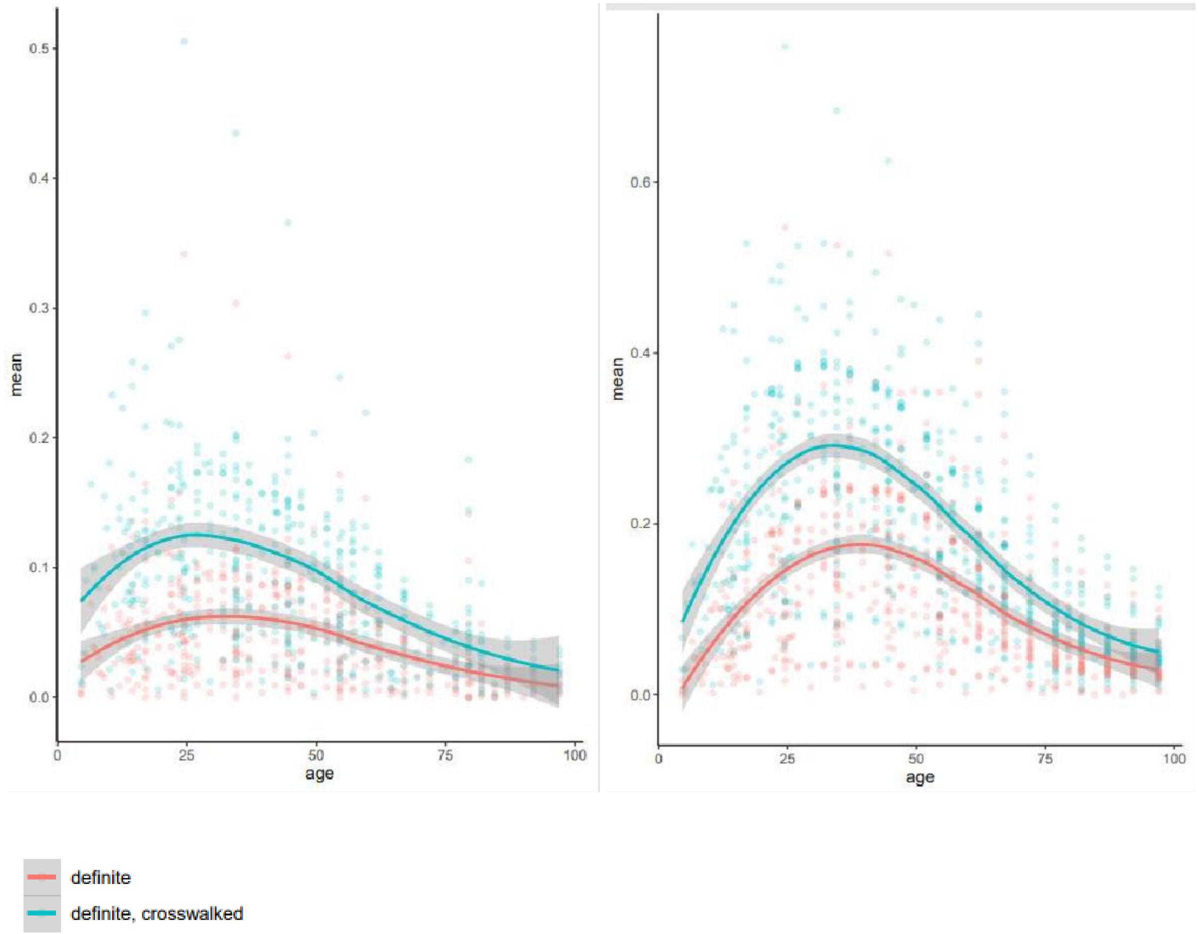

For GBD 2021, same as in 2019, we used multi-country survey unit-record data from 19 countries in the Lift the Burden survey series provided by our collaborators on the time symptomatic of various headache types. This source provided greater granularity of time symptomatic data, as we had used summary measures from survey reports instead of microdata in the past. This source also provided data on probable, definite, and total migraine, eliminating the need to back calculate time symptomatic for probable migraine. Using the MR-BRT regression method, we calculated the proportion of time symptomatic is 0.093 for definite migraine and 0.066 for probable migraine.

Tension-type headache

For this round of the GBD, like in 2019, we replicated the modelling process for migraine headache and ran separate DisMod-MR models for definite TTH, probable TTH, and the total TTH category, setting an upper bound on remission of 0.5 across all models. After running the separate models, we then scaled the results of probable and definite headache to the total headache envelope to ensure consistency.

Because some data sources, especially earlier data from before ICHD became the standard (the initial criteria were published in 1988), largely report on definite TTH, we also adjusted studies that reported only on definite TTH to the total TTH category in order to better inform that model. Initially, all data that reported on both definite and total TTH were used in regression models by sex in order to derive an age- and sex-specific adjustment. These sex-specific models resulted in an implausible age pattern for females

such that the age pattern of the age-split datapoints was the inverse of the original data. Consequently, we ran a regression model to derive an age-specific adjustment that was applied to both sexes. The adjustment is shown in the graphs below.

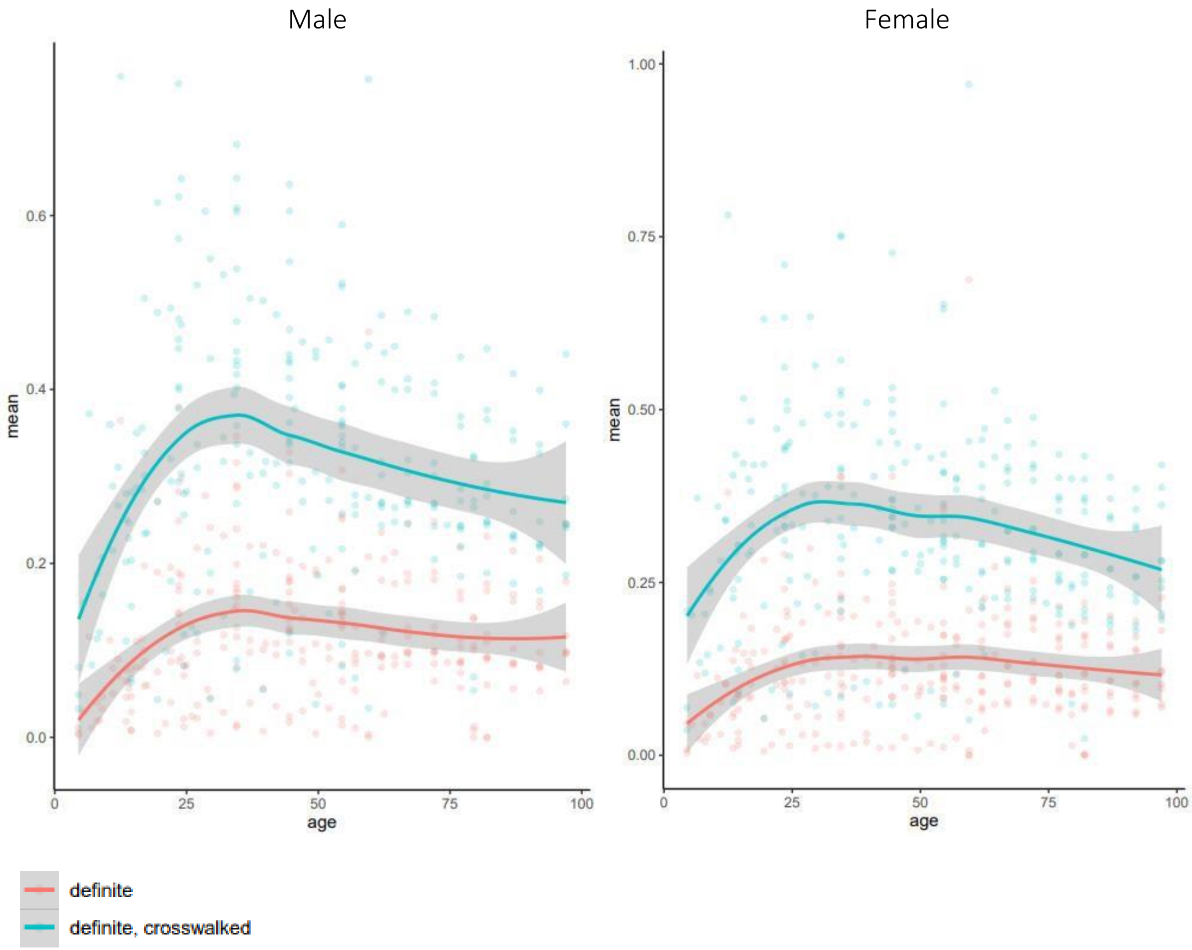

For GBD 2021 we used the results from the same meta-analysis of Lift the Burden unit-record data on the time symptomatic of headache, which also reported estimates for probable, definite, and total TTH. Using MR-BRT, we calculated the proportion of time symptomatic is 0.029 for definite TTH and 0.021 for probable TTH.

Medication overuse headache

Prior settings in the DisMod-MR model included an upper bound on remission of 0.4. In GDB 2017, to determine the proportion of time over a year spent with medication overuse headache, we meta-analysed the two available studies on frequency and used the one available study on duration. The result of the meta-analysis on frequency gave an estimate of 250.83 attacks per year, and the available source on duration estimated an average duration of 18.59 hours. From this data we estimated that the proportion of time symptomatic for medication overuse headache was 0.532. We made no substantive changes in the modelling strategy since GBD 2017.

Medication overuse headache split

As medication overuse headache can develop from migraine or tension-type headache, we split medication overuse into sequelae of both primary headache disorders. Based on a 2017 meta-analysis of three sources, 73.2% (63.7–81.0) of medication overuse headache is assigned to medication overuse headache due to migraine. The forest plot is shown below.

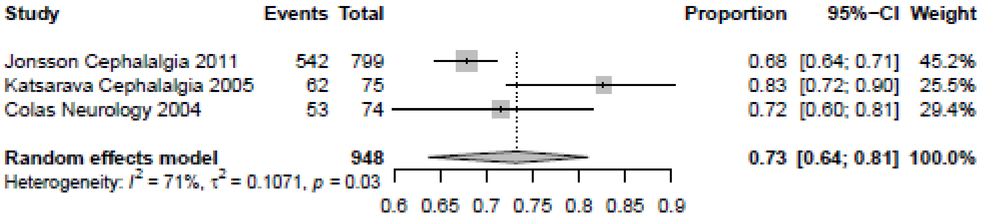

References

[1] Kim B-K, Chung YK, Kim J-M, Lee K-S, Chu MK. Prevalence, clinical characteristics and disability of migraine and probable migraine: A nationwide population-based survey in Korea. *Cephalalgia* 2013; **33**: 1106–16.

[2] Lantéri-Minet M, Valade D, Géraud G, Chautard M, Lucas C. Migraine and probable migraine – results of FRAMIG 3, a French nationwide survey carried out according to the 2004 IHS classification. *Cephalalgia*; **25**: 1146–58.

[3] Pfaffenrath V, Fendrich K, Vennemann M, *et al.* Regional variations in the prevalence of migraine and tension-type headache applying the new IHS criteria: the German DMKG Headache Study. *Cephalalgia*; **29**: 48–57.

[4] Rasmussen BK, Jensen R, Olesen J. A Population-Based Analysis of the Diagnostic Criteria of the International Headache Society. *Cephalalgia* 1991; **11**: 129–34.

[5] Fendrich K, Vennemann M, Pfaffenrath V, *et al.* Headache Prevalence Among Adolescents — The German DMKG Headache Study. *Cephalalgia* 2007; **27**: 347–54.

[6] Vos T, Lim SS, Abbafati C, *et al.* Global burden of 369 diseases and injuries in 204 countries and territories, 1990–2019: a systematic analysis for the Global Burden of Disease Study 2019. *The Lancet* 2020; **396**: 1204–22.

Hearing impairment

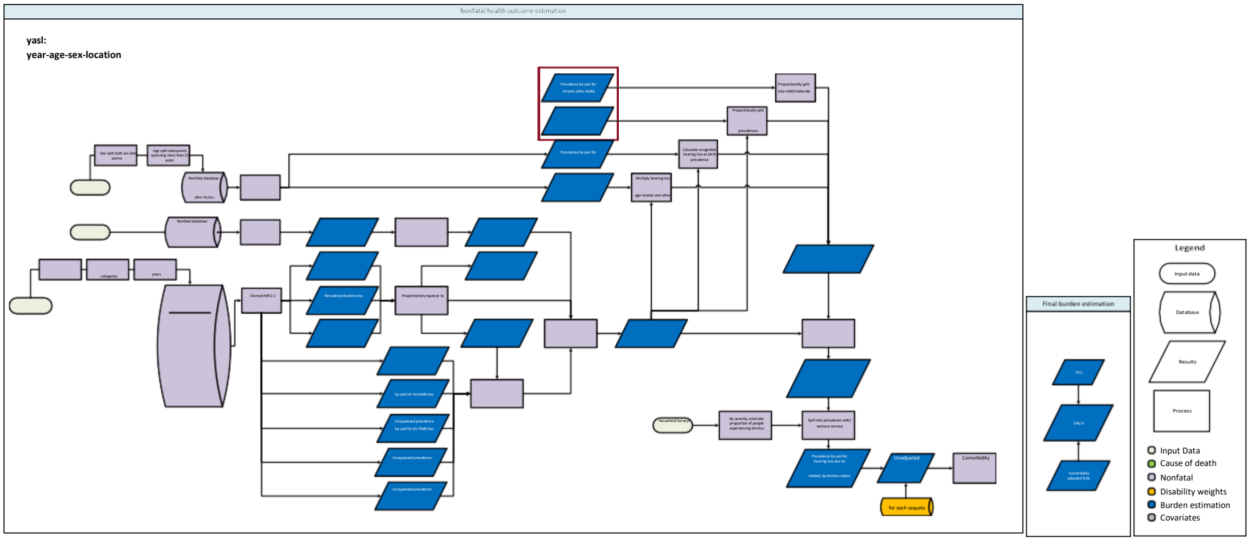

Case definition

Hearing impairment is defined as the biologically irreversible loss of the ability to perceive externally produced sounds, either through damage to the middle or inner ear or to neural circuitry underlying hearing. Hearing loss can be caused by genetics, altered neonatal development, loss of inner ear hair cells, or infection, and is common during aging. Hearing impairment is an estimation of the prevalence of hearing loss at a range of severities, as measured by the softest sound that an individual can hear in their better ear, taken as the average across frequencies from 500 to 4000 Hertz.

Hearing impairment is modelled for every year, age, sex, and location in the following severity categories:

Table 1: Severity thresholds of hearing loss

Severity thresholds of interest for hearing loss	
Severity	Threshold (in decibels)
None	0–19
Mild	20–34
Moderate	35–49
Moderately severe	50–64
Severe	65–79
Profound	80–94
Complete	95+

We modelled the following causes of hearing loss: congenital, meningitis, otitis, and age-related and other. Congenital hearing loss is defined as hearing loss present at birth. Age-related and other hearing loss includes causes not identified as meningitis, otitis, or congenital. This includes presbycusis, the gradual loss of hearing with age, caused by breakdown of sensory receptors in the inner ear. For all causes, we estimate hearing loss with and without tinnitus, the perception of noise or ringing in the ears.

Unadjusted estimates of the prevalence of hearing loss due to meningitis and chronic otitis media are produced separately as part of each underlying cause's modelling process, as described in their respective

sections. Along with the congenital and age-related aetiologies, these unadjusted estimates are incorporated into the overall hearing loss model, as detailed below.

Input data and processing

Studies on hearing loss typically report the prevalence of hearing loss by severity, in categories that are mutually exclusive and exhaustive. The severity grouping that an individual is put into depends on the softest decibel level at which they can hear a sound. However, these severity groupings are not standardised across literature. For example, one study may report the prevalence of mild, moderate, and severe hearing loss across the range of decibels. Another study may simply report the prevalence of the study population with no hearing loss, and those that have hearing loss, regardless of range. To standardise severity groupings, we established seven mutually exclusive and exhaustive categories that the GBD would use to model and report the severity of hearing loss. These are referred to as "severity-specific envelopes". The range of decibel values applicable to each severity category can be seen in table 1.

For the estimation of severity-specific envelopes, we used prevalence measurements and individual-level data extracted from published surveys identified in a series of systematic reviews, or from sources provided by the GBD collaborator network.

Data sources up to 2008 were identified by a published systematic review (http://www.ncbi.nlm.nih.gov/pubmed/19444763). For GBD 2013, we conducted a systematic review covering 2008–2013 with the following search terms:

> (hearing impairment[Title/Abstract] OR deafness[Title/Abstract] OR hearing loss[Title/Abstract]) AND (prevalence[Title/Abstract]) AND ("2008"[PDAT] : "3000"[PDAT]) AND (cross sectional OR survey)

For GBD 2016, we conducted an additional systematic review using the following search terms:

(hearing impairment[Title/Abstract] OR deafness[Title/Abstract] OR hearing loss[Title/Abstract] OR audiometry[Title/Abstract]) AND (prevalence[Title/Abstract]) AND ("2008/11/26"[PDAT]: "3000"[PDAT]) AND (cross sectional OR survey)

This was conducted on November 30, 2016, and returned 239 results, of which 17 were accepted.

For GBD 2021, we conducted an additional systematic review using the following search terms:

> hearing imp*[Title/Abstract] OR deaf*[Title/Abstract] OR hearing loss[Title/Abstract] OR audio*[Title/Abstract]) AND (prevalen*[Title/Abstract]) AND ("2016/11/16"[PDAT] : "3000"[PDAT]) AND (cross sectional OR survey)

This was conducted on November 5, 2019, and returned 431 results, of which 17 were accepted. The PRISMA diagram for this systematic review is shown below.

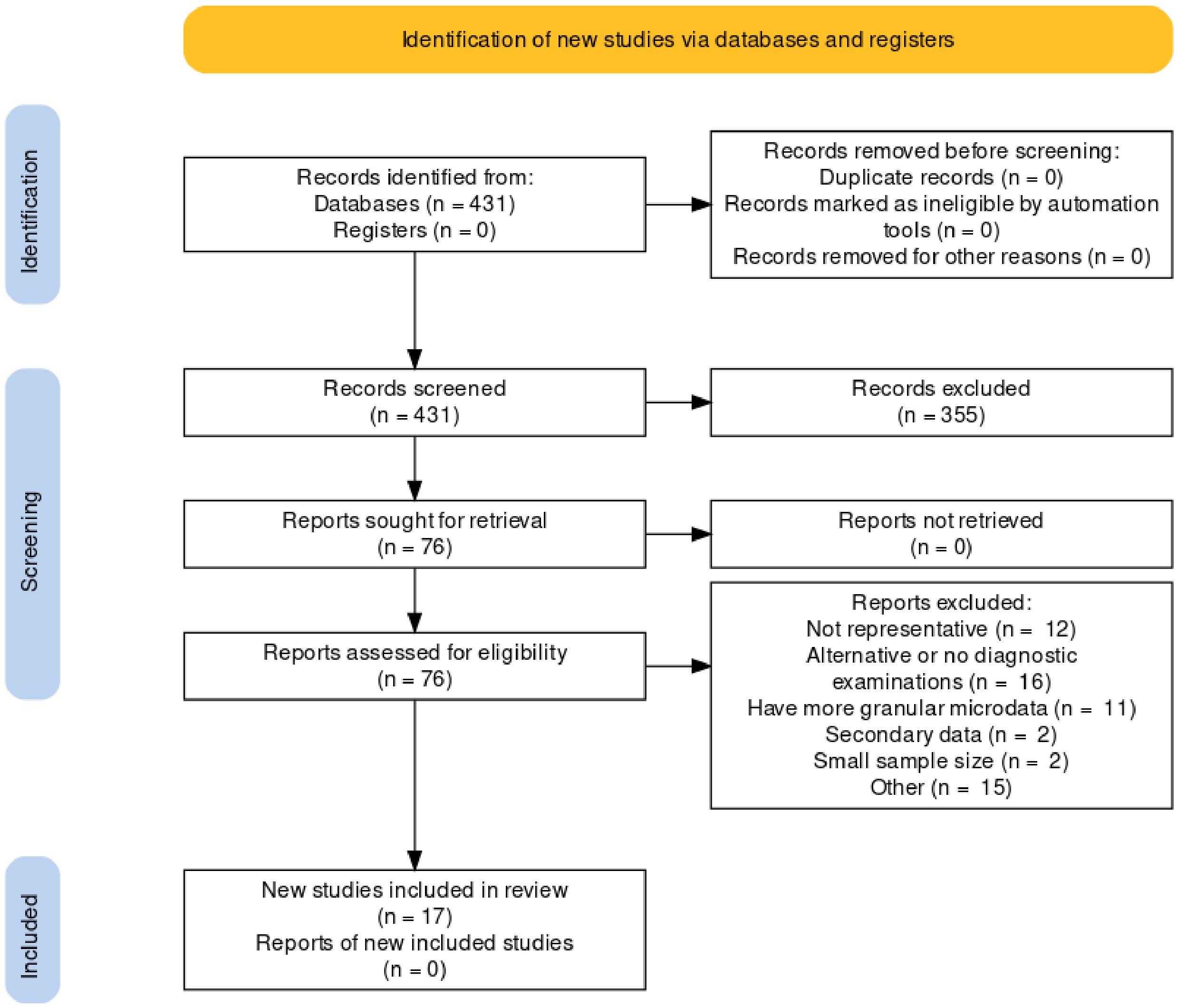

In addition to the search-string hits above, we identified household surveys that measured hearing loss – the United States National Health and Examination Surveys (NHANES) and the Health Survey for England (HSE) – and extracted prevalence measurements from individual-level data.

Self-reported hearing loss data were excluded. This includes censuses in the Integrated Public Use Microdata Series (IPUMS), the WHO Studies on Global Ageing and Adult Health (SAGE), and the WHO Multi-Country Survey Study on Health and Responsiveness (MCSS). Self-reported use of hearing aids (such as in MCSS, SAGE, and NHANES), however, was used to estimate hearing aid coverage.

Table 2: Data inputs

Cause/impairment name	Measure	Total sources	Countries with data
Hearing loss	All measures	271	89
	Prevalence	267	89
	Other	11	2
Age-related and other hearing loss	All measures	58	34

	Proportion	58	34

For studies that did not report prevalence by sex, datapoints were split by sex based by running a regression on the log ratio of female/male prevalence within each severity-specific dataset using MR-BRT (meta-regression—Bayesian, regularised, trimmed), then applying this ratio to non-specific data (Table 3). Datapoints that also reported data in wide age groups (>25 years) were split into 5-year age bins by applying the age pattern of the best severity-specific GBD 2019 model.

Table 3. Sex split coefficients for hearing loss models, with 95% UIs, log-space

**Model**	**Sex split model coefficient, log-space (95% UI)**
Hearing loss, 0–19 dB	0.0171 (–0.0163 to 0.0505)
Hearing loss, 20–34 dB	–0.211 (–0.583 to 0.161)
Hearing loss, 35–49 dB	–0.240 (–0.511 to 0.031)
Hearing loss, 35+ dB	–0.139 (–0.48 to 0.202)
Hearing loss, 50–64 dB	–0.468 (–0.629 to –0.307)
Hearing loss, 65–79 dB	–0.383 (–0.536 to –0.230)
Hearing loss, 80–94 dB	–0.0738 (–0.562 to 0.414)
Hearing loss, 95+ dB	–0.149 (–0.317 to 0.019)

Where studies reported hearing loss spanning multiple thresholds (eg, 80+, rather than 80–94 and 95+) or severity categories that did not align with GBD thresholds, we crosswalked data with the MR-BRT methodology to the appropriate GBD severity categories. A description of the MR-BRT methodology can be found in its respective section.

To create adjustment factors between alternate and reference threshold categories, we used microdata extracted from NHANES surveys. These data reported the exact decibel at which each person experienced hearing loss. We estimated the prevalence of each alternate and reference severity category by aggregating microdata into groups specific to age and sex. The prevalent population for each alternate or reference category was composed of every individual that fell within the range of decibels for a given severity. Adjustment factors were estimated as the logit difference between the prevalence of an alternate category and the prevalence of its corresponding reference category. A table of each adjustment factor can be found below.

Table 4: **MR-BRT crosswalk adjustment factors**

Reference Category (dB)	Alternate Category (dB)	Gamma	Beta coefficient, logit (95% UI)*	Adjustment factor**
0-19	0-24	0	0.60 (0.54 to 0.67)	1.82 (1.72 to 1.95)
	0-25	0	0.70 (0.64 to 0.77)	2.01 (1.90 to 2.16)
	0-29	0.23	1.13 (0.68 to 1.59)	3.10 (1.97 to 4.90)
	0-30	0.21	1.24 (0.83 to 1.68)	3.46 (2.29 to 5.37)
	0-39	0.91	1.67 (–0.04 to 3.58)	5.31 (0.96 to 35.87)
	0-40	0.96	1.71 (–0.05 to 3.53)	5.53 (0.95 to 34.12)

0-19	10-25	2.06	–1.34 (–1.40 to –1.36)	0.26 (0.25 to 0.26)
20-34	0-24	2.50	3.40 (–1.46 to 8.28)	29.96 (0.23 to 3944.19)
	0-25	2.45	3.49 (–1.53 to 8.29)	32.79 (0.22 to 3983.83)
	0-29	2.30	3.82 (–0.85 to 8.29)	45.60 (0.43 to 3983.83)
	0-30	2.27	3.89 (–0.24 to 8.42)	48.91 (0.79 to 4536.90)
	0-39	1.95	4.48 (0.61 to 8.55)	88.23 (1.84 to 5166.75)
	0-40	1.91	4.50 (0.86 to 8.14)	90.02 (2.36 to 3428.92)
	15-24	.42	0.28 (0.28 to 0.29)	1.32 (1.32 to 1.34)
	20-39	0.13	0.27 (0.02 to 0.52)	1.31 (1.02 to 1.68)
	20-40	0.15	0.29 (0.003 to 0.59)	1.34 (1.00 to 1.80)
	20-200	0.41	0.52 (–0.35 to 1.32)	1.68 (0.70 to 3.74)
	21-39	0.20	0.12 (–0.29 to 0.52)	1.13 (0.75 to 1.68)
	25-39	0.35	–0.39 (–1.04 to 0.34)	0.68 (0.35 to 1.40)
	26-30	0	–1.42 (–1.42 to –1.42)	0.24 (0.24 to 0.24)
	26-40	0.43	–0.50 (–1.36 to 0.28)	0.61 (0.26 to 1.32)
	26-99	0.84	–0.03 (–1.65 to 1.73)	0.97 (0.19 to 5.64)
	26-200	0.84	–0.03 (–1.74 to 1.54)	0.97 (0.18 to 4.66)
	30-40	0.56	–1.06 (–2.24 to 0.007)	0.35 (0.11 to 1.01)
	30-200	0.96	–0.37 (–2.12 to 1.43)	0.69 (0.12 to 4.18)
35-49	0-39	2.45	5.18 (0.16 to 10.08)	177.68 (1.17 to 23 860.99)
	0-40	2.42	5.24 (0.41 to 10.17)	188.67 (1.51 to 26 108.08)
	20-39	0.71	1.45 (0.04 to 2.85)	4.26 (1.04 to 17.29)
	20-40	0.69	1.49 (0.10 to 2.88)	4.44 (1.11 to 17.81)
	21-39	0.66	1.31 (0.02 to 2.67)	3.71 (1.02 to 14.44)
	25-39	0.54	0.76 (–0.27 to 1.93)	2.14 (0.76 to 6.89)
	26-40	0.51	0.67 (–0.30 to 1.75)	1.95 (0.74 to 5.75)
	30-40	0.47	0.09 (-0.89 to 1.05)	1.09 (0.41 to 2.86)
	31-50	0.52	0.10 (0.29 to 0.74)	1.11 (1.34 to 2.10)
	35-60	0	0.38 (0.38 to 0.39)	1.46 (1.46 to 1.48)

	40-64	0.37	–0.10 (–0.85 to 0.61)	0.90 (0.43 to 1.84)
	40-69	0.40	–0.04 (–0.82 to 0.811)	0.96 (0.44 to 2.25)
	41-55	0.32	–0.45 (–1.06 to 0.23)	0.64 (0.35 to 1.26)
	41-60	0.35	–0.29 (–0.99 to 0.37)	0.75 (0.37 to 1.45)
	41-70	0.44	–0.12 (–1.06 to 0.76)	0.89 (0.35 to 2.14)
50-64	31-60	0	0.82 (0.82 to 0.82)	2.27 (2.27 to 2.27)
50-64	35-60	0.03	1.52 (1.51 to 1.53)	4.57 (4.53 to 4.62)
50-64	40-64	0.27	1.13 (0.58 to 1.68)	3.10 (1.79 to 5.37)
	40-69	0.29	1.22 (0.64 to 1.80)	3.39 (1.90 to 6.05)
	41-55	0.4	0.72 (–0.09 to 1.53)	2.05 (0.91 to 4.62)
	41-60	0.31	0.92 (0.30 to 1.55)	2.51 (1.35 to 4.71)
	41-70	0.32	1.13 (0.49 to 1.77)	3.10 (1.63 to 5.87)
	41-80	0	1.27 (1.26 to 1.28)	3.56 (3.53 to 3.60)
	51-70	0.18	0.06 (–0.31 to 0.42)	1.06 (0.73 to 1.52)
	55-69	0.29	–0.42 (–1.00 to 0.15)	0.66 (0.37 to 1.16)
	56-70	0.33	–0.43 (–1.10 to 0.24)	0.65 (0.33 to 1.27)
65-79	40-69	0.77	2.44 (0.92 to 3.99)	11.47 (2.51 to 54.05)
	41-80	0	2.61 (2.59 to 2.63)	13.60 (13.33 to 13.87)

	51-70	0.67	1.35 (0.01 to 2.68)	3.86 (1.01 to 14.59)
	55-69	0.69	0.86 (–0.53 to 2.24)	2.36 (0.59 to 9.39)
	56-70	0.66	0.84 (–0.47 to 2.16)	2.32 (0.63 to 8.67)
	61-80	0.19	0.35 (–0.04 to 0.72)	1.42 (0.96 to 2.05)
	61-99	0.14	0.46 (0.17 to 0.75)	1.58 (1.19 to 2.12)
	65-84	0.02	0.03 (–0.01 to 0.08)	1.03 (0.99 to 1.08)
	70-89	0.21	–0.20 (–0.63 to 0.22)	0.82 (0.53 to 1.25)
	70-94	0.21	–0.20 (–0.62 to 0.24)	0.82 (0.54 to 1.27)
	70-95	0.21	–0.20 (–0.63 to 0.23)	0.82 (0.53 to 1.26)
	71-90	0.3	–0.26 (–0.86 to 0.34)	0.77 (0.42 to 1.40)
	71-99	0.3	–0.16 (–0.75 to 0.44)	0.85 (0.47 to 1.55)
	71-200	0.31	–0.19 (–0.81 to 0.42)	0.83 (0.44 to 1.52)
80-94	61-99	1.01	1.58 (–0.42 to 3.58)	4.85 (0.66 to 35.87)
	65-84	0.91	0.92 (–0.89 to 2.73)	2.51 (0.41 to 15.33)
	70-89	0.81	0.54 (–1.06 to 2.14)	1.72 (0.35 to 8.50)
	70-94	0.73	0.44 (–1.01 to 1.88)	1.55 (0.36 to 6.55)
	70-95	0.73	0.44 (–1.00 to 1.89)	1.55 (0.37 to 6.62)
	71-90	0.61	0.25 (–0.96 to 1.45)	1.28 (0.38 to 4.26)
	71-99	0.61	0.37 (–0.83 to 1.58)	1.45 (0.44 to 4.85)
	71-200	0.66	0.41 (–0.88 to 1.71)	1.51 (0.41 to 5.53)
	80-200	0	0.00 (–0.04 to 0.04)	1.00 (0.96 to 1.04)
	81-90	0	–0.027(–0.32 to 0.26)	0.97 (0.72 to 1.30)
	81-99	0	$–3.92e^{-16}$ (–0.04 to 0.03)	1.00 (0.96 to 1.03)
	81-200	0	0.00 (–0.04 to 0.04)	1.00 (0.96 to 1.04)
	85-200	0	$–4.37e^{-24}$ (–0.04 to 0.04)	1.00 (0.96 to 1.04)
	90-99	0	0.00 (–0.03 to 0.03)	1.00 (0.97 to 1.03)
	90-200	0	0.00 (–0.03 to 0.03)	1.00 (0.97 to 1.03)
35-200	15-200	0	2.70 (2.69 to 2.70)	14.88 (14.73 to 14.88)
35-200	20-200	0.15	1.79 (1.48 to 2.10)	5.99 (4.39 to 8.17)
	25-200	0	1.17 (1.16 to 1.17)	3.22 (3.19 to 3.22)
	26-200	0.14	1.02 (0.73 to 1.31)	2.77 (2.08 to 3.71)
	26-99	0.14	1.02 (0.73 to 1.31)	2.77 (2.08 to 3.71)

	30-200	0.07	0.55 (0.40 to 0.70)	1.73 (1.49 to 2.01)
	31-200	0.05	0.43 (0.33 to 0.54)	1.54 (1.39 to 1.72)
	31-99	0.04	0.44 (0.34 to 0.54)	1.55 (1.40 to 1.72)
	40-200	0.04	–0.49 (–0.58 to –0.39)	0.61 (0.56 to 0.68)
	40-99	0.05	–0.48 (–0.59 to –0.38)	0.62 (0.55 to 0.68)
	41-200	0.09	–0.59 (–0.78 to –0.39)	0.55 (0.46 to 0.68)
	41-99	0.10	–0.58 (–0.78 to –0.39)	0.56 (0.46 to 0.68)
95-2000	61-99	0.80	2.42 (0.84 to 4.03)	11.25 (2.32 to 56.26)
	71-99	0.90	0.65 (–1.14 to 2.43)	1.92 (0.32 to 11.36)
	71-200	0.88	0.60 (–1.13 to 2.33)	1.82 (0.32 to 10.28)
	80-200	0.22	0.08 (–0.34 to 0.52)	1.08 (0.71 to 1.68)
	81-99	0.21	0.08 (–0.35 to 0.50)	1.08 (0.70 to 1.65)
	81-200	0.18	0.05 (–0.30 to 0.41)	1.05 (0.74 to 1.51)
	85-200	0	0.00 (–0.04 to 0.04)	1.00 (0.96 to 1.04)
	90-99	0	0.00 (–0.02 to 0.02)	1.00 (0.98 to 1.02)
	90-200	0	0.00 (–0.02 to 0.02)	1.00 (0.98 to 1.02)
	91-99	0	0.00 (–0.03 to 0.02)	1.00 (0.97 to 1.02)
	91-200	0	0.00 (–0.02 to 0.02)	1.00 (0.98 to 1.02)
	95-99	0	0.00 (–0.02 to 0.02)	1.00 (0.98 to 1.02)
	96-99	0	0.00 (–0.02 to 0.02)	1.00 (0.98 to 1.02)

**MR-BRT crosswalk adjustments can be interpreted as the factor the alternative case definition is adjusted by to reflect what it would have been had it been measured using the reference case definition. If the log/logit beta coefficient is negative, then the alternative is adjusted up to the reference. If the log/logit beta coefficient is positive, then the alternative is adjusted down to the reference.*

***The adjustment factor column is the exponentiated beta coefficient. For log beta coefficients, this is the relative rate between the two case definitions. For logit beta coefficients, this is the relative odds between the two case definitions.*

Modelling strategy

We modelled the prevalence of hearing loss over five steps. First, we ran three DisMod-MR 2.1 (disease model—Bayesian meta-regression) models to estimate the total prevalence of the following levels of hearing by y-a-s-l: normal hearing (0–19 dB), mild hearing loss (20–34 dB), and moderate hearing loss and above (35+ dB). For normal hearing loss (0–19 dB), DisMod-MR 2.1 had trouble fitting prevalence values close to 100% in very young ages. Initial models attempted to follow lower prevalence datapoints in teen and middle-aged populations, and resulting, estimates of the prevalence of normal hearing in infants were implausible in the face of the data. As a solution, we modelled all data adjusted to the normal hearing loss category as 1–prevalence, to accommodate for the fact that DisMod-MR 2.1 interacts better with datapoints at lower values. We then took the complement of the fitted model at the draw level to obtain normal hearing prevalence estimates. Next, we rescaled the prevalence estimates from the three models (0–19, 20–34, 35+) to sum to 1 for every year, age, sex, and location. We estimated prevalence of normal hearing for the purpose of correctly scaling the other two models only, and hence it did not form part of further analysis.

These three models used Socio-demographic Index (SDI) as a covariate. SDI was also used as a covariate in GBD 2017 and GBD 2019. The estimated betas are shown in the table below.

Table 4: Covariates

Model	Covariate name	Measure	Beta value	Exponentiated value
Hearing loss impairment at 0-19 dB	Socio-demographic Index	Prevalence	0.038 (0.00017 to 0.011)	1.00 (1.00 to 1.01)
Hearing loss impairment at 35+ dB	Socio-demographic Index	Prevalence	–0.073 (–0.26 to –0.002)	0.93 (0.77 to 1.00)
Hearing loss impairment at 95+ dB	Socio-demographic Index	Prevalence	–1.79 (–1.99 to –1.3)	0.17 (0.14 to 0.27)

Second, we ran five additional DisMod-MR 2.1 models for each severity level of hearing loss greater than mild hearing loss: moderate (35–49 dB), moderately severe (50–64 dB), severe (65–79 dB), profound (80–94 dB), and complete (95+ dB). We then rescaled the prevalence estimates from these models to fit within the prevalence estimated for 35+ dB in the first step. By the end of the second step, we had estimated prevalence of six severity levels of hearing loss, including mild (20–34 dB).

Third, we ran two additional DisMod-MR 2.1 models to (1) estimate the proportion of the hearing impaired that use a hearing aid, deemed “hearing aid coverage”, and (2) estimate the proportion of hearing loss across all severities that is attributable to age-related and other factors.

Fourth, we adjusted the prevalence of each of the six hearing loss severity levels estimated in steps one and two to account for hearing aid use. To do this, we made the assumption that the use of a hearing aid reduces the severity of impairment by one category. The model used to estimate hearing aid coverage

represents *all* severity categories. To estimate the proportion of hearing aid coverage for *each* severity category, we used data obtained from the Nord-Trondelag study and NHANES surveys. These two sources provided detailed information on hearing aid coverage among the impaired by age, sex, and most important, severity. We ran a logistic regression on age with binary indicators for severity levels and sex. Outputs of this regression were the proportion of individuals at every severity of hearing impairment that used a hearing aid. We assumed that 0% of people in the completely deaf category (95+) used a hearing aid. We then took estimates of hearing aid coverage that were produced in step 3 and scaled the estimate by dividing the value produced in each location by the value produced for Norway. This was to correct for any bias created by using adjustment factors calculated primarily with data from Norway. From there, we multiplied the scaled value of hearing aid coverage for each location by each of the six proportions of severity-specific coverage. This gave us the proportion of individuals in each severity category that use a hearing aid. Then, we shifted the identified fraction of people in each severity category that used a hearing aid to the category directly below. This provided the adjusted prevalence of six severity levels of all-cause hearing loss.

Fifth, we estimated the prevalence of hearing loss due to multiple causes: otitis media, congenital, meningitis, and age-related and other causes not classified elsewhere. In GBD 2017, we estimated the prevalence of hearing loss for each subtype of meningitis (pneumococcal, *H influenzae* type B meningitis, meningococcal, and other bacterial), but from GBD 2019 onward, we estimated the prevalence of hearing loss for meningitis as a whole. See the meningitis cause write-up for further details. For congenital hearing loss, we assumed that all hearing loss occurring at the time of birth is of congenital nature. We also assumed that all hearing loss due to otitis media is at the mild or moderate level. Because data on the aetiology of hearing loss are more stable in younger ages, up to the age of 20, we implemented proportional squeezes to scale cause-specific hearing loss prevalence to the total prevalence of each severity level. Above age 20, we subtracted the prevalence of congenital hearing loss, meningitis, and otitis from the total and called any remainder age-related and other hearing loss. Since we ensured that congenital prevalence was constant in each age group for every location, year, and sex combination after conducting the proportional squeeze, the sum of the prevalence of all hearing loss aetiologies sometimes exceeded the total prevalence of some severity levels.

Finally, we estimated the percentage of people experiencing tinnitus. We determined the proportion of people suffering from tinnitus using data from NHANES years that asked about the frequency each survey respondent heard ringing, roaring, and/or buzzing (1999, 2001, 2003, and 2011–2012). We labelled anyone with mild hearing loss and ringing, roaring, or buzzing “at least once a month” as a mild hearing loss with tinnitus case. Anyone with moderate hearing through to severe hearing loss and ringing, roaring, or buzzing “at least once a day” was labelled as a moderate hearing loss with tinnitus case. Anyone with complete hearing loss who responded that they “almost always” had ringing or buzzing was labelled as a complete hearing loss with tinnitus case. Using the data from NHANES, we calculated confidence intervals assuming a binomial distribution. We assumed the same distribution of tinnitus across all aetiologies of hearing loss. This is the same strategy used in previous GBD cycles.

Table 5: Health states and disability weights

Health state name	Health state description	Disability weight
Hearing loss, mild	has great difficulty hearing and understanding another person talking in a noisy place (for example, on an urban street).	0.010 (0.004–0.019)
Hearing loss, mild, with ringing	has great difficulty hearing and understanding another person talking in a noisy place (for example, on an urban street), and sometimes has annoying ringing in the ears.	0.021 (0.012–0.036)

Hearing loss, moderate	is unable to hear and understand another person talking in a noisy place (for example, on an urban street), and has difficulty hearing another person talking even in a quiet place or on the phone.	0.027 (0.015–0.042)
Hearing loss, moderate, with ringing	is unable to hear and understand another person talking in a noisy place (for example, on an urban street), and has difficulty hearing another person talking even in a quiet place or on the phone, and has annoying ringing in the ears for more than 5 minutes at a time, almost every day.	0.074 (0.048–0.107)
Hearing loss, moderately severe	(custom DW from hearing loss impairment envelope)	0.092 (0.064–0.129)
Hearing loss, moderately severe, with ringing	(custom DW from hearing loss impairment envelope)	0.167 (0.114–0.231)
Hearing loss, severe	is unable to hear and understand another person talking, even in a quiet place, and unable to take part in a phone conversation. Difficulties with communicating and relating to others cause emotional impact at times (for example worry or depression).	0.158 (0.104–0.227)
Hearing loss, severe, with ringing	is unable to hear and understand another person talking, even in a quiet place, and unable to take part in a phone conversation, and has annoying ringing in the ears for more than 5 minutes at a time, almost every day. Difficulties with communicating and relating to others cause emotional impact at times (for example worry or depression).	0.261 (0.174–0.361)
Hearing loss, profound	is unable to hear and understand another person talking, even in a quiet place, is unable to take part in a phone conversation, and has great difficulty hearing anything in any other situation. Difficulties with communicating and relating to others often cause worry, depression, and loneliness.	0.204 (0.134–0.288)
Hearing loss, profound, with ringing	is unable to hear and understand another person talking, even in a quiet place, is unable to take part in a phone conversation, has great difficulty hearing anything in any other situation, and has annoying ringing in the ears for more than 5 minutes at a time, several times a day. Difficulties with communicating and relating to others often cause worry, depression, or loneliness.	0.277 (0.182–0.388)
Hearing loss, complete	cannot hear at all in any situation, including even the loudest sounds, and cannot communicate verbally or use a phone. Difficulties with communicating and relating to others often cause worry, depression, or loneliness.	0.215 (0.143–0.307)
Hearing loss, complete, with ringing	cannot hear at all in any situation, including even the loudest sounds, and cannot communicate verbally or use a phone, and has very annoying ringing in the ears for more than half of the day. Difficulties with communicating and relating to others often cause worry, depression, or loneliness.	0.316 (0.211–0.436)

Heart failure impairment

Flowcharts

Overall modelling strategy

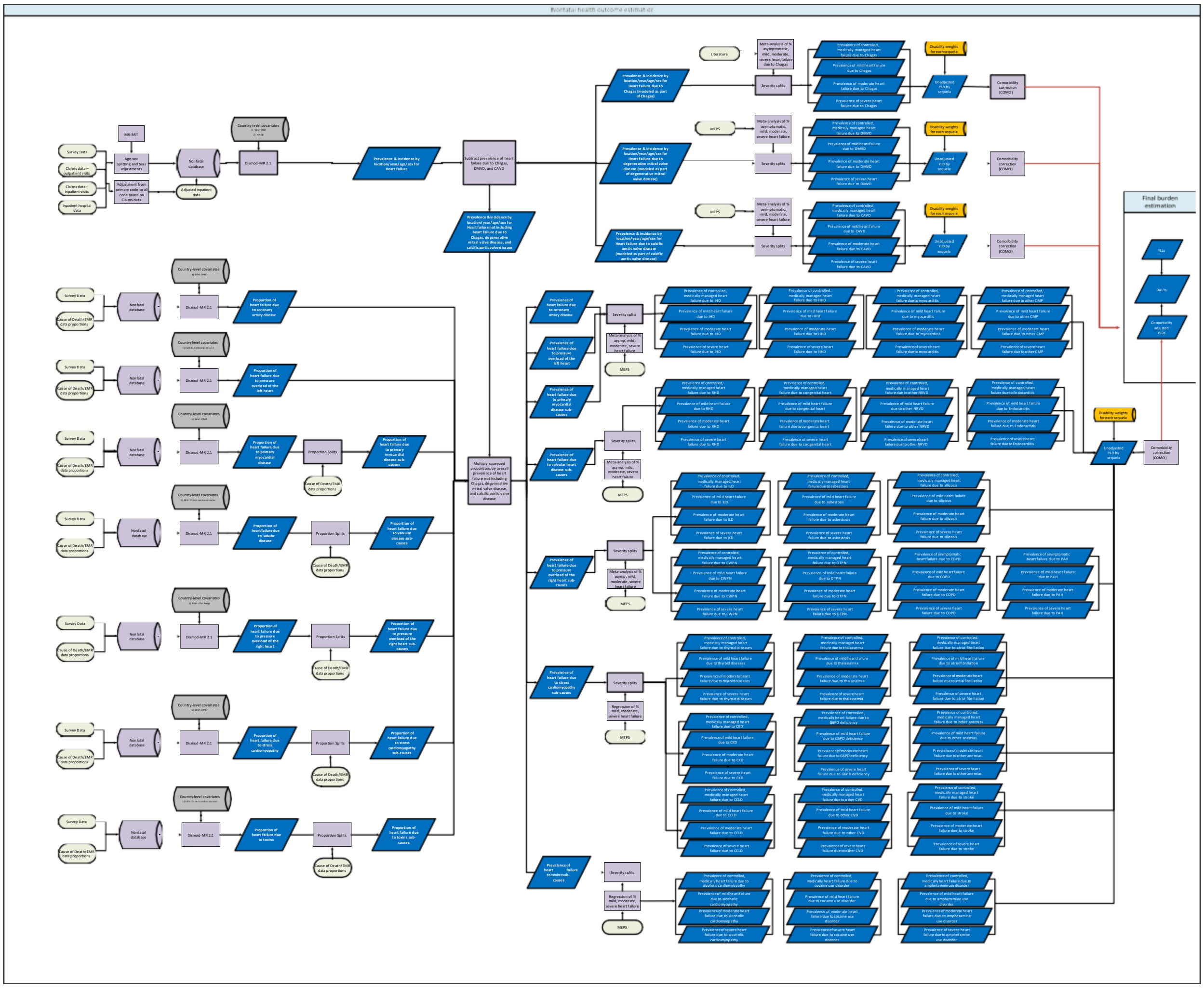

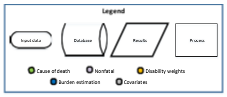

Abbreviations

DMVD: degenerative mitral valve disease; CAVD: calcific aortic valve disease; IHD: ischaemic heart disease; CMP: cardiomyopathy and myocarditis; HHD: hypertensive heart disease; ILD: interstitial lung disease; CWPN: coal workers pneumoconiosis; OTPN: other pneumoconiosis; COPD: chronic obstructive pulmonary disease; RHD: rheumatic heart disease; CVD: cardiovascular disease; NRVD: non-rheumatic valve disease; PAH: pulmonary arterial hypertension; CKD: chronic kidney disease; CCLD: cirrhosis and other chronic liver diseases.

Proportion splits and correction factor estimation

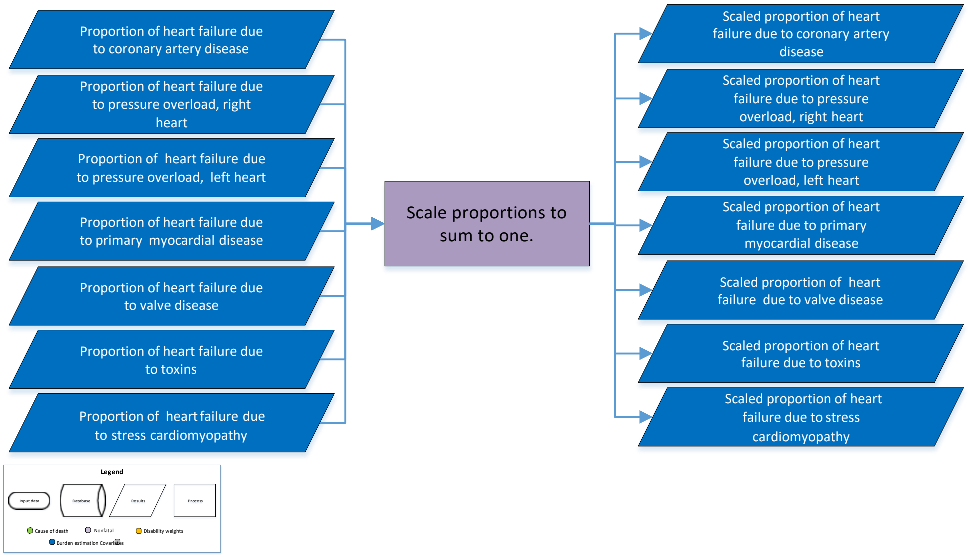

Case definition

The GBD case definition for heart failure impairment data sources includes studies in which heart failure was diagnosed clinically using structured criteria such as the Framingham or European Society of Cardiology criteria. Beginning in GBD 2016, we used ACC/AHA Stage C and above to capture both persons who are currently symptomatic and those who have been diagnosed with heart failure but are currently asymptomatic.

Framingham criteria (1): Must fulfill two major criteria or one major and two minor criteria.
Major criteria: Paroxysmal nocturnal dyspnoea, neck vein distention, rales, radiographic cardiomegaly, acute pulmonary oedema, S3 gallop, increased central venous pressure (>16 cm H_2O at right atrium), hepatojugular reflux; weight loss >4.5 kg in 5 days in response to treatment.
Minor criteria: bilateral ankle oedema, nocturnal cough, dyspnoea on ordinary exertion, hepatomegaly, pleural effusion, decrease in vital capacity by one-third from maximum recorded, tachycardia (heart rate >120 beats/min).

European Society of Cardiology (2): Typical signs (elevated jugular venous pressure, pulmonary crackles and peripheral oedema) and symptoms (eg, breathlessness, ankle swelling, and fatigue) caused by a structural and/or functional cardiac abnormality, resulting in a reduced cardiac output and/or elevated intracardiac pressures at rest or during stress.

Input data

A systematic review was performed GBD 2016 and updated in GBD 2020, along with an unstructured review in 2019. In 2016, the search terms used were: "heart failure"[TIAB] AND (epidemiology[MeSH Terms] OR prevalence[TIAB] OR incidence[TIAB] OR mortality[TIAB]) AND ("1990/01/01"[PDAT] : "2016/09/02"[PDAT]) NOT “animal model” NOT rat NOT mice NOT diabetes[TIAB] NOT “renal transplant”[TIAB]. The dates of the search were 01/01/1990 through 09/02/2016. 37,891 initial hits were returned, and 57 sources were added. An unstructured review yielded an additional 30 sources, of which six were extracted. In 2019, a review of 8 systematic review articles yielded 519 sources to review, of which 14 were extracted. In 2020, the search terms were: "heart failure"[TIAB] OR “cardiac failure”[TIAB])

AND (epidemiology[MeSH Terms] OR prevalence[TIAB] OR incidence[TIAB] OR “excess mortality”[TIAB] OR “case fatality”[TIAB]) AND ("2016/01/01"[PDAT] : "2020/1/2"[PDAT]) NOT “animal model” NOT rat NOT mice NOT diabetes[TIAB] NOT “renal transplant”[TIAB]. 4,469 initial hits were returned and 27 sources were added.

Figure 1: PRISMA 2020 flow diagram

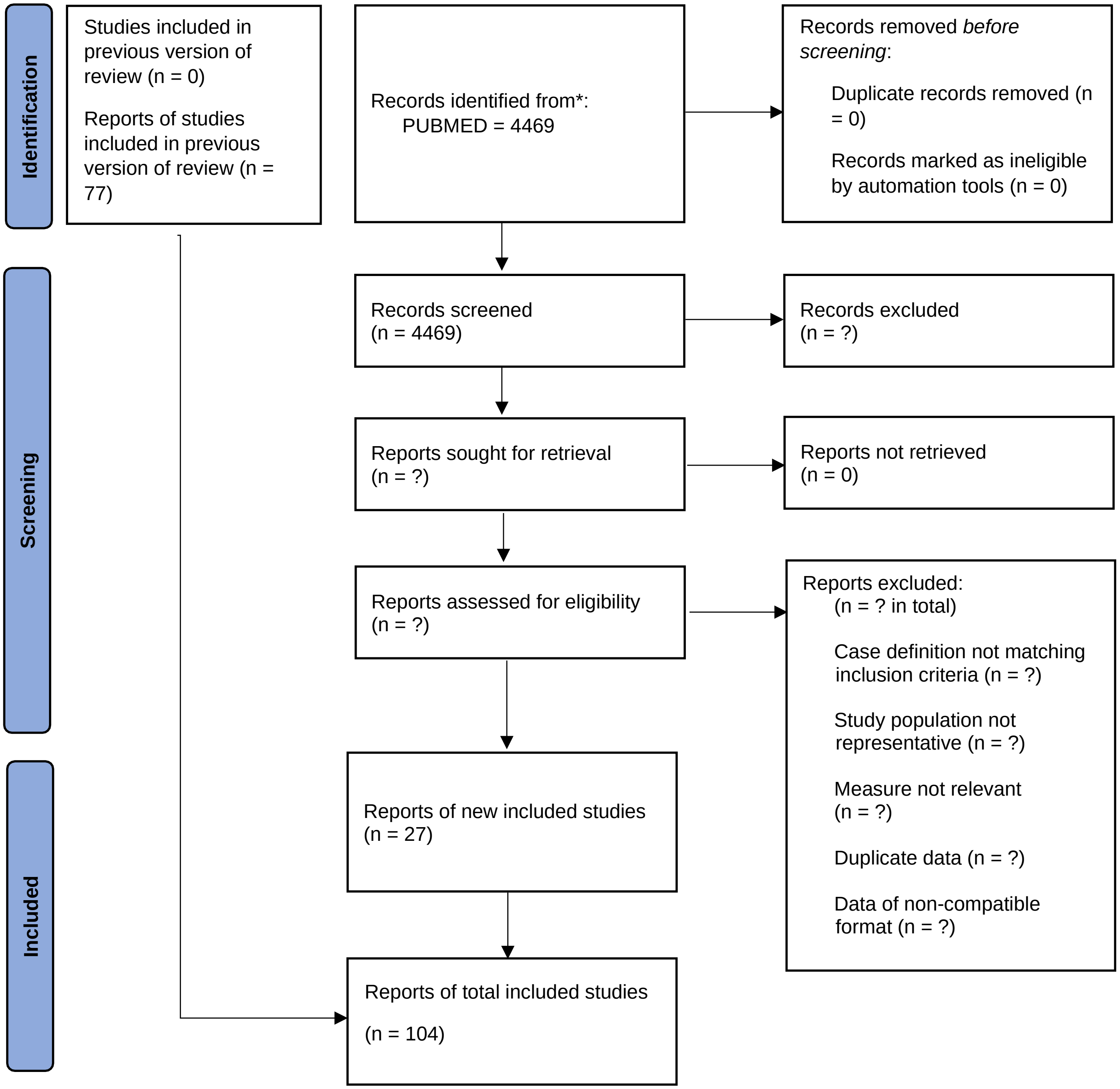

The final dataset also included inpatient hospital data and claims data from the USA. Inpatient hospital data were corrected for readmission, primary diagnosis to any diagnosis ratios, and inpatient to outpatient utilisation ratios using adjustment factors calculated from individual-level claims data. This methodology is detailed elsewhere in the appendix. Inpatient data were excluded if the facilities were not representative of the national population.

Additionally, we used the following data sources to estimate the proportion of heart failure attributable to each aetiology: vital registry data from Mexico, Brazil, Taiwan, Colombia, and the USA; inpatient admissions from Friuli Venezia, Italy; and linked vital registry data from Friuli Venezia, Italy.

Table 1: Source counts for heart failure

Measure	Total sources	Countries with data
Prevalence	217	39
Incidence	44	15
Standardised mortality ratio	2	2
With-condition mortality rate	661	23
Proportion	53	49

Table 2: ICD codes for source counts for heart failure

ICD Codes	ICD description
086.0	Chagas disease with heart involvement
402.01, 402.11, 402.3, 402.7, 402.91	Hypertensive heart disease with heart failure
416	Pulmonary arterial hypertension
425.6	Cardiomyopathy in Chagas disease
428	Heart failure
B572	Chagas disease (chronic) with heart involvement
I09.81	Rheumatic heart failure
I11.0, I11.2	Hypertensive heart disease with heart failure
I27.1	Kyphoscoliotic heart disease
I50	Heart failure
J81, J81.0, J81.1	Pulmonary oedema

Input data were adjusted when there was systematic bias between definitions of heart failure. We adjusted data from inpatient facilities, in which heart failure was identified by ICD codes. We did not adjust claims data or data from epidemiological studies. Consistent with general practices of the GBD, we used the modelling software meta-regression—Bayesian, regularised, trimmed (MR-BRT) to adjust data from inpatient facilities to claims or literature data. Details on MR-BRT and bias adjustment can be found elsewhere in the appendix.

Briefly, we used a network meta-analysis to compare prevalence between our gold-standard definition (physician diagnosis using structured criteria) and alternate definition (ICD diagnosis from inpatient facilities). We modelled the logit difference in prevalence between HF definitions using age (scaled to the observed distribution, mean=75 and SD=12) as a covariate, and applied the adjustment factor to all data from inpatient facilities to correct for systematic bias. Uncertainty from the original data source and crosswalk were propagated to the final estimate. Table 3 shows MR-BRT crosswalk adjustment factors. Figure 2 illustrates the adjustment for inpatient data in Belgium.

MR-BRT was used to split both-sex datapoints into sex-specific estimates. This methodology is detailed elsewhere in the appendix. We also split datapoints where the age range was greater than 25 years. Age splitting was based on the global sex-specific age pattern from a DisMod model that only used input data from scientific literature with less than a 25-year age range.

Case-fatality (CFR) data extracted from a review of published literature were transformed into excess mortality rate (EMR), under the assumption that deaths among those with a diagnosis of heart failure were caused by heart failure. The transformation between CFR and EMR was made using the formula: EMR = –(log(1 – CFR))/(time (years))

In an effort to provide greater guidance to DisMod on the expected pattern of EMR, the transformed EMR data described above were used as inputs to a MR-BRT model (Figure 3). We modelled log(EMR) on sex and a cubic spline for age. We included Healthcare Access and Quality (HAQ) Index as a covariate and specified a prior of a negative coefficient on the association between HAQ Index and EMR. Results from this model were then predicted for each location year, sex, and ten-year age groups. We included HAQ Index as a country-level covariate in DisMod to inform EMR with a mean and standard deviation produced from MR-BRT. However, even without this setting, DisMod would tend to estimate a coefficient that was consistent with the MR-BRT analysis.

Table 3: MR-BRT crosswalk adjustment factors for heart failure prevalence

$$Estimated\ Reference\ Def = invlogit(logit(Alternative\ Def) - Beta_{Alternative\ Def} - Beta_{Age_{scaled}} * Age\ Scaled)$$

To scale the age variable, mean=75 and SD=12

Data input	Reference or alternative case definition	Gamma	Beta coefficient, logit (95% CI)
Structured criteria	Reference	0.04	---
Inpatient data	Alternate		–0.249 (–0.441, –0.057)
Age, scaled			0.093 (0.081, 0.105)

Figure 1: Funnel plot for crosswalk of inpatient data, calculated when age (scaled) = 0

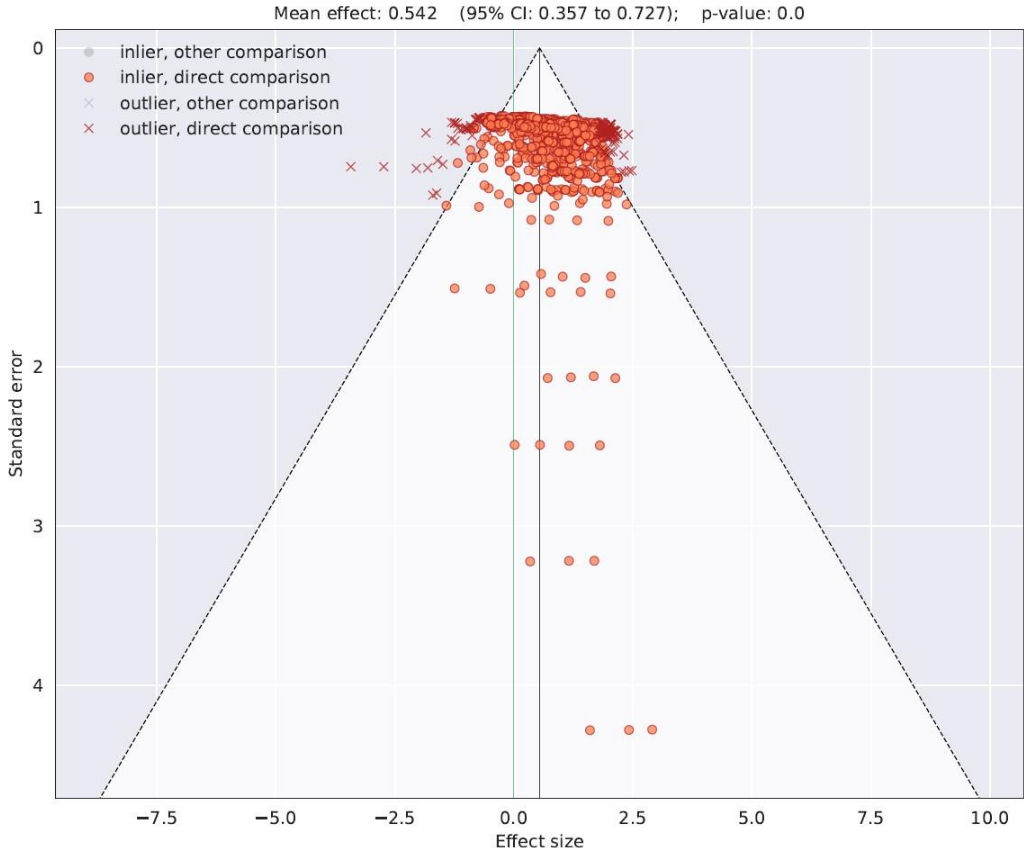

Figure 2: Effect of bias adjustment in Belgium

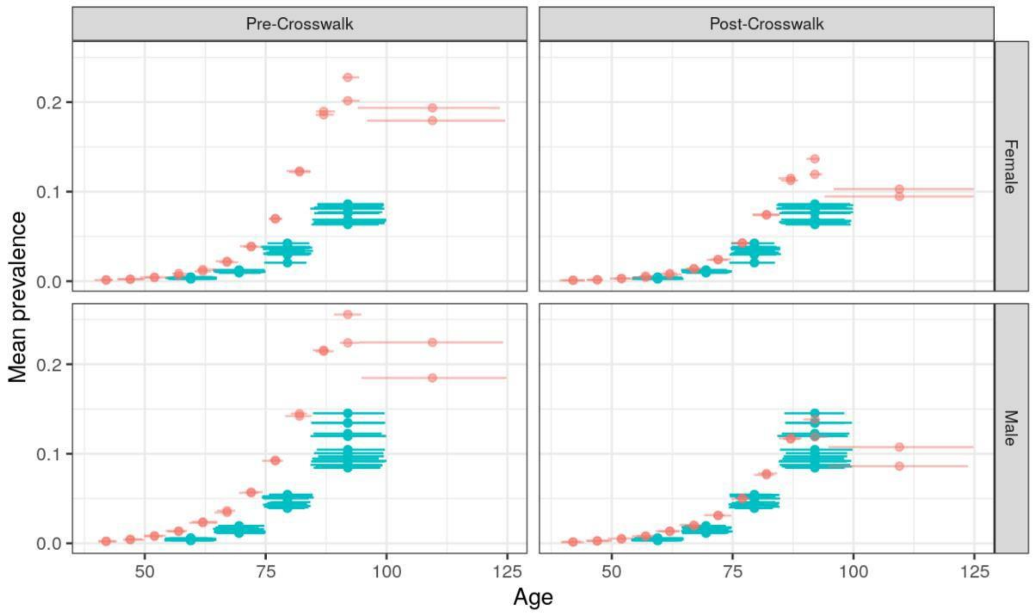

Figure 3: MR-BRT model predictions for excess mortality rate (EMR)

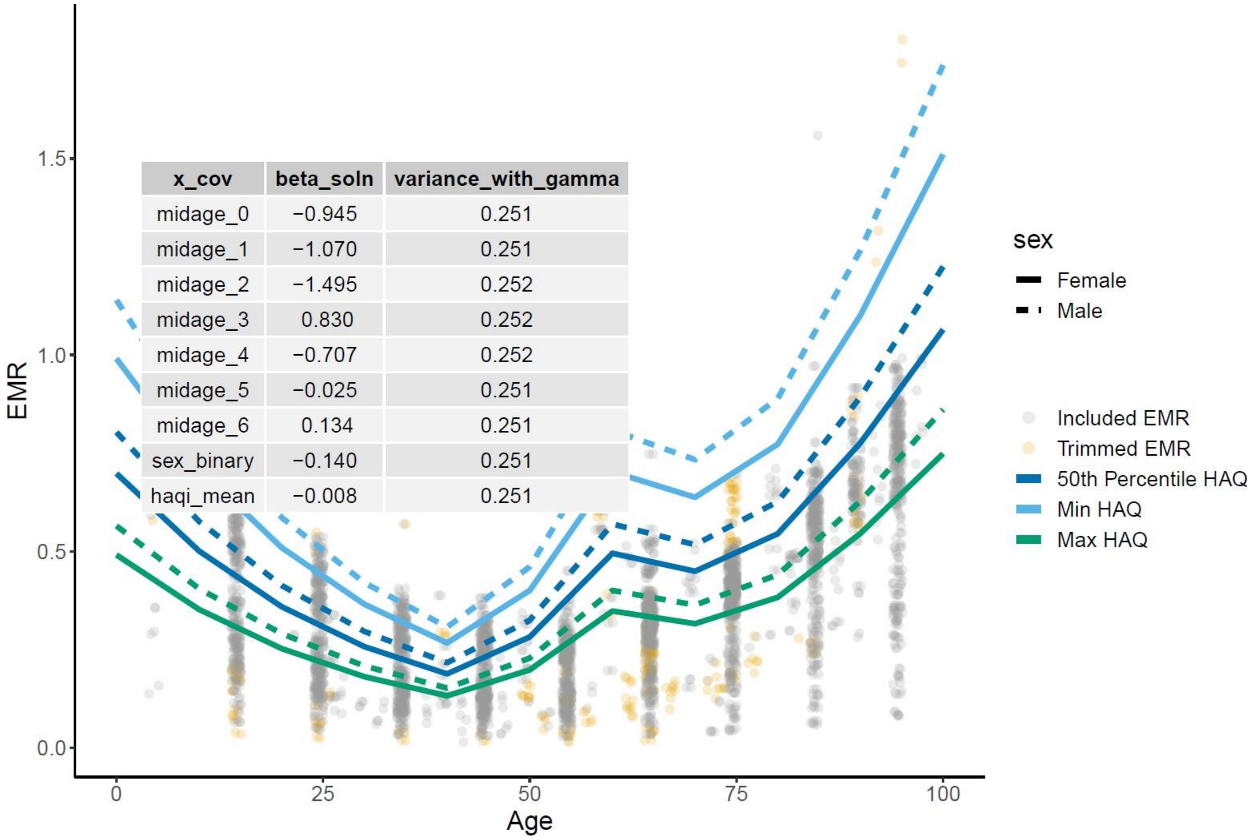

Severity split inputs

These estimates were then split into asymptomatic, mild, moderate, and severe heart failure based on an analysis of MEPS data, with the exception of Chagas disease. MEPS is the only available population-based source that links EQ5D to ICD codes, allowing the application of GBD's standard disability methods. For Chagas, which is not represented in MEPS, we based the severity splits on a meta-analysis of NYHA class among persons diagnosed with heart failure due to Chagas disease in areas where Chagas is endemic (3-6). Disability weights were established for these severities using the standard approach for GBD 2020.

Table 4. Severity distribution, details on the severity levels for heart failure in GBD 2020 and the associated disability weight (DW) with that severity

Severity level	Lay description	DW (95% CI)
Controlled, medically managed	Has been diagnosed with clinical heart failure, a chronic disease that requires medication every day and causes some worry but minimal interference with daily activities.	0.049 (0.031–0.072)
Mild	Is short of breath and easily tires with moderate physical activity, such as walking uphill or more than a quarter-mile on level ground. The person feels comfortable at rest or during activities requiring less effort.	0.041 (0.026–0.062)

Moderate	Is short of breath and easily tires with minimal physical activity, such as walking only a short distance. The person feels comfortable at rest but avoids moderate activity.	0.072 (0.047–0.103)
Severe	Is short of breath and feels tired when at rest. The person avoids any physical activity, for fear of worsening the breathing problems.	0.179 (0.122–0.251)

Modelling strategy

To estimate the burden of overall heart failure and apportion this burden to each of 27 underlying causes (Table 5), we first estimated the overall prevalence of heart failure and then the proportion of heart failure each cause was responsible for. The latter process includes an initial assessment of the fraction of heart failure cases attributable to each of seven high-level parent cause groupings, followed by further division into the detailed causes within each of these groupings. The selection for aetiological causes was based on a review of the literature, a quantitative analysis of the causes most commonly co-occurring with HF in 93 location-years of death certificates and inpatient records, and expert opinion regarding diseases that lead to clinical heart failure. In our review of the literature, we identified diseases whose biological mechanisms are proven to cause heart failure. In our quantitative analysis, we assessed death certificate records with both underlying and contributing causes of death, and hospital records with primary and non-primary diagnoses recorded. With these data, we restricted to patients with heart failure diagnoses, and identified all other diseases recorded in each record. For each country, we tallied the number of times each disease was diagnosed alongside heart failure and identified the 20 most common diseases. Figure 4 is an illustrative example, showing the 20 most commonly co-occurring causes in Italian hospital data. In our expert consultations, we asked disease experts to review and augment our aetiology list. Though hundreds of diseases can result in heart failure, the 27 causes included represent common, clinically relevant, and quantifiable entities that can be stably estimated, and include an “other” category to ensure estimates are collectively exhaustive and mutually exclusive.

Table 5: Heart failure aetiologies and proportion models

**Proportion model**	**Aetiology**
**Coronary artery disease**	Ischaemic heart disease
**Pressure overload, left heart**	Hypertensive heart disease
**Pressure overload, right heart**	COPD<br>Interstitial lung disease and pulmonary sarcoidosis<br>Pulmonary arterial hypertension<br>Silicosis<br>Asbestosis<br>Coal workers pneumoconiosis<br>Other pneumoconiosis
**Valve diseases**	Rheumatic heart disease<br>Congenital heart abnormalities<br>Other non-rheumatic valvular diseases<br>Endocarditis<br>Degenerative mitral disease<br>Calcific aortic disease
**Primary myocardial disease**	Myocarditis

	Other cardiomyopathy
**Toxins**	Alcoholic cardiomyopathy<br>Cocaine use disorders<br>Amphetamine use disorders<br>Chagas
**Stress cardiomyopathies**	Thyroid disease<br>Thalassaemias<br>G6PD deficiency<br>Other haemoglobinopathies and haemolytic anaemias<br>Other cardiovascular and circulatory disorders<br>Atrial fibrillation<br>Chronic kidney disease<br>Cirrhosis and other chronic liver diseases<br>Acute stroke (subarachnoid haemorrhage, intracerebral haemorrhage, ischaemic)<br>Chronic stroke (subarachnoid haemorrhage, intracerebral haemorrhage, ischaemic)

Figure 4: 20 most commonly co-occurring diseases with heart failure in Italy hospital data

YLL cause	count
Garbage Code	184505
Hypertensive heart disease	40884
Atrial fibrillation and flutter	30721
Ischemic heart disease	28023
Diabetes mellitus type 2	16934
Chronic obstructive pulmonary disease	14967
Chronic kidney disease	12909
Ischemic stroke	8267
Alzheimer's disease and other dementias	4785
Non-rheumatic calcific aortic valve disease	4460
Urinary tract infections	4138
Other cardiovascular and circulatory diseases	3605
Cirrhosis and other chronic liver diseases	3322
Non-rheumatic degenerative mitral valve disease	3266
Lipoprotein metabolism and other lipidaemias disorders	3201
Obesity	3118
Other lower respiratory infections	3048
Rheumatic heart disease	2664
Peripheral artery disease	2252
Other endocrine, metabolic, blood, and immune disorders	2057

Prevalence estimation

Overall prevalence of AHA/ACC stage C or D heart failure was estimated in DisMod-MR 2.1 using literature data, hospital data, and claims data. We set a prior of no remission and capped excess mortality at 1. All data adjustments were done outside of DisMod-MR 2.1, described above. Coefficients of covariates included in the DisMod-MR 2.1 model of heart failure prevalence are listed in Table 6.

Table 6: Coefficients for covariates included in the DisMod-MR model of the overall prevalence of heart failure

Study covariate	Parameter	Beta	Exponentiated beta
Healthcare Access and Quality Index	Excess mortality rate	–0.008 (–0.008 to –0.008)	0.99 (0.99 to 0.99)

Estimates for the prevalence of heart failure due to Chagas, degenerative mitral valve disease, and calcific aortic valve disease were generated separately because the methods for those diseases explicitly estimate the burden of heart failure due to those causes. The methods appendix sections for each of these diseases contain additional details. We subtracted the prevalence of heart failure due to these causes from the overall heart failure estimates to give an adjusted prevalence of heart failure due to all other aetiologies.

The GBD estimates separately the prevalence of acute stroke (defined as 30 days or less after the initial stroke) and chronic stroke (defined as 30 days or more after the initial stroke). To estimate the burden of heart failure due to stroke, we similarly needed to provide acute and chronic estimates. Due to the temporal aspect of acute stroke, we estimated the prevalence of heart failure due to this cause separately from others. We reviewed the literature (7) and assumed that 5% of acute strokes resulted in heart failure. Similarly to Chagas, DMVD, and CAVD, we subtracted the prevalence of heart failure due to acute stroke from the overall heart failure estimates to give an adjusted prevalence of heart failure due to all other aetiologies. Heart failure due to chronic stroke was estimated using the same methodology as other aetiologies.

Aetiological fraction estimation

We used data that relate each disease to the risk of heart failure and death to estimate the proportion of heart failure attributable to each cause. To do this, we utilised the epidemiological relationship between prevalence, cause-specific mortality, and excess mortality, described in Equation 1. The prevalence of heart failure due to each aetiology was then scaled into a proportion.

Equation 1:

$$Prevalence_{\ HF\ due\ to\ aetiology} = \frac{Cause\ Specific\ Mortality\ Rate_{\ HF\ due\ to\ aetiology}}{Excess\ Mortality\ Rate_{\ HF\ due\ to\ aetiology}}$$

First, we calculated the cause-specific mortality rate (CSMR) for heart failure due to each aetiology. We used age-, sex-, and location-specific CSMR (post CoDCorrect) for each aetiology, multiplied by the fraction of deaths that also involved heart failure (Equation 2). This fraction was a modelled quantity, informed by person-level vital registry (VR) data from the USA, Mexico, Brazil, Taiwan, and Colombia, data sources available to the GBD study which contained the underlying cause of death as well as all codes in the causal chain. From these sources, we calculated the fraction of underlying deaths from each aetiology in which heart failure was coded in the causal chain. These data were modelled in MR-BRT to generate age- and sex-specific estimates of this proportion. For hypertensive heart disease, alcoholic cardiomyopathy, and other cardiomyopathy, we set the proportion to be 1, as all deaths due to these causes involve heart failure.

Equation 2:

$$CSMR_{HF\ due\ to\ aetiology} = CSMR_{aetiology} * Proportion\ deaths\ with\ HF_{aetiology}$$

Next, we estimated the excess mortality rate (EMR) for heart failure due to each aetiology. We used uniquely identified person-level hospital discharge data for the entire Italian region of Friuli Venezia Giulia, linked to all death records from the region, the only location available to the GBD study with population-level linked data of this kind. Inpatient data contained all primary and non-primary diagnoses associated with the visit, and mortality data contained the underlying cause of death as well as all codes in the causal chain. We identified patients with heart failure due to each aetiology as individuals with hospital-coded heart failure concurrent or after a hospital code of the aetiology. Excess mortality rate for heart failure due to each aetiology was calculated by subtracting the background mortality rate from the mortality rate of persons with heart failure due to that aetiology. We modelled this quantity in MR-BRT to generate age- and sex-specific estimates of this value (Figure 5a and 5b). Due to the small number of deaths in younger ages, we assumed equal EMR across aetiologies for ages under 45.

Figure 5a: Modeled excess mortality for ischaemic heart disease

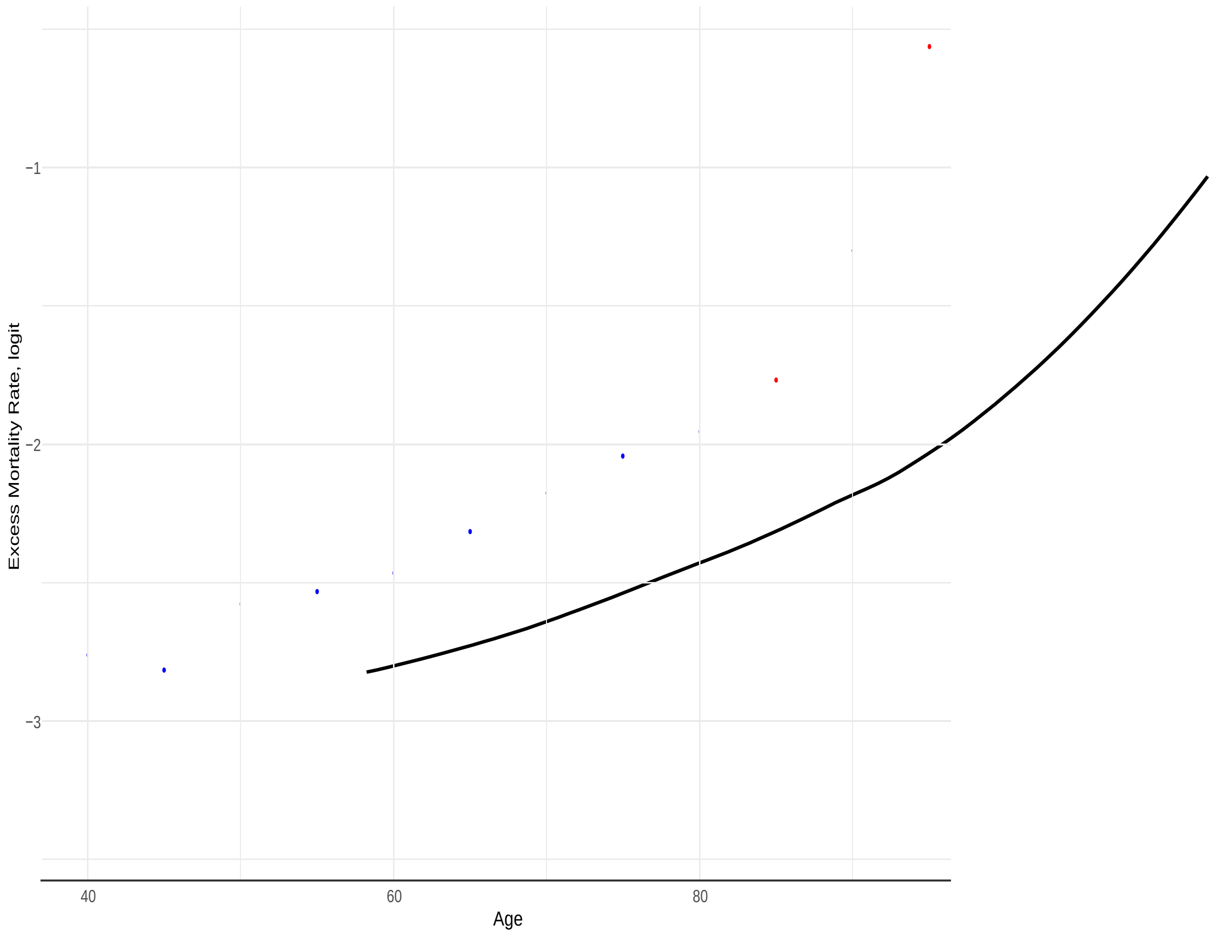

Figure 5b: Modeled excess mortality for chronic obstructive pulmonary disease

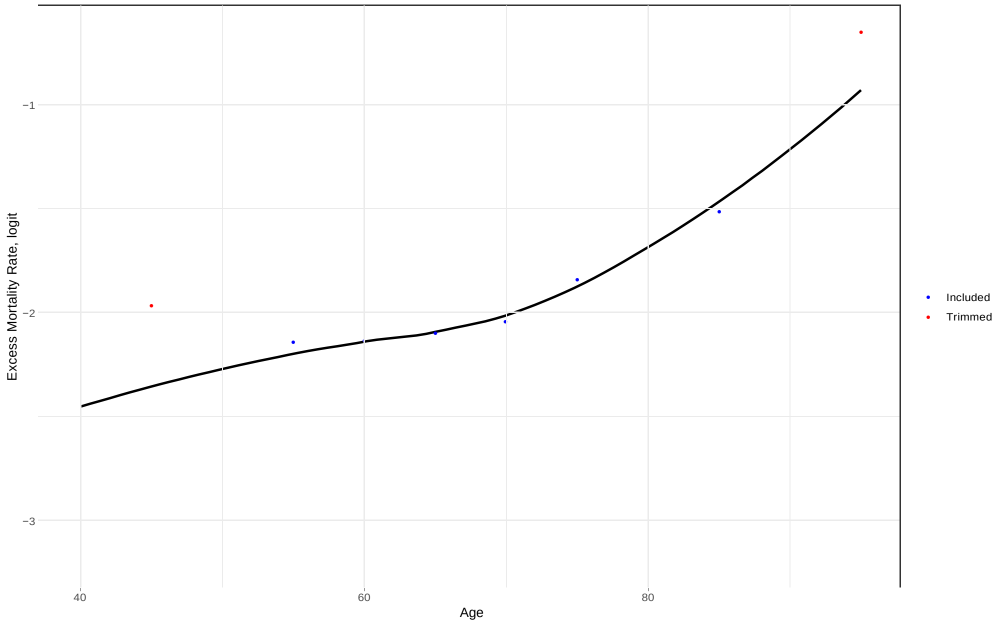

We calculated the prevalence of heart failure due to each aetiology using Equation 1. These were scaled to sum to one, generating the estimated proportions of heart failure due to each aetiology.

These proportions, along with literature data, were used to inform DisMod-MR 2.1 models for the seven broadest and mutually exclusive and collectively exhaustive cause groupings: coronary artery disease, pressure overload of the left heart, pressure overload of the right heart, valve diseases, primary myocardial diseases, toxins, and stress cardiomyopathies (Table 3). An exception to this approach was made for sub-Saharan Africa, where we excluded the proportion estimates generated from death data, relying instead on published literature to determine the proportions of heart failure aetiologies. This decision was based on expert opinion that local patterns differed significantly from what would have been determined from death data and the unique availability of the THESUS-HF study, a large-scale, prospective, echocardiographic study of heart failure aetiologies in multiple African countries, which provided these proportions (8). Table 7 shows the coefficients for the covariates included in the DisMod models for the seven main sub-cause proportion envelopes.

Table 7: Coefficients for covariates included in the DisMod-MR models for the seven main sub-cause proportion envelopes

Sub-cause	Covariate	Parameter	Beta	Exponentiated beta
Heart failure due to coronary artery disease	Log-transformed age-standardised SEV scalar: IHD	Proportion	0.75 (0.75–0.75)	2.12 (2.12–2.12)
Heart failure due to pressure overload of the left heart	Systolic blood pressure (mmHg)	Proportion	7.2E-5 (2.4E-6 – 2.5E-4)	1.00 (1.00–1.00)

Heart failure due to pressure overload of the right heart	Log-transformed age-standardised SEV scalar: COPD	Proportion	0.75 (0.75–0.75)	2.12 (2.12–2.12)
Heart failure due to primary myocardial disease	Log-transformed age-standardised SEV scalar: CMP	Proportion	0.75 (0.75–0.75)	2.12 (2.12–2.12)
Heart failure due to valve diseases	Log-transformed SEV scalar: other cardiovascular	Proportion	0.76 (0.75–0.77)	2.13 (2.12–2.16)
Heart failure due to stress cardiomyopathies	Log-transformed age-standardised SEV scalar: CVD	Proportion	0.75 (0.75–0.76)	2.12 (2.12–2.13)
Heart failure due to toxins	Age-standardised SEV for alcohol use	Proportion	1.25 (1.25–1.25)	3.49 (3.49–3.49)

The results of these seven proportion models were scaled to sum to one.

After scaling the seven proportion models to one, we scaled each aetiology within its respective proportion model, with the exception of heart failure due to coronary artery disease and pressure overload of the left heart, which each represent one aetiology. Figure 6 shows the global results of these proportion models in 2020, including all aetiologies.

Figure 6: Global proportion of heart failure due to each aetiology, 2020

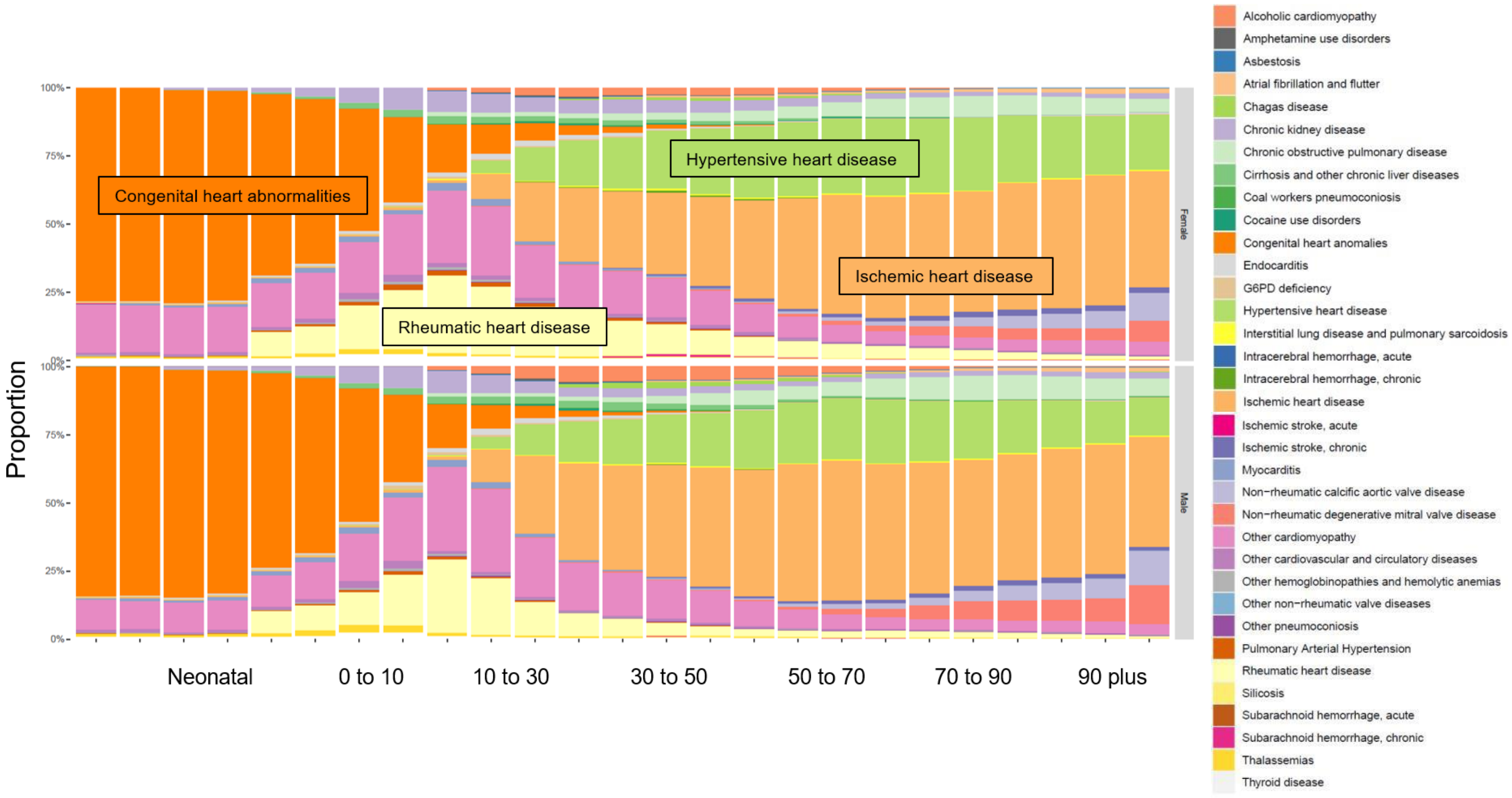

Models were evaluated based on expert opinion, comparison of results with other rounds of GBD, and model fit.

Limitations

Our estimation of the aetiological causes of heart failure makes several assumptions and has several limitations. First, we assume that each case of heart failure only has one cause. While comorbidity, or heart failure resulting from confluent causes, is possible, this assumption was made to improve the utility of the estimates and understand the most common and clinically relevant drivers of heart failure. Second, we rely on individually linked inpatient and mortality records from a small region of Italy to calculate aetiology-specific EMR. The framework allows us to augment with more locations when they become available. Third, we rely on multiple cause of death VR data from five countries to inform use the proportion of deaths that contain heart failure in all countries. This approach allows us to produce estimates for all locations and can similarly be updated to include more detailed health record and claims data from additional locations as they become available.

References

1) http://www.framinghamheartstudy.org/share/protocols/soe0_03s_protocol.pdf
2) 2016 ESC Guidelines for the diagnosis and treatment of acute and chronic heart failure: The Task Force for the diagnosis and treatment of acute and chronic heart failure of the European Society of Cardiology (ESC) Developed with the special contribution of the Heart Failure Association (HFA) of the ESC. Eur Heart J 2016; 37 (27): 2129-2200.

3) Sabino EC, Ribeiro AL, et al, Ten-year incidence of Chagas cardiomyopathy among asymptomatic Trypanosoma cruzi-seropositive former blood donors. Circulation. 2013 Mar 12;127(10):1105-15.
4) Ribeiro AL et al. Brain natriuretic peptide and left ventricular dysfunction in Chagas disease. Lancet. 2002 Aug 10;360(9331):461-2.
5) Ribeiro AL, personal communication. NYHA Class Information in a Cohort of Chagas dilated cardiomyopathy; Hospital das Clínicas da UFMG.
6) Ribeiro AL, personal communication. Cohort of patients attending the Chagas' disease outpatient clinic of Evandro Chagas Hospital (Oswaldo Cruz Foundation, Rio de Janeiro, Brazil).
7) Norberg, Erik, et al. "Impact of Acute Cardiac Complications After Subarachnoid Hemorrhage on Long-Term Mortality and Cardiovascular Events." *Neurocritical Care*, vol. 29, no. 3, 2018, pp. 404–412. doi:10.1007/s12028-018-0558-0.
8) Damasceno A, Mayosi BM, Sani M, Ogah OS, Mondo C, Ojji D, Dzudie A, Kouam CK, Suliman A, Schrueder N, Yonga G, Ba SA, Maru F, Alemayehu B, Edwards C, Davison BA, Cotter G, Sliwa K. The Causes, Treatment, and Outcome of Acute Heart Failure in 1006 Africans From 9 Countries. Results of the Sub-Saharan Africa Survey of Heart Failure. Arch Intern Med. 2012;172(18):1386-1394.

HIV/AIDS

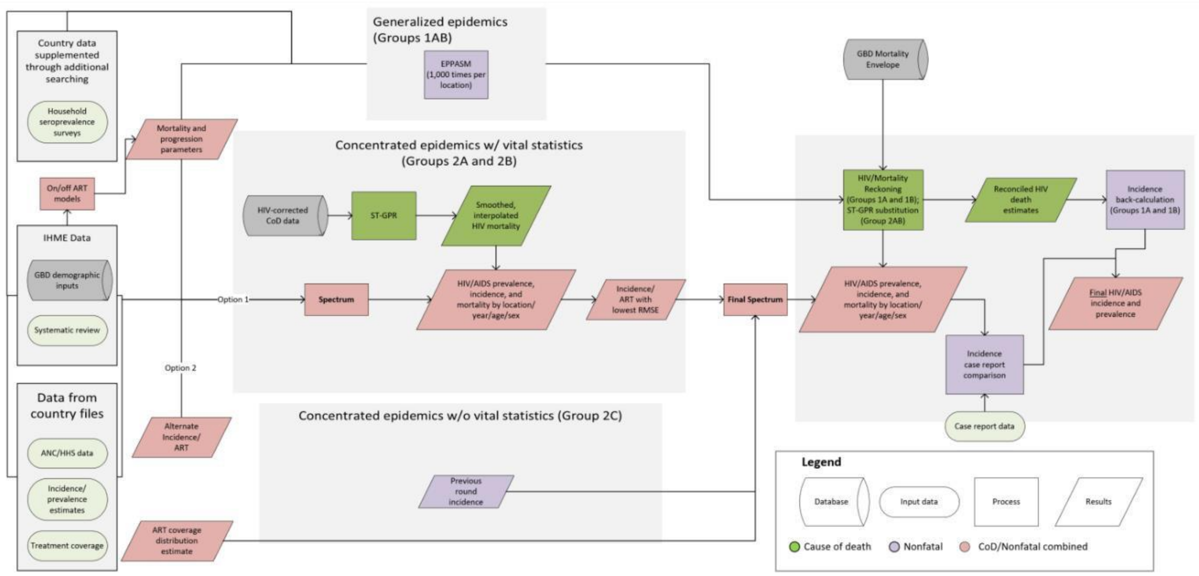

Case definition

Infection with the human immunodeficiency virus (HIV) causes influenza-like symptoms during the acute period following infection and can lead to acquired immunodeficiency syndrome (AIDS) if untreated. HIV attacks the immune system of its host, leaving infected individuals more susceptible to opportunistic infections like tuberculosis. Although there are two different subtypes of HIV, HIV-1 and HIV-2, no distinction is made in our estimation process or presentation of results. For HIV, ICD-10 codes are B20-B24, C46-C469, D84.9; ICD-9 codes are 042-044, 112-118 (after 1980), 130 (after 1980), 136.3-136.8 (after 1980), 176.0-176.9 (after 1980), 279 (after 1980); and ICD-9 BTL codes are B184-B185.

Input data

Household seroprevalence surveys

Geographically representative HIV seroprevalence survey results were used as inputs to the model for countries with generalised HIV epidemics where available.

GBD demographic inputs

Location-specific population, fertility, migration, and HIV-free survival rates from GBD 2021 were used as inputs in modelling all locations.

Data from countries

The files compiled by UNAIDS for their HIV/AIDS estimation process were one of our sources of data for producing estimates of HIV burden. The files are often built by within-country experts with the support of UNAIDS, which publishes estimates annually on behalf of countries and only shares their files when permission is granted. The files contain the HIV-specific information which is needed to run the Spectrum,[1] and Estimation and Projection Package-Age Sex[2] (EPP-ASM) models.

Spectrum and EPP-ASM require the following input data: AIDS mortality among people living with HIV with and without ART, CD4 progression among people living with HIV not on ART, ART coverage among adults and children, cotrimoxazole coverage among children, coverage of breastfeeding among women living with HIV, prevention of mother-to-child transmission coverage, and CD4 thresholds for treatment eligibility. EPP-ASM additionally uses HIV prevalence data from surveillance sites and representative surveys. Antenatal care (ANC), incidence, prevalence, and treatment coverage data from UNAIDS were used in modelling for all locations. We extracted all of these data from the proprietary format used by UNAIDS.

Changes for GBD 2021

We supplemented the antenatal care and treatment coverage data available through processing done by the Local Burden of Disease team,[3] and retrieving data on adult antiretroviral (ART) treatment coverage rates from country reports, respectively. The addition of ANC sites affected 33 countries, while ART data were added in 45 countries. During the Local Burden of Disease alignment process, the antenatal care clinic prevalence estimates were corrected in a number of facets. There were 17 estimates with placeholder sample sizes that were corrected, duplicate observations in Togo were removed, 123 additional observations were added, 1491 non-ANC observations were removed, and 232 points were outliered based on comparison reports of HIV burden in a given area.

We did not have country UNAIDS files for 40 locations, many of them countries with small populations and/or low HIV prevalence. As in previous rounds, we generated regional averages of all needed inputs in these locations. This enabled us to run Spectrum for every GBD location.

Vital registration data

We used all available sources of vital registration and sample registration data from the GBD Causes of Death database after garbage code redistribution and HIV/AIDS mis-coding correction[4] in Group 2 countries and India. There are two different cause of death data sources for HIV/AIDS in China: the Disease Surveillance Points (DSP) system and the Notifiable Infectious Disease Reporting (NIDR) system. Both systems are administered by the Chinese Center for Disease Control and Prevention, but the

reported number of deaths due to HIV is significantly lower in DSP. Therefore, we have used the provincial-level ratio of deaths due to HIV/AIDS from NIDR to those from DSP, choosing the larger ratio between years 2013 and 2014, and scaled the reported deaths in the DSP system, which is in turn used in the spatiotemporal Gaussian process regression (ST-GPR).

Case notifications data

We searched for case notifications data using the ECDC database and country reports series in countries with four- and five-star vital registration data. We identified 59 countries with available information.

On-ART literature data

Data were identified by using search string: "hiv"[MeSH Terms] OR "hiv"[All Fields]) AND ("mortality"[Subheading] OR "mortality"[All Fields] OR "mortality"[MeSH Terms]) AND antiretroviral[All Fields] AND ("therapy"[Subheading] OR "therapy"[All Fields] OR "therapeutics"[MeSH Terms] OR "therapeutics"[All Fields]) in PubMed.

To be included, studies must include only HIV-positive people over the age of 15 who receive antiretroviral therapy (ART) but who were ART-naïve prior to the study. In addition, studies must report either a duration-specific (time since initiation of ART) mortality proportion or a hazard ratio across age or sex, and must not include children.

For duration-specific survival data, studies must report uncertainty on mortality estimates or provide stratum-specific sample sizes and must include duration-specific data to allow for calculation of 0–6, 7–12, or 13–24-month conditional mortality. In addition, studies must either report separate mortality and loss-to-follow-up (LTFU) curves, be corrected for LTFU using vital registration data or double sampling or be conducted in a high-income setting. Finally, studies must report the percentage of participants who are male and the median age of participants.

Hazard ratio data for ages or sexes can only be used if the hazard ratios are controlled for other variables of interest (age, sex, and CD4 category). In GBD 2021, we included 61 studies, 13 of which were new this cycle. Of these studies, we added ten to inform the estimation age-sex hazard ratios, and three studies informed LFTU curves.

PRISMA flow diagram for GBD 2021 on-ART systematic review

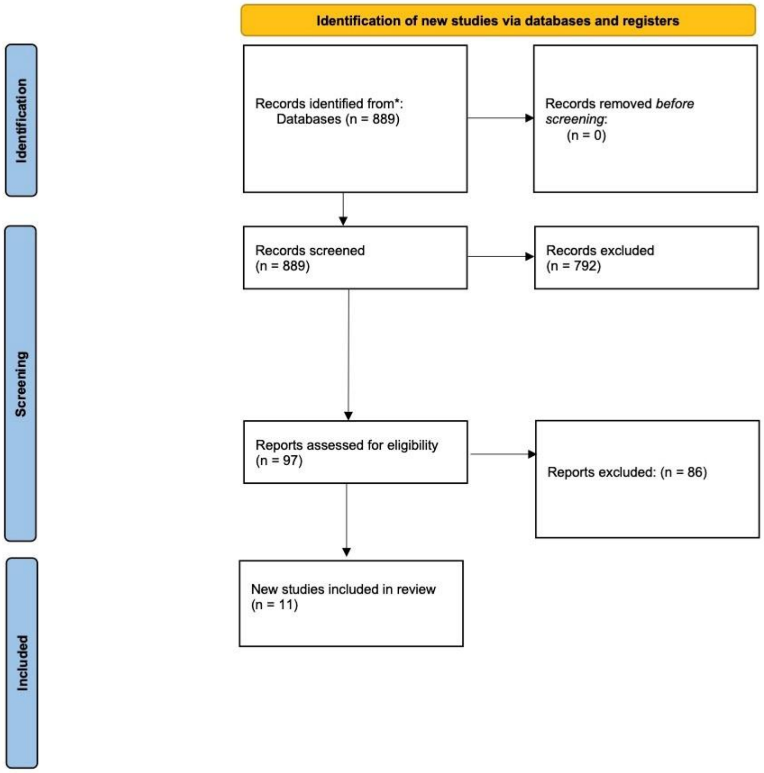

*Note: This systematic review was an update to the GBD 2019 review and doubled as a historical review of sources to capture previously missed studies. As a result, the number of sources being reviewed and excluded are ongoing.

From: Page MJ, McKenzie JE, Bossuyt PM, Boutron I, Hoffmann TC, Mulrow CD, et al. The PRISMA 2020 statement: an updated guideline for reporting systematic reviews. BMJ 2021;372:n71. doi: 10.1136/bmj.n71

For more information, visit: http://www.prisma-statement.org/

Off-ART literature data

In GBD 2013, we systematically reviewed the literature on mortality without ART to characterise uncertainty in the progression and death rates. We searched terms related to pre-ART or ART-naive survival since seroconversion.[5] After screening, we identified 13 cohort studies that included the cohorts used by UNAIDS, from which we extracted survival at each one-year point after infection. Screening for additional, recently published studies in GBD 2015, GBD 2016, and GBD 2017 identified no new cohort studies for inclusion in this analysis. We did not search for new studies in GBD 2019 or GBD 2021.

Severity splits and disability weights

The basis of the GBD disability weight survey assessments are lay descriptions of sequelae highlighting major functional consequences and symptoms. The lay descriptions and disability weights for HIV/AIDS severity levels are shown below.

**Severity level**	**Lay description**	**DW (95% CI)**

Symptomatic HIV	Has weight loss, fatigue, and frequent infections.	0.274 (0.184–0.377)
AIDS with antiretroviral treatment	Has occasional fevers and infections. The person takes daily medication that sometimes causes diarrhoea.	0.078 (0.052–0.111)
AIDS without antiretroviral treatment	Has severe weight loss, weakness, fatigue, cough and fever, and frequent infections, skin rashes, and diarrhoea.	0.582 (0.406–0.743)

Modelling strategy

Countries were divided into groups: Groups 1A and 1B, and 2A, 2B, and 2C.

Group 1 includes countries with HIV prevalence data from antenatal clinics or nationally- or subnationally-representative population-based seroprevalence surveys. Group 1A included countries with a peak of at least 0.5% prevalence, and Group 1B includes countries with a peak of at least 0.25% prevalence and vital registration completeness less than 65%.

The remaining countries made up Group 2,which are further subdivided in Group 2A, 2B and 2C based on availability of vital registration data. Group 2A consisted of countries with high-quality vital registration data; Group 2B consisted of countries with any available vital registration data, which was generally lower-quality; and Group 2C countries were those without any vital registration data. Quality was measured based on a star rating system as described elsewhere.[6]

On-ART

First, we corrected reported probabilities of death for loss to follow-up using an approach developed by Verguet and colleagues.[6] Verguet and colleagues used tracing and follow-up studies to empirically estimate the relationship between death in loss to follow-up (LTFU) and the rate of LTFU.
To create estimates of age-specific hazard ratios, we synthesised hazard ratio data in five broad age groups: 15–25, 25–35, 35–45, 45–55, and 55–100, and modelled the data using DisMod-MR 2.1.

To create estimates of sex-specific hazard ratios, we use the *metan* function in Stata to create estimates of relative risks separately by region, using female as the reference group.
The age and sex hazard ratios were applied to the study-level mortality rates, accounting for the distribution of ages and sexes in the mortality data. We then subtracted HIV-free mortality from the model life table process to calculate study-level age-sex HIV-specific mortality.

Changes for GBD 2021
To synthesise the age-sex-split study-level data into estimates of conditional probability of death over initial CD4 count for GBD 2021, we replaced the use of DisMod[6] in favour of the meta-regression—Bayesian, regularised, trimmed (MR-BRT) model.[7] This model is a mixed effects meta-regression that

accounts for between-study heterogeneity. We ran MR-BRT models for each age group (15–25, 25–35, 35–45, 45–55, or 55–100), sex (male or female), duration since ART initiation (0–6, 7–12, or 13–24 months) and super-region (sub-Saharan Africa, high-income, or other) strata.

Off-ART

Following UNAIDS assumptions, no-ART mortality is modelled as shown in the figure below.

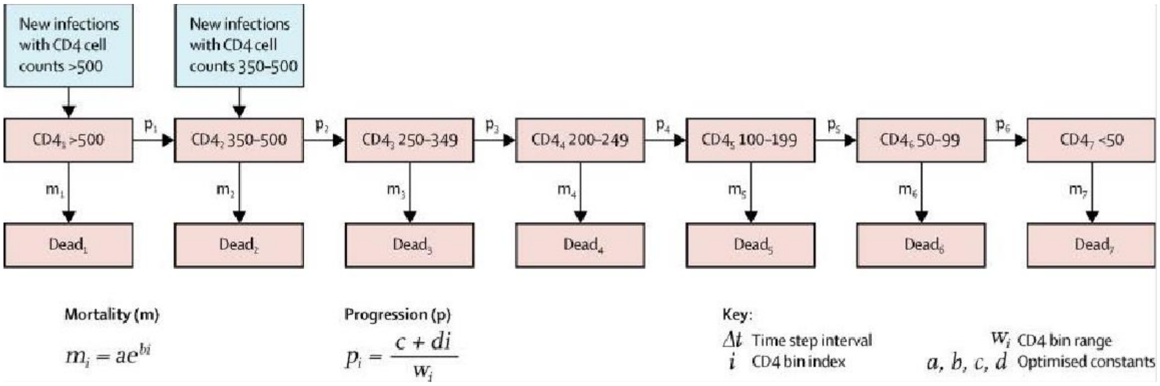

The death and progression rates between CD4 categories vary by age according to four age groups: 15–24 years, 25–34 years, 35–44 years, and 45 years or older. We modelled the logit of the conditional probability of death between years in these studies using the following formula:

$$logit(m_{ijk}) = \beta_0 + \sum_{i=1}^{4} \beta_{1i}a_i + \sum_{j=1}^{12} \beta_{2j}t_j + u_k + \epsilon_{ijk}$$

In the formula, *m* is conditional probability of death from year t_j to t_{j+1}, a_i is an indicator variable for age group at seroconversion (15–24 years, 25–34 years, 35–44 years, and 45 years or older), t_j is an indicator variable of year since seroconversion, and u_k is a study-level random effect.

By sampling the variance-covariance matrix of the regression coefficients and the study-level random effect, we generated 1000 survival curves for each age group that capture the systematic variation in survival across the available studies. For each of the 1000 survival curves, we used a framework modelled after the UNAIDS optimisation framework in which we find a set of progression and death rates that minimises the sum of the squared errors for the fit to the survival curve.[8,9]

Finally, in cases where estimated on-ART mortality rates were higher than off-ART mortality rates, we replaced our estimated on-ART mortality rates by the corresponding off-ART mortality rates to account for progression to lower CD4 categories. This ensured individuals would not experience higher mortality when they entered treatment in Spectrum or EPP-ASM.

GBD 2021 HIV burden estimation overview

We used two different components to derive year-, age- and sex-specific estimates of HIV incidence, prevalence, and mortality depending on locations' availability of data and extent of HIV burden, as described below:

1. **EPP-ASM was used to estimate incidence, prevalence, and mortality that are consistent with serosurveillance data from antenatal care clinics and/or prevalence surveys.**

Spectrum is a compartmental HIV progression model used to generate age-sex-specific incidence, prevalence, and death rates from input incidence and prevalence curves and assumptions about intervention scale-up and local variation in epidemiology. This model was used for all Group 2 countries, and in conjunction with EPP-ASM for India.

EPP-ASM model

For GBD 2021, we continued to use our modified version of EPP-ASM both to improve the fit to age-sex-specific prevalence survey data among adults and to generate paediatric estimates. We built a paediatric module in EPP-ASM that mirrored early updates to the paediatric module in Spectrum.[10] This child module included CD4 progression and CD4-specific mortality rates taken from a model fit to survival data from IeDEA and child initiation of ART based on ART distribution data from IeDEA. Perinatal and breastfeeding transmission was calculated as a function of prevalence among pregnant women and PMTCT programme data. We were thus able to utilise EPP-ASM to produce HIV incidence, prevalence, and mortality estimates for all ages. Additionally, we improved fit to prevalence data through allowing flexibility in the age distribution of incidence over time. We parameterised the ratio of incidence among ages 15–24:25+ as a constant before year 2000 and a linear regression thereafter. This allowed for the shifts in the age distribution of incidence observed over the course of the HIV epidemic to be reflected in our results. Finally, we utilised GBD demographic inputs and substituted in our own assumptions about HIV progression rates and on/off-ART mortality.

To incorporate uncertainty in our demographic and progression parameters, we ran EPP-ASM with separate draws of CD4 progression, on- and off-ART mortality rates, fertility, and HIV-free mortality. This process produced 1000 posterior distributions for each of the locations that make up Group 1. For every location in the group, we sampled one draw from each of the sets of EPP-ASM results to create a final distribution of 1000 draws. By sampling one draw from each set, we ensured that the distribution of mortality parameters dictating the relationship between incidence and prevalence aligned with those used in the GBD demographics estimates.

We also continued to use the approach implemented in GBD 2019 to address selection bias resulting from temporal and geographical variation in ANC reporting. The ANC data which EPP-ASM uses cannot be assumed as representative of HIV prevalence in the full population. This is especially the case when there are minimal or no nationally representative prevalence surveys to anchor estimates, as in the early epidemic.[11]

EPP-ASM has embedded approaches to adjust for the bias associated with using prevalence among ANC-site-attending pregnant women to estimate prevalence among the both-sexes population. For the bias between pregnant women and the national both-sexes population, it makes assumptions around the difference in total fertility rate among HIV-positive and HIV-negative women, and the difference in prevalence between men and women. For the bias associated with the data coming from ANC sites, the specification of the likelihood of observed ANC data includes random intercepts for each clinic. The

random intercepts allow each site's baseline prevalence to vary randomly around the overall mean prevalence. In other words, factors that could drive differences between sites' HIV prevalence levels are "adjusted" for.

However, the embedded approach does not explicitly account for the fact that the location of the clinic in space may also drive its HIV prevalence level. For example, we might expect rural sites to be more correlated than urban sites. Thus, to further adjust for this bias, we used an offset term that represents the difference in the prevalence among the national, both-sexes population and the prevalence among the female, pregnant population associated with an ANC site location. The offset term was derived for each location as the difference between the adjusted prevalence in a given site-year and the adjusted national prevalence in that year. These estimates are adjusted for covariates that are thought to influence prevalence, for example, access to health-care facilities, malaria incidence, and male circumcision.

Thus, our final strategy for estimating the likelihood of the observed ANC data was:

$$\boldsymbol{W_{st} = \varphi^{-1}(\rho_t) + \vartheta_{st} + u_s + e_{st}}$$

$$\boldsymbol{e_{st} \sim N(0, \sigma_{st}^2)}$$

$$\boldsymbol{u_s \sim N(0, \sigma_s^2)}$$

Where:

W_{st} = the probit transformed prevalence among ANC-attending pregnant women at site *s* and time *t*
ρ_t= the national prevalence adjusted to represent prevalence among pregnant women from the model simulation
ϑ_{st} = the offset term representing the difference between the adjusted prevalence in a given site-year and the adjusted national prevalence in that year
φ^{-1} = probit transformation
e_{st} = site-year-specific error term
u_s = site-specific intercept

Spectrum

For GBD 2013, we created an exact replica of Spectrum in Python. This enabled us to run thousands of iterations of the model at once on our computing cluster and allowed for more flexible input data structures. Additionally, we scaled all input values by a uniformly sampled factor between 0.9 and 1.1 to generate estimates with realistic ranges of uncertainty. For example, if treatment retention rates across CD4 categories were 0.906, 0.759, 0.787, 0.795, 0.785, 0.756, 0.813, and 0.700, we multiplied each number by an array of equivalent size that contained factors ranging from 0.9 to 1.1. At each draw, the array would contain different, randomly selected factors in the same range. Further, we previously improved our sex-specific modelling strategy in Spectrum by sex-splitting incidence based on a model fit to the sex ratio of prevalence observed in countries with representative surveys and updated the Spectrum paediatric module to reflect changes made by UNAIDS.[10] Our child module was revised to include CD4 progression and CD4-specific mortality rates taken from a model fit to survival data from IeDEA. Finally, we updated child initiation of ART to include data on ART distribution from IeDEA. These changes were retained in GBD 2021.

ART coverage distribution

Spectrum determines the number of people initiating ART treatment across each CD4 category based on eligibility criteria, and the number of expected deaths and untreated people. In other words, groups with a large proportion of people living with HIV and high numbers of expected deaths initiated the most individuals into treatment.

We improved the basis for this distribution using survey microdata and country-level wealth information. Three relevant surveys were available: Uganda AIS 2011 and Kenya AIS 2007 and 2012. These surveys conducted CD4 count measurements and include a question regarding the amount of time that an individual receiving ART had been enrolled in treatment. Survey data provide cross-sectional CD4 count information; however, the Spectrum modelling framework tracks individuals by categorical CD4 count at the initiation of treatment. To crosswalk the cross-sectional survey data into estimates of CD4 count at treatment initiation, we built a model using relevant cohort data which tracked changes in CD4 count after initiation of treatment to translate an individual's current CD4 count and duration on treatment into CD4 count at initiation of treatment. The functional form for changes in CD4 count as a function of duration on treatment was a natural spline on duration with knots at 3, 12, 24, and 36 months, and an interaction between initial CD4 count and duration.

After crosswalking, we predicted the probability of being on treatment as a function of individual income (measured through an asset-based index), stratified by CD4 count, age, and sex. The results of this prediction were translated into country-specific age-sex-year-CD4 count probabilities of coverage using a conversion factor between individual income and lag-distributed GDP per capita. We used stochastic frontier analysis to constrain the maximum possible coverage for a given degree of income and CD4 count.

Predicted probabilities of coverage were input to Spectrum to inform the distribution, and not the overall level, of ART treatment by CD4 count. Within Spectrum, the probabilities of coverage are converted to counts of expected individuals on treatment in each CD4 count group. These are scaled to the distribution across CD4 count groups to match the input data on the number of people on ART coming from UNAIDS country files. In cases where the predicted number of individuals initiating treatment exceeds the total number of untreated individuals in a CD4 count group, we reallocate treatment evenly to other CD4 count groups.

Countries with seroprevalence surveys and antenatal clinic data (Groups 1ABC)

53 countries – as well as subnational locations in India, Kenya, Ethiopia, Nigeria, and South Africa – were included in Group 1 with available antenatal care clinic (ANC) data and/or least one geographically representative HIV seroprevalence survey. For all these locations we used EPP-ASM, which was updated to incorporate the new ANC bias adjustment.

In EPP-ASM, the transmission rate, r(t), is a simple transmission model applied at each time step (1/10 of a year) to the population. 'r' represents the number of new cases expected to emanate from a single case. Over 3000 iterations, a new r(t) is drawn, the full epidemic is determined and compared to the observed prevalence data to determine its likelihood. Beyond the end of the data, a prior distribution on r(t) helps to determine how we should expect the epidemic to behave. This assumption was different in EPP-ASM versus EPP. In EPP-ASM in most countries, we extended a random walk into the future based

on the ‘r-hybrid’ r(t). The r-hybrid assumes a logistic decay until the year 2003, a linear interpolation until year 2008, and a random walk form after this.

Changes for GBD 2021

For India, Comoros, São Tomé and Príncipe, and Mauritania (new Group 1B countries), we used EPP-ASM to model HIV burden for GBD 2021. For India, we used EPP-ASM in combination with Spectrum, to be able to capitalise on SRS data. The SRS data were used to inform age and sex distribution. In addition, we used an ‘equilibrium prior,’ for r(t) rather than ‘r-hybrid’ for India, which provided a better fit to the comparatively lower magnitude of the epidemic.[12] The equilibrium prior extends into the future with a rate of change following a normal distribution with a mean equal to the value of r expected when the proportion of the population infected is saturated, ie, the epidemic has stabilised.

When age-sex-specific prevalence data included a zero proportion (no observed positive HIV tests), a binomial likelihood was used in place of the normal likelihood. Prior, imputation of a half positive observation was used to allow for probit transformation. This improved the fit to the zero proportion data while minimally impacting fits in non-zero prevalence age-sex strata. South Africa, India, Kenya, Gambia, Niger, Burundi, Ethiopia, Rwanda, Ghana, São Tomé and Príncipe, Senegal, and Sierra Leone were affected by this change.

The HIV/mortality reckoning process is intended as a method of reconciling separate estimates of HIV mortality (and its resulting effect on estimates of HIV-free and all-cause mortality) in Group 1 countries by averaging estimates of HIV mortality from the model life table process and EPP-ASM. Additional details on the reckoning can be found elsewhere.[13]

Since EPP-ASM produces HIV incidence, prevalence, and deaths that are consistent with one another over time, the reckoning process results in death numbers that are no longer consistent with the incidence and prevalence produced in Spectrum. To recreate this consistency, we recalculated incidence for all Group 1 locations using reckoned deaths and prevalence produced by EPP-ASM. The updated incidence is calculated by aggregating counts of new infections, HIV deaths from EPP-ASM, and HIV deaths after reckoning at the year-sex level. The difference between reckoned HIV deaths and HIV deaths from EPP-ASM is added to EPP-ASM incidence, and we calculate the ratio between updated incidence and EPP-ASM incidence. Age-specific counts of new infections are then scaled by their corresponding sex-year ratios.

Countries with vital registration data (Group 2A and 2B)

Vital registration is one of the highest-quality sources of data on HIV burden in many countries, so generating estimates that are consistent with these data with necessary adjustment to account for any potential under-reporting is critical. We identified 121 countries – as well as 760 subnational locations from China, Japan, Indonesia, India, Mexico, Sweden, the Philippines, Poland, Italy, the UK, Ukraine, Russia, New Zealand, Iran, Norway, and the USA – with usable points of vital registration data, verbal autopsy (VA) data, or sample registration system (SRS) data. In India, Vietnam, and Indonesia, we used SRS and VA data, respectively, as input mortality for CIBA. For India, we extracted the CIBA-derived age-

sex distribution of incidence but scaled the level to match the adult incidence rate estimated from EPP for each state.

We imputed missing years of data to generate a complete time series for HIV from the estimated start year of the epidemic using ST-GPR. We analysed mortality trends using ST-GPR starting in 1981, the year that HIV was first identified in the USA.[13] For ST-GPR, we adjusted the lambda (time weight) and GPR scale according to the completeness of vital registration data, with 4- and 5-star quality VR using parameters designed to follow the data more closely. Separately by country/age group, we fit a piecewise linear spline with a single knot located at the empirical peak year of death rate using robust regression. The model includes fixed effects on region, age, and sex. Following this, we ran space-time residual smoothing, in which time, age, and space weights are used to inform smoothing of the residuals between datapoints and the linear regression estimate. From this process, we generated space-time estimates with the applied weights, along with the median absolute deviation (MAD) of the space-time estimates from the data. The MAD was calculated at various levels of the geographical hierarchy (eg, subnational and national), and was added into the data variance term. The data variance and space-time estimates were then analysed using Gaussian process regression to return a final estimate of mortality along with uncertainty.

Although Spectrum produces HIV mortality estimates that are within the realm of possibility in most countries using the incidence curves provided in the UNAIDS country files, it is a deterministic model that has not yet been integrated into an optimisable framework. Therefore, in order to "fit" it to vital registration data, we need to adjust input incidence. In contrast to GBD 2019 and previous cycles, in addition to adjusting input incidence, we determined the most plausible best treatment input based on fit to vital registration as well.

Changes for GBD 2021

For GBD 2021, we then created a grid of incidence and treatment options and reran Spectrum for each using each of these options, rather than using the CIBA-adjusted incidence for our final Spectrum run in all locations. The incidence options included the CIBA-adjusted incidence and the non-CIBA adjusted incidence from the initial Spectrum run, both using the most recent data and the last cycle, in addition to incidence data available from public-use UNAIDS files. The CIBA process is described in more detail in the GBD 2019 writeup.[6] The adult ART options included the data available from public-use UNAIDS files. Where these data were provided in terms of the number of people on treatment, we created additional treatment options by dividing the number on treatment by prevalence, as estimated by the current and previous GBD cycles. We ran Spectrum on every combination of incidence and treatment options, and then determined the root mean squared error of the resulting mortality relative to the vital registration data.

Finally, to produce location-, year-, age-, and sex-specific estimates of HIV incidence, prevalence, and mortality, we ran a final Spectrum run using the incidence and treatment option that resulted in the best fit to VR data, or the lowest RMSE.

Adjustment to case notification data

To estimate final incidence, we scaled the Spectrum output incidence up to the level of observed case notifications data, with a five-year lag to account for the difference between infection and detection.

This was done in countries with 4- and 5-star vital registration systems, with available case notification data.

Countries without prevalence or deaths data (Group 2C)

32 countries had neither geographically representative seroprevalence surveys nor reliable vital registration systems. To produce estimates of HIV burden in these countries, we used Spectrum to produce estimates of burden. As above, the estimates of incidence, prevalence, and mortality were incorporated into the rest of the machinery via the reckoning process.

Changes for GBD 2021
Group 2C countries no longer sampled bias adjustment ratios from other Group 2 countries within the same super-region.

References

1 Stover J, Glaubius R, Mofenson L, *et al.* Updates to the Spectrum/AIM model for estimating key HIV indicators at national and subnational levels. *AIDS Lond Engl* 2019; **33**: S227–34.

2 Eaton JW, Brown T, Puckett R, *et al.* The Estimation and Projection Package Age-Sex Model and the r-hybrid model: new tools for estimating HIV incidence trends in sub-Saharan Africa. *AIDS Lond Engl* 2019; **33 Suppl 3**: S235–44.

3 Dwyer-Lindgren L, Cork MA, Sligar A, *et al.* Mapping HIV prevalence in sub-Saharan Africa between 2000 and 2017. *Nature* 2019; **570**: 189–93.

4 Kyu HH, Jahagirdar D, Cunningham M, *et al.* Accounting for misclassified and unknown cause of death data in vital registration systems for estimating trends in HIV mortality. *J Int AIDS Soc* 2021; **24**: e25791.

5 Murray CJL, Ortblad KF, Guinovart C, *et al.* Global, regional, and national incidence and mortality for HIV, tuberculosis, and malaria during 1990–2013: a systematic analysis for the Global Burden of Disease Study 2013. *The Lancet* 2014; **384**: 1005–70.

6 Vos T, Lim SS, Abbafati C, *et al.* Global burden of 369 diseases and injuries in 204 countries and territories, 1990–2019: a systematic analysis for the Global Burden of Disease Study 2019. *The Lancet* 2020; **396**: 1204–22.

7 Zheng P, Barber R, Sorensen RJD, Murray CJL, Aravkin AY. Trimmed Constrained Mixed Effects Models: Formulations and Algorithms. *J Comput Graph Stat* 2021; **0**: 1–13.

8 Ghys PD, Zaba B, Prins M. Survival and mortality of people infected with HIV in low and middle income countries: results from the extended ALPHA network. *AIDS Lond Engl* 2007; **21 Suppl 6**: S1-4.

9 Hallett TB, Zaba B, Todd J, *et al.* Estimating incidence from prevalence in generalised HIV epidemics: methods and validation. *PLoS Med* 2008; **5**: e80.

10 MAHY M, PENAZZATO M, CIARANELLO A, *et al.* Improving estimates of children living with HIV from the Spectrum AIDS Impact Model. *AIDS Lond Engl* 2017; **31**: S13–22.

11	Ng M, Gakidou E, Murray CJ, Lim SS. A comparison of missing data procedures for addressing selection bias in HIV sentinel surveillance data. *Popul Health Metr* 2013; **11**: 12.

12	Eaton JW, Brown T, Puckett R, *et al.* The Estimation and Projection Package Age-Sex Model and the r-hybrid model: new tools for estimating HIV incidence trends in sub-Saharan Africa. *AIDS Lond Engl* 2019; **33**: S235–44.

13	Pneumocystis Pneumonia --- Los Angeles. https://www.cdc.gov/mmwr/preview/mmwrhtml/june_5.htm (accessed Feb 4, 2021).

Hookworm disease

Flowchart

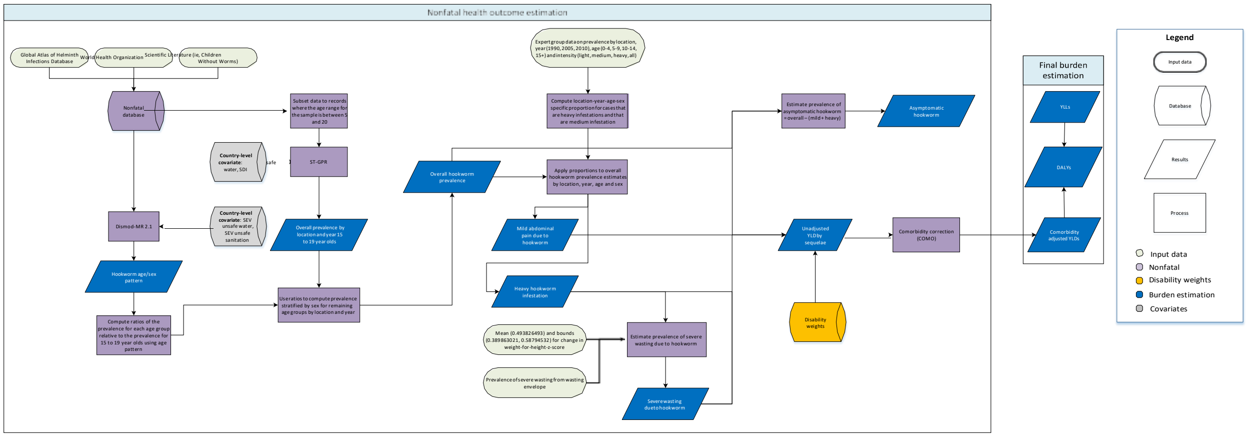

Input data and methodological summary

Case definition

Hookworm disease is a helminthic disease caused by intestinal parasites in the roundworm group, *Ancylostoma duodenale* and *Necator americanus* that can cause abdominal pain, intestinal blood loss leading to anemia, and malnutrition. It is one of the three intestinal nematode infections, or soil-transmitted helminthiases (STH), that we model in GBD. Diagnosis is made by examination of stool by microscope or PCR, with or without concentration procedures. The ICD-10 codes for hookworm disease are B76–B76.9.

We used the following case definition for GBD 2021:

Quantity of interest	Reference or alternative	Definition
Hookworm	Reference	Diagnosis made by examination of stool using Kato-Katz technique, resulting in positive for intestinal helminth eggs of type hookworm.

Input data

The primary input data for this model were from the Global Atlas of Helminth Infections (GAHI) database and the Expanded Special Project for the Elimination of Neglected Tropical Diseases (ESPEN). The GAHI and ESPEN databases include surveys and studies conducted to measure the prevalence of STH.[1] Each record in the database contained metadata (ie, location, year, age range, sex) of each study sample and the prevalence of hookworm in that sample.

We supplemented the GAHI and ESPEN data with survey data collected in a literature review performed by Children Without Worms (2006-2016), which included countries outside of sub-Saharan Africa, and additional data provided by the World Health Organization (WHO). For all input data, we excluded datapoints where the age range of the sample was unknown and retained only those surveys utilising the Kato-Katz diagnostic method.

Table 1: Data inputs for hookworm morbidity modelling by parameter.

Measure	Countries with data	New sources	Total sources
All measures	140	40	208
Prevalence	80	40	207
Proportion	134	0	1

Geographical restrictions

We conducted a literature review (last updated for GBD 2017) to determine the geographical extent of the disease and classify locations based on whether the disease is absent or present in each year. Locations that were geographically restricted in any given year did not have estimates made for them. Of note, we did not attempt a complete systematic review since a single high-quality source could offer sufficient evidence of presence. Evidence of absence or presence was not available for every location for each year. Assumptions made for missing years took into consideration the epidemiological characteristics of the disease.

If evidence indicated disease presence for two non-consecutive years, we assumed presence for all years between the two. If evidence indicated disease absence for two non-consecutive years, we assumed absence for all years between the two. If evidence indicated a change in status (ie, from absent to present, or present to absent) between two non-consecutive years, then we conducted targeted searches to ascertain the relevant year of introduction or elimination for that location. In the cases where presence or absence information was missing for the start or end years of our study interval without evidence of any introduction or elimination events within the interval, we applied the status of the first and last presence/absence observations, respectively, to all years between the interval bound and the observation year. Table 2 shows the search strings and associated yield for each of the databases queried.

Table 2. Geographical restriction search strings

Database	Search string	Yield
PubMed	(Ascariasis[Title/Abstract] OR Ascaris[Title/Abstract] OR "A. lumbricoides"[Title/Abstract] OR Ascaris[MeSH] OR Trichuris[Title/Abstract] OR Trichuriasis[Title/Abstract] OR "Whip Worm"[Title/Abstract] OR "T. trichura"[Title/Abstract] OR Trichuris[MeSH] OR Hookworm[Title/Abstract] OR "A. duodenale"[Title/Abstract] OR "Ancylostoma duodenale"[Title/Abstract] OR ancylostomiasis[Title/Abstract] OR "N. americanus"[Title/Abstract] OR	2376

	"Necator americanus"[Title/Abstract] OR necatoriasis[Title/Abstract] OR Ancylostoma [MeSH] OR Necator[MeSH]) AND (prevalence[Title/Abstract] OR incidence[Title/Abstract] OR epidemiology[Title/Abstract] OR surveillance[Title/Abstract]) NOT(Animals[MeSH] NOT Humans[MeSH])	
Web of Science	(Ascariasis OR Ascaris OR A. lumbricoides OR Trichuris OR Trichuriasis OR Whip Worm OR T. trichura OR Hookworm OR A. duodenale OR Ancylostoma duodenale OR anclyostomiasis OR N. americanus OR Necator americanus OR necatoriasis) AND TOPIC:(prevalence OR incidence OR epidemiology OR surveillance) NOTTOPIC: ((Animals NOT Humans))<br>Timespan: 1980-2016. Indexes: SCI-EXPANDED, SSCI, A&HCI, ESCI.	2266
SCOPUS	TITLE-ABS_KEY (ascariasis OR ascaris OR a. lumbricoides OR trichuris OR trichuriasis OR whip worm OR t. trichura OR hookworm OR a. duodenale OR ancylostoma duodenale OR anclyostomiasis OR n. americanus OR necator americanus OR necatoriasis) AND PUBYEAR>1979	29

These papers classified location-years for all locations and years present in the literature. We only utilised papers that are explicitly concerned with hookworm. Additionally, systematic literature reviews, meta-analyses, national health statistics publications, and collaborator input supported classification of location-years not present in the literature review wherever possible.

Modelling strategy

DisMod-MR

In the estimation of overall morbidity due to hookworm we implemented a three-stage modelling framework. The first stage of the modelling process used a DisMod Bayesian meta-regression model (DisMod-MR), to generate a global age-sex curve to disaggregate all-age, both-sex prevalence data. DisMod-MR is an integrated meta-regression framework that allows multiple datasets to be used within a singular analysis regardless of age-binning, sources, and geographies. As a result, a variety of differently aggregated information combines to generate a consensus output. Our final model contained all processed GAHI data as input informed by two country-level covariates (ie, SEV for unsafe water and unsafe sanitation).

Table 3a. Covariates. Summary of covariates used in the hookworm DisMod-MR model.

Covariate	Type	Parameter	Exponentiated beta (95% UI)
SEV unsafe water	Country-level	Proportion	4.44 (4.36–4.48)
SEV unsafe sanitation	Country-level	Proportion	4.44 (4.36–4.48)

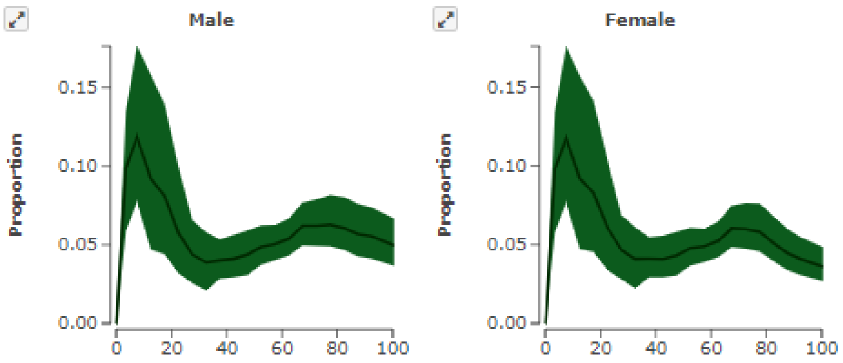

Figure 1: Global age-specific prevalence estimates for males (left) and females (right) for the year 2010. Proportion (prevalence) is on the Y-axis, and age in years on the X-axis. Screenshot from EpiViz tool.

Figure 1 shows the age-specific variation in prevalence rates, differentiated by sex. When considered as a global aggregate, we see that reported male and female prevalence are very similar. Prevalence peaks among young adults, followed by a decline and then stabilising during adulthood. These age-sex curves are similar to what has been reported in the literature.[2,3]

ST-GPR

We then utilise a spatiotemporal Gaussian process regression (ST-GPR) to generate a complete time series of estimates for each location where there are no geographical restrictions. ST-GPR attempts to model non-linear trends utilising a Gaussian process to fit a trend. We ran an age-restricted ST-GPR model, using all data with age bins between 5 and 20 because these data fall within the peak in prevalence across all age groups, the majority of data fall within these age ranges, and these data provide sufficient statistical power for our model. This left us with 280 site-years of input data. The following were the model specifications:

$$Prevalence = Proportion\ Safe\ Water + Sociodemographic\ Index + (1|level\ 2) + (1|level\ 3)$$

Levels 2 and 3 refer to GBD location hierarchies, or random effects for region and location. Notably, the covariates for the model were safe water or proportion of population with access to improved water sources and Socio-demographic Index (SDI). Improved toilet types and improved water sources are defined by the Joint Monitoring Programme.[4] The following hyperparameters were used: st-lambda = 0.25, st-omega = 2, st-zeta = 0.005, gpr-scale = 15. We selected these hyperparameters as they provided more weight to country-level data rather than region-level data when estimating the prevalence for a given location-year. In other words, these hyperparameters ensure that the Gaussian process regressions follow country-specific data rather than region-specific data when estimating a time series for a location.

Table 3b. Covariates. Summary of covariates used in the hookworm ST-GPR model

Covariate	Beta coefficient, log (95% UI)	Standard error	Exponentiated beta (95% UI)
Improved water	–2.490 (–5.028 to 0.048)	1.295	0.083 (0.006–1.049)
SDI	–7.610 (–12.23 to –2.976)	2.364	0.0005 ($4.81*10^{-6}$ – 0.051)

Imputation

The final stage of the overall prevalence modelling process is to impute the remaining age groups by borrowing information from the DisMod-MR global age-sex pattern and ST-GPR time series, by first assuming that the estimates from ST-GPR are representative of the 15–19-year-old age group. Each additional age group is assigned a ratio representing how much larger or smaller the prevalence is compared to the prevalence of the reference group (15–19-year-olds) using the DisMod-MR global age-sex pattern. The following is the computation for each age group:

$$Ratio = \frac{prevalence_{[age\ start] to\ [age\ end]}}{prevalence_{15\ to\ 19}}$$

With a ratio for every age group by sex, we multiplied the ratio by the ST-GPR location-year estimates to impute estimates for the remaining age groups.

Health states/sequelae

The table below shows the list of sequelae due to hookworm and the associated disability weights (DW). Prevalence of medium infection and heavy infection mapped to *mild abdominopelvic problems* and *heavy infestation of hookworm,* respectively. Light infection or asymptomatic were not attributed any disability.

Table 4. Severity distribution, details on the severity levels of hookworm and the associated disability weight (DW) with that severity

Sequela	Lay description	DW (95% CI)
Mild abdominopelvic problems	Has some pain in the belly that causes nausea but does not interfere with daily activities	0.011 (0.005–0.021)
Heavy infestation	Has cramping pain and a bloated feeling in the belly	0.027 (0.015–0.044)
Severe wasting	Is extremely skinny and has no energy	0.128 (0.082–0.183)
Asymptomatic hookworm disease	N/A	N/A
Mild anaemia	Feels slightly tired and weak at times, but this does not interfere with normal daily activities	0.004 (0.001–0.008)
Moderate anaemia	Feels moderate fatigue, weakness, and shortness of breath after exercise, making daily activities more difficult	0.052 (0.034–0.076)
Severe anaemia	Feels very weak, tired, and short of breath, and has problems with activities that require physical effort or deep concentration	0.149 (0.101–0.210)

Following computations of location-year-age-sex-specific prevalence of hookworm, we leverage information from the 2010 Expert Group (EG) data to conduct sequelae splits. The 2010 EG data provided estimates for heavy infestation, mild abdominopelvic problems, and asymptomatic hookworm by location and for 1990, 2005, and 2010. These three values add up to *all cases* of hookworm. Thus, for heavy infestation and mild abdominopelvic problems, we computed the proportion of cases that belong to our sequelae of interest over *all cases* of hookworm. More specifically, the following is the computation by heavy infestation and mild abdominopelvic problems:

$$Proportion_{sequelae} = \frac{prevalence_{sequelae}}{prevalence_{all\,cases}}$$

This calculates proportions for every location, year, and age group available. The EG data only had four age groups (0–4, 5–9, 10–14, 15+ years), so we applied the 15+ age group proportion for all remaining age groups. In addition, for the years 1995 and 2000, we applied the 1990 proportions, and for years 2015, 2019 and 2020-2021, we applied the 2010 proportions. Using these location-year-age-specific proportions, we multiplied the total hookworm estimates to compute heavy infestation and mild abdominopelvic prevalence. To estimate the prevalence of asymptomatic hookworm, prevalence of mild and heavy infestation were each subtracted from the overall hookworm prevalence.

The final step in the modelling process was to estimate the prevalence of severe wasting due to hookworm in age groups 1–5 months, 6–11 months, 12–23 months and 2–4 years. This was done separately using 1000 draws of prevalence of heavy infestation due to hookworm and the wasting envelope prevalence. The initial step in determining prevalence of severe wasting due to hookworm was generating 1000 draws of change in weight-for-height z-score per heavy prevalent case from a random normal distribution with mean = 0.493826493 and standard deviation = 0.04972834 (calculated from upper and lower bounds of the mean estimate). The mean, upper, and lower bounds were based on a published article.[5] The prevalence of severe wasting due to hookworm was then obtained as a function of change in weight-for-height z-score. The following are the computations:

$$Prevalence_{wasting\ due\ to\ hookworm} = wasting - \Phi(\Phi^{-1}(\text{wasting}) - \text{z score} * \text{heavy infestation})$$

Where Φ is the standard normal cumulative distribution function and Φ^{-1} is the inverse standard normal cumulative distribution function. Finally, the age- and sex-specific anaemia prevalence for hookworm was analysed as part of overall anaemia causal attribution for GBD 2021. The description of the details of the anaemia analysis are in the "Anaemia impairment" section. Briefly, after estimating total anaemia, a series of counterfactual distributions are generated based on the age- and sex-specific prevalence of each anaemia-causing condition, and the quantitative effect that the condition has on haemoglobin concentration in the blood, a "haemoglobin shift," that was derived by meta-analysing cohort studies, observational studies, or trials comparing the haematological status of those with as compared to without the disease. Due to limited data on haemoglobin shift, all were assumed to be invariant over age, sex, location, and year.

Changes from GBD 2019

The major change from GBD 2019 was in specifying new covariates for the ST-GPR global prevalence model, specifically in removing the WHO STH MDA covariate due to noise in the data causing sharp fluctuations in estimates. In future modelling, we plan to re-incorporate MDA coverage either as a covariate and/or by relating treatment to the distribution of severity after developing methods to account for noise in the underlying data.

There were also data changes between the rounds. New data inputs from WHO and ESPEN added to the model. In addition, nationally tagged data in Nigeria and the Philippines were re-tagged to appropriate subnational locations.

We did not apply any adjustments for the COVID pandemic to hookworm due to a lack of available data quantifying the impacts of the pandemic on NTD epidemiology.

Limitations

As we attempt to improve the modelling processes for hookworm, we recognise that there are several limitations. We only include studies where Kato-Katz identifies infected individuals. Future updates to the model will include a systematic review for within-study comparisons of diagnostic performance to facilitate a crosswalk model.

A secondary limitation to our data is that several included studies are not nationally representative, and therefore at a location level, the data are highly heterogeneous . Numerous studies within the database come from districts or townships, and in some cases, the studies were done in known areas where prevalence is high.

Furthermore, we made a large assumption that the global age-sex distributions were applicable to all locations. While we believe that prevalence should peak among young adults and slowly decline afterward, there is likely variation across regions and locations. Given that our data are among adolescents or all-ages, it is very difficult to build an age trend at granular location levels. Thus, we allowed DisMod-MR to disaggregate our heterogeneous data in an effort to provide sensible age-sex curves.

We believe that more work will improve our sequelae split methods. Since the EG data do not provide all estimation years and age groups, several assumptions had to be made. Thus, we will explore conducting literature searches to provide novel datapoints for sequelae estimations.

References

1. London School of Hygiene and Tropical Medicine. Global Atlas of Helminth Infections – Soil Transmitted Helminths. London, United Kingdom: London School of Hygiene and Tropical Medicine.
2. Riess H, Clowes P, Kroidl, Kowuor D, Nsojo A, Mangu C, Schule S, Mansmann U, Geldmacher C, Mhina S, Maboko L, Hoelscher M, Saathoff E. Hookworm Infection and Environmental Factors in Mbeya Region, Tanzania: A Cross-Sectional, Population-Based Study. *PLoS Neglected Tropical Diseases*. 2013. 7. e2408.
3. Pullan R, Kabatereine N, Quinnell R, Brooker S. Spatial and Genetic Epidemiology of Hookworm in a Rural Community in Uganda. *PLoS Neglected Tropical Diseases*. 2010. 4. e713.
4. "Improved and Unimproved Water Sources and Sanitation Facilities." *WHO / UNICEF Joint Monitoring Programme: Wat/san Categories*. The WHO/UNICEF, n.d. Web. 08 June 2016.
5. Hall A, Hewitt G, Tuffrey V, de Silva N. A review and meta-analysis of the impact of intestinal worms on child growth and nutrition. *Maternal and Child Nutrition*. 2008. 4. 118-236.

Infertility (impairment)

Flowchart

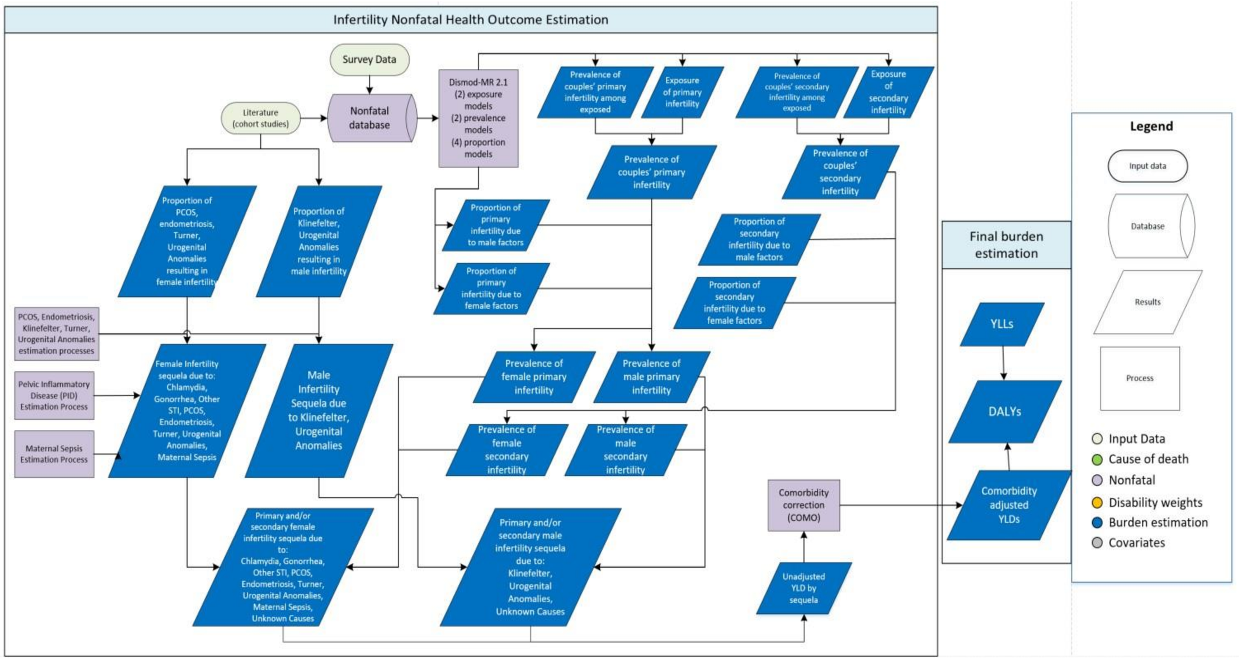

Input data and methodological summary for infertility impairment

Case definition

Infertility encompasses both primary infertility and secondary infertility, which are defined as inability to conceive a child and have a livebirth (primary: first child, secondary: additional children) via vaginal intercourse without using contraceptives.

In GBD, the following case definitions are used for infertility:

- Primary infertility is diagnosed in a couple who have not had a livebirth, desire a child, have been in a union for five years or longer, and are not using contraceptives at the time of the survey.
- Secondary infertility is diagnosed in a couple who last had a livebirth more than five years ago, desire a child, have been in a union for five years or longer, and are not using contraceptives at the time of the survey.

These case definitions agree with definitions of infertility for use with household survey data that were proposed and described in detail by Mascarenhas and colleagues.[1] For GBD purposes, however, we extend this analysis to then estimate the proportion of primary and secondary infertility in couples that are attributable to females and males, and we execute a “causal attribution” process to assign cases of primary female infertility, primary male infertility, secondary female infertility, and secondary male infertility to likely underlying causes and classify the remainder as idiopathic (ie, unknown causes).

Input data

Our primary data sources are population surveys. For GBD 2021, relative to prior rounds of GBD, we added 43 new data sources which included data for three new countries. Data were extracted for women in five-year age groups between 15 and 49 from population-based surveys including the Demographic and Health Surveys (DHS), World Fertility Surveys (WFS), Reproductive Health Surveys

(RHS), Family and Fertility Survey (FFS), and others (EUR, NSF, PCD, PFM). Such surveys only ask fertility-related questions to women in a marriage or union. Even though only women are interviewed, we treated the responses as a proxy for the infertility of couples in unions because the questions are not structured in a way that it is possible to determine which partner is the cause of the couples' inability to conceive a child.

The combination of variables in surveys that were used to construct datasets for each of four models (primary "impairment" and "exposure" and secondary "impairment" and "exposure") are illustrated in the table below. As described below, prevalence of primary and secondary infertility in the population is estimated by multiplying prevalence among those with the "impairment" of infertility by the prevalence of the "exposure".

Table 1: Data extraction definitions used in estimation of infertility

Model name	Indicator	Numerator	Denominator
**Primary infertility (exposure)**	At risk for primary infertility among all women	Married, not using contraception and wants a child OR married and has had a livebirth (A+B+C)	All women surveyed
**Primary infertility (impairment)**	Primary infertility among married women	Married 5+ years, not using contraception, wants a child, and has not had a livebirth (A)	Numerator + Married 5+ years and has had a livebirth (A+B)
**Secondary infertility (exposure)**	At risk for secondary infertility among all women	Married, has had a livebirth, not using contraception, and wants a child OR married, had a birth in the last 5 years, and has had more than one livebirth (A+B+C)	All women surveyed
**Secondary infertility (impairment)**	Secondary infertility among married women	Married 5+ years, had a livebirth more than 5 years ago but not in the last five years, not using contraception, and wants a child (A)	Numerator + Married 5+ years, had a livebirth more than 5 years ago and had a live birth within the last 5 years (A+B)

A second dataset informed estimates of which component of primary and secondary infertility was due to female and male factors, respectively. To obtain data on the sex breakdown for infertility, we used data from a systematic review of the literature conducted for GBD 2010 which used the following search string:

Causes[Title/abstract] AND infertility[Title] NOT mouse NOT murine NOT rat NOT rodent

This produced 626 hits from PubMed and studies were excluded according to the following criteria:

1. studies not representative of the national population;
2. studies that provide no raw data,
3. studies that provide only estimates;
4. studies performed before 1970;
5. case studies or studies with sample size less than 50;
6. studies that provide no data on the sex of the partner responsible for infertility among couples.

The majority of excluded studies were excluded because of the latter criterion. In total, 15 studies were included in our analysis for the sex breakdown among infertile couples. Infertility among couples was reported as due to one of the following causes: male factor, female factor, both, or unknown. Data reporting couples' infertility due to both partners were allocated to both male factor and female factor, and data reporting couples with infertility of unknown cause were allocated to male and female factors based on the proportion observed in other couples in the study.

The total number of data sources included in the analysis are shown in table 2.

Table 2. Data inputs used to estimate infertility

Measures	Total sources	New sources	Countries with data
Prevalence	374	42	117
Other	18	1	15

Data sources specific to the infertility impairment estimation process did not change between GBD 2019 and GBD 2021, although data sources may have changed for estimating the prevalence of one or more disease for which infertility is a sequela, leading to shifts in the attribution of infertility to different GBD diseases. See the "Congenital birth defects", "Gynaecological diseases", "Pelvic inflammatory disease", and "Maternal disorders" portions of the "Non-fatal modelling methods" section in this appendix for updates on data sources for GBD causes that result in infertility.

Klinefelter's and Turner's syndrome cases were all considered to be infertile, but for all other causes of infertility, we required data inputs for estimating the prevalence of infertility due to each disease. The proportion of reproductive-age adults with urogenital anomalies with infertility was taken from Ching and colleagues 2011 and Davies and colleagues 2010 [2,3] (see also "Congenital birth defects" section in this appendix.) The proportions of endometriosis and polycystic ovarian syndrome cases with infertility were taken from the Australian Longitudinal Women's Health Study (ALWHS)[4] (see also the "Gynaecological diseases" section of this appendix). For STIs and maternal sepsis, the proportion of incident cases that go on to develop infertility were obtained from studies by Weström and colleagues[5,6] and were used to determine the proportion of PID cases that are caused by STIs and go on to develop infertility and puerperal sepsis that go on to develop infertility, respectively (see also the "Maternal disorders" sections in this appendix). The application of these proportions is described further in the "Causal attribution" section of this write-up.

Data processing

The processing of infertility data is unchanged in GBD 2021 from GBD 2019, and consists of age-splitting, only. Individual-level data from surveys were extracted and aggregated into GBD age groups, but for any summary measure extracted from published reports that referred to an age range that did not entirely fit within a GBD age group, the observation was split to be multiple age-specific datapoints based on the age pattern predicted by DisMod-MR 2.1 models from the previous round. All extracted indicators were sex-specific and used similar case definitions and data collection methods, so sex-splitting and crosswalking were not required.

Modelling strategy

We estimated the prevalence of primary and secondary infertility by sex and cause in three steps: 1) estimation of couples' infertility [four DisMod-MR 2.1 models], 2) estimation of infertility by sex [four DisMod-MR 2.1 models], and 3) causal attribution of infertility. All DisMod-MR 2.1 models were run as single parameter models (either prevalence or proportion). We assumed zero infertility prior to age 15 or after age 50 years.

In previous rounds, we tested the predictive value of several covariates in all DisMod models: the prevalence of pelvic inflammatory diseases, the risk-weighted prevalence of smoking, obesity, and alcohol use as measured by the summary exposure values (SEV) for smoking, body-mass index, and alcohol consumption (%), and the age-standardised death rate (lnASDR) of sexually transmitted infections (STIs) as country-level covariates. None of these covariates were statistically significant; therefore, no predictive covariates were used in the final models. This choice was carried over to GBD 2021.

Estimation of couples' infertility

To estimate the prevalence of primary and secondary infertility among couples, we first ran four DisMod-MR 2.1 models to estimate the four parameters detailed above: (1) prevalence of primary infertility exposure, (2) proportion of primary infertility among the exposed, (3) prevalence of secondary infertility exposure, and (4) proportion of secondary infertility among the exposed.

We then multiplied estimates of the proportion of infertility among the exposed by the prevalence of exposure to obtain the prevalence of primary infertility among couples and the prevalence of secondary infertility among couples.

Estimation of infertility by sex

Next, we ran four DisMod-MR 2.1 models to estimate the prevalence of primary and secondary infertility by sex: (1) proportion of primary infertility attributable to female factor, (2) proportion of secondary infertility attributable to female factor, (3) proportion of primary infertility attributable to male factor, and (4) proportion of secondary infertility attributable to male factor. Because infertility in some couples is attributable to both partners rather than just one, the sum of the proportions due to each partner can exceed one. When the sum of the proportions estimated by DisMod is lower than one, however, we re-scale them to sum to one. We multiplied the prevalence of primary and secondary infertility derived in step 1 by the proportion due to male and female factors to estimate the following: (1) Female primary infertility (prevalence), (2) Male primary infertility (prevalence), (3) Female secondary infertility (prevalence), (4) Male secondary infertility (prevalence).

Causal attribution of infertility

There are nine identified causes of infertility in the GBD 2021 cause list: pelvic inflammatory disease (PID) due to sexually transmitted infections (STIs), including chlamydia, gonorrhoea, and other STIs; maternal sepsis; polycystic ovarian syndrome (PCOS); endometriosis; congenital Klinefelter syndrome; congenital Turner syndrome; and congenital urogenital anomalies. For each disease, we first determine the total prevalence of infertility due to that disease for each year-age-sex-location combination, and then we assign those to primary and secondary infertility, by sex, according to their natural history, as described below.

Total infertility by cause

For Klinefelter syndrome and Turner syndrome, all prevalent cases of these diseases were considered to be infertile throughout the reproductive years.

For urogenital anomalies, endometriosis, and PCOS, the prevalence of infertility among prevalent disease cases (either alone or in combination with another sequela) were derived from the published sources described above. We applied the proportion of prevalent urogenital anomalies, endometriosis, and PCOS cases with each infertility-related sequela to the prevalence of each disease estimated in DisMod-MR 2.1. This resulted in the prevalence of each infertility-related sequela due to each disease, as listed in the table below. See the "Congenital birth defects" and "Gynaecological diseases" sections in the "Non-fatal modelling methods" portion of this appendix for details on the DisMod models.

For STIs and maternal sepsis, we estimated the prevalence of infertility due to these causes by first estimating incidence of these diseases, applying a published estimate of the proportion of incident cases that go on to develop infertility, and then using these incidence estimates as inputs to an additional DisMod-MR 2.1 compartmental model to estimate the prevalence in the relevant age-groups over time. For STIs, we started with four custom models output from the PID estimation process: the prevalence and incidence of PID, the proportion of PID due to chlamydia, the proportion of PID due to gonorrhoea, and the proportion of PID due to other STIs. See the pelvic inflammatory diseases section in the non-fatal "Modelling methods" portion of this appendix for details about the data inputs and modelling strategy. We then multiplied each proportion model by the PID envelope model to get the following outputs: the prevalence and incidence of PID due to chlamydia, the prevalence and incidence of PID due to gonorrhoea, and the prevalence and incidence of PID due to other STIs. Next, we took an estimate of the proportion of incident PID cases that go on to experience infertility from the analysis of Weström and colleagues,[5] and applied it to the incident cases of PID due to each STI and used DisMod-MR 2.1 to calculate the corresponding prevalence for each subsequent age group through their reproductive years, assuming zero remission or excess mortality. This process produced the following models: the prevalence of infertility due to chlamydia, the prevalence of infertility due to gonorrhoea, and the prevalence of infertility due to other STIs. For maternal sepsis, estimates of puerperal sepsis incidence were taken directly from DisMod (see the "Non-fatal modelling methods: Maternal disorders" section of this appendix for details of DisMod maternal sepsis modelling). These were multiplied by an estimate of the proportion of incident cases that go on to develop infertility, also taken from Westrom,[6] and the product was input into a subsequent DisMod compartmental model to estimate prevalence of infertility due to maternal sepsis over age and time.

Table 3: Infertility sequela by cause

Cause Group	Cause	Sequelae*
Sexually transmitted infections (STIs)	PID due to chlamydia	• Infertility due to chlamydia
	PID due to gonorrhoea	• Infertility due to gonorrhoea
	PID due to other STI	• Infertility due to other STI
Gynaecological diseases	Endometriosis	• Mild abdominal pain and infertility due to endometriosis<br>• Moderate abdominal pain and infertility due to endometriosis<br>• Severe abdominal pain and infertility due to endometriosis<br>• Infertility due to endometriosis

	Polycystic ovarian syndrome	• Infertility due to PCOS<br>• Hirsutism & infertility due to PCOS
Maternal disorders	Maternal sepsis	• Infertility due to puerperal sepsis
Congenital birth defects	Congenital Turner	• Heart diseases with infertility due to Turner syndrome<br>• Infertility due to Turner syndrome
	Congenital urogenital anomalies	• Infertility due to genital anomalies<br>• Atypical genitalia and infertility due to genital anomalies<br>• Infertility and recurrent urinary tract infections or other abdominal issues due to genital anomalies<br>• Infertility and impotence due to genital anomalies<br>• Atypical genitalia, recurrent urinary tract infections or other abdominal issues and infertility due to genital anomalies<br>• Atypical genitalia, infertility, and impotence due to genital anomalies<br>• Infertility, impotence, and recurrent urinary tract infections or other abdominal issues and impotence due to genital anomalies<br>• Atypical genitalia, infertility, impotence, and recurrent urinary tract infections or other abdominal issues and impotence due to genital anomalies
	Congenital Klinefelter	• Borderline intellectual disability with infertility due to Klinefelter syndrome<br>• Mild intellectual disability with infertility due to Klinefelter syndrome<br>• Infertility due to Klinefelter syndrome

*Each of these diseases has other sequela that are not related to infertility that are not listed in this table.

Sex- and stage-specific attribution of infertility

Once the infertility-related sequelae for each disease are estimated, the models are split into primary or secondary infertility for females or males.

Infertility by stage – female

The following diseases can cause infertility in females: chlamydia, gonorrhoea, other STIs, endometriosis, PCOS, maternal sepsis, congenital Turner syndrome, and congenital urogenital anomalies.

To split the female infertility sequela into primary or secondary infertility, we aggregate the primary and secondary female impairment envelopes, and then calculate the proportion of the aggregate envelope that is primary infertility and the proportion that is secondary infertility. We multiply the primary and secondary proportions by the prevalence of each female infertility sequela for PCOS, endometriosis, gonorrhoea, chlamydia, and other STIs to split each sequela into primary or secondary sequela. All female infertility sequelae from congenital Turner and congenital urogenital syndromes are assigned to female primary infertility. The infertility sequela from maternal sepsis is assigned to female secondary infertility.

Next, we estimate the portion of female infertility that is due to unknown causes, by stage. To do this, we sum the prevalence of all female primary infertility sequelae for comparison to 95% of the female primary infertility impairment envelope. If the prevalence of the summed female primary sequelae is less than 95% of the female primary envelope, then the difference between the prevalence of the summed sequelae and the envelope is assigned to "primary female infertility due to other causes", also known as idiopathic primary female infertility. If the sum of the female primary infertility sequelae is greater than 95% of the envelope, each individual sequela is scaled to 95% of its prevalence estimates, then the scaled sequelae are aggregated. We assign the difference between the rescaled, aggregated female primary infertility sequela and the female primary infertility impairment envelope to idiopathic primary female infertility. The same process is used to estimate idiopathic secondary female infertility, substituting the female primary sequelae and envelope for their secondary counterparts.

Finally, we determine the cases of endometriosis and PCOS sequelae that were misallocated to sequelae with infertility. To do this, we aggregate the primary and secondary sequelae for each disease and compare to the prevalence of the sequelae prior to being split into primary and secondary. If the prevalence of the original sequelae is greater than the prevalence of the aggregated type-specific sequelae, we assign the excess cases back to the disease, and not to the infertility-related sequela due to the disease. The excess cases are not included in years lived with disability (YLDs) or disability-adjusted life-years (DALYs) estimates for infertility, but rather for PCOS or endometriosis.

Infertility by stage – male

The following diseases can cause infertility in males: congenital Klinefelter syndrome and congenital urogenital anomalies.

To estimate male primary infertility due to unknown causes, we aggregate the prevalence of all Klinefelter and urogenital anomalies sequelae and subtract the summed value from the male primary infertility impairment envelope. We assign the remaining prevalence to idiopathic male primary infertility impairment. All Klinefelter and urogenital anomalies sequelae are assigned to primary infertility.

There are no known specific causes of secondary infertility in males, so we assigned the entire secondary male infertility impairment envelope to idiopathic secondary male infertility impairment.

Table 4: Infertility sequelae by sex, stage, and cause

Cause	Female	Male
[PID due to] chlamydia	• Primary/secondary infertility due to chlamydia	NA
[PID due to] gonorrhoea	• Primary/secondary infertility due to gonorrhoea	NA
[PID due to] other STI	• Primary/secondary infertility due to other STI	NA
Endometriosis	• Mild abdominal pain and primary/secondary infertility due to endometriosis	NA

	• Moderate abdominal pain and primary/secondary infertility due to endometriosis<br>• Severe abdominal pain and primary/secondary infertility due to endometriosis<br>• Primary/secondary Infertility due to endometriosis	
Polycystic ovarian syndrome	• Primary/secondary infertility due to PCOS<br>• Hirsutism and primary/secondary infertility due to PCOS	NA
Maternal sepsis	• Secondary infertility due to puerperal sepsis	NA
Congenital Turner	• Heart diseases with primary infertility due to Turner syndrome<br>• Primary infertility due to Turner syndrome	NA
Congenital Klinefelter	• NA	• Borderline intellectual disability with primary infertility due to Klinefelter syndrome<br>• Mild intellectual disability with primary infertility due to Klinefelter syndrome<br>• Primary infertility due to Klinefelter syndrome
Unknown	• Idiopathic primary and secondary infertility	
Congenital urogenital anomalies	• Primary infertility due to genital anomalies<br>• Atypical genitalia and primary infertility due to genital anomalies<br>• Primary infertility and recurrent urinary tract infections or other abdominal issues due to genital anomalies<br>• Primary infertility and impotence due to genital anomalies<br>• Atypical genitalia, recurrent urinary tract infections or other abdominal issues, and primary infertility due to genital anomalies<br>• Atypical genitalia, primary infertility, and impotence due to genital anomalies<br>• Primary infertility, impotence, and recurrent urinary tract infections or other abdominal issues and impotence due to genital anomalies<br>• Atypical genitalia, primary infertility, impotence, and recurrent urinary tract infections or other abdominal issues and impotence due to genital anomalies	

Sequelae and disability weights

Every person with infertility is assumed to experience the health state as determined from the GBD disability weights survey. The lay descriptions of primary and secondary infertility are below.

Table 5: Health states used in estimation of YLDs due to infertility

Health state name	Health state description	Disability weight
Infertility, primary	This person wants to have a child and has a fertile partner, but the couple cannot conceive.	0.008 (0.003–0.015)
Infertility, secondary	This person has at least one child and wants to have more children. The person has a fertile partner, but the couple cannot conceive.	0.005 (0.002–0.011)

References

1 Mascarenhas, Maya N., et al. National, regional, and global trends in infertility prevalence since 1990: a systematic analysis of 277 health surveys. *PLoS medicine* 9.12 (2012): e1001356.

2 Ching CB, Wood HM, Ross JH, Gao T, Angermeier KW. The Cleveland Clinic experience with adult hypospadias patients undergoing repair: their presentation and a new classification system. *BJU Int*. 2011; 107(7): 1142–6.

3 Davies MC, Liao L-M, Wilcox DT, Woodhouse CRJ, Creighton SM. Anorectal malformations: what happens in adulthood?. *BJU Int*. 2010; 106(3): 398–404.

4 Loxton D, Dobson A, Byles J, Tooth L. Australian Longitudinal Study on Women's Health (ALSWH). http://www.alswh.org.au/.

5 Weström L, Joesoef R, Reynolds G, Hagdu A, Thompson SE. Pelvic inflammatory disease and fertility. A cohort study of 1,844 women with laparoscopically verified disease and 657 control women with normal laparoscopic results. *Sex Transm Dis* 1992; **19**: 185–92.

6 Weström LV. Chlamydia and its effect on reproduction. *J Br Fer Soc* 1996; **1**: 23–30.

Inflammatory bowel disease

Flowchart

Inflammatory Bowel Disease

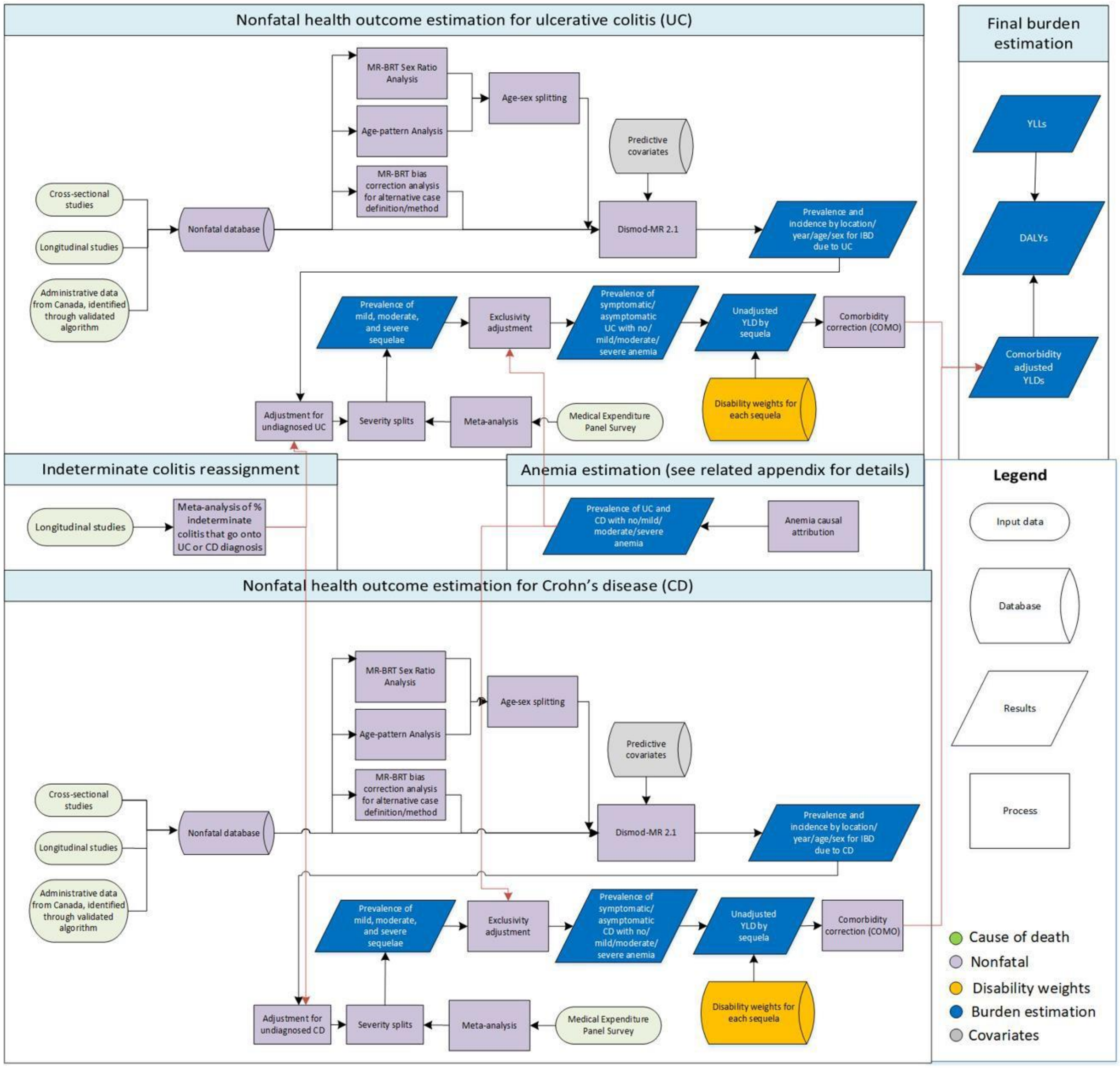

Input data and methodological summary for inflammatory bowel disease

Case definition

Inflammatory bowel disease is a group of chronic diseases resulting from non-infectious inflammation of the colon and gastrointestinal tract. These include Crohn's disease (inflammation throughout the gastrointestinal tract, but predominantly the small and large intestine) and ulcerative colitis (inflammation of the colon and rectum). These diseases result in episodes of abdominal pain, gastrointestinal bleeding, diarrhea, and potential additional complications, with many patients also experiencing intervening asymptomatic periods.

The reference case definition includes cases that are diagnosed by endoscopy, imaging studies, or biopsy in a patient with appropriate clinical signs and symptoms, or cases that are identified through a patient database (including both inpatient and outpatient care) using the International Classification of Diseases (ICD)-based case-identification algorithm that is validated through detailed chart review. As non-reference standard, we also included studies that extracted cases from a patient database without using a validated algorithm.

In some cases of inflammatory bowel disease, neither Crohn's disease nor ulcerative colitis can be definitively diagnosed, and a diagnosis of indeterminate colitis is applied, indefinitely, or until definitive features of Crohn's or ulcerative colitis declare themselves.

ICD codes are K50 for Crohn's disease, K51 for ulcerative colitis, and K52 for indeterminate colitis.

Overall strategy

As in GBD 2019, we utilised two databases for inflammatory bowel disease as inputs to two separate, complete compartmental DisMod models: ulcerative colitis and Crohn's disease. We then adjusted both for the proportion of indeterminate colitis cases thought to represent undiagnosed ulcerative colitis and Crohn's, and then applied distributions of the frequency of symptoms.

Input data and data processing

Input data

For GBD 2016, a systematic literature review was conducted to capture studies of prevalence and incidence for all inflammatory bowel diseases. A PubMed search was conducted using the following search string: (("crohn disease"[MeSH Terms] OR ("crohn"[All Fields] AND "disease"[All Fields]) OR "crohn disease"[All Fields] OR ("crohn's"[All Fields] AND "disease"[All Fields]) OR "crohn's disease"[All Fields]) OR ("colitis, ulcerative"[MeSH Terms] OR ("colitis"[All Fields] AND "ulcerative"[All Fields]) OR "ulcerative colitis"[All Fields] OR ("ulcerative"[All Fields] AND "colitis"[All Fields])) OR (Inflammatory[All Fields] AND Bowl[All Fields]) OR (("irritable bowel syndrome"[MeSH Terms] OR ("irritable"[All Fields] AND "bowel"[All Fields] AND "syndrome"[All Fields]) OR "irritable bowel syndrome"[All Fields]) AND ("diarrhoea"[All Fields] OR "diarrhea"[MeSH Terms] OR "diarrhea"[All Fields]))) AND ("epidemiology"[Subheading] OR "epidemiology"[All Fields] OR "epidemiology"[MeSH Terms]) AND (("2016"[PDAT]) NOT (animals[MeSH] NOT humans[MeSH])).

The exclusion criteria were:

1. Studies clearly not representative of a geographically defined general population.
2. Studies that did not provide primary data on epidemiological parameters, eg, a commentary piece.

In GBD 2019, we re-reviewed and excluded studies that included diagnosis by self-report or did not provide sufficient information about the study population to derive crude prevalence or incidence estimates.

For GBD 2021, we added additional data from peer-reviewed publications identified via a systematic review that was conducted by Ng and colleagues in 2017.[1] We also added administrative data from Canada that were extracted using a validated algorithm.[2]

In addition to the aforementioned prevalence and incidence data extracted from population-based studies, we also used in previous rounds of GBD prevalence estimates extracted from hospital inpatient discharge and claims data processed by IHME, which we will refer to as clinical informatics data throughout this report. As a chronic disorder that is primarily treated in outpatient settings, IBD cases are poorly captured in inpatient discharge data. To address this, IHME has historically modelled correction factors (ie, ratios of inpatient admissions to total cases identified in both inpatient and outpatient care) using MarketScan medical insurance claims data from the United States. Despite this adjustment, however, we noted a large discrepancy between the adjusted inpatient discharge data and the reference data in locations where we had both data sources, suggesting a systematic bias in the ratio of inpatient admissions to total cases observed in the commercially insured population. In GBD 2021, we attempted to improve the correction factors by modelling them using multiple claims sources – from Poland and USA MarketScan data. The correction factors and age-sex patterns, however, were so different between the two data sources that correction factor models using both of them would not converge. From this, we inferred that there were substantial differences in coding practices and sites of care compared to the actual disease burden. Until we have more data to derive more robust correction factors, we decided to exclude clinical informatics data in GBD 2021.

Table 1. Data inputs for inflammatory bowel disease morbidity modelling by parameter

	**Countries with data**	**New sources**	**Total sources**
Prevalence	42	29	92
Incidence	67	25	174
Other	2	0	16

Data processing

For both ulcerative colitis and Crohn's disease models, the non-reference (or "alternative") datapoints were adjusted toward the reference, which we refer to as "stringent criteria" in shorthand, using a meta-regression approach called meta-regression—Bayesian, regularised, trimmed (MR-BRT) analysis. Specifically, we first identified studies that reported data using both reference and non-reference methods (Figure 1). We then calculated the logit difference between the two datapoints for each study and used as an input to MR-BRT to estimate pooled logit-difference. This adjustment factor was applied to all datapoints collected with non-reference methods. The process of adjusting for non-reference data using MR-BRT with the logit-transformation method is described below:

50. Identify datapoints with overlapping year, age, sex, and location between non-reference and reference data within the same study.

[1]Ng SC, Shi HY, Hamidi N, Underwood FE, Tang W, Benchimol EI, et al. Worldwide incidence and prevalence of inflammatory bowel disease in the 21st century: a systematic review of population-based studies. *The Lancet*. 2018 23;390(10114):2769–78.

[2]Coward S, Clement F, Benchimol EI, Bernstein CN, Avina-Zubieta JA, Bitton A, Carroll MW, Hazlewood G, Jacobson K, Jelinski S, Deardon R, Jones JL, Kuenzig ME, Leddin D, McBrien KA, Murthy SK, Nguyen GC, Otley AR, Panaccione R, Rezaie A, Rosenfeld G, Peña-Sánchez JN, Singh H, Targownik LE, Kaplan GG. Past and Future Burden of Inflammatory Bowel Diseases Based on Modeling of Population-Based Data. *Gastroenterology*. 2019 Apr;156(5):1345-1353.e4.

51. Logit-transform overlapping datapoints of alternative and reference case definitions.
52. Convert overlapping datapoints into a difference in logit space using the following equation: $logit(altnerative) - logit(reference)$.
53. Use the delta method to compute standard errors of overlapping datapoints in logit space, then calculate standard error of logit difference using the following equation: $\sqrt{(variance\ of\ alternative) + (variance\ of\ reference)}$
54. Using MR-BRT, conduct a random effects meta-regression to obtain the pooled logit difference of alternative to reference.
55. Apply the pooled logit difference to all datapoints from studies that only employed non-reference methods using the following equation: $new_{estimate} = inverse.logit((logit(alternative)) - (pooled\ logit\ difference))$.
56. Calculate new standard errors using the delta method, accounting for gamma (between-study heterogeneity).

Figure 1. Studies reported both reference and non-reference (alternative) data used in MR-BRT

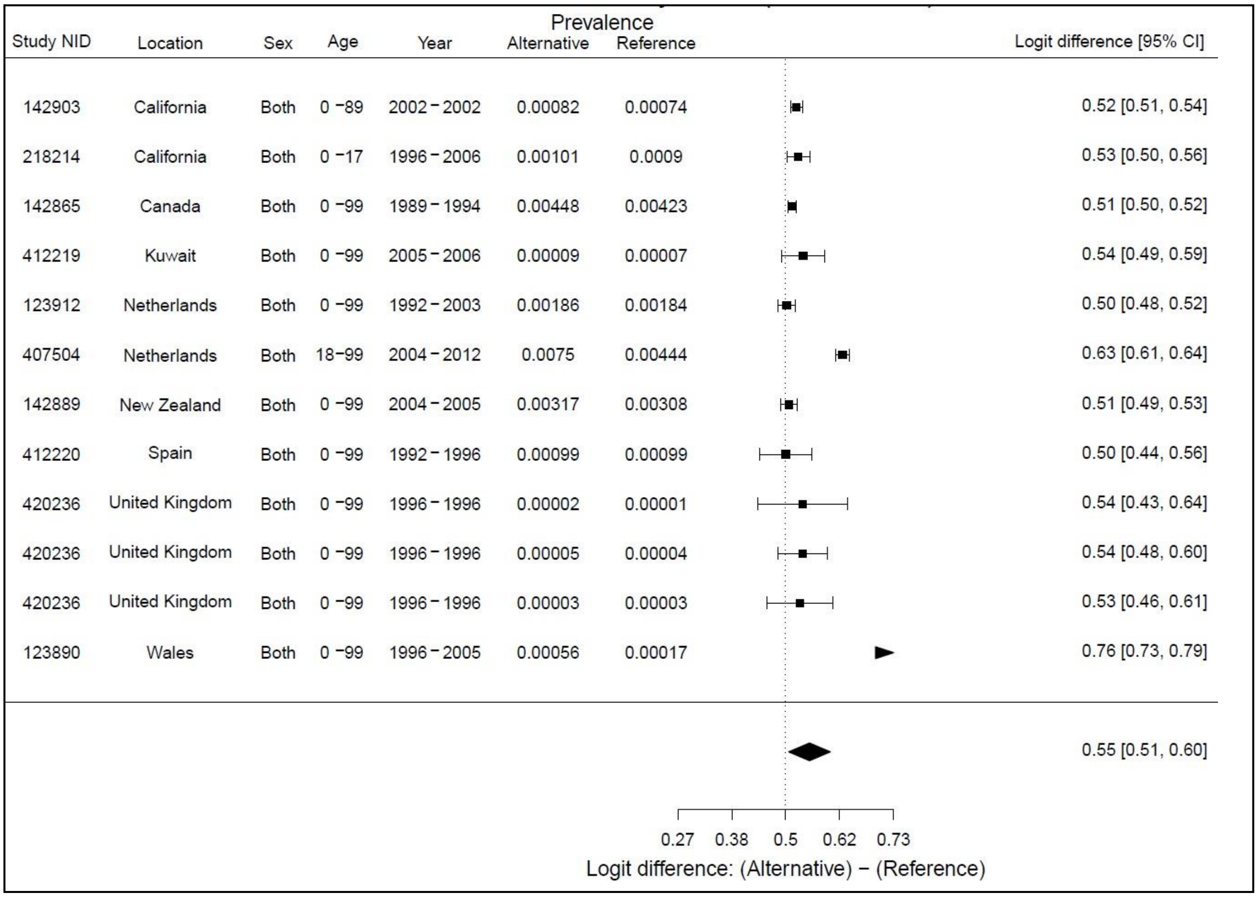

This bias adjustment method allows a more direct comparison between the reference and non-reference data. Prior to GBD 2019, we adjusted alternative case definitions or study design characteristics to the reference standard by creating binary covariates for these alternative groups and estimating a fixed effect for these covariates in our DisMod meta-regression modelling process. This amounts to adjusting data using an ecological comparison and is vulnerable to compositional bias; if data from different location-years were collected using different methods or case definitions, true spatiotemporal

differences in epidemiology can be erroneously adjusted, and differences truly due to differences in methods can be erroneously estimated as differences in underlying epidemiology. By using matches of reference and non-reference estimates, we were able to bypass compositional bias and adjust non-reference datapoints more accurately, particular given that our matched reference and non-reference data were measured on the same sample.

The table below shows bias correction factors estimated using MR-BRT.

Table 2. MR-BRT crosswalk adjustment factors for inflammatory bowel disease

Data input	Reference or alternative case definition	Gamma	Beta coefficient, logit (95% CI)	Adjustment factor*
Stringent criteria	Ref	0	--	--
ICD-code based administrative data	Alt		0.06 (0.04–0.09)	1.07 (1.04–1.10)

**MR-BRT crosswalk adjustments can be interpreted as the factor the alternative case definition is adjusted by to reflect what it would have been had it been measured using the reference case definition. If the log/logit beta coefficient is negative, then the alternative is adjusted up to the reference. If the log/logit beta coefficient is positive, then the alternative is adjusted down to the reference.*
***The adjustment factor column is the exponentiated beta coefficient. For log beta coefficients, this is the relative rate between the two case definitions. For logit beta coefficients, this is the relative odds between the two case definitions.*

We split datapoints where the age range was greater than 25 years using the super-region age patterns informed by the datapoints with fine age groups (ie, ages 5–9, 10–14, and 15–20…). We also split data reported for both sexes using the pooled sex ratio estimated from studies that reported prevalence or incidence in males and females separately. For prevalence, the ratios of female to male cases derived from MR-BRT analysis were 0.91 (95% UI 0.60–1.38) and 1.13 (95% UI 0.36–3.53) for ulcerative colitis and Crohn's disease, respectively. For incidence, the ratios of female to male cases derived from MR-BRT analysis were 0.92 (0.65–1.30) and 1.20 (0.59–2.45) for ulcerative colitis and Crohn's disease, respectively.

We excluded any data for subnational locations under the age of 20 years that had excessive influence on the estimation of pseudo-random effects and the subnational prior distribution and led the model to ignore more abundant data in older age groups; this occurred in some subnational locations in Japan and the USA.

Modelling strategy

Modelling

The modelling strategy for all inflammatory bowel disease encompasses two separate DisMod models for ulcerative colitis and Crohn's disease, which are then adjusted to account for inflammatory bowel disease due to indeterminate colitis.

<u>Non-infective inflammatory bowel disease due to ulcerative colitis, pre-adjustment (for indeterminate colitis)</u>

Similar to GBD 2019, we ran a DisMod-MR 2.1 model to produce estimates by age, sex, year, and country. Prior settings included setting incidence to zero for ages 0 to 2 and 0.00025 for ages 80 to 100, remission to zero for all ages, and excess mortality rate (EMR) to 0.2 for all ages. The minimum coefficient of variation at the regional, super-regional, and global level was set at 0.8. Predictive covariates included Socio-demographic Index on incidence and Healthcare Access and Quality Index on EMR.

Non-infective inflammatory bowel disease due to Crohn's disease, pre-adjustment (for indeterminate colitis)

Similar to GBD 2019, we ran a DisMod-MR 2.1 model to produce estimates by age, sex, year, and country. Prior settings included setting incidence to zero for ages 0 to 2 and 0.00025 for ages 80 to 100, remission to zero for all ages, and excess mortality rate (EMR) to 0.2 for all ages. The minimum coefficient of variation at the regional, super-regional, and global level was set at 0.8. Predictive covariates included Socio-demographic Index on incidence and Healthcare Access and Quality Index on EMR.

Betas and exponentiated values for predictive covariates (which can be interpreted as an odds ratio) are shown in the table below.

Table 3. Covariates. Summary of covariates used in the inflammatory bowel disease DisMod-MR meta-regression models

Ulcerative colitis

Covariate	Parameter	Exponentiated beta (95% uncertainty interval)
Socio-demographic Index	Incidence	5.70 (4.57–6.73)
Healthcare Access and Quality Index	Excess mortality rate	0.60 (0.37–0.98)

Crohn's disease

Covariate	Parameter	Exponentiated beta (95% uncertainty interval)
Socio-demographic Index	Incidence	7.31 (7.20–7.38)
Healthcare Access and Quality Index	Excess mortality rate	0.61 (0.38–0.98)

Indeterminate colitis adjustment

After running DisMod-MR, the model outputs of ulcerative colitis and Crohn's disease were adjusted to account for indeterminate colitis. This approach assumed that all indeterminate colitis cases would be ultimately confirmed as either ulcerative colitis or Crohn's disease. Specifically, we identified studies that reported the proportion of indeterminate colitis cases in the total number of IBD cases. Then, we ran a

meta-regression model in R to find a pooled proportion of indeterminate colitis attributable to the burden of ulcerative colitis and Crohn's disease. Both ulcerative colitis and Crohn's disease model outputs were adjusted using the pooled proportion at the draw level. The adjusted estimates were then combined to estimate the total burden of IBD.

Severity split & disability weight

The basis of the GBD disability weight survey assessments are lay descriptions of sequelae highlighting major functional consequences and symptoms. For GBD 2021, we used the Medical Expenditure Panel Survey to find the proportion of ulcerative colitis and Crohn's disease asymptomatic versus symptomatic during a given four-week period. The lay descriptions and disability weights for sequelae associated with inflammatory bowel disease are shown below.

Table 4. Severity distribution, details on the severity levels for inflammatory bowel disease in GBD 2021 and the associated disability weight (DW) with that severity

Severity split	Lay description	DW (95% CI)
Crohn's disease, currently asymptomatic	--	0
Crohn's disease, symptomatic	This person has cramping abdominal pain, has diarrhoea several times a day, and feels very tired for two months every year. When the person does not have symptoms, there is anxiety about them returning.	0.231 (0.156–0.32)
Ulcerative colitis, currently asymptomatic	--	0
Ulcerative colitis, symptomatic	This person has cramping abdominal pain, has diarrhoea several times a day, and feels very tired for two months every year. When the person does not have symptoms, there is anxiety about them returning.	0.231 (0.156–0.32)

The age-specific anaemia prevalence for symptomatic cases of ulcerative colitis and Crohn's disease was analysed as part of overall anaemia causal attribution for GBD 2021. The details of the anaemia analysis are described separately in the "Anaemia impairment" section. Briefly, after estimating total anaemia, a series of counterfactual distributions are generated based on the age- and sex-specific prevalence of each anaemia-causing condition and the quantitative effect that the condition has on haemoglobin concentration in the blood, a so-called "haemoglobin shift," that was derived by meta-analysing cohort studies, observational studies, or trials comparing the haemotological status of those with as compared to without the disease. Due to limited data on haemoglobin shift, all were assumed invariant over age, sex, location, and year.

Inguinal, femoral, and abdominal hernia

Flowchart

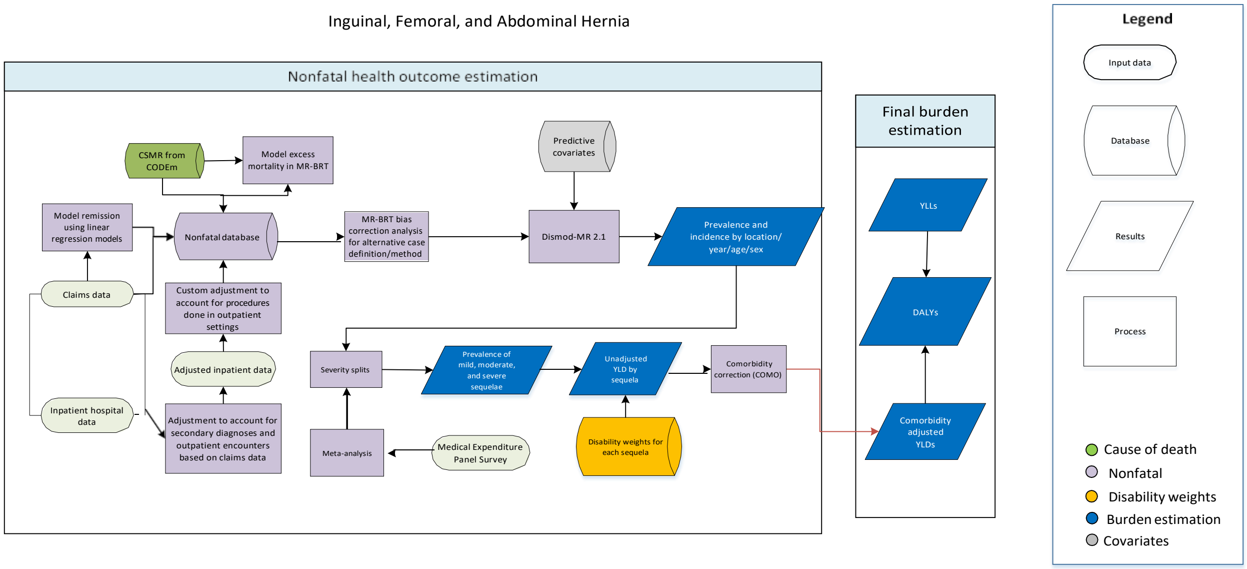

Input data and methodological summary for inguinal, femoral, and abdominal hernia

Case definition

Hernia refers to the protrusion of an abdominal internal organ through an opening in the tissue that holds it in place, regardless of symptoms. Inguinal, femoral, and abdominal hernia comprises the disorders in which portions of the digestive tract protrude through defects in the walls of the abdominal cavity. These occasionally lead to life-threatening acute complications, but more commonly are asymptomatic or cause chronic or intermittent pain. Symptomatic hernia is surgically repaired.

ICD-10 codes are K40, K41, K42, K44, K45, and K46 and all their 4-digit and 5-digit constituents. The ICD-9 codes are 550, 551, 552, 553 and their constituents, with the exceptions of 551.1-3, 552.1-3, and 553.1-3. The procedure codes for hernia repair are 43336-43337, 44050, 49491-49492, 49495-49496, 49500-49501, 49505, 49507, 49525, 49540, 49550, 49553, 49555, 49557, 49560-49561, 49565-49566, 49568, 49570, 49572, 49585, 49587, 49590, 49650-49653, and 54640.

Overall strategy

In GBD 2017, two databases were developed for inguinal, femoral, and abdominal hernia to separately model total (symptomatic + asymptomatic cases) and symptomatic cases. In GBD 2019, the DisMod model for symptomatic cases was dropped, and we only modelled total cases of hernia in DisMod; an updated severity distribution was then applied as described below. This GBD 2019 approach was carried forward in GBD 2021.

Input data and data processing

Inputs

Like GBD 2019, the model included prevalence data from hospital discharges and claims. In GBD 2021, we newly added additional years of data from USA claims (year 2017) and Poland claims (year 2018), as well as hospital discharges in Greece, Armenia, Chile, Ecuador, Argentina, Italy, Brazil, and Spain.

Table 1. Data Inputs for inguinal, femoral, and abdominal hernia morbidity modelling by parameter

	**Countries with data**	**New sources**	**Total sources**
Prevalence	49	34	325
Other	1	0	15

Inputs to our non-fatal modelling also included cause-specific mortality rate (CSMR) estimates taken from our fatal modelling process (see CoD cause-specific modelling description for hernia in this appendix) and remission and excess mortality rates (EMR) estimates modelled outside of DisMod (see the remission and EMR data processing sections below).

Prevalence data processing

The data processing approach was largely similar to GBD 2019. Specifically, we extracted prevalent cases of hernia for the total hernia database from claims data in the same manner as in GBD 2019—extracting prevalent cases from claims data if an individual had one inpatient or two outpatient encounters with a hernia ICD code as any diagnosis. We assumed that individuals with either an inpatient encounter with a hernia ICD code or an outpatient encounter with both hernia ICD code and procedural code for hernia repair was symptomatic, and that most symptomatic cases of hernia were treated in an inpatient setting. Consequently, we summed the inpatient and outpatient encounters with procedures in the USA claims data, estimated the ratio of this sum to all encounters with hernia ICD codes, and applied this ratio to international hospital discharge data to estimate total hernia cases for populations for which individual-level claims data were not available.

Although better able to capture the relationship between inpatient and outpatient care with the aforementioned custom correction factors, USA claims data were still regarded as suffering from selection bias due to commercial health insurance status. Thus, total hernia prevalence data extracted from USA claims from the year 2000 and from the years 2010–2017 were ultimately adjusted to total hernia prevalence data from hospital discharges. This was done in MR-BRT using the logit-transformation method is described below:

1. Identify datapoints with overlapping year, age, sex, and location between commercial claims (non-reference data type) and hospital discharges (reference data type).
2. Logit-transform overlapping datapoints of alternative and reference data types.
3. Convert overlapping datapoints into a difference in logit space using the following equation: $logit(altnerative) - logit(reference)$.
4. Use the delta method to compute standard errors of overlapping datapoints in logit space, then calculate standard error of logit difference using the following equation: $\sqrt{(variance\ of\ alternative) + (variance\ of\ reference)}$.
5. Using MR-BRT, conduct a random effects meta-regression to obtain the pooled logit difference of alternative to reference.
6. Apply the pooled logit difference to all datapoints of alternative case definitions using the following equation:

$$new_{estimate} = inverse.logit((logit(alternative)) - (pooled\ logit\ difference)).$$

7. Calculate new standard errors using the delta method, accounting for gamma (between-study heterogeneity).

The table below shows bias correction factors of alternative case definitions using MR-BRT.

Table 2. MR-BRT crosswalk adjustment factors for inguinal, femoral, and abdominal hernia

Data input	Reference or alternative case definition	Gamma	Covariate	Beta coefficient, logit (95% CI)	Adjustment factor*
Hospital + non-USA claims	Ref	0.001		---	---
USA claims from year 2000	Alt		Age (continuous from 0 to 95+)	–0.03 (–0.033 to –0.027)	0.97 (0.968 to 0.973)
			Intercept	4.04 (4.01 to 4.08)	57.00 (55.10 to 58.95)
USA claims from years 2010–2017	Alt		Age (continuous from 0 to 95+)	–0.02 (–0.026 to –0.022)	0.976 (0.974 to 0.978)
			Intercept	4.47 (4.37 to 4.57)	87 (70.17 to 96.31)

**MR-BRT crosswalk adjustments can be interpreted as the factor the alternative case definition is adjusted by to reflect what it would have been had it been measured using the reference case definition. If the log/logit beta coefficient is negative, then the alternative is adjusted up to the reference. If the log/logit beta coefficient is positive, then the alternative is adjusted down to the reference.*
***The adjustment factor column is the exponentiated beta coefficient. For log beta coefficients, this is the relative rate between the two case definitions. For logit beta coefficients, this is the relative odds between the two case definitions.*

Datapoints with an age-standardised prevalence greater than two median absolute deviations from the median of the age-standardised prevalence for all inpatient and non-USA claims data were marked as outliers and excluded from analysis.

Remission processing

In several rounds prior to GBD 2019, we used remission estimates derived from a single, large study of mean wait times for elective surgical repair in OECD countries conducted by Siciliani and colleagues. Starting in GBD 2019, we aimed to better inform DisMod on the increasing pattern of remission with greater access to quality health care. To do so, we used remission data from the USA claims, defined as the number of people with a hernia repair procedure code among all people with hernia diagnosis, and regressed against Healthcare Access and Quality (HAQ) Index and sex with an assumption that hernia does not resolve on its own without a surgical repair, so remission is 0 at a theoretical HAQ Index value of 0. In GBD 2021, we updated this by including an additional covariate, age, to capture age variations in

remission. The results from the regression model were then used to predict remission estimates for each location, year, and sex for ages 0, 10, 20…100.

Figure 1. Predicted remission in function of age, sex, and HAQ Index

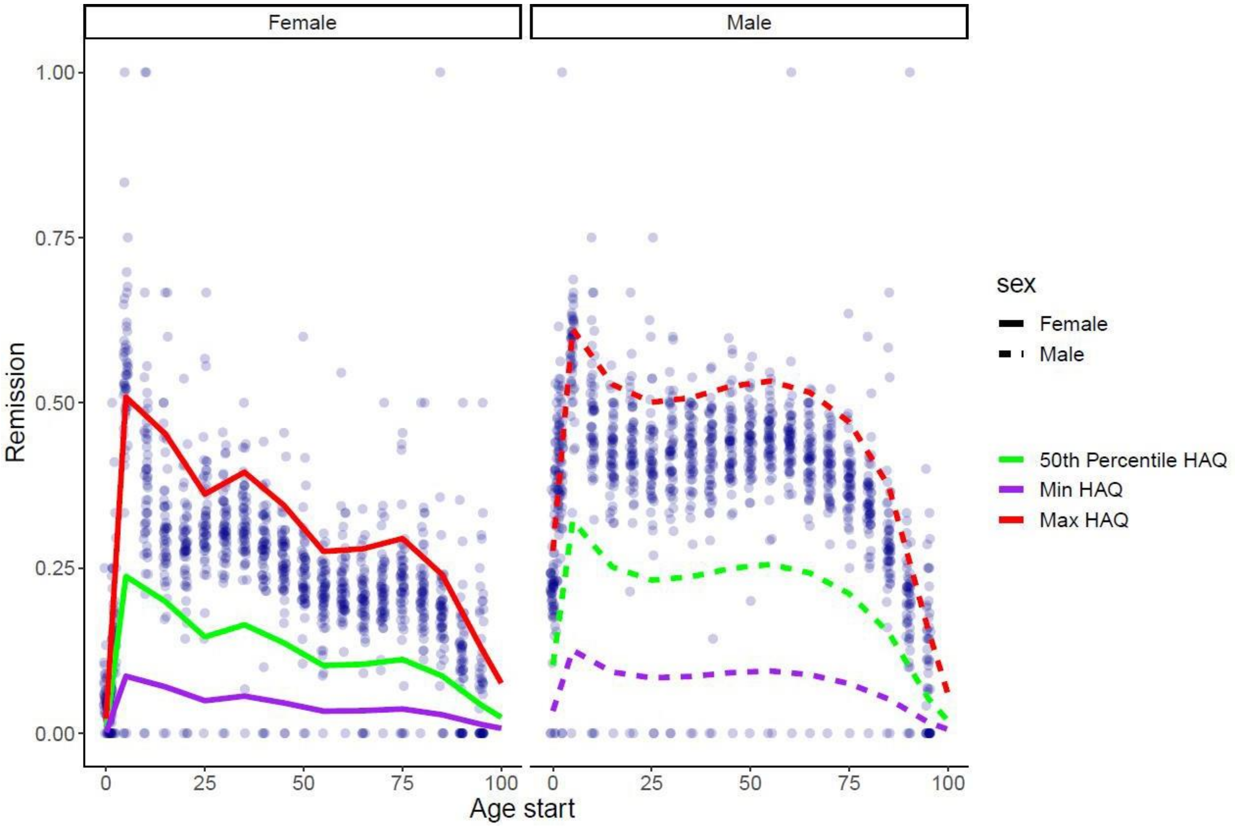

EMR processing

In GBD 2017, EMR inputs were produced by matching prevalence datapoints with their corresponding CSMR values within the same age, sex, year, and location (by dividing CSMR by prevalence). For short-duration conditions (remission >1), the corresponding prevalence was derived by running an initial model and then applying the same CSMR/prevalence method. However, this method of producing EMR inputs demonstrated a rather unrealistic pattern of EMR compared to an expected pattern of decreasing EMR with greater access to quality health care. Such unexpected patterns often signal inconsistencies between CSMR estimates and the measures of prevalence and/or incidence. Thus, in an effort to provide greater guidance on the expected pattern of EMR, in GBD 2019, EMR data produced per above in GBD 2017 were modelled by age, sex, and HAQ Index using MR-BRT, with a prior on HAQ Index having a negative coefficient. In GBD 2021, we employed the same MR-BRT method to predict EMR for each location, year, sex, and for ages 0, 10, 20….100, these predictions were used as inputs to our non-fatal model, below.

Modelling strategy

Modelling

Similar to previous rounds, we ran a DisMod-MR 2.1 model to produce estimates by age, sex, year, and location. Inputs to DisMod for hernia include prevalence, remission, CSMR and EMR inputs processed as described above. We changed several DisMod settings in GBD 2021. First, we removed the prior on EMR, which had a bound of 0 to 0.00002 between ages 0 and 15. We also removed an upper bound of incidence rate at 0.01 between ages 0 and 20. This was to capture high incident cases of hernia in children. The minimum coefficient of variation at the regional, super-regional, and global level was set at 0.8. In contrast to GBD 2019, we allowed birth prevalence of hernia. We used smoking prevalence and mean BMI as predictive covariates for prevalence. The HAQ Index and lag-distributed income (log transformed) covariates were applied to EMR and remission, respectively. Betas and exponentiated values for these predictive covariates (which can be interpreted as an odds ratio) are shown in the table below.

Table 3. Covariates. Summary of predictive covariates used in the total inguinal, femoral, and abdominal hernia DisMod-MR meta-regression model

Covariate	Parameter	Exponentiated beta (95% uncertainty interval)
Smoking prevalence	Prevalence	6.26<br>(5.98 to 6.53)
Mean BMI	Prevalence	0.86<br>(0.86 to 0.87)
Healthcare Access and Quality Index	Excess mortality rate	0.99<br>(0.99 to 0.99)
LDI (I$ per capita)	Remission	1.65<br>(1.65 to 1.65)

Severity split and disability weight

The DisMod model of symptomatic hernia used in GBD 2017 was dropped in GBD 2019, and symptom occurrence and severity distribution were estimated from Medical Expenditure Panel Survey (MEPS) data using standard GBD methodology. MEPS is an overlapping panel survey of the non-institutionalised USA population that collects data on respondents' health service interactions. Panels are initiated every year. Each panel is two years long and consists of five rounds. In 2000, MEPS began using 12-Item Short Form Surveys (SF-12) to collect data on functional health status. The SF-12 survey is administered twice per panel (about once per year).

In order to translate SF-12 scores into GBD disability weights, 62 lay descriptions for conditions representing the full range of disability weight values (from most mild to most severe) were selected. A convenience sample of respondents was then asked to complete an SF-12 form for an individual with the health state described in the lay descriptions of these conditions. Composite mental and physical SF-12 score was regressed on GBD disability weight to derive the relationship between disability weight and SF-12 score. Individual respondent scores were then regressed on reported conditions to obtain a

comorbidity-corrected condition-specific disability weight. The distribution of these condition-specific weights was used to derive the proportion of individuals with the conditions that fall within each GBD severity category.

The basis of the GBD disability weight survey assessments are lay descriptions of sequelae highlighting major functional consequences and symptoms. Prevalent cases of symptomatic hernia were divided according to severity distributions derived from data from the MEPS to assign them to mild, moderate, and severe sequelae. Asymptomatic cases were assigned no disability. The lay descriptions and disability weights for inguinal, abdominal, and femoral hernia are shown below.

Table 4. Severity distribution, details on the severity levels for Inguinal, femoral, and abdominal hernia in GBD 2019 and the associated disability weight (DW) with that severity

Severity level	Lay description	DW (95% CI)
Asymptomatic	--	0
Mild	This person has some pain in the belly that causes nausea but does not interfere with daily activities.	0.011 (0.005–0.021)
Moderate	This person has pain in the belly and feels nauseous. The person has difficulties with daily activities.	0.114 (0.080–0.159)
Severe	This person has severe pain in the belly and feels nauseated. The person is anxious and unable to carry out daily activities.	0.324 (0.219–0.442)

Severity	Distribution
Asymptomatic	0.356 (0.351–0.362)
Mild	0.326 (0.252–0.390)
Moderate	0.160 (0.117–0.208)
Severe	0.158 (0.123–0.193)

Injuries

Flowchart

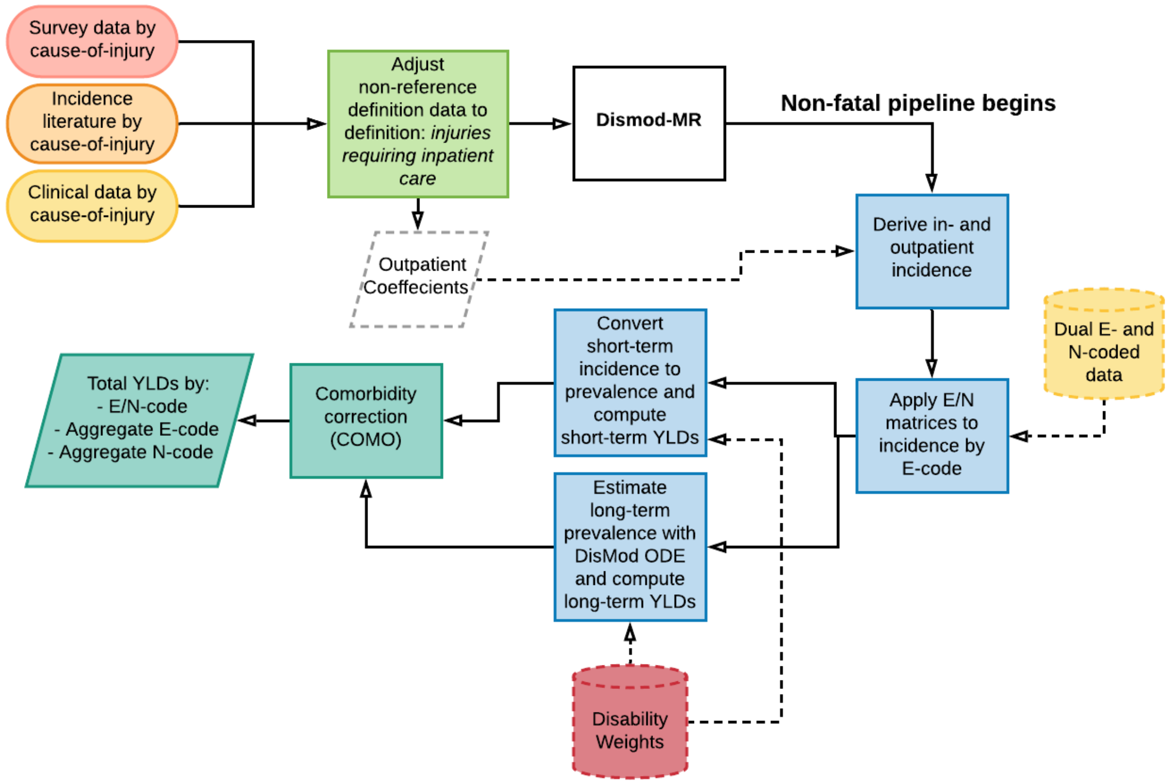

Case definition

For GBD 2021, the Injuries estimation process for non-fatal health outcomes encompasses a range of 29 causes, including transport injuries, falls, drowning, self-harm, interpersonal violence, and animal contact (excluding sexual violence, as described in a separate Appendix section). Injury incidence is defined using ICD-9 codes E000-E999 and ICD-10 chapters V to Y. Chapters S and T in ICD-10 and codes 800-999 in ICD9 are used to estimate morbidity. Fatal discontinuities, defined as changes in deaths due to sudden, unexpected spikes in mortality that depart from the underlying mortality trend, are also included in the injuries framework and include the following causes: police conflict and executions ("state actor violence"), conflict and terrorism, and exposure to forces of nature. Specific non-fatal methods for these causes are outlined further below.

Each of these 29 causes of injury can result in a variety of physical injury sequelae (eg, traumatic brain injury), which we call the "nature of injury." Although the initial models are at the "cause of injury" level (eg, drowning), each cause of injury is distributed into cause-nature pairs to capture the disability that develops from the resulting nature of injury. We report incidence, prevalence, and years lived with disability (YLDs) due to injuries at the cause-nature pair level.

We make additional distinctions between inpatient and outpatient injuries and between short-term and long-term injuries. Inpatient injuries are defined as injuries that lead to overnight hospitalisation, whereas outpatient injuries are defined as ones treated in outpatient settings or emergency care. We define short-term injuries as injuries lasting less than one year and long-term injuries as those lasting longer than one year, at which point we assume lifelong disability.

Input data

Model inputs

To estimate morbidity from injuries, we use data from hospital records, emergency department records, insurance claims, and population-representative surveys to produce YLDs by country, year, sex, age, external cause-of-injury, and nature-of-injury category. Many countries report hospital data using a mix of cause-of-injury and nature-of-injury codes. In order to retain as much of the data as possible, we include all datasets that had at least 15% of cases coded to the cause of injury. In GBD 2015, we chose 45% as the threshold but have since lowered the threshold to 15%. We made this distinction after assessing the proportions of major injury causes (road injuries and falls) in each of the data sources. We concluded that there were no obvious differences between country data with 15%–45% coverage of external cause codes and those with more than 45% coverage. Below the 15% threshold, the external cause of injury coding became more disproportionate when compared to sources with higher external cause of injury coding. We assessed the raw hospital data to make sure that there was no disproportionate coding to certain causes in the 15%–45% cause-of-injury coding range. We increased the cause-specific injury cases from these datasets proportionately to sum to the total number of injury cases.

Conflict, war and executions, and police conflict data are obtained from the Uppsala Conflict Data Program [2], the International Institute for Strategic Studies [3], the Armed Conflict Location and Event Dataset [4], the Social Conflict Analysis Database [5], the Global Terrorism Database [29] and vital

registration systems. Disaster data are obtained from the International Disaster Database from the Center for Research on the Epidemiology of Disasters [6]. Supplemental sources, such as collaborator accounts and news reports, are also used as sources for these causes.

Data searches

GBD 2021 utilised the same data as GBD 2019 [1] with some updates to existing data and additions of new data. For GBD 2021, hospital and emergency department records were supplemented with more recent and available site-years. GBD 2021 included more granular age groups under 5 years, and inpatient sources were reformatted to include the new age groups. We applied correction factors to account for repeat hospital visits within a three-month time window (derived from USA claims data) to the incidence estimates to avoid double-counting multiple health service contacts for the same injury (*Clinical data processing and estimation is described in detail in a separate section of this Appendix)*. We incorporated a correction for access to health care facilities to account for individuals who sustain an injury but do not have access to a hospital or health care facility. This correction is based on the Healthcare Access and Quality (HAQ) Index [28].

Infrequently, datapoints were marked as outliers when a datapoint did not follow the age or time pattern as expected based on subject-matter or in-country experts and/or if the incidence rate of people sustaining an injury from a certain cause of injury was not plausible based on subject-matter or in-country experts. Table 1 contains information about data coverage for each cause of injury, not including fatal discontinuities: state actor violence, exposure to forces of nature, and conflict and terrorism.

Table 1. Data inputs for injuries incidence modelling

**Cause**	**Total sources**	**Countries with data**
Road injuries	301	77
**Pedestrian road injuries**	**177**	**22**
Cyclist road injuries	186	22
**Motorcyclist road injuries**	**179**	**22**
Motor vehicle road injuries	187	22
**Other road injuries**	**174**	**18**
Other transport injuries	191	20
**Falls**	**234**	**40**
Drowning	204	27
**Fire, heat, and hot substances**	**217**	**34**
Poisonings	214	35
**Poisoning by carbon monoxide**	**163**	**20**

Poisoning by other means	165	21
**Exposure to mechanical forces**	**191**	**24**
Unintentional firearm injuries	187	20
**Other exposure to mechanical forces**	**190**	**23**
Adverse effects of medical treatment	346	49
**Animal contact**	**226**	**33**
Venomous animal contact	189	22
**Non-venomous animal contact**	**190**	**23**
Pulmonary aspiration and foreign body in airway	188	21
**Foreign body in eyes**	**196**	**20**
Foreign body in other body part	201	23
**Environmental heat and cold exposure**	**182**	**24**
Other unintentional injuries	168	20
**Self-harm**	**210**	**29**
Self-harm by firearm	164	18
**Self-harm by other specified means**	**166**	**21**
Interpersonal violence	209	32
**Physical violence by firearm**	**188**	**21**
Physical violence by sharp object	171	25
**Physical violence by other means**	**165**	**22**

Modelling strategy

As in previous GBD iterations, two categories of injury severity were separately modelled for each injury: injuries warranting inpatient care (inpatient) and injuries warranting other health care (outpatient). Injuries warranting inpatient care refer to injury cases of sufficient severity to require inpatient care if there are no restrictions in access to health care. Injuries warranting other health care refer to injury cases of sufficient severity to require health care attention but not hospitalisation. This category includes emergency department visits. To best measure the burden of injuries, the GBD 2021 estimates excluded trivial injuries by restricting morbidity analysis to cases warranting some form of health care in a system with full access to health care. We intended to include cases with injuries that did not receive care in areas with restricted access to health care but that would have warranted some type of health care in a system with full access to health care. In some surveys, after asking about recall of injuries in the past month or year, respondents were further probed on whether they sought care and why they did not. This allowed us to include cases who cited financial or geographical barriers as reasons for not seeking care.

Cause-of-injury incidence

The list of unique (ie, not counting aggregate categories like road injuries or interpersonal violence) cause-of-injury categories did not change from the 29 unique causes in GBD 2019 [1]. We treat police conflict and executions ("state actor violence") as a typical cause of injury rather than as a fatal discontinuity; however, the cause is modelled using the fatal discontinuity estimation strategy using incidence-to-mortality ratios because we do not have incidence data.

The majority of incidence data exist for external causes of injury. Incidence for cause-of-injury categories was modelled using Bayesian meta-regression method DisMod-MR 2.1 *(DisMod-MR 2.1 estimation is described in detail in a separate section of this Appendix)*. Multiple datasets from hospital and emergency/outpatient departments, insurance claims, and surveys were fed into these incidence models. We separately estimated two categories of injury severity: inpatient and outpatient injuries.

Excess mortality modelling (EMR)

In previous rounds, priors on excess mortality rate (EMR) were estimated in DisMod-MR 2.1 by matching prevalence datapoints with their corresponding CSMR values within the same age, sex, year, location (by dividing CSMR by prevalence). For many injuries, DisMod-MR 2.1 estimated a rather unrealistic pattern of EMR since we expect a pattern of decreasing EMR with greater access to quality health care. An example of this is shown in Figure 1 below. Such unexpected patterns often signal inconsistencies between CSMR estimates and the measures of prevalence and/or incidence. For injuries with large fatal and non-fatal inconsistencies, we implemented a new EMR modelling method described in the following section. These injuries included drowning, falls, poisoning by carbon monoxide, interpersonal violence, assault by firearm, assault by sharp object, assault by other means, self-harm, self-harm by firearm, other transport injuries, and all road injuries.

To provide greater guidance to DisMod-MR 2.1 on the expected pattern of EMR, EMR data generated through a previous iteration was modelled using the meta-regression—Bayesian, regularised, trimmed (MR-BRT) tool with age, sex, and HAQ Index as predictors *(MR-BRT is described in detail in a separate section of this Appendix)*. An upper bound of zero was included for HAQ Index based on the a priori assumption that greater access to healthcare leads to decreased mortality [29]. For violence by firearm, sharp object, and other means, the mortality rate of homicide by firearm was also included as a covariate, since we assume that country-years with higher rates of violence by firearm are prone generally to more fatal violence. Results from MR-BRT were then used to predict EMR for each location-year, sex and for ages 0, 10, 20 … 100. For the 16 injuries using EMR inputs modelled from MR-BRT, we set the trimming parameter to trim 10% of the datapoints, added a cubic-spline on age with knots set by data density, and a fixed effect on sex. The final MR-BRT predictions were then uploaded as EMR input data to DisMod-MR 2.1 models for these injuries.

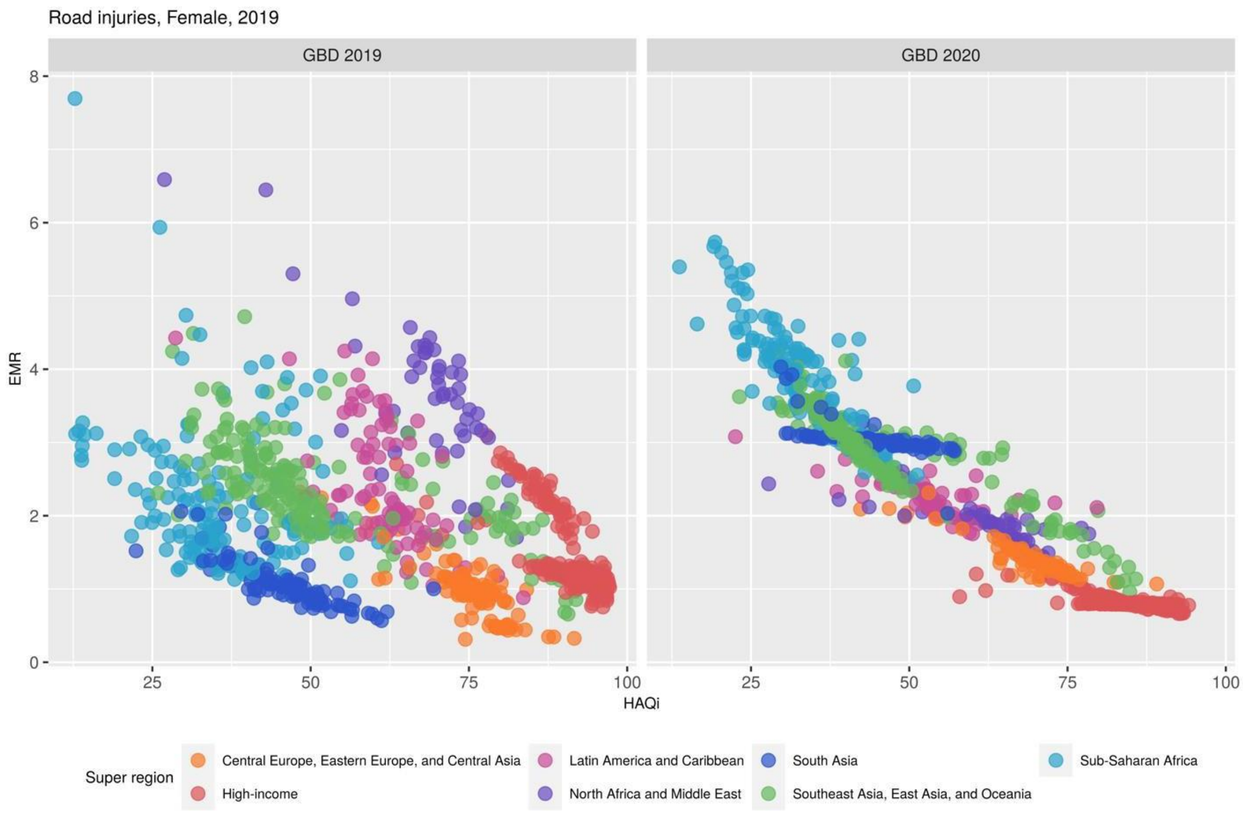

Figure 1. EMR estimates versus HAQ Index by location, for female road injuries DisMod-MR 2.1 results. The left plot shows the EMR estimates from DisMod-MR in GBD 2019, while the right plot shows the EMR estimates from DisMod-MR in GBD 2021 after implementing the new EMR method. On the left, South Asia is an example of a region in which locations are not following an expected pattern of health access versus EMR, in that it has lower estimates of EMR than some high-income locations. On the right, a more visible trend is shown between EMR and HAQ Index across all locations.

Adjusting data

For GBD 2021, we derived coefficients to adjust type of care (inpatient or outpatient only) and presence of care-seeking behaviour (care vs no care) to maximise data included in inpatient-only and outpatient-only models for every injury. This was performed out of DisMod-MR 2.1, using adjustment coefficients derived from a network analysis on World Health Survey data on road injuries spanning over 50 countries. First, ST-GPR was used to estimate the proportion of people who were able to receive care for their injuries using the ratio of individuals who received in- or outpatient care to all injured individuals who did or did not receive care. These proportions allowed us to adjust data to the definition "injuries that received inpatient or outpatient care." Then, MR-BRT was used to adjust "received care" incidence and outpatient incidence both to inpatient incidence using inpatient versus outpatient incidence comparisons from the United States National Hospital Ambulatory Medical Care Survey. This process is summarised in Figure 1, and an example of a MR-BRT output can be seen in Figure 2. Country-level covariates are shown in Table 2.

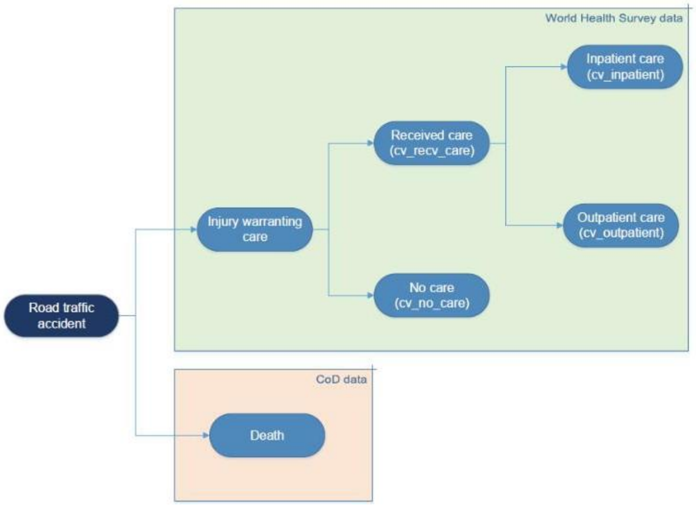

Figure 2. Overview of data adjustment process using road injuries data from World Health Survey data

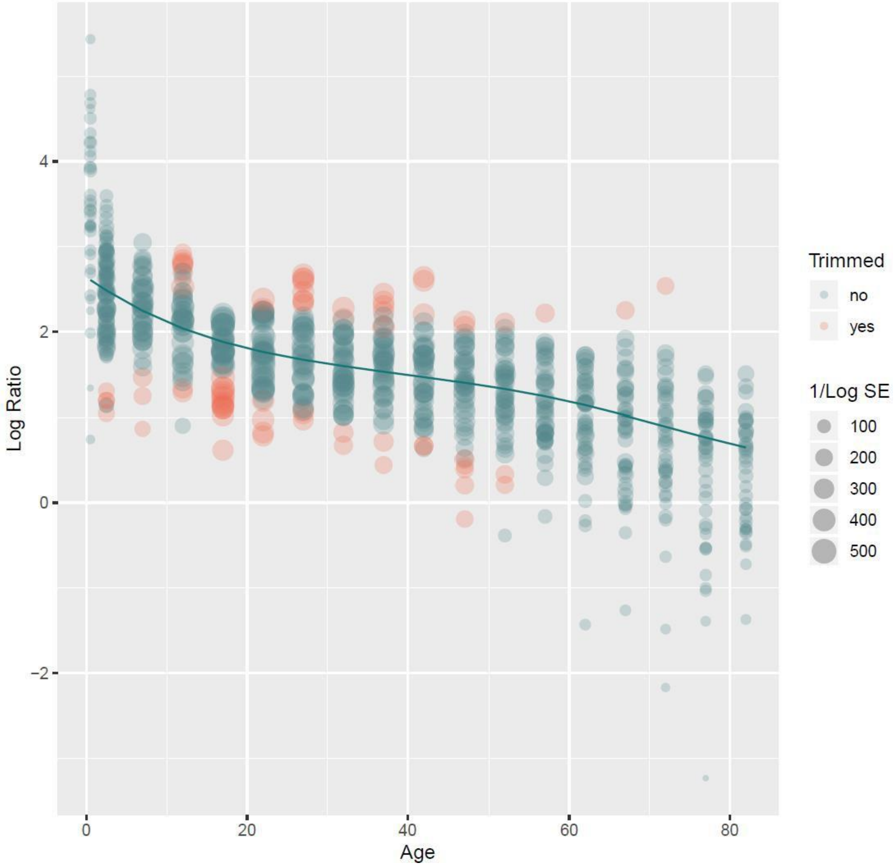

Figure 3. MR-BRT model for road injuries by age. The y-axis shows the log of the ratio of outpatient cases to inpatient cases for each age along the x-axis. This shows how outpatient or ED visits without admission are more probable per inpatient admission in younger ages, while in the oldest ages, it is less likely for a road injury case to be seen only as an outpatient relative to each observed inpatient admission. The red datapoints show data that were trimmed by MR-BRT. See Figures 5–16 for MR-BRT plots for other injuries.

Table 2. Country-level covariates for DisMod-MR 2.1 incidence models for injuries

Model	Covariate	Exponentiated Value
Road injuries (EMR)	Log-transformed age-standardised SEV scalar: Road Inj	3.48 (3.46 — 3.49)
	Vehicles - 2+4 wheels (per capita)	1.25 (1.23 — 1.27)
**Pedestrian road injuries by road vehicle (EMR)**	**Log-transformed age-standardised SEV scalar: Pedest**	**3.06 (2.66 — 3.45)**
Cyclist road injuries (EMR)	Log-transformed age-standardised SEV scalar: Cyclist	3.39 (3.16 — 3.49)
	Vehicles - 2+4 wheels (per capita)	1.25 (1.22 — 1.27)
**Motorcyclist road injuries (EMR)**	**Log-transformed age-standardised SEV scalar: Mot Cyc**	**3.33 (3.06 — 3.48)**
	Vehicles - 2 wheels (per capita)	1.72 (1.67 — 1.76)
Motor vehicle road injuries (EMR)	Log-transformed age-standardised SEV scalar: Mot Veh	2.19 (2.12 — 2.31)
	Vehicles - 4 wheels (per capita)	1.13 (1.10 — 1.15)
**Other road injuries (EMR)**	**Log-transformed age-standardised SEV scalar: Oth Road**	**2.17 (2.12 — 2.25)**
Other transport injuries (EMR)	Log-transformed age-standardised SEV scalar: Oth Trans	3.26 (2.84 — 3.48)
**Falls**	**Log-transformed age-standardised SEV scalar: Falls**	**3.48 (3.46 — 3.49)**
Drowning (EMR)	Log-transformed age-standardised SEV scalar: Drown	3.23 (2.83 — 3.48)
	Coastal Population within 10km (proportion)	1.37 (1.25 — 1.50)
**Fire, heat, and hot substances**	**Log-transformed age-standardised SEV scalar: Fire**	**3.42 (3.30 — 3.49)**
	Indoor Air Pollution (All Cooking Fuels)	0.70 (0.64 — 0.89)

Poisonings	Log-transformed age-standardised SEV scalar: Poison	3.38 (3.22 — 3.48)
**Poisoning by carbon monoxide (EMR)**	**Log-transformed SEV scalar: Poison**	**2.74 (2.22 — 3.34)**
Poisoning by other means	Log-transformed SEV scalar: Poison	3.21 (2.78 — 3.48)
**Exposure to mechanical forces**	**Log-transformed age-standardised SEV scalar: Mech**	**3.48 (3.47 — 3.49)**
Unintentional firearm injuries	Log-transformed age-standardised SEV scalar: Mech Gun	3.12 (2.66 — 3.47)
**Other exposure to mechanical forces**	**Log-transformed age-standardised SEV scalar: Oth Mech**	**3.45 (3.39 — 3.49)**
Adverse effects of medical treatment	Socio-demographic Index	1.65 (1.65 — 1.65)
**Animal contact**	**Log-transformed age-standardised SEV scalar: Animal**	**3.46 (3.41 — 3.49)**
	LDI (I$ per capita)	0.74 (0.74 — 0.74)
Venomous animal contact	Log-transformed age-standardised SEV scalar: Venom	2.17 (2.12 — 2.27)
**Non-venomous animal contact**	**Log-transformed age-standardised SEV scalar: Non Ven**	**3.47 (3.43 — 3.49)**
Pulmonary aspiration and foreign body in airway	Log-transformed age-standardised SEV scalar: F Body Asp	3.16 (2.64 — 3.48)
**Foreign body in eyes**	**—**	**—**
Foreign body in other body part	Log-transformed SEV scalar: Oth F Body	2.74 (2.25 — 3.27)
**Environmental heat and cold exposure**	**Population-weighted mean temperature**	**1.00 (1.00 — 1.01)**
	90th percentile climatic temperature in the given country-year.	2.63 (2.46 — 2.72)
Other unintentional injuries	Log-transformed age-standardised SEV scalar: Oth Unint	2.45 (2.15 — 2.90)
**Self-harm (EMR)**	**Log-transformed age-standardised SEV scalar: Self Harm**	**2.60 (2.42 — 2.80)**
Self-harm by firearm (EMR)	Log-transformed age-standardised SEV scalar: Self Other	2.25 (2.12 — 2.57)
**Self-harm by other specified means**	**Log-transformed age-standardised SEV scalar: Self Harm**	**3.41 (3.29 — 3.49)**
Interpersonal violence (EMR)	Log-transformed age-standardised SEV scalar: Violence	3.17 (2.94 — 3.39)
**Assault by firearm (EMR)**	**Log-transformed age-standardised SEV scalar: Viol Gun**	**2.64 (2.40 — 2.91)**

Assault by sharp object (EMR)	Log-transformed age-standardised SEV scalar: Viol Knife	2.26 (2.14 — 2.40)
**Assault by other means (EMR)**	**Log-transformed age-standardised SEV scalar: Oth Viol**	**3.43 (3.34 — 3.49)**

Incidence of fatal discontinuities

Due to the sporadic nature of the incidence of injuries and a lack of time trend that results from fatal discontinuities, DisMod-MR 2.1 was not used to model incidence due to fatal discontinuities, including state actor violence, exposure to forces of nature (ie, natural disaster), and conflict and terrorism. Instead, incidence-to-mortality ratios were averaged over super-region, year, and sex to limit the variability in the ratios applied to fatal discontinuities. For disaster incidence, the incidence-to-mortality ratio was calculated as an average of road injuries and drowning if there was a water-related natural disaster in that specific country-year noted in the International Disaster Database [6]. For conflict and terrorism, the incidence-to-mortality ratio was calculated as an average of the road injuries and interpersonal violence causes. We treated executions and police conflict as similar to the fatal discontinuities in that we imputed the incidence using the incidence-to-mortality ratio of interpersonal violence. These incidence-to-mortality ratios were applied to mortality estimates from shock events from the Cause of Death database and shocks modelling process to calculate fatal discontinuity injuries incidence.

Analysis to inform nature-of-injury category hierarchy and long-term probability of injuries

Similar to GBD 2019, we used follow-up data obtained from a pooled dataset of six follow-up studies from China, the Netherlands, and the US (see Table 3) [1]. These studies followed patients for at least one year after the injury. We also used the Medical Expenditure Panel Survey (MEPS) [7]. MEPS is a large-scale overlapping continuous panel survey of the US non-institutionalised population that collects information on use and cost of health care and SF-12 responses. SF-12 responses are elicited twice over the two-year period that any individual is part of the study. Thus, MEPS offered the benefit of including health state measures of non-injured and destined to be injured and the benefit of having pre-injury and post-injury SF-12 responses. We pooled all available MEPS data over a 19-year span.

The follow-up studies used different patient-reported outcome measures to assess health status, namely the SF-36, Version 1 SF-12, and the EQ-5D. To enable comparison across the six datasets, it was necessary to analyse the data in a standardised patient-reported outcome measure. First, we mapped all patient-reported outcome measures to Version 2 SF-12 (SF-12v2). Second, we normalised the health status measurements by mapping the SF-12 scores to a corresponding disability weight based on several opportunistic surveys asking respondents to score SF-12 based on the lay descriptions for a selection of 60 GBD health states. We ran a regression of logit-transformed disability weight on nature-of-injury category and age group and never-injured status. The pooled dataset informed both the nature-of-injury category hierarchy and the long-term probability of injuries, discussed below.

Table 3. Details of injury follow-up surveys used in GBD 2021

Dataset	Year	Type of data collected	Type of patients	Setting	Sample size* and response
**Guangdong follow-up survey, China[9]**	2006–2007	Follow-up survey among sample of ISS patients	Patients (15+ years) who were hospitalised that had been injured by road traffic injury, fall, blunt or penetrating trauma	Based on three national injury surveillance hospitals in Zhuhai, Guangdong Province in China	998 (response 87%)
LIS follow-up survey, Netherlands[10]	**2001–2002**	**Follow-up survey among stratified sample of ISS patients (oversampling less common, severe injuries)**	**Patients (15+ years) who visited the Emergency Department of a hospital and were discharged to the home environment and patients who were admitted to hospital**	**Based on 17 public hospitals in the Netherlands**	**8,564 (response 37%)**
**LIS follow-up survey, Netherlands[11]**	2007–2008	Follow-up survey among stratified sample of ISS patients (oversampling less common, severe injuries)	Patients (15+ years) who visited the Emergency Department of a hospital and were discharged to the home environment and patients who were admitted to hospital	Based on 15 public hospitals in the Netherlands	8,057 (response 36%)
NSCOT – National study on Costs and Outcomes of Trauma, USA[12]	**2001–2002**	**A prospective cohort study was conducted among a sample of adult trauma patients treated at Level I trauma centres and non-trauma centre hospitals**	**Patients treated for a moderate to severe injury (as defined by at least one injury of an Abbreviated Injury Scale (AIS) score of 3 or greater**	**Based on 69 hospitals in 12 states in the US**	**5,191 (response 61%)**
**SCTBIFR – South Carolina Traumatic Brain injury Follow-up Registry, USA[13]**	1999–2002	A prospective cohort study was conducted among injured in-patients with a traumatic brain injury-related injury	Patients (15+ years) who were admitted to hospitals and met the CDC case definition of TBI – trauma to the head associated with altered consciousness, amnesia, neurological abnormalities, skull fracture, intracranial lesion, or death	Discharged from all nonfederal in-state acute care hospitals	7,613 (response 28%)

Burns outcome study, Netherlands[14]	**2003–2006**	**A multicentre prospective cohort was conducted among adult (severe) burn patients**	**Injury patients who sustained severe burns**	**Three public hospitals with specialised burn units.**	**311 (response 78%)**

*number of patients that met the inclusion criteria; response rate = percentage of patients who responded to the follow-up survey (in case of multiple follow-up times the response rate of the first follow-up moment is reported).

Nature-of-injury category hierarchy

Multiple injuries can occur in one individual. For GBD 2021, a nature-of-injuries severity hierarchy was developed to establish a one-to-one relationship between cause-of-injury and nature-of-injury category. This means that in the case of multiple injuries the nature-of-injury category that was likely to be responsible for the largest burden was selected. To construct the hierarchy, we used data from the pooled dataset of follow-up studies [9–14]. The output of the regression of logit-transformed disability weight on nature-of-injury category and individual characteristics of the follow-up studies were used to calculate the mean long-term disability attributable to each nature-of-injury category. The ranking of nature-of-injury categories by their long-term disability weights formed the basis of our severity hierarchy. Hierarchies were developed separately, for injuries warranting inpatient care and injuries warranting other health care.

Table 4. Nature-of-injury hierarchies: combination of empirical hierarchies estimated from pooled follow-up studies and expert adjustments, for inpatient and outpatient injuries

Rank	Inpatient Hierarchy	Outpatient Hierarchy
1	Spinal cord lesion below neck level	Fracture of pelvis
**2**	**Amputation of lower limbs, bilateral**	**Fracture of patella, tibia or fibula, or ankle**
3	Amputation of upper limbs, bilateral	Fracture of hip
**4**	**Spinal cord lesion at neck level**	**Fracture of skull**
5	Fracture of hip	Amputation of thumb
**6**	**Fracture of femur, other than femoral neck**	**Fracture of vertebral column**
7	Amputation of upper limb, unilateral	Multiple fractures, dislocations, crashes, wounds, sprains, and strains
**8**	**Amputation of lower limb, unilateral**	**Internal haemorrhage in abdomen and pelvis**
9	Multiple fractures, dislocations, crashes, wounds, sprains, and strains	Fracture of femur, other than femoral neck
**10**	**Effect of different environmental factors**	**Dislocation of hip**
11	Fracture of patella, tibia or fibula, or ankle	Amputation of toe/toes
**12**	**Moderate-Severe traumatic brain injury**	**Fracture of hand (wrist and other distal part of hand)**
13	Fracture of foot bones except ankle	Amputation of fingers (excluding thumb)
**14**	**Internal haemorrhage in abdomen and pelvis**	**Burns, <20% of total burned surface area without lower airway burns**
15	Crush injury	Dislocation of knee
**16**	**Minor traumatic brain injury**	**Contusion in any part of the body**
17	Fracture of pelvis	Minor traumatic brain injury
**18**	**Nerve injury**	**Foreign body in respiratory system**
19	Severe chest injury	Severe chest injury

**20**	**Dislocation of hip**	**Drowning and non-fatal submersion**
**21**	Burns, >= 20% total burned surface area or >= 10% burned surface are if head/neck or hands/wrist involved w/o lower airway burns	Asphyxiation
**22**	**Lower airway burns**	**Poisoning requiring urgent care**
**23**	Fracture of skull	Effect of different environmental factors
**24**	**Amputation of thumb**	**Foreign body in GI and urogenital system**
**25**	Fracture of hand (wrist and other distal part of hand)	Fracture of sternum and/or fracture of one or more ribs
**26**	**Fracture of vertebral column**	**Nerve injury**
**27**	Contusion in any part of the body	Fracture of face bones
**28**	**Open wound(s)**	**Dislocation of shoulder**
**29**	Amputation of toe/toes	Injury to eyes
**30**	**Dislocation of knee**	**Fracture of clavicle, scapula, or humerus**
**31**	Amputation of fingers (excluding thumb)	Fracture of radius and/or ulna
**32**	**Drowning and non-fatal submersion**	**Fracture of foot bones except ankle**
**33**	Asphyxiation	Foreign body in ear
**34**	**Burns, <20% total burned surface area without lower airway burns**	**Muscle and tendon injuries, including sprains and strains lesser dislocations**
**35**	Muscle and tendon injuries, including sprains and strains lesser dislocations	Superficial injury of any part of the body
**36**	**Fracture of face bones**	**Open wound(s)**
**37**	Foreign body in respiratory system	Complications following therapeutic procedures
**38**	**Poisoning requiring urgent care**	
**39**	Foreign body in GI and urogenital system	
**40**	**Fracture of sternum and/or fracture of one or more ribs**	
**41**	Dislocation of shoulder	
**42**	**Injury to eyes**	
**43**	Fracture of clavicle, scapula, or humerus	
**44**	**Fracture of radius and/or ulna**	
**45**	Foreign body in ear	
**46**	**Superficial injury of any part of the body**	
**47**	Complications following therapeutic procedures	

Cause-nature matrices

Because injury disability is linked more to the nature of injury than to the cause of injury, matrices were generated to map the proportion of each cause-of-injury category that results in a particular nature-of-injury category. These matrices are based on a collection of dual-coded (ie, both cause-of-injury and nature-of-injury coded) hospital and emergency department datasets [27]. The data for this step came from inpatient, outpatient, and emergency room discharge data from Argentina, Brazil, Bulgaria, China, Chile, Colombia, Cyprus, Czech Republic, Denmark, Egypt, Estonia, Georgia, Great Britain, Hungary, Iceland, Iran, Italy, India, Kyrgyzstan, Latvia, Malta, Mauritius, Mexico, Mozambique, Netherlands, New Zealand, Norway, Philippines, Portugal, Slovenia, Spain, Sweden, Macedonia, Uganda, United States, and

Zambia. We applied our nature-of-injury severity hierarchy above to assert that every observation had one cause of injury and one nature of injury.

Dirichlet models were used to estimate all the nature-of-injury category proportions for one cause of injury simultaneously. These models allow for consistent borrowing of information across age, sex, inpatient/outpatient, and high/low-income countries and assert that the nature-of-injury proportions within a cause-of-injury category must add up to 1. One cause-nature matrix was created for each combination of injury warranting hospital admission versus injury warranting other health care, high/low-income countries (a binary variable based on GBD super-region), male/female, and age category. Applying these matrices to our cause-of-injury incidence from DisMod-MR 2.1, we produced cases of injury warranting hospital admission and incidence of injury warranting other health care by cause and nature of injury. For causes that are subsets of other causes (child and parent causes), the cause-nature matrix was applied directly to the child causes. Afterward, the incidences of the child cause-nature combinations were scaled to sum to the incidence of the parent cause.

<u>Probability of permanent health loss</u>

Disability due to injury was assumed to affect all cases in the short-term with a proportion having long-term (permanent) outcomes. The probability of long-term outcomes was needed to estimate the incidence and subsequently the prevalence of cases with permanent health loss. In our conceptual model, individuals who suffer from a non-fatal injury will, in the long-term, return to either full or partial health. If one-year post-injury patients return to a health status with more disability than their pre-injury health status, injury patients are assumed to have permanent disability from their injury. The difference between the pre-injury health states and health status one year after injury is assumed to be their permanent level of injury-related disability. We assessed the probability of developing permanent health loss using the pooled dataset of follow-up studies [9–14] and the MEPS [7] that were also used to generate the nature-of-injury hierarchy. To assess the probability of permanent health loss, we estimated the effects using a logit-linear mixed effects regression:

$$\begin{aligned} Logit(DW)_{im} = \alpha &+ \beta(injuries_{im}) + \beta(never\ injured_i) + \beta(never\ injured_i * age_i) \\ &+ \beta(fracture\ of\ pelvis_i) + \beta(fracture\ of\ pelvis_i * age_i) + \beta(poisoning_i * age_i) \\ &+ \beta(moderate\ to\ severe\ TBI_i * age_i) + RE_c + RE_i \end{aligned}$$

where we included dummies for all the nature-of-injury categories ($injuries_{im}$), with the reference category being no injury (from MEPS dataset). We also included a dummy for never injured prior to the current injury, age, interactions between age and never-injured status, and interactions with three long-term nature-of-injury categories that were found to significantly vary with age: pelvis fractures, poisonings, and moderate/severe traumatic brain injuries. In notation, subscript m refers to patient-reported outcome measure, i refers to individual, and c refers to country. Random effects (RE) were included to control for variation between countries and individuals.

After predicting overall disability at one-year follow-up, we estimated a counterfactual by setting all observations to "no injury," the reference group for $\beta(injuries_{im})$ in our model. The disability attributable to the nature of injury at one year was assumed to be the difference between our counterfactual of no injury and predicted disability with injury. The probability of treated long-term outcomes was estimated via the ratio of this attributable disability relative to the long-term disability weight for that injury.

$$Probability\ of\ long-term\ disability = \frac{with\ injury\ disability_{im} - counterfactual\ disability_{im}}{DW_m}$$

We developed estimates of the probability of permanent health loss by nature-of-injury category, injury severity level (injuries warranting inpatient admission and injuries warranting other health care), and age. These probabilities are shown in Figure 3 for three selected age groups (25-30, 50-55, 75-80) and selected nature-of-injury categories by inpatient and outpatient. Moderate-severe TBI and spinal cord lesions only have inpatient injury long-term probabilities, and nerve injury, open wounds, and severe chest injury have long-term probabilities of zero for outpatient cases.

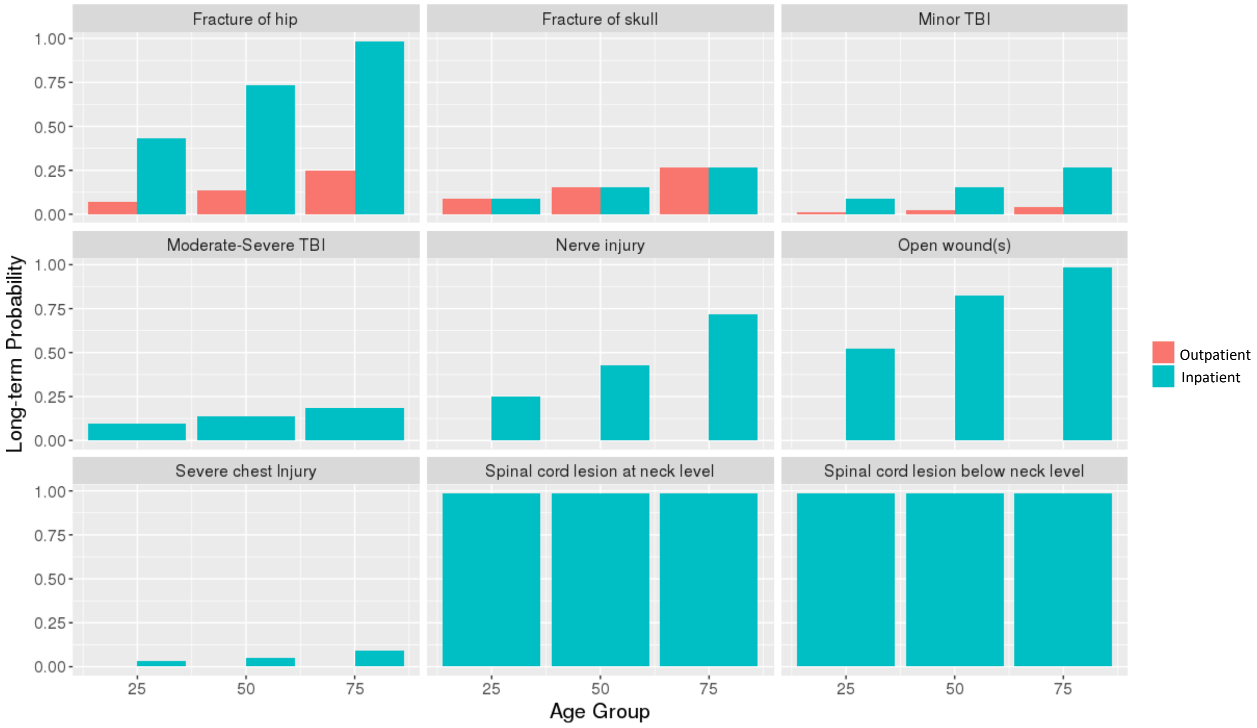

Figure 4. Long-term probabilities derived from the MEPS data for selected nature of injuries and age groups

Disability associated with treated and untreated cases

For many nature-of-injury categories, a separate disability weight is used for treated versus untreated cases. To estimate the percent treated for injuries in a given location-year, we used the Healthcare Access and Quality (HAQ) Index [28] with the same strategy described for the probability of permanent health loss. We chose a reasonable cutoff for the HAQ Index (75 on a scale of 0 – 100) as the threshold at and above which 100% of injuries were treated. This value captured most OECD countries for all years back to 1980. We then scaled all remaining location-years between 10% and 100% treated based on their HAQ Index value and used that as the percent treated in a given location-year. This was done at the draw level to propagate uncertainty. We made the decision to ignore any long-term disability from injuries with implausibly high estimates of long-term disability.

Custom disability weight adjustments

Traumatic brain injury (TBI)

An analysis was performed to create two custom combined disability weights (DWs) for long-term traumatic brain injury (TBI) from the GBD 2013 minor, moderate, and severe long-term TBI DWs [30]. Minor long-term TBI is defined as episodes of headaches, memory problems, and difficulty concentrating, moderate TBI also includes dizziness and anxiety, and severe TBI includes dependence on others. Custom weights were computed for two reasons: First, while mild TBI can be isolated using ICD codes, there was no meaningful way to distinguish between moderate and severe TBI within ICD codes, which would have been necessary for the E-N matrix of the injuries pipeline. Second, the severity of an incident case of TBI might not necessarily align with the severity of the long-term outcome. For instance, a case of TBI categorized as minor after the incident could lead to moderate or severe long-term outcomes. Data from a follow-up study of TBI patients [31] detailed by severity of TBI incident as well as severity of long-term outcomes were used to inform logit models that estimated the proportion of minor incident TBI (N27) and moderate/severe incident TBI (N28) cases that resulted in minor, moderate, and severe long-term outcomes. The logit models' distributions of outcome severity of the initial TBI incident were then used to create new weighted combinations of the minor, moderate, and severe long-term TBI DWs, producing two custom DWs for the Minor TBI n-code (N27) and Moderate/Severe TBI n-code (N28), shown in the table below. These custom DWs were only applied to the proportion of TBI cases estimated to have long-term outcomes.

For example, from the described analysis we found that out of all minor TBI incident cases with long-term outcomes, approximately 77% of those long-term outcomes were minor, approximately 21% were moderate, and approximately 2% were severe. So, the combined DW for minor TBI (N27) would be weighted as 77% of the original minor long-term TBI DW, 21% of the original moderate long-term TBI DW, and 2% of the original severe long-term TBI DW.

Table 5. Disability weights for long-term TBI, before and after custom adjustment

	Minor TBI, long-term	Moderate TBI, long-term	Severe TBI, long-term
**Original DW**	0.094 (0.063-0.133)	0.231 (0.156-0.324)	0.637 (0.462-0.786)
**Combined DW**	0.132 (0.090-0.182)	0.164 (0.112-0.226)	

Spinal cord injury (SCI)

Spinal cord lesions are grouped into two nature of injuries (n-codes): lesions at the neck level (N33), and lesions below the neck level (N34), where neck level is defined as at the level of the cervical spinal cord. To determine the disability weight of each of these n-codes, different levels of severity and their frequency were accounted for. Data was used from a study [32] that reported on the distribution of spinal cord injuries by their severity after 1 year of recovery, with severity graded according to the American Spinal Injury Association (ASIA) Impairment Scale score. The frequency of each grade of severity after 1 year was calculated. Each grade of severity was assigned two corresponding disability weight, one for a treated injury and one for an untreated injury. A grade of E was treated as having full health (a disability weight of 0).

Table 6. ASIA Impairment Scale score and proportions mapped to GBD health state descriptions for long-term treated spinal cord injuries

ASIA Impairment Scale Score after 1 year	Proportion after 1 year	GBD Health state lay description (at neck level, treated)	GBD Health State lay description (below neck level, treated)
A	50.2%	is paralyzed from the neck down, with no feeling or control over any part of the body below the neck, and no urine or bowel control.	is paralyzed from the waist down, cannot feel or move the legs and has difficulties with urine and bowel control. The person uses a wheelchair to move around.
B	7.4%	is paralyzed from the neck down and cannot feel or move the arms and legs.	is paralyzed from the waist down and cannot feel or move the legs. The person uses a lightweight and comfortable wheelchair to move around.
C	14.0%	is paralyzed from the neck down and cannot feel or move the arms and legs.	is paralyzed from the waist down and cannot feel or move the legs. The person uses a lightweight and comfortable wheelchair to move around.
D	27.1%	has some difficulty in moving around, and difficulty in lifting and holding objects, dressing	has some difficulty in moving around, and difficulty in lifting and holding objects, dressing and

		and sitting upright, but is able to walk without help	sitting upright, but is able to walk without help.
E	1.3%	N/A	N/A

Table 7. ASIA Impairment Scale score and proportions mapped to GBD health state descriptions for long-term untreated spinal cord injuries

ASIA Impairment Scale Score after 1 year	Proportion after 1 year	GBD Health state lay description (at neck level, untreated)	GBD Health State lay description (below neck level, untreated)
A	50.2%	is paralyzed from the neck down, with no feeling or control over any part of the body below the neck, and no urine or bowel control. Arms and legs are in fixed, bent positions, and the person gets frequent infections and pressure sores.	is paralyzed from the waist down, cannot feel or move the legs and has difficulties with urine and bowel control. Legs are in fixed, bent positions, and the person gets frequent infections and pressure sores.
B	7.4%	is paralyzed from the neck down and cannot feel or move the arms and legs. Arms and legs are in fixed, bent positions, and the person gets frequent infections and pressure sores.	is paralyzed from the waist down and cannot feel or move the legs. Legs are in fixed, bent positions, and the person gets frequent infections and pressure sores.
C	14.0%	is paralyzed from the neck down and cannot feel or move the arms and legs. Arms and legs are in fixed, bent positions, and the person gets frequent infections and pressure sores.	is paralyzed from the waist down and cannot feel or move the legs. Legs are in fixed, bent positions, and the person gets frequent infections and pressure sores.
D	27.1%	has some difficulty in moving around, and difficulty in lifting and holding objects, dressing and sitting upright, but is able to walk without help	has some difficulty in moving around, and difficulty in lifting and holding objects, dressing and sitting upright, but is able to walk without help.
E	1.3%	N/A	N/A

Afterward, a weighted average of these disability weights was calculated based on the frequency of each grade of severity, and used as the final disability weight. This process for calculating a final disability weight was conducted separately for lesions at versus below neck level.

Table 8. Disability weights associated with long-term SCI

Health state	Disability weight
Spinal cord lesion at neck level (treated)	0.589 (0.415-0.748)
Spinal cord lesion at neck level (untreated)	0.732 (0.544-0.871)
Spinal cord lesion below neck level (treated)	0.296 (0.198-0.414)
Spinal cord lesion below neck level (untreated)	0.623 (0.434-0.777)

Duration of short-term health loss

To determine the duration for treated cases of short-term injury, we analysed patient responses from two Dutch Injury Surveillance System follow-up studies conducted from 2001–2003 and 2007–2009 [8]. These studies collected data at 2.5, 5, 9, and 24 months post-injury to determine whether injury patients were still experiencing problems due to their injury. If not, the patients were asked how many days they had experienced problems. The injury patients that still reported having problems one year after the injury were assumed to be captured in our analysis of permanent disability. The duration for treated cases of short-term injury was estimated for injuries warranting inpatient admission and injuries warranting other health care separately. The estimates were supplemented by expert-driven estimates of short-term duration for nature-of-injury categories that did not appear in the Dutch dataset and untreated injuries.

Calculation of prevalence from incidence data – short-term injury

For short-term injury outcomes, which were assumed to be less than one year in duration, the prevalence for each cause-of-injury/nature-of-injury/severity-level grouping was approximated by the incidence for that grouping multiplied by the associated nature-of-injury/severity-level-specific duration.

Calculation of prevalence from incidence data – permanent health loss

For permanent health loss, we assumed no remission and thus integrated incidence over time to arrive at prevalence estimates. We used DisMod-MR ODE (ie, the “engine” of DisMod-MR 2.1) to carry out this integration for each combination of cause of injury and nature of injury by country, year, and sex. For this step we used random effects meta-analysis to pool data on standardised mortality ratios derived from literature reviews for spinal cord injury, burns covering more than 20% of the body, moderate to severe traumatic brain injury, hip fracture, and multiple significant injuries [14–26]. Here we include examples of these meta-analyses: hip fractures and traumatic brain injuries.

Figure 5. Meta-analyses of standardised mortality ratios derived from literature reviews: hip fractures and traumatic brain injury

For all other nature-of-injury categories, we assumed no long-term excess mortality. For the incidence estimates derived from fatal discontinuities – "exposure to forces of nature" and "conflict and terrorism" – we did not use DisMod-MR 2.1 as discontinuities by definition violate the assumption of a steady state in DisMod-MR 2.1 to estimate prevalence from incidence. For these two cause-of-injury categories, we coded the differential equations from DisMod ODE that determine the relationship between incidence, remission, mortality risk, and prevalence into Python and streamed out the prevalence from the incidence in the years of war or disaster by integrating over one year at a time.

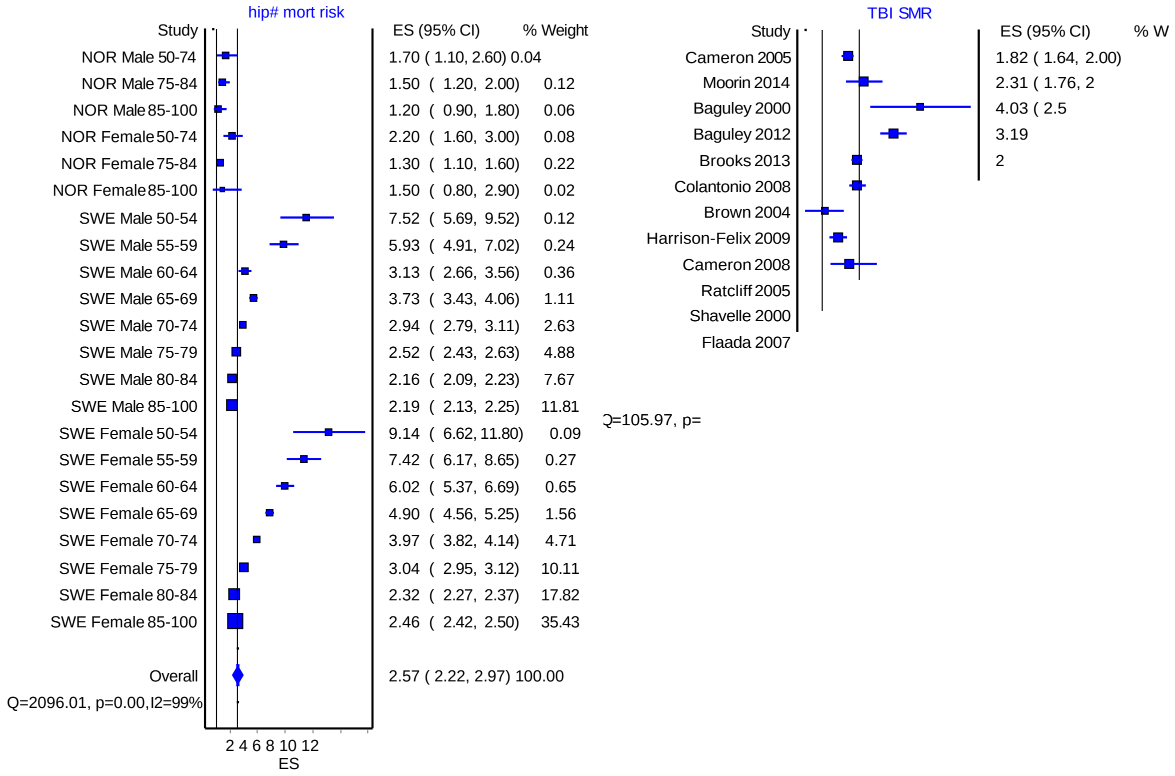

MR-BRT models (continued)

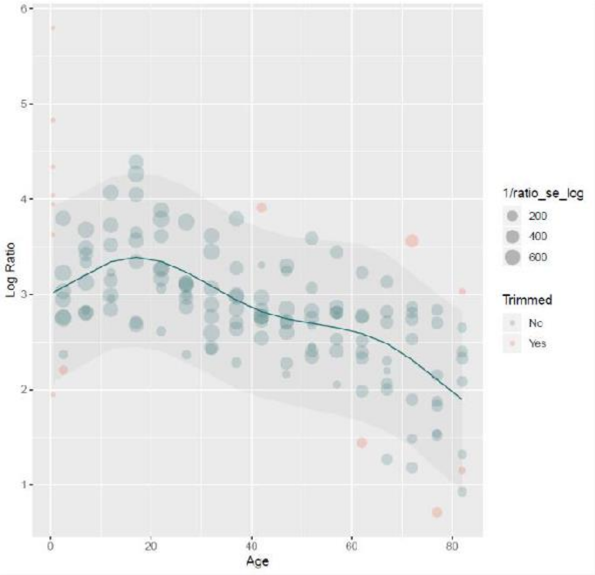

Figure 6. MR-BRT model for animal contact

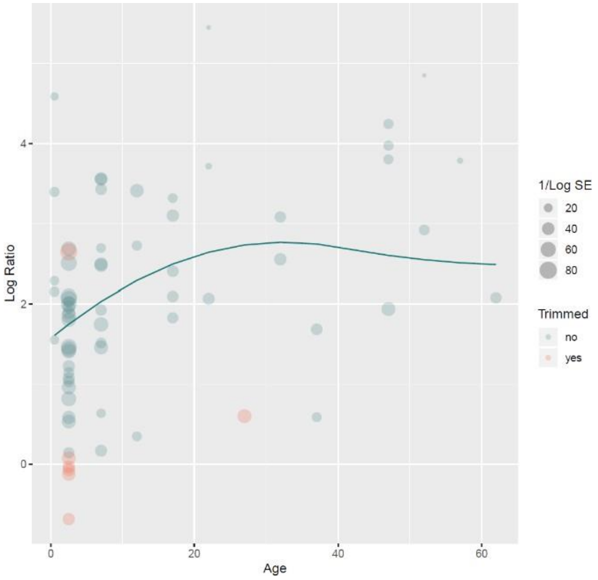

Figure 7. MR-BRT model for drowning

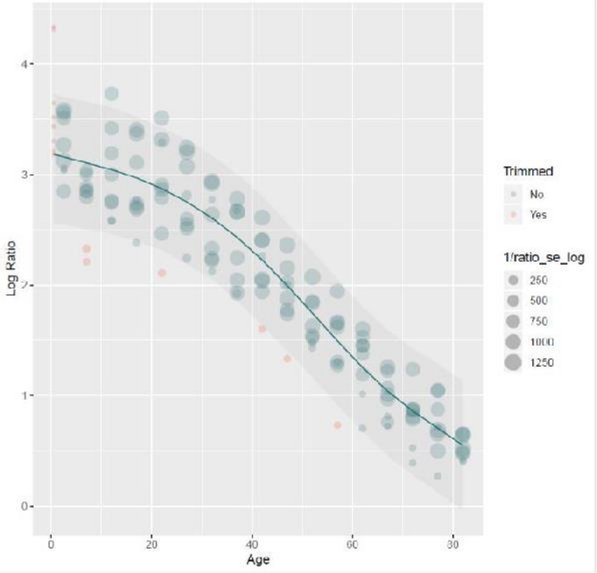

Figure 8. MR-BRT model for falls

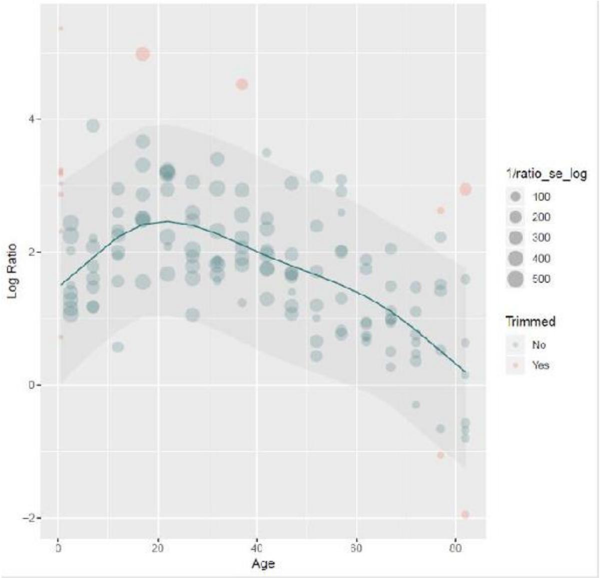

Figure 9. MR-BRT model for fire, heat, and hot substances

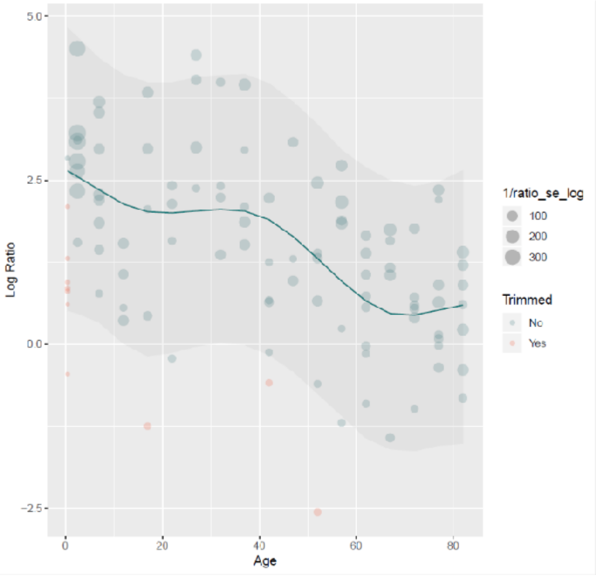

Figure 10. MR-BRT model for pulmonary aspiration and foreign body in airway

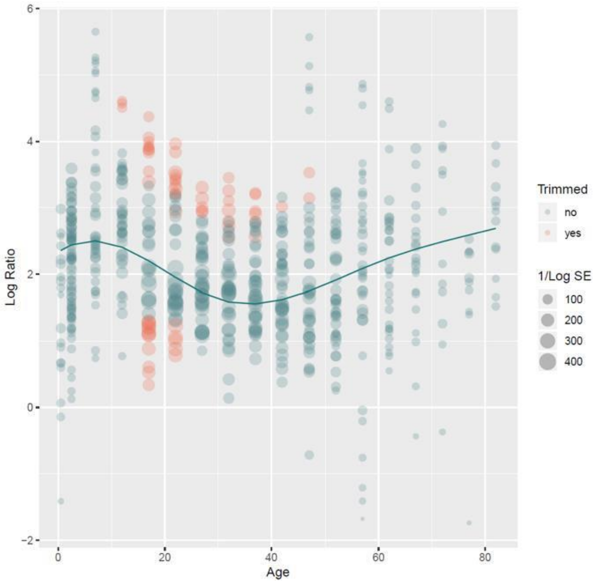

Figure 11. MR-BRT model for interpersonal violence

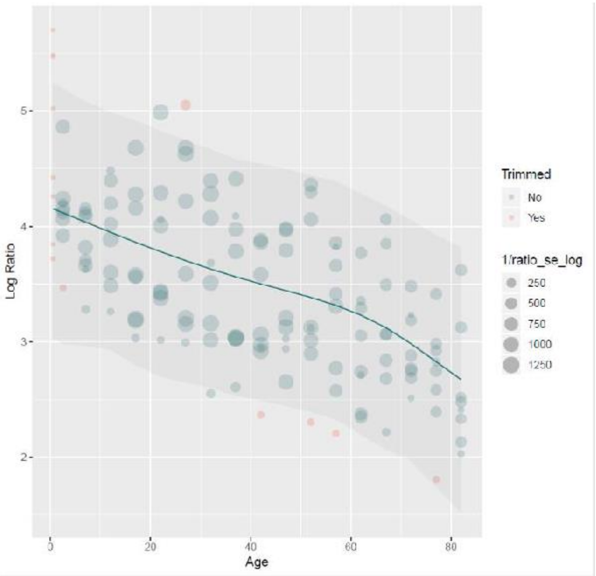

Figure 12. MR-BRT model for exposure to mechanical forces

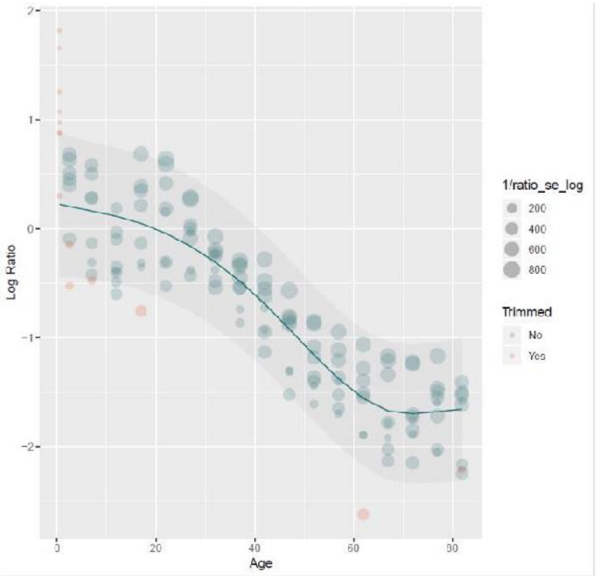

Figure 13. MR-BRT model for adverse effects of medical treatment

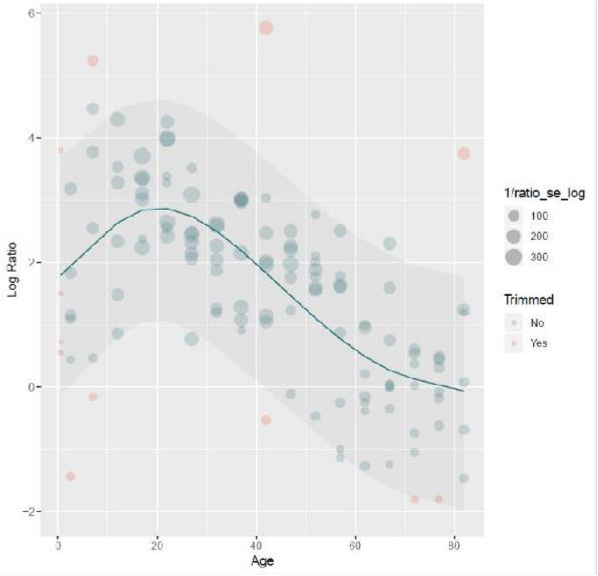

Figure 14. MR-BRT model for exposure to forces of nature

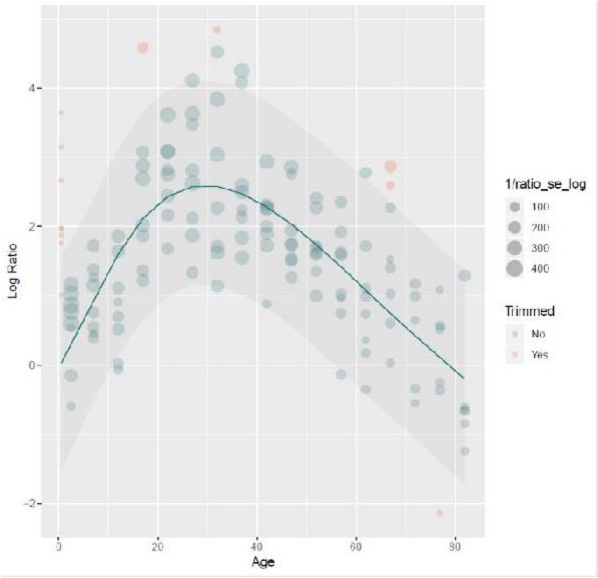

Figure 15. MR-BRT model for poisonings

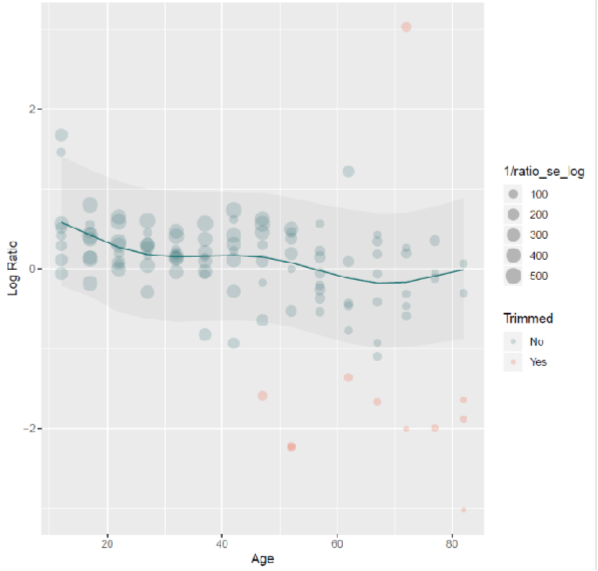

Figure 16. MR-BRT model for self-harm

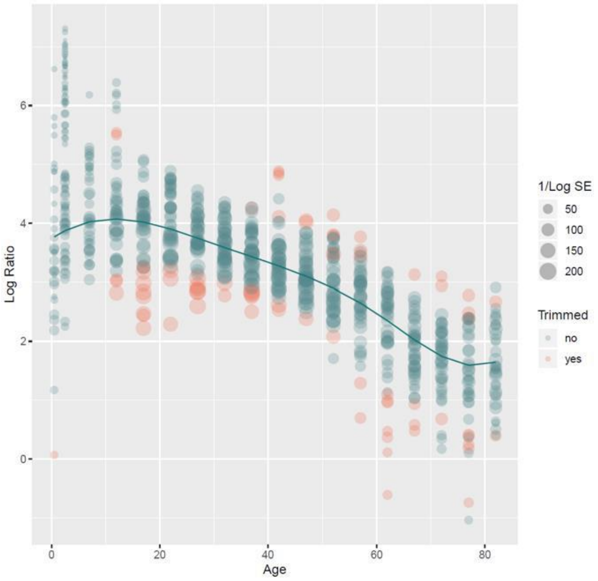

Figure 17. MR-BRT model for other unintentional injuries

References

1. Global burden of 369 diseases and injuries in 204 countries and territories, 1990–2019: a systematic analysis for the Global Burden of Disease Study 2019. Vos, Theo et al. The Lancet, Volume 396, Issue 10258, 1204 – 1222.
2. Department of Peace and Conflict Research, Uppsala University. UCDP Nonstate Conflict Dataset. Uppsala, Sweden: Department of Peace and Conflict Research, Uppsala University.
3. International Institute for Strategic Studies. International Institute for Strategic Studies Armed Conflict Database. London, United Kingdom: International Institute for Strategic Studies.
4. Climate Change and African Political Stability Project (CCAPS). Armed Conflict Location and Event Dataset, Realtime - Robert S. Strauss Center as referenced in Raleigh, Clionadh, Andrew Linke, Havard Hegre and Joakim Karlsen. 2010. Introducing ACLED-Armed Conflict Location and Event Data. Journal of Peace Research 47(5), 1-10.
5. Salehyan I, Hendrix CS, Hamner J, Case C, Linebarger C, Stull E, Williams J, Robert S. Strauss Center for International Security and Law. Social Conflict in Africa: A New Database. Int Interact. 2012; 38(4): 503-511.
6. Centre for Research on the Epidemiology of Disasters (CRED). EM-DAT: The OFDA/CRED International Disaster Database. Brussels, Belgium: Catholic University of Leuven.
7. Agency for Healthcare Research and Quality. United States Medical Expenditure Panel Survey. Rockville, United States: Agency for Healthcare Research and Quality.
8. Polinder S, van Beeck EF, Essink-Bot ML, Toet H, Looman CW, Mulder S, Meerding WJ. Functional outcome at 2.5, 5, 9, and 24 months after injury in the Netherlands. J Trauma. 2007; 62(1): 133-41.
9. Chinese Center for Disease Control and Prevention (CCDC). China Zhuhai Study 2006-2007 - China CDC.
10. Consumer Safety Institute (Netherlands). Netherlands Injury Surveillance System 2002.
11. Consumer Safety Institute (Netherlands). Netherlands Injury Surveillance System 2008.
12. Mackenzie EJ, Rivara FP, Jurkovich GJ, et al. The National Study on Costs and Outcomes of Trauma. J Trauma 2007; 63: S54-67; discussion S81-86.
13. CDC, Medical University of South Carolina, South Carolina Department of Disabilities and Special Needs, South Carolina Department of Health and Environmental Control. South Carolina Traumatic Brain Injury Follow-up Registry 1999-2013. USA.
14. van Loey NE, van Beeck EF, Faber BW, van de Schoot R, Bremer M. Health-Related Quality of Life After Burns: A Prospective Multicentre Cohort Study With 18 Months Follow-Up. J Trauma. 2011; 72(2): 513-520.
15. Strauss D, Shavelle R, DeVivo MJ, Day S. An analytic method for longitudinal mortality studies. J Insur Med 2000; 32: 217–25.
16. Shavelle R, Strauss D. Comparative mortality of adults with traumatic brain injury in California, 1988–97. J Insur Med 2000; 32: 163–6.
17. Baguley IJ, Nott MT, Howle AA, et al. Late mortality after severe traumatic brain injury in New South Wales: a multicentre study. Med J Aust 2012; 196: 40–5.
18. Middleton JW, Dayton A, Walsh J, Rutkowski SB, Leong G, Duong S. Life expectancy after spinal cord injury: a 50-year study. Spinal Cord 2012; 50: 803–11.
19. Brooks JC, Strauss DJ, Shavelle RM, Paculdo DR, Hammond FM, Harrison-Felix CL. Long-term disability and survival in traumatic brain injury: results from the National Institute on Disability and Rehabilitation Research Model Systems. Arch Phys Med Rehabil 2013; 94: 2203–9.
20. Baguley I, Slewa-Younan S, Lazarus R, Green A. Long-term mortality trends in patients with traumatic brain injury. Brain Inj 2000; 14: 505–12.

21. Ratcliff G, Colantonio A, Escobar M, Chase S, Vernich L. Long-term survival following traumatic brain injury. Disabil Rehabil 2005; 27: 305–14.
22. Frankel HL, Coll JR, Charlifue SW, et al. Long-term survival in spinal cord injury: a fifty year investigation. Spinal Cord 1998; 36: 266–74.
23. Harrison-Felix CL, Whiteneck GG, Jha A, DeVivo MJ, Hammond FM, Hart DM. Mortality over four decades after traumatic brain injury rehabilitation: a retrospective cohort study. Arch Phys Med Rehabil 2009; 90: 1506–13.
24. Moorin R, Miller TR, Hendrie D. Population-based incidence and 5-year survival for hospital-admitted traumatic brain and spinal cord injury, Western Australia, 2003-2008. J Neurol 2014; 261: 1726–34.
25. Colantonio A, Escobar MD, Chipman M, et al. Predictors of postacute mortality following traumatic brain injury in a seriously injured population. J Trauma 2008; 64: 876–82.
26. Flaada JT, Leibson CL, Mandrekar JN, et al. Relative risk of mortality after traumatic brain injury: a population-based study of the role of age and injury severity. J Neurotrauma 2007; 24: 435–45.
27. GBD 2016 Traumatic Brain Injury and Spinal Cord Injury Collaborators. Global, regional, and national burden of traumatic brain injury and spinal cord injury, 1990–2016: a systematic analysis for the Global Burden of Disease Study 2016. The Lancet Neurology. 26 Nov 2018.
28. GBD 2017 SDG Collaborators. Measuring progress from 1990 to 2017 and projecting attainment to 2030 of the health-related Sustainable Development Goals for 195 countries and territories: a systematic analysis for the Global Burden of Disease Study 2017. The Lancet. 8 Nov 2018; 392:2091–138.
29. National Consortium for the Study of Terrorism and Responses to Terrorism (START). Global Terrorism Database. College Park , MD, United States of America: University of Maryland, 2018.
30. Salomon JA, Haagsma JA, Davis A, Noordhout CM de, Polinder S, Havelaar AH, et al. Disability weights for the Global Burden of Disease 2013 study. The Lancet Global Health. 2015 Nov 1;3(11):e712–23.
31. Radboud University Medical Center. Netherlands Radboud University Brain Injury Cohort Study 1998-2011.
32. Marino RJ, Ditunno JF, Donovan WH, Maynard F. Neurological Recovery After Traumatic Spinal Cord Injury: Data From the Model Spinal Cord Injury Systems. Arch Phys Med Rehabil 1999;80:1391-6.

Interstitial lung disease and pulmonary sarcoidosis (ILD)

Flowchart

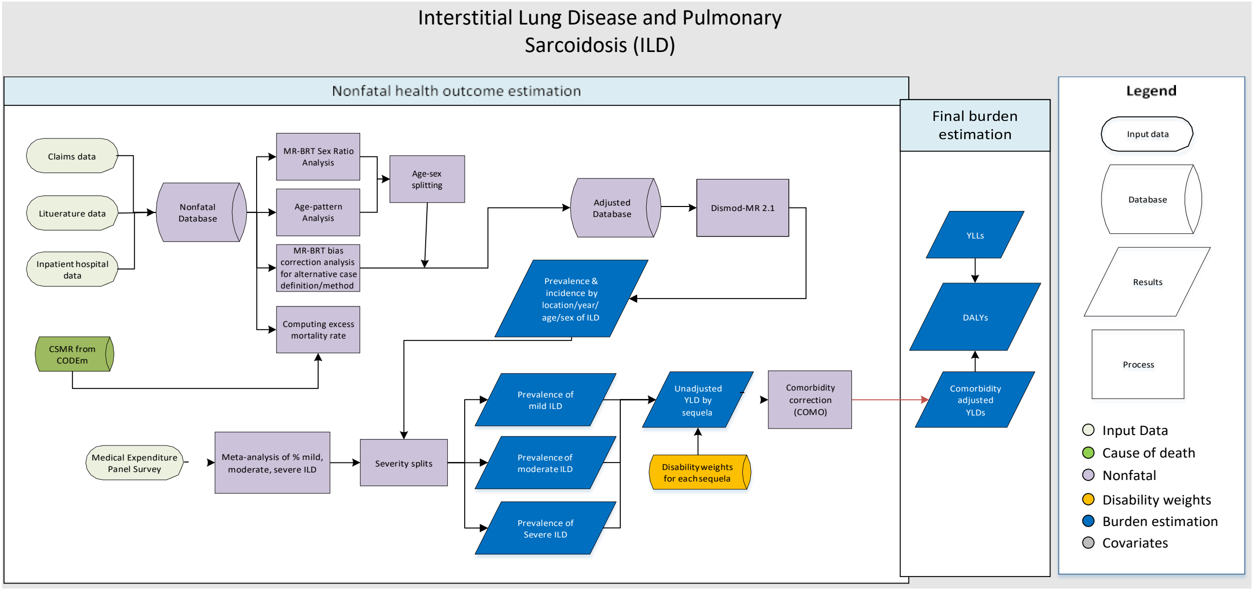

Case definition

Interstitial lung diseases and pulmonary sarcoidosis are a collection of chronic respiratory diseases that impair lung function and oxygen uptake through scarring and/or inflammation. The relevant ICD codes are D86 and J84. For interstitial lung disease, we use the American Thoracic Society as the gold standard definition.

Input data

Model Inputs

No systematic review of the literature was conducted for ILD for the Global Burden of Disease 2021 study. These reviews are done on a rotating basis, and updates will be made for a future iteration. The last systematic review was conducted for GBD 2017 and used the following search string: ("lung diseases, interstitial"[MeSH Terms] OR "sarcoidosis, pulmonary"[MeSH Terms] OR "idiopathic pulmonary fibrosis"[MeSH Terms]) AND (prevalence[Ti] OR incidence[Ti] OR remission[Ti]) AND (("1990/01/01"[PDAT] : "3000/12/31"[PDAT]) AND "humans"[MeSH Terms]).

Data used to make estimates of ILD are from three sources. The first is literature data from previous systematic reviews – usually from smaller-scale studies of prevalence or incidence. The second data type is claims data for the United States (MarketScan), Poland, and Taiwan (province of China). The sources and preparation of these data are described elsewhere. The third data type is adjusted hospital inpatient records. Because these records only report primary diagnosis, a priori adjustments are made based on location and health-care access and quality.

Data inputs for interstitial lung disease and pulmonary sarcoidosis

Parameter	Countries with data	New sources	Total sources
Prevalence	45	0	304
Incidence	16	0	27

Remission	0	0	0
Other	3	0	17

Data processing

There were no major changes to data processing for ILD in GBD 2021.

Age and sex split

In some cases, data are reported by only age or only sex, but not both. For example, a study may have included the proportion of males and females with ILD and then separately reported the proportion of both sexes in smaller age bins (eg, age 40-45, 45-50, etc.) that have ILD. In these cases, we perform an age-sex split by utilising proportions within the study to disaggregate the data.

When no information by sex in a study is present, we instead perform a sex-split on the data by applying separate sex proportions. The sex split analysis was carried out using a tool called MR-BRT [1] (meta-regression—Bayesian, regularised, trimmed). When data are aggregated into age categories larger than 25 years, we split the data into smaller age bins based on the global age pattern from an initial DisMod-MR 2.1 model[1] (disease model—Bayesian meta-regression; details on this method can be found in appendix 1, section 4.5 of the citation)

Bias adjustments

In GBD 2019, we improved the bias adjustment methods by utilising a MR-BRT model outside of DisMod to allow a more direct comparison between different case definitions and/or study designs.

We made a series of adjustments to data that don't completely match our case definition. Data that only report idiopathic pulmonary fibrosis (IPF) or sarcoidosis under-report estimates of ILD in a population. USA claims data from 2000 tend to differ from other years of USA claims data. We make adjustments to these data to reflect these possible variations. The adjustment is a logit-transformation method in MR-BRT. The general process is described below:

1. Identify datapoints with overlapping year, age, sex, and location between reference and alternative definitions.
2. Logit transform overlapping datapoints of alternative and reference case definitions
3. Convert overlapping datapoints into a difference in logit space using the following equation: $logit(altnerative) - logit(reference)$
4. Use the delta method to compute standard errors of overlapping datapoints in logit space, then calculate standard error of logit difference using the following equation: $\sqrt{(variance\ of\ alternative) + (variance\ of\ reference)}$
5. Using MR-BRT, conduct a random effects meta-regression to obtain the pooled logit difference of alternative to reference
6. Apply the pooled logit difference to all data oints of alternative case definitions using the following equation:
$$new_{estimate} = inverse.\,logit((logit(alternative)) - (pooled\ logit\ difference))$$
7. Calculate new standard errors using the delta method, accounting for gamma (between-study heterogeneity)

MR-BRT crosswalk adjustment factors

Data input	Status	Gamma	Beta coefficient, logit (95% UI)*	Adjustment factor**
All ILD (inclusive of IPF and sarcoidosis)	Ref	0.23	---	---
Only IPF	Alt	-	-1.46 (-2.09 - -0.79)	0.23
Only sarcoidosis	Alt	-	-1.07 (-1.71 - -0.40)	0.34
USA MarketScan 2000 (vs reference of other years of MarketScan)	Alt	0	-0.31 (-0.32 - -0.29)	0.73

**MR-BRT crosswalk adjustments can be interpreted as the factor the alternative case definition is adjusted by to reflect what it would have been had it been measured using the reference case definition. If the log/logit beta coefficient is negative, then the alternative is adjusted up to the reference. If the log/logit beta coefficient is positive, then the alternative is adjusted down to the reference.*
***The adjustment factor column is the exponentiated beta coefficient. For log beta coefficients, this is the relative rate between the two case definitions. For logit beta coefficients, this is the relative odds between the two case definitions.*

Modelling strategy

Estimates for ILD are produced using a standard DisMod-MR 2.1 approach. We use prior settings of zero remission and we constrain the super-region random effects to-0.5 to 0.5 to ensure model stability.

We employed predictive covariates to improve estimation in locations with scarce prevalence data. These were income per capita and the Healthcare Access and Quality (HAQ) Index. The priors on HAQ Index were model outputs from the MR-BRT modelling on EMR as described in the next section.

Variable name	Measure	Beta	Exponentiated
LDI (I$ per capita)	excess mortality rate	-0.2 (-0.2 to -0.2)	0.82 (0.82–0.82)
Healthcare Access and Quality Index	excess mortality rate	-0.014 (-0.014 — -0.014)	0.99 (0.99 — 0.99)

Predicted excess mortality rate with MR-BRT

Similar to other causes, we include estimates of cause-specific mortality rate (CSMR) and excess mortality rate (EMR) as model inputs. In previous rounds, priors on excess mortality rate (EMR) were estimated in DisMod by matching prevalence datapoints with their corresponding CSMR values within the same age, sex, year, location (by dividing CSMR by prevalence).

However, for many causes, DisMod estimated a rather unrealistic pattern of EMR compared to an expected pattern of decreasing EMR with greater access to quality health care. Such unexpected patterns often signal inconsistencies between CSMR estimates and the measures of prevalence and/or incidence.

To provide greater guidance to DisMod on the expected pattern of EMR, EMR data generated in the previous round were modelled using the MR-BRT approach by age and sex with a prior on Healthcare Access and Quality (HAQ) Index having a negative coefficient. Results from MR-BRT were then predicted for each location year, sex, and for ages 0, 10, 20 ….100. We included HAQ Index as a country-level covariate to inform EMR with a mean and standard deviation produced from MR-BRT.

Correction to GBD 2019

There was an error in our estimates in GBD 2019, where the incidence of ILD was greater than the prevalence across many locations. This error was caused by a miscoded remission setting, where we had individuals recovering from ILD. This was corrected in GBD 2021, which contributed to a large reduction in overall incidence and a reduction to 0% remission.

Severity splits

Data to inform estimates of the severity gradient due to ILD are derived from previous analyses of the Medical Expenditure Panel Survey (MEPS). The table below illustrates the lay descriptions and disability weights associated with different levels of severity of interstitial lung disease.

Severity level	Lay description	DW (95% CI)
Mild	Has cough and shortness of breath after heavy physical activity, but is able to walk long distances and climb stairs.	0.019 (0.011–0.033)
Moderate	Has cough, wheezing, and shortness of breath, even after light physical activity. The person feels tired and can walk only short distances or climb only a few stairs.	0.225 (0.153–0.312)
Severe	Has cough, wheezing, and shortness of breath all the time. The person has great difficulty walking even short distances or climbing any stairs, feels tired when at rest, and is anxious.	0.408 (0.273–0.556)

[1] Vos T, Lim SS, Abbafati C, *et al.* Global burden of 369 diseases and injuries in 204 countries and territories, 1990–2019: a systematic analysis for the Global Burden of Disease Study 2019. *The Lancet* 2020; 396: 1204–22. doi: https://doi.org/10.1016/S0140-6736(20)30925-9

Invasive non-typhoidal Salmonella (iNTS)

Flowchart

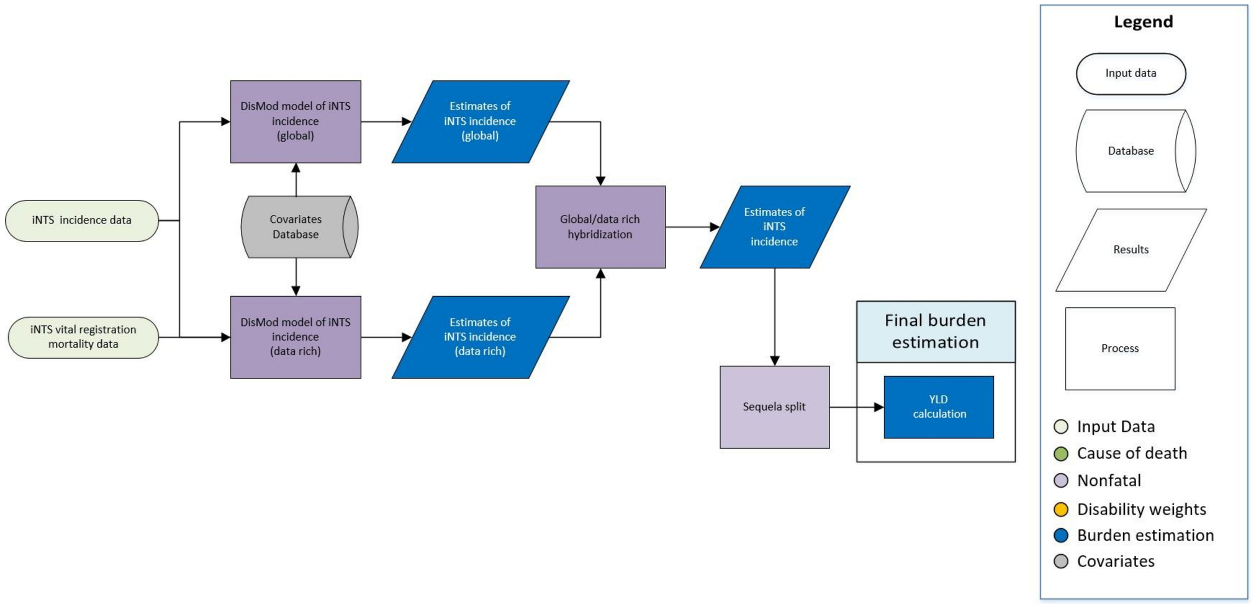

Case definition

Non-typhoidal salmonella infections are typically associated with diarrhoea. When these bacteria invade a typically sterile site like blood, they produce invasive non-typhoidal salmonella (iNTS) disease. Whereas non-typhoidal salmonella infections typically produce diarrhoeal illness, iNTS is typically febrile and can manifest in diverse symptoms that vary with severity and the exact site of the infection. Blood culture is the standard diagnostic for iNTS and has good sensitivity and specificity. We thus define a case of iNTS as any blood-culture-confirmed non-typhoidal salmonella infection of a normally sterile site.

Input data

Model inputs

We conducted a systematic review for studies of iNTS incidence for GBD 2017, including sources that provided iNTS incidence rates derived from either active surveillance or, more commonly, hospital- or clinic-based surveillance with adjustments for health-care utilisation. Studies of special populations (eg, people living with HIV/AIDS) were excluded. In total, we found 34 sources meeting our inclusion criteria. Updates to systematic reviews are performed on an ongoing schedule across all GBD causes; an update for iNTS was not performed for GBD 2021 and will be performed in the next one to two iterations.

Table 1: Data inputs for invasive non-typhoidal salmonella

Measure	Total sources	Countries with data
All measures	1921	88
Incidence	1921	88

Severity splits

Given the typical severity of iNTS and the breadth of potential symptoms and manifestations, we assign all cases to the severe acute infectious disease episode health state, with a disability weight of 0.133 (0.088–0.19)

Table 2: Severity distribution for invasive non-typhoidal salmonella

Sequela	Description	Disability weight
Severe acute infectious disease episode	Has a high fever and pain, and feels very weak, which causes great difficulty with daily activities.	0.133 (0.088–0.19)

Modelling strategy

We modelled incidence using two DisMod models: 1) a model that includes only incidence data, used to produce estimates for moderate- and high-burden regions; and 2) a model that includes additional incidence estimates derived from vital registration data from data-rich counties, used to produce estimates for low-burden regions. Both DisMod models used HIV mortality rate, malaria incidence adjusted for antimalarial coverage and drug effectiveness, and the summary exposure values (SEV) for sanitation as country-level covariates. We used no study-level covariates in the models.

We estimated prevalence as the product of incidence times duration. We estimated the duration of iNTS based on duration parameters reported in the scientific literature, with reported duration parameters including mean, median, range, standard deviation, and interquartile range. Because studies differed in how they reported duration, we were unable to use a simple meta-analysis approach. Toleverage information on duration from all studies, we used approximate Bayesian computation (ABC). ABC employs a simple grid search in which we assumed that iNTS duration, in days, follows a negative binomial distribution with a one-day offset such that the resulting distribution had a minimum possible value of one-day. We used a random negative binomial generator that took three inputs: the length of the randomly generated vector, *N*, the number of trials, *n*, and the probability of success in each trial, *p*. We trialed combinations of values of *n* and *p* using a simple grid search. For each combination, and for each duration datapoint, we generated 10,000 vectors from an offset random negative binomial distribution, where the length of each vector equaled the sample size of the study. Thus, each vector represented a random realisation of a possible distribution of durations for a given study. We estimated deviations between these realisations and the corresponding input data using an empirical cumulative distribution, and selected the best combination of values for *n* and *p* based on the root mean squared error. We estimated a mean duration of 7 days (95% CI: 1–24).

Changes from GBD 2019 to GBD 2021

We have made no substantive changes to our modelling strategy for iNTS between GBD 2019 and GBD 2021.

Ischaemic heart disease

Flowchart

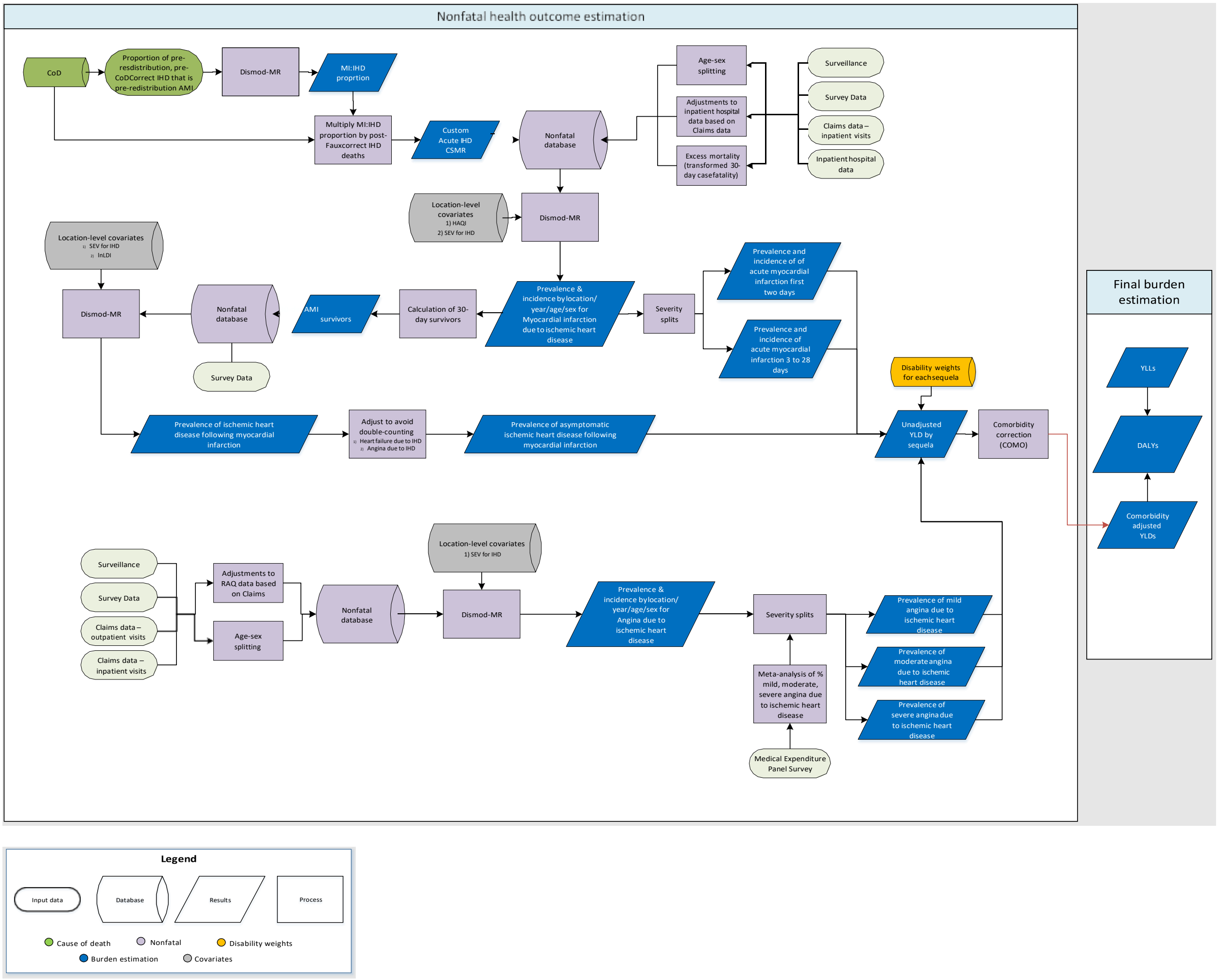

Input data and methodological summary for ischaemic heart disease

Case definition

Ischaemic heart disease (IHD) is a disease that limits the supply of blood to the heart. IHD is typically due to the narrowing of the coronary arteries, usually due to atherosclerosis, which limits blood flow. GBD estimates IHD as the aggregate of discrete sequelae, consisting of myocardial infarction (heart attacks), angina (stable ischaemic heart disease manifesting as chest pain), or ischaemic cardiomyopathy (heart failure due to IHD). For GBD 2021, we modelled prevalence and incidence of acute myocardial infarction (MI) as well as the prevalence of chronic ischaemic heart disease (IHD).

Case definitions:

1) Acute myocardial infarction (MI): Definite and possible MI according to the fourth universal definition of myocardial infarction:[4]
    a. When there is clinical evidence of myocardial necrosis in a clinical setting consistent with myocardial ischaemia or
    b. Detection of a rise and/or fall of cardiac biomarker values and with at least one of the following: i) symptoms of ischaemia, ii) new or presumed new ST-segment-T wave changes or new left bundle branch block, iii) development of pathological Q waves in the ECG, iv) imaging evidence of new loss of viable myocardium or new regional wall motion abnormality, or v) identification of an intracoronary thrombus by angiography or autopsy.
    c. Sudden (abrupt) unexplained cardiac death, involving cardiac arrest or no evidence of a non-coronary cause of death.

The prevalence of MI is considered to last from the onset of the event to 28 days after the event and is divided into an acute phase (0–2 days) and subacute phase (3–28 days). We also included unstable angina when reported separately as specified in the fourth universal definition.

2) Chronic IHD
    a. Stable angina: clinically diagnosed stable exertional angina pectoris or definite angina pectoris according to the Rose Angina Questionnaire (RAQ), physician diagnosis, or taking nitrate medication for the relief of chest pain.
    b. Asymptomatic ischaemic heart disease following myocardial infarction; survival to 28 days following incident MI. The GBD study does not use estimates based on ECG evidence for prior MI, due to its limited specificity and sensitivity (1).

Reference and alternate definitions of acute myocardial infarction and stable angina are shown in Tables 1a and 1b. ICD codes mapped to acute myocardial infarction and stable angina are listed in Tables 2a and 2b.

Table 1a: Reference and alternate definitions for acute myocardial infarction

Quantity of interest	Reference or Alternate	Definition
Incidence of acute myocardial infarction (MI)	Reference	Definite and possible MI according to the third universal definition of myocardial infarction; includes recurrent cases and cases who died before reaching medical care
Incidence of acute myocardial infarction (MI)	Alternate	Cases diagnosed prior to regular use of troponin as part of the standard case definition
Incidence of acute myocardial infarction (MI)	Alternate	First-ever cases only
Incidence of acute myocardial infarction (MI)	Alternate	Includes only persons who survived to the hospital

Table 1b: Reference and alternate definitions for stable angina

Quantity of interest	Reference or Alternate	Definition
Prevalence of angina due to ischaemic heart disease	Reference	Stable exertional or definite angina pectoris as identified by clinician diagnosis and subsequent ICD coding in claims or outpatient data
Prevalence of angina due to ischaemic heart disease	Alternate	Stable exertional angina pectoris as defined using the Rose Angina Questionnaire

Table 2a: ICD codes mapped to acute myocardial infarction

ICD code	Description
410	Acute myocardial infarction
411.0	Post-myocardial infarction syndrome
412	Old myocardial infarction
I21	Acute myocardial infarction
I22	Subsequent ST elevation (STEMI) and non-ST elevation (NSTEMI) myocardial infarction
I23	Certain current complications following ST elevation (STEMI) and non-ST elevation (NSTEMI) myocardial infarction (within the 28-day period)
I24.1	Dressler's syndrome

Table 2b: ICD codes mapped to stable angina

ICD code	Description
413	Angina pectoris
I20	Angina pectoris

Input data

Myocardial infarction

To update the current GBD database for GBD 2021, a systematic review was done for myocardial infarction. The following databases were searched: EMBASE, PubMed, and Virtual Health Library (VHL). The dates of the search were 01/01/2020 – 03/31/2020. The search strings used were ((“myocardial infarction”[tiab] AND (incidence OR “case fatality” OR “excess mortality”)) OR (“acute coronary syndrome”[tiab] AND (incidence OR “case fatality” OR “excess mortality”)) OR (angina[tiab] AND (incidence OR prevalence OR “case fatality” OR “excess mortality”))) AND ("2020/01/01"[PDAT] : "20/03/31"[PDAT]) NOT rat[tiab] NOT mice[tiab] NOT monkey[tiab] NOT pig[tiab] NOT animals[tiab]. The findings were reported according to the PRISMA (Preferred Reporting Items for Systematic reviews and Meta-Analyses) statement. Figure 1 shows the PRISMA diagram for the systematic review. In the diagram, screening refers to reviewing of the title and abstract of an article for relevant information, not screening of the entire article. Reasons for exclusion include non-representativeness, use of different case definitions, studies reporting age-standardised data and data reported in a non-compatible format. In total, 2849 studies were returned, 579 from EMBASE, 2231 from PubMed, and 39 from VHL. 18 articles were extracted.

The total source counts for non-fatal ischaemic heart disease (including acute myocardial infarction and chronic ischaemic heart disease) are shown in Table 3 below by measure. The “Other” category includes primarily case fatality proportion data, but also includes one source that reports remission and another that reports standardised mortality ratio.

Table 3: Data inputs for ischaemic heart disease morbidity modelling by parameter

	Countries with data	New sources	Total sources
Incidence	58	111	406
Prevalence	147	3	92
Remission	0	0	0
Other	30	75	183

Figure 1: PRISMA 2020 flow diagram

From: Page MJ, McKenzie JE, Bossuyt PM, Boutron I, Hoffmann TC, Mulrow CD, et al. The PRISMA 2020 statement: an updated guideline for reporting systematic reviews. *BMJ* 2021;372:n71. doi: 10.1136/bmj.n71

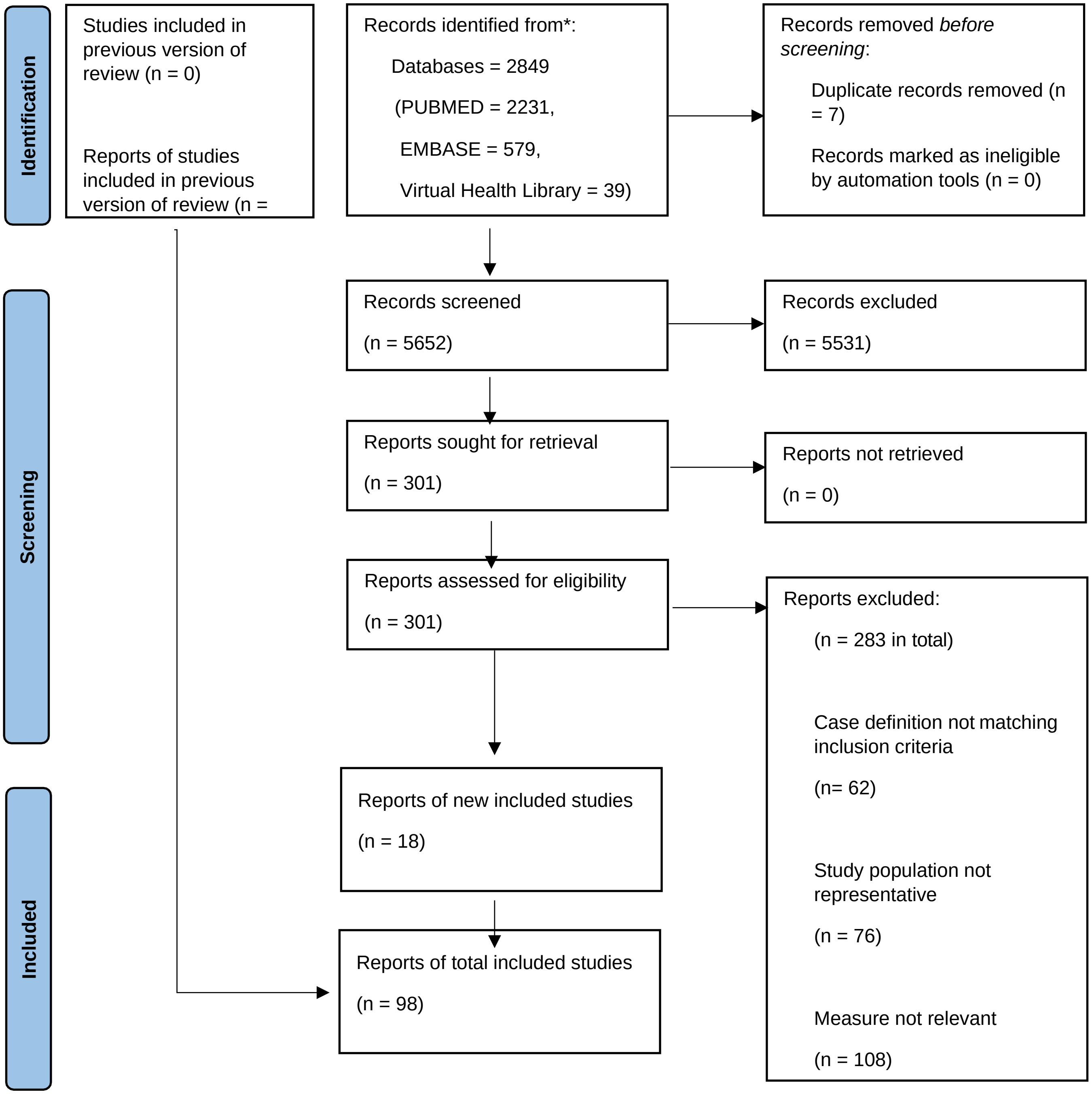

We also performed searches for GBD 2013, 2015, and 2019. Search terms, parameters, and results returned will be provided on request.

Apart from inpatient hospital and inpatient claims data, we did not include any data from sources other than the literature for myocardial infarction.

The 30-day case fatality proportion of acute myocardial infarction was extracted from the literature and myocardial infarction registries. We expressed 30-day case fatality proportion as a rate (excess mortality rate) using the rate equation:

$$excess\ mortality\ rate = \frac{-\log(1 - case\ fatality\ proportion)}{30/365}$$

Case fatality proportion was expressed as excess mortality rate under the assumption that death within 30 days of an acute myocardial infarction event would be due to the event.

We adjusted incidence measurements for myocardial infarction literature data with alternative definitions to agree with our case reference definition using MR-BRT (meta-regression—Bayesian, regularised, trimmed) modelling tool. MR-BRT and the process of data adjustment are discussed elsewhere in the appendix. For myocardial infarction, we adjusted using multiple different covariates: a covariate to capture only first-ever MI, using studies where all events were included as the reference; a covariate to adjust estimates from studies that only included non-fatal cases that survived the event in time to reach health care, using sources that included fatal and non-fatal cases as reference; and a covariate to adjust for studies that did not use troponin measurements in their case diagnosis, using sources that did include troponin measurements in their diagnostic method. The coefficients for myocardial infarction reported in Table 4a can be used to calculate adjustment factors for alternative definitions. The formula for computing adjustment factors is given in Equation 1 below. We also included a standardised age variable (age-scaled) and a sex variable in the regression to adjust for the possibly of bias. Splines were not used in this model.

Equation 1: Calculation of adjustment factors without splines:

$$Estimated\ Reference\ Def = invlogit(logit(Alternative\ Def) - [\sum_{a=0}^{b} Beta_{Alternative\ Def_a} * I(Alternate\ Def_a)] - Beta_{sex} * I(Sex) - Beta_{age} * age_{scaled})$$

$I(.)$ = Indicator function, b = Number of alternate definitions used

Table 4: MR-BRT crosswalk adjustment factors for myocardial infarction

Data input	Measure	Reference or alternative case definition	Gamma	Beta coefficient, logit (95% CI)*	Adjustment factor**
Any event, fatal and non-fatal events, used troponin	Incidence	Ref	---	---	
First-ever MI, intercept	Incidence	Alt	0.52	–0.87 (–2.03 to –0.25)	2.36 (1.20 to 3.52)
First-ever MI, age-scaled	Incidence	Alt		0.09 (0.07 to 0.12)	
First-ever MI, male	Incidence	Alt		0.71	

				(0.69 to 0.72)	
Non-fatal MI, intercept	Incidence	Alt		–0.76 (–1.91 to –0.37)	2.12 (0.96 to 3.27)
Non-fatal MI, age-scaled	Incidence	Alt		0.14 (0.12 to 0.17)	
Non-fatal MI, male	Incidence	Alt		0.19 (0.14 to 0.24)	
Troponin not used as part of definition, intercept	Incidence	Alt		–0.83 (–2.16 to –0.47)	2.27 (0.94 to 3.60)
Troponin not used as part of definition; age-scaled	Incidence	Alt		–0.13 (–0.12 to –0.14)	
Troponin not used as part of definition, sex (male)	Incidence	Alt		0.22 (0.22 to 0.23)	

**MR-BRT crosswalk adjustments can be interpreted as the factor the alternative case definition is adjusted by to reflect what it would have been had it been measured using the reference case definition. If the log/logit beta coefficient is negative, then the alternative is adjusted up to the reference. If the log/logit beta coefficient is positive, then the alternative is adjusted down to the reference.*

***The adjustment factor column is the exponentiated beta coefficient. For log beta coefficients, this is the relative rate between the two case definitions. For logit beta coefficients, this is the relative odds between the two case definitions.*

Once the crosswalk was performed, we split incidence and excess mortality datapoints where the age range was greater than 25 years. Age splitting was based on the global sex-specific age pattern from a DisMod model that only used as input data incidence or excess mortality information from scientific literature with less than a 25-year age range. Datapoints included in the model used to generate the age pattern could use either the reference or alternate case definition.

<u>Asymptomatic ischaemic heart disease following myocardial infarction</u>

No systematic review was performed for asymptomatic ischaemic heart disease following myocardial infarction in GBD 2021. The primary input for this model is 28-day survivors calculated from the excess mortality estimates for the myocardial infarction model. To calculate survivors of incident acute myocardial infarction, we first retrieve the incidence and excess mortality rate estimates from the Dismod-MR 2.1 model of acute myocardial infarction. We then express excess mortality rate (EMR) as cross-sectional case fatality proportion (CFP) using the inverse of the rate-equation $CFP = \frac{EMR}{12+EMR}$. The estimated incidence is then multiplied by $(1 - CFP)$ to calculate the number of incident cases that survived the initial 28 days of the event.

We also included data for excess mortality and standardised mortality ratio to inform the estimates of survival after myocardial infarction. This data came from literature reviews as well as a custom analysis in collaboration with the PURE study.[1]

Stable angina

A specific systematic review for angina pectoris was not performed for GBD 2021. Updates to systematic reviews are performed on an ongoing schedule across all GBD causes; an update for angina will be performed in the next one to two iterations.

Prior to GBD 2021, a systematic review for stable angina was last performed for GBD 2013. The search terms used were: (Angina Pectoris/epidemiology[Mesh] OR Angina Pectoris/mortality[Mesh]) AND (prevalence[Title/Abstract] OR incidence[Title/Abstract]) AND ("2010"[Date - Publication] : "3000"[Date - Publication])

We included survey data (including National Health and Nutrition Examination surveys and World Health Study questionnaires) which included the RAQ items. Prevalence of angina was calculated using the standard algorithm to determine whether the RAQ was positive or negative by either grade 1 or 2 criteria of definite angina.[2]

We included USA administrative health system data using the ICD codes for inpatient and outpatient billing for stable angina (table 2b) from the private payor data from Truven MarketScan analytics; preparation of these data is described in the “Claims data” section of the non-fatal appendix. Stable angina is expected to be rare in inpatient but common in outpatient data, as it is a condition usually managed on an outpatient basis, except for specific surgical interventions. This discrepancy leads to implausible correction factors based on inpatient/outpatient information from claims data (~150X); thus, adjusted data cannot be used. Including uncorrected data in the model is likely to lead to incorrect estimates, as hospitalisation and procedure rates are likely to vary between geographies based on access to and patterns of care.

We adjusted prevalence data obtained from survey data using the RAQ using claims data as a reference since the RAQ has been shown to be neither sensitive nor specific. Specifics on the crosswalking process are discussed elsewhere in the GBD methods appendix. Table 5 shows the coefficients adjustments made to the alternative definition. Figure 2 shows the spline on age implemented in the crosswalk model by sex.

We split angina prevalence datapoints where the age range was greater than 25 years. Age-splitting was based on the global sex-specific age pattern from a DisMod model that only used prevalence input data from scientific literature with less than a 25-year age range and administrative claims data. We excluded prevalence data with broad age ranges where it was impossible to age-split due to small sample size, as these data caused the known age pattern for increased risk of angina to be masked in the estimates generated from DisMod.

Equation 2: Calculation of adjustment factors with splines:

$$Estimated\ Reference\ Def = invlogit(logit(Alternative\ Def) - [\sum_{s=0}^{b} Beta_{Alternative\ Def, spline\ basis_s} * Spline\ basis_s(age\_scaled)] - Beta * I(Sex))$$

$I(.)$ = Indicator function, b = Number of spline bases used

Table 5: MR-BRT crosswalk adjustment factors for angina prevalence

Data input	Reference or alternate case definition	Gamma	Beta coefficient, log/logit (95% UI)*	Adjustment factor**
USA claims data	Reference	0.05	---	---
Rose Angina Questionnaire	Alt		1.97 (1.61 to 2.32)	0.14 (–0.25 to 0.50)
Age (scaled) spline 0	Alt		5.407 (0.02 to 5.45)	
Age (scaled) spline 1	Alt		1.815 (0.02 to 1.84)	
Age (scaled) spline 2	Alt		0.809 (0.78 to 0.83)	
Age (scaled) spline 3	Alt		–0.544 (–0.57 to –0.52)	
Age (scaled) spline 4	Alt		–0.274 (–0.31 to –0.24)	
Age (scaled) spline 5	Alt		–0.285 (–0.33 to –0.24)	
Sex (male)	Alt		–0.80 (–0.81 to –0.80)	

**MR-BRT crosswalk adjustments can be interpreted as the factor the alternative case definition is adjusted by to reflect what it would have been had it been measured using the reference case definition. If the log/logit beta coefficient is negative, then the alternative is adjusted up to the reference. If the log/logit beta coefficient is positive, then the alternative is adjusted down to the reference.*

***The adjustment factor column is the exponentiated beta coefficient. For log beta coefficients, this is the relative rate between the two case definitions. For logit beta coefficients, this is the relative odds between the two case definitions.*

Figure 2: Age-scaled spline for adjustment of RAQ angina prevalence data

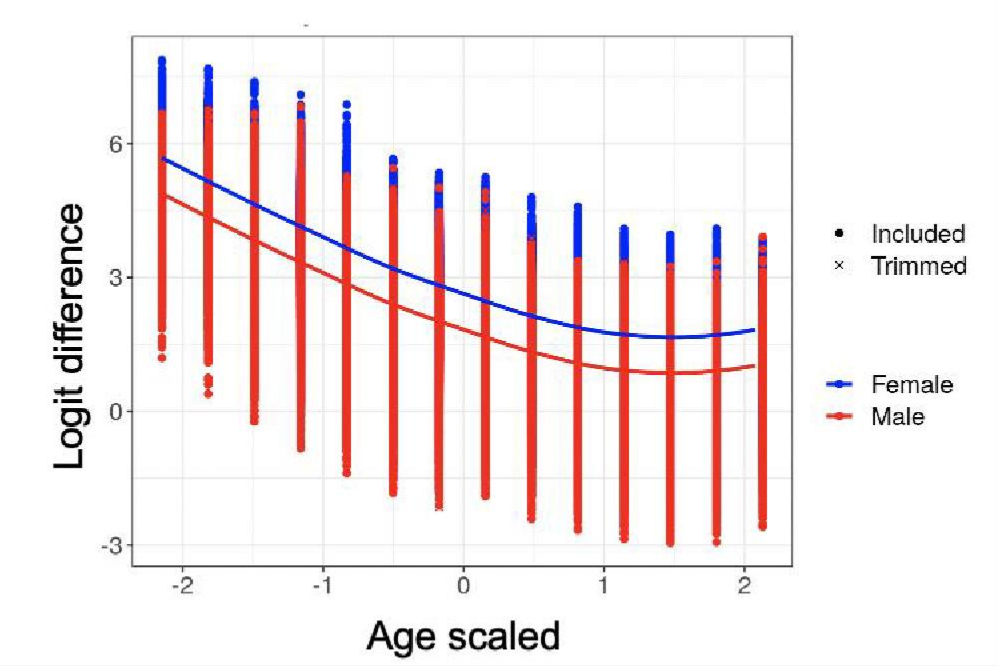

Modelling strategy

Myocardial infarction

- We first calculated custom cause-specific mortality estimates using cause of death data post garbage code redistribution, generating age-sex-country-year-specific proportions of IHD deaths that were due to MI (acute IHD) versus those due to other causes of IHD (chronic IHD). Estimates of this proportion for all locations were then generated using a DisMod proportion-only model. Due to a high degree of variability in pre-redistribution coding practices by location, we used the global age-, sex-, and year-specific proportions of acute deaths in subsequent calculations. The global proportions were multiplied by post-CoDCorrect IHD deaths by location to generate CSMR estimates for MI. These data, along with incidence and excess mortality data, informed a DisMod model to estimate the prevalence and incidence of myocardial infarction due to ischaemic heart disease.
- These estimates were split proportionally by time into estimates for days 1–2 and days 3–28 post-event. Disability weights were assigned to each of these two groupings.
- We set a value prior of one month for remission (11/13) from the MI model. We also set a value prior for the maximum excess mortality rate of 10 for all ages. We included the Healthcare Access and Quality (HAQ) Index as a fixed-effect country-level covariate on excess mortality, forcing an inverse relationship. We also included the log-transformed age-standardised SEV scalar for IHD as a fixed-effect country-level covariate for incidence.

Table 6. Covariates. Summary of covariates used in the myocardial infarction DisMod-MR meta-regression model

Covariate	Parameter	Beta	Exponentiated beta

Healthcare Access and Quality (HAQ) Index	Excess mortality rate	–0.03 (–0.03 to –0.03)	0.97 (0.97 to 0.97)
Log-transformed age-standardised SEV scalar: IHD	Incidence	0.75 (0.75 to 0.76)	2.12 (2.12 to 2.14)

Severity split inputs

Acute myocardial infarction was split into two severity levels by length of time since the event – days 1 and 2 versus days 3 through 28. Disability weights were established for these two severities using the standard approach for GBD 2021.

Table 7. Severity distribution, details on the severity levels for myocardial infarction in GBD 2021 and the associated disability weight (DW) with that severity

Severity level	Lay description	DW (95% CI)
Acute myocardial infarction, days 1–2	Has severe chest pain that becomes worse with any physical activity. The person feels nauseated, short of breath, and very anxious.	0.432 (0.288–0.579)
Acute myocardial infarction, days 3–28	Gets short of breath after heavy physical activity, and tires easily, but has no problems when at rest. The person has to take medication every day and has some anxiety.	0.074 (0.049–0.105)

Asymptomatic chronic ischaemic heart disease

- Excess mortality estimates from the myocardial infarction model were used to generate data of the incidence of surviving 28 days post-event.
- We used these data, along with the estimates of CSMR due to chronic IHD (the other part of the proportion described in step 1) and excess mortality data in a DisMod model to estimate the prevalence of persons with IHD following myocardial infarction. This estimate included subjects with angina and heart failure; a proportion of this prevalence was removed to avoid double-counting based on evidence from the literature.[3] The result of this step generates estimates of asymptomatic ischaemic heart disease following myocardial infarction.
- We set a value prior of 0 for remission for all ages.
- We also included the log-transformed, age-standardised SEV scalar for IHD as a fixed effect, country-level covariate on prevalence and LDI (I$ per capita) as a fixed-effect country-level covariate on excess mortality, forcing an inverse relationship for LDI.

Table 8. Covariates. Summary of covariates used in the asymptomatic chronic ischaemic heart disease DisMod-MR meta-regression model

Covariate	Parameter	Beta	Exponentiated beta
LDI (I$ per capita)	Excess mortality rate	–0.36 (–0.46 to –0.26)	0.70 (0.63 to 0.77)
Log-transformed age-standardised SEV scalar: IHD	Prevalence	1.00 (0.75 to 1.25)	2.72 (2.12 to 3.49)

Severity split inputs

Asymptomatic ischaemic heart disease following myocardial infarction was all assigned to the asymptomatic severity level. No disability weight is assigned to this level.

Table 9. Severity distribution, details on the severity levels for asymptomatic ischaemic heart disease following myocardial infarction in GBD 2021 and the associated disability weight (DW) with that severity

Severity level	Lay description	DW (95% CI)
Asymptomatic ischaemic heart disease		N/A

<u>Stable angina</u>

- We used prevalence data from the literature and USA claims databases, along with data on mortality risk to estimate the prevalence and incidence of angina for all locations. Data which used the Rose Angina Questionnaire to determine prevalence of angina were adjusted using MR-BRT as described above.
- The proportion of mild, moderate, and severe angina was determined by the standard approach for severity splitting for GBD 2021 that used the Medical Expenditure Panel Survey (MEPS) to map angina ICD codes (see table 2b) to quality of life metrics to quantify disability. More information on methodology on the proportion split using MEPS can be found in the appendix section 4.7: Severity distribution.
- We included a value prior of 0 for remission for all ages. We also included a value prior of 1 for excess mortality for all ages.
- We also included the log-transformed, age-standardised SEV scalar for IHD as a fixed effect, country-level covariate on prevalence and LDI (I$ per capita) as a fixed effect, country-level covariate on excess mortality, forcing an inverse relationship LDI.

Table 10. Covariates. Summary of covariates used in the Angina DisMod-MR meta-regression model

Covariate	Parameter	Beta	Exponentiated beta
Log-transformed age-standardised SEV scalar: IHD	Prevalence	–0.54 (–0.97 to –0.12)	0.58 (0.38 to 0.88)
LDI (I$ per capita)	Excess mortality rate	1.25 (1.24 to 1.25)	3.47 (3.45 to 3.49)

Severity split inputs

Angina was split into asymptomatic, mild, moderate, and severe groups using information from MEPS. Disability weights were established for these severities using the standard approach for GBD 2021.

Table 11. Severity distribution, details on the severity levels for angina pectoris in GBD 2019 and the associated disability weight (DW) with that severity

Severity level	Lay description	DW (95% CI)
Asymptomatic angina		N/A
Mild angina	Has chest pain that occurs with strenuous physical activity, such as running or lifting heavy objects. After a brief rest, the pain goes away.	0.033 (0.02–0.052)
Moderate angina	Has chest pain that occurs with moderate physical activity, such as walking uphill or more than half a kilometer (around a quarter-mile) on level ground. After a brief rest, the pain goes away.	0.08 (0.052–0.113)
Severe angina	Has chest pain that occurs with minimal physical activity, such as walking only a short distance. After a brief rest, the pain goes away. The person avoids most physical activities because of the pain.	0.167 (0.11–0.24)

There have been no substantive changes in the modelling strategy for myocardial infarction, asymptomatic ischaemic heart disease following myocardial infarction, and angina from GBD 2019.

References

[1] Hamilton Health Sciences, McMaster University (Canada), Population Health Research Institute (PHRI).

[2] Lawlor DA, Adamson J, Ebrahim S. Performance of the WHO Rose angina questionnaire in post-menopausal women: Are all of the questions necessary? *Journal of Epidemiology & Community Health* 2003;57:538-541.

[3] Doll JA, Tang F, Cresci S, Ho PM, Maddox TM, Spertus JA, Wang TY. Change in Angina Symptom Status After Acute Myocardial Infarction and Its Association With Readmission Risk: An Analysis of the Translational Research Investigating Underlying Disparities in Acute Myocardial Infarction Patients' Health Status (TRIUMPH) Registry. *J Am Heart Assoc*. 2016 Jun 13;5(6):e003205. doi: 10.1161/JAHA.116.003205. PMID: 27412898; PMCID: PMC4937266.

[4] Thygesen K, Alpert JS, Jaffe AS, Chaitman BR, Bax JJ, Morrow DA, White HD; Executive Group on behalf of the Joint European Society of Cardiology (ESC)/American College of Cardiology (ACC)/American Heart Association (AHA)/World Heart Federation (WHF) Task Force for the Universal Definition of Myocardial Infarction. Fourth Universal Definition of Myocardial Infarction (2018). *J Am Coll Cardiol*. 2018 Oct 30;72(18):2231-2264. doi: 10.1016/j.jacc.2018.08.1038. Epub 2018 Aug 25. PMID: 30153967.

Leprosy

Flowchart

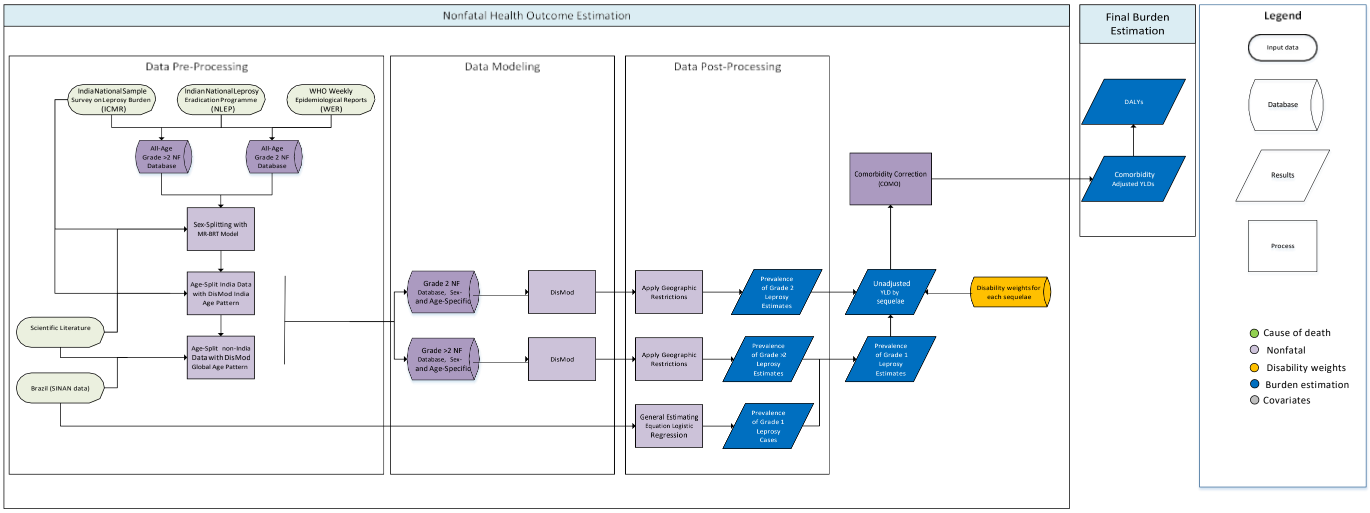

Input data and methodological summary

Case definition

Leprosy, also known as Hansen's disease, is a chronic bacterial infection caused by *Mycobacterium leprae*, primarily affecting the nervous system, skin, respiratory tract, and eyes. Transmission facilitates through prolonged contact with fluid from the nose and mouth of an infected individual. Leprosy can be diagnosed based on clinical manifestations, such as hypopigmented or reddish skin lesions with loss of sensation or thickening of the peripheral nerves accompanied by loss of sensation, and/or a positive skin smear for acid-fast bacilli. Left untreated, leprosy can progress to paralysis, blindness, painful neuropathy, and physical deformity. The ICD-10 code for leprosy is A30.9.

We used the following case definitions for GBD 2021:

Quantity of interest	Reference or alternative	Definition
Leprosy	Reference	An incident case of leprosy is defined as one identified through case notification or surveillance systems or via clinical diagnosis. Clinical diagnosis of leprosy can be based on clinical manifestations, such as hypopigmented or reddish skin lesion with loss of sensation, with or without involvement of peripheral nerves, and/or confirmation involving a skin-smear or biopsy.

Input data

Table 1: Data inputs for leprosy morbidity modelling by parameter

Measure	Countries with data	New sources	Total sources
All measures	144	110	1595
Prevalence	121	0	692

Incidence	144	110	1547

General methodology

The non-fatal estimation process for leprosy began with nationally representative case notification data published by the World Health Organization (WHO) or Ministries of Health. The analysis was implemented in three steps: (1) data pre-processing, (2) data modelling, and (3) post-processing, which included applying geographical restrictions and quantification of sequela.

Model inputs

Five data sources inform estimates of leprosy incidence and prevalence by grade-classification:

(i) WHO Weekly Epidemiological Record (WER) reports disaggregated by Grade 2 and less than Grade 2 disability from 2000 to 2018. Data from 1990–2000 were not disaggregated by grade and we hope to split it to use in future cycles.

(ii) Indian National Leprosy Eradication Programme (NLEP) subnational incidence data were used from 2010–2017.

(iii) The 2010–2011 India National Sample Survey on Leprosy Burden (ICMR) prevalence data were used to estimate prevalence in India subnationals, and to inform sex- and age-split models.

(iv) Data from Brazil's Information System for Notifiable Diseases (SINAN) informed the age-split models and the severity split model to disaggregate less than Grade 2 estimates into Grade 1 and Grade 0 estimates. These data were not used in the main prevalence models due to concerns that hospital-based reporting might over-represent prevalence at the subnational and national level.

(v) Relevant scientific literature (sci-lit) was used to inform the sex- and age-split models.

First, data reported in both sexes were split into male and female incidence inputs. To sex-split our both-sex data points, we used sex-specific inputs in a Bayesian meta-regression (MR-BRT) model to derive a ratio of female leprosy incidence to both leprosy incidence (sci-lit data). The resultant log ratio was applied to both-sex datapoints to calculate out females, and males were calculated via subtraction. The result was a higher prevalence of leprosy among males as opposed to females and is consistent with published gender disparity in leprosy cases.[1-3]

Table 2: MR-BRT crosswalk adjustment factors for leprosy

Data input	Reference or alternative case definition	Gamma	Beta coefficient, log ratio (95% CI)*	Adjustment factor**
Female data	Ref	0.126	---	---
Both data	Alt		0.257 (-0.53 – 1.05)	1.28

****MR-BRT crosswalk adjustments can be interpreted as the factor the alternative case definition is adjusted by to reflect what it would have been had it been measured using the reference case definition. If the log/logit beta coefficient is negative, then the alternative is adjusted up to the reference. If the log/logit beta coefficient is positive, then the alternative is adjusted down to the reference.***

***The adjustment factor column is the exponentiated beta coefficient. For log beta coefficients, this is the relative rate between the two case definitions. For logit beta coefficients, this is the relative odds between the two case definitions.*

We then split all-age case data into age-specific observations using two age patterns derived by a DisMod Bayesian meta-regression model (DisMod-MR), one specific for India (derived using ICMR and Indian scientific literature) and another global age pattern for non-India locations (derived using SINAN and non-Indian scientific literature). Both age patterns were developed using single-parameter incidence models in DisMod-MR. Uncertainty is propagated throughout the sex- and age-splitting processes, such that final sex- and age-specific incidence estimates reflect uncertainty of the original data.

The age patterns can be found below:

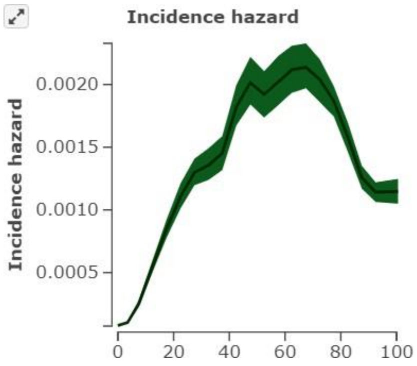

Figure 1a. Global age-pattern for leprosy used to split non-India all-age data into age-specific datapoints for further modelling

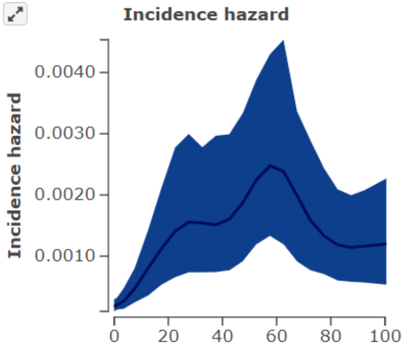

Figure 1b. India age-pattern for leprosy used to split India all-age data into age-specific datapoints for further modelling

Modelling strategy

We used a compartmental model in DisMod-MR to derive prevalence of leprosy from incident case reports. The reported case data were grade-specific, allowing us to implement two models, Grade <2 and Grade 2. In the Grade <2 Leprosy model, we assumed no incident cases among children less than 2 years old and an average duration of 2 years, to account for the broad spectrum of disability associated with Grade 1 and the availability of treatment. In the Grade 2 model, we assumed no incident cases occurring

among children less than 2 years old and assume no remission, as Grade 2 leprosy causes permanent disfigurement and/or disability.

Estimates of Grade <2 leprosy were disaggregated into Grade 1 and Grade 0 estimates using age- and sex-specific data reported by Brazil. Using these data, proportions of cases of Grade 1 and Grade 0 were calculated via logistic regression using a generalised estimating equation to account for repeated measures among the subjects in that cohort.

Table 3a. Covariates. Summary of covariates used in the leprosy DisMod-MR less than Grade 2 model

Covariate	Type	Parameter	Exponentiated beta (95% UI)
Latitude 15 to 30 (proportion)	Country-level	Prevalence	0.68 (0.64–0.71)
Healthcare Access and Quality Index	Country-level	Prevalence	0.08 (0.076–0.085)

Table 3b. Covariates. Summary of covariates used in the leprosy DisMod-MR grade 2 model

Covariate	Type	Parameter	Exponentiated beta (95% UI)
Latitude 15 to 30 (proportion)	Country-level	Prevalence	2.80 (1.53–12.13)
Healthcare Access and Quality Index	Country-level	Prevalence	0.048 (0.011–0.067)

Results post-processing

Geographical restrictions were applied to generate zero estimates in countries for which transmission is not considered endemic. We do not account for imported cases of leprosy.

Table 3. Severity distribution, details on the severity levels for leprosy and the associated disability weight (DW) with that severity

Severity level	Lay description	DW (95% CI)
Disfigurement level 1 due to Leprosy	Has a slight, visible physical deformity that others notice, which causes some worry and discomfort.	0.011 (0.005–0.021)
Disfigurement level 2 due to Leprosy	Has a visible physical deformity that causes others to stare and comment. As a result, the person is worried and has trouble sleeping and concentrating.	0.067 (0.044–0.096)

Changes from GBD 2019 to GBD 2021

The leprosy model had some revisions in GBD 2021. Specifically, there were changes in data inputs and processing.

Data inputs increased to include WHO WER data through 2018. In data processing, an updated MR-BRT method was used to sex-split the both-sex data (methods described elsewhere in this appendix). Additionally, in GBD 2019, we assumed no incident cases for Grade 2 and Grade <2 in ages younger than 15. The current prior assumes no incident cases in children less than 2 years old in Grade <2 and Grade 2.

There were two covariates used in GBD 2019, Socio-demographic Index (SDI) and Healthcare Access and Quality (HAQ) Index. Due to concerns of collinearity between SDI and HAQ Index, SDI was removed and proportion of the population living in latitude 15 to 30 was added in GBD 2021.

We did not apply any adjustments for the COVID pandemic to leprosy due to a lack of available data quantifying the impacts of the pandemic on NTD epidemiology.

References

1. Kumar R, Singhasivanon P, Sherchand JB, *et al.* Gender difference in socio-epidemiological factors for leprosy in the most hyper-endemic district of Nepal. *Nepal Med Coll J* 2004; 6: 98–105.
2. Peters ES, Eshiet AL. Male-female (sex) differences in leprosy patients in south eastern Nigeria: females present late for diagnosis and treatment and have higher rates of deformity. *Lepr Rev* 2002; 73: 262–7.
3. Ramos JM, Martínez-Martín M, Reyes F, Lemma D, Belinchón I, Gutiérrez F. Gender differential on characteristics and outcome of leprosy patients admitted to a long-term care rural hospital in South-Eastern Ethiopia. *Int J Equity Health* 2012; 11: 56.

Low back pain

Flowchart

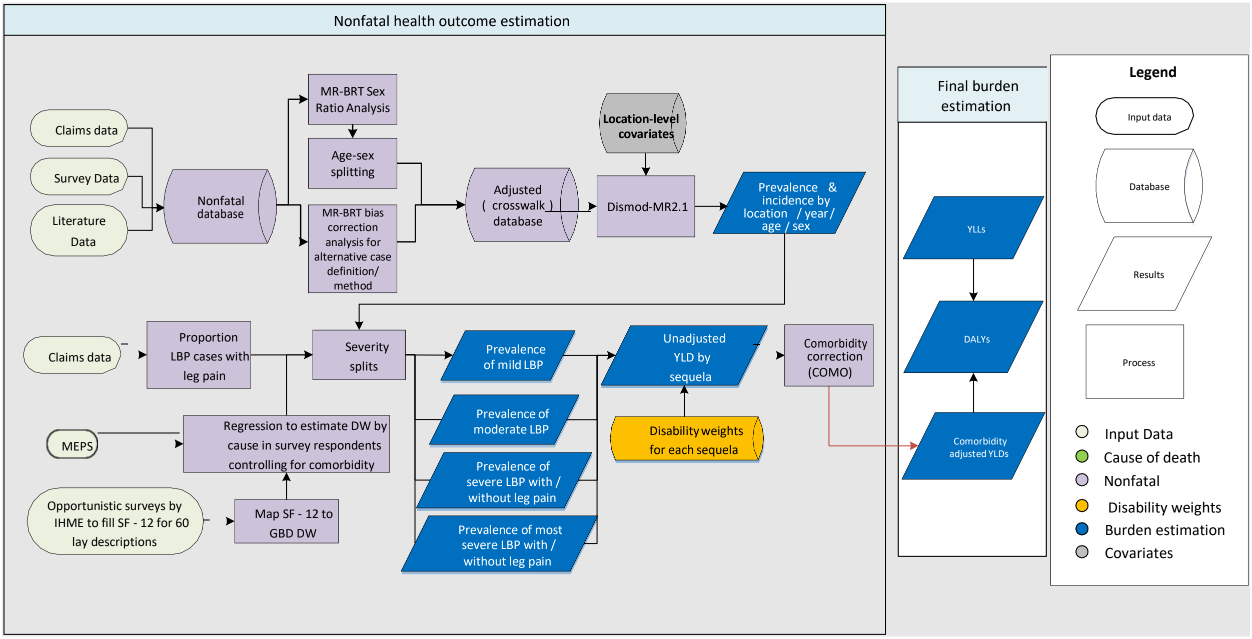

Input data and methodological summary for low back pain

Case definition

Low back pain (LBP) is defined as low back pain (with or without pain referred into one or both lower limbs) that lasts for at least one day. The "low back" is defined as the area on the posterior aspect of the body from the lower margin of the twelfth ribs to the lower gluteal folds.

The case definitions accepted for low back pain are shown below.

Reference or alternative	Definition
Reference	Taiwan claims data
Alternative	current low back pain that lasts for at least 3 months (chronic)
Alternative	low back pain that lasts for at least one day within the last 1 week to 1 month
Alternative	low back pain that lasts for at least one day within the last 2 months to 1 year
Alternative	current low back pain in a study population of schoolchildren that lasts for at least one day
Alternative	current low back pain that lasts for at least one day and is activity-limiting
Alternative	Includes a broader anatomical region (eg, low back and upper back, low back and neck, etc.)
Alternative	USA claims data 2000
Alternative	USA claims data 2010–2012, 2014

ICD-10 codes for LBP are M54.3, M54.4 and M54.5. The ICD-9 code is 724.

Input data

The last systematic review was conducted for GBD 2021 utilising the following search string:

(((Back Pain[MeSH] OR "back pain"[TiAb]) AND (prevalen*[TiAb] OR inciden*[TiAb]) AND (2017/09/01[PDAT] : 3000[PDAT]))

OR(((prevalen* OR inciden*) AND ("neck pain" OR "neck ache" OR "neckache" OR "cervical pain" OR Neck Pain[Mesh])) AND (2017/12/20[PDAT] : 3000[PDAT]))

)

NOT (animals[MeSH] NOT humans[MeSH])

This returned 1346 entries, of which 35 low back pain sources were extracted.

Exclusion criteria were:

1. Sub-populations clearly not representative of the national population
2. Not a population-based study
3. Low sample size (less than 150)
4. Review rather than original studies

Figure 2: PRISMA diagram of low back pain systematic review from 2021

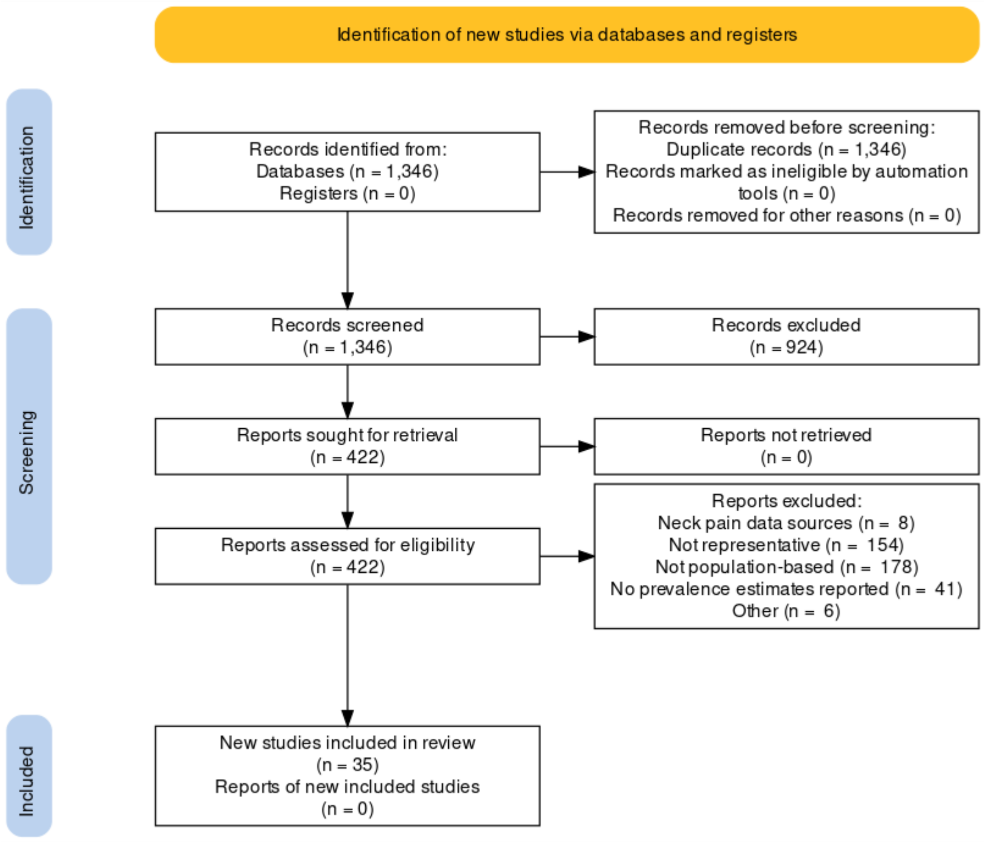

Additional information was derived from unit record data of surveys in the GHDx, GBD's repository of population health data including the World Health surveys and national health surveys. Opportunistically, additional studies encountered during data review were added for GBD 2019. In addition, data from USA claims data for 2000, 2010–2012, and 2014–2016 by state were included.

Table 1: Data Inputs for low back pain

Measure	Total sources	Countries with data	New sources
All measures	501	104	39
Prevalence	484	103	39
Incidence	4	3	0
Remission	3	2	0
Other	15	1	0

Age and sex splitting

Reported estimates of prevalence were split by age and sex where possible. First, if studies reported prevalence for broad age groups by sex (eg, prevalence in 15- to 65-year-old males and females separately), and also by specific age groups for both sexes combined (eg, prevalence in 15- to 30-year-olds, then in 31- to 65-year-olds, for males and females combined), age-specific estimates were split by sex using the reported sex ratio and bounds of uncertainty. Second, prevalence data for both sexes that could not be split using a within-study ratio were split using a sex ratio derived from a meta-analysis of existing sex-specific data using MR-BRT (meta-regression—Bayesian, regularised, trimmed). The female to male ratio was 1.19 (1.03 to 1.40). Finally, after the application of bias adjustments, where studies reported estimates across age groups spanning 25 years or more, these were split into five-year age groups using the prevalence age pattern estimated by DisMod-MR 2.1 (disease model—Bayesian meta-regression) in GDB 2019.

Data adjustment

We corrected for bias among studies that defined low back pain with too broad an anatomical region, episode duration of greater than three months, recall periods of one week to one month, recall periods between two months and one year, or as activity-limiting LBP, as well as studies conducted among schoolchildren. We added three additional covariates for claims data in the USA from the year 2000 and from 2010 onward and for Taiwan claims data. These adjustment factors were estimated as the logit difference between the prevalence of alternate case definition data and that of the reference definition for comparable age, sex, year, and location calculated using the MR-BRT network crosswalk adjustment method. Unadjusted low back pain prevalence data are often already close to 1, especially for older age groups, and a logit difference strategy ensures that any prevalence data requiring adjustment to a higher value do not exceed 1. Claims data from Taiwan and data on chronic low back pain (duration greater than 3 months) were not included in the final model, as we were unable to find matches to inform a reliable crosswalk. Moreover, in GBD 2021, data on low back pain in schoolchildren and studies reporting back pain in a broad anatomical region were not adjusted, as we could not find matches to inform a crosswalk. Betas and exponentiated values for these covariates are shown in the table below:

Table 2: Crosswalk adjustment factors for LBP

Data input	Reference or alternative case definition	Gamma	Beta coefficient, logit (95% CI)*	Adjustment factor**
Point prevalence	Ref	0.59	---	---
Recall periods of 1 week to 1 month	Alt		0.03 (–0.03 to 0.09)	1.03 (0.97 to 1.092)
Recall periods between 2 months and one year	Alt		0.73 (0.68 to 0.78)	2.08 (1.97 to 02.18)
Activity-limiting LBP	Alt		–1.65 (–1.66 to –1.63)	0.19 (0.19 to 0.20)
USA claims data – 2000	Alt		–1.28 (–1.59 to –0.97)	0.28 (0.20 to 0.37)
USA claims data – 2010–2012, 2014–2017	Alt		–0.66 (–0.81 to –0.51)	0.52 (0.44 to 0.60)

**MR-BRT crosswalk adjustments can be interpreted as the factor the alternative case definition is adjusted by to reflect what it would have been had it been measured using the reference case definition. If the log/logit beta coefficient is negative, then the alternative is adjusted up to the reference. If the log/logit beta coefficient is positive, then the alternative is adjusted down to the reference.*

***The adjustment factor column is the exponentiated beta coefficient. For log beta coefficients, this is the relative rate between the two case definitions. For logit beta coefficients, this is the relative odds between the two case definitions.*

After adjusting data for case definition, we outliered data with a median absolute deviation of 1.5 or more above the age-standardised mean. This was done in a systematic way to cull data that were implausibly high.

Modelling strategy

Prior settings in the DisMod-MR model included setting excess mortality to 0, and it was assumed that there was no incidence or prevalence of low back pain before the age of 5 years. We made no substantive changes in the modelling strategy from GBD 2019. We included the SEV scalar for low back pain as a country covariate. This combines the exposure measures for risks estimated to impinge on LBP in GBD: occupational ergonomic exposure and increased BMI. We set bounds of 0.75 to 1.25 as the SEV is constructed in a way that if our risk estimates are accurate the value should be 1.

Table 3. Covariates. Summary of covariates used in the LBP DisMod-MR meta-regression model

Covariate	Type	Parameter	Log beta (95% uncertainty interval)	Exponentiated beta (95% uncertainty interval)

Log-transformed age-standardised SEV scalar: Back pain	Country-level	Prevalence	0.75 (0.75–0.76)	2.12 (2.12–2.14)

Severity and disability

The basis of the GBD disability weight survey assessments are lay descriptions of sequelae highlighting major functional consequences and symptoms. The lay descriptions and disability weights for LBP severity levels are shown below.

Table 4. Severity distribution, details on the severity levels for LBP in GBD 2019 and the associated disability weight (DW) with that severity.

Severity level	Lay description	DW (95% CI)
Low back pain, mild	This person has mild back pain, which causes some difficulty dressing, standing, and lifting things.	0.020 (0.011–0.035)
Low back pain, moderate	This person has moderate back pain, which causes difficulty dressing, sitting, standing, walking, and lifting things.	0.054 (0.035–0.079)
Low back pain, severe without leg pain	This person has severe back pain, which causes difficulty dressing, sitting, standing, walking, and lifting things. The person sleeps poorly and feels worried.	0.272 (0.182–0.373)
Low back pain, severe with leg pain	This person has severe back and leg pain, which causes difficulty dressing, sitting, standing, walking, and lifting things. The person sleeps poorly and feels worried.	0.325 0.219–0.446)
Low back pain, most severe without leg pain	This person has constant back pain, which causes difficulty dressing, sitting, standing, walking, and lifting things. The person sleeps poorly, is worried, and has lost some enjoyment in life.	0.372 (0.250–0.506)
Low back pain, most severe with leg pain	This person has constant back and leg pain, which causes difficulty dressing, sitting, standing, walking, and lifting things. The person sleeps poorly, is worried, and has lost some enjoyment in life.	0.384 (0.256–0.518)

The severity distributions are derived from an analysis of the Medical Expenditure Panel Surveys (MEPS) in the USA. MEPS is an overlapping continuous panel survey of the United States non-institutionalised population whose primary purpose is to collect information on the use and cost of health care. Panels are two years long and are conducted in five rounds, which are conducted every five to six months. A new panel begins annually, while the last panel is in its second year. Each panel typically contains about 30,000 to 35,000 individual respondents. (http://www.meps.ahrq.gov/survey_comp/hc_data_collection.jsp)

MEPS was initiated in 1996 but only began collecting health status data in the form of SF-12 responses in 2000. We used data from 2000–2014 in our analysis. Respondents self-administer the SF-12 twice per panel, at rounds 2 and 4, typically about a year apart. Only adults 18 years and older completed the SF-12. MEPS also usually collects information on diagnoses based on self-report of reasons for encounters with health services. In addition, diagnoses are derived through additional questions on “problems that

bother you" or conditions that led to "disability days," ie, days out of role due to illness. Professional coders translate the verbatim text into three-digit ICD-9 codes. The main reason for LBP being measured in MEPS relates to health care contact. From MEPS, the severity distribution for LBP without leg pain and with leg pain were derived as shown in the below table.

Table 5. Severity distribution, details on the distribution of severity splits for LBP in GBD 2019 with and without leg pain

Severity level	Distribution without leg pain	Distribution with leg pain
Low back pain, mild	0.41 (0.31–0.53)	0.27 (0.19–0.37)
Low back pain, moderate	0.35 (0.25–0.44)	0.36 (0.28–0.43)
Low back pain, severe	0.10 (0.08–0.12)	0.14 (0.10–0.16)
Low back pain, most severe	0.14 (0.09–0.20)	0.23 (0.15–0.32)

We used USA claims data (2012) to derive the proportion of cases with low back pain who report leg pain. The proportions were different by age group as shown in Figure 2. The proportion in each severity level in each age group is calculated by multiplying the proportion in the severity level and the proportion with or without leg pain.

Figure 2: Proportion of LBP with leg pain

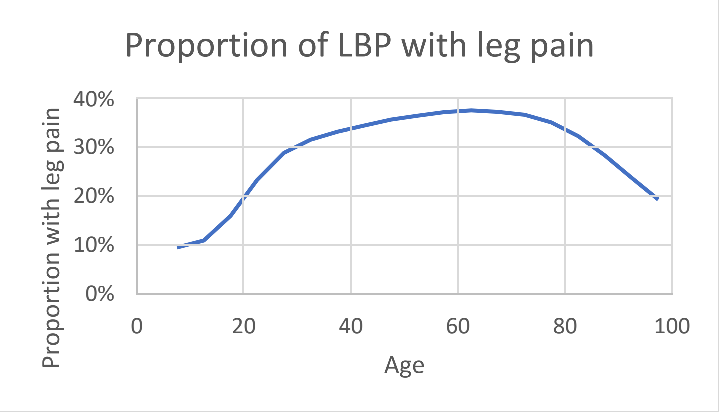

Table 6. Proportion of LBP with leg pain

Age (years)	Proportion with leg pain
5–9	9.4% (9.1–9.8)
10–14	10.9% (10.7–11.1)
15–19	15.9% (15.8–16.1)
20–24	23.2% (23.0–23.4)
25–29	28.8% (28.6–28.9)
30–34	31.4% (31.3–31.6)
35–39	33.1% (32.9–33.2)
40–44	34.3% (34.2–34.4)
45–49	35.5% (35.4–35.6)

50–54	36.4% (36.3–36.5)
55–59	37.1% (37.0–37.2)
60–64	37.4% (37.3–37.5)
65–69	37.1% (36.9–37.3)
70–74	36.5% (36.4–36.7)
75–79	35.0% (34.8–35.2)
80–84	32.1% (31.9–32.4)
85–89	28.3% (28.0–28.5)
90–94	23.7% (23.2–24.2)
95–100	19.2% (18.2–20.2)

Lower respiratory infections (LRI)

Flowchart

Lower respiratory infections

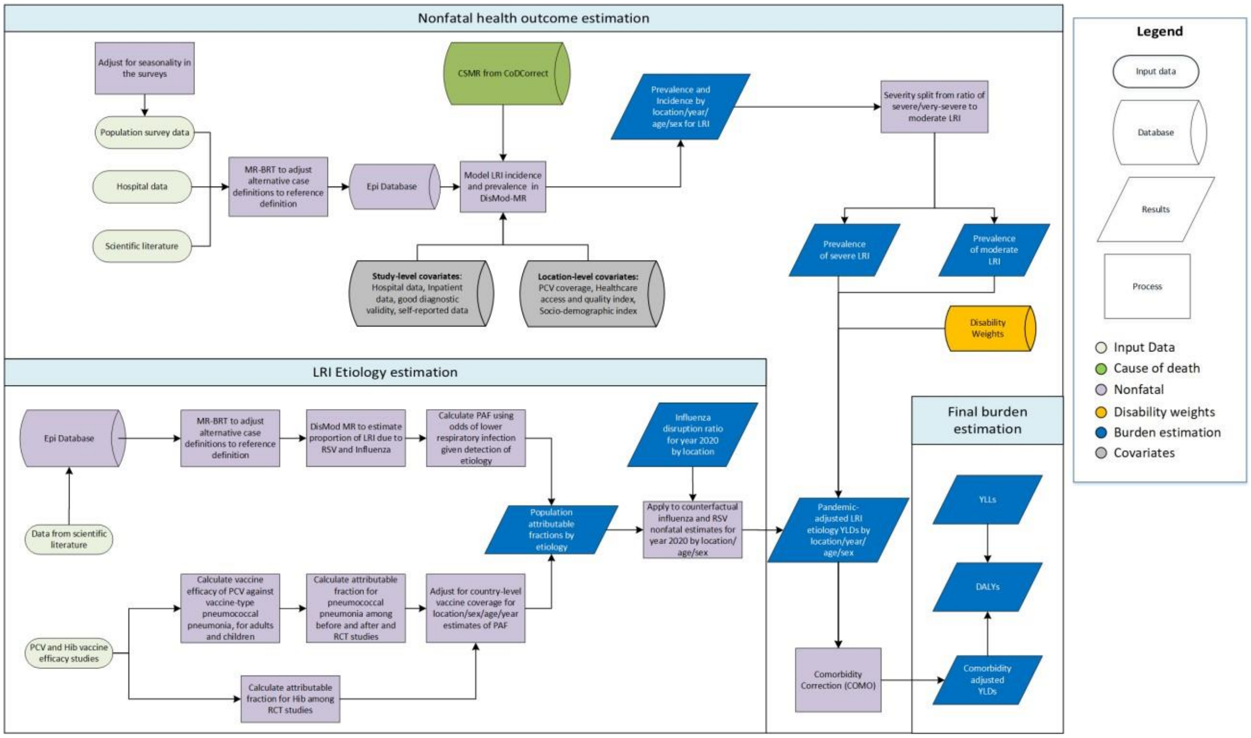

Input data and methodological summary for lower respiratory infections

Case definition

Lower respiratory infections (LRI) are defined by the GBD study as pneumonia or bronchiolitis. Symptoms include cough, fever, and shortness of breath. Included in the GBD modelling were cases meeting ICD-9 diagnostics criteria for LRI (079.82, 466-469, 470.0, 480-481.9, 482.0-482.89, 483.0-483.9, 484.1-484.2,

484.6-484.7, 487-490.9, 510-511.9, 513.0-513.9) and ICD-10 diagnostic criteria for LRI (A48.1, A70, B96.0-96.1, B97.21, B97.4-B97.6, J09-J11.89, J12-J13.9, J14-J14.0, J15-J15.8, J20-J21.9, J85.1, J91.0, P23.0-P23.4, U04-U04.9). In addition, the following garbage codes were redistributed entirely to LRI in ICD-9 (482, 482.9-483, 484, 484.3-484.5, 484.8-486.9, 770.0, V12.61) and ICD-10 (J15.9, J1-J19.6, J22-J22.9, P23, P23.5-P23.9). The GBD case definition of LRI does not include tuberculosis or COVID-19; although these pathogens can infect the lower respiratory tract, they are modelled separately due to their individual public health significance.

Table 1: Case definitions accepted for lower respiratory infections

**Quantity of interest**	**Reference or Alternative**	**Definition**
Incidence or prevalence of lower respiratory infections	Reference	Clinician-diagnosed episode of pneumonia or bronchiolitis
Incidence or prevalence of lower respiratory infections	Alternative	Hospitalised episodes of lower respiratory infection (ICD-9 codes 073.0-073.6, 079.82, 466-469, 480-489, 513.0, and 770.0 and ICD-10 codes A48.1, J09-J22, J85.1, P23-P23.9, and U04)
Prevalence of lower respiratory infections	Alternative	Maternal-reported symptoms of under-5 acute lower respiratory infections including cough with difficulty breathing, fever, and symptoms in the chest.

Input data

Overall LRI

Input data included all data used in GBD 2019 and new data identified in our updated systematic review, newly acquired surveys, and new claims and inpatient data. These data measure lower respiratory infection incidence and prevalence. They come from a systematic literature review, hospital inpatient and outpatient data, claims data from the USA, and population-representative surveys.

Prisma diagram:

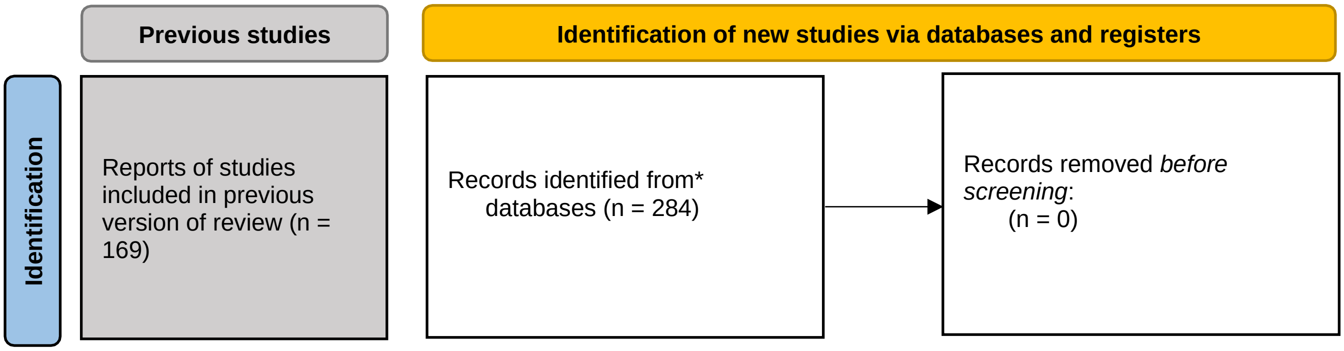

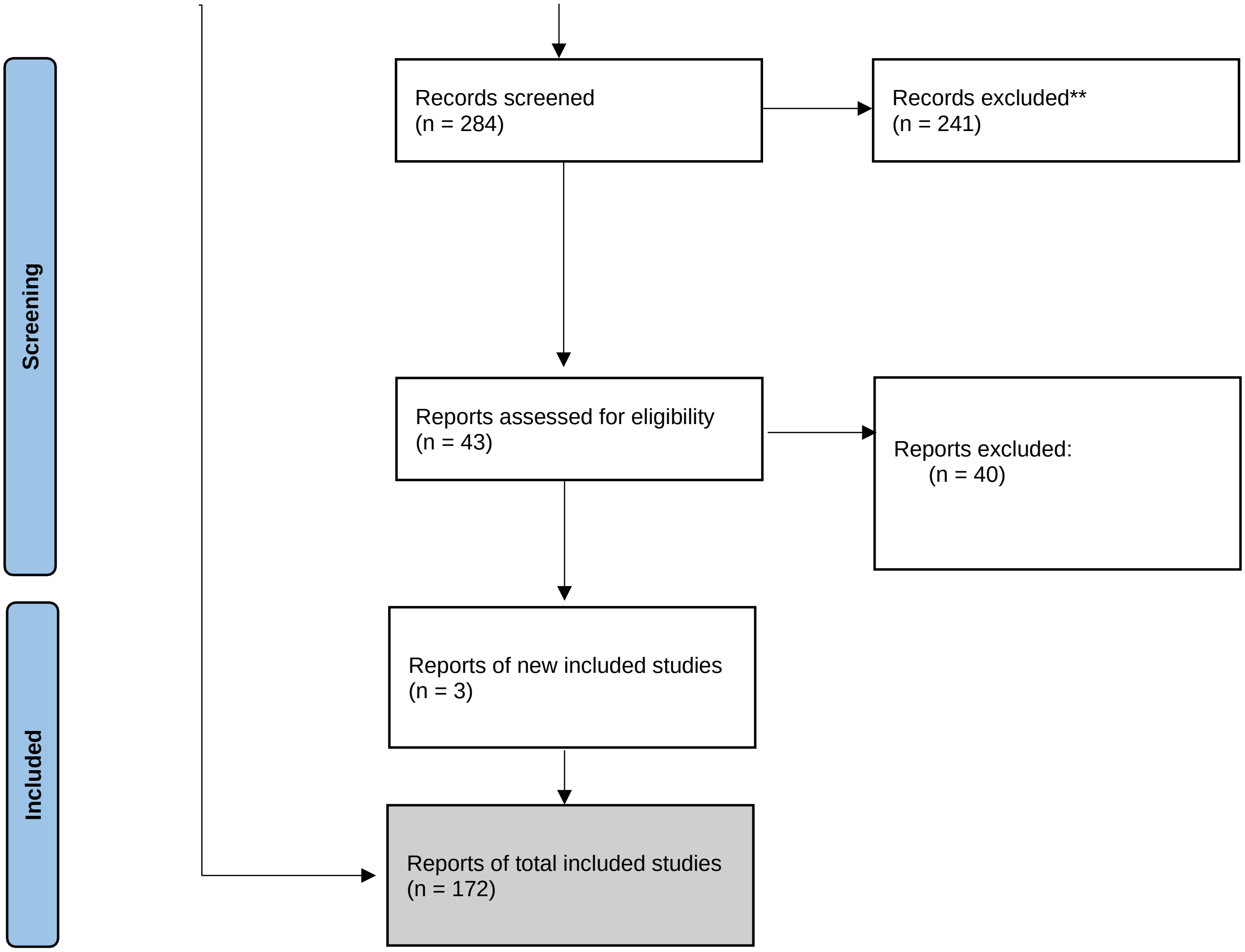

Table 1. Unique source counts for lower respiratory infections by parameter

	Countries with data	New sources	Total sources
Prevalence	156	71	968
Incidence	162	2058	2058
Remission	0	0	0
Other	132	110	389

Data were outliered or excluded if we found them unreasonable when compared to regional, super-regional, and global rates.

To estimate the non-fatal burden of LRI, we also used self-reported prevalence of LRI symptoms from population-representative surveys, such as the Demographic and Health Survey and the Multiple Indicator Cluster Survey. When possible, we extracted survey data by one-year age group and by sex. We

converted these data from two-week period prevalence to point prevalence. The equation for this adjustment is:

$$1)\ Point\ Prevalence = \frac{Period\ Prevalence * Duration}{(Recall\ Period + Duration - 1)}$$

We accepted four survey definitions for the prevalence of symptoms of LRI: 1) Cough with difficulty breathing with symptoms in the chest with a fever was our gold standard, but we also accepted 2) Cough with difficulty breathing with symptoms in the chest *without* fever, 3) Cough with difficulty breathing with fever, and 4) Cough with difficulty breathing *without* fever. To make these definitions comparable, we identified the surveys that met the best case definition (definition 1). Within these surveys, we calculated the ratio of the prevalence of the best case definition to the prevalence of the alternate definitions. This ratio was used as the dependent variable in a meta-regression. The results from that meta-regression were used to adjust the prevalence and uncertainty for all the surveys that reported alternate case definitions **(Table 2a)**.

Table 2a: MR-BRT crosswalk adjustment factors for lower respiratory infections, surveys

Data input	Reference or alternative case definition	Gamma	Crosswalk covariate	Beta coefficient, logit (95% UI)	Adjustment Factor**
Cough, with difficulty breathing and fever	ref	--	--	--	--
Survey, chest without fever	alt	0.17	intercept	–0.48 (–1.28 to 0.32)	0.62
Survey, difficulty breathing without fever	alt	0.51	intercept	–0.82 (–2.22 to 0.58)	0.44
Survey, difficulty breathing with fever	alt	0.22	intercept	–0.58 (–1.5 to 0.34)	0.56

Survey data were adjusted for seasonality. An inclusion criterion for scientific literature is a study duration longer than one year to avoid bias in the seasonal timing of LRI. Surveys are frequently conducted over several months. To account for seasonal variation in LRI symptom prevalence, we fit a generalised additive model with a forced periodicity for each GBD region. The model is mixed-effects with random effects on each country. The model accounts for the year of the survey and the case definition used. The percentage difference between the monthly model-fit LRI prevalence and the mean fitted LRI prevalence is a scalar to adjust survey data by month and geography.

In addition to survey data, hospital inpatient and USA inpatient claims data were included in the LRI modelling. These data are adjusted prior to modelling for readmissions and multiple diagnoses. To make the data more consistent in the modelling process, we converted all incidence data to prevalence. We found the ratio of the prevalence of LRI in hospitalisation records to the prevalence of LRI in our case definition (clinician-diagnosed pneumonia or bronchiolitis) for locations that contained data on both these prevalence values. We then regressed this ratio in a meta-regression to predict the adjustment

factor for hospitalisation data to make them compatible with the reference case definition for our modelling. This meta-regression considered the Socio-demographic Index (SDI) as a predictor of this ratio for inpatient data, assuming that location-years with higher values of SDI are more likely to have access to health care, making this ratio smaller in those location-years **(Table 2b)**. Similarly, age was considered a predictor for hospital-based studies, and data were adjusted accordingly using age midpoint **(Table 2b)**.

Claims data for GBD 2019 include MarketScan (USA), and data from Taiwan (province of China), Poland, and Russia. MarketScan data are retrieved by IHME's Clinical Informatics Team. As with inpatient clinical data, these data are converted first to prevalence, then compared to the reference definition for LRI using a meta-regression model **(Table 2b)**. Taiwan claims data were dropped as there were no reference data to match with and because the values there were systematically different from those in the USA.

Table 2b: MR-BRT crosswalk adjustment factors for lower respiratory infections: clinical inpatient, hospital-based studies, and inpatient claims to reference

Data input	Reference or alternative case definition	Gamma	Crosswalk covariate	Beta coefficient, logit (95% UI)*	Adjustment factor**
Clinician-diagnosed pneumonia or bronchiolitis	ref		--	--	--
Clinical, inpatient	alt	1.43	sdi_0	2.79 (0.2 to 5.38)	16.23
Clinical, inpatient	alt		sdi_1	4.87 (2.31 to 7.43)	129.85
Clinical, inpatient	alt		sdi_2	1.08 (–1.49 to 3.65)	2.94
Clinical, inpatient	alt		sdi_3	0.02 (–2.43 to 2.47)	1.02
Literature, hospital-based	alt	0.30	age_mid_0	1.06 (–0.31 to 2.42)	2.87
Literature, hospital-based	alt		age_mid_1	1.98 (–0.42 to 4.38)	7.23
Literature, hospital-based	alt		age_mid_2	1.31 (–0.11 to 2.74)	3.72
Literature, hospital-based	alt		age_mid_3	0.95 (–0.2 to 2.1)	2.59
Self-report	alt	0.81	Intercept	–1.19 (–2.98 to 0.6)	0.30
Claims, MarketScan	alt	0.87	intercept	1.14 (–0.69 to 2.97)	3.13

**MR-BRT crosswalk adjustments can be interpreted as the factor the alternative case definition is adjusted by to reflect what it would have been had it been measured using the reference case definition. If the log/logit beta coefficient is negative, then the alternative is adjusted up to the reference. If the log/logit beta coefficient is positive, then the alternative is adjusted down to the reference.*

***The adjustment factor column is the exponentiated beta coefficient. For log beta coefficients, this is the relative rate between the two case definitions. For logit beta coefficients, this is the relative odds between the two case definitions.*

We performed a systematic review of the duration of symptoms of LRI. We sought consistency with our case definition of LRI and defined our duration as the time between the onset of symptoms to the resolution of increased work of breathing. Although crucial, there were very limited data on spatial, temporal, or age-specific duration, which may vary based on severity, aetiology, and treatment. We identified 485 titles from PubMed and extracted six studies which were used in a meta-analysis (mean duration 7.79 days [6.2–9.64]). We used this as the duration of LRI in our conversions from period to point prevalence and for the conversion between incidence and prevalence.

Severity splits

The distribution of moderate (85%) and severe (15%) lower respiratory infections is determined by a meta-analysis of the ratio of severe to all LRI from studies that report the incidence of moderate and severe lower respiratory infections.

We used the health states of acute infectious disease episode, moderate and severe, with the lay descriptions and disability weight values shown in **Table 3** below:

Table 3. Severity distribution, details on the severity levels for lower respiratory infections and the associated disability weight (DW) with that severity.

Severity level	Lay description	DW (95% CI)
Moderate	Has a fever and aches and feels weak, which causes some difficulty with daily activities.	0.051 (0.032–0.074)
Severe	Has a high fever and pain and feels very weak, which causes great difficulty with daily activities.	0.133 (0.088–0.19)

Aetiologies

Input data for aetiology estimation consisted of multiple cause of death, vital registration, hospital discharge, and microbial data, as well as the aforementioned systematic literature review and a separate, targeted review pulling data from citations found in meta-analyses. For data sources that provided ICD codes (multiple cause of death, vital registration, hospital discharge, and some microbial data), these codes were used to identify patients with lower respiratory tract infections and the culprit pathogen, when detailed. For the microbial data that did not provide ICD codes, we identified pathogens associated with LRI based on the type of sample that was collected from the patient. Samples we deemed related to LRI included sputum, aspirates from the lower respiratory tract, and pleural fluid. We excluded samples from the eyes, ears, nose, or throat.

Table 4: ICD codes used to identify LRI cases with known aetiology

**Type of LRI**	**ICD-10 code(s)**	**ICD-9 code(s)**

LRI due to *Bordetella pertussis*	A37-A37.9	033-033.9, 484.3
LRI due to *Legionella spp.*	A48.1-A48.2	--
LRI due to *Actinomyces*	--	039.1
LRI due to *Chlamydia spp.*	A70, J16.0, P23.1	073-073.9, 483.1, 484.2
LRI due to *Streptococcus pneumoniae*	J13-J13.9, J15.4, J20.2	481-481.9, 482.3
LRI due to *Haemophilus influenzae*	J14-J14.0, J20.1	482.2
LRI due to *Klebsiella pneumoniae*	J15.0	482.0
LRI due to *Pseudomonas spp.*	--	482.1
LRI due to *Pseudomonas aeruginosa*	J15.1, P23.5	--
LRI due to *Staphylococcus aureus*	J15.2, P23.2	482.4
LRI due to Group B *Streptococcus*	J15.3, P23.3	--
LRI due to *Escherichia coli*	J15.5, P23.4	--
LRI due to *Mycoplasma pneumoniae*	J15.7, J20.0	483.0
LRI due to *Francisella tularensis*	--	484.4
LRI due to *Bacillus anthracis*	--	484.5
LRI due to virus	--	079.6-079.7, 480-480.9, 484.0-484.1, 487-489
LRI due to coronaviruses	B34.2, B97.2, J12.8	--
LRI due to respiratory syncytial virus	B97.4, J12.1, J20.5, J21.0	--
LRI due to influenza viruses	J09-J11.8	--
LRI due to parainfluenza viruses	J12.2, J20.4	--
LRI due to adenoviruses	J12.0	--
LRI due to rhinoviruses	J20.6	--
LRI due to other virus	J12, J12.3, J12.9, J17.0, J17.2-J17.8, J20.3, J20.7-J20.8, J21.1	--

Data on pathogens cultured from human infections were solicited from a wide array of international stakeholders (representing every inhabited continent). These included research hospitals, surveillance networks, and infection databases maintained by private laboratories and medical technology

companies. For a full list of non-literature sources used for our estimates, please refer to the following article appendix (section 2).1 For cases in which both a bacterial and a non-bacterial pathogen were detected, the bacterial pathogen was preferentially recorded. Only co-infections of bacterial plus bacterial, or non-bacterial plus non-bacterial, were recorded as polymicrobial.

Due to the documented challenge in the microbiological identification of some LRI culprit pathogens,[2,3] we supplemented these data with estimates of the PAF of pneumonia due to *Streptococcus pneumoniae* (pneumococcus), which was calculated based on vaccine efficacy data reported in 18 high-quality vaccine probe studies.

We conducted a systematic literature review of PCV efficacy studies until January 2020. For PCV studies, we extracted, if available, the distribution of pneumococcal pneumonia serotypes and the serotypes included in the PCV used in the study. Four new studies were identified for GBD 2021, which were all extracted only from PCV efficacy studies. PCV trial data are also frequently limited to younger populations. To understand the contribution of pneumococcal pneumonia in older populations, we also included PCV efficacy studies that used before-after approaches.

---

[1] Murray CJ, Ikuta KS, Sharara F, Swetschinski L, Aguilar GR, Gray A, Han C, Bisignano C, Rao P, Wool E, Johnson SC. Global burden of bacterial antimicrobial resistance in 2019: a systematic analysis. *The Lancet*. 2022 Feb 12;399(10325):629-55.
[2] Ewig S, Schlochtermeier M, Goïke N, Niederman MS. Applying sputum as a diagnostic tool in pneumonia: limited yield, minimal impact on treatment decisions. *Chest*. 2002 May 1;121(5):1486-92.
[3] Ogawa H, Kitsios GD, Iwata M, Terasawa T. Sputum Gram stain for bacterial pathogen diagnosis in community-acquired pneumonia: a systematic review and Bayesian meta-analysis of diagnostic accuracy and yield. *Clinical Infectious Diseases*. 2020 Jul 27;71(3):499-513.

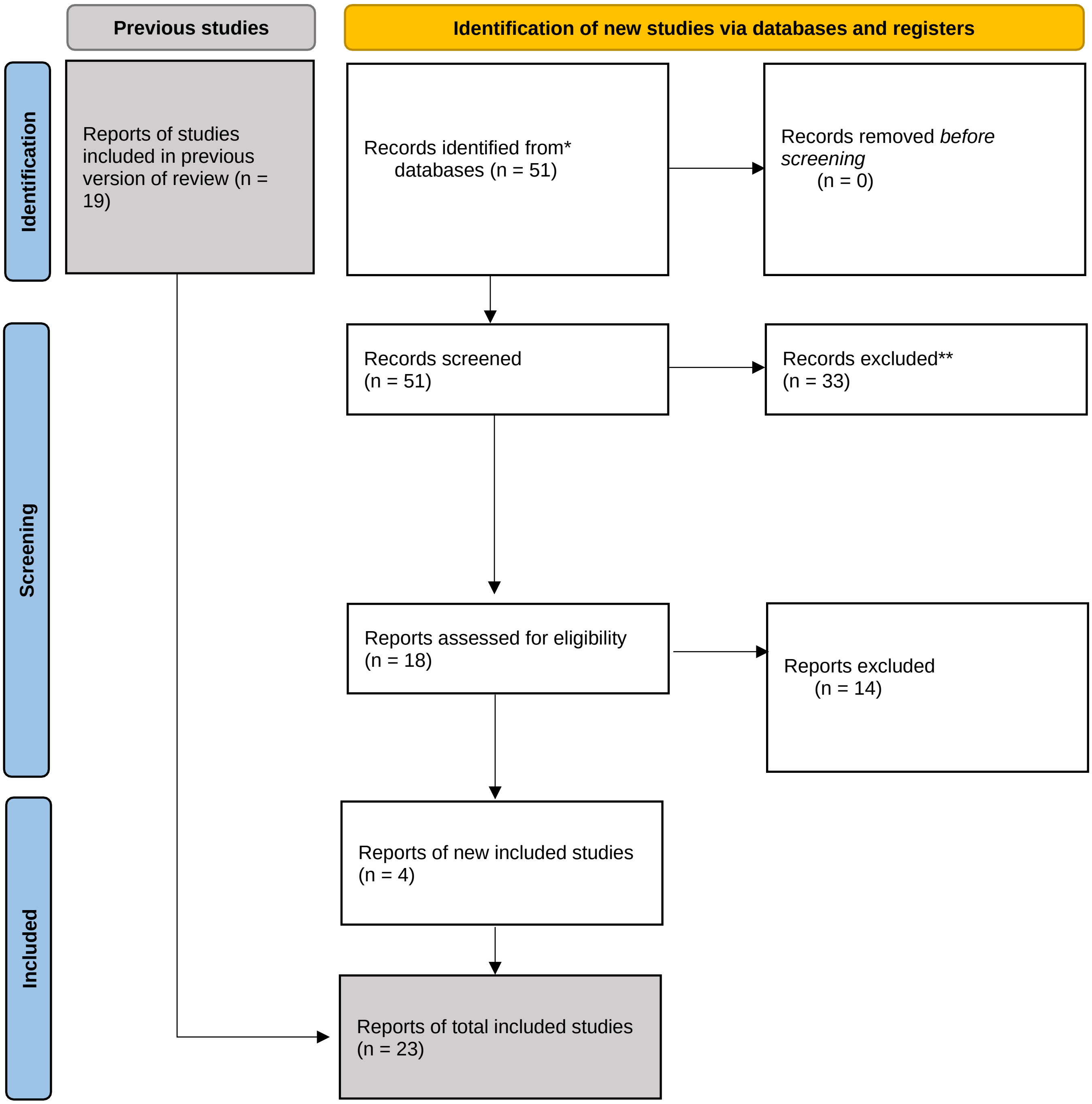

Figure 2. PCV vaccine efficacy systematic review flowchart

Table 5: Data inputs for lower respiratory infections morbidity modelling by parameter

	**Countries with data**	**New sources**	**Total sources**
Incidence	0	0	0
Prevalence	156	71	970

Remission	0	0	0
Other	146	114	633

Modelling strategy

Overall LRI

The non-fatal lower respiratory infection burden is modelled in DisMod-MR, a Bayesian meta-regression modelling framework. DisMod-MR produces estimates of the incidence, prevalence, and remission of LRI for each age, sex, geographical location, and year. We defined the time to recovery as an average of 10 days (5–15 days), which corresponds with a remission of 36.5. The models are informed by country-level covariates (**Table 6**).

Table 6. Covariates used in the lower respiratory infections DisMod-MR meta-regression model

Covariate	Type	Parameter	Exponentiated beta (95% uncertainty interval)
Socio-demographic Index	Country-level	Prevalence	0.14 (0.14–0.14)
Healthcare Access and Quality Index	Country-level	Excess mortality	0.37 (0.14–0.95)

We adjusted overall LRI incidence and prevalence estimates for 2020 and 2021 to account for the reductions in influenza and RSV cases associated with the COVID-19 pandemic, as described elsewhere in this appendix.

Aetiology estimation

We estimated mutually exclusive proportions of LRI cases attributable to the following set of pathogens: *Acinetobacter baumannii*, *Chlamydia* spp., *Enterobacter* spp., *Escherichia coli*, fungi, group B *Streptococcus*, *Haemophilus influenzae*, influenza, *Klebsiella pneumoniae*, *Legionella* spp., *Mycoplasma* spp., polymicrobial infections, *Pseudomonas aeruginosa*, respiratory syncytial virus (RSV), *Staphylococcus aureus*, *Streptococcus pneumoniae*, and other viruses, as well as a residual "other pathogen" category. These proportions were estimated for five aggregate age groups: neonatal, post-neonatal to 5 years, 550 years, 50–70 years, and 70 years or older. Unspecified or unknown pathogens were excluded from the analysis.

We estimated LRI aetiologies separately from overall LRI mortality and morbidity using two distinct counterfactual modelling strategies to estimate population attributable fractions (PAFs), described in detail below. The PAF represents the relative reduction in LRI mortality if there was no exposure to a given aetiology. We calculated uncertainty of our PAF estimates from 1000 draws of each parameter using normal distributions in log space.

Streptococcus pneumoniae

For *Streptococcus pneumoniae* (pneumococcal pneumonia), we calculated the population attributable fraction using a vaccine probe design[1] due to the documented challenge in the microbiological

[1] Feikin DR, Scott JA, Gessner BD. Use of vaccines as probes to define disease burden. *The Lancet*. 2014 May 17;383(9930):1762-70.

identification of this pathogen.[Error! Bookmark not defined.,Error! Bookmark not defined.] The ratio of vaccine efficacy ag ainst all pneumonia (non-pathogen-specific) to vaccine-type, pathogen-specific disease represents the fraction of pneumonia cases attributable to each pathogen.

To estimate the PAF for pneumococcal pneumonia, we calculated study-level PAFs as the ratio of vaccine efficacy against all pneumonia to vaccine-type pathogen-specific pneumonia (Equations 1 & 2). For pneumococcal pneumonia, we used only the vaccine efficacy against vaccine-type pneumococcal pneumonia. This value was available in three studies and was calculated separately for children and adults, pooling the results of the Cutts[1] and Madhi[2] studies for children and using the Bonten[3] study for adults. Vaccine efficacy for all pneumonia was available at the study level. To estimate the PAF for pneumococcal pneumonia, we included RCTs and before-and-after vaccine introduction longitudinal studies.

For pneumococcal pneumonia, we adjusted the PAF by vaccine serotype coverage. Finally, we used an age distribution of PAF modelled in MR-BRT to determine the PAF by age. Because of an absence of data describing vaccine efficacy against Hib in children older than 2 years, we did not attribute Hib to episodes of LRI in ages 5 years and older.

We used a vaccine probe design to estimate the PAF for pneumococcal pneumonia and (Hib) by first calculating the ratio of vaccine efficacy against all pneumonia to pathogen-specific pneumonia at the study level (Equation 1).[Error! Bookmark not defined.,4,5] We then adjusted this estimate by vaccine coverage and expected vaccine performance to estimate country- and year-specific PAF values (Equation 2).

$$1)\quad PneumoPAF_{Base} = \frac{VE_{all\ pneumonia}}{VE_{vt\_pneumococcal\_pneumonia} * Cov_{Serotype}}$$

$$2)\quad PAF_{Pneumo} = PneumoPAF_{Base} * \frac{(1 - Cov_{PCV} * VE_{all\ pneumonia})}{(1 - PneumoPAF_{Base} * Cov_{PCV} * VE_{all\_pneumonia})}$$

---

[1] Cutts FT, Zaman SM, Enwere GY, Jaffar S, Levine OS, Okoko JB, Oluwalana C, Vaughan A, Obaro SK, Leach A, McAdam KP. Efficacy of nine-valent pneumococcal conjugate vaccine against pneumonia and invasive pneumococcal disease in The Gambia: randomised, double-blind, placebo-controlled trial. *The Lancet*. 2005 Mar 26;365(9465):1139-46.

[2] Madhi SA, Kuwanda L, Cutland C, Klugman KP. The impact of a 9-valent pneumococcal conjugate vaccine on the public health burden of pneumonia in HIV-infected and-uninfected children. *Clinical infectious diseases*. 2005 May 15;40(10):1511-8.

[3] Bonten MJ, Huijts SM, Bolkenbaas M, Webber C, Patterson S, Gault S, van Werkhoven CH, van Deursen AM, Sanders EA, Verheij TJ, Patton M. Polysaccharide conjugate vaccine against pneumococcal pneumonia in adults. *New England Journal of Medicine*. 2015 Mar 19;372(12):1114-25.

[4] O'Brien KL, Wolfson LJ, Watt JP, Henkle E, Deloria-Knoll M, McCall N, Lee E, Mulholland K, Levine OS, Cherian T. Burden of disease caused by Streptococcus pneumoniae in children younger than 5 years: global estimates. *The Lancet*. 2009 Sep 12;374(9693):893-902.

[5] Watt JP, Wolfson LJ, O'Brien KL, Henkle E, Deloria-Knoll M, McCall N, Lee E, Levine OS, Hajjeh R, Mulholland K, Cherian T. Burden of disease caused by Haemophilus influenzae type b in children younger than 5 years: global estimates. *The Lancet*. 2009 Sep 12;374(9693):903-11.

Where $VE_{all\_pneumonia}$ is the vaccine efficacy against non-specific pneumonia, $VE_{vt\_pneumococcal\_pneumonia}$ is the vaccine efficacy against vaccine-type pneumococcal pneumonia, $Cov_{serotype}$ is the serotype-specific vaccine coverage for PCV,[1] and Cov_{PCV} is the PCV coverage.

We used the PAF_{Pneumo} as an input to our aetiology estimation model, described below, where it represented the proportion of LRI incidence attributable to *Streptococcus pneumoniae*. The remainder, $1 - PAF_{Pneumo}$, represented "non-pneumococcus" LRI, and was represented as a composite of all of the non-*Streptococcus pneumoniae* pathogens we estimated as well as the residual "other pathogens" category.

Aetiology estimation model: MEPCO

Aetiology proportions were calculated using an entirely new method from that applied in previous rounds of the GBD. Working from the assumption that aetiologies would follow a multinomial distribution, we estimated aetiology fractions using a method previously described as multinomial estimation of partial and composite observations (MEPCO).**Error! Bookmark not defined.** Briefly, we constructed a n etwork model with the dependent variable as the log ratio of cases between different pathogens. Due to vastly different aetiology proportions among neonates relative to other ages, we estimated neonatal aetiologies separately. While the model estimates both the proportions of hospital- and community-acquired LRI cases attributable to each aetiology, for the GBD we only report community-acquired aetiologies, as hospital-acquired infection would occur with a non-LRI underlying cause.

Table 7: Covariates used in aetiology modelling

Covariate	Model
Age group (neonatal, post-neonatal to 5, 5–50, 50–70, 70 plus)	Non-neonatal
Healthcare Access and Quality Index	Neonatal, non-neonatal
Community vs. hospital-acquired infection	Neonatal, non-neonatal
Proportion of people who as infants were vaccinated against pneumococcus	Non-neonatal
Proportion of population age 15 or younger vaccinated against pneumococcus	Neonatal, non-neonatal
Proportion of people who as infants were vaccinated against *Haemophilus influenzae* type B	Non-neonatal
Proportion of population age 15 or younger vaccinated against *Haemophilus influenzae* type B	Neonatal, non-neonatal

Due to inconsistencies in which pathogens are tested for and reported by different data sources, each data source contained partial observations of the possible outcomes of the underlying multinomial distribution. Certain data sources like the vaccine probe estimates represent compositional observations, where pathogens like "not *S. pneumoniae"* represent aggregates of more detailed pathogens.

[1] Johnson HL, Deloria-Knoll M, Levine OS, Stoszek SK, Freimanis Hance L, Reithinger R, Muenz LR, O'Brien KL. Systematic evaluation of serotypes causing invasive pneumococcal disease among children under five: the pneumococcal global serotype project. *PLoS medicine*. 2010 Oct 5;7(10):e1000348.

To use both partial and compositional data, we constructed a network model with the dependent variable as the log ratio of cases between different pathogens and estimated over a flexible parameterisation of multinomial parameters using a maximum likelihood approach. Consider a given infectious syndrome with a multinomial distribution of n mutually exclusive, collectively exhaustive aetiologies with probabilities $p = (p_1, \ldots, p_n)$, so that each $p_j \in (0,1)$ and $\sum_j p_j = 1$. The likelihood of an observation of $c = (c_1, \ldots, c_n)$, where c_j = number of cases of pathogen j in a total sample of N infections ($\sum_j c_j = N$), is:

$$P(c|p) = N! \prod_{j=1}^{n} \frac{p_j^{c_j}}{c_j!} \quad (1)$$

We modelled the probabilities using a composition of a link function with a linear predictor:

$$p_{i,j} = \exp(x_{i,j}^T \beta_j) \quad (2)$$

for observations i, a vector of covariates $x_{i,j}$, and a vector of coefficients β_j for each pathogen j. However, we did not observe these probabilities directly. Rather, we observed ratios between sums of these probabilities, which reduce to ratios between sums of cases within each study. These observations therefore take the form:

$$y = \frac{cases\ of\ pathogen\ A}{cases\ of\ pathogen\ B} = \frac{\sum_{j=1}^{n} w_{i,j}^a \exp(x_{i,j}^T \beta_j)}{\sum_{j=1}^{n} w_{i,j}^b \exp(x_{i,j}^T \beta_j)} \quad (3)$$

where $w_{i,j}^a$ is a weight of 0 or 1 that selects the mutually exclusive, collectively exhaustive most-detailed pathogens that make up observed pathogen A, which may be a composite observation. For example, for the "non-*Streptococcus pneumoniae*" pathogen, $w_{i,j}$ would be 1 for *Acinetobacter baumannii*, *Chlamydia* spp., *Enterobacter* spp., *Escherichia coli*, fungi, group B *Streptococcus*, *Haemophilus influenzae*, influenza, *Klebsiella pneumoniae*, *Legionella* spp., *Mycoplasma* spp., polymicrobial infections, *Pseudomonas aeruginosa*, respiratory syncytial virus (RSV), *Staphylococcus aureus*, other viruses, and the residual "other pathogen" category and 0 for *Streptococcus pneumoniae* . We dropped all observations where either the numerator or denominator had zero observed cases in order to make this calculation and a forthcoming log transform possible. This may bias the model towards overestimating less common pathogens.

It is not possible to infer all coefficients β_j from the observations, since they are all relative. However, if we fix all of the coefficients for one pathogen to zero as a reference group, then we obtain a well-posed inverse problem, as long as there is enough data to estimate the remaining coefficients. Without loss of generality, we assumed $\beta_1 = 0$ for all elements and obtain estimates of the remaining $\beta_2, \ldots, \beta_n$ by minimising the sum of the residuals between log-transformed observations y and corresponding log-transformed predictions from equation 3:

$$\min_{\beta_2,\ldots,\beta_n} f(\beta) := \sum_i \frac{1}{\sigma_i^2} \left[\ln(y_i) - \ln\left(\sum_{j=1}^{n} w_{i,j}^a\, exp(x_{i,j}^T \beta_j)\right) + \ln\left(\sum_{j=1}^{n} w_{i,j}^b\, exp(x_{i,j}^T \beta_j)\right)\right]^2 \quad (4)$$

where σ_i^2 are variances corresponding to the datapoints. Equation 4 is a non-linear likelihood minimisation problem that that we optimised using a standard implementation of the Gauss-Newton method.[1] We then re-normalised the optimal coefficients to obtain final predictions of the probabilities of each pathogen:

$$p_{i,j} = \frac{\exp(x_{i,j}^T \beta_j)}{\sum_{\hat{j}} \exp(x_{i,\hat{j}}^T \beta_{\hat{j}})} \tag{5}$$

To quantify the uncertainty of this estimate, we used asymptotic statistics to obtain the posterior distribution of $(\beta_2, \dots, \beta_n)$. Specifically, using the Gauss-Newton Hessian approximation gave us the asymptotic information matrix for all β_j except for the reference pathogen, allowing us to sample draws of $\beta = (\beta_1 = 0, \beta_2, \dots, \beta_n)$. For each β draw and given feature x, we obtained a corresponding draw of p using equation 6.3.1.5.

This network regression with covariates framework allowed us to use partial and composite data that reported on one or only a few pathogens, or that reported multiple pathogens aggregated together. Networks, however, can be unstable with sparse data, and stable estimates have in some cases required the use of Bayesian priors in these models. In particular, we imposed Gaussian priors with mean 0 and non-zero variance on all coefficients except intercepts, to bias the model away from spurious effects driven by data sparsity. For the neonatal model, a prior standard deviation of 0.2 was used. For the non-neonatal model, we used a standard deviation of 0.1.

Changes from GBD 2019

The method of aetiology calculation is entirely new for all aetiologies except for *Streptococcus pneumoniae,* using the method described here.**Error! Bookmark not defined.** For *Streptococcus pneumoniae*, we r etained the vaccine probe approach but made key methodological improvements to the method, which had been implemented in previous rounds of the GBD. The base pneumococcal PAF no longer includes vaccine efficacy against invasive pneumococcal disease multiplied by an adjustment factor; instead, we use only the vaccine efficacy against vaccine-type pneumococcal pneumonia. In addition, there is no longer *Haemophilus influenzae* type B adjustment in the pneumococcal PAF. Pneumococcus PAF data based on the vaccine efficacy studies was directly incorporated in our models and supplanted the pneumococcus data collected from literature data and microbiology labs, which was removed due to the potential for underestimation.

[1] Nocedal J, Wright SJ, editors. *Numerical Optimization*, 2nd edn. New York: Springer-Verlag, 2006 DOI:10.1007/978-0-387-40065-5.

Lymphatic filariasis

Flowchart

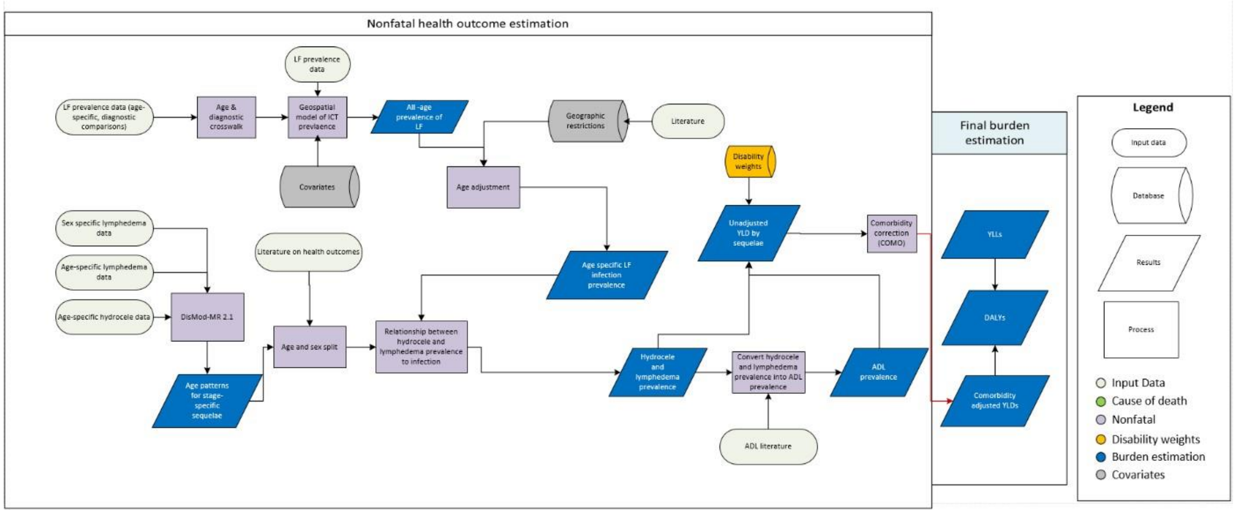

Input data and methodological summary

Case definition

Lymphatic filariasis (LF) is a neglected tropical disease in which threadlike nematodes invade the lymphatic system. The worms responsible – *Wuchereria bancrofti*, *Brugia malayi,* and *Brugia timori* – are spread from human to human via mosquitoes. Chronic infection can lead to lymphoedema (a swelling of the legs, also known in its more extreme manifestation as elephantiasis), hydrocele (a collection of fluid in the sac around the testicles), as well as recurrent debilitating episodes of acute adenolymphangitis.

We used the following case definition for GBD 2021:

Quantity of interest	Reference or alternative	Definition
Lymphatic filariasis	Reference	Prevalent cases of lymphatic filariasis as confirmed through antigenaemia (ICT) diagnostic testing.
Lymphatic filariasis	Alternative	Prevalent cases of lymphatic filariasis as confirmed through microfilariaemia (MF) diagnostic testing.

Input data

A systematic review of literature for GBD 2016 in the PubMed database was done on October 14, 2016, for prevalence and incidence data using the search (Lymphatic filariasis AND prevalence) OR (Lymphatic filariasis AND (prevalence OR incidence OR "mass drug administration" OR MDA OR coverage)) OR (Lymphoedema, hydrocele) OR (Transmission Assessment Survey (TAS)) OR (Lymphatic filariasis AND mapping). This literature review was updated again in May 2019. Additional data on LF infection prevalence collected under the Global Programme for the Elimination of Lymphatic Filariasis were obtained through the Expanded Special Project for Elimination of Neglected Tropical Diseases and the World Health Organization.

Table 1. Total data source counts

Measure	Countries with data	New sources	Total sources
All measures	72	0	565
Prevalence	72	0	565

Modelling strategy

We first model the prevalence of LF infection represented by immunochromatographic test (ICT) using a geospatial model, as described by Cromwell et al. (2020), to generate an estimate of all-age prevalence. We then relate the prevalence of LF infection to the prevalence of hydrocele and lymphoedema, and ADL.

Model of LF infection prevalence

Covariates

The geospatial model relied on covariates at the 5 × 5-km grid-cell resolution to represent environmental factors associated with LF transmission, including elevation, precipitation, vegetation, and temperature, as well as socioeconomic measures potentially associated with vector-borne disease burden. Geospatial estimates of population coverage with insecticide-treated bed nets (ITN), indoor residual spraying, and LF MDA (of any drug regimen) were included to account for interventions known to reduce transmission, and malaria (*Plasmodium falciparum* and *Plasmodium vivax*) prevalence and incidence were included as proxies for exposure to vector-borne disease. VIF analysis was performed to identify the set of covariates for modelling. The final analyses included a total of 23 covariates for Africa, 22 covariates for south Asia, 21 covariates for southeast Asia and 21 covariates for Hispaniola.

Age & diagnostic adjustment

In order to derive a global estimate of LF infection using data reported across different age and diagnostic categories, reflecting all-age infection prevalence, we used age and diagnostic crosswalk models to adjust the input data prior to the main modelling analysis. Due to the introduction and rapid adoption of ICT card tests in the mid-2000s and their higher sensitivity, data derived from identification of MF by blood microscopy were first adjusted to be comparable with ICT prevalence estimates. Prevalence measured in a single age group (typically adults in baseline surveys or children in TAS) were adjusted to reflect all-age prevalence. We identified peer-reviewed published surveys that reported prevalence in at least two age groups in the same study population. The non-linear age-dependent relationship between MF and ICT prevalence was then calculated using surveys that reported both measures by fitting a logistic regression model with a basis spline on the ratio of ICT to MF prevalence by age. The age crosswalk model was similarly structured and was fit using surveys reporting ICT prevalence for multiple age groups.

Geostatistical analysis

Bayesian geostatistical models were fit separately for each of the following modelling regions based on a review of LF endemicity: (1) Africa and Yemen, including Madagascar, São Tomé and Príncipe, and

Comoros; (2) South and southeast Asia; and (3) the island of Hispaniola. We first employed an ensemble method to select covariates, capture possible non-linear effects, and account for the complex interactions among them. For each modelling region, we fit three sub-models to predict prevalence of LF for geo-referenced datapoints, with cross validation: generalised additive models (GAM), generalised boosted models (GBM), and lasso regression. All sub-models included country-level fixed effects. We modelled LF infection prevalence using a spatially and temporally explicit generalised linear mixed effects model via integrated nested Laplace approximation (INLA). The spatiotemporal variation beyond that described by the included covariates was modelled as a Gaussian process with covariance as a Kronecker product of the spatial and temporal error processes. Spatial covariance was modelled using a Matérn function, and the temporal covariance was modelled using a first- or second-order autoregressive function. Predictions were generated using the in-sample sub-model predictions as covariates and summarising 1000 samples from the posterior distribution as the mean; 95% uncertainty intervals (UIs) were generated from the 2·5th and 97·5th percentiles. This model was fit in R-INLA using stochastic partial differential equations (SPDE) to model the spatiotemporal processes.

Model validation was performed using spatially stratified five-fold out-of-sample cross validation, with examination of mean bias, mean absolute error, total error variance (root-mean-square error, RMSE), 95% data coverage within prediction intervals, and correlations of observed to predicted values. Geostatistical methods were not practical for estimating the prevalence of LF infection for the following locations due to small area (<25 km^2), missing covariate data, or limited geo-referenced data: American Samoa, Brazil, Cook Islands, Fiji, French Polynesia, Guyana, Kiribati, Maldives, Marshall Islands, New Caledonia, Niue, Palau, Samoa, Tonga, Tuvalu, Vanuatu, and Wallis and Futuna. Instead, Bayesian time series models for endemic IUs were fit to estimate annual national prevalence (Cromwell et al. (2020), Appendix 2). We masked all final model outputs for which land cover was classified as “barren or sparsely vegetated” on the basis of 2013 MODIS satellite data (the most recent year available), as well as areas in which total population density was less than ten individuals per 5 × 5-km grid cell in 2015.

To estimate of the number of infected individuals from the 5 × 5-km model predictions, the total number of cases per country was calculated first by multiplying grid-cell-level prevalence by the grid-cell-level population estimate produced by WorldPop, then aggregating those case estimates to national boundaries by draw. The mean total cases infected was calculated across the 1000 draws of case totals and the UI was constructed from the 2·5th and 97·5th percentiles. WHO regional totals were produced by aggregating up to regional boundaries, also by draw. Mean case estimates from the non-MBG locations were produced by applying the model-predicted national prevalence (mean, 2·5th and 97·5th percentile values) to the national population estimates produced for the Global Burden of Disease study or other sources for the relevant IU populations.

Lymphoedema and hydrocele modelling

For lymphoedema and hydrocele, we reviewed published studies on the prevalence of hydrocele or lymphoedema, as well as programme monitoring data for which LF infection and hydrocele or lymphoedema prevalence were reported in the same study population. We first adjusted data on lymphoedema reported in both males and females to be sex-specific. We do not model the prevalence of hydrocele in females. We then adjusted any all-age lymphoedema and hydrocele data to be age-specific according to five-year age groups using age patterns modelled from age-specific data in DisMod-MR 2.1. Two separate disability models were implemented, one for lymphoedema and one for hydrocele – the process essentially the same. The age patterns can be found below in Figure 1. The community-level

prevalence reported in studies for which hydrocele or lymphoedema were also reported was used as a covariate (adjusted to represent ICT prevalence) to predict prevalence of hydrocele and lymphoedema. The age-specific national estimates of ICT prevalence estimated by the geospatial model were then used to predict national hydrocele and lymphoedema prevalence. Overall prevalence of LF infection was predicted accounting for the impact of MDA on prevalence – we further restricted countries at least five years post-elimination from the estimates.

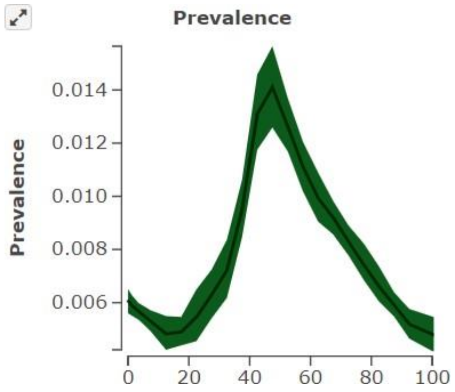

Figure 1a. Global age-pattern for lymphoedema due to LF used to split all-age data into age-specific datapoints for further modelling

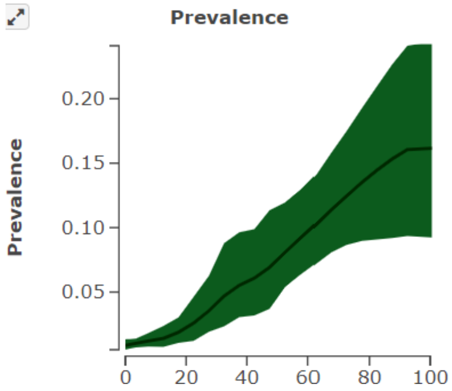

Figure 1b. Global age-pattern for hydrocele due to LF used to split all-age data into age-specific datapoints for further modelling

ADL prevalence estimates

After prevalence of lymphoedema and hydrocele were estimated, we assumed the following for prevalent lymphoedema cases: 95% experience a total of four episodes per year, with an average duration of 7 days. For prevalent hydrocele, we assume 70% of cases experience a total of two episodes per year, with an average duration of 7 days.

Table 2. Severity distribution, details on the severity levels for lymphatic filariasis and the associated disability weight (DW) with that severity

Sequela	Lay description	DW (95% CI)
Lymphoedema	Has swollen legs with hard and thick skin, which causes difficulty in moving around	0.109 (0.073–0.154)
Hydrocele	Has swelling and tenderness in the testicles and pain during urination	0.128 (0.086–0.18)
Acute adenolymphangitis due to lymphatic filariasis	Has a fever and aches and feels weak, which causes some difficulty with daily activities	0.051 (0.032–0.074)

Changes from GBD 2019 to GBD 2021

There were no substantive changes implemented in GBD 2021. We did not apply any adjustments for the COVID pandemic to lymphatic filariasis due to a lack of available data quantifying the impacts of the pandemic on NTD epidemiology.

References

1. Cromwell EA, Schmidt CA, Kwong KT, *et al.* The global distribution of lymphatic filariasis, 2000–18: a geospatial analysis. *The Lancet Global Health* 2020; 8: e1186–94.

Major depressive disorder

Flowchart

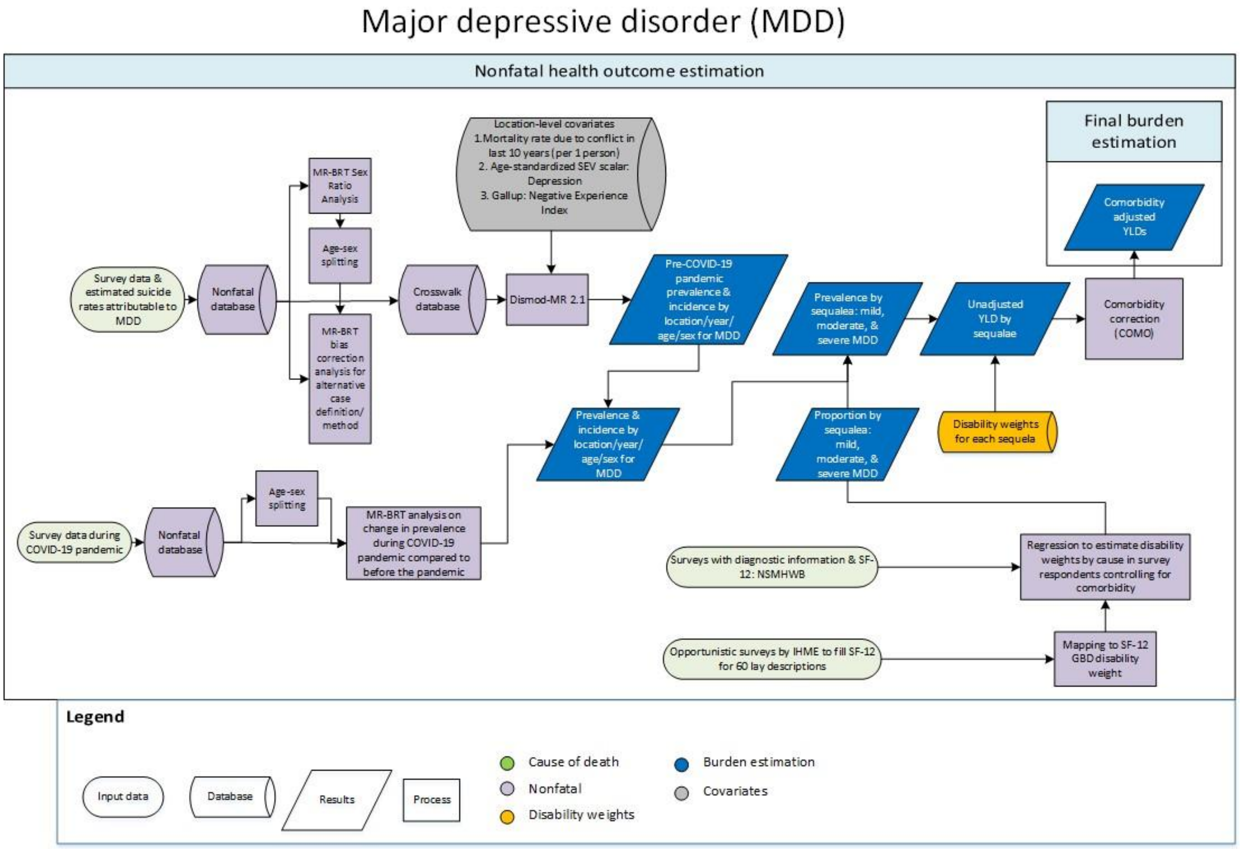

Input data and methodological summary for major depressive disorder

Case definition

Major depressive disorder (MDD) is an episodic mood disorder involving the experience of one or more major depressive episode(s). Included in the GBD disease modelling were cases meeting diagnostic criteria for MDD according to the Diagnostic and Statistical Manual of Mental Disorders (DSM) or the equivalent diagnosis of recurrent depression in the International Classification of Diseases (ICD).[1,2] These were identified by the following codes: DSM-IV-TR: 296.21–24, 296.31–34; ICD-10: F32.0–9, F33.0–9; excluding those cases due to a general medical condition or substance-induced cases.[1,2] Different versions of DSM (DSM-III, DSM-III-R, DSM-IV, DSM-IV-TR, DSM-5, and DSM-5-TR) and ICD (ICD-9, ICD-10, and ICD-11) were accepted.

According to DSM-IV-TR criteria, MDD involves the presence of at least one major depressive episode, which is the experience of either depressed mood or loss of interest/pleasure, for most of every day, for at least two weeks. This must represent a change from the person’s baseline and impaired functioning observed across social, occupational, and educational domains.

In addition to one of the two symptoms above, four out of the following seven criteria must also be met to make a diagnosis:

- change in eating, appetite, or weight

- excessive sleeping or insomnia
- agitated or slow motor activity
- fatigue
- feeling worthless or inappropriately guilty
- trouble concentrating
- repeated thoughts about death

MDD was modelled as an episodic disorder with the average length of a major depressive episode (ie, duration) specified. This method has been discussed in greater detail in previous publications.[3,4]

Input data

The epidemiological systematic literature review for MDD was conducted in three stages involving electronic searches of the peer-reviewed literature (ie, via PsycInfo, Embase, and PubMed), the grey literature, and expert consultation. For mental disorders, we update our GBD electronic database searches on a rolling basis. An electronic search was not required for GBD 2021. The next update will be conducted in the next round of GBD. The grey literature searches and expert consultation were conducted for GBD 2021.

The GBD inclusion criteria stipulated that: 1) the publication year must be from 1980 onward; 2) "caseness" must be based on clinical threshold as established by the DSM or ICD; 3) sufficient information must be provided on study method and sample characteristics to assess the quality of the study; and 4) study samples must be representative of the general population (ie, inpatient or pharmacological treatment samples, case studies, veterans, or refugee samples were excluded). No limitation was set on the language of publication. Methods used for this systematic review have been reported in greater detail elsewhere.[3] Table 1 summarises data inputs by parameter for MDD.

Table 1: Data inputs for MDD morbidity modelling by parameter

**Parameter**	**Countries with data**	**New sources**	**Total sources**
Incidence	3	1	3
Prevalence	112	82	573
Remission	0	0	0
Other	14	1	25

Age-sex splitting

The extracted data underwent three types of age-sex splitting processes:

16. Where possible, estimates were further split by sex and age based on the available data. For instance, if studies reported prevalence for broad age groups by sex (eg, prevalence in 15–65-year-old males and females separately), and also by specific age groups but for both sexes combined (eg, prevalence in 15–30-year-olds, then in 31–65-year-olds, for males and females combined); age-specific estimates were split by sex using the reported sex-ratio and bounds of uncertainty.

17. A meta-regression—Bayesian, regularised, trimmed (MR-BRT) analysis was used to split the remaining both-sex estimates in the dataset. For each parameter, sex-specific estimates were matched by location, mid-age, and year. A MR-BRT network meta-analysis was then used to estimate pooled sex ratios. Given evidence to suggest that the sex-ratio in depression varies with age,[5-7] we also tested for an age interaction in the model. We found that the sex difference in MDD decreased significantly with age, ie, prevalence in males (compared to females) increased significantly with increasing age. The global sex-ratio (at the mean mid-age of data informing the sex-ratio model) was estimated as 0.54 (95% uncertainty interval [UI]: 0.36–0.80) while Figure 1 shows the estimated male-to-female prevalence ratio by age. Age-specific sex ratios were used to split both-sex estimates in the dataset.

Figure 1. Sex ratios by age for MDD

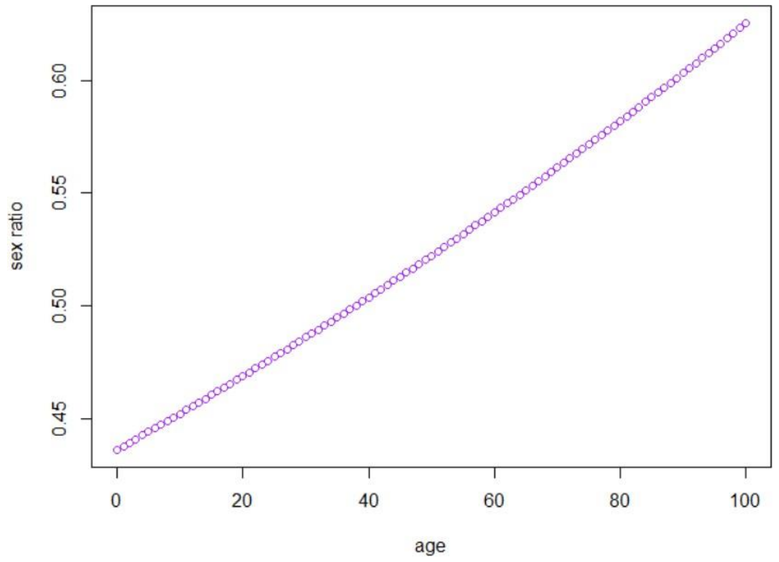

18. Studies reporting prevalence estimates across age groups spanning 25 years or more were split into five-year age groups using the prevalence age pattern estimated by DisMod-MR 2.1. The DisMod-MR model used to estimate the age pattern did not contain any previously age-split data.

Bias corrections/crosswalks

Estimates with known biases were adjusted/crosswalked accordingly prior to DisMod-MR 2.1. For each crosswalk of interest, pairs of the reference and the alternative estimates were matched by age, sex, location, and year. This was done for both within-study (where possible) and between-study pairs. These pairs were then used as inputs in a MR-BRT network meta-analysis. The MR-BRT analysis produced a pooled ratio between the reference estimates and alternative estimates, which was used to adjust all alternative estimates in the dataset. Reference data informing the prevalence of MDD consisted of estimates reporting past-month/point prevalence of MDD using a diagnostic tool that was administered by a clinician. Four adjustment ratios were used for alternative data:

1. A past-year recall ratio adjusted all datapoints derived from past-year prevalence toward the level they would have been if the study had captured point/past-month prevalence. The latter prevalence period is less affected by recall bias.
2. A symptom scale ratio adjusted all datapoints derived using a symptom scale toward the level they would have been if the scale had strictly adhered to DSM or ICD thresholds for MDD.
3. A World Health Survey ratio adjusted all World Health Survey data downwards towards the level they would have been had the study strictly adhered to DSM or ICD thresholds for MDD. The World Health Surveys are surveys conducted by the World Health Organization in close to 70 countries. While these surveys capture useful information on the prevalence of depression, they make use of a symptom scale which does not fully meet DSM and ICD criteria for MDD. This adjustment works essentially in the same way as the previous symptom scale adjustment.
4. A lay-interviewer ratio was used to adjust all prevalence estimates derived from trained lay-interviewers towards the level they would have been if the estimate was derived from clinically trained interviewers (eg, psychologist or psychiatrist). We consider interviews conducted by clinicians to be more sensitive to detecting cases of MDD, particularly in locations where western-based mental health case definitions and instruments are yet to be fully validated.

See Table 2 for adjustment factors used for MDD. The estimated UIs around the adjustment ratio incorporate Gamma, which represents the between-study variance across all input data in the model. This added uncertainty widens the UIs for crosswalks with significant fixed effects.

Table 2: MR-BRT crosswalk adjustment factors for MDD

Data input	Reference or alternative case definition	Gamma	Beta coefficient, log (95% UI)*	Adjustment factor**
Population survey	Reference: past-month/point prevalence, from a diagnostic tool, administered by a clinician	0.43		
Population survey	Alternative: past-year prevalence		0.72 (–0.17 to 1.55)	2.06 (0.84–4.73)
Population survey	Alternative: symptom scale		1.05 (0.16 to 1.88)	2.85 (1.17–6.55)
Population survey	Alternative: World Health Survey data		0.70 (–0.18 to 1.54)	2.00 (0.83–4.69)
Population survey	Alternative: lay-interviewer diagnosis		–0.18 (–1.08 to 0.64)	0.83 (0.34–1.91)

**MR-BRT crosswalk adjustments can be interpreted as the factor the alternative case definition is adjusted by to reflect what it would have been had it been measured using the reference case definition. If the log/logit beta coefficient is negative, then the alternative is adjusted up to the reference. If the log/logit beta coefficient is positive, then the alternative is adjusted down to the reference.*

***The adjustment factor column is the exponentiated beta coefficient. For log beta coefficients, this is the relative rate between the two case definitions. For logit beta coefficients, this is the relative odds between the two case definitions.*

Attributable suicide estimates

Given that MDD is an established risk factor for suicide,[8] we supplemented the available data on excess mortality with estimated suicide rates (by age, sex, year, and location) attributable to MDD. These were

estimated using GBD's comparative risk assessment methodology whereby the current health status was compared with a theoretical minimum risk exposure defined as the counterfactual status of the absence of MDD in the population. Population attributable fractions (PAFs) were estimated using this established formula:

$$PAF = \frac{p\,(RR - 1)}{p\,(RR - 1) + 1}$$

P referred to the exposure distribution, which in this case was the DisMod-MR 2.1 prevalence rates of MDD by age, sex, location, and year. Relative risk referred to the pooled relative risk of suicide due to MDD obtained from an existing systematic review and meta-analysis.[8] These are also summarised in Figure 2. Age, sex, year, and location-specific PAFs were multiplied by their corresponding GBD suicide rate to estimate the proportion of suicide cases attributable to MDD. These were entered as cause-specific mortality rates in our epidemiological model for MDD.

Figure 2. Forest plot showing relative risk of suicide due to MDD

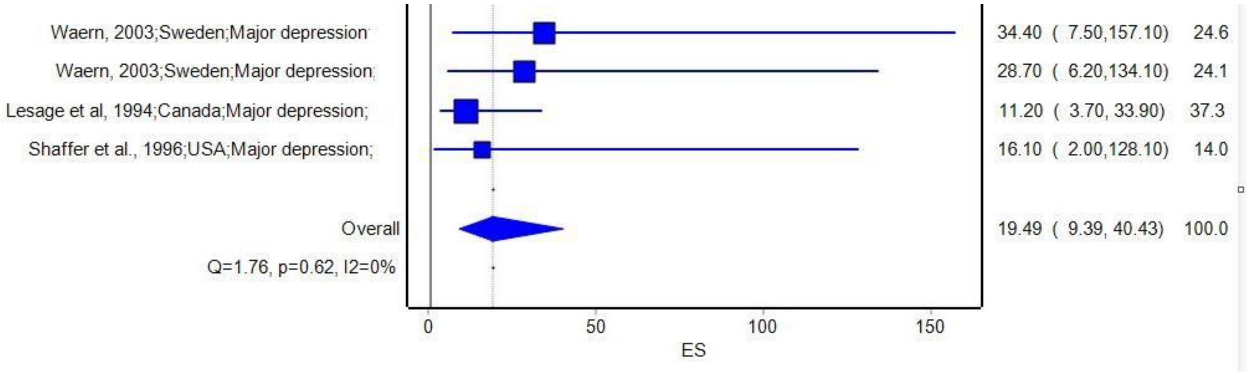

Impact of the COVID-19 pandemic

The emergence of the COVID-19 pandemic in 2020 has raised many questions around the resulting impacts on mental health. In GBD 2021, we sought to quantify the impact of COVID-19 on the prevalence and burden of MDD and anxiety disorders for the years 2020 and 2021.

We first conducted a systematic literature review to identify studies reporting on MDD or anxiety disorder prevalence during the COVID-19 pandemic published between 1 January 2020 and 29 January 2021. The search was conducted in three stages involving electronic searches of the peer-reviewed literature (ie, via PubMed), the grey literature (ie, via COVID-19: living map of the evidence by Eppi-centre, The DEPRESSD Project, WHO-COVID-19, COVID-minds, MedRxiv, and PsyArXiv), and expert consultation. The following search terms were used to develop search strings across all databases searched: 'mental health', 'mental disorders', 'anxiety disorder', 'depressive disorder', 'anxiety', 'depress*', 'psycholog' and 'novel coronavirus', 'covid', 'covid-19', 'nCoV', '2019nCoV', 'coronavirus', 'coronavi*', 'SARS-COV-2' 'SARSCoV2', 'outbreak', 'epidemic', 'pandemic', and 'prevalence', 'impact', 'outcome', 'effect', 'percentage'.[9]

We conducted an update to the systematic literature review in two stages. First, in July 2022, we conducted a review of reviews by searching for systematic reviews in PubMed published since 1 January 2021. Next, in August 2022, we conducted electronic searches of the peer-reviewed literature (ie, via

PubMed), the grey literature (ie, via COVID-19: living map of the evidence by Eppi-centre, WHO-COVID-19, and COVID-minds), and expert consultation. Studies reporting data during 2021 and 2022 were prioritised in this update.

The inclusion criteria used closely mirrored the criteria used more broadly within the GBD to ensure consistency in measurement. Studies had to report the prevalence of MDD during the COVID-19 pandemic and have a pre-pandemic baseline. Longitudinal studies using samples that were representative of the general population were preferred, but cross-sectional studies conducted during the COVID-19 pandemic were also accepted if comparable pre-COVID prevalence data could be identified. Studies reporting on probable depressive disorders using established screening measures (eg, the Patient Health Questionnaire-9) were also included due to lack of available data using reference case definitions for MDD. Additionally, studies using screening measures of psychological distress or both depression and anxiety together (eg, the Kessler-6) were included and were to be controlled for in analyses.

The first search generated 5683 records, and the update generated 5569 records (after duplicates were removed). The title/abstract screening across both searches reduced the number of relevant records to 2544 studies, of which 64 met criteria for inclusion for MDD.

Modelling strategy

The modelling strategy used in GBD 2021 was the same as GBD 2019, with the addition of COVID-19 adjustment. The COVID-19 adjustment was applied to the modelled prevalence estimates for 2020 and 2021, after the standard epidemiological modelling analysis to estimate prevalence by age, sex, location, and year had been undertaken.

DisMod-MR 2.1 was used to model the (pre-COVID-19) epidemiological data for MDD. Adjustments to model priors or the dataset were made where appropriate. Where outliers were identified in the data, we reassessed the study's methodology and quality before a decision was made to exclude or include the data.

Data across all epidemiological parameters were initially included in the modelling process. However, given that the few incidence datapoints available typically excluded cases of MDD at baseline, new major depressive episodes in people with previous episodes were not counted and incidence was underestimated. For this reason, we chose to exclude all raw incidence data in the final model and instead allowed DisMod-MR to calculate incidence based on data from other parameters. We assumed no incidence and prevalence before age 3. This minimum age of onset was corroborated with expert feedback and existing MDD literature.[3] An average remission rate for a major depressive episode of 1.45 (1.3–1.6) was used. This was derived from the five longitudinal studies[10-14] fitting a lognormal curve with least squared differences to data on the proportion of incident cases still fulfilling the case definition for MDD at intervals over a one-year period (See Figure 3). As data were only available for a follow-up of one year, a decision had to be made about the maximum allowable duration of an episode. Setting this at 40 years, the average duration implied by the lognormal fit was 0.65 (0.59–0.70) of a year.[15]

Figure 3. Time to recovery in episodes of MDD from five studies

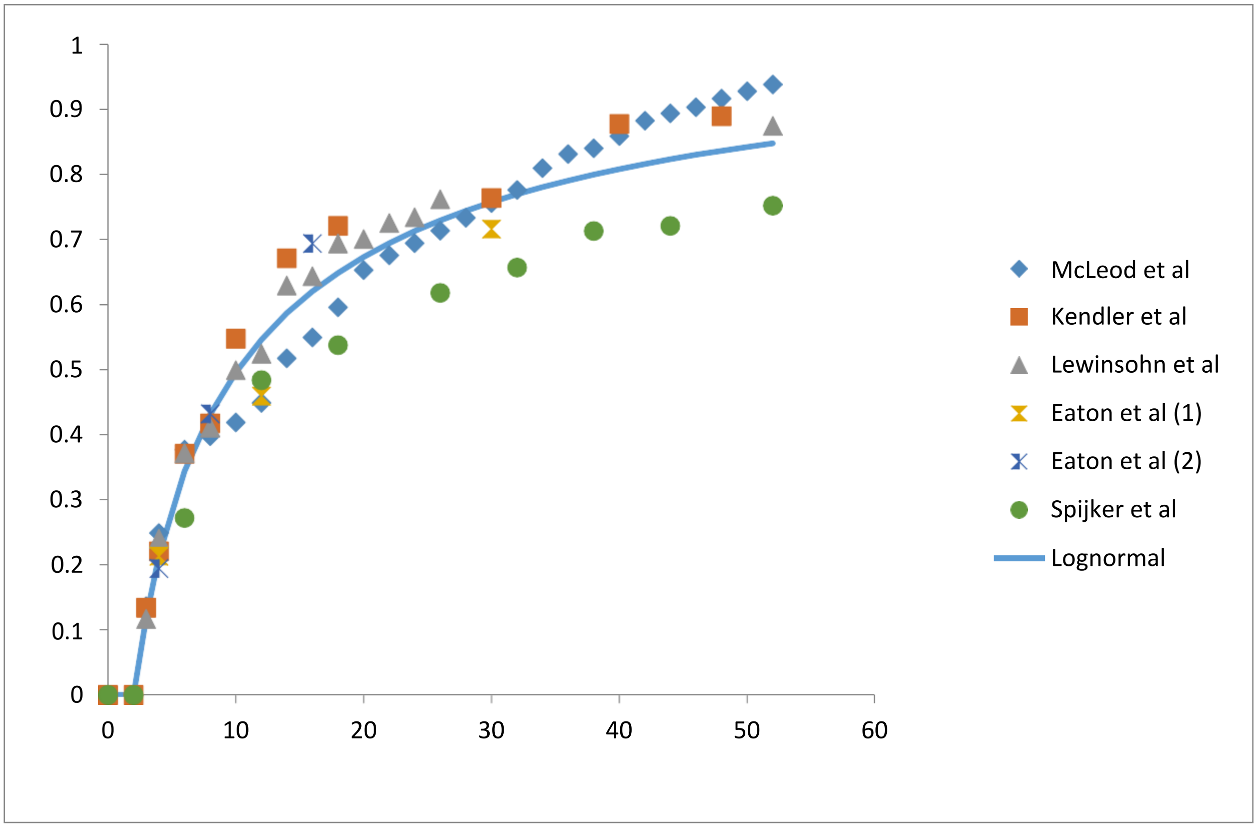

The following location-level covariates were used to inform the estimation of prevalence:

1. The mean war mortality rate in the previous ten years: This covariate identified, for each GBD location, the mean mortality rate in the previous ten years due to war and terrorism. It was used given the existing evidence to show a positive association between conflict status and the prevalence of MDD.[16,17]
2. An age-standardised summary exposure value (SEV) scalar: This made use of the fraction of MDD burden caused by childhood sexual abuse and intimate partner violence (two risk factors of MDD included in the GBD study) to inform the estimation of prevalence.
3. A Gallup negative experience index: The Gallup initiative conducts comprehensive and comparable national surveys across a wide range of countries worldwide.[18] This index measured respondents' past-day experiences of physical pain, worry, sadness, stress, and anger. The Gallup covariate was included as a means to test for a correlation between negative emotions at a location level and MDD prevalence. Data from the Gallup negative experience index was modelled using the spatiotemporal Gaussian process regression (ST-GPR) to produce estimates for all years and locations required by DisMod-MR. The log of the modelled output was used as the covariate in DisMod-MR due to skewedness of the data. The relationship detected was in the expected direction (ie, the higher the negative emotion, the higher the prevalence rate), although the association with MDD prevalence was marginally positive.

A summary of covariates and exponentiated values for MDD is shown in Table 3.

Table 3. Summary of covariates used in the MDD DisMod-MR meta-regression model

Covariate	Type	Parameter	Beta (95% UI)	Exponentiated beta (95% UI)
Mean war mortality rate in the previous 10 years	Location-level	Prevalence	0.50 (0.07–0.94)	1.64 (1.07–2.56)
Log-transformed age-standardised SEV scalar: depression	Location-level	Prevalence	1.19 (1.11–1.25)	3.29 (3.03–3.48)
Gallup: negative experience index	Location-level	Prevalence	0.01 (0.00–0.05)	1.01 (1.00–1.05)

Impact of the COVID-19 pandemic

Prevalence data from the COVID-19 systematic review were first analysed separately to the above DisMod-MR 2.1 analysis in order to investigate the change in prevalence of MDD during the COVID-19 pandemic.[9] The logit difference between pre-pandemic prevalence and prevalence during the pandemic was calculated for all eligible input data. A model to estimate the adjustment to prevalence was developed via a two-step process. In step one, an indicator model was run to develop an index for the impact of COVID-19. We conducted a meta-regression via MR-BRT to predict the logit difference in prevalence from changes in human mobility (as captured by anonymous cell phone mobility data) and the IHME daily COVID-19 mortality rate, controlling for studies that compared mid-pandemic prevalence from a market research and quota sampling methodology against a prevalence from a random sample. We used the coefficients for these two indicators to calculate a single COVID-19 impact indicator for MDD. In step two, we developed a final model via backward elimination to regress the COVID-19 impact indicator, and interactions between this indicator and age and sex. Bias covariates were also treated as interactions against the indicator except for studies that compared mid-pandemic prevalence from a market research and quota sampling methodology against a prevalence from a random sample, which were controlled for via a binary covariate on the change in logit prevalence. The least significant covariate was iteratively removed until no improvement was seen in the Akaike information criterion (See Table 4). This model was then used to predict the logit change in prevalence for every day of the years 2020 and 2021 by age, sex, and location. The 2020 and 2021 age-specific, sex-specific, and location-specific MDD prevalence estimated by DisMod-MR 2.1 (informed by prevalence data prior to 2020) was then adjusted by the predicted logit change from the MR-BRT model for every day of the years 2020 and 2021. Annual point prevalence estimates for 2020 and 2021 were then calculated as the average daily prevalence for the year.

Table 4. Meta-regression coefficients on the change in MDD logit prevalence over the course of the COVID-19 pandemic

Covariate	Coefficient	Uncertainty interval	*p*
COVID-19 impact indicator	0.865	0.637 to 1.093	<0.001
Human mobility*	–0.200	–0.439 to –0.057	-
COVID-19 mortality rate*†	–69.600	–101.513 to –43.169	-
Mean or midpoint age	–0.026	–0.029 to –0.024	<0.001
Proportion female	0.082	–0.014 to 0.179	0.095
Combined depressive and	0.296	0.027 to 0.565	0.031

anxiety disorder symptoms			
Market research and quota sampling vs. market research and quota sampling	–2.118	–3.227 to –1.009	<0.001
Market research and quota sampling vs. random sampling	–0.976	–1.298 to –0.654	<0.001

* Coefficients were estimated using the coefficient of the COVID-19 impact index multiplied by the coefficient of the COVID-19 impact indicators from the indicator model. †Square-root-transformed before analysis to correct for positive skew.

Severity splits and disability weights

The GBD disability weight survey assessments include lay descriptions of sequelae highlighting major functional consequences and symptoms. The lay descriptions and disability weights for MDD severity levels are shown in Table 5. To determine the proportion of people with MDD within each of the severity levels, estimates from the Australian National Survey of Mental Health and Wellbeing of Adults (NSMHWB, conducted in 1997)[19] were used. The proportion of MDD cases falling within each severity level were as follows: asymptomatic 13% (10–17), mild 59% (49–69), moderate 17% (13–22), and severe 10% (3–20). The same severity distribution and disability weights were applied to the pre-COVID-19 and post-COVID-19 prevalent cases of MDD.

Table 5. Severity distribution, details on the severity levels for MDD, and the associated disability weight with that severity

Severity level	Lay description	Disability weight (95% UI)
Mild	Feels persistent sadness and has lost interest in usual activities. The person sometimes sleeps badly, feels tired, or has trouble concentrating but still manages to function in daily life with extra effort.	0.145 (0.099–0.209)
Moderate	Has constant sadness and has lost interest in usual activities. The person has some difficulty in daily life, sleeps badly, has trouble concentrating, and sometimes thinks about harming himself (or herself).	0.396 (0.267–0.531)
Severe	Has overwhelming, constant sadness and cannot function in daily life. The person sometimes loses touch with reality and wants to harm or kill himself (or herself).	0.658 (0.477–0.807)

The addition of the COVID-19 adjustment has meant that the prevalence of MDD increased in GBD 2021 compared to GBD 2019. The pandemic has created an environment where many determinants of mental health are also impacted. Social restrictions, lockdowns, school and business closures, loss of livelihood, and decreases in economic growth all have the potential to significantly impact on mental health. In GBD 2021, we responded to this by incorporating a method to estimate the impact of COVID-19 on the prevalence and burden of MDD. That said, several limitations to this work need to be acknowledged. Data coverage was limited to high-income countries, with location-specific predictions relying on two COVID-19 indicators in the model – human mobility and IHME-estimated daily COVID-19 mortality. Our analysis relied on data from symptom scales capturing probable cases of MDD as very few diagnostic mental health surveys have been conducted during the COVID-19 pandemic. Our estimation of the

impact of COVID-19 on mental disorders is still underway, with further improvements to be made as new epidemiological studies are published, and as we progress through various stages of the pandemic.

More broadly, across our entire epidemiological modelling process, we still have a large number of locations with no high-quality raw data available. It is also difficult to quantify and remove all variation due to measurement error in our epidemiological estimates. While we have improved the methodology used to account for known sources of bias, in some cases, we still have very few datapoints to inform these adjustments. Finally, there is a paucity of research on the risk factors of mental disorders which can be used as predictive covariates in our epidemiological models.

References

1. American Psychiatric Association. *Diagnostic and Statistical Manual of Mental Disorders (DSM)*. Washington: American Psychiatric Association, 1952.

2. World Health Organization. *The ICD-10 Classification of Mental and Behavioural Disorders. Clinical descriptions and diagnostic guidelines*. Geneva: World Health Organization; 1992.

3. Ferrari AJ, Charlson FJ, Norman RE, et al. Burden of depressive disorders by country, sex, age, and year: findings from the global burden of disease study 2010. *PLoS Med* 2013; **10**(11): e1001547.

4. Ferrari AJ, Charlson FJ, Norman RE, et al. The epidemiological modelling of major depressive disorder: application for the Global Burden of Disease Study 2010. *PloS One* 2013; **8**(7).

5. Salk RH, Hyde JS, Abramson LY. Gender differences in depression in representative national samples: meta-analyses of diagnoses and symptoms. *Psychological Bulletin* 2017; **143**(8): 783.

6. Patten SB, Williams JV, Lavorato DH, Wang JL, Bulloch AG, Sajobi T. The association between major depression prevalence and sex becomes weaker with age. *Social psychiatry and psychiatric epidemiology* 2016; **51**(2): 203-10.

7. Patten SB, Wang JL, Williams JV, et al. Descriptive epidemiology of major depression in Canada. *The Canadian Journal of Psychiatry* 2006; **51**(2): 84-90.

8. Ferrari AJ, Norman RE, Freedman G, et al. The burden attributable to mental and substance use disorders as risk factors for suicide: findings from the Global Burden of Disease Study 2010. *PLoS One* 2014; **9**(4): e91936.

9. Santomauro DF, Whiteford HA, Ferrari AJ. Depression and anxiety during COVID-19 - Authors' reply. *Lancet* 2022; **399**(10324): 518-9.

10. Eaton WW, Anthony JC, Gallo J, et al. Natural history of Diagnostic Interview Schedule/DSM-IV major depression. The Baltimore Epidemiologic Catchment Area follow-up. *Arch Gen Psychiatry* 1997; **54**(11): 993-9.

11. Kendler KS, Walters EE, Kessler RC. The prediction of length of major depressive episodes: results from an epidemiological sample of female twins. *Psychol Med* 1997; **27**(1): 107-17.

12. Lewinsohn PM, Clarke GN, Seeley JR, Rohde P. Major depression in community adolescents: age at onset, episode duration, and time to recurrence. *J Am Acad Child Adolesc Psychiatry* 1994; **33**(6): 809-18.

13. McLeod JD, Kessler RC, Landis KR. Speed of recovery from major depressive episodes in a community sample of married men and women. *J Abnorm Psychol* 1992; **101**(2): 277-86.

14. Spijker J, de Graaf R, Bijl RV, Beekman AT, Ormel J, Nolen WA. Duration of major depressive episodes in the general population: results from The Netherlands Mental Health Survey and Incidence Study (NEMESIS). *Br J Psychiatry* 2002; **181**: 208-13.

15. Vos T, Haby MM, Barendregt JJ, Kruijshaar M, Corry J, Andrews G. The burden of major depression avoidable by longer-term treatment strategies. *Archives of General Psychiatry* 2004; **61**(11): 1097-103.

16. Karam E, Bou GM. Psychosocial consequences of war among civilian populations. *Current Opinion in Psychiatry* 2013; **16**(413–419).

17. Steel Z, Chey T, Silove D, Marnane C, Bryant RA, van Ommeren M. Association of torture and other potentially traumatic events with mental health outcomes among populations exposed to mass conflict and displacement: a systematic review and meta-analysis. *Jama* 2009; **302**(5): 537-49.

18. Gallup G. The Gallup Poll: Public Opinion 2003: Rowman & Littlefield; 2004.

19. Australian Bureau of Statistics. National Survey of Mental Health and Wellbeing of Adults 1997. Canberra: Australian Bureau of Statistics.

Malaria

Flowchart

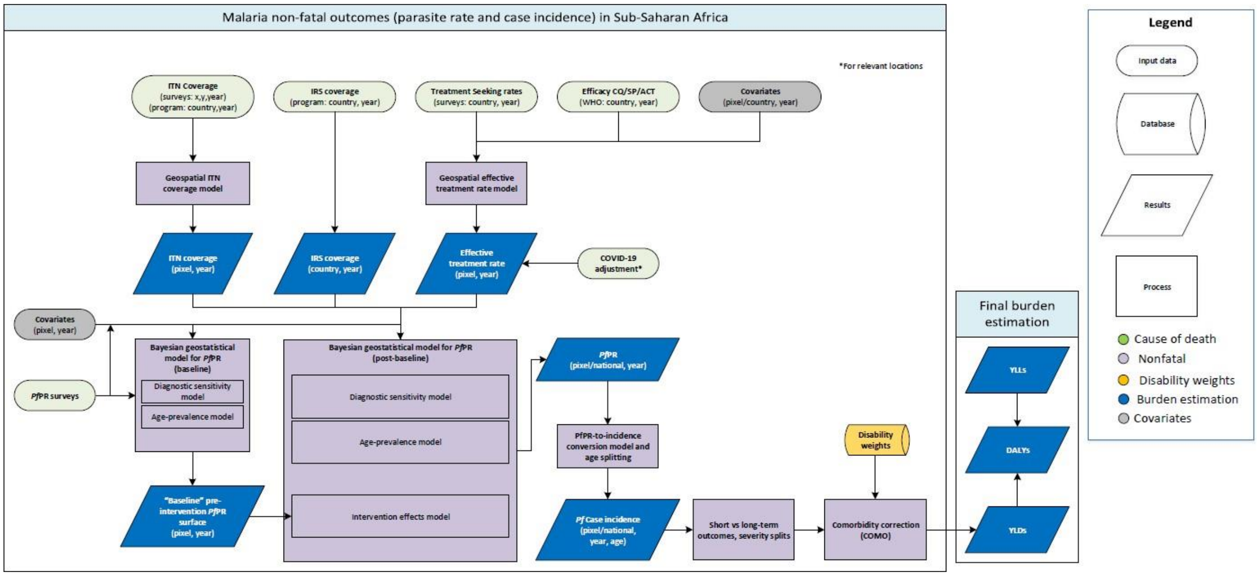

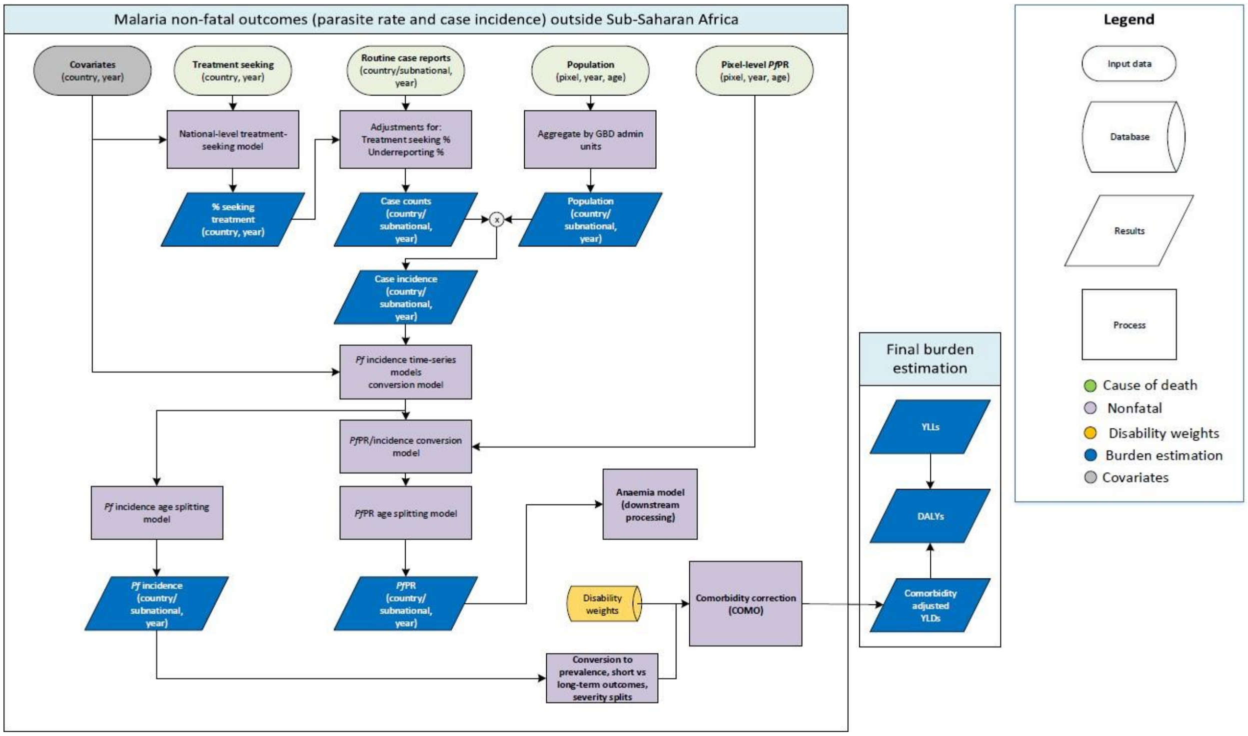

Input data and methodological summary for malaria

Case definition

Malaria is a mosquito-borne infectious disease caused by *Plasmodium* parasites. Symptoms include fever, chills, headaches, myalgias, nausea, anemia, and vomiting. In severe malaria, symptoms can progress to cerebral malaria (impaired consciousness, seizures, and residual neurologic deficits), severe anemia, liver dysfunction, coagulopathy, renal failure, respiratory distress, hypoglycemia, multi-organ

dysfunction, and death.. Microscopy is considered the gold-standard diagnostic approach for the purposes of GBD. The relevant ICD-10 codes are B50-B54.

Malaria

Quantity of interest	Reference or Alternative	Definition
Malaria	Reference	Prevalence of people with detectable *P. falciparum* or *P. vivax* parasites through microscopy and/or rapid diagnostic tests and clinical symptoms for malaria (any of fever, diarrhoea, vomiting). This definition includes ICD-10 codes B50-B54.
Malaria	Alternative	Prevalence of people in malaria-endemic locations with clinical symptoms (any of fever, diarrhoea, vomiting) for whom diagnostic testing was either inconclusive or unavailable.
Disability due to cerebral malaria in children under 5	Reference	Proportion of children under 5 with cerebral malaria who go on to have long-term disability (motor impairment, intellectual disability, seizures, and blindness).

Input data

Primary data inputs were:

(i) Routine malaria case reports from national routine surveillance systems. These were obtained at the national level from the WHO World Malaria Report and at the subnational administrative level, wherever possible, via an exhaustive search of published and grey literature sources along with online data portals hosted by national ministries of health. Each retained record consisted of an annual count of malaria cases along with a distinction between confirmed and unconfirmed diagnoses, and differentiation by malaria parasite species.

(ii) Cross-sectional, geolocated, and community-representative observations of infection prevalence for *Plasmodium falciparum* (referred to hereafter as *P. falciparum* parasite rate, *Pf*PR).

These malaria epidemiological metrics were augmented in the modelling by:

(iii) Malaria Atlas Project (MAP) modelled estimates of malaria control intervention population coverage (ITNs, IRS, and effective treatment with an antimalarial drug) resolved to 5 km x 5 km pixel-year level (for sub-Saharan Africa) and country-year level (outside sub-Saharan Africa).

(iv) A large suite of environmental, sociodemographic, and economic covariates resolved to 5 km x 5 km pixel-year level (for sub-Saharan Africa) and country-year level (outside sub-Saharan Africa).

Table 1: Data Inputs for malaria morbidity modelling by parameter

Measure	Countries with data	New sources	Total sources
Incidence	104	1989	4948
Prevalence	85	86	1702
Other	121	414	7280

Modelling strategy

The suitability, availability, and quality of *Pf*PR and routine case reporting data, as well as detailed intervention coverage information, differ markedly inside versus outside sub-Saharan Africa. As such, we developed separate modelling strategies for countries inside sub-Saharan Africa versus those outside. The exceptions were Botswana, Cabo Verde, Comoros, Djibouti, Eritrea, Ethiopia, Mauritania, Mauritius, Namibia, Sao Tome and Principe, Senegal, South Africa, and Swaziland. Despite being part of Africa, these countries exhibit epidemiological trends and have data availability/quality more akin to non-African settings.

PfPR and case incidence modelling: Africa
Modelling was conducted in the following steps:

(i) The large assembly of geolocated *Pf*PR surveys maintained by MAP was used in a Bayesian spatiotemporal geostatistical model to predict *Pf*PR for every pixel-year in sub-Saharan Africa, representing an update to earlier work (Bhatt et al Nature, Gething et al NEJM). The model considered (i) *Pf*PR survey participant age ranges and diagnostic type; (ii) coverage of ITNs, IRS, and effective antimalarial drug coverage, and how these metrics changed through time at each date and prediction location; (iii) environmental conditions at each date and prediction location (including density of vegetation, temperature, humidity, rainfall, elevation, and proximity to populated areas). The outcome was a predicted space-time "cube" of *Pf*PR, standardised to the 2–10 age range, for each year 1980–2022.

(ii) The *Pf*PR cube was then converted into an equivalent cube of the predicted incidence rate of clinical malaria. This conversion was achieved using an established model (Cameron et al Nature Communications) taking seasonality, treatment level, and recent prevalence trends at the location level into account and provided estimates stratified first into three broad age bins (0–5; 5–15; <15) and then into the final 23 GBD 2021 age bins.

*Pf*PR and case incidence modelling: outside Africa
Malaria-endemic countries outside Africa tend to have less *Pf*PR data than those inside, in part because prevalence is generally lower. Furthermore, *Pf*PR surveys are rare in areas of lower prevalence and thus this metric becomes an inefficient way to measure malaria risk. In contrast, routine surveillance systems outside Africa are generally stronger, meaning that reports of malaria cases from health systems are more reliable and provide some insight into the total malaria burden in the community. Modelling outside Africa was carried out in the following steps:

(i) National and subnational case reports were first subject to adjustments to identify and minimise bias. Bias in reported case numbers arises from various sources. First, a fraction of cases in the community will fail to seek treatment or will attend a private or informal health care provider that will not provide a record of that case to the routine surveillance system. We adjusted for these factors by modelling the fraction of cases seeking care from different provider categories based on data from nationally representative cross-sectional household surveys (primarily from the Demographic and Health Survey (DHS) program and the Multiple Indicator Cluster Survey program) (Battle et al, 2016). Another factor for which we must adjust is cases reaching formal clinics that may not be subject to a confirmatory diagnostic test. We adjusted for this by assuming the fraction of unconfirmed cases that were truly

malaria would equal the fraction of positives among all those tested. A final factor we adjust for is incomplete data as many routine surveillance systems fail to capture all case reports, with facilities/regions missing from the national totals in a given year. We adjusted for this based on reporting completeness statistics published nationally by the WHO.

(ii) These adjusted routine case reports were georeferenced using digitised administrative boundary data using a spatial database of such boundaries collated and maintained by MAP.

(iii) Each case report was converted into an estimate of clinical incidence rate by dividing it by the estimated population in each unit, with the latter quantity derived by combining high-resolution gridded population data and the aforementioned administrative boundaries.

(iv) Bayesian time-series models were then applied to the case reports for each country to impute incidence rates for years with missing data. The results from this analysis, in conjunction with the adjusted case reports, constitute the incidence values delivered for GBD 2021.

(v) The incidence rate for each country-year was then converted to an inferred *Pf*PR value using the same model described earlier (Cameron et al). This allowed us to utilise these polygon-level surveillance data and the *Pf*PR point-level data (where present) within the same modelling framework.

(vi) The combined *Pf*PR survey point data and (pseudo) *Pf*PR administrative unit data were then used in a Bayesian spatiotemporal geostatistical model to predict *Pf*PR at pixel-year level across all countries. As for the Africa model, *Pf*PR was standardised by age and diagnostic type and informed by a wide suite of covariates. An additional mechanism was developed to allow polygon (ie, administrative unit) and point (ie, survey) data to be used jointly to infer the predicted space-time surfaces.

(vii) The predicted *Pf*PR cube was then adjusted to ensure that, after conversion to pixel-level incidence, the incidence counts per country-year would precisely match the incidence results from step (iv). The summarised *Pf*PR values (ie, population-weighted and tallied for each country-year) from the adjusted *Pf*PR cube constitute the *Pf*PR values delivered for GBD 2021.

Total malaria cases by country, year, sex

The pixel-level predictions of clinical incidence rate (both inside and outside of Africa) were combined with high-resolution gridded population data to estimate total cases per pixel-year. These were then aggregated to GBD national/subnational areas. Inside sub-Saharan Africa, for countries endemic for *P. vivax* and *P. falciparum*, we calculated the number of cases due to *P. vivax* by applying the fraction of *P. vivax* and *P. falciparum* obtained from WHO and a literature review (Battle et al., 2019; Weiss et al., 2019). Outside sub-Saharan Africa we followed the identical procedure for *P. vivax* and *P. falciparum.* Final age-splitting was accomplished using age-versus-incidence rate relationships gleaned from the paper by Cameron and colleagues (2015).

Determining YLDs for malaria

As in GBD 2019, we used a two-step process for determining malaria severity. For acute cases, severity splits for mild, moderate, and severe malaria were produced by analysis of MEPS data. These sequelae and their associated disability weights are presented below.

Table 3. Severity distribution, details on the severity levels for malaria and the associated disability weight (DW) with that severity

Severity level	Lay description	DW (95% CI)
Mild malaria	Has a low fever and mild discomfort but no difficulty with daily activities.	0.006 (0.002–0.012)
Moderate malaria	Has a fever and aches and feels weak, which causes some difficulty with daily activities.	0.051 (0.032–0.074)
Severe malaria	Has a high fever and pain and feels very weak, which causes great difficulty with daily activities.	0.133 (0.088–0.19)

To determine long-term neurological burden due to malaria, we use the work by Roca-Felter and colleagues (2008) that examined the number of uncomplicated cases that led to longer-term impairment. Analytically, this means multiplying incidence estimates (described in the section above for persons under 20 by 0.00029 (0.000077–0.00057). This adjusted case estimate is then combined with excess mortality rates derived from all-cause mortality and standardised mortality ratios for neonatal encephalopathy (NE) in a DisMod model to produce prevalence estimates of long-term sequelae for all estimation years. Implicit in this process is an assumption that the disability and trend of impairment due to severe malaria follow NE. The subsequent severity splitting follows NE as well.

To determine the burden of acute (short-term) malaria, the incidence estimation results are combined and converted to prevalence by matching each draw with a draw of duration of clinical illness. Consistent with GBD 2019, we use a uniform distribution between 14 and 28 days for duration.

Changes from GBD 2019 to GBD 2021

The methodology between GBD 2019 and GBD 2021 has stayed the same except for the inclusion of a COVID-19 adjustment.

A COVID-19 adjustment was applied to the years 2020 and 2021 utilizing PULSE surveys, from the WHO, conducted by country government officials on healthcare service disruption ("Pulse survey", 2020; "Second round", 2021; "Third round", 2021). This adjustment was applied to antimalarial effective treatment rates, which are used in the prevalence estimation process, and subsequently incidence. Please see Annex 1 of the 2022 World Malaria Report or the recent article by Dzianach et al., (2023) for further details on how the adjustments were derived. Currently these adjustments have only been applied to 33 countries located in Africa (see full list below) due to the lack of a complimentary approach to introducing the adjustments for countries outside of Africa. At the time our estimates were generated, this was the best data available to help account for the impact of COVID-19 on malaria. We anticipate that in the future new data will become available, such as in the form of PR surveys and routine surveillance data, that will reflect COVID-19 impacts.

COVID-19 adjustments were applied to the following countries: Angola, Burundi, Benin, Burkina Faso, Central African Republic, Côte d'Ivoire, Cameroon, Democratic Republic of the Congo, Congo, Ethiopia (and subnationals), Gabon, Ghana, Guinea, Guinea-Bissau, Equatorial Guinea, Kenya (and subnationals), Liberia, Madagascar, Mali, Mozambique, Malawi, Niger, Nigeria (and subnationals), Rwanda, Sudan, Sierra Leone, Somalia, South Sudan, Chad, Togo, United Republic of Tanzania, Uganda, and Zambia

References

1. Battle, K.E. et al. Treatment-seeking rates in malaria endemic countries. *Malar J* **15**, 20 (2016).

2. Battle, K.E. et al. Mapping the global endemicity and clinical burden of *Plasmodium vivax*, 2000-17: a spatial and temporal modelling study. *The Lancet*, doi:10.1016/S0140-6736(19)31096-7 (2019).
3. Bhatt, S. et al. The effect of malaria control on *Plasmodium falciparum* in Africa between 2000 and 2015. *Nature* (2015).
4. Cameron, E. et al. Defining the relationship between infection prevalence and clinical incidence of *Plasmodium falciparum* malaria. *Nature Communications* 6:8170 (2015).
5. Dzianach, P.A.; Rumisha, S.F.; Lubinda, J.; Saddler, A.; Van den Berg, M.; Gelaw, Y.A., Harris, J.R.; Vargas-Ruiz, C.A.; Cameron, E.;Gething, P.W.; Weiss, D.J. Evaluating COVID-19 related disruptions to healthcare accessibility in 2020-2021 and its potential effects on malaria burden in Sub-Saharan Africa. *Trop. Med. Infect. Dis.* **2023**, *7*, x. https://doi.org/10.3390/xxxxx
6. Gething, P. W. et al. Mapping *Plasmodium falciparum* Mortality in Africa between 1990 and 2015. *New England Journal of Medicine* 375, 2435-2445 (2016).
7. Pulse survey on continuity of essential health services during the COVID-19 pandemic: interim report, 27 August 2020. Geneva: World Health Organization; 2020 (https://www.who.int/publications/i/item/WHO-2019-nCoV-EHS_continuity-survey-2020.1).
8. Roca-Feltrer, A. et al. Estimates of the burden of malaria morbidity in Africa in children under the age of 5 years. *Tropical Medicine & International Health*, doi: 10.1111/j.1365-3156.2008.02076(13)771-83 (2008).
9. Second round of the national pulse survey on continuity of essential health services during the COVID-19 pandemic: January–March 2021. Geneva: World Health Organization; 2021 (https://apps.who.int/iris/bitstream/handle/10665/340937/WHO-2019-nCoV-EHScontinuity-survey-2021.1-eng.pdf?sequence=1&isAllowed=y).
10. Third round of the global pulse survey on continuity of essential health services during the COVID-19 pandemic. Geneva: World Health Organization; 2022 (https://www.who.int/publications/i/item/WHO-2019-nCoV-EHS_continuity-survey-2022.1).
11. Weiss, D. J. et al. Mapping the global prevalence, incidence, and mortality of *Plasmodium falciparum*, 2000-17: a spatial and temporal modelling study. *The Lancet*, doi:10.1016/S0140-6736(19)31097-9 (2019).
12. World Health Organization. (2022). World malaria report 2022. World Health Organization. https://apps.who.int/iris/handle/10665/365169. License: CC BY-NC-SA 3.0 IGO

PRISMA 2009 Flow Diagram for PR and API literature review

From: Moher D, Liberati A, Tetzlaff J, Altman DG, The PRISMA Group (2009). *P*referred *R*eporting *I*tems for *S*ystematic Reviews and *M*eta-*A*nalyses: The PRISMA Statement. PLoS Med 6(7): e1000097. doi:10.1371/journal.pmed1000097

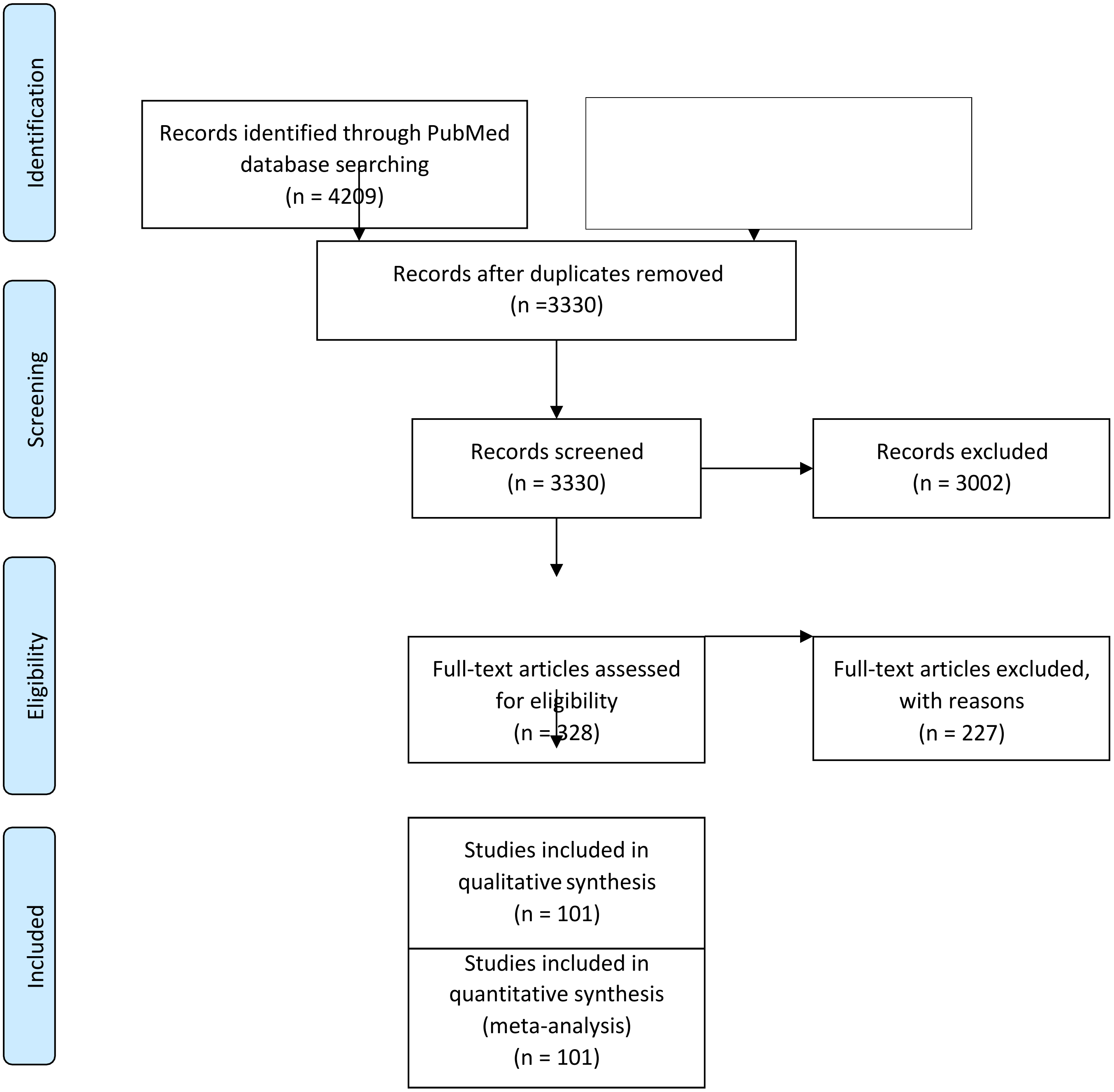

Maternal disorders

Maternal disorders non-fatal burden estimation includes estimation of disability due to seven direct obstetric complications: 1) abortion and miscarriage; 2) ectopic pregnancy; 3) obstructed labour and uterine rupture; 4) maternal haemorrhage; 5) maternal sepsis and other maternal infections; 6) maternal hypertensive disorders; and 7) other [direct] maternal disorders. These correspond to seven of nine subcauses of maternal death for which we estimate fatal burden. We do not estimate non-fatal burden related to the diseases and injuries underlying indirect maternal deaths and maternal deaths aggravated by HIV/AIDS, based on the premise that non-fatal burden associated with these diseases and injuries is captured in the respective underlying GBD cause.

Flowchart

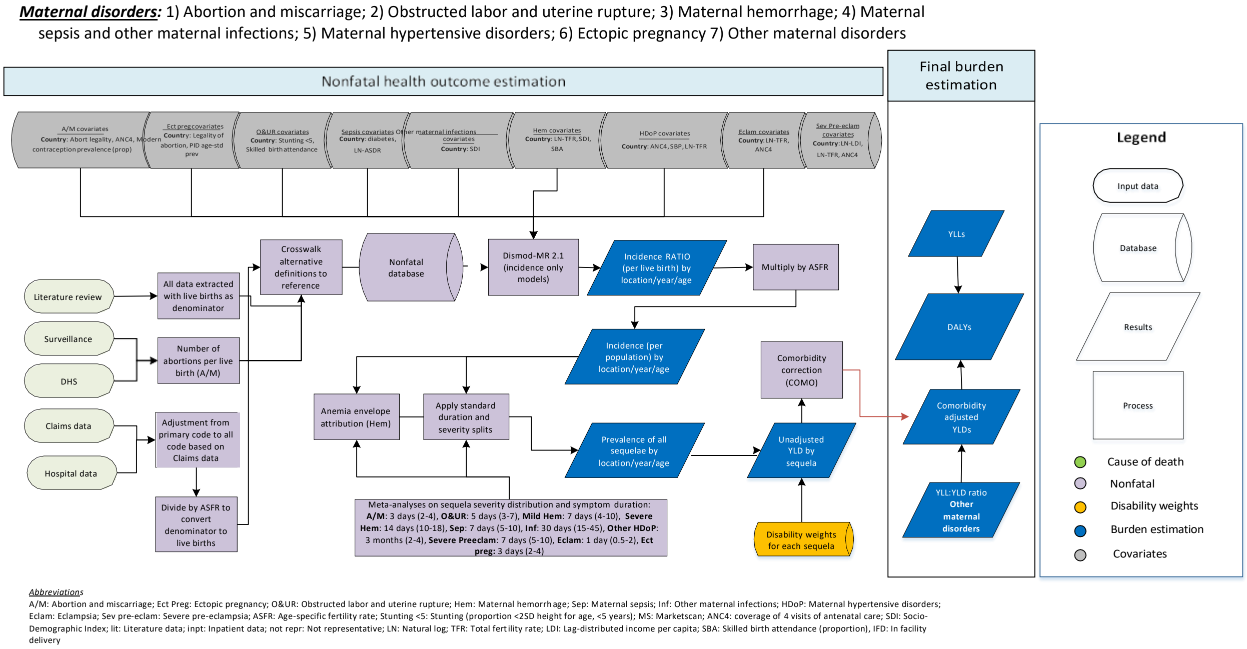

Input data and methodological summary

Case definition

Maternal disorders are direct obstetric complications of pregnancy, childbirth, and the postpartum period:

1) Abortion is defined as elective or medically indicated termination of pregnancy at any gestational age, regardless of symptoms or complications (abortion), or spontaneous loss of pregnancy before 24 weeks of gestation (miscarriage) with complications requiring medical care. Miscarriages that do not require medical care are not included in this cause.
2) Ectopic pregnancy is defined as a pregnancy that implants outside of the uterine cavity, generally presenting with abdominal pain and vaginal bleeding.
3) Obstructed labour and uterine rupture.
    a. Acute event includes failure to progress (no advance of the presenting part of the fetus despite strong uterine contractions), cephalopelvic disproportion (fetal size that is too

large for maternal pelvic dimensions), non-vertex fetal positioning during labour (any fetal position besides head down during labour; excludes non-vertex positioning during antepartum period), and uterine rupture during labour (non-surgical breakdown of uterine wall during labour and delivery). Perineal lacerations without any of the above conditions are excluded from the case definition. (*Estimation of the incidence and short-term disability due to these conditions is described in this appendix section*.)

   b. Obstetric fistula is defined as an abnormal opening between the vagina and the bladder or rectum with involuntary escape of urine, flatus, and/or faeces following childbirth. (*The non-fatal burden of fistulas is included in the non-fatal burden of obstructed labour in reporting, but estimation is described in a separate appendix section on "Fistula – impairment."*)

4) Maternal haemorrhage includes heavier than expected postpartum bleeding (>500 ml following vaginal delivery or >1000 ml after cesarean delivery) or antepartum bleeding (vaginal bleeding from any cause at or beyond 20 weeks of gestation and prior to onset of labour), or placental disorders with haemorrhage regardless of blood volume lost or timing of bleeding event. Placental disorders without haemorrhage are included with other [direct] maternal disorders.
5) Maternal sepsis and other maternal infections encompasses maternal sepsis during or following labour and delivery (defined as temperature <36°C or >38°C and clinical signs of shock [systolic blood pressure <90 mmHg and tachycardia >120 bpm]) as well as other maternal infections believed to have a close epidemiological relationship with pregnancy, including genitourinary tract infections (excluding sexually transmitted diseases), obstetrical wound infections, and breast infections related to childbirth and lactation.
6) Hypertensive disorders of pregnancy includes several subcategories:
   a. Gestational hypertension is the new onset of hypertension in a pregnant person after 20 weeks' gestation, as defined by having a blood pressure measured >140/90 mmHg on more than one occasion.
   b. Preeclampsia is defined by hypertension [>140/90 mmHg] and proteinuria [≥0.3 g/l], with or without signs of end-organ damage.
   c. Severe preeclampsia is defined by preeclampsia with severe hypertension [>160/100 mmHg] or other signs of end organ damage [liver: low platelets, elevated liver enzymes, coagulation issues; kidney: elevated creatinine; central nervous system: headaches or visual disturbances], syndrome of hypertension, elevated liver enzymes, low platelets [HELLP syndrome]).
   d. Eclampsia is defined as hypertension and seizures, with or without proteinuria.

   This definition excludes chronic hypertension in a pregnant person (hypertension present prior to 20 weeks' gestation) unless superimposed preeclampsia develops.

7) Other [direct] maternal disorders includes a variety of different obstetric complications. The most common of these in ICD-10-coded vital registration sources in terms of number of deaths include O88 (obstetric embolism), O26 (maternal care for other conditions predominantly related to pregnancy), O90 (complications of the puerperium, not elsewhere classified), O75 (other complications of labour and delivery, not elsewhere classified), C58 (malignant neoplasm of placenta), and O36 (maternal care for other fetal problems).

Input data

Systematic literature reviews have been completed annually since GBD 2010 and use a consolidated search string for all components of maternal burden estimation. These were updated on May 10, 2019, using the search string below.

(((("Postpartum Hemorrhage" OR "Uterine Hemorrhage") OR (maternal[Title/Abstract] OR pregnan*[Title/Abstract] OR mothers) AND (haemorrhag*[Title/Abstract] OR hemorrhag*[Title/Abstract]) NOT "case report"[All fields]) OR (("induced abortion" OR "Therapeutic abortion" OR "legal Abortion" OR "medical abortion" OR "miscarriage" OR "Abortion, Induced"[Mesh] OR "Abortion, Therapeutic"[Mesh] OR "Abortion, Legal"[Mesh] OR "ectopic Pregnancy") NOT ("case report"[Title/Abstract] OR "birth defect"[Title/Abstract] OR congenital[Title/Abstract])) OR ("obstructed labour" OR "obstructed labor" OR "labour dystocia" OR "labor dystocia" OR dystocia OR "cephalopelvic disproportion" OR "cephalo-pelvic disproportion") OR (("obstetric fistula" OR "vesicovaginal fistula") OR "rectovaginal fistula") OR (("Puerperal Infection"[Mesh] OR "Puerperal Infection" OR ((maternal[Title/Abstract] OR pregnan*[Title/Abstract]) AND (Sepsis OR infection[Title/Abstract]))) NOT "case report") OR ((pre-eclampsia[Title/Abstract] OR preeclampsia[Title/Abstract] OR eclampsia[Title/Abstract] OR Pre-Eclampsia[Mesh] OR Eclampsia[Mesh] OR "Hypertension, Pregnancy-Induced"[Mesh] OR "pregnancy induced hypertension"[Title/Abstract] OR "gestational hypertension"[Title/Abstract] OR "Hypertensive disorders of pregnancy"[Title/Abstract]) NOT ("case report" OR "kidney donor"[Title/Abstract] OR "kidney donors"[Title/Abstract] OR polymorphism*[Title/Abstract] OR endotheli*[Title/Abstract]))) OR((("maternal mortality"[Title/Abstract] OR "maternal death"[Title/Abstract] OR "maternal deaths"[Title/Abstract] OR "MM"[Title/Abstract] OR "confidential enquiry"[Title/Abstract] OR "confidential inquiry"[Title/Abstract] OR ((obstetric[Title/Abstract] OR pregnan*[Title/Abstract]) AND (etiology[Title/Abstract] OR cause[Title/Abstract] OR pattern[Title/Abstract]) AND (death[Title/Abstract] OR mortality[Title/Abstract])))) NOT (fetal[Title/Abstract] OR newborn*[Title/Abstract] OR neonatal[Title/Abstract] OR "case report" [Title/Abstract] OR "case study" [Title/Abstract] OR pathogenesis[Title/Abstract] OR thromboprophylaxis[Title/Abstract])) OR ((("maternal mortality"[Title/Abstract] OR "maternal death"[Title/Abstract] OR "maternal deaths"[Title/Abstract] OR "MMR"[Title/Abstract]) AND ("Afghanistan"[Title/Abstract] OR "Albania"[Title/Abstract] OR "Algeria"[Title/Abstract] OR "Andorra"[Title/Abstract] OR "Angola"[Title/Abstract] OR "Antigua and Barbuda"[Title/Abstract] OR "Argentina"[Title/Abstract] OR "Armenia"[Title/Abstract] OR "Azerbaijan"[Title/Abstract] OR "Bahrain"[Title/Abstract] OR "Bangladesh"[Title/Abstract] OR "Barbados"[Title/Abstract] OR "Belarus"[Title/Abstract] OR "Belize"[Title/Abstract] OR "Benin"[Title/Abstract] OR "Bhutan"[Title/Abstract] OR "Bolivia"[Title/Abstract] OR "Bosnia and Herzegovina"[Title/Abstract] OR "Botswana"[Title/Abstract] OR "Brazil"[Title/Abstract] OR "Brunei"[Title/Abstract] OR "Bulgaria"[Title/Abstract] OR "Burkina Faso"[Title/Abstract] OR "Burundi"[Title/Abstract] OR "Cambodia"[Title/Abstract] OR "Cameroon"[Title/Abstract] OR "Cape Verde"[Title/Abstract] OR "Central African Republic"[Title/Abstract] OR "Chad"[Title/Abstract] OR "China"[Title/Abstract] OR "Colombia"[Title/Abstract] OR "Comoros"[Title/Abstract] OR "Congo"[Title/Abstract] OR "Costa Rica"[Title/Abstract] OR "Croatia"[Title/Abstract] OR "Cuba"[Title/Abstract] OR "Cyprus"[Title/Abstract] OR "Côte d'Ivoire"[Title/Abstract] OR "Democratic Republic of the Congo"[Title/Abstract] OR "Djibouti"[Title/Abstract] OR "Dominica"[Title/Abstract] OR "Dominican Republic"[Title/Abstract] OR "Ecuador"[Title/Abstract] OR "Egypt"[Title/Abstract] OR "El Salvador"[Title/Abstract] OR "Equatorial Guinea"[Title/Abstract] OR "Eritrea"[Title/Abstract] OR "Ethiopia"[Title/Abstract] OR "Federated States of Micronesia"[Title/Abstract] OR "Fiji"[Title/Abstract] OR "Gabon"[Title/Abstract] OR "Georgia"[Title/Abstract] OR "Ghana"[Title/Abstract] OR "Grenada"[Title/Abstract] OR "Guatemala"[Title/Abstract] OR "Guinea"[Title/Abstract] OR "Guinea-Bissau"[Title/Abstract] OR "Guyana"[Title/Abstract] OR "Haiti"[Title/Abstract] OR "Honduras"[Title/Abstract] OR "India"[Title/Abstract] OR "Indonesia"[Title/Abstract] OR "Iran"[Title/Abstract] OR "Iraq"[Title/Abstract] OR "Jamaica"[Title/Abstract] OR "Jordan"[Title/Abstract] OR "Kazakhstan"[Title/Abstract] OR "Kenya"[Title/Abstract] OR "Kiribati"[Title/Abstract] OR "Kuwait"[Title/Abstract] OR "Kyrgyzstan"[Title/Abstract] OR "Laos"[Title/Abstract] OR "Latvia"[Title/Abstract] OR "Lebanon"[Title/Abstract] OR "Lesotho"[Title/Abstract] OR "Liberia"[Title/Abstract] OR "Libya"[Title/Abstract] OR "Lithuania"[Title/Abstract] OR "Macedonia"[Title/Abstract] OR "Madagascar"[Title/Abstract] OR "Malawi"[Title/Abstract] OR "Malaysia"[Title/Abstract] OR "Maldives"[Title/Abstract] OR "Mali"[Title/Abstract] OR "Malta"[Title/Abstract] OR "Marshall Islands"[Title/Abstract] OR "Mauritania"[Title/Abstract] OR "Mauritius"[Title/Abstract] OR "Moldova"[Title/Abstract] OR "Mongolia"[Title/Abstract] OR "Montenegro"[Title/Abstract] OR "Morocco"[Title/Abstract] OR "Mozambique"[Title/Abstract] OR "Myanmar"[Title/Abstract] OR "Namibia"[Title/Abstract] OR "Nepal"[Title/Abstract] OR "Nicaragua"[Title/Abstract] OR "Niger"[Title/Abstract] OR "Nigeria"[Title/Abstract] OR "North Korea"[Title/Abstract] OR "Oman"[Title/Abstract] OR "Pakistan"[Title/Abstract] OR "Palestine"[Title/Abstract] OR "Panama"[Title/Abstract] OR "Papua New Guinea"[Title/Abstract] OR "Paraguay"[Title/Abstract] OR "Peru"[Title/Abstract] OR "Philippines"[Title/Abstract] OR "Qatar"[Title/Abstract] OR "Romania"[Title/Abstract] OR "Russia"[Title/Abstract] OR "Rwanda"[Title/Abstract] OR "Saint Lucia"[Title/Abstract] OR "Saint Vincent and the Grenadines"[Title/Abstract] OR "Samoa"[Title/Abstract] OR "Saudi Arabia"[Title/Abstract] OR "Senegal"[Title/Abstract] OR "Serbia"[Title/Abstract] OR "Seychelles"[Title/Abstract] OR "Sierra Leone"[Title/Abstract] OR

"Singapore"[Title/Abstract] OR "Solomon Islands"[Title/Abstract] OR "Somalia"[Title/Abstract] OR "South Africa"[Title/Abstract] OR "South Sudan"[Title/Abstract] OR "Sri Lanka"[Title/Abstract] OR "Sudan"[Title/Abstract] OR "Suriname"[Title/Abstract] OR "Swaziland"[Title/Abstract] OR "Syria"[Title/Abstract] OR "São Tomé and Principe"[Title/Abstract] OR "Taiwan"[Title/Abstract] OR "Tajikistan"[Title/Abstract] OR "Tanzania"[Title/Abstract] OR "Thailand"[Title/Abstract] OR "The Bahamas"[Title/Abstract] OR "The Gambia"[Title/Abstract] OR "Timor-Leste"[Title/Abstract] OR "Togo"[Title/Abstract] OR "Tonga"[Title/Abstract] OR "Trinidad and Tobago"[Title/Abstract] OR "Tunisia"[Title/Abstract] OR "Turkmenistan"[Title/Abstract] OR "Uganda"[Title/Abstract] OR "Ukraine"[Title/Abstract] OR "United Arab Emirates"[Title/Abstract] OR "Uruguay"[Title/Abstract] OR "Uzbekistan"[Title/Abstract] OR "Vanuatu"[Title/Abstract] OR "Venezuela"[Title/Abstract] OR "Vietnam"[Title/Abstract] OR "Yemen"[Title/Abstract] OR "Zambia"[Title/Abstract] OR "Zimbabwe"[Title/Abstract])) NOT ("demographic and health survey"[Title/Abstract] OR "demographic and health surveys "[Title/Abstract] OR DHS[Title/Abstract] OR "reproductive health survey"[Title/Abstract] OR "reproductive health surveys"[Title/Abstract] OR RHS[Title/Abstract])) OR ((HIV[Title/Abstract] OR "Acquired Immunodeficiency Syndrome"[Title/Abstract] OR AIDS[Title/Abstract]) AND (pregnan*[Title/Abstract] OR "postpartum"[Title/Abstract] OR "post partum"[Title/Abstract]) AND ("mortality"[Title/Abstract] OR "death"[Title/Abstract]) NOT "case report")) AND (2017/07/01[PDat] : 3000[PDat]) NOT (animals[MeSH] NOT humans[MeSH]))

Figure 1: PRISMA 2020 flow diagram

From: Page MJ, McKenzie JE, Bossuyt PM, Boutron I, Hoffmann TC, Mulrow CD, et al. The PRISMA 2020 statement: an updated guideline for reporting systematic reviews. BMJ 2021;372:n71. doi: 10.1136/bmj.n71

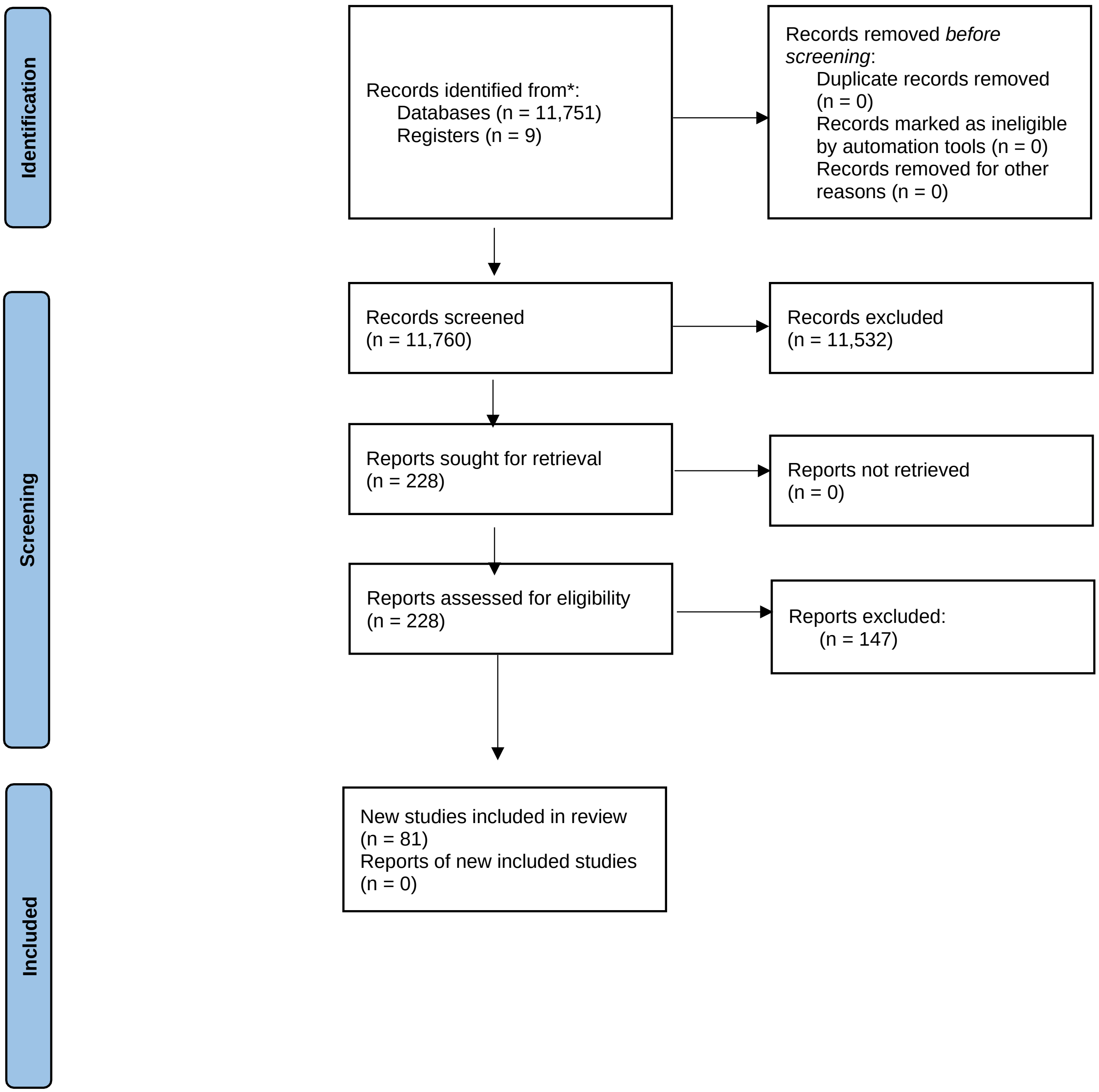

This search produced 11,751 hits for title and abstract review. Of these, 228 were selected for full-text review and 81 were extracted for inclusion in the models.

In addition, we searched ministry of health websites for pregnancy complication data and used Confidential Enquiry and other sources used in our maternal mortality analyses when they presented data on pregnancy complications. We also performed snowball searches for abortion reporting and surveillance data systems, finding multiple such systems throughout high-income countries and several geographies in central and eastern Europe. The table below summarises the number of sources used in each model by cause.

Table 1. Data sources used in estimation of non-fatal pregnancy complications

Cause/impairment name	Measure	Total sources	Countries with data
Maternal haemorrhage	All measures	495	88
Maternal haemorrhage	Incidence	495	88
Maternal sepsis and other maternal infections	All measures	420	79
Maternal sepsis and other maternal infections	Incidence	420	79
Maternal hypertensive disorders	All measures	554	105
Maternal hypertensive disorders	Incidence	554	105
Maternal obstructed labour and uterine rupture	All measures	395	76
Maternal obstructed labour and uterine rupture	Prevalence	33	26
Maternal obstructed labour and uterine rupture	Incidence	349	59
Maternal obstructed labour and uterine rupture	Other	13	6
Ectopic pregnancy	All measures	345	59
Ectopic pregnancy	Incidence	345	59
Maternal abortion and miscarriage	All measures	621	59
Maternal abortion and miscarriage	Incidence	621	59

Hospital discharge data were used, as were claims data from Poland and Singapore as well as MarketScan in the United States. These data were extracted and processed as described in the appendix section on claims, inpatient, and outpatient data, including use of primary-to-any inpatient ratio to correct for under-reporting of pregnancy complications in hospital datasets that rely only on primary discharge codes, and inpatient-to-outpatient ratio. Processing of clinical administrative data (ie, hospital and claims) were based on ICD-9 and ICD-10 codes as listed in the table below. We only used inpatient data, corrected for location-year-specific Healthcare Access and Quality (HAQ) Index value for most models, with four exceptions: hypertensive disorders of pregnancy (total), abortion and miscarriage, ectopic pregnancy, and other maternal infections.

All data were either extracted as incidence ratio (number of events/livebirth) or, if data were only available with population as the denominator, they were converted to incidence ratio using GBD 2021 age-specific fertility rate (number of livebirths/population). The reason is that most literature and surveillance data are expressed in terms of number of events per livebirth rather than per population. Hospital and claims data, which were centrally processed for all GBD 2021 causes to have population as the denominator, were transformed to have livebirths as the denominator by dividing by age-specific fertility rate (ASFR; livebirths per population).

Table 2. Maternal ICD codes

Non-fatal model	ICD-10 code	ICD-9 code
Ectopic pregnancy	O00	633
Maternal abortive outcome	N96, O01-O08	630-632, 634-636, 638
Maternal haemorrhage	O20, O43.2, O44-O46, O62.2, O67, O72	640-641, 661.2, 665, 666
Hypertensive disorders of pregnancy	O11-16	642 (excluding 642.0-642.2)
Severe pre-eclampsia	O14.1	642.5
Eclampsia	O15	642.6
Obstructed labour and uterine rupture	O64-O66, O70-O71, O83-O84	652.7, 653, 659.0, 660, 664-665, 669.5
Maternal sepsis	O85	646.5-646.6, 659.3, 670
Other maternal infections	O23, O41.1-O41.9, O86, O91	658.4, 659.2, 672

We also use input data to calculate incidence rate, prevalence, and severity of these disorders after completion of the DisMod-MR 2.1 models. We use data to estimate the proportion of maternal haemorrhage that is mild and severe, the proportion of hypertensive disorders of pregnancy that are long-term sequelae, and the proportion of puerperal sepsis cases that continue on to develop secondary infertility. We rely on expert opinion to determine the duration of each of the maternal disorders in order to calculate prevalence.

Data processing

Previously we derived empirical age patterns and performed all crosswalks in DisMod-MR 2.1. Our data processing approach changed for GBD 2019 such that all of this occurred prior to DisMod-MR 2.1 modelling, and we continued with these pre-modelling approaches in GBD 2021.

The first step of data processing was age-splitting. For any datum that did not entirely fit within a GBD age group, the observation was split to be multiple age-specific datapoints based on the age pattern predicted by GBD 2019 Decomposition 1 DisMod-MR 2.1 models.

The second step was to develop and apply adjustment factors to correct systematic biases in data collected using non-reference ("alternate") case definitions or collection methods. This process is referred to as "crosswalking". In accordance with GBD 2021 principles for data processing, to make data comparable, we began by evaluating the number of observations of each alternate type that matched in year, age, sex, and location of the population sampled with a corresponding observation of reference type. We considered within-study matches, where the same source reported both an alternative and reference type, as well as between-study matches, where the alternative and reference type were reported by different sources. For the disorders where we crosswalked based on data source type, we assessed the relative levels of the data and chose the reference data source to be the one with the most plausible values. The ratio of the two observations was then calculated, the standard error of the ratio was calculated using the delta method, and these ratios were modelled in log space using meta-regression—Bayesian, regularised, trimmed (MR-BRT), a meta-analytic tool developed for GBD 2019. The details of each of the crosswalks are described below by disorder. Across all disorders, all data sources

that only reported event rates for severe maternal morbidity or “near miss” were excluded, as a reliable crosswalk model could not be developed.

The results of the crosswalks are shown in the section below. In each graph, the effect size is the log of the adjustment coefficient estimated by the model, the x-axis is the age, and the points are the input ratios. The X points are trimmed by the model. If the adjustment factor is negative, then the alternative is underestimating the incidence of the specific disorder, relative to the reference, whereas when it is positive, the inverse is true.

Abortion and miscarriage

Surveillance data are the reference category for abortion and miscarriage. USA claims data were the only claims data in this dataset, and they had similar levels to the USA inpatient so we only ran one crosswalk. All clinical data were crosswalked to surveillance data by age.

Figure 1. Clinical to surveillance for abortion and miscarriage

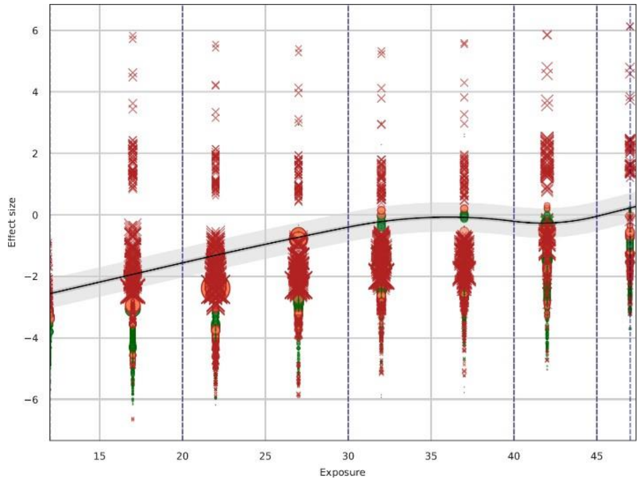

According to this model, clinical data underestimate the number of abortions compared to surveillance until age 45, where the crosswalk changes direction and clinical data overestimate the number of abortion relative to surveillance.

Ectopic pregnancy

We used hospital data adjusted for the inpatient-to-outpatient ratio for ectopic pregnancy. Claims data were the reference category. We crosswalked hospital data to claims by age.

Figure 7. Hospital data to claims data for ectopic pregnancy

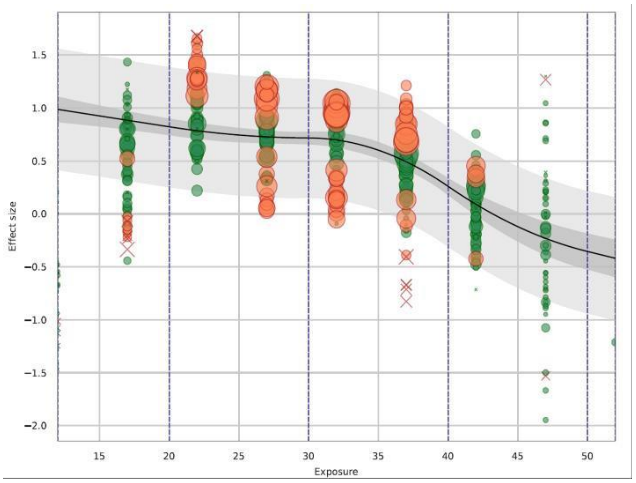

According to this model, hospital data overestimated the number of ectopic pregnancies for most of the reproductive age groups. The adjustment factor decreases with age, and after age 45, the inverse relationship is true.

Maternal haemorrhage

For maternal haemorrhage, the reference is all cases of maternal haemorrhage including post-partum bleeding ≥500 ml in vaginal births and ≥1000 ml in Caesarean sections and any amount of bleeding prior to birth. All data sources that reported only on antepartum haemorrhage (APH) or postpartum haemorrhage (PPH) were crosswalked to total haemorrhage by age. We included only within-study matches for this model, as many sources provided data for total haemorrhage as well as each subtype. We excluded severe cases of haemorrhage from this analysis due to sparsity of data.

Figure 3. PPH to all haemorrhage

Figure 4. APH to all haemorrhage

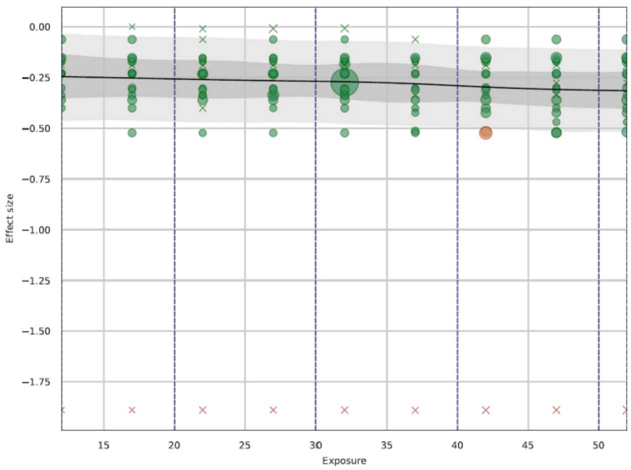

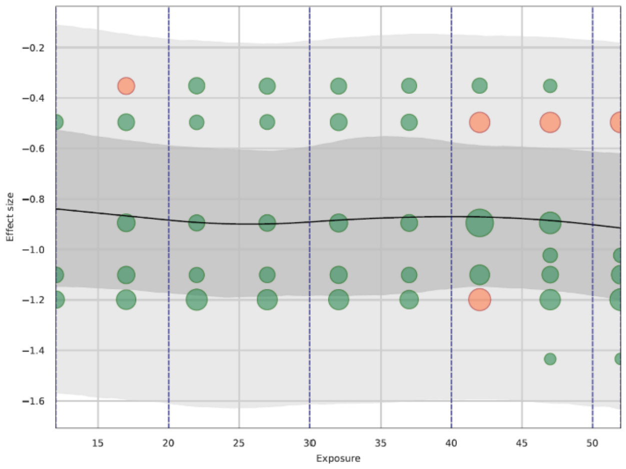

The age-specific crosswalk was retained for consistency across all maternal pregnancy complications even though it was not significant in this case. The model shows that there is no difference in the adjustment factor between the different ages.

Puerperal sepsis

Puerperal sepsis cases reported in literature studies that included data collected from a variety of sites and matched our case definition were the reference category. We crosswalked claims data to inpatient data by age. After this adjustment, we crosswalked all of the clinical data to the reference data by age. The age pattern for the claims to inpatient crosswalk was significant, with an increase with age until age 40. The age pattern of clinical to literature was slightly decreasing with age.

Figure 5. Claims to inpatient hospital

Figure 6. Clinical to lit. for puerperal sepsis

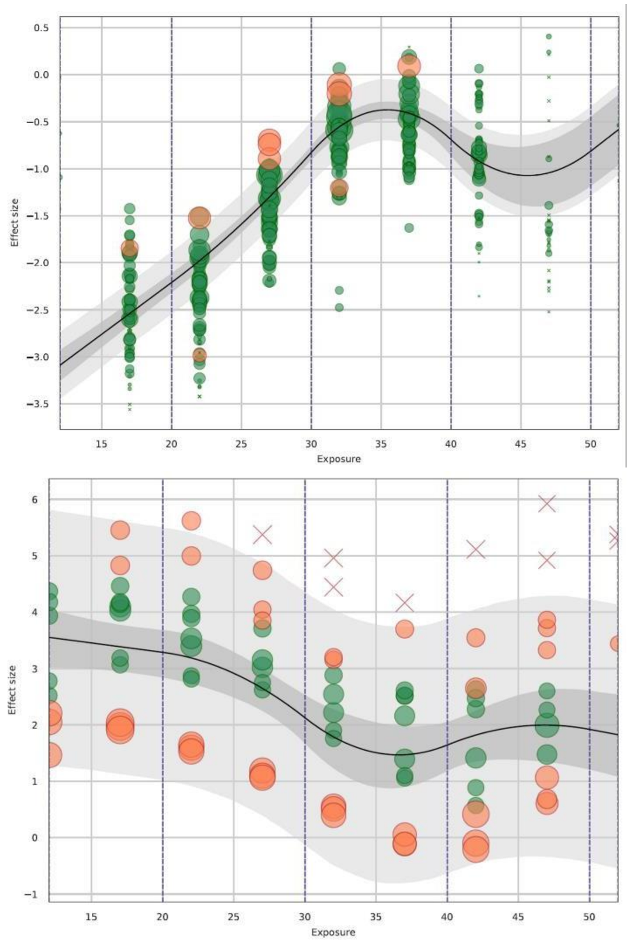

According to the model, claims data underestimated the number of sepsis cases, whereas clinical data overestimated the number of sepsis episodes relative to literature data.

Other maternal infections

Inpatient hospital data were the reference for other maternal infections. We crosswalked claims data to inpatient hospital data by age.

Figure 7. Claims to inpatient hospital data for other maternal infections

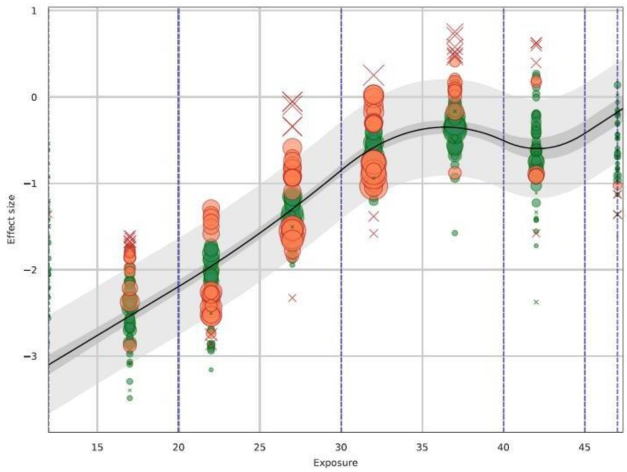

The model shows that claims data underestimate cases of maternal infections throughout the different age groups. The age pattern shows a steep increase in the ratio from ages 10 to 35. After age 35 the two data sources have more similar values, with the adjustment factor being closer to 0.

Hypertensive disorders of pregnancy

For the overall hypertensive disorders of pregnancy (HDoP), any sources that reported only on pre-eclampsia (PE) or pregnancy-induced hypertension (PIH) were crosswalked to total HDoP. We excluded studies reporting chronic hypertension from this process due to insufficient data.

Figure 8. PE to all HDoP

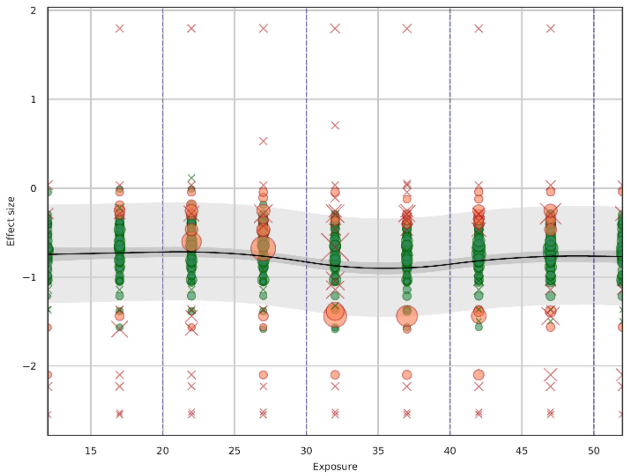

Figure 9. PIH to all HDoP

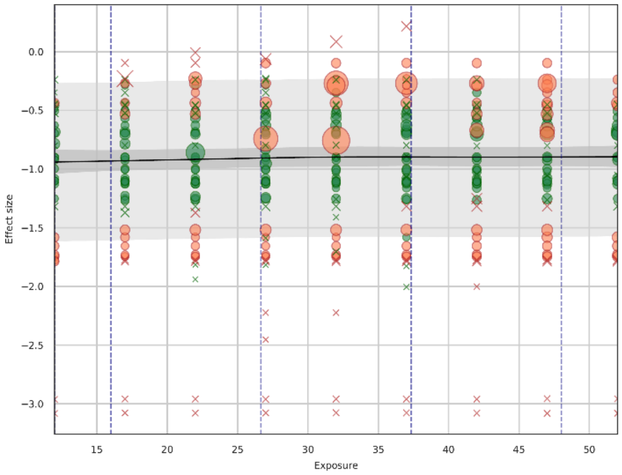

This crosswalk was again completed using only within-study matches and in an age-specific manner, although the age pattern was not significant.

Severe pre-eclampsia

We crosswalked claims data to inpatient hospital data for severe pre-eclampsia.

Figure 10. Claims to inpatient data for severe pre-eclampsia

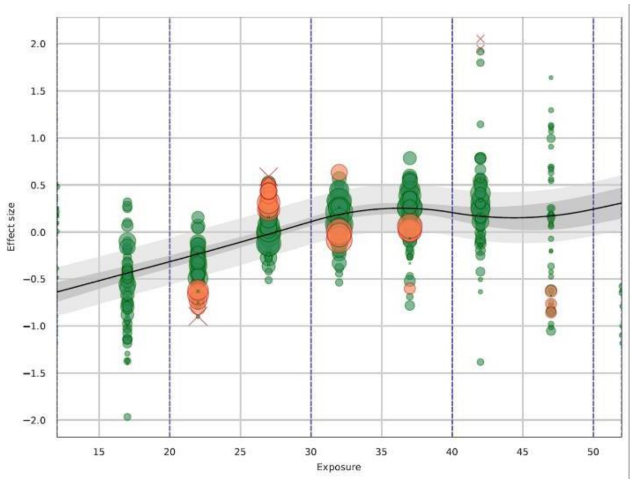

The crosswalk had a significant age pattern with a slight increase in the ratio of claims to inpatient data with age (mostly from 10 to 35). The model shows that claims underestimate the incidence of severe pre-eclampsia relative to hospital data in ages 10 to 35. After age 35, the two data sources converge, with a slight overestimation of claims data relative to hospital data in the oldest age group.

Eclampsia

For eclampsia, we considered the cases reported in literature studies that included data collected from a variety of sites and matched our case definition as the reference. We adjusted claims data to inpatient hospital data and then adjusted all of the clinical data to the reference data. These crosswalks were age-specific.

Figure 11. Claims data to inpatient hospital data **Figure 12. Clinical to lit. data for eclampsia**

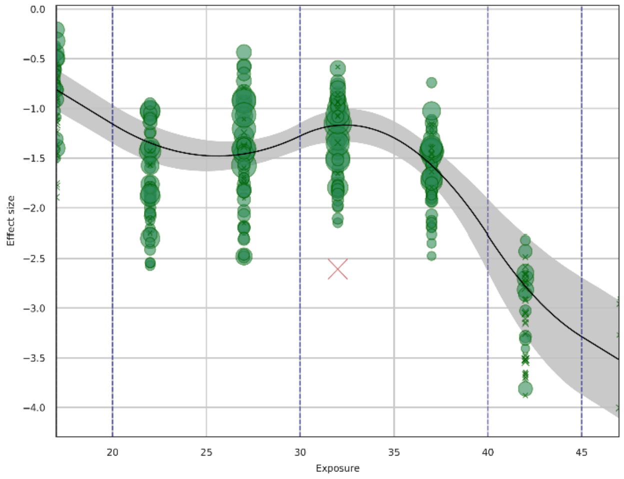

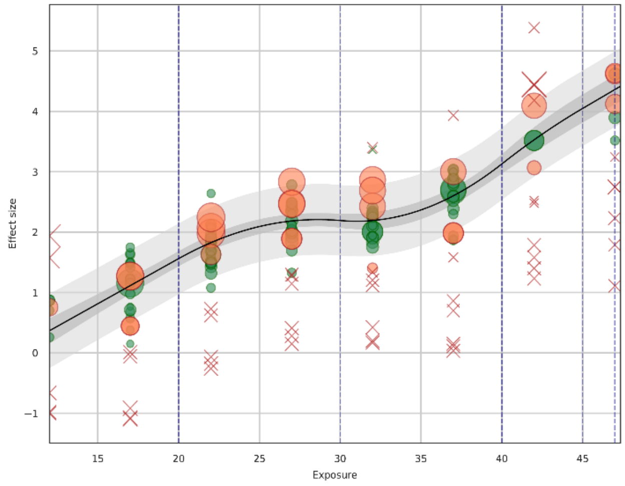

Both crosswalks had significant and opposite age patterns and directions. The claims to inpatient ratio decreases, indicating that claims data underestimated the incidence of eclampsia relative to hospital data, and this underestimation grew larger with age. The clinical to literature crosswalk increases with age, indicating that the clinical data overestimated the incidence of eclampsia and that this difference grew larger with age.

Modelling strategy

Modelling incidence ratios

We used the datasets described above to estimate incidence ratio for each specified pregnancy complication for each year-age-location combination in the GBD 2021 estimation framework using DisMod-MR 2.1. A series of country covariates were chosen to help drive the magnitude of estimates in areas of sparse or absent data. We included the respective log-transformed maternal mortality ratio (MMR) for each maternal disorder that was estimated in GBD 2019 as a predictive covariate for almost every model. Puerperal sepsis and ectopic pregnancy used the log-transformed age-standardised death rate (LN-ASDR) as a covariate, instead of MMR. The coefficients of the covariates in each model are shown below. No specific age or slope priors were used. All models were run with a time window of five years.

Model	Covariate name	Beta value	Exponentiated beta value
Maternal haemorrhage	Skilled birth attendance (proportion)	–0.0024 (–0.0061 to –0.00013)	1.00 (0.99 to 1.00)
	Socio-demographic Index	–0.1 (–0.1 to –0.1)	0.90 (0.90 to 0.90)
	MMR due to maternal haemorrhage	1.00 (0.042 to 1.94)	2.72 (1.04 to 6.99)
Maternal hypertensive disorders	Antenatal care (4 visits) coverage (proportion)	–0.000033 (–0.00012 to –7.7e-7)	1.00 (1.00 to 1.00)
	MMR due to maternal hypertensive disorders	1.02 (0.057 to 2.00)	2.77 (1.06 to 7.39)
	Age-standardised SEV for high blood pressure	0.000042 (0.0000033 to 0.00016)	1.00 (1.00 to 1.00)
	Age-standardised SEV for high body-mass index	2.00 (2.00 to 2.00)	7.38 (7.38 to 7.38)
Eclampsia	Antenatal care (4 visits) coverage (proportion)	–1.84 (–1.86 to –1.81)	0.16 (0.16 to 0.16)
	MMR due to maternal hypertensive disorders	1.00 (0.054 to 1.95)	2.71 (1.06 to 7.01)
	Age-standardised SEV for high body-mass index	2.00 (1.98 to 2.00)	7.36 (7.27 to 7.39)
Obstructed labour	Skilled birth attendance (proportion)	–0.0061 (–0.017 to –0.00021)	0.99 (0.98 to 1.00)
	Age-standardised SEV for child stunting	0.0097 (0.00052 to 0.031)	1.01 (1.00 to 1.03)
	MMR due to obstructed labour	1.01 (0.061 to 1.96)	2.74 (1.06 to 7.11)
Abortion and miscarriage	Legality of abortion	0.018 (0.017 to 0.019)	1.02 (1.02 to 1.02)
	Contraception (modern) prevalence (proportion)	–0.0011 (–0.0027 to –0.000055)	1.00 (1.00 to 1.00)
Ectopic pregnancy	Legality of abortion	–0.00036 (–0.00085 to –0.000012)	1.00 (1.00 to 1.00)
	Pelvic inflammatory disease age-standardised prevalence	0.50 (0.064 to 0.93)	1.65 (1.07 to 2.53)
Severe pre-eclampsia	Antenatal care (4 visits) coverage (proportion)	–0.0068 (–0.021 to –0.00021)	0.99 (0.98 to 1.00)
	MMR due to maternal hypertensive disorders	1.01 (0.059 to 1.96)	2.75 (1.06 to 7.12)
	Age-standardised SEV for high body-mass index	1.99 (1.98 to 2.00)	7.32 (7.21 to 7.39)
Maternal sepsis	Diabetes age-standardised prevalence (proportion)	1.86 (1.55 to 2.00)	6.42 (4.71 to 7.36)
	Maternal sepsis and other maternal infections (lnASDR)	0.058 (0.026 to 0.095)	1.06 (1.03 to 1.10)
Other maternal infections	Socio-demographic Index	–0.011 (–0.032 to –0.00037)	0.99 (0.97 to 1.00)
	Log-transformed age-standardised SEV scalar: HIV	0.021 (0.0034 to 0.039)	1.02 (1.00 to 1.04)
	MMR due to sepsis and other maternal infections	1.00 (0.039 to 1.98)	2.71 (1.04 to 7.28)

If the exponentiated beta coefficient is smaller than 1, then the covariate is negatively associated with the outcome; if it is greater than 1, then the inverse is true.

Estimating incidence rates, prevalence, and YLDs

After completion of DisMod-MR 2.1 models, all age-specific incidence ratios were then converted to incidence rates by multiplying by ASFR and then to prevalence by applying globally assumed durations of disability for each pregnancy complication.

Maternal haemorrhage was split between moderate (500 to <1000 ml blood loss) and severe (≥1000 ml blood loss) on the basis of a meta-analysis of 19 studies.[1] Data on the average duration of acute symptoms were not available, so after consultation with clinician collaborators, we assigned a duration of seven days (+/–3) for moderate haemorrhage and 14 days (+/– 4) for severe haemorrhage. The age-specific anaemia prevalence for maternal haemorrhage was also analysed as part of overall anaemia causal attribution for GBD 2021. The details of the anaemia analysis are described separately in the "Anaemia impairment" section. Briefly, after estimating total anaemia, a series of counterfactual distributions are generated based on the age- and sex-specific prevalence of each anaemia-causing condition and the quantitative effect that the condition has on haemoglobin concentration in the blood, a so-called "haemoglobin shift," that was derived by meta-analysing cohort studies, observational studies, or trials comparing the haemotological status of those with as compared to without the disease. Due to limited data on haemoglobin shift, all were assumed to be invariant over age, sex, location, and year.

For abortion and miscarriage, prevalence was calculated assuming incident cases have acute disability that persists for an average of three days (+/–1). The same was calculated for ectopic pregnancy. Obstructed labour was assigned a duration of five days (+/–2). Again, these determinations were based on clinical expert determination as we could not identify any data to inform this.

Hypertensive disorders of pregnancy (HDoP) was estimated in three models. The duration of severe pre-eclampsia was assigned to be 7 days (+/–2), and other HDoP was assigned a duration of three months (2–4). Eclampsia was a separate model, assigned a duration of one day (+/–1). The disability weight for eclampsia and severe pre-eclampsia is estimated as a combination of the disability weights for hypertensive disorders of pregnancy and the respective specific condition. A large number of those with severe pre-eclampsia go on to have long-term sequelae of the condition,[2] as do those with eclampsia.[3,4] We estimate these long-term sequelae by using the prevalence results of severe pre-eclampsia and eclampsia as input data for two full-compartment DisMod-MR 2.1 models. 62% (57–67) of the severe pre-eclampsia cases are estimated to be long-term sequelae. For eclampsia, we estimate that 6.5% (6.1–6.9) of the cases continue on to long-term sequelae in data-rich locations, whereas 11% (10.8–12) in not data-rich. We apply these percentages to the outputs of the severe pre-eclampsia and eclampsia DisMod-MR 2.1 models and use the resulting dataset as the input for the long-term sequelae models.

Maternal sepsis and other maternal infections were also estimated separately. Maternal sepsis was assigned a duration of five days (+/– 2) and, based on the same data identified in our review of pelvic inflammatory disease[5] (PID; described separately), 9% (7.7–10) of incident cases of puerperal sepsis were estimated to continue on to have secondary infertility due to maternal sepsis. We apply this proportion to the incidence results of puerperal sepsis and use them as input data for a full-compartment DisMod-MR 2.1 model. Other maternal infections were assigned a wide potential duration of 15 to 45 days (mean 30).

The sequelae, health states, lay descriptions, and disability weights for each maternal disorder are listed in table 3. Disability weights in GBD were calculated from two large surveys carried out in 2010 and 2013 as described in the Disability weight section of the appendix. We assigned abdominopelvic pain of varying severity to approximate the disability from maternal haemorrhage, obstructed labour, ectopic pregnancy, and abortion and miscarriage. We used two health states to estimate the disability weight due to eclampsia (moderate abdominal pain and severe epilepsy). Tension-type headaches and mild

motor plus cognitive impairment were used for severe pre-eclampsia. When two or more health states were combined for one sequela, we calculated the disability weight as described in the Disability weight section of the Diseases and Injuries appendix.

Table 2: Health states and disability weights for each of the non-fatal maternal disorders

Sequela	Health state name	Health state description	Disability weight
Maternal haemorrhage (<1 L blood lost)	Abdominopelvic problem, moderate	Has pain in the belly and feels nauseous. The person has difficulties with daily activities.	0.114 (0.078–0.159)
Maternal haemorrhage (>1L blood lost)	Abdominopelvic problem, severe	Has severe pain in the belly and feels nauseous. The person is anxious and unable to carry out daily activities.	0.324 (0.22–0.442)
Mild anaemia due to maternal haemorrhage	Anaemia, mild	Feels slightly tired and weak at times, but this does not interfere with normal daily activities.	0.004 (0.001–0.008)
Moderate anaemia due to maternal haemorrhage	Anaemia, moderate	Feels moderate fatigue, weakness, and shortness of breath after exercise, making daily activities more difficult.	0.052 (0.034–0.076)
Severe anaemia due to maternal haemorrhage	Anaemia, severe	Feels very weak, tired, and short of breath, and has problems with activities that require physical effort or deep concentration.	0.149 (0.101–0.209)
Severe pre-eclampsia	Moderate abdominal pain, tension-type headaches, mild motor plus cognitive impairment	Has pain in the belly and feels nauseous. The person has difficulties with daily activities. Has a moderate headache that also affects the neck, which causes difficulty in daily activities. Has some difficulty in moving around but is able to walk without help. The person is slow in learning at school. As an adult, the person has some difficulty doing complex or unfamiliar tasks but otherwise functions independently.	0.174 (0.120–0.239)
Eclampsia	Moderate abdominal pain and severe epilepsy	Has pain in the belly and feels nauseous. The person has difficulties with daily activities. Has sudden seizures with violent muscle contractions and stiffness, loss of consciousness, and loss of urine or bowel control. Between seizures the person has memory loss and difficulty concentrating.	0.602 (0.427–0.753)
Long-term sequelae of severe pre-eclampsia	Tension-type headaches, mild motor plus cognitive impairment	Has a moderate headache that also affects the neck, which causes difficulty in daily activities. Has some difficulty in moving around but is able to walk without help. The person is slow in learning at school. As an adult, the person has some difficulty doing complex or unfamiliar tasks but otherwise functions independently.	0.067 (0.041–0.103)
Long-term sequelae of eclampsia	Tension-type headaches, mild motor plus cognitive impairment	Has a moderate headache that also affects the neck, which causes difficulty in daily activities. Has some difficulty in moving around but is able to walk without help. The person is slow in learning at school. As an adult, the person has some difficulty doing complex or unfamiliar tasks but otherwise functions independently.	0.067 (0.041–0.103)
Other hypertensive disorders of pregnancy	Generic uncomplicated disease: worry and daily medication	Has a chronic disease that requires medication every day and causes some worry but minimal interference with daily activities.	0.049 (0.031–0.072)
Puerperal sepsis	Infectious disease, acute episode, severe	Has a high fever and pain, and feels very weak, which causes great difficulty with daily activities.	0.133 (0.088–0.19)

Infertility due to puerperal sepsis	Infertility, secondary	Has at least one child and wants to have more children. The person has a fertile partner, but the couple cannot conceive.	0.005 (0.002–0.011)
Other maternal infections	Infectious disease, acute episode, moderate	Has a fever and aches, and feels weak, which causes some difficulty with daily activities.	0.051 (0.032–0.074)
Obstructed labour, acute event	Abdominopelvic problem, severe	Has severe pain in the belly and feels nauseous. The person is anxious and unable to carry out daily activities.	0.324 (0.22–0.442)
Rectovaginal fistula	Rectovaginal fistula	Has an abnormal opening between her vagina and rectum causing flatulence and faeces to escape through the vagina. The person gets infections in her vagina and has pain when urinating.	0.501 (0.339–0.657)
Vesicovaginal fistula	Vesicovaginal fistula	Has an abnormal opening between the bladder and the vagina, which makes her unable to control urination. The woman is anxious and depressed.	0.342 (0.227–0.478)
Maternal abortive outcome	Abdominopelvic problem, moderate	Has pain in the belly and feels nauseous. The person has difficulties with daily activities.	0.114 (0.078–0.159)
Ectopic pregnancy	Abdominopelvic problem, moderate	Has pain in the belly and feels nauseous. The person has difficulties with daily activities.	0.114 (0.159–0.078)

Uncertainty and model selection

For all explicitly modelled maternal disorders, uncertainty bounds include uncertainty due to input data, crosswalks of non-reference data, uncertainty in numerical solutions (posteriors) of each DisMod-MR 2.1 model, duration of symptoms, and proportion of all persons with each type of symptom.

In consultation with GBD researchers and collaborators, final models were selected on a combination of qualitative and quantitative goodness of fit to input data, plausibility of geographical and temporal trends, consistency of age pattern, and, when available, comparison with other published studies on the epidemiology of pregnancy complications. Directionality, magnitude, and plausibility of adjustment factors and predictive covariates were also considered in the process of model development. Of note, due to the nature of statistical modelling, final results do not always cover the values reported in input data.

Other direct and indirect maternal causes

We estimated YLDs for other [direct] maternal disorders using the YLD-to-YLL ratio approach, where the ratio of YLD:YLL were pooled for all the causes in the list above and multiplied by the YLL for other [direct] maternal disorders. For other subcauses of maternal disorders, including late maternal death, indirect maternal disorders, and maternal death complicated by HIV/AIDS, we did not estimate any non-fatal burden based on the premise that the associated disability is captured in the respective causes.

References

1 Sloan N, Durocher J, Aldrich T, Blum J, Winikoff B. What measured blood loss tells us about postpartum bleeding: a systematic review. *BJOG* 2010; **117**: 788–800.

2	Roes EM, Raijmakers MT, Schoonenberg M, Wanner N, Peters WH, Steegers EA. Physical well-being in women with a history of severe preeclampsia. *J Matern Fetal Neonatal Med* 2005; **18**: 39–45.

3	Okanloma KA, Moodley J. Neurological complications associated with the pre-eclampsia/eclampsia syndrome. *Int J Gynaecol Obstet* 2000; **71**: 223–5.

4	Usta IM, Sibai BM. Emergent management of puerperal eclampsia. *Obstet Gynecol Clin North Am* 1995; **22**: 315–35.

5	Westrom LV. Chlamydia and its effect on reproduction. *J Br Fer Soc* 1996; **1**: 23–30.

Measles

Flowchart

Measles

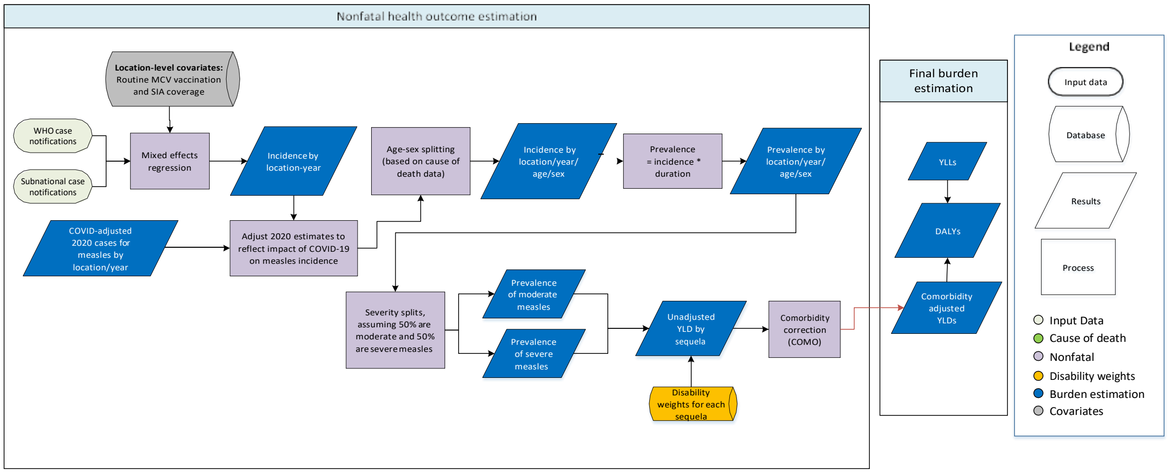

Case definition

Measles is a systemic illness caused by infection with the highly contagious measles virus. It is typically characterized by fever, cough, conjunctivitis, rhinitis and a diffuse maculopapular rash. While infection in healthy children can be benign and self-limited, important complications include pneumonia, encephalitis, diarrhea, and death. For measles, ICD-10 codes are B05-B05.9, Z24.4, and ICD-9 codes are 055-055.9, 484.0, V04.2, V73.2.

Measles

Quantity of interest	Reference or Alternative	Definition
Measles case fatality rate	Reference	Ratio of fatal cases of measles over total confirmed cases of measles in the sample
Measles incidence	Reference	Cases reported by national measles surveillance systems to WHO. Cases may be diagnosed via laboratory confirmation (including IgM or PCR) or

		clinical diagnosis following presentation of symptoms including cough, runny nose, fever, conjunctivitis, and red, blotchy skin

Input data

Model inputs

The custom measles incidence model primarily leverages the relationship between direct reports of measles case notifications annually released by the World Health Organization (WHO) in the Joint Reporting Form (JRF), modelled estimates of measles-containing-vaccine (MCV) vaccination coverage proportions for doses 1 and 2, and supplementary immunisation campaign (SIA) coverage to produce global estimates of measles cases. We supplement the national, JRF-reported case notifications with subnational case notifications from national health agencies in United States and Japan when complete and publicly available. New in GBD 2021, we added subnational data for Brazil, Great Britain, Indonesia, Italy, Poland, and South Africa and added additional years of subnational data from Japan. We also leverage WHO preliminary weekly case notification data from 2020 and 2021 to capture the COVID-associated changes to measles incidence. In total for GBD 2021, we included complete case notifications through December 31, 2021, adding in supplemental notifications from outbreaks in 2018 and 2019 where available. For high-income, central Europe/eastern Europe/central Asia and Latin America and the Caribbean super-regions as well as WHO-verified measles elimination locations outside of these super-regions, modelled estimates of measles incidence are replaced directly by case notifications as reported to WHO in years which have case notifications available after the model is fit, assuming complete reporting in these locations. In China and Jordan, modelled estimates of measles incidence are replaced by case notifications because of the strength of surveillance systems in these locations. Table 1 contains counts of all non-fatal input data used in the measles model. The case notification data is classified as other in Table 1 due to standard GBD practices for classifying source counts.

Table 1: Data inputs for measles morbidity modelling by parameter

	**Countries with data**	**New sources**	**Total sources**
Incidence	194	0	194
Prevalence	0	0	0
Remission	0	0	0
Other	200	273	7633

Modelling strategy

The general modelling approach used for GBD 2021 is similar to that used in GBD 2019. First, we make estimates of measles cases (ie, direct counts) in every location, using a mixed-effects linear regression model and the case notification inputs. This model uses measles case notifications as the dependent variable with GBD 2019 estimates of five-year rolling mean routine measles vaccination rates (first- and second-dose measles-containing vaccines) and five-year lagged coverage of supplementary immunisation activities (SIAs) as predictors. We use rolling means of MCV coverage calculated over the preceding five-year interval in order to better capture population-level vaccine-derived immunity among under-5-year-olds. This approach now incorporates coverage both in the current year and in recent years. The five-year

duration of the lag on supplementary immunisation activities was chosen from models tested with five-, seven-, and ten-year lags because it had the best out-of-sample performance in a five-fold cross validation framework.

In more detail, log-transformed incidence rates were regressed on the log of the proportion unvaccinated with first- and second-dose measles-containing vaccine (calculated using five-year rolling mean coverage), and additional SIA coverage lagged by one, two, three, four, and five years, with super-region, region, and country-level random effects:

$$Y_{ij} = \beta_0 + \beta_1 (1\text{-}MCV1_{ij}) + \beta_2 (1\text{-}MCV2_{ij}) + \beta_{a3} SIA_{aij} + u_j + e_{ij},$$

In the equation above, Y_{ij} is the natural log of measles incidence rate per 100,000 people; β_0 is the fixed-effect intercept; β_1 is the fixed-effects slope on the log-transformed proportion unvaccinated with first-dose measles vaccine (calculated using rolling mean coverage over the preceding five years); β_2 is the fixed-effects slope on the log-transformed proportion unvaccinated with second-dose measles vaccine coverage (similarly calculated using rolling mean five-year coverage); β_{a3} is the fixed-effects slope on supplementary measles immunization campaign coverage (administered doses over the target population of all under-15s) lagged by a=1-5 years; u_j is the location-level random effects; e_{ij} is the residual; i is the year; and j is the location. We assume a universal 95% attack rate in the absence of vaccination by generating a standard random effect consistent with this assumption and then applying that random effect in all years and locations when generating predictions from the model. From the fitted model, 1000 incidence predictions (draws) were generated for all ages, sexes, locations, and years using the estimated variance-covariance matrix. For locations detailed above that use case notifications directly, 1000 incidence draws are generated from a binomial distribution. We then adjusted all-age, both-sex measles incidence and prevalence estimates for 2020 and 2021 to account for the reductions in measles cases associated with the COVID-19 pandemic, as described elsewhere in this appendix.

These both-sex/all-age measles case estimates for every location were split into age- and sex-specific cases counts by utilising age-sex distributions, updated to include the new GBD 2021 under-5 age groups, obtained from cause of death modelling in CODEm. For all countries, we produced estimates for all age groups between six months and 64 years, under the assumption that all individuals born before 1957 are immune to measles. This is a change in the ages modelled from earlier GBD estimates, which were produced for all age groups between post-neonatal and 59 years. In rare cases, all in under-5 age groups, where a modelled incidence rate draw exceeded 0.9, we capped that incidence draw at 0.9. Prevalence rates were then calculated by multiplying case predictions at the draw level by an average case duration of ten days and dividing by GBD-estimated population in each location; incidence rates were computed by draw by dividing estimated cases by population in each location. All draw-level results were then summarised by the mean of the draws with 95% uncertainty intervals (2.5th and 97.5th percentiles of all draws).

Severity splits

We assume 50% of measles cases were acute episodes of moderate infectious disease and 50% were acute episodes of severe infectious disease. The lay descriptions and disability weights for measles severity levels derived from the GBD disability weights study are shown in Table 2.

Table 2. Severity distribution, details on the severity levels for measles in GBD 2021 and the associated disability weight (DW) with that severity

Severity level	Lay description	DW (95% CI)
Moderate	Has a fever and aches, and feels weak, which causes some difficulty with daily activities.	0.051 (0.032–0.074)
Severe	Has a high fever and pain, and feels very weak, which causes great difficulty with daily activities.	0.133 (0.088–0.19)

Meningitis

Flowchart

Input data and methodological summary for meningitis

Case definition

Meningitis is a disease caused by inflammation of the meninges, the protective membrane surrounding the brain and spinal cord, and is typically caused by an infection in the cerebrospinal fluid. Symptoms include headache, fever, stiff neck, and sometimes seizures. Included in the GBD modelling were cases meeting ICD-10 diagnostic criteria for meningitis due to bacteria or viruses (A39-A39.9, A87-A87.9, G00.0-G00.8, and G00-G03.0).

The case definitions accepted for meningitis are shown below.

Quantity of interest	Reference or alternative	Definition
Incidence of meningitis	Reference	Meningitis from inpatient hospital in clinical data or from literature, where cases are diagnosed by antigen test, blood test, cerebrospinal fluid test, polymerase chain reaction test, or latex agglutination test.
Incidence of meningitis	Alternative	Meningitis from private claims data.
Incidence of meningitis	Alternative	Cases detected by epidemiological surveillance.

Input data

Overall meningitis

Input data included all data previously used from GBD 2019, new data identified in our updated systematic review, newly acquired surveys, and new claims and inpatient data. Meningitis incidence data come from a systematic literature review, hospital inpatient and outpatient data, claims data from the USA, and surveillance data. In addition, sequelae and severity splits for bacterial meningitis were informed by a meta-analysis from Edmond and colleagues,[1] while sequelae and severity splits for viral meningitis were informed by a meta-analysis from Hudson and colleagues.[2]

The PubMed search string below was used to look for the incidence of meningitis cases, meningitis aetiology proportions, and the case-fatality rates of meningitis aetiologies (described in more detail in next section).

("meningitis"[MeSH Terms] OR "meningitis"[Title/Abstract]) AND ("case fatality rate"[Title/Abstract] OR “case fatality ratio”[Title/Abstract] OR “mortality”[MeSH] OR "mortality"[Title/Abstract] OR "fatal*"[Title/Abstract] OR “inciden*”[Title/Abstract] OR “cases”[Title/Abstract]) AND ("Meningitis, Haemophilus"[MeSH Terms] OR "Haemophilus"[Title/Abstract] OR “Hib meningitis”[Title/Abstract] OR "Meningitis, Pneumococcal"[MeSH Terms] OR "Pneumo*"[Title/Abstract] OR "Meningitis, Meningococcal"[MeSH Terms] OR "Meningococcal"[Title/Abstract] OR “Neisseria meningitidis”[MeSH Terms] OR “Neisseria meningitidis”[Title/Abstract] OR "Meningitis, Viral"[MeSH Terms] OR "Viral Meningitis"[Title/Abstract] OR "Streptococcus agalactiae"[MeSH Terms] OR "Streptococcus agalactiae"[Title/Abstract] OR “Group B Strep*”[Title/Abstract] OR “GBS”[Title/Abstract] NOT ("animals"[MeSH Terms] NOT "humans"[MeSH Terms]) AND (1990[DP] : 3000[DP]))

We ran this search string on 3/11/2020 for GBD 2021.

The inclusion criteria stipulated that: (1) the publication year must be between 1980 and the present year; (2) “caseness” was based on presence of bacterial pathogens in blood (with additional clinical presentation of meningitis) or cerebrospinal fluid, as diagnosed by culture, antigen test, polymerase chain reaction test, or latex agglutination test, or Gram staining; (3) sufficient information must be provided on study method and sample characteristics to assess the quality of the study; and (4) study samples must be representative of the general population. No limitation was set on the language of publication. We identified 265 studies after title-abstract screening, of which 133 met our inclusion criteria and were extracted. We excluded studies that were unrepresentative of the general population, studies that used animals as subjects, and studies (for incidence) with study population under 100.

[1] Edmond K, Clark A, Korczak VS, Sanderson C, Griffiths UK, Rudan I. Global and regional risk of disabling sequelae from bacterial meningitis: a systematic review and meta-analysis. *The Lancet Infectious Diseases*. 2010 May 1;10(5):317-28.

[2] Hudson JA, Broad J, Martin NG, Sadarangani M, Galal U, Kelly DF, Pollard AJ, Kadambari S. Outcomes beyond hospital discharge in infants and children with viral meningitis: a systematic review. *Reviews in Medical Virology*. 2020 Mar;30(2):e2083.

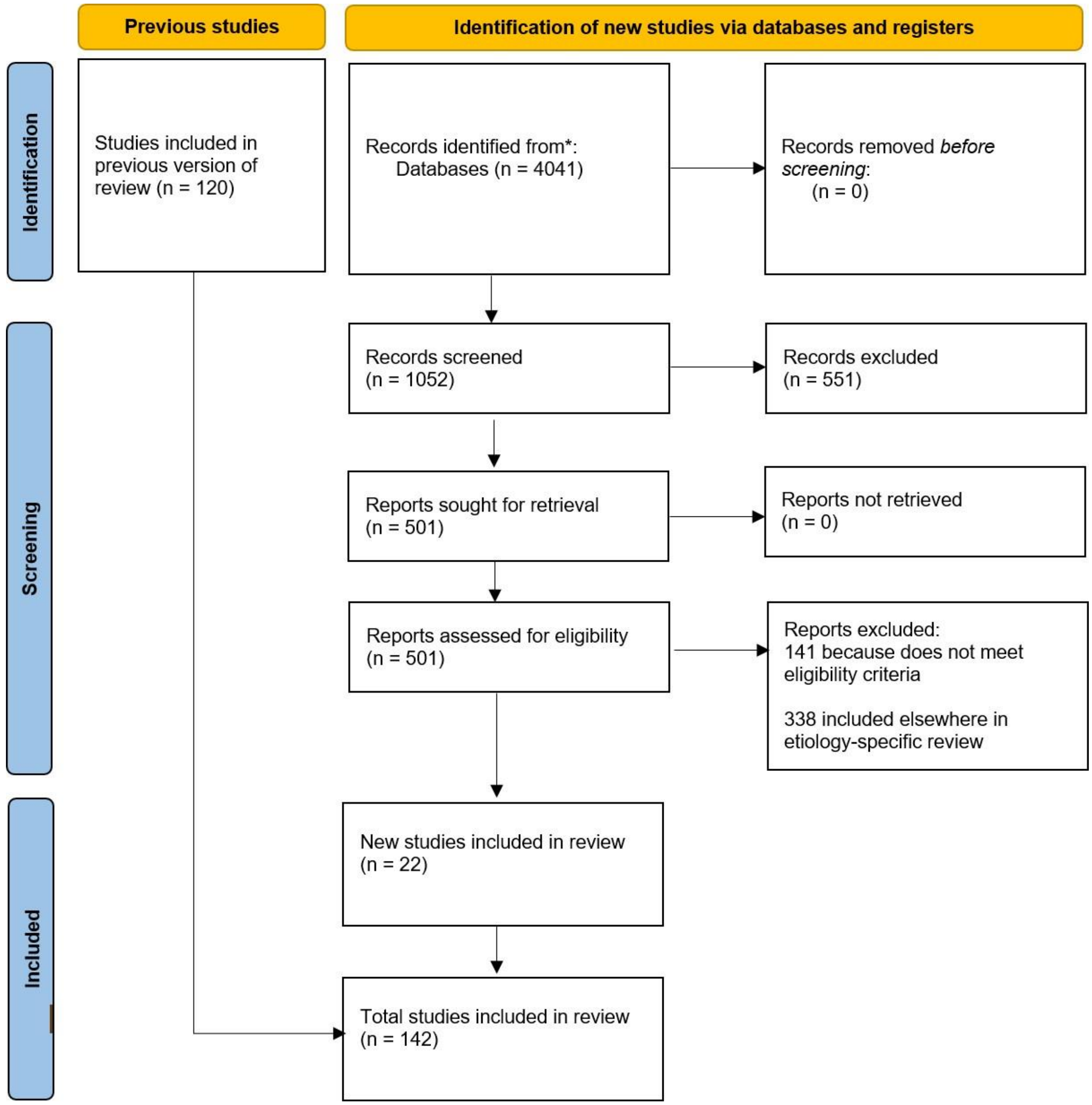

Figure 1: PRISMA diagram for meningitis 2021 systematic review for incidence

**Note: This systematic review was an update to the GBD 2019 review and doubled as a historical review of sources to capture previously missed studies. As a result, the number of sources being reviewed and excluded is ongoing.*

Table 1: Data inputs for meningitis morbidity modelling by parameter

	Countries with data	New sources	Total sources
Incidence	82	208	553
Prevalence	0	0	0
Remission	0	0	0
Other	124	484	990

Data were outliered or excluded if we found them unreasonable when compared to regional, super-regional, and global rates.

Aetiology proportions

Input data for aetiology estimation consisted of multiple cause of death, vital registration, hospital discharge, and microbial data, as well as the aforementioned systematic literature review and a separate, targeted review pulling data from citations found in meta-analyses. For data sources that provided ICD codes (multiple cause of death, vital registration, hospital discharge, and some microbial data), these codes were used to identify patients with meningitis and the culprit pathogen, when detailed. For the microbial data that did not provide ICD codes, we identified pathogens associated with meningitis using cerebrospinal fluid samples. The table below documents the ICD codes used to identify meningitis cases with known aetiology.

**Type of meningitis**	**ICD 10 code(s)**	**ICD9 code(s)**
Meningitis due to *Listeria*	A32.1	--
Meningitis due to *Neisseria meningitidis*	A39-A39.0	036-036.1, 320.5-320.8
Meningitis due to *Haemophilus*	G00.0	320.0
Meningitis due to *Streptococcus pneumoniae*	G00.1	320.1
Meningitis due to Group B *Streptococcus*	G00.2	320.2
Meningitis due to *Staphylococcus aureus*	G00.3	320.3
Meningitis due to virus	A87-A87.9, G03.0	047-049.9

Data on pathogens cultured from human infections were solicited from a wide array of international stakeholders (representing every inhabited continent). These included research hospitals, surveillance networks, and infection databases maintained by private laboratories and medical technology companies. For a full list of non-literature sources used for our estimates, please refer to the referenced article appendix (section 2).[1]

Bias corrections – incidence data

Hospital data were flagged with a covariate for inpatient hospital data and were used as the reference category. Claims data were flagged with year-specific covariates. Surveillance data were flagged with a covariate. For GBD 2021, an additional covariate was added to note surveillance with a broad definition, including suspected or viral meningitis. As described later, in non-fatal data modelling, we estimate bacterial meningitis first and add viral at the very end. Therefore, in our initial non-fatal data processing, we aim to include bacterial meningitis only to avoid double-counting viral cases. This "broadly defined" category was applied only to certain surveillance sources. The crosswalk allowed for composite

[1] Murray CJ, Ikuta KS, Sharara F, Swetschinski L, Aguilar GR, Gray A, Han C, Bisignano C, Rao P, Wool E, Johnson SC. Global burden of bacterial antimicrobial resistance in 2019: a systematic analysis. *The Lancet*. 2022 Feb 12;399(10325):629-55.

definitions, such that a source that is both a surveillance source and broadly defined would have both adjustments additively applied. Both claims and surveillance data were crosswalked to the reference category.

Table 2a: MR-BRT crosswalk adjustment factors for meningitis incidence

Data input	Reference or alternative case definition	Gamma	Basis function	B-spline coefficient, logit (95% UI)*	Adjustment factor **
Inpatient hospital (CF2)	Ref		---	---	---
Claims, inpatient only	Alt	0.00	intercept	0.78 (0.49 to 1.08)	2.19
			age_mid_0	–0.63 (–1.29 to 0.03)	0.53
			age_mid_1	0.42 (–0.60 to 1.44)	1.53
			age_mid_2	–0.56 (–1.08 to –0.04)	0.57
			age_mid_3	–0.48 (–0.84 to –0.11)	0.62
Claims, inpatient only, year 2000	Alt	0.00	intercept	0.88 (0.37 to 1.39)	2.41
			age_mid_0	–1.15 (–2.33 to 0.02)	0.32
			age_mid_1	0.63 (–1.22 to 2.48)	1.87
			age_mid_2	–1.39 (–2.26 to –0.51)	0.25
			age_mid_3	–0.87 (–1.49 to –0.26)	0.42
Surveillance	Alt	0.00	intercept	–3.46 (–4.29 to –2.63)	0.03
			HAQ Index	–0.05 (–0.06 to –0.05)	0.95
Broadly defined	Alt	0.65	intercept	5.75 (3.37 to 8.14)	317
			HAQ Index	0.04 (0.03 to 0.04)	1.04

**MR-BRT crosswalk adjustments can be interpreted as the factor the alternative case definition is adjusted by to reflect what it would have been had it been measured using the reference case definition. If the log/logit beta coefficient is negative, then the alternative is adjusted up to the reference. If the log/logit beta coefficient is positive, then the alternative is adjusted down to the reference.*

***The adjustment factor column is the exponentiated beta coefficient. For log beta coefficients, this is the relative rate between the two case definitions. For logit beta coefficients, this is the relative odds between the two case definitions.*

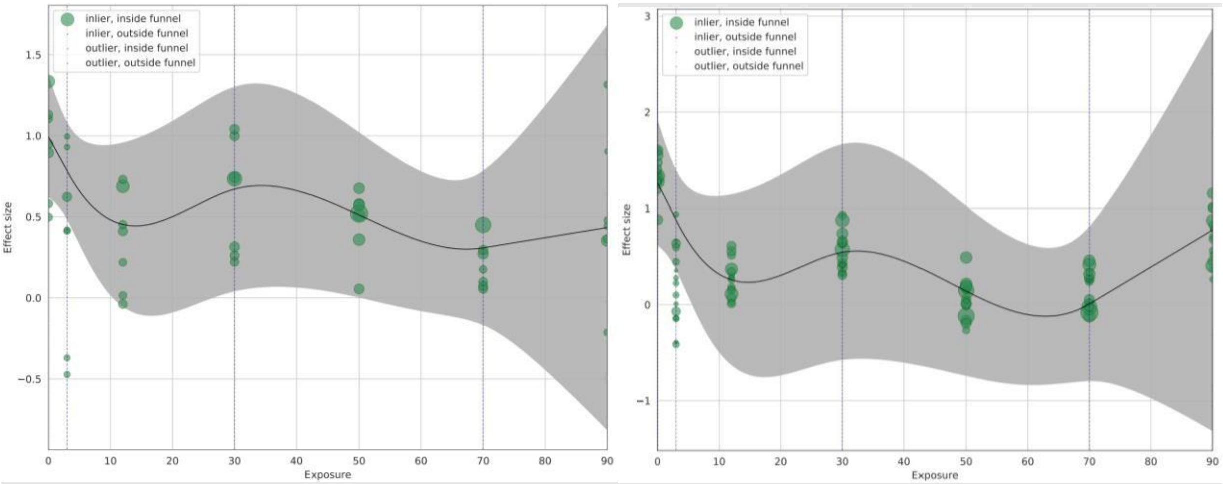

Figure 2a: Cubic spline on age midpoint for MarketScan claims crosswalk (left) and MarketScan 2000 claims crosswalk (right). Exposure is age midpoint; effect size is the adjustment factor in logit space.

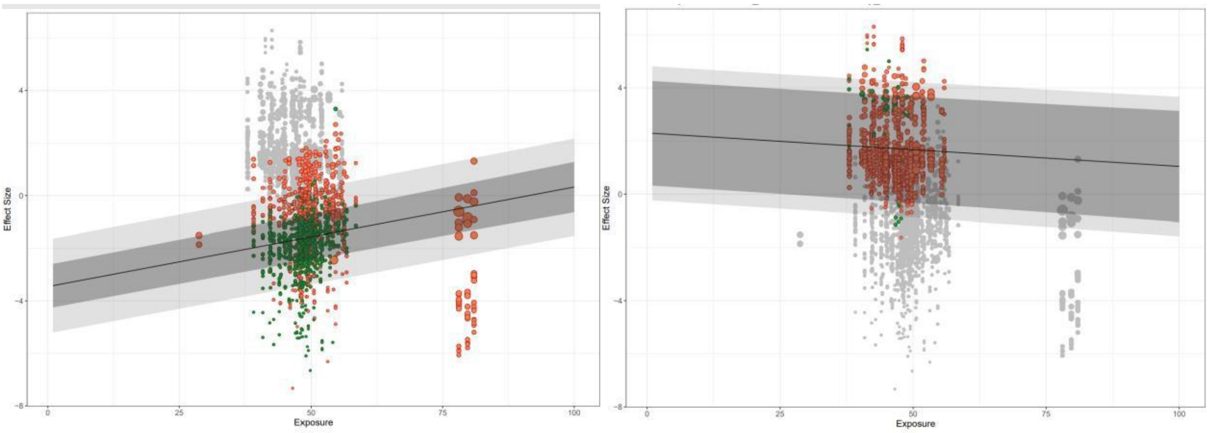

Figure 2b: Regression on Healthcare Access and Quality Index for surveillance data (left) and surveillance data that are broadly defined (right). Exposure is Healthcare Access and Quality Index; effect size is the difference between alternative and reference in logit space. Circles are data used in the regression; crosses are trimmed data. Green datapoints fall inside the funnel; red datapoints fall outside the funnel. The dark gray shading shows uncertainty not including between-study heterogeneity; the light gray shading shows uncertainty including between-study heterogeneity.

Modelling strategy: overall meningitis

Non-fatal outcomes were modelled using a combination of custom models and DisMod-MR 2.1. First, the overall incidence and prevalence of bacterial meningitis were modelled to estimate the short-term morbidity due to acute infection. This DisMod model had a set duration (1/remission) of four weeks with a range ±2 weeks. We also imposed caps on excess mortality for neonates and elders based on the highest excess mortality estimates from GBD 2019. We used the function in DisMod-MR 2.1 to pull in cause-specific mortality rate (CSMR) data from our CODEm and CoDCorrect analyses. We calculated excess mortality rate to estimate priors by dividing CSMR by prevalence, calculated from remission and incidence. To help inform trends where we lack data, we applied country-level covariates for proportion of the population at the subnational and country levels that lives within the meningitis belt in sub-

Saharan Africa,[1] coverage of MenAfriVac vaccine initiative, coverage of Hib3, and coverage of PCV3. Betas and exponentiated values (which can be interpreted as an odds ratio) are shown in the tables below country-level covariates. After bacterial meningitis was modelled using DisMod, viral meningitis incidence was calculated using the age-sex-location-year-specific ratios of bacterial:viral meningitis from the aetiology analysis (described below).

Disability weights

The basis of the GBD disability weight survey assessments is lay descriptions of sequelae highlighting major functional consequences and symptoms. The lay descriptions and disability weights for sequelae associated with meningitis are shown below.

Table 3. Severity distribution, details on the severity levels for meningitis and the associated disability weight (DW) with that severity

Severity split	Lay description	DW (95% CI)
Acute meningitis	This person has a high fever and pain, and feels very weak, which causes great difficulty with daily activities.	0.133 (0.088–0.19)
Acute viral meningitis	This person has a high fever and pain, and feels very weak, which causes great difficulty with daily activities.	0.133 (0.088–0.19)
Mild behaviour problems	This person is hyperactive and has difficulty concentrating, remembering things, and completing tasks.	0.045 (0.028–0.066)
Mild hearing loss	This person has great difficulty hearing and understanding another person talking in a noisy place (for example, on an urban street).	0.01 (0.004–0.019)
Mild hearing loss with ringing	This person has great difficulty hearing and understanding another person talking in a noisy place (for example, on an urban street), and sometimes has annoying ringing in the ears.	0.021 (0.012–0.036)
Moderate hearing loss	This person is unable to hear and understand another person talking in a noisy place (for example, on an urban street), and has difficulty hearing another person talking even in a quiet place or on the phone.	0.027 (0.015–0.042)
Moderate hearing loss with ringing	This person is unable to hear and understand another person talking in a noisy place (for example, on an urban street), and has difficulty hearing another person talking even in a quiet place or on the phone, and has annoying ringing in the ears for more than 5 minutes at a time, almost every day.	0.074 (0.048–0.107)
Moderately severe hearing loss	(custom DW from hearing loss impairment envelope)	

[1] Centers for Disease Control (CDC). *CDC health information for international travel 2016: the yellow book*. New York City, United States: Oxford University Press, USA, 2016.

Moderately severe hearing loss with ringing	(custom DW from hearing loss impairment envelope)	
Severe hearing loss	This person is unable to hear and understand another person talking, even in a quiet place, and unable to take part in a phone conversation. Difficulties with communicating and relating to others cause emotional impact at times (for example worry or depression).	0.158 (0.105–0.227)
Profound hearing loss	This person is unable to hear and understand another person talking, even in a quiet place, is unable to take part in a phone conversation, and has great difficulty hearing anything in any other situation. Difficulties with communicating and relating to others often cause worry, depression, or loneliness.	0.204 (0.134–0.288)
Complete hearing loss	This person cannot hear at all in any situation, including even the loudest sounds, and cannot communicate verbally or use a phone. Difficulties with communicating and relating to others often cause worry, depression, or loneliness.	0.215 (0.144–0.307)
Severe hearing loss with ringing	This person is unable to hear and understand another person talking, even in a quiet place, is unable to take part in a phone conversation, and has annoying ringing in the ears for more than 5 minutes at a time, almost every day. Difficulties with communicating and relating to others cause emotional impact at times (for example, worry or depression).	0.261 (0.175–0.36)
Profound hearing loss with ringing	This person is unable to hear and understand another person, even in a quiet place, is unable to take part in a phone conversation, has great difficulty hearing anything in any other situation, and has annoying ringing in the ears for more than 5 minutes at a time, several times a day. Difficulties with communicating and relating to others often cause worry, depression, or loneliness.	0.277 (0.182–0.387)
Complete hearing loss with ringing	This person cannot hear at all in any situation, including even the loudest sounds, and cannot communicate verbally or use a phone, and has very annoying ringing in the ears for more than half of the day. Difficulties with communicating and relating to others often cause worry, depression, or loneliness.	0.316 (0.212–0.435)
Moderate motor impairment	This person has some difficulty in moving around, and difficulty in lifting and holding objects, dressing, and sitting upright, but is able to walk without help.	0.061 (0.04–0.089)

Moderate motor plus cognitive impairments	This person has some difficulty in moving around, holding objects, dressing, and sitting upright, but can walk without help. This person has low intelligence and is slow in learning to speak and to do simple tasks.	0.203 (0.134–0.29)
Long-term mild motor impairment	This person has some difficulty in moving around but is able to walk without help.	0.01 (0.005–0.02)
Borderline intellectual disability	This person is slow in learning at school. As an adult, the person has some difficulty doing complex or unfamiliar tasks but otherwise functions independently.	0.011 (0.005–0.02)
Severe motor impairment	This person is unable to move around without help, and is not able to lift or hold objects, get dressed, or sit upright.	0.402 (0.268–0.545)
Epilepsy	(combined DW)	NA
Blindness	Is completely blind, which causes great difficulty in some daily activities, worry and anxiety, and great difficulty going outside the home without assistance.	0.187 (0.124–0.26)
Mild intellectual disability	This person has low intelligence and is slow in learning at school. As an adult, the person can live independently, but often needs help to raise children and can only work at simple, supervised jobs.	0.043 (0.026–0.065)
Monocular distance vision loss	This person is blind in one eye and has difficulty judging distances.	0.017 (0.009–0.029)
Mild motor plus cognitive impairments	This person has some difficulty in moving around but is able to walk without help. The person is slow in learning at school. As an adult, the person has some difficulty doing complex or unfamiliar tasks but otherwise functions independently.	0.031 (0.018–0.05)
Severe motor plus cognitive impairments	This person cannot move around without help, and cannot lift or hold objects, get dressed, or sit upright. The person also has very low intelligence, speaks few words, and needs constant supervision and help with all daily activities.	0.542 (0.37–0.702)
Moderate vision impairment	The person has vision problems that make it difficult to recognise faces or objects across a room.	0.031 (0.019–0.049)
Severe vision impairment	The person has severe vision loss, which causes difficulty in daily activities, some emotional impact (for example, worry), and some difficulty going outside the home without assistance.	0.184 (0.125–0.258)

Table 4a. Covariates. Summary of covariates used in the meningitis DisMod-MR meta-regression model

Covariate	Type	Parameter	Exponentiated beta (95% uncertainty interval)

Hib3 vaccine coverage (proportion)	Country-level	Incidence	0.61 (0.60–0.62)
PCV3 coverage (proportion)	Country-level	Incidence	0.58 (0.57–0.59)
Meningitis belt (proportion)	Country-level	Incidence	7.12 (6.71–7.37)
Proportion of total population covered by MenAfriVac initiative (meningitis meningococcal type A vaccine)	Country-level	Incidence	0.96 (0.93–1.00)
Healthcare Access and Quality Index	Country-level	Excess mortality	1.00 (0.99–1.00)

Modelling strategy: aetiology estimation

We estimated mutually exclusive proportions of meningitis cases attributable to the following set of pathogens: *Escherichia coli,* group B *Streptococcus, Haemophilus influenzae, Klebsiella pneumoniae, Listeria monocytogenes, Neisseria meningitidis,* polymicrobial infections, *Staphylococcus aureus, Streptococcus pneumoniae,* and viruses, as well as a residual "other pathogen" category. These proportions were estimated for five aggregate age groups: neonatal, post-neonatal to 5 years, 5–50 years, 50–70 years, and 70 years or older.

Aetiology proportions were calculated using an entirely new method from that applied in previous rounds of the GBD. Working from the assumption that aetiologies would follow a multinomial distribution, we estimated aetiology fractions using a method previously described as multinomial estimation of partial and composite observations (MEPCO).[1] Briefly, we constructed a network model with the dependent variable as the log ratio of cases between different pathogens and estimated over a flexible parameterisation of multinomial parameters using a maximum likelihood approach. Due to the unique pattern of meningitis in neonates, particularly the high prevalence of group B *Streptococcus*, we modelled neonatal and adult meningitis aetiology proportions separately.

The following covariates were used in our models:

**Covariate**	**Model**
Age group (neonatal, post-neonatal to 5, 5–50, 50–70, 70 plus)	Non-neonatal
Healthcare Access and Quality Index	Neonatal, non-neonatal
Proportion of people who as infants were vaccinated with PCV	Non-neonatal

Proportion of population age 15 or younger vaccinated against pneumococcus	Neonatal, non-neonatal
Proportion of people who as infants were vaccinated against *Haemophilus influenzae* type B	Non-neonatal
Proportion of population age 15 or younger vaccinated against *Haemophilus influenzae* type B	Neonatal, non-neonatal
Proportion of population covered by '10-'15 MenAfriVac rollout for meningococcal meningitis	Neonatal, non-neonatal

In order to use both partial and compositional data, we constructed a network model with the dependent variable as the log ratio of cases between different pathogens and estimated over a flexible parameterisation of multinomial parameters using a maximum likelihood approach. Consider a given infectious syndrome with a multinomial distribution of n mutually exclusive, collectively exhaustive aetiologies with probabilities $p = (p_1, \dots, p_n)$, so that each $p_j \in (0,1)$ and $\sum_j p_j = 1$. The likelihood of an observation of $c = (c_1, \dots, c_n)$, where c_j = number of cases of pathogen j in a total sample of N infections ($\sum_j c_j = N$), is:

$$P(c|p) = N! \prod_{j=1}^{n} \frac{p_j^{c_j}}{c_j!} \tag{1}$$

We modelled the probabilities using a composition of a link function with a linear predictor:

$$p_{i,j} = \exp(x_{i,j}^T \beta_j) \tag{2}$$

for observations i, a vector of covariates $x_{i,j}$, and a vector of coefficients β_j for each pathogen j. However, we did not observe these probabilities directly. Rather, we observed ratios between sums of these probabilities, which reduce to ratios between sums of cases within each study. These observations therefore take the form:

$$y = \frac{cases\ of\ pathogen\ A}{cases\ of\ pathogen\ B} = \frac{\sum_{j=1}^{n} w_{i,j}^{a} \exp(x_{i,j}^T \beta_j)}{\sum_{j=1}^{n} w_{i,j}^{b} \exp(x_{i,j}^T \beta_j)} \tag{3}$$

where $w_{i,j}^a$ is a weight of 0 or 1 that selects the mutually exclusive, collectively exhaustive most-detailed pathogens that make up observed pathogen A, which may be a composite observation. For example, for the "other bacterial, non-GBS" pathogen, $w_{i,j}$ would be 1 for *Staphyloccocus aureus, S. pneumoniae, Haemophilus influenzae, Neisseria meningitidis, Listeria monocytogenes, K. pneumoniae, E. coli*, and other pathogens and 0 for GBS and virus. We dropped all observations where either the numerator or denominator had zero observed cases in order to make this calculation and a forthcoming log transform possible. This may bias the model towards overestimating less common pathogens.

It is not possible to infer all coefficients β_j from the observations, since they are all relative. However, if we fix all of the coefficients for one pathogen to 0 as a reference group, then we obtain a well-posed inverse problem, as long as there is enough data to estimate the remaining coefficients. Without loss of

generality, we assumed $\beta_1 = 0$ for all elements and obtain estimates of the remaining $\beta_2, \dots, \beta_n$ by minimising the sum of the residuals between log-transformed observations y and corresponding log-transformed predictions from equation 3:

$$\min_{\beta_2,\dots,\beta_n} f(\beta) := \sum_i \frac{1}{\sigma_i^2}\left[\ln(y_i) - \ln\left(\sum_{j=1}^{n} w^a_{i,j}\, exp(x_{i,j}^T \beta_j)\right) + \ln\left(\sum_{j=1}^{n} w^b_{i,j}\, exp(x_{i,j}^T \beta_j)\right)\right]^2 \quad (4)$$

where σ_i^2 are variances corresponding to the datapoints. Equation 4 is a non-linear likelihood 632ptimized632on problem that that we 632ptimized using a standard implementation of the Gauss-Newton method.[1] We then re-normalised the optimal coefficients to obtain final predictions of the probabilities of each pathogen:

$$p_{i,j} = \frac{\exp(x_{i,j}^T \beta_j)}{\sum_{j} \exp(x_{i,\hat{j}}^T \beta_{\hat{j}})} \quad (5)$$

To quantify the uncertainty of this estimate, we used asymptotic statistics to obtain the posterior distribution of $(\beta_2, \dots, \beta_n)$. Specifically, using the Gauss-Newton Hessian approximation gave us the asymptotic information matrix for all β_j except for the reference pathogen, allowing us to sample draws of $\beta = (\beta_1 = 0, \beta_2, \dots, \beta_n)$. For each β draw and given feature x, we obtained a corresponding draw of p using equation 6.3.1.5.

This network regression with covariates framework allowed us to use partial and composite data that reported on one or only a few pathogens, or that reported multiple pathogens aggregated together. Networks, however, can be unstable with sparse data, and stable estimates have in some cases required the use of Bayesian priors in these models. In particular, we imposed Gaussian priors with mean 0 and non-zero variance on all coefficients except intercepts, to bias the model away from spurious effects driven by data sparsity. For the neonatal model, a prior standard deviation of 0.2 was used. For the non-neonatal model, we used a standard deviation of 0.1.

Finally, aetiology case proportions were converted into YLD proportions. In previous GBD rounds, bacterial meningitis YLDs were split into aetiology-attributable YLDs assuming that the distribution of permanent disabling sequelae by aetiology is the same as the distribution of cases by aetiology. However, now that viral meningitis is estimated as a part of the GBD, this method would have determined viral meningitis to have the most YLDs of any aetiology because it has the highest case proportion. However, this is not plausible because viral meningitis is less severe than bacterial meningitis.[1] Instead, we estimate viral and bacterial sequelae separately, as described below. We retain the assumption that within bacterial meningitis, the relative case proportions match the relative YLD proportions.

Modelling strategy: long-term sequelae estimation

[1] Nocedal J, Wright SJ, editors. *Numerical Optimization*, 2nd edn. New York: Springer-Verlag, 2006. DOI:10.1007/978-0-387-40065-5.

We split the long-term sequelae among survivors of acute infection. We calculated the acute-phase survival proportion by applying the excess mortality (estimated by the acute meningitis DisMod model) to incidence; excess mortality was converted to case-fatality rate by $e^{(-\text{excess mortality} \times 1/(\text{excess mortality} + \text{remission}))}$. We multiplied this proportion times the bacterial and viral incidence, to get the incidence of meningitis-with-survival for bacterial and viral meningitis, respectively.

The survivors were then subject for long-term sequelae by applying the post-discharge proportions of health consequences calculated by a meta-analysis by Edmond and colleagues for bacterial meningitis, and Hudson and colleagues for viral meningitis.[1,1] We calculated the proportion of acute meningitis survivors who experience major or minor long-term impairments for all aetiologies. This proportion was based off a regression of log-transformed GDP and ratio values from Edmonds and colleagues. The regression is shown below:

$$y = -0.347 \ln(GDP) + 1.28$$

For viral meningitis, we used a single global, meta-analysed proportion value for viral meningitis: because viral is not treatable by antibiotics, we assume that the proportion with sequelae does not vary greatly by country. The proportion with any impairments was further split into specific impairments according to the proportions from the aetiology-relevant systematic review, which were grouped into vision loss, hearing loss, motor/cognitive impairments, and epilepsy.

All subsequently described steps were performed in parallel for bacterial and viral meningitis. The calculated incidence of long-term sequelae was then converted to prevalence by two different approaches. For the sequelae not associated with excess mortality, which were vision loss, hearing loss, intellectual disability, motor impairment, and behavioural problems, the incidence of each age was cumulatively added up to the subsequent age (assuming half-cycle) to construct prevalence at each age. If the sequela is associated with excess mortality (epilepsy and moderate-to-severe cognitive impairments), the calculated incidence was used as in input to the ODE solver together with the corresponding mortality parameters (excess mortality data from the epilepsy envelope DisMod model, and standardised mortality ratio data from a neonatal encephalopathy meta-analysis, converted to excess mortality using all-cause mortality estimates) to estimate the prevalence. Vision loss, hearing loss, and epilepsy estimates were squeezed and severity split centrally.

To calculate YLDs attributable to all meningitis, bacterial and viral attributable YLDs were ultimately summed. To calculate proportion of YLDs attributable to viral meningitis, the viral meningitis YLDs were divided by the summed YLDs. To calculate proportion of YLDs attributable to each bacterial aetiology, the case proportions estimated as described in the aetiology section were squeezed to the overall bacterial meningitis YLDs, such that the sum of the proportion of YLDs attributable to each bacterial aetiology would equal the proportion of YLDs attributable to all bacterial aetiologies.

Motor neuron diseases

Flowchart

Motor neuron diseases

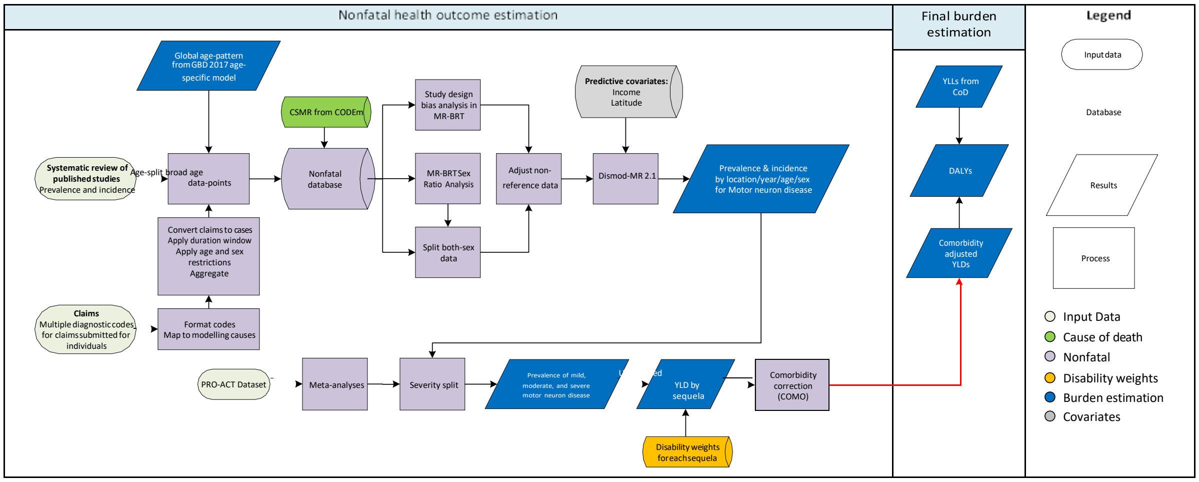

Case definition

Motor neuron diseases (MND) are a set of chronic, degenerative, and progressive neurological conditions typified by the destruction of upper and/or lower motor neurons and the subsequent deterioration of voluntary muscle activity. The most common MND is amyotrophic lateral sclerosis (ALS). The El Escorial Criteria are the gold standard diagnostic criteria. The ICD-10 code corresponding to motor neuron diseases is G12.

Input data and data processing

A full systematic review was last conducted for GBD 2015 and will be updated in a future round of GBD. The following search string guided our search, which resulted in 3,146 hits with 58 sources meeting extraction criteria: (1) the study is a representative population-based study with well-defined sample, (2) reports on prevalence, incidence, remission, excess mortality, relative risk of mortality, standardised mortality ratio, or with-condition mortality rate for motor neuron diseases in aggregate or a specified motor neuron disease.

(('motor neuron disease'[MeSH Terms] OR ('motor'[All Fields] AND 'neuron'[All Fields] AND 'disease'[All Fields]) OR 'motor neuron disease'[All Fields] OR ('motor'[All Fields] AND 'neuron'[All Fields] AND 'diseases'[All Fields]) OR 'motor neuron diseases'[All Fields]) OR ('amyotrophic lateral sclerosis'[MeSH Terms] OR ('amyotrophic'[All Fields] AND 'lateral'[All Fields] AND 'sclerosis'[All Fields]) OR 'amyotrophic lateral sclerosis'[All Fields]) OR ALS[All Fields] OR ('motor neuron disease'[MeSH Terms] OR ('motor'[All Fields] AND 'neuron'[All Fields] AND 'disease'[All Fields]) OR 'motor neuron disease'[All Fields] OR ('primary'[All Fields] AND 'lateral'[All Fields] AND 'sclerosis'[All Fields]) OR 'primary lateral sclerosis'[All Fields]) OR ('Politics Life Sci'[Journal] OR 'pls'[All Fields]) OR ('muscular atrophy, spinal'[MeSH Terms] OR ('muscular'[All Fields] AND 'atrophy'[All Fields] AND 'spinal'[All Fields]) OR 'spinal muscular atrophy'[All Fields] OR ('progressive'[All Fields] AND 'muscular'[All Fields] AND 'atrophy'[All Fields]) OR 'progressive muscular atrophy'[All Fields]) OR PBP[All Fields] OR ('pseudobulbar palsy'[MeSH Terms] OR ('pseudobulbar'[All Fields] AND 'palsy'[All Fields]) OR 'pseudobulbar palsy'[All Fields])) AND (('epidemiology'[Subheading] OR 'epidemiology'[All Fields] OR 'epidemiology'[MeSH Terms]) OR population-based[All Fields])

Data from the systematic review were manually extracted for GBD 2015. For GBD 2021, as in 2019 datapoints referring to broad age groups were split according to the age-pattern estimated for that datum's location in a preliminary model that used only age-specific data. For GBD 2019, all previously extracted studies were reviewed and assigned a design variable to indicate if the case definition was limited to ALS only or encompassed all MND. This was maintained for GBD 2021.

Beyond data from the systematic review, as in previous rounds of GBD, we made use of claims data as obtained and processed by the GBD Clinical Informatics team and described in a separate section of this appendix. These data link claims for all inpatient and outpatient encounters for a single individual, and provide primary and secondary diagnoses for all encounters. An individual was extracted from claims data as a prevalent case if they had any MND code as any diagnosis in one or more inpatient encounters or two or more outpatient encounters.

Total sources used for modelling in GBD 2021 are listed in the table below:

Measure	Total sources	Countries with data
All measures	73	18
Prevalence	24	1
Incidence	48	18
Remission	0	0
Other	1	1

In GBD 2021, all sex-specific data were used to estimate a pooled sex-ratio using a MR-BRT (meta-regression—Bayesian, regularised, trimmed) model[1] (Additional information can be found in appendix 1, section 4.4.1 of the cited paper). This ratio was combined with sex-specific population estimates for the year-age-location combinations corresponding to each datapoint reported for both sexes combined, to estimate sex-specific datapoints prior to modelling. These were applied by calculating male prevalence:

$$prev_{male} = prev_{both} * \frac{pop_{both}}{(pop_{male} + ratio * pop_{female})}$$

and then calculating female prevalence:

$$prev_{female} = ratio * prev_{male}$$

(Or the equivalent equations for incidence or other epidemiological measure.)

Two pre-modelling adjustments were then made to adjust for systematic biases in some data sources: data reporting on ALS only and data from USA claims in the year 2000 (the U.S. Marketscan database covers a commercially insured sub-population from all U.S. states). Two studies of ALS only were found to be closely matched in year, age, sex and time with three studies of MND more broadly, and the log-ratios for all matched pairs were entered into a MR-BRT meta-analysis. Commercial claims data from the USA in 2000 were matched to USA claims data from later years with more complete coverage of the population, and these log-ratios were entered into a separate MR-BRT model.

MR-BRT Crosswalk Adjustment Factors

Data input	Reference or alternative case definition	Beta Coefficient, Log (95% CI)*	Adjustment factor**
Surveys of all MND using combined clinical, imaging, electrophysiology and imaging criteria<br>OR<br>Claims data from location-years other than USA 2000	Ref	---	---
USA claims from year 2000	Alt	-0.026 (-1.2 to 1.1)	0.97 (0.31 to 3.1)
Surveys limited to ALS only	Alt	-0.13 (-0.23 to -0.029)	0.88 (0.79 to 0.97)

**MR-BRT crosswalk adjustments can be interpreted as the factor the alternative case definition is adjusted by to reflect what it would have been had it been measured using the reference case definition. If the log/logit beta coefficient is negative, then the alternative is adjusted up to the reference. If the log/logit beta coefficient is positive, then the alternative is adjusted down to the reference.*
***The adjustment factor column is the exponentiated beta coefficient. For log beta coefficients, this is the relative rate between the two case definitions. For logit beta coefficients, this is the relative odds between the two case definitions.*

After extraction and processing, some studies were marked as outliers and excluded on a case-by-case basis if they were inconsistent with established regional or temporal trends or if concerns about study quality were identified during extraction and processing.

Modelling strategy

We use DisMod-MR 2.1[1] (disease model—Bayesian meta-regression, details on this method can be found in appendix 1, section 4.5 of the citation) as the main analytical tool for MND estimation. Inputs included prevalence and incidence data, as described above, as well as the cause-specific mortality rate (CSMR) estimated in the GBD causes of death analysis, and excess mortality rate (EMR) obtained by dividing CSMR by prevalence datapoints. Prior settings are limited to 0 remission at all ages and maximum incidence of 0.0004. We also constrain the super-region random effects for prevalence and incidence to -0.5 and 0.5 to account for spurious inflation of regional differences.

We employed the following covariates to improve model predictions:

Covariate	Measure	Beta coefficient (95% CI)	Exponentiated
Absolute value of average latitude	Prevalence	0.037 (0.036 to 0.038)	1.04 (1.04 to 1.04)
LDI (I$ per capita)	Excess mortality rate	-0.5 (-0.5 to -0.5)	0.61 (0.61 to 0.61)

Although there are no known cures for MND, we expect disease management to differ globally – largely as a function of available resources. To capture this, we use the natural log of lagged distributed income per capita as a proxy to capture this relationship in the estimation of excess mortality.

As described in the literature, extreme latitude may be associated with higher prevalence and incidence of motor neuron disease, although the pathway to explain the association is not understood. Our operationalisation of latitude is created by taking the absolute value of a population-weighted average of latitude by country. The underlying population distribution rasters are part of the Gridded Population of the World dataset.

Severity splits

To calculate severity and disability due to MND we analysed a dataset from Pooled Resource Open-access ALS Clinical Trials (PRO-ACT). PRO-ACT is a large ALS clinical trials dataset, with a total of 8,635 ALS patient records from multiple completed clinical trials. Among these, we conducted the final analysis with n=4838 (56%) of the patients with complete ALS Function Rating Score (ALSFRS) with average follow-up time of 184 days (min: -22, max: 648), in which 2,999 (62%) received experimental (medication) treatments and 1,301 (27%) received placebo (in these trials, the medications tested were found to be no better than placebo with respect to their effects on ALS progressions).

The ALSFRS is an instrument for evaluating the functional status of patients with amyotrophic lateral sclerosis. It can be used to monitor functional changes in a patient over time. It measures (1) speech, (2) salivation, (3) swallowing, (4) handwriting, (5) cutting food and handling utensils (with or without gastrostomy), (6) dressing and hygiene, (7) turning in bed and adjusting bed clothes, (8) walking, (9) climbing stairs, and (10) breathing. Each task is rated on a 5-point scale from 0 = can't do, to 4 = normal ability. Individual item scores are summed to produce a reported total score of between 0 and 40 (worst to best). ALSFRS has been revised to ALSFRS-R, which includes 12 questions (ALSFRS Q10 changes to (10) Dyspnoea, (11) Orthopnea, and (12) Respiratory insufficiency), with individual item scores summed to a score between 0 and 48.

In order to eliminate any bias from the treatment effects on the ALSFRS, only the first observation at the time of trial is selected. If the first observation is missing at the time of trial (or prior), the next non-missing observation is selected to be included in the final analysis.

We subsequently mapped ALSFRS scores into GBD severities, and sequelae into different combinations of speech problems, chronic obstructive pulmonary disease, and motor impairment using the following logic:

Motor impairment

The ALSFRS assess motor function of the legs through questions on walking (Q8) and stair climbing (Q9).

Combined score	Severity level
8	None
5-7	Mild
2-4	Moderate
0-1	Severe

The ALSFRS also assesses motor impairment through questions on handwriting (Q4), cutting food and handling utensils (Q5), and dressing and hygiene (Q6).

Combined score	Severity level
12	None
9-11	Mild
3-8	Moderate
0-2	Severe

After determining case severity on these two separate metrics, we aggregate by taking the most severe ranking (eg, severe + mild = a severe case).

Respiratory problems:

Question 10 of the ALSFRS describes breathing difficulty as a function of MND.

ALSFRS score	Description	Severity level
4	Normal	None
3	Shortness of breath with minimal exertion	Mild
2	Shortness of breath at rest	Moderate
0-1	Intermittent ventilator assistance required/ventilator-dependent	Severe

Speech problems

Speech impairment due to MND is derived from ALSFRS question 1, which describes speech impediments. A score of 4 on this question denotes no impairment, while all other values suggest some impairment.

Creating sequelae

After determining the severity status of each case for the three symptom umbrellas, we subsequently estimated the relative proportion of each combination of symptom class and their respective severities. Those without any symptoms (eg, no severity) were categorised as having worry about the diagnosis for disability estimation. The following table displays the various sequelae and their associated proportions.

Sequela	Proportion (Mean)	Proportion (Lower)	Proportion (Upper)
Mild motor impairment, mild respiratory problems and speech problems due to motor neuron disease	0.01779	0.01658	0.01909
Mild motor impairment, moderate respiratory problems and speech problems due to motor neuron disease	0.00270	0.00225	0.00324
Mild motor impairment, severe respiratory problems and speech problems due to motor neuron disease	0.00082	0.00059	0.00113
Mild motor impairment, and speech problems due to motor neuron disease	0.02052	0.01922	0.02190
Moderate motor impairment, mild respiratory problems and speech problems due to motor neuron disease	0.03377	0.03210	0.03552
Moderate motor impairment, moderate respiratory problems and speech problems due to motor neuron disease	0.00715	0.00640	0.00799

Moderate motor impairment, severe respiratory problems and speech problems due to motor neuron disease	0.00286	0.00240	0.00342
Moderate motor impairment, and speech problems due to motor neuron disease	0.03041	0.02883	0.03208
Severe motor impairment, mild respiratory problems and speech problems due to motor neuron disease	0.05242	0.05035	0.05457
Severe motor impairment, moderate respiratory problems and speech problems due to motor neuron disease	0.02247	0.02111	0.02392
Severe motor impairment, severe respiratory problems and speech problems due to motor neuron disease	0.01365	0.01259	0.01479
Severe motor impairment and speech problems due to motor neuron disease	0.04765	0.04567	0.04970
Mild respiratory problems and speech problems due to motor neuron disease	0.01157	0.01060	0.01263
Moderate respiratory problems and speech problems due to motor neuron disease	0.00142	0.00111	0.00182
Severe respiratory problems and speech problems due to motor neuron disease	0.00023	0.00013	0.00043
Speech problems due to motor neuron disease	0.02457	0.02315	0.02608
Mild motor impairment and mild respiratory problems due to motor neuron disease	0.02245	0.02109	0.02389
Mild motor impairment and moderate respiratory problems due to motor neuron disease	0.00275	0.00230	0.00329
Mild motor impairment and severe respiratory problems due to motor neuron disease	0.00068	0.00047	0.00097
Mild motor impairment due to motor neuron disease	0.10388	0.10103	0.10681
Moderate motor impairment and mild respiratory problems due to motor neuron disease	0.06744	0.06511	0.06985
Moderate motor impairment and moderate respiratory problems due to motor neuron disease	0.01302	0.01199	0.01413
Moderate motor impairment and severe respiratory problems due to motor neuron disease	0.00412	0.00356	0.00477
Moderate motor impairment due to motor neuron disease	0.20136	0.19760	0.20518
Severe motor impairment and mild respiratory problems due to motor neuron disease	0.06902	0.06666	0.07146
Severe motor impairment and moderate respiratory problems due to motor neuron disease	0.02000	0.01872	0.02137
Severe motor impairment and severe respiratory problems due to motor neuron disease	0.01062	0.00969	0.01163
Severe motor impairment due to motor neuron disease	0.15037	0.14702	0.15378
Mild respiratory problems due to motor neuron disease	0.00643	0.00571	0.00723
Moderate respiratory problems due to motor neuron disease	0.00044	0.00028	0.00069
Severe respiratory problems due to motor neuron disease	0.00005	0.00001	0.00017
Asymptomatic, but worry about diagnosis due to motor neuron disease	0.03738	0.03562	0.03921

To determine disability due to these sequelae, we use the standard multiplicative aggregation formula as described in the main text. The following table provides description and disability weight assigned to the sequelae as appropriate.

Symptom group	Severity level	Lay description	DW (95%)
Respiratory problems	Asymptomatic		
Respiratory problems	Mild	Has cough and shortness of breath after heavy physical activity, but is able to walk long distances and climb stairs.	0.019 (0.011–0.033)
Respiratory problems	Moderate	Has cough, wheezing, and shortness of breath, even after light physical activity. The person feels tired and can walk only short distances or climb only a few stairs.	0.225 (0.153–0.31)
Respiratory problems	Severe	Has cough, wheezing, and shortness of breath all the time. The person has great difficulty walking even short distances or climbing any stairs, feels tired when at rest, and is anxious.	0.408 (0.273–0.556)
Motor impairment	Asymptomatic		
Motor impairment	Mild	Has some difficulty in moving around but is able to walk without help.	0.01 (0.005–0.019)
Motor impairment	Moderate	Has some difficulty in moving around and difficulty in lifting and holding objects, dressing, and sitting upright, but is able to walk without help.	0.061 (0.04–0.089)
Motor impairment	Severe	Is unable to move around without help, and is not able to lift or hold objects, get dressed, or sit upright.	0.402 (0.268–0.545)
Speech problems	No		
Speech problems	Yes	Has difficulty speaking, and others find it difficult to understand.	0.051 (0.032–0.078)
Asymptomatic, but worry	Yes	Has a disease diagnosis that causes some worry but minimal interference with daily activities.	0.012 (0.006–0.023)

[1] Vos T, Lim SS, Abbafati C, *et al.* Global burden of 369 diseases and injuries in 204 countries and territories, 1990–2019: a systematic analysis for the Global Burden of Disease Study 2019. *The Lancet* 2020; 396: 1204–22. doi: https://doi.org/10.1016/S0140-6736(20)30925-9

Multiple sclerosis (MS)

Flowchart

Multiple sclerosis

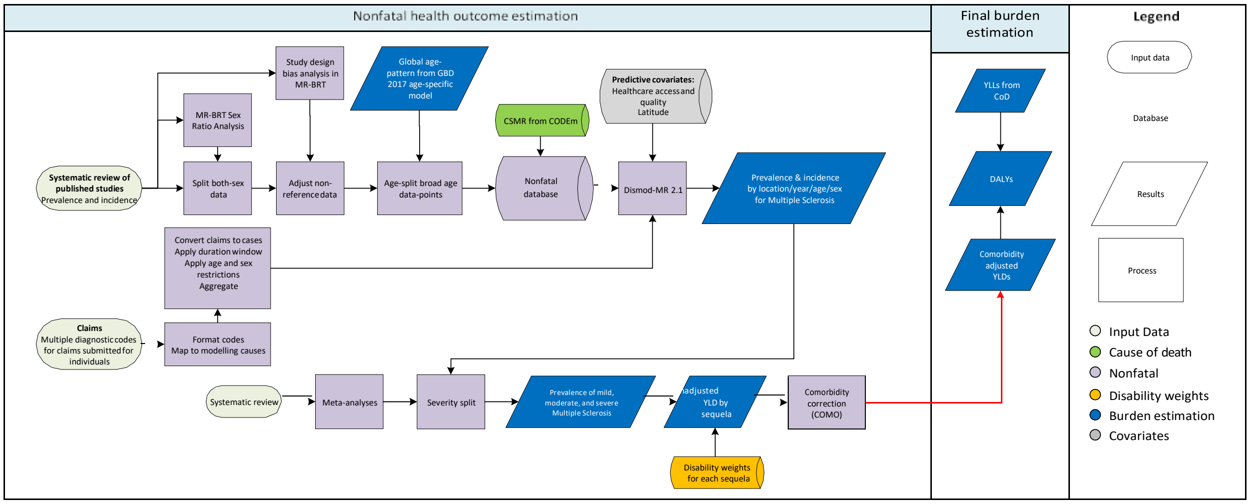

Input data and methodological summary

Case definition

Multiple sclerosis is a chronic, degenerative, and progressive neurological condition typified by damage by the immune system to the myelin sheaths surrounding neurons in the brain and spinal cord. McDonald's criteria for diagnosis are considered the contemporary gold standard. For GBD 2021, as for previous rounds, diagnosis by McDonald's criteria, other published criteria (such as Poser, Schumacher, or McAllen criteria), and clinical neurological exam are all treated as reference. The ICD-10 code for MS is G35.

Input data and processing

The data underpinning estimates of burden due to MS are generally of two types. The first are representative, population-based, cross-sectional or longitudinal studies reported in peer-reviewed journals and identified via a search-string-based review, last updated for GBD 2017 and described in previous reports. Estimates of epidemiological measures (prevalence, incidence, *etc.*) were manually extracted from these publications. The second type are claims data as obtained and processed by the GBD Clinical Informatics team and described in a separate section of this appendix. New data added in GBD 2021 included Polish claims (2017–2018), and an additional year of USA claims (2017). These data link claims for all inpatient and outpatient encounters for a single individual, and provide primary and secondary diagnoses for all encounters. An individual was extracted from claims data as a prevalent case if they had any peptic ulcer disease code as any diagnosis in one or more inpatient encounters or two or more outpatient encounters.

The total number of sources used for modelling in GBD 2021 are listed in the table below:

Measure	Total sources	Countries with data
All measures	253	53
Prevalence	211	46
Incidence	86	24
Remission	0	0
Other	29	20

For studies that reported epidemiological measures (generally prevalence or incidence) by age for both sexes combined, and also by sex for all ages combined, we calculated the sex ratio of cases in that study and applied it to the age-specific measures to estimate age-sex-specific measures.

To estimate sex-specific measures from studies that reported only for both sexes combined, we modelled the log sex ratio in MR-BRT (meta-regression—Bayesian, regularised, trimmed) model[1] (Additional information can be found in appendix 1, section 4.4.1 of the cited paper) using all sex-specific measurements from all other studies in the database and combined these with the GBD sex-specific population estimates for the relevant age-group. For prevalence, this estimate was 0.63 (0.069 to 1.2); for incidence this estimate was 0.86 (0.53 to 1.2). These were applied by calculating male prevalence:

$$prev_{male} = prev_{both} * \frac{pop_{both}}{(pop_{male} + ratio * pop_{female})}$$

and then calculating female prevalence:

$$prev_{female} = ratio * prev_{male}$$

(Equivalent equations were used for incidence.)

A pre-modelling bias adjustment was then made to data from USA claims in the year 2000—a dataset that only covers a small commercially insured sub-population. This adjustment was modelled as difference in logit prevalence between USA claims data and reference data matched on year, age, sex and location. The estimated mean logit differences were applied to the USA claims data for 2000 prior to modelling in DisMod-MR 2.1[1] (disease model—Bayesian meta-regression, details on this method can be found in appendix 1, section 4.5 of the citation) (below).

The process of adjusting for non-reference data using MR-BRT with the logit-transformation method is described below:

1. Identify datapoints with overlapping year, age, sex, and location between claims (alternative case definition) and other (reference case definition)
2. Logit transform overlapping datapoints of alternative and reference case definitions
3. Convert overlapping datapoints into a difference in logit space using the following equation: $logit(altnerative) - logit(reference)$

4. Use the delta method to compute standard errors of overlapping datapoints in logit space, then calculate standard error of logit difference using the following equation:
   $\sqrt{(variance\ of\ alternative) + (variance\ of\ reference)}$
5. Using MR-BRT, conduct a random effects meta-regression to obtain the pooled logit difference of alternative to reference
6. Apply the pooled logit difference to all datapoints of alternative case definitions using the following equation:
   $$new_{estimate} = inverse.\,logit((logit(alternative)) - (pooled\ logit\ difference))$$
7. Calculate new standard errors using the delta method, accounting for gamma (between-study heterogeneity)

The table below shows bias correction factors.

MR-BRT Crosswalk Adjustment Factors for Multiple sclerosis

Data input	Reference or alternative data	Gamma	Beta Coefficient, Logit difference (95% CI)*	Adjustment factor**
McDonald's diagnostic criteria<br>OR<br>Other published diagnostic criteria<br>OR<br>Clinical neuro exam<br>OR<br>Claims for location-years other than USA 2000	Reference	0.32	---	---
Data from USA claims in 2000	Alternative		-0.57 (-1.79 to 0.62)	0.36 (0.14 to 0.65)

**MR-BRT crosswalk adjustments can be interpreted as the factor the alternative case definition is adjusted by to reflect what it would have been had it been measured using the reference case definition. If the log/logit beta coefficient is negative, then the alternative is adjusted up to the reference. If the log/logit beta coefficient is positive, then the alternative is adjusted down to the reference.*
***The adjustment factor column is the exponentiated beta coefficient. For log beta coefficients, this is the relative rate between the two case definitions. For logit beta coefficients, this is the relative odds between the two case definitions.*

Subsequently, datapoints for samples spanning 25 years of age or more were disaggregated into 5-year age groups by applying the age-pattern observed in the global fit for the GBD 2019 best model for Multiple sclerosis.
After extraction and processing, some studies were marked as outliers and excluded on a case-by-case basis if they were inconsistent with established regional or temporal trends or if concerns about study quality were identified during extraction and processing.

Modelling strategy

Compartmental model
We used DisMod-MR 2.1 as the main analytical tool for the MS estimation process. Inputs included

prevalence and incidence data, as described above, as well as the cause-specific mortality rate (CSMR) estimated in the GBD causes of death analysis, and excess mortality rate (EMR) obtained by dividing CSMR by prevalence datapoints. Prior settings included zero remission for all ages, no incidence or excess mortality for persons under 5 years old, and incidence limited to less than 0.000005 after the age of 60 years. We also constrained the random effects for prevalence, incidence, and excess mortality to a minimum of -1 and a maximum of 1 for all locations except Greenland, United States, and Canada, where location random effects for incidence were constrained to -6, 3 and 3, respectively and Albania whose location random effect was constrained between a minimum of -0.25 and a maximum of 2.

We employed the following covariates to improve model predictions:

Covariate	Measure	Beta Coefficient, Log (95% CI)	Adjustment factor**
Absolute value of average latitude	prevalence	0.032 (0.022 to 0.035)	1.03 (1.02 to 1.04)
Absolute value of average latitude	incidence	0.018 (0.012 to 0.023)	1.02 (1.01 to 1.02)
Healthcare Access and Quality Index	excess mortality rate	-0.036 (-0.042 to -0.034)	0.97 (0.96 to 0.97)

**MR-BRT crosswalk adjustments can be interpreted as the factor the alternative case definition is adjusted by to reflect what it would have been had it been measured using the reference case definition. If the log/logit beta coefficient is negative, then the alternative is adjusted up to the reference. If the log/logit beta coefficient is positive, then the alternative is adjusted down to the reference.*

***The adjustment factor column is the exponentiated beta coefficient. For log beta coefficients, this is the relative rate between the two case definitions. For logit beta coefficients, this is the relative odds between the two case definitions.*

As described in the literature, extreme latitude is associated with higher prevalence and incidence of MS, although the pathway to explain the association is not understood. Our operationalisation of latitude is created by a population-weighted average of latitude by country and taking the absolute value. The underlying population distribution rasters are part of the Gridded Population of the World dataset.

Although there are no known cures for MS, we expect disease management to differ globally – largely as a function of available resources. To capture this, we use the Healthcare Access and Quality Index covariate to capture this relationship in the estimation of excess mortality.

Severity splits

As we have done since GBD 2013, we used Kurtzke's Expanded Disability Status Scale (EDSS) to determine severity splits for MS. The EDSS scores corresponding to each severity are as follows:

Asymptomatic: EDSS = 0
Mild: 0 < EDSS ≤ 3.5
Moderate: 3.5 < EDSS ≤ 6.5
Severe: 6.5 < EDSS ≤ 9.5

The table below illustrates severity levels, lay descriptions, and DWs.

Severity level	Lay description	DW (95% CI)
Asymptomatic	-	0<br>(0-0)
Mild	Has mild loss of feeling in one hand, is a little unsteady while walking, has slight loss of vision in one eye, and often needs to urinate urgently.	0.183<br>(0.124–0.253)
Moderate	Needs help walking, has difficulty with writing and arm coordination, has loss of vision in one eye and cannot control urinating.	0.463<br>(0.313–0.613)
Severe	Has slurred speech and difficulty swallowing. The person has weak arms and hands, very limited and stiff leg movement, has loss of vision in both eyes and cannot control urinating.	0.719<br>(0.534–0.858)

Because not all sources had information on the number of cases with EDSS stage 0, instead reporting on a mild category, we implemented a two-step meta-analysis strategy. First, we subsetted the studies to those that reported on the number of cases with EDSS stage 0, and did meta-analyses on the proportion of asymptomatic and mild cases. Then, we conducted meta-analyses on the full dataset to get the proportion mild, moderate, and severe, and we squeezed the asymptomatic and mild categories from the previous meta-analyses into the mild category established by the meta-analysis on the full dataset.

The following figures provide the result of the first meta-analysis on the asymptomatic and mild categories.

Figure 1. Asymptomatic cases of MS

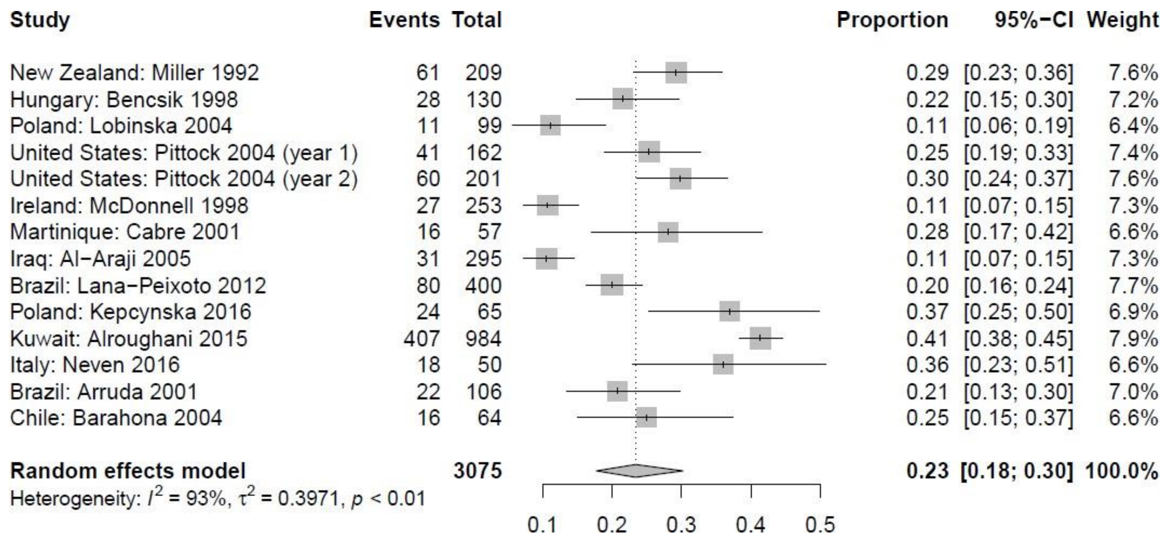

Figure 2. Mild cases of MS

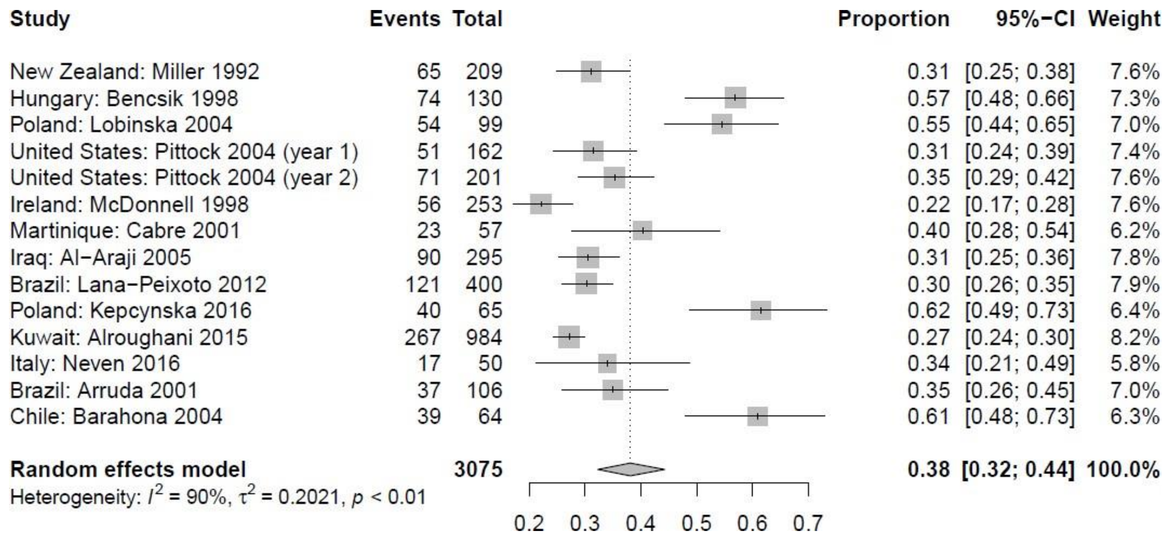

The following figures provide the result of the second meta-analysis on the mild, moderate, and severe categories.

Figure 3. Mild cases of MS (including both asymptomatic and mild categories)

Study	Events	Total	Proportion	95%-CI	Weight
Ita ly: Casetta 1998	221	394	0.56	[0.51; 0.61)	3.6%
**Japan: Houzen 2003**	20	31	0.65	(0.45; 0.81)	2.9%
**Hungary: Bencsik 2001**	119	**248**	0.48	[0.42; 0.54)	**3.5%**
Spain: Arribas 1 999	31	54	0.57	[0 .43 ; 0.71)	3 .2%
Spain: Pardo 1997	26	43	0.60	[0.44; 0.75]	**3.1%**
**Taiwan: Tsai 2004**	27	41	0.66	[0.49; 0.80)	**3.1%**
**Brazil: da Silva 2016**	**84**	208	0.40	[0.34; 0.47)	**3.5%**
**Romania: Maier 2016**	258	351	0.74	[0.69; 0.78)	**3.5%**
**Iran: Torabipour 2014**	282	332	0.85	[0.81; 0.89)	**3.5%**
Iran: Ayatolla hi 2013	41	51	0.80	[0.67; 0.90)	3.0%
**Nether1and s: Karampampa2013**	121	263	0.46	(0.40; 0.52)	**3.5%**
**Iran: Harand 2012**	47	78	0.60	[0.49; 0.71)	**3.3%**
France: Kobelt 2009	529	1334	0.40	[0.37; 0.42)	3.6%
**New Zealand: Miller 1992**	126	209	0.60	[0.53; 0.67)	**3.5%**
Hunga ry: Bencsik 1998	102	130	0. 78	[0.70 ; 0 . 8 5]	3 . 4%
**Poland: Lobinska 2004**	65	99	0.66	[0.55; 0.75]	**3.4%**
United States: Pittock **2004** (yea r 1)	92	162	0.57	[0.49; 0.65]	**3.5%**
United States: Pittock **2004** (yea r 2)	131	201	0.65	[0.58; 0.72)	**3.5%**
Ire land: McDonnell 1998	83	**253** -	0.33	[0.27; 0.39)	**3.5%**
**Martinique: Cabre 2001**	39	57	0.68	[0.55; 0.80)	3.2%
Iraq: AI- Araji 2005	121	295	0.41	[0.35; 0.47)	3.6%
**Brazil:Lana-Peixoto2012**	201	400	0.50	(0.45; 0.55]	3.6%
Poland: Kepcynska 2016	64	65	0.98	[0.92; 1.00]	**1.4%**
**Kuwait: Alroughani 2015**	674	984	0.68	[0.65; 0.71)	3.6%
Italy: Neven 2016	35	50	0.70	[0.55; 0.82)	**3.1%**
Brazil: Arruda 2001	59	106	0.56	[0.46; 0.65]	3.4%
**Ch.ile : Ba rahona 2004**	55	64	0.86	[0.75; 0.93)	3.0%
**Turkey: Akdemir 2017**	1339	1584	0.85	[0.83; 0.86]	3.6%
Zaka ria: Egypt2016	518	950	0.55	[0.51; 0.58)	3.6%
**Pearson: New Zealand2016**	597	1727	0.35	[0.32; 0.37)	3.6%
**Randomeffects model**		10764	0.62	[0.55; 0.69)	100.0%

Heterogeneity: ,2 =98%. 1 =0.6023, $p < 0.01$

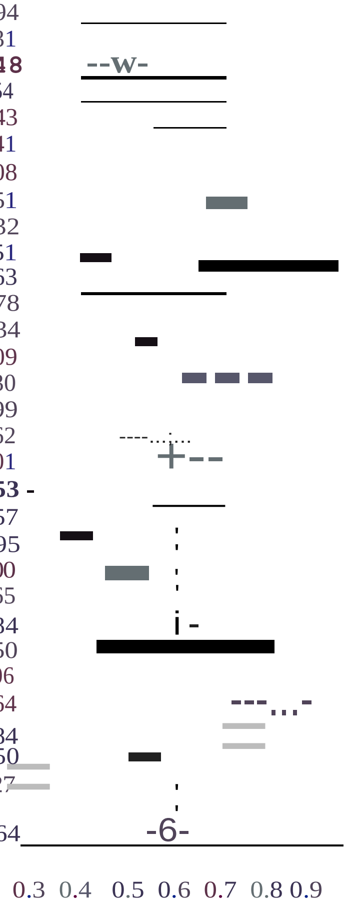

Figure 4. Moderate cases of MS

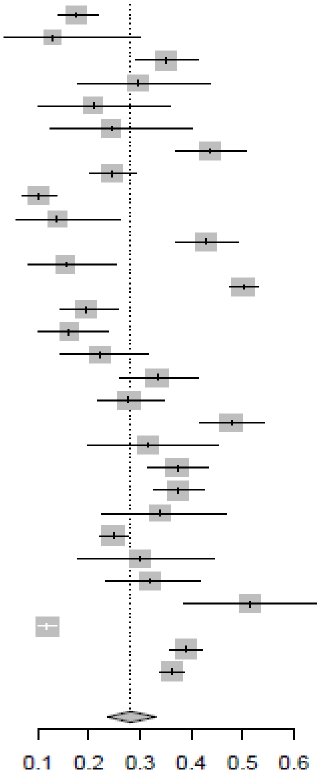

Study	Events	Total	Proportion	95%-CI	Weight
Italy: Casetta 1998	69	394	0.18	[0.14; 0.22]	3.6%
Japan: Houzen 2003	4	31	0.13	[0.04; 0.30]	2.1%
Hungary: Bencsik 2001	87	248	0.35	[0.29; 0.41]	3.6%
Spain: Arribas 1999	16	54	0.30	[0.18; 0.44]	3.0%
Spain: Pardo 1997	9	43	0.21	[0.10; 0.36]	2.7%
Taiwan: Tsai 2004	10	41	0.24	[0.12; 0.40]	2.8%
Brazil: da Silva 2016	91	208	0.44	[0.37; 0.51]	3.6%
Romania: Maier 2016	86	351	0.25	[0.20; 0.29]	3.6%
Iran: Torabipour 2014	33	332	0.10	[0.07; 0.14]	3.4%
Iran: Ayatollahi 2013	7	51	0.14	[0.06; 0.26]	2.6%
Netherlands: Karampampa 2013	113	263	0.43	[0.37; 0.49]	3.6%
Iran: Harandi 2012	12	78	0.15	[0.08; 0.25]	3.0%
France: Kobelt 2009	669	1334	0.50	[0.47; 0.53]	3.7%
New Zealand: Miller 1992	41	209	0.20	[0.14; 0.26]	3.5%
Hungary: Bencsik 1998	21	130	0.16	[0.10; 0.24]	3.3%
Poland: Lobinska 2004	22	99	0.22	[0.14; 0.32]	3.3%
United States: Pittock 2004 (year 1)	54	162	0.33	[0.26; 0.41]	3.5%
United States: Pittock 2004 (year 2)	56	201	0.28	[0.22; 0.35]	3.5%
Ireland: McDonnell 1998	121	253	0.48	[0.42; 0.54]	3.6%
Martinique: Cabre 2001	18	57	0.32	[0.20; 0.45]	3.1%
Iraq: Al−Araji 2005	110	295	0.37	[0.32; 0.43]	3.6%
Brazil: Lana−Peixoto 2012	150	400	0.38	[0.33; 0.42]	3.7%
Poland: Kepcynska 2016	22	65	0.34	[0.23; 0.47]	3.2%
Kuwait: Alroughani 2015	244	984	0.25	[0.22; 0.28]	3.7%
Italy: Neven 2016	15	50	0.30	[0.18; 0.45]	3.0%
Brazil: Arruda 2001	34	106	0.32	[0.23; 0.42]	3.4%
Chile: Barahona 2004	33	64	0.52	[0.39; 0.64]	3.2%
Turkey: Akdemir 2017	187	1584	0.12	[0.10; 0.13]	3.7%
Zakaria: Egypt 2016	370	950	0.39	[0.36; 0.42]	3.7%
Pearson: New Zealand 2016	625	1727	0.36	[0.34; 0.39]	3.7%
**Random effects model**		**10764**	**0.28**	**[0.24; 0.33]**	**100.0%**

Heterogeneity: $I^2 = 96\%$, $\tau^2 = 0.3752$, $p < 0.01$

Figure 5. Severe cases of MS

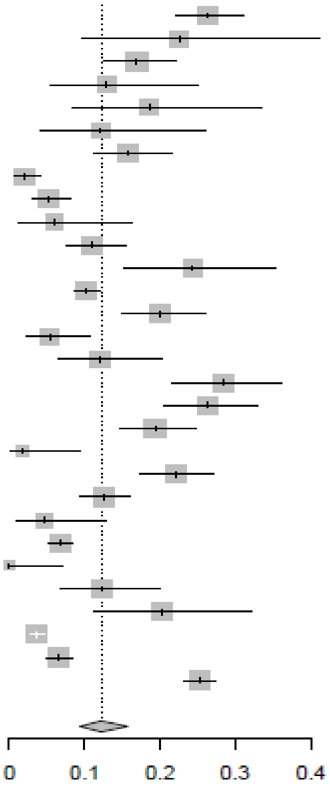

Study	Events	Total	Proportion	95%-CI	Weight
Italy: Casetta 1998	104	394	0.26	[0.22; 0.31]	3.9%
Japan: Houzen 2003	7	31	0.23	[0.10; 0.41]	3.0%
Hungary: Bencsik 2001	42	248	0.17	[0.12; 0.22]	3.8%
Spain: Arribas 1999	7	54	0.13	[0.05; 0.25]	3.1%
Spain: Pardo 1997	8	43	0.19	[0.08; 0.33]	3.1%
Taiwan: Tsai 2004	5	41	0.12	[0.04; 0.26]	2.8%
Brazil: da Silva 2016	33	208	0.16	[0.11; 0.22]	3.7%
Romania: Maier 2016	7	351	0.02	[0.01; 0.04]	3.1%
Iran: Torabipour 2014	17	332	0.05	[0.03; 0.08]	3.6%
Iran: Ayatollahi 2013	3	51	0.06	[0.01; 0.16]	2.4%
Netherlands: Karampampa 2013	29	263	0.11	[0.08; 0.15]	3.7%
Iran: Harandi 2012	19	78	0.24	[0.15; 0.35]	3.5%
France: Kobelt 2009	136	1334	0.10	[0.09; 0.12]	3.9%
New Zealand: Miller 1992	42	209	0.20	[0.15; 0.26]	3.8%
Hungary: Bencsik 1998	7	130	0.05	[0.02; 0.11]	3.1%
Poland: Lobinska 2004	12	99	0.12	[0.06; 0.20]	3.4%
United States: Pittock 2004 (year 1)	46	162	0.28	[0.22; 0.36]	3.8%
United States: Pittock 2004 (year 2)	53	201	0.26	[0.20; 0.33]	3.8%
Ireland: McDonnell 1998	49	253	0.19	[0.15; 0.25]	3.8%
Martinique: Cabre 2001	1	57	0.02	[0.00; 0.09]	1.4%
Iraq: Al−Araji 2005	65	295	0.22	[0.17; 0.27]	3.8%
Brazil: Lana−Peixoto 2012	50	400	0.12	[0.09; 0.16]	3.8%
Poland: Kepcynska 2016	3	65	0.05	[0.01; 0.13]	2.4%
Kuwait: Alroughani 2015	66	984	0.07	[0.05; 0.08]	3.9%
Italy: Neven 2016	0	50	0.00	[0.00; 0.07]	0.9%
Brazil: Arruda 2001	13	106	0.12	[0.07; 0.20]	3.4%
Chile: Barahona 2004	13	64	0.20	[0.11; 0.32]	3.4%
Turkey: Akdemir 2017	58	1584	0.04	[0.03; 0.05]	3.8%
Zakaria: Egypt 2016	62	950	0.07	[0.05; 0.08]	3.9%
Pearson: New Zealand 2016	436	1727	0.25	[0.23; 0.27]	4.0%
**Random effects model**		**10764**	**0.12**	**[0.09; 0.16]**	**100.0%**

Heterogeneity: $I^2 = 95\%$, $\tau^2 = 0.5542$, $p < 0.01$

[1] Vos T, Lim SS, Abbafati C, *et al.* Global burden of 369 diseases and injuries in 204 countries and territories, 1990–2019: a systematic analysis for the Global Burden of Disease Study 2019. *The Lancet* 2020; 396: 1204–22. doi: https://doi.org/10.1016/S0140-6736(20)30925-9

Acute myocarditis

Flowchart

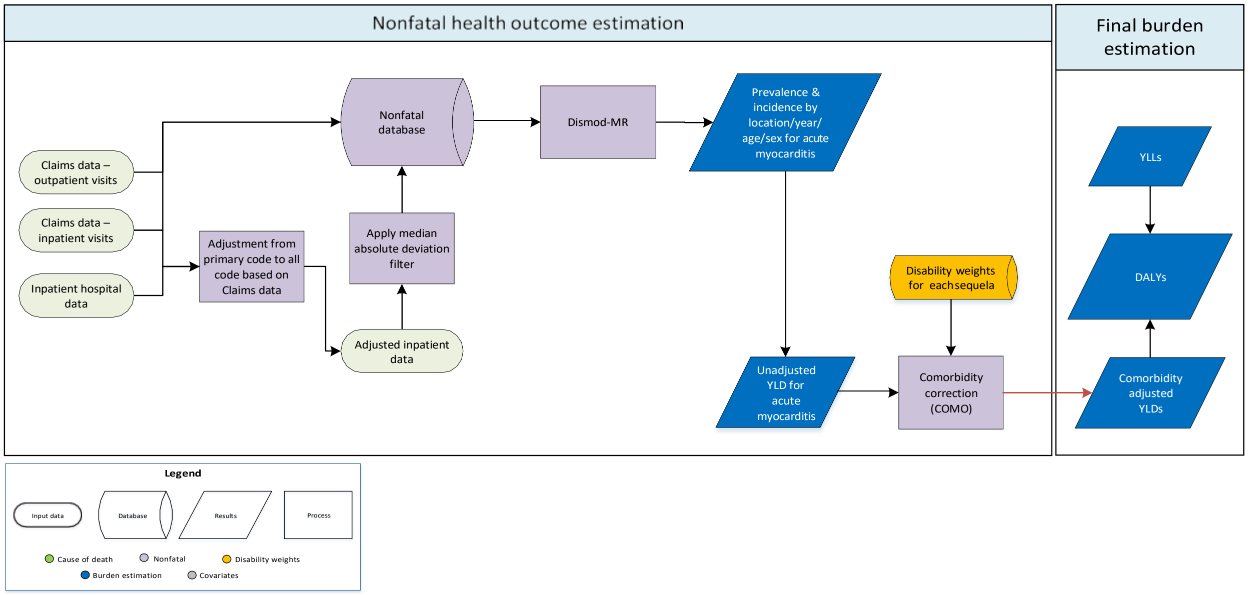

Input data and methodological summary

Case definition

Myocarditis is inflammation of the myocardium or middle layer of the heart wall muscles. It can be caused by viral infections, autoimmune conditions, and other non-ischaemic causes and can result in reduced ability of the heart to pump blood to the body. Acute myocarditis was defined for GBD as the acute and time-limited symptoms of myocarditis separate from its chronic heart-failure-related sequelae. Heart failure due to myocarditis is estimated separately in GBD (see methods for heart failure). Symptoms of acute myocarditis can be nonspecific and include a flu-like or gastrointestinal syndrome, followed by anginal-type chest pain, arrhythmias, syncope, or heart failure.

The ICD codes included for myocarditis are B33.2, I40–I41.9, I51.4 for ICD-10 and 422–422.9 for ICD-9.

Input data

Model inputs

The preferred data sources for acute myocarditis were hospital admission data and other health facility data identifying cases of acute myocarditis. We have performed a systematic review of myocarditis in past cycles of GBD (GBD 2013 – see below) and found no sources that matched our criteria for modelling of the disease. As a result, we currently only use hospital admission incidence data to estimate acute myocarditis incidence and prevalence. Table 1 shows the source counts for acute myocarditis.

Table 1: Source counts for acute myocarditis

Measure	Total sources	Countries with data
Incidence	315	44

A systematic review was performed for GBD 2013 and updated for GBD 2015. A systematic review was not performed for GBD 2021.

The GBD 2015 search terms included (cardiomyopathy AND epidemiology [MeSH Subheading]) OR (myocarditis AND epidemiology [MeSH Subheading]) OR (cardiomyopathy AND (incidence OR prevalence OR "case fatality")) OR (myocarditis AND (incidence OR prevalence OR "case fatality"))

- Dates included in search: 1/1/2013–3/16/2015
- Number of initial hits: 3598
- Number of sources included: 0

The GBD 2013 search terms included: (hasabstract[text] AND Humans[MeSH] AND middle age[MeSH])) OR 21) AND ((cardiomyopathy/epidemiology[MeSH] OR cardiomyopathy/mortality[MeSH]) AND (prevalence[Title/Abstract] OR incidence[Title/Abstract]) AND ("2010"[Date - Publication] : "3000"[Date - Publication]) AND (hasabstract[text] AND Humans[MeSH] AND middle age[MeSH]))

We used inpatient hospital data adjusted for readmission, primary to any diagnosis, and inpatient to outpatient utilisation based on correction factors generated using USA claims data. More information on how correction factors were made for this adjustment can be found in the "Claims data" section of the non-fatal appendix. We excluded all outpatient data, as they were implausibly low when compared with inpatient data from the same locations and with claims data. Inpatient hospital datapoints that were more than two-fold higher or 0.5-fold lower than the median absolute deviation[1] value for high-income North America, central Europe, and western Europe for that age-sex group were excluded.

Severity splits and disability weights

Table 2: Details on the severity levels for acute myocarditis in GBD 2021 and the associated disability weight with that severity

Severity level	Lay description	Disability weight (95% CI)
Acute myocarditis	Has a fever and aches, and feels weak, which causes some difficulty with daily activities.	0.051 (0.032–0.074)

Modelling strategy

For GBD 2021, we estimated acute myocarditis using a DisMod-MR Bayesian meta-regression model, setting a minimum of 3 and maximum of 5 as value priors on remission to establish an average duration

of three months. We set a value prior of 0 for all ages on excess mortality. For GBD 2021, the only country-level covariate used was Healthcare Access and Quality (HAQ) Index on excess mortality.

Table 3 below gives the parameters, betas, and exponentiated betas for study-level and country-level covariates used in the model.

Table 3: Summary of covariates used in the acute myocarditis DisMod-MR meta-regression model

Study covariate	Parameter	Beta	Exponentiated beta
HAQ Index	Excess mortality rate	–0.56 (–0.98 to –0.12)	0.57 (0.37–0.89)

No substantive changes were made to the modelling approach for GBD 2021.

References

[1] Huber, P.J. (2011). Robust Statistics. In: Lovric, M. (eds) *International Encyclopedia of Statistical Science*. Springer, Berlin, Heidelberg. https://doi.org/10.1007/978-3-642-04898-2_594

Non-alcoholic fatty liver disease without cirrhosis

Flowchart

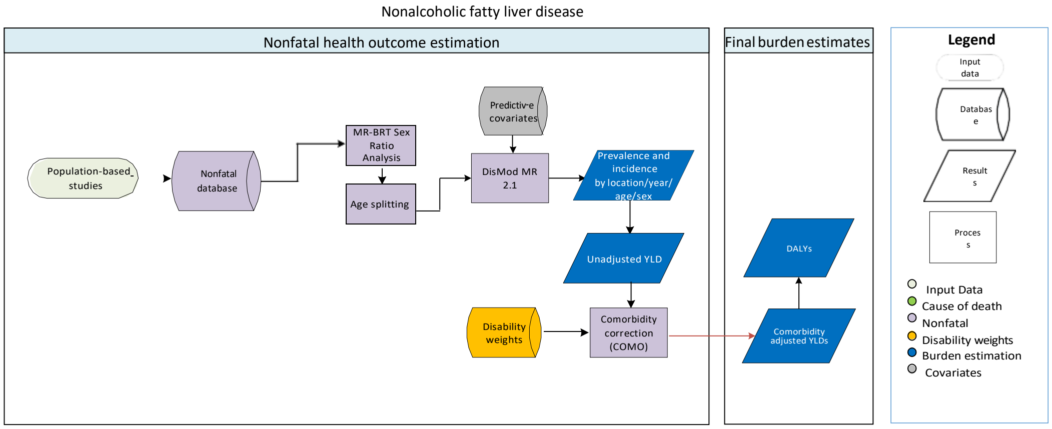

Input data and methodological summary for non-alcoholic fatty liver disease

Case definition

Non-alcoholic fatty liver disease (NAFLD) encompasses the spectrum of non-alcoholic fatty liver disease including fat deposition without cirrhosis and cirrhosis (ie, scarring of the liver) that can result from longstanding and progressive fat deposition and inflammation.

NAFLD without cirrhosis includes all degrees of NAFLD that have not progressed to cirrhosis, although we refer to it simply as “NAFLD” in this appendix section. Modelling details of cirrhosis due to NAFLD can be found in the “Cirrhosis” section of this appendix.

A consensus statement was published by experts in the field of hepatology in June 2023, revising the nomenclature for this cause of disease to metabolic dysfunction-associated steatosis of the liver (MASLD).[1] This consensus was adopted after the GBD 2021 analysis concluded and we continue to refer to this cause of disease as “NAFLD” for the GBD 2021 study report. The updated name will be proposed to the GBD Scientific Council to consider for adoption in the next GBD iteration.

Input data

We use population-based studies that report the prevalence of NAFLD. The following inclusion criteria were used:

(1) Sample size greater than 100.
(2) Sample representative of general population for location.
(3) Sufficient description of methods to assess study quality.
(4) Does not exclude comorbidities.
(5) NAFLD diagnosed by ultrasound (USS) or other diagnostic imaging modality.

The last systematic review was performed for GBD 2017, using the search string below.

("Steatohepatitides"[Title/Abstract]) OR ("NAFLD"[Title/Abstract] OR "NAFL"[Title/Abstract] OR "NASH"[Title/Abstract] OR)) AND ("prevalence"[Title/Abstract] OR "incidence"[Title/Abstract) AND ("1990/01/01"[PDAT] : "2017/07/26"[PDAT]) NOT (animals[MeSH] NOT humans[MeSH]))

Table 20: Data inputs for NAFLD

Measure	Total sources	New sources	Countries with data
Prevalence	52	0	17
Other	35	11	19

Although biopsy provides the gold-standard clinical case definition, this invasive procedure is not typically employed in population-based surveys or screening programmes. In consultation with GI experts, we thus chose ultrasound or other imaging study as our reference case diagnostics. We excluded any studies using

[1] Rinella ME, Lazarus JV, Ratziu V, Francque SM, Sanyal AJ, Kanwal F, Romero D, Abdelmalek MF, Anstee QM, Arab JP, Arrese M, Bataller R, Beuers U, Boursier J, Bugianesi E, Byrne C, Castro Narro GE, Chowdhury A, Cortez-Pinto H, Cryer D, Cusi K, El-Kassas M, Klein S, Eskridge W, Fan J, Gawrieh S, Guy CD, Harrison SA, Kim SU, Koot B, Korenjak M, Kowdley K, Lacaille F, Loomba R, Mitchell-Thain R, Morgan TR, Powell E, Roden M, Romero-Gómez M, Silva M, Singh SP, Sookoian SC, Spearman CW, Tiniakos D, Valenti L, Vos MB, Wong VW, Xanthakos S, Yilmaz Y, Younossi Z, Hobbs A, Villota-Rivas M, Newsome PN; NAFLD Nomenclature consensus group. A multi-society Delphi consensus statement on new fatty liver disease nomenclature. *Hepatology*. 2023 Jun 24. doi: 10.1097/HEP.0000000000000520. Epub ahead of print. PMID: 37363821.

serum diagnostics or fatty liver indexes and scores to diagnose NAFLD. Studies were excluded if they ascertained cases only among patients with GI distress or in specialty outpatient clinics, or if they excluded patients with comorbidities.

Since the majority of NAFLD cases are asymptomatic, we generally preferred studies with active case-finding methods and did not make use of administrative data from hospitals or claims, which severely underestimate NAFLD prevalence. An exception to this is that we accepted Asian studies pooling data from general checkups, where participation in checkups is high and ultrasound is a part of the checkup regimen (eg, South Korea, Japan, and some parts of China). Data were marked as outliers and excluded if we found they differed substantially when compared to regional, super-regional, and global rates.

Data processing

Because we produce sex-specific estimates, we adjusted data that reported on both sexes into male and female sex-specific estimates. We identified studies that reported sex-specific prevalence and calculated the log ratio of female to male prevalence, then modelled these log ratios in a meta-regression tool, meta-regression—Bayesian, regularised, trimmed (MR-BRT). We then used the modelled sex ratio to adjust "both"-sex data values to expected "male" and "female" values. We calculated the male values as $val_{male} = val_{both} * \frac{pop_{both}}{(pop_{male} + ratio * pop_{female})}$. We calculated female values $val_{female} = ratio * val_{male}$.

We adjusted broad age-group data into five-year age bins using an estimated age pattern. Data in which the age range was greater than 25 years was categorised as broad age-range data. We assumed the age distribution in the study sample was the same as the estimated population in GBD 2017. We also assumed that the ratios of age-specific prevalence to full-age prevalence was the same as the model from GBD 2017.

Modelling strategy

DisMod model

We made no changes to the modelling strategy from GBD 2019. We modelled prevalence and incidence of NAFLD using DisMod-MR 2.1. Prior settings include zero incidence from age 0 to 5, excess mortality bound between 0 and 1 for all ages, and remission bound between 0 and 1 for all ages.

Several factors known to be associated with NAFLD prevalence in prior studies, for which we have prevalence estimates available for all GBD year-age-sex-location combinations, were employed as predictive covariates. Associations between predictive covariates and NAFLD prevalence for year-age-sex-location combinations with NAFLD prevalence data are used to help predict NAFLD prevalence for year-age-sex-location combinations with few or no data. A table of predictive covariates and their coefficients is shown below.

Summary of covariates used in the NAFLD DisMod-MR meta-regression model

Covariate	Parameter	Exponentiated beta

		(95% uncertainty interval)
Mean BMI	Prevalence	1.16 (1.11–1.20)
Prevalence of obesity	Prevalence	1.61 (0.46–6.25)
Age-standardised SEV* for high fasting plasma glucose	Prevalence	2.72 (2.49–2.97)

*Estimation of scaled exposure variables (SEVs) is described in a separate appendix section

Adjustment

In GBD 2017, we evaluated how different study definitions of alcohol consumption influenced NAFLD estimates in the regression tool. We compared alternative definitions to the most frequently used definition: 70 grams and 140 grams per week for men and women, respectively. The effect of these adjustments were insignificant and dropped in the final model.

We performed a post-hoc adjustment of estimates from DisMod to account for study exclusions of individuals with high alcohol consumption. The study samples often reflect the prevalence in low- or non-consumers of alcohol, not a general population, so we adjust final estimates. We multiplied location-year-sex-age-specific prevalence estimates from the NAFLD DisMod model by the proportion of the general population that consumes <70 g (female) and <140 g (male) of alcohol per week to approximate data for the general population. This proportion is estimated by the alcohol risk factor team and is year, age, sex, and location specific.

Disability weights

Cases of NAFLD without cirrhosis are asymptomatic and assigned a disability weight of zero.

Neonatal disorders

Morbidity due to neonatal disorders is modelled as five individual causes: neonatal preterm birth complications, neonatal encephalopathy due to birth asphyxia and trauma, neonatal sepsis and other neonatal infections, haemolytic disease and other neonatal jaundice, and other neonatal disorders. Each cause is modelled separately due to differences in data availability and pathology, though many input data types and modelling approaches are shared across the causes.

Neck pain

Flowchart

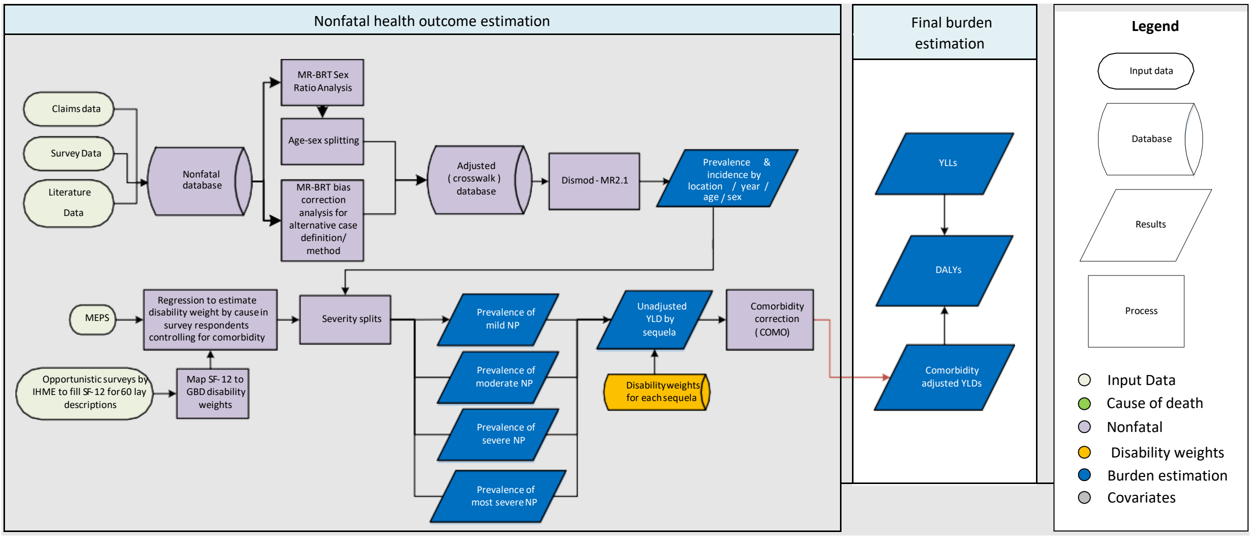

Input data and methodological summary for neck pain

Case definition

The list of accepted neck pain definitions is found below.

Reference or alternative	Case definition
Reference	current neck pain (+/- pain referred into the upper limb(s)) that lasts for at least one day. This includes Taiwan claims data, which align with the reference definition
Alternative	current pain (+/- pain referred into the upper limb(s)) that includes the neck in addition to a broader anatomical region (eg, neck and back) that lasts for at least one day
Alternative	current neck pain (+/- pain referred into the upper limb(s)) that lasts for at least 3 months (chronic)
Alternative	current neck pain (+/- pain referred into the upper limb(s)) that lasts for at least one day in the last 1 week to 1 month
Alternative	current neck pain (+/- pain referred into the upper limb(s)) that lasts for at least one day in the last 2 months to 1 year
Alternative	current neck pain (+/- pain referred into the upper limb(s)) among a study population of schoolchildren that lasts for at least one day
Alternative	current neck pain (+/- pain referred into the upper limb(s)) that lasts for at least one day and is activity-limiting
Alternative	USA claims data

The ICD-10 code for neck pain is M54.2. The ICD-9 code is 723.1.

Input data

Ovid MEDLINE, EMBASE, CINAHL, CAB abstracts, WHOLIS, and SIGLE databases were searched for GBD 2010, and PUBMED was searched through October 2017 for GBD 2017. There were no age, sex, or language restrictions. The terms neck pain, neck ache, neckache, and cervical pain individually and combined with each of the following terms: prevalen*, inciden*, cross-sectional, cross sectional, epidemiol*, survey, population-based, population based, population study, population sample.

Exclusion criteria were:

1. Sub-populations clearly not representative of the national population
2. Not a population-based study
3. Studies on a specific type of neck pain (eg, following neck fracture)
4. Low sample size (less than 150)
5. Review rather than original studies

An update systematic review was conducted for neck pain and low back pain simultaneously in GBD 2021, using the following search string:

(((Back Pain[MeSH] OR "back pain"[TiAb]) AND (prevalen*[TiAb] OR inciden*[TiAb]) AND (2017/09/01[PDAT] : 3000[PDAT]))

OR(((prevalen* OR inciden*) AND ("neck pain" OR "neck ache" OR "neckache" OR "cervical pain" OR Neck Pain[Mesh])) AND (2017/12/20[PDAT] : 3000[PDAT]))

)

NOT (animals[MeSH] NOT humans[MeSH])

This returned 1346 entries, of which eight neck pain sources were extracted.

Figure 1: PRISMA diagram of neck pain systematic review from 2021

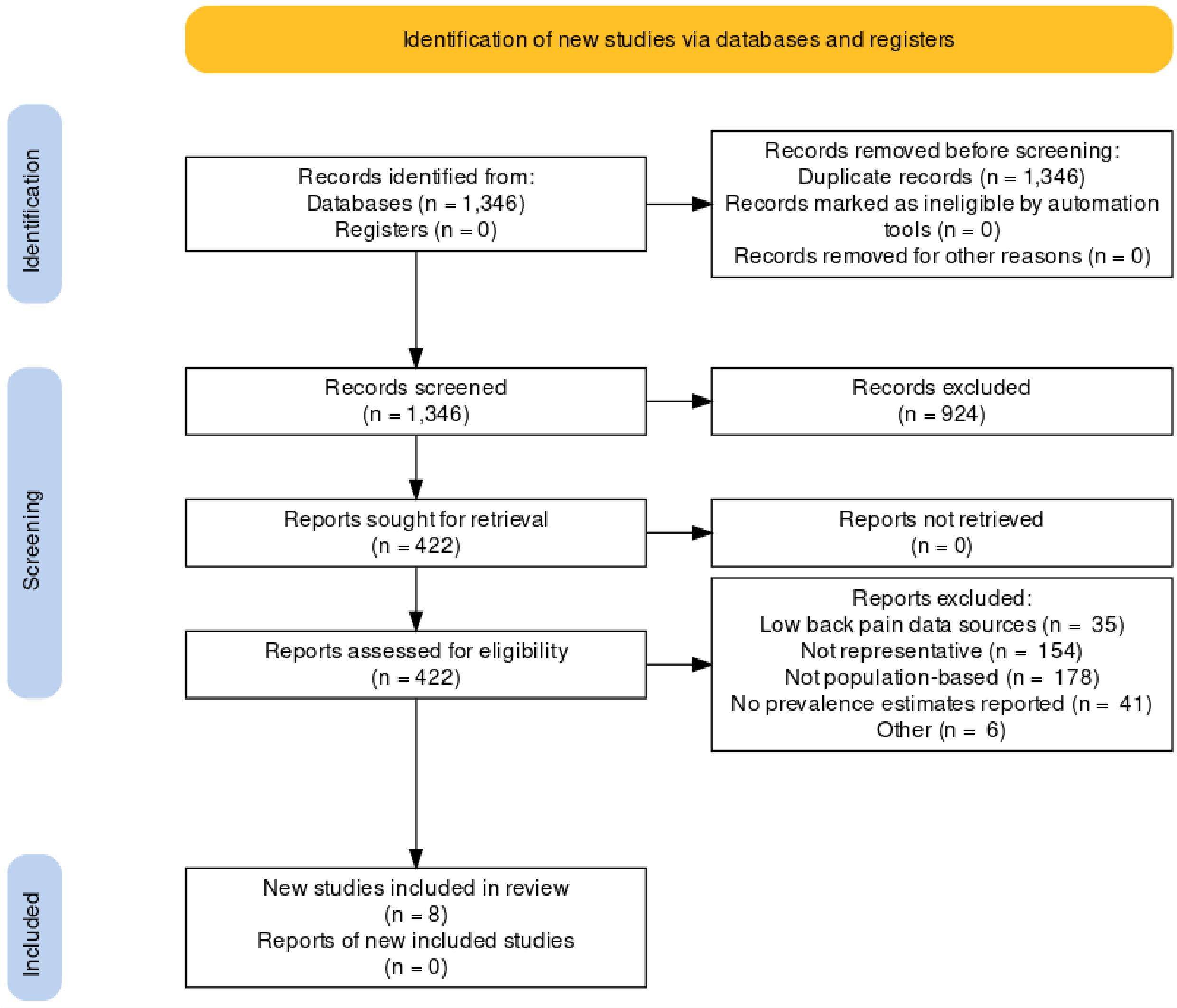

Additional information was derived from unit record data of surveys in the GHDx, GBD's repository of population health data including National Health and Nutrition Examination Survey (NHANES) and National Health Interview Survey (NHIS) in the USA. Opportunistically, additional studies encountered during data review were added for GBD 2019. In addition, data from USA claims data for 2000 and 2010–2015 by state and Taiwan claims data from 2016 were included.

Table 1: Data inputs for neck pain

Measure	Total sources	Countries with data	New sources
All measures	100	29	25
Prevalence	85	29	25
Remission	1	1	0
Other	15	1	0

Age and sex splitting

Reported estimates of prevalence were split by age and sex where possible. First, if studies reported prevalence for broad age groups by sex (eg, prevalence in 15- to 65-year-old males and females separately), and also by specific age groups for both sexes combined (eg, prevalence in 15- to 30-year-olds, then in 31- to 65-year-olds, for males and females combined), age-specific estimates were split by sex using the reported sex ratio and bounds of uncertainty. Second, prevalence data for both sexes that could not be split using a within-study ratio were split using a sex ratio derived from a meta-analysis of existing sex-specific data using MR-BRT (meta-regression—Bayesian, regularised, trimmed[1]). The female to male ratio was 1.47 (1.19 to 1.83). Finally, after the application of bias adjustments, where studies reported estimates across age groups spanning 25 years or more, these were split into five-year age groups using the prevalence age pattern estimated by DisMod-MR 2.1 (disease model—Bayesian meta-regression[2]) in GBD 2019.

Data adjustment

We used MR-BRT to calculate adjustment factors to correct for biases introduced by alternative case definitions. These alternative case definitions were studies that reported a too broad of an anatomical region, episode duration of greater than three months, recall periods of one week to one month, recall periods between two months and one year, activity-limiting neck pain, and studies conducted among schoolchildren. We added three additional covariates for claims data in the USA from the year 2000 and from 2010 onward and for Taiwan claims data. The mean and standard error for the coefficients were calculated using a two-step MR-BRT network crosswalk adjustment method. The covariate for claims data from Taiwan was not included in the final adjustments, as we were unable to find matches to inform a reliable crosswalk. MarketScan claims data were not included in the final model, as fluctuations in ICD coding prevented the construction of a reliable crosswalk. Betas and exponentiated values (which can be interpreted as an odds ratio) for these two covariates are shown in the table below:

Table 2: MR-BRT crosswalk adjustment factors for neck pain

Data input	Reference or alternative case definition	Gamma	Beta coefficient, logit (95% CI)*	Adjustment factor**
Point prevalence	Ref		---	---
Anatomical region too broad	Alt	0.12	0.97 (0.76 to 1.18)	2.63 (2.13 to 3.25)

[1] Vos T, Lim SS, Abbafati C, *et al.* Global burden of 369 diseases and injuries in 204 countries and territories, 1990–2019: a systematic analysis for the Global Burden of Disease Study 2019. *The Lancet* 2020; **396**: 1204–22. doi: https://doi.org/10.1016/S0140-6736(20)30925-9

Details found in appendix 1, section 4.4.1

[2] Vos T, Lim SS, Abbafati C, *et al.* Global burden of 369 diseases and injuries in 204 countries and territories, 1990–2019: a systematic analysis for the Global Burden of Disease Study 2019. *The Lancet* 2020; **396**: 1204–22. doi: https://doi.org/10.1016/S0140-6736(20)30925-9

Episode duration ≥3 months	Alt	0.12	–0.78 (–0.91 to –0.65)	0.46 (0.40 to 0.52)
Recall periods of 1 week to 1 month	Alt	0	1.13 (1.08 to 1.19)	3.10 (2.94 to 3.29)
Recall periods between 2 months and one year	Alt	0	1.68 (1.63 to 1.73)	5.37 (5.10 to 5.64)
Studies among schoolchildren	Alt	0.12	1.07 (0.78 to 1.36)	2.92 (2.18 to 3.90)
Activity-limiting neck pain	Alt	0	–1.13 (–1.14 to –1.12)	0.32 (0.32 to 0.33)

MR-BRT crosswalk adjustments can be interpreted as the factor the alternative case definition is adjusted by to reflect what it would have been had it been measured using the reference case definition. If the log/logit beta coefficient is negative, then the alternative is adjusted up to the reference. If the log/logit beta coefficient is positive, then the alternative is adjusted down to the reference.

*****The adjustment factor column is the exponentiated beta coefficient. For log beta coefficients, this is the relative rate between the two case definitions. For logit beta coefficients, this is the relative odds between the two case definitions.***

After adjusting data for case definition, we outliered data with a median absolute deviation of 2 or more above or below the age-standardised mean. This was done in a systematic way to identify and remove data that were implausibly high or low.

Modelling strategy

Prior settings in the DisMod-MR model included setting excess mortality to 0, and it was assumed that there was no incidence or prevalence of neck pain before the age of 5 years. We made no substantive changes in the modelling strategy from GBD 2019.

Severity and disability

The basis of the GBD disability weight survey assessments are lay descriptions of health states highlighting major functional consequences and symptoms. The lay descriptions and disability weights for neck pain severity levels are shown below.

Table 3. Severity distribution, details on the severity levels for NP in GBD 2021 and the associated disability weight (DW) with that severity.

Severity level	Lay description	DW (95% CI)	Proportions
Neck pain, mild	This person has neck pain, and has difficulty turning the head and lifting things	0.052 (0.036–0.074)	0.67 (0.57–0.75)
Neck pain, moderate	This person has constant neck pain, and has difficulty turning the head, holding arms up, and lifting things	0.112 (0.079–0.162)	0.12 (0.08–0.19)
Neck pain, severe	This person has severe neck pain, and difficulty turning the head and lifting things. The person	0.226 (0.147–0.323)	0.06 (0.05–0.07)

	gets headaches and arm pain, sleeps poorly, and feels tired and worried		
Neck pain, most severe	This person has constant neck pain and arm pain, and difficulty turning the head, holding arms up, and lifting things. The person gets headaches, sleeps poorly, and feels tired and worried	0.300 0.199–0.434)	0.15 (0.11–0.20)

The severity distributions are derived from an analysis of the Medical Expenditure Panel Surveys (MEPS) in the USA. MEPS is an overlapping continuous panel survey of the United States non-institutionalised population whose primary purpose is to collect information on the use and cost of health care. Panels are two years long and are conducted in five rounds, which are conducted every five to six months. A new panel begins annually, while the last panel is in its second year. Each panel typically contains about 30,000 to 35,000 individual respondents (http://www.meps.ahrq.gov/survey_comp/hc_data_collection.jsp).

MEPS was initiated in 1996 but only began collecting health status data in the form of SF-12 responses in 2000. We used data from 2000–2014 for our analysis, which was last updated in GBD 2019. Respondents self-administer the SF-12 twice per panel, at rounds two and four, typically about a year apart. Only adults 18 years and older completed the SF-12. MEPS also usually collects information on diagnoses based on self-report of reasons for encounters with health services. In addition, diagnoses are derived through additional questions on "problems that bother you" or conditions that led to "disability days," ie, days out of role due to illness. Professional coders translate the verbatim text into three-digit ICD-9 codes. The main reason for neck pain being measured in MEPS relates to health-care contact.

In order to derive a crosswalk of SF-12 values into a scale comparable with that used by the GBD disability weights, small studies on convenience samples were conducted asking respondents to fill in SF-12 to reflect 62 lay descriptions of diverse severity that were used to derive the GBD disability weights. From these responses a relationship between SF-12 summary score and the GBD DWs was derived. With regression methods, average disability weights were calculated for each of 156 conditions for which there were corresponding diagnoses in MEPS, while controlling for any co-morbid other condition by adding dummy variables for each condition. As our case definition is for point prevalence of neck pain, we ignored the proportion of MEPS respondents with a neck pain diagnosis for whom in our regression we found no disability attributable to neck pain. For the remaining cases we binned the amount of DW attributed to neck pain across the four health states assuming thresholds at the midpoints between DW values.

Neonatal preterm birth complications

Input data and methodological summary

Short gestational age and low birthweight are highly correlated risk factors associated with poor child health outcomes. The “low birthweight and short gestation” (LBWSG) risk factor quantifies the burden of disease attributable to increased risk of death and disability due to 1) less than ideal birthweight (“low birthweight”) and 2) shorter than ideal length of gestation (“short gestation”).

Within GBD, attributable burden is generally estimated separately for each individual risk factor, but the combined burden attributable to multiple risk factors is of general interest. In GBD, attributable burden due to multiple risk factors is typically estimated through a “mediation analysis” that is applied after independent estimation of each risk factor’s exposure, relative risk, theoretical minimum risk exposure level (TMREL), and population attributable fraction (PAF). In the mediation analysis, a “mediation factor” adjusts the PAF of each risk factor by the amount of attributable burden mediated through the other GBD risk factors. While mediation may be common, direct quantification of the joint exposure, relative risk, and PAF of the combined risk factors is conceptually more straightforward.

In GBD 2016, LBWSG became the first (and, as of GBD 2021, only) group of GBD risk factors in which combined attributable burden is quantified by direct estimation of the joint exposure, relative risk, TMREL, and PAF of multiple risk factors. After first directly estimating the joint exposure, relative risk, TMREL, and PAF of birthweight and gestational age together, we then separate out the independent PAFs due to birthweight only or gestational age only. Because of this modelling strategy, the joint GBD risk factor quantifying the burden of disease due to both less than ideal birthweight (“low birthweight”) and shorter than ideal gestational age (“short gestation”) is grouped into a single “parent” risk factor termed “low birthweight and short gestation”. LBWSG is disaggregated into two “child” risk factors: “low birthweight for gestation” and “short gestation for birthweight”. Low birthweight for gestation quantifies the burden of disease attributable to less than ideal birthweight, after adjusting for the influence of gestational age. Likewise, short gestation for birthweight quantifies the burden of disease attributable to shortened gestational age, after adjusting for the influence of birthweight.

Ideally, the model for joint exposure and joint relative risk would be fully continuous. To simplify the computation for the analysis, a grid of 500-gram and two-week units (“bins”) is used as the LBWSG dimensions and to approximate a fully continuous joint distribution model (see Figure 1).

Flowchart

Neonatal preterm (cause) and Low birth weight and short gestation (risk factor) estimation flowchart

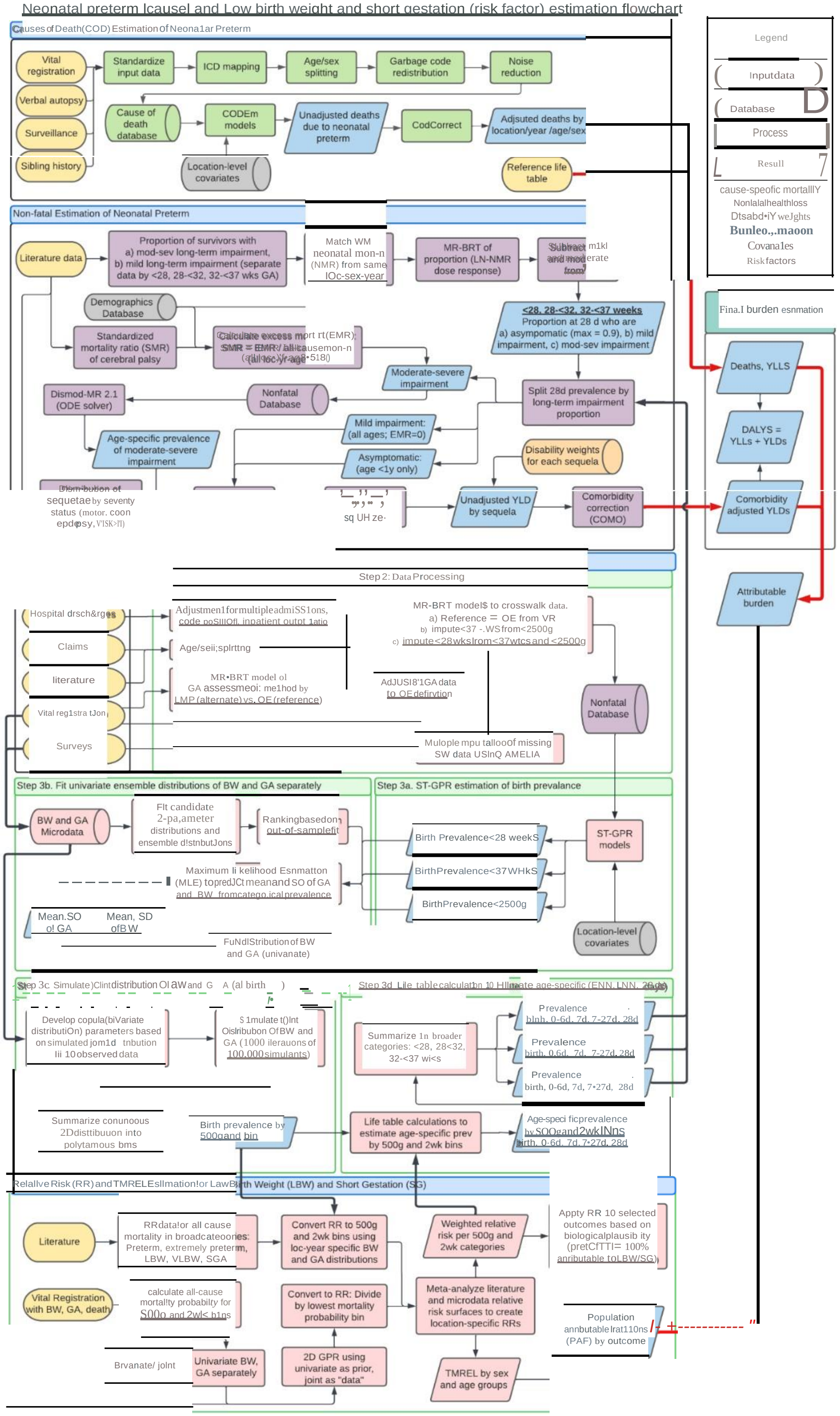

Figure 1. Fully continuous analysis of joint gestational age and birthweight (left) is approximated with a grid of birthweight and gestational age with 500-gram and two-week "bins" (right)

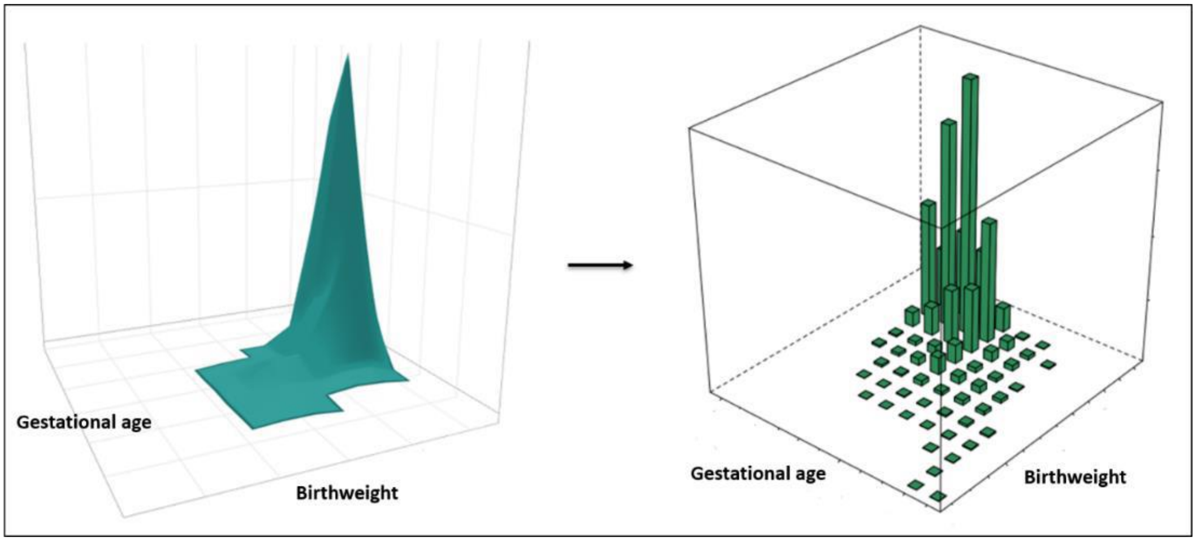

Case definition

"Low birthweight" has historically referred to any birthweight less than 2500 grams, dichotomising birthweight into two categories: "normal" and "low". In the context of the GBD LBWSG risk factor, low birthweight refers to any birthweight less than the birthweight TMREL (the birthweight that minimises risk at the population level). Because LBWSG is estimated in a grid of 500-gram and 2-week bins, any 500-gram birthweight unit less than the TMREL, which was determined as [38, 40) weeks and [3500, 4000) g for the LBWSG parent risk factor, is considered "low birthweight". This includes, for example, birthweight of [2500, 3000) grams, which the traditional, dichotomous definition of "low birthweight" would not include.

Like birthweight, gestational age is typically classified into broad categories. "Preterm" is used to describe any newborn baby born less than 37 completed weeks of gestation. In the GBD context, "short gestation" is used to refer to all gestational ages below the gestational age TMREL.

Exposure

In LBWSG, exposure refers to the portion of the joint distribution of gestational age and birthweight less than the TMREL, by location/year/sex (l/y/s), from birth to the end of the neonatal period. Modelling LBWSG exposure can be summarised in three steps:

A. Model univariate gestational age and birthweight distributions at birth, by l/y/s
B. Model joint distributions of gestational age and birthweight at birth, by l/y/s
C. Model joint distributions from birth to the end of the neonatal period, by l/y/s

Table 1. Analytical steps in estimation of years lived with disability (YLDs) due to preterm birth

Step	Summary of exposure modelling strategy
**Step A**<br>Model univariate distributions at birth	1. Model mean gestational age, prevalence of gestational age <28 weeks, and prevalence of gestational age <37 weeks, by l/y/s<br>2. Model mean birthweight and prevalence of birthweight <2500 grams, by l/y/s<br>3. Model univariate gestational age and birthweight distributions separately at birth, by l/y/s
**Step B**<br>Model joint distributions at birth	1. Use copulae to model the correlation structure of the joint distribution of gestational age and birthweight, globally<br>2. Model the joint distribution of gestational age and birthweight, by location/year/sex at birth, by applying the globally modelled correlation structure to the location/year/sex-specific univariate models of gestational age and birthweight distributions
**Step C**<br>Model joint distributions from birth to 28 days	1. Model all-cause mortality rates by gestational age and birthweight<br>2. Model gestational age and birthweight distributions of surviving neonates for all l/y/s from birth to end of the neonatal period, using all-cause mortality rates by gestational age and birthweight

Input data and data processing

Input data needed to model univariate gestational age and birthweight distributions at birth (Step A):

- Prevalence of preterm birth (<37 weeks), by l/y/s
- Prevalence of preterm birth (<28 weeks), by l/y/s
- Mean gestational age, by l/y/s
- Gestational age microdata
- Prevalence of low birthweight (<2500 grams), by l/y/s
- Mean birthweight, by l/y/s
- Birthweight microdata

To model joint distributions of gestational age and birthweight (Step B), joint microdata of gestational age and birthweight are also required. Additional inputs to modelling joint distributions from birth to 28 days (Step C) are all-cause mortality by l/y/s and joint birthweight and gestational age microdata linked to mortality outcomes.

Prevalence of extremely preterm birth (<28 weeks) and preterm birth (<37 weeks) were modelled using vital registration, survey, and clinical data. For the preterm models, only inpatient and insurance claims data were included from clinical informatics datasets; outpatient data were excluded because they were more likely to capture repeated visits by the same child rather than unique visits. Prevalence of low birthweight (<2500 grams) was modelled using only vital registration and survey data.

Literature review

Before GBD 2016, available preterm birth data were sourced by a technical working group. In GBD 2016 and GBD 2017, we conducted systematic reviews to identify additional sources beyond the data already used in the models. The PubMed database was searched using the following search string:

> ((("Infant, Premature"[Mesh] OR ("infant"[All Fields] AND "premature"[All Fields]) OR "premature infant"[All Fields] OR ("preterm"[All Fields] AND "infant"[All Fields]) OR "preterm infant"[All Fields] OR ("infant, newborn"[MeSH Terms] OR ("infant"[All Fields] AND "newborn"[All Fields]) OR "newborn infant"[All Fields] OR ("newborn"[All Fields] AND "infant"[All Fields])) AND (premature[All Fields] OR preterm[All Fields]) OR "premature birth"[MeSH Terms] OR ("premature"[All Fields] AND "birth"[All Fields]) OR "premature birth"[All Fields] OR ("preterm"[All Fields] AND "birth"[All Fields]) OR "preterm

birth"[All Fields]) ((("Infant, Premature"[Mesh] OR ("infant"[All Fields] AND "premature"[All Fields]) OR "premature infant"[All Fields] OR ("preterm"[All Fields] AND "infant"[All Fields]) OR "preterm infant"[All Fields] OR ("infant, newborn"[MeSH Terms] OR ("infant"[All Fields] AND "newborn"[All Fields]) OR "newborn infant"[All Fields] OR ("newborn"[All Fields] AND "infant"[All Fields])) AND (premature[All Fields] OR preterm[All Fields]) OR "premature birth"[MeSH Terms] OR ("premature"[All Fields] AND "birth"[All Fields]) OR "premature birth"[All Fields] OR ("preterm"[All Fields] AND "birth"[All Fields]) OR "preterm birth"[All Fields]) AND ("1985"[PDAT] : "3000"[PDAT]) AND "humans"[MeSH Terms].

The exclusion criteria were studies that did not provide primary data on epidemiological parameters, non-representative studies (eg, only high-risk pregnancies), and reviews. Table 2 shows the search hits, number of full-texts reviewed, and number of extracted sources.

Table 2. LBWSG search hits, full-text review, extracted sources

Search	Hits	Full-text review	Extracted	Search date
GBD 2017	16 174	2200	154	6/6/2017

Table 3. Input data for exposure models

Input data	Exposure
Source count (total)	2230
Number of countries with data	176

Data processing

Any data that didn't fit a GBD age group were split into age groups using a model that was run using only age-specific data. Starting in GBD 2019, as was the case with all other non-fatal analyses,we applied empirical age and sex ratios from previous models to disaggregate observations that did not entirely fit in one GBD age category or sex. Ratios were determined by dividing the result for a specific age and sex by the result for the aggregate age and sex specified in a given observation.

Low birthweight (<2500 grams) data were extracted from literature, vital registration systems, and surveys. Survey data (most commonly from DHS and MICS) were observed to have high missingness of birthweight responses. We evaluated the patterns of missingness and found a number of distinct patterns that suggested non-random omission of birthweight observations. We therefore imputed missing birthweight values using the Amelia II (Version 1.7.6) package in R. Birthweight was predicted using the following variables also in the DHS surveys: urbanicity, sex, birthweight recorded on card, birth order, maternal education, paternal education, child age, child weight, child height, mother's age at birth, mother's weight, shared toilet facility, and household water treated.

After imputation, we completed a number of additional steps to standardise the dataset by applying a series of crosswalks. "Crosswalking" is a process of reducing non-random bias by adjusting non-standard data to the likely value had the data been collected using a reference definition, technique, or sample. Three crosswalks were applied for birthweight and gestational age data, all of the statistical models for which were developed using meta-regression—Bayesian, regularised, trimmed (MR-BRT).

First was a crosswalk for method of gestational age assessment that included three separate models. All microdata that reported GA and both obstetric estimate (OE) and last menstrual period were crosswalked to OE using the relationship derived from USA GA microdata (Figure 2). This crosswalk was developed with a spline on LMP in order to reliably match on the data that needed to be crosswalked.

Next, for all data that were only categorical, we adjusted all gestational age data to a reference definition of obstetric estimate (OE), which also included tabulations of the crosswalked microdata above. Two alternate definitions regularly appeared, and both were crosswalked separately. These were

last menstrual period (LMP) for each of <37 weeks' and <28 weeks' gestation (Tables 4 and 5) and other measure of gestation age (Table 6 and 7).

The second set of crosswalks adjusted data derived from clinical administrative sources (ie, hospital discharges and insurance claims) to matched vital registration data using OE (Tables 8 and 9).

The third set of crosswalks served to "square the input dataset" to ensure that every location-year with data had an observation for each of <2500 g (birthweight), <37 weeks, and <28 weeks. This process used relationships between input data types to maximise the volume of data later input to models. Low birthweight data (<2500 g) were crosswalked to preterm (<37 weeks) data (Table 10), preterm to extremely preterm (Table 11), and extremely preterm to preterm (Table 12).

Figure 2. MR-BRT OE-LMP crosswalk adjustment factor by LMP-reported gestational age

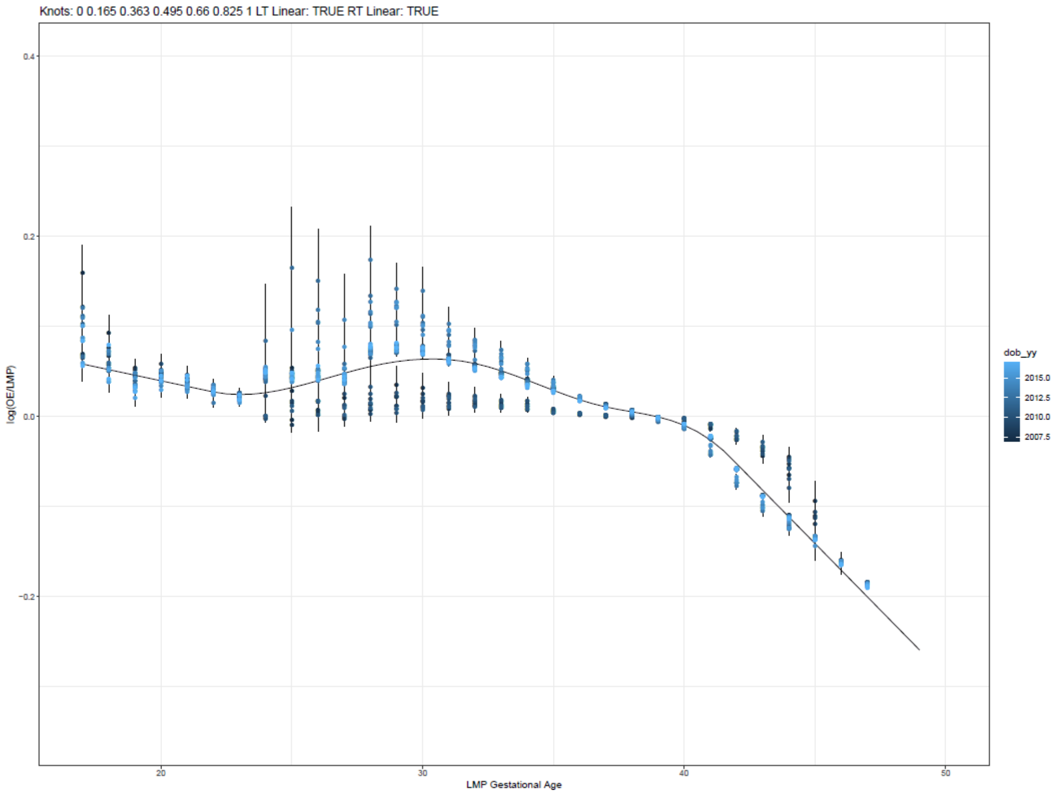

Table 4. MR-BRT OE-LMP crosswalk adjustment factor for preterm birth (<37 weeks' gestation)

Data input	Reference or alternative case definition	Gamma	Beta coefficient, log (95% CI)	Adjustment factor*
Obstetric estimate	Reference	0.01	---	---
Last menstrual period	Alt		0.187 (0.142, 0.231)	1.205 (1.153, 1.260)

Table 5. MR-BRT OE-LMP crosswalk adjustment factor for extremely preterm (<28 weeks' gestation)

Data input	Reference or alternative case definition	Gamma	Beta coefficient, log (95% CI)	Adjustment factor*
Obstetric estimate	Reference	0.00	---	---
Last menstrual period	Alt		0.0284 (0.268, 0.300)	1.328 (1.308, 1.349)

Table 6. MR-BRT OE-other measure crosswalk adjustment factor for preterm birth (<37 weeks' gestation)

Data input	Reference or alternative case definition	Gamma	Beta coefficient, log (95% CI)	Adjustment factor*
Obstetric estimate	Reference	0.10	---	---
Other measurement	Alt		–0.243 (–0.494, 0.009)	0.785 (0.610, 1.01)

Table 7. MR-BRT OE-other measure crosswalk adjustment factor for extremely preterm birth (<28 weeks' gestation)

Data input	Reference or alternative case definition	Gamma	Beta coefficient, log (95% CI)	Adjustment factor*
Obstetric estimate	Reference	0.37	---	---
Other measurement	Alt		0.154 (–0.486, 0.793)	1.166 (0.615, 2.210)

Table 8. MR-BRT VR-claims crosswalk adjustment factor for preterm birth (<37 weeks' gestation)

Data input	Reference or alternative case definition	Gamma	Beta coefficient, log (95% CI)	Adjustment factor*
Vital registration	Reference	0.07	---	---
Insurance claims	Alt		–0.712 (–0.909, –0.515)	0.491 (0.403, 0.597)

Table 9. MR-BRT VR-insurance claims crosswalk adjustment factor for extremely preterm birth (<28 weeks' gestation)

Data input	Reference or alternative case definition	Gamma	Beta coefficient, log (95% CI)	Adjustment factor*
Vital registration	Reference	0.02	---	---
Insurance claims	Alt		–1.258 (–1.447, –1.07)	0.284 (0.235, 0.344)

Table 10. MR-BRT low birthweight to preterm birth (<37 weeks' gestation)

Data input	Reference or alternative case definition	Gamma	Beta coefficient, log (95% CI)	Adjustment factor*
Preterm birth	Reference	0.08	---	---
Low birthweight	Alt		–0.479 (–0.518, –0.440)	0.620 (0.596, 0.644)

Table 11. MR-BRT preterm (<37 weeks' gestation) to extremely preterm (<28 weeks' gestation)

Data input	Reference or alternative case definition	Gamma	Beta coefficient, log (95% CI)	Adjustment factor*
28 weeks	Reference	0.06	---	---
37 weeks	Alt		3.221 (3.161, 3.281)	25.053 (23.600, 26.604)

Table 12. MR-BRT extremely preterm (<28 weeks' gestation) to preterm (<37 weeks' gestation)

Data input	Reference or alternative case definition	Gamma	Beta coefficient, log (95% CI)	Adjustment factor*
37 weeks	Reference	0.05	---	---
28 weeks	Alt		–3.208 (–3.266, –3.150)	0.0404 (0.0381, 0.0428)

**MR-BRT crosswalk adjustments can be interpreted as the factor the alternative case definition is adjusted by to reflect what it would have been had it been measured using the reference case definition. If the log/logit beta coefficient is negative, then the alternative is adjusted up to the reference. If the log/logit beta coefficient is positive, then the alternative is adjusted down to the reference.*

***The adjustment factor column is the exponentiated beta coefficient. For log beta coefficients, this is the relative rate between the two case definitions. For logit beta coefficients, this is the relative odds between the two case definitions.*

These data adjustments had the effect of dramatically increasing the size of each of the modelling datasets and are primarily responsible for most changes in preterm estimates between GBD 2019 and GBD 2021. After all crosswalks, we performed a deduplication step on GA models. Namely, if low birthweight data in countries that were 1) categorised as "data-rich" locations in cause of death modelling or had at least ten consecutive years of vital registration data recording gestational age, and 2) had both preterm birth and low birthweight data, crosswalked low birthweight data were outliered so that the model was informed only by the gestational age data.

Modelling strategy

Step 1A: Model univariate birthweight and gestational age distributions at birth, by l/y/s

Microdata are the ideal data source for modelling distributions; however, microdata are not widely available for birthweight and are scarcer for gestational age. Categorical prevalence data are more readily available from a wider range of locations and years for low birthweight (<2500 g), extremely preterm (<28 weeks of gestation), and preterm birth (<37 weeks of gestation). Because categorical prevalence has wider availability than microdata, we use prevalence data to assist in modelling birthweight and gestational age ensemble distributions.

Ensemble distribution models can be constructed with three pieces of information: mean of the distribution, variance of the distribution, and the weights of the distributions being used in the ensemble. To model mean and variance for all l/y/s for birthweight and gestational age, we first used spatiotemporal Gaussian process regression (ST-GPR) models to model prevalence of low birthweight, extremely preterm and preterm birth for all l/y/s at birth. To model mean birthweight for all l/y/s, OLS linear regression was used to regress mean birthweight on log-transformed low birthweight prevalence. This model was then used to predict mean birthweight for all l/y/s, using the prevalence of low birthweight (<2500 g) modelled for all l/y/s in ST-GPR. Similarly, to model gestational age mean for all l/y/s, OLS linear regression model was used to regress mean gestational age on log-transformed preterm prevalence. Mean gestational age for all l/y/s was predicted using the preterm birth (<37 weeks) estimate modelled in ST-GPR.

Global ensemble weights for gestational age were derived by using all available gestational age and birthweight microdata in Table 13 to select the ensemble weights. The distribution families included in the optimisation process were exponential, gamma, gumbel, Weibull, log-normal, normal, mirrored gamma, and mirrored gumbel. As an advancement in GBD 2021, ensemble weights were fit that specifically targeted the fit at 28 weeks and 37 weeks for gestational age and 1500 grams and 2500 grams for low birthweight. In previous GBD cycles the fit of these models had been optimised to reduce error across the entire distribution. Additionally, as an improvement in GBD 2021, this ensemble weight fitting strategy optimised on all microdata sources simultaneously, as opposed to separately.

For each l/y/s, given the mean and ensemble weights, the variance was optimised to minimise error on the prevalence of preterm birth (<37 weeks) for the gestational age distribution and prevalence of low birthweight (<2500 grams) for the birthweight distribution.

Step 1B: Model joint birthweight and gestational age distributions at birth, by l/y/s

To model the joint distribution of gestational age and birthweight from separate distributions, information was needed about the correlation between the two distributions. Distributions of gestational age and birthweight are not independent; the Spearman correlation for each country where joint microdata were available (Table 13), pooling across all years of data available, ranged from 0.25 to 0.49. The overall Spearman correlation was 0.38, pooling across all countries in the dataset.

Table 13. Summary of microdata inputs

Location	Years of data	Total births*	Format of data	Spearman correlation	Used in ensemble weight selection	Used in copula parameter selection	Used in relative risk models
BRA	2016	2,854,380	Microdata	0.37	Yes	Yes	No
ECU	2003–2015	2,473,039	Microdata	0.34	Yes	Yes	No
ESP	1990–2014	8,537,220	Microdata	0.42	Yes	Yes	No
JPN	1995–2015	23,644,506	Tabulations	0.41	No	No	Yes
MEX	2008–2012	10,256,117	Microdata	0.35	Yes	Yes	No

NOR	1990–2014	1,489,210	Microdata	0.44	Yes	Yes	Yes
NZL	1990–2016	1,600,501	Microdata	0.25	Yes	Yes	Yes
SGP	1993–2015	972,775	Tabulations	0.41	No	No	Yes
TWN	1998–2002	1,331,760	Tabulations	0.38	No	No	Yes
URY	1996–2014	698,622	Microdata	0.49	Yes	Yes	No
USA	1990–2014	81,929,879	Microdata	0.38	Yes	Yes	Yes

** Pooled across all years and sexes, excluding data missing year of birth, gestational age, or birthweight*

Joint distributions between the birthweight and gestational age marginal distributions were modelled with copulae. The Copula and VineCopula packages in R were used to select the optimal copula family and copula parameters to model the joint distribution, using joint microdata from the country-years in Table 13. The copula family selected from the microdata was "Survival BB8", with theta parameter set to 1.75 and delta parameter set to 1.

The joint distribution of birthweight and gestational age per location-year-sex was modelled using the global copula family and parameters selected and the location-year-sex gestational age and birthweight distributions. The joint distribution was simulated 100 times to capture uncertainty. Each simulation consisted of 10,000 simulated joint birthweight and gestational age datapoints. Each joint distribution was divided into 500 g by two-week bins to match the categorical bins of the relative risk surface. Birth prevalence was then calculated for each 500 g by two-week bin.

Step 1C: Model joint distributions from birth to the end of the neonatal period, by l/y/s

Early neonatal prevalence and late neonatal prevalence were estimated using life table approaches for each 500 g and two-week bin. Using the all-cause early neonatal mortality rate for each location-year-sex, births per location-year-sex-bin, and the relative risks for each location-year-sex-bin in the early neonatal period, the all-cause early neonatal mortality rate was calculated for each location-year-sex-bin. The early neonatal mortality rate per bin was used to calculate the number of survivors at seven days and prevalence in the early neonatal period. Using the same process, the all-cause late neonatal mortality rate for each location-year-sex was paired with the number of survivors at seven days and late neonatal relative risks per bin to calculate late neonatal prevalence and survivors at 28 days.

Step 2: Model impairment proportions

Using mild impairment proportion and moderate-to-severe impairment proportion data, we ran a single mixed-effects linear regression model, regressing on Healthcare Access and Quality (HAQ) Index and with a dummy variable on each gestational age and proportion type, to generate country-year-sex-specific estimates of both parameters for each gestational age (Figure 3). The remainder of 1 – (mild proportion + moderate-severe proportion) was assigned to asymptomatic proportion, by gestational age. The maximum sum of the mild and moderate-severe proportions was capped at 90%.

Figure 3. Preterm birth mild, moderate-severe impairment regression on HAQ Index (log), by gestational age

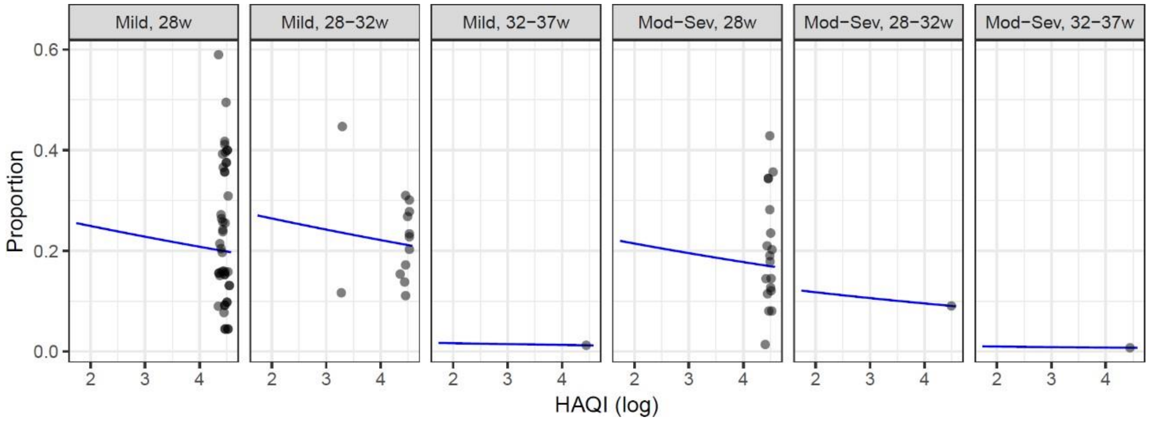

Step 3: Model long-term impairment at all ages

Asymptomatic, mild, and moderate-severe impairment proportions at 28 days, modelled in Step 2, were applied to prevalence at 28 days. Prevalence of survivors of extremely preterm birth, very preterm birth, and moderate-to-late preterm birth to 28 days was estimated in the modelling step described in Step 1C. Asymptomatic prevalence was assumed to be the same from birth to one year as at 28 days. Asymptomatic prevalence was set to zero after one year, as no burden is assumed after the first year of life. Mild prevalence was assumed to be the same at all GBD age groups as at 28 days. This was both a pragmatic decision in terms of reducing complexity of subsequent modelling steps, but also reflects a lack of data and therefore an assumption of no excess mortality among those born preterm who develop mild impairment.

The sum of asymptomatic and mild impairment in the early and late neonatal periods was subtracted from the neonatal preterm birth envelope estimates for each gestational age in the early and late neonatal periods, respectively, to estimate moderate-severe impairment.

Standardised mortality ratios of cerebral palsy were used as input data to model the prevalence of neonatal encephalopathy for ages older than the neonatal period based on the assumption that complications of prematurity are one of the reasons that young children go on to develop cerebral palsy. These data were used across all four neonatal causes and for many other causes in the GBD study. We ran a meta-analysis for a 0–19 age group and a 20–99 age group, and the SMR values were converted to EMR using the formula:

$$EMR = (\text{locaton-sex-age-specific } all\text{-}cause\ mortality\ rate) * (\text{age-specific } SMR - 1)$$

To model moderate-severe prevalence at older ages, a DisMod-MR 2.1 model was run on the existing moderate-severe prevalence estimate (eg, prevalence in the early neonatal period), and on excess mortality estimates derived from the standard mortality ratios (SMR) of cerebral palsy. Remission and incidence were set to zero. The input dataset was entirely complete as every location had an input datum for early neonatal prevalence as well as specific values for EMR at every age-location-sex-year, so we did not specify location-level covariates and the model was set to not pass any priors for any parameter during the estimation cascade, functionally meaning the final estimate age-location-sex-year was not informed by any adjacent locations or years.

Step 4: Split into sequelae

Asymptomatic cases were by definition assigned no disability weight and therefore no YLDs. Mild impairment and moderate-severe impairment due to neonatal preterm birth are split into the sequelae listed in Table 14. The proportion for mild sequelae was split equally between motor and motor plus cognitive impairment. The proportions for each moderate/severe sequela were extracted from a study by Badawi and colleagues and are listed in Table 14. The proportions were the same across gestational age categories.

Prematurity was additionally assessed to be a cause of vision loss via development of retinopathy of prematurity. The proportion of infants born with prematurity and surviving to the end of the neonatal period who go onto develop retinopathy of prematurity is applied to prevalence of preterm birth at 28 days. Proportional splits were estimated by regressing proportion of ROP among preterm infants on natural-log-transformed neonatal mortality rate from 55 studies in 19 countries. The prevalence of infants with ROP is then split into five vision sequelae of varying severity: asymptomatic, mild, moderate, severe, and complete vision loss (blindness). The proportional splits of retinopathy of prematurity by severity are also listed in the table below and are the same across gestational age categories. The mild impairment estimates are split into two sequelae, and the moderate-to-severe impairment estimates are split into 14 sequelae. The mild sequelae were derived by splitting the mild prevalence equally. The proportions for each moderate/severe sequela were extracted from a study by Badawi and colleagues[1] and are listed in the table below in descending order. These proportions were also used to split impairments into sequelae across the other neonatal causes.

Table 14. Proportion of each sequela of neonatal preterm birth

Sequelae of neonatal preterm birth	Mild	Moderate-Severe
Mild motor impairment	0.500	
Mild motor plus cognitive impairment	0.500	
Moderate motor only		0.173
Moderate motor impairment + epilepsy		0.100
Moderate motor impairment + blindness		0.018
Moderate motor impairment + blindness + epilepsy		0.009
Moderate motor impairment + blindness + cognitive impairment		0.032
Moderate motor impairment + epilepsy + cognitive impairment		0.183
Moderate motor impairment + blindness + epilepsy + cognitive impairment		0.017
Severe motor only		0.152
Severe motor impairment + epilepsy		0.033
Severe motor impairment + blindness		0.006
Severe motor impairment + blindness + epilepsy		0.003
Severe motor impairment + blindness + cognitive impairment		0.038
Severe motor impairment + epilepsy + cognitive impairment		0.216
Severe motor impairment + blindness + epilepsy + cognitive impairment		0.020
Mild retinopathy of prematurity	0.07	0.07
Moderate retinopathy of prematurity	0.19	0.19
Severe retinopathy of prematurity	0.13	0.13
Retinopathy of prematurity with blindness	0.26	0.26

Step 5: Use disability weights to calculate YLDs

Each sequela is associated with a health state, which is used to calculate YLDs. The disability weights for all the health states of all the neonatal disorders are listed in the table below. Some health states are combined using a multiplicative approach to calculate the disability of certain sequelae.

Table 15. Disability weights and lay descriptions by health state

Health state	Description	Disability weight
Motor impairment, mild	Has some difficulty in moving around but is able to walk without help.	0.01 (0.005–0.019)
Motor impairment, moderate	Has some difficulty in moving around, and difficulty in lifting and holding objects, dressing and sitting upright, but is able to walk without help.	0.061 (0.040–0.089)
Motor impairment, severe	Is unable to move around without help, and is not able to lift or hold objects, get dressed or sit upright.	0.402 (0.268–0.545)
Motor plus cognitive impairments, mild	Has some difficulty moving around but is able to walk without help. The person is slow in learning at school. As an adult, the person has some difficulty doing complex or unfamiliar tasks but otherwise functions independently.	0.031 (0.018–0.050)
Motor plus cognitive impairments, moderate	Has some difficulty in moving around, holding objects, dressing, and sitting upright, but can walk without help. The person has low intelligence and is slow in learning to speak and to do simple tasks.	0.203 (0.134–0.290)
Motor plus cognitive impairments, severe	Cannot move around without help, and cannot lift or hold objects, get dressed, or sit upright. The person also has very low intelligence, speaks few words, and needs constant supervision and help with all daily activities.	0.542 (0.374–0.702)
Distance vision blindness	Is completely blind, which causes great difficulty in some daily activities, worry and anxiety, and great difficulty going outside the home without assistance.	0.187 (0.124–0.260)
Epilepsy, less severe (seizures < once per month)	Has sudden seizures two to five times a year, with violent muscle contractions and stiffness, loss of consciousness, and loss of urine or bowel control.	0.263 (0.173–0.367)
Epilepsy, severe (seizures ≥ once per month)	Has sudden seizures one or more times each month, with violent muscle contractions and stiffness, loss of consciousness, and loss of urine or bowel control. Between seizures the person has memory loss and difficulty concentrating.	0.552 (0.375–0.71)
Abdominopelvic problem, severe (proxy for EHB without kernicterus)	Has severe pain in the belly and feels nauseous. The person is anxious and unable to carry out daily activities.	0.324 (0.220–0.442)

Neonatal encephalopathy due to birth asphyxia and trauma

Flowchart

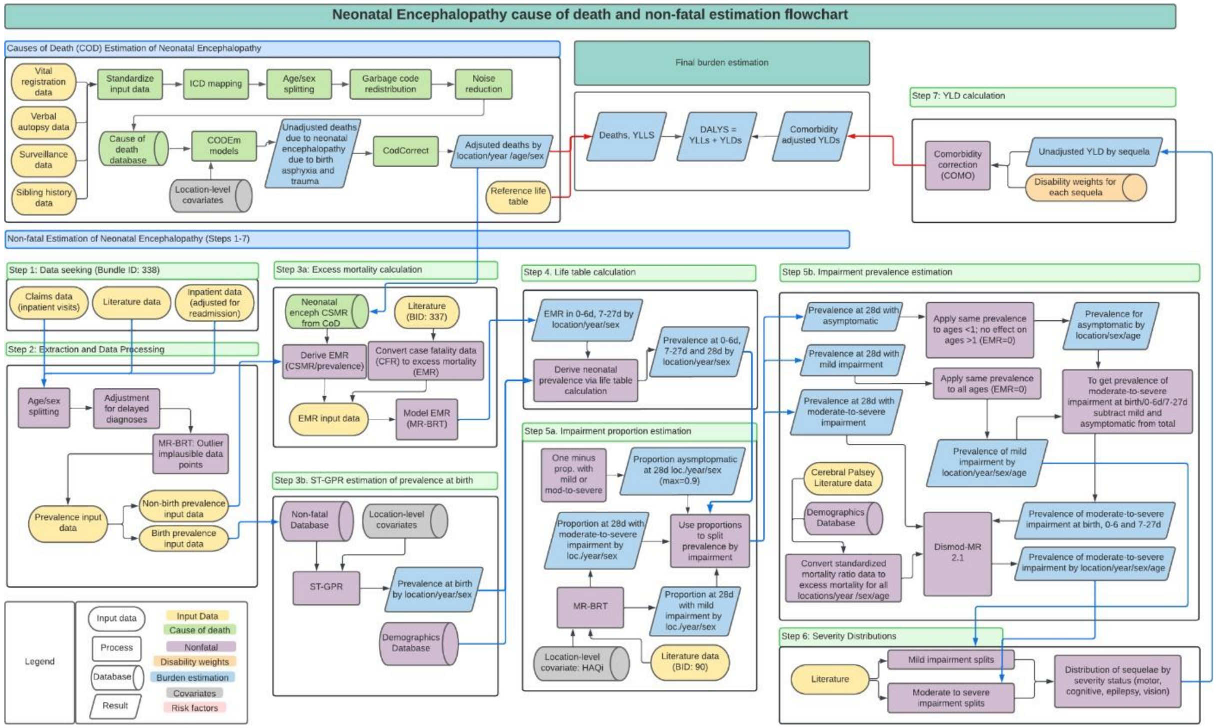

Case definition

Neonatal encephalopathy (NE) due to birth asphyxia and birth trauma is defined in the GBD 2021 non-fatal analyses as injury to the brain in the first few moments or days of life in an infant born at term. The case definition does not include trauma that is not associated with brain injury due to inconsistent coding in clinical administrative datasets. NE is often used interchangeably with the term hypoxic-ischaemic encephalopathy (HIE), but the terms are not strictly synonymous because it is believed that only a subset of NE cases are actually triggered by a hypoxic or ischemic event. NE has multiple aetiologies and is defined by its symptoms – abnormal neurological function, including reduced level of consciousness, seizures, depression of tone and reflexes, or difficulty maintaining respiration.

Modelling strategy

Modelling the non-fatal burden of neonatal encephalopathy occurs in six main steps.

Table 16. Analytical steps in estimation of YLDs due to neonatal encephalopathy due to birth asphyxia and trauma

Step	Summary of modelling strategy
1	Model NE prevalence at birth using ST-GPR
2	Estimate NE prevalence in the early neonatal period, late neonatal period, and at exactly 28 days using a life table algorithm
3	Model case fatality ratio and asymptomatic, mild, and moderate-severe impairment proportions at 28 days using mixed effect regressions, then split prevalence at 28 days by severity of impairment
4	Model impairment prevalence at younger and older ages based on 28-day impairment prevalence
5	Split mild and moderate/severe impairment prevalence into sequelae
6	Apply disability weights to each sequela to calculate YLDs

Table 17. Input data – neonatal encephalopathy due to birth asphyxia and trauma

Measure	Countries with data	New sources	Total sources
Proportion	64	130	424

Step 1: Model NE prevalence at birth using ST-GPR

In previous GBD rounds we used DisMod MR 2.1 to model neonatal encephalopathy prevalence at birth, early neonatal, and late neonatal periods. In GBD 2021, we amended our modelling process to use spatiotemporal Gaussian process regression (ST-GPR) to model the prevalence of neonatal encephalopathy at birth and produced estimates in the early and late neonatal periods using a life table algorithm.

Input data and data processing

Prevalence

We sourced data on prevalence of neonatal encephalopathy at birth from literature and clinical informatics data.

A systematic review for NE was last completed for GBD 2015. The PubMed database was searched using the following search string:

> ((("infant"[Title/Abstract] OR "newborn"[Title/Abstract] OR "newborn infant"[Title/Abstract]) AND ("encephalopathy"[Title/Abstract] OR "neonatal encephalopathy"[Title/Abstract] OR "perinatal asphyxia"[Title/Abstract] OR "asphyxia neonatorum"[Title/Abstract] OR "newborn encephalopathy"[Title/Abstract] OR "hypoxic ischaemic encephalopathy"[Title/Abstract] OR ("birth trauma"[Title/Abstract] AND "birth asphyxia"[Title/Abstract]))) AND ("2012"[PDAT] : "3000"[PDAT]) AND "humans"[MeSH Terms])

The exclusion criteria were studies that did not provide primary data on epidemiological parameters, non-representative studies (eg, only high-risk pregnancies), and reviews. We extracted 60 studies from this review.

Clinical informatics data (hospital and claims) formed the bulk of the input data for the neonatal encephalopathy birth prevalence model, including inpatient hospital and inpatient claims data. Using the meta-regression—Bayesian, regularised, trimmed (MR-BRT) tool, we modelled and applied a correction factor to account for multiple hospital admissions for a single case of neonatal encephalopathy. We did not include outpatient data in the model because we do not believe it to be representative of the true prevalence of neonatal encephalopathy. This is because neonates with neonatal encephalopathy in the countries where hospital data were available are almost sure to be admitted to the hospital, whereas outpatient data are more likely to capture repeated visits by the same child as they grow.

In GBD 2021, clinical informatics data were processed to reflect the discrete under-5 age groups within the GBD study. Because many sources are not linked across years, these splits led to implausible age patterns and an under-ascertainment of cases at birth. Based on the assumption that all cases present in older age groups in the <1-year GBD age groups would necessarily have been present at birth, we adjusted the under-1 inpatient data to back add cases that first "appeared" at older ages to the numerator of the prevalence in younger ages.

In GBD 2019, we standardised data processing to be the same across all sources of clinical informatics data (inpatient hospital and claims data) by ensuring the codes included in claims data matched the codes included in hospital data. This approach standardised the clinical data, but we still observed substantial heterogeneity between clinical and literature data. Investigation of the root cause of the heterogeneity led to a second change: exclusion of those with a solitary discharge diagnosis of P20 (intrauterine hypoxia) from being counted as cases of NE.

Both of these changes technically create a mismatch between GBD mapping of ICD codes for neonatal encephalopathy for non-fatal versus mortality analyses, but we believe this is likely a more accurate representation of how the codes are used. These changes in clinical mapping and processing eliminated the need for crosswalking between clinical informatics source types, but also had the consequence of limiting the size of the dataset because not all sources contained the necessary level of detail to make the necessary distinctions. Significant heterogeneity in NE data from clinical sources remains and is a priority research area going forward in GBD.

We applied empirical sex ratios derived from clinical informatics data to disaggregate literature observations that were sex-aggregated. We calculated these ratios as the pooled sex distribution of birth prevalence from all clinical informatics data sources, which are disaggregated by sex. It is our intention to update this splitting process annually.

Lastly, because of significant residual heterogeneity in input data, especially from clinical administrative sources, we used an MR-BRT model to identify outliers in the birth prevalence data. We logit transformed our prevalence data and calculated standard errors using the delta method and fit a cubic spline on the Healthcare Access and Quality (HAQ) Index with fixed effects on sex and age group with a 40% trimming parameter. All trimmed data were marked as outliers in the model. In addition, in GBD 2021, we outliered all inpatient hospital data from the USA as they were implausibly low compared to both USA claims data and clinical data from similar countries.

Modelling strategy

Due to inconsistencies in estimates of neonatal encephalopathy prevalence between GBD rounds when modelling in DisMod MR-2.1, we significantly altered our approach to estimating neonatal encephalopathy prevalence for GBD 2021. In previous GBD rounds we ran a DisMod MR-2.1 model to estimate prevalence at birth and in the early neonatal and late neonatal periods. We then interpolated these results to estimate the prevalence at 28 days. For GBD 2021 we implemented a demographic life table modelling approach to produce estimates for each of these age groups.

We first modelled the prevalence of neonatal encephalopathy at birth using spatiotemporal Gaussian process regression (ST-GPR), a three-step modelling procedure for generating estimates for every location, year, age, and sex in the GBD study. The first step of the ST-GPR process is an ensemble linear mixed-effects regression of our data on a set of potentially predictive covariates taken from the GBD study covariates database. We tested every combination of these covariates in individual, sex-specific mixed-effects linear regressions with nested random effects at the super-region, region, and location levels. We then evaluated and ranked each of these sub-models by their out-of-sample root-mean-squared error (RMSE). Finally, to produce initial estimates for every location, year, age, and sex in the analysis, we averaged the 50 top-performing models where the estimated coefficients were 1) statistically significant at $p < 0.05$, and 2) in the expected direction. We tested the following covariates in the ensemble prior: antenatal care coverage (1+ visits), in-facility delivery, lag-distributed income per capita, livebirths among women aged 35+ years, total fertility rate, maternal care and immunisation, Socio-demographic Index, Healthcare Access and Quality Index, maternal education (6+ years and 12+ years), ambient particulate matter summary exposure value (SEV), household air pollution SEV, low birthweight SEV, short gestation SEV, and smoking SEV. The covariates selected in each ensemble prior, including their frequency and relative influence (indicated by the standardised beta coefficients for each covariate), are displayed in Figure 4.

Figure 4. Standardised betas from ST-GPR ensemble stage 1 prior

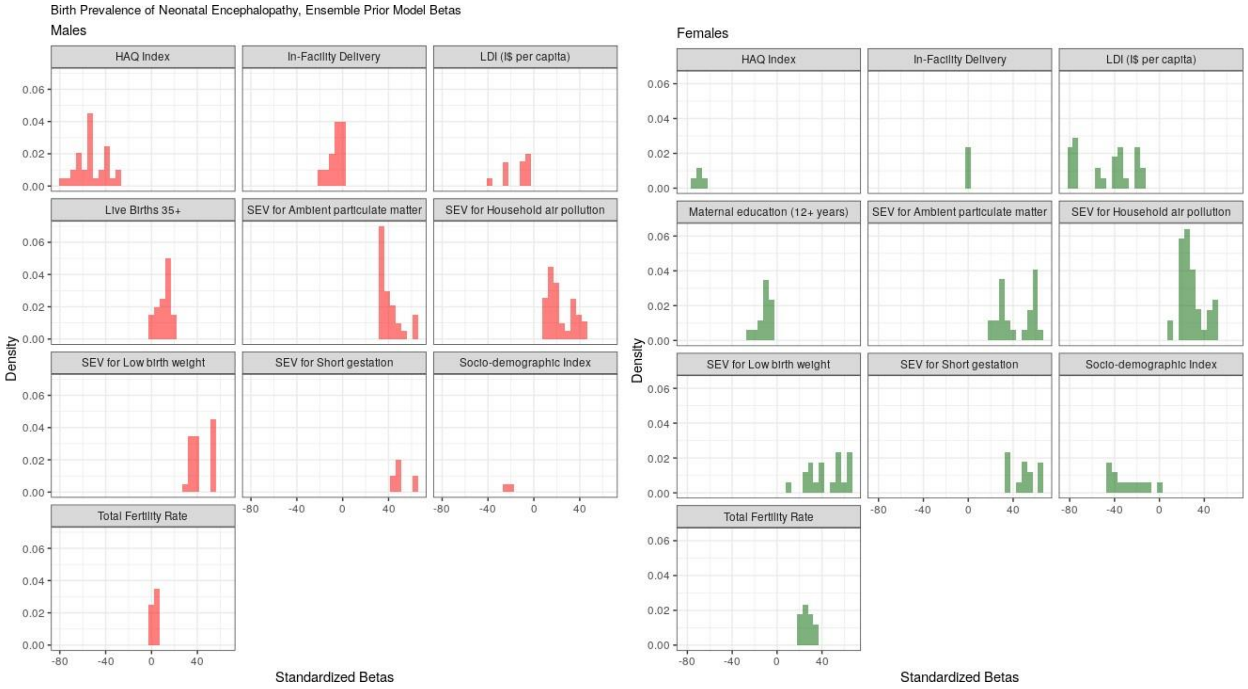

The second, spatiotemporal smoothing step of ST-GPR calculates the residual between our stage 1 regression estimate and each of our observed datapoints and then smooths this residual, drawing

strength over space and time and producing a revised stage 2 estimate of birth prevalence for every location, year, and sex. The third step of ST-GPR is a Gaussian process regression, using the stage 2 estimates as a prior and the observed datapoints and their variance to 1) further smooth the residual between the stage 2 predictions and observed data and produce a final mean estimate for each location, year, and sex, and 2) estimate uncertainty around this mean estimate, quantified by taking 1000 draws from the posterior Gaussian process. More detailed information on the ST-GPR modelling process can be found in the main text methods appendix.

Step 2: Estimate NE prevalence in the early neonatal, late neonatal, and 28-day periods using a life table algorithm

Excess mortality data modelling

Our life table algorithm requires excess mortality estimates for every location, year, age, and sex. To generate these estimates, we modelled excess mortality in MR-BRT, using excess mortality data calculated from case-fatality ratio data as well as derived excess mortality from our prevalence data and modelled cause-specific mortality rates.

We extracted case fatality ratio (CFR) data from literature as the proportion of deaths in the neonatal period (<28 days of life) among cases of neonatal encephalopathy. We did not conduct a separate literature review for this CFR data; rather, it was extracted whenever identified from the search described above. In order to use this CFR data for the life table algorithm detailed below, CFR was transformed into an excess mortality rate (EMR) using the formula:

$$EMR = -\frac{\ln(1 - CFR)}{\frac{days\ of\ observation\ period}{365}}$$

This is analogous to the transformation of cumulative incidence (proportion) to an incidence rate (person-year denominator). The denominator in this equation is the number of days in the observation period for the datapoint – for example, data that followed newborns with neonatal encephalopathy for one year would have a denominator of 1.

Additionally, we calculated excess mortality data wherever we have prevalence data by taking the cause-specific mortality rate estimates from the GBD cause of death analysis divided by the corresponding prevalence datapoint in a given location, year, age, and sex.

For our MR-BRT model, we log-transformed all EMR data and calculated standard errors using the delta method. We fit a cubic spline on the HAQ Index with fixed effects on age group and sex. From this, we generated 1000 draws of estimated EMR for every location, year, age, and sex in the analysis.

Life table algorithm

The next step in our modelling process is a life table algorithm that uses our estimates of birth prevalence of neonatal encephalopathy, excess mortality data, and mortality data from the GBD mortality analysis to generate prevalence estimates in the early and late neonatal age groups and at exactly 28 days for every location, year, and sex.

We began with cases at birth derived from our birth prevalence model and calculated cases at the end of the early neonatal period using the equation:

$$n_7 = n_0\, e^{-EMR_{0-7} t_7}$$

Where n is the number of cases at age 0 or 7 days, EMR_{0-7} is the modelled excess mortality rate for the early neonatal period, and t_7 is the duration of the early neonatal period in years. We repeated this calculation to get cases at the end of the late neonatal period. Prevalence at exactly 28 was a straightforward calculation of cases at 28 days divided by population at 28 days.

To calculate the period prevalence in the early and late neonatal periods, we first summed the person-years of cases who lived to the end of the period and the person-years of the cases who died during the period. We then divided that sum by the sum of the person-years of the general population who lived to the end of the period and the person-years of the general population who died during the period, as follows:

$$p_{0-7} = \frac{n_7 t_{0-7} + n_0(1 - e^{-EMR_{0-7}t_7})a_{0-7}}{N_7 t_{0-7} + N_0 ACMR_{0-7} t_{0-7} a_{0-7}}$$

In this equation, a_{0-7} represents the average years lived in the age interval $[0,7)$ by persons who die in this interval, and $ACMR_{0-7}$ represents the all-cause mortality ratio in that interval.

We computed the population (N_7) at the end of the early and late neonatal periods by using the all-cause mortality rate as follows:

$$N_7 = N_0 (1 - ACMR_{0-7}t_{0-7})$$

In total, we estimated prevalence in the early and late neonatal periods and at exactly 28 days for every location, year, and sex through this method. This method incorporated uncertainty from the initial estimates of birth prevalence and excess mortality but did not include uncertainty from population estimates.

Step 3: Model impairment proportions at 28 days, then split prevalence at 28 days by severity of impairment

Infants who survive neonatal encephalopathy may go on to experience long-term disability or impairment. We categorised impairment for neonatal encephalopathy into three severities: asymptomatic, mild, and moderate-to-severe impairment.

Input data

Data on the proportion of cases of neonatal encephalopathy that go on to develop mild impairment and moderate-to-severe impairment were extracted from a systematic literature review that was last completed in GBD 2013 and updated in GBD 2015. The same search string described above was used to identify impairment data. In GBD 2021, we identified and extracted data from two additional studies to inform our estimates of mild and moderate-to-severe impairment proportion.

Modelling strategy

In GBD 2019, we modelled the proportion of mild impairment and moderate-severe impairment using a linear mixed-effects regression of log impairment proportion on HAQ Index. In GBD 2021, we updated the model to use a more appropriate logit transformation of the proportion data and changed the method from a linear regression to a MR-BRT model, which allowed us to fit the data using a more-flexible quadratic, monotonically increasing spline on HAQ Index and to weight data by their standard

error. These changes led to an increase in the moderate-severe proportion at HAQ Index below approximately 80 and to an increase in the mild proportion at every value of HAQ Index. Figure 5 demonstrates the impact of these changes by showing the predicted impairment proportion values for each severity by level of HAQ Index for GBD 2019 and GBD 2021.

Figure 5. MR-BRT model of impairment proportions

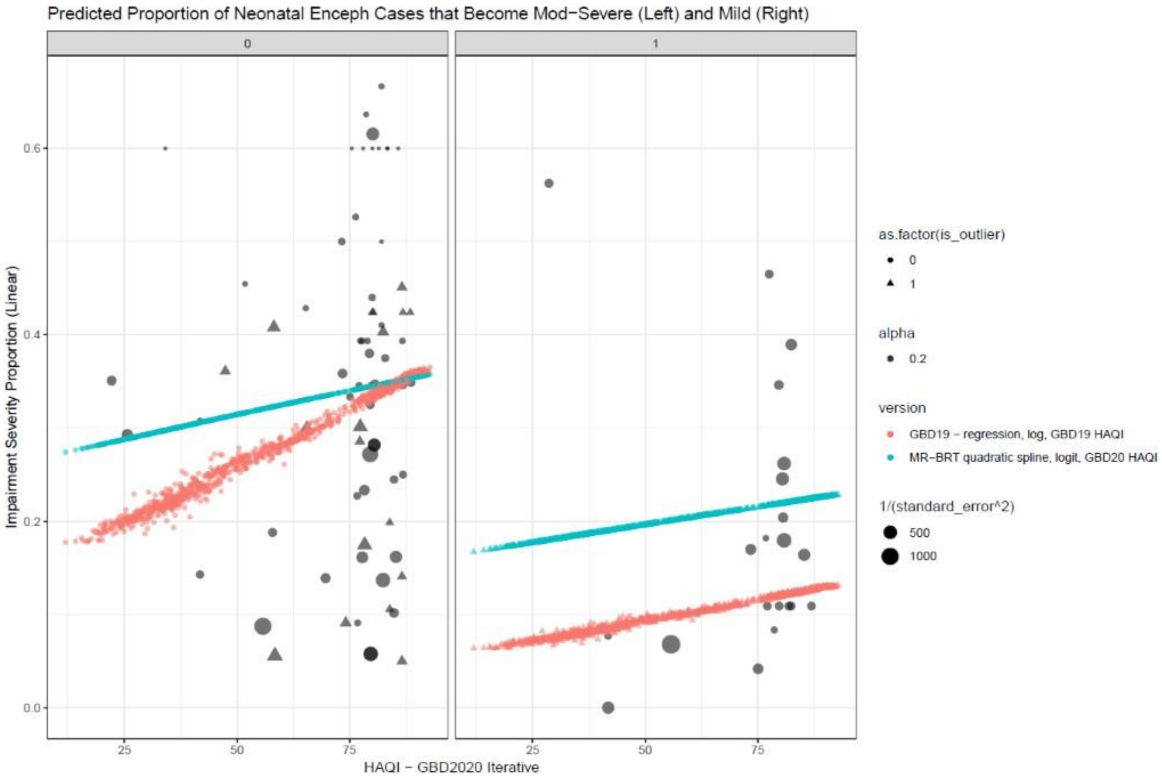

From this MR-BRT model, we generated 1000 draws of estimates of proportion mild and moderate-to-severe impairment for every unique location-year combination. We checked that every location, year, draw pairing of mild and moderate-to-severe impairment proportion never summed to greater than 90%, reserving at least 10% of long-term impairment as asymptomatic. However, no draw pairings summed to greater than 90% and so no draws were adjusted. For every location, year, draw we assigned the remainder proportion, calculated as 1 – (mild impairment proportion + moderate-to-severe impairment proportion), as the proportion with asymptomatic impairment.

We multiplied our estimated impairment proportions by the prevalence at 28 days calculated from our life table algorithm in Step 2 to generate impairment-specific prevalence estimates. Asymptomatic prevalence was extended to other ages based on the assumption that asymptomatic prevalence at 28 days is the same as at early neonatal, late neonatal, and post-neonatal, and that there is no burden and therefore no asymptomatic prevalence after 1 year of age. Mild prevalence was extended to other ages based on the assumption that the mild prevalence at 28 days is the same as the mild prevalence at all other ages. This assumption is grounded in the lack of excess mortality and remission among those born with mild neonatal encephalopathy (ie, no one can develop the disease after birth, no one dies from it, and no one recovers from it, so the number of cases is constant across age).

<u>Step 4: Model long-term impairment prevalence at all ages</u>

Input data

Standardised mortality ratios of cerebral palsy were used as input data to model the prevalence of neonatal encephalopathy for ages older than the neonatal period based on the assumption that brain damage in the neonatal period is one of the reasons that young children go on to develop cerebral palsy. These data were used across all four neonatal causes and for many other causes in the GBD study. We ran a meta-analysis for a 0–19 age group and a 20–99 age group, and the SMR values were converted to EMR using the formula:

$$EMR = (\text{locaton-sex-age-specific } all\text{-}cause\ mortality\ rate) * (\text{age-specific } SMR - 1)$$

Modelling strategy

To estimate the prevalence of moderate-severe impairment at other ages, we needed to account for excess mortality. Because there is excess mortality, the number of cases of moderate-severe impairment declines with age. The sum of asymptomatic and mild impairment prevalence in the early and late neonatal periods was subtracted from the neonatal encephalopathy envelope prevalence estimates (Step 1) to estimate moderate-severe impairment prevalence. This reflects the assumption that all deaths in the early and late neonatal period were among those with moderate-severe impairment, and all newborns who developed asymptomatic or mild neonatal sepsis did not experience excess mortality.

To model moderate-severe prevalence at older ages, a DisMod-MR 2.1 model was run on the existing moderate-severe prevalence estimate (eg, prevalence in the early neonatal period), and on excess mortality estimates derived from the standard mortality ratios (SMR) of cerebral palsy. Remission and incidence were set to zero. The input dataset was entirely complete as every location had an input datum for early neonatal prevalence as well as specific values for EMR at every age-location-sex-year, so we did not specify location-level covariates and the model was set to not pass any priors for any parameter during the estimation cascade, functionally meaning the final estimate age-location-sex-year was not informed by any adjacent locations or years.

Step 5: Split mild and moderate-to-severe prevalence into sequelae

The mild impairment estimates are split into two sequelae, and the moderate-to-severe impairment estimates are split into 14 sequelae. The mild sequelae were derived by splitting the mild prevalence equally. The proportions for each moderate/severe sequela were extracted from a study by Badawi and colleagues[1] and are listed in the table below in descending order. These proportions were also used to split impairments into sequelae across the other neonatal causes.

Table 18. Health states by severity

Sequelae of neonatal encephalopathy	Mild	Moderate-severe
Mild motor impairment	0.500	
Mild motor plus cognitive impairment	0.500	
Moderate motor only		0.173
Moderate motor impairment + epilepsy		0.100
Moderate motor impairment + blindness		0.018
Moderate motor impairment + blindness + epilepsy		0.009
Moderate motor impairment + blindness + cognitive impairment		0.032
Moderate motor impairment + epilepsy + cognitive impairment		0.183
Moderate motor impairment + blindness + epilepsy + cognitive impairment		0.017
Severe motor only		0.152
Severe motor impairment + epilepsy		0.033
Severe motor impairment + blindness		0.006

Severe motor impairment + blindness + epilepsy		0.003
Severe motor impairment + blindness + cognitive impairment		0.038
Severe motor impairment + epilepsy + cognitive impairment		0.216
Severe motor impairment + blindness + epilepsy + cognitive impairment		0.020

Step 6: Use disability weights to calculate YLDs

Each sequela is associated with a health state, which is used to calculate YLDs. The health states used for neonatal encephalopathy are largely the same as the health states for other neonatal causes (see Table 19. Disability weights and lay descriptions by health state for list). Some health states were combined to calculate the burden of certain sequelae.

Table 19. Disability weights and lay descriptions by health state

Health state	Description	Disability weight
Motor impairment, mild	Has some difficulty in moving around but is able to walk without help.	0.01 (0.005–0.019)
Motor impairment, moderate	Has some difficulty in moving around, and difficulty in lifting and holding objects, dressing and sitting upright, but is able to walk without help.	0.061 (0.040–0.089)
Motor impairment, severe	Is unable to move around without help, and is not able to lift or hold objects, get dressed or sit upright.	0.402 (0.268–0.545)
Motor plus cognitive impairments, mild	Has some difficulty moving around but is able to walk without help. The person is slow in learning at school. As an adult, the person has some difficulty doing complex or unfamiliar tasks but otherwise functions independently.	0.031 (0.018–0.050)
Motor plus cognitive impairments, moderate	Has some difficulty in moving around, holding objects, dressing, and sitting upright, but can walk without help. The person has low intelligence and is slow in learning to speak and to do simple tasks.	0.203 (0.134–0.290)
Motor plus cognitive impairments, severe	Cannot move around without help, and cannot lift or hold objects, get dressed or sit upright. The person also has very low intelligence, speaks few words, and needs constant supervision and help with all daily activities.	0.542 (0.374–0.702)
Distance vision blindness	Is completely blind, which causes great difficulty in some daily activities, worry and anxiety, and great difficulty going outside the home without assistance.	0.187 (0.124–0.260)
Epilepsy, less severe (seizures < once per month)	Has sudden seizures two to five times a year, with violent muscle contractions and stiffness, loss of consciousness, and loss of urine or bowel control.	0.263 (0.173–0.367)
Epilepsy, severe (seizures ≥ once per month)	Has sudden seizures one or more times each month, with violent muscle contractions and stiffness, loss of consciousness, and loss of urine or bowel control. Between seizures the person has memory loss and difficulty concentrating.	0.552 (0.375–0.71)
Abdominopelvic problem, severe (proxy for EHB without kernicterus)	Has severe pain in the belly and feels nauseous. The person is anxious and unable to carry out daily activities.	0.324 (0.220–0.442)

Haemolytic disease and other neonatal jaundice

Flowchart

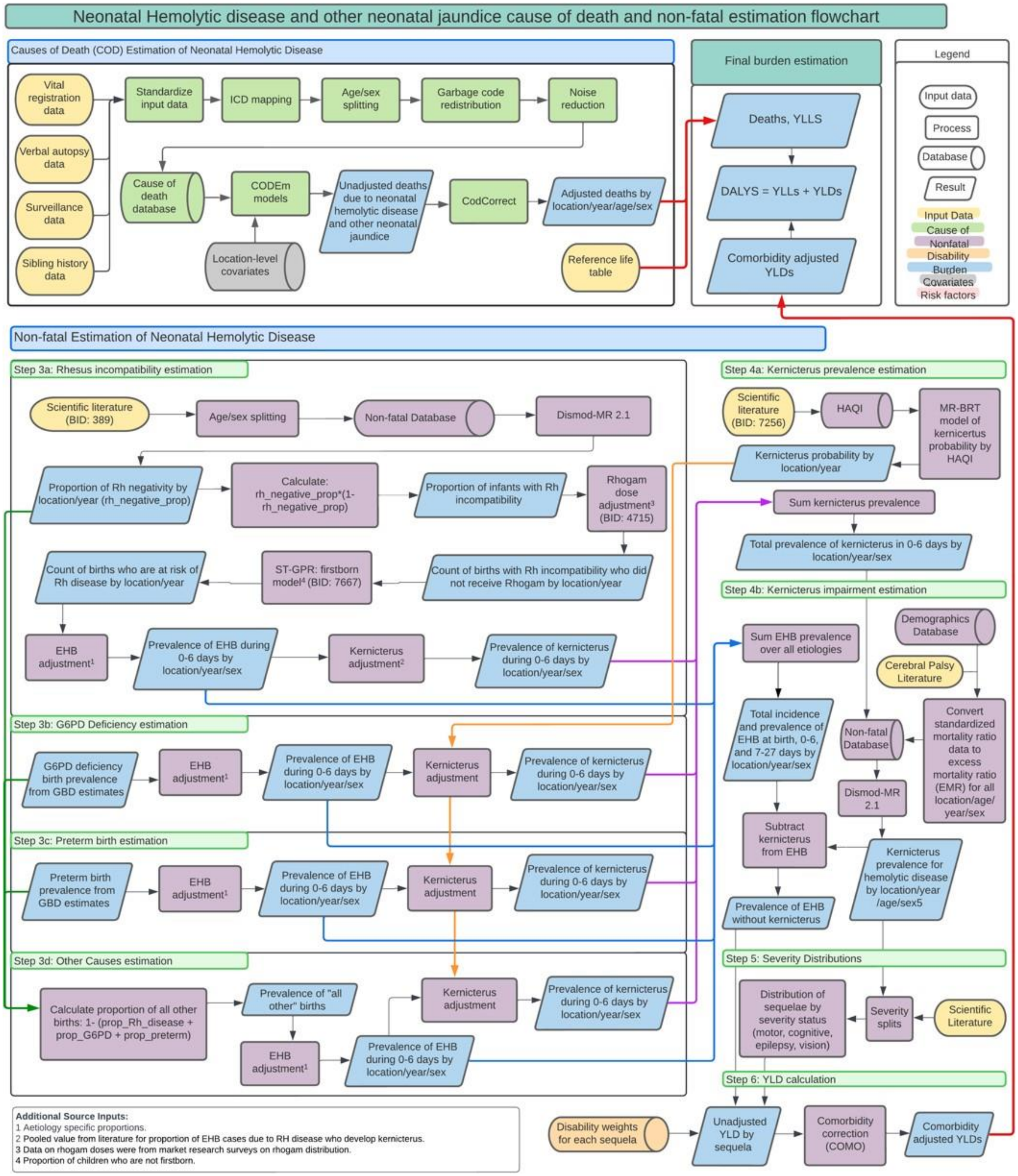

Case definition

Haemolytic disease of the newborn and other neonatal jaundice refers to several aetiologies by which an infant develops extreme hyperbilirubinaemia (EHB) and can then go on to develop kernicterus. We define jaundice as serum bilirubin >5 mg/dL and EHB as >25 mg/dL in the neonatal period. Kernicterus is defined as bilirubin-induced brain injury following an EHB episode and is a clinical diagnosis. GBD estimates are limited to incidence, prevalence, and YLDs due to EHB and kernicterus. We classify EHB

that does not progress to kernicterus as mild impairment and kernicterus as moderate/severe impairment. The aetiologies that inform our estimates for EHB and kernicterus are Rhesus (Rh) disease, preterm birth, glucose-6-phosphate dehydrogenase deficiency (G6PD), and other causes.

Modelling strategy

Modelling the non-fatal burden of haemolytic disease occurs in eight main steps.

Table 20. Analytical steps in estimation of YLDs due to haemolytic disease and other neonatal jaundice

Step	Summary of modelling approach
1	Prevalence of EHB due to Rh disease:<br>a. Rh-negativity prevalence<br>b. Non-firstborn prevalence<br>c. Rhogam availability for Rh-incompatible pregnancies
2	Prevalence of EHB due to G6PD deficiency, preterm birth complications, and other causes
3	Proportion of EHB due to Rh disease who develop kernicterus
4	Proportion of EHB due to G6PD deficiency, preterm birth complications, and other causes who develop kernicterus
5	Prevalence (all ages) of kernicterus (accounting for increased long-term mortality)
6	Prevalence (in neonates only) of EHB without kernicterus
7	Split moderate/severe impairment (kernicterus) prevalence into sequelae
8	Apply disability weights to each sequela to calculate YLDs

Table 21. Input data – haemolytic disease and other neonatal jaundice

Measure	Countries with data	New sources	Total sources
Prevalence	50	0	56
Proportion	188	854	1102

USA claims data and hospital data were not included in the haemolytic disease modelling process because they are not coded separately by aetiology. We are working to develop an analytical framework whereby these data could be incorporated into GBD estimates.

<u>Step 1: EHB due to Rh disease</u>

Birth prevalence of EHB due to Rh disease is estimated using the following equation:

$$\text{EHB Prevalence} = \text{Rh negative prevalence} * (1 - \text{Rh negative prevalence}) * \left(1 - \frac{\text{2010 Rhogam doses}}{\text{2010 Rh-incompatible babies}}\right) * (\text{non-firstborn prevalence}) * 0.15$$

The three components included in the above equation are Rh negativity prevalence, Rhogam (Rh0 immune globulin) doses, which is a medication to prevent RhD isoimmunisation in mothers, and non-firstborn prevalence. Rh negativity was used to estimate the prevalence of Rh-incompatible pregnancies. The number of Rhogam doses was used to calculate the proportion of Rh-incompatible pregnancies that are protected by Rhogam. Non-firstborn prevalence was used to further quantify births who are at risk of Rhesus disease as the RhD isoimmunisation in mothers affects births after the firstborn. The inputs and analytical approach that inform each of these components are described below.

Step 1a: Rh negativity prevalence

Rh negativity prevalence was extracted from literature based on the following search, first completed as a systematic review for GBD 2010. The systematic review was last updated in GBD 2019 to include additional years since GBD 2010. The PubMed database was searched using the search string below on February 7, 2019, and returned 466 results. 39 were screened for full-text review, and eight were ultimately extracted. The exclusion criteria were studies that did not provide primary data on epidemiological parameters, non-representative studies (eg, only high-risk pregnancies), and reviews.

> ((newborn[Title/Abstract] OR neonat*[Title/Abstract]) AND (haemolytic[Title/Abstract] OR hemolytic[Title/Abstract] OR hyperbilirubin*[Title/Abstract] OR jaundice[Title/Abstract] OR "glucose-6"[Title/Abstract] OR G6PD[Title/Abstract] OR EHB[Title/Abstract] OR phototherapy[Title/Abstract] OR "ABO incompatibility"[Title/Abstract] OR "RH incompatibility"[Title/Abstract] OR "rh blood group system"[Title/Abstract] OR Rhesus[Title/Abstract] OR "erythroblastosis fetalis"[Title/Abstract] OR kernicterus[Title/Abstract]) AND (prevalen*[Title/Abstract] OR inciden*[Title/Abstract] OR mortality[Title/Abstract] OR severity[Title/Abstract] OR "long term"[Title/Abstract])) AND (2015/05/01[PDAT] : 3000[PDAT]) NOT "Case Reports"[PT]

Figure 6. PRISMA flow diagram for Rh-negativity systematic review

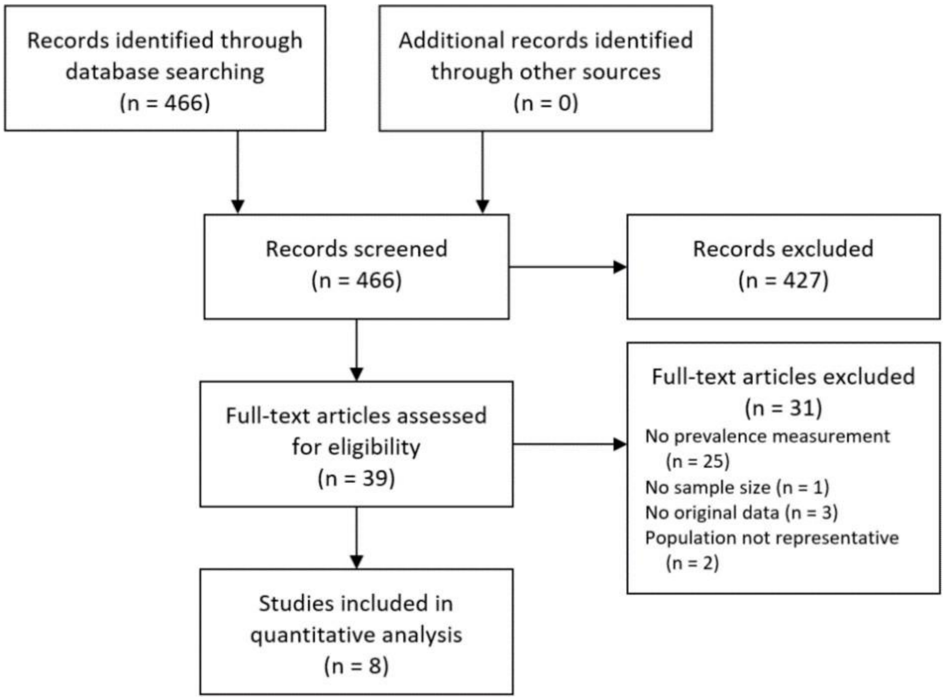

The literature included for quantitative analysis were studies on blood group typing and distribution, blood donors, Rh factor distribution, RhD blood antigens, blood group alloimmunisation, blood transfusion, and efficacy of antibody screening. We extracted data on Rh negativity prevalence covering a total of 49 countries and three subnational locations.

A single-parameter DisMod-MR 2.1 model was run on Rh negativity prevalence. In our estimation for Rh incompatible pregnancies, we assumed that Rh negativity prevalence did not change over time and that it did not vary by age.

Step 1b: Non-firstborn prevalence

Previously, we extracted prevalence of non-firstborn births only from the Demographic Health Surveys (DHS) program's population-representative child and birth history modules. For GBD 2021, we significantly expanded our non-firstborn data, identifying and including numerous additional surveys

from more recent years and including vital registration data from the 2018 United Nations (UN) Demographic Yearbook for higher-income countries. From the surveys, we extracted variables on birth order and child's date of birth to find the proportion of babies who are not firstborns for the years of their birth. The numerator is the number of children who are not firstborn and the denominator is all the children born that year. From the UN Demographic Yearbook, we extracted birth order proportions from tabulated country-level data. The table below shows the number of unique source-location-years, totaling 10 273 and spanning years 1970 to 2018, broken down by GBD super-region.

Table 22. Input data: non-firstborn prevalence

GBD super-region	Unique source-location-years
North Africa and Middle East	283
Sub-Saharan Africa	3758
Central Europe, eastern Europe, and central Asia	357
High-income	297
South Asia	2268
Latin America and Caribbean	821
Southeast Asia, east Asia, and Oceania	2489

For GBD 2021, we ran an ST-GPR model on non-firstborn prevalence with total fertility rate as a covariate to generate estimates of non-firstborn prevalence for each location-year. This ST-GPR model represents a departure from GBD 2019, in which a mixed-effects linear regression was used to estimate non-firstborn prevalence.

Step 1c: Rhogam doses

Data on the distribution of Rhogam doses were reported by the Marketing Research Bureau as cited in Bhutani and colleagues.[2] The report included the number of Rhogam doses distributed to 138 countries in 2010. The table below shows the number of countries for which we had data, broken down by GBD super-region:

Table 23. Input data: Rhogam doses

GBD super-region	Countries with data
North Africa and Middle East	17
Sub-Saharan Africa	45
Central Europe, eastern Europe, and central Asia	19
High-income	2
South Asia	5
Latin America and Caribbean	27
Southeast Asia, east Asia, and Oceania	23

The proportion of Rhogam doses distributed in 2010 to Rh-incompatible pregnancies in 2010 was used as a constant over time for our estimation of the prevalence of babies who are not protected by Rhogam. We made the assumption that countries without data had complete Rhogam coverage if their NMR was less than 5, and zero Rhogam coverage otherwise. This Rhogam coverage assumption is based on the assumption made in the Bhutani and colleagues[2] study.

Extreme hyperbilirubinemia (EHB) probability

The 0.15 multiplier used in the EHB prevalence formula, also cited in Bhutani and colleagues[2], was derived from Zipursky and colleagues[3] , which cited the Clark[4] study on trials for anti-D gammaglobulin before widespread availability of Rhogam; this multiplier was used to represent the proportion of babies at risk for Rh disease who go on to develop EHB. We do not have corresponding information on the proportion of babies at risk for Rh disease who only develop jaundice (and not EHB), which prevents our being able to estimate overall jaundice.

Step 2: EHB due to G6PD deficiency, neonatal preterm birth, and other causes

Input data

The data used to estimate EHB due to non-Rh disease were prevalence of G6PD deficiency and prevalence of neonatal preterm birth, both of which came from corresponding GBD 2021 models, and the proportion of cases who develop EHB. Birth prevalence estimates for G6PD deficiency are described in the appendix section on "Haemoglobinopathies and haemolytic anaemias", and neonatal preterm birth is described in the first neonatal section above. The proportion of cases that develop EHB for each of these causes, also cited in Bhutani and colleagues[2], were derived from combined Canada and Denmark population studies[9-15] that specified causes for EHB (not including Rh disease because of effective national Rh prophylaxis programs) and unpublished data that were further provided by study authors Sgro and Ebbesen. These aetiology-specific EHB proportions are listed in the table below.

Table 24. Proportion of cases of G6PD, preterm birth, and other causes that develop EHB

Aetiology	EHB proportion
G6PD deficiency	0.0013<br>(0.00085, 0.002)
Neonatal preterm birth	0.00045<br>(0.00029, 0.0007)
All other births	0.00038<br>(0.00033, 0.00163)

Modelling strategy

To model the prevalence of EHB due to G6PD deficiency, preterm, and other causes, we started with birth prevalence results for these three conditions. Birth prevalence estimates for G6PD deficiency and neonatal preterm birth came from the corresponding GBD 2021 models of those two conditions. The birth prevalence of other causes was based on the assumption that all babies who do not have any of the three modelled conditions (Rh, G6PD deficiency, and preterm birth) still have some probability of developing EHB. We therefore summed the birth prevalence of Rh disease, G6PD deficiency, and preterm births (as calculated in previous steps), and subtracted this from 1 to get the birth prevalence of all other causes as follows:

$$\text{"Other" birth prevalence} = $$
$$1 - (\text{Rh birth prevalence} + \text{G6PD birth prevalence} + \text{pre-term birth prevalence})$$

We calculated prevalence of EHB by multiplying each birth prevalence estimate by the aetiology-specific scalar from the table above, representing the proportion of children who are expected to develop EHB.

Step 3: Kernicterus prevalence for Rhesus disease

We used 0.072 (0.038, 0.112)[5-8] as the proportion of cases that develop kernicterus from EHB due to Rhesus disease. This is a pooled proportion derived from a study[5] on a large series of pregnancies

affected with Rh disease, case studies[6] at a New Haven, Connecticut, USA hospital, exchange transfusion controlled trials[7] in London with Rh incompatible patients, and studies[8] on serum bilirubin testing from two Boston hospitals, all of which were published in the 1950s to 1970s before widespread availability and initiation of Rhogam, phototherapy, and exchange transfusion. This pooled proportion was cited in the Bhutani and colleagues[2] study.

Step 4: Kernicterus prevalence for G6PD deficiency, neonatal preterm birth, and other causes

Input data

Data on the probability of kernicterus were extracted from literature based on the following search, first completed as a systematic review for GBD 2019. This search was also designed to identify data on the probability of EHB and prevalence of neonatal jaundice as a whole. The PubMed database was searched using the search string below on April 25, 2019, returning 2212 results. 151 were screened for full-text review, and 36 were extracted.

((newborn[Title/Abstract] OR neonat*[Title/Abstract]) AND (haemolytic[Title/Abstract] OR hemolytic[Title/Abstract] OR hyperbilirubin*[Title/Abstract] OR jaundice[Title/Abstract] OR icter*[Title/Abstract] OR "exchange transfusion"[Title/Abstract] OR “acute bilirubin encephalopathy” [Title/Abstract] OR EHB[Title/Abstract] OR phototherapy[Title/Abstract] OR kernicterus[Title/Abstract]) AND (prevalen*[Title/Abstract] OR inciden*[Title/Abstract] OR mortality[Title/Abstract] OR severity[Title/Abstract] OR "long term"[Title/Abstract])) AND (1980[PDAT] : 3000[PDAT]) NOT "Case Reports"[PT]

We included data in our model of kernicterus probability if the total serum bilirubin level in study participants was directly specified or could be reasonably inferred, and if the outcome matched our case definition of kernicterus (bilirubin-induced brain dysfunction). The exclusion criteria were studies that did not provide primary data on epidemiological parameters, non-representative studies (eg, only high-risk pregnancies), and reviews. In GBD 2021, we identified and extracted five new literature sources on the probability of kernicterus given an initial level of total serum bilirubin and included them in the meta-analysis.

Figure 7. PRISMA flow diagram for kernicterus proportion systematic review

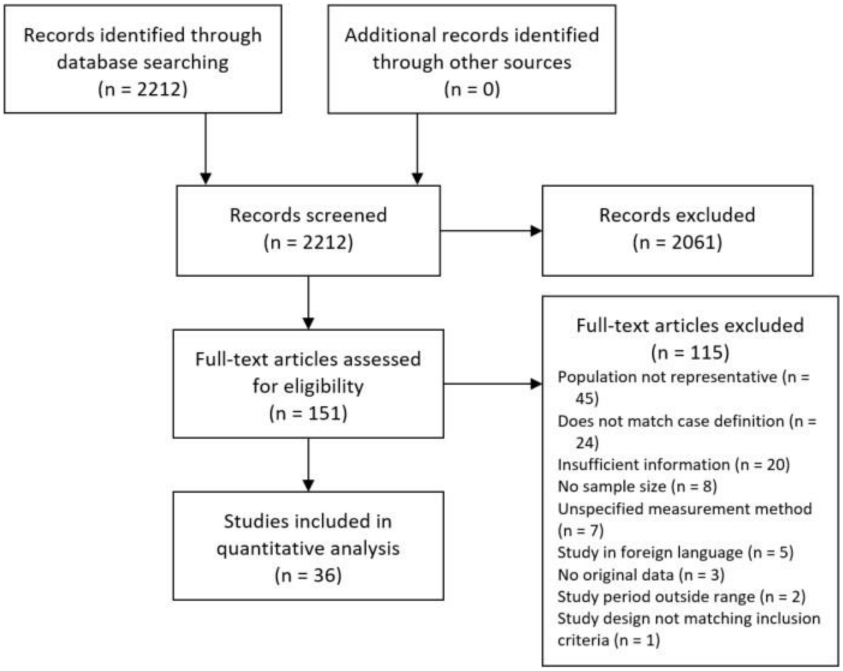

Modelling strategy

In GBD 2017, we had calculated kernicterus prevalence with the same approach used to calculate EHB prevalence – in this case by multiplying EHB prevalence by literature-derived scalars representing the proportion of EHB cases that develop kernicterus. Starting in GBD 2019, using the data that were extracted from our systematic reviews described above, we instead modelled kernicterus probability as a function of HAQ Index and initial total serum bilirubin level (TSB), and generated location-year-specific kernicterus proportions. These proportions were used to calculate kernicterus from non-Rhesus EHB, For Rh disease EHB; however, we continued to use a pooled value from literature of 0.072 (0.038, 0.112)[5–8] for the proportion of cases who develop kernicterus.

To go into more detail about the modelling approach to estimate these new location-year-specific kernicterus proportions, we used the extracted data to develop a monotonic cubic spline model in MR-BRT, with 10% trimming and covariates for the HAQ Index and TSB, with a spline on the HAQ Index. The model results are shown in Figure 8 below, the first figure in logit space and the second figure in linear space. We used from this model the probability of kernicterus when initial TSB is 25 mg/dL, which is the minimum EHB threshold, to represent the probability of kernicterus among those with EHB, pairing with location-year-specific HAQ Index values.

Figure 8. Predicted kernicterus proportion for total serum bilirubin levels as a function of HAQ Index

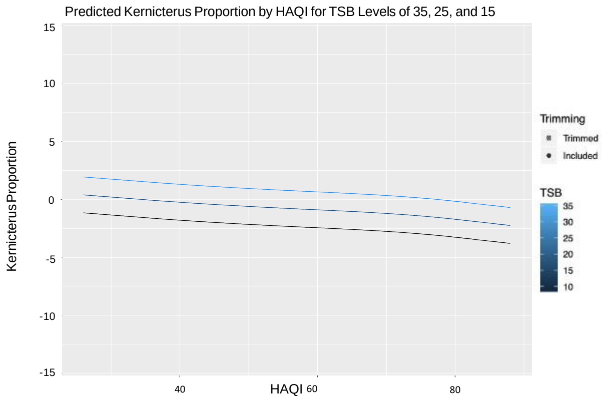

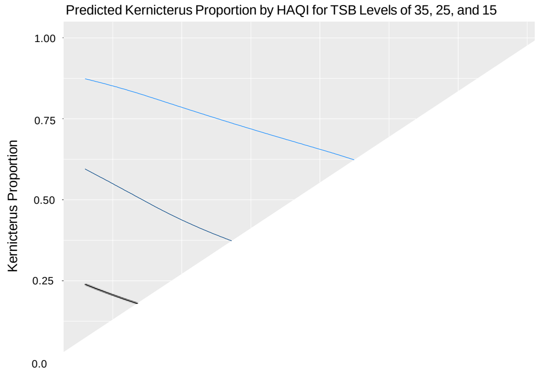

Finally, we calculated total kernicterus prevalence across aetiologies in the 0–6-day period by summing kernicterus prevalence from its four aetiologies: Rh disease, G6PD, preterm birth complications, and other causes. In previous rounds we excluded preterm birth complications from this calculation because we assumed that all disability due to preterm birth complications was already captured in our preterm models; however, this led to inconsistencies with our calculations of EHB. Thus, it was included in GBD 2021 according to the following equation.

$$\begin{aligned}\text{Total Kernicterus prevalence} &= (\text{Kernicterus prevalence due to Rh disease}) \\ &+ (\text{Kernicterus prevalence due to G6PD}) \\ &+ (\text{Kernicterus prevalence due to preterm birth complications}) \\ &+ (\text{Kernicterus prevalence due to other causes})\end{aligned}$$

The inclusion of preterm birth complications for GBD 2021 led to a very modest increase in total kernicterus prevalence.

Step 5: Kernicterus prevalence at all ages (moderate/severe impairment)

Input data

Standardised mortality ratios of cerebral palsy were used as input data to model the prevalence of kernicterus for ages older than the neonatal period based on the assumption that acute bilirubin encephalopathy (ie, kernicterus) is one of the reasons that young children go on to develop cerebral palsy. These data were used across all four neonatal causes and for many other causes in the GBD study.

We ran a meta-analysis for a 0–19 age group and a 20–99 age group, and the SMR values were converted to EMR using the formula:

$$EMR = (\text{locaton-sex-age-specific } \mathit{all\text{-}cause\ mortality\ rate}) * (\text{age-specific } SMR - 1)$$

Modelling strategy

To model moderate-severe (kernicterus) prevalence at older ages, a DisMod-MR 2.1 model was run on the existing moderate-severe prevalence estimate (eg, prevalence in the early neonatal period), and on excess mortality estimates derived from the standard mortality ratios (SMR) of cerebral palsy. Remission and incidence were set to zero. The input dataset was entirely complete as every location had an input datum for early neonatal prevalence as well as specific values for EMR at every age-location-sex-year, so we did not specify location-level covariates and the model was set to not pass any priors for any parameter during the estimation cascade, functionally meaning the final estimate age-location-sex-year was not informed by any adjacent locations or years.

<u>Step 6: EHB without kernicterus (mild impairment)</u>

We represent mild impairment as impairment due to having EHB alone (no progression to kernicterus). To estimate this, we summed EHB prevalence across all four aetiologies, and then subtracted the summed kernicterus prevalence across the four aetiologies (excluding preterm). This was estimated for the 0–6-day and 7–27-day age groups. Prevalence of EHB without kernicterus from the post-neonatal period onward was assumed to be zero.

Step 7: Split into health states and pair with disability weights to calculate YLDs

The kernicterus estimates were split into 14 sequelae corresponding to moderate and severe disability, and the EHB without kernicterus estimate was associated with one sequela with mild disability. The proportions for each moderate/severe sequela were extracted from a study by Badawi and colleagues[1] and are listed in the table below in descending order. These proportions were also used to split impairments into sequelae across the other neonatal causes.

Table 25. Health states of haemolytic disease and other neonatal jaundice by severity

Sequelae of neonatal encephalopathy	EHB	Kernicterus
Severe abdominopelvic problem	1.000	
Moderate motor only		0.173
Moderate motor impairment + epilepsy		0.100
Moderate motor impairment + blindness		0.018
Moderate motor impairment + blindness + epilepsy		0.009
Moderate motor impairment + blindness + cognitive impairment		0.032
Moderate motor impairment + epilepsy + cognitive impairment		0.183
Moderate motor impairment + blindness + epilepsy + cognitive impairment		0.017
Severe motor only		0.152
Severe motor impairment + epilepsy		0.033
Severe motor impairment + blindness		0.006
Severe motor impairment + blindness + epilepsy		0.003
Severe motor impairment + blindness + cognitive impairment		0.038
Severe motor impairment + epilepsy + cognitive impairment		0.216
Severe motor impairment + blindness + epilepsy + cognitive impairment		0.020

Step 8: Split into health states and pair with disability weights to calculate YLDs

Each sequela was associated with a unique GBD health state and paired with corresponding disability weights to calculate YLDs. The health states that were used for neonatal encephalopathy are largely the same as the health states for other neonatal causes (see Table 26. Disability weights and lay descriptions by health state). Some health states were combined to calculate the burden of certain sequelae.

Table 26. Disability weights and lay descriptions by health state

Health state	Description	Disability weight
Motor impairment, mild	Has some difficulty in moving around but is able to walk without help.	0.01 (0.005–0.019)
Motor impairment, moderate	Has some difficulty in moving around, and difficulty in lifting and holding objects, dressing and sitting upright, but is able to walk without help.	0.061 (0.040–0.089)
Motor impairment, severe	Is unable to move around without help, and is not able to lift or hold objects, get dressed or sit upright.	0.402 (0.268–0.545)
Motor plus cognitive impairments, mild	Has some difficulty moving around but is able to walk without help. The person is slow in learning at school. As an adult, the person has some difficulty doing complex or unfamiliar tasks but otherwise functions independently.	0.031 (0.018–0.050)
Motor plus cognitive impairments, moderate	Has some difficulty in moving around, holding objects, dressing, and sitting upright, but can walk without help. The person has low intelligence and is slow in learning to speak and to do simple tasks.	0.203 (0.134–0.290)
Motor plus cognitive impairments, severe	Cannot move around without help, and cannot lift or hold objects, get dressed or sit upright. The person also has very low intelligence, speaks few words, and needs constant supervision and help with all daily activities.	0.542 (0.374–0.702)
Distance vision blindness	Is completely blind, which causes great difficulty in some daily activities, worry and anxiety, and great difficulty going outside the home without assistance.	0.187 (0.124–0.260)
Epilepsy, less severe (seizures < once per month)	Has sudden seizures two to five times a year, with violent muscle contractions and stiffness, loss of consciousness, and loss of urine or bowel control.	0.263 (0.173–0.367)
Epilepsy, severe (seizures ≥ once per month)	Has sudden seizures one or more times each month, with violent muscle contractions and stiffness, loss of consciousness, and loss of urine or bowel control. Between seizures the person has memory loss and difficulty concentrating.	0.552 (0.375–0.71)
Abdominopelvic problem, severe (proxy for EHB without kernicterus)	Has severe pain in the belly and feels nauseous. The person is anxious and unable to carry out daily activities.	0.324 (0.220–0.442)

Neonatal sepsis and other neonatal infections

Flowchart

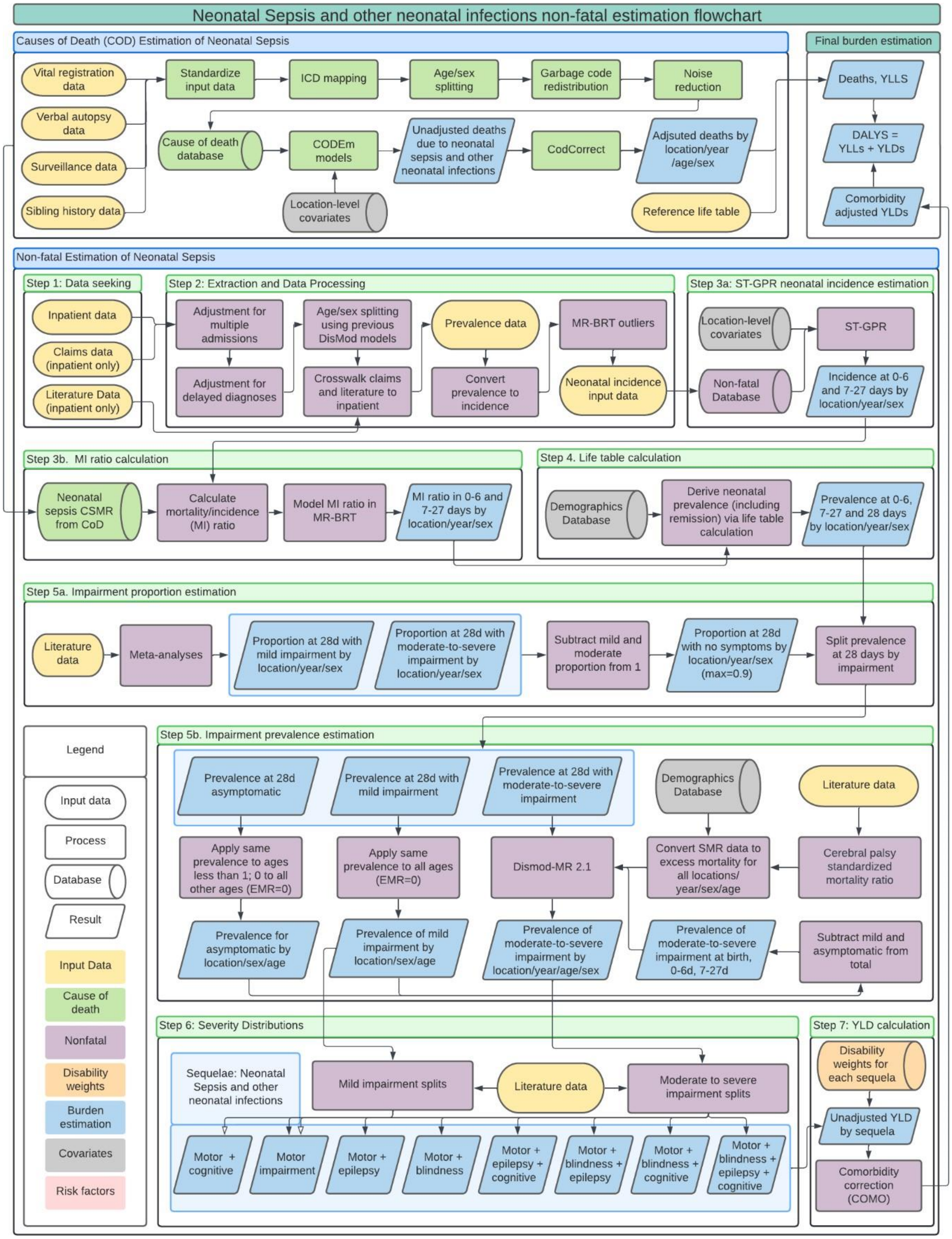

Case definition

Neonatal sepsis and other neonatal infections are infections during the neonatal period that advance to a systemic bloodstream infection (sepsis) and infections that occur during the neonatal period that are not already modelled separately in the GBD.

Modelling strategy

Modelling the non-fatal burden of neonatal sepsis and other neonatal infections occurs in six main steps:

Table 27. Analytical steps in estimation of YLDs due to neonatal sepsis and other neonatal infections

Step	Summary of modelling strategy
1	Model neonatal sepsis incidence in the early and late neonatal periods in ST-GPR
2	Estimate neonatal sepsis prevalence in the early neonatal period, late neonatal period, and at exactly 28 days using a life table algorithm
3	Meta-analyse asymptomatic, mild, and moderate-severe impairment proportions at 28 days, then split prevalence at 28 days by severity of impairment
4	Model impairment prevalence at younger and older ages based on 28-day impairment prevalence
5	Split mild and moderate-severe impairment prevalence into sequelae
6	Apply disability weights to each sequela to calculate YLDs

Table 28. Input data – neonatal sepsis and other neonatal infections

Measure	Countries with data	New sources	Total sources
Incidence	47	35	328
Other (proportion and case fatality rate)	18	0	17

Step 1: Model neonatal sepsis incidence in the early and late neonatal periods in ST-GPR

In previous GBD rounds we used DisMod MR 2.1 to model neonatal sepsis prevalence in the early and late neonatal periods. In GBD 2021, we amended this modelling process to use spatiotemporal Gaussian process regression (ST-GPR) to model the incidence of neonatal sepsis in these age groups. We then used these as inputs in a life table algorithm to produce estimates of neonatal sepsis prevalence in the early neonatal period, late neonatal period, and at 28 days.

Input data

We extracted data on prevalence and incidence of neonatal sepsis and other neonatal infections from literature and clinical informatics data. All prevalence data were then converted to incidence before being input to ST-GPR.

A systematic literature review for neonatal sepsis was last completed for GBD 2015. The PubMed database was searched using the following search string:

> (("infant"[Title/Abstract] OR "newborn"[Title/Abstract] OR "newborn infant"[Title/Abstract]) AND ("neonatal sepsis"[All Fields] OR "neonatal septicaemia"[All Fields] OR "neonatal meningitis"[All Fields] OR "early sepsis"[All Fields] OR "early septicaemia"[All Fields] OR "tetanus"[All Fields] OR "meningitis"[All Fields] OR "sepsis"[All Fields])) AND ("2012"[PDAT] : "3000"[PDAT]) AND "humans"[MeSH Terms]

To be included, published data sources had to report on specific infections, or groups of infections, and provide diagnostic criteria for how cases were identified. The exclusion criteria were studies that did not provide primary data on epidemiological parameters (eg, a commentary piece), non-representative studies (eg, only high-risk pregnancies, nosocomial infection rates, preterm infants, ICU populations), and review articles. We did not find any studies that reported on all neonatal infections, only sepsis.

Clinical informatics data (hospital and claims) formed the bulk of the input data for the neonatal sepsis envelope model. Only inpatient data were included from these datasets because we believe they are more representative of the true prevalence of neonatal sepsis than outpatient data; infants with neonatal sepsis in the countries from which hospital data were available are almost sure to be admitted to the hospital, whereas outpatient data are more likely to capture repeated visits by the same child as they grow. Clinical data processing is described separately.

Data processing

In GBD 2021, clinical informatics data were processed to reflect the discrete under-5 age groups within the GBD study. Because many sources are not linked across years, these splits led to implausible age patterns and an under-ascertainment of cases at birth. Based on the assumption that all cases present in older age groups in the <1-year GBD age groups would necessarily have been present at birth, we adjusted the under-1 inpatient data to back-add cases that first "appeared" at older ages to the numerator of the prevalence in younger ages.

Starting in GBD 2019, we applied empirical age ratios from previous DisMod-MR 2.1 models to disaggregate observations that did not entirely fit in one GBD age category. We calculated these ratios by dividing the result for a specific age and sex by the result for the aggregate age and sex specified in a given observation. It is our intention to update this splitting process annually.

In GBD 2021, our reference case definition for neonatal sepsis data was inpatient hospital data. We crosswalked data from inpatient claims and literature data to the reference definition using MR-BRT before modelling in ST-GPR. The adjustment factors applied were as follows:

Table 29. MR-BRT crosswalk adjustment factors for neonatal sepsis and other neonatal infections

Data input	Reference or alternative case definition	Gamma	Beta coefficient, log (95% CI)*	Adjustment factor**
Hospital data	Reference	--	--	--
Claims data	Alternate	0.74	0.25 (–0.44 to 0.94)	1.28 (0.64–2.56)
Literature data	Alternate	5.83	–1.64 (–4.99 to 1.71)	0.19 (0.01–5.53)

**MR-BRT crosswalk adjustments can be interpreted as the factor the alternative case definition is adjusted by to reflect what it would have been had it been measured using the reference case definition. If the log/logit beta coefficient is negative, then the alternative is adjusted up to the reference. If the log/logit beta coefficient is positive, then the alternative is adjusted down to the reference.*
***The adjustment factor column is the exponentiated beta coefficient. For log beta coefficients, this is the relative rate between the two case definitions. For logit beta coefficients, this is the relative odds between the two case definitions.*

Lastly, because of significant residual heterogeneity in input data, especially from clinical administrative sources, we used an MR-BRT model to identify outliers in the data. We log-transformed our incidence data and calculated standard errors using the delta method and fit a cubic spline on the Healthcare Access and Quality (HAQ) Index with fixed effects on age group, a prior of decreasing monotonicity, and a 30% trimming parameter. All trimmed data were marked as outliers in the model.

Modelling strategy

Due to inconsistencies in estimates of neonatal sepsis prevalence between GBD rounds when modelling in DisMod MR-2.1, we significantly amended our modelling approach for GBD 2021. In previous GBD rounds, we ran a DisMod MR-2.1 model to estimate prevalence of neonatal sepsis in the early and late neonatal periods directly. We then interpolated these results to estimate the prevalence at 28 days. For

GBD 2021, we amended this process to model the incidence of neonatal sepsis in the early and late neonatal periods and use a life table algorithm to calculate prevalence in these age groups and at 28 days. Unlike other neonatal cause models, we did not estimate birth prevalence for neonatal sepsis and other neonatal infections.

We first modelled the incidence of neonatal sepsis and other neonatal infections in the early and late neonatal periods using spatiotemporal Gaussian process regression (ST-GPR), a three-step modelling procedure for generating estimates for every location, year, age, and sex in the GBD study. The first step of the ST-GPR process is an ensemble linear mixed-effects regression of our data on a set of potentially predictive covariates taken from the GBD study covariates database. We tested every combination of these covariates in individual, sex-specific mixed-effects linear regressions with nested random effects at the super-region, region, and location levels. We then evaluated and ranked each of these sub-models by their out-of-sample root-mean-squared error (RMSE). Finally, to produce initial estimates for every location, year, age, and sex in the analysis, we averaged the 50 top-performing models where the estimated coefficients were 1) statistically significant at $p < 0.05$, and 2) in the expected direction. We tested the following covariates in the ensemble prior: lag-distributed income per capita, Sociodemographic Index, Healthcare Access and Quality Index, unsafe water SEV, unsafe sanitation SEV, maternal care and immunisation index, livebirths among women aged 35+ years, preterm birth SEV, low birthweight SEV, short gestation SEV, smoking SEV, mortality due to war and conflict, and neonatal CSMR.

The second, spatiotemporal smoothing step of ST-GPR calculates the residual between our stage 1 regression estimate and each of our observed datapoints and then smooths this residual, drawing strength over space, age, and time and producing a revised stage 2 estimate for every location, year, age, and sex. The third step of ST-GPR is a Gaussian process regression, using the stage 2 estimates as a prior and the observed datapoints and their variance to 1) further smooth the residual between the stage 2 predictions and observed data and produce a final mean estimate for each location, year, age, and sex, and 2) estimate uncertainty around this mean estimate, quantified by taking 1000 draws from the posterior Gaussian process. More detailed information on the ST-GPR modelling process can be found in the main text methods appendix.

Step 2: Estimate neonatal sepsis prevalence in the early neonatal period, late neonatal period, and at exactly 28 days using a life table algorithm

Mortality/incidence ratio modelling

Our life table algorithm requires mortality-to-incidence (MI) ratio estimates for every location, year, age, and sex. To generate these estimates, we modelled the MI ratio in MR-BRT, using MI ratio data derived from our incidence estimates and modelled cause-specific mortality rates. We log-transformed all MI ratio data and calculated standard errors using the delta method. We fit a monotonically decreasing cubic spline on the Healthcare Access and Quality Index with fixed effects on age and sex and a 20% trimming parameter. From this model we generated 1000 draws of estimated MI ratio for every location, year, age, and sex included in the analysis.

Life table algorithm

The next step in our modelling process is a life table algorithm that uses estimates of population at birth and in the early and late neonatal periods, incidence of neonatal sepsis in the early and late neonatal periods, mortality-to-incidence ratios, remission estimates, and mortality data from the GBD mortality

analysis to generate prevalence estimates in the early and late neonatal age groups and at exactly 28 days for every location, year, and sex.

Early neonatal age group calculations

We first calculated incident cases of neonatal sepsis in the early neonatal period using the equation:

$$cases_{enn} = births * (1 - e^{-Incenn*t_{0-7}})$$

where Inc_{Enn} is the modelled incidence rate in the early neonatal period and $t_{0\text{-}7}$ is the number of days in the early neonatal period. We then calculated remitted cases in the early neonatal period as

$$remitted\ cases_{enn} = cases_{enn} * (1 - e^{-Remrate*t0-6})$$

where *Remrate* is the remission rate. We generated 1000 draws of the remission rate from a normal distribution with mean 40 and standard deviation of 5.1, approximating a mean remission rate of 40 with confidence interval (30–50). Next, we calculated deaths among cases in the early neonatal period as

$$deaths_{enn} = cases_{enn} * MIratio_{enn}$$

Where $MIratio_{enn}$ is the modelled MI ratio for a given location, year, age, and sex. Finally, we calculated the population vulnerable to infection after the neonatal period as the population at birth minus all-cause deaths in the early neonatal period and the surviving cases of neonatal sepsis from the early neonatal period (which we assume cannot be re-infected):

$$population_{7days} = births - (all\ cause\ mortality_{enn} + survivors_{enn})$$

Late neonatal age group calculations

The equations for the late neonatal period calculations mirror those used in the early neonatal period, beginning with the calculated population at 7 days described above. The equations follow the same order as in the early neonatal period:

$$cases_{lnn} = population_{7days} * (1 - e^{-Inclnn*t_{7-27}})$$
$$remitted\ cases_{lnn} = cases_{lnn} * (1 - e^{-Remrate*t7-27})$$
$$deaths_{lnn} = cases_{lnn} * MIratio_{lnn}$$

28 days calculations

Finally, to estimate prevalence at exactly 28 days, we first calculated the population at 28 days as:

$$population_{28days} = population_{7days} + survivors_{enn} - all\ cause\ mortality_{lnn}$$

Where $population_{7days}$ is the calculated population at the end of the early neonatal period, $survivors_{enn}$ are the surviving early neonatal cases of sepsis (which are not included in the $population_{7days}$ estimate), and $all\ cause\ mortality_{lnn}$ is the total all-cause deaths in the late neonatal period. We then calculated prevalence at 28 days as:

$$prevalence_{28days} = \frac{survivors_{enn} + survivors_{lnn}}{population_{28days}}$$

Step 3: Model impairment proportions at 28 days, then split prevalence at 28 days by severity of impairment

Infants who survive neonatal sepsis may go on to experience long-term disability or impairment. We categorised impairment for neonatal sepsis and other neonatal infections into three severities: asymptomatic, mild, and moderate-to-severe impairment.

Input data

Data on the proportion of cases of neonatal sepsis that go on to develop mild impairment and moderate-to-severe impairment were extracted from a systematic literature review that was last completed in GBD 2013 and updated in GBD 2015. The same search string described above was used to identify impairment data.

Modelling strategy

Using mild impairment proportion and moderate-to-severe impairment proportion data, we ran separate meta-analyses to generate estimates of both parameters. The remainder of 1 – (mild proportion + moderate-severe proportion) was assigned as the asymptomatic proportion.

Table 30. Proportion of mild and moderate-to-severe impairment of neonatal sepsis and other neonatal infections at 28 days

Parameter	Estimate (95% UI)
Mild impairment proportion	10.2% (7.2–12.9)
Moderate-to-severe impairment proportion	4.3% (2.5–6.0)

Figure 9. Mild impairment meta-analysis

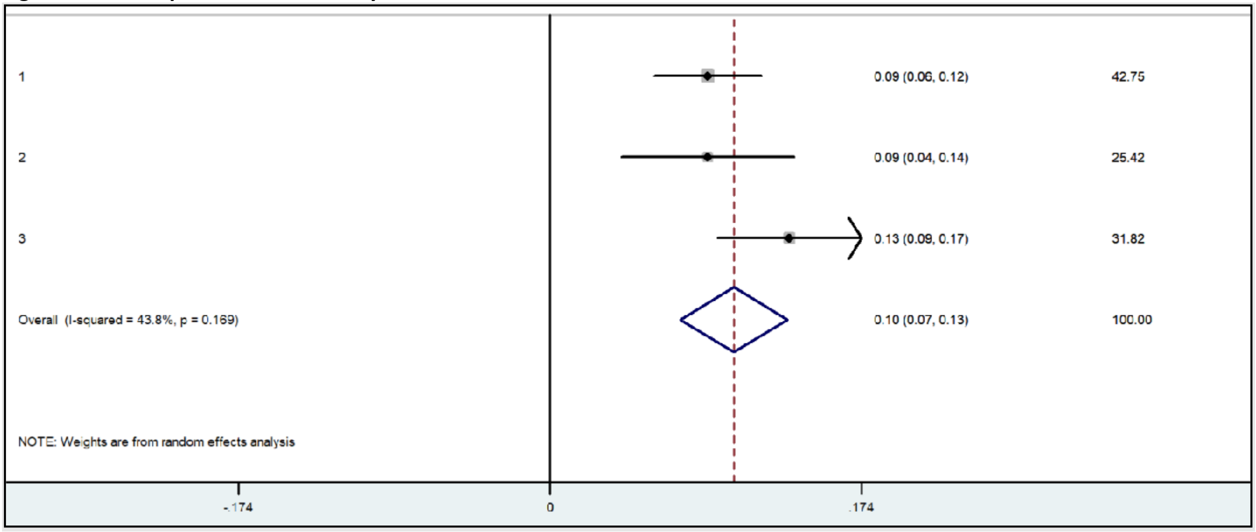

Figure 10. Moderate-to-severe impairment meta-analysis

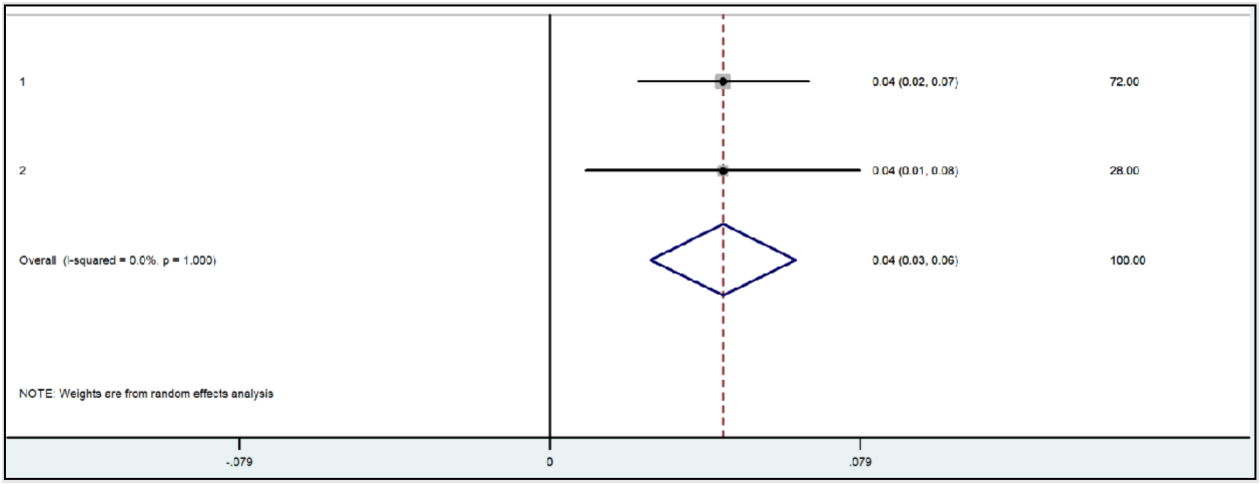

We multiplied our estimated impairment proportions by the prevalence at 28 days calculated from our life table algorithm in Step 2 to generate impairment-specific prevalence estimates. Asymptomatic prevalence was extended to other ages based on the assumption that asymptomatic prevalence at 28 days is the same as at early neonatal, late neonatal, and post-neonatal, and that there is no burden and therefore no asymptomatic prevalence after 1 year of age. Mild prevalence was extended to other ages based on the assumption that the mild prevalence at 28 days is the same as the mild prevalence at all other ages. This assumption is grounded in the lack of excess mortality and remission among those who develop mild neonatal sepsis (ie, no one can develop the disease after the neonatal period, no one dies from it, and no one recovers from it, so the number of cases is constant across age).

Step 4: Model long-term impairment prevalence at all ages

Input data

Standardised mortality ratios of cerebral palsy were used as input data to model the prevalence of neonatal sepsis for ages older than the neonatal period based on the assumption that severe sepsis is one of the reasons that young children go on to develop cerebral palsy. These data were used across all four neonatal causes and for many other causes in the GBD study. We ran a meta-analysis for a 0–19 age group and a 20–99 age group, and the SMR values were converted to EMR using the formula:

$$EMR = (location\text{-}sex\text{-}age\text{-}specific\,all\text{-}cause\,mortality\,rate) * (age\text{-}specific\,SMR - 1)$$

Modelling strategy

To estimate the prevalence of moderate-severe impairment at other ages, we needed to account for excess mortality. Because there is excess mortality, the number of cases of moderate-severe impairment declines with age. The sum of asymptomatic and mild impairment in the early and late neonatal periods was subtracted from the neonatal sepsis envelope prevalence estimates (Step 1) in the early and late neonatal periods to estimate moderate-severe impairment. This reflects the assumption that all deaths in the early and late neonatal period were among those with moderate-severe impairment, and all newborns who developed asymptomatic or mild neonatal sepsis did not experience excess mortality.

To model moderate-severe prevalence at older ages, a DisMod-MR 2.1 model was run on the existing moderate-severe prevalence estimate (eg, prevalence in the early neonatal period), and on excess mortality estimates derived from the standard mortality ratios (SMR) of cerebral palsy. Remission and incidence were set to zero. The input dataset was entirely complete as every location had an input

datum for early neonatal prevalence as well as specific values for EMR at every age-location-sex-year, so we did not specify location-level covariates and the model was set to not pass any priors for any parameter during the estimation cascade, functionally meaning the final estimate age-location-sex-year was not informed by any adjacent locations or years.

Step 5: Split mild and moderate-severe impairment prevalence into sequelae

The mild impairment estimates are split into two sequelae, and the moderate-to-severe impairment estimates are split into 14 sequelae. The mild sequelae were derived by splitting the mild prevalence equally. The proportions for each moderate/severe sequela were extracted from a study by Badawi and colleagues[1] and are listed in the table below in descending order. These proportions were also used to split impairments into sequelae across the other neonatal causes.

Table 31. Health states by severity

Sequelae of neonatal sepsis and other neonatal infections	Mild	Moderate-severe
Mild motor impairment	0.500	
Mild motor plus cognitive impairment	0.500	
Moderate motor only		0.173
Moderate motor impairment + epilepsy		0.100
Moderate motor impairment + blindness		0.018
Moderate motor impairment + blindness + epilepsy		0.009
Moderate motor impairment + blindness + cognitive impairment		0.032
Moderate motor impairment + epilepsy + cognitive impairment		0.183
Moderate motor impairment + blindness + epilepsy + cognitive impairment		0.017
Severe motor only		0.152
Severe motor impairment + epilepsy		0.033
Severe motor impairment + blindness		0.006
Severe motor impairment + blindness + epilepsy		0.003
Severe motor impairment + blindness + cognitive impairment		0.038
Severe motor impairment + epilepsy + cognitive impairment		0.216
Severe motor impairment + blindness + epilepsy + cognitive impairment		0.020

Step 6: Use disability weights to calculate YLDs

Each sequela is associated with a health state, which is used to calculate YLDs. The health states used for neonatal encephalopathy are largely the same as the health states for other neonatal causes (see Table 32. Disability weights and lay descriptions by health state for list). Some health states were combined to calculate the burden of certain sequelae.

Table 32. Disability weights and lay descriptions by health state

Health state	Description	Disability weight
Motor impairment, mild	Has some difficulty in moving around but is able to walk without help.	0.01 (0.005–0.019)
Motor impairment, moderate	Has some difficulty in moving around, and difficulty in lifting and holding objects, dressing, and sitting upright, but is able to walk without help.	0.061 (0.040–0.089)
Motor impairment, severe	Is unable to move around without help, and is not able to lift or hold objects, get dressed or sit upright.	0.402 (0.268–0.545)
Motor plus cognitive impairments, mild	Has some difficulty moving around but is able to walk without help. The person is slow in learning at school. As an adult, the person has some difficulty doing complex or unfamiliar tasks but otherwise functions independently.	0.031 (0.018–0.050)

Motor plus cognitive impairments, moderate	Has some difficulty in moving around, holding objects, dressing, and sitting upright, but can walk without help. The person has low intelligence and is slow in learning to speak and to do simple tasks.	0.203 (0.134–0.290)
Motor plus cognitive impairments, severe	Cannot move around without help, and cannot lift or hold objects, get dressed or sit upright. The person also has very low intelligence, speaks few words, and needs constant supervision and help with all daily activities.	0.542 (0.374–0.702)
Distance vision blindness	Is completely blind, which causes great difficulty in some daily activities, worry and anxiety, and great difficulty going outside the home without assistance.	0.187 (0.124–0.260)
Epilepsy, less severe (seizures < once per month)	Has sudden seizures two to five times a year, with violent muscle contractions and stiffness, loss of consciousness, and loss of urine or bowel control.	0.263 (0.173–0.367)
Epilepsy, severe (seizures ≥ once per month)	Has sudden seizures one or more times each month, with violent muscle contractions and stiffness, loss of consciousness, and loss of urine or bowel control. Between seizures the person has memory loss and difficulty concentrating.	0.552 (0.375–0.71)
Abdominopelvic problem, severe (proxy for EHB without kernicterus)	Has severe pain in the belly and feels nauseous. The person is anxious and unable to carry out daily activities.	0.324 (0.220–0.442)

Other neonatal disorders

In addition to the neonatal disorders described above, there are many diverse types of neonatal disorders with a range of severities and associated sequelae. Because these other neonatal disorders are diverse in their underlying causes and risk factors as well as in their associated health outcomes, modelling them together in a DisMod-MR 2.1 model would not produce reliable estimates of prevalence or excess mortality. Instead, we calculated the YLDs caused by other neonatal disorders directly using a YLD/YLL ratio.

We calculated the ratio of YLDs to YLLs across the specified neonatal disorders for which non-fatal outcomes were modelled, using YLL estimates from the GBD 2021 cause of death (CoD) analysis. We then multiplied this YLD/YLL ratio by the YLL estimate for other neonatal disorders from the GBD 2021 CoD analysis, providing us with an estimate of the YLDs associated with other neonatal disorders.

A full list of the ICD codes classified as other neonatal disorders in the mortality analysis are provided below. The codes that made up the largest proportion of deaths were P52: intracranial nontraumatic haemorrhage of newborn, P29: cardiovascular disorders originating in the perinatal period, and P00: newborn (suspected to be) affected by maternal conditions that may be unrelated to present pregnancy.

ICD9 codes:

760, 760.0-760.6, 760.8-760.9, 761, 761.2-761.6, 764, 766, 770, 771, 772, 772.0, 775, 775.0, 775.4-775.9, 776, 776.0-776.5, 776.7-776.9, 777, 777.0-777.4, 777.7-777.9, 778, 779, 779.3, 779.6-779.8

ICD10 codes:

P00, P01, P01.2-01.6, P01.8-01.9, P04, P04.0-04.2, P04.5-04.6, P04.8-04.9, P05, P08, P09, P19, P29, P50, P51, P52, P53, P54, P60, P61, P61.0-61.1, P61.3-61.6, P61.8-61.9, P70, P70.1, P70.3-70.4, P70.8-70.9, P71, P72, P74, P75, P76, P78, P80, P81, P83, P84, P92, P93, P94, P96, P96.3-96.4, P96.8

References

1 Badawi N, Felix JF, Kurinczuk JJ, *et al.* Cerebral palsy following term newborn encephalopathy: a population-based study. *Dev Med Child Neurol* 2005; **47**: 293–8.

2 Bhutani VK, Zipursky A, Blencowe H, *et al.* Neonatal hyperbilirubinemia and Rhesus disease of the newborn: incidence and impairment estimates for 2010 at regional and global levels. *Pediatr Res* 2013; **74 Suppl 1**: 86–100.

3 Zipursky A. The universal prevention of Rh immunization. *Clin Obstet Gynecol* 1971;14:869–84.

4 Clarke CA: Prevention of Rhesus iso-immunization. *Seminars in Hematology* 6:201, 1969.

5 Walker W. Haemolytic Disease of the Newborn. In: *Recent Advances in Paediatrics*, 4th edn. London, UK: JA Churchill, 1970.

6 Vaughan VC. Kernicterus in erythroblastosis fetalis. *J Pediatr* 1946; **29**: 462–73.

7 Mollison PL, Cutbush M. Exchange transfusion in haemolytic disease of the newborn. *Lancet* 1948; **2**: 522–7.

8 Hsia DY, Allen FH Jr, Gellis SS, Diamond LK. Erythroblastosis fetalis. VIII. Studies of serum bilirubin in relation to kernicterus. *N Engl J Med* 1952;247:668–71.

9 Ebbesen F. Recurrence of kernicterus in term and near-term infants in Denmark. *Acta Paediatr* 2000;89:1213–7.

10 Ebbesen F, Andersson C, Verder H, et al. Extreme hyperbilirubinaemia in term and near-term infants in Denmark. *Acta Paediatr* 2005;94:59–64.

11 Ebbesen F, Bjerre JV, Vandborg PK. Relation between serum bilirubin levels =450 µmol/L and bilirubin encephalopathy; a Danish population-based study. *Acta Paediatr* 2012;101:384–9.

12 Sgro M, Campbell D, Barozzino T, Shah V. Acute neurological findings in a national cohort of neonates with severe neonatal hyperbilirubinemia. *J Perinatol* 2011;31:392–6.

13 Sgro M, Campbell D, Shah V. Incidence and causes of severe neonatal hyperbilirubinemia in Canada. *CMAJ* 2006;175:587–90.

14 Sgro M, Campbell DM, Kandasamy S, Shah V. Incidence of chronic bilirubin encephalopathy in Canada, 2007-2008. *Pediatrics* 2012;130: e886–90.

15 Vandborg PK, Hansen BM, Greisen G, Jepsen M, Ebbesen F. Follow-up of neonates with total serum bilirubin levels = 25mg/dL: a Danish population-based study. *Pediatrics* 2012;130:61–6.

Neoplasms

This general framework for the GBD 2021 cancer estimation applies to all malignant neoplasms (ie, cancers) except for non-melanoma skin cancer (including basal cell carcinoma and squamous cell carcinoma); benign and in situ neoplasms (including

intestinal; cervical and uterine; and other benign neoplasms); and myelodysplastic, myeloproliferative, and other haemopoietic neoplasms.

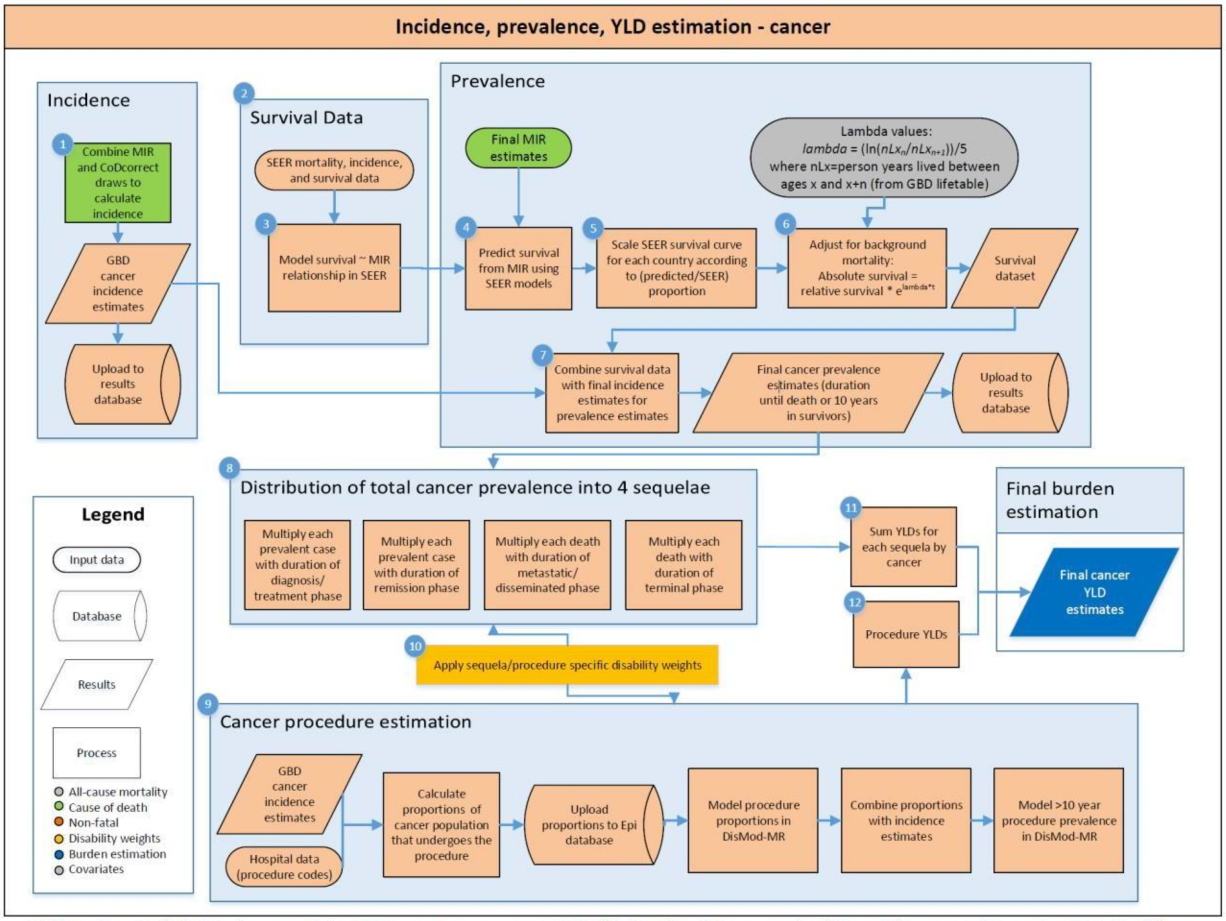

Abbreviations: DisMod-MR, disease model – Bayesian meta-regression; GBD, Global Burden of Disease study; MIR, mortality-to-incidence ratio; SEER, Surveillance, Epidemiology, and End Results Program; YLD, years lived with disability.

Input data and methodological appendix

Case definition

For GBD 2021, incidence, prevalence, and disability are estimated for all cancers and benign neoplasms as defined in ICD-10 (C00–D49). The associated ICD codes for neoplasms estimated for GBD 2021 are listed elsewhere in the GBD summary papers. Prevalence for cancers are estimated for a maximum of ten years after incidence, as in GBD 2013, GBD 2015, GBD 2016, GBD 2017, and GBD 2019.[1–5] Prevalence extending beyond the ten-year period is only estimated for permanent sequelae resulting from five treatment-related surgical procedures (cystectomy, laryngectomy, mastectomy, prostatectomy, and stoma).

To estimate disability for each cancer, total prevalence is split into four sequelae: 1. diagnosis and primary therapy phase; 2. controlled phase; 3. metastatic phase; and 4. terminal phase. The diagnosis and primary therapy phase represents the time from the onset of symptoms to the end of treatment. The controlled phase represents the time between finishing primary treatment and the earliest of either: cure (defined as recurrence- and progression-free survival after ten years); death from another cause; or progression to the metastatic phase. The metastatic phase represents the time period of intensive treatment for metastatic disease, as determined for each cancer by evaluating data from SEER[6] (Surveillance, Epidemiology, and End Results Program) averages (Table 3). The terminal phase represents the one-month period prior to death. Each of these four sequelae has a separate disability weight, which are the same across cancer types (Error! Reference source not found.). Because of the long-term disability associated w ith certain treatment-related procedures, additional disability beyond these four sequelae is estimated for five cancers: breast cancer (disability due to mastectomy), larynx cancer (disability due to laryngectomy), colon and rectum cancer (disability due to stoma), bladder cancer (disability due to incontinence from cystectomy), and prostate cancer (disability due to either incontinence or impotence from prostatectomy).

Input data

Cancer incidence is directly estimated from cancer mortality estimates using mortality-to-incidence ratios (MIRs). Data sources for cancer mortality are described elsewhere in the GBD summary articles. Data sources used to estimate the proportion of cancer patients undergoing surgical procedures and to adjust procedure sequelae will be listed below.

Table 1a. Data inputs for neoplasms morbidity modelling by parameter

Cause	Prevalence sources	Incidence sources	Deaths sources	All measures sources
Neoplasms	344	5599	4856	10 289
Lip and oral cavity cancer	3	3448	4191	7232
Nasopharynx cancer	3	3569	3736	7229
Other pharynx cancer	3	3593	4101	7288
Oesophageal cancer	3	3697	4538	7708
Stomach cancer	3	3768	4527	7708
Colon and rectum cancer	3	3779	4635	7805
Liver cancer	3	3804	4235	7788

Hepatoblastoma	3	3804	4235	7788
Gallbladder and biliary tract cancer	3	3632	4141	7275
Pancreatic cancer	3	3729	4281	7442
Larynx cancer	3	3695	4480	7650
Tracheal, bronchus, and lung cancer	3	3745	4654	7776
Malignant neoplasm of bone and articular cartilage	3	3529	3663	7130
Malignant skin melanoma	3	3523	3772	7184
Non-melanoma skin cancer	3	1462	3645	5110
Mesothelioma	3	1568	2083	3625
Neuroblastoma and other peripheral nervous cell tumours	3	2578	2815	5355
Soft tissue and other extraosseous sarcomas	3	2699	2837	5476
Breast cancer	3	3757	4677	7820
Cervical cancer	3	3681	4501	7630
Uterine cancer	3	3691	4498	7638
Ovarian cancer	3	3697	4274	7413
Prostate cancer	3	3749	4596	7703
Testicular cancer	3	3505	3590	6999
Kidney cancer	3	3499	4218	7215
Bladder cancer	3	3866	4129	7570
Eye cancer	3	2712	2863	5511
Retinoblastoma	3	2499	2796	5239
Other eye cancer	3	2331	2836	5110
Brain and central nervous system cancer	3	3652	4215	7367
Thyroid cancer	3	3658	4107	7346
Other malignant neoplasms	3	3597	3789	7271
Hodgkin lymphoma	3	3596	3762	7256
Non-Hodgkin lymphoma	3	3740	4275	7458
Burkitt lymphoma	3	3579	82	3591
Other non-Hodgkin lymphoma	3	3575	2770	6377
Multiple myeloma	3	3582	3160	6628
Leukaemia	3	3696	4273	7627
Other neoplasms	344	0	2827	3171

Table 1b. Data Inputs for liver cancer subtypes morbidity modelling by parameter

Cause	Proportion data sources
Liver cancer	268
Liver cancer due to alcohol use	96
Liver cancer due to hepatitis B	267
Liver cancer due to hepatitis C	266
Liver cancer due to NASH	93
Liver cancer due to other causes	55

Table 2: MR-BRT crosswalk adjustment factors for model inputs for liver cancer aetiology proportions

Model	Crosswalk	Reference	Alternative	Gamma	Beta coefficient	Adjustment factor
Liver cancer due to alcohol	Sex split	male proportion	Both-sex proportion	0	–0.998	0.368
Liver cancer due to hepatitis B	Sex split	male proportion	Both-sex proportion	0.07	–0.421	0.656
Liver cancer due to hepatitis C	Sex split	male proportion	Both-sex proportion	0.42	0.299	1.349
Liver cancer due to other causes	Sex split	male proportion	Both-sex proportion	0.37	0.260	1.297
Liver cancer due to NASH	Sex split	male proportion	Both-sex proportion	0	0.090	1.095
Liver cancer due to NASH	NASH definition	Explicit NASH	Implicit NASH	0.91	–0.322	0.725

Additional detail on the MR-BRT method can be found elsewhere in the GBD summary articles.

Modelling strategy

Estimation of cancer mortality and MIR estimation has been described elsewhere in the GBD summary articles. As both the fatal and non-fatal estimation processes utilise these same modelled MIR estimates, the MIR estimation process is detailed again below for convenience. To summarise, incidence and mortality data from cancer registries were matched by cancer, age, sex, year, and location to generate input MIRs, which are then used to obtain MIR estimates using one of two MIR modelling approaches, depending on the cancer. In the non-fatal process, these estimated MIRs are then used to transform the final GBD cancer mortality estimates into GBD incidence estimates.

MIR data processing

For all causes that existed in GBD 2019, data-cleaning steps for MIR estimation were the same as for GBD 2019. For each cancer, MIRs from locations in Healthcare Access and Quality Index (HAQ Index) quintiles 1–4 were dropped if they were below the median of MIRs from locations in HAQ Index quintile 5. We also dropped MIRs from locations in HAQ Index quintiles 1–4 if the MIRs were above an outlier threshold calculated as the third quartile + 1.5 * IQR (inter-quartile range). We dropped all MIR data that were based on fewer than 15 incident cases to avoid excessive variation in the ratio due to small numbers. An exception to this threshold was made for mesothelioma and acute myeloid leukaemia, where instead we dropped MIRs that were based on fewer than ten cases because of lower data availability for these two cancers. For the lower end of the age spectrum where cancers are generally rarer, we also aggregated incidence and mortality to the youngest five-year age bin where SEER[7] reported at least 50 cases from 1990 to 2015, to avoid unstable MIR predictions in young age groups because of too few cases or deaths. The MIR estimates in this SEER-based minimum age-bin were then copied down to all younger GBD age groups estimated for that cancer.

For the nine new cancer causes first estimated in GBD 2021, additional data processing steps were used to help stabilise the input data and MIR estimates. First, data were aggregated across sexes and across bins of ten calendar years. Data were then only excluded if it had 0 cases. As cancer registry mortality data were limited for the new cancer causes Burkitt lymphoma and retinoblastoma, we supplemented with mortality data from vital registration systems where available. For these two causes, cancer registry incidence was matched with vital registration mortality by age-sex-year-location. These cancer registry-vital registration matched inputs were processed the same as the standard matched inputs.

Since MIRs can be above 1, especially in older age groups and for cancers with low cure rates, we used the 95th percentile (by age group) of the cleaned dataset (detailed above) to cap the MIR input data. These "upper cap" values were used to allow MIRs over 1 in some age groups but to constrain the MIRs to a maximum level. The addition of new data for GBD 2021 led to slightly different upper caps compared to GBD 2019 (see upper cap values for GBD 2021 below). New for GBD 2021, the upper caps for paediatric age groups (under 20 years) were increased to 1 (regardless of the 95th percentile) to allow for more model flexibility in the distribution of MIRs across locations.

**Age group (years):**	**0–4**	**5–9**	**10–14**	**15–19**	**20–24**	**25–29**	**30–34**	**35–39**	**40–44**	**45–49**
**Upper cap:**	1.00	1.00	1.00	1.00	1.04	0.949	0.936	0.888	0.928	0.950

**Age group (years):**	**50–54**	**55–59**	**60–64**	**65–69**	**70–74**	**75–79**	**80–84**	**85–89**	**90–94**	**95+**
**Upper cap:**	0.962	0.992	1.02	1.06	1.11	1.18	1.27	1.36	1.48	1.61

Any MIR values over this upper cap were Winsorised to the cap value. To run the logit model in ST-GPR, the input data were first divided by the upper caps to get proportional data ranging from 0 to 1. Model predictions from ST-GPR were then rescaled back to MIRs by multiplying the scaled predictions by the upper caps. To constrain the MIRs at the lower end, we used the fifth percentile of the cancer and age-specific cleaned MIR input data to Winsorise all model predictions below this lower cap.

MIR data modelling

As in previous GBD cycles, MIRs for most cancers were estimated with a three-step modelling approach using the general GBD spatiotemporal Gaussian process regression (ST-GPR) approach. These used logit-transformed MIR as the outcome, with covariates for sex, categorical age group, and HAQ Index as a covariate in the linear mixed effects model.[8]

$$\text{logit}\,(MIR_{c,a,s,t}) = \alpha + \beta_1(HAQIndex)_{c,t} + \sum_{a}^{A} \beta_2 I_a + \beta_3 I_s + \epsilon_{c,a,s,t}$$

MIR: mortality-to-incidence ratio
c: country (or subnational for subnationally modelled locations), a: age group, t: time (years); s: sex
HAQIndex: Healthcare Access and Quality Index
I: indicator variable
$\epsilon_{c,a,s,t}$: error term

Results from the final linear model were used as input for spatiotemporal smoothing and a Gaussian process regression. The ST-GPR model has three main hyper-parameters that control for smoothing across time, age, and geography. These hyper-parameter values were unchanged for GBD 2021. The time adjustment parameter lambda (λ) aims to borrow strength from neighbouring time points (ie, the value in this year is highly correlated with the value in the previous year but less so further back in time) and was set to 0.05. The age adjustment parameter omega (ω) borrows strength from data in neighbouring age groups and was set to 0.5. The space adjustment parameter zeta (ζ) aims to borrow strength across the hierarchy of geographical locations and was set to 0.01. For the remaining parameters in the Gaussian process regression, we set amplitude to 1 (influences fluctuation from the mean function) and set the scale value to 10 (influences the time distance over which points are correlated). Additional details on ST-GPR are described elsewhere in the GBD summary papers. These models were used to obtain MIR estimates for all combinations of GBD age, sex, year, cause, and location. Datapoints were outliered manually if they clearly influenced the model in an unrealistic way. For example, a datapoint was marked as an outlier if it created a single-year, single age group spike in model predictions that was inconsistent with the trend suggested by surrounding datapoints.

For eight of the nine cancer causes that are newly estimated in GBD 2021, we modelled MIRs using a negative binomial regression approach. The exception was “other non-Hodgkin lymphoma”, which was modelled using the ST-GPR methods described above due to greater data availability for this cause. The negative binomial approach was used for most of the newly estimated cancer causes because it allows modelling of count data with overdispersion (meaning the mean and variance are allowed to differ in the underlying distribution), which was determined to be needed due to the relatively rare deaths for these cancer causes. MIRs were estimated for each age-sex-year-location using a negative binomial regression run in R (version 3.5.0) using glm.nb from the MASS package. We used categorical age and HAQ Index as covariates and offset by the logarithm of cases.

Incidence estimation

For all cancers except retinoblastoma, the final GBD cancer mortality estimates (after CoDCorrect adjustment) were transformed to incidence estimates by using the MIRs specific to that cancer cause.

Final mortality estimates at the 1000-draw level were divided by the modelled MIR estimates (also at the 1000-draw level) to generate 1000 draws of incidence estimates (which provides an estimated mean incidence with 95% uncertainty interval). It was assumed that uncertainty in the MIR is independent of uncertainty in the estimated mortality.

For retinoblastoma, the incidence estimation approach above was used for all locations except those in the high-income super-region. For high-income countries, death from retinoblastoma is extremely rare, which can lead to estimated MIRs close to zero and underestimation of incidence with the above approach (due to a numerator close to zero). To address this, alternative MIRs specific to locations in the high-income super-region were estimated by matching CODEm mortality estimates with cancer registry incidence data for these locations; incidence draws for these locations were then estimated as detailed above, dividing the CoDCorrect estimates by these adjusted MIRs (rather than the globally informed MIRs). To avoid potential subsequent overestimation of incidence in these locations, the incidence rates were Winsorised to the 2.5th and 97.5th percentiles of incidence rates across countries in the high-income super-region.

Prevalence estimation

After transforming the final GBD cancer mortality estimates to incidence estimates (step 1 in the general cancer flowchart), incidence was combined with annual relative survival estimates from one to ten years after diagnosis (step 7 in the flowchart). Previous reports suggest that the value of (1 – MIR) may serve as a proxy for five-year relative survival, with the exact correlation varying slightly by cancer type.[9] Because this correlation varies, we trained cancer-specific prediction models to estimate five-year survival from MIRs, using data from SEER. We used SEER*Stat[10] to obtain mortality,[11] incidence,[12] and relative survival[12] statistics from the nine SEER registries reporting from 1980 to 2014 (through 2014 so that all years have at least five years of follow-up time; step 2), by cancer type, sex, five-year time periods (eg, 1980–1984, 1985–1989, etc.), and five-year age groups (except combining 80+). For each cancer, we modelled five-year relative survival with MIRs calculated from SEER mortality and incidence, using a generalised linear model with a quasibinomial family and logit link, weighted by the number of index cases (step 3).

To reduce variability due to small samples, we only included MIRs based on at least 25 incident cases (except for the rarer cancers mesothelioma and acute myeloid leukaemia, where MIRs based on at least ten cases were included). These models were then applied to the GBD MIR estimates to predict an estimated five-year survival for each age/sex/year/location (step 4). To prevent unrealistic values, predicted five-year survival values were Winsorised to be between 0% and 100% survival. Unlike GBD 2017 (but similar to GBD 2019), we did not require the estimated survival to be greater than the all-ages worst-case survival scenario from SurvCan and USA 1950 survival data,[13,14] since age-specific survival could be plausibly lower than for those all-ages scenarios.

To generate yearly survival estimates up to ten years, we downloaded SEER sex- and age-specific annual one- through ten-year relative survival data from persons diagnosed between 2001 and 2010 (2001 through 2010 so that all cases had at least five years of follow-up, with half having the full ten years of follow-up).[12] A proportional scalar was calculated as the predicted GBD five-year survival estimate divided by the SEER five-year survival statistic, and was then used to generate yearly survival estimates by scaling the one- to ten-year SEER curve to the GBD survival predictions under the proportional hazards assumption (step 5).

The estimated relative survival is next transformed into absolute survival estimates (step 6 and 7 in the flowchart). To account for background mortality in the relative survival estimates, GBD 2021 lifetables were used to calculate lambda (λ) values:

$$\lambda = \frac{\ln\left(\frac{nLx_n}{nLx_{n+1}}\right)}{5}$$

nLx = person-years lived between ages x and x+n (from GBD lifetable).

GBD 2021 lifetables are described elsewhere in the GBD summary papers. Absolute survival was then calculated using an exponential survival function:

$$absolute\ survival = relative\ survival * e^{\lambda * t}$$

t = time (in years)

Absolute survival is combined with incidence to estimate the prevalence at each year after diagnosis, which is then split into the four sequelae (step 8 in the flowchart).

Disability estimation

For the purposes of calculating disability due to cancer, survivors beyond ten years were considered cured. For this group, the survivor population prevalence was divided into two sequelae: (1) diagnosis and primary therapy phase; and (2) controlled phase (or remission). For the population that did not survive beyond ten years, the yearly prevalence was divided into four sequelae by assigning fixed durations for each of the: (1) diagnosis and primary therapy phase, (3) disseminated/metastatic phase, and (4) terminal phase, and assigning any remaining prevalence to the (2) controlled phase (step 8 in the flowchart). Except for the new cancer causes added in GBD 2021, the duration of these four sequelae remained the same as for GBD 2013, GBD 2015, GBD 2016, GBD 2017, and GBD 2019.[1–5] Table 3 lists the duration of each, along with the sources used to determine their length. For the diagnosis and primary therapy phase, the duration was taken from primary literature or expert opinion. For the disseminated/metastatic phase, the duration was taken from primary literature, or as the median survival time reported by SEER for the persons described in the note column.

Table 3. Duration of four prevalence sequelae by cancer

	Diagnosis and primary therapy phase (months)*	Controlled phase, or remission	Disseminated/ metastatic phase (months)*	Note for disseminated/ metastatic phase	Terminal (months)
Oesophageal cancer	5.0[15]	The remission phase duration is calculated based on	4.6[16]	SEER Summary Stage 1977 (Distant site/node involved) 1995–2000	1.0

Stomach cancer	5.2[15]	the remaining time after attributing other sequelae durations.	3.9[16]	SEER Summary Stage 1977 (distant site/node involved) 1995–2000	1.0
Liver cancer	4.0		2.5[16]	SEER Summary Stage 1977 (distant site/node involved) 1995–2000	1.0
Hepatoblastoma	6.0		23.1[16]	SEER Summary Stage 1977 (distant site/node involved) 1995–2000	1.0
Larynx cancer	5.3[15]		8.8[16]	SEER Stage IVc	1.0
Tracheal, bronchus, and lung cancer	3.3[17]		4.5[16]	SEER Summary Stage 1977 (distant site/node involved) 1995–2000	1.0
Breast cancer	3.0[17]		17.7[16]	SEER Summary Stage 1977 (distant site/node involved) 1995–2000	1.0
Cervical cancer	4.8[15]		9.2[16]	SEER Summary Stage 1977 (distant site/node involved) 1995–2000	1.0
Uterine cancer	4.6[15]		11.6[16]	SEER Summary Stage 1977 (distant site/node involved) 1995–2000	1.0
Prostate cancer	4.0[17]	The remission phase duration is calculated based on the remaining time after attributing other sequelae durations.	30.4[16]	SEER Summary Stage 1977 (distant site/node involved) 1995–2000	1.0
Colon and rectum cancer	4.0[17]		9.7[16]	SEER Summary Stage 1977 (distant site/node involved) 1995–2000	1.0
Lip and oral cavity cancer	5.3[15]		9.3[16]	SEER Stage IVc	1.0
Nasopharynx cancer	5.3[15]		13.2[16]	SEER Stage IVc	1.0
Other pharynx cancer	5.3[15]		7.9[16]	SEER Stage IVc	1.0
Gallbladder and biliary tract cancer	4.0		3.5[16]	SEER Summary Stage 1977 (distant site/node involved) 1995–2000	1.0
Pancreatic cancer	4.1[15]		2.5[16]	SEER Summary Stage 1977 (distant site/node involved) 1995–2000	1.0
Malignant skin melanoma	2.9[18]		7.2[16]	SEER Summary Stage 1977 (distant site/node involved) 1995–2000	1.0

Ovarian cancer	3.2[17]		25.6[16]	SEER Summary Stage 1977 (distant site/node involved) 1995–2000	1.0
Testicular cancer	3.7[15]		19.5[16]	SEER Stage III	1.0
Kidney cancer	5.3[15]		5.4[16]	SEER Summary Stage 1977 (distant site/node involved) 1995–2000	1.0
Bladder cancer	5.1[15]		5.8[16]	SEER Summary Stage 1977 (distant site/node involved) 1995–2000	1.0
Brain and central nervous system cancer	5.0		6.9[16]	SEER median age-standardised survival all patients, all years	1.0
Thyroid cancer	3.0		19.4[16]	SEER Stage IVc	1.0
Mesothelioma	4.0		7.8[16]	SEER Summary Stage 1977 (distant site/node involved) 1995–2000	1.0
Hodgkin lymphoma	3.7[17]		26.0[19]	Literature	1.0
Non-Hodgkin lymphoma	3.7[17]		7.7[19]	Literature	1.0
Other non-Hodgkin lymphoma	6.0		41.0[16]	SEER Summary Stage 1977 (distant site/node involved) 1995–2000	1.0
Burkitt lymphoma	6.0	The remission phase duration is calculated based on the remaining time after attributing other sequelae durations.	8.8[16]	SEER Summary Stage 1977 (distant site/node involved) 1995–2000	1.0
Multiple myeloma	7.0[15]		36.8[16]	SEER median age-standardised survival all patients, all years	1.0
Leukaemia	5.0[15]		43.7[16]	SEER median age-standardised survival all patients, all years	1.0
Acute lymphoid leukaemia	12.0		7.0[16]	SEER median age-standardised survival all patients, all years	1.0
Acute myeloid leukaemia	6.0		4.6[16]	SEER median age-standardised survival all patients, all years	1.0
Chronic lymphoid leukaemia	6.0		48[20]	SEER median age-standardised survival all patients, all years	1.0
Chronic myeloid leukaemia	6.0		4.6[16]	SEER median age-standardised survival for	1.0

				AML (patients with CML die in blast crisis, which is treated like AML) all patients, all years	
Other leukaemia	6.0		48.0[20]	SEER median age-standardised survival all patients, all years	1.0
Other malignant neoplasms	4.4 (mean of other cancer durations)		15.8[16]	SEER median age-standardised survival all patients, all years	1.0
Malignant neoplasm of bone and articular cartilage	10.0		19.8[16]	SEER Summary Stage 1977 (distant site/node involved) 1995–2000	1.0
Neuroblastoma and other peripheral nervous cell tumours	10.0		47.4[16]	SEER Summary Stage 1977 (distant site/node involved) 1995–2000	1.0
Soft tissue and other extraosseous sarcomas	10.0		10.7[16]	SEER Summary Stage 1977 (distant site/node involved) 1995–2000	1.0
Eye cancer	2.9		16.0[16]	SEER median age-standardised survival all patients, all years	1.0
Other eye cancers	2.9	The remission phase duration is calculated based on the remaining time after attributing other sequelae durations.	16.0[16]	SEER median age-standardised survival all patients, all years	1.0
Retinoblastoma	6.0		6.4[21]	Literature	1.0

* Superscripts refer to references used to inform these values. Durations without a superscript are based on expert opinion.

For cancer-specific procedure sequelae, hospital data were used to estimate the number of cancer patients undergoing mastectomy, laryngectomy, stoma, prostatectomy, or cystectomy (step 9 in the flowchart). Input data for these proportions remained the same as in GBD 2013, GBD 2015, GBD 2016, GBD 2017, and GBD 2019.[1–5] Proportions were generated by dividing the rate of procedures generated from the diagnostic codes in the hospital dataset and the coverage population by the GBD age- and sex-specific disease incidence rates for that country. Diagnostic codes used are listed in Table 4:

Table 4. Procedure codes used to estimate cancer procedure proportions

Procedure	Cancer	Procedure code (ICD-9-CM[22])
Mastectomy	Breast cancer	854, 8541, 8542, 8543, 8544, 8545, 8546, 8547, 8548
Laryngectomy	Larynx cancer	301, 303, 304, 3029
Stoma	Colon and rectum cancer	461, 4610, 4611, 4613, 4862
Cystectomy	Bladder cancer	5771, 5779
Prostatectomy	Prostate	603, 604, 605, 606, 6062

To estimate procedure-related disability for each of these five cancers, the procedure proportions (proportion of each cancer population that undergo these procedures) from hospital data from the USA,[23,24] Canada,[25] and Mexico[26] were used as input for a proportion model in DisMod-MR 2.1 to estimate the proportions for all locations, by age, year, and by sex. Details of DisMod-MR 2.1 and clinical and claims data processing are available elsewhere in the GBD summary papers.

Since colostomy or ileostomy procedures are done for reasons other than cancer, a literature review was conducted to determine the proportion of ostomies due to colon and rectum cancer. Based on the results of the literature review that an average of 58% of ostomies are done for colon and rectum cancer, the "all cause" colostomy proportions were multiplied by 0.58.[27–29]

The final procedure proportions were applied to the incident cases of the respective cancers and multiplied with the proportion of the incident population surviving for ten years to determine the incident cases of the cancer population that underwent procedures and that survived beyond ten years. These estimates of survivors at ten years were then used as an input for DisMod-MR 2.1, with a remission specification of zero and an excess mortality rate prior of 0 to 0.1, as well as with increasing both the age of the population and the year by ten years to reflect prevalence after that population has survived ten years. The results from this model are incidence and lifetime prevalent cases of persons with these cancer-related sequelae who have survived beyond ten years.

Since disability associated with prostatectomy comes from impotence and incontinence, and not from the prostatectomy itself, 18% of the prostatectomy prevalence was assumed to have incontinence and 55% was assumed to have impotence, based on a literature review done for GBD 2013.[30–37] Cases were assigned disability for either impotence or incontinence, but no cases were assigned disability from both.

We assumed that for the population surviving up to ten years, only the prevalence population being in remission experiences additional disability due to procedures (eg, women suffering from metastatic breast cancer do not experience additional disability due to a mastectomy during this phase). To estimate the prevalence of the cancer population in remission during the first ten years after diagnosis with and without procedure-related disability, we multiplied the prevalence of the population in the remission phase with the proportion of the population undergoing a procedure. This step allowed us to estimate disability during the remission phase for both the population experiencing disability due to the remission phase alone, as well as the population experiencing disability from the remission phase and the additional procedure-related disability.

YLD estimation

Lastly, the procedure sequelae prevalence and general sequelae prevalence were multiplied with their respective disability weights (Table 5) to obtain the number of YLDs for each sequelae (steps 11 and 12 in the flowchart). Summing these sequelae-specific YLDs then provides the total YLD estimate associated with each cancer cause.

Table 5. Lay description and disability weights

Health state	Lay description	Disability weight (95% uncertainty interval)
Cancer, diagnosis and primary therapy *All cancers except non-melanoma skin cancer*	This person has pain, nausea, fatigue, weight loss and high anxiety.	0.288 (0.193–0.399)
Cancer, controlled phase *All cancers except non-melanoma skin cancer*	This person has a chronic disease that requires medication every day and causes some worry but minimal interference with daily activities.	0.049 (0.031–0.072)
Cancer, metastatic *All cancers except non-melanoma skin cancer*	This person has severe pain, extreme fatigue, weight loss and high anxiety.	0.451 (0.307–0.600)
Terminal phase, with medication *All cancers except non-melanoma skin cancer*	This person has lost a lot of weight and regularly uses strong medication to avoid constant pain. The person has no appetite, feels nauseous, and needs to spend most of the day in bed.	0.540 (0.377–0.687)
Mastectomy *Breast cancer*	This person had one of her breasts removed and sometimes has pain or swelling in the arms.	0.036 (0.020–0.057)
Stoma *Colon and rectum cancer*	This person has a pouch attached to an opening in the belly to collect and empty stools.	0.095 (0.063–0.131)
Laryngectomy *Larynx cancer*	This person has difficulty speaking, and others find it difficult to understand.	0.051 (0.032–0.078)
Urinary incontinence *Bladder cancer; Prostate cancer*	This person cannot control urinating.	0.139 (0.094–0.198)

Impotence<br>*Prostate cancer*	This person has difficulty in obtaining or maintaining an erection.	0.017<br>(0.009–0.030)

Non-melanoma skin cancer (squamous and basal cell carcinoma)

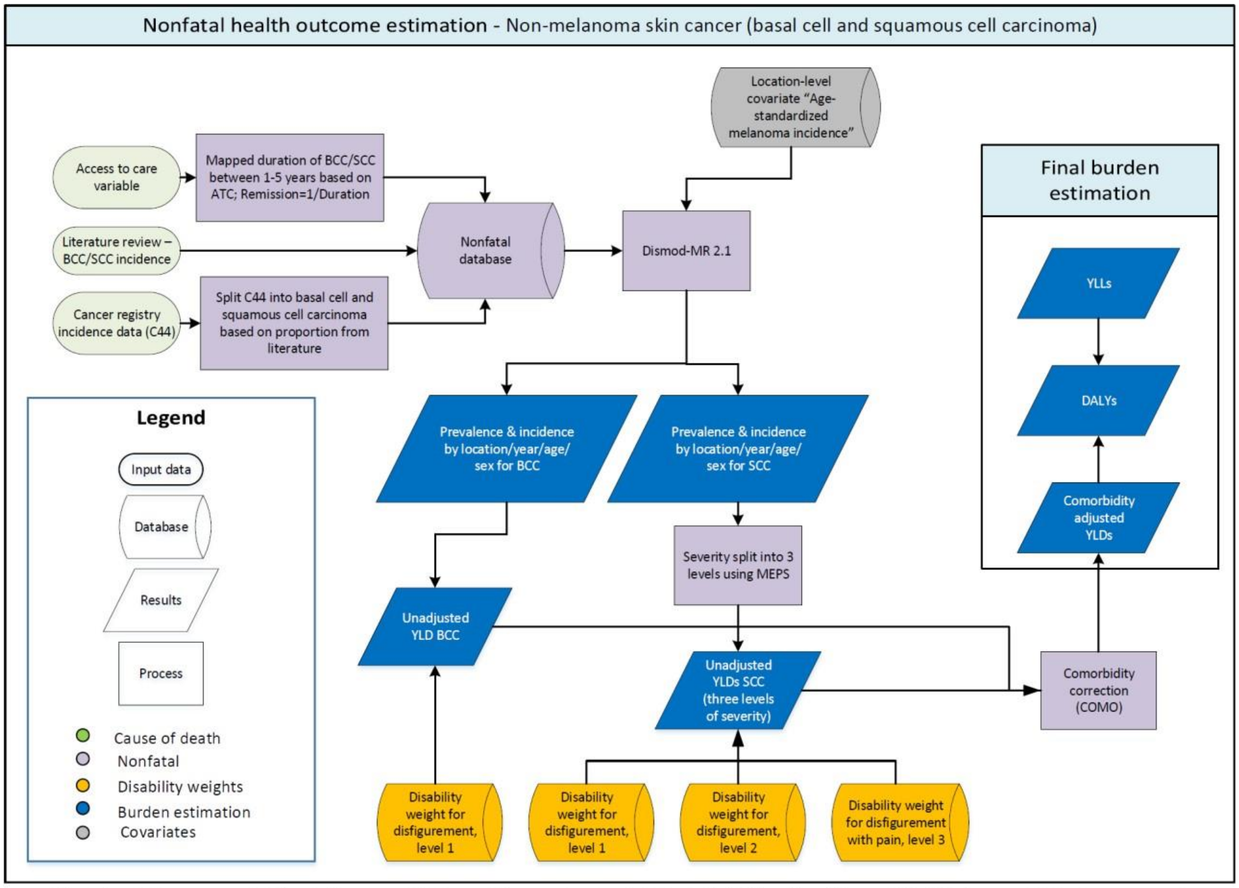

Abbreviations: ATC, access to care; BCC, basal cell carcinoma; COMO, comorbidity correction microsimulation; DALY, disability-adjusted life year; DisMod-MR, disease model, Bayesian meta-regression; MEPS, Medical Expenditure Panel Survey; SCC, squamous cell carcinoma; YLD, years lived with disability; YLL, years of life lost.

Case definition

Non-melanoma skin cancer (NMSC) is defined as squamous cell carcinoma and basal cell carcinoma. NMSC does not include other types of skin cancer (eg, melanoma, Merkel cell carcinoma).

Input data

We estimated squamous cell carcinoma and basal cell carcinoma incidence by using data from cancer registries, primary literature, clinical data, and insurance claims. Only cancer registries that were listed in Cancer Incidence in Five Continents (CI5)[38–48] as registering squamous cell carcinoma or basal cell carcinoma were included in the cancer registry incidence data. For GBD 2021, clinical data processing

and adjustment methods were updated to base correction factors on hospital data from Poland,[49] using age splines with frequency-based knots. This reduced the size and uncertainty of the correction factors compared to GBD 2019, which had previously been based on claims data from MarketScan.[50] As in GBD 2019, these clinical data were adjusted for the HAQ Index of the location and inpatient data accounts for outpatient encounters. Additional details on clinical and claims data processing are available elsewhere in the GBD summary papers.

Modelling strategy

For cancer registry data reported at the three-digit level (ie, C44: Other and unspecified malignant neoplasm of skin), fixed proportions reported in Karagas and colleagues were used to split C44 into squamous cell carcinoma and basal cell carcinoma.[51] These data, along with data from clinical and literature sources, were input into DisMod-MR 2.1 models to estimate incidence and prevalence for both squamous cell carcinoma and basal cell carcinoma. Prevalence was calculated as a function of two extreme scenarios (duration of one versus five years). Country-, age-, sex-, and year-specific duration was estimated using a country-age-sex-year-specific relative access-to-care score.

The access-to-care score was based on the melanoma mortality-to-incidence ratio:

$$Access\ to\ care = 1 - \frac{Age\ standardised\ MIR_{cys} - Age\ standardised\ MIR_{min}}{Age\ standardised\ MIR_{max} - Age\ standardised\ MIR_{min}}$$

c = country; y = year; s = sex

Age-standardised MIR_{min} = lowest MIR for all countries and years

Age standardised MIR_{max} = highest MIR for all countries and years

Remission was calculated as the inverse of these duration estimates and used as additional input for DisMod-MR 2.1. New for GBD 2021, the squamous cell carcinoma DisMod model included modelled priors of the excess mortality rate (EMR) as input data, which were generated using the GBD meta-regression—Bayesian, regularised, trimmed (MR-BRT) method, which is detailed elsewhere in the GBD summary articles. The EMR estimates were extracted from a baseline DisMod model and subsequently modelled using the MR-BRT approach by age and sex with a prior on HAQ Index having a negative coefficient. Results from MR-BRT were then predicted for each location, year, sex, and for ages 0, 10, 20, … 100. We included HAQ Index as a country-level covariate to inform EMR with a mean and standard deviation produced from MR-BRT. These modelled EMR priors were then included as input data for the final GBD 2021 DisMod model.

To reflect differing degrees of disability due to squamous cell carcinoma we used three levels of severity that were derived from MEPS (Medical Expenditure Panel Survey),[52] resulting in fixed proportions of 80% mild disfigurement, 15% moderate, and 5% severe. For basal cell carcinoma, disability severity was split into 60% asymptomatic (without disability) and 40% with mild disfigurement. Prevalence was multiplied by distinct disability weights (Table 6) to generate YLDs.

Table 6. Lay description and disability weights, non-melanoma skin cancer (squamous and basal cell carcinoma)

Cancer disability, severity	Health state	Lay description	Disability weight (95% uncertainty interval)
Cutaneous squamous cell carcinoma, mild	Disfigurement, level 1	This person has a slight, visible physical deformity that others notice, which causes some worry and discomfort.	0.011 (0.005–0.021)
Cutaneous squamous cell carcinoma, moderate	Disfigurement, level 2	This person has a visible physical deformity that causes others to stare and comment. As a result, the person is worried and has trouble sleeping and concentrating.	0.067 (0.044–0.096)
Cutaneous squamous cell carcinoma, severe	Disfigurement, level 3, with itch/pain	This person has an obvious physical deformity that is very painful and itchy. The physical deformity makes others uncomfortable, which causes the person to avoid social contact, feel worried, sleep poorly, and think about suicide.	0.576 (0.401–0.731)
Disfigurement due to basal cell carcinoma	Disfigurement, level 1	This person has a slight, visible physical deformity that others notice, which causes some worry and discomfort.	0.011 (0.005–0.021)

Myelodysplastic, myeloproliferative, and other haemopoietic neoplasms

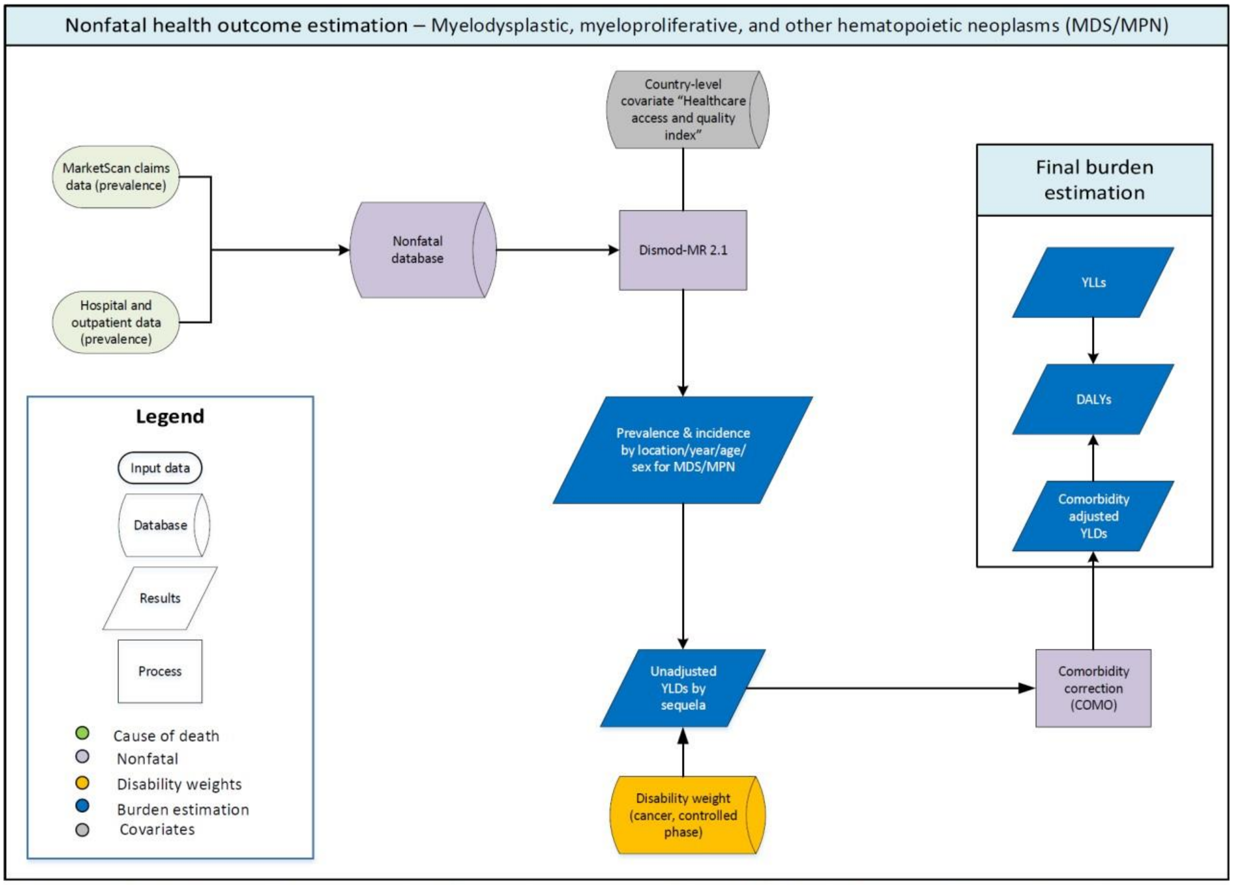

Abbreviations: DisMod-MR, disease model – Bayesian meta-regression; COMO, comorbidity correction microsimulation; DALY, disability-adjusted life-year; MDS/MPN, myelodysplastic, myeloproliferative, and other hematopoietic neoplasms; YLD, years lived with disability; YLL, years of life lost.

Case definition

Myelodysplastic, myeloproliferative, and other haemopoietic neoplasms (MDS/MPN) comprise a wide variety of diseases and outcomes, including ICD-10 codes D45, D46, and D47. These were modelled together as a single group for GBD 2021 (the same as for GBD 2017 and GBD 2019).

Input data

We estimated MDS/MPN deaths using vital registration data (as outlined above). We did not use cancer registry data for these neoplasms, as it has only been reported within some cancer registries since 2001 and is recognised to be under-reported.[53] We estimated MDS/MPN incidence and prevalence using hospital and outpatient prevalence data from various health systems worldwide. For GBD 2021, clinical data processing and adjustment methods were updated to base correction factors on hospital data from Poland,[49] using age splines with frequency-based knots. This reduced the size and uncertainty of the correction factors compared to GBD 2019, which had previously been based on claims data from MarketScan.[50] As in GBD 2019, these clinical data were adjusted for the HAQ Index of the location and inpatient data accounts for outpatient encounters. Additional details on clinical and claims data processing are available elsewhere in the GBD summary papers.

Modelling strategy

We modelled deaths for all locations and years, by age and by sex, using CODEm. As MDS/MPN can be a precursor to leukaemia, our MDS/MPN CODEm model used many of the same covariate priors as the CODEm model for acute myeloid leukaemia. CODEm methods, parameters, and covariates are described elsewhere in the GBD summary articles.

We modelled the incidence and prevalence of these diseases for all combinations of location, age, year, and sex using a prevalence model in DisMod-MR 2.1. For DisMod model specifications, cause-specific mortality rates came from the CODEm model, remission was specified to be zero, and the excess mortality rate was set to be inversely related to the HAQ Index covariate.

While this broad category of haematological neoplasms is heterogeneous in its components' severity or propensity for transformation to leukaemia, modelling the subtypes of MDS/MPN separately was not feasible for GBD 2021. This is a limitation and an area of desired future improvement as data availability improves. For GBD 2021, the "cancer, controlled phase" disability weight was assigned for all MDS/MPN cases (see Table 5).

Benign and in situ intestinal neoplasms; benign and in situ cervical and uterine neoplasms; other benign and in situ neoplasms

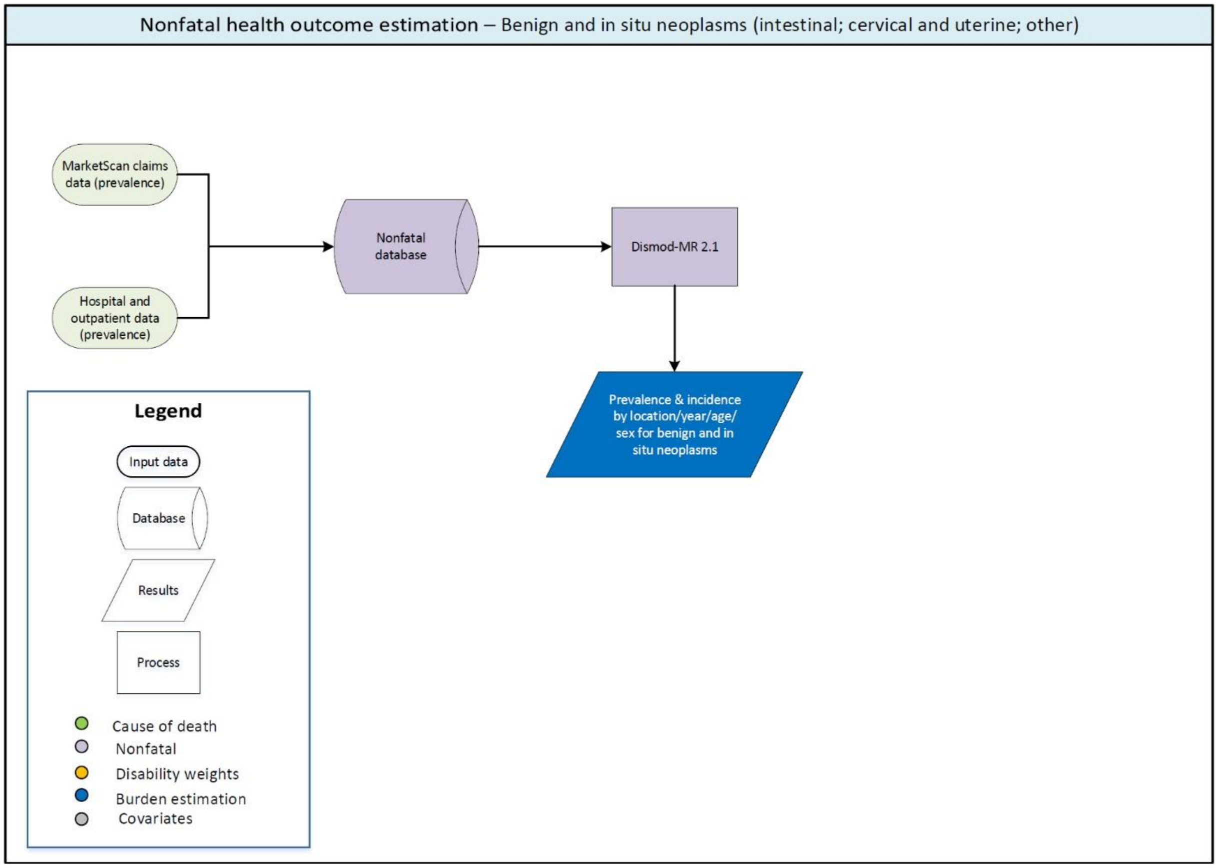

Abbreviations: DisMod-MR, disease model – Bayesian meta-regression.

Case definition

For GBD 2021, we estimated three categories of benign and in situ neoplasms: (1) intestinal neoplasms; (2) cervical and uterine neoplasms; and (3) other benign and in situ neoplasms. Benign and in situ intestinal neoplasms were defined as any non-invasive intestinal growth of the digestive system beyond the stomach, from duodenum to anus. Benign and in situ cervical and uterine neoplasms were defined as any non-invasive cervical or uterine growth, except for uterine fibroids, which are modelled as a separate GBD cause. Other benign and in situ neoplasms were defined as any non-invasive neoplasms not covered by other GBD causes, such as lipomas, benign breast neoplasms, and non-melanoma skin neoplasms other than basal cell carcinoma or squamous cell carcinoma.

Input data

To estimate the prevalence of each of these categories for all locations, by age, year, and sex, the prevalence of these neoplasms from clinical data was used as input for a prevalence model in DisMod-MR 2.1. We estimated incidence and prevalence for each of these benign neoplasms using hospital and outpatient prevalence data from various health systems worldwide. For GBD 2021, clinical data processing and adjustment methods were updated to base correction factors on hospital data from Poland,[49] using age splines with frequency-based knots. This reduced the size and uncertainty of the correction factors compared to GBD 2019, which had previously been based on claims data from MarketScan.[50] As in GBD 2019, these clinical data were adjusted for the HAQ Index of the location and inpatient data accounts for outpatient encounters. Additional details on clinical and claims data processing are available elsewhere in the GBD Capstones.

Of these three causes, we only estimated deaths for “other benign and in situ neoplasms”, as this cause includes neoplasms such as low-grade and other central nervous system neoplasms. Though non-malignant, these tumors can cause death from physically impairing vital nervous system functions. We estimated these deaths using vital registration data (as outlined above). We did not use cancer registry data for these neoplasms for GBD 2021, as these are not consistently captured by these systems.

Modelling strategy

In the DisMod model for benign and in situ intestinal neoplasms, excess mortality rate was specified to be zero, and remission was allowed to vary from 0 to 1. In the DisMod model for benign and in situ cervical and uterine neoplasms, excess mortality rate was specified to be zero, and remission was allowed to vary from 0 to 0.75. In the DisMod model for other benign and in situ neoplasms, excess mortality rate was specified to be zero, and remission was allowed to vary from 0 to 1.

All three of these benign and in situ neoplasms are by definition benign and localised. As such, no deaths or disability were attributed to their occurrence in GBD 2021. The exception was for “other benign and in situ neoplasms”, where deaths were included for a subset of this broad category that includes neoplasms such as low-grade brain and central nervous system neoplasms. Deaths were modelled in CODEm, as described above. Because these are only a subset of the total neoplasms included in the cause, we did not assign any disability to this cause for GBD 2021. This is an area of desired future improvement as data availability improves.

References

1 Global Burden of Disease Cancer Collaboration. The Global Burden of Cancer 2013. *JAMA Oncology* 2015; **1**: 505–27.

2 Global Burden of Disease Cancer Collaboration. Global, Regional, and National Cancer Incidence, Mortality, Years of Life Lost, Years Lived With Disability, and Disability-Adjusted Life-years for 32 Cancer Groups, 1990 to 2015: A Systematic Analysis for the Global Burden of Disease Study. *JAMA Oncology* 2017; **3**: 524–48.

3 Global Burden of Disease Cancer Collaboration. Global, Regional, and National Cancer Incidence, Mortality, Years of Life Lost, Years Lived With Disability, and Disability-Adjusted Life-Years for 29 Cancer Groups, 1990 to 2016: A Systematic Analysis for the Global Burden of Disease Study. *JAMA Oncology* 2018; **4**: 1553–68.

4 Global Burden of Disease Cancer Collaboration. Global, Regional, and National Cancer Incidence, Mortality, Years of Life Lost, Years Lived With Disability, and Disability-Adjusted Life-Years for 29 Cancer Groups, 1990 to 2017: A Systematic Analysis for the Global Burden of Disease Study. *JAMA Oncology* 2019; **5**: 1749–68.

5 Global Burden of Disease 2019 Cancer Collaboration. Cancer Incidence, Mortality, Years of Life Lost, Years Lived With Disability, and Disability-Adjusted Life Years for 29 Cancer Groups From 2010 to 2019: A Systematic Analysis for the Global Burden of Disease Study 2019. *JAMA Oncology* 2022;8(3):420–444. doi:10.1001/jamaoncol.2021.6987. https://jamanetwork.com/journals/jamaoncology/fullarticle/2787350 (accessed March 23, 2022).

6 Surveillance, Epidemiology, and End Results (SEER) Program (www.Seer.Cancer.Gov) SEER*Stat Database: Incidence - SEER 18.

7 Surveillance, Epidemiology, and End Results (SEER) Program (www.seer.cancer.gov) SEER*Stat Database: Incidence - SEER 18 Regs Research Data + Hurricane Katrina Impacted Louisiana Cases, Nov 2017 Sub (1973-2015 varying) - Linked To County Attributes - Total U.S., 1969-2016 Counties, National Cancer Institute, DCCPS, Surveillance Research Program, released April 2018, based on the November 2017 submission.

8 Barber RM, Fullman N, Sorensen RJD, *et al.* Healthcare Access and Quality Index based on mortality from causes amenable to personal health care in 195 countries and territories, 1990–2015: a novel analysis from the Global Burden of Disease Study 2015. *The Lancet* 2017; **390**: 231–66.

9 Asadzadeh Vostakolaei F, Karim-Kos HE, Janssen-Heijnen MLG, Visser O, Verbeek ALM, Kiemeney LALM. The validity of the mortality to incidence ratio as a proxy for site-specific cancer survival. *Eur J Public Health* 2011; **21**: 573–7.

10 Surveillance Research Program, National Cancer Institute SEER*Stat software (seer.cancer.gov/seerstat) version 8.3.4.

11 Surveillance, Epidemiology, and End Results (SEER) Program (www.seer.cancer.gov) SEER*Stat Database: Mortality - All COD, Aggregated With State, Total U.S. (1969-2015) <Katrina/Rita Population Adjustment>, National Cancer Institute, DCCPS, Surveillance Research Program, released December 2017. Underlying mortality data provided by NCHS (www.cdc.gov/nchs).

12 Surveillance, Epidemiology, and End Results (SEER) Program (www.seer.cancer.gov) SEER*Stat Database: Incidence - SEER 9 Regs Research Data, Nov 2016 Sub (1973-2014) <Katrina/Rita Population Adjustment> - Linked To County Attributes - Total U.S., 1969-2015 Counties, National Cancer Institute, DCCPS, Surveillance Research Program, released April 2017, based on the November 2016 submission.

13 Sankaranarayanan R, Swaminathan R, Brenner H, *et al.* Cancer survival in Africa, Asia, and Central America: a population-based study. *The Lancet Oncology* 2010; **11**: 165–73.

14 National Center for Health Statistics, Centers for Disease Control and, Prevention. US Mortality Files. 61-Year Trends in U.S. Cancer Death Rates. http://seer.cancer.gov/archive/csr/1975_2010/results_merged/topic_historical_mort_trends.pdf. .

15 Neal RD, Din NU, Hamilton W, *et al.* Comparison of cancer diagnostic intervals before and after implementation of NICE guidelines: analysis of data from the UK General Practice Research Database. *Br J Cancer* 2014; **110**: 584–92.

16 Surveillance, Epidemiology, and End Results (SEER) Program (www.seer.cancer.gov) SEER*Stat Database: Incidence - SEER 18 Regs Research Data + Hurricane Katrina Impacted Louisiana Cases, Nov 2012 Sub (1973-2010 varying) - Linked To County Attributes - Total U.S., 1969-2011 Counties, National Cancer Institute, DCCPS, Surveillance Research Program, Surveillance Systems Branch, released April 2013, based on the November 2012 submission.

17 Allgar VL, Neal RD. Delays in the diagnosis of six cancers: analysis of data from the National Survey of NHS Patients: Cancer. *Br J Cancer* 2005; **92**: 1959–70.

18 Neal RD, Cannings-John R, Hood K, *et al.* Excision of malignant melanomas in North Wales: effect of location and surgeon on time to diagnosis and quality of excision. *Fam Pract* 2008; **25**: 221–7.

19 Kewalramani T, Nimer SD, Zelenetz AD, *et al.* Progressive disease following autologous transplantation in patients with chemosensitive relapsed or primary refractory Hodgkin's disease or aggressive non-Hodgkin's lymphoma. *Bone Marrow Transplant* 2003; **32**: 673–9.

20 Esteban D, Tovar N, Jiménez R, *et al.* Patients with relapsed/refractory chronic lymphocytic leukaemia may benefit from inclusion in clinical trials irrespective of the therapy received: a case-control retrospective analsysis. *Blood Cancer J* 2015; **5**: e356.

21 Kopelman JE, McLean IW, Rosenberg SH. Multivariate analysis of risk factors for metastasis in retinoblastoma treated by enucleation. *Ophthalmology* 1987; **94**: 371–7.

22 ICD - ICD-9-CM - International Classification of Diseases, Ninth Revision, Clinical Modification. 2021; published online Nov 3. https://www.cdc.gov/nchs/icd/icd9cm.htm (accessed Dec 15, 2022).

23 National Cancer Institute (United States). United States SEER Cancer Data 1973-2010. Bethesda, United States: National Cancer Institute (United States).

24 National Center for Health Statistics (NCHS), Centers for Disease Control and Prevention (CDC), US Census Bureau. United States National Hospital Discharge Survey. Hyattsville, United States: National Center for Health Statistics (NCHS), Centers for Disease Control and Prevention (CDC). .

25 Canadian Institute for Health Information (CIHI). Canada Discharge Abstract Database 1994-2009. Ottawa, Canada: Canadian Institute for Health Information (CIHI). .

26 Ministry of Health (Mexico). Mexico Ministry of Health Hospital Discharges 2000-2012. Mexico City, Mexico: Ministry of Health (Mexico). .

27 Canova C, Giorato E, Roveron G, Turrini P, Zanotti R. Validation of a stoma-specific quality of life questionnaire in a sample of patients with colostomy or ileostomy. *Colorectal Dis* 2013; **15**: e692-698.

28 Caricato M, Ausania F, Ripetti V, Bartolozzi F, Campoli G, Coppola R. Retrospective analysis of long-term defunctioning stoma complications after colorectal surgery. *Colorectal Dis* 2007; **9**: 559–61.

29 Erwin-Toth P, Thompson SJ, Davis JS. Factors impacting the quality of life of people with an ostomy in North America: results from the Dialogue Study. *J Wound Ostomy Continence Nurs* 2012; **39**: 417–22; quiz 423–4.

30 Catalona WJ, Carvalhal GF, Mager DE, Smith DS. Potency, continence and complication rates in 1,870 consecutive radical retropubic prostatectomies. *J Urol* 1999; **162**: 433–8.

31 Donnellan SM, Duncan HJ, MacGregor RJ, Russell JM. Prospective assessment of incontinence after radical retropubic prostatectomy: objective and subjective analysis. *Urology* 1997; **49**: 225–30.

32 Eastham JA, Kattan MW, Rogers E, *et al.* Risk factors for urinary incontinence after radical prostatectomy. *J Urol* 1996; **156**: 1707–13.

33 Kundu SD, Roehl KA, Eggener SE, Antenor JAV, Han M, Catalona WJ. Potency, continence and complications in 3,477 consecutive radical retropubic prostatectomies. *J Urol* 2004; **172**: 2227–31.

34 Potosky AL, Davis WW, Hoffman RM, *et al.* Five-Year Outcomes After Prostatectomy or Radiotherapy for Prostate Cancer: The Prostate Cancer Outcomes Study. *JNCI Journal of the National Cancer Institute* 2004; **96**: 1358–67.

35 Sacco E, Prayer-Galetti T, Pinto F, *et al.* Urinary incontinence after radical prostatectomy: incidence by definition, risk factors and temporal trend in a large series with a long-term follow-up. *BJU Int* 2006; **97**: 1234–41.

36 Stanford JL, Feng Z, Hamilton AS, *et al.* Urinary and Sexual Function After Radical Prostatectomy for Clinically Localized Prostate CancerThe Prostate Cancer Outcomes Study. *JAMA* 2000; **283**: 354–60.

37 Walsh PC, Marschke P, Ricker D, Burnett AL. Patient-reported urinary continence and sexual function after anatomic radical prostatectomy. *Urology* 2000; **55**: 58–61.

38 Doll R, Payne P, Waterhouse J, editors. Cancer Incidence in Five Continents, Vol. I. Geneva: Union Internationale Contre le Cancer, 1966 https://publications.iarc.fr/Non-Series-Publications/Other-Non-Series-Publications/Cancer-Incidence-In-Five-Continents-Volume-I-1966 (accessed Feb 24, 2021)..

39 Doll R, Muir CS, Waterhouse JA. Cancer Incidence in Five Continents, Vol. II. Geneva: Union Internationale Contre le Cancer, 1970. .

40 Waterhouse J, Muir C, Correa P, Powell J. Cancer Incidence in Five Continents III. Lyon: IARC; 1976. .

41 Waterhouse J, Muir C, Shanmugaratnam K, Powell J. Cancer Incidence in Five Continents IV. Lyon: IARC; 1982. .

42 Muir C, Mack T, Powell J, Whelan S. Cancer Incidence in Five Continents V. Lyon: IARC; 1987. .

43 Parkin D, Muir C, Whelan S, Gao Y, Ferlay J, Powell J. Cancer Incidence in Five Continents VI. Lyon: IARC; 1992. .

44 Parkin D, Whelan S, Ferlay J, Raymond L, Young J. Cancer Incidence in Five Continents VII. Lyon: IARC; 1997. .

45 Parkin D, Whelan S, Ferlay J, Teppo L, Thomas D. Cancer Incidence in Five Continents VIII. Lyon: IARC; 2002. .

46 Curado M, Edwards B, Shin H, et al. Cancer Incidence in Five Continents IX. Lyon: IARC; 2007. http://www.iarc.fr/en/publications/pdfs-online/epi/sp160/CI5vol9-A.pdf. .

47 Forman D, Bray F, Brewster D, et al. Cancer Incidence in Five Continents X. http://ci5.iarc.fr. Published 2013. .

48 Bray F, Colombet M, Mery L, Piñeros M, Znaor A, Zanetti R and Ferlay J, editors (2017). Cancer Incidence in Five Continents, Vol. XI. Lyon: International Agency for Research on Cancer. .

49 National Health Fund (Poland). Poland National Health Fund Patient Claims 2016. .

50 Truven Health Analytics. United States MarketScan Commercial Claims and Encounters Database 2011. Ann Arbor, United States of America: Truven Health Analytics. .

51 Karagas MR, Greenberg ER, Spencer SK, Stukel TA, Mott LA. Increase in incidence rates of basal cell and squamous cell skin cancer in New Hampshire, USA. *International Journal of Cancer* 1999; **81**: 555–9.

52 Medical Expenditure Panel Survey Home. Accessed November 15, 2019. https://meps.ahrq.gov/mepsweb/. .

53 Cogle CR, Craig BM, Rollison DE, List AF. Incidence of the myelodysplastic syndromes using a novel claims-based algorithm: high number of uncaptured cases by cancer registries. *Blood* 2011; **117**: 7121–5.

Non-rheumatic valvular heart diseases. Calcific aortic valve disease, degenerative mitral valve disease, other non-rheumatic valve diseases

Flowchart

Calcific aortic valve and degenerative mitral valve disease

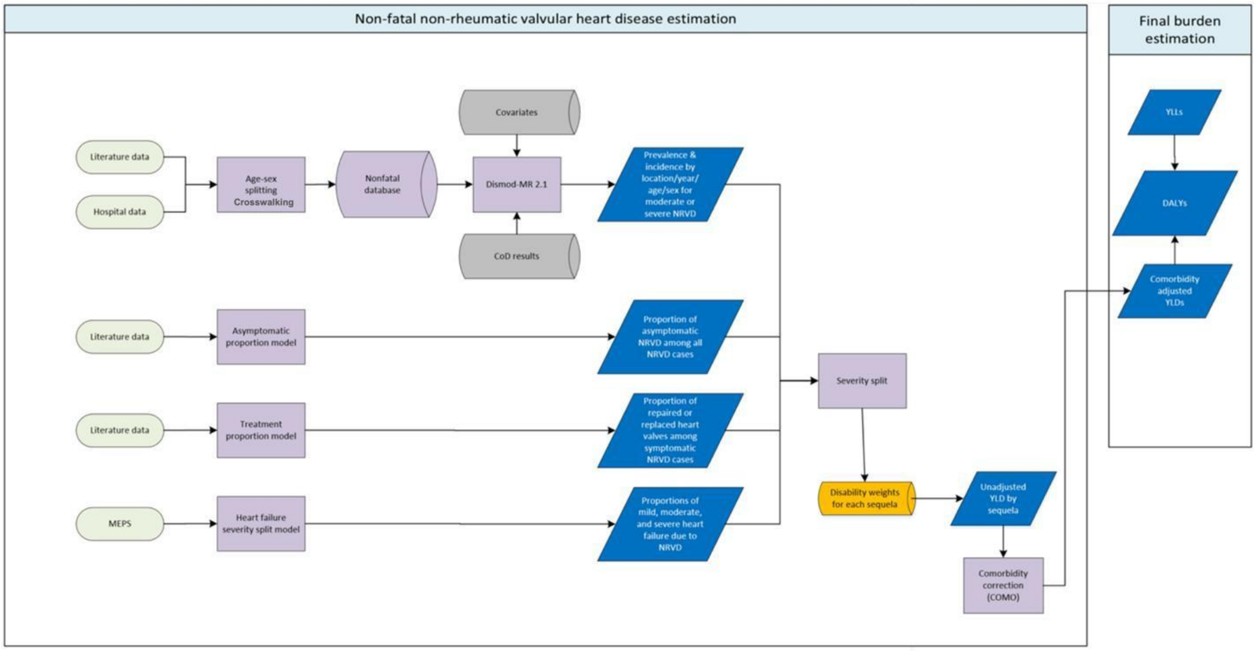

Input data and methodological appendix

Case definitions

Non-rheumatic valvular heart disease

The non-rheumatic valve diseases (NRVD) are a group of cardiac conditions characterised by damage to at least one of the four heart valves. Estimates of NRVD in the GBD do not include valve disease with an aetiology that was congenital, rheumatic, or infectious. Valve disease due to these aetiologies is modelled as part of other causes in the GBD. All NRVD models were restricted to persons at or above the age of 15 to exclude congenital valve disorders. This age restriction is consistent with other progressive cardiovascular diseases modelled in the GBD.

Calcific aortic valve disease

Non-rheumatic calcific aortic valve disease (CAVD) is a condition where the aortic valve in the heart becomes stiff and hard due to the buildup of calcium deposits. CAVD was defined as physician diagnosis based on echocardiographic findings of haemodynamically moderate or severe aortic stenosis or regurgitation according to criteria from the American Heart Association and American College of Cardiology (Table 1). DMVD did not include disease with an aetiology that was congenital, rheumatic, or infectious. Mild haemodynamic aortic stenosis or regurgitation was not included in our case definition because mildly abnormal haemodynamic parameters are difficult to differentiate from non-pathological stenosis and/or regurgitation and are generally not reported in population-based studies.

Table 21: AHA/ACC definitions of moderate/severe aortic stenosis and regurgitation

Stenosis	Maximum jet velocity ≥3 m/s<br>Mean pressure gradient ≥20 mmHg
Regurgitation	Central jet mitral regurgitation ≥25% of the left ventricular outflow tract<br>Vena contracta ≥0.3 cm<br>Regurgitant volume ≥30 mL/beat

	Regurgitant fraction ≥30%<br>Angiography grade ≥2+

Degenerative mitral valve disease

Non-rheumatic degenerative mitral valve disease (DMVD) is a condition where the mitral valve, which separates the two left chambers of the heart, becomes damaged due to weakening of the valve tissue, leading to leakage of blood across the valve. DMVD was defined as physician diagnosis based on echocardiographic findings of haemodynamically moderate or severe mitral regurgitation according to criteria from the American Heart Association and American College of Cardiology (Table 2). DMVD did not include disease with an aetiology that was congenital, rheumatic, infectious, traumatic, carcinoid, or functional (ie, secondary to left ventricular remodelling due to heart failure from another cause). Mitral valve stenosis was considered to have rheumatic aetiology and therefore was not included. Haemodynamically mild mitral regurgitation was not included in our case definition because mild disease is challenging to differentiate from non-pathological regurgitation and is generally not reported in population-based studies.

Table 2: AHA/ACC definitions of moderate/severe mitral regurgitation

Regurgitation	Central jet mitral regurgitation >20% of the left atrium<br>Vena contracta ≥0.7 cm<br>Regurgitant volume ≥60 mL/beat<br>Regurgitant fraction ≥50%<br>Effective regurgitant orifice ≥0.4 cm^2<br>Angiography grade ≥2+

Other non-rheumatic valve disease

Other non-rheumatic valve diseases is a residual category that captures non-rheumatic, non-congenital valve disorders of the tricuspid and pulmonary valves. This includes tricuspid regurgitation, tricuspid stenosis, pulmonary regurgitation, and pulmonary stenosis. Other non-rheumatic valve diseases did not include tricuspid or pulmonary valve disease with an aetiology that was congenital, rheumatic, infectious, traumatic, carcinoid, or functional (ie, secondary to heart failure due to another cause).

Input data

A systematic review was performed for GBD 2017. We searched PubMed using the following search strings:

Calcific aortic valve disease

("aortic stenosis"[Title/Abstract] OR "aortic regurgitation"[Title/Abstract]) NOT ("Transcatheter Aortic Valve Replacement"[MeSH] OR "Transcatheter aortic valve implantation"[KEYWORD]) AND (epidemiology[MeSH Major Topic] OR epidemiology[Subheading] OR epidemiology[MeSH Terms] OR prevalence[Title/Abstract] OR mortality[Title/Abstract]) NOT (animals[MeSH] NOT humans[MeSH]) AND ("1980/1/01"[PDAT] : "2017/12/31"[PDAT]) NOT Comment[ptyp] NOT Case Reports[ptyp]

Degenerative mitral valve disease

("mitral stenosis"[Title/Abstract] OR "mitral regurgitation"[Title/Abstract]) AND ("epidemiology"[MeSH Major Topic] OR "epidemiology"[Subheading] OR "epidemiology"[MeSH Terms] OR prevalence

[Title/Abstract] OR mortality[Title/Abstract]) NOT (animals[MeSH] NOT humans[MeSH]) AND ("1980/1/01"[PDAT] : "2017/12/31"[PDAT]) NOT Comment[ptyp] NOT Case Reports[ptyp]

Other non-rheumatic valve disease

We did not conduct a literature review for other non-rheumatic valve diseases as all estimates were produced as part of the overall heart failure modelling process.

Data on the prevalence of calcific aortic valve and degenerative mitral valve disease were also obtained from inpatient hospital data. These data were adjusted for multiple visits, non-primary diagnoses, and inpatient to outpatient utilisation ratios. Descriptions of search strategies for hospital and claims data and the methodology used to process these data are included elsewhere. Inpatient hospital data were excluded below age 30 or if the age series for a given hospital data source was implausible. Prevalence data from both inpatient and outpatient hospital claims were used in the USA. The final source counts are shown in table 3.

Data on treatment, haemodynamic severity, and asymptomatic status of NRVD were collected from a subset of the studies reporting prevalence as shown in table 4 and 5.

Table 3: Source counts for prevalence models

	Measure	Total sources	Countries with data
Calcific aortic valve disease	Prevalence	260	37
Calcific aortic valve disease	With-condition mortality rate	4	4
Degenerative mitral valve disease	Prevalence	210	32
Degenerative mitral valve disease	With-condition mortality rate	4	3

Table 4: Data on the proportion of individuals haemodynamically moderate or severe valve disease who were haemodynamically moderate

	Measure	Total sources	Countries with data
Calcific aortic valve disease and degenerative mitral valve disease	Proportion	15	4

Table 5: Data on treated calcific aortic and degenerative mitral valve disease

	Measure	Total sources	Countries with data
Calcific aortic valve disease and degenerative mitral valve disease	Proportion	5	5

Data processing

We used the modelling software meta-regression—Bayesian, regularised, trimmed (MR-BRT) to correct for biases in data types. We used a network meta-analysis to adjust inpatient hospital data and MarketScan data to literature data for each cause. Tables 6a and 6b show MR-BRT crosswalk adjustment factors for CAVD and DMVD, respectively. The formula for computing adjustment factors for prevalence is given in equation 1 below.

MR-BRT was used to split both-sex datapoints into sex-specific estimates. This methodology is detailed elsewhere in the appendix. Age-splitting was based on the global sex-specific age pattern obtained from a single-parameter DisMod-MR model done using prevalence data that reported age ranges of less than 25 years collected through systematic review, hospital inpatient/outpatient databases, and survey microdata.

Equation 1: Calculation of adjustment factors:

$$Estimated\ Reference\ Def = invlogit(logit(Alternative\ Def) - Beta_{Alternative\ Def} - Beta_{Age_{scaled}} * Age\ Scaled)$$

Table 6a: MR-BRT adjustment factors for calcific aortic valve disease

Data input	Reference or alternative case definition	Gamma	Beta coefficient, logit (95% CI)
Literature	Reference	0.05	---
Inpatient	Alternate		–0.88 (–1.1 to –0.66)
MarketScan	Alternate		–0.32 (–0.55 to –0.093)
Age, scaled (inpatient)			–0.35 (–0.46 to –0.24)
Age, scaled (MarketScan)			–0.16 (–0.27 to 0.046)

Table 6b: MR-BRT adjustment factors for degenerative mitral valve disease

Data input	Reference or alternative case definition	Gamma	Beta coefficient, logit (95% CI)
Literature	Reference	0.03	---
Inpatient	Alternate		–1.0 (–1.3 to –0.76)
MarketScan	Alternate		–0.50 (–0.75 to –0.26)
Age, scaled (inpatient)			–0.99 (–1.1 to –0.85)
Age, scaled (MarketScan)			–0.41 (–0.55 to –0.27)

MR-BRT crosswalk adjustments can be interpreted as the factor the alternative case definition is adjusted by to reflect what it would have been had it been measured using the reference case definition. If the log/logit beta coefficient is negative, then the alternative is adjusted up to the reference. If the log/logit beta coefficient is positive, then the alternative is adjusted down to the reference.

The adjustment factor column is the exponentiated beta coefficient. For log beta coefficients, this is the relative rate between the two case definitions. For logit beta coefficients, this is the relative odds between the two case definitions.

Overview

We used parallel modelling strategies to model CAVD and DMVD. For other non-rheumatic valve diseases, we estimated non-fatal burden as part of the overall heart failure estimation process. This method is used for most heart failure aetiologies and is described in detail in the appendix section on heart failure.

We first ran cause-specific models to estimate the prevalence and incidence of combined haemodynamically moderate and severe CAVD and DMVD. We then estimated the proportion of those with prevalent disease who were haemodynamically moderate. We next estimated the proportion of those with haemodynamically severe disease who were treated with valve repair or replacement procedures. The remaining proportion – those with untreated symptomatic disease – was split into four proportions: 1) controlled, medically managed; 2) mild; 3) moderate; and 4) severe heart failure. All proportions were calculated and converted to population prevalence at the draw level, thus propagating uncertainty from each step through to all subsequent steps. Population prevalence estimates for each severity level are necessary to accurately calculate the burden for these diseases. Figure 1 visualises this framework. Each of these modelling steps is outlined in greater detail below.

Figure 1: Modelling framework for calcific aortic valve disease and degenerative mitral valve disease

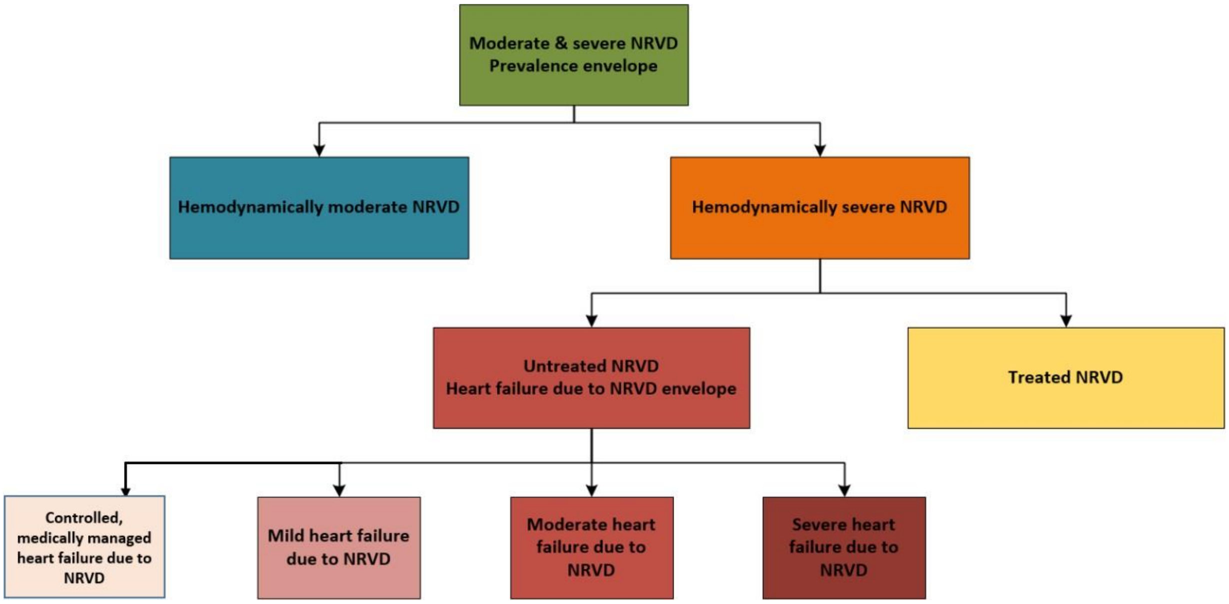

Prevalence envelope

We separately modelled the overall prevalence of CAVD and DMVD in DisMod-MR 2.1. We used cause-specific mortality rates from the fatal modelling process as inputs. These two models estimate the prevalence of these two valve diseases for each age, sex, location, and year. Covariates included in the DisMod models for prevalence of calcific aortic valve and degenerative mitral valve disease are presented in tables 7a and 7b. In each model, we set value priors of 0 for incidence from ages 0 to 15 and a value prior to 0 for remission for all ages.

Table 7a: Covariates and resulting coefficients for CAVD DisMod-MR model

Covariate	Integrand	Coefficients	Exponentiated coefficients
Mean BMI	Prevalence	0.17 (0.17 to 0.18)	1.19 (1.18 to 1.20)
Smoking prevalence	Prevalence	0.0046 (0.00017 to 0.014)	1.00 (1.00 to 1.01)

HAQ Index	Excess mortality rate	–0.03<br>(–0.035 to –0.029)	0.97<br>(0.97 to 0.97)

Table 7b: Covariates and resulting coefficients for DMVD DisMod-MR model

Covariate	Integrand	Coefficients	Exponentiated coefficients
LDI	Excess mortality rate	–0.17 (–0.19 to –0.14)	0.85 (0.83 to 0.87)

Haemodynamically moderate proportion
We estimated the proportion of individuals with overall valve disease who were haemodynamically moderate. There was a total of five data sources (Belgium, Iceland, the Netherlands, Spain, and USA) that reported the proportion of individuals who were haemodynamically moderate. Due to the sparsity of the data, we were not able to generate separate estimates of the haemodynamically moderate proportion for CAVD and DMVD.

We modelled a proportion with uncertainty that varied by age with the following regression:

$$logit(y) = \beta_0 + \beta_1 age + \gamma$$

Where y is the proportion of haemodynamically moderate disease, *age* is the midpoint age foreach datapoint, and γ is a random effect for each data source. The regression coefficients are reported in Table 8.

Table 8: Haemodynamically moderate NRVD regression coefficients.

Covariate	Coefficients	Transformed coefficients
Intercept (β_0)	6.6 (4.9 to 8.4)	0.998 (0.992 to 0.999)
Age (β_1)	–0.07 (–0.093 to –0.047)	0.932 (0.911 to 0.954)

Figure 2: Results of model for haemodynamically moderate NRVD

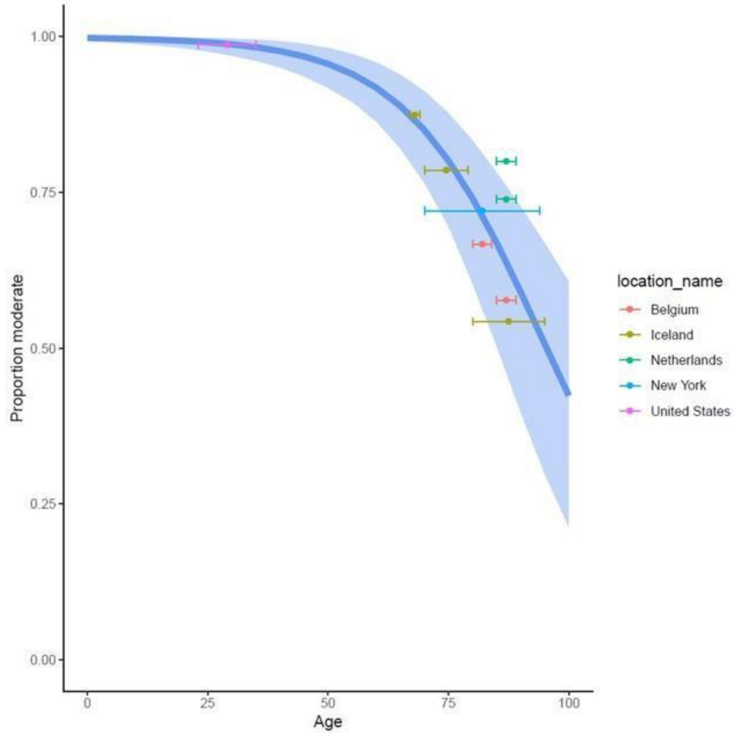

The prevalence of those with haemodynamically moderate valve disease and the prevalence of those with haemodynamically severe disease were calculated using the prevalence envelope and the proportion of those with haemodynamically moderate disease for each five-year age group, sex, location, and year.

Treated proportion

We estimated the proportion of individuals with haemodynamically severe disease who had been treated with valve replacement or repair. We assumed that these procedures were not performed on any individuals with haemodynamically moderate disease. The number of datapoints is reported in Table 9.

These data were all from relatively high-income geographies, yet it is important that we capture the difference in treatment probability based on the likelihood of access to care. Because of this challenge, we ran a regression using the Healthcare Access and Quality (HAQ) Index predicting the level of treatment and set a prior that the proportion of individuals with a valve replacement or repair was zero where HAQ Index was equal to zero. This assumption allowed us to estimate an increasing relationship between HAQ Index and proportion treated, where the estimated proportion treated was based on data where HAQ Index was high. We used the regression equation:

$$logit(y) = \alpha + \beta_1 * haqi + \beta_2 * age + \beta_3 * severity$$

where y is the proportion of individuals with haemodynamically severe disease who had a valve replacement or repair, $haqi$ is the Healthcare Access and Quality Index, age is the midpoint of the age range for a given datapoint, and $severity$ is an indicator variable to adjust for datapoints where the denominator of the proportion treated included both haemodynamically moderate and

haemodynamically severe individuals. The prevalence of those with treated valve disease and the prevalence of those with untreated haemodynamically severe disease were calculated using the prevalence of haemodynamically severe disease and the proportion of those with treated valve disease. The results of this regression are reported in Table 9 and plotted for three ages in Figure 2.

Table 9: Treated calcific aortic valve and degenerative mitral valve disease regression coefficients

**Covariate**	**Coefficients**	**Transformed coefficients**
Intercept (β_0)	–4.69 (–5.90 to –3.43)	0.009 (0.003 to 0.032)
HAQI (β_1)	0.080 (0.070 to 0.089)	1.083 (1.073 to 1.093)
Age (β_2)	–0.029 (–0.04 to –0.015)	0.971 (0.957 to 0.985)
Severity (β_3)	–0.947 (–1.40 to –0.54)	0.377 (0.246 to 0.578)

Figure 3: Results of treatment model for three ages

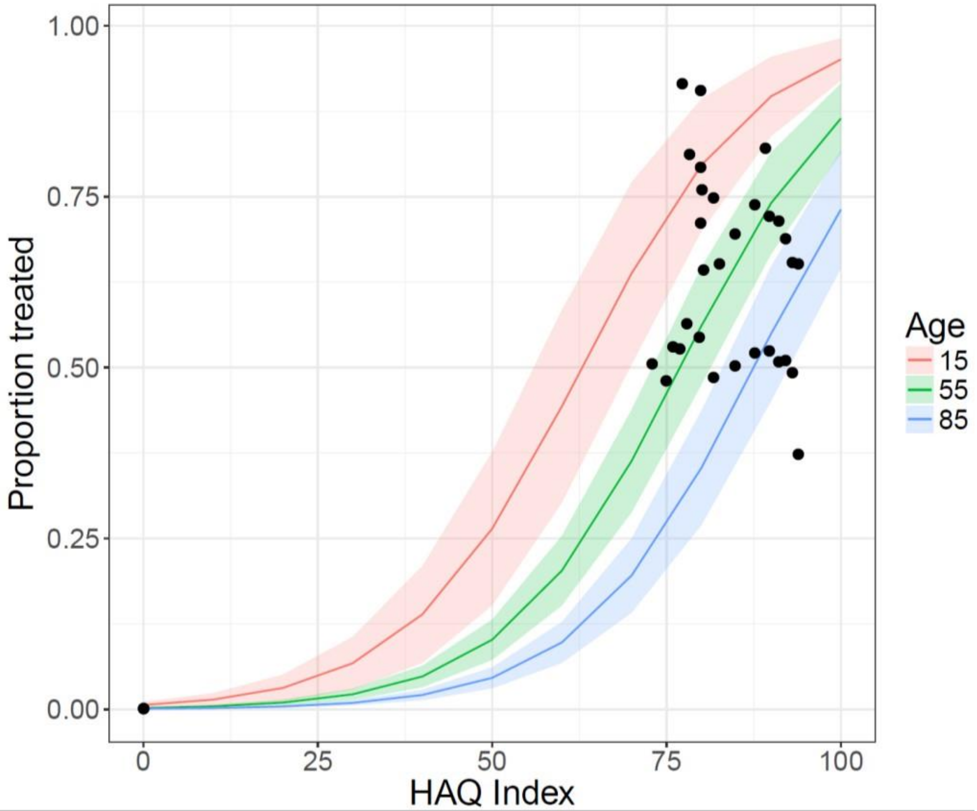

To obtain final estimates for the sequelae of interest, we multiplied the prevalence estimates from the overall CAVD and DMVD prevalence models by the proportion estimated as having haemodynamically moderate disease by age, sex, year, and location to generate the prevalence of haemodynamically moderate and haemodynamically severe CAVD and DMVD. We then multiplied the prevalence estimates for haemodynamically severe disease by the proportion treated with valve replacement or repair procedures by age, sex, year, and location to generate the estimates of prevalence of treated and untreated haemodynamically severe disease. We assumed that all untreated haemodynamically severe

disease would result in heart failure. The proportions of 1) controlled, medically managed, 2) mild, 3) moderate, and 4) severe heart failure due to valve disease were estimated using the approach described in the heart failure section of the appendix. These proportions are based on an analysis of MEPS, a population-based survey that links EQ5D to ICD codes, allowing the application of GBD's standard disability methods.

As the conditions included in the other NRVD category in GBD are rare and usually not recognised until they have become symptomatic, all burden is captured in the four heart failure sequelae. This method is used for most cardiovascular diseases that cause heart failure and is described in detail in the appendix section on heart failure.

The health states, lay descriptions, and associated disability weights for the NRVD sequelae are shown in Table 10.

Table 10: Sequelae, health state lay descriptions, and associated disability weights

Sequela	Health state name	Health state lay description	Disability weight
Asymptomatic non-rheumatic valve disease	Asymptomatic	--	0
Non-rheumatic valve disease after treatment	Generic uncomplicated disease: worry and daily medication	Has a chronic disease that requires medication every day and causes some worry but minimal interference with daily activities.	0.049 (0.031–0.072)
Controlled, medically managed heart failure due to non-rheumatic valve disease	Generic uncomplicated disease: worry and daily medication	Has been diagnosed with clinical heart failure, a chronic disease that requires medication every day and causes some worry but minimal interference with daily activities.	0.049 (0.031–0.072)
Mild heart failure due to non-rheumatic valve disease	Heart failure, mild	Is short of breath and easily tires with moderate physical activity, such as walking uphill or more than a quarter mile on level ground. The person feels comfortable at rest or during activities requiring less effort.	0.041 (0.026–0.062)
Moderate heart failure due to non-rheumatic valve disease	Heart failure, moderate	Is short of breath and easily tires with minimal physical activity, such as walking only a short distance. The person feels comfortable at rest but avoids moderate activity.	0.072 (0.047–0.103)

Severe heart failure due to non-rheumatic valve disease	Heart failure, severe	Is short of breath and feels tired when at rest. The person avoids any physical activity, for fear of worsening the breathing problems.	0.179 (0.122–0.251)

There were no substantive updates to the model for GBD 2021.

Nutritional deficiencies

Nutritional deficiencies is a parent cause for the non-fatal estimation of the following subcauses:

1. vitamin A deficiency
2. iodine deficiency
3. dietary iron deficiency
4. protein-energy-malnutrition
5. other nutritional deficiencies

Since these five subcauses are modelled separately with differences in case definition, input data, strategy, and severity distribution analysis, we present each subcause sequentially.

Vitamin A deficiency

Flowchart

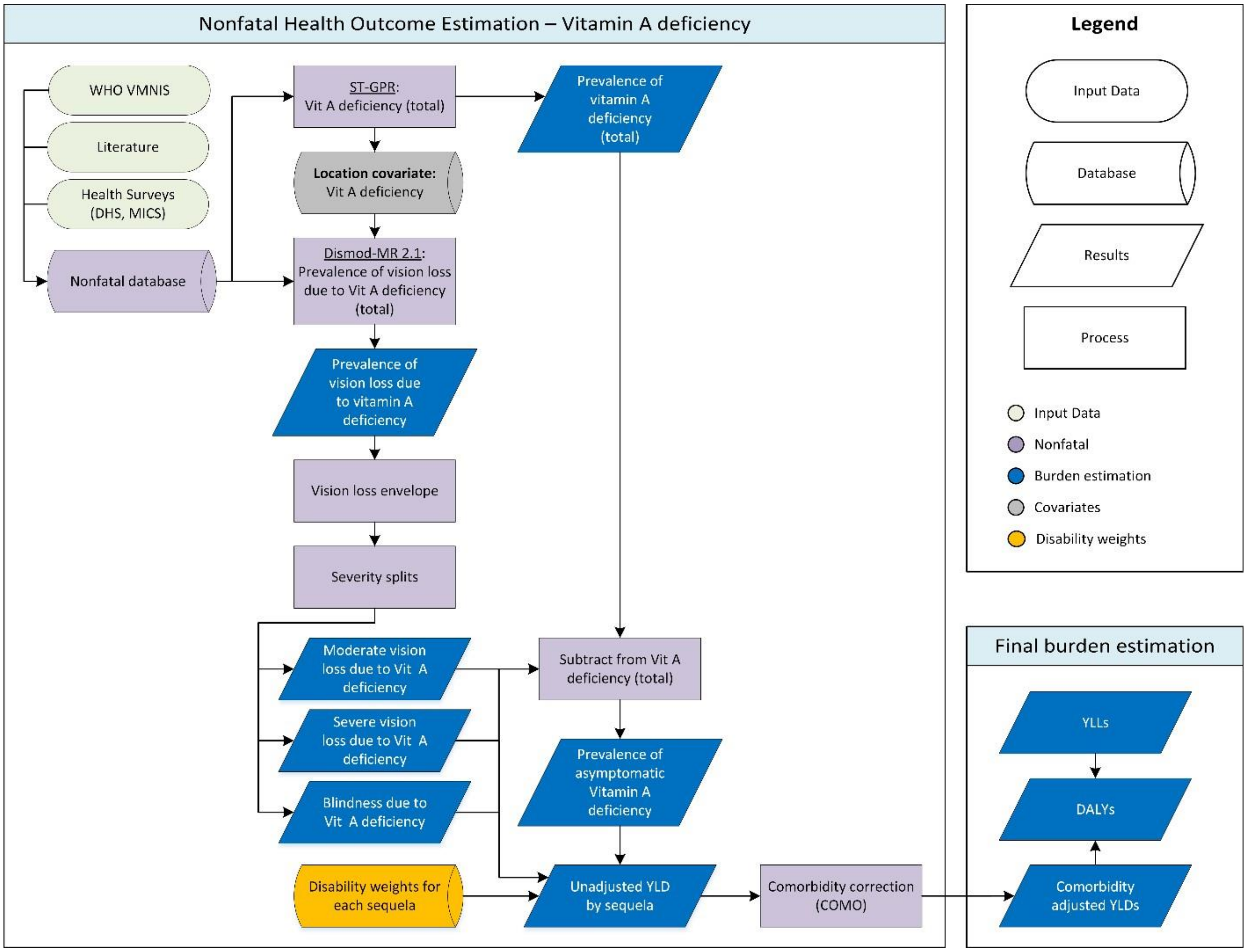

Case definition

Vitamin A deficiency is a condition due to low dietary intake or bioavailability of vitamin A that is inadequate to satisfy physiological needs, which is characterized by low serum or/and breast milk retinol or /and retinol binding concentration, or /and clinical symptoms such as night blindness, xerophthalmia. In GBD 2021, the case definition of vitamin A deficiency is the prevalence of serum retinol concentration < 0.7 µmol/L.

In GBD 2021, the assessment of vitamin A deficiency burden involves the quantification of total vitamin A deficiency, anemia due to vitamin A deficiency as well as blindness and vision loss due to vitamin A deficiency, which are associated with corneal ulcerations and corneal scars.

Input data

For GBD 2021, we used data from the WHO Vitamin and Mineral Nutrition Information System, health surveys such as DHS and MICS, and studies identified through literature review for thevitamin A deficiency model. We used data from the WHO Vitamin and Mineral Nutrition Information System for the vision loss model. Table 1 provides a summary of data inputs for vitamin A deficiency modelling. A systematic review was last conducted for GBD 2013. The PubMed search terms were: ((vitamin A

deficiency[Title/Abstract] AND prevalence[Title/Abstract]) AND (“2009”[Date – Publication] : “2013”[Date – Publication])). Exclusion criteria were:

1. Studies that were not population-based, eg, hospital or clinic-based studies
2. Studies that did not provide primary data on epidemiological parameters, eg, commentaries
3. Review articles
4. Case series
5. Self-reported cases

Table 1: Data inputs for vitamin A deficiency morbidity modelling by parameter.

Measure	Total sources	Countries with data
All measures	320	101
Prevalence	46	27
Proportion	274	96

Modelling strategy

No major changes to the modelling strategy for vitamin A deficiency were made in GBD 2021 as compared to GBD 2019. However, the covariates used in the vitamin A deficiency model were updated in GBD 2021. Specifically, the vitamin A deficiency model now uses logit SDI instead of SDI, and no longer includes vitamin A supplementation coverage as a covariate in the vitamin A deficiency model. The prevalence of vitamin A deficiency was used as a location-level covariate to guide prevalence estimates of vision loss due to vitamin A deficiency. The difference between total vitamin A deficiency and vision loss due to vitamin A deficiency is considered asymptomatic. We also attribute a portion of the anemia hemoglobin shift to vitamin A deficiency for children younger than 15 years old. Total vitamin A deficiency is separately considered as a risk factor in the GBD comparative risk assessment analysis.

We estimated the age- and sex-specific prevalence of vitamin A deficiency (serum retinol < 0.7 µmol/L). In GBD 2019, we updated the deficiency data processing steps to include a separate sex ratio model (using meta-regression–Bayesian, regularised, trimmed (MR-BRT)) and a separate age pattern model (using DisMod-MR) which were used to split both-sex and all-age data prior to modelling. Additionally, in GBD 2019, we moved vitamin A deficiency to ST-GPR to utilise its superior time trends. In GBD 2021, the vitamin A ST-GPR model utilized three location-level covariates: age-specific stunting SEV, Socio-demographic Index (logit scale), and the availability of retinol activity equivalent (RAE) units in foods. Vitamin A supplementation was omitted as a covariate in the vitamin A deficiency model due to its lack of significance in the ST-GPR model. We also observed that when the coverage of vitamin A supplementation was included as a covariate in the vitamin A deficiency STGPR model, an unusual temporal trend was observed. Since GBD 2019, we have introduced the assumption that the duration of vitamin A deficiency is one year, which implies that prevalence and incidence are equal.

The vision loss due to vitamin A deficiency model was run as a single-parameter meta-regression on prevalence in DisMod-MR with vitamin A deficiency prevalence as a location-level covariate. The case definition for vision loss due to vitamin A deficiency is aligned with the WHO Vitamin and Mineral Nutrition Information System database’s definition of a corneal scar. In GBD 2019 we modelled the sex

ratio for vision loss due to vitamin A deficiency outside of DisMod-MR using MR-BRT and applied this ratio to split both-sex data prior to DisMod-MR modelling.

Table 2. Covariates. Summary of covariates used in the vitamin A deficiency models(GBD 2021)

Vitamin A model	Modelling strategy	Covariate	Type	Parameter
Vitamin A Deficiency	ST-GPR	Vitamin A rae unadjusted (g)	Country-level	Prevalence
	ST-GPR	Stunting SEV	Country-level	Prevalence
	ST-GPR	SDI	Country-level	Prevalence
Vision loss	DisMod-MR	Vitamin A deficiency (age standardised)	Country-level	Prevalence

Our GBD results include explicit estimates of total vitamin A deficiency, although those without vision loss are assumed to be asymptomatic. Description of how our estimates of total vision loss described above are parsed into moderate vision loss, severe vision loss, and blindness can be found in the modelling description for the "vision loss impairment". Sequelae and corresponding disability weights for each of the health states associated with vitamin A deficiency are shown in Table 3.

Table 3. Severity, lay description, and disability weight (DW)

Sequela	Health state name	Lay description	Disability weight
Asymptomatic vitamin A deficiency	Asymptomatic	--	--
Moderate vision impairment loss due to vitamin A deficiency	Distance vision, moderate impairment	Has vision problems that make it difficult to recognise faces or objects across a room.	0.031 (0.019–0.049)
Severe vision impairment loss due to vitamin A deficiency	Distance vision, severe impairment	Has severe vision loss, which causes difficulty in daily activities, some emotional impact (for example worry), and some difficulty going outside the home without assistance.	0.184 (0.125–0.258)
Blindness due to vitamin A deficiency	Distance vision blindness	Is completely blind, which causes great difficulty in some daily activities, worry and anxiety, and great difficulty going outside the home without assistance.	0.187 (0.124–0.26)
Mild anaemia due to vitamin A deficiency	Mild anaemia	Feels slightly tired and weak at times, but this does not interfere with normal daily activities.	0.004 (0.001 – 0.008)
Moderate anaemia due to vitamin A deficiency	Moderate anaemia	Feels moderate fatigue, weakness, and shortness of breath after exercise, making daily activities more difficult.	0.052 (0.034 – 0.076)

Severe anaemia due to vitamin A deficiency	Severe anaemia	Feels very weak, tired and short of breath, and has problems with activities that require physical effort or deep concentration.	0.149<br>(0.101 – 0.209)

Iodine deficiency

Flowchart

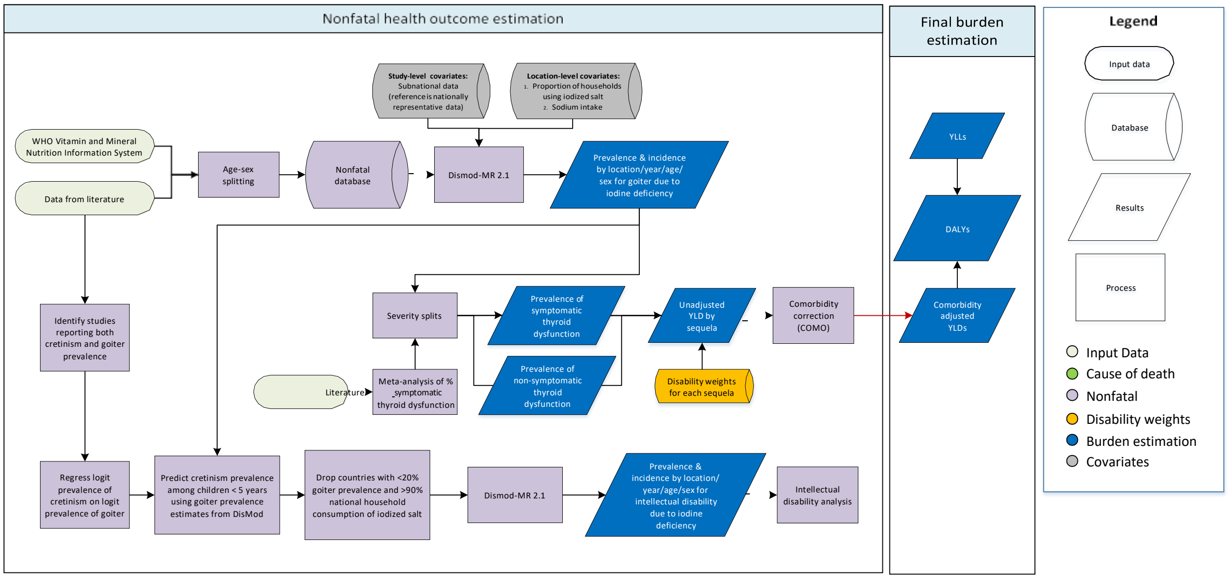

Case definition

Iodine deficiency is a condition characterized by impaired production of thyroid hormones due to insufficient iodine intake which results in adverse health ranging from thyroid gland enlargement(goiter) to severe physical and mental retardation. Our assessment of the non-fatal burden of iodine deficiency includes estimates of only the subset of iodine deficiency associated with visible goitre (grade 2) and its associated sequelae, including thyroid dysfunction, heart failure, and intellectual disability (historically referred to as “cretinism”). It does not include estimates of sub-clinical iodine deficiency or non-visible goitre (grade 1) induced by iodine deficiency.

Input data

For GBD 2021, data from the WHO Vitamin and Mineral Nutrition Information System and published studies were used for the visible goitre model (Table 1). The extraction and accompanying systematic review were last conducted for GBD 2013. The PubMed search terms were: ((iodine deficiency[Title/Abstract] AND prevalence[Title/Abstract]) AND (“2009”[Date – Publication] : “2013”[Date – Publication]))

The exclusion criteria were:

17. Studies that were not population-based, eg, hospital or clinic-based studies
18. Studies that did not provide primary data on epidemiological parameters, eg, commentaries
19. Review articles
20. Case series
21. Self-reported cases

Updates to systematic reviews are performed on an ongoing schedule across all GBD causes; an update for iodine deficiency will be performed in the next iteration

Table 1: Data Inputs for iodine deficiency morbidity modelling by parameter.

Measure	Total sources	Countries with data
All measures	207	81
Prevalence	201	78
Incidence	0	0
Remission	0	0
Other	6	4

All input data for iodine deficiency is already in our gold-standard case definition (prevalence of visible goitre and prevalence of intellectual disability due to iodine deficiency), so no bias corrections are needed.

Modelling strategy

The iodine deficiency modelling strategy includes iodine deficiency and associated sequelae heart failure, thyroid dysfunction, and intellectual disability. The process is comprised of two models for visible goitre and intellectual disability due to iodine deficiency and severity splits for the other sequela. No changes to the modelling strategy were made in GBD 2021.

In GBD 2019, we implemented a two-step process to estimating the prevalence of grade 2 goitre which we carried into GBD 2021. We first used all available data to construct an age pattern model that captured the prevalence age-trend in the data, which was used to split data spanning an age range greater than 25 years into narrower age bins. Then we modelled the prevalence of visible goitre using the new age split data. In this model, we introduced several new assumptions: visible goitre incidence can be non-decreasing across age (ie, we removed a decreasing slope prior), a small amount of remission is possible, and birth prevalence is not possible. These assumptions were based on evidence in the literature showing that the highest levels of visible goitre are in middle aged people and were prompted by observing that the previously strict parameters were limiting the predictive power of the model. We also continued to use proportion of households using iodised salt and sodium intake as country-level covariates. The coefficients for these covariates are in the table below.

Table 2. Visible goitre covariates. Summary of covariates used in the visible goitre DisMod-MR meta-regression model

Covariate	Type	Parameter	Exponentiated beta (95% UI)

Proportion of households using iodised salt	Country-level	Prevalence	0.0028 (0.0024 – 0.0034)
Sodium intake	Country-level	Prevalence	1.11 (1.08-1.13)

For GBD 2021, no changes were made to the strategy for the intellectual disability model. Consistent with the GBD 2017 and GBD 2019 approach, we estimated the prevalence of intellectual disability due to iodine deficiency (cretinism) by regressing datapoints from studies reporting both cretinism and goitre prevalence in the same population. To do so, we first transformed cretinism prevalence and goitre prevalence into logit space, regressed the logit prevalence of cretinism on the logit prevalence of goitre, and predicted for all national locations using the goitre estimates from the DisMod-MR 2.1 model above. We dropped locations with total goitre prevalence less than 20% and locations with household iodised salt consumption greater than 90%. We kept observations in children younger than 5 years and used these data as incidence input in a second DisMod-MR 2.1 to generate location-year-age-sex-specific estimates. This was combined with relative risk (RR) and standardised mortality ratio (SMR) data on intellectual disability identified in the literature review described above. We modelled with zero remission, zero incidence after age 5, and proportion of households using iodised salt as a covariate on incidence (Table 3). We repeated the dropout criteria of total goitre prevalence and iodised salt consumption on the DisMod-MR 2.1 output.

Table 3. Intellectual disability due to iodine deficiency covariates. Summary of covariates used in the intellectual disability due to iodine deficiency DisMod-MR meta-regression model

Covariate	Type	Parameter	Exponentiated beta (95% UI)
Proportion of households using iodised salt	Country-level	Incidence	0.14 (0.14-0.14)

The severity split distribution did not change for GBD 2021. Initial severity proportions are: visible goitre without symptoms of thyroid dysfunction (proportion=0.915, 95% confidence interval (CI): 0.904–0.926); goitre with symptoms of thyroid dysfunction (proportion=0.085, 95% confidence interval (CI): 0.084–0.086). Additionally, we split the intellectual disability due to iodine deficiency model into severe and profound ID using ID proportion assumptions. Everyone with ID is assumed to have thyroid dysfunction, while heart failure is assumed to only occur in people with profound intellectual disability (which we split into mild, moderate and severe heart failure). Heart failure attributable to iodine deficiency was modelled separately, and the methods for this outcome are presented separately in the section for heart failure and its etiologies. Table 4 provides details on the severity states downstream of iodine deficiency.

Table 4. Severity distribution, details on the severity levels for iodine deficiency in GBD 2019 and the associated disability weight (DW) with that severity.

Sequela	Health state name	Lay description	Disability weight
Visible goitre without symptoms	Disfigurement, level 1	Has a slight, visible physical deformity that others notice, which causes some worry and discomfort.	0.011 (0.005–0.021)

Visible goitre with symptoms without intellectual disability or heart failure	Iodine-deficiency goitre	Has a large mass in the front of the neck. The person sometimes has weakness and fatigue, constipation and weight gain.	0.199 (0.133–0.276)
Visible goitre with severe intellectual disability due to iodine deficiency	Intellectual disability / mental retardation, severe	Has very low intelligence and cannot speak more than a few words, needs constant supervision and help with most daily activities, and can do only the simplest tasks.	0.326 (0.233–0.438)*
	Iodine-deficiency goitre	(see above)	
Visible goitre with profound intellectual disability due to iodine deficiency	Intellectual disability / mental retardation, profound	Has very low intelligence, has almost no language, and does not understand even the most basic requests or instructions. The person requires constant supervision and help for all activities.	0.358 (0.252–0.475)*
	Iodine-deficiency goitre	(see above)	
Visible goitre with profound intellectual disability and mild heart failure due to iodine deficiency	Intellectual disability / mental retardation, profound	(see above)	0.384 (0.276–0.502)*
	Iodine-deficiency goitre	(see above)	
	Heart failure, mild	Is short of breath and easily tires with moderate physical activity, such as walking uphill or more than a quarter-mile on level ground. The person feels comfortable at rest or during activities requiring less effort.	
Visible goitre with profound intellectual disability and moderate heart failure due to iodine deficiency	Intellectual disability / mental retardation, profound	(see above)	0.403 (0.293–0.524)*
	Iodine-deficiency goitre	(see above)	
	Heart failure, moderate	Is short of breath and easily tires with minimal physical activity, such as walking only a short distance. The person feels comfortable at rest but avoids moderate activity.	
Visible goitre with profound intellectual disability with severe heart failure due to iodine deficiency	Intellectual disability / mental retardation, profound	(see above)	0.471 (0.344–0.602)*
	Iodine-deficiency goitre	(see above)	
	Heart failure, severe	Is short of breath and feels tired when at rest. The person avoids any physical activity, for fear of worsening the breathing problems.	

Dietary iron deficiency

Flowchart

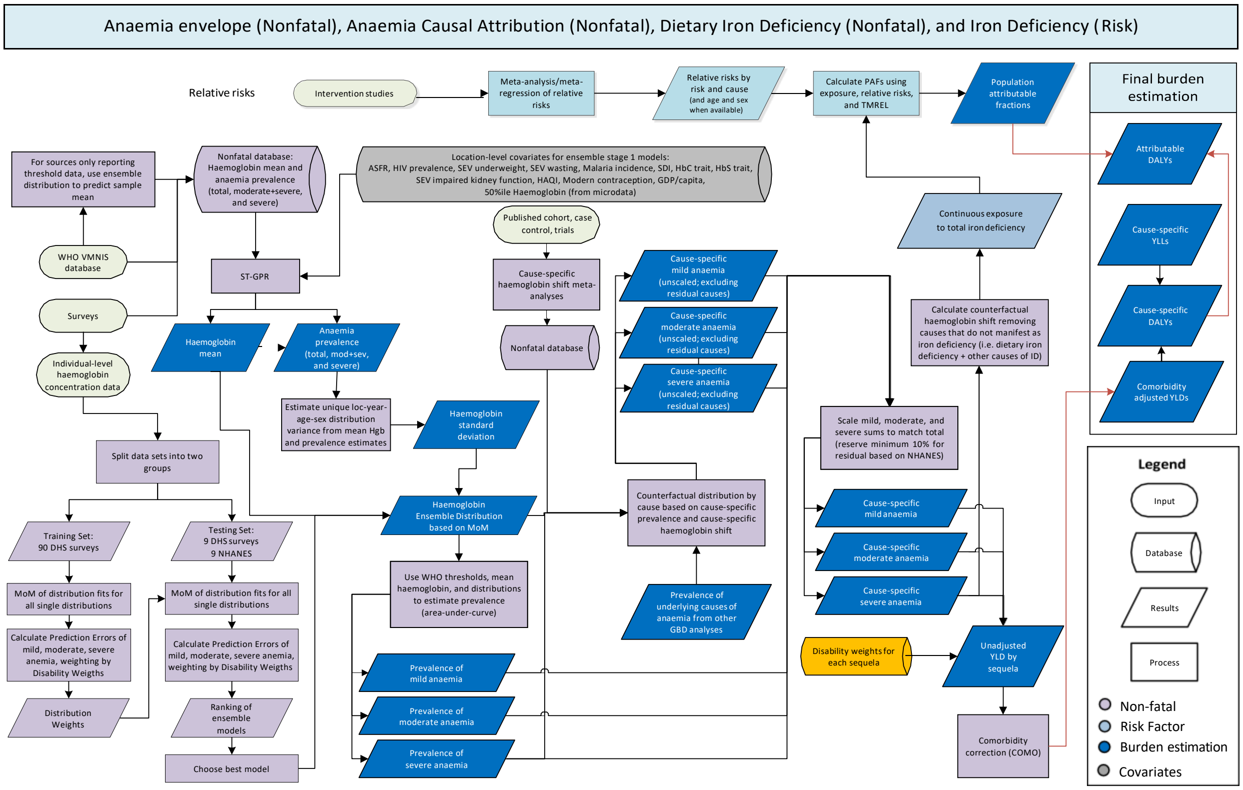

Case definition

Dietary iron deficiency in the GBD cause analysis is defined as mild, moderate, or severe anemia that is the result of inadequate dietary intake of iron, but not due to other causes of inadequate absolute or functional iron availability to meet the body's needs.

Methodological summary

Dietary iron deficiency was quantified as an output of the GBD Anaemia Causal Attribution framework. The GBD anaemia model has two main steps – estimation of the anaemia envelope and causal attribution – both of which inherently impact estimates of iron deficiency. See the methodological description of "Anaemia (Impairment)" for detailed description of the analytic approach and inputs.

Briefly, the first step is estimating anaemia envelope – the prevalence of mild, moderate, and severe anaemia prevalence for each GBD location, age-group, sex, and year. The inputs to the envelope model are mean and standard deviation (SD) of haemoglobin [Hb] concentration. Mean haemoglobin is modelled directly in ST-GPR, and standard deviation is estimated using a variance optimisation algorithm that takes as inputs the modelled mean haemoglobin estimates and estimates of the prevalence of severe, moderate+severe, and total anaemia (also modelled in ST-GPR). For every location, year, age, and sex we anchor the distributions at the estimated mean [Hb] value and find the variance value that minimises the error between our ST-GPR estimates of severe, moderate+severe, and total anaemia and the corresponding values implied by a given mean and variance [Hb] combination.

Individual-level data sources are then used to develop a set of ensemble distribution weights using method of moments, which are then paired with mean and SD results to produce estimates of the entire distribution of haemoglobin for each population group. A population group is a specific geography, sex, age-group, and year combination. The second step is anaemia causal attribution, which generates counterfactual haemoglobin distributions for each cause of anaemia based on the cause-level prevalence (or incidence, in the case of maternal haemorrhage) estimates from the respective GBD analyses and cause-specific haemoglobin shifts that were determined via meta-analysis for each cause. The counterfactual distribution methods used the same ensemble distribution weights as the overall anaemia envelope because there is inadequate data to guide alternate distributions for each subcause. Mild, moderate, and severe anaemia were assigned to each cause based on the difference between the counterfactual and observed haemoglobin distributions in each population group. The sum of severity-specific prevalence was then summed to match the total, with a minimum residual of 10%,[1,2] and then the remainder was distributed between five GBD causes using fixed proportion redistribution methods: 1) dietary iron deficiency (GBD cause), 2) other haemoglobinopathies and haemolytic anaemias, 3) other infectious diseases, 4) other neglected tropical disease, and 5) endocrine, metabolic, blood, and immune disorders.

It is important to take note of the difference between “dietary iron deficiency” as a GBD cause and “iron deficiency” as a GBD risk. Many GBD causes lead to anaemia that clinically manifests as iron deficiency (or microcytosis), but where inadequate intake is not the underlying problem. Examples include neglected tropical diseases such as hookworm, malaria, and schistosomiasis, gastrointestinal disorders, cirrhosis, maternal haemorrhage, menstrual disorders, uterine fibroids, and vitamin A deficiency. The name “dietary iron deficiency” is intended to differentiate, therefore, between inadequate dietary intake of iron and haemorrhagic or disorders of iron metabolism. Additionally, because we have yet to include 100% of anaemia causes, estimates should be interpreted to also include some acute and chronic haemorrhagic states for which supplementation may be helpful, but poor nutritional intake is not the only underlying problem. Examples include malabsorption syndromes, other micronutrient deficiencies besides vitamin A deficiency, and injuries with associated acute blood loss anaemia. “Iron deficiency” exposure as estimated for the GBD risk factors analysis, in contrast, includes a combined assessment of the magnitude of haemotologic insult from all causes that manifest as iron deficiency. Our goal is to systematically add all causes of anaemia as specific inputs to GBD Anaemia Causal Attribution, including inadequate iron intake, and eliminate the need for residual attribution.

Protein-energy malnutrition

Flowchart

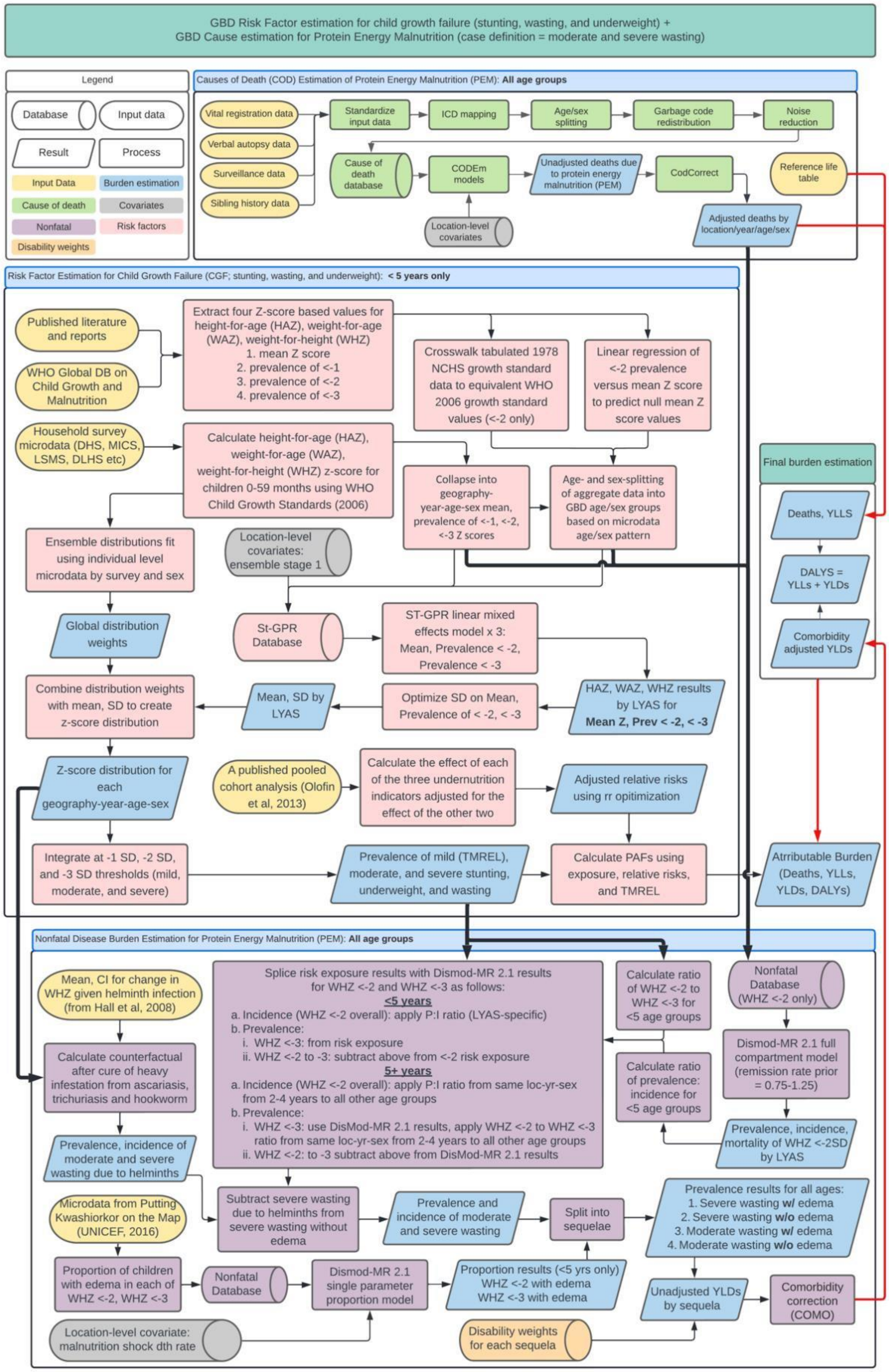

Case definition

Protein-energy malnutrition (PEM) includes moderate and severe acute malnutrition, commonly referred to as "wasting," and was defined in terms of weight-for-height Z-scores (WHZ) on the WHO 2006 growth standard for children. We quantified non-fatal PEM burden in four mutually exclusive and collectively exhaustive categories, reflecting distinct gradations of disability that can occur: moderate wasting ***without oedema*** (WHZ < -2SD to < -3 SD), moderate wasting ***with oedema*** (WHZ < -2SD to < -3 SD), severe wasting ***without oedema*** (WHZ < -3SD), and severe wasting ***with oedema*** (WHZ < -3SD). The aggregate of categories that include "oedema" can be considered equivalent to the disease state commonly referred to as "kwashiorkor" and severe wasting can likewise be considered equivalent to "marasmus." For PEM, ICD 10 codes are E40-E46.9, E64.0, and ICD 9 codes are 260-263.9.

This classification reflects a moderate shift from GBD 2015, when moderate wasting without oedema was not included in our non-fatal estimates, and by definition is associated with higher prevalence estimates than previously published by GBD. The other GBD 2015 categories – kwashiorkor, marasmus, and severe wasting – have unchanged case definitions, but have been renamed for clarity and consistency. This revised GBD 2016 case definition more closely aligns with other and allows for better application to the international nutrition community's programming and estimates related to non-fatal PEM. This change continued into GBD 2019.

Input data and data processing

The input data for this model come in two primary streams. First, we used individual-level and tabulated child anthropometry data from health surveys, literature, and national reports, and centralised them to inform the prevalence of WHZ decrement in each category corresponding to our case definitions. For details on estimation of wasting (WHZ <-2 and WHZ <-3) data identification and processing, see the methodological description of "Child Growth Failure" in the GBD 2021 Risk Factors appendix. Second, to inform the proportion of children under 5 years who have signs of organ failure manifested as oedema (ie, kwashiorkor), we used a compiled dataset of surveys conducted using Standardised Monitoring and Assessment of Relief and Transitions (SMART) methods. All data were extracted with the most detailed standard demographic identifiers available, including age, sex, country, year, and subnational location if available. No alternate case identifications were identified for oedema data so no crosswalks were required or performed.

Table 1a. Dataset contents for total wasting (moderate + severe, with and without oedema)

	**Prevalence**
Site-years (total)	1781
Number of countries with data	160
Number of GBD regions with data (out of 21 regions)	21
Number of GBD super-regions with data (out of seven super-regions)	7

Table 1b. Dataset contents for proportion of oedema among total wasting

	**Proportion**
Site-years (total)	240
Number of countries with data	45
Number of GBD regions with data (out of 21 regions)	12
Number of GBD super-regions with data (out of seven super-regions)	6

Table 2c. Dataset contents for proportion of oedema among severe wasting

	Proportion
Site-years (total)	240
Number of countries with data	45
Number of GBD regions with data (out of 21 regions)	12
Number of GBD super-regions with data (out of seven super-regions)	6

Modelling Strategy

We used five parallel models to inform our estimates, all of which produced age-sex-specific results: 1) Prevalence of WHZ <-2 in children under 5 years in ST-GPR, 2) Prevalence of WHZ <-3 in children under 5 years in ST-GPR, 3) Proportion of those with WHZ <-2 who have oedema in under 5 years in DisMod-MR 2.1, 4) Proportion of those with WHZ <-3 who have oedema in under 5 years in DisMod-MR 2.1, and 5) Prevalence, incidence, and excess mortality of WHZ <-2 in all ages in DisMod-MR 2.1.

Using available information from scientific publications, which suggest the mean duration of illness is nine months, and conversations with collaborators and nutrition experts, we applied what we consider a plausible set of remission rate bounds of 0.25–1.25 (# of remitted cases of PEM per person-year of illness) to the final of the five models. These bounds allowed DisMod-MR to mathematically derive an internally consistent solution for incidence, prevalence, remission, excess mortality, and cause-specific mortality using all available data. This could only be done for the aggregate PEM definition (prevalence of WHZ <-2) to ensure that the case definition for prevalence matched that of the mortality results. The incidence-to-prevalence ratio derived from the final model was applied equally across all the categories of non-fatal PEM. Future work in systematically evaluating longitudinal datasets on nutrition and growth failure will allow us to improve the empirical basis for PEM incidence estimates, including improved resolution for the component categories.

For details on estimation of wasting (WHZ <-2 and WHZ <-3) estimation, see the methodological description of "Child Growth Failure" in the GBD 2021 Risk Factors appendix. Location-level covariate effects for each of the three DisMod-MR 2.1 models are shown in the tables below. The two DisMod-MR models shown below for proportion of oedema among total and severe wasting were most recently run in GBD 2019. For GBD 2021, model results were imputed for from the GBD 2019 models, applying results from parent age groups to new, increasingly disaggregated age groups.

Table 2a: Location-level covariate effects for proportion of oedema among total wasting

Measure	Covariate	Beta value	Exponentiated
Proportion	Energy unadjusted (kcal)	-1 (-1 - -1)	0.37 (0.37–0.37)
Proportion	Malnutrition shock log-transformed mortality rate	1 (1 - 1)	2.72 (2.72 – 2.72)

Table 2b. Location-level covariate effects for proportion of oedema among severe wasting

Measure	Covariate	Beta Value	Exponentiated
Proportion	energy unadjusted(kcal)	-1 (-1 - -1)	0.37 (0.37–0.37)

Proportion	Malnutrition shock log-transformed mortality rate	1 (1 - 1)	2.72 (2.72–2.72)

Table 2c. Location-level covariate effects for total wasting (moderate + severe, with and without oedema)

Measure	Covariate	Beta Value	Exponentiated
Prevalence	Sanitation (prop access)	-0.00044 (-0.0016 — -0.000015)	1.00 (1.00 — 1.00)
Prevalence	Socio-demographic Index	-0.87 (-0.92 - -0.80)	0.42 (0.40 — 0.45)
Prevalence	Malnutrition Shock, log-trans mortality rate	0.0028 (0.00042 — 0.0055)	1.00 (1.00 — 1.01)
Excess mortality rate	Healthcare Access and Quality Index	-0.064 (-0.064 — -0.063)	0.94 (0.94 – 0.94)

The results of the first four models were used for children under 5 years. Arithmetic transformations were performed to ensure that the final results fit into the mutually exclusive, collectively exhaustive categories of moderate and severe wasting, with and without oedema. We assumed zero prevalence of oedema in people over 5 years old. The results of the final model were used for all age groups 5 years and older and the proportion of moderate versus severe wasting in each of those age groups was derived from the first set of models.

As a final step, we subtracted a number of cases of PEM where the underlying aetiology is severe worm infestation. See the appendix section on “Neglected Tropical Diseases” for more details of that process. Briefly, because both worms and PEM can cause wasting, we needed to divide out the wasting envelope to attribute wasting to both PEM and worms. We determined the amount of wasting attributable to worms by referencing Hall and colleagues[1] to determine the mean and confidence interval estimates of the z-score shift. We then calculated the counterfactual wasting prevalence given no worms, according to the z-score shift. From this, we calculated the fraction of wasting that is attributable to worms and assigned the remainder of wasting to PEM. We assumed no oedema due to worms and the same prevalence-to-incidence ratio as in each of the other models.

We applied disability weights from the GBD disability weight survey to the prevalence of the above sequelae according to their corresponding health state and severity level. The sequelae, along with their lay descriptions and disability weights for health states derived from the GBD disability weights study, are shown below. We assumed that those with moderate wasting, but no oedema, did not have any direct disability due to this condition.

Table 3. Sequelae, severity, lay description, and DWs

Sequela	Health state name	Lay description	DW (95% CI)
Moderate wasting without oedema	Asymptomatic	--	--
Moderate wasting with oedema	Kwashiorkor	Is very tired and irritable and has diarrhoea.	0.051 (0.031–0.079)
Severe wasting without oedema	Severe wasting	Is extremely skinny and has no energy.	0.128 (0.082–0.183)
Severe wasting with oedema	Kwashiorkor + severe wasting	Is very tired and irritable and has diarrhoea.	0.051 (0.031–0.079)
		Is extremely skinny and has no energy.	0.128 (0.082–0.183)

Following the assignment of disability weights to the various sequelae, the resulting years lived with disability (YLDs) go through the comorbidity simulator, which accounts for any comorbidity and corrects accordingly. The final outputs are comorbidity-adjusted YLDs, which are combined with years of life lost (YLLs) for final disability-adjusted life years (DALYs).

References

[1] Hall A, Hewitt G, Tuffrey V, de Silva N. A review and meta-analysis of the impact of intestinal worms on child growth and nutrition. Maternal and Child Nutrition. 2008. 4. 118-236.

Other nutritional deficiencies

Other nutritional deficiencies encompass a wide variety of causes of morbidity, ranging from vitamin deficiencies to other nutritional anaemias. In GBD 2019, as done previously, we treat these causes as a single category, given their relatively limited burden, diversity in underlying causes and risk factors, and data availability. Instead of modelling them in a traditional modelling format, we calculate the YLDs associated with other nutritional deficiencies using a YLD/YLL ratio.

The first input for this non-fatal portion of other nutritional deficiencies burden is the YLL estimates from the GBD 2019 causes of death (CoD) analysis. The causes and their associated ICD-10 codes that constitute other nutritional deficiencies for CoD are listed below. Additionally, CoD includes specific models for protein-energy malnutrition, another nutritional cause of morbidity and mortality; as protein-energy malnutrition has a specific non-fatal model that results in YLDs, we can calculate the YLD/YLL ratio for protein-energy malnutrition. We multiply the YLL estimates for other nutritional deficiencies from CoD by the YLD/YLL ratio for PEM, providing us with an estimate of the YLDs associated with other nutritional deficiencies. There were no changes in modelling strategy for other nutritional deficiencies from GBD 2017.

Table 1. Definitions, ICD-10 codes and descriptions included in the other nutritional deficiencies model

GBD cause	ICD-10 code
Other nutritional deficiencies	D51-D52.0 (vitamin B12 deficiency anaemia and folate deficiency anaemia)
Other nutritional deficiencies	D52.8-D53.9 (other nutritional anaemias)
Other nutritional deficiencies	D64.3 (other sideroblastic anaemias)
Other nutritional deficiencies	E51-E61.9 (thiamine, niacin, other B group vitamins, ascorbic acid, vitamin D, other vitamin, dietary calcium, dietary selenium, dietary zinc, and other nutrient element deficiencies)

Other nutritional deficiencies	E63-E64.0 (other nutritional deficiencies and sequelae of protein-calorie malnutrition)
Other nutritional deficiencies	E64.2-E64.9 (sequelae of vitamin C deficiency, rickets, other nutritional deficiencies, and unspecified nutritional deficiencies)
Other nutritional deficiencies	M12.1-M12.19 (Kaschin-Beck disease)

References

1 Centers for Disease Control and Prevention (CDC). Iron deficiency--United States, 1999-2000. *MMWR Morb Mortal Wkly Rep* 2002; **51**: 897–9.

2 Looker AC, Dallman PR, Carroll MD, Gunter EW, Johnson CL. PRevalence of iron deficiency in the united states. *JAMA* 1997; **277**: 973–6.

3 Murray-Kolb LE, Chen L, Chen P, Shapiro M, Caulfield L. CHERG Iron Report: Maternal Mortality, Child Mortality, Perinatal Mortality, Child Cognition, and Estimates of Prevalence of Anemia due to Iron Deficiency | GHDx. 2013. http://ghdx.healthdata.org/record/cherg-iron-report-maternal-mortality-child-mortality-perinatal-mortality-child-cognition-and (accessed Nov 12, 2019).

Onchocerciasis

Flowchart

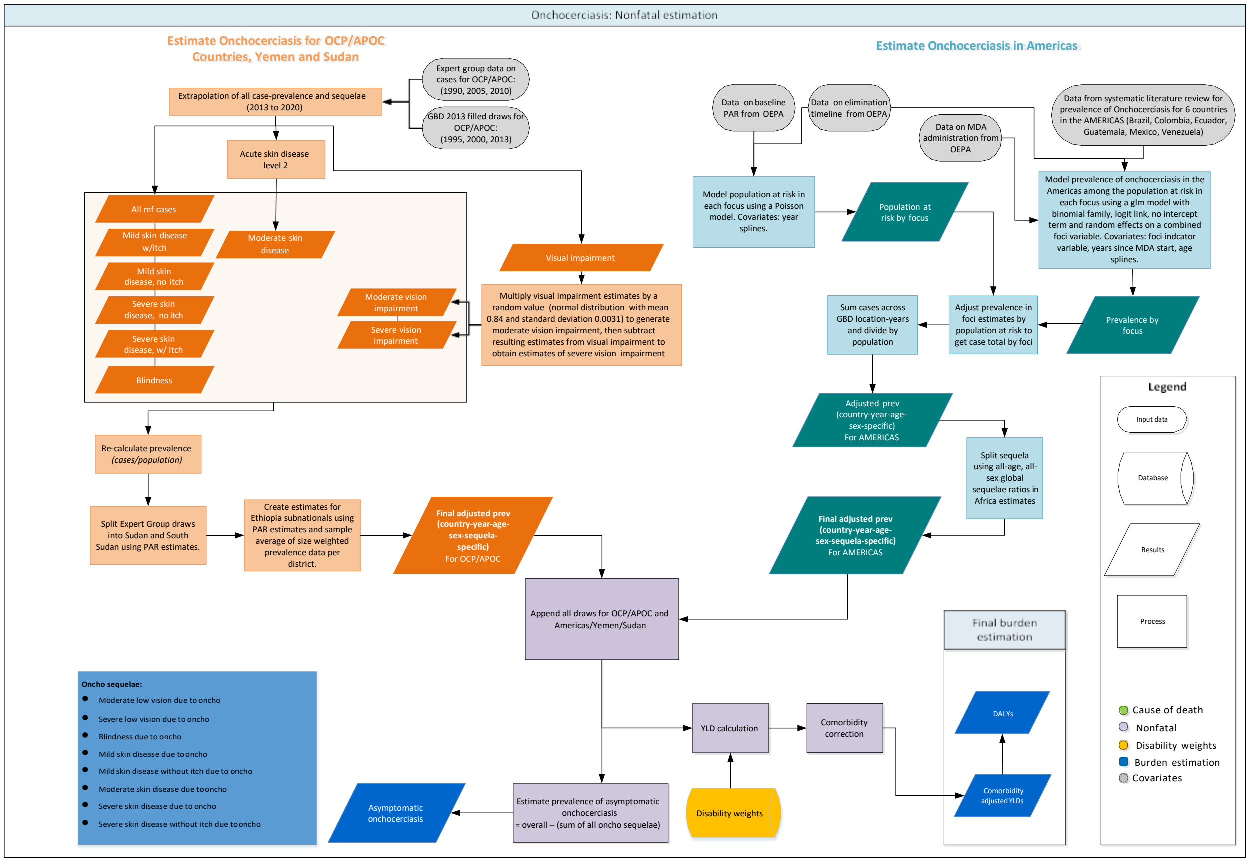

Input data and methodological summary

Case definition

Onchocerciasis, also known as river blindness, is a parasitic disease caused by *Onchocerca volvulus*. It is transmitted via the bite of one of several species of *Similium* blackflies that have historically bred in fast-moving freshwater rivers and tributaries throughout sub-Saharan Africa, Central America, and South America. Clinical manifestations includes pruritic and/or disfiguring skin disease, vision loss, and blindness. Diagnosis can be made by skin snip biopsy to identify larvae, surgical removal of nodules and exam for adult worms, slit lamp exam of anterior part of the eye where larvae or lesions caused by them are visible, and antibody tests (mostly useful to visitors to areas with parasites). The ICD-10 code for onchocerciasis is B73.

We used the following case definition for GBD 2021:

Quantity of interest	Reference or alternative	Definition
Onchocerciasis	Reference	Presence of *O. volvulus* microfilariae in a skin snip under microscopy.

Input data

Model inputs

Table 1: Source counts

Measure	Countries with data	New sources	Total sources
All measures	32	0	351
Prevalence	32	0	345
Population	6	0	6

Prevalence data prepared by the GBD 2010 expert group (EG) were used for modelling the non-fatal outcomes resulting from onchocerciasis in Africa. This included 1000 draws of infection and morbidity (visual impairment, blindness, and skin conditions) cases with confidence intervals categorised by country, age, and sex for years 1990, 1995, 2000, 2005, 2010, and 2013. Details about the materials and methods used by the EG to generate these draws can be found elsewhere [1-5]. These data represented all African countries included in the African Programme for Onchocerciasis Control (APOC) and the Onchocerciasis Control Programme (OCP) for which initial Rapid Epidemiological Mapping of Onchocerciasis (REMO) assessments demonstrated a need for community-directed treatment with ivermectin (CDTI) (defined as having a prevalence of skin nodules greater than 20%). Four countries – Rwanda, Mozambique, Kenya, and Gabon – were designated as hypo-endemic countries after initial REMO assessments and not included due to sparsity of cases and paucity of data. Estimates for Sudan from GBD 2010 were reassigned to South Sudan in GBD 2013 after its independence in 2011 since REMO assessments indicated that the vast majority of cases occurred in that area of the former Sudan. The tables below show the countries included in each programme and the number of corresponding GBD locations they represent.

	APOC countries	OCP countries
Countries included	Angola, Burundi, Cameroon, Central African Republic, Chad, Congo, Democratic Republic of Congo, Ethiopia, Equatorial Guinea, Liberia, Malawi, Nigeria, South Sudan, Tanzania, and Uganda	Benin, Burkina Faso, Côte d'Ivoire, Ghana, Guinea Bissau, Guinea, Mali, Niger, Senegal, Sierra Leone, and Togo
Hypo-endemic countries not included	Rwanda, Mozambique, Kenya, Gabon, Sudan	
GBD countries & subnational locations provided by EG	15	11
GBD world regions	3	1

Prevalence data for modelling non-fatal outcomes resulting from onchocerciasis in the Americas was extracted via a systematic literature review. Web of Science, Scopus, and PubMed were searched with the following search strings:

Database	Search string	Yield
PubMed	(oncho*[Title/Abstract] OR "river blindness"[Title/Abstract] OR "O. volvulus"[Title/Abstract] OR "robles disease"[Title/Abstract] OR "blinding filariasis"[Title/Abstract] OR "coast erysipelas"[Title/Abstract] OR "sowda" [Title/Abstract] OR "nodding	986

	syndrome”[Title/Abstract]) AND (“1980”[Date – Publication] : “2016”[Date – Publication]) AND (epidemiology[Title/Abstract] OR prevalence[Title/Abstract] OR incidence[Title/Abstract] OR surveillance[Title/Abstract] OR”MDA”[Title/Abstract] OR “Mass Drug Administration”[Title/Abstract] OR “Community-directed treatment with ivermectin”[Title/Abstract] OR “CDTI”[Title/Abstract] OR “mass treatment”[Title/Abstract] OR “multiple ivermectin treatments”[Title/Abstract] OR “monthly doses of ivermectin”[Title/Abstract] OR “large scale treatment”[Title/Abstract] OR REMO[Title/Abstract] OR “Rapid epidemiological mapping of onchocerciasis”[Title/Abstract] OR APOC[Title/Abstract] OR “African Programme for Onchocerciasis Control”[Title/Abstract] OR OCP[Title/Abstract] OR “Onchocerciasis Control Programme”[Title/Abstract]) NOT(Animals[MeSH] NOT Humans[MeSH])	
Web of Science	TS=(oncho* OR "river blindness" OR "O. volvulus" OR "robles disease" OR "blinding filariasis" OR "coast erysipelas" OR sowda OR “nodding syndrome”) AND TS=(epidemiology OR prevalence OR incidence OR surveillance OR MDA OR “Mass Drug Administration” OR “Community-directed treatment with ivermectin” OR CDTI OR “mass treatment” OR “multiple ivermectin treatments” OR “monthly doses of ivermectin” OR “large scale treatment” OR REMO OR “Rapid epidemiological mapping of onchocerciasis” OR APOC OR “African Programme for Onchocerciasis Control” OR OCP OR “Onchocerciasis Control Programme”) NOT TS=((Animals NOT Humans))	1144
SCOPUS	(TITLE-ABS-KEY(oncho* OR "river blindness" OR "O. volvulus" OR "robles disease" OR "blinding filariasis" OR "coast erysipelas")) AND TITLE-ABS-KEY(epidemiology OR prevalence OR incidence OR surveillance OR MDA OR "Mass Drug Administration" OR "Community-directed treatment with ivermectin" OR CDTI OR "mass treatment" OR "multiple ivermectin treatments" OR "monthly doses of ivermectin" OR "large scale treatment" OR REMO OR "Rapid epidemiological mapping of onchocerciasis" OR APOC OR "African Programme for Onchocerciasis Control" OR OCP OR "Onchocerciasis Control Programme") AND NOT KEY(Animals NOT Humans) AND PUBYEAR > 1979	2000

This yielded 4130 results in total, which was reduced to 2502 after removing duplicates. The title and abstracts were screened for inclusion or exclusion with the following criteria:

Exclusion criteria:

- Pre-1980
- Non-original source
- Non-representative population
    - Vulnerable populations (eg, slum-dwellers, prisoners, orphans, high-risk jobs, etc.)
    - Hospital-based samples (including saved stool samples)
    - Non-native peoples (eg, migrants, expats, nomads, etc.)
    - Immunosuppression/illness (eg, HIV, TB, CA, RA, asthma, malaria, handicap, etc.)
- Non-human population
- Does not meet case definition
- Case-control study

61 articles were identified for full text screening and extraction from the historically endemic American countries: Guatemala, Brazil, Ecuador, Venezuela, Mexico, and Colombia.

Severity splits/sequelae

The table below shows the list of common clinical manifestations of onchocerciasis and the sequelae to which they have been mapped along with the lay description and the associated disability weight (DW) of each sequela.

Clinical manifestation	Sequela name	Lay description	DW
Uveitis; punctate keratitis; optic neuritis; torpid Iritis; onchochorioretinitis	Moderate vision impairment	"has vision problems that make it difficult to recognize faces or objects across a room"	0.031 (0.019–0.049)
Sclerosing keratitis; optic neuropathy; optic atrophy; choroidoretinopathy; cataracts	Severe vision impairment	"has severe vision loss, which causes difficulty in daily activities, some emotional impact (for example worry), and some difficulty going outside the home without assistance"	0.184 (0.125–0.258)
Blindness	Blindness	"is completely blind, which causes great difficulty in some daily activities, worry and anxiety, and great difficulty going outside the home without assistance"	0.187 (0.124–0.260)
Acute papular onchodermatitis; onchocercomata (subcutaneous nodules)	Mild skin disease	"has a slight, visible physical deformity that is sometimes sore or itchy. Others notice the deformity, which causes some worry and discomfort"	0.027 (0.015–0.042)
Chronic papular onchodermatitis; lichenified onchodermatitis ("sowda"); lymphadenopathy	Mild skin disease without itch	"has a slight, visible physical deformity that others notice, which causes some worry and discomfort"	0.011 (0.005–0.021)
Skin atrophy; depigmentation ("leopard skin")	Moderate skin disease	"has a visible physical deformity that is sore and itchy. Other people stare and comment, which causes the person to worry. The person has trouble sleeping and concentrating"	0.188 (0.124–0.267)
Hanging groin; lymphoedema	Severe skin disease without itch	"has an obvious physical deformity that makes others uncomfortable, which causes the person to avoid social contact, feel worried, sleep poorly, and think about suicide"	0.405 (0.275–0.546)
	Asymptomatic onchocerciasis	NA	NA

Modelling strategy

The non-fatal modelling for onchocerciasis included six major steps. In the first step, GBD 2010 prevalence was exponentially extrapolated to obtain GBD 2021 estimates. Acute skin disease level 2 was mapped to the moderate skin disease sequela. Uncertainty was quantified and provided by the EG for all estimates except OCP cases of visual impairment and blindness. Uncertainty was added during the splitting process of visual impairment into moderate and severe vision impairment sequelae. The process includes multiplying visual impairment estimates by a random value from a normal distribution with mean 0.84 and standard deviation 0.0031 to generate moderate visual impairment (details of calculation described elsewhere [6]). The resulting estimates are subtracted from visual impairment to generate severe vision impairment. Prevalence of sequelae was calculated by dividing the cases by the population.

The second step in modelling morbidity due to onchocerciasis begins with the process of estimating the prevalence of onchocerciasis in GBD subnational locations. In Nigeria, we assume no subnational prevalence variation, and thus subnational prevalence estimates are set equal to national estimates.

Third, since EG draws were provided before the independence of South Sudan in 2011, Sudan estimates from the EG were partitioned between Sudan and South Sudan. Population at risk (PAR) estimates pre- and post-Abu Hamed foci elimination in 2015 in Sudan were used to proportionally split cases between the two countries [2]. REMO maps showing definite needs for community-directed treatment with ivermectin (CTDI) were digitised and overlaid with population per pixel rasters to produce estimates of PAR pre-Abu Hamed elimination. Post-Abu Hamed elimination in 2015, REMO maps were edited to remove the foci as a definite CDTI areas and estimates were reproduced.

In the fourth step, prevalence in the Ethiopia subnationals was estimated separately and appended to the Africa model. Subnational draws were split proportionally based on sample size weighted prevalence from prevalence data, using population at risk estimates derived from digitising a map of onchocerciasis endemic districts in 2015 from Meribo and colleagues to convert into case space [3]. A proportion of cases falling into each subnational was then used to split national case numbers provided by EG draws into each subnational.

In the fifth step, prevalence of onchocerciasis in Yemen was modelled separately and combined with the Africa model. Due to limited data, this was done utilising one datapoint from the Ministry of Health published in 1991 only accounting for population change [23]. Furthermore, the global age-sex trend was imposed to produce age-sex-specific estimates. The clinical manifestations of onchocerciasis in Yemen differ from those observed in other regions, almost exclusively consisting of onchodermatitis without ocular involvement [24]. All cases of onchocerciasis in Yemen were mapped to mild skin disease due to onchocerciasis without itch, though future efforts will seek to identify additional data to better inform severity distributions in Yemen.

In the sixth step, prevalence of onchocerciasis in the Americas was modelled separately and combined with the Africa and Yemen models. For the GBD estimation period, onchocerciasis is known to have occurred in six countries of Central and southern America: Mexico, Guatemala, Colombia, Ecuador, Brazil and Venezuela. The epidemiology of onchocerciasis is very different in these countries than in Africa because it has only occurred in relatively small, well-defined foci. These foci have been mapped and thoroughly monitored since the early 1990s with the formation of the Onchocerciasis Elimination Program

of the Americas (OEPA) and all of the prevalence surveys conducted are only representative of these areas. Additionally, certain foci are geographically continuous across national boundaries. Therefore, we modelled onchocerciasis in these countries at the focus level among the population at risk in each focus instead of at the national level.

Population at risk for each focus was modelled using data from OEPA on baseline population at risk [7] and data from OEPA and peer-reviewed studies on dates of elimination in each focus [7-22]. This was done with a Poisson model using year splines as a covariate, and 1000 draws of the population at risk were drawn from the predicted mean and standard error. The prevalence of disease among the population at risk was subsequently modelled using a generalised linear model with a binomial family, logit link, no intercept term, and random effects on a combined-foci variable created by grouping foci by geographical contiguity and nearness when data were sparse. Covariates included an indicator term on the foci, the number of years since MDA began, and splines on age. 1000 draws of prevalence were calculated from 1000 draws of beta values from the variance-covariance matrix and adjusted by the estimated population at risk in each focus-year to determine the number of cases. The cases were then summed by GBD geography and year and divided by national population to find the national prevalence. While the model predicted case values very close to zero in the countries where elimination has occurred, these were overwritten to zero values for all years after certified elimination. The ratio of global all-age, all-sex prevalence of each sequela to the all-cases prevalence from the Africa estimates was applied to all-cases prevalence from the Americas to calculate prevalence of each sequelae.

Lastly, to estimate the prevalence of asymptomatic onchocerciasis, the prevalence of morbidity (vision loss, blindness and skin conditions) was subtracted from the overall onchocerciasis prevalence. Moderate vision impairment, severe vision impairment, and blindness estimates were each multiplied by a factor of 8/33 (details of calculation described elsewhere [6]) before subtraction to account for cases that have concurring symptoms.

Changes from GBD 2019

We have made no substantive changes in the modelling strategy in GBD 2021. We did not apply any adjustments for the COVID-19 pandemic to onchocerciasis due to a lack of available data quantifying the impacts of the pandemic on NTD epidemiology.

References

1. Noma M, Zouré HGM, Tekle AH, Enyong PAI, Nwoke BEB, Remme JHF. The geographic distribution of onchocerciasis in the 20 participating countries of the African Programme for Onchocerciasis Control: (1) priority areas for ivermectin treatment. *Parasit Vectors* 2014; 7:325.
2. Zarroug IMA, Hashim K, ElMubark WA, *et al.* The First Confirmed Elimination of an Onchocerciasis Focus in Africa: Abu Hamed, Sudan. *Am J Trop Med Hyg* 2016; 95:1037–40.
3. Meribo K, Kebede B, Feleke SM, *et al.* Review of Ethiopian Onchocerciasis Elimination Programme. *Ethiop Med J* 2017; 55: 55–63.
4. Coffeng LE, Stolk WA, Hoerauf A, *et al.* Elimination of African onchocerciasis: modeling the impact of increasing the frequency of ivermectin mass treatment. *PLoS One* 2014; 9:e115886.
5. Coffeng LE, Stolk WA, Zouré HG, Veerman JL, Agblewonu KB, Murdoch ME, Noma M, Fobi G, Richardus JH, Bundy DA, Habbema D, de Vlas SJ, Amazigo UV. African Programme For

Onchocerciasis Control 1995-2015: model-estimated health impact and cost. PLoS Negl Trop Dis. 2013; 7(1): e2032.
6. Causes of blindness and vision impairment in 2020 and trends over 30 years, and prevalence of avoidable blindness in relation to VISION 2020: the Right to Sight: an analysis for the Global Burden of Disease Study. *Lancet Glob Health* 2020; 9: e144–60.
7. Murdoch ME, Asuzu MC, Hagan M, Makunde WH, Ngoumou P, Ogbuagu KF, Okello D, Ozoh G, Remme J. Onchocerciasis: the clinical and epidemiological burden of skin disease in Africa. Ann Trop Med Parasitol. 2002; 96(3): 283-296.
8. Brieger WR, Awedoba AK, Eneanya CI, Hagan M, Ogbuagu KF, Okello DO, Ososanya OO, Ovuga EB, Noma M, Kale OO, Burnham GM, Remme JH. The effects of ivermectin on onchocercal skin disease and severe itching: results of a multicentre trial. Trop Med Int Health. 1998; 3(12): 951-61.
9. México. http://www.oepa.net/Mexico.htm (accessed July 7, 2017).
10. Guatemala. http://www.oepa.net/guatemala.html (accessed July 7, 2017).
11. Venezuela. http://www.oepa.net/venezuela.html (accessed July 7, 2017).
12. Colombia. http://www.oepa.net/colombia.html (accessed July 7, 2017).
13. Ecuador. http://www.oepa.net/ecuador.html (accessed July 7, 2017).
14. Rodríguez-Pérez MA, Unnasch TR, Domínguez-Vázquez A, *et al.* Lack of Active Onchocerca volvulus Transmission in the Northern Chiapas Focus of Mexico. *The American Journal of Tropical Medicine and Hygiene* 2010; 83: 15–20.
15. Rodríguez-Pérez MA, Domínguez-Vázquez A, Unnasch TR, *et al.* Interruption of Transmission of Onchocerca volvulus in the Southern Chiapas Focus, México. *PLOS Neglected Tropical Diseases* 2013; 7: e2133.
16. Rodríguez-Pérez MA, Unnasch TR, Domínguez-Vázquez A, *et al.* Interruption of Transmission of Onchocerca volvulus in the Oaxaca Focus, Mexico. *The American Journal of Tropical Medicine and Hygiene* 2010; 83: 21–7.
17. Cruz-Ortiz N, Gonzalez RJ, Lindblade KA, *et al.* Elimination of Onchocerca volvulus Transmission in the Huehuetenango Focus of Guatemala. Journal of Parasitology Research. 2012. https://www.hindawi.com/journals/jpr/2012/638429/abs/ (accessed July 7, 2017).
18. Jr FR, Rizzo N, Espinoza CED, *et al.* One Hundred Years After Its Discovery in Guatemala by Rodolfo Robles, Onchocerca volvulus Transmission Has Been Eliminated from the Central Endemic Zone. *The American Journal of Tropical Medicine and Hygiene* 2015; 93: 1295–304.
19. Gonzalez RJ, Cruz-Ortiz N, Rizzo N, *et al.* Successful interruption of transmission of Onchocerca volvulus in the Escuintla-Guatemala focus, Guatemala. *PLoS Negl Trop Dis* 2009; 3: e404.
20. Lindblade KA, Arana B, Zea-Flores G, *et al.* Elimination of Onchocercia volvulus transmission in the Santa Rosa focus of Guatemala. *Am J Trop Med Hyg* 2007; 77: 334–41.
21. Convit J, Schuler H, Borges R, *et al.* Interruption of Onchocerca volvulus transmission in Northern Venezuela. *Parasites & Vectors* 2013; 6: 289.
22. WHO | WHO declares Ecuador free of onchocerciasis (river blindness). WHO. http://www.who.int/neglected_diseases/ecuador_free_from_onchocerciasis/en/ (accessed July 7, 2017).
23. Onchocerciasis and its control. Report of a WHO Expert Committee on Onchocerciasis Control. World Health Organ Tech Rep Ser. 1995; 852:1-104.
24. Al-Kubati A-S, Mackenzie CD, Boakye D, *et al.* Onchocerciasis in Yemen: moving forward towards an elimination program. *International Health* 2018; 10: i89–96.

Opioid use disorders

Flowchart

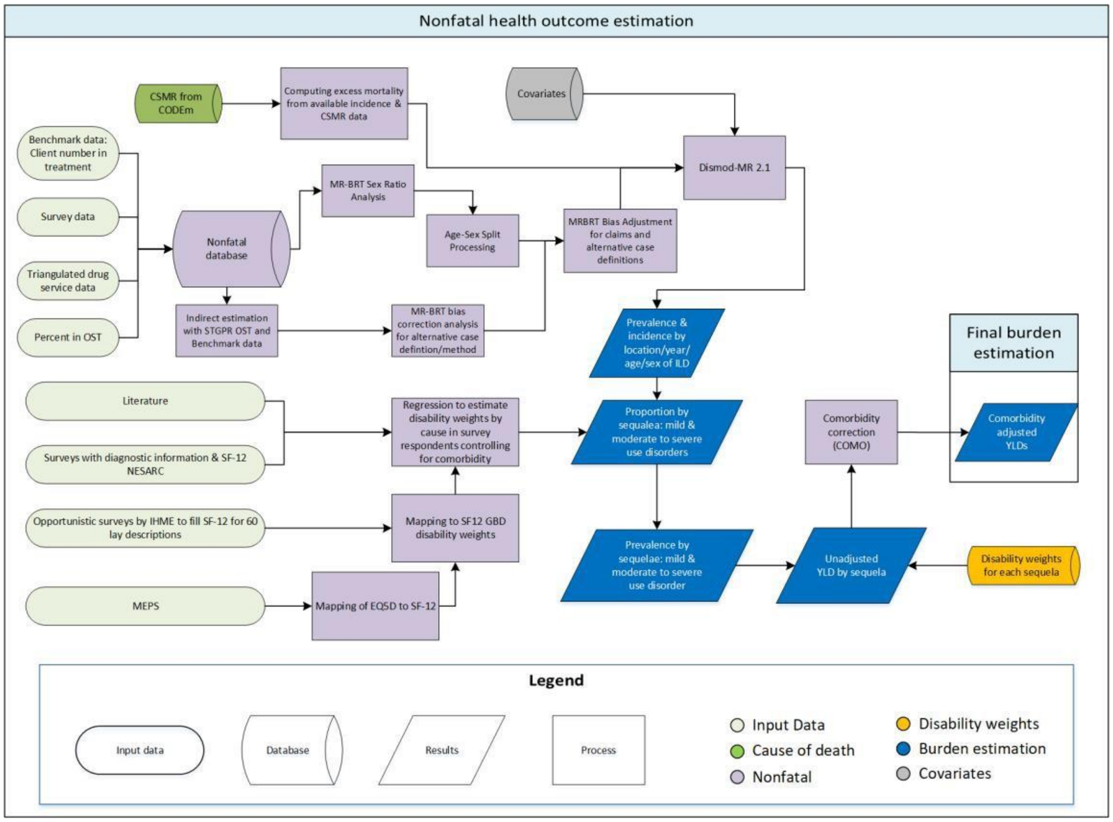

Input data and methodological summary for opioid use disorders

Case definition

We define opioid use disorders as "a maladaptive pattern of opioid abuse, leading to clinically significant impairment or distress that includes symptoms of dependence, such as withdrawal symptoms or progressive tolerance." Opioid dependence is a substance-related disorder involving a dysfunctional pattern of opioid use. Included in the GBD disease modelling were cases meeting the Diagnostic and Statistical Manual of Mental Disorders (DSM-IV-TR) or the International Classification of Diseases (ICD-10) diagnostic criteria for opioid dependence (DSM: 304.00; ICD: F11.2), excluding those cases due to a general medical condition.[1,2] To meet the DSM-IV TR criteria, at least three of the following symptoms must be experienced within the same 12-month period:

- Tolerance, characterised by either
  - a need for increased amounts of the substance to achieve intoxication; or
  - markedly diminished effect with continued use of the same amount of the substance;
- Withdrawal, characterised by either
  - Withdrawal symptoms characteristic to dependence; or
  - the same (or similar) substance is taken to avoid withdrawal symptoms;
- Substance taken in progressively larger amounts or for longer period;
- Persistent desire or unsuccessful efforts to reduce substance use;

- Disproportionate time dedicated to obtaining the substance;
- Other important activities are given up because of the substance use; and
- Substance use is continued despite knowledge of physical or psychological problems occurring as a result of the substance.

Input data

Systematic reviews were conducted in GBD 2010 and GBD 2017. The first review was repeated in GBD 2013 and GBD 2016 to update literature sources. The GBD 2017 review was targeted towards Maori and non-Maori populations in New Zealand, and cases in China, using primarily the China National Knowledge Infrastructure database. The inclusion criteria stipulated that 1) the publication year must be from 1980 onward; 2) "caseness" must be based on clinical threshold as established by the DSM or ICD; 3) sufficient information must be provided on study method and sample characteristics to assess the quality of the study; and (4) study samples must be representative of the general population (ie, inpatient or pharmacological treatment samples, case studies, veterans or refugee samples were excluded). No limitation was set on the language of publication. Methods used for this systematic review have been reported in greater detail elsewhere.[3,4]

In GBD 2021, we included data utilising sources of opioid users in substitution therapy and literature surveys of percentage of drug users in substitution therapy. Inclusion criteria for these sources were 1) currently in substitution treatment; 2) primary drug of use disorder was opioids, including synthetic opiates. We used these data to construct indirect estimates of prevalence of opioid use disorder. For details on these data and process, please see the IHME-indirect data creation.

Table 1: Data inputs for opioid use disorders morbidity modelling by parameter

Parameters	Countries with data	New sources	Total sources
Prevalence	46	459	553
Incidence	0	0	0
Remission	6	0	8
Other	16	0	41

Age and sex splitting

Reported estimates of prevalence were split by age and sex where possible. First, if studies reported prevalence for broad age groups by sex (eg, prevalence in 15- to 65-year-old males and females separately), and also by specific age groups for both sexes combined (e.g., prevalence in 15- to 30-year-olds, then in 31- to 65-year-olds, for males and females combined), age-specific estimates were split by sex using the reported sex ratio and bounds of uncertainty. Second, prevalence data for both sexes that could not be split using a within-study ratio were split using a sex ratio derived from a meta-analysis of existing sex-specific data using MR-BRT[5] (meta-regression—Bayesian, regularised, trimmed, described in

appendix 1, section 4.4.1 of the reference). The female to male ratio was 0.61 (0.51 to 0.73) for ages 20 and above and 1.12 (0.92 to 1.35) for ages below 20. Finally, after the application of bias adjustments, where studies reported estimates across age groups spanning 25 years or more, these were split into five-year age groups using the prevalence age pattern estimated by DisMod-MR 2.1[6] (disease model—Bayesian meta-regression tool) on all data prior to age-splitting. More details on DisMod-MR 2.1 can be found in appendix 1, section 4.5 of the reference article.

IHME-indirect data creation

Prevalence data include data created by IHME using an indirect multiplier method. This is a method of matching two datasets with partial information about opioid users to estimate the total population. This is already the primary type of data in previous iterations of opioid use disorder modelling.

The first source of data comes primarily from government records of the number of people with opioid dependence in substitution therapy. The second source of data comes from literature sources that describe the percentage of people with opioid dependence in treatment.

We run the data on percentage of dependents in treatment through our spatiotemporal Gaussian process regression tool[6] (ST-GPR, details in appendix 1, section 4.3.3 of the reference) model to get coverage estimates by every year, location, and sex. We assume treatment percentage doesn't differ by age due to data constraints.

We then calculate the following to get the total population of people with opioid dependence:

Opioid population = Number in treatment / ST-GPR estimated coverage; year, sex, location

This opioid population estimate is divided by the total population in each location-year-sex grouping to get an indirect estimate of opioid prevalence.

Data adjustment

The prevalence dataset included datapoints of both use and dependence estimated using "direct" or "indirect" survey methods. "Direct" methods of measuring opioid dependence predominantly involve surveys of the general population that ask if respondents use or are dependent on opioids. Surveys tend to underestimate the prevalence of the most harmful and stigmatised forms of illicit drug use in ways that probably vary between countries and cultures.[7] "Indirect" methods are considered superior, but they use different sources of data to indirectly estimate the total number of drug users (methods include "multiplier methods," back-projection and capture-recapture methods) that are often poorly documented. In GBD 2019, direct surveys of opioid dependence were adjusted by a factor derived from MR-BRT by the logit differences to indirect literature data. In GBD 2021, we modified this approach to use the IHME-indirect data instead of the indirect literature to create the adjustment factor instead. This round, we updated our data adjustment process by matching direct surveys to the IHME-indirect created data to increase the amount of information available for creating adjustment factors using MR-BRT. The beta and exponentiated value for this covariate are shown in the table below:

Table 2: MR-BRT crosswalk adjustment factors for opioid use disorder

Data input	Status	Gamma	Beta coefficient, logit*	Adjustment factor**
IHME-indirect created opioid dependence data	Ref	0.24	---	---
Opioid dependence – direct method	Alt		-1.07	0.25

**MR-BRT crosswalk adjustments can be interpreted as the factor the alternative case definition is adjusted by to reflect what it would have been had it been measured using the reference case definition. If the log/logit beta coefficient is negative, then the alternative is adjusted up to the reference. If the log/logit beta coefficient is positive, then the alternative is adjusted down to the reference.*

***The adjustment factor column is the exponentiated beta coefficient. For log beta coefficients, this is the relative rate between the two case definitions. For logit beta coefficients, this is the relative odds between the two case definitions.*

Modelling strategy

Prior settings in DisMod included assuming no incidence and excess mortality before age 15. This minimum age of onset was corroborated with expert feedback and existing literature on opioid dependence. We also assumed no incidence after age 64 as supported by data from various sources including the European Monitoring Centre for Drugs and Drug Addiction.[8] An upper limit of 0.2 was placed on remission consistent with limits in the dataset. These settings were retained for GBD 2021.

As in GBD 2019, age-standardised prevalence of intravenous drug use and log-transformed estimates of defined daily doses for statistical purposes (SDDD; consumption per day per million population) of prescribed opioid analgesics were included as country-level covariates. SDDD were modelled in GBD 2017 via spatiotemporal Gaussian process regression (ST-GPR) using data supplied by the International Narcotics Control Board (INCB). These 2017 estimates were carried forward into 2021. Subnational estimates for the USA were estimated by crosswalking national estimates with the state/national ratios of opioid prescriptions per 100 persons supplied by the Centers for Disease Control and Prevention.

We continued generated excess mortality rate data (EMR) using the MR-BRT approach by age and sex with a prior on Healthcare Access and Quality (HAQ) Index having a negative coefficient. Results from MR-BRT were then predicted for each location year, sex, and for ages 0, 10, 20 ….100. We included HAQ Index as a country-level covariate to inform EMR with a mean and standard deviation produced from MR-BRT. In previous rounds, priors on excess mortality rate (EMR) were estimated in DisMod by matching prevalence datapoints with their corresponding CSMR values within the same age, sex, year, location (by dividing CSMR by prevalence). However, for many causes, DisMod estimated a rather unrealistic pattern of EMR compared to an expected pattern of decreasing EMR with greater access to quality health care. Such unexpected patterns often signal inconsistencies between CSMR estimates and the measures of prevalence and/or incidence.

For opioid use disorder, the MR-BRT analysis for paired prevalence and CSMR data did not find any effect of HAQ Index under the condition of a negative prior. As such, across high and low HAQ Index locations predicted EMR was the same, following the EMR trend of the high-income and central Europe, eastern Europe, and central Asia super-regions, where the majority of data come from. It did lead to estimates of prevalence following those of cause of deaths estimates more closely.

However, estimates in Afghanistan and Iran, two countries in the otherwise low-prevalence north Africa and the Middle East super-region with some of the highest prevalence input data in the world, were constrained significantly. These two locations have low values of the intravenous drug use and prescription opioid covariates, resulting in country priors that were far lower than their prevalence data. Intravenous drug use was also included as a country-level covariate on EMR with bounds set between 0 and 2.

Table 3. Covariates. Summary of covariates used in the opioid use disorders DisMod-MR meta-regression model

Covariate	Parameter	Beta, log (95% uncertainty interval)	Exponentiated beta (95% uncertainty interval)
Intravenous drug use (age-standardised proportion)	Prevalence	0.26 (0.036 – 0.47)	1.29 (1.04 — 1.60)
Opioids per million population per day (10-year lag)	Prevalence	0.097 (0.084 — 0.11)	1.10 (1.09 — 1.12)
Intravenous drug use (age-standardised proportion)	Excess mortality rate	1.92 (1.81 — 2.00)	6.84 (6.12 — 7.36)

Note, a bound was set on the coefficient for opioids per million per day in an effort to make the model follow the high prevalence data in Iran and Afghanistan more closely.

Severity and disability

The basis of the GBD disability weight survey assessments are lay descriptions of health states highlighting major functional consequences and symptoms. The lay descriptions and disability weights for opioid dependence severity levels are shown below.

Table 4. Severity distribution, details on the severity levels for opioid use disorders in GBD 2021 and the associated disability weight (DW) with that severity

Severity level	Lay description	DW (95% CI)
Mild	Uses heroin (or methadone) daily and has difficulty controlling the habit. When not using, the person functions normally.	0.335 (0.221–0.473)
Moderate to severe	Uses heroin daily and has difficulty controlling the habit. When the effects wear off, the person feels severe nausea, agitation, vomiting, and fever. The person has a lot of difficulty in daily activities.	0.697 (0.510–0.843)

The proportion of people with opioid dependence within each of the severity levels was determined based on available data from US National Epidemiological Survey on Alcohol and Related Conditions (NESARC), conducted in two waves from 2001–2002 and 2004–2005,[9] and the Comorbidity and Trauma study conducted in 2005–2008.[10] NESARC is a direct household survey. As such, it is expected to underestimate moderate to severe cases of drug dependence. The estimated distribution of opioid dependent cases by severity were asymptomatic (16%, 13–19), mild (37%, 20–55), and moderate/severe (47%, 29–64).

References

1. American Psychiatric Association. Diagnostic and Statistical Manual of Mental Disorders (DSM-IV-TR). 4th, Text Revision ed Washington DC: American Psychiatric Association; 2000.
2. World Health Organization. The ICD-10 Classification of Mental and Behavioural Disorders. Clinical descriptions and diagnostic guidelines Geneva: World Health Organization; 1992.
3. Degenhardt L, Bucello C, Calabria B, Nelson P, Roberts A, Hall W, et al. What data are available on the extent of illicit drug use and dependence globally? Results of four systematic reviews. Drug and alcohol dependence. 2011.
4. Calabria B, Degenhardt L, Briegleb C, Vos T, Hall W, Lynskey M, et al. Systematic review of prospective studies investigating “remission” from amphetamine, cannabis, cocaine or opioid dependence. Addictive Behaviors. 2010.
5. Murray CJL, Aravkin AY, Zheng P, et al. Global burden of 87 risk factors in 204 countries and territories, 1990–2019: a systematic analysis for the Global Burden of Disease Study 2019. The Lancet 2020; 396: 1223–49. doi: https://doi.org/10.1016/S0140-6736(20)30752-2
6. Vos T, Lim SS, Abbafati C, et al. Global burden of 369 diseases and injuries in 204 countries and territories, 1990–2019: a systematic analysis for the Global Burden of Disease Study 2019. The Lancet 2020; 396: 1204–22. doi: https://doi.org/10.1016/S0140-6736(20)30925-9
7. Shand FL, Degenhardt L, Slade T, Nelson EC. Sex differences amongst dependent heroin users: Histories, clinical characteristics and predictors of other substance dependence. Addictive behaviors. 2011; 36(1): p. 27-36.

8. European Montioring Centre for Drugs and Drug Addiction. Lisbon, Portugal; 2014.
9. Grant BF, Dawson DA. National Institute on Alcohol Abuse and Alcoholism. Alcohol Health & Research World. 2006; 29(2): p. 74.
10. Shand FL, Slade T, Degenhardt L, Baillie A, Nelson EC. Opioid dependence latent structure: two classes with differing severity? Addiction. 2011; 106(3): p. 590-8.

Oral disorders

This document describes the non-fatal disease burden modelling process for GBD 2021 for each of edentulism, caries of deciduous teeth, caries of permanent teeth, chronic periodontal disease, and other oral disorders.

Input data

Data seeking and systematic literature reviews were completed for all oral disorders together, given the overlap in data types and data sources that inform the models. An initial literature review was done by the Expert Group for GBD 2010 in PubMed, Embase, Latin American and Caribbean Health Sciences (LILACS), and Scientific Electronic Library Online (SciELO), including published articles as well as the results of national and subnational reports. An updated systematic review was last completed on February 11, 2018, for GBD 2017 in PubMed and Embase. The search strings used are below:

PubMed: ((((Deciduous caries[Title/Abstract]) OR (milk caries[Title/Abstract]) OR (baby caries[Title/Abstract]) OR (caries[Title/Abstract]) OR (dental health[Title/Abstract]) OR (oral health[Title/Abstract])) OR ((Permanent caries[Title/Abstract]) OR (caries prevalence[Title/Abstract]) OR (dental health[Title/Abstract]) OR (oral health[Title/Abstract])) OR ((Periodontal disease[Title/Abstract]) OR (periodontitis[Title/Abstract]) OR (periodontal[Title/Abstract])) OR ((Edentulism[Title/Abstract]) OR (edentulous[Title/Abstract]) OR (endentulousness[Title/Abstract]) OR (severe tooth loss[Title/Abstract]) OR (total tooth loss[Title/Abstract]) OR (complete tooth loss[Title/Abstract]))) AND ((prevalence[Title/Abstract]) OR (incidence[Title/Abstract])) AND (2013/06/01[PDat] : 2016/12/31[PDat]))

Embase: 'deciduous caries':ab,ti OR 'milk caries':ab,ti OR 'baby caries':ab,ti OR caries:ab,ti OR 'permanent caries':ab,ti OR 'caries prevalence':ab,ti OR 'dental health':ab,ti OR 'oral health':ab,ti OR 'periodontal disease':ab,ti OR periodontitis:ab,ti OR periodontal:ab,ti OR edentulism:ab,ti OR edentulous:ab,ti OR endentulousness:ab,ti OR 'severe tooth loss':ab,ti OR 'total tooth loss':ab,ti OR 'complete tooth loss':ab,ti AND (prevalence:ab,ti OR incidence :ab,ti) AND [2008-2016]/py AND [humans]/lim AND [embase]/lim NOT [medline]/lim

For GBD 2019, we completed a targeted systematic review of LILACS and SciELO, focusing first on articles from the most recent period, from 2014 to 2018, which were subject to full text screening. The search used for LILACS and SciELO was the same:

LILACS/SciELO: "(deciduous caries OR milk caries OR baby caries OR caries OR dental health OR oral health OR permanent caries OR caries prevalence OR periodontal disease OR periodontitis OR periodontal OR edentulism OR edentulous OR edentulousness OR complete tooth loss OR tooth loss OR toothloss OR number of teeth OR dentate OR edentate) AND (prevalence OR incidence OR survey OR epidemiology)".

A total of 1696 citations were identified after deduplication, 147 were selected for full text review, and 77 new sources were extracted from the following countries: Argentina (1), Brazil (47), Chile (5), Colombia (5), Cuba (5), Ecuador (1), El Salvador (1), Honduras (1), Mexico (5), Peru (5), and Venezuela (1).

For GBD 2021, we reviewed the Global Health Data Exchange (https://ghdx.healthdata.org) for oral health surveys and national reports with oral epidemiology data. A total of 105 new sources were identified and extracted for inclusion in GBD 2021 models. We eliminated many datapoints to avoid repetition in the dataset, while striving to maintain as much data detail as possible. Redundancy tended to arise in three data descriptors: age, gender, and urbanicity. Our order of preference for maintaining detail was age, followed by gender, then urbanicity. Additionally, many of the studies presented dmft or DMFT scores, which represent lifetime prevalence and were often described as "caries experience". For the purposes of measuring the burden of disability from dental caries, we considered only data on current prevalence to be relevant, and thus converted lifetime prevalence data to current prevalence and incidence where possible. The complete dataset contents for each model are shown in tables for each cause in the corresponding sections below.

Table 1. Total number of sources and countries with data for oral disorders, by measure

Measure name	GBD 2021 sources	GBD 2021 new sources	# of countries	# of regions	# of super-regions
-	966	105	133	21	7
Prevalence	887	71	132	21	7
Incidence	197	54	68	19	7
Other	37	0	13	10	5

Edentulism

Flowchart

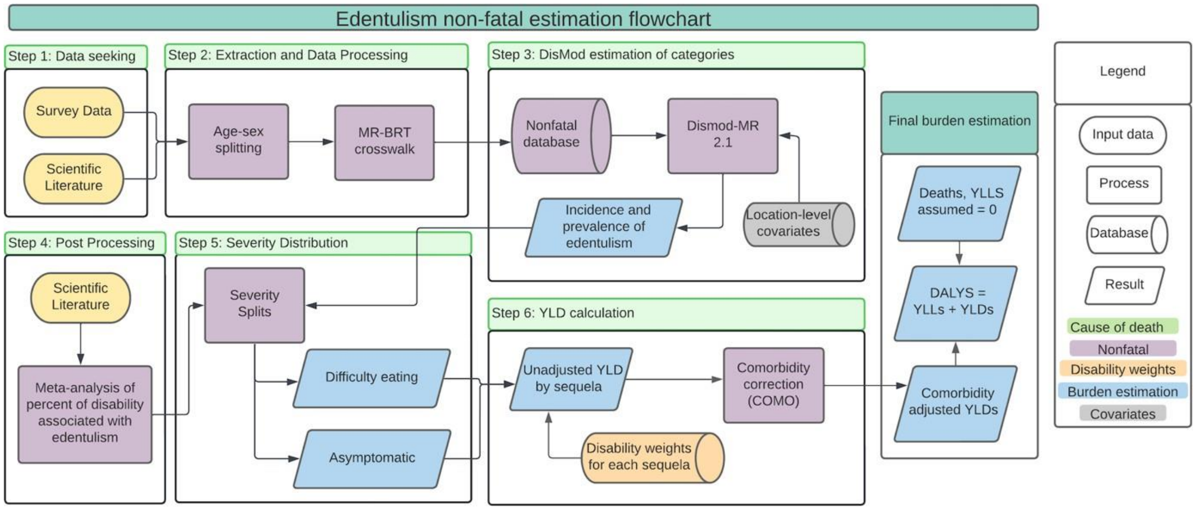

Case definition

The case definition of edentulism includes any individual with zero remaining permanent teeth; toothlessness of infancy is not included. The assessment of this disease includes quantification of the prevalence of the disease as well as estimation of the major sequelae: asymptomatic toothlessness and symptomatic toothlessness leading to "great difficulty in eating meat, fruits, and vegetables". A small body of evidence has begun to emerge that implicates edentulousness as predisposing individuals to increased risk for ischaemic cardiovascular events, including myocardial infarction and stroke. These data are sparse but have been included in models estimating the excess mortality of those with complete tooth loss. Given that the association is believed to be ecological rather than causal, however, edentulism has not been estimated as an underlying cause of death, and it is not included in the risk factor analysis for cardiovascular diseases.

Input data and data processing

Details of the systematic literature reviews appear earlier in this write-up. In addition to published studies, we also utilised self-report data on toothlessness from World Health Survey (WHS) for 47 countries as well as a number of national oral health surveys identified through the Global Health Data Exchange.

Table 1: Total number of sources and countries with data for edentulism, by measure

Measure name	GBD 2021 sources	GBD 2021 new sources	# of Countries	# of regions	# of super-regions
-	310	57	94	21	7
Prevalence	299	47	94	21	7
Incidence	14	10	6	5	3

Age-sex splitting

The first step of data processing was age splitting. For any datum that did not entirely fit within a GBD sex or age group, the observation was split to be multiple age-sex-specific datapoints based on the age and sex pattern predicted by previous DisMod-MR 2.1 models. It is our intention to update this age-sex splitting with each cycle of GBD.

Crosswalks in meta-regression—Bayesian, regularised, trimmed (MR-BRT)

Prior to GBD 2021, we crosswalked self-reported (ie, WHS) data on toothlessness to the reference definition of oral examination; but at present, we no longer found a statistically significant difference between self-report and oral examination, so no adjustment of self-report data was completed.

Figure 1: Funnel plot showing logit-transformed ratio of edentulism prevalence for alternate (self-report) versus reference (oral examination)

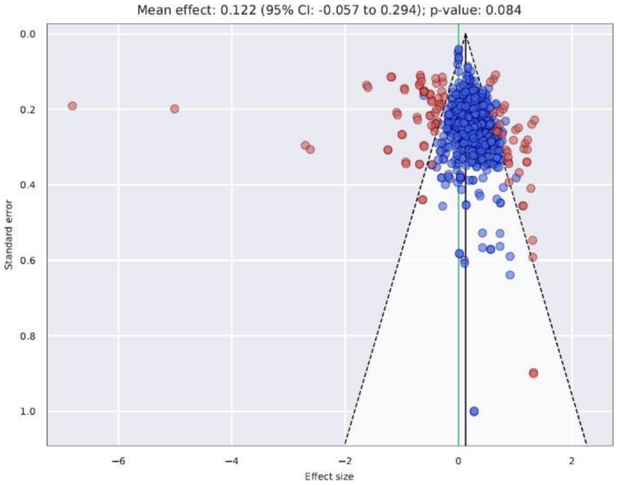

.

Modelling strategy

Estimates for the prevalence of edentulism were calculated for each location/year/sex/age using DisMod-MR 2.1. As would be expected for an irreversible condition, remission was fixed at zero for all ages. Mortality and relative risk were both fixed at zero before age 30, as any excess cardiovascular events resulting from severe tooth loss would not be expected at younger ages. We also assigned incidence and prevalence to be zero during childhood. Incidence was allowed to rise beginning at age 15, which was chosen based on the age at which the permanent dentition is expected to have fully formed in all individuals. The random effect limits for all locations were bounded at +/- 1.

As mentioned above, the criteria for diagnosis of edentulism are straightforward, and bias in the dataset was considered negligible. Thus, no study-level covariates were used in modelling the prevalence of edentulism. We included two location-level covariates in the model: 1) log-transformed lag-distributed income (LDI) with a minimum beta value of 0.02, and 2) log-transformed age-standardised summary exposure value (SEV) scalar of cardiovascular disease (CVD) in recognition of the common risk factors between CVD and tooth loss.

Table 4: Covariate, parameter, beta, and exponentiated beta values for edentulism

Covariate name	Measure	Beta (UI)	Exponentiated beta (UI)
LDI (I$ per capita)	Prevalence	–0.217 (–0.225 to –0.209)	0.805 (0.798–0.811)
Age- and sex-specific SEV for smoking	Prevalence	0.048 (0.002 to 0.123)	1.05 (1.002–1.131)
Age- and sex-specific SEV for high fasting plasma glucose	Prevalence	0.263 (0.068 to 0.466)	1.301 (1.07–1.594)

Models were vetted based on the plausibility of the results, the extent to which estimates fit the data, and the plausibility of the range of estimates across location hierarchies.

Severity distributions and disability weights

The disability weight used for symptomatic toothlessness leading to "great difficulty in eating meats, fruits, and vegetables" is 0.067 (0.045–0.095) as determined by the GBD disability survey. We considered all those with severe tooth loss and no access to dentures to experience this disability. However, the proportion of those with edentulism and severe tooth loss who have dentures has not been studied extensively.

In order to estimate the proportion of edentulous individuals with no access to dentures, we completed a supplemental literature review of dentures prevalence for GBD 2010. Only six systematic surveys of dentures prevalence were identified, all in high- and middle-income countries. All were completed since 2000. After extracting the data from the studies, we performed linear regressions of denture presence and denture absence against health system access (HSA), a standardised covariate of treatment availability used in many disease estimation models. From the results of the regression, the prevalence of no dentures was calculated for all super-regions. We then completed a population-weighted average of all countries in the super-region based on 2003 populations, the average year of the dentures studies. Uncertainties for the prevalence of dentures were calculated by finding the standard deviation and standard error of the calculated prevalence values.

The estimated prevalence of dentures in each location was used to calculate the proportion of individuals with asymptomatic edentulism and severe tooth loss (ie, those who have access to dentures) and difficulty eating due to edentulism and severe tooth loss (ie, those without access to dentures). This latter sequela was included as a cause of years lost due to disability (YLDs).

Caries of permanent teeth and caries of deciduous teeth

Separate estimates of caries of deciduous teeth and caries of permanent teeth

The natural histories of deciduous and permanent caries share many similarities, but they also share some important differences. Age patterns of decay in permanent and deciduous dentition are distinct, and duration of a carious lesion in deciduous teeth also tends to be shorter than an untreated episode of permanent caries. Sugar consumption and feeding with formula are both associated with development of deciduous caries, while sugar consumption is associated with the development of caries of permanent teeth. Finally, it is unclear whether the gender patterns and regional differences are the same for both deciduous and permanent caries. For all of these reasons, we elected to model deciduous caries and permanent caries as separate entities and then add the estimates together for an overall estimation of the global burden of dental caries. This is the modelling approach which has been taken in each iteration since GBD 2010.

Flowchart

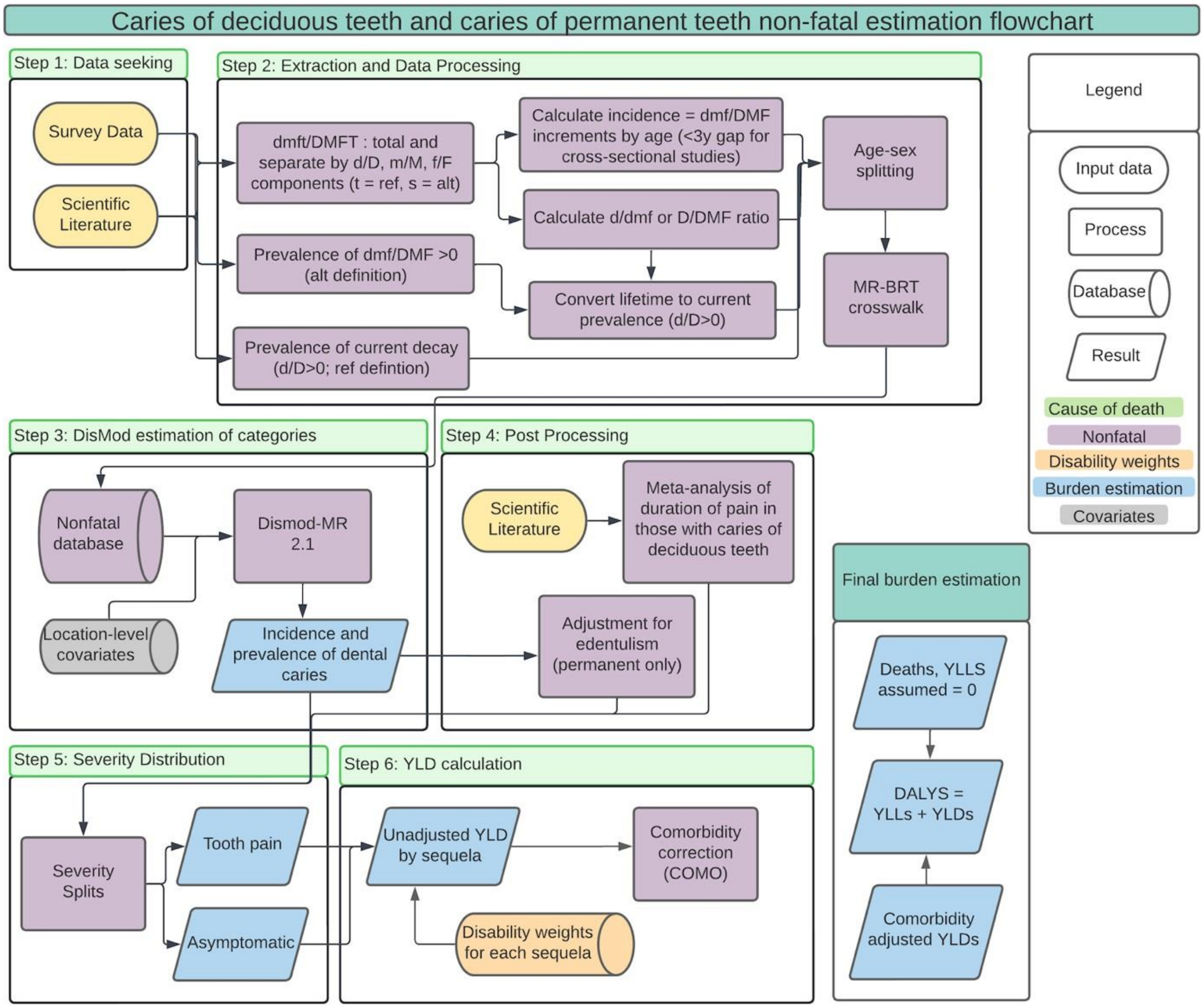

Case definition

The case definition for dental caries is "teeth with unmistakable coronal cavity at dentin level, root cavity in cementum that feel soft or leathery to probing, temporary or permanent restorations, or missing teeth extracted due to a caries lesion". Excluded definitions include crowns with isolated cosmetic defects, stained enamel pits, or fissures without visible cavitation or softening, fluorosis, and abrasion lesions. This definition corresponds to an ICD-9 code of 521.0 and an ICD-10 code of K02.3–K02.9. Most caries are subclinical in the sense that they do not cause symptoms a majority of the time. Once a carious lesion develops, it will occasionally recede without intervention, but often it worsens with time and eventually requires either filling or extraction.

Public health dentists commonly measure dental caries using the dmft/DMFT index, which is an incremental measure of the proportion of unhealthy teeth and is also a measure of an individual's lifetime prevalence of caries. Lowercase letters (dmft) are used for deciduous dentition and uppercase letters (DMFT) for permanent dentition. D is for decayed, M for missing, F for filled, and T for teeth. The maximum dmft score is 20 and the maximum DMFT score is 32. Furthermore, some dentists prefer to measure dental caries in terms of tooth surfaces, rather than number of teeth, and report their results

using an analogous dmfs/DMFS index. The maximum dmfs score is 88, and the maximum DMFS score is 128 or 148 depending on whether the third molars are counted.

The DMFT index is easy to measure, and inter-rater reliability is high. However, the primary shortcoming of the DMFT is that it does not discriminate well between current and past caries. Strategies we employed to maximally utilise dmf/DMF data for estimating the prevalence of burden due to permanent caries are described below.

Input data and data processing

The approach for systematic literature review is described above. The reference definition for this model was presence of one or more teeth with current decay (for prevalence), whereas each additional carious tooth was counted as a separate incident event.

Table 1: Total number of sources and countries with data for caries of deciduous teeth, by measure

Measure name	GBD 2021 sources	GBD 2021 new sources	# of countries	# of regions	# of super-regions
-	411	28	89	20	7
Prevalence	371	23	88	20	7
Incidence	104	21	41	16	7
Other	22	0	13	10	5

Table 2: Total number of sources and countries with data for caries of permanent teeth, by measure

Measure name	GBD 2021 sources	GBD 2021 new sources	# of countries	# of regions	# of super-regions
-	283	56	91	20	7
Prevalence	246	26	88	20	7
Incidence	115	47	57	18	7

Converting lifetime to current prevalence

Many of the studies presented dmft or DMFT scores, which represent lifetime prevalence and were often described as "caries experience". For the purposes of measuring the burden of disability from dental caries, we converted lifetime prevalence data to current prevalence for individuals aged 20 years and under. We did this by multiplying the observed lifetime prevalence by the ratio of d/D to dmf/DMF. When d/dmf or D/DMF information was available from the same study, this ratio was applied. When not available from the same study, the pooled ratio from the closest matching GBD geography was used for the multiplication (country, region, super-region, global).

Calculation of incidence from dmft/DMFT increment

Whereas in the deciduous dentition, a vast majority of the dmf index is accounted for by caries, tooth loss is a major contributor to the DMF index for the permanent dentition. Caries of permanent teeth may not necessarily be the primary driver of this tooth loss, as other factors such as periodontal disease

and trauma may contribute significantly. Thus, we performed the conversions of incremental dmf/DMF scores to incidence values for permanent caries only in individuals aged 20 years or less and for all ages in the case of deciduous caries. For longitudinal studies, the difference between the dmf/DMF score in the initial versus subsequent examination was taken to be equivalent to the number of incident caries over that time period. This assumes a negligible proportion of dmf/DMF increment is due to trauma in children. For cross-sectional studies examining children of different ages, we only calculated incidence when the gap in age was ≤3 years given the propensity for strong cohort effects in caries epidemiology.

Age and sex splitting

For any datum that did not entirely fit within a GBD sex or age group, the observation was split to be multiple age-sex-specific datapoints based on the age and sex pattern predicted by previous DisMod-MR 2.1 models. It is our intention to update this with each cycle of GBD.

Crosswalks in MR-BRT

We then crosswalked alternative to reference definitions. To make data comparable, we began by evaluating the number of observations of each alternate definition that matched with a corresponding observation from the reference definition. Owing to the significant heterogeneity in data on caries incidence and prevalence, we limited the comparisons to only "within"-study matches, where a match was defined as both methods of ascertainment being performed in the identical study population. The ratio of alternative to reference was calculated and logit-transformed. Standard error of the ratio was calculated using the delta method. Sex was included as a fixed effect and, for prevalence only, midpoint of age as a spline. The adjustment factors and spline plots for the crosswalks are shown below.

The funnel plot is for demonstration only; the final crosswalk was derived from the MR-BRT model represented by the spline plot in Figure 1. For deciduous caries, we also adjusted data that were DMFS-derived (ie, calculated from d/dmfs as opposed to d/dmft), even though data are comparatively sparse, because there is strong suggestion of age-specific relationship in these two measures. This sub-analysis would be strengthened by additional data. We elected to adjust rather than drop the alternate data because the age groups involved are comparatively data-sparse. We intend to focus on identifying additional data for early childhood caries for the next GBD systematic data extraction effort.

Figure 1: Funnel plot (left) and spline plot by age (right) showing logit-transformed ratio of deciduous caries prevalence for alternate (converted from d/dmf) versus reference (d>0)

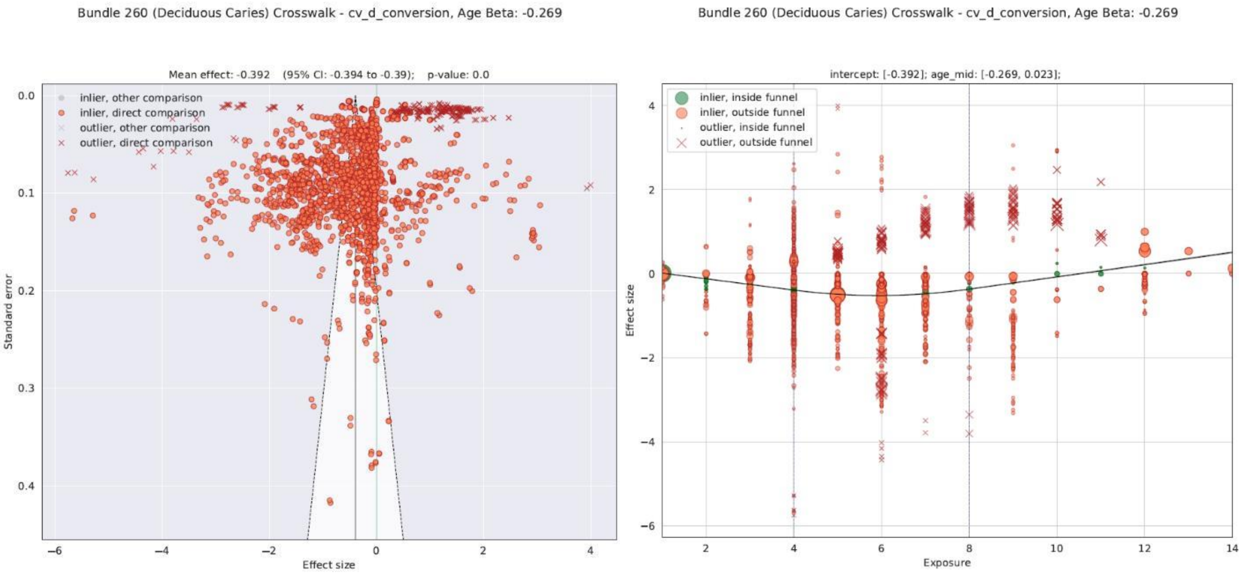

Figure 2: Funnel plot (left) and spline plot by age (right) showing logit-transformed ratio of deciduous caries incidence for alternate (dmfs measurement) versus reference (dmft measurement)

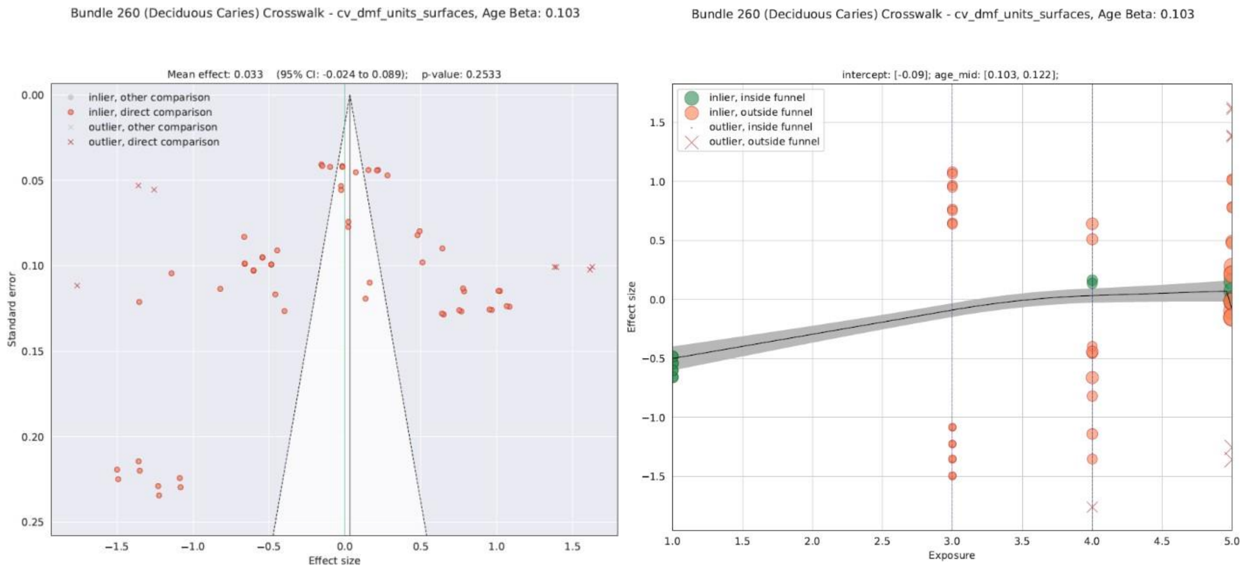

The same crosswalks were evaluated for data on caries of permanent teeth. The results of those crosswalks are shown in Figures 3 and 4, respectively.

Figure 3: Funnel plot (left) and spline plot by age (right) showing logit-transformed ratio of caries of permanent teeth prevalence for alternate (converted from D/DMF) versus reference (D>0)

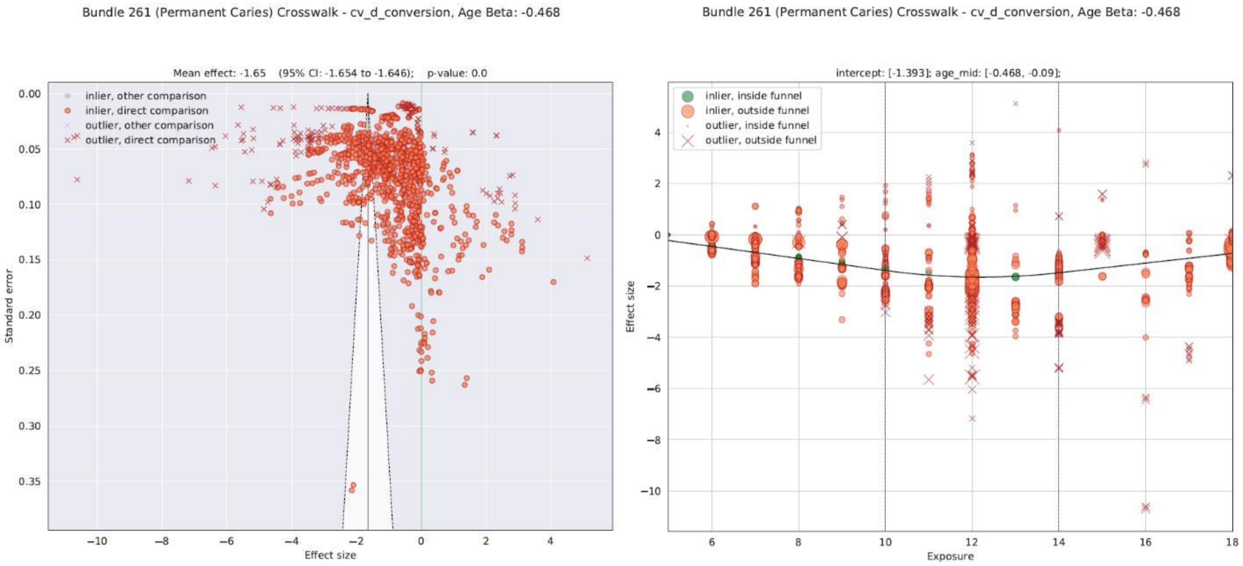

Figure 4: Funnel plot (left) and spline plot by age (right) showing logit-transformed ratio of caries of permanent teeth prevalence for alternate (DMFS measurement) versus reference (DMFT measurement)

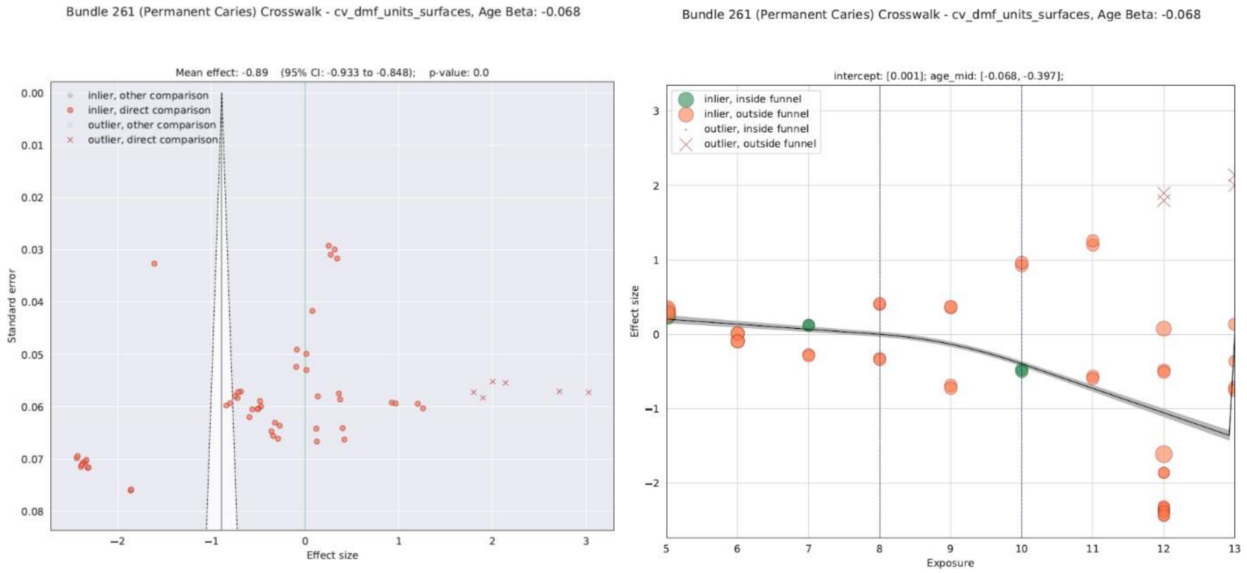

For prevalence data on caries of permanent teeth, there was a significant and age-dependent difference in prevalence data derived from measurement of surfaces as compared to teeth, so DMFS-derived prevalence data were adjusted to the reference of teeth as shown in Figure 4. As described above, the D/DMF conversion was only completed for data from children under 13 years of age because these were the only age groups from which we felt confident in deriving accurate measures of relationships between D/DMFS and D/DMFT. There were insufficient data to inform an assessment of whether or not there is a difference between cohort-based caries incidence and incidence derived from DMF increment.

Modelling strategy

DisMod model development

Serious health consequences of caries were also assumed to be uncommon and death very rare. We therefore assigned excess mortality to be zero from age 0 to 100. For both types of caries, most of the model settings were similar. The primary difference was in value priors. We assumed zero incident caries in infants under 1 year old and similarly zero incident deciduous caries from age 11 onward. For permanent caries, we assumed zero incident cases in children under 5. Location-level covariates were assigned separately on prevalence and incidence. Sugar availability in food from the GBD diet analysis was used as a covariate on incidence with a positive beta, while prevalence was assigned log-transformed LDI with a negative beta to reflect the association with access to dental care.

Table 3: Covariate, parameter, beta, and exponentiated beta values for dental caries of deciduous teeth

Covariate name	Measure	Beta (UI)	Exponentiated beta (UI)
LDI (I$ per capita)	Prevalence	–0.116 (–0.134 to –0.095)	0.89 (0.875–0.909)
Age- and sex-specific SEV for high sweetened beverages	Incidence	0.37 (0.022 to 0.878)	1.448 (1.023–2.407)

Table 4: Covariate, parameter, beta, and exponentiated beta values for dental caries of permanent teeth

Covariate name	Measure	Beta (UI)	Exponentiated beta (UI)
LDI (I$ per capita)	Prevalence	–0.171 (–0.216 to –0.131)	0.843 (0.806–0.877)
Age- and sex-specific SEV for high sweetened beverages	Incidence	0.3 (0.024 to 0.697)	1.35 (1.024–2.008)

Although studies were screened carefully during data extraction to ensure that they specified whether they were measuring permanent or deciduous caries, some datapoints were marked as outliers during modelling due to their high prevalence values in young ages, as it was deemed likely that some of these studies were reporting deciduous in addition to permanent caries. As with deciduous caries, models for permanent caries were vetted based on the plausibility of the results, the extent to which estimates fit the data, and the plausibility of the range of estimates across location hierarchies.

Correction for edentulism

One systematic source of bias in the literature was the exclusion of edentate individuals from the study populations, which leads to systematic overestimation of caries prevalence when modelled over the entire population. To account for this bias, we used our GBD estimates of edentulism prevalence to adjust YLD estimates for caries of permanent teeth. Final DisMod-MR 2.1 estimates of edentulism prevalence were paired with the corresponding results for caries of permanent teeth by age group, sex, location, and year to adjust for the proportion of the population that was excluded from the denominator of permanent caries models. No adjustment was made to the estimates of caries of deciduous teeth.

Severity distributions and disability weights

As described above, the GBD definition of disability associated with symptomatic dental caries is “this person has a toothache, which causes some difficulty eating”. The disability weight associated with this condition is 0.01 (0.005–0.019), as derived from the GBD disability weights study.

Not all those with dental caries experience this disability all the time. We considered only those with active dentinal decay to experience symptomatic tooth pain. Those with deciduous caries who had undergone exfoliation or had their cavities filled were considered to have no disability. Likewise, those with permanent caries who had received fillings, had their cavities extracted, or lost a carious tooth altogether were considered to have no disability. Thus, two additional pieces of information are required to complete the calculation of YLDs: proportion with symptoms and duration of disability.

To determine which segment of the population has ongoing tooth pain and the proportion of time spent with tooth pain, we considered several different options. First, we examined the data on dental caries symptoms and disability from the Medical Expenditure Panel Survey (MEPS) conducted by the USA Department of Health and Human Services in 2000–2009. MEPS data were widely used in GBD 2010 analyses. Respondents to the survey are asked about all medical conditions. Conditions for which provider care was sought are reported by the respondents at every round, and respondents also report problems for which they did not see a provider if the symptoms were "bothering" them. Conditions can be added to the condition roster if 1) they are reported as a reason for a medical event, 2) the condition was reported as the reason for one or more disability days, or 3) the condition was "bothering" the person during the reference period. Conditions are then recorded as verbatim text and coded to ICD-9CM 3rd digit codes by professional medical coders. These ICD-9 codes were mapped to GBD causes, including dental caries. From the MEPS, symptomatic caries in the previous year were reported by 48.4% (44.3–52.9) of the respondents. This number is in agreement with our DisMod-MR 2.1 estimates of 1–2 years' duration in high-income North America for permanent caries if we consider people to only have symptoms at the end of a course of caries. The two primary shortcomings of using this approach are 1) it does not provide enough detail to differentiate between the experiences of those with deciduous versus permanent caries, and 2) it indicates the proportion of those with caries who were symptomatic during the previous year, but it does not provide information on the amount of time during that year spent with symptoms (ie, one day versus 12 months). The approach described below addresses both issues.

To determine duration, we adapted the method employed by the Australian Burden of Disease (AusBoD) Study in 1996. For total duration, we used the posterior estimates of duration from final DisMod-MR 2.1 models. For those with symptoms, we split this total duration into two distinct phases of caries disability. The "initial" phase is characterised by *periodic* pain that we assigned to occur an average of one hour per day. The "terminal" phase is a period of *constant* symptoms at the end of an episode. The length of the terminal phase was determined by literature review as described by the AusBoD group. For deciduous caries, we used a study by Mason and colleagues of children in the UK presenting to a casualty ward with tooth pain.[1] The length of time each child had been experiencing tooth pain was recorded. Based on the distribution of time courses, a log-normal distribution was plotted that approximated the average duration of *constant* symptoms at 27.6 days leading up to seeking care. For permanent caries, a similar study of the tooth pain experience of adults in New Zealand who presented to hospital dental departments and an emergency clinic[2] resulted in an estimated 55.2 days spent in the terminal phase of caries. For those with severe disease, the length of time spent in the terminal phase was subtracted from the total duration to determine the amount of time spent in the initial phase. For those with mild disease, we considered the entire duration to be spent in the initial phase. These calculations were last completed as part of the GBD 2013 analysis.

To determine proportion with symptoms, we completed a supplemental literature review of tooth pain and caries. We identified a total of 21 studies with data about the prevalence of pain. The studies were

grouped according to the type of dentition studied (deciduous or permanent) and the location of the study group (high-income or low- and middle-income countries). We extracted data on the proportion in each group who described symptoms of pain related to their caries as well as a subset who described their symptoms as being severe. The proportions in each group were weighted according to sample size to give estimates of the relative sizes of three groups: asymptomatic, mild, and severe. The results of this meta-analysis are illustrated in the table below.

We considered asymptomatic individuals to experience no disability. Those with mild disease spent the entire duration in the initial phase of disease (one hour of pain per day). Those with severe disease spent a majority of the duration in the initial phase followed by a period of time in the terminal phase (constant pain). YLDs were calculated by multiplying the prevalence, duration, proportion, and disability weight for each age, country, sex, and year.

Table 7: Duration and distribution of severity for tooth pain due to caries of deciduous and permanent teeth

	# of studies	% symptomatic of total	% severe among symptomatic	% mild of total	% severe of total	% asymptomatic of total
Deciduous caries						
Data-rich	5	0.35	0.257	0.26	0.09	0.65
All others	4	0.555	0.438	0.312	0.243	0.445
Permanent caries						
Data-rich	6	0.602	0.315	0.412	0.189	0.398
All others	6	0.954	0.548	0.432	0.521	0.046
Duration of phases						
Initial phase				1 hour per day		
Terminal phase (deciduous caries)				27.6 days		
Terminal phase (permanent caries)				55.2 days		

Chronic periodontal disease

Flowchart

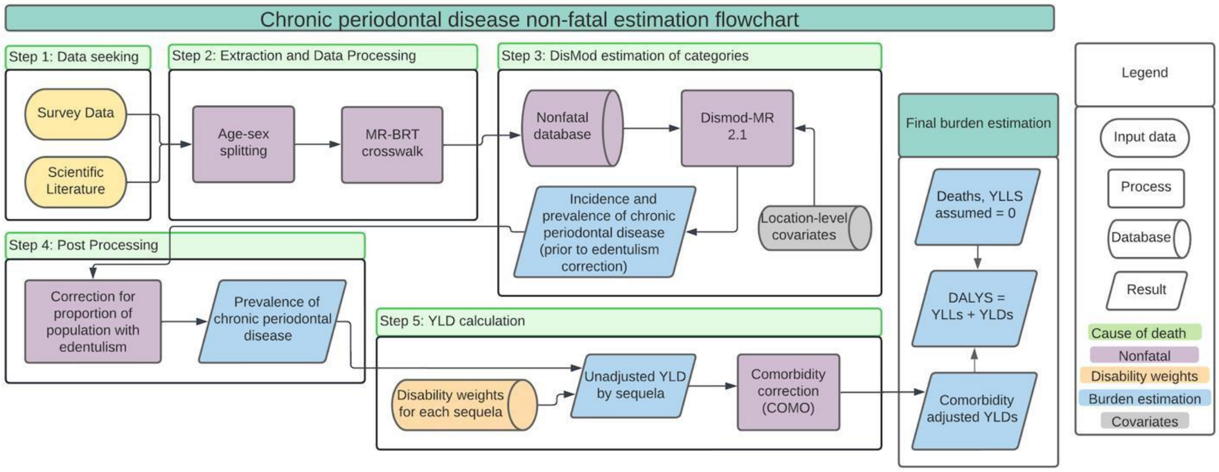

Case definition

Chronic periodontal disease is caused by chronic bacterial infection around the teeth. Symptoms of gingivitis, the mildest form of the disease, include swelling, redness, and propensity of the gums to bleed when perturbed. If the infection is not treated appropriately, it will eventually spread below the gum line, leading to a chronic inflammatory state of the periodontal tissues. Over time, there will be loss of gingival tissue and alveolar bone destruction. Teeth will become loose and may need to be extracted.

The GBD definition of disability associated with symptomatic severe periodontal disease is "bad breath, a bad taste in the mouth, and gums that bleed a little from time to time, but which does not interfere with daily activities". The ICD-10 codes for periodontal disease are K05.0–K05.6, and the ICD-9 codes are 523.0–523.9.

Defining periodontal disease in a meaningful, reproducible manner has been an ongoing challenge for public health dentists. Attachment loss (AL) and pocket depth (PD) have emerged as the most common metrics of periodontal health measurement. AL is measured as the difference between the distance from the gingival margin to the bottom of the pocket and the distance from the cemento-enamel junction to the bottom of the pocket.

The Community Periodontal Index (CPI) is a classification system that was developed by WHO as a standardised method of periodontal health measurement. CPI classification is based on the examination of all teeth present in the mouth for absence or presence of gingival bleeding and absence or presence of periodontal pockets. A standard-sized probe is used, with depth markings from 0.5 to 11.5 mm. The probe is inserted into the sulcus between a tooth and the gingiva until it meets resistance. The surrounding area is then explored with the probe to determine the maximum depth of the pocket. Multiple areas around each tooth are probed. Pocket scores range from 0 to 2 in order of increasing severity. When the CPI method was employed, we considered those with Class 2 only (pocket of 6 mm or more). Additionally, loss of attachment may be collected for specific index teeth by dividing the mouth in sextants. The two molars in each posterior sextant are paired for recording and, if one is missing, there is no replacement. If no index tooth is present in a sextant qualifying for examination, all the teeth that are present in that sextant are examined and the highest score is recorded as the score for the sextant. We excluded studies in which the study population was reported as the number of sextants rather than the number of individuals surveyed. CPI is a modification of Community Periodontal Index of Treatment Needs (CPITN) that does not include the assessment of periodontal treatment needs. Also, Class 2 of CPI is equivalent Class 4 of CPITN.

In 2007, a new CDC proposal for gold-standard diagnosis of severe, chronic periodontitis was published.[1] This standard specified that a stricter definition of the condition should be implemented. This more exclusive definition of chronic periodontal disease includes $\geq$2 interproximal sites with AL $\geq$6 mm ***AND*** $\geq$1 interproximal site with PD $\geq$5 mm. This definition has not been adopted by GBD.

A small body of evidence has begun to emerge that implicates chronic periodontal disease as predisposing individuals to increased risk for ischaemic cardiovascular events including myocardial infarction and stroke. These data are sparse but have been included in models estimating the excess mortality of those with chronic periodontal disease. Given that the association is believed to be ecological rather than causal, however, periodontal disease has not been estimated as an underlying cause of death and it is not included in the risk factor analysis for cardiovascular diseases.

Input data

Details of the systematic review are provided above. We implemented a hierarchical preference for case definitions. We included the following definitions of severe periodontal disease commonly found in the literature:

1. CPITN – Class 4 only
2. CPI – Class 3 only
3. Clinical AL >6 mm
4. Clinical AL >5 mm
5. Clinical AL >4 mm
6. Gingival PD >5 mm

If more than one type of data was included in a study, our first preference was for CPITN = 4, followed by AL >6 mm, and PD >5. All were considered equivalently as reference definitions with no additional crosswalking performed. For those sources that did not provide data on any of the components of CPITN Class 4, but did provide data on CPITN Class 3, AL >5 mm, or AL >4 mm, we utilised these data after crosswalking in MR-BRT as described below.

Table 1: Total number of sources and countries with data for chronic periodontal disease, by measure

Measure name	GBD 2021 sources	GBD 2021 new sources	# of countries	# of regions	# of super-regions
-	119	4	54	18	7
Prevalence	119	4	54	18	7

Age-sex splitting

For any datum that did not entirely fit within a GBD sex or age group, the observation was split to be multiple age-sex-specific datapoints based on the age and sex pattern predicted by previous DisMod-MR 2.1 models. It is our intention to update with each cycle of GBD.

Crosswalks in MR-BRT

We then crosswalked alternative to reference definitions. To make data comparable, we began by evaluating the number of observations of each alternate definition that matched with a corresponding observation from the reference definition. All alternative definitions were mutually exclusive with one another, so three separate crosswalks were performed using only within-study matches, defined as both methods of ascertainment being performed in the identical study population. The ratio of alternative to reference was calculated and logit-transformed. Standard error of the ratio was calculated using the delta method. Sex was included as a fixed effect and, for prevalence only, midpoint of age as a spline. The total number of matches, the adjustment factors, and the spline plots for periodontal disease crosswalks are shown below.

Figure 1: Funnel plot (left) and spline plot by age (right) showing logit-transformed ratio of chronic periodontal diseases prevalence as measured with alternate (top = CPITN 3; middle = attachment loss >4 mm; bottom = attachment loss >5 mm) versus reference (CPITN 4, AL =>6 mm)

Bundle 262 (Periodontal Disease) Crosswalk. cv_<.piclass3, AQt'! Beta: 0.65

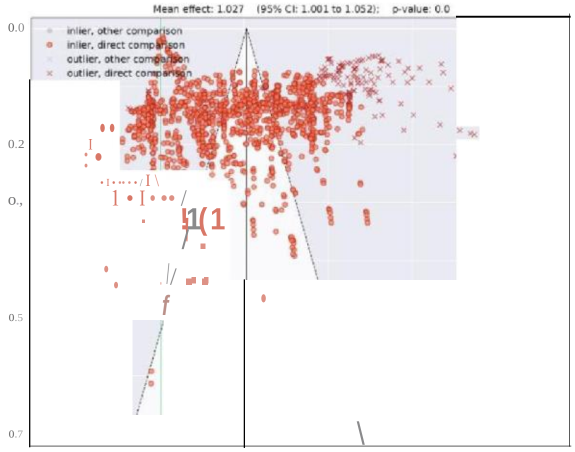

Bundle 262 !Periodontal Disease) Crosswalk - cv_cpiclassJ, Age Beta : 0.65

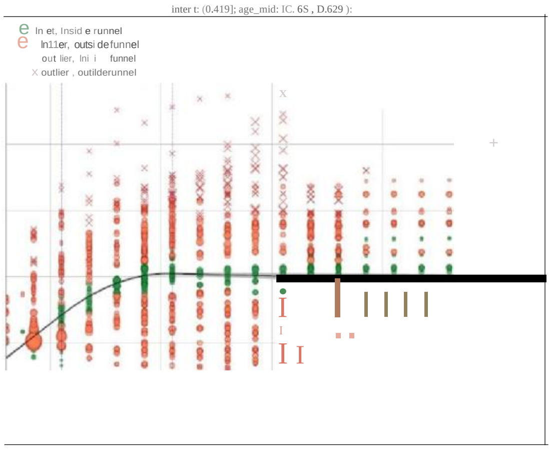

Bundle 262 (Periodontal Diseas e) Crosswalk - cv_atchloss4ormore. Age Beta: 0.793

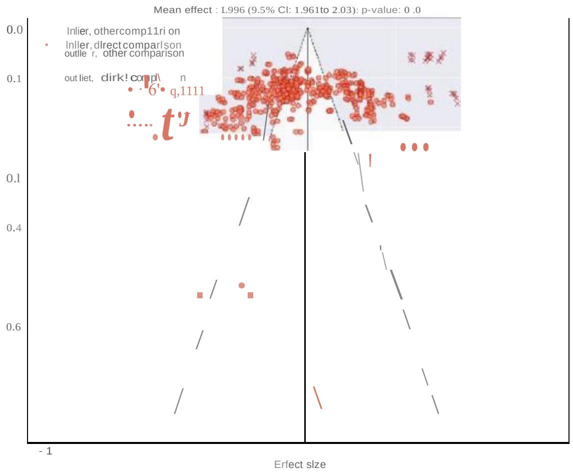

Bund le 2 6 2 (Pe rio dont a l Disease) Crosswalk - cv _atc hloss4 or m ore, Age Beta: 0.793

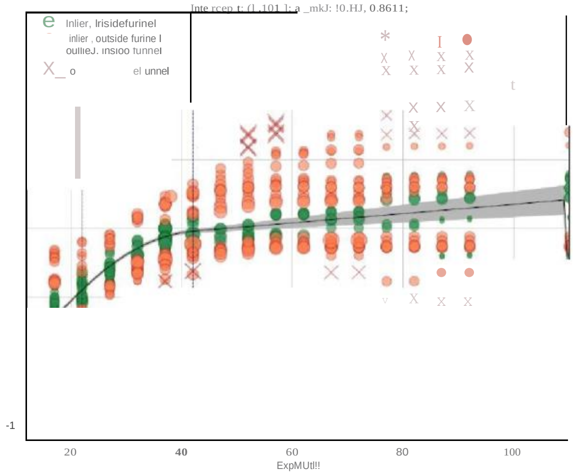

Bun dle 2 62 (Pe r i odo nta l Disease } Cross w alk cv_atchl oS55o rm ore , Age Beta: 0 .629

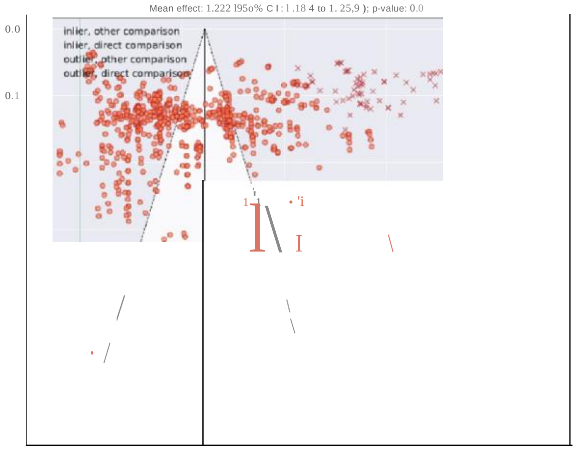

Bundle 262 (Periodo ntal Disease) Cro,;swalk- cv_atchlosIo5ormore , Age Beta : 0.629

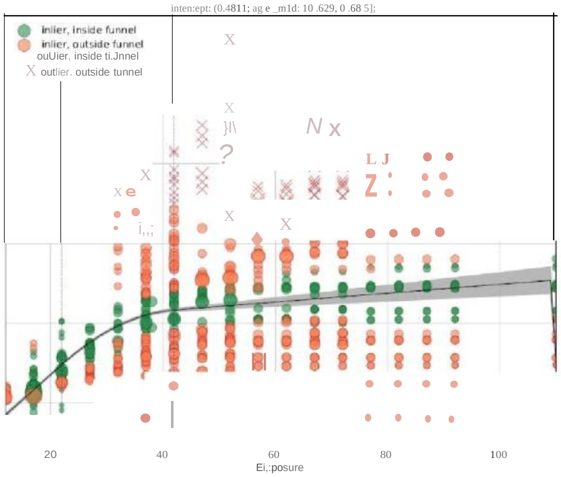

Modelling strategy

First, estimates for the prevalence of chronic periodontal disease were generated for each location/year/sex/age using DisMod-MR 2.1. Mortality was fixed to zero, and relative risk was fixed to 1.0 before age 30, as any excess cardiovascular events that occur in those with severe tooth loss would not be expected at young ages. Incidence and prevalence were assigned to be zero until age 8, as periodontal disease is largely considered to be a disease of adulthood. Incidence was allowed to rise beginning at age 9, based on the youngest age at which there was a non-zero point estimate for prevalence in the dataset. Additional bounds were assigned for incidence, remission, and excess mortality to improve plausibility in the DisMod estimates. Remission was bounded from 0 to 0.05, excess mortality rate from 0 to 0.0001, and incidence from 0 to 0.05. We considered these bounds to reasonably reflect the natural history of the disease. Three location-level covariates were used as shown in the table below.

Table 4: Covariate, parameter, beta, and exponentiated beta values for chronic periodontal diseases

Covariate name	Measure	Beta (UI)	Exponentiated beta (UI)
LDI (I$ per capita)	Prevalence	0.21 (0.156–0.261)	1.234 (1.169–1.298)
Age-standardised SEV for smoking	Prevalence	0.171 (0.01–0.464)	1.187 (1.01–1.591)
Age-standardised SEV for high fasting plasma glucose	Prevalence	0.39 (0.032–0.985)	1.476 (1.032–2.678)

Models were vetted based on the plausibility of the results, the extent to which estimates fit the data, and the plausibility of the range of estimates across location hierarchies.

Correction for edentulism

One systematic source of bias in the literature was the exclusion of edentate individuals from the study populations, which leads to systematic overestimation of periodontal disease prevalence when modelled over the entire population. To account for this bias, we used our GBD estimates of edentulism prevalence to adjust YLD estimates for chronic periodontal disease. Final DisMod-MR 2.1 estimates of edentulism prevalence were paired with the corresponding results for periodontal disease by age group, sex, location, and year to adjust for the proportion of the population that was excluded from the denominator of periodontal disease models.

Severity distributions and disability weights

We considered all estimated prevalent cases of chronic periodontal disease to experience the disability described by “bad breath, a bad taste in the mouth, and gums that bleed a little from time to time, but this does not interfere with daily activities”. The GBD disability survey differentiated between those who experience pain and those who do not, but the calculated disability weight was the same for both forms of the condition, 0.007 (0.003–0.014).

Other oral disorders

Other oral disorders encompass a wide variety of dental, tongue, and jaw disorders and malformations, including all oral disorders that are not included in the case definitions of permanent or deciduous

dental caries, periodontal disease, or edentulism and severe tooth loss. All data on the prevalence of other oral disorders were obtained from the United States MEPS, a nationally representative survey conducted yearly from 1996 to 2011 by the US Agency for Healthcare Research and Quality. These data were modelled in DisMod-MR 2.1 using a prevalence-only model. Disability weights and severity distribution for these causes were also derived from MEPS.

Table 2: Total number of sources and countries with data for other oral disorders, by measure

Measures	Total sources	Countries with data
All measures	19	1
Prevalence	16	1

References

1. C Mason, SR Porter, G Madland, J Parry. Early management of dental pain in children and adolescents. *J Dent.* Jan 1997; 25(1): 31-4.
2. RA Whyman, ET Treasure, KM Ayers. Dental disease levels and reasons for emergency clinic attendance in patients seeking relief of pain in Auckland. *NZ Dent J.* Dec 1996; 92(410): 114-7
3. RC Page and PI Eke. Case Definitions for Use in Population-Based Surveillance of Periodontitis. *J Periodont*. Jul 2007. 78(7 Suppl): 1387-99
4. Petersen PE, Baez RJ. Oral health surveys: basic methods. Geneva: World Health Organization; 2013.

Osteoarthritis

Flowchart

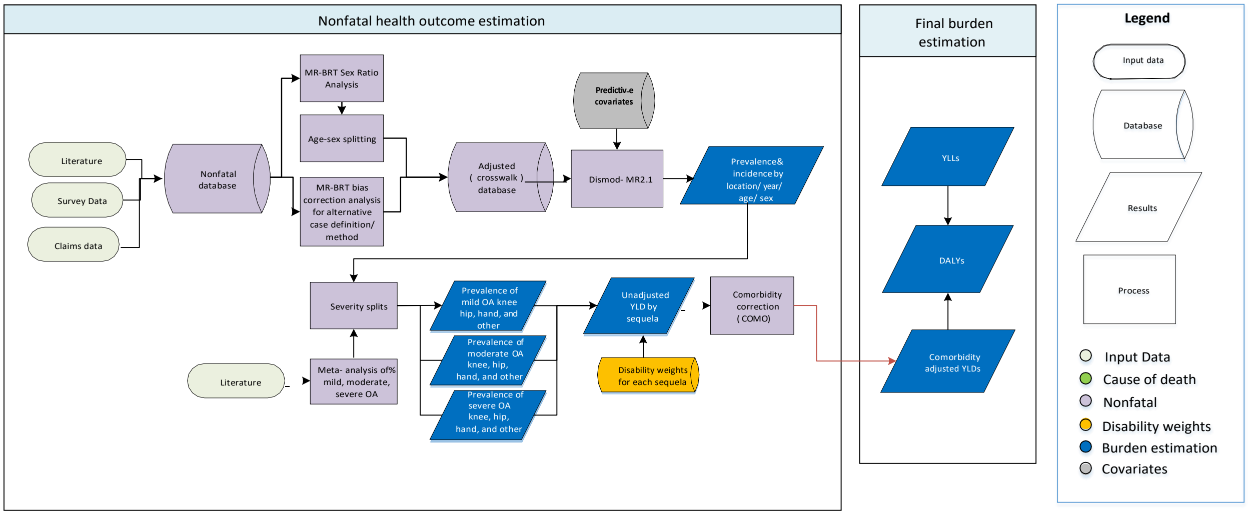

Input data and methodological summary for osteoarthritis

Case definition

Osteoarthritis (OA) is the most common form of arthritis, involving chronic inflammation, breakdown, and structural changes of whole joints. For the GBD study, four individual sites hip, knee, hand, and other sites were separately estimated. The hip, knee, and hand are the most common sites of OA. OA in the larger joints, such as the hip and knee, are considered to produce the greatest disability. Failure of these joints can lead to the need for joint replacement surgery, if available, and thus contributes to a significant proportion of the high direct health care costs attributable to arthritis. OA of the spine is also common; however, it was considered that any symptoms and disability related to the cervical, thoracic, and/or lumbar spine would be captured in the estimates of low back pain and neck pain.

The osteoarthritis (OA) reference case definition is symptomatic osteoarthritis radiologically confirmed as Kellgren-Lawrence grade 2–4. Prior to GBD 2019, we only estimated OA of the hip and knee. For GBD 2019, two new sites of OA were added, OA of the hand, with the same reference criteria present in any single hand joint type, and OA other, with the same reference criteria present in any joint other than those of the hand, hip, knee, or spine. Grade 2 symptomatic requires one defined osteophyte in the affected joint and pain for at least one month out of the last 12. Grade 3–4 symptomatic requires osteophytes and joint space narrowing in the affected joint with deformity also present for grade 4, and pain for at least one month out of the last 12 months.

ICD-10 codes for OA of the hip, knee, hand, and other are M16, M17, M18, and M19, respectively. The ICD-9 code for OA is 715, without specific codes for various sites.

The case definitions accepted for osteoarthritis are shown in the table below.

Reference or alternative	Definition
Alternative	Self-reported symptomatic OA physician diagnosis without the use of radiography
Alternative	Self-reported pain only without physician diagnosis
Alternative	Radiographically-confirmed OA without pain or without mention of the presence of pain
Alternative	USA claims data
Alternative	Taiwan claims data

Input data

The most recent systematic review for OA hip and OA knee was conducted in 2017 for studies published between 2013 and 2017. A systematic review of the prevalence, incidence, and mortality was performed on MEDLINE, EMBASE, CINAHL, CAB Abstracts, WHO Library (WHOLIS) and OpenSIGLE. For prevalence and incidence, the following search terms were used: (osteoarth* OR gonarthr*) AND (prevalen* OR inciden* OR cross-sectional OR cross sectional OR epidemiol* OR survey OR population-based OR population based OR population study OR population sample OR cohort OR follow-up OR follow up OR longitudinal OR regist*) AND (list of names of all GBD countries).

Exclusion criteria were:

1. Sub-populations clearly not representative of the national population

2. Not a population-based study
3. Low sample size (less than 150)
4. Review rather than original studies

We identified 1,864 articles and extracted data from 26. These studies were from 19 locations: Australia, Brazil, Canada, China, Ecuador, Egypt, India, Iran, United Kingdom, France, Japan, United States, Mongolia, Portugal, Spain, Mexico, Turkey, Venezuela, and Vietnam.

Figure 1: PRISMA diagram of osteoarthritis systematic review from 2013–2017

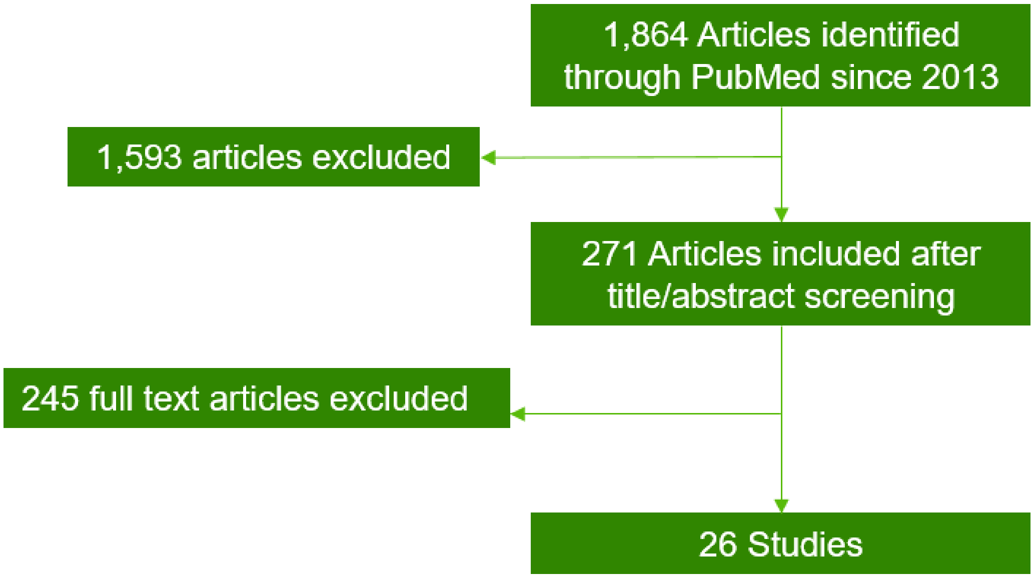

All existing sources used in the hip and knee models were re-reviewed for mention of prevalence and incidence of OA hand or OA other specifically. In order to gather more input data on prevalence for the new OA hand and OA other models, a broad systematic review was also conducted in 2019 specifically for data on these sites. A PubMed search was conducted for studies published between 1980 and 2019 using the following search terms: (("osteoarthritis" AND ("epidemiology" OR "prevalence")) AND "humans") AND ("population" OR "population groups" OR ("population" AND "groups")).

Figure 2: PRISMA diagram of osteoarthritis systematic review from 1980–2019

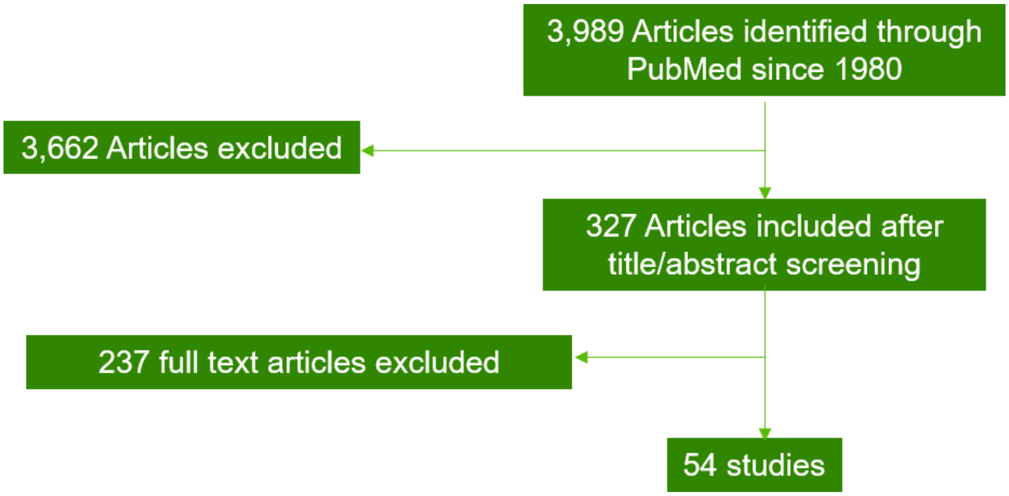

As in past rounds of the GBD, we decided not to use hospital inpatient data as we considered it would not be representative of true prevalence, and that variation between countries in the proportion of true prevalent cases captured in hospital inpatient data system would likely vary more than can be captured by a single crosswalk in DisMod-MR 2.1. Data from USA claims data for 2000 and 2010–2016 by state and Taiwan claims data from 2016 were included. There were very few sources identified through data re-review and systematic review for OA other, with minimal overlap in reported site. As a result, USA claims data constituted the only data input source for this model.

Table 1: Data Inputs for osteoarthritis morbidity modelling by parameter.

	**Countries with data**	**Total sources**
Total	44	173
Prevalence	44	161
Incidence	6	11
Remission	0	0
Other	2	2

Age and sex splitting

Reported estimates of prevalence were split by age and sex where possible. First, if studies reported prevalence for broad age groups by sex (eg, prevalence in 15- to 65-year-old males and females separately), and also by specific age groups but for both sexes combined (eg, prevalence in 15- to 30-year-olds, then in 31- to 65-year-olds, for males and females combined), age-specific estimates were split by sex using the reported sex ratio and bounds of uncertainty. Second, input data reporting prevalence of OA for both sexes that could not be split using a within-study ratio were split using a sex ratio derived from a meta-analysis of existing sex-specific data for each type of OA using MR-BRT (meta-regression—Bayesian, regularised, trimmed). The female to male ratio was 1.10 (1.09 to 1.12) for the hip, 1.44 (1.43 to 1.45) for the knee, and 2.36 (2.33 to 2.38) for the hand. There weren't any both sex input data for OA other. Finally, after the application of bias adjustments, where studies on OA hip and OA knee reported

estimates across age groups spanning 25 years or more, these were split into five-year age groups using the prevalence age pattern estimated by DisMod-MR 2.1 (disease model—Bayesian meta-regression) for each type of OA in GBD 2019. Wide age bin data for OA hand were split into five-year age groups using the prevalence age pattern of the USA claims input data. There weren't any wide age bin input data for OA other.

Data adjustment

For OA hip and OA knee, we marked studies that reported on X-rays only, self-reported OA with pain, or self-reported OA with no information on pain. Other studies identified cases of OA through a review of medical charts. We assumed that these cases were diagnosed by X-ray with pain present. We added three additional covariates for claims data in the USA from the year 2000 and from 2010 onward and for Taiwan claims data. For all these alternative case definitions we derived adjustment factors using MR-BRT. Claims data from Taiwan were excluded from the model, as we did not have data on the reference case definition from Taiwan to inform a reliable adjustment. Betas and exponentiated values (which can be interpreted as an odds ratio) for these two covariates are shown in the table below:

Table 2: MR-BRT crosswalk adjustment factors for OA hip

Data input	Reference or alternative case definition	Gamma	Beta Coefficient, Log (95% UI) *	Adjustment factor**
Radiography with pain	Ref	0.26	---	---
Radiography only	Alt		1.09 (0.89 to 1.28)	2.96 (2.44 to 3.6)
Self-reported OA with pain	Alt		1.32 (1.15 to 1.48)	3.73 (3.16 to 4.39)
Self-reported OA, no mention of pain	Alt		1.60 (1.18 to 2.01)	4.94 (3.26 to 7.49)
USA Claims data – 2000	Alt		-2.50 (-2.96 to -2.01)	0.082 (0.052 to 0.13)
USA Claims data – 2010–2016	Alt		-2.03 (-2.08 to -1.97)	0.13 (0.12 to 0.14)

**MR-BRT crosswalk adjustments can be interpreted as the factor the alternative case definition is adjusted by to reflect what it would have been had it been measured using the reference case definition. If the log/logit beta coefficient is negative, then the alternative is adjusted up to the reference. If the log/logit beta coefficient is positive, then the alternative is adjusted down to the reference.*

***The adjustment factor column is the exponentiated beta coefficient. For log beta coefficients, this is the relative rate between the two case definitions. For logit beta coefficients, this is the relative odds between the two case definitions.*

Table 3: MR-BRT crosswalk adjustment factors for OA knee

Data input	Reference or alternative case definition	Gamma	Beta Coefficient, Log (95% UI)*	Adjustment factor**
Radiography with pain	Ref	0.38	---	---
Radiography only	Alt		0.21 (0.14 to 0.27)	1.23 91.15 to 1.32)
Self-reported OA with pain	Alt		0.063 (-0.027 to 0.15)	1.065 (0.97 to 1.17)
Self-reported OA, no mention of pain	Alt		-0.77 (-0.81 to -0.72)	0.46 (0.44 to 0.48)
USA Claims data – 2000	Alt		-2.26 (-2.64 to -1.88)	0.10 (0.072 to 0.15)
USA Claims data – 2010–2016	Alt		-1.60 (-2.43 to -0.77)	0.20 (0.088 to 0.46)

**MR-BRT crosswalk adjustments can be interpreted as the factor the alternative case definition is adjusted by to reflect what it would have been had it been measured using the reference case definition. If the log/logit beta coefficient is negative, then the alternative is adjusted up to the reference. If the log/logit beta coefficient is positive, then the alternative is adjusted down to the reference.*

***The adjustment factor column is the exponentiated beta coefficient. For log beta coefficients, this is the relative rate between the two case definitions. For logit beta coefficients, this is the relative odds between the two case definitions.*

For OA hand, we allowed for alternatives to two dimensions of case definition: affected joint and diagnostic criteria. These alternative case definitions concerned studies reporting on the presence of OA in any single joint type (eg, distal interphalangeal), present in the first carpometacarpal joint of the thumb specifically, present in multiple joint types, or diagnosed as generalised hand OA. Adjustments were also considered for studies that used X-rays, studies in which a physician diagnosed OA without X-rays, studies that used reported pain, and studies that used self-report. We added two additional covariates for claims data in the USA from the year 2000 and from 2010 onward. The mean and standard error for the coefficients were calculated using the MR-BRT crosswalk adjustment method. Data concerning the presence of OA in the thumb base and through self-report were not included in the final model, as we were unable to find matches to inform a reliable crosswalk. Claims data in the USA were not included in the final model for the same reason. Betas and exponentiated values (which can be interpreted as an odds ratio) for these two covariates are shown in the table below:

Table 4: MR-BRT crosswalk adjustment factors for OA hand

Data input	Reference or alternative case definition	Gamma	Beta Coefficient, Log (95% UI)*	Adjustment factor**
Radiography with pain in a single joint type	Ref	0.36	---	---
OA in a single joint type	Alt		0.32 (0.29 to 0.34)	1.37 (1.34 to 1.40)

OA in multiple joint types	Alt		0.32 (0.30 to 0.34)	1.38 (1.35 to 1.41)
Generalised hand OA	Alt		-0.74 (-0.80 to -0.68)	0.48 (0.45 to 0.51)
Radiography only	Alt		1.09 (1.03 to 1.15)	2.97 (2.79 to 3.16)
Physician diagnosis only	Alt		0.58 (0.51 to 0.65)	1.78 (1.66 to 1.92)
Pain only	Alt		0.055 (0.0077 to 0.10)	1.06 (1.01 to 1.11)
Radiography with pain	Alt		0.31 (0.23 to 0.39)	1.36 (1.26 to 1.48)
Physician diagnosis with pain	Alt		0.28 (0.20 to 0.35)	1.32 (1.22 to 1.42)

**MR-BRT crosswalk adjustments can be interpreted as the factor the alternative case definition is adjusted by to reflect what it would have been had it been measured using the reference case definition. If the log/logit beta coefficient is negative, then the alternative is adjusted up to the reference. If the log/logit beta coefficient is positive, then the alternative is adjusted down to the reference.*

***The adjustment factor column is the exponentiated beta coefficient. For log beta coefficients, this is the relative rate between the two case definitions. For logit beta coefficients, this is the relative odds between the two case definitions.*

Modelling strategy

For OA hip and OA knee, prior settings in the DisMod-MR model included setting remission to 0, and it was assumed that there was no incidence or prevalence of OA before the age of 30 years. We assumed that excess mortality is zero. While there are some data on excess mortality risk, the values of hazard ratios or standardised mortality ratios are close to one, with some studies reporting mean estimates less than one. We made few substantive changes in the modelling strategy from GBD 2019. We included Mean BMI and the SEV scalar for osteoarthritis as country covariates on prevalence. The OA SEV scalar combines the exposure measures for risks estimated to impinge on OA in GBD: increased BMI.

Table 5. Covariates. Summary of covariates used in the OA hip and OA knee DisMod-MR meta-regression models

Covariate	Beta, log (95% Uncertainty Interval), OA Hip	Exponentiated beta (95% Uncertainty Interval), OA Hip	Beta, log (95% Uncertainty Interval), OA Knee	Exponentiated beta (95% Uncertainty Interval), OA Knee
Mean BMI	0.98 (0.86 to 1.00	2.66 (2.37 to 2.72)	0.72 (0.54 to 0.91)	2.06 (1.72 to 2.48)
Log-transformed age-standardised SEV scalar: OA	1.89 (0.0017 to 2.00)	6.62 (1.00 to 7.38)	0.77 (0.75 to 0.81)	2.16 (2.12 to 2.24)

For the OA hand and OA other models, settings in DisMod-MR included setting remission to 0, and assuming no incidence or prevalence of OA before the age of 30 years. In addition, we included the SEV scalar for OA as a country covariate on prevalence for OA other in order to provide a basis for some geographic variation in a model that only has input data in the USA. This covariate was not used in the OA hand model because we did not have reason to believe that there is a reliable relationship between increased BMI and OA in hand joints.

Table 6. Covariates. Summary of covariates used in the OA other DisMod-MR meta-regression model

Covariate	Beta, log (95% Uncertainty Interval)	Exponentiated beta (95% Uncertainty Interval)
Log-transformed age-standardised SEV scalar: OA	1.23 (1.20 to 1.25)	3.43 (3.32 to 3.49)

Severity and disability

The basis of the GBD disability weight survey assessments are lay descriptions of sequelae highlighting major functional consequences and symptoms. The lay descriptions and disability weights for OA severity levels are shown below.

Table 7. Severity distribution, details on the severity levels for OA in GBD 2019 and the associated disability weight (DW) with that severity.

Severity level	Lay description	DW (95% CI)
Asymptomatic		0
Mild	This person has pain in the leg, which causes some difficulty running, walking long distances, and getting up and down.	0.023 (0.013–0.037)
Moderate	This person has moderate pain in the leg, which makes the person limp, and causes some difficulty walking, standing, lifting and carrying heavy things, getting up and down, and sleeping.	0.079 (0.054–0.110)
Severe	This person has severe pain in the leg, which makes the person limp and causes a lot of difficulty walking, standing, lifting and carrying heavy things, getting up and down, and sleeping.	0.165 (0.112–0.232)

In past GBD rounds, to determine the proportion of people with OA within each of the severity levels, four studies representing the High-income, South Asia, and Southeast Asia, East Asia, and Oceania super regions provided information on the severity of OA. In GBD 2017, data from the USA Osteoarthritis Initiative study were included as well. The OA Initiative is a large cohort study that follows individuals with OA of the knee recruited from four centres around the USA. In all five studies, severity was

classified based on the Western Ontario and McMaster Universities Arthritis Index (WOMAC) with scores 0–5 taken as mild, 6–13 as moderate, and 14 and higher as severe. Estimates were pooled across studies using a random effects meta-analysis model. The pooled percentages were mild 47.0% (42.2–51.9), moderate 35.9% (31.3–40.7), and severe 17.1% (12.9–21.6) pooled between patient and physician ratings in a study from Bangladesh, which we apply to low- and middle-income countries. The pooled proportions from three high-income countries were mild 74.3% (64.8–82.7), moderate 24.3% (16.4–33.1), and severe 1.1% (0.6–1.7). After streaming out 1,000 draws assuming a binomial distribution, percentages were scaled to sum to 1 at each draw. For the sake of consistency, the same severity distribution and disability weights were applied to OA hand and OA other, to be reconsidered in the subsequent modelling round.

Other cardiovascular disease

Flowchart

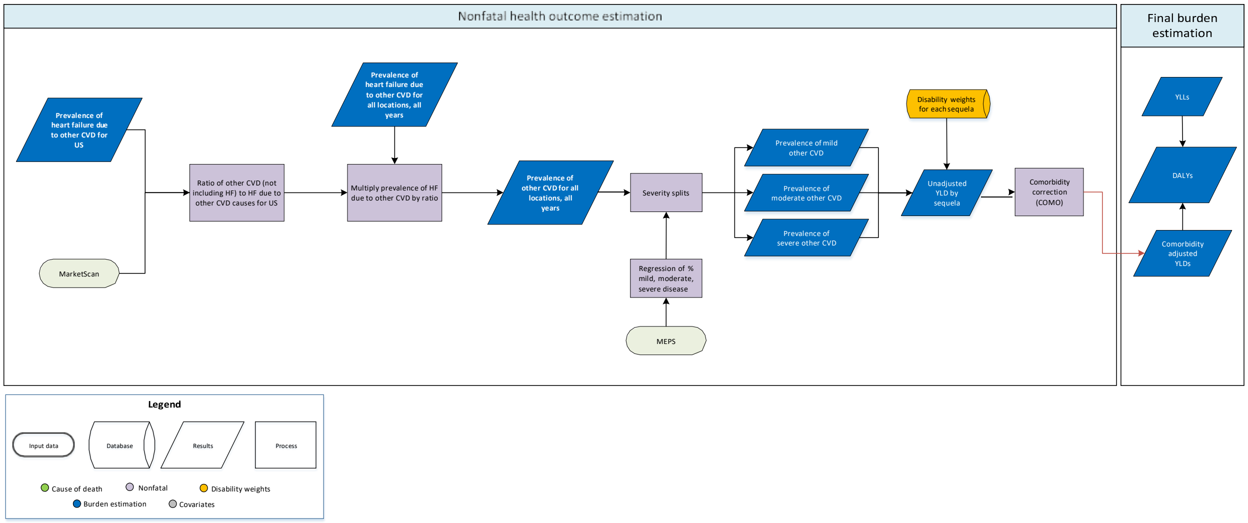

Case definition

Other cardiovascular disease is a residual category resulting from the GBD approach of estimating the total burden of all causes. Prevalence estimates are produced in order to provide YLDs consistent with the estimated YLLs from the death modelling process and to enable the calculation of DALYs.

Conditions included in this cause, based on ICD codes used for both fatal and non-fatal modelling, are other diseases of pulmonary vessels; acute pericarditis; other diseases of pericardium; pericarditis in diseases classified elsewhere; paroxysmal tachycardia; cardiac septal defect, acquired; rupture of chordae tendineae, not elsewhere classified; rupture of papillary muscle, not elsewhere classified; intracardiac

thrombosis, not elsewhere classified; cerebral amyloid angiopathy; other aneurysm; other disorders of arteries and arterioles; diseases of capillaries; disorders of arteries, arterioles, and capillaries in diseases classified elsewhere; phlebitis and thrombophlebitis; portal vein thrombosis; other venous embolism and thrombosis; varicose veins of lower extremities; varicose veins of other sites; other disorders of veins; non-specific lymphadenitis; other non-infective disorders of lymphatic vessels and lymph nodes; other disorders of circulatory system in diseases classified elsewhere. As of GBD 2021, codes for pulmonary arterial hypertension (PAH) are no longer included, as PAH is modelled separately.

Input data

As this is a residual category, we used inpatient and outpatient claims data from the USA (MarketScan) and modelled estimates from heart failure due to other cardiovascular disease to estimate prevalence of other cardiovascular disease. MarketScan replaced data from the Medical Expenditure Panel Survey, used in GBD 2019 and before. Details on MarketScan and methods used to extract cause-specific prevalence estimates are detailed in the “Claims data” section of the appendix.

Severity split inputs

The proportions of asymptomatic, mild, moderate, and severe cases for other cardiovascular diseases were determined by the standard approach for severity splitting for GBD 2021 that utilised the Medical Expenditure Panel Survey (MEPS) to map other cardiovascular diseases ICD codes (see table 1) to quality of life metrics to quantify disability. More information on methodology on the proportion split using MEPS can be found in the appendix section 4.7: Severity distribution. The table below includes lay descriptions and disability weights for the severity levels of other cardiovascular disease for GBD 2021.

Severity level	Lay description	DW (95% CI)
Asymptomatic		N/A
Mild	Is short of breath and easily tires with moderate physical activity, such as walking uphill or more than a quarter-mile on level ground. The person feels comfortable at rest or during activities requiring less effort.	0.041 (0.026–0.062)
Moderate	Is short of breath and easily tires with minimal physical activity, such as walking only a short distance. The person feels comfortable at rest but avoids moderate activity.	0.072 (0.047–0.103)
Severe	Is short of breath and feels tired when at rest. The person avoids any physical activity, for fear of worsening the breathing problems.	0.179 (0.122–0.251)

Source counts

**Measure**	Total sources	Countries with data
Proportion	19	1

Modelling strategy

To obtain prevalence estimates of other cardiovascular disease, we used MarketScan data combined with prevalence estimates of heart failure due to other CVD for the USA to estimate the ratio of the prevalence of heart failure due to other CVD causes in 2015 to the prevalence of other CVD causes in 2015. We then applied this ratio to the age-, sex-, and year-specific prevalence estimates for heart failure due to other CVD causes for all locations to generate prevalence estimates of other cardiovascular disease. Estimation of heart failure due to other CVD is detailed elsewhere in the appendix.

In GBD 2021, updates to heart failure methods made between GBD 2017 and GBD 2019 were applied to this cause. Estimates of heart failure due to other CVD causes are now informed by person-level multiple cause of death data from the USA, Mexico, Colombia, Brazil, and Taiwan (province of China), linked longitudinal data from Italy, and cause-specific mortality estimates from all locations. This substantial methodological improvement led to changes in estimates of heart failure due to other CVD, and therefore changes in estimates of other CVD.

Other chronic respiratory diseases

In addition to the chronic respiratory diseases, there are other types of chronic respiratory diseases with a range of severities and associated sequelae. Because these chronic respiratory diseases are diverse in their underlying causes and risk factors, as well as in their associated health outcomes, modelling them together in a DisMod-MR model would not produce reliable estimates of prevalence. Instead, we calculated the YLDs caused by other chronic respiratory diseases directly using a YLD/YLL ratio as a 'place holder'.

We calculated the ratio of YLDs to YLLs across the specified chronic respiratory diseases for which non-fatal outcomes were modelled, using YLL estimates from the GBD 2021 cause of death analysis. We then multiplied this YLD/YLL ratio by the YLL estimates for other chronic respiratory diseases.

Other digestive diseases

In addition to specified digestive diseases including inguinal, femoral, and abdominal hernia, inflammatory bowel disease, gastro-oesophageal reflux disease, gastritis and duodenitis, peptic ulcer disease, gallbladder and biliary diseases, appendicitis, paralytic ileus and intestinal obstruction, vascular intestinal disorders, and pancreatitis, there are a number of other types of digestive diseases with a range of severities and associated sequelae. Because these digestive diseases are diverse in their underlying causes and risk factors, as well as in their associated health outcomes, modelling them together in a DisMod-MR model would not produce reliable estimates of prevalence. Instead, we calculated the YLDs caused by other digestive diseases directly using an YLD/YLL ratio as a "placeholder".

We calculated the ratio of YLDs to YLLs across the specified digestive diseases for which non-fatal outcomes were modelled, using YLL estimates from the GBD 2021 cause of death analysis. We then multiplied this YLD/YLL ratio by the YLL estimates for other digestive diseases.

Other drug use disorders

Flowchart

Other drug use disorders

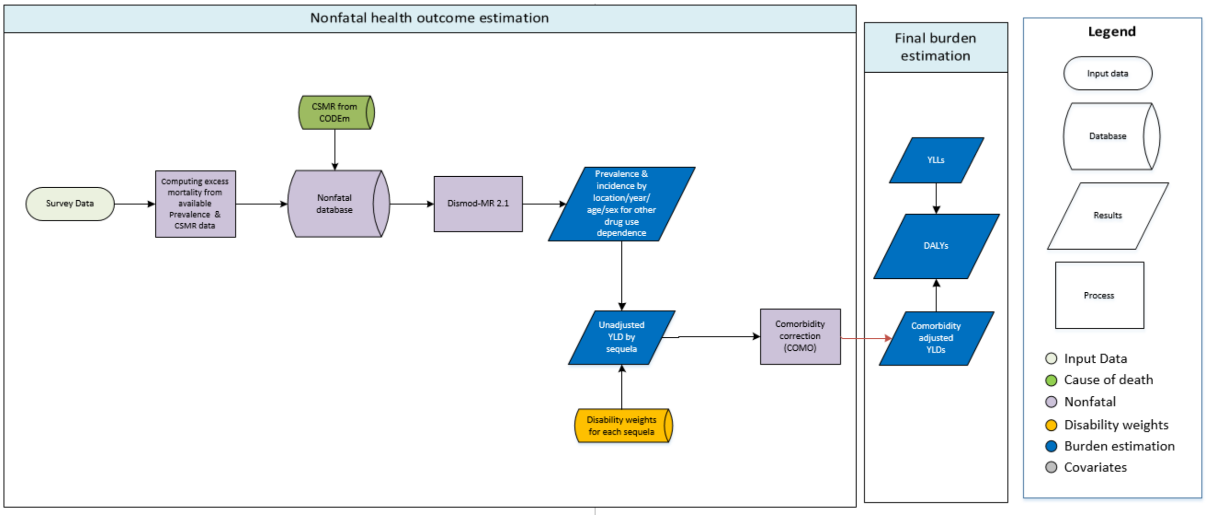

Input data and methodological summary for other drug use disorders

Case definition

In addition to the four drug use disorders for which we specifically estimate non-fatal burden (opioid, cocaine, amphetamine, and cannabis dependence), we also estimate the burden attributable to a residual cause of "other drug use disorders." This is made up of an aggregate group of other forms of drug dependence. Included in the Global Burden of Disease (GBD) modelling were cases meeting the Diagnostic and Statistical Manual of Mental Disorders (DSM-IV-TR)[1] or the International Classification of Diseases (ICD-10)[2] diagnostic criteria for:

- Hallucinogen dependence
- Inhalant or solvent dependence
- Sedative dependence
- Tranquiliser dependence
- Other medicines, drugs, substance dependence

According to DSM-IV TR criteria, dependence involves a maladaptive pattern of substance use, leading to clinically significant impairment or distress. At least three of the following symptoms must be experienced within the same 12-month period:

- Tolerance, characterised by either
  - a need for increased amounts of the substance to achieve intoxication; or
  - markedly diminished effect with continued use of the same amount of the substance;
- Withdrawal, characterised by either
  - Withdrawal symptoms characteristic to dependence; or
  - the same (or similar) substance is taken to avoid withdrawal symptoms;
- Substance taken in progressively larger amounts or for longer periods;
- Persistent desire or unsuccessful efforts to reduce substance use;

- Disproportionate time dedicated to obtaining the substance;
- Other important activities are given up because of the substance use; and
- Substance use is continued despite knowledge of physical or psychological problems occurring as a result of the substance.

Input data

Prevalence estimates were obtained from the Australian National Survey of Mental Health and Wellbeing (NSMHWB) conducted in 1997,[3] and the US National Epidemiological Survey on Alcohol and Related Conditions (NESARC), conducted in two waves in 2001–2002[4] and 2004–2005.[5] Given that other forms of drug dependence often co-occur with the four types of drug dependence for which we estimate non-fatal burden (opioid, cocaine, amphetamine, and cannabis dependence), an adjustment for co-morbidity is important so as not to overestimate the overall burden attributable to drug dependence. Participants meeting criteria for any other form of drug dependence from each of the surveys used were counted as a prevalent case only if they did not simultaneously meet criteria for opioid, cocaine, amphetamine, or cannabis dependence.

Table 1: Data inputs for other drug use disorders morbidity modelling by parameter

Measure	Total sources	Countries with data
All measures	4	2
Prevalence	4	2

Modelling strategy

The GBD 2021 epidemiological modelling strategy made use of our disease model—Bayesian meta-regression tool[7] (DisMod-MR 2.1). Information on DisMod-MR 2.1 can be found in appendix 1, section 4.5 of the reference article.

A number of additional expert priors were used in order to run a full parameter model. We assumed no incidence before age 14, a maximum of 0.0004 on incidence from the age of 60 years onward, and a maximum remission of 0.2. These priors were corroborated with expert feedback and existing literature on drug use disorders including the European Monitoring Centre for Drugs and Drug Addiction.[6] Finally, cause-specific mortality rates (CSMR) from the GBD 2021 cause of death model for other drug use disorders were included as datapoints in the DisMod-MR model. A YLL to YLD ratio analysis had been used to calculate prevalence prior to GBD 2016. This step was removed for GBD 2021 to avoid double-counting. As a result, YLDs were decreased globally.

Severity and disability

The basis of the GBD disability weight survey assessments are lay descriptions of sequelae highlighting major functional consequences and symptoms. The average disability weight estimated for cocaine and amphetamine dependence was applied to all cases in this residual group of other drug use disorders. The cocaine and amphetamine lay descriptions and disability weights are shown below.

Table 2. Severity distribution, details on the severity levels for amphetamine use and cocaine use disorders in GBD 2021, and the associated disability weight (DW) with that severity

Severity level	Lay description	DW (95% CI)
**Amphetamine dependence**		
Mild	Uses stimulants (drugs) at least once a week and has some difficulty controlling the habit. When not using, the person functions normally.	0.079 (0.051–0.114)
Moderate to severe	Uses stimulants (drugs) and has difficulty controlling the habit. The person sometimes has depression, hallucinations, and mood swings, and has difficulty in daily activities.	0.486 (0.329–0.637)
**Cocaine dependence**		
Mild	Uses cocaine at least once a week and has some difficulty controlling the habit. When not using, the person functions normally.	0.116 (0.074–0.165)
Moderate to severe	Uses cocaine and has difficulty controlling the habit. The person sometimes has mood swings, anxiety, paranoia, hallucinations, and sleep problems, and has some difficulty in daily activities.	0.479 (0.324–0.634)

References

1. American Psychiatric Association. Diagnostic and Statistical Manual of Mental Disorders (DSM-IV-TR). 4th, Text Revision ed. Washington DC: 2000.

2. World Health Organization. The ICD-10 Classification of Mental and Behavioural Disorders. Clinical descriptions and diagnostic guidelines. Geneva: 1992.

3. Australia Bureau of Statistics. Australia National Survey of Mental Health and Wellbeing 1997. Canberra: 1997.

4. National Institute on Alcohol Abuse and Alcoholism (NIAAA), National Institutes of Health (NIH). United States National Epidemiologic Survey on Alcohol and Related Conditions 2001-2002. 2002.

5. National Institute on Alcohol Abuse and Alcoholism (NIAAA), National Institutes of Health (NIH). United States National Epidemiologic Survey on Alcohol and Related Conditions 2004-2005. 2005.

6. European Monitoring Centre for Drugs and Drug Addiction. Lisbon, Portugal: 2014.

7. Vos T, Lim SS, Abbafati C, et al. Global burden of 369 diseases and injuries in 204 countries and territories, 1990–2019: a systematic analysis for the Global Burden of Disease Study 2019. The Lancet 2020; 396: 1204–22. doi: https://doi.org/10.1016/S0140-6736(20)30925-9

Other intestinal infectious diseases

In addition to the intestinal infectious diseases described above, there are many diverse types of intestinal infectious diseases. Because these intestinal infectious diseases are diverse in their underlying causes and risk factors as well as in their associated health outcomes, modelling them together in a DisMod-MR model would not produce reliable estimates of prevalence or excess mortality. Instead, we calculated the YLDs caused by intestinal infectious diseases directly using a YLD/YLL ratio.

We calculated the ratio of YLDs to YLLs across the specified intestinal infectious diseases for which nonfatal outcomes were modelled, using YLL estimates from the GBD 2021 cause of death (CoD) analysis. We then multiplied this YLD/YLL ratio by the YLL estimates for other intestinal infectious diseases from the GBD 2021 CoD analysis, providing us with an estimate of the YLDs associated with other intestinal infectious diseases.

Other mental disorders

Other mental disorders: Personality disorders

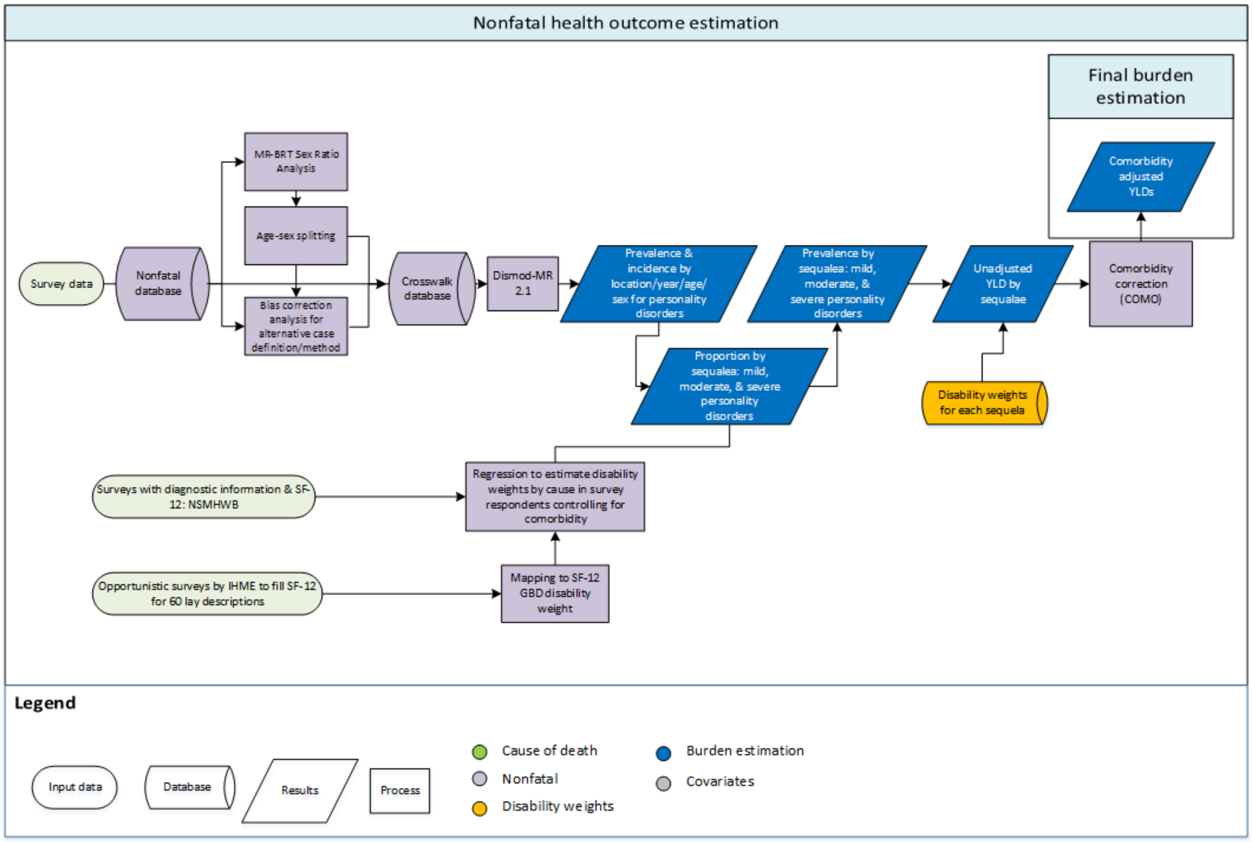

Input data and methodological summary for other mental disorders

Case definition

In addition to the individual mental disorders for which we estimate burden, we also estimate the non-fatal burden attributable to a residual cause of "other mental disorders." This is made up of an aggregate group of personality disorders. Personality disorders are characterised by pervasive, inflexible and maladaptive patterns of behaviour and inner experience which are markedly different from what is considered to be acceptable in the individual's culture. These disorders tend to be chronic and are associated with significant distress or disability. Included in GBD 2021 were cases meeting diagnostic criteria for personality disorders according to the Diagnostic and Statistical Manual of Mental Disorders (DSM-IV-TR: 300.3, 301.0; 301.2, 301.22, 301.5–301.9), or the equivalent diagnosis in the International Classification of Diseases (ICD-10: F60).[1,2] The aggregated group of DSM personality disorders used in GBD 2021 captured any of the following;

- Paranoid personality disorder
- Schizoid personality disorder
- Schizotypal personality disorder
- Antisocial personality disorder
- Borderline personality disorder
- Histrionic personality disorder

- Narcissistic personality disorder
- Avoidant personality disorder
- Dependent personality disorder
- Obsessive-compulsive personality disorder
- Personality disorder not otherwise specified

Input data

Prevalence estimates for the above personality disorders were obtained from the US National Epidemiological Survey on Alcohol and Related Conditions (NESARC, conducted in two waves from 2001–2002 and 2004–2005)[3] and the Australian National Survey of Mental Health and Wellbeing of Adults (NSMHWB, conducted in 1997).[4] Given that personality disorders often co-occur with other mental and substance use disorders, an adjustment for comorbidity is important so as not to overestimate the overall burden attributable to mental and substance use disorders. Participants meeting criteria for any type of personality disorder from the NESARC and NSMHWB surveys were counted as a prevalent case only if they did not simultaneously meet criteria for another mental and substance use disorders featured in GBD 2021. Table 1 summarises data inputs by parameter for other mental disorders.

Table 1: Data Inputs for other mental disorders morbidity modelling by parameter

**Parameter**	**Countries with data**	**New sources**	**Total sources**
Incidence	0	0	0
Prevalence	2	0	3
Remission	0	0	0
Other	0	0	0

Bias corrections/crosswalks

Estimates with known biases were adjusted/crosswalked accordingly prior to DisMod-MR 2.1. A NESARC: NSMHWB prevalence ratio of 2.04 (95% uncertainty interval [UI]: 1.82–2.34) was used to adjust all datapoints derived from NESARC toward the level of datapoints from the NSMHWB. The latter survey was made up of a more representative list of personality disorders and produced estimates along the levels of what we would expect for personality disorders. As this ratio was informed by only two data sources it was estimated outside of the meta-regression—Bayesian, regularised, trimmed (MR-BRT) analysis typically used for bias correction in GBD 2021.

Modelling Strategy

We have made no substantive changes in the modelling strategy from GBD 2019.

After the above data processes were applied, DisMod MR 2.1 was used to model the epidemiological data for personality disorders. Adjustments to model priors or the dataset were made where appropriate. Where outliers were identified in the data, we reassessed the study's methodology and quality before a decision was made to exclude or include the data.

As we only had prevalence data available, a number of expert priors were used in order to run a full-parameter model. We assumed no incidence and prevalence before age 14. This minimum age of onset was corroborated with expert feedback and DSM criteria highlighting the fact that personality disorders typically become recognisable during adolescence and early adulthood. Remission was set to a maximum of 0.01, given that these are understood to be chronic disorders with little or no complete remission. Excess mortality was set to 0 in this model, in the absence of mortality data required for DisMod-MR 2.1 modelling purposes. Given the sparsity of data, we applied a restriction on location random-effects of -0.1 to 0.1 to further guide prevalence estimation.

Severity splits and disability weights

The GBD disability weight survey assessments include lay descriptions of sequelae highlighting major functional consequences and symptoms. The lay descriptions and disability weights applied to the personality disorders within this residual group are shown below and were those estimated for anxiety disorders (See Table 2). To determine the proportion of people with personality disorders within each of the severity levels, the NSMHWB survey was used to estimate the proportion of cases asymptomatic (30%, 28%–32%), mild (41%, 33%–47%), moderate (15%, 11%–20%) and severe (14%, 10%–18%).

Table 2. Severity distribution, details on the severity levels for other mental disorders and the associated disability weight with that severity

Severity level	Lay description	Disability weight (95% UI)
Mild	Feels mildly anxious and worried, which makes it slightly difficult to concentrate, remember things, and sleep. The person tires easily but is able to perform daily activities.	0.03 (0.018–0.046)
Moderate	Feels anxious and worried, which makes it difficult to concentrate, remember things, and sleep. The person tires easily and finds it difficult to perform daily activities.	0.133 (0.091–0.186)
Severe	Constantly feels very anxious and worried, which makes it difficult to concentrate, remember things, and sleep. The person has lost pleasure in life and thinks about suicide.	0.523 (0.362–0.677)

There were no significant changes in GBD 2021 results for other mental disorders compared to GBD 2019. In this model, global prevalence was exclusively estimated using prevalence estimates from two surveys from the United States and Australia where we had unit record data available to estimate the prevalence of personality disorders, excluding those not simultaneously meeting criteria for another mental or substance use disorder. The sparsity of data leads to modelled prevalence estimates with large uncertainty bounds, which are sensitive to model re-runs and small changes to model settings. We are currently undertaking a literature review of population-survey data on the epidemiology of personality disorders across low-, middle-, and high-income countries with the aim of providing more robust and globally representative burden estimates for personality disorders in future GBD studies.

Other musculoskeletal disorders (Other MSK)

Flowchart

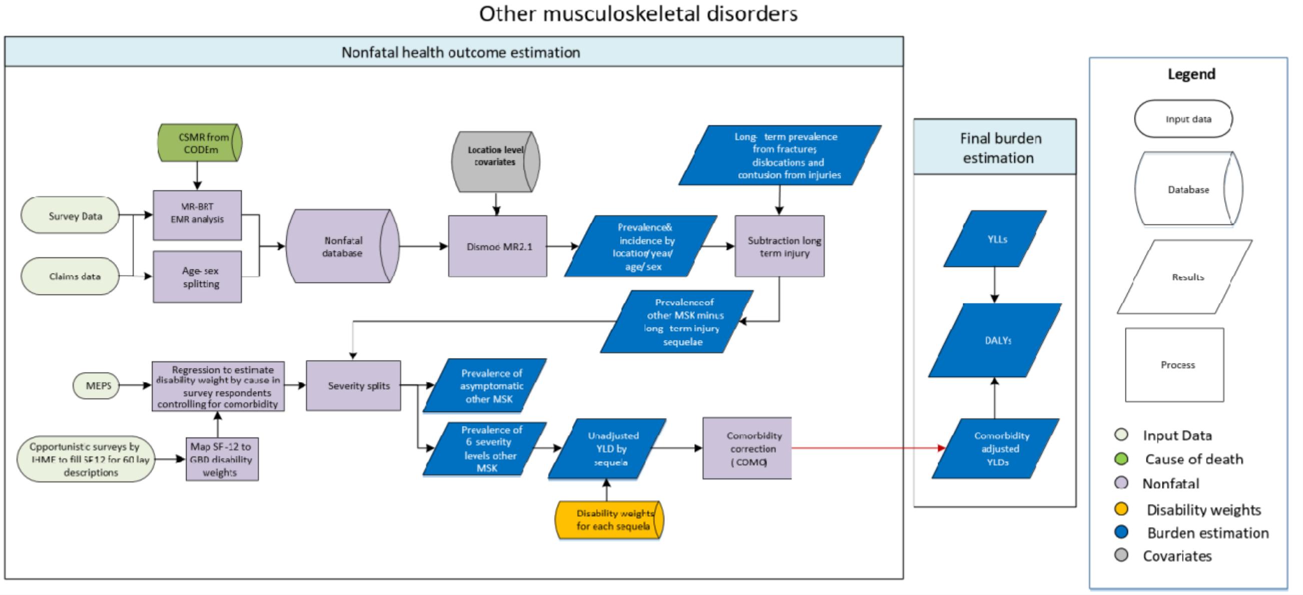

Input data and methodological summary for other MSK

Case definition

Other musculoskeletal disorders is a heterogeneous rest category comprising a wide range of disorders of muscles, bones, and ligaments that are not included in the five GBD defined musculoskeletal diseases – rheumatoid arthritis, osteoarthritis, low back pain, neck pain, and gout – and are not captured as long-term sequelae of injuries.

The utilised case definitions for other musculoskeletal disorders are listed below.

**Reference or alternative**	**Definition**
Reference	Prevalence of any of the following conditions: lupus erythematosus, infectious arthropathies, inflammatory polyarthropathies, other joint disorders, systemic connective tissue disorders, deforming dorsopathies, spondylopathies, disorders of the muscles, disorders of synovium and tendon, other soft tissue disorders, disorders of bone density and structure, osteomyelitis, other otseopathies, chondropathies, other disorders of the musculoskeletal system and connective tissue not included under gout, rheumatoid arthritis, osteoarthritis, low back pain, or neck pain
Alternative	USA claims data 2010–2015
Alternative	USA claims data 2000

The table below provides detail of the ICD-10 and ICD-9 codes included in this category.

ICD-10 codes	ICD-9 codes
L93—Lupus erythematosus<br>M00-M02—Infectious arthropathies<br>M08, M11-M13—Inflammatory polyarthropathies<br>M20-M25—Other joint disorders<br>M30-M35—Systemic connective tissue disorders<br>M40-M43—Deforming dorsopathies<br>M45-M46—Spondylopathies<br>M60 -M63—Disorders of muscles<br>M65-M68—Disorders of synovium and tendon<br>M70- M73, M75-M79—Other soft tissue disorders<br>M80-M85—Disorders of bone density and structure<br>M86—Osteomyelitis<br>M87-M90—Other osteopathies<br>M91-M94—Chondropathies<br>M95-M99—Other disorders of the MSK system and connective tissue	710.0<br>711<br>712–713<br>716–719<br>710.1–710.9<br>737<br>720–721<br>725<br>726–728<br>729<br>733.0-2<br>730.1–730.3, 730.7-9<br>731, 733.3-9<br>732<br>734–736, 738–739

Input data

The above ICD codes were used to extract other MSK prevalence from USA claims data for 2000 and 2010–2016 by state. The systematic review concentrated on finding health surveys that measured an overall amount of musculoskeletal disorders and reported information to distinguish a rest category that was not OA, RA, gout, or low back or neck pain. These data sources are based on self-reported musculoskeletal conditions or symptoms and did not use the listed ICD codes.

Table 1. Data inputs

Measure	Total sources	Countries with data
All measures	71	23
Prevalence	68	23
Proportion	15	1

Age and sex splitting

Reported estimates of prevalence were split by age and sex where possible. First, if studies reported prevalence for broad age groups by sex (eg, prevalence in 15- to 65-year-old males and females separately), and by specific age groups but for both sexes combined (eg, prevalence in 15- to 30-year-olds, then in 31- to 65-year-olds, for males and females combined), age-specific estimates were split by sex using the reported sex ratio and bounds of uncertainty. Second, prevalence data for both sexes that could not be split using a within-study ratio were split using a sex ratio derived from a meta-analysis of existing sex-specific data using MR-BRT (meta-regression— Bayesian, regularised, trimmed) The female to male ratio was 1.37 (1.37–1.38). Finally, where studies reported estimates across age groups spanning 25 years or more, these were split into five-year age groups using the prevalence age pattern estimated by DisMod-MR 2.1 in GBD 2017.

Data adjustment

In previous rounds, we used two study covariates to adjust claims data from the USA by state from the year 2000 and from 2010 onward. For GBD 2019 onward, we did not carry out bias adjustments for claims data because claims sources are more likely to capture all of the ICD codes included in the other MSK category and reflect the assumed mutual exclusivity of component disorders than study and survey data. In future rounds of the GBD, we intend to begin the process of modelling certain component disorders independently in order to more accurately reflect their prevalence and reduce variability of input data for the remaining disorders in the other MSK model.

Modelling strategy

Prior settings in the DisMod-MR model included the assumption of no incidence or prevalence of other MSK before the age of 10 years. In the absence of any meaningful data on incidence and remission for such a heterogeneous category of disorders, we made a rather arbitrary decision of remission of 0.5–1, ie, an average duration of 1–2 years. We also included the Socio-demographic Index country covariate with bounds set at –1 and 1. These settings were retained for GBD 2021.

Despite its inconsistencies between CSMR and prevalence prior to the inclusion of the modelled EMR data, the final other MSK model both excludes predicted data for the EMR (excess mortality rate) parameter and has the GBD 2017 DisMod-MR EMR calculation disabled. This is because the input data for the EMR MR-BRT analysis represented a narrow range of relatively high Healthcare Access and Quality (HAQ) Index locations, which resulted in far greater predicted EMR in data sparse, lower HAQ Index locations than in prior rounds, suppressing prevalence to implausibly low levels. Data for cause-specific mortality rate were also excluded from the model (arguing that the pattern of mortality comes from autoimmune diseases which constitute only a small fraction of the non-fatal manifestations captured in this residual category), a 15-year time window was set, and bounds of 0 to 0 were set on EMR, while retaining the HAQ Index country covariate on the parameter.

Table 2. Covariates. Summary of covariates used in the other MSK DisMod-MR meta-regression model

Covariate	Type	Parameter	Exponentiated beta (95% uncertainty interval)
Healthcare Access and Quality Index	Country-level	Excess mortality rate	0.95 (0.94–0.96)
Socio-demographic Index	Country-level	Prevalence	2.71 (2.69–2.72)

Severity and disability

The basis of the GBD disability weight survey assessments are lay descriptions of health states highlighting major functional consequences and symptoms. The lay descriptions and disability weights

for other MSK severity levels are shown below. They include the three levels of health states that are used for osteoarthritis and rheumatoid arthritis, each.

Table 3. Severity distribution, details on the severity levels for other MSK in GBD 2019 and the associated disability weight (DW) with that severity.

Severity level	Lay description	DW (95% CI)	Proportions
Asymptomatic			0.28 (0.27–0.29)
Musculoskeletal problems, lower limbs, mild	This person has pain in the leg, which causes some difficulty running, walking long distances, and getting up and down.	0.023 (0.013–0.040)	0.22 (0.15–0.30)
Musculoskeletal problems, upper limbs, mild	This person has mild pain and stiffness in the arms and hands. The person has some difficulty lifting, carrying, and holding things.	0.028 (0.017–0.046)	0.20 (0.15–0.29)
Musculoskeletal problems, upper limbs, moderate	This person has moderate pain and stiffness in the arms and hands, which causes difficulty lifting, carrying, and holding things, and trouble sleeping because of the pain.	0.115 (0.079–0.163)	0.10 (0.06–0.15)
Musculoskeletal problems, lower limbs, severe	This person has severe pain in the leg, which makes the person limp and causes a lot of difficulty walking, standing, lifting and carrying heavy things, getting up and down, and sleeping.	0.163 (0.109–0.224)	0.06 (0.04–0.07)
Musculoskeletal problems, generalised, moderate	This person has pain and deformity in most joints, causing difficulty moving around, getting up and down, and using the hands for lifting and carrying. The person often feels fatigue.	0.312 (0.201–0.438)	0.07 (0.06–0.08)
Musculoskeletal problems, generalised, severe	This person has severe, constant pain and deformity in most joints, causing difficulty moving around, getting up and down, eating, dressing, lifting, carrying, and using the hands. The person often feels sadness, anxiety, and extreme fatigue.	0.572 (0.370–0.758)	0.07 (0.07–0.08)

The severity distributions were derived from an analysis of the Medical Expenditure Panel Surveys (MEPS) in the USA. MEPS is an overlapping continuous panel survey of the United States non-institutionalised population whose primary purpose is to collect information on the use and cost of health care. Panels are two years long and are conducted in five rounds, which are conducted every five to six months. A new panel begins annually, while the last panel is in its second year

(http://www.meps.ahrq.gov/survey_comp/hc_data_collection.jsp). Each panel typically contains about 30,000 to 35,000 individual respondents.

MEPS was initiated in 1996 but only began collecting health status data in the form of 12-Item Short Form Survey (SF-12) responses in 2000. For GBD 2016, we used data from 2000–2014. Respondents self-administer the SF-12 twice per panel, at rounds two and four, typically about a year apart. Only adults 18 years and older completed the SF-12. MEPS also usually collects information on diagnoses based on self-report of reasons for encounters with health services. In addition, diagnoses are derived through additional questions on "problems that bother you" or conditions that led to "disability days," ie, days out of role due to illness. Professional coders translate the verbatim text into three-digit ICD-9 codes. The main reason for other MSK being measured in MEPS relates to health care contact.

To derive a crosswalk of SF-12 values into a scale comparable with that used by the GBD disability weights, small studies on convenience samples were conducted asking respondents to fill in SF-12 to reflect 62 lay descriptions of diverse severity that were used to derive the GBD disability weights. From these responses a relationship between SF-12 summary score and the GBD DWs was derived. With regression methods, average disability weights were calculated for each of 156 conditions for which there were corresponding diagnoses in MEPS, while controlling for any comorbid other condition by adding dummy variables for each condition. We binned the amount of DW attributed to other MSK across the seven health states assuming thresholds at the midpoints between DW values.

Other neglected tropical diseases

Other neglected tropical diseases is a residual category in addition to the specific neglected tropical diseases causes that were modelled separately. There are many diverse types of neglected tropical diseases included in this category, which are encompassed by the following ICD-10 codes:

A68 Relapsing fevers

A68.0 Louse-borne relapsing fever

A68.1 Tick-borne relapsing fever

A68.9 Relapsing fever, unspecified

A69.2 Lyme disease

A69.20 Lyme disease, unspecified

A69.21 Meningitis due to Lyme disease

A69.22 Other neurologic disorders in Lyme disease

A69.23 Arthritis due to Lyme disease

A69.29 Other conditions associated with Lyme disease

A69.5 There is not this code in ICD10 site, but we have this in mortality data

A69.8 Other specified spirochetal infections

A69.9 Spirochetal infection, unspecified

A75 Typhus fever

A75.0 Epidemic louse-borne typhus fever due to Rickettsia prowazekii

A75.1 Recrudescent typhus [Brill's disease]

A75.2 Typhus fever due to Rickettsia typhi

A75.3 Typhus fever due to Rickettsia tsutsugamushi

A75.9 Typhus fever, unspecified

A77 Spotted fever [tick-borne rickettsioses]

A77.0 Spotted fever due to Rickettsia rickettsii

A77.1 Spotted fever due to Rickettsia conorii

A77.2 Spotted fever due to Rickettsia siberica

A77.3 Spotted fever due to Rickettsia australis

A77.4 Ehrlichiosis

A77.40 Ehrlichiosis, unspecified

A77.41 Ehrlichiosis chafeensis [E. chafeensis]

A77.49 Other ehrlichiosis

A77.8 Other spotted fevers

A77.9 Spotted fever, unspecified

A78 Q fever

A79 Other rickettsioses

A79.0 Trench fever

A79.1 Rickettsialpox due to Rickettsia akari

A79.8 Other specified rickettsioses

A79.81 Rickettsiosis due to Ehrlichia sennetsu

A79.89 Other specified rickettsioses

A79.9 Rickettsiosis, unspecified

A92 Other mosquito-borne viral fevers

A92.0 Chikungunya virus disease

A92.1 O'nyong-nyong fever

A92.2 Venezuelan equine fever

A92.3 West Nile virus infection

A92.30 West Nile virus infection, unspecified

A92.31 West Nile virus infection with encephalitis

A92.32 West Nile virus infection with other neurologic manifestation

A92.39 West Nile virus infection with other complications

A92.4 Rift Valley fever

A92.8 Other specified mosquito-borne viral fevers

A92.9 Mosquito-borne viral fever, unspecified

A93 Other arthropod-borne viral fevers, not elsewhere classified

A93.0 Oropouche virus disease

A93.1 Sandfly fever

A93.2 Colorado tick fever

A93.8 Other specified arthropod-borne viral fevers

A94 Unspecified arthropod-borne viral fever

A94.0 Unspecified arthropod-borne viral fever

A96 Arenaviral haemorrhagic fever

A96.0 Junin haemorrhagic fever

A96.1 Machupo haemorrhagic fever

A96.2 Lassa fever

A96.8 Other arenaviral haemorrhagic fevers

A96.9 Arenaviral haemorrhagic fever, unspecified

A98 Other viral haemorrhagic fevers, not elsewhere classified

A98.0 Crimean-Congo haemorrhagic fever

A98.1 Omsk haemorrhagic fever

A98.2 Kyasanur Forest disease

A98.3 Marburg virus disease

A98.5 Haemorrhagic fever with renal syndrome

A98.8 Other specified viral haemorrhagic fevers

B33.0 Epidemic myalgia

B33.1 Ross River disease

B60 Other protozoal diseases, not elsewhere classified

B60.0 Babesiosis

B60.1 Acanthamebiasis

B60.10 Acanthamebiasis, unspecified

B60.11 Meningoencephalitis due to Acanthamoeba (culbertsoni)

B60.12 Conjunctivitis due to Acanthamoeba

B60.13 Keratoconjunctivitis due to Acanthamoeba

B60.19 Other acanthamebic disease

B60.2 Naegleriasis

B60.8 Other specified protozoal diseases

B67.5 Echinococcus multilocularis infection of liver

B67.6 Echinococcus multilocularis infection, other and multiple sites

B67.61 Echinococcus multilocularis infection, multiple sites

B67.69 Echinococcus multilocularis infection, other sites

B67.7 Echinococcus multilocularis infection, unspecified

B70 Diphyllobothriasis and sparganosis

B70.0 Diphyllobothriasis

B70.1 Sparganosis

B71 Other cestode infections

B71.0 Hymenolepiasis

B71.1 Dipylidiasis

B71.8 Other specified cestode infections

B71.9 Cestode infection, unspecified

B74.3 Loiasis

B74.4 Mansonelliasis

B74.8 Other filariases

B74.9 Filariasis, unspecified

B75 Trichinellosis

B83 Other helminthiases

B83.0 Visceral larva migrans

B83.1 Gnathostomiasis

B83.2 Angiostrongyliasis due to Parastrongylus cantonensis

B83.3 Syngamiasis

B83.4 Internal hirudiniasis

B83.8 Other specified helminthiases

P37.1 Congenital toxoplasmosis

Because these neglected tropical diseases are diverse in their underlying causes and risk factors, as well as in their associated health outcomes, modelling them together in a DisMod-MR model would not produce reliable estimates of prevalence or excess mortality. Instead, we calculated the YLDs caused by neglected tropical diseases directly using a YLD/YLL ratio.

We calculated the ratio of YLLs for other neglected tropical diseases to the sum of YLLs across the specified neglected tropical disease, using YLL estimates from the GBD 2019 cause of death analysis. We then multiplied this ratio by the YLDs estimates for the specified neglected tropical diseases from the GBD 2019 non-fatal analysis, providing us with an estimate of the YLDs associated with other neglected tropical diseases. The YLDs of anaemia due to other neglected tropical diseases were estimated using a different approach (see anaemia documentation for details).

Changes from GBD 2019 to GBD 2021

We have made no substantive changes in the modelling strategy from GBD 2019.

We did not apply any adjustments for the COVID-19 pandemic to other neglected tropical diseases due to a lack of available data quantifying the impacts of the pandemic on NTD epidemiology.

Other neurological disorders

In addition to the neurological disorders described above, there are many diverse types of neurological disorders with a range of severities and associated sequelae. Because these neurological disorders are

diverse in their underlying causes and risk factors as well as in their associated health outcomes, modelling them together in a DisMod-MR model would not produce reliable estimates of prevalence or excess mortality. Instead, we calculated the YLDs caused by neurological disorders directly using a YLD/YLL ratio.

We calculated the ratio of YLDs to YLLs across the specified neurological disorders for which non-fatal outcomes were modelled, using YLL estimates from the GBD 2020 cause of death (CoD) analysis. We then multiplied this YLD/YLL ratio by the YLL estimates for other neurological disorders from the GBD 2020 CoD analysis, providing us with an estimate of the YLDs associated with other neurological disorders.

Other sense organ diseases

Flowchart

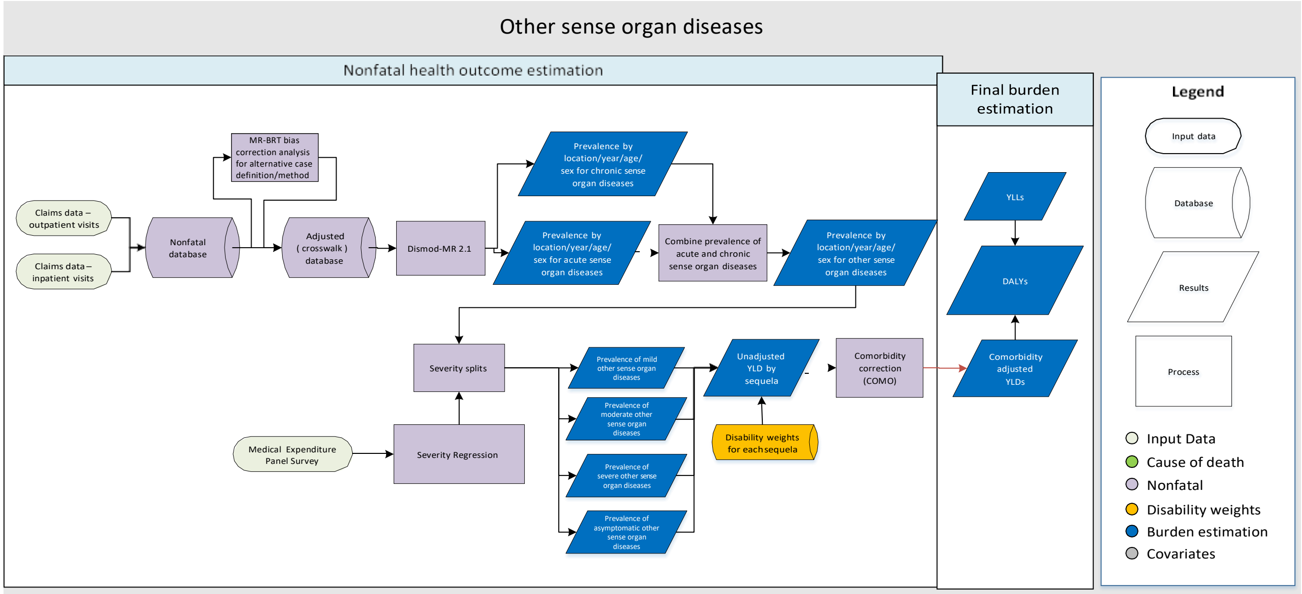

Case definition

Other sense organ disease is a residual cause capturing both acute and chronic eye and ear conditions that do not map to other causes but lead to non-trivial morbidity. These include the following ICD-9 codes: 077, 360, 364, 370-77, 379, 380, 386, and 388, which encompass a plethora of eye and ear disorders and conditions.

Table 1. ICD-9 codes included in other sense organs disease category.

ICD Code	Description
077	Other diseases of conjunctiva due to viruses and chlamydiae
360	Disorders of the globe

364	Disorders of iris and ciliary body
370-77	Keratitis, corneal opacity and other disorders of cornea, disorders of conjunctiva, inflammation of eyelids, other disorders of eyelids, disorders of the lacrimal system, disorders of the orbit, disorders of optic nerve and visual pathways
379	Other disorders of eye
380	Disorders of external ear
386	Vertiginous syndromes and other disorders of vestibular system
388	Other disorders of ear

Input data

Model inputs

For GBD 2021, we used claims data from the USA and Poland to model other sense organ diseases; these conditions do not appear in inpatient hospital data. We tested the inclusion of Taiwan and Russia claims data as well, but ultimately outliered these data sources. ICD-9 codes were assigned at the five-digit level to either acute or chronic conditions as listed elsewhere in the appendix table of all ICD codes.

Table 2. Data Inputs for other sense organ diseases morbidity modelling by parameter

Measure	New sources	Countries with data	Total sources
All measures	3	4	47
Prevalence	3	4	32
Incidence	3	4	32
Proportion	0	1	15

Modelling strategy

For GBD 2021, data were extracted separately for the chronic and acute conditions included in other sense organ diseases. Chronic data were extracted as prevalence, and acute data as incidence. We then ran two separate DisMod-MR 2.1 models[1] (disease model—Bayesian meta-regression, details on this method can be found in appendix 1, section 4.5 of the citation). The chronic model, with prevalence data, was run as a prevalence-only model. The acute model was run as a full compartmental model with incidence data, assuming zero excess mortality and duration of one week (remission = 52). In both models, to correct for systematically lower data from 2000 USA claims, we used a study-level covariate to crosswalk the 2000 USA claims data using a Bayesian meta-regression tool called MR-BRT[1] (meta-regression—Bayesian, regularised, trimmed; additional information can be found in appendix 1, section 4.4.1 of the cited paper). Since the only data sources are from the USA and Poland, we did not use any country-level covariates in this model.

Table 3. MR-BRT crosswalk adjustment factors for acute other sense organ diseases

Data input	Reference or alternative case definition	Gamma	Beta coefficient, logit (95% CI)*	Adjustment factor**
USA MarketScan (2010 onward)	Reference	0.18	---	---
USA MarketScan 2000	Alternative		–0.42 (–0.78 to –0.07)	0.66

MR-BRT crosswalk adjustments can be interpreted as the factor the alternative case definition is adjusted by to reflect what it would have been had it been measured using the reference case definition. If the log/logit beta coefficient is negative, then the alternative is adjusted up to the reference. If the log/logit beta coefficient is positive, then the alternative is adjusted down to the reference.

*****The adjustment factor column is the exponentiated beta coefficient. For log beta coefficients, this is the relative rate between the two case definitions. For logit beta coefficients, this is the relative odds between the two case definitions.***

Table 4. MR-BRT crosswalk adjustment factors for chronic other sense organ diseases

Data input	Reference or alternative case definition	Gamma	Beta coefficient, logit (95% CI)*	Adjustment factor**
USA MarketScan (2010 onward)	Reference	0.18	---	---
USA MarketScan 2000	Alternative		–0.54 (–0.90 to –0.19)	0.37

MR-BRT crosswalk adjustments can be interpreted as the factor the alternative case definition is adjusted by to reflect what it would have been had it been measured using the reference case definition. If the log/logit beta coefficient is negative, then the alternative is adjusted up to the reference. If the log/logit beta coefficient is positive, then the alternative is adjusted down to the reference.

*****The adjustment factor column is the exponentiated beta coefficient. For log beta coefficients, this is the relative rate between the two case definitions. For logit beta coefficients, this is the relative odds between the two case definitions.***

We then aggregated chronic and acute prevalence outputs, resulting in the prevalence of other sense organ diseases by country, age, year, and sex.

Severity splits and disability weights

The basis of the GBD disability weight survey assessments are lay descriptions of sequelae highlighting major functional consequences and symptoms. Severity splits for other sense organ diseases were calculated via the Medical Expenditure Panel Survey (MEPS) regression borrowing from disability weights used for infectious disease for acute other sense organ diseases and from vertigo and physical disfigurement for chronic other sense organ disease.[2]

Severity distributions are listed in the table below, and provide details on the severity levels for other sense organ diseases in GBD 2021 and the associated disability weight (DW) with that severity.

Table 5. Disability weights for chronic and acute other sense organ disease severity levels.

Chronic:

Severity	Proportion	Health state	Disability weight
Moderate (vertigo)	0.21 (0.15–0.28)	Has short spells of dizziness and loss of balance; between spells the person is worried the spells will occur again	0.113 (0.078–0.159)
Mild (disfigurement)	0.37 (0.30–0.42)	This person has slight physical deformity which causes some worry and discomfort	0.011 (0.005–0.021)
Asymptomatic	0.42 (0.41–0.44)	Asymptomatic	N/A

Acute:

Severity	Proportion	Health state	Disability weight
Moderate (moderate infectious disease)	0.25 (0.18–0.32)	Has a fever and aches, and feels weak, which causes some difficulty with daily activities	0.05 (0.033–0.073)
Mild (mild infectious disease)	0.30 (0.23–0.37)	This person has low fever and mild discomfort but no difficulty with daily activities	0.006 (0.002–0.012)
Asymptomatic	0.45 (0.43–0.46)	Asymptomatic	N/A

[1] Vos T, Lim SS, Abbafati C, *et al.* Global burden of 369 diseases and injuries in 204 countries and territories, 1990–2019: a systematic analysis for the Global Burden of Disease Study 2019. *The Lancet* 2020; 396: 1204–22. doi: https://doi.org/10.1016/S0140-6736(20)30925-9

[2] Salomon JA, Haagsma JA, Davis A, *et al.* Disability weights for the Global Burden of Disease 2013 study. *Lancet Global Health* 2015; 3: e712-23. doi: https://doi.org/10.1016/S2214-109X(15)00069-8

Other skin and subcutaneous diseases

Flowchart for other skin and subcutaneous diseases (OSSD)

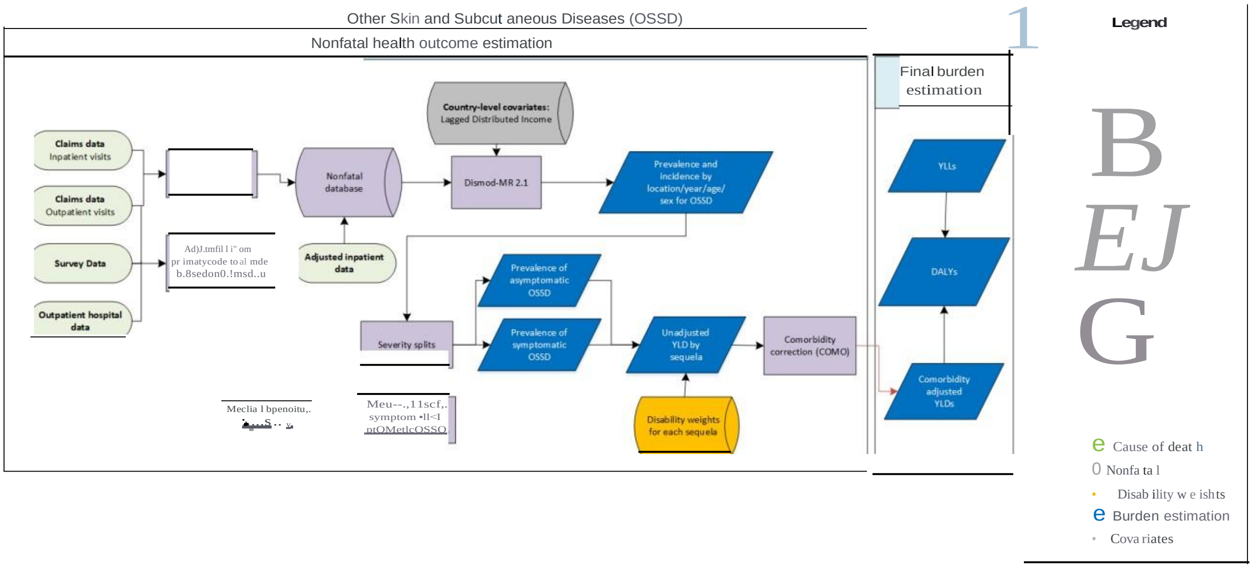
Other Skin and Subcutaneous Diseases (OSSD)
Nonfatal health outcome estimation
Final burden estimation
Legend
Claims data Inpatient visits
Claims data Outpatient visits
Survey Data
Outpatient hospital data
Country-level covariates: Lagged Distributed Income
Nonfatal database
Dismod-MR 2.1
Prevalence and incidence by location/year/age/sex for OSSD
Adjusted inpatient data
Prevalence of asymptomatic OSSD
Prevalence of symptomatic OSSD
Severity splits
Unadjusted YLD by sequela
Comorbidity correction (COMO)
Disability weights for each sequela
YLLs
DALYs
Comorbidity adjusted YLDs
Burden estimation

Input data and methodological summary for OSSD

Case definition

The other skin and subcutaneous diseases category encompasses a large group of skin conditions not captured in other skin categories: other viral infections characterised by skin and mucous membrane lesions, not elsewhere classified (B08), unspecified viral infection characterised by skin and mucous membrane lesions (B09), pediculosis and phthiriasis (B85), myiasis (B87), other infestations (B88), sarcoidosis of skin (D86.3), porphyria cutanea tarda (E80.1), other and unspecified porphyria (E80.2), pemphigus (L10), other acantholytic disorders (L11), pemphigoid (L12), other bullous disorders (L13), bullous disorders in diseases classified elsewhere (L14), lichen simplex chronicus and prurigo (L28), pityriasis rosea (L42), lichen planus (L43), other papulosquamous disorders (L44), papulosquamous disorders in diseases classified elsewhere (L45), exfoliation due to erythematous conditions according to extent of body surface involved (L49), erythema multiforme (L51), erythema nodosum (L52), other erythematous conditions (L53), erythema in diseases classified elsewhere (L54), other acute skin changes due to ultraviolet radiation (L56), skin changes due to chronic exposure to nonionising radiation (L57), other disorders of skin and subcutaneous tissue related to radiation (L59), nail disorders (L60), nail disorders in diseases classified elsewhere (L62), androgenic alopecia (L64), other nonscarring hair loss (L65), cicatricial alopecia [scarring hair loss] (L66), hair color and hair shaft abnormalities (L67), hypertrichosis (L68), rosacea (L71), follicular cysts of skin and subcutaneous tissue (L72), other follicular disorders (L73), eccrine sweat disorders (L74), apocrine sweat disorders (L75), vitiligo (L80), other disorders of pigmentation (L81), seborrheic keratosis (L82), acanthosis nigricans (L83), corns and callosities (L84), other epidermal thickening (L85), keratoderma in diseases classified elsewhere (L86), transepidermal elimination disorders (L87), atrophic disorders of skin (L90), hypertrophic disorders of skin (L91), granulomatous disorders of skin and subcutaneous tissue (L92), other localised connective tissue disorders (L94), vasculitis limited to skin, not elsewhere classified (L95), and other disorders of skin and subcutaneous tissue in diseases classified elsewhere (L99).

Quantity of interest	Reference or Alternative	Definition
Other skin and subcutaneous diseases	Reference	Other skin and subcutaneous diseases as indicated by claims data since 2010 and hospital outpatient data.
Other skin and subcutaneous diseases	Alternative	Other skin and subcutaneous diseases as indicated by claims data in 2000.

Input data

In the GBD 2010 study, a systematic review of the literature was conducted using PubMed and Google Scholar to capture epidemiological data for skin diseases not captured in the other skin categories. The inclusion criteria stipulated that studies (1) must be published between 1980 and 2012; (2) must provide data on the incidence or prevalence; (3) must use samples representative of the general population (ie, samples derived from the experimental arm of clinical trials or based in dermatology clinics were excluded); (4) must use a sample size larger than 100; and (5) must provide sufficient information on study method and sample characteristics to assess the quality of the study. For GBD 2013, the GBD 2010 search strategy was replicated to capture epidemiological studies published between 2012 and 2013. Data from

USA claims for 2000 and 2010–2016 by USA state and Taiwan (province of China) claims data for 2016 were included in GBD 2019 as well. Data were outliered or excluded if we found them unreasonable when compared to regional, super-regional, and global rates.

Table 1: Data inputs for OSSD morbidity modelling by parameter

**Cause/impairment name**	**Measure**	**Countries with data**	**New sources**	**Total sources**
Other skin and subcutaneous diseases	All measures	2	3	37
Other skin and subcutaneous diseases	Prevalence	2	3	22
Other skin and subcutaneous diseases	Proportion	1	0	15

Table 2: MR-BRT crosswalk adjustment factors for OSSD

Data input	Reference or alternative case definition	Gamma	Beta coefficient, logit* (95% CI)	Adjustment factor**
USA MarketScan 2010–2016, outpatient data	Reference	0.05	---	---
USA MarketScan 2000	Alternative		–0.18 (–0.64 to 0.29)	0.46

****MR-BRT crosswalk adjustments can be interpreted as the factor the alternative case definition is adjusted by to reflect what it would have been had it been measured using the reference case definition. If the log/logit beta coefficient is negative, then the alternative is adjusted up to the reference. If the log/logit beta coefficient is positive, then the alternative is adjusted down to the reference.***

*****The adjustment factor column is the exponentiated beta coefficient. For log beta coefficients, this is the relative rate between the two case definitions. For logit beta coefficients, this is the relative odds between the two case definitions.***

Modelling strategy

DisMod-MR 2.1, a Bayesian meta-regression tool, was used to estimate prevalence by age, sex, year, and geography (subnational, country, region, super-region) for skin and other subcutaneous diseases.

We assumed remission of one, implying a duration of 12 months. Similar to GBD 2017, we used a time window of 25 years to determine which datapoints were used for a particular year of fit.

In GBD 2021, we replaced our within-DisMod crosswalks with crosswalks completed using the MR-BRT modelling tool. We adjusted USA MarketScan 2000 data toward the level of other prevalence datapoints which were more representative of the general population. In addition, log-transformed lagged distributed income (LDI) was used as a country-level covariate to guide estimates for locations with few or no data.

We have made no substantive changes in the modelling strategy from GBD 2019.

Table 3. Severity distribution, details on the severity levels for OSSD and the associated disability weight (DW) with that severity

Sequela	Severity level	Lay description	DW (95% CI)
Asymptomatic other skin and subcutaneous diseases	Asymptomatic		0
Symptomatic other skin and subcutaneous diseases	Disfigurement, level 1	The person has a slight, visible physical deformity that others notice, which causes some worry and discomfort.	0.011 (0.005–0.021)

Table 4. Covariates. Summary of covariates used in the OSSD DisMod-MR meta-regression model

Covariate	Type	Parameter	Exponentiated beta (95% uncertainty interval)
LDI (I$ per capita)	Country-level	Prevalence	1.35 (1.35–1.35)

Other unspecified infectious diseases

Flowchart

Anemia Impairment Estimation

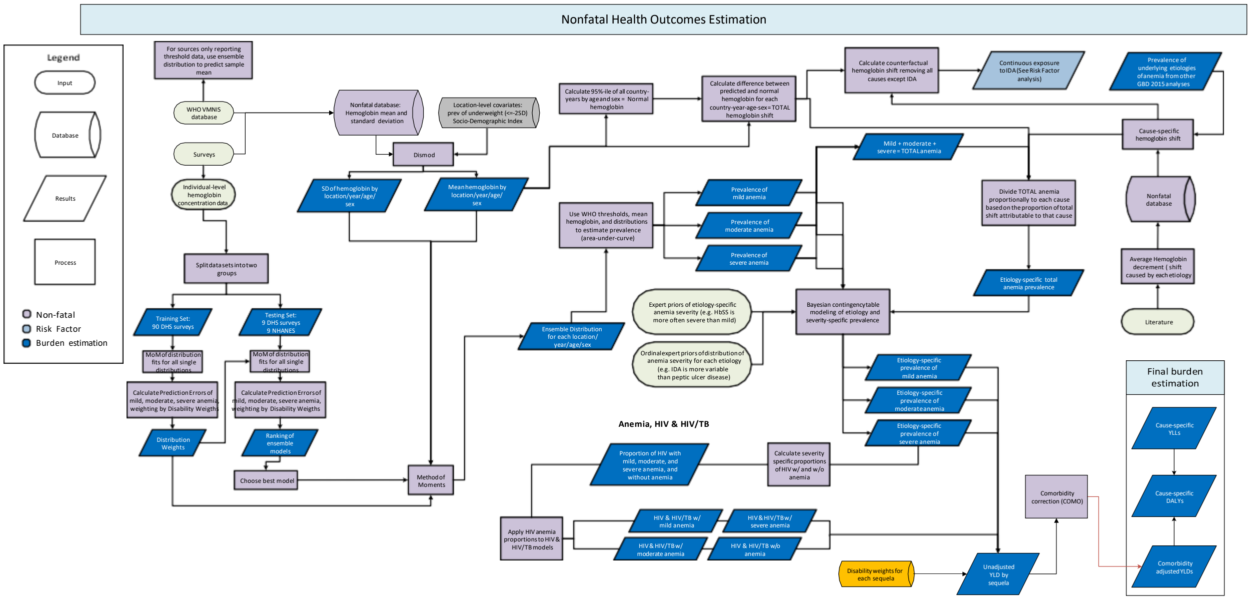

Input data and methodological summary for other unspecified infectious diseases

For GBD 2021, we estimate other unspecified infectious diseases using the residual anaemia impairment envelope based on a fixed proportion of redistribution. The resulting models of mild anaemia due to other infectious diseases, moderate anaemia due to other infectious diseases, and severe anaemia due to other infectious diseases go into our central computation to generate YLDs based on our prevalence values.

Causes for which allocation of residual anaemia envelope was based on fixed proportion redistribution methods*:
Iron-deficiency anaemia (IDA)
Other infectious diseases
Other neglected tropical diseases
Other endocrine, nutrition, blood, and immune disorders
Other haemoglobinopathies and haemolytic anemias

* A minimum of 10% of all anaemia was assigned to residual categories based on analysis of NHANES-III data from the United States.

References

1. Kassebaum NJ. The Global Burden of Anemia. *Hematology/Oncology Clinics* 2016; **30**: 247–308.
2. Kassebaum NJ, Jasrasaria R, Naghavi M, *et al.* A systematic analysis of global anemia burden from 1990 to 2010. *Blood* 2014; **123**: 615–24.

Other urinary diseases

In addition to specified urinary diseases including urolithiasis, urinary tract infections and interstitial nephritis, and benign prostatic hyperplasia, there are other types of urinary diseases with a range of severities and associated sequelae. Because these urinary diseases are diverse in their underlying causes and risk factors as well as in their associated health outcomes, modelling them together in a DisMod-MR model would not produce reliable estimates of prevalence. Instead, we calculated the YLDs caused by other urinary disorders directly using an YLD/YLL ratio as a 'placeholder'.

We calculated the ratio of YLDs to YLLs across the specified urinary diseases for which non-fatal outcomes were modelled, using YLL estimates from the GBD 2021 cause of death analysis. We then multiplied this YLD/YLL ratio by the YLL estimates for other urinary diseases.

Otitis media

Flowchart

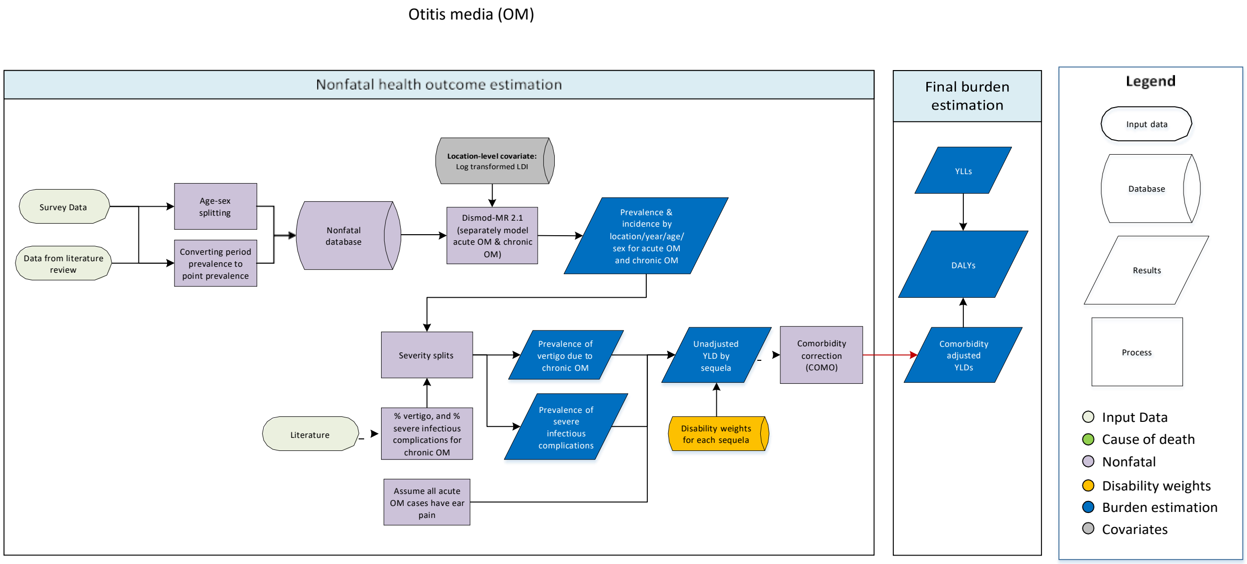

Case definition

Otitis media is an infection of the middle ear space. We included acute otitis media, chronic otitis media, and hearing loss due to chronic otitis media in the GBD non-fatal outcome modelling. Hearing loss due to chronic otitis media estimation is included in the hearing loss report provided separately. The ICD-10 codes are H65-H75.83, and ICD-9 codes are 381-384.9.

Quantity of interest	Reference or Alternative	Definition
Incidence of acute otitis media	Reference	Cases of acute otitis media from clinical diagnosis, surveys, or literature.

Incidence of chronic otitis media	Reference	Cases of chronic otitis media from surveys or literature.
Prevalence of acute otitis media	Reference	Cases of acute otitis media from clinical diagnosis, surveys, or literature.
Prevalence of chronic otitis media	Reference	Cases of chronic otitis media from surveys or literature.
Remission of chronic otitis media	Reference	The rate at which chronic otitis media cases stop meeting the ICD diagnostic criteria.

Input data

A systematic review of the incidence and prevalence of otitis media was conducted for GBD 2021. The PubMed search terms were: (otitis media[Title/Abstract] AND (inciden*[Title/Abstract] OR prevalen*[Title/Abstract] OR remission[Title/Abstract] OR duration[Title/Abstract]) AND ("2017/10/01"[PDAT] : "3000"[PDAT]) NOT (animals[MESH] NOT humans[MESH]))

The exclusion criteria were:

1. Studies that were not population-based, eg, hospital or clinic-based studies
2. Studies that did not provide primary data on epidemiological parameters, eg, commentaries
3. Studies with a sample size of less than 150
4. Reviews
5. Case series

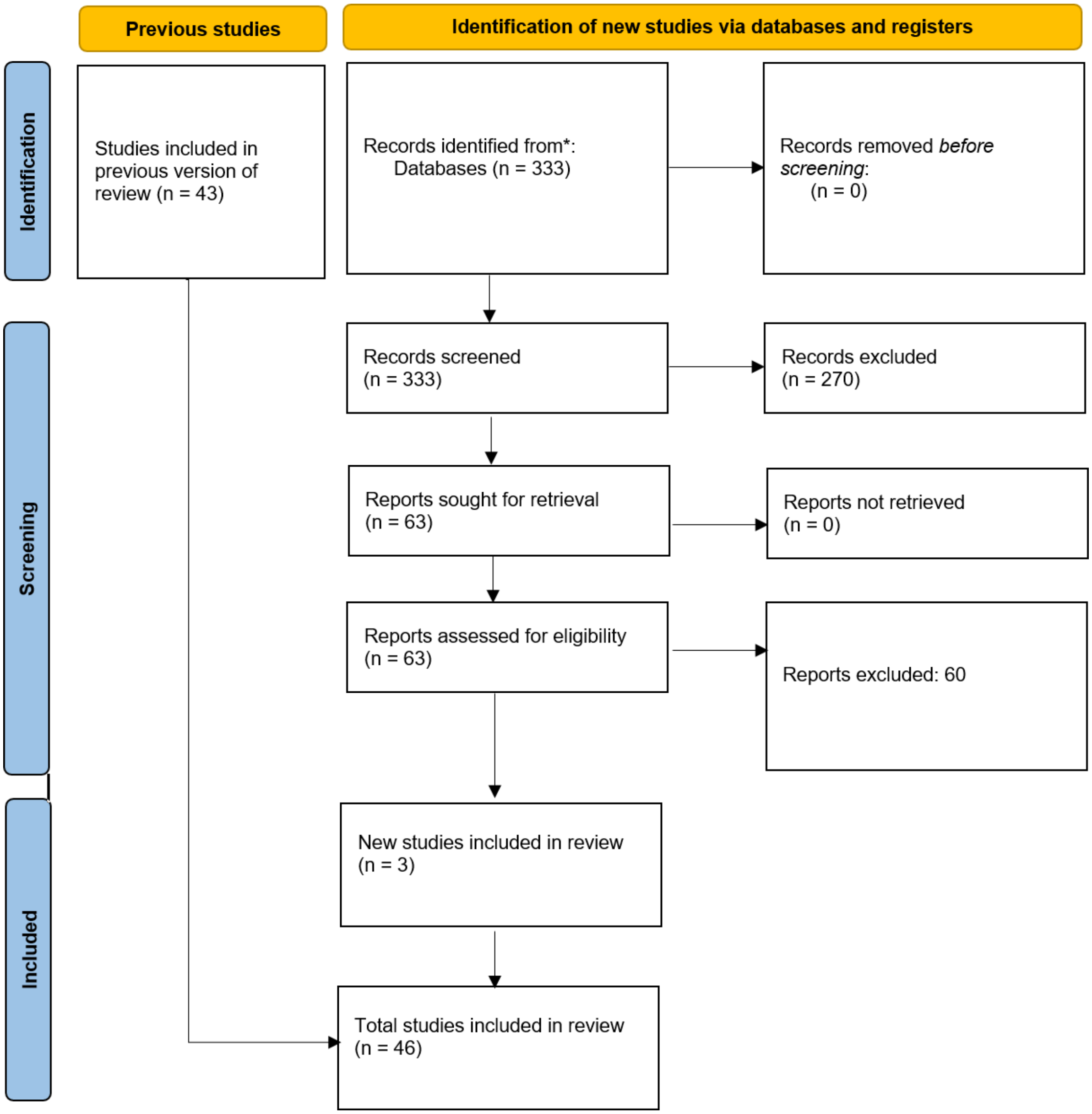

Figure 1 PRISMA diagram for otitis media 2021 systematic review of incidence and prevalence sources.

In addition, CF3-corrected data from inpatient and outpatient claims were included in the acute otitis model.

Table 1: Data Inputs for otitis media morbidity modelling by parameter.

	**Countries with data**	**New sources**	**Total sources**
Incidence	10	3	52
Prevalence	21	3	33
Remission	4	0	5
Other	0	0	0

Modelling strategy

We assume that all acute otitis media cases would experience ear pain. The severity distributions for chronic otitis media based on the study by Lin and colleagues (2009) were as follows: (i) vertigo (2.9%, 95% CI: 2.4–3.6), and (ii) severe infectious complications (0.05%, 95% CI: 0.01–0.2). We assumed that all chronic otitis media cases experience either mild or moderate hearing loss. The lay descriptions and disability weights for severity levels derived from the GBD disability weights study are shown below.

Table 2. Severity distribution, details on the severity levels for otitis media and the associated disability weight (DW) with that severity.

Severity level	Lay description	DW (95% CI)
Acute otitis media	Has an earache that causes some difficulty with daily activities.	0.013 (0.007–0.024)
Severe infectious complications due to chronic otitis media	Has an earache that causes some difficulty with daily activities.	0.013 (0.009–0.019)
Mild hearing loss due to chronic otitis media	Has great difficulty hearing and understanding another person talking in a noisy place (for example, on an urban street).	0.01 (0.004–0.019)
Moderate hearing loss due to chronic otitis media	Is unable to hear and understand another person talking in a noisy place (for example, on an urban street), and has difficulty hearing another person talking even in a quiet place or on the phone.	0.027 (0.015–0.042)
Mild hearing loss with ringing due to chronic otitis media	Has great difficulty hearing and understanding another person talking in a noisy place (for example, on an urban street), and sometimes has annoying ringing in the ears.	0.021 (0.012–0.036)
Moderate hearing loss with ringing due to chronic otitis media	Is unable to hear and understand another person talking in a noisy place (for example, on an urban street), and has difficulty hearing another person talking even in a quiet place or on the phone, and has annoying ringing in the ears for more than 5 minutes at a time, almost every day.	0.074 (0.049–0.107)
Vertigo with mild hearing loss due to chronic otitis media	*	0.122 (0.079–0.17)
Vertigo with mild hearing loss and ringing due to chronic otitis media	*	0.132 (0.086–0.184)
Vertigo with moderate hearing loss due to chronic otitis media	*	0.137 (0.089–0.189)

Vertigo with moderate hearing loss and ringing due to chronic otitis media	*	0.179<br>(0.12–0.247)

* See the hearing loss report for the lay descriptions and disability weights for different severity levels.

We modelled acute and chronic otitis media as separate non-fatal health outcomes using DisMod-MR 2.1. Log-transformed LDI covariate was used as a country-level covariate to model chronic otitis media.

Table 4a. Covariates. Summary of covariates used in the acute otitis media DisMod-MR meta-regression model

Covariate	Type	Parameter	Exponentiated beta (95% CI)
Sex	Study-level	Prevalence	0.99 (0.66–1.50)
Sex	Study-level	Incidence	0.79 (0.78–0.80)

Table 4b. Covariates. Summary of covariates used in the chronic otitis media DisMod-MR meta-regression model

Covariate	Type	Parameter	Exponentiated beta (95% CI)
Log LDI	Country-level	Prevalence	0.72 (0.63–0.82)
Sex	Study-level	Prevalence	1.14 (0.98–1.32)
Sex	Study-level	Incidence	1.26 (0.66–2.49)

Reference

Lin, Y. S., Lin, L. C., Lee, F. P., & Lee, K. J. (2009). The prevalence of chronic otitis media and its complication rates in teenagers and adult patients. *Otolaryngology-Head and Neck Surgery*, *140*(2), 165-170.

Pancreatitis

Flowchart

Pancreatitis

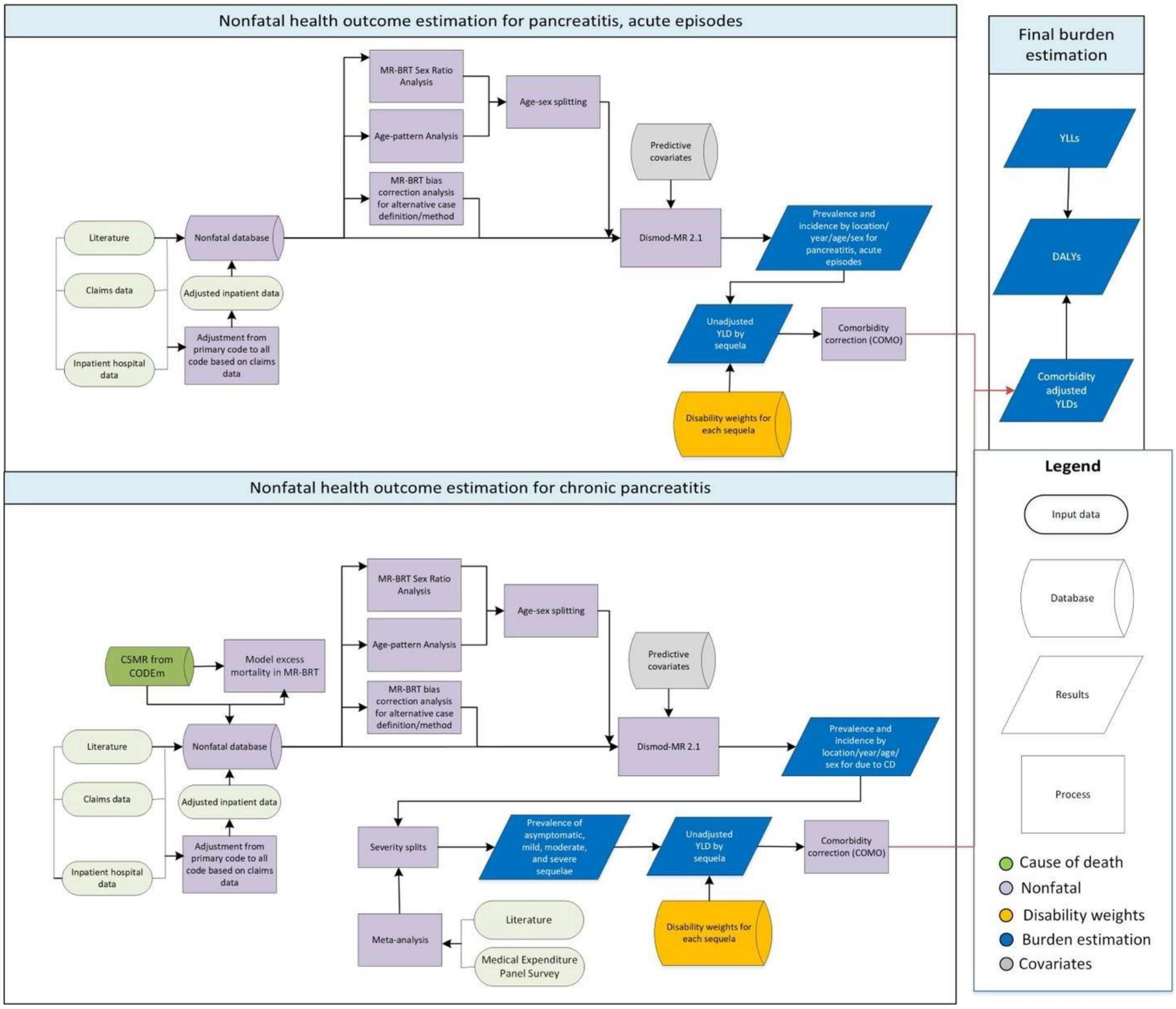

Input data and methodological summary for chronic pancreatitis and pancreatitis, acute episodes

Case definition

Pancreatitis is the inflammation of the pancreas, acutely or chronically. Acute pancreatitis involves active inflammation and injury to the pancreas, generally presenting with severe upper abdominal pain and nausea, inappropriate release of pancreatic contents, and a systemic inflammatory response with fever, low blood pressure, and, in some cases, failure of one or more organs. Chronic pancreatitis involves permanent damage to the pancreas from longstanding or recurrent inflammation; this produces chronic or episodic abdominal pain and nausea and ultimately failure of the pancreas to produce and release digestive enzymes and hormones, leading to chronic diarrhoea, poor absorption of nutrients from food, and diabetes.

Individuals with chronic pancreatitis can have superimposed episodes of acute pancreatitis, but acute episodes can also occur in individuals without chronic pancreatitis. In early rounds of GBD, we modelled

acute and chronic pancreatitis together, but starting in GBD 2017, we developed separate models for these two diseases.

ICD-10 codes are K85 for acute and K86 for chronic pancreatitis. ICD-9 code 577.0 corresponds to acute pancreatitis, and 577 and the remainder of its four-digit and five-digit constituents refer to chronic or unspecified pancreatitis.

Overall strategy

Two databases were used as inputs to two separate, complete compartmental DisMod models: pancreatitis, acute episodes, and chronic pancreatitis.

Input data and data processing

Input data

For GBD 2013, a systematic literature review was conducted to capture studies of prevalence and incidence of pancreatitis throughout the world. This search was updated for GBD 2015 and again for GBD 2016. A PubMed search was conducted using the following search terms: Pancreatitis[Title/Abstract] OR "Pancreatitis"[Mesh] OR "Pancreatitis, Acute Necrotizing"[Mesh] OR "Pancreatitis, Chronic"[Mesh]) AND (prevalence[Title/Abstract] OR incidence[Title/Abstract]) AND ("2010/01/01"[Date - Publication] : "2016/11/01"[Date - Publication]) NOT(animals[MeSH] NOT humans[MeSH])) NOT("comment"[Publication Type])

The exclusion criteria were:

1. Studies clearly not representative of the national population (ie, alcoholics or smokers).
2. Studies that did not provide primary data on epidemiological parameters (eg, a commentary piece).

Studies were added to the acute database if they measured the incidence of acute pancreatitis as defined by appropriate ICD codes, or by a combination of clinical, biochemical, and radiographic criteria. The acute database included studies that measured incidence of first episode of acute pancreatitis only, and studies that measured incidence of all acute pancreatitis, including recurrent episodes. Studies that included individuals with underlying chronic pancreatitis were excluded from the acute database. Studies were added to the chronic database if they employed appropriate ICD codes or appropriate clinical, biochemical, and radiographic criteria of chronic pancreatitis. Some studies reported incidence of acute and chronic disease separately and data were extracted to both databases, but those few studies that reported only a single combined estimate for both acute and chronic disorders were excluded.

In GBD 2017, the acute database included literature data extracted as prevalence from six countries, such as Ireland, Japan, and Poland. These data were excluded from analysis in both GBD 2019 and GBD 2021 because they did not meet the inclusion criteria for the acute database.

In addition to the literature studies, both databases included administrative data that were extracted as incidence for acute and prevalence for chronic. In GBD 2021, we newly added additional years of data from USA claims (year 2017) and Poland claims (year 2018), as well as hospital discharges in Greece, Armenia, Chile, Ecuador, Argentina, Italy, Brazil, and Spain.

Table 1. Data inputs for pancreatitis morbidity modelling by parameter

	Countries with data	New sources	Total sources
Prevalence	47	34	307
Incidence	51	38	367
Other	1	0	15

Inputs to our non-fatal modelling of chronic pancreatitis also included cause-specific mortality rate (CSMR) estimates taken from our fatal modelling process (see CoD cause-specific modelling description for pancreatitis in this appendix) and excess mortality rate (EMR) estimates modelled outside of DisMod (see the EMR data processing section below).

Incidence and prevalence input processing

Hospital discharge data provide observations about encounters, generally with only the primary diagnostic code for the encounter. Claims data, on the other hand, link claims for all inpatient and outpatient encounters for a single individual and provide primary and secondary diagnoses for all encounters.

Similar to GBD 2019, in the acute database, an individual was extracted from claims data as an incident case if that individual had one or more inpatient encounters with an appropriate ICD code as any diagnosis; readmissions within 30 days were assumed to be for the same episodes of illness. Hospital discharges were included only if the primary discharge diagnosis was a code for acute pancreatitis, and incident cases were estimated from number of discharges using a correction factor (ie, correction factor 1) from claims data.

In the chronic database, individuals were extracted from claims data as prevalent cases if they had at least one inpatient or two outpatient encounters with a chronic pancreatitis ICD code as any diagnosis. Hospital discharge data were processed by extracting discharges with an appropriate ICD code as primary diagnosis and adjusting using a correction factor (ie, correction factor 3) derived from claims data. Specifically, we modelled from the ratio of inpatient claims with chronic pancreatitis as primary diagnosis to total prevalent cases of chronic pancreatitis seen in claims data.

In GBD 2019, we improved the bias adjustment methods to allow a more direct comparison between different case definitions and/or study designs. In GBD 2017, we used data from published studies that employed rigorous case definitions as our reference standard for acute pancreatitis and adjusted clinical administrative data toward this reference standard by marking administrative data with binary covariates and estimating a fixed effect for this covariate in our DisMod meta-regression modelling process. This amounts to adjusting data using an ecological comparison and is vulnerable to compositional bias; if data from different location-years were collected using different methods or case definitions, true spatiotemporal differences in epidemiology can be erroneously adjusted, and differences truly due to differences in methods can be erroneously estimated as differences in underlying epidemiology. In GBD

2019, we avoided this risk by making pre-modelling bias adjustments and dropping data types that could not be rigorously adjusted. This was done by conducting a meta-regression of the relationship between datapoints matched on year, age, sex, and location, but differing with regard to one or more study design characteristic. This pre-modelling bias adjustment approach was used in GBD 2021.

As in GBD 2017, we desired to use data from published studies that identified cases through detailed chart review as the reference standard for the acute pancreatitis model. These studies used a combination of clinical presentation, biochemical, and radiographic findings to validate a case definition, which we refer to as “stringent criteria” in shorthand. Using the stringent criteria, ideally, we would then adjust other ICD-code-based administrative data. However, the number of matched pairs between reference and alternative (based on year, age, sex, and location) was small and yielded highly uncertain adjustment factors for the alternative case definitions. As a result, a new case definition was adopted in GBD 2019: diagnosis of acute pancreatitis as indicated by ICD code in a clinical encounter. Other case definitions and study design characteristics were adjusted toward this new reference standard. This choice of reference and adjustment approach remained the same in GBD 2021.

The chronic pancreatitis model used ICD-code-based administrative data as the reference standard in GBD 2017 due to scant literature data that were available. In GBD 2019, we attempted to employ the new bias adjustment method for chronic pancreatitis using the more rigorous case definition based on clinical, biochemical, and radiographic findings, but, as in the acute pancreatitis model, we could not find an adequate number of comparison pairs to inform reliable adjustment factors. Therefore, we decided to use the same ICD-based administrative data as the reference standard in GBD 2019 and GBD 2021, adjusting other case definitions and study design characteristics to this reference standard.

For both acute and chronic pancreatitis models, the USA claims data from the year 2000 and from the years 2010–2017 were each adjusted to the reference to adjust for selection bias due to commercial insurance.

The table below shows bias correction factors estimated using MR-BRT.

Table 2. MR-BRT crosswalk adjustment factors for pancreatitis

Acute pancreatitis episode: Incidence

Data input	Reference or alternative case definition	Gamma	Beta coefficient, log (95% CI)	Adjustment factor*
ICD-code based administrative data	Ref	0.30		
USA claims from year 2000	Alt		–0.18 (–1.12, 0.75)	0.83 (0.33, 2.12)
USA claims from years 2010–2017	Alt		0.19 (–0.44, 0.82)	1.21 (0.65, 2.26)
Stringent criteria	Alt		–0.22 (–1.05, 0.60)	0.80 (0.35, 1.82)

**Adjustment factor is the transformed beta coefficient in normal space and can be interpreted as the factor by which the alternative case definition is adjusted to reflect what it would have been if measured as the reference.*

Chronic pancreatitis: incidence

Data input	Reference or alternative case definition	Gamma	Beta coefficient, log (95% CI)	Adjustment factor*
ICD-code based administrative data	Ref	0.61		
Stringent criteria	Alt		–0.66 (–2.14, 0.82)	0.52 (0.12, 2.28)
[truncated: 1,247,753 more chars]
